# Mutational concordance analysis provides supportive information for double cancer diagnosis

**DOI:** 10.1186/s12885-021-07899-1

**Published:** 2021-02-19

**Authors:** Keiichi Hatakeyama, Takeshi Nagashima, Akifumi Notsu, Keiichi Ohshima, Sumiko Ohnami, Shumpei Ohnami, Yuji Shimoda, Akane Naruoka, Koji Maruyama, Akira Iizuka, Tadashi Ashizawa, Hirotsugu Kenmotsu, Tohru Mochizuki, Kenichi Urakami, Yasuto Akiyama, Ken Yamaguchi

**Affiliations:** 1 Medical Genetics Division, Shizuoka Cancer Center Research Institute, Sunto-gun, Shizuoka, 411-8777 Japan; 2 Cancer Diagnostics Research Division, Shizuoka Cancer Center Research Institute, Sunto-gun, Shizuoka, 411-8777 Japan; 3 SRL Inc., Shinjuku-ku, Tokyo, 163-0409 Japan; 4 Clinical Research Center, Shizuoka Cancer Center, Sunto-gun, Shizuoka, 411-8777 Japan; 5 Drug Discovery and Development Division, Shizuoka Cancer Center Research Institute, Sunto-gun, Shizuoka, 411-8777 Japan; 6 Experimental Animal Facility, Shizuoka Cancer Center Research Institute, Sunto-gun, Shizuoka, 411-8777 Japan; 7 Immunotheraphy Division, Shizuoka Cancer Center Research Institute, Sunto-gun, Shizuoka, 411-8777 Japan; 8 Division of Genetic Medicine Promotion, Shizuoka Cancer Center, Sunto-gun, Shizuoka, 411-8777 Japan; 9 Shizuoka Cancer Center, Sunto-gun, Shizuoka, 411-8777 Japan

**Keywords:** Whole exome sequencing, Double cancer, Tumor mutational burden, Poisson distribution

## Abstract

**Background:**

Mutation analysis using next-generation sequencing highlights the features of tumors with somatic alterations. However, the mutation profile of double cancer remains unclear. Here, we analyzed tumors derived from the same patient using whole exome sequencing (WES) to investigate the coherence of somatic mutations in double cancer.

**Methods:**

First, the tumor mutational burden (TMB) was investigated using WES of 5521 tumor specimens from a Japanese pan-cancer cohort. The frequencies of mutation concordance were then compared in these cancers. Finally, we calculated the expected value of mutational concordance fitting a Poisson distribution to determine the relationship between double and metastatic cancers.

**Results:**

In all, 44, 58, and 121 paired samples were diagnosed as double cancer, multifocal lesions (derived from identical tissues), and metastasis, respectively. Our analysis revealed that common somatic mutations were almost entirely absent in double cancer, whereas primary tumors and metastatic foci harbored several identical alterations. Concordance of the mutation profile in the same patient reflects the tumor origin and development, suggesting the potential for identifying double cancer based on common somatic mutations. Furthermore, according to a Poisson distribution, double cancer could be discriminated based on paired samples from the same patient. The probability of double cancer with more than 10 mutations was ≤1 part-per-billion (ppb, 10^− 9^). In multifocal lesions, 74% of tumor pairs accumulated ≤10 common mutations, implying a difference in tumor origin within identical tissues.

**Conclusions:**

These findings indicate that counting common somatic mutations can indicate the differences in origin between tumors derived from the same patient. Our mutation coherence analysis can thus provide beneficial information for diagnosing double cancer.

**Supplementary Information:**

The online version contains supplementary material available at 10.1186/s12885-021-07899-1.

## Background

Determining the primary cancerous organ is essential for adequate treatment. Multiple cancers within the same patient need to be identified as double/multifocal cancers or other forms of cancer, such as metastasis or cancers of unknown primary origin. Although the primary organ in these cancers is generally determined based on clinicopathological findings (e.g., immunohistochemical staining, tissue specificity, and shape of tumor cells), there are only a few studies on alternative approaches that can support these findings.

Recently, pan-cancer analysis of metastatic solid tumors revealed that the mutation profile of metastatic lesions reflects that of the primary tumor types [1]. This partial mutational concordance between primary and metastatic tumors suggests the potential ability for discriminating the primary site. For instance, some studies have tracked somatic mutations in a few metastatic cancers in the same patient [2, 3]. However, the incidence of mutational concordance in double cancer remains unclear due to the lack of similar mutation analysis in the same patient. Therefore, investigating mutational concordance in double cancer is important for understanding the tendency of mutation accumulation in tumors from the same patient.

In the current study, we performed next-generation sequencing in 44 double cancers, 58 multifocal lesions, and 121 metastatic cancers to compare the mutation profiles of multiple cancers in the same patient. The tumor mutational burden, frequencies of mutation concordance among cancers, and the relationship between double and metastatic cancers were determined.

## Methods

### Ethics statement

The age range of all patients was 11–98 years. If the patients were under 18 years of age, informed consent was obtained from their parents and written informed consent was obtained from patients aged 18 years and above; all aspects of this study were approved by the Institutional Review Committee of Shizuoka Cancer Center (authorization number 25–33). All experiments using clinical samples were performed in accordance with the approved Japanese ethical guidelines (Human Genome/Gene Analysis Research, 2017, provided by the Ministry of Health, Labor, and Welfare; https://www.mhlw.go.jp/stf/seisakunitsuite/bunya/hokabunya/kenkyujigyou/i-kenkyu/index.html).

### Clinical samples

In our analysis, double cancer, multifocal lesions (derived from identical tissues), and metastatic tumors were extracted from the Japanese pan-cancer cohort (project HOPE) comprising 5521 tumor specimens [4]. These samples had been clinicopathologically diagnosed by a pathologist after surgery. Tumors and their surrounding tissues (≥ 0.1 g) were dissected from surgical specimens immediately after resection of the lesion at the Shizuoka Cancer Center Hospital. Tumor samples were visually assessed by a clinical pathologist at our hospital when tumor content was ≥50%, and were not filtered further by pathophysiological features or cancer type. Peripheral blood was also collected as a paired control to exclude germline mutations. The details of the experimental protocols used have been described previously [4–9]. Briefly, tumor and normal tissues were immediately frozen in liquid nitrogen prior to DNA extraction. DNA was extracted from the tissues and peripheral blood samples using a QIAamp DNA Blood Mini Kit (Qiagen, Venlo, The Netherlands). Purified DNA was quantified using a NanoDrop system and a Qubit 2.0 Fluorometer (Thermo Fisher Scientific, Waltham, MA).

### Datasets for analysis of somatic alterations

To analyze the sequencing data derived from freshly frozen clinical samples, we used the pipeline described in our previous report [4]. Thereafter, the data were submitted to the National Bioscience Database Center (NBDC) Human Database as ‘Controlled-Access Data’ (https://humandbs.biosciencedbc.jp/en/). In this study, a part of the dataset containing double cancer, multifocal lesions, and metastatic cancer was extracted for analyzing somatic alterations, including TMB (Table S1).

### Poisson distribution

The Poisson distribution was adopted to estimate the frequency of mutation concordance. This discrete probability distribution can express the probability of a given number of events occurring in a fixed interval of time (or space). The formula for the Poisson probability mass function is

$$ p\left(k;\lambda \right)=\frac{e^{-\lambda }{\lambda}^k}{k}\mathrm{for}\kern0.5em k=0,\kern0.5em 1,\kern0.5em 2,\dots $$

Where *λ* is the shape parameter, which indicates the average number of concordant mutations in the given time interval, and *k* is the number of occurrences. In our study, the Poisson distribution was used to describe the distribution of concordant mutations (rare events) in somatic alterations within the same patient.

### Statistical analysis

To calculate the frequency of matched and mismatched mutation positions in pair samples, the matched mutations in paired samples were divided by all the detected somatic nonsynonymous/synonymous mutations. Welch’s *t*-test was performed to compare the concordance rate of somatic mutation and TMB. The concordance rate was determined by dividing the number of concordant mutations by the number of total mutations derived from the same patient. The results were considered significant at *p* < 0.01.

## Results

### Tissue distribution in double cancer and metastatic cancer

**Fig. 1:**
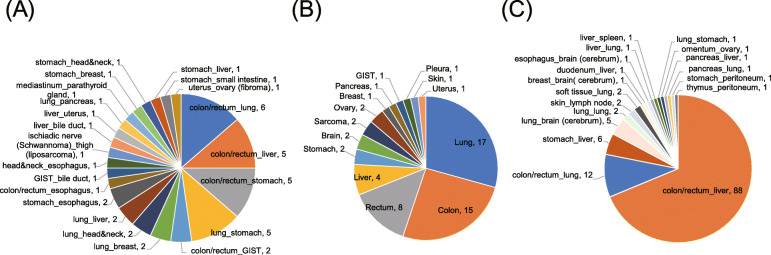
Sample profile in double cancer and metastatic cancer. **a** Distribution of tissues in 44 pairs of double cancer. The pair from the same patient is represented as before and after the underscore. **b** Distribution of tissues in 58 pairs of multifocal tumors derived from identical tissues. **c** Distribution of metastatic foci in each primary site. These pairs (*n* = 111) are composed of the same number of primary sites and metastatic foci. The pair in the same patient is represented as before and after the underscore (primary_metastatic)

We extracted 44 pairs of double cancer, 58 pairs of multifocal lesions derived from identical tissues, and 121 pairs of primary site and metastatic foci in the same patient from the Japanese version of The Cancer Genome Atlas dataset. The extracted samples received a definitive diagnosis postoperatively. Double cancer sometimes occurred in the colorectum (43%, 19/44), lung (39%, 17/44), and stomach (34%, 15/44) (Fig. 1a), and 69% of multifocal lesions derived were from identical tissues such as the lung and colorectum (Fig. 1b). Of the metastatic cancers, 74% were liver metastases derived from colorectal cancer (Fig. 1c).

### TMB profile in the same patient

**Fig. 2:**
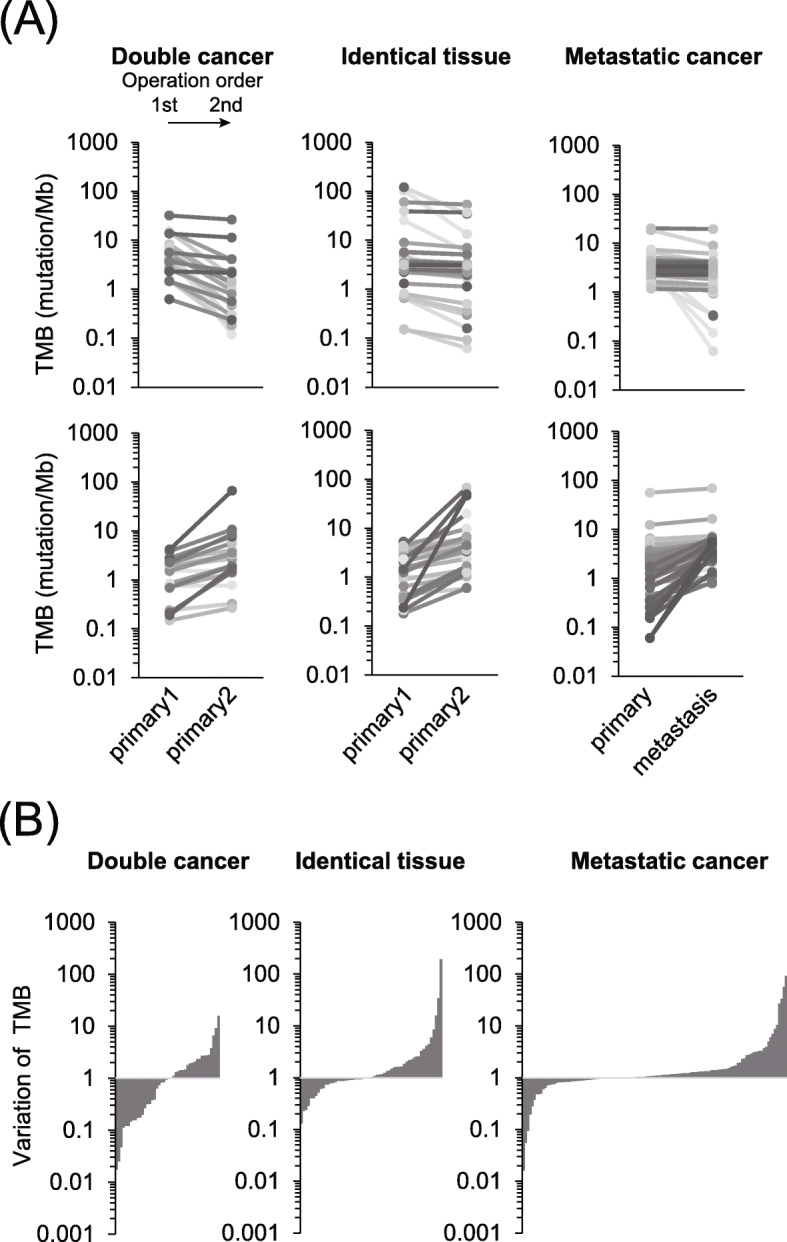
Comparison of mutation frequency in tumors derived from the same patient. **a** Transition of tumor mutational burden (TMB) in double/multifocal cancer and metastatic cancer. Primary 1 and 2 in double/multifocal cancer were determined in the first and second operation, respectively. Samples with decreased and increased TMB during primary 2 and metastasis are represented on the upper and lower panels, respectively. **b** Variation of TMB in the three cancer types. This rate represents an increase of TMB in primary 2 or metastasis when it exceeds 1.0

To trace the accumulation of somatic alterations in double, multifocal, and metastatic cancer, TMB transition was investigated in the same patient. No TMB bias was observed between operation orders in double and multifocal cancers; in contrast, TMB was inclined to increase in metastatic foci (Fig. 2a and b). Of metastatic cancers, 63% (76/121 pairs) showed high accumulation of somatic mutations compared to the primary site, whereas an even proportion was found in double (50%, 22/44 pairs) and multifocal (52%, 30/58 pairs) cancers (Fig. 2b). These results suggest that most double and multifocal cancers were independently developed in each tissue or location, whereas somatic mutations in metastatic foci were accumulated according to tumor progression.

### Features of common mutations in paired samples

**Fig. 3:**
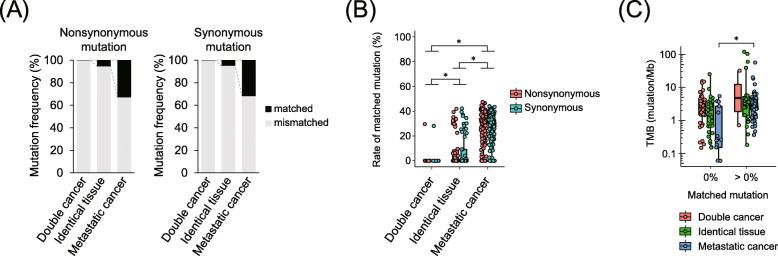
Common mutation pattern in double cancer, metastatic cancer, and multifocal lesions derived from identical tissues. **a** Frequency of matched and mismatched mutation positions in paired samples. **b** Concordance rate of nonsynonymous and synonymous mutations in each sample. **c** Comparison of tumor mutational burden (TMB) between tumors with or without matched mutations. The asterisk represents significant differences corrected with Bonferroni’s method (*p* < 0.01/3)

The frequency of common mutations between double/multifocal cancer and metastatic cancer was compared to confirm the recurrent somatic mutations in the same patient. In metastatic cancers, over 30% of the mutations were identical to those at the primary site, whereas 99.8% of the somatic mutations in double cancers were inconsistent in the same patient (Fig. 3a and Table S2). In multifocal cancers, 5% of the mutations were consistent. Although the concordance rate for nonsynonymous and synonymous mutations was significantly increased in multifocal cancer and metastatic cancer compared to double cancer (*p* < 0.001), this rate for each sample was widely distributed within 0–40% (Fig. 3b). Notably, in lesions derived from identical tissues, this concordance rate was bimodal. In all cancer types, in pairs without common mutations, TMB was significantly lower only in metastatic cancers (*p* < 0.001, Fig. 3c), suggesting that the mutational concordance rate of metastatic cancer was influenced by the number of somatic mutations in the tumor. Taken together, the inconsistency of mutations is a unique feature of double cancers, regardless of the TMB.

### Discrimination of double cancer based on Poisson distribution

**Fig. 4:**
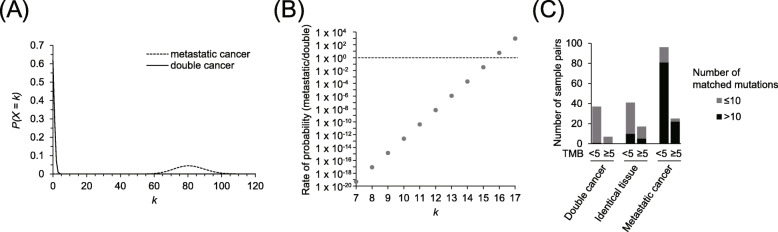
Mutational concordance analysis in double cancer, metastatic cancer, and multifocal lesions derived from identical tissues. **a** Poisson distribution of common mutation in double cancer and metastatic cancer. For Poisson distribution calculation, parameter λ is defined as the average of common mutations in paired samples. **b** Estimation of metastatic cancer with probability calculated from Poisson distribution. This rate represents an increase in the probability of metastasis when it exceeds 1.0. **c** Number of samples with more than 10 common mutations in tumor mutational burden (TMB)-low (< 5 mutations/Mb) tumors. *k*, event of common mutations occurring in paired samples

Mutation analysis of cancers derived from the same patient revealed that the somatic mutations of double cancer were independent in each individual tumor. To estimate the event probability of common mutations in double cancer, a Poisson distribution was applied to the number of somatic mutations matched in paired samples derived from the same patient. In double cancer, the probability of having > 10 common mutations was < 1.0 part-per-billion (ppb) (Fig. 4a), and this probability was 10^12^ times lower than that for metastatic cancer; however, the probabilities of double and metastatic cancer with 16 common mutations were similar (Fig. 4b). These results indicated that the origin of tumors derived from the same patient with ≤10 common mutations was disparate. To investigate the tumor origin in multifocal lesions and metastatic cancer, sample pairs with ≤10 common mutations were counted (Fig. 4c). Our analysis estimated that 74% (43/58) of the pairs had different tumor origins within identical tissues. Although the mutational concordance rate in metastatic cancers was influenced by TMB (Fig. 3c), 12% (3/25) of metastases without TMB-low features had ≤10 common mutations.

## Discussion

Based on clinicopathological findings, pathologists categorically diagnose whether tumors are double cancer or not. However, only a few studies have focused on approaches that can support these diagnoses. Recently, WES has enabled exhaustive mutation detection in more than 20,000 genes, and molecular profiling based on somatic alterations has been found to reveal the tumor characteristics. Thus, mutation analysis can discriminate double/multifocal and metastatic cancers with a different perspective compared to the clinicopathological findings. We thus analyzed the somatic mutations in paired samples derived from the same patient.

Tumor progression is considered to promote the accumulation of somatic mutations [10]. Although tumors in metastatic foci were inclined to increase the TMB, 37% of metastases were associated with a minor decrease in mutations (see Fig. 2b). Cell populations in solid tumors usually maintain mutation heterogeneity, despite harboring essential driver alterations [10, 11]. These findings raise the possibility that cell populations with particular mutations proliferate in metastatic foci. In support of this hypothesis, approximately 30% of the mutations were identical to those in the primary tumor, which is rare in the case of double cancer. We concluded that the number of common somatic mutations rather than the TMB is the intrinsic difference between double and metastatic cancers in the same patient.

Our analysis revealed that common mutations were extremely low in double cancer derived from the same patient. This discordance of somatic mutations implies that the generation of sporadic mutations was a random event in an interval of time, except for an essential driver mutation. Thus, it is reasonable to assume that the expected common mutations in double cancer follow a Poisson distribution. A difference in distribution was observed between double and metastatic cancers. In double cancers, few common mutations (average < 1.0) accounted for a greater proportion of somatic alterations, whereas almost all metastatic cancers harbored 60–100 common mutations. For the first time, these data show that discordance of somatic mutations in double cancer is higher than that in metastatic cancer derived from the same patient. Molecular profiling focusing on mutational concordance is thus a promising approach to support cancer diagnosis.

This mutational concordance analysis is characterized by discriminating double cancer based on the number of concordant mutations, regardless of TMB. For instance, the probability of double cancer with more than 5 and 10 mutations was 0.013% and ≤ 1 ppb, respectively. In our samples, 96.9 and 87.9% of the tumors harbored ≥5 and ≥ 10 mutations, respectively; thus, mutational concordance analysis can facilitate high discrimination of double cancer for most cancers.

In our analysis, one patient with double cancer showed high concordance with somatic mutations. Based on the probability estimated by Poisson distribution (9.59 × 10^− 24^% in *k* = 20), this pair case (derived from the ischiadic nerve and thigh) is unlikely to be double cancer. Additionally, three metastatic cases with intermediate/high TMB (≥5 mutations/Mb) and few common mutations were identified. These cases may include tumors with different tumor origins based on the mutational concordance. To clarify whether these were double cancers or not, these cases should be clinicopathologically verified in further studies.

The mutational concordance rate of multifocal cancers was bimodal and often included samples with a few common mutations, suggesting the presence of tumors generated from different tumor origins in identical tissues from the same patient. Various reports have recently revealed that the pattern of mutation accumulation differs in normal tissues within identical tissues [12–15]. This diversity can underlie cancer heterogeneity and may affect the mutation concordance rate within identical tissues. Enumeration of somatic mutations using WES allows us to distinguish between tumors with the same tumor origin and those without. In clinicopathological diagnosis, it is sometimes difficult to distinguish between multiple cancers, the primary lesion, and its metastatic lesions based on histological images [16]. Our analysis would thus be useful in the diagnosis of lesions in identical tissues, though multifocal cancers need to be validated in more cases.

## Conclusions

The present study investigated the molecular profiling of tumors derived from the same patient to distinguish between double and metastatic cancers using next-generation sequencing. The TMB evaluated using WES tended to increase in metastatic foci. Somatic mutations in double cancer were independently generated in each tissue, leading to most mutational discrepancies. Mutational concordance analysis based on Poisson distribution helped distinguish several common mutations between double and metastatic cancers. Furthermore, our approach could estimate multifocal cancers with different tumor origins in identical tissues. Thus, mutational concordance analysis is useful for understanding the tendency of mutation accumulation within tumors in the same patient; it is also a promising approach for supporting clinical cancer diagnosis.

## Supplementary Information

**Additional file 1: Table S1.** Sample list used in the main analysis.

**Additional file 2: Table S2.** Common somatic mutations in paired samples from the same patient.

## Data Availability

Although the WES data were submitted to the NBDC as ‘Controlled-Access Data’ (Research ID, hum0127.v1; https://humandbs.biosciencedbc.jp/en/), parts of the datasets generated and/or analyzed during the current study are not available publicly, but are available from the corresponding author on reasonable request.

## Abbreviations

WES: whole exome sequencing
TMB: tumor mutational burden
