# Supplementary material for: Mutational concordance analysis provides supportive information for double cancer diagnosis
Source: BMC Cancer. 2021 Feb 19;21:181. doi: 10.1186/s12885-021-07899-1 (PMC7893960; doi:10.1186/s12885-021-07899-1)
Supplement: Supplementary file 1 — Additional file 1: Table S1. Sample list used in the main analysis. [file 12885_2021_7899_MOESM1_ESM.pdf]

Mutational concordance analysis provides supportive information for double cancer diagnosis

Keiichi Hatakeyama, Takeshi Nagashima, Akifumi Notsu, Keiichi Ohshima, Sumiko Ohnami, Shumpei Ohnami, Yuji Shimoda, Akane Naruoka, Koji Maruyama, Akira Iizuka, Tadashi Ashizawa, Hirotsugu Kenmotsu, Tohru Mochizuki, Kenichi Urakami, Yasuto Akiyama and Ken Yamaguchi

Table S1 Sample list used in main analysis

						Number of total mutation		Number of common mutation	
ID	Operation date (yyyy/mm/dd)	Cancer type	Status	Tissue	TMB	Nonsynonymous	Synonymous	Nonsynonymous	Synonymous
SCC1	2017/11/1	double_cancer	primary1	colon (ascending)	3.28096	71	40	0	0
SCC2	2017/11/1	double_cancer	primary2	stomach	0.537253	12	6	0	0
SCC3	2015/2/5	double_cancer	primary1	colon (transverse)	2.30388	44	32	0	0
SCC4	2015/6/23	double_cancer	primary2	lung	8.58338	200	78	0	0
SCC5	2016/8/8	double_cancer	primary1	colon (sigmoid)	5.6328	132	55	0	0
SCC6	2016/9/16	double_cancer	primary2	lung	4.14955	95	40	0	0
SCC7	2015/3/27	double_cancer	primary1	colon (rectum)	3.66429	90	32	48	48
SCC8	2017/3/1	double_cancer	primary2	pancreas	2.90752	68	31	48	48
SCC9	2017/11/27	double_cancer	primary1	esophagus	15.0431	314	195	0	0
SCC10	2017/3/1	double_cancer	primary2	head&neck (gingiva)	1.84365	41	21	0	0
SCC11	2014/8/25	double_cancer	primary1	stomach	0.215523	6	1	0	0
SCC12	2014/10/6	double_cancer	primary2	lung	1.49947	29	19	0	0
SCC13	2015/1/26	double_cancer	primary1	pancreas	2.05136	49	20	0	0
SCC14	2015/4/20	double_cancer	primary2	lung	5.6196	131	58	0	0
SCC15	2015/1/19	double_cancer	primary1	liver	7.23138	157	88	0	0
SCC16	2015/6/17	double_cancer	primary2	colon (rectum)	1.16399	29	10	0	0
SCC17	2015/11/17	double_cancer	primary1	lung	0.150929	2	3	0	0
SCC18	2015/11/17	double_cancer	primary2	esophagus	2.0341	41	26	0	0
SCC19	2017/11/15	double_cancer	primary1	stomach	5.53707	136	50	0	0
SCC20	2017/11/15	double_cancer	primary2	breast	0.954303	23	9	0	0
SCC21	2014/7/14	double_cancer	primary1	lung	13.7398	291	137	0	0
SCC22	2014/10/1	double_cancer	primary2	stomach	11.4199	254	131	0	0
SCC23	2015/6/5	double_cancer	primary1	soft tissue (ischiadic nerve)	0.653755	15	7	13	13
SCC24	2015/7/24	double_cancer	primary2	soft tissue (thigh)	1.39292	30	16	13	13
SCC25	2016/9/20	double_cancer	primary1	colon (sigmoid)	5.22963	124	51	0	0
SCC26	2016/10/27	double_cancer	primary2	stomach	0.215582	4	3	0	0
SCC27	2014/10/24	double_cancer	primary1	stomach	4.47901	94	50	0	0
SCC28	2015/1/6	double_cancer	primary2	lung	1.49676	34	15	0	0
SCC29	2017/3/1	double_cancer	primary1	stomach	1.57544	33	20	0	0
SCC30	2017/4/26	double_cancer	primary2	lung	3.29333	79	32	0	0
SCC31	2014/8/19	double_cancer	primary1	uterus (corpus)	2.15945	49	20	0	0
SCC32	2015/6/10	double_cancer	primary2	liver	2.94873	61	37	0	0
SCC33	2014/4/2	double_cancer	primary1	colon (transverse)	2.48553	46	27	0	0
SCC34	2014/5/23	double_cancer	primary2	liver	3.95622	77	45	0	0
SCC35	2017/10/23	double_cancer	primary1	colon (rectum)	1.77792	36	24	0	0
SCC36	2017/12/21	double_cancer	primary2	lung	3.44168	77	39	0	0
SCC37	2016/4/13	double_cancer	primary1	breast	1.46245	34	15	0	0
SCC38	2016/7/13	double_cancer	primary2	lung	1.49424	32	18	0	0
SCC39	2014/7/17	double_cancer	primary1	colon (cecum)	1.90866	36	23	0	0
SCC40	2014/9/1	double_cancer	primary2	liver	2.7175	58	30	0	0
SCC41	2016/9/13	double_cancer	primary1	breast	0.728738	18	6	0	0
SCC42	2016/11/9	double_cancer	primary2	lung	0.772907	19	7	0	0
SCC43	2017/3/3	double_cancer	primary1	stomach	8.26416	170	110	0	0
SCC44	2017/3/3	double_cancer	primary2	colon (transverse)	1.29508	22	22	0	0
SCC45	2015/5/25	double_cancer	primary1	colon (rectum)	2.58731	56	30	0	0
SCC46	2016/2/5	double_cancer	primary2	lung	0.794343	17	9	0	0
SCC47	2014/10/27	double_cancer	primary1	liver	3.17096	66	35	0	0
SCC48	2014/12/15	double_cancer	primary2	lung	0.470831	9	6	0	0
SCC49	2017/5/16	double_cancer	primary1	esophagus	2.3727	51	29	0	0
SCC50	2017/5/16	double_cancer	primary2	colon (sigmoid)	2.47411	53	30	0	0
SCC51	2014/11/4	double_cancer	primary1	esophagus	4.85995	105	50	0	0
SCC52	2014/11/4	double_cancer	primary2	stomach	0.151955	2	3	0	0
SCC53	2017/6/14	double_cancer	primary1	stomach	0.716991	14	10	0	0
SCC54	2017/6/14	double_cancer	primary2	colon (descending)	1.99523	40	27	0	0
SCC55	2014/7/23	double_cancer	primary1	lung	3.69696	77	37	0	0
SCC56	2014/9/11	double_cancer	primary2	head&neck (parotid gland)	2.33376	51	25	0	0
SCC57	2015/9/24	double_cancer	primary1	stomach	2.86292	57	36	0	0
SCC58	2015/11/18	double_cancer	primary2	head&neck (hypopharynx)	7.51301	172	76	0	0
SCC59	2016/3/31	double_cancer	primary1	stomach	31.944	725	351	1	1
SCC60	2016/3/31	double_cancer	primary2	colon (cecum)	26.3009	560	329	1	1
SCC61	2015/1/5	double_cancer	primary1	colon (transverse)	3.88636	88	43	0	0
SCC62	2015/3/17	double_cancer	primary2	lung	10.6087	238	112	0	0
SCC63	2014/4/15	double_cancer	primary1	colon (ascending)	0.094942	1	2	0	0
SCC64	2014/5/26	double_cancer	primary2	liver	1.54236	31	16	0	0
SCC65	2015/11/26	double_cancer	primary1	pancreas	0.337674	8	3	0	0
SCC66	2016/6/22	double_cancer	primary2	mediastinum	0.627258	14	7	0	0
SCC67	2014/2/13	double_cancer	primary1	skin	0.629192	10	8	0	0
SCC68	2014/2/13	double_cancer	primary2	unknown	0.190859	2	4	0	0
SCC69	2018/1/25	double_cancer	primary1	colon (ascending)	3.11817	80	26	0	0
SCC70	2018/1/25	double_cancer	primary2	stomach	1.30114	27	17	0	0
SCC71	2015/2/25	double_cancer	primary1	stomach	0.84467	23	5	0	0
SCC72	2015/2/25	double_cancer	primary2	colon (rectum)	1.96225	38	26	0	0
SCC73	2014/3/10	double_cancer	primary1	liver	2.80129	59	28	0	0
SCC74	2016/10/7	double_cancer	primary2	colon (cecum)	4.02391	94	40	0	0
SCC75	2017/6/12	double_cancer	primary1	stomach	0.177749	3	3	0	0
SCC76	2017/6/12	double_cancer	primary2	small intestine	0.266267	6	3	0	0
SCC77	2015/10/20	double_cancer	primary1	esophagus	1.5086	30	21	0	0
SCC78	2015/10/20	double_cancer	primary2	stomach	0.181869	5	1	0	0
SCC79	2016/6/7	double_cancer	primary1	colon (sigmoid)	4.18437	92	45	0	0
SCC80	2016/7/25	double_cancer	primary2	lung	66.0803	1501	701	0	0
SCC81	2014/2/13	double_cancer	primary1	lung	2.35163	53	21	0	0
SCC82	2014/4/7	double_cancer	primary2	head&neck (mouth)	2.20553	42	29	0	0
SCC83	2014/6/2	double_cancer	primary1	liver	6.71123	141	65	0	0
SCC84	2015/2/2	double_cancer	primary2	lung	0.029563	1	0	0	0
SCC85	2015/4/6	double_cancer	primary1	lung	15.1172	333	172	0	0
SCC86	2016/7/21	double_cancer	primary2	stomach	4.0134	84	50	0	0
SCC87	2015/11/18	primary	primary	colon (transverse)	6.35789	121	88	110	81
SCC88	2016/1/12	metastasis	metastasis	liver	6.56671	125	95	110	81
SCC89	2015/3/26	primary	primary	colon (ascending)	1.17517	26	13	22	10
SCC90	2015/6/29	metastasis	metastasis	liver	4.47816	97	48	22	10
SCC91	2016/3/16	primary	primary	colon (rectum)	3.67472	81	43	63	24
SCC92	2016/5/12	metastasis	metastasis	liver	3.26902	75	31	63	24
SCC93	2017/2/10	primary	primary	colon (rectum)	3.49397	83	36	47	21
SCC94	2018/1/5	metastasis	metastasis	lung	2.51926	56	29	47	21
SCC95	2015/2/20	primary	primary	colon (sigmoid)	3.33043	67	44	45	30

SCC96	2015/4/20	metastasis	metastasis	liver	2.86775	59	37	45	30
SCC97	2014/11/25	primary	primary	lung	0.154092	3	2	0	0
SCC98	2016/12/22	metastasis	metastasis	brain (cerebrum)	5.01171	114	54	0	0
SCC99	2017/10/30	primary	primary	colon (sigmoid)	1.9314	44	21	23	12
SCC100	2017/12/14	metastasis	metastasis	lung	4.42702	92	57	23	12
SCC101	2015/3/10	primary	primary	lung	1.70543	38	18	0	0
SCC102	2016/8/10	metastasis	metastasis	lung	0.365346	10	2	0	0
SCC103	2015/3/24	primary	primary	skin (face)	18.4795	326	268	151	123
SCC104	2015/3/24	metastasis	metastasis	lymph node	8.83414	158	135	151	123
SCC105	2016/3/28	primary	primary	colon (rectum)	3.49593	81	38	63	19
SCC106	2017/9/25	metastasis	metastasis	lung (rt_S10)	4.98466	115	50	63	19
SCC107	2017/11/6	primary	primary	colon (rectum)	3.5551	81	37	54	23
SCC108	2017/12/21	metastasis	metastasis	liver	3.18747	68	39	54	23
SCC109	2014/8/7	primary	primary	colon (rectum)	2.9611	65	30	59	23
SCC110	2014/9/22	metastasis	metastasis	liver	2.74868	63	26	59	23
SCC111	2015/12/4	primary	primary	colon (sigmoid)	2.6656	56	32	0	0
SCC112	2016/2/1	metastasis	metastasis	liver	0.648341	4	18	0	0
SCC113	2017/2/3	primary	primary	colon (rectum)	2.46059	53	30	47	26
SCC114	2017/3/21	metastasis	metastasis	liver	2.97097	64	35	47	26
SCC115	2017/10/26	primary	primary	colon (sigmoid)	2.4229	52	30	35	17
SCC116	2017/9/4	metastasis	metastasis	lung	2.42953	56	25	35	17
SCC117	2017/6/30	primary	primary	stomach	2.80764	49	45	32	24
SCC118	2017/6/30	metastasis	metastasis	liver	2.56302	50	35	32	24
SCC119	2017/6/7	primary	primary	colon (ascending)	1.9946	46	21	35	20
SCC120	2017/7/13	metastasis	metastasis	liver	3.52054	76	43	35	20
SCC121	2014/4/16	primary	primary	colon (sigmoid)	0.93377	17	13	15	10
SCC122	2014/5/7	metastasis	metastasis	liver	3.08961	58	36	15	10
SCC123	2017/6/27	primary	primary	colon (ascending)	4.75561	94	64	78	54
SCC124	2017/8/14	metastasis	metastasis	liver	5.11883	101	71	78	54
SCC125	2015/4/7	primary	primary	liver	1.28596	27	16	25	12
SCC126	2017/6/21	metastasis	metastasis	lung	3.81407	91	36	25	12
SCC127	2016/9/15	primary	primary	colon (rectum)	0.928971	18	13	14	8
SCC128	2016/11/14	metastasis	metastasis	liver	5.70616	125	65	14	8
SCC129	2015/6/17	primary	primary	colon (sigmoid)	4.30269	98	47	21	9
SCC130	2016/4/11	metastasis	metastasis	liver	2.06192	48	22	21	9
SCC131	2016/10/28	primary	primary	colon (rectum)	1.99247	44	23	34	17
SCC132	2016/12/14	metastasis	metastasis	liver	5.74558	131	61	34	17
SCC133	2017/3/7	primary	primary	esophagus	2.24091	48	27	26	15
SCC134	2017/10/3	metastasis	metastasis	brain (cerebrum)	3.09296	65	35	26	15
SCC135	2015/2/20	primary	primary	colon (rectum)	3.19747	65	40	20	13
SCC136	2017/1/26	metastasis	metastasis	lung	2.03134	38	30	20	13
SCC137	2014/9/3	primary	primary	lung	12.4003	276	130	265	128
SCC138	2016/6/7	metastasis	metastasis	brain (cerebrum)	16.3701	362	185	265	128
SCC139	2015/1/28	primary	primary	colon (rectum)	0.08953	2	1	0	0
SCC140	2015/9/9	metastasis	metastasis	liver	5.52514	123	58	0	0
SCC141	2017/2/14	primary	primary	stomach	1.4244	28	20	23	10
SCC142	2017/12/13	metastasis	metastasis	peritoneum	1.23766	26	16	23	10
SCC143	2016/4/13	primary	primary	pancreas	0.935662	21	10	13	5
SCC144	2016/4/13	metastasis	metastasis	liver	1.0287	24	10	13	5
SCC145	2017/2/2	primary	primary	colon (ascending)	3.87532	84	48	53	24
SCC146	2017/3/27	metastasis	metastasis	liver	3.22468	74	34	53	24
SCC147	2014/7/28	primary	primary	stomach	56.0475	1125	632	1046	578
SCC148	2016/1/27	metastasis	metastasis	liver	68.5604	1473	834	1046	578
SCC149	2017/9/12	primary	primary	colon (cecum)	3.42236	73	41	39	22
SCC150	2017/10/30	metastasis	metastasis	liver	3.62047	76	45	39	22
SCC151	2017/8/25	primary	primary	colon (descending)	5.04885	116	54	51	23
SCC152	2017/10/30	metastasis	metastasis	liver	3.76361	88	38	51	23
SCC153	2014/11/19	primary	primary	colon (rectum)	2.2124	54	19	39	13
SCC154	2015/5/7	metastasis	metastasis	liver	1.97668	42	24	39	13
SCC155	2014/10/21	primary	primary	colon (rectum)	3.42875	76	35	61	26
SCC156	2014/12/22	metastasis	metastasis	liver	3.69309	83	37	61	26
SCC157	2014/11/21	primary	primary	stomach	6.35027	130	70	46	25
SCC158	2016/2/1	metastasis	metastasis	liver	6.71294	153	71	46	25
SCC159	2015/4/22	primary	primary	colon (sigmoid)	3.39976	76	35	54	24
SCC160	2016/1/7	metastasis	metastasis	liver	3.99433	82	52	54	24
SCC161	2016/11/7	primary	primary	colon (rectum)	2.9399	71	28	53	25
SCC162	2017/1/30	metastasis	metastasis	liver	2.56218	57	30	53	25
SCC163	2017/11/15	primary	primary	colon (rectum)	2.79219	62	32	38	19
SCC164	2018/1/15	metastasis	metastasis	liver	3.34627	80	33	38	19
SCC165	2014/8/22	primary	primary	lung	3.07476	60	37	37	19
SCC166	2016/7/12	metastasis	metastasis	brain (cerebrum)	3.57324	78	41	37	19
SCC167	2015/6/8	primary	primary	colon (sigmoid)	2.41629	56	23	30	17
SCC168	2015/6/8	metastasis	metastasis	liver	2.03555	41	27	30	17
SCC169	2017/4/7	primary	primary	colon (descending)	2.2358	47	29	34	25
SCC170	2017/6/23	metastasis	metastasis	liver	3.29933	63	48	34	25
SCC171	2017/4/4	primary	primary	skin (thigh)	0.264405	3	6	0	0
SCC172	2017/4/4	metastasis	metastasis	lymph node	0.83021	15	13	0	0
SCC173	2016/3/30	primary	primary	colon (ascending)	3.41859	70	44	55	29
SCC174	2016/5/16	metastasis	metastasis	liver	3.16286	68	38	55	29
SCC175	2015/1/30	primary	primary	colon (rectum)	3.20344	58	48	45	39
SCC176	2016/2/22	metastasis	metastasis	liver	4.80561	97	65	45	39
SCC177	2016/8/18	primary	primary	colon (sigmoid)	0.303213	7	3	2	0
SCC178	2016/9/29	metastasis	metastasis	liver	2.52948	57	26	2	0
SCC179	2015/2/16	primary	primary	stomach	4.11791	100	33	81	25
SCC180	2015/2/16	metastasis	metastasis	liver	4.70373	109	45	81	25
SCC181	2015/9/14	primary	primary	colon (rectum)	0.060417	2	0	0	0
SCC182	2016/5/12	metastasis	metastasis	liver	3.38099	71	40	0	0
SCC183	2015/12/22	primary	primary	colon (rectum)	2.70719	61	31	50	20
SCC184	2016/2/29	metastasis	metastasis	liver	3.05874	72	31	50	20
SCC185	2017/3/10	primary	primary	colon (cecum)	2.68613	55	36	39	19
SCC186	2017/5/24	metastasis	metastasis	liver	3.35732	71	42	39	19
SCC187	2016/4/8	primary	primary	colon (sigmoid)	1.732	34	24	28	16
SCC188	2016/5/19	metastasis	metastasis	liver	6.8701	158	68	28	16
SCC189	2015/9/17	primary	primary	colon (rectum)	6.69629	150	72	8	3
SCC190	2015/11/11	metastasis	metastasis	liver	3.9708	86	45	8	3
SCC191	2014/2/21	primary	primary	soft tissue (foot)	1.43492	29	16	24	9
SCC192	2014/8/15	metastasis	metastasis	lung	1.42189	31	14	24	9
SCC193	2017/7/6	primary	primary	colon (rectum)	3.19767	66	42	38	33

SCC194	2017/9/4	metastasis	metastasis	liver	3.36761	60	52	38	33
SCC195	2014/10/9	primary	primary	colon (rectum)	2.14764	41	27	38	21
SCC196	2016/6/20	metastasis	metastasis	liver	2.94949	64	34	38	21
SCC197	2015/8/11	primary	primary	soft tissue (abdominal wall)	0.153357	3	2	0	0
SCC198	2017/8/31	metastasis	metastasis	lung	1.33133	33	12	0	0
SCC199	2016/5/13	primary	primary	colon (ascending)	2.79185	54	38	33	21
SCC200	2016/6/30	metastasis	metastasis	liver	3.82813	81	43	33	21
SCC201	2015/2/19	primary	primary	colon (rectum)	2.41417	54	24	48	21
SCC202	2015/5/18	metastasis	metastasis	liver	4.74458	101	54	48	21
SCC203	2016/1/29	primary	primary	colon (rectum)	3.1684	71	36	59	30
SCC204	2017/2/6	metastasis	metastasis	liver	5.08089	120	53	59	30
SCC205	2017/4/3	primary	primary	colon (rectum)	2.49678	57	28	41	21
SCC206	2017/6/7	metastasis	metastasis	liver	2.97521	65	34	41	21
SCC207	2014/7/9	primary	primary	colon (cecum)	2.25824	48	24	26	16
SCC208	2015/3/24	metastasis	metastasis	liver	2.04166	40	27	26	16
SCC209	2014/8/19	primary	primary	liver	0.030573	0	1	0	0
SCC210	2015/2/2	metastasis	metastasis	spleen	5.1126	113	58	0	0
SCC211	2016/2/26	primary	primary	colon (sigmoid)	4.11854	90	49	68	37
SCC212	2016/4/28	metastasis	metastasis	liver	3.95925	82	47	68	37
SCC213	2016/12/16	primary	primary	colon (rectum)	2.78225	64	30	42	20
SCC214	2017/12/6	metastasis	metastasis	liver	2.33014	55	24	42	20
SCC215	2017/1/5	primary	primary	colon (rectum)	3.25316	72	38	46	22
SCC216	2017/8/30	metastasis	metastasis	liver	3.00836	65	37	46	22
SCC217	2016/8/26	primary	primary	colon (cecum)	0.216348	5	2	0	0
SCC218	2016/10/5	metastasis	metastasis	liver	2.44891	49	31	0	0
SCC219	2016/6/16	primary	primary	colon (rectum)	4.25312	97	42	58	24
SCC220	2018/1/22	metastasis	metastasis	liver	4.14305	96	46	58	24
SCC221	2017/6/1	primary	primary	colon (sigmoid)	3.11777	62	44	45	28
SCC222	2017/8/1	metastasis	metastasis	liver	3.05073	66	37	45	28
SCC223	2015/6/15	primary	primary	colon (ascending)	4.36865	96	51	77	40
SCC224	2016/8/15	metastasis	metastasis	lung	4.45646	96	53	77	40
SCC225	2014/8/20	primary	primary	colon (sigmoid)	3.61697	73	44	60	40
SCC226	2015/2/4	metastasis	metastasis	lung	3.97337	85	49	60	40
SCC227	2016/7/13	primary	primary	colon (rectum)	4.72654	99	59	47	32
SCC228	2016/9/1	metastasis	metastasis	liver	3.50048	69	45	47	32
SCC229	2014/4/17	primary	primary	lung	0.465342	7	8	3	2
SCC230	2016/2/15	metastasis	metastasis	lung	0.772472	16	10	3	2
SCC231	2015/10/7	primary	primary	thymus	1.14073	27	11	13	5
SCC232	2016/10/20	metastasis	metastasis	peritoneum	1.0585	21	14	13	5
SCC233	2016/11/11	primary	primary	colon (sigmoid)	3.67316	89	34	67	21
SCC234	2016/12/19	metastasis	metastasis	liver	4.10306	102	35	67	21
SCC235	2017/3/22	primary	primary	colon (ascending)	2.95935	65	33	37	16
SCC236	2017/5/1	metastasis	metastasis	liver	4.52616	98	56	37	16
SCC237	2016/2/26	primary	primary	colon (rectum)	2.97427	67	32	34	15
SCC238	2016/4/19	metastasis	metastasis	liver	2.1077	46	25	34	15
SCC239	2015/7/9	primary	primary	stomach	0.640632	13	8	11	3
SCC240	2015/7/9	metastasis	metastasis	liver	3.27629	77	31	11	3
SCC241	2015/10/14	primary	primary	colon (sigmoid)	4.28698	96	46	89	42
SCC242	2015/12/17	metastasis	metastasis	liver	5.12421	115	59	89	42
SCC243	2016/9/15	primary	primary	lung	6.20469	141	64	128	54
SCC244	2018/1/12	metastasis	metastasis	brain (cerebrum)	7.439	170	78	128	54
SCC245	2016/2/15	primary	primary	colon (rectum)	3.46388	73	44	51	28
SCC246	2016/3/18	metastasis	metastasis	liver	3.37903	75	40	51	28
SCC247	2015/1/7	primary	primary	colon (descending)	3.74063	82	41	75	34
SCC248	2015/1/7	metastasis	metastasis	liver	3.63237	78	42	75	34
SCC249	2014/5/30	primary	primary	colon (rectum)	1.5344	34	11	29	9
SCC250	2014/7/31	metastasis	metastasis	liver	4.22762	86	44	29	9
SCC251	2014/3/28	primary	primary	colon (rectum)	4.82063	95	58	68	42
SCC252	2016/6/2	metastasis	metastasis	liver	5.89408	120	70	68	42
SCC253	2014/6/4	primary	primary	colon (rectum)	2.42539	47	25	39	22
SCC254	2015/1/29	metastasis	metastasis	lung	2.65613	55	33	39	22
SCC255	2014/3/17	primary	primary	colon (cecum)	1.98457	40	20	33	16
SCC256	2014/4/21	metastasis	metastasis	liver	2.93087	57	36	33	16
SCC257	2016/12/1	primary	primary	colon (rectum)	3.24931	73	36	62	30
SCC258	2017/1/23	metastasis	metastasis	liver	3.3212	76	37	62	30
SCC259	2014/5/28	primary	primary	breast	2.77925	58	27	49	22
SCC260	2016/12/27	metastasis	metastasis	brain (cerebrum)	6.20047	130	75	49	22
SCC261	2015/4/10	primary	primary	colon (rectum)	1.45574	29	18	21	10
SCC262	2015/6/1	metastasis	metastasis	lung	1.33216	30	14	21	10
SCC263	2017/2/20	primary	primary	colon (sigmoid)	5.35468	126	56	96	47
SCC264	2017/10/23	metastasis	metastasis	liver	6.4833	137	81	96	47
SCC265	2017/8/16	primary	primary	colon (sigmoid)	2.46644	58	25	46	19
SCC266	2017/9/28	metastasis	metastasis	liver	2.60607	54	33	46	19
SCC267	2016/2/4	primary	primary	colon (sigmoid)	3.86879	85	44	70	34
SCC268	2016/4/11	metastasis	metastasis	liver	5.12651	109	64	70	34
SCC269	2015/8/3	primary	primary	colon (anal canal)	3.87711	86	40	59	30
SCC270	2015/10/5	metastasis	metastasis	liver	3.82227	80	44	59	30
SCC271	2017/11/7	primary	primary	colon (rectum)	2.64225	59	27	49	24
SCC272	2017/12/20	metastasis	metastasis	liver	2.68843	59	32	49	24
SCC273	2015/9/24	primary	primary	colon (sigmoid)	3.90065	81	49	63	36
SCC274	2015/11/11	metastasis	metastasis	liver	5.43725	115	63	63	36
SCC275	2015/9/7	primary	primary	colon (rectum)	3.34239	72	36	3	1
SCC276	2016/1/25	metastasis	metastasis	liver	0.89454	21	9	3	1
SCC277	2016/2/9	primary	primary	duodenum	0.840186	16	12	2	2
SCC278	2016/2/9	metastasis	metastasis	liver	1.03479	22	13	2	2
SCC279	2016/11/28	primary	primary	lung	20.1906	452	225	417	187
SCC280	2017/6/5	metastasis	metastasis	stomach	19.4485	446	205	417	187
SCC281	2017/9/6	primary	primary	colon (cecum)	3.31015	75	37	60	27
SCC282	2017/10/4	metastasis	metastasis	liver	4.71177	107	51	60	27
SCC283	2015/9/11	primary	primary	stomach	2.65697	54	34	12	5
SCC284	2015/9/11	metastasis	metastasis	liver	1.09006	21	14	12	5
SCC285	2016/12/22	primary	primary	colon (rectum)	5.01511	116	53	44	17
SCC286	2017/2/17	metastasis	metastasis	liver	5.12827	118	55	44	17
SCC287	2017/4/28	primary	primary	colon (cecum)	3.93948	83	50	14	10
SCC288	2017/6/16	metastasis	metastasis	liver	4.52423	82	70	14	10
SCC289	2017/5/29	primary	primary	colon (rectum)	3.37581	74	40	52	28
SCC290	2017/7/26	metastasis	metastasis	liver	3.72471	77	48	52	28
SCC291	2017/11/9	primary	primary	colon (ascending)	1.58657	33	20	15	10

SCC292	2018/2/1	metastasis	metastasis	liver	4.52757	92	57	15	10
SCC293	2014/2/10	primary	primary	colon (cecum)	2.83917	52	36	44	25
SCC294	2014/2/10	metastasis	metastasis	liver	3.54591	68	46	44	25
SCC295	2017/6/7	primary	primary	colon (sigmoid)	2.95384	72	28	62	27
SCC296	2017/7/14	metastasis	metastasis	liver	4.06508	88	47	62	27
SCC297	2015/6/16	primary	primary	lung	2.42638	50	32	39	23
SCC298	2016/8/16	metastasis	metastasis	brain (cerebrum)	2.53988	54	28	39	23
SCC299	2014/7/22	primary	primary	pancreas	0.302593	4	5	0	0
SCC300	2015/8/12	metastasis	metastasis	lung	1.12807	22	15	0	0
SCC301	2014/12/26	primary	primary	colon (ascending)	4.44371	94	44	79	31
SCC302	2015/3/3	metastasis	metastasis	liver	4.00443	95	39	79	31
SCC303	2016/7/22	primary	primary	liver	5.46896	117	65	0	0
SCC304	2016/7/22	metastasis	metastasis	liver	2.63288	64	24	0	0
SCC305	2015/2/13	primary	primary	colon (sigmoid)	3.07889	68	31	58	29
SCC306	2015/4/1	metastasis	metastasis	liver	3.61155	77	42	58	29
SCC307	2016/3/8	primary	primary	colon (sigmoid)	2.4598	60	23	34	15
SCC308	2016/4/18	metastasis	metastasis	liver	2.55735	55	31	34	15
