# Supplementary material for: Mutational concordance analysis provides supportive information for double cancer diagnosis
Source: BMC Cancer. 2021 Feb 19;21:181. doi: 10.1186/s12885-021-07899-1 (PMC7893960; doi:10.1186/s12885-021-07899-1)
Supplement: Supplementary file 2 — Additional file 2: Table S2. Common somatic mutations in paired samples from the same patient. [file 12885_2021_7899_MOESM2_ESM.pdf]

Mutational concordance analysis provides supportive information for double cancer diagnosis

Keiichi Hatakeyama, Takeshi Nagashima, Akifumi Notsu, Keiichi Ohshima, Sumiko Ohnami, Shumpei Ohnami, Yuji Shimoda, Akane Naruoka, Koji Maruyama, Akira Iizuka, Tadashi Ashizawa, Hirotsugu Kenmotsu, Tohru Mochizuki, Kenichi Urakami, Yasuto Akiyama and Ken Yamaguchi

Table S2 Somatic mutation list identified in this study

Gene symbol	chromosome	Position/Ref//Var	mutation type	double cancer	metastasis
KRAS	chr12	25398284/C//A	nonsynonymous	4	16
KRAS	chr12	25398284/C//T	nonsynonymous	0	20
KRAS	chr12	25398285/C//A	nonsynonymous	4	11
APC	chr5	112175639/C//T	nonsynonymous	2	11
PIK3CA	chr3	178936091/G//A	nonsynonymous	3	10
TP53	chr17	7577120/C//T	nonsynonymous	2	11
KRAS	chr12	25398281/C//T	nonsynonymous	2	8
CDC27	chr17	45266610/T//G	synonymous	1	9
APC	chr5	112173917/C//T	nonsynonymous	1	8
TP53	chr17	7578212/G//A	nonsynonymous	1	8
APC	chr5	112175211/TAAAAG//T	nonsynonymous	0	9
APC	chr5	112175348/G//T	nonsynonymous	2	6
KRAS	chr12	25398285/C//T	nonsynonymous	0	8
TP53	chr17	7577538/C//T	nonsynonymous	1	6
TP53	chr17	7577539/G//A	nonsynonymous	0	7
TP53	chr17	7577548/C//T	nonsynonymous	2	5
PCBP1	chr2	70315174/T//A	nonsynonymous	0	6
APC	chr5	112151204/C//T	nonsynonymous	2	4
TP53	chr17	7577094/G//A	nonsynonymous	0	6
CDC27	chr17	45214544/T//C	synonymous	0	6
CDC27	chr17	45219255/A//G	synonymous	0	6
CDC27	chr17	45219276/A//G	synonymous	0	6
POTEC	chr18	14543057/T//G	nonsynonymous	1	5
PLEC	chr8	144995427/A//G	synonymous	0	6
APC	chr5	112128143/C//T	nonsynonymous	0	6
HIST1H2AE	chr6	26217526/C//T	synonymous	2	4
TP53	chr17	7574003/G//A	nonsynonymous	0	6
TP53	chr17	7577121/G//A	nonsynonymous	3	2
PIK3CA	chr3	178936082/G//A	nonsynonymous	1	4
BAGE3	chr21	11049592/C//T	synonymous	0	5
CDC27	chr17	45214582/A//G	synonymous	0	5
FBXW7	chr4	153249385/G//A	nonsynonymous	1	4
APC	chr5	112174631/C//T	nonsynonymous	1	4
PLEC	chr8	144995388/T//C	synonymous	0	5
PLEC	chr8	144995396/G//C	nonsynonymous	0	5
PLEC	chr8	144995466/G//A	synonymous	0	5
TPSD1	chr16	1306918/C//T	synonymous	1	4
ITIH2	chr10	7776987/C//T	synonymous	0	4
CDC27	chr17	45266571/T//A	synonymous	0	4
APC	chr5	112175426/G//T	nonsynonymous	0	4
	chr1	77042635/C//G	synonymous	0	4
HCFC1	chrX	153220993/G//T	nonsynonymous	0	4
APC	chr5	112175255/G//T	nonsynonymous	0	4
OBSCN	chr1	228504627/C//T	synonymous	0	4
OR2M5	chr1	248309230/C//T	nonsynonymous	0	4
FIGN	chr2	164467533/G//A	nonsynonymous	0	4
TMEM132C	chr12	129028565/C//T	synonymous	0	4
SOX9	chr17	70119791/C//T	nonsynonymous	0	4
NXF5	chrX	101096868/C//T	synonymous	0	4
TP53	chr17	7576897/G//A	nonsynonymous	0	4
FBXW7	chr4	153245446/G//A	nonsynonymous	0	4
COL14A1	chr8	121238908/C//T	nonsynonymous	0	4
CNTNAP2	chr7	147675028/G//A	nonsynonymous	0	4
INF2	chr14	105176511/T//C	nonsynonymous	3	1
KDM6B	chr17	7750989/G//C	synonymous	0	4
GLRB	chr4	158065029/C//T	synonymous	0	4
ANO3	chr11	26484617/C//T	synonymous	0	4
BAGE3	chr21	11049584/C//T	nonsynonymous	0	4
TP53	chr17	7578190/T//C	nonsynonymous	1	3
KCNJ10	chr1	160011923/G//A	nonsynonymous	0	4
TGIF1	chr18	3457672/CCTTT//C	nonsynonymous	0	4
QTRT2	chr3	113804718/G//T	nonsynonymous	0	4
APC	chr5	112151184/A//G	synonymous	0	4
APC	chr5	112151261/C//T	nonsynonymous	0	4
PCDHB7	chr5	140554081/C//T	synonymous	0	4
TP53	chr17	7578263/G//A	nonsynonymous	1	3
TPSD1	chr16	1306921/G//A	synonymous	1	3
TPSD1	chr16	1306927/C//T	synonymous	1	3

CDC27	chr17	45214510/C//G	synonymous	0	4
RANGAP1	chr22	41650337/C//T	nonsynonymous	0	4
APC	chr5	112175576/C//T	nonsynonymous	0	4
PLEC	chr8	144995457/C//G	synonymous	1	3
CAPN12	chr19	39226927/T//C	nonsynonymous	0	4
CD93	chr20	23065344/G//A	nonsynonymous	0	4
SMAD4	chr18	48591904/C//T	nonsynonymous	0	4
	chr4	26322448/A//C	synonymous	1	2
KRTAP10-11	chr21	46066636/G//A	synonymous	2	1
MEP1A	chr6	46806766/G//A	nonsynonymous	1	2
KCNH4	chr17	40318310/C//A	synonymous	0	3
ATXN2L	chr16	28848110/G//T	synonymous	0	3
COL20A1	chr20	61951636/G//A	synonymous	1	2
PLCH2	chr1	2429964/G//A	nonsynonymous	0	3
CTNNB1	chr3	41266113/C//T	nonsynonymous	1	2
LAMB2	chr3	49163400/G//A	nonsynonymous	1	2
DOCK3	chr3	51315142/C//T	nonsynonymous	0	3
KIAA1549	chr7	138545904/G//A	nonsynonymous	0	3
GAPVD1	chr9	128064536/C//T	nonsynonymous	1	2
GDPD5	chr11	75146556/C//T	nonsynonymous	0	3
KRAS	chr12	25380283/C//T	nonsynonymous	1	2
COL6A1	chr21	47423400/C//T	nonsynonymous	1	2
SPTA1	chr1	158623113/G//A	nonsynonymous	0	3
FAM90A1	chr12	8374605/C//T	nonsynonymous	1	2
PIK3CA	chr3	178928079/G//A	nonsynonymous	1	2
POTEE	chr2	132021599/C//T	synonymous	1	2
OTOGL	chr12	80771719/G//A	nonsynonymous	1	2
TMEM8B	chr9	35853671/C//T	nonsynonymous	1	2
FLG	chr1	152285238/A//G	synonymous	1	2
KRTAP10-6	chr21	46012360/G//A	synonymous	1	2
PCDHA6	chr5	140209359/C//T	synonymous	0	3
PCDH17	chr13	58208417/C//T	synonymous	0	3
KRAS	chr12	25378562/C//T	nonsynonymous	1	2
ZNF717	chr3	75787186/C//T	nonsynonymous	0	3
CDC27	chr17	45216231/T//C	synonymous	0	3
KCNC1	chr11	17758115/C//T	nonsynonymous	2	1
C8orf22	chr8	49987926/C//A	synonymous	1	2
FBXW7	chr4	153250883/G//A	nonsynonymous	1	2
SATB2	chr2	200173527/C//T	nonsynonymous	0	3
PIK3CA	chr3	178916876/G//A	nonsynonymous	0	3
ANP32C	chr4	165118820/G//A	nonsynonymous	0	3
POTEC	chr18	14543098/C//T	synonymous	0	3
OR7D2	chr19	9296570/C//T	nonsynonymous	0	3
TP53	chr17	7577559/G//T	nonsynonymous	0	3
ZNF814	chr19	58385426/A//T	synonymous	0	3
ZNF814	chr19	58385442/T//C	nonsynonymous	0	3
LRFN1	chr19	39804645/G//T	synonymous	1	2
ADARB2	chr10	1405429/G//A	nonsynonymous	0	3
RYR1	chr19	39008210/C//T	synonymous	0	3
CDC27	chr17	45216141/T//G	synonymous	0	3
CDC27	chr17	45216210/A//G	synonymous	0	3
CDC27	chr17	45216216/A//G	synonymous	0	3
CDC27	chr17	45219309/A//G	synonymous	0	3
CSMD1	chr8	2857618/C//T	nonsynonymous	1	2
GPR132	chr14	105517426/G//C	nonsynonymous	0	3
CNN2	chr19	1037679/C//T	nonsynonymous	1	2
CNN2	chr19	1037681/G//A	nonsynonymous	1	2
SLAMF6	chr1	160456412/A//G	synonymous	0	3
PTK2B	chr8	27291021/C//T	nonsynonymous	1	2
CNTNAP4	chr16	76482757/G//A	nonsynonymous	0	3
LHPP	chr10	126176971/G//GTC	synonymous	1	2
ZNF595	chr4	53360/C//G	synonymous	0	3
TPTE	chr21	10959806/G//T	synonymous	2	1
AMER1	chrX	63412095/G//A	nonsynonymous	1	2
TP53	chr17	7577551/C//T	nonsynonymous	0	3
SDR9C7	chr12	57323196/G//A	synonymous	0	3
FAM196B	chr5	169310626/G//A	nonsynonymous	0	3
IRX4	chr5	1879887/G//A	nonsynonymous	0	3
MS4A15	chr11	60540955/C//T	nonsynonymous	1	2
DDB1	chr11	61099067/T//C	nonsynonymous	0	3

PLG	chr6	161135860/C//T	synonymous	0	3
MEX3A	chr1	156046781/C//A	nonsynonymous	0	3
PAX4	chr7	127255505/G//A	nonsynonymous	0	3
APC	chr5	112175328/C//A	nonsynonymous	1	2
EPHA10	chr1	38201002/G//T	nonsynonymous	0	2
CHRNB2	chr1	154543824/G//A	synonymous	0	2
INSRR	chr1	156821096/G//A	synonymous	0	2
RCOR3	chr1	211444638/C//G	nonsynonymous	0	2
EHD3	chr2	31483550/C//T	nonsynonymous	0	2
SLC9A4	chr2	103130668/AG//A	nonsynonymous	0	2
TTN	chr2	179640287/C//T	nonsynonymous	0	2
TNS1	chr2	218683202/G//A	nonsynonymous	0	2
ATG16L1	chr2	234200896/G//A	nonsynonymous	0	2
SRGAP3	chr3	9055479/C//T	nonsynonymous	0	2
NBEAL2	chr3	47036753/G//A	nonsynonymous	0	2
FOXP1	chr3	71101777/G//A	nonsynonymous	0	2
TF	chr3	133485259/A//C	nonsynonymous	0	2
ZBTB49	chr4	4323071/G//A	synonymous	0	2
FAT4	chr4	126370617/C//A	nonsynonymous	0	2
TRIML2	chr4	189012848/C//T	synonymous	0	2
HTR1A	chr5	63257100/C//T	synonymous	0	2
PIK3R1	chr5	67589028/G//A	nonsynonymous	0	2
POU5F2	chr5	93076993/G//A	nonsynonymous	0	2
CDC25C	chr5	137666841/C//G	nonsynonymous	0	2
PCDHA3	chr5	140182272/G//A	nonsynonymous	0	2
CDYL	chr6	4892459/C//T	synonymous	0	2
COL11A2	chr6	33133517/C//T	nonsynonymous	0	2
PTCHD4	chr6	47847083/C//T	synonymous	0	2
	chr7	32209485/C//A	synonymous	0	2
URGCP	chr7	43916971/G//A	synonymous	0	2
ZNF655	chr7	99170615/A//G	nonsynonymous	0	2
RELN	chr7	103138555/C//A	nonsynonymous	0	2
TRPV5	chr7	142622807/C//G	synonymous	0	2
CNTNAP2	chr7	147092836/C//T	nonsynonymous	0	2
CSMD1	chr8	2855561/C//T	synonymous	0	2
PKHD1L1	chr8	110498978/G//A	nonsynonymous	0	2
	chr9	85858022/C//T	synonymous	0	2
CACNA1B	chr9	140878665/C//T	nonsynonymous	0	2
MLLT10	chr10	21827815/G//T	nonsynonymous	0	2
MLLT10	chr10	21962662/G//T	nonsynonymous	0	2
	chr10	22701422/G//A	synonymous	0	2
RHOBTB1	chr10	62671228/C//T	nonsynonymous	0	2
TET1	chr10	70450959/G//A	synonymous	0	2
UBTD1	chr10	99330294/C//A	synonymous	0	2
HPS1	chr10	100190958/C//A	nonsynonymous	0	2
C10orf91	chr10	134261320/T//A	nonsynonymous	0	2
DCHS1	chr11	6646598/C//A	nonsynonymous	0	2
C12orf4	chr12	4645224/C//T	nonsynonymous	0	2
GALNT8	chr12	4854666/G//A	nonsynonymous	0	2
DENND5B	chr12	31600643/T//C	nonsynonymous	0	2
FZD10	chr12	130648325/C//T	nonsynonymous	0	2
LIG4	chr13	108861526/C//T	synonymous	0	2
AHNAK2	chr14	105404488/G//A	nonsynonymous	0	2
ATP10A	chr15	26107846/C//T	nonsynonymous	0	2
JMJD7-PLA2G4B	chr15	42137247/C//T	synonymous	0	2
TP53BP1	chr15	43699735/G//A	nonsynonymous	0	2
OTOA	chr16	21737848/C//T	nonsynonymous	0	2
CA7	chr16	66887318/G//A	nonsynonymous	0	2
NFAT5	chr16	69704149/T//G	synonymous	0	2
TUBB3	chr16	90001664/G//A	nonsynonymous	0	2
FBXL20	chr17	37425075/C//G	synonymous	0	2
KIAA1468	chr18	59925837/G//A	nonsynonymous	0	2
TUBB4A	chr19	6501414/G//A	synonymous	0	2
TSHZ3	chr19	31768116/T//C	synonymous	0	2
ZNF566	chr19	36940744/T//A	nonsynonymous	0	2
PWP2	chr21	45548209/G//A	nonsynonymous	0	2
XAGE5	chrX	52847228/A//G	synonymous	0	2
TGIF2LX	chrX	89177717/C//T	synonymous	0	2
ARMCX5-GPRASP2	chrX	101970562/G//C	nonsynonymous	0	2
L1CAM	chrX	153130580/G//A	synonymous	0	2

CTNNB1	chr3	41266104/G//T	nonsynonymous	2	0
GOLIM4	chr3	167747615/C//A	nonsynonymous	1	1
DCAF8	chr1	160209571/A//C	nonsynonymous	1	1
KRTAP9-1	chr17	39346413/G//T	nonsynonymous	1	1
CDK5RAP3	chr17	46058835/G//C	synonymous	1	1
ZNF729	chr19	22497166/C//G	nonsynonymous	1	1
SFPQ	chr1	35656463/T//G	nonsynonymous	0	2
COL11A1	chr1	103488309/T//C	nonsynonymous	0	2
HMCN1	chr1	186024744/A//G	nonsynonymous	0	2
PIGR	chr1	207112682/C//T	nonsynonymous	0	2
NVL	chr1	224484239/G//A	nonsynonymous	0	2
OBSCN	chr1	228466581/G//A	nonsynonymous	0	2
CAPN9	chr1	230921750/T//C	nonsynonymous	0	2
NCOA1	chr2	24928080/A//G	nonsynonymous	0	2
NRXN1	chr2	50780066/C//T	nonsynonymous	0	2
SLC19A3	chr2	228552909/T//C	synonymous	0	2
USP19	chr3	49153922/A//C	synonymous	0	2
P2RY12	chr3	151056630/G//C	nonsynonymous	0	2
GK2	chr4	80328689/A//C	nonsynonymous	0	2
AP3B1	chr5	77458740/T//A	synonymous	0	2
PCDHA7	chr5	140215887/C//T	nonsynonymous	0	2
TECPR1	chr7	97863207/C//T	nonsynonymous	0	2
FUT10	chr8	33230150/T//G	nonsynonymous	0	2
TACC1	chr8	38677246/A//G	nonsynonymous	0	2
SNTG1	chr8	51362279/C//A	nonsynonymous	0	2
ZNF189	chr9	104170803/A//C	nonsynonymous	0	2
AGAP5	chr10	75457330/G//A	nonsynonymous	0	2
MED17	chr11	93545012/T//A	synonymous	0	2
SCN4B	chr11	118015783/C//T	nonsynonymous	0	2
PAN2	chr12	56722320/G//A	nonsynonymous	0	2
THSD1	chr13	52952405/G//A	nonsynonymous	0	2
ANKRD34C	chr15	79587135/G//C	nonsynonymous	0	2
SELENOS	chr15	101814653/T//C	synonymous	0	2
MVP	chr16	29855907/A//T	synonymous	0	2
ANKRD11	chr16	89352579/G//A	synonymous	0	2
DHPS	chr19	12787936/T//A	nonsynonymous	0	2
OR7C1	chr19	14910475/G//A	synonymous	0	2
MYT1	chr20	62858789/C//T	synonymous	0	2
KRTAP10-11	chr21	46066648/C//A	synonymous	0	2
MYH9	chr22	36737424/T//C	nonsynonymous	0	2
PADI2	chr1	17410325/C//T	nonsynonymous	0	2
KIRREL	chr1	158064855/C//G	nonsynonymous	0	2
B3GALNT2	chr1	235611649/C//T	synonymous	0	2
SNTG2	chr2	1320143/C//T	nonsynonymous	0	2
CYP1B1	chr2	38301682/G//A	nonsynonymous	0	2
ZEB2	chr2	145162543/C//G	nonsynonymous	0	2
BSN	chr3	49695570/G//A	nonsynonymous	0	2
PSAPL1	chr4	7435401/C//A	synonymous	0	2
PACRGL	chr4	20709442/G//A	nonsynonymous	0	2
FBXL7	chr5	15937262/C//T	synonymous	0	2
VCAN	chr5	82815665/C//T	nonsynonymous	0	2
IGF2BP3	chr7	23401206/G//A	synonymous	0	2
NOM1	chr7	156746891/G//A	nonsynonymous	0	2
ARFGEF1	chr8	68128877/T//C	nonsynonymous	0	2
ZFHX4	chr8	77764890/G//A	synonymous	0	2
CSMD3	chr8	113256754/C//A	nonsynonymous	0	2
KCNA4	chr11	30034125/G//T	nonsynonymous	0	2
SLC22A8	chr11	62782446/C//T	synonymous	0	2
FDXACB1	chr11	111745735/C//T	nonsynonymous	0	2
KIRREL3	chr11	126333111/C//T	nonsynonymous	0	2
PLXNC1	chr12	94543282/G//A	nonsynonymous	0	2
ANKS1B	chr12	100200273/C//T	nonsynonymous	0	2
TCP11L2	chr12	106715478/T//C	nonsynonymous	0	2
NOVA1	chr14	27064755/G//A	synonymous	0	2
KCNH5	chr14	63447728/C//T	synonymous	0	2
AHNAK2	chr14	105415096/C//A	nonsynonymous	0	2
	chr16	4776826/C//T	synonymous	0	2
GTF3C1	chr16	27495625/G//A	nonsynonymous	0	2
ABCC12	chr16	48125077/G//A	nonsynonymous	0	2
DEF8	chr16	90030936/C//T	synonymous	0	2

MYO19	chr17	34881161/G//C	synonymous	0	2
KRT10	chr17	38976357/C//A	nonsynonymous	0	2
LPIN2	chr18	2931392/C//A	nonsynonymous	0	2
SMAD4	chr18	48593557/G//C	nonsynonymous	0	2
SERPINB12	chr18	61234021/G//A	nonsynonymous	0	2
MUC16	chr19	9064395/G//A	nonsynonymous	0	2
KCNA7	chr19	49573770/G//A	synonymous	0	2
TMEM150B	chr19	55832377/C//T	nonsynonymous	0	2
SOGA1	chr20	35441193/G//A	nonsynonymous	0	2
ACOT8	chr20	44477307/C//T	synonymous	0	2
HELZ2	chr20	62195369/G//A	synonymous	0	2
RTEL1	chr20	62305429/C//T	nonsynonymous	0	2
CHRDL1	chrX	109931971/G//T	nonsynonymous	0	2
SPANXN1	chrX	144337399/C//A	synonymous	0	2
HCFC1	chrX	153215719/C//T	synonymous	0	2
FAM43B	chr1	20879390/G//A	synonymous	0	2
CSMD2	chr1	34192195/C//T	nonsynonymous	0	2
LRP8	chr1	53729891/GAA//G	nonsynonymous	0	2
LRRC8D	chr1	90401062/G//A	nonsynonymous	0	2
ZNF644	chr1	91405282/A//T	nonsynonymous	0	2
GJA8	chr1	147380211/C//T	synonymous	0	2
CTSK	chr1	150769212/G//T	synonymous	0	2
PGLYRP4	chr1	153312955/G//A	synonymous	0	2
C1orf27	chr1	186355130/T//G	nonsynonymous	0	2
BRINP3	chr1	190068067/C//G	nonsynonymous	0	2
TMEM206	chr1	212588083/C//T	nonsynonymous	0	2
OBSCN	chr1	228557713/G//A	nonsynonymous	0	2
ALLC	chr2	3727535/G//A	synonymous	0	2
LRRTM4	chr2	77746471/C//G	nonsynonymous	0	2
SMYD1	chr2	88387508/C//A	synonymous	0	2
IL36A	chr2	113763355/A//G	synonymous	0	2
CNTN4	chr3	3095610/C//T	synonymous	0	2
KLHL40	chr3	42727901/G//A	nonsynonymous	0	2
QRICH1	chr3	49081831/G//A	nonsynonymous	0	2
HGD	chr3	120363212/T//C	nonsynonymous	0	2
STXBP5L	chr3	120976137/G//A	nonsynonymous	0	2
ZIC1	chr3	147128362/T//A	nonsynonymous	0	2
MCF2L2	chr3	182897388/T//TC	nonsynonymous	0	2
DVL3	chr3	183885438/A//G	synonymous	0	2
DLG1	chr3	196846370/G//C	nonsynonymous	0	2
JAKMIP1	chr4	6066667/T//C	synonymous	0	2
ADH1C	chr4	100266028/T//G	nonsynonymous	0	2
ADH1C	chr4	100266031/G//A	synonymous	0	2
ADH1C	chr4	100266055/A//G	synonymous	0	2
TRAPPC11	chr4	184605914/T//C	nonsynonymous	0	2
IRX1	chr5	3600203/G//A	nonsynonymous	0	2
IRX1	chr5	3600241/C//T	synonymous	0	2
AMACR	chr5	33989511/G//A	nonsynonymous	0	2
HMGCS1	chr5	43294904/G//C	nonsynonymous	0	2
HCN1	chr5	45262314/G//T	synonymous	0	2
GZMA	chr5	54401418/T//C	nonsynonymous	0	2
ADAMTS6	chr5	64520764/T//C	synonymous	0	2
LVRN	chr5	115319118/C//A	nonsynonymous	0	2
	chr6	10586488/C//T	synonymous	0	2
NEDD9	chr6	11190948/G//A	nonsynonymous	0	2
HIST1H2BK	chr6	27114593/C//T	synonymous	0	2
ADGRB3	chr6	69348962/G//A	nonsynonymous	0	2
COL12A1	chr6	75852994/T//C	nonsynonymous	0	2
SASH1	chr6	148865333/G//C	nonsynonymous	0	2
LFNG	chr7	2566474/C//G	nonsynonymous	0	2
PCLO	chr7	82580041/T//C	nonsynonymous	0	2
C7orf34	chr7	142637557/C//A	nonsynonymous	0	2
OR2A25	chr7	143771517/G//A	nonsynonymous	0	2
ZNF596	chr8	195648/A//G	synonymous	0	2
NKX2-6	chr8	23563997/C//G	nonsynonymous	0	2
TMEM70	chr8	74888513/C//T	synonymous	0	2
FAM84B	chr8	127569083/G//A	synonymous	0	2
GLIS3	chr9	3898700/G//C	nonsynonymous	0	2
IFNA10	chr9	21207013/C//A	nonsynonymous	0	2
NUTM2G	chr9	99697680/C//T	nonsynonymous	0	2

PTPN3	chr9	112172532/C//T	nonsynonymous	0	2
OR1N1	chr9	125289123/G//C	synonymous	0	2
INPP5E	chr9	139327015/G//A	nonsynonymous	0	2
LARP4B	chr10	888998/C//G	nonsynonymous	0	2
DHTKD1	chr10	12155009/G//T	synonymous	0	2
MYOZ1	chr10	75399702/C//A	nonsynonymous	0	2
WAPL	chr10	88203107/G//A	synonymous	0	2
ZFYVE27	chr10	99517046/C//T	nonsynonymous	0	2
MUC5B	chr11	1262118/G//A	synonymous	0	2
LRRC55	chr11	56954996/C//T	synonymous	0	2
SCYL1	chr11	65293767/A//T	nonsynonymous	0	2
FAT3	chr11	92616069/C//T	synonymous	0	2
PKNOX2	chr11	125301239/C//T	nonsynonymous	0	2
KRT6C	chr12	52864990/C//T	nonsynonymous	0	2
MYO1A	chr12	57423379/C//T	synonymous	0	2
ATP2B1	chr12	90049658/G//T	synonymous	0	2
POP5	chr12	121019026/G//A	nonsynonymous	0	2
TUBA3C	chr13	19748143/C//T	nonsynonymous	0	2
DCLK1	chr13	36428681/C//T	synonymous	0	2
CSNK1A1L	chr13	37678579/G//T	nonsynonymous	0	2
EDNRB	chr13	78492700/C//T	synonymous	0	2
ZIC2	chr13	100635292/G//A	nonsynonymous	0	2
SPTB	chr14	65241196/C//T	nonsynonymous	0	2
UNC79	chr14	94088370/A//G	synonymous	0	2
DYNC1H1	chr14	102516145/A//G	nonsynonymous	0	2
GABRG3	chr15	27777790/C//A	synonymous	0	2
SLC12A1	chr15	48539603/C//G	nonsynonymous	0	2
CSPG4	chr15	75982669/C//T	nonsynonymous	0	2
WFIKKN1	chr16	683835/C//G	synonymous	0	2
ABCC11	chr16	48239437/G//A	synonymous	0	2
CNGB1	chr16	57991262/A//G	nonsynonymous	0	2
DHX8	chr17	41598247/G//A	synonymous	0	2
FZD2	chr17	42636513/T//C	nonsynonymous	0	2
ITGB3	chr17	45376687/C//A	nonsynonymous	0	2
COL1A1	chr17	48275858/G//A	nonsynonymous	0	2
PDE6G	chr17	79618097/C//T	synonymous	0	2
GPS1	chr17	80013953/G//A	nonsynonymous	0	2
PTPRM	chr18	8394498/G//A	synonymous	0	2
PCSK4	chr19	1483395/G//A	nonsynonymous	0	2
ZNF729	chr19	22499415/C//T	nonsynonymous	0	2
RELB	chr19	45540730/C//T	synonymous	0	2
SYT3	chr19	51133064/G//A	nonsynonymous	0	2
KIR2DL4	chr19	55316507/C//A	nonsynonymous	0	2
PEG3	chr19	57335705/G//A	nonsynonymous	0	2
BPIFA2	chr20	31761975/G//A	synonymous	0	2
BRWD1	chr21	40568642/G//A	nonsynonymous	0	2
GGT5	chr22	24622072/C//T	nonsynonymous	0	2
TCF20	chr22	42610387/T//A	nonsynonymous	0	2
CELSR1	chr22	46859820/G//T	synonymous	0	2
ZRSR2	chrX	15833854/C//T	synonymous	0	2
DACH2	chrX	86067981/G//A	nonsynonymous	0	2
NXF3	chrX	102335091/C//T	synonymous	0	2
AGTR2	chrX	115304168/C//A	nonsynonymous	0	2
NSDHL	chrX	152037638/G//A	nonsynonymous	0	2
ZNF208	chr19	22155980/C//A	nonsynonymous	1	1
NBR1	chr17	41332806/T//C	synonymous	1	1
DNM3	chr1	172100387/G//A	nonsynonymous	0	2
NRXN1	chr2	50692579/C//T	nonsynonymous	0	2
LRRFIP2	chr3	37114363/C//T	nonsynonymous	0	2
HESX1	chr3	57233833/T//G	nonsynonymous	0	2
DOCK2	chr5	169230209/C//T	nonsynonymous	0	2
EYS	chr6	65300609/T//A	synonymous	0	2
TRDN	chr6	123539760/G//T	nonsynonymous	0	2
NPSR1	chr7	34867074/C//A	synonymous	0	2
CYP11B1	chr8	143957293/G//A	nonsynonymous	0	2
ZNHIT2	chr11	64885081/G//A	synonymous	0	2
MRGPRF	chr11	68773040/G//A	synonymous	0	2
NECTIN1	chr11	119548403/G//A	nonsynonymous	0	2
VWA5A	chr11	124012438/G//A	synonymous	0	2
DCT	chr13	95121241/G//A	synonymous	0	2

RASGRP1	chr15	38803840/C//A	nonsynonymous	0	2
ITPRIPL2	chr16	19126678/G//A	nonsynonymous	0	2
PDPR	chr16	70187366/G//A	nonsynonymous	0	2
ZFHX3	chr16	72828961/C//T	synonymous	0	2
PIK3R5	chr17	8793353/G//A	nonsynonymous	0	2
KIF18B	chr17	43005433/G//A	nonsynonymous	0	2
ELAVL1	chr19	8028423/G//A	nonsynonymous	0	2
OLFM2	chr19	9965137/C//T	nonsynonymous	0	2
ZNF799	chr19	12501526/A//G	synonymous	0	2
ASPHD2	chr22	26830286/T//C	synonymous	0	2
GCNT3	chr15	59911157/C//A	synonymous	0	2
NGRN	chr15	90814933/A//G	synonymous	0	2
SIRT7	chr17	79871660/C//T	nonsynonymous	0	2
SAFB2	chr19	5613522/A//C	nonsynonymous	0	2
HNRNPCL4	chr1	12907296/C//T	nonsynonymous	0	2
ZNF845	chr19	53855052/A//G	nonsynonymous	1	1
PIK3CD	chr1	9776641/GTACCTGTATGGCAGC	nonsynonymous	0	2
MTFR1L	chr1	26150240/G//C	nonsynonymous	0	2
HFM1	chr1	91731617/G//C	nonsynonymous	0	2
LMX1A	chr1	165173110/GGAAGACTCAAGAT	nonsynonymous	0	2
PPFIA4	chr1	203023000/G//A	synonymous	0	2
SNTG2	chr2	1320048/G//T	nonsynonymous	0	2
CRIPT	chr2	46850992/G//C	nonsynonymous	0	2
MDH1	chr2	63831904/G//C	nonsynonymous	0	2
REEP1	chr2	86459917/A//G	synonymous	0	2
NMI	chr2	152132380/T//C	nonsynonymous	0	2
LRP2	chr2	169995844/G//T	synonymous	0	2
DUSP19	chr2	183943507/G//C	synonymous	0	2
CALCRL	chr2	188248026/C//A	nonsynonymous	0	2
CDK5R2	chr2	219825321/C//A	nonsynonymous	0	2
SPEG	chr2	220338298/C//G	nonsynonymous	0	2
TBC1D5	chr3	17549971/G//C	nonsynonymous	0	2
SLC25A38	chr3	39431042/C//T	synonymous	0	2
COL7A1	chr3	48616686/C//A	nonsynonymous	0	2
ITIH1	chr3	52820346/T//C	synonymous	0	2
ADCY5	chr3	123166599/G//C	nonsynonymous	0	2
EPHB3	chr3	184295786/C//T	synonymous	0	2
FAM160A1	chr4	152499004/T//C	nonsynonymous	0	2
TKTL2	chr4	164393220/G//C	nonsynonymous	0	2
SLC25A2	chr5	140683334/T//C	nonsynonymous	0	2
TCERG1	chr5	145843177/T//C	nonsynonymous	0	2
	chr6	56484766/G//A	synonymous	0	2
PHTF2	chr7	77469611/A//G	synonymous	0	2
WDR91	chr7	134896249/C//G	synonymous	0	2
PKHD1L1	chr8	110447538/C//T	nonsynonymous	0	2
GPR20	chr8	142367897/C//A	nonsynonymous	0	2
TYRP1	chr9	12694266/G//A	nonsynonymous	0	2
CUBN	chr10	16981010/C//A	nonsynonymous	0	2
ERCC6	chr10	50666792/T//G	synonymous	0	2
PTEN	chr10	89720683/C//T	synonymous	0	2
PAMR1	chr11	35515766/C//T	nonsynonymous	0	2
OR8K5	chr11	55927421/C//T	nonsynonymous	0	2
CDC42BPG	chr11	64595208/C//T	nonsynonymous	0	2
DPP3	chr11	66264890/G//A	nonsynonymous	0	2
STT3A	chr11	125479485/G//GTA	synonymous	0	2
SLCO1B7	chr12	21207422/C//A	nonsynonymous	0	2
RASSF8	chr12	26218155/A//C	nonsynonymous	0	2
CDK4	chr12	58144541/G//A	nonsynonymous	0	2
SETD1B	chr12	122242665/CA//C	nonsynonymous	0	2
MTMR6	chr13	25840338/C//A	nonsynonymous	0	2
RNASE10	chr14	20979254/G//T	nonsynonymous	0	2
SIX4	chr14	61190085/G//C	nonsynonymous	0	2
SLC8A3	chr14	70635083/A//T	synonymous	0	2
NRDE2	chr14	90754540/AACAGAACGGTCTGG	nonsynonymous	0	2
SLC24A5	chr15	48431282/T//G	nonsynonymous	0	2
ITGAM	chr16	31336343/G//A	nonsynonymous	0	2
ZNF469	chr16	88502616/G//C	nonsynonymous	0	2
TP53	chr17	7578211/C//A	nonsynonymous	0	2
TNFRSF13B	chr17	16852173/G//A	synonymous	0	2
TMEM132E	chr17	32963198/C//A	nonsynonymous	0	2

LLGL2	chr17	73564753/G//C	nonsynonymous	0	2
	chr18	29672602/C//G	synonymous	0	2
CDH19	chr18	64218502/A//C	synonymous	0	2
ADGRE1	chr19	6937328/G//C	nonsynonymous	0	2
ATG4D	chr19	10655810/A//T	synonymous	0	2
YIPF2	chr19	11034671/G//T	synonymous	0	2
MYO9B	chr19	17283768/T//G	synonymous	0	2
ZNF708	chr19	21476910/G//T	synonymous	1	1
ZNF91	chr19	23578232/C//G	synonymous	0	2
ATP4A	chr19	36045951/T//A	nonsynonymous	0	2
PAF1	chr19	39879955/C//T	nonsynonymous	0	2
KLK4	chr19	51411981/C//G	nonsynonymous	0	2
NCOA6	chr20	33345761/G//C	nonsynonymous	0	2
TAF4	chr20	60587907/C//A	nonsynonymous	0	2
SEC14L6	chr22	30921089/C//G	synonymous	0	2
APOBEC3G	chr22	39479831/G//A	nonsynonymous	0	2
GNL3L	chrX	54567760/C//T	nonsynonymous	0	2
COL4A5	chrX	107845151/G//A	nonsynonymous	0	2
WDPCP	chr2	63631512/C//A	nonsynonymous	0	2
TTC30A	chr2	178482957/A//C	nonsynonymous	0	2
GNAT1	chr3	50231618/C//A	nonsynonymous	0	2
ADPRH	chr3	119306313/C//T	nonsynonymous	0	2
FAT4	chr4	126370300/G//A	nonsynonymous	0	2
GLRA1	chr5	151239470/G//C	nonsynonymous	0	2
TNXB	chr6	32037382/G//A	synonymous	0	2
MFSD4B	chr6	111587808/C//T	nonsynonymous	0	2
NPC1L1	chr7	44579814/G//A	nonsynonymous	0	2
C7orf72	chr7	50191073/C//A	synonymous	0	2
PTPRZ1	chr7	121653099/C//T	synonymous	0	2
PRSS3P2	chr7	142481353/G//A	synonymous	0	2
SEC16A	chr9	139371928/G//A	nonsynonymous	0	2
C9orf163	chr9	139379280/C//T	nonsynonymous	0	2
DIP2C	chr10	436703/G//A	nonsynonymous	0	2
ITIH2	chr10	7769786/C//T	nonsynonymous	0	2
NRG3	chr10	84745186/G//A	nonsynonymous	0	2
B4GALNT4	chr11	377106/G//A	synonymous	0	2
CAPN1	chr11	64953421/G//A	nonsynonymous	0	2
P2RY6	chr11	73008494/C//T	nonsynonymous	0	2
C12orf71	chr12	27234970/G//A	synonymous	0	2
TINF2	chr14	24709873/T//G	nonsynonymous	0	2
SLX4	chr16	3646264/C//A	nonsynonymous	0	2
C16orf52	chr16	22086861/C//T	nonsynonymous	0	2
ZNF689	chr16	30616089/G//A	synonymous	0	2
DPEP3	chr16	68014114/G//A	nonsynonymous	0	2
ANKFN1	chr17	54403618/G//T	synonymous	0	2
WIPI1	chr17	66425048/G//A	nonsynonymous	0	2
CCDC114	chr19	48800655/C//T	nonsynonymous	0	2
ZNF274	chr19	58723941/G//A	nonsynonymous	0	2
PTPRA	chr20	2945774/C//T	nonsynonymous	0	2
CFAP61	chr20	20243672/C//T	nonsynonymous	0	2
KRTAP19-8	chr21	32410628/T//A	synonymous	0	2
EP300	chr22	41568593/G//C	nonsynonymous	0	2
ZNF645	chrX	22292363/C//A	nonsynonymous	0	2
PLP1	chrX	103041612/G//A	nonsynonymous	0	2
SLC25A43	chrX	118540544/C//T	nonsynonymous	0	2
BRS3	chrX	135574491/C//T	nonsynonymous	0	2
CTNNB1	chr3	41266137/C//T	nonsynonymous	2	0
PLEKHO1	chr1	150131552/C//T	nonsynonymous	0	2
IQGAP3	chr1	156517950/C//T	nonsynonymous	0	2
ASTN1	chr1	176838102/G//A	synonymous	0	2
KCNT2	chr1	196459043/A//G	nonsynonymous	0	2
F13B	chr1	197025007/G//A	nonsynonymous	0	2
AGT	chr1	230839054/C//A	nonsynonymous	0	2
OTOF	chr2	26695380/G//A	synonymous	0	2
HK2	chr2	75115122/G//A	nonsynonymous	0	2
PTCD3	chr2	86364250/A//G	nonsynonymous	0	2
ANKRD23	chr2	97505494/G//C	synonymous	0	2
GRM7	chr3	7188209/G//A	nonsynonymous	0	2
GOLGA4	chr3	37370007/A//C	nonsynonymous	0	2
KLHL40	chr3	42730433/C//T	synonymous	0	2

KALRN	chr3	124376289/C//T	synonymous	0	2
LINC01565	chr3	128292158/G//T	synonymous	0	2
TRPC1	chr3	142521045/A//T	nonsynonymous	0	2
MED12L	chr3	151112529/C//T	synonymous	0	2
STOX2	chr4	184930527/C//T	nonsynonymous	0	2
MAP3K1	chr5	56177043/A//G	synonymous	0	2
APC	chr5	112175216/G//T	nonsynonymous	0	2
PCDHGA4	chr5	140734755/C//T	synonymous	0	2
HAVCR1	chr5	156479518/G//A	nonsynonymous	0	2
HIST1H1B	chr6	27834574/T//C	synonymous	0	2
FGD2	chr6	36979617/C//T	nonsynonymous	0	2
RPL7L1	chr6	42847690/G//A	nonsynonymous	0	2
XPO5	chr6	43540293/G//A	nonsynonymous	0	2
SLC35B2	chr6	44223037/G//A	synonymous	0	2
GPNMB	chr7	23299622/C//T	nonsynonymous	0	2
GCK	chr7	44187439/T//C	synonymous	0	2
KEL	chr7	142643391/C//T	nonsynonymous	0	2
SSPO	chr7	149500346/C//T	synonymous	0	2
CYP11B1	chr8	143957763/G//T	nonsynonymous	0	2
RAG1	chr11	36596007/A//G	nonsynonymous	0	2
TIGD3	chr11	65124634/C//T	nonsynonymous	0	2
LRP5	chr11	68115647/C//T	nonsynonymous	0	2
DYNC2H1	chr11	103270487/C//T	nonsynonymous	0	2
IGSF9B	chr11	133789848/G//A	nonsynonymous	0	2
KRT74	chr12	52962039/G//A	synonymous	0	2
ANHX	chr12	133810751/C//T	synonymous	0	2
TUBA3C	chr13	19748203/C//T	nonsynonymous	0	2
THSD1	chr13	52952713/G//A	synonymous	0	2
ZFYVE26	chr14	68256063/A//C	nonsynonymous	0	2
IGDCC4	chr15	65686780/C//T	synonymous	0	2
STRA6	chr15	74487998/G//T	nonsynonymous	0	2
WSCD1	chr17	6023845/G//A	nonsynonymous	0	2
ACOX1	chr17	73945334/C//A	synonymous	0	2
CBX8	chr17	77768549/C//A	nonsynonymous	0	2
LMNB2	chr19	2434320/C//T	nonsynonymous	0	2
CCDC151	chr19	11533199/C//A	synonymous	0	2
PSG7	chr19	43439848/G//A	synonymous	0	2
NTF4	chr19	49564984/G//A	nonsynonymous	0	2
	chr19	50758485/G//A	synonymous	0	2
SNAP25	chr20	10280022/G//A	nonsynonymous	0	2
TUBB1	chr20	57598806/G//A	synonymous	0	2
MTG2	chr20	60770943/G//A	nonsynonymous	0	2
KRTAP10-6	chr21	46012351/G//A	synonymous	0	2
PBDC1	chrX	75396797/C//T	nonsynonymous	0	2
NRK	chrX	105153812/C//T	nonsynonymous	0	2
TRPC5	chrX	111090567/C//T	nonsynonymous	0	2
F8	chrX	154157303/T//C	nonsynonymous	0	2
PUM1	chr1	31532277/G//A	nonsynonymous	0	2
CNGA3	chr2	99006129/C//T	nonsynonymous	0	2
TUBA3E	chr2	130952725/C//T	nonsynonymous	0	2
BAZ2B	chr2	160289748/T//C	nonsynonymous	0	2
CFAP65	chr2	219894901/G//A	synonymous	0	2
FGD5	chr3	14861538/C//T	synonymous	0	2
PLCD1	chr3	38051771/G//A	synonymous	0	2
DCHS2	chr4	155157404/C//T	synonymous	0	2
APC	chr5	112175247/CTG//C	nonsynonymous	0	2
FAT2	chr5	150947998/G//C	nonsynonymous	0	2
FARS2	chr6	5368987/G//A	nonsynonymous	0	2
SLC17A1	chr6	25813380/G//A	synonymous	0	2
OR2J2	chr6	29142214/T//G	nonsynonymous	0	2
PIK3CG	chr7	106508080/C//T	nonsynonymous	0	2
OR6B1	chr7	143701287/G//C	synonymous	0	2
CDK9	chr9	130550544/G//A	nonsynonymous	0	2
LAMC3	chr9	133917080/G//A	nonsynonymous	0	2
CXCL12	chr10	44876285/G//A	synonymous	0	2
IFIT2	chr10	91065878/C//A	nonsynonymous	0	2
CFAP46	chr10	134679564/G//A	nonsynonymous	0	2
TH	chr11	2189715/C//T	synonymous	0	2
LARGE2	chr11	45947896/AAGGTG//A	nonsynonymous	0	2
MIR4690	chr11	65403796/G//C	synonymous	0	2

DDI1	chr11	103908445/A//G	nonsynonymous	0	2
PKNOX2	chr11	125280091/G//A	nonsynonymous	0	2
FLI1	chr11	128628033/C//T	synonymous	0	2
FGF23	chr12	4479729/C//T	nonsynonymous	0	2
DCAF4	chr14	73420880/C//T	nonsynonymous	0	2
DICER1	chr14	95570148/G//A	synonymous	0	2
RYR3	chr15	34021137/C//T	synonymous	0	2
SETD1A	chr16	30975492/C//T	synonymous	0	2
SLC12A3	chr16	56918001/G//A	synonymous	0	2
SLC52A1	chr17	4936433/A//AAC	nonsynonymous	0	2
KIF2B	chr17	51900881/C//T	nonsynonymous	0	2
CACNG1	chr17	65040805/G//A	nonsynonymous	0	2
SMAD2	chr18	45372045/T//A	nonsynonymous	0	2
CHST8	chr19	34263100/G//A	nonsynonymous	0	2
DNAAF3	chr19	55670471/C//T	nonsynonymous	0	2
RNF114	chr20	48568736/G//A	synonymous	0	2
TENM1	chrX	123657272/G//T	nonsynonymous	0	2
BCAP31	chrX	152967557/C//A	nonsynonymous	0	2
APC	chr5	112175482/GA//G	nonsynonymous	2	0
CCNL2	chr1	1323222/A//G	synonymous	0	2
MIB2	chr1	1558926/G//A	nonsynonymous	0	2
PEX10	chr1	2337971/G//A	synonymous	0	2
AJAP1	chr1	4772564/C//T	nonsynonymous	0	2
CHD5	chr1	6204126/A//G	nonsynonymous	0	2
CHD5	chr1	6214887/C//T	nonsynonymous	0	2
RPL22	chr1	6253081/C//T	nonsynonymous	0	2
	chr1	6947740/C//T	synonymous	0	2
CAMTA1	chr1	7811313/C//T	nonsynonymous	0	2
SLC2A7	chr1	9067389/G//A	nonsynonymous	0	2
C1orf127	chr1	11008316/G//A	nonsynonymous	0	2
ANGPTL7	chr1	11255039/C//T	nonsynonymous	0	2
MTOR	chr1	11319309/C//T	nonsynonymous	0	2
DISP3	chr1	11580795/G//A	nonsynonymous	0	2
DISP3	chr1	11595706/G//A	synonymous	0	2
VPS13D	chr1	12378269/G//A	nonsynonymous	0	2
AADACL3	chr1	12776258/C//A	nonsynonymous	0	2
PRAMEF12	chr1	12837662/C//T	nonsynonymous	0	2
PRAMEF2	chr1	12919856/T//C	nonsynonymous	0	2
PDPN	chr1	13910281/C//T	synonymous	0	2
PRDM2	chr1	14068567/G//A	nonsynonymous	0	2
PLEKHM2	chr1	16044389/C//T	synonymous	0	2
HSPB7	chr1	16342222/G//A	synonymous	0	2
FAM131C	chr1	16385073/C//T	synonymous	0	2
NECAP2	chr1	16774426/G//A	synonymous	0	2
CROCC	chr1	17272037/G//A	nonsynonymous	0	2
ARHGEF10L	chr1	17939588/C//T	synonymous	0	2
KLHDC7A	chr1	18809211/G//A	nonsynonymous	0	2
UBR4	chr1	19487516/T//G	nonsynonymous	0	2
PLA2G2F	chr1	20470022/G//A	nonsynonymous	0	2
KIF17	chr1	21014001/C//T	synonymous	0	2
EPHA8	chr1	22915661/G//A	nonsynonymous	0	2
GALE	chr1	24123628/C//T	nonsynonymous	0	2
MYOM3	chr1	24390605/G//A	synonymous	0	2
GRHL3	chr1	24669504/C//T	nonsynonymous	0	2
RHD	chr1	25627475/C//T	synonymous	0	2
UBXN11	chr1	26624477/G//T	synonymous	0	2
AIM1L	chr1	26672536/C//T	nonsynonymous	0	2
ARID1A	chr1	27106879/C//T	nonsynonymous	0	2
THEMIS2	chr1	28208733/C//T	nonsynonymous	0	2
THEMIS2	chr1	28209091/G//A	nonsynonymous	0	2
SMPDL3B	chr1	28285221/G//A	nonsynonymous	0	2
ADGRB2	chr1	32204909/C//A	nonsynonymous	0	2
ADGRB2	chr1	32207065/C//T	nonsynonymous	0	2
KHDRBS1	chr1	32503521/C//T	nonsynonymous	0	2
DCDC2B	chr1	32677773/G//T	nonsynonymous	0	2
NCDN	chr1	36024809/G//T	nonsynonymous	0	2
NCDN	chr1	36031117/C//T	synonymous	0	2
CLSPN	chr1	36209073/CAT//C	nonsynonymous	0	2
TEKT2	chr1	36553678/G//A	nonsynonymous	0	2
PPT1	chr1	40546147/G//A	synonymous	0	2

KCNQ4	chr1	41300686/G//A	nonsynonymous	0	2
	chr1	43149039/A//G	synonymous	0	2
P3H1	chr1	43212424/C//T	nonsynonymous	0	2
PTPRF	chr1	44057118/GGGCAGCCTGC//G	nonsynonymous	0	2
TCTEX1D4	chr1	45271700/G//A	nonsynonymous	0	2
MAST2	chr1	46489619/C//T	nonsynonymous	0	2
TTC39A	chr1	51753908/C//T	nonsynonymous	0	2
PRPF38A	chr1	52874248/C//T	nonsynonymous	0	2
ZYG11B	chr1	53236740/G//A	nonsynonymous	0	2
GLIS1	chr1	54060472/G//A	nonsynonymous	0	2
LRRC42	chr1	54417904/C//T	nonsynonymous	0	2
PLPP3	chr1	56990205/G//A	nonsynonymous	0	2
PATJ	chr1	62374152/C//T	nonsynonymous	0	2
PGM1	chr1	64120143/G//A	synonymous	0	2
LRRC7	chr1	70225906/A//T	nonsynonymous	0	2
LRRC7	chr1	70484416/C//T	synonymous	0	2
ZRANB2-AS1	chr1	71512328/C//T	synonymous	0	2
GBP5	chr1	89728086/T//C	nonsynonymous	0	2
LRRC8B	chr1	90049861/G//A	nonsynonymous	0	2
AGL	chr1	100356892/C//T	nonsynonymous	0	2
SASS6	chr1	100573550/C//T	nonsynonymous	0	2
VCAM1	chr1	101196794/C//T	synonymous	0	2
RNPC3	chr1	104068882/C//T	synonymous	0	2
KCNA3	chr1	111216388/G//A	synonymous	0	2
IGSF3	chr1	117119904/G//A	synonymous	0	2
GJA8	chr1	147380414/C//T	nonsynonymous	0	2
FAM63A	chr1	150969846/G//A	synonymous	0	2
THEM4	chr1	151861769/A//G	nonsynonymous	0	2
KPRP	chr1	152733509/C//T	nonsynonymous	0	2
CHRNB2	chr1	154548282/C//A	synonymous	0	2
PBXIP1	chr1	154920768/G//A	nonsynonymous	0	2
EFNA3	chr1	155058923/G//A	synonymous	0	2
THBS3	chr1	155175005/C//T	nonsynonymous	0	2
ASH1L	chr1	155313212/C//T	nonsynonymous	0	2
GON4L	chr1	155823276/AT//A	nonsynonymous	0	2
ARHGEF2	chr1	155931598/C//T	nonsynonymous	0	2
CD5L	chr1	157805813/C//T	nonsynonymous	0	2
OR10K1	chr1	158436255/C//T	nonsynonymous	0	2
MNDA	chr1	158817524/G//A	nonsynonymous	0	2
COPA	chr1	160293298/C//A	nonsynonymous	0	2
ITLN2	chr1	160914916/C//T	synonymous	0	2
DDR2	chr1	162731065/G//A	nonsynonymous	0	2
LMX1A	chr1	165218816/G//A	nonsynonymous	0	2
POU2F1	chr1	167381535/C//T	nonsynonymous	0	2
ADCY10	chr1	167829118/C//T	nonsynonymous	0	2
CCDC181	chr1	169391241/G//A	nonsynonymous	0	2
SELP	chr1	169565287/C//T	synonymous	0	2
	chr1	170983403/T//C	synonymous	0	2
PRRC2C	chr1	171501663/G//T	nonsynonymous	0	2
TNN	chr1	175087702/G//A	nonsynonymous	0	2
PAPPA2	chr1	176563812/C//T	nonsynonymous	0	2
PAPPA2	chr1	176668468/A//G	synonymous	0	2
CEP350	chr1	180012272/C//T	nonsynonymous	0	2
MR1	chr1	181018344/A//G	nonsynonymous	0	2
TSEN15	chr1	184023541/G//A	synonymous	0	2
HMCN1	chr1	185969203/T//C	synonymous	0	2
HMCN1	chr1	186114603/C//T	nonsynonymous	0	2
BRINP3	chr1	190068141/G//A	synonymous	0	2
BRINP3	chr1	190129925/G//A	nonsynonymous	0	2
CFHR3	chr1	196749055/A//G	nonsynonymous	0	2
ZBTB41	chr1	197168659/A//C	nonsynonymous	0	2
CRB1	chr1	197297729/C//T	nonsynonymous	0	2
PTPRC	chr1	198723415/C//T	nonsynonymous	0	2
CSRP1	chr1	201465312/G//A	synonymous	0	2
NAV1	chr1	201618293/C//A	nonsynonymous	0	2
KLHL12	chr1	202887311/G//A	synonymous	0	2
ADORA1	chr1	203097939/C//T	synonymous	0	2
PPP1R15B	chr1	204379124/A//G	synonymous	0	2
NFASC	chr1	204923462/G//T	nonsynonymous	0	2
TMCC2	chr1	205210948/A//G	nonsynonymous	0	2

PIGR	chr1	207107907/G//A	synonymous	0	2
NEK2	chr1	211848846/C//T	synonymous	0	2
KCNK2	chr1	215259772/G//A	synonymous	0	2
LYPLAL1	chr1	219383902/A//G	synonymous	0	2
TLR5	chr1	223284383/C//T	nonsynonymous	0	2
FBXO28	chr1	224345314/C//T	nonsynonymous	0	2
EPHX1	chr1	226019589/G//A	nonsynonymous	0	2
LEFTY1	chr1	226074635/C//T	nonsynonymous	0	2
ACBD3	chr1	226349343/C//T	nonsynonymous	0	2
MIXL1	chr1	226413369/C//T	synonymous	0	2
OBSCN	chr1	228404751/C//T	synonymous	0	2
OBSCN	chr1	228482568/C//T	nonsynonymous	0	2
HIST3H2A	chr1	228645529/A//C	synonymous	0	2
COG2	chr1	230827260/G//A	nonsynonymous	0	2
SIPA1L2	chr1	232649812/G//A	nonsynonymous	0	2
PCNX2	chr1	233314840/G//A	nonsynonymous	0	2
KCNK1	chr1	233802496/C//T	nonsynonymous	0	2
NID1	chr1	236144919/G//A	synonymous	0	2
NID1	chr1	236205344/G//A	nonsynonymous	0	2
NID1	chr1	236212188/C//T	synonymous	0	2
CHRM3	chr1	240071457/G//A	nonsynonymous	0	2
AKT3	chr1	243716231/A//T	nonsynonymous	0	2
KIF26B	chr1	245848872/G//A	nonsynonymous	0	2
CNST	chr1	246811011/C//T	nonsynonymous	0	2
NLRP3	chr1	247607348/C//T	nonsynonymous	0	2
OR2T8	chr1	248084484/G//A	synonymous	0	2
OR2L3	chr1	248224907/C//T	synonymous	0	2
OR2L13	chr1	248262870/C//T	nonsynonymous	0	2
OR2G6	chr1	248685134/C//A	nonsynonymous	0	2
PXDN	chr2	1637944/A//C	synonymous	0	2
PXDN	chr2	1653244/C//T	nonsynonymous	0	2
LPIN1	chr2	11955273/G//A	nonsynonymous	0	2
SMC6	chr2	17919559/G//A	synonymous	0	2
HS1BP3	chr2	20818792/G//A	synonymous	0	2
ADCY3	chr2	25054533/G//A	nonsynonymous	0	2
DNMT3A	chr2	25467450/C//A	synonymous	0	2
C2orf70	chr2	26798783/G//A	nonsynonymous	0	2
KCNK3	chr2	26951259/C//T	synonymous	0	2
DPYSL5	chr2	27167644/C//T	nonsynonymous	0	2
DPYSL5	chr2	27169897/C//T	synonymous	0	2
PREB	chr2	27354543/G//A	nonsynonymous	0	2
CAD	chr2	27465631/C//T	synonymous	0	2
SLC30A3	chr2	27480097/C//T	synonymous	0	2
FOSL2	chr2	28635015/A//G	synonymous	0	2
PLB1	chr2	28812936/C//T	nonsynonymous	0	2
ALK	chr2	29436950/G//A	synonymous	0	2
ALK	chr2	29498070/G//A	nonsynonymous	0	2
CAPN13	chr2	30980996/T//C	nonsynonymous	0	2
BIRC6	chr2	32664600/C//T	nonsynonymous	0	2
FEZ2	chr2	36818091/A//G	synonymous	0	2
DHX57	chr2	39070320/C//T	nonsynonymous	0	2
ABCG8	chr2	44099258/G//A	nonsynonymous	0	2
PRKCE	chr2	45879483/G//A	nonsynonymous	0	2
EPAS1	chr2	46605103/G//A	synonymous	0	2
	chr2	47397926/G//T	synonymous	0	2
SPTBN1	chr2	54852032/G//A	nonsynonymous	0	2
EML6	chr2	55191850/C//T	nonsynonymous	0	2
OTX1	chr2	63283112/C//T	synonymous	0	2
PNO1	chr2	68385138/C//T	synonymous	0	2
FIGLA	chr2	71017615/G//A	synonymous	0	2
VAX2	chr2	71148369/G//A	nonsynonymous	0	2
MPHOSPH10	chr2	71371667/C//T	nonsynonymous	0	2
DYSF	chr2	71740898/G//A	synonymous	0	2
SLC4A5	chr2	74474229/T//C	nonsynonymous	0	2
SLC4A5	chr2	74492333/C//T	nonsynonymous	0	2
MRPL53	chr2	74699326/G//A	nonsynonymous	0	2
SEMA4F	chr2	74907289/C//T	nonsynonymous	0	2
CTNNA2	chr2	80801387/G//A	nonsynonymous	0	2
DNAH6	chr2	84777082/G//A	synonymous	0	2
RETSAT	chr2	85571732/G//A	nonsynonymous	0	2

SH2D6	chr2	85663600/C//T	synonymous	0	2
KDM3A	chr2	86677038/C//T	nonsynonymous	0	2
FABP1	chr2	88425744/G//A	nonsynonymous	0	2
PROM2	chr2	95941838/C//T	nonsynonymous	0	2
ASTL	chr2	96795829/G//A	synonymous	0	2
FER1L5	chr2	97361561/C//T	nonsynonymous	0	2
CNNM4	chr2	97427884/G//T	nonsynonymous	0	2
ACTR1B	chr2	98275383/G//A	synonymous	0	2
ACTR1B	chr2	98275853/C//T	nonsynonymous	0	2
ZAP70	chr2	98340516/C//T	nonsynonymous	0	2
NCK2	chr2	106498388/C//T	synonymous	0	2
ST6GAL2	chr2	107423228/G//A	nonsynonymous	0	2
BUB1	chr2	111430269/C//T	nonsynonymous	0	2
FBLN7	chr2	112922609/C//T	synonymous	0	2
SLC20A1	chr2	113417093/A//G	nonsynonymous	0	2
STEAP3	chr2	120020700/C//T	nonsynonymous	0	2
TFCP2L1	chr2	122038782/C//T	nonsynonymous	0	2
CLASP1	chr2	122227441/C//T	nonsynonymous	0	2
MYO7B	chr2	128334255/C//T	nonsynonymous	0	2
MYO7B	chr2	128381686/G//A	nonsynonymous	0	2
WDR33	chr2	128477132/C//T	nonsynonymous	0	2
POLR2D	chr2	128610503/G//A	nonsynonymous	0	2
GPR39	chr2	133402902/G//A	nonsynonymous	0	2
TMEM163	chr2	135308176/G//A	synonymous	0	2
LCT	chr2	136590758/C//T	nonsynonymous	0	2
THSD7B	chr2	137988629/G//A	nonsynonymous	0	2
LRP1B	chr2	141143453/GTTAC//G	synonymous	0	2
KIF5C	chr2	149866663/C//T	synonymous	0	2
NEB	chr2	152482152/G//A	nonsynonymous	0	2
NEB	chr2	152484315/C//T	nonsynonymous	0	2
ARL5A	chr2	152684745/G//T	synonymous	0	2
FMNL2	chr2	153475605/C//T	synonymous	0	2
TANC1	chr2	160007039/C//A	synonymous	0	2
TANC1	chr2	160035450/C//T	synonymous	0	2
TANC1	chr2	160087070/C//T	synonymous	0	2
COBLL1	chr2	165551036/C//T	nonsynonymous	0	2
CSRNP3	chr2	166536026/C//T	synonymous	0	2
XIRP2	chr2	168099276/C//T	synonymous	0	2
LRP2	chr2	170025181/G//A	nonsynonymous	0	2
DLX2	chr2	172967148/T//C	nonsynonymous	0	2
ITGA6	chr2	173349898/A//G	nonsynonymous	0	2
PDK1	chr2	173423568/T//C	nonsynonymous	0	2
TTN	chr2	179404240/C//T	nonsynonymous	0	2
TTN	chr2	179436431/C//T	nonsynonymous	0	2
TTN	chr2	179438273/G//A	nonsynonymous	0	2
TTN	chr2	179594237/C//T	nonsynonymous	0	2
	chr2	179614378/G//T	synonymous	0	2
TTN	chr2	179666963/G//A	nonsynonymous	0	2
ZNF804A	chr2	185803801/A//T	synonymous	0	2
	chr2	189454467/C//T	synonymous	0	2
COL5A2	chr2	189898939/G//A	nonsynonymous	0	2
PGAP1	chr2	197767333/A//G	synonymous	0	2
SATB2	chr2	200213744/G//T	nonsynonymous	0	2
ICA1L	chr2	203684477/C//T	nonsynonymous	0	2
	chr2	210569351/C//T	synonymous	0	2
XRCC5	chr2	217055041/G//A	nonsynonymous	0	2
TNS1	chr2	218713695/C//T	synonymous	0	2
TNS1	chr2	218745630/C//T	nonsynonymous	0	2
GPBAR1	chr2	219127539/C//T	nonsynonymous	0	2
BCS1L	chr2	219527335/G//A	synonymous	0	2
STK36	chr2	219545354/C//T	nonsynonymous	0	2
CDK5R2	chr2	219825185/G//A	nonsynonymous	0	2
CHPF	chr2	220404386/C//T	nonsynonymous	0	2
PAX3	chr2	223085058/C//T	nonsynonymous	0	2
SGPP2	chr2	223423242/G//A	synonymous	0	2
SCG2	chr2	224463321/G//A	nonsynonymous	0	2
SERPINE2	chr2	224856526/G//A	nonsynonymous	0	2
NYAP2	chr2	226273739/G//A	nonsynonymous	0	2
IRS1	chr2	227660794/A//G	synonymous	0	2
SLC19A3	chr2	228563732/G//A	synonymous	0	2

SPATA3	chr2	231867434/C//T	nonsynonymous	0	2
ECEL1	chr2	233344842/C//T	synonymous	0	2
DGKD	chr2	234358738/G//A	nonsynonymous	0	2
RBM44	chr2	238726253/A//G	nonsynonymous	0	2
PER2	chr2	239164301/G//A	nonsynonymous	0	2
HDAC4	chr2	240002870/C//T	nonsynonymous	0	2
GPC1	chr2	241401664/G//A	nonsynonymous	0	2
GPC1	chr2	241402778/G//A	synonymous	0	2
	chr2	241500512/G//T	synonymous	0	2
RNPEPL1	chr2	241516004/G//A	synonymous	0	2
SNED1	chr2	242009624/C//T	synonymous	0	2
IL5RA	chr3	3139842/G//A	nonsynonymous	0	2
SRGAP3	chr3	9036074/G//A	synonymous	0	2
TADA3	chr3	9821949/C//T	synonymous	0	2
ATP2B2	chr3	10392245/C//T	nonsynonymous	0	2
ATP2B2	chr3	10420024/G//A	synonymous	0	2
GRIP2	chr3	14581777/C//T	synonymous	0	2
NR2C2	chr3	15064742/C//T	nonsynonymous	0	2
HACL1	chr3	15643210/C//A	synonymous	0	2
KCNH8	chr3	19295378/C//T	synonymous	0	2
OXSM	chr3	25833095/G//A	nonsynonymous	0	2
TGFBR2	chr3	30713601/C//T	nonsynonymous	0	2
GPD1L	chr3	32200587/G//A	nonsynonymous	0	2
TMPPE	chr3	33134910/C//T	nonsynonymous	0	2
TRANK1	chr3	36898755/G//A	nonsynonymous	0	2
TRANK1	chr3	36898892/G//A	nonsynonymous	0	2
ITGA9	chr3	37514923/G//A	nonsynonymous	0	2
DLEC1	chr3	38150955/C//T	synonymous	0	2
HHATL	chr3	42739841/G//A	synonymous	0	2
TOPAZ1	chr3	44283818/G//A	synonymous	0	2
TGM4	chr3	44945458/A//G	nonsynonymous	0	2
CDCP1	chr3	45152127/A//T	nonsynonymous	0	2
LZTFL1	chr3	45877152/G//A	nonsynonymous	0	2
CCR9	chr3	45943064/G//A	nonsynonymous	0	2
SCAP	chr3	47456657/G//A	nonsynonymous	0	2
SCAP	chr3	47462192/G//A	nonsynonymous	0	2
PLXNB1	chr3	48459679/C//T	nonsynonymous	0	2
CELSR3	chr3	48697683/G//A	synonymous	0	2
LAMB2	chr3	49160340/C//T	nonsynonymous	0	2
BSN	chr3	49680058/G//A	nonsynonymous	0	2
APEH	chr3	49713615/A//G	nonsynonymous	0	2
MST1	chr3	49721510/C//T	nonsynonymous	0	2
UBA7	chr3	49850517/C//T	nonsynonymous	0	2
TRAIP	chr3	49869404/G//A	nonsynonymous	0	2
SLC38A3	chr3	50255226/A//G	nonsynonymous	0	2
SEMA3B	chr3	50313252/G//A	nonsynonymous	0	2
NAT6	chr3	50334402/G//A	nonsynonymous	0	2
GRM2	chr3	51749927/G//A	nonsynonymous	0	2
POC1A	chr3	52130607/C//T	nonsynonymous	0	2
TLR9	chr3	52255688/C//T	nonsynonymous	0	2
DNAH1	chr3	52417963/G//A	synonymous	0	2
GLT8D1	chr3	52730292/C//T	nonsynonymous	0	2
ITIH3	chr3	52840156/T//G	nonsynonymous	0	2
SLMAP	chr3	57817251/C//T	nonsynonymous	0	2
FLNB	chr3	58092419/A//T	nonsynonymous	0	2
ADAMTS9	chr3	64527073/C//T	nonsynonymous	0	2
SUCLG2	chr3	67426243/G//A	synonymous	0	2
MITF	chr3	69928359/G//A	nonsynonymous	0	2
GBE1	chr3	81548277/C//T	nonsynonymous	0	2
MINA	chr3	97686349/G//T	nonsynonymous	0	2
ADGRG7	chr3	100328717/C//T	nonsynonymous	0	2
ZBTB11	chr3	101384051/T//C	nonsynonymous	0	2
BOC	chr3	112991274/G//A	nonsynonymous	0	2
ZDHHC23	chr3	113679644/C//T	synonymous	0	2
ARHGAP31	chr3	119133521/G//A	synonymous	0	2
TIMMDC1	chr3	119236129/G//A	nonsynonymous	0	2
GSK3B	chr3	119562094/G//A	synonymous	0	2
KALRN	chr3	124415056/A//G	synonymous	0	2
CHST13	chr3	126261410/C//T	nonsynonymous	0	2
MGLL	chr3	127413938/G//A	synonymous	0	2

EEFSEC	chr3	128060483/C//T	synonymous	0	2
TF	chr3	133473497/C//T	nonsynonymous	0	2
NME9	chr3	138023764/C//T	nonsynonymous	0	2
PRR23B	chr3	138739249/C//T	synonymous	0	2
NMNAT3	chr3	139280119/G//A	synonymous	0	2
TRPC1	chr3	142496524/T//C	nonsynonymous	0	2
CPB1	chr3	148575288/C//T	synonymous	0	2
CPA3	chr3	148614490/C//T	nonsynonymous	0	2
HLTF	chr3	148768095/C//T	nonsynonymous	0	2
IGSF10	chr3	151154752/G//A	nonsynonymous	0	2
P2RY1	chr3	152553832/C//T	synonymous	0	2
SHOX2	chr3	157815959/G//A	nonsynonymous	0	2
SLC7A14	chr3	170184995/C//T	nonsynonymous	0	2
SPATA16	chr3	172674522/T//C	synonymous	0	2
ZNF639	chr3	179051896/C//T	nonsynonymous	0	2
ACTL6A	chr3	179287968/T//C	synonymous	0	2
KLHL6	chr3	183226233/C//T	nonsynonymous	0	2
PARL	chr3	183551527/C//T	synonymous	0	2
DVL3	chr3	183887975/C//T	synonymous	0	2
DVL3	chr3	183888005/C//T	synonymous	0	2
ABCF3	chr3	183905738/G//A	nonsynonymous	0	2
PSMD2	chr3	184018068/GAGA//G	nonsynonymous	0	2
CHRD	chr3	184099602/C//T	nonsynonymous	0	2
TP63	chr3	189587113/C//T	nonsynonymous	0	2
CCDC50	chr3	191075810/G//A	nonsynonymous	0	2
	chr3	191093190/G//A	synonymous	0	2
	chr3	191093296/A//C	synonymous	0	2
FGF12	chr3	192126114/C//T	synonymous	0	2
SMCO1	chr3	196242161/G//A	synonymous	0	2
PAK2	chr3	196554136/C//T	nonsynonymous	0	2
RUBCN	chr3	197421364/T//C	synonymous	0	2
LMLN	chr3	197703506/G//A	nonsynonymous	0	2
PDE6B	chr4	619806/G//A	nonsynonymous	0	2
RGS12	chr4	3317925/C//T	nonsynonymous	0	2
WFS1	chr4	6303619/G//A	synonymous	0	2
MAN2B2	chr4	6588854/C//T	nonsynonymous	0	2
SORCS2	chr4	7738820/C//T	synonymous	0	2
ABLIM2	chr4	8031410/G//A	nonsynonymous	0	2
ACOX3	chr4	8394094/G//A	synonymous	0	2
TRMT44	chr4	8448217/A//G	nonsynonymous	0	2
ZNF518B	chr4	10445109/G//A	synonymous	0	2
C1QTNF7	chr4	15444220/G//T	nonsynonymous	0	2
CC2D2A	chr4	15517628/G//A	nonsynonymous	0	2
FAM184B	chr4	17638188/C//T	nonsynonymous	0	2
SOD3	chr4	24801217/C//T	nonsynonymous	0	2
PDGFRA	chr4	55144660/C//A	nonsynonymous	0	2
PPAT	chr4	57272846/C//T	nonsynonymous	0	2
NPFFR2	chr4	73013562/C//T	synonymous	0	2
ANKRD17	chr4	73959897/T//C	synonymous	0	2
USO1	chr4	76695935/G//T	nonsynonymous	0	2
WDFY3	chr4	85599417/C//T	nonsynonymous	0	2
WDFY3	chr4	85781647/G//A	nonsynonymous	0	2
GPRIN3	chr4	90170733/C//T	nonsynonymous	0	2
PDLIM5	chr4	95496971/G//A	nonsynonymous	0	2
SGMS2	chr4	108820744/C//T	nonsynonymous	0	2
AP1AR	chr4	113184239/G//A	synonymous	0	2
ANK2	chr4	114276425/C//T	synonymous	0	2
ARSJ	chr4	114823957/T//C	nonsynonymous	0	2
ADAD1	chr4	123333813/T//C	synonymous	0	2
FAT4	chr4	126238049/C//T	synonymous	0	2
SLC25A31	chr4	128694556/C//T	nonsynonymous	0	2
JADE1	chr4	129783065/G//A	synonymous	0	2
FREM3	chr4	144621516/G//A	nonsynonymous	0	2
POU4F2	chr4	147561183/G//A	synonymous	0	2
FAM160A1	chr4	152577524/A//G	nonsynonymous	0	2
DCHS2	chr4	155254007/G//A	nonsynonymous	0	2
GUCY1B3	chr4	156721041/G//A	synonymous	0	2
CTSO	chr4	156850723/G//A	nonsynonymous	0	2
AADAT	chr4	171008289/C//T	nonsynonymous	0	2
TENM3	chr4	183721222/C//T	synonymous	0	2

STOX2	chr4	184930976/C//T	nonsynonymous	0	2
PLEKHG4B	chr5	156331/C//T	synonymous	0	2
SLC6A18	chr5	1232881/C//T	nonsynonymous	0	2
LPCAT1	chr5	1474142/G//A	nonsynonymous	0	2
LPCAT1	chr5	1494867/C//T	synonymous	0	2
ICE1	chr5	5489358/G//A	nonsynonymous	0	2
MTRR	chr5	7885828/C//A	synonymous	0	2
CTNND2	chr5	11117604/G//A	synonymous	0	2
TRIO	chr5	14368882/C//T	synonymous	0	2
TRIO	chr5	14492757/G//A	nonsynonymous	0	2
FAM105A	chr5	14610433/G//A	synonymous	0	2
PRDM9	chr5	23524457/C//T	nonsynonymous	0	2
DROSHA	chr5	31483682/G//A	nonsynonymous	0	2
DROSHA	chr5	31526322/G//A	nonsynonymous	0	2
DNAJC21	chr5	34937648/C//T	nonsynonymous	0	2
GDNF	chr5	37815852/G//A	synonymous	0	2
ZNF131	chr5	43161739/C//T	synonymous	0	2
ITGA2	chr5	52344280/T//C	nonsynonymous	0	2
HSPB3	chr5	53751772/G//A	synonymous	0	2
ZSWIM6	chr5	60837776/G//A	synonymous	0	2
DIMT1	chr5	61694568/T//C	nonsynonymous	0	2
SREK1	chr5	65460650/G//A	nonsynonymous	0	2
SREK1	chr5	65465922/C//T	nonsynonymous	0	2
ARHGEF28	chr5	73190300/C//T	synonymous	0	2
IQGAP2	chr5	75991425/G//A	synonymous	0	2
WDR41	chr5	76734101/T//C	nonsynonymous	0	2
JMY	chr5	78610649/T//C	synonymous	0	2
CMYA5	chr5	79025998/C//T	synonymous	0	2
MSH3	chr5	80150074/G//A	nonsynonymous	0	2
HAPLN1	chr5	82937450/C//T	synonymous	0	2
ELL2	chr5	95234256/G//A	nonsynonymous	0	2
WDR36	chr5	110432816/A//G	nonsynonymous	0	2
WDR36	chr5	110443087/C//T	synonymous	0	2
MCC	chr5	112389496/C//T	nonsynonymous	0	2
PRR16	chr5	120022211/C//T	nonsynonymous	0	2
PHAX	chr5	125939766/C//T	nonsynonymous	0	2
MEGF10	chr5	126732228/C//T	synonymous	0	2
FBN2	chr5	127674668/G//A	synonymous	0	2
CXCL14	chr5	134910258/G//A	synonymous	0	2
PCDHA1	chr5	140167558/C//T	synonymous	0	2
PCDHA7	chr5	140215534/G//A	synonymous	0	2
PCDHA10	chr5	140237744/C//T	nonsynonymous	0	2
PCDHA12	chr5	140257249/G//A	nonsynonymous	0	2
PCDHA13	chr5	140264013/G//A	synonymous	0	2
PCDHB2	chr5	140476467/C//T	nonsynonymous	0	2
PCDHB7	chr5	140553938/G//A	nonsynonymous	0	2
PCDHB11	chr5	140580989/C//T	nonsynonymous	0	2
PCDHB12	chr5	140590500/C//T	nonsynonymous	0	2
SLC25A2	chr5	140683097/C//T	synonymous	0	2
PCDHGB5	chr5	140778596/A//G	nonsynonymous	0	2
PCDHGC5	chr5	140869635/A//G	synonymous	0	2
ARAP3	chr5	141033935/G//T	nonsynonymous	0	2
KIAA0141	chr5	141318214/C//A	nonsynonymous	0	2
PCDH12	chr5	141335611/G//A	synonymous	0	2
ARHGAP26	chr5	142311642/G//A	synonymous	0	2
GRXCR2	chr5	145252474/G//A	nonsynonymous	0	2
PPP2R2B	chr5	145969776/C//A	nonsynonymous	0	2
PPARGC1B	chr5	149216434/C//A	nonsynonymous	0	2
CSF1R	chr5	149441165/C//T	synonymous	0	2
GALNT10	chr5	153795373/C//T	nonsynonymous	0	2
LARP1	chr5	154179594/C//T	nonsynonymous	0	2
PWWP2A	chr5	159520917/G//A	nonsynonymous	0	2
C1QTNF2	chr5	159776682/G//A	synonymous	0	2
ATP10B	chr5	160097657/A//G	nonsynonymous	0	2
WWC1	chr5	167896016/C//T	synonymous	0	2
RARS	chr5	167922324/T//C	nonsynonymous	0	2
DOCK2	chr5	169141391/G//A	nonsynonymous	0	2
DRD1	chr5	174869160/C//T	nonsynonymous	0	2
EIF4E1B	chr5	176072983/G//T	synonymous	0	2
HK3	chr5	176315758/A//G	nonsynonymous	0	2

LMAN2	chr5	176761318/C//T	nonsynonymous	0	2
B4GALT7	chr5	177035600/C//T	nonsynonymous	0	2
CLK4	chr5	178045682/T//C	nonsynonymous	0	2
GRM6	chr5	178413949/C//T	nonsynonymous	0	2
SQSTM1	chr5	179251192/A//G	nonsynonymous	0	2
IRF4	chr6	395874/C//T	nonsynonymous	0	2
ECI2	chr6	4117645/C//T	nonsynonymous	0	2
PPP1R3G	chr6	5086716/T//C	nonsynonymous	0	2
HFE	chr6	26091636/C//T	synonymous	0	2
HIST1H1E	chr6	26156605/C//T	synonymous	0	2
HIST1H2AG	chr6	27100868/G//A	synonymous	0	2
ZSCAN26	chr6	28239827/G//T	nonsynonymous	0	2
MAS1L	chr6	29455256/C//T	nonsynonymous	0	2
PRRC2A	chr6	31603002/C//T	nonsynonymous	0	2
CFB	chr6	31915554/G//A	nonsynonymous	0	2
TNXB	chr6	32038046/G//A	synonymous	0	2
HLA-DOB	chr6	32782846/G//A	synonymous	0	2
SYNGAP1	chr6	33405668/G//A	nonsynonymous	0	2
ZBTB9	chr6	33423975/C//T	synonymous	0	2
IP6K3	chr6	33694610/C//T	nonsynonymous	0	2
ANKS1A	chr6	34985468/G//A	nonsynonymous	0	2
ANKS1A	chr6	35047660/G//A	synonymous	0	2
DEF6	chr6	35280418/C//T	nonsynonymous	0	2
CLPS	chr6	35762986/G//A	synonymous	0	2
CMTR1	chr6	37440230/C//T	synonymous	0	2
ZFAND3	chr6	38029481/G//A	synonymous	0	2
TFEB	chr6	41658904/C//T	synonymous	0	2
PPP2R5D	chr6	42978460/C//T	nonsynonymous	0	2
MEA1	chr6	42980911/ACCT//A	nonsynonymous	0	2
TTBK1	chr6	43251044/G//A	nonsynonymous	0	2
POLR1C	chr6	43488496/G//T	nonsynonymous	0	2
TMEM151B	chr6	44243253/G//A	synonymous	0	2
TDRD6	chr6	46655966/G//T	nonsynonymous	0	2
ANKRD66	chr6	46721477/G//A	nonsynonymous	0	2
ADGRF5	chr6	46834818/C//T	nonsynonymous	0	2
ADGRF1	chr6	46984453/A//G	synonymous	0	2
OPN5	chr6	47763121/C//T	nonsynonymous	0	2
TFAP2B	chr6	50810989/G//A	nonsynonymous	0	2
MCM3	chr6	52144299/G//A	synonymous	0	2
GCM1	chr6	52999120/G//A	synonymous	0	2
LRRC1	chr6	53778689/G//A	nonsynonymous	0	2
KCNQ5	chr6	73904551/C//T	nonsynonymous	0	2
CD109	chr6	74519763/C//T	nonsynonymous	0	2
MDN1	chr6	90382036/C//A	nonsynonymous	0	2
CASP8AP2	chr6	90578058/C//T	synonymous	0	2
TSTD3	chr6	99979245/C//T	nonsynonymous	0	2
SIM1	chr6	100868667/T//A	nonsynonymous	0	2
SIM1	chr6	100895212/G//A	synonymous	0	2
SCML4	chr6	108042146/C//T	nonsynonymous	0	2
SEC63	chr6	108224188/C//T	nonsynonymous	0	2
ARMC2	chr6	109274427/G//A	synonymous	0	2
MFSD4B	chr6	111587534/A//G	nonsynonymous	0	2
GOPC	chr6	117894771/T//C	synonymous	0	2
TPD52L1	chr6	125583981/C//T	nonsynonymous	0	2
MED23	chr6	131923402/T//C	nonsynonymous	0	2
IFNGR1	chr6	137519545/C//T	nonsynonymous	0	2
NHSL1	chr6	138745241/C//T	nonsynonymous	0	2
NHSL1	chr6	138745520/C//T	nonsynonymous	0	2
UTRN	chr6	144794859/G//A	nonsynonymous	0	2
PLEKHG1	chr6	151152558/C//A	nonsynonymous	0	2
AKAP12	chr6	151671536/G//A	synonymous	0	2
SYNE1	chr6	152651168/C//T	synonymous	0	2
SYNE1	chr6	152688391/C//T	nonsynonymous	0	2
TAGAP	chr6	159457414/G//A	synonymous	0	2
GPR31	chr6	167570665/G//A	nonsynonymous	0	2
ERMARD	chr6	170176775/C//T	synonymous	0	2
ERMARD	chr6	170181558/A//C	nonsynonymous	0	2
DLL1	chr6	170597536/G//A	nonsynonymous	0	2
DNAAF5	chr7	814702/G//A	synonymous	0	2
GPER1	chr7	1131813/G//T	nonsynonymous	0	2

GPER1	chr7	1132095/C//T	nonsynonymous	0	2
INTS1	chr7	1518071/C//T	nonsynonymous	0	2
INTS1	chr7	1542763/C//T	synonymous	0	2
CARD11	chr7	2946339/C//T	nonsynonymous	0	2
FBXL18	chr7	5540809/C//T	nonsynonymous	0	2
FBXL18	chr7	5541425/C//T	nonsynonymous	0	2
USP42	chr7	6193714/G//A	synonymous	0	2
FAM220A	chr7	6370486/C//T	synonymous	0	2
C7orf26	chr7	6639512/T//C	synonymous	0	2
GLCCI1	chr7	8126132/G//T	nonsynonymous	0	2
VWDE	chr7	12409215/G//A	nonsynonymous	0	2
DGKB	chr7	14724985/G//A	synonymous	0	2
HDAC9	chr7	18687629/A//G	synonymous	0	2
MACC1	chr7	20201439/T//G	nonsynonymous	0	2
NFE2L3	chr7	26224957/A//G	nonsynonymous	0	2
HOXA1	chr7	27135386/C//T	nonsynonymous	0	2
	chr7	29519791/A//G	synonymous	0	2
NOD1	chr7	30492035/C//T	nonsynonymous	0	2
CRHR2	chr7	30695572/C//T	synonymous	0	2
BMPER	chr7	34118464/C//T	synonymous	0	2
EEPD1	chr7	36338647/G//A	synonymous	0	2
AOAH	chr7	36571781/T//C	nonsynonymous	0	2
AMPH	chr7	38431481/C//T	synonymous	0	2
HECW1	chr7	43447306/C//T	synonymous	0	2
AEBP1	chr7	44152298/G//A	nonsynonymous	0	2
NPC1L1	chr7	44578946/C//T	synonymous	0	2
MYO1G	chr7	45007334/G//A	nonsynonymous	0	2
CCM2	chr7	45104079/G//A	synonymous	0	2
ADCY1	chr7	45632428/G//A	nonsynonymous	0	2
UPP1	chr7	48141448/C//T	nonsynonymous	0	2
ABCA13	chr7	48390322/C//A	synonymous	0	2
POM121L12	chr7	53103907/G//A	synonymous	0	2
PSPH	chr7	56087301/G//A	synonymous	0	2
TRIM50	chr7	72734178/C//T	nonsynonymous	0	2
FZD9	chr7	72848989/G//A	nonsynonymous	0	2
GTF2IRD1	chr7	73932543/G//A	nonsynonymous	0	2
GTF2IRD1	chr7	74005220/C//T	nonsynonymous	0	2
TMEM120A	chr7	75617060/G//A	nonsynonymous	0	2
MAGI2	chr7	77762214/G//A	synonymous	0	2
SRI	chr7	87838694/G//A	synonymous	0	2
GTPBP10	chr7	90014301/G//A	nonsynonymous	0	2
AKAP9	chr7	91708659/C//T	synonymous	0	2
LMTK2	chr7	97834784/G//A	nonsynonymous	0	2
TECPR1	chr7	97860657/A//G	nonsynonymous	0	2
NPTX2	chr7	98254445/C//T	synonymous	0	2
TRRAP	chr7	98606091/C//T	synonymous	0	2
SMURF1	chr7	98643415/G//A	nonsynonymous	0	2
NYAP1	chr7	100086994/C//A	synonymous	0	2
ACTL6B	chr7	100246418/C//T	nonsynonymous	0	2
ZAN	chr7	100385658/G//A	nonsynonymous	0	2
CUX1	chr7	101845347/A//G	nonsynonymous	0	2
PIK3CG	chr7	106509677/G//A	synonymous	0	2
SLC26A3	chr7	107417074/T//A	synonymous	0	2
LAMB1	chr7	107601664/G//A	nonsynonymous	0	2
PPP1R3A	chr7	113519199/G//T	nonsynonymous	0	2
MET	chr7	116414986/C//A	nonsynonymous	0	2
CTTNBP2	chr7	117431724/C//A	nonsynonymous	0	2
AASS	chr7	121741474/C//T	nonsynonymous	0	2
CADPS2	chr7	122027158/G//A	synonymous	0	2
GPR37	chr7	124404177/A//T	nonsynonymous	0	2
OPN1SW	chr7	128414648/G//A	synonymous	0	2
TSPAN33	chr7	128806708/C//T	synonymous	0	2
SMO	chr7	128851571/A//G	nonsynonymous	0	2
PLXNA4	chr7	131825574/C//T	synonymous	0	2
PLXNA4	chr7	131895797/G//A	nonsynonymous	0	2
ATP6V0A4	chr7	138430005/G//A	synonymous	0	2
CLEC2L	chr7	139221053/C//T	nonsynonymous	0	2
BRAF	chr7	140501350/G//A	nonsynonymous	0	2
PRSS1	chr7	142459627/G//A	nonsynonymous	0	2
TRPV6	chr7	142573641/A//G	nonsynonymous	0	2

TRPV5	chr7	142626668/A//G	synonymous	0	2
CLCN1	chr7	143027948/G//A	nonsynonymous	0	2
ZYX	chr7	143087676/C//T	synonymous	0	2
ZNF212	chr7	148951153/A//G	nonsynonymous	0	2
SSPO	chr7	149497422/G//A	nonsynonymous	0	2
ASIC3	chr7	150746024/C//T	nonsynonymous	0	2
SLC4A2	chr7	150772423/C//T	synonymous	0	2
ABCF2	chr7	150920903/G//A	nonsynonymous	0	2
ZNF596	chr8	192995/A//G	nonsynonymous	0	2
CSMD1	chr8	3000088/G//A	nonsynonymous	0	2
AGPAT5	chr8	6614816/C//T	synonymous	0	2
MSRA	chr8	10285674/G//A	nonsynonymous	0	2
C8orf74	chr8	10558013/G//A	synonymous	0	2
NEIL2	chr8	11637363/G//A	nonsynonymous	0	2
DLC1	chr8	12957249/C//T	nonsynonymous	0	2
INTS10	chr8	19690804/C//T	nonsynonymous	0	2
ATP6V1B2	chr8	20068778/G//A	nonsynonymous	0	2
DOK2	chr8	21767026/G//A	synonymous	0	2
SORBS3	chr8	22428640/G//A	nonsynonymous	0	2
DOCK5	chr8	25159867/G//A	synonymous	0	2
SCARA3	chr8	27516497/C//A	synonymous	0	2
KIF13B	chr8	28974353/G//A	nonsynonymous	0	2
DCTN6	chr8	30038139/G//A	nonsynonymous	0	2
WRN	chr8	31014947/G//A	nonsynonymous	0	2
	chr8	32600250/C//T	synonymous	0	2
WHSC1L1	chr8	38162915/C//T	nonsynonymous	0	2
ADAM9	chr8	38899582/C//A	synonymous	0	2
KAT6A	chr8	41789986/T//C	nonsynonymous	0	2
KAT6A	chr8	41844985/A//C	nonsynonymous	0	2
POTEA	chr8	43147879/C//T	synonymous	0	2
PRKDC	chr8	48739379/G//A	nonsynonymous	0	2
RB1CC1	chr8	53558352/T//C	synonymous	0	2
OPRK1	chr8	54142384/C//T	nonsynonymous	0	2
XKR4	chr8	56270333/A//G	nonsynonymous	0	2
ASPH	chr8	62415937/C//T	nonsynonymous	0	2
PSKH2	chr8	87081839/C//T	nonsynonymous	0	2
GEM	chr8	95272706/C//T	nonsynonymous	0	2
KIAA1429	chr8	95503886/C//T	nonsynonymous	0	2
CPQ	chr8	98041654/C//T	nonsynonymous	0	2
SPAG1	chr8	101232558/G//T	nonsynonymous	0	2
PKHD1L1	chr8	110464495/G//A	nonsynonymous	0	2
SYBU	chr8	110587662/G//A	nonsynonymous	0	2
RAD21	chr8	117864799/C//T	nonsynonymous	0	2
COL14A1	chr8	121256226/G//A	nonsynonymous	0	2
KLHL38	chr8	124658238/G//T	nonsynonymous	0	2
TRIB1	chr8	126445785/G//A	nonsynonymous	0	2
EFR3A	chr8	132982819/G//A	nonsynonymous	0	2
KCNQ3	chr8	133141590/C//T	synonymous	0	2
ZFAT	chr8	135490788/G//A	synonymous	0	2
KCNK9	chr8	140630726/G//T	synonymous	0	2
DENND3	chr8	142178613/C//T	nonsynonymous	0	2
CYP11B2	chr8	143994722/G//A	nonsynonymous	0	2
TOP1MT	chr8	144407645/C//A	nonsynonymous	0	2
	chr8	144671525/C//T	synonymous	0	2
CCDC166	chr8	144789883/G//A	nonsynonymous	0	2
MAPK15	chr8	144798571/C//T	synonymous	0	2
FAM83H	chr8	144810191/G//A	synonymous	0	2
FAM83H	chr8	144810228/C//T	nonsynonymous	0	2
SCRIB	chr8	144885852/G//A	nonsynonymous	0	2
EPPK1	chr8	144945186/G//A	nonsynonymous	0	2
MROH1	chr8	145255402/G//A	nonsynonymous	0	2
TONSL	chr8	145665825/C//T	nonsynonymous	0	2
SMARCA2	chr9	2086866/G//A	nonsynonymous	0	2
JAK2	chr9	5073782/G//A	nonsynonymous	0	2
RIC1	chr9	5763525/G//A	nonsynonymous	0	2
MLANA	chr9	5892525/C//T	synonymous	0	2
GLDC	chr9	6602197/G//A	nonsynonymous	0	2
NFIB	chr9	14146701/G//A	synonymous	0	2
FREM1	chr9	14747343/C//T	synonymous	0	2
TTC39B	chr9	15307161/G//T	nonsynonymous	0	2

ADAMTSL1	chr9	18675909/C//T	synonymous	0	2
ELAVL2	chr9	23762155/C//T	synonymous	0	2
AQP3	chr9	33443780/G//A	synonymous	0	2
KIAA1161	chr9	34372695/G//A	nonsynonymous	0	2
CD72	chr9	35615933/C//T	synonymous	0	2
TLN1	chr9	35717288/G//T	synonymous	0	2
ZNF658	chr9	40784534/C//T	nonsynonymous	0	2
PRKACG	chr9	71628604/G//A	synonymous	0	2
TRPM3	chr9	73150903/G//A	nonsynonymous	0	2
GDA	chr9	74817659/G//A	nonsynonymous	0	2
PCSK5	chr9	78682947/G//A	nonsynonymous	0	2
PRUNE2	chr9	79320987/G//T	nonsynonymous	0	2
GNAQ	chr9	80537236/C//T	synonymous	0	2
UBQLN1	chr9	86279960/G//A	nonsynonymous	0	2
SPATA31E1	chr9	90497949/C//A	nonsynonymous	0	2
CENPP	chr9	95375395/C//T	synonymous	0	2
NINJ1	chr9	95887337/G//A	synonymous	0	2
PHF2	chr9	96427988/C//T	nonsynonymous	0	2
TRMO	chr9	100667175/C//T	nonsynonymous	0	2
INVS	chr9	102988437/C//T	nonsynonymous	0	2
CYLC2	chr9	105767054/G//T	nonsynonymous	0	2
OR13C8	chr9	107331777/C//T	nonsynonymous	0	2
ZNF883	chr9	115759645/G//A	nonsynonymous	0	2
RNF183	chr9	116059984/G//A	nonsynonymous	0	2
WHRN	chr9	117186653/G//A	synonymous	0	2
TNC	chr9	117819616/G//A	synonymous	0	2
PHF19	chr9	123632128/C//T	nonsynonymous	0	2
GSN	chr9	124076269/G//A	nonsynonymous	0	2
	chr9	126164083/C//T	synonymous	0	2
RALGPS1	chr9	129724598/C//T	synonymous	0	2
CFAP157	chr9	130475408/C//A	nonsynonymous	0	2
LCN2	chr9	130912643/G//A	nonsynonymous	0	2
GOLGA2	chr9	131022740/G//A	synonymous	0	2
PKN3	chr9	131469674/C//T	synonymous	0	2
SH3GLB2	chr9	131783434/G//A	synonymous	0	2
PRRC2B	chr9	134357816/A//T	nonsynonymous	0	2
POMT1	chr9	134388668/C//T	synonymous	0	2
NTNG2	chr9	135073926/G//A	nonsynonymous	0	2
CFAP77	chr9	135374097/C//T	nonsynonymous	0	2
GTF3C5	chr9	135933334/C//T	synonymous	0	2
DBH	chr9	136508690/C//T	synonymous	0	2
OLFM1	chr9	138011487/C//T	synonymous	0	2
LCN1	chr9	138413404/C//A	nonsynonymous	0	2
KCNT1	chr9	138648714/C//T	synonymous	0	2
UBAC1	chr9	138836905/C//T	nonsynonymous	0	2
QSOX2	chr9	139108571/G//A	synonymous	0	2
NOTCH1	chr9	139391420/C//T	synonymous	0	2
FBXW5	chr9	139835491/G//A	synonymous	0	2
NPDC1	chr9	139935011/G//A	synonymous	0	2
ANAPC2	chr9	140080722/C//T	nonsynonymous	0	2
SSNA1	chr9	140083536/A//G	nonsynonymous	0	2
EXD3	chr9	140204144/G//A	synonymous	0	2
ARRDC1-AS1	chr9	140510143/G//A	synonymous	0	2
CACNA1B	chr9	140865850/G//A	nonsynonymous	0	2
CACNA1B	chr9	140952557/A//G	nonsynonymous	0	2
CACNA1B	chr9	140952699/C//T	synonymous	0	2
CACNA1B	chr9	140968449/G//A	synonymous	0	2
ADARB2	chr10	1405265/G//A	synonymous	0	2
ADARB2	chr10	1405675/C//T	nonsynonymous	0	2
PITRM1	chr10	3200272/C//T	nonsynonymous	0	2
IL15RA	chr10	6008166/G//A	synonymous	0	2
ITIH5	chr10	7618596/G//A	nonsynonymous	0	2
ITIH2	chr10	7780627/G//A	synonymous	0	2
UPF2	chr10	11994247/C//T	nonsynonymous	0	2
UCMA	chr10	13275743/G//A	nonsynonymous	0	2
FRMD4A	chr10	13735925/C//T	nonsynonymous	0	2
SKIDA1	chr10	21804267/G//A	nonsynonymous	0	2
ARMC3	chr10	23250949/G//A	nonsynonymous	0	2
ANKRD26	chr10	27328913/G//A	nonsynonymous	0	2
ARMC4	chr10	28149592/C//T	nonsynonymous	0	2

MTPAP	chr10	30625741/G//A	synonymous	0	2
PARD3	chr10	34420422/C//T	nonsynonymous	0	2
PARD3	chr10	34759020/T//A	nonsynonymous	0	2
	chr10	35456687/C//T	synonymous	0	2
ANKRD30A	chr10	37482193/T//C	synonymous	0	2
RET	chr10	43610086/G//A	nonsynonymous	0	2
FXYD4	chr10	43869149/C//T	synonymous	0	2
C10orf10	chr10	45473258/C//T	nonsynonymous	0	2
GPRIN2	chr10	46999202/C//T	nonsynonymous	0	2
RBP3	chr10	48390514/G//A	nonsynonymous	0	2
GDF10	chr10	48429469/C//T	synonymous	0	2
ARHGAP22	chr10	49654545/C//T	synonymous	0	2
WDFY4	chr10	49988012/G//A	nonsynonymous	0	2
WDFY4	chr10	50025390/C//T	nonsynonymous	0	2
C10orf71	chr10	50534659/G//A	nonsynonymous	0	2
OGDHL	chr10	50953527/G//A	nonsynonymous	0	2
RHOBTB1	chr10	62671216/G//A	nonsynonymous	0	2
RHOBTB1	chr10	62671280/G//A	synonymous	0	2
COL13A1	chr10	71686853/C//T	nonsynonymous	0	2
DNAJB12	chr10	74104849/G//A	nonsynonymous	0	2
ZSWIM8	chr10	75548539/G//A	nonsynonymous	0	2
KCNMA1	chr10	78669755/G//A	nonsynonymous	0	2
	chr10	82173622/C//T	synonymous	0	2
SH2D4B	chr10	82298247/C//A	nonsynonymous	0	2
CDHR1	chr10	85971411/A//G	nonsynonymous	0	2
LRIT2	chr10	85984362/G//A	nonsynonymous	0	2
LDB3	chr10	88485941/G//A	nonsynonymous	0	2
SORBS1	chr10	97096333/C//T	nonsynonymous	0	2
SORBS1	chr10	97143773/G//A	nonsynonymous	0	2
RRP12	chr10	99139502/C//T	nonsynonymous	0	2
ZFYVE27	chr10	99508078/G//A	nonsynonymous	0	2
SFRP5	chr10	99527417/G//A	nonsynonymous	0	2
LOXL4	chr10	100020707/C//T	nonsynonymous	0	2
CNNM1	chr10	101090683/C//T	synonymous	0	2
ABCC2	chr10	101590172/G//A	nonsynonymous	0	2
LDB1	chr10	103868933/C//T	synonymous	0	2
GBF1	chr10	104119976/C//T	nonsynonymous	0	2
FBXL15	chr10	104181837/C//A	synonymous	0	2
HABP2	chr10	115344049/G//A	synonymous	0	2
DCLRE1A	chr10	115610251/C//T	nonsynonymous	0	2
PRLHR	chr10	120354098/C//T	nonsynonymous	0	2
EIF3A	chr10	120825050/G//A	nonsynonymous	0	2
ATE1	chr10	123503355/G//A	nonsynonymous	0	2
IKZF5	chr10	124753609/C//T	nonsynonymous	0	2
	chr10	126715188/C//A	synonymous	0	2
MKI67	chr10	129897386/G//A	synonymous	0	2
MKI67	chr10	129904842/C//A	nonsynonymous	0	2
C10orf91	chr10	134259251/G//A	synonymous	0	2
B4GALNT4	chr11	379669/G//A	nonsynonymous	0	2
B4GALNT4	chr11	380184/G//A	synonymous	0	2
PKP3	chr11	404684/G//A	synonymous	0	2
LRRC56	chr11	551689/G//A	nonsynonymous	0	2
SLC25A22	chr11	793561/G//A	synonymous	0	2
KRTAP5-1	chr11	1605789/C//T	nonsynonymous	0	2
OSBPL5	chr11	3114849/C//T	synonymous	0	2
MRGPRG	chr11	3239272/C//T	nonsynonymous	0	2
ARFIP2	chr11	6501638/A//C	nonsynonymous	0	2
ZNF214	chr11	7023941/T//C	synonymous	0	2
RBMXL2	chr11	7110448/C//T	nonsynonymous	0	2
CYB5R2	chr11	7694029/T//C	nonsynonymous	0	2
DENND5A	chr11	9165680/G//A	nonsynonymous	0	2
SBF2	chr11	10022513/G//A	nonsynonymous	0	2
GALNT18	chr11	11314641/G//A	nonsynonymous	0	2
TEAD1	chr11	12923505/G//A	nonsynonymous	0	2
INSC	chr11	15198703/C//T	nonsynonymous	0	2
MRGPRX4	chr11	18195449/G//A	nonsynonymous	0	2
NELL1	chr11	21250929/G//A	nonsynonymous	0	2
C11orf74	chr11	36669698/C//T	nonsynonymous	0	2
LRP4	chr11	46924362/G//A	synonymous	0	2
NDUFS3	chr11	47603710/C//T	nonsynonymous	0	2

TRIM49B	chr11	49053261/C//A	nonsynonymous	0	2
TRIM49B	chr11	49059392/C//T	nonsynonymous	0	2
TRIM64C	chr11	49080540/C//T	nonsynonymous	0	2
OR8U8	chr11	56143251/C//T	nonsynonymous	0	2
ZDHHC5	chr11	57466712/C//T	nonsynonymous	0	2
OR9I1	chr11	57886281/G//A	synonymous	0	2
DTX4	chr11	58949795/G//A	nonsynonymous	0	2
MS4A4A	chr11	60068501/G//A	nonsynonymous	0	2
TMEM138	chr11	61136107/G//A	nonsynonymous	0	2
AHNAK	chr11	62284193/A//G	synonymous	0	2
LRRN4CL	chr11	62455775/G//A	nonsynonymous	0	2
TAF6L	chr11	62546400/C//T	nonsynonymous	0	2
TMEM223	chr11	62558383/G//A	synonymous	0	2
SLC22A6	chr11	62747054/G//A	nonsynonymous	0	2
SLC22A6	chr11	62751925/G//A	nonsynonymous	0	2
PLCB3	chr11	64026410/C//T	nonsynonymous	0	2
PLCB3	chr11	64030200/C//T	nonsynonymous	0	2
NRXN2	chr11	64394007/G//A	nonsynonymous	0	2
MEN1	chr11	64571764/C//T	synonymous	0	2
CDC42BPG	chr11	64608108/G//A	nonsynonymous	0	2
FRMD8	chr11	65164417/C//T	synonymous	0	2
LTBP3	chr11	65310589/G//A	synonymous	0	2
EFEMP2	chr11	65635353/C//T	synonymous	0	2
CATSPER1	chr11	65793525/C//T	nonsynonymous	0	2
RAB1B	chr11	66039857/C//T	nonsynonymous	0	2
PELI3	chr11	66241212/G//A	nonsynonymous	0	2
CCDC87	chr11	66360527/G//A	synonymous	0	2
SPTBN2	chr11	66472741/G//A	nonsynonymous	0	2
SPTBN2	chr11	66476435/G//A	nonsynonymous	0	2
SPTBN2	chr11	66483361/G//A	synonymous	0	2
GRK2	chr11	67048933/C//T	synonymous	0	2
PITPNM1	chr11	67267593/G//A	nonsynonymous	0	2
TBX10	chr11	67401799/G//A	nonsynonymous	0	2
C11orf24	chr11	68029455/A//G	synonymous	0	2
LRP5	chr11	68192764/C//T	synonymous	0	2
CPT1A	chr11	68548192/C//T	synonymous	0	2
MRGPRF	chr11	68772896/G//A	synonymous	0	2
FGF3	chr11	69625252/G//A	nonsynonymous	0	2
SHANK2	chr11	70506047/G//A	synonymous	0	2
FAM86C1	chr11	71507065/G//A	synonymous	0	2
NUMA1	chr11	71729533/G//A	nonsynonymous	0	2
P2RY6	chr11	73007862/G//A	nonsynonymous	0	2
UCP3	chr11	73718000/C//T	nonsynonymous	0	2
XRRA1	chr11	74562149/C//T	nonsynonymous	0	2
THAP12	chr11	76061857/G//A	synonymous	0	2
EMSY	chr11	76169274/G//A	nonsynonymous	0	2
CCDC90B	chr11	82985784/C//A	nonsynonymous	0	2
DLG2	chr11	83252900/C//T	nonsynonymous	0	2
GRM5	chr11	88300321/G//A	nonsynonymous	0	2
FAT3	chr11	92526087/C//T	nonsynonymous	0	2
MTNR1B	chr11	92715012/A//G	nonsynonymous	0	2
AMOTL1	chr11	94563358/G//A	nonsynonymous	0	2
TRPC6	chr11	101375399/G//A	nonsynonymous	0	2
GRIA4	chr11	105795276/T//C	nonsynonymous	0	2
SLC35F2	chr11	107673871/G//A	synonymous	0	2
NCAM1	chr11	113075144/C//T	synonymous	0	2
ANKK1	chr11	113269866/C//T	nonsynonymous	0	2
REXO2	chr11	114314650/G//A	nonsynonymous	0	2
IL10RA	chr11	117864097/C//A	nonsynonymous	0	2
PHLDB1	chr11	118527475/G//A	nonsynonymous	0	2
TREH	chr11	118530640/A//G	nonsynonymous	0	2
NLRX1	chr11	119045182/T//C	synonymous	0	2
TMEM136	chr11	120198259/C//T	nonsynonymous	0	2
ARHGEF12	chr11	120318617/T//C	nonsynonymous	0	2
CRTAM	chr11	122720827/C//T	nonsynonymous	0	2
C11orf63	chr11	122828072/C//T	nonsynonymous	0	2
SCN3B	chr11	123513204/C//T	nonsynonymous	0	2
SRPRA	chr11	126134397/C//T	synonymous	0	2
KCNJ1	chr11	128709546/C//T	nonsynonymous	0	2
	chr11	128807494/C//T	synonymous	0	2

ARHGAP32	chr11	128840874/G//A	nonsynonymous	0	2
ARHGAP32	chr11	128844502/C//T	nonsynonymous	0	2
NFRKB	chr11	129756216/C//T	nonsynonymous	0	2
IGSF9B	chr11	133795735/C//T	nonsynonymous	0	2
NINJ2	chr12	674532/G//A	nonsynonymous	0	2
WNT5B	chr12	1755163/G//A	synonymous	0	2
CACNA1C	chr12	2714278/C//T	nonsynonymous	0	2
CACNA1C	chr12	2775934/C//T	nonsynonymous	0	2
VWF	chr12	6128864/C//T	synonymous	0	2
TAPBPL	chr12	6562860/C//T	synonymous	0	2
TAPBPL	chr12	6567981/G//A	nonsynonymous	0	2
CHD4	chr12	6702703/G//A	nonsynonymous	0	2
ATN1	chr12	7046503/C//A	synonymous	0	2
A2ML1	chr12	9016535/G//A	synonymous	0	2
CLEC1A	chr12	10224005/C//A	nonsynonymous	0	2
EPS8	chr12	15807121/C//T	nonsynonymous	0	2
PIK3C2G	chr12	18658225/G//A	nonsynonymous	0	2
ST8SIA1	chr12	22440139/C//T	nonsynonymous	0	2
SOX5	chr12	23818479/G//A	nonsynonymous	0	2
LRMP	chr12	25236399/C//T	nonsynonymous	0	2
AMN1	chr12	31882048/G//A	synonymous	0	2
KMT2D	chr12	49431640/G//A	nonsynonymous	0	2
FAM186A	chr12	50727706/G//A	synonymous	0	2
TMPRSS12	chr12	51236808/G//A	nonsynonymous	0	2
SLC4A8	chr12	51879575/G//A	nonsynonymous	0	2
ACVR1B	chr12	52370312/C//T	nonsynonymous	0	2
KRT81	chr12	52681089/G//A	synonymous	0	2
KRT86	chr12	52700070/T//A	synonymous	0	2
KRT84	chr12	52772147/C//A	nonsynonymous	0	2
KRT5	chr12	52908758/T//A	nonsynonymous	0	2
KRT71	chr12	52943925/C//T	nonsynonymous	0	2
KRT2	chr12	53041558/C//T	nonsynonymous	0	2
KRT3	chr12	53185037/G//A	synonymous	0	2
KRT79	chr12	53215714/G//A	nonsynonymous	0	2
NPFF	chr12	53900912/T//C	synonymous	0	2
ANKRD52	chr12	56647936/G//A	nonsynonymous	0	2
SRGAP1	chr12	64377776/G//T	synonymous	0	2
LEMD3	chr12	65633992/C//T	synonymous	0	2
LRRC10	chr12	70003992/C//T	synonymous	0	2
PTPRB	chr12	70933438/A//G	synonymous	0	2
PTPRQ	chr12	81007382/C//A	nonsynonymous	0	2
CCER1	chr12	91348012/G//A	nonsynonymous	0	2
CEP83	chr12	94725499/G//A	nonsynonymous	0	2
NR2C1	chr12	95445560/G//A	nonsynonymous	0	2
METAP2	chr12	95879695/C//T	synonymous	0	2
SCYL2	chr12	100704846/C//T	nonsynonymous	0	2
SLC17A8	chr12	100774601/G//A	nonsynonymous	0	2
SLC5A8	chr12	101603549/G//A	synonymous	0	2
UTP20	chr12	101705872/G//T	nonsynonymous	0	2
CMKLR1	chr12	108686412/C//T	nonsynonymous	0	2
SELPLG	chr12	109016787/G//T	synonymous	0	2
SELPLG	chr12	109017772/C//T	synonymous	0	2
DAO	chr12	109278846/C//T	nonsynonymous	0	2
MMAB	chr12	109999241/G//A	nonsynonymous	0	2
MVK	chr12	110028579/G//A	synonymous	0	2
TCHP	chr12	110345437/T//C	nonsynonymous	0	2
SH2B3	chr12	111885823/C//A	nonsynonymous	0	2
ATXN2	chr12	111923067/A//G	synonymous	0	2
BRAP	chr12	112097155/G//A	synonymous	0	2
FBXW8	chr12	117426663/G//A	nonsynonymous	0	2
FBXO21	chr12	117612586/G//A	synonymous	0	2
KSR2	chr12	118298140/G//A	nonsynonymous	0	2
CIT	chr12	120128209/C//T	nonsynonymous	0	2
GATC	chr12	120894978/C//T	synonymous	0	2
KDM2B	chr12	121947522/C//T	nonsynonymous	0	2
BCL7A	chr12	122468625/G//A	nonsynonymous	0	2
PITPNM2	chr12	123481031/G//T	nonsynonymous	0	2
TCTN2	chr12	124184297/G//A	nonsynonymous	0	2
DNAH10	chr12	124402166/G//A	nonsynonymous	0	2
DNAH10	chr12	124414233/C//T	nonsynonymous	0	2

AACS	chr12	125613927/G//A	synonymous	0	2
GLT1D1	chr12	129431955/G//A	synonymous	0	2
MMP17	chr12	132335658/C//A	nonsynonymous	0	2
PUS1	chr12	132426160/A//G	nonsynonymous	0	2
PUS1	chr12	132426358/C//T	synonymous	0	2
P2RX2	chr12	133197906/G//A	nonsynonymous	0	2
POLE	chr12	133226471/G//A	nonsynonymous	0	2
PXMP2	chr12	133272574/G//A	nonsynonymous	0	2
TUBA3C	chr13	19748265/G//T	nonsynonymous	0	2
FGF9	chr13	22255181/G//A	nonsynonymous	0	2
SPATA13	chr13	24823618/G//A	synonymous	0	2
RNF17	chr13	25363492/C//T	nonsynonymous	0	2
ATP8A2	chr13	26125492/C//T	nonsynonymous	0	2
LNX2	chr13	28155508/T//C	synonymous	0	2
RFC3	chr13	34395350/C//T	nonsynonymous	0	2
NBEA	chr13	36245124/A//G	nonsynonymous	0	2
SMAD9	chr13	37422955/C//T	nonsynonymous	0	2
SUPT20H	chr13	37622057/G//A	nonsynonymous	0	2
MTRF1	chr13	41814546/G//A	nonsynonymous	0	2
DNAJC15	chr13	43643080/C//T	nonsynonymous	0	2
NUFIP1	chr13	45523896/T//C	nonsynonymous	0	2
SLC25A30	chr13	45970135/C//T	nonsynonymous	0	2
SPERT	chr13	46287518/C//T	nonsynonymous	0	2
SIAH3	chr13	46357973/G//A	nonsynonymous	0	2
ZC3H13	chr13	46563172/G//A	synonymous	0	2
ESD	chr13	47351614/G//A	nonsynonymous	0	2
ARL11	chr13	50205037/C//T	nonsynonymous	0	2
SERPINE3	chr13	51915353/C//T	synonymous	0	2
SERPINE3	chr13	51918597/G//A	nonsynonymous	0	2
CKAP2	chr13	53036581/C//T	nonsynonymous	0	2
PCDH17	chr13	58208729/G//A	synonymous	0	2
GPC5	chr13	92380905/T//C	synonymous	0	2
ABCC4	chr13	95705359/G//A	nonsynonymous	0	2
MBNL2	chr13	97995346/C//A	nonsynonymous	0	2
FARP1	chr13	99098350/A//G	nonsynonymous	0	2
SLC15A1	chr13	99340750/G//A	synonymous	0	2
ZIC5	chr13	100624006/C//A	synonymous	0	2
GGACT	chr13	101184754/C//T	nonsynonymous	0	2
CCDC168	chr13	103390167/C//A	nonsynonymous	0	2
MYO16	chr13	109672199/T//C	synonymous	0	2
COL4A1	chr13	110833684/C//T	synonymous	0	2
COL4A2	chr13	111155778/C//T	nonsynonymous	0	2
ANKRD10	chr13	111532193/C//T	nonsynonymous	0	2
ARHGEF7	chr13	111927931/C//T	nonsynonymous	0	2
MCF2L	chr13	113719372/G//A	synonymous	0	2
TEP1	chr14	20836641/G//A	synonymous	0	2
KLHL33	chr14	20898551/C//T	nonsynonymous	0	2
NDRG2	chr14	21487317/C//T	nonsynonymous	0	2
ZNF219	chr14	21559624/C//T	nonsynonymous	0	2
LRP10	chr14	23346393/G//A	nonsynonymous	0	2
REM2	chr14	23352466/C//T	synonymous	0	2
PRMT5	chr14	23397808/T//C	nonsynonymous	0	2
EFS	chr14	23826425/C//A	synonymous	0	2
EFS	chr14	23828095/G//A	nonsynonymous	0	2
EFS	chr14	23829051/C//T	synonymous	0	2
MHRT	chr14	23886423/G//A	synonymous	0	2
MYH7	chr14	23899793/G//A	synonymous	0	2
ZFHX2	chr14	23994299/C//T	nonsynonymous	0	2
AP1G2	chr14	24033256/G//A	nonsynonymous	0	2
CPNE6	chr14	24545577/G//A	nonsynonymous	0	2
DCAF11	chr14	24584867/C//T	nonsynonymous	0	2
ADCY4	chr14	24795367/G//A	nonsynonymous	0	2
NFATC4	chr14	24845773/C//A	nonsynonymous	0	2
STRN3	chr14	31381313/G//A	nonsynonymous	0	2
RALGAPA1	chr14	36143846/T//C	nonsynonymous	0	2
FAM179B	chr14	45433489/C//T	nonsynonymous	0	2
ATP5S	chr14	50792433/T//A	nonsynonymous	0	2
NIN	chr14	51224413/G//A	nonsynonymous	0	2
TMX1	chr14	51713832/C//T	nonsynonymous	0	2
BMP4	chr14	54417562/G//A	nonsynonymous	0	2

SAMD4A	chr14	55241681/C//T	nonsynonymous	0	2
PELI2	chr14	56763647/C//T	synonymous	0	2
SYNE2	chr14	64545210/A//G	synonymous	0	2
SYNE2	chr14	64593399/C//T	synonymous	0	2
ESR2	chr14	64727411/G//A	synonymous	0	2
MTHFD1	chr14	64908984/C//T	nonsynonymous	0	2
SPTB	chr14	65249177/G//A	nonsynonymous	0	2
SPTB	chr14	65266525/G//A	nonsynonymous	0	2
EXD2	chr14	69704478/G//A	synonymous	0	2
GALNT16	chr14	69795289/G//C	nonsynonymous	0	2
SLC39A9	chr14	69890814/C//T	nonsynonymous	0	2
PROX2	chr14	75321889/C//T	synonymous	0	2
CIPC	chr14	77572171/G//A	synonymous	0	2
NOXRED1	chr14	77872282/G//T	synonymous	0	2
NRXN3	chr14	79175831/G//A	nonsynonymous	0	2
NRXN3	chr14	79270067/C//T	nonsynonymous	0	2
FLRT2	chr14	86089128/C//T	nonsynonymous	0	2
ZC3H14	chr14	89030023/T//G	nonsynonymous	0	2
ZC3H14	chr14	89068286/C//T	nonsynonymous	0	2
NRDE2	chr14	90744664/G//A	synonymous	0	2
SLC24A4	chr14	92908448/G//A	nonsynonymous	0	2
ITPK1	chr14	93407858/G//A	synonymous	0	2
UNC79	chr14	94069683/C//T	nonsynonymous	0	2
UNC79	chr14	94083610/G//A	nonsynonymous	0	2
BDKRB2	chr14	96703496/G//A	nonsynonymous	0	2
BCL11B	chr14	99641923/G//A	nonsynonymous	0	2
CYP46A1	chr14	100157473/G//A	nonsynonymous	0	2
RTL1	chr14	101347803/C//T	nonsynonymous	0	2
RTL1	chr14	101351094/G//A	nonsynonymous	0	2
DYNC1H1	chr14	102505491/C//T	nonsynonymous	0	2
CINP	chr14	102825778/C//T	nonsynonymous	0	2
TECPR2	chr14	102891388/G//A	synonymous	0	2
KLC1	chr14	104145843/C//T	synonymous	0	2
KIF26A	chr14	104638991/G//A	nonsynonymous	0	2
KIF26A	chr14	104642879/A//G	nonsynonymous	0	2
AKT1	chr14	105236655/G//A	synonymous	0	2
AHNAK2	chr14	105411829/G//A	nonsynonymous	0	2
AHNAK2	chr14	105411932/C//G	nonsynonymous	0	2
AHNAK2	chr14	105412263/G//A	synonymous	0	2
AHNAK2	chr14	105420774/C//A	synonymous	0	2
PACS2	chr14	105834860/C//T	nonsynonymous	0	2
CYFIP1	chr15	22940794/C//T	synonymous	0	2
GABRA5	chr15	27185209/G//A	nonsynonymous	0	2
OCA2	chr15	28202839/C//T	nonsynonymous	0	2
OCA2	chr15	28270047/G//A	nonsynonymous	0	2
	chr15	31362183/G//A	synonymous	0	2
OTUD7A	chr15	31779675/G//A	synonymous	0	2
SLC12A6	chr15	34549914/G//A	nonsynonymous	0	2
NUTM1	chr15	34648103/G//A	nonsynonymous	0	2
GJD2	chr15	35044897/T//C	nonsynonymous	0	2
EXD1	chr15	41501704/G//A	synonymous	0	2
NDUFAF1	chr15	41688934/C//T	synonymous	0	2
MAPKBP1	chr15	42104310/C//T	synonymous	0	2
TMEM87A	chr15	42519026/G//A	nonsynonymous	0	2
EPB42	chr15	43503645/C//T	nonsynonymous	0	2
TGM5	chr15	43527663/G//A	synonymous	0	2
TUBGCP4	chr15	43695964/G//A	synonymous	0	2
TP53BP1	chr15	43767762/C//T	nonsynonymous	0	2
MAP1A	chr15	43818200/C//A	nonsynonymous	0	2
DUOX2	chr15	45388081/C//T	nonsynonymous	0	2
	chr15	45411248/G//A	synonymous	0	2
DUOX1	chr15	45444127/C//T	nonsynonymous	0	2
HDC	chr15	50546389/G//A	nonsynonymous	0	2
MYO5C	chr15	52487584/C//T	nonsynonymous	0	2
WDR72	chr15	53992134/A//G	synonymous	0	2
PRTG	chr15	55971599/G//A	nonsynonymous	0	2
TCF12	chr15	57565358/C//T	nonsynonymous	0	2
CGNL1	chr15	57816920/C//T	nonsynonymous	0	2
CCNB2	chr15	59406777/C//T	synonymous	0	2
VPS13C	chr15	62174893/G//A	nonsynonymous	0	2

VPS13C	chr15	62219414/G//A	nonsynonymous	0	2
CA12	chr15	63637822/C//T	synonymous	0	2
DAPK2	chr15	64218220/G//A	synonymous	0	2
UACA	chr15	70976621/G//A	nonsynonymous	0	2
THSD4	chr15	72069784/C//T	synonymous	0	2
NR2E3	chr15	72105812/G//A	synonymous	0	2
PML	chr15	74325692/G//A	nonsynonymous	0	2
ISLR2	chr15	74426468/G//A	nonsynonymous	0	2
ACSBG1	chr15	78474329/G//A	synonymous	0	2
DNAJA4	chr15	78566648/C//T	synonymous	0	2
ABHD17C	chr15	81041981/C//T	nonsynonymous	0	2
AKAP13	chr15	86278308/C//T	synonymous	0	2
ACAN	chr15	89381906/C//T	nonsynonymous	0	2
ACAN	chr15	89381931/G//A	synonymous	0	2
HAPLN3	chr15	89422332/G//A	nonsynonymous	0	2
IQGAP1	chr15	91020406/G//A	synonymous	0	2
IGF1R	chr15	99486166/C//T	nonsynonymous	0	2
ADAMTS17	chr15	100591783/C//T	nonsynonymous	0	2
LRRK1	chr15	101562104/G//T	nonsynonymous	0	2
CHSY1	chr15	101775564/C//T	nonsynonymous	0	2
RHBDF1	chr16	109251/C//T	nonsynonymous	0	2
FAM234A	chr16	314043/C//T	nonsynonymous	0	2
RGS11	chr16	319368/G//A	nonsynonymous	0	2
CAPN15	chr16	597834/GCCCGCCAGC//G	nonsynonymous	0	2
RHOT2	chr16	720472/T//C	nonsynonymous	0	2
BAIAP3	chr16	1397936/C//T	nonsynonymous	0	2
CLCN7	chr16	1497053/C//T	nonsynonymous	0	2
CLCN7	chr16	1498415/T//C	nonsynonymous	0	2
CLCN7	chr16	1502802/C//T	nonsynonymous	0	2
EME2	chr16	1825645/G//A	nonsynonymous	0	2
TSC2	chr16	2122238/G//A	synonymous	0	2
RNPS1	chr16	2313103/C//T	nonsynonymous	0	2
TBC1D24	chr16	2547065/G//A	nonsynonymous	0	2
SRRM2	chr16	2815594/C//T	nonsynonymous	0	2
SRRM2	chr16	2816224/C//T	nonsynonymous	0	2
FLYWCH1	chr16	2988325/C//T	nonsynonymous	0	2
PAQR4	chr16	3021806/C//T	nonsynonymous	0	2
PKMYT1	chr16	3024311/G//A	nonsynonymous	0	2
MEFV	chr16	3299521/G//A	synonymous	0	2
NLRC3	chr16	3614763/C//T	synonymous	0	2
TRAP1	chr16	3740965/C//T	nonsynonymous	0	2
CREBBP	chr16	3820616/C//T	synonymous	0	2
GLIS2	chr16	4382446/G//A	synonymous	0	2
CORO7-PAM16	chr16	4438021/C//T	nonsynonymous	0	2
UBALD1	chr16	4659508/C//T	synonymous	0	2
GLYR1	chr16	4873881/C//T	nonsynonymous	0	2
PPL	chr16	4933333/G//A	synonymous	0	2
SEC14L5	chr16	5041924/G//A	nonsynonymous	0	2
PARN	chr16	14693804/T//C	nonsynonymous	0	2
SMG1	chr16	18823089/A//G	synonymous	0	2
SMG1	chr16	18863401/C//T	synonymous	0	2
UMOD	chr16	20359827/C//T	nonsynonymous	0	2
EEF2K	chr16	22237178/C//T	nonsynonymous	0	2
USP31	chr16	23093817/G//A	nonsynonymous	0	2
CACNG3	chr16	24358104/T//C	synonymous	0	2
AQP8	chr16	25232848/C//A	nonsynonymous	0	2
HS3ST4	chr16	26147031/C//T	nonsynonymous	0	2
HS3ST4	chr16	26147454/G//A	nonsynonymous	0	2
GTF3C1	chr16	27500493/G//A	synonymous	0	2
C16orf92	chr16	30035070/G//A	synonymous	0	2
CD2BP2	chr16	30364373/C//T	nonsynonymous	0	2
TBC1D10B	chr16	30371083/C//T	nonsynonymous	0	2
ZNF48	chr16	30407099/A//G	nonsynonymous	0	2
ZNF771	chr16	30429192/G//A	nonsynonymous	0	2
SETD1A	chr16	30978852/G//A	nonsynonymous	0	2
SETD1A	chr16	30982725/T//C	nonsynonymous	0	2
TGFB1I1	chr16	31485194/C//T	nonsynonymous	0	2
ITFG1	chr16	47195721/G//A	nonsynonymous	0	2
ITFG1	chr16	47196506/C//A	nonsynonymous	0	2
ABCC11	chr16	48209244/C//T	nonsynonymous	0	2

LONP2	chr16	48304049/C//T	nonsynonymous	0	2
PAPD5	chr16	50245299/G//A	nonsynonymous	0	2
NKD1	chr16	50659407/C//T	synonymous	0	2
NKD1	chr16	50667422/C//T	synonymous	0	2
IRX6	chr16	55362746/C//T	nonsynonymous	0	2
SLC6A2	chr16	55703536/A//G	nonsynonymous	0	2
	chr16	57793059/C//T	synonymous	0	2
ELMO3	chr16	67235523/C//T	nonsynonymous	0	2
FHOD1	chr16	67271561/G//A	synonymous	0	2
ZDHHC1	chr16	67435027/G//A	synonymous	0	2
FAM65A	chr16	67573806/C//T	synonymous	0	2
CENPT	chr16	67862225/C//T	nonsynonymous	0	2
SLC12A4	chr16	67980372/G//A	synonymous	0	2
HAS3	chr16	69148767/C//T	synonymous	0	2
COG8	chr16	69366757/C//T	nonsynonymous	0	2
SF3B3	chr16	70569301/G//A	nonsynonymous	0	2
SF3B3	chr16	70597923/G//A	synonymous	0	2
HYDIN	chr16	71163565/T//C	nonsynonymous	0	2
ZFHX3	chr16	72831708/G//A	nonsynonymous	0	2
WDR59	chr16	74908124/G//A	nonsynonymous	0	2
CDYL2	chr16	80718786/G//A	synonymous	0	2
BCO1	chr16	81324155/C//T	synonymous	0	2
MBTPS1	chr16	84088051/C//T	synonymous	0	2
ATP2C2	chr16	84495648/G//A	nonsynonymous	0	2
IRF8	chr16	85946798/G//A	nonsynonymous	0	2
FOXF1	chr16	86544851/G//T	nonsynonymous	0	2
PIEZO1	chr16	88782210/C//T	nonsynonymous	0	2
PIEZO1	chr16	88789274/G//A	nonsynonymous	0	2
ACSF3	chr16	89169104/G//A	synonymous	0	2
CPNE7	chr16	89655117/C//T	nonsynonymous	0	2
CDK10	chr16	89758884/C//T	nonsynonymous	0	2
TCF25	chr16	89965178/C//T	synonymous	0	2
TUBB3	chr16	90001622/G//A	nonsynonymous	0	2
RPH3AL	chr17	96970/G//A	nonsynonymous	0	2
SLC43A2	chr17	1496473/G//A	synonymous	0	2
PRPF8	chr17	1564296/C//T	nonsynonymous	0	2
RPA1	chr17	1779058/C//A	synonymous	0	2
RTN4RL1	chr17	1840332/G//A	nonsynonymous	0	2
SMG6	chr17	2203334/G//A	nonsynonymous	0	2
SGSM2	chr17	2276772/C//T	nonsynonymous	0	2
CLUH	chr17	2595865/G//A	synonymous	0	2
ZZEF1	chr17	3916822/C//T	nonsynonymous	0	2
ANKFY1	chr17	4098363/C//T	nonsynonymous	0	2
MED11	chr17	4635186/C//G	nonsynonymous	0	2
CAMTA2	chr17	4875641/A//G	synonymous	0	2
WSCD1	chr17	6012920/C//A	synonymous	0	2
	chr17	7121886/T//C	synonymous	0	2
DVL2	chr17	7129587/C//T	nonsynonymous	0	2
ELP5	chr17	7160344/G//A	nonsynonymous	0	2
GPS2	chr17	7217802/C//G	synonymous	0	2
NEURL4	chr17	7224931/C//T	nonsynonymous	0	2
SPEM1	chr17	7324353/G//A	nonsynonymous	0	2
CHD3	chr17	7804615/G//A	synonymous	0	2
CHD3	chr17	7806722/C//T	nonsynonymous	0	2
CHD3	chr17	7808941/C//T	nonsynonymous	0	2
PER1	chr17	8052875/C//T	nonsynonymous	0	2
MYH10	chr17	8448859/G//A	synonymous	0	2
NTN1	chr17	8926116/C//T	synonymous	0	2
DNAH9	chr17	11648158/G//T	synonymous	0	2
TRPV2	chr17	16327059/C//T	nonsynonymous	0	2
CCDC144A	chr17	16667414/T//C	synonymous	0	2
MPRIP	chr17	17030117/G//A	synonymous	0	2
RAI1	chr17	17698079/A//T	nonsynonymous	0	2
RAI1	chr17	17700591/G//A	synonymous	0	2
MYO15A	chr17	18051846/C//T	nonsynonymous	0	2
ALKBH5	chr17	18098328/G//A	nonsynonymous	0	2
FLII	chr17	18154314/G//A	synonymous	0	2
MAPK7	chr17	19284605/G//A	synonymous	0	2
RNF112	chr17	19318388/C//T	synonymous	0	2
ULK2	chr17	19746541/A//G	synonymous	0	2

KCNJ12	chr17	21319254/C//T	synonymous	0	2
FOXN1	chr17	26851609/C//T	nonsynonymous	0	2
SSH2	chr17	27957926/G//A	nonsynonymous	0	2
MYO1D	chr17	30981516/C//T	nonsynonymous	0	2
LIG3	chr17	33319026/C//A	nonsynonymous	0	2
SLFN13	chr17	33771725/C//T	synonymous	0	2
AATF	chr17	35413969/G//A	synonymous	0	2
GPR179	chr17	36484994/G//A	synonymous	0	2
GPR179	chr17	36491526/G//A	nonsynonymous	0	2
ARHGAP23	chr17	36614481/G//A	nonsynonymous	0	2
PNMT	chr17	37825892/C//T	synonymous	0	2
MED24	chr17	38182539/A//G	nonsynonymous	0	2
KRT39	chr17	39122918/C//T	nonsynonymous	0	2
KRT37	chr17	39578450/G//T	synonymous	0	2
KRT35	chr17	39635733/G//A	nonsynonymous	0	2
GAST	chr17	39871686/G//A	synonymous	0	2
HAP1	chr17	39881396/G//A	nonsynonymous	0	2
GHDC	chr17	40342259/C//T	nonsynonymous	0	2
STAT3	chr17	40491345/C//T	nonsynonymous	0	2
WNK4	chr17	40936504/C//T	synonymous	0	2
CNTD1	chr17	40951222/G//A	nonsynonymous	0	2
DUSP3	chr17	41847137/G//A	nonsynonymous	0	2
HDAC5	chr17	42188128/C//T	synonymous	0	2
FZD2	chr17	42636116/C//A	nonsynonymous	0	2
CCDC43	chr17	42759407/A//C	nonsynonymous	0	2
KANSL1	chr17	44115975/G//A	nonsynonymous	0	2
KANSL1	chr17	44248233/C//T	nonsynonymous	0	2
NGFR	chr17	47588006/G//A	synonymous	0	2
SPATA20	chr17	48629529/C//T	nonsynonymous	0	2
TRIM25	chr17	54985913/C//T	synonymous	0	2
TSPOAP1	chr17	56395764/G//A	synonymous	0	2
TSPOAP1	chr17	56396614/G//A	synonymous	0	2
MTMR4	chr17	56585883/A//G	nonsynonymous	0	2
TEX14	chr17	56693650/G//A	nonsynonymous	0	2
MED13	chr17	60042364/T//C	synonymous	0	2
MAP3K3	chr17	61771045/C//T	nonsynonymous	0	2
CSHL1	chr17	61987817/G//A	nonsynonymous	0	2
CSHL1	chr17	61988595/C//T	synonymous	0	2
SCN4A	chr17	62026884/G//A	nonsynonymous	0	2
ERN1	chr17	62126408/C//T	synonymous	0	2
AXIN2	chr17	63532613/G//A	nonsynonymous	0	2
SLC16A6	chr17	66270122/C//T	nonsynonymous	0	2
ABCA10	chr17	67145200/T//C	nonsynonymous	0	2
SDK2	chr17	71434181/C//T	nonsynonymous	0	2
KIF19	chr17	72324574/G//A	nonsynonymous	0	2
KIF19	chr17	72345455/C//T	nonsynonymous	0	2
GPR142	chr17	72365658/G//T	nonsynonymous	0	2
OTOP3	chr17	72939804/A//G	nonsynonymous	0	2
RECQL5	chr17	73627574/G//A	synonymous	0	2
UNC13D	chr17	73832920/C//T	nonsynonymous	0	2
WBP2	chr17	73847668/A//G	nonsynonymous	0	2
FOXJ1	chr17	74133452/G//A	synonymous	0	2
SPHK1	chr17	74381491/T//C	synonymous	0	2
RHBDF2	chr17	74468801/G//A	synonymous	0	2
	chr17	75316383/C//T	synonymous	0	2
DNAH17	chr17	76455859/G//A	synonymous	0	2
TIMP2	chr17	76851906/G//A	nonsynonymous	0	2
CCDC40	chr17	78063635/C//T	synonymous	0	2
RNF213	chr17	78268635/G//A	nonsynonymous	0	2
RNF213	chr17	78305896/C//T	nonsynonymous	0	2
RNF213	chr17	78314093/G//A	nonsynonymous	0	2
CEP131	chr17	79180578/C//T	nonsynonymous	0	2
TEPSIN	chr17	79207259/C//T	nonsynonymous	0	2
DLGAP1	chr18	3814117/G//A	nonsynonymous	0	2
DLGAP1	chr18	3879421/G//A	synonymous	0	2
EPB41L3	chr18	5438113/C//A	synonymous	0	2
LAMA1	chr18	7017295/C//T	synonymous	0	2
LRRC30	chr18	7231979/G//A	synonymous	0	2
MTCL1	chr18	8826172/C//T	nonsynonymous	0	2
ANKRD12	chr18	9258556/C//A	nonsynonymous	0	2

RALBP1	chr18	9535807/C//T	nonsynonymous	0	2
NAPG	chr18	10550171/C//T	nonsynonymous	0	2
LAMA3	chr18	21484551/C//T	nonsynonymous	0	2
DSG1	chr18	28913647/C//T	synonymous	0	2
GAREM1	chr18	29867399/C//T	synonymous	0	2
SLC14A2	chr18	43207002/C//T	synonymous	0	2
PSTPIP2	chr18	43595892/C//T	nonsynonymous	0	2
ST8SIA5	chr18	44260358/G//A	nonsynonymous	0	2
PIAS2	chr18	44424698/T//C	synonymous	0	2
MYO5B	chr18	47462659/G//A	nonsynonymous	0	2
CXXC1	chr18	47813200/G//A	nonsynonymous	0	2
DCC	chr18	50683754/G//A	synonymous	0	2
ZNF236	chr18	74680263/G//A	nonsynonymous	0	2
ATP9B	chr18	76873322/A//T	nonsynonymous	0	2
NFATC1	chr18	77170458/G//A	synonymous	0	2
MISP	chr19	758304/C//T	nonsynonymous	0	2
ARID3A	chr19	929811/C//T	nonsynonymous	0	2
ABCA7	chr19	1044724/C//T	nonsynonymous	0	2
ARHGAP45	chr19	1080047/C//T	nonsynonymous	0	2
PCSK4	chr19	1482046/G//A	synonymous	0	2
PCSK4	chr19	1489817/C//T	nonsynonymous	0	2
SLC39A3	chr19	2732908/G//A	synonymous	0	2
NFIC	chr19	3453832/G//A	synonymous	0	2
DOHH	chr19	3496585/G//A	synonymous	0	2
TJP3	chr19	3747890/C//T	synonymous	0	2
TMIGD2	chr19	4292743/C//T	synonymous	0	2
HDGFRP2	chr19	4475493/G//A	synonymous	0	2
PTPRS	chr19	5206800/G//A	synonymous	0	2
PTPRS	chr19	5219383/G//A	synonymous	0	2
FUT5	chr19	5867383/C//T	synonymous	0	2
RFX2	chr19	5997093/G//A	nonsynonymous	0	2
SLC25A41	chr19	6430031/C//T	nonsynonymous	0	2
TUBB4A	chr19	6495528/C//T	nonsynonymous	0	2
ARHGEF18	chr19	7535154/C//T	synonymous	0	2
TIMM44	chr19	7992542/C//T	synonymous	0	2
TIMM44	chr19	7998383/G//A	synonymous	0	2
MUC16	chr19	8999514/C//T	nonsynonymous	0	2
FBXL12	chr19	9922362/C//T	nonsynonymous	0	2
PIN1	chr19	9949167/C//T	synonymous	0	2
COL5A3	chr19	10090677/C//G	nonsynonymous	0	2
KEAP1	chr19	10602724/A//G	nonsynonymous	0	2
YIPF2	chr19	11034643/C//T	nonsynonymous	0	2
SMARCA4	chr19	11135038/C//T	nonsynonymous	0	2
ZNF627	chr19	11727740/G//A	nonsynonymous	0	2
ZNF627	chr19	11728117/C//T	nonsynonymous	0	2
ZNF799	chr19	12502833/C//T	nonsynonymous	0	2
DHPS	chr19	12790709/C//T	nonsynonymous	0	2
HOOK2	chr19	12874135/G//T	synonymous	0	2
HOOK2	chr19	12874493/G//A	nonsynonymous	0	2
HOOK2	chr19	12874566/C//T	synonymous	0	2
TRMT1	chr19	13216176/G//A	nonsynonymous	0	2
TRMT1	chr19	13226585/G//A	synonymous	0	2
ZSWIM4	chr19	13923949/G//A	nonsynonymous	0	2
CC2D1A	chr19	14034569/C//T	nonsynonymous	0	2
NOTCH3	chr19	15280904/C//T	nonsynonymous	0	2
BRD4	chr19	15349686/C//T	synonymous	0	2
AKAP8L	chr19	15512155/G//A	nonsynonymous	0	2
WIZ	chr19	15559005/C//T	synonymous	0	2
OR10H5	chr19	15905651/G//A	nonsynonymous	0	2
OR10H5	chr19	15905664/C//T	nonsynonymous	0	2
CYP4F2	chr19	16000370/G//A	nonsynonymous	0	2
CYP4F11	chr19	16025652/C//T	nonsynonymous	0	2
CPAMD8	chr19	17010311/C//T	nonsynonymous	0	2
MYO9B	chr19	17265212/G//A	nonsynonymous	0	2
PDE4C	chr19	18343843/G//A	synonymous	0	2
KIAA1683	chr19	18375876/G//A	nonsynonymous	0	2
KIAA1683	chr19	18378115/C//T	nonsynonymous	0	2
ISYNA1	chr19	18547548/C//T	synonymous	0	2
ZNF429	chr19	21719918/A//C	nonsynonymous	0	2
ZNF43	chr19	21992349/G//A	nonsynonymous	0	2

ZNF208	chr19	22154966/G//A	nonsynonymous	0	2
ANKRD27	chr19	33116785/C//T	nonsynonymous	0	2
CEP89	chr19	33392244/G//A	nonsynonymous	0	2
LRP3	chr19	33697122/C//T	synonymous	0	2
PEPD	chr19	33878815/C//T	nonsynonymous	0	2
ZNF30	chr19	35434709/G//A	nonsynonymous	0	2
SCN1B	chr19	35524650/C//T	synonymous	0	2
	chr19	35608226/G//A	synonymous	0	2
FFAR1	chr19	35842685/G//A	synonymous	0	2
GAPDHS	chr19	36033850/C//T	synonymous	0	2
KMT2B	chr19	36223184/C//T	nonsynonymous	0	2
KMT2B	chr19	36224677/C//T	nonsynonymous	0	2
IGFLR1	chr19	36230938/C//T	nonsynonymous	0	2
ARHGAP33	chr19	36271686/C//T	nonsynonymous	0	2
APLP1	chr19	36361862/G//T	nonsynonymous	0	2
ZFP82	chr19	36884709/G//A	nonsynonymous	0	2
ZNF850	chr19	37241329/C//T	nonsynonymous	0	2
ZNF568	chr19	37441527/G//A	nonsynonymous	0	2
SIPA1L3	chr19	38621398/C//T	synonymous	0	2
CAPN12	chr19	39232422/G//A	synonymous	0	2
CCER2	chr19	39401701/G//A	synonymous	0	2
LRFN1	chr19	39804941/G//A	nonsynonymous	0	2
PAF1	chr19	39876894/G//A	nonsynonymous	0	2
FCGBP	chr19	40397981/C//T	nonsynonymous	0	2
ZNF546	chr19	40521492/C//T	nonsynonymous	0	2
AKT2	chr19	40741015/C//T	nonsynonymous	0	2
LTBP4	chr19	41122917/G//A	nonsynonymous	0	2
AXL	chr19	41765522/C//T	nonsynonymous	0	2
BCKDHA	chr19	41928113/G//A	nonsynonymous	0	2
CEACAM5	chr19	42219139/G//A	nonsynonymous	0	2
CEACAM3	chr19	42300608/C//T	synonymous	0	2
ERF	chr19	42754483/C//T	nonsynonymous	0	2
MEGF8	chr19	42848675/C//A	nonsynonymous	0	2
MEGF8	chr19	42866653/G//A	nonsynonymous	0	2
IRGQ	chr19	44096770/G//A	nonsynonymous	0	2
SMG9	chr19	44252177/C//T	nonsynonymous	0	2
NECTIN2	chr19	45375315/G//A	synonymous	0	2
TOMM40	chr19	45404303/C//T	nonsynonymous	0	2
TRAPPC6A	chr19	45681430/G//C	nonsynonymous	0	2
MARK4	chr19	45805908/G//A	synonymous	0	2
ERCC2	chr19	45855468/C//T	nonsynonymous	0	2
FBXO46	chr19	46215041/G//A	synonymous	0	2
DMPK	chr19	46281814/C//T	nonsynonymous	0	2
SYMPK	chr19	46329601/G//A	nonsynonymous	0	2
PGLYRP1	chr19	46526006/C//T	nonsynonymous	0	2
PPP5C	chr19	46857085/C//A	nonsynonymous	0	2
PNMAL2	chr19	46997467/G//T	nonsynonymous	0	2
PNMAL2	chr19	46997853/G//A	synonymous	0	2
C5AR1	chr19	47823602/G//A	nonsynonymous	0	2
GRIN2D	chr19	48908245/C//T	synonymous	0	2
NTN5	chr19	49174152/G//A	nonsynonymous	0	2
TULP2	chr19	49398656/C//T	nonsynonymous	0	2
HRC	chr19	49657426/C//T	nonsynonymous	0	2
TRPM4	chr19	49693531/G//A	nonsynonymous	0	2
TEAD2	chr19	49845810/T//C	nonsynonymous	0	2
PRR12	chr19	50128890/G//A	synonymous	0	2
SCAF1	chr19	50156900/C//A	nonsynonymous	0	2
CPT1C	chr19	50204865/C//T	nonsynonymous	0	2
MED25	chr19	50333092/G//A	nonsynonymous	0	2
SIGLEC11	chr19	50461952/G//A	synonymous	0	2
NAPSA	chr19	50861862/C//T	nonsynonymous	0	2
POLD1	chr19	50905496/G//A	synonymous	0	2
MYBPC2	chr19	50949184/G//A	nonsynonymous	0	2
SYT3	chr19	51128813/C//T	nonsynonymous	0	2
KLK1	chr19	51325005/G//A	nonsynonymous	0	2
SIGLEC12	chr19	52002731/C//T	nonsynonymous	0	2
FPR3	chr19	52327714/G//A	nonsynonymous	0	2
PPP2R1A	chr19	52714686/C//T	synonymous	0	2
ZNF534	chr19	52941831/G//A	nonsynonymous	0	2
FAM90A27P	chr19	53786091/C//T	synonymous	0	2

ZNF331	chr19	54080055/C//T	nonsynonymous	0	2
CNOT3	chr19	54656196/G//A	synonymous	0	2
PPP1R12C	chr19	55602909/G//A	synonymous	0	2
KMT5C	chr19	55855042/T//C	synonymous	0	2
SSC5D	chr19	56011423/C//T	nonsynonymous	0	2
NLRP5	chr19	56538969/C//T	nonsynonymous	0	2
ZSCAN5B	chr19	56701936/T//C	nonsynonymous	0	2
ZSCAN5A	chr19	56733046/G//A	synonymous	0	2
PEG3	chr19	57327119/C//A	nonsynonymous	0	2
ZIK1	chr19	58101682/G//A	nonsynonymous	0	2
TRIM28	chr19	59060583/C//A	synonymous	0	2
SDCBP2	chr20	1293150/G//A	nonsynonymous	0	2
SNRPB	chr20	2443387/CAGG//C	nonsynonymous	0	2
TMC2	chr20	2573021/G//T	nonsynonymous	0	2
VPS16	chr20	2845636/C//A	synonymous	0	2
FASTKD5	chr20	3128266/T//C	nonsynonymous	0	2
SLC4A11	chr20	3209814/C//T	nonsynonymous	0	2
PROKR2	chr20	5282861/A//G	nonsynonymous	0	2
TRMT6	chr20	5919235/G//A	synonymous	0	2
LRRN4	chr20	6021904/G//A	nonsynonymous	0	2
FERMT1	chr20	6060153/G//A	nonsynonymous	0	2
LAMP5	chr20	9510396/G//A	nonsynonymous	0	2
JAG1	chr20	10622245/C//T	nonsynonymous	0	2
PCSK2	chr20	17462599/G//A	nonsynonymous	0	2
RIN2	chr20	19970781/C//T	nonsynonymous	0	2
RALGAPA2	chr20	20586008/G//A	nonsynonymous	0	2
SSTR4	chr20	23016585/G//A	synonymous	0	2
NINL	chr20	25459654/G//A	synonymous	0	2
REM1	chr20	30072111/C//T	nonsynonymous	0	2
ID1	chr20	30193250/G//T	nonsynonymous	0	2
	chr20	30449212/G//A	synonymous	0	2
COMMD7	chr20	31291215/C//T	nonsynonymous	0	2
BPIFB4	chr20	31671224/T//C	nonsynonymous	0	2
SNTA1	chr20	31998068/C//T	synonymous	0	2
E2F1	chr20	32265070/C//T	nonsynonymous	0	2
MYH7B	chr20	33588608/C//T	nonsynonymous	0	2
CEP250	chr20	34085877/C//T	synonymous	0	2
SLA2	chr20	35243729/C//T	nonsynonymous	0	2
TGM2	chr20	36784336/G//A	nonsynonymous	0	2
KIAA1755	chr20	36859722/G//A	nonsynonymous	0	2
PPP1R16B	chr20	37531387/C//T	synonymous	0	2
DHX35	chr20	37634984/T//C	nonsynonymous	0	2
MAFB	chr20	39317290/C//T	synonymous	0	2
TOP1	chr20	39721132/C//A	nonsynonymous	0	2
PTPRT	chr20	40790065/C//T	nonsynonymous	0	2
L3MBTL1	chr20	42162678/A//G	nonsynonymous	0	2
JPH2	chr20	42747161/C//T	synonymous	0	2
HNF4A	chr20	43043174/G//A	nonsynonymous	0	2
HNF4A	chr20	43047159/C//T	synonymous	0	2
KCNS1	chr20	43727023/G//A	synonymous	0	2
MATN4	chr20	43927113/G//T	nonsynonymous	0	2
ACOT8	chr20	44477278/C//T	nonsynonymous	0	2
ZNF335	chr20	44598139/C//T	synonymous	0	2
SLC12A5	chr20	44680470/C//T	nonsynonymous	0	2
NCOA5	chr20	44698965/G//A	synonymous	0	2
PREX1	chr20	47295928/C//T	nonsynonymous	0	2
ZNFX1	chr20	47888006/G//A	nonsynonymous	0	2
ZNFX1	chr20	47892414/T//C	synonymous	0	2
BCAS4	chr20	49434784/C//A	nonsynonymous	0	2
KCNG1	chr20	49621073/G//A	nonsynonymous	0	2
RBM38	chr20	55982789/G//A	nonsynonymous	0	2
PMEPA1	chr20	56227642/C//T	nonsynonymous	0	2
GNAS	chr20	57429130/G//A	synonymous	0	2
ZNF831	chr20	57766692/C//T	synonymous	0	2
HRH3	chr20	60791420/G//A	nonsynonymous	0	2
OGFR	chr20	61443584/G//A	nonsynonymous	0	2
COL9A3	chr20	61451288/C//T	nonsynonymous	0	2
DIDO1	chr20	61512312/C//T	nonsynonymous	0	2
DIDO1	chr20	61542781/G//A	nonsynonymous	0	2
GID8	chr20	61572891/G//T	nonsynonymous	0	2

COL20A1	chr20	61937317/C//T	nonsynonymous	0	2
EEF1A2	chr20	62127247/G//A	nonsynonymous	0	2
HELZ2	chr20	62196470/C//T	synonymous	0	2
HELZ2	chr20	62197378/G//A	nonsynonymous	0	2
GMEB2	chr20	62223387/G//A	nonsynonymous	0	2
RTEL1	chr20	62324280/C//T	synonymous	0	2
ZGPAT	chr20	62366035/G//A	nonsynonymous	0	2
UCKL1	chr20	62577878/T//C	nonsynonymous	0	2
CHODL	chr21	19629133/C//T	synonymous	0	2
NCAM2	chr21	22841105/G//A	nonsynonymous	0	2
CYYR1	chr21	27852623/G//A	nonsynonymous	0	2
KRTAP11-1	chr21	32253539/C//T	nonsynonymous	0	2
MRPS6	chr21	35514908/C//T	synonymous	0	2
PRDM15	chr21	43230527/G//A	nonsynonymous	0	2
ZBTB21	chr21	43411305/G//A	nonsynonymous	0	2
ABCG1	chr21	43711290/C//T	synonymous	0	2
TRPM2	chr21	45825109/G//A	nonsynonymous	0	2
ITGB2	chr21	46308804/G//A	synonymous	0	2
FAM207A	chr21	46363734/G//A	nonsynonymous	0	2
POFUT2	chr21	46689893/G//A	synonymous	0	2
COL6A1	chr21	47406938/C//T	synonymous	0	2
COL6A2	chr21	47531946/G//A	nonsynonymous	0	2
FTCD	chr21	47574081/C//T	nonsynonymous	0	2
DIP2A	chr21	47965120/C//T	synonymous	0	2
CCT8L2	chr22	17071852/T//C	nonsynonymous	0	2
MICAL3	chr22	18300316/G//A	nonsynonymous	0	2
PRODH	chr22	18900740/A//C	nonsynonymous	0	2
CLTCL1	chr22	19226881/C//A	nonsynonymous	0	2
TXNRD2	chr22	19864625/C//T	synonymous	0	2
TANGO2	chr22	20052095/C//T	synonymous	0	2
SLC7A4	chr22	21385523/C//T	nonsynonymous	0	2
BCR	chr22	23615871/G//T	nonsynonymous	0	2
SMARCB1	chr22	24176339/G//A	nonsynonymous	0	2
CABIN1	chr22	24459602/C//T	nonsynonymous	0	2
CABIN1	chr22	24574185/G//A	synonymous	0	2
SUSD2	chr22	24584295/C//T	nonsynonymous	0	2
GGT1	chr22	25011033/C//T	synonymous	0	2
GGT1	chr22	25016352/G//A	nonsynonymous	0	2
CRYBB2	chr22	25623853/C//T	synonymous	0	2
MYO18B	chr22	26219577/G//A	nonsynonymous	0	2
SEZ6L	chr22	26688560/G//A	nonsynonymous	0	2
TPST2	chr22	26937137/C//T	nonsynonymous	0	2
TPST2	chr22	26937534/C//T	synonymous	0	2
TTC28	chr22	28392157/C//T	nonsynonymous	0	2
C22orf31	chr22	29456508/G//A	synonymous	0	2
AP1B1	chr22	29746114/G//A	nonsynonymous	0	2
NEFH	chr22	29879545/C//T	synonymous	0	2
LARGE1	chr22	34022242/G//A	synonymous	0	2
RBFOX2	chr22	36155968/G//A	nonsynonymous	0	2
FOXRED2	chr22	36897297/G//A	nonsynonymous	0	2
FOXRED2	chr22	36900732/G//A	synonymous	0	2
BAIAP2L2	chr22	38481329/C//T	nonsynonymous	0	2
TMEM184B	chr22	38622803/G//A	synonymous	0	2
KCNJ4	chr22	38823715/G//A	synonymous	0	2
SGSM3	chr22	40802205/C//T	nonsynonymous	0	2
ZC3H7B	chr22	41735192/G//A	synonymous	0	2
NAGA	chr22	42458918/G//A	synonymous	0	2
SCUBE1	chr22	43603577/C//T	nonsynonymous	0	2
EFCAB6	chr22	43985983/G//A	synonymous	0	2
PNPLA5	chr22	44276749/C//T	nonsynonymous	0	2
SAMM50	chr22	44386258/G//A	nonsynonymous	0	2
PARVB	chr22	44527442/C//T	nonsynonymous	0	2
NUP50	chr22	45574516/A//G	synonymous	0	2
TRABD	chr22	50635992/C//A	nonsynonymous	0	2
TUBGCP6	chr22	50656153/C//T	synonymous	0	2
TUBGCP6	chr22	50656534/C//T	synonymous	0	2
TUBGCP6	chr22	50656654/C//T	nonsynonymous	0	2
HDAC10	chr22	50687124/C//T	synonymous	0	2
NCAPH2	chr22	50956231/C//T	nonsynonymous	0	2
SCO2	chr22	50962301/G//A	synonymous	0	2

PLCXD1	chrX	205418/C//T	nonsynonymous	0	2
PPP2R3B	chrX	322273/G//A	nonsynonymous	0	2
ASMTL	chrX	1537994/T//C	nonsynonymous	0	2
ASMT	chrX	1734089/C//T	synonymous	0	2
MAGED1	chrX	51643343/G//A	nonsynonymous	0	2
HUWE1	chrX	53561125/C//T	nonsynonymous	0	2
AMER1	chrX	63410103/G//A	nonsynonymous	0	2
MTMR8	chrX	63551454/C//A	nonsynonymous	0	2
STARD8	chrX	67942449/C//T	synonymous	0	2
FOXO4	chrX	70322030/C//A	synonymous	0	2
HDX	chrX	83616611/G//A	nonsynonymous	0	2
GLRA4	chrX	102974160/C//T	nonsynonymous	0	2
CHRDL1	chrX	109931890/T//C	nonsynonymous	0	2
PAK3	chrX	110406161/G//A	nonsynonymous	0	2
KLHL13	chrX	117033329/C//T	nonsynonymous	0	2
NKRF	chrX	118724761/C//T	synonymous	0	2
ATP11C	chrX	138884438/G//A	nonsynonymous	0	2
MAMLD1	chrX	149639108/C//T	synonymous	0	2
L1CAM	chrX	153129444/G//A	synonymous	0	2
FLNA	chrX	153582589/G//A	synonymous	0	2
KDM5D	chrY	21869154/C//T	nonsynonymous	0	2
LCE1E	chr1	152760075/A//G	synonymous	1	1
GPR161	chr1	168066328/C//T	nonsynonymous	0	2
SWT1	chr1	185240519/G//A	nonsynonymous	0	2
HMCN1	chr1	185902887/G//A	nonsynonymous	0	2
LYST	chr1	235896886/G//C	nonsynonymous	0	2
GPR45	chr2	105858864/G//A	synonymous	0	2
NBEAL1	chr2	204000577/G//C	nonsynonymous	0	2
ADAM23	chr2	207413038/C//A	nonsynonymous	0	2
PASK	chr2	242079443/C//T	nonsynonymous	0	2
TRANK1	chr3	36884208/T//C	synonymous	0	2
ALS2CL	chr3	46727075/C//T	synonymous	0	2
FLNB	chr3	58116558/G//A	nonsynonymous	0	2
CLSTN2	chr3	140167507/C//T	nonsynonymous	0	2
ZNF518B	chr4	10446518/C//T	nonsynonymous	0	2
GABRA4	chr4	46979105/G//T	nonsynonymous	0	2
TXK	chr4	48069695/C//T	nonsynonymous	0	2
KIAA1211	chr4	57193837/C//T	nonsynonymous	0	2
SHROOM3	chr4	77661969/G//A	synonymous	0	2
ENPEP	chr4	111441161/C//G	synonymous	0	2
	chr4	155411654/C//T	synonymous	0	2
TERT	chr5	1268691/G//T	nonsynonymous	0	2
TRPC7	chr5	135601916/C//A	nonsynonymous	0	2
PCDHB1	chr5	140431238/G//A	synonymous	0	2
PCDHGA2	chr5	140719867/A//G	synonymous	0	2
BTN2A2	chr6	26390936/G//A	nonsynonymous	0	2
DOPEY1	chr6	83835322/C//T	nonsynonymous	0	2
DCBLD1	chr6	117890910/C//A	synonymous	0	2
SASH1	chr6	148865112/C//T	nonsynonymous	0	2
SYNE1	chr6	152702497/C//G	nonsynonymous	0	2
FNDC1	chr6	159650954/C//T	nonsynonymous	0	2
GDF6	chr8	97156774/C//T	synonymous	0	2
CSMD3	chr8	113353912/A//G	nonsynonymous	0	2
TBC1D31	chr8	124096564/C//T	nonsynonymous	0	2
TG	chr8	133980207/G//A	nonsynonymous	0	2
MPDZ	chr9	13113947/C//T	nonsynonymous	0	2
CKS2	chr9	91930086/CATGTT//C	nonsynonymous	0	2
ZNF462	chr9	109734437/C//T	synonymous	0	2
SUSD1	chr9	114874061/G//T	nonsynonymous	0	2
COL5A1	chr9	137620531/G//A	nonsynonymous	0	2
LHX3	chr9	139092464/C//T	nonsynonymous	0	2
NET1	chr10	5498989/A//T	synonymous	0	2
OPTN	chr10	13167560/G//C	nonsynonymous	0	2
GPR158	chr10	25887170/C//T	nonsynonymous	0	2
SYT15	chr10	46967489/G//T	nonsynonymous	0	2
JMJD1C	chr10	64974457/G//A	synonymous	0	2
NDST2	chr10	75564562/C//T	synonymous	0	2
CFAP43	chr10	105923958/C//T	nonsynonymous	0	2
MUC6	chr11	1016776/C//T	nonsynonymous	0	2
SSRP1	chr11	57100230/G//A	nonsynonymous	0	2

FLRT1	chr11	63884882/G//A	synonymous	0	2
	chr12	2613691/C//T	synonymous	0	2
FGFR1OP2	chr12	27117668/G//C	synonymous	0	2
KIF21A	chr12	39696768/T//C	nonsynonymous	0	2
PDZRN4	chr12	41582464/G//A	synonymous	0	2
KRT84	chr12	52771925/C//T	nonsynonymous	0	2
CCER1	chr12	91348463/G//A	synonymous	0	2
PITPNM2	chr12	123494508/C//T	nonsynonymous	0	2
TMEM132C	chr12	129181799/G//T	nonsynonymous	0	2
FREM2	chr13	39263318/C//T	nonsynonymous	0	2
AKAP11	chr13	42893308/G//A	nonsynonymous	0	2
CCDC168	chr13	103399942/C//T	synonymous	0	2
TMCO3	chr13	114203785/G//A	nonsynonymous	0	2
CGNL1	chr15	57730331/G//A	nonsynonymous	0	2
C15orf32	chr15	93015500/C//T	nonsynonymous	0	2
PGPEP1L	chr15	99514312/C//G	nonsynonymous	0	2
BAIAP3	chr16	1395354/G//C	nonsynonymous	0	2
MYH13	chr17	10248675/C//T	synonymous	0	2
COL1A1	chr17	48272117/C//T	nonsynonymous	0	2
ZNF236	chr18	74672718/G//A	nonsynonymous	0	2
MUM1	chr19	1360869/G//A	nonsynonymous	0	2
MKNK2	chr19	2042828/C//G	nonsynonymous	0	2
NFIC	chr19	3381846/C//T	nonsynonymous	0	2
FBN3	chr19	8131120/C//T	nonsynonymous	0	2
GMIP	chr19	19746370/C//T	nonsynonymous	0	2
ZNF675	chr19	23836886/G//T	synonymous	0	2
ZNF541	chr19	48047513/C//T	nonsynonymous	0	2
BPIFB4	chr20	31672750/G//A	nonsynonymous	0	2
MMP9	chr20	44641108/C//T	nonsynonymous	0	2
KCNB1	chr20	48098820/G//A	synonymous	0	2
KCNG1	chr20	49621205/C//T	nonsynonymous	0	2
RFPL2	chr22	32588988/G//A	nonsynonymous	0	2
ASMTL	chrX	1546924/C//T	synonymous	0	2
GRIK3	chr1	37324749/G//A	nonsynonymous	0	2
SZT2	chr1	43911607/T//C	synonymous	0	2
PIAS3	chr1	145585689/C//T	synonymous	0	2
TTC24	chr1	156555600/G//A	nonsynonymous	0	2
SPTA1	chr1	158612230/C//T	nonsynonymous	0	2
TNN	chr1	175046577/G//A	nonsynonymous	0	2
NMNAT2	chr1	183247737/C//T	nonsynonymous	0	2
SLC41A1	chr1	205779339/G//A	synonymous	0	2
LEFTY1	chr1	226075317/G//A	synonymous	0	2
NRXN1	chr2	50758479/G//A	nonsynonymous	0	2
LRP1B	chr2	141819727/G//A	synonymous	0	2
CRYGB	chr2	209007437/C//T	synonymous	0	2
UNC80	chr2	210858102/C//T	nonsynonymous	0	2
CCDC71	chr3	49200295/G//A	synonymous	0	2
CAMKV	chr3	49898393/T//C	synonymous	0	2
HYAL3	chr3	50332752/G//A	synonymous	0	2
DOCK3	chr3	51264853/G//A	nonsynonymous	0	2
LRTM1	chr3	54952771/G//A	synonymous	0	2
FOXP1	chr3	71064770/G//A	nonsynonymous	0	2
	chr3	156009817/C//T	synonymous	0	2
SEL1L3	chr4	25823612/A//C	synonymous	0	2
SHROOM3	chr4	77662615/C//T	nonsynonymous	0	2
GK2	chr4	80327881/G//A	nonsynonymous	0	2
FHDC1	chr4	153897467/G//T	nonsynonymous	0	2
LMBRD2	chr5	36105221/T//A	nonsynonymous	0	2
ERBIN	chr5	65321777/A//G	nonsynonymous	0	2
APC	chr5	112175407/AC//A	nonsynonymous	0	2
APC	chr5	112175745/CT//C	nonsynonymous	0	2
FBN2	chr5	127614507/C//T	nonsynonymous	0	2
SHROOM1	chr5	132158745/G//A	nonsynonymous	0	2
ARAP3	chr5	141041713/C//T	synonymous	0	2
CLK4	chr5	178030851/C//T	synonymous	0	2
NUP153	chr6	17661999/T//G	nonsynonymous	0	2
TNFAIP3	chr6	138199848/C//T	synonymous	0	2
GRM1	chr6	146350618/C//T	synonymous	0	2
NOX3	chr6	155750023/G//A	synonymous	0	2
HGF	chr7	81355310/C//T	nonsynonymous	0	2

ZAN	chr7	100377162/C//T	nonsynonymous	0	2
GRM8	chr7	126882872/C//T	synonymous	0	2
DENND2A	chr7	140301577/G//A	synonymous	0	2
CNTNAP2	chr7	146825886/T//G	nonsynonymous	0	2
DLGAP2	chr8	1616675/C//T	nonsynonymous	0	2
CSMD1	chr8	3205573/C//T	nonsynonymous	0	2
ASAP1	chr8	131088627/C//G	nonsynonymous	0	2
TG	chr8	134128912/G//A	nonsynonymous	0	2
GAPVD1	chr9	128094307/G//A	nonsynonymous	0	2
PLPP7	chr9	134183544/G//A	nonsynonymous	0	2
PRRC2B	chr9	134358213/C//T	synonymous	0	2
ARHGAP21	chr10	24874155/C//T	nonsynonymous	0	2
SVIL	chr10	29788094/C//T	synonymous	0	2
MRGPRX1	chr11	18956125/G//A	synonymous	0	2
PACSIN3	chr11	47204064/C//T	nonsynonymous	0	2
SSRP1	chr11	57097812/C//T	nonsynonymous	0	2
INTS4	chr11	77692580/C//T	nonsynonymous	0	2
CCDC83	chr11	85576266/G//A	synonymous	0	2
FAT3	chr11	92086978/G//A	nonsynonymous	0	2
BSX	chr11	122848537/G//A	synonymous	0	2
IGSF9B	chr11	133792490/C//T	nonsynonymous	0	2
AKAP3	chr12	4736349/A//T	synonymous	0	2
OLR1	chr12	10319305/C//A	synonymous	0	2
ANKRD52	chr12	56641925/G//A	synonymous	0	2
SVOP	chr12	109313524/G//A	nonsynonymous	0	2
PABPC3	chr13	25670728/T//C	nonsynonymous	0	2
ATP8A2	chr13	26273469/G//A	synonymous	0	2
BRCA2	chr13	32912807/G//A	nonsynonymous	0	2
MYCBP2	chr13	77732230/A//G	synonymous	0	2
NALCN	chr13	101707798/C//T	nonsynonymous	0	2
CMTM5	chr14	23848689/A//G	nonsynonymous	0	2
CDC42BPB	chr14	103400173/G//A	nonsynonymous	0	2
ATP10A	chr15	25924912/T//G	nonsynonymous	0	2
SPTBN5	chr15	42143294/G//A	nonsynonymous	0	2
EPB42	chr15	43498739/G//A	synonymous	0	2
CHRNB4	chr15	78921761/C//T	nonsynonymous	0	2
ADAMTS7	chr15	79054874/G//A	nonsynonymous	0	2
HDDC3	chr15	91475331/C//G	nonsynonymous	0	2
UNC45A	chr15	91493395/G//T	synonymous	0	2
ITGAX	chr16	31382732/G//A	nonsynonymous	0	2
TSNAXIP1	chr16	67859987/C//A	synonymous	0	2
MPO	chr17	56356442/G//A	nonsynonymous	0	2
MRC2	chr17	60742265/C//T	nonsynonymous	0	2
CTDP1	chr18	77474948/G//A	synonymous	0	2
MOB3A	chr19	2078472/C//T	nonsynonymous	0	2
DUS3L	chr19	5787133/C//T	nonsynonymous	0	2
ARMC6	chr19	19166661/G//A	nonsynonymous	0	2
SHANK1	chr19	51169888/G//A	nonsynonymous	0	2
CD33	chr19	51738500/C//A	synonymous	0	2
MCM8	chr20	5974312/C//T	nonsynonymous	0	2
SEL1L2	chr20	13912406/G//A	synonymous	0	2
MMP24	chr20	33862134/G//A	nonsynonymous	0	2
DSCAM	chr21	41414371/G//A	synonymous	0	2
MYO18B	chr22	26351313/C//A	synonymous	0	2
ASPHD2	chr22	26830091/G//A	synonymous	0	2
RFPL2	chr22	32586994/C//T	nonsynonymous	0	2
RBMXL3	chrX	114424866/G//A	nonsynonymous	0	2
FMR1NB	chrX	147063000/G//T	synonymous	0	2
GPR50	chrX	150348653/C//T	nonsynonymous	0	2
SFN	chr1	27190247/G//A	nonsynonymous	0	2
PLEKHA6	chr1	204199576/C//T	nonsynonymous	0	2
PSME4	chr2	54197692/C//T	nonsynonymous	0	2
SFTPB	chr2	85895292/G//A	synonymous	0	2
ST6GAL2	chr2	107460028/C//T	nonsynonymous	0	2
AMER3	chr2	131520676/C//A	nonsynonymous	0	2
LRRN1	chr3	3887847/G//A	nonsynonymous	0	2
SCN11A	chr3	38936379/G//T	nonsynonymous	0	2
SLC9C1	chr3	111981886/C//T	nonsynonymous	0	2
SOX14	chr3	137484329/G//T	nonsynonymous	0	2
ZMAT3	chr3	178785600/G//T	synonymous	0	2

FXR1	chr3	180685919/G//A	nonsynonymous	0	2
ZNF518B	chr4	10446350/C//T	nonsynonymous	0	2
SLC34A2	chr4	25671422/T//C	synonymous	0	2
FRYL	chr4	48564969/C//T	synonymous	0	2
SPATA18	chr4	52917854/G//C	synonymous	0	2
MCUB	chr4	110608666/G//T	synonymous	0	2
ARSJ	chr4	114824634/G//A	nonsynonymous	0	2
CTNND2	chr5	10973744/A//G	nonsynonymous	0	2
ZNF366	chr5	71756416/T//C	nonsynonymous	0	2
RASA1	chr5	86672283/TA//T	nonsynonymous	0	2
DND1	chr5	140050879/C//T	synonymous	0	2
FGF18	chr5	170883614/G//A	synonymous	0	2
ADAMTS2	chr5	178581070/C//T	synonymous	0	2
ATXN1	chr6	16327189/C//A	synonymous	0	2
HSPA1L	chr6	31778643/C//A	nonsynonymous	0	2
RIMS1	chr6	72806829/G//T	synonymous	0	2
MDN1	chr6	90468573/G//C	nonsynonymous	0	2
CDHR3	chr7	105644966/C//G	synonymous	0	2
KIAA1549	chr7	138603515/C//T	nonsynonymous	0	2
PXDNL	chr8	52233403/C//A	nonsynonymous	0	2
ZFAT	chr8	135490850/C//T	nonsynonymous	0	2
NRBP2	chr8	144918340/A//G	nonsynonymous	0	2
CDC37L1	chr9	4679812/G//T	synonymous	0	2
WNK2	chr9	96055116/G//T	nonsynonymous	0	2
LCNL1	chr9	139877888/T//G	synonymous	0	2
ITGA8	chr10	15646332/C//G	nonsynonymous	0	2
OR5B12	chr11	58206724/T//A	nonsynonymous	0	2
AMOTL1	chr11	94599103/A//G	synonymous	0	2
LRRK2	chr12	40653312/G//A	synonymous	0	2
PA2G4	chr12	56505284/C//T	nonsynonymous	0	2
MAP3K9	chr14	71199973/G//A	nonsynonymous	0	2
TRPM1	chr15	31339375/C//T	nonsynonymous	0	2
ACAN	chr15	89398415/A//T	nonsynonymous	0	2
DNAH9	chr17	11835360/G//A	synonymous	0	2
CD226	chr18	67614075/G//C	nonsynonymous	0	2
CBARP	chr19	1236020/C//T	nonsynonymous	0	2
ZNF709	chr19	12595507/C//A	synonymous	0	2
DAND5	chr19	13084480/C//T	synonymous	0	2
PDE4C	chr19	18321876/C//G	nonsynonymous	0	2
SIRPA	chr20	1905506/G//A	nonsynonymous	0	2
TCF20	chr22	42606234/A//T	nonsynonymous	0	2
FTHL17	chrX	31089841/C//A	nonsynonymous	0	2
AFF2	chrX	148062286/G//T	nonsynonymous	0	2
CHD5	chr1	6212524/C//A	nonsynonymous	0	2
RERE	chr1	8421820/C//A	synonymous	0	2
EXOSC10	chr1	11132229/C//A	nonsynonymous	0	2
VPS13D	chr1	12378286/A//T	nonsynonymous	0	2
TMEM51	chr1	15541865/C//T	synonymous	0	2
AKR7A3	chr1	19612823/C//A	synonymous	0	2
HTR1D	chr1	23519623/G//T	synonymous	0	2
ASAP3	chr1	23767610/T//C	nonsynonymous	0	2
SFN	chr1	27190066/G//A	synonymous	0	2
ADGRB2	chr1	32209896/G//C	nonsynonymous	0	2
ZMYM4	chr1	35864526/G//A	nonsynonymous	0	2
KANK4	chr1	62739327/G//T	synonymous	0	2
	chr1	67394582/A//C	synonymous	0	2
FPGT-TNNI3K	chr1	74954908/A//T	nonsynonymous	0	2
ERICH3	chr1	75037615/C//A	nonsynonymous	0	2
LHX8	chr1	75614269/C//A	synonymous	0	2
MSH4	chr1	76262831/C//G	nonsynonymous	0	2
ADGRL4	chr1	79356884/G//T	nonsynonymous	0	2
GBP7	chr1	89607329/T//A	synonymous	0	2
TGFBR3	chr1	92327097/C//G	synonymous	0	2
SLC30A7	chr1	101361838/A//C	nonsynonymous	0	2
MAB21L3	chr1	116666872/G//T	nonsynonymous	0	2
TTF2	chr1	117618069/G//T	nonsynonymous	0	2
HDGF	chr1	156714083/C//A	nonsynonymous	0	2
CD1E	chr1	158325808/C//A	nonsynonymous	0	2
OR6Y1	chr1	158517761/C//A	synonymous	0	2
SPTA1	chr1	158612691/G//T	nonsynonymous	0	2

SPTA1	chr1	158648246/C//A	nonsynonymous	0	2
FASLG	chr1	172635001/A//T	nonsynonymous	0	2
TNN	chr1	175086182/A//T	nonsynonymous	0	2
RFWD2	chr1	175957433/T//C	nonsynonymous	0	2
ASTN1	chr1	176926953/A//T	nonsynonymous	0	2
BRINP2	chr1	177250008/G//T	nonsynonymous	0	2
SEC16B	chr1	177902353/G//C	nonsynonymous	0	2
CACNA1E	chr1	181686338/C//T	synonymous	0	2
KCNT2	chr1	196448370/T//A	nonsynonymous	0	2
NEK7	chr1	198247197/G//C	nonsynonymous	0	2
IGFN1	chr1	201175838/G//A	nonsynonymous	0	2
ARL8A	chr1	202107134/G//A	synonymous	0	2
LGR6	chr1	202273732/C//A	nonsynonymous	0	2
LAX1	chr1	203734577/G//A	synonymous	0	2
IRF6	chr1	209974709/T//A	nonsynonymous	0	2
PROX1	chr1	214170032/C//G	nonsynonymous	0	2
CENPF	chr1	214830446/G//T	nonsynonymous	0	2
USH2A	chr1	215960185/G//T	nonsynonymous	0	2
HIST3H2BB	chr1	228645823/C//G	synonymous	0	2
CHRM3	chr1	240072512/G//A	synonymous	0	2
FMN2	chr1	240286487/C//A	nonsynonymous	0	2
PLD5	chr1	242263997/T//C	nonsynonymous	0	2
C1orf101	chr1	244747231/T//C	nonsynonymous	0	2
SMYD3	chr1	245927427/G//A	synonymous	0	2
OR2M7	chr1	248487220/G//T	synonymous	0	2
OR2T4	chr1	248525769/C//A	nonsynonymous	0	2
OR2T1	chr1	248570384/G//T	nonsynonymous	0	2
SLC30A3	chr2	27485845/G//C	synonymous	0	2
	chr2	27851313/G//A	synonymous	0	2
GPN1	chr2	27858005/A//C	nonsynonymous	0	2
HEATR5B	chr2	37284496/C//G	nonsynonymous	0	2
NRXN1	chr2	50463927/T//A	synonymous	0	2
CCDC88A	chr2	55523569/C//T	nonsynonymous	0	2
EXOC6B	chr2	72411226/T//C	nonsynonymous	0	2
LRRTM1	chr2	80529396/C//A	nonsynonymous	0	2
LRRTM1	chr2	80530742/A//G	nonsynonymous	0	2
MRPL35	chr2	86437838/G//T	synonymous	0	2
SMYD1	chr2	88387486/G//T	synonymous	0	2
TEX37	chr2	88828810/G//C	nonsynonymous	0	2
SNRNP200	chr2	96949050/C//A	nonsynonymous	0	2
VWA3B	chr2	98866845/C//G	nonsynonymous	0	2
ZC3H6	chr2	113088767/C//T	nonsynonymous	0	2
ACTR3	chr2	114691893/A//G	nonsynonymous	0	2
GPR148	chr2	131486845/G//C	nonsynonymous	0	2
TMEM163	chr2	135309638/G//T	nonsynonymous	0	2
LCT	chr2	136575266/T//A	nonsynonymous	0	2
LCT	chr2	136581525/C//A	nonsynonymous	0	2
SCN3A	chr2	165948805/C//G	nonsynonymous	0	2
SCN9A	chr2	167149789/G//T	synonymous	0	2
XIRP2	chr2	168107311/A//G	nonsynonymous	0	2
LRP2	chr2	170129550/C//A	nonsynonymous	0	2
TTN	chr2	179463475/C//T	nonsynonymous	0	2
TTN	chr2	179485026/C//A	nonsynonymous	0	2
TTN	chr2	179547565/G//A	nonsynonymous	0	2
TTN	chr2	179550271/T//A	nonsynonymous	0	2
TTN	chr2	179596128/G//T	nonsynonymous	0	2
SLC40A1	chr2	190428466/C//A	nonsynonymous	0	2
MYO1B	chr2	192206295/G//C	synonymous	0	2
HECW2	chr2	197183391/C//A	synonymous	0	2
PARD3B	chr2	206480263/G//T	nonsynonymous	0	2
PIKFYVE	chr2	209190574/A//T	nonsynonymous	0	2
MYL1	chr2	211158451/G//C	nonsynonymous	0	2
CPS1	chr2	211540525/C//A	nonsynonymous	0	2
ABCA12	chr2	215840523/C//A	nonsynonymous	0	2
CFAP65	chr2	219888009/C//A	nonsynonymous	0	2
COL4A4	chr2	227985872/G//A	synonymous	0	2
GPR55	chr2	231775621/C//A	nonsynonymous	0	2
BTD	chr3	15686612/G//T	nonsynonymous	0	2
STAC	chr3	36570420/T//G	nonsynonymous	0	2
XYLB	chr3	38437001/A//T	nonsynonymous	0	2

SCN11A	chr3	38951602/C//A	nonsynonymous	0	2
ZNF662	chr3	42955820/G//T	nonsynonymous	0	2
SLC6A20	chr3	45817309/G//C	nonsynonymous	0	2
CELSR3	chr3	48699319/C//T	nonsynonymous	0	2
USP19	chr3	49148217/G//A	nonsynonymous	0	2
POC1A	chr3	52183383/G//A	nonsynonymous	0	2
ZNF717	chr3	75787885/C//G	nonsynonymous	0	2
HTR1F	chr3	88040450/A//T	nonsynonymous	0	2
GOLGB1	chr3	121414823/C//G	nonsynonymous	0	2
PRR23A	chr3	138724764/T//C	nonsynonymous	0	2
ZNF518B	chr4	10445561/C//G	nonsynonymous	0	2
CLRN2	chr4	17516844/C//G	synonymous	0	2
SLIT2	chr4	20591307/C//A	nonsynonymous	0	2
YIPF7	chr4	44624484/C//G	nonsynonymous	0	2
IGFBP7	chr4	57976223/C//A	nonsynonymous	0	2
SCARB2	chr4	77097682/C//T	nonsynonymous	0	2
RAP1GDS1	chr4	99339905/T//A	nonsynonymous	0	2
UCP1	chr4	141484332/A//G	nonsynonymous	0	2
FSTL5	chr4	162841607/G//A	synonymous	0	2
AADAT	chr4	170990300/C//A	nonsynonymous	0	2
SNX25	chr4	186263193/G//A	nonsynonymous	0	2
MTNR1A	chr4	187455092/C//T	nonsynonymous	0	2
ICE1	chr5	5464912/C//G	nonsynonymous	0	2
DNAH5	chr5	13820544/G//A	nonsynonymous	0	2
DNAH5	chr5	13901681/A//G	synonymous	0	2
RAI14	chr5	34823757/G//A	nonsynonymous	0	2
EGFLAM	chr5	38418259/G//A	nonsynonymous	0	2
MROH2B	chr5	41008715/G//T	nonsynonymous	0	2
FNIP1	chr5	131007720/C//A	nonsynonymous	0	2
PCDHA2	chr5	140176423/G//T	nonsynonymous	0	2
PCDHA4	chr5	140187486/T//A	nonsynonymous	0	2
PCDHA5	chr5	140201827/C//A	nonsynonymous	0	2
PCDHA12	chr5	140255577/C//A	nonsynonymous	0	2
ABLIM3	chr5	148577960/G//C	nonsynonymous	0	2
CSF1R	chr5	149440436/C//A	nonsynonymous	0	2
FAT2	chr5	150922572/C//A	nonsynonymous	0	2
HAVCR1	chr5	156464346/C//G	synonymous	0	2
DOCK2	chr5	169101423/C//A	synonymous	0	2
HRH2	chr5	175110521/C//A	synonymous	0	2
RAB24	chr5	176729444/C//A	synonymous	0	2
SLC22A23	chr6	3456218/C//T	synonymous	0	2
HIST1H4C	chr6	26104268/A//G	synonymous	0	2
UHRF1BP1	chr6	34825965/C//T	nonsynonymous	0	2
TDRD6	chr6	46657805/A//G	nonsynonymous	0	2
ADGRF5	chr6	46827007/C//A	nonsynonymous	0	2
PKHD1	chr6	51768810/C//A	nonsynonymous	0	2
EYS	chr6	66204829/A//C	nonsynonymous	0	2
SNAP91	chr6	84269927/G//T	nonsynonymous	0	2
HTR1E	chr6	87725628/T//A	nonsynonymous	0	2
GRIK2	chr6	102130487/T//C	synonymous	0	2
AIM1	chr6	106999768/A//G	nonsynonymous	0	2
EPM2A	chr6	145948833/C//A	synonymous	0	2
STXBP5	chr6	147680334/C//T	nonsynonymous	0	2
LATS1	chr6	150004316/G//C	nonsynonymous	0	2
TNRC18	chr7	5353058/C//A	synonymous	0	2
CREB5	chr7	28610048/C//A	nonsynonymous	0	2
NPC1L1	chr7	44579349/A//T	nonsynonymous	0	2
ZNF107	chr7	64168438/G//T	nonsynonymous	0	2
ANKIB1	chr7	91991584/G//C	nonsynonymous	0	2
ZAN	chr7	100366281/A//T	nonsynonymous	0	2
GPR22	chr7	107115491/T//A	nonsynonymous	0	2
KCND2	chr7	119915005/C//T	nonsynonymous	0	2
GRM8	chr7	126173756/C//A	nonsynonymous	0	2
WEE2	chr7	141418917/G//C	nonsynonymous	0	2
OR9A2	chr7	142723347/G//T	nonsynonymous	0	2
OR2A2	chr7	143807107/T//A	synonymous	0	2
CUL1	chr7	148457524/G//C	nonsynonymous	0	2
SLC4A2	chr7	150767104/C//T	synonymous	0	2
SLC4A2	chr7	150767107/CTCTGACCAGA//C	nonsynonymous	0	2
CSMD1	chr8	2820776/C//A	nonsynonymous	0	2

	chr8	18666343/C//A	synonymous	0	2
FNTA	chr8	42939847/T//C	synonymous	0	2
PRKDC	chr8	48701591/C//G	nonsynonymous	0	2
CLVS1	chr8	62212743/G//T	nonsynonymous	0	2
SLCO5A1	chr8	70744153/C//T	synonymous	0	2
ZFHX4	chr8	77763273/C//A	nonsynonymous	0	2
LRRCC1	chr8	86025171/G//C	nonsynonymous	0	2
CNGB3	chr8	87591331/T//A	synonymous	0	2
PKHD1L1	chr8	110425663/A//G	nonsynonymous	0	2
	chr8	114389032/G//C	synonymous	0	2
SAMD12	chr8	119593092/A//G	synonymous	0	2
DEPTOR	chr8	120977579/G//T	nonsynonymous	0	2
ZFAT-AS1	chr8	135612565/C//G	synonymous	0	2
ZFAT-AS1	chr8	135612630/C//T	synonymous	0	2
ZFAT	chr8	135612747/C//T	nonsynonymous	0	2
ZFAT	chr8	135613729/C//G	nonsynonymous	0	2
ZFAT	chr8	135613760/C//T	synonymous	0	2
ZFAT	chr8	135614179/C//G	nonsynonymous	0	2
FAM135B	chr8	139380161/G//C	synonymous	0	2
MROH5	chr8	142505489/G//T	synonymous	0	2
LYPD2	chr8	143833806/C//G	synonymous	0	2
ZC3H3	chr8	144620978/C//A	nonsynonymous	0	2
CCDC166	chr8	144789088/C//T	nonsynonymous	0	2
TONSL	chr8	145667801/G//C	synonymous	0	2
RFX3	chr9	3288257/T//C	synonymous	0	2
RRAGA	chr9	19049986/T//A	nonsynonymous	0	2
	chr9	36276922/A//T	synonymous	0	2
TMEM252	chr9	71152335/G//C	nonsynonymous	0	2
VPS13A	chr9	79936407/G//T	nonsynonymous	0	2
SPATA31C2	chr9	90747621/G//A	synonymous	0	2
WNK2	chr9	96021465/A//T	nonsynonymous	0	2
SVEP1	chr9	113252047/C//A	nonsynonymous	0	2
RGS3	chr9	116276820/G//A	synonymous	0	2
OR1Q1	chr9	125377472/C//G	synonymous	0	2
OR1B1	chr9	125391222/A//G	nonsynonymous	0	2
LCN9	chr9	138557700/C//A	synonymous	0	2
NOTCH1	chr9	139409060/GC//G	nonsynonymous	0	2
CACNB2	chr10	18789761/G//T	nonsynonymous	0	2
SKIDA1	chr10	21805270/G//A	synonymous	0	2
ZEB1	chr10	31815730/C//T	synonymous	0	2
A1CF	chr10	52595889/G//C	nonsynonymous	0	2
DUSP13	chr10	76861669/G//T	synonymous	0	2
RBM20	chr10	112572225/G//T	synonymous	0	2
GPAM	chr10	113931957/G//A	synonymous	0	2
DMBT1	chr10	124395581/G//A	nonsynonymous	0	2
CUZD1	chr10	124596394/C//A	nonsynonymous	0	2
MKI67	chr10	129899585/C//A	nonsynonymous	0	2
KNDC1	chr10	134997384/C//A	synonymous	0	2
MUC5B	chr11	1278761/C//A	nonsynonymous	0	2
OR51S1	chr11	4870247/G//A	synonymous	0	2
OR51L1	chr11	5020447/A//G	nonsynonymous	0	2
OR51V1	chr11	5221004/A//G	synonymous	0	2
OR51B2	chr11	5345261/C//A	nonsynonymous	0	2
DCHS1	chr11	6661662/C//T	nonsynonymous	0	2
RBMXL2	chr11	7111247/C//T	nonsynonymous	0	2
C11orf58	chr11	16760356/G//T	nonsynonymous	0	2
SLC6A5	chr11	20628563/C//G	synonymous	0	2
ANO5	chr11	22232858/A//G	nonsynonymous	0	2
HIPK3	chr11	33308441/C//A	nonsynonymous	0	2
OR4C3	chr11	48347219/T//C	nonsynonymous	0	2
TRIM49B	chr11	49059135/C//A	nonsynonymous	0	2
TRIM64C	chr11	49080622/A//G	nonsynonymous	0	2
OR5D18	chr11	55587141/G//T	synonymous	0	2
OR8K5	chr11	55927379/G//T	synonymous	0	2
OR8K5	chr11	55927549/A//T	nonsynonymous	0	2
OR5R1	chr11	56185472/C//A	synonymous	0	2
OR5M9	chr11	56230581/G//T	nonsynonymous	0	2
OR10V1	chr11	59481189/T//C	nonsynonymous	0	2
TCN1	chr11	59620441/G//C	synonymous	0	2
FADS1	chr11	61578349/C//A	synonymous	0	2

SLC22A6	chr11	62749411/G//A	nonsynonymous	0	2
PLA2G16	chr11	63357680/G//T	synonymous	0	2
TRPT1	chr11	63993025/G//A	synonymous	0	2
CDC42BPG	chr11	64601973/G//A	nonsynonymous	0	2
ATG2A	chr11	64675238/T//C	nonsynonymous	0	2
RNASEH2C	chr11	65486798/C//A	synonymous	0	2
CTSF	chr11	66332107/C//A	nonsynonymous	0	2
FAT3	chr11	92533247/A//T	nonsynonymous	0	2
SLC36A4	chr11	92917632/T//A	nonsynonymous	0	2
CEP295	chr11	93416837/G//T	nonsynonymous	0	2
ATM	chr11	108213947/A//C	nonsynonymous	0	2
NLRX1	chr11	119045506/T//C	synonymous	0	2
GRIK4	chr11	120690570/C//A	nonsynonymous	0	2
GRIK4	chr11	120852871/G//A	nonsynonymous	0	2
NTM	chr11	132177612/T//C	synonymous	0	2
OPCML	chr11	132307246/C//A	nonsynonymous	0	2
RERG	chr12	15261942/C//A	synonymous	0	2
COL2A1	chr12	48383568/A//G	synonymous	0	2
KRT79	chr12	53216153/C//A	nonsynonymous	0	2
OR6C75	chr12	55759253/G//T	nonsynonymous	0	2
HELB	chr12	66718866/G//T	nonsynonymous	0	2
OTOGL	chr12	80651698/T//G	nonsynonymous	0	2
PTPRQ	chr12	81043451/A//G	synonymous	0	2
EPYC	chr12	91396267/T//C	nonsynonymous	0	2
FAM71C	chr12	100042387/C//G	synonymous	0	2
NAA25	chr12	112509722/C//A	nonsynonymous	0	2
HECTD4	chr12	112746659/T//A	synonymous	0	2
SETD1B	chr12	122265953/C//T	nonsynonymous	0	2
HIP1R	chr12	123340864/C//A	synonymous	0	2
TMEM132D	chr12	130184653/C//A	nonsynonymous	0	2
TMEM132D	chr12	130184823/C//A	nonsynonymous	0	2
PIWIL1	chr12	130833898/G//C	nonsynonymous	0	2
NBEA	chr13	35730265/G//T	nonsynonymous	0	2
SOHLH2	chr13	36748947/C//A	nonsynonymous	0	2
AKAP11	chr13	42876025/A//G	nonsynonymous	0	2
ERICH6B	chr13	46124139/T//A	nonsynonymous	0	2
ATP7B	chr13	52548606/C//A	synonymous	0	2
PCDH17	chr13	58298856/C//A	nonsynonymous	0	2
CCDC168	chr13	103410788/A//C	nonsynonymous	0	2
OR11H6	chr14	20692168/A//C	synonymous	0	2
LRFN5	chr14	42356155/C//A	nonsynonymous	0	2
MDGA2	chr14	47343334/T//A	nonsynonymous	0	2
CGRRF1	chr14	55004525/A//T	nonsynonymous	0	2
C14orf37	chr14	58605202/C//A	nonsynonymous	0	2
KIAA0586	chr14	58909512/A//T	nonsynonymous	0	2
RHOJ	chr14	63757666/C//T	nonsynonymous	0	2
CHURC1-FNTB	chr14	65521353/C//A	nonsynonymous	0	2
GALNT16	chr14	69791433/G//A	synonymous	0	2
PROX2	chr14	75325138/A//T	nonsynonymous	0	2
RPS6KA5	chr14	91526874/G//A	synonymous	0	2
CCDC88C	chr14	91739252/G//T	nonsynonymous	0	2
SETD3	chr14	99872906/C//T	nonsynonymous	0	2
NIPA1	chr15	23049107/C//A	nonsynonymous	0	2
GABRB3	chr15	26825601/C//A	nonsynonymous	0	2
KATNBL1	chr15	34437613/C//A	nonsynonymous	0	2
TP53BP1	chr15	43766960/G//A	nonsynonymous	0	2
WDR72	chr15	54003101/T//C	nonsynonymous	0	2
ADAM10	chr15	58974515/T//C	nonsynonymous	0	2
NEO1	chr15	73570499/G//T	nonsynonymous	0	2
CHRNA3	chr15	78909398/C//A	nonsynonymous	0	2
MEX3B	chr15	82335938/C//G	nonsynonymous	0	2
NTRK3	chr15	88678509/G//T	synonymous	0	2
LRRC28	chr15	99926345/C//T	synonymous	0	2
ADAMTS17	chr15	100657103/C//A	nonsynonymous	0	2
WDR24	chr16	735508/C//A	nonsynonymous	0	2
ZNF598	chr16	2051608/C//T	nonsynonymous	0	2
PKD1	chr16	2161136/C//T	synonymous	0	2
BICDL2	chr16	3078086/G//T	synonymous	0	2
UBALD1	chr16	4664821/C//A	synonymous	0	2
ERCC4	chr16	14029212/A//G	nonsynonymous	0	2

ZNF843	chr16	31447015/A//T	synonymous	0	2
	chr16	31476600/G//T	synonymous	0	2
FTO	chr16	53859862/C//T	synonymous	0	2
VAC14	chr16	70726804/C//A	nonsynonymous	0	2
IST1	chr16	71950412/G//A	nonsynonymous	0	2
PSMD7	chr16	74339224/C//T	nonsynonymous	0	2
KLHDC4	chr16	87744948/C//A	nonsynonymous	0	2
ZFPM1	chr16	88555480/G//T	nonsynonymous	0	2
MYBBP1A	chr17	4451951/A//T	synonymous	0	2
NLRP1	chr17	5487007/C//T	nonsynonymous	0	2
WSCD1	chr17	5993699/C//G	nonsynonymous	0	2
TP53	chr17	7578469/C//A	nonsynonymous	0	2
MYH4	chr17	10356576/C//A	nonsynonymous	0	2
MYH2	chr17	10447429/G//T	nonsynonymous	0	2
DNAH9	chr17	11833256/C//G	nonsynonymous	0	2
FBXW10	chr17	18671949/G//T	nonsynonymous	0	2
ULK2	chr17	19728500/G//A	synonymous	0	2
KIAA0100	chr17	26947641/C//A	synonymous	0	2
TOP2A	chr17	38546373/G//A	synonymous	0	2
GHDC	chr17	40341850/C//A	synonymous	0	2
SLC25A39	chr17	42397446/C//A	nonsynonymous	0	2
	chr17	49123901/C//T	synonymous	0	2
CA10	chr17	49710994/C//A	synonymous	0	2
MRPS23	chr17	55917211/T//A	nonsynonymous	0	2
ABCA10	chr17	67218716/C//A	nonsynonymous	0	2
TBC1D16	chr17	77984543/C//A	nonsynonymous	0	2
L3MBTL4	chr18	5956236/G//A	nonsynonymous	0	2
PTPRM	chr18	7906549/T//G	nonsynonymous	0	2
ASXL3	chr18	31187580/C//G	nonsynonymous	0	2
HNRNPM	chr19	8551017/G//T	nonsynonymous	0	2
MUC16	chr19	9070314/T//C	nonsynonymous	0	2
ECSIT	chr19	11616997/G//C	synonymous	0	2
PDE4C	chr19	18332115/G//T	synonymous	0	2
ANKRD27	chr19	33098684/C//A	nonsynonymous	0	2
ZNF573	chr19	38229596/G//C	nonsynonymous	0	2
MEGF8	chr19	42838302/G//C	synonymous	0	2
NANOS2	chr19	46417556/G//T	synonymous	0	2
CCDC8	chr19	46915654/C//T	synonymous	0	2
KCNJ14	chr19	48965568/A//T	nonsynonymous	0	2
SPHK2	chr19	49131436/A//T	synonymous	0	2
POLD1	chr19	50905326/G//C	synonymous	0	2
SIGLEC14	chr19	52148732/G//T	nonsynonymous	0	2
ZNF613	chr19	52448027/C//T	synonymous	0	2
PRKCG	chr19	54387498/G//A	nonsynonymous	0	2
NDUFA3	chr19	54606163/G//T	synonymous	0	2
KIR3DL1	chr19	55341789/C//T	synonymous	0	2
HSPBP1	chr19	55789060/C//G	nonsynonymous	0	2
NLRP8	chr19	56466869/C//A	nonsynonymous	0	2
ZSCAN5A	chr19	56735187/T//A	nonsynonymous	0	2
PEG3	chr19	57328051/C//A	nonsynonymous	0	2
ZIM2	chr19	57334187/G//A	nonsynonymous	0	2
GPCPD1	chr20	5559080/G//T	nonsynonymous	0	2
ACSS1	chr20	25038556/C//G	synonymous	0	2
ZNF337	chr20	25657150/G//A	synonymous	0	2
	chr20	35421837/C//A	synonymous	0	2
TTI1	chr20	36641100/G//T	synonymous	0	2
CHD6	chr20	40076561/C//G	nonsynonymous	0	2
PTPRT	chr20	40710572/G//A	nonsynonymous	0	2
ZSWIM1	chr20	44511699/C//G	nonsynonymous	0	2
ARFGEF2	chr20	47585711/G//T	nonsynonymous	0	2
CABLES2	chr20	60971418/C//A	nonsynonymous	0	2
KCNQ2	chr20	62076018/G//A	synonymous	0	2
HELZ2	chr20	62195052/T//A	nonsynonymous	0	2
TPTE	chr21	10944742/G//T	synonymous	0	2
SAMSN1	chr21	15882666/G//C	nonsynonymous	0	2
C21orf91	chr21	19165676/T//C	synonymous	0	2
DSCAM	chr21	42080507/G//A	synonymous	0	2
TMPRSS3	chr21	43792845/C//G	synonymous	0	2
AIRE	chr21	45711025/C//G	nonsynonymous	0	2
CCDC116	chr22	21987450/G//T	synonymous	0	2

SEZ6L	chr22	26761338/C//A	nonsynonymous	0	2
FOXRED2	chr22	36892089/C//A	nonsynonymous	0	2
MGAT3	chr22	39883425/T//G	nonsynonymous	0	2
EFCAB6	chr22	43924828/A//G	nonsynonymous	0	2
TAB3	chrX	30870917/C//A	nonsynonymous	0	2
FAM47B	chrX	34962178/C//G	synonymous	0	2
KDM6A	chrX	44945108/A//T	nonsynonymous	0	2
CACNA1F	chrX	49072873/A//C	nonsynonymous	0	2
GSPT2	chrX	51488437/G//T	nonsynonymous	0	2
RRAGB	chrX	55757982/A//G	nonsynonymous	0	2
CHM	chrX	85302540/G//A	synonymous	0	2
PCDH11X	chrX	91090714/G//C	nonsynonymous	0	2
SAGE1	chrX	134992313/G//A	nonsynonymous	0	2
SPANXD	chrX	140785632/G//T	nonsynonymous	0	2
FLNA	chrX	153593044/G//A	synonymous	0	2
F8	chrX	154088827/T//A	synonymous	0	2
PRDM16	chr1	3347458/G//A	nonsynonymous	0	2
CASP9	chr1	15831193/C//T	nonsynonymous	0	2
MYOM3	chr1	24419473/C//T	nonsynonymous	0	2
HFM1	chr1	91733357/A//T	nonsynonymous	0	2
CREG1	chr1	167511467/G//A	synonymous	0	2
MAP3K21	chr1	233482322/G//A	nonsynonymous	0	2
HOXD12	chr2	176965257/G//A	synonymous	0	2
AAMP	chr2	219129266/G//A	nonsynonymous	0	2
SLC6A20	chr3	45800504/C//T	nonsynonymous	0	2
QRICH1	chr3	49114190/TTG//T	nonsynonymous	0	2
BSN	chr3	49701983/G//A	synonymous	0	2
ATR	chr3	142266595/G//A	nonsynonymous	0	2
TM4SF18	chr3	149051052/T//G	nonsynonymous	0	2
TRIML2	chr4	189022244/G//A	nonsynonymous	0	2
OSMR	chr5	38925448/G//A	synonymous	0	2
DDX41	chr5	176939620/C//T	nonsynonymous	0	2
PTCHD4	chr6	47847282/G//A	nonsynonymous	0	2
TINAG	chr6	54186103/C//G	nonsynonymous	0	2
L3MBTL3	chr6	130392142/C//T	nonsynonymous	0	2
TAAR9	chr6	132859485/C//T	synonymous	0	2
CCDC170	chr6	151894591/C//A	synonymous	0	2
DNAAF5	chr7	794233/C//T	synonymous	0	2
STAG3	chr7	99778178/G//A	synonymous	0	2
ACHE	chr7	100490017/C//T	synonymous	0	2
ATP6V0A4	chr7	138417706/G//A	synonymous	0	2
TMEM178B	chr7	140912481/G//A	nonsynonymous	0	2
LGI3	chr8	22005642/A//G	synonymous	0	2
MCM4	chr8	48875347/C//T	synonymous	0	2
ZNF623	chr8	144733070/G//A	nonsynonymous	0	2
CLTA	chr9	36211661/C//T	nonsynonymous	0	2
BICD2	chr9	95482989/TCTC//T	nonsynonymous	0	2
WNK2	chr9	96055334/A//T	nonsynonymous	0	2
AKNA	chr9	117120450/G//A	synonymous	0	2
ANKRD30A	chr10	37431046/G//A	synonymous	0	2
C10orf71	chr10	50531955/C//T	synonymous	0	2
SRSF8	chr11	94801131/T//C	synonymous	0	2
KCNA5	chr12	5154394/G//A	nonsynonymous	0	2
NACA	chr12	57114715/T//C	nonsynonymous	0	2
SLC16A7	chr12	60168559/G//A	synonymous	0	2
ORAI1	chr12	122079491/A//G	nonsynonymous	0	2
HTR2A	chr13	47466707/G//A	nonsynonymous	0	2
	chr14	65417778/G//A	synonymous	0	2
ISLR2	chr15	74426447/C//T	nonsynonymous	0	2
SLX4	chr16	3632599/G//A	nonsynonymous	0	2
DOC2A	chr16	30017814/A//C	nonsynonymous	0	2
HYDIN	chr16	70995856/G//A	synonymous	0	2
MBTPS1	chr16	84129261/G//A	nonsynonymous	0	2
ABR	chr17	961996/G//A	nonsynonymous	0	2
MYO15A	chr17	18022871/C//T	nonsynonymous	0	2
NGFR	chr17	47590321/G//A	nonsynonymous	0	2
ITGB4	chr17	73733432/C//T	nonsynonymous	0	2
CBX8	chr17	77769058/G//A	synonymous	0	2
DENND1C	chr19	6477288/C//T	nonsynonymous	0	2
CST2	chr20	23807255/G//T	nonsynonymous	0	2

KRTAP27-1	chr21	31709720/G//A	synonymous	0	2
TMEM39B	chr1	32557556/G//A	nonsynonymous	0	2
WDR77	chr1	111986541/G//C	nonsynonymous	0	2
OR2M5	chr1	248309246/G//T	nonsynonymous	0	2
TRIB2	chr2	12880757/G//A	nonsynonymous	0	2
LRP2	chr2	170068568/A//T	nonsynonymous	0	2
TTN	chr2	179403751/G//A	nonsynonymous	0	2
HECW2	chr2	197185133/T//C	synonymous	0	2
MAP2	chr2	210557684/G//T	nonsynonymous	0	2
RUFY4	chr2	218940309/C//T	nonsynonymous	0	2
SLC4A3	chr2	220493871/C//T	nonsynonymous	0	2
KLHL18	chr3	47378159/G//A	nonsynonymous	0	2
TBC1D1	chr4	38016321/C//G	synonymous	0	2
TLR6	chr4	38829781/A//T	nonsynonymous	0	2
ENOPH1	chr4	83381215/A//AGACTTACT	nonsynonymous	0	2
NUDT9	chr4	88356254/C//T	nonsynonymous	0	2
CCSER1	chr4	91234082/G//A	nonsynonymous	0	2
UNC5C	chr4	96106239/C//T	nonsynonymous	0	2
ANK2	chr4	114163368/G//A	synonymous	0	2
PCDH18	chr4	138451498/C//T	nonsynonymous	0	2
PCDHA3	chr5	140182025/C//T	nonsynonymous	0	2
PCDHA5	chr5	140202029/T//C	synonymous	0	2
PCDHB11	chr5	140581573/C//T	synonymous	0	2
PCDHGB1	chr5	140729862/G//T	nonsynonymous	0	2
FAM71B	chr5	156589500/G//A	synonymous	0	2
HIST1H3C	chr6	26045947/A//G	synonymous	0	2
ZNF292	chr6	87970811/T//C	synonymous	0	2
CDC40	chr6	110530416/A//C	nonsynonymous	0	2
THBS2	chr6	169632238/G//A	nonsynonymous	0	2
DLL1	chr6	170598736/G//T	nonsynonymous	0	2
FKBP6	chr7	72744246/C//T	nonsynonymous	0	2
SEMA3D	chr7	84642152/G//A	nonsynonymous	0	2
ADAM22	chr7	87759746/C//T	nonsynonymous	0	2
KIAA1549	chr7	138601708/G//A	synonymous	0	2
SSPO	chr7	149522197/C//T	synonymous	0	2
DLGAP2	chr8	1626412/C//T	nonsynonymous	0	2
CSMD1	chr8	2944703/G//A	nonsynonymous	0	2
ADAM28	chr8	24184140/G//A	nonsynonymous	0	2
ADAM18	chr8	39442090/G//A	synonymous	0	2
PENK	chr8	57354129/C//T	nonsynonymous	0	2
WASHC5	chr8	126068993/C//T	nonsynonymous	0	2
PTPRD	chr9	8449762/G//T	nonsynonymous	0	2
PHF2	chr9	96439019/C//A	synonymous	1	1
POLR3A	chr10	79777416/C//T	nonsynonymous	0	2
LDB1	chr10	103869712/C//T	nonsynonymous	0	2
COL17A1	chr10	105816860/G//A	synonymous	0	2
OR4C16	chr11	55340116/GATCA//G	nonsynonymous	0	2
INTS5	chr11	62417296/G//A	nonsynonymous	0	2
PCNX3	chr11	65384943/G//A	synonymous	0	2
GRK2	chr11	67051322/C//T	synonymous	0	2
ST8SIA1	chr12	22354607/G//A	nonsynonymous	0	2
PRKAG1	chr12	49399286/C//T	nonsynonymous	0	2
TBX5	chr12	114841612/G//A	nonsynonymous	0	2
SLITRK5	chr13	88329003/G//A	nonsynonymous	0	2
CCDC168	chr13	103396902/C//A	nonsynonymous	0	2
ATP10A	chr15	25959230/C//T	synonymous	0	2
OTUD7A	chr15	31776456/G//A	nonsynonymous	0	2
C15orf65	chr15	55710600/T//C	synonymous	0	2
PTPN9	chr15	75798093/G//A	synonymous	0	2
AP3B2	chr15	83333604/G//A	nonsynonymous	0	2
CAPN15	chr16	601548/C//T	synonymous	0	2
RHBDL1	chr16	727886/G//A	nonsynonymous	0	2
CREBBP	chr16	3786125/T//C	nonsynonymous	0	2
ZNF423	chr16	49670758/C//T	nonsynonymous	0	2
NOD2	chr16	50746117/G//C	synonymous	0	2
ADGRG1	chr16	57687155/G//A	nonsynonymous	0	2
ALOX12	chr17	6909894/C//A	nonsynonymous	0	2
TP53	chr17	7577130/A//G	nonsynonymous	0	2
AARSD1	chr17	41103884/C//T	nonsynonymous	0	2
SPPL2C	chr17	43923925/C//T	synonymous	0	2

HOXB3	chr17	46629479/C//T	nonsynonymous	0	2
PSMA8	chr18	23713838/C//T	synonymous	0	2
SLC14A2	chr18	43246115/C//T	synonymous	0	2
KANK2	chr19	11303842/G//T	nonsynonymous	0	2
ZNF536	chr19	30935058/C//T	nonsynonymous	0	2
FAM187B	chr19	35719438/G//A	nonsynonymous	0	2
CD3EAP	chr19	45912282/G//A	synonymous	0	2
PRPF31	chr19	54625276/C//T	synonymous	0	2
KIR2DL1	chr19	55286780/C//A	nonsynonymous	0	2
SLC27A5	chr19	59021214/C//T	nonsynonymous	0	2
PRNP	chr20	4679944/G//A	synonymous	0	2
CST1	chr20	23731299/G//A	nonsynonymous	0	2
SMARCB1	chr22	24133988/T//C	nonsynonymous	0	2
SF3A1	chr22	30737727/G//A	nonsynonymous	0	2
SLC35E4	chr22	31042989/C//T	nonsynonymous	0	2
RFPL2	chr22	32586754/A//T	synonymous	0	2
MXRA5	chrX	3235606/C//T	nonsynonymous	0	2
NHS	chrX	17744831/G//T	nonsynonymous	0	2
ZNF138	chr7	64292280/A//G	synonymous	2	0
	chr1	6111702/C//T	synonymous	0	2
HRNR	chr1	152193605/C//A	nonsynonymous	0	2
PGLYRP3	chr1	153270589/G//A	nonsynonymous	0	2
CFAP45	chr1	159842861/C//T	nonsynonymous	0	2
LHX9	chr1	197896850/T//A	nonsynonymous	0	2
PSME4	chr2	54176365/T//G	nonsynonymous	0	2
COL6A3	chr2	238243486/G//A	synonymous	0	2
GRM7	chr3	7620446/C//T	nonsynonymous	0	2
SLC22A14	chr3	38348789/G//T	nonsynonymous	0	2
NLGN1	chr3	173996842/C//T	nonsynonymous	0	2
FBXW7	chr4	153247289/G//A	nonsynonymous	0	2
TLR3	chr4	187005807/T//C	nonsynonymous	0	2
SLC1A3	chr5	36679839/G//T	nonsynonymous	0	2
CARD6	chr5	40852540/A//G	nonsynonymous	0	2
APC	chr5	112175133/C//A	nonsynonymous	0	2
JADE2	chr5	133914336/G//A	nonsynonymous	0	2
PCDHB5	chr5	140515207/C//T	nonsynonymous	0	2
ARAP3	chr5	141051796/C//G	synonymous	0	2
PRELID1	chr5	176733145/A//C	nonsynonymous	0	2
MDN1	chr6	90457158/C//T	nonsynonymous	0	2
DNAH11	chr7	21920447/T//G	nonsynonymous	0	2
EVX1	chr7	27284697/C//T	nonsynonymous	0	2
MPDZ	chr9	13121844/A//T	nonsynonymous	0	2
PCSK5	chr9	78853887/G//A	nonsynonymous	0	2
OR1N1	chr9	125288841/G//A	synonymous	0	2
OR1B1	chr9	125391391/G//A	nonsynonymous	0	2
HPX	chr11	6452905/G//T	synonymous	0	2
OR5P2	chr11	7818171/C//T	nonsynonymous	0	2
MFRP	chr11	119213352/G//A	synonymous	0	2
CHD4	chr12	6705221/C//T	nonsynonymous	0	2
PTPRQ	chr12	80904201/T//A	nonsynonymous	0	2
EP400	chr12	132514266/C//T	nonsynonymous	0	2
PCK2	chr14	24569430/C//A	synonymous	0	2
	chr14	35515685/C//T	synonymous	0	2
FLRT2	chr14	86088234/C//T	nonsynonymous	0	2
TTC7B	chr14	91211166/G//A	synonymous	0	2
ATP10A	chr15	26026194/C//T	nonsynonymous	0	2
OCA2	chr15	28116339/C//T	synonymous	0	2
HAUS2	chr15	42851532/T//C	synonymous	0	2
VEZF1	chr17	56058146/G//A	nonsynonymous	0	2
SMAD4	chr18	48584560/C//T	nonsynonymous	0	2
HNRNPL	chr19	39336502/C//A	synonymous	0	2
PRX	chr19	40903079/G//A	nonsynonymous	0	2
KIR3DL3	chr19	55239218/G//A	nonsynonymous	0	2
SUN5	chr20	31571645/C//T	synonymous	0	2
KRTAP13-4	chr21	31802793/G//A	nonsynonymous	0	2
RRP1B	chr21	45092225/G//A	nonsynonymous	0	2
FAM47A	chrX	34148222/T//C	nonsynonymous	0	2
AJAP1	chr1	4772695/T//G	synonymous	0	2
GJB3	chr1	35250741/C//A	synonymous	0	2
MPL	chr1	43805146/G//A	nonsynonymous	0	2

PTPRF	chr1	44069536/G//A	nonsynonymous	0	2
USP24	chr1	55627881/G//A	synonymous	0	2
SPTA1	chr1	158592962/C//A	synonymous	0	2
C2orf71	chr2	29295432/C//G	nonsynonymous	0	2
C2orf71	chr2	29296972/G//A	synonymous	0	2
LIPT1	chr2	99779177/G//C	nonsynonymous	0	2
THSD7B	chr2	138208561/C//T	nonsynonymous	0	2
DCLK3	chr3	36763056/G//T	nonsynonymous	0	2
TRPC1	chr3	142522979/C//G	nonsynonymous	0	2
ZIC1	chr3	147128336/T//G	nonsynonymous	0	2
LIPH	chr3	185229397/T//C	nonsynonymous	0	2
ANK2	chr4	114275101/G//A	nonsynonymous	0	2
TTC23L	chr5	34864626/A//G	synonymous	0	2
PCDHA6	chr5	140208003/C//T	synonymous	0	2
PCDHB3	chr5	140481830/G//A	nonsynonymous	0	2
PCDHGA5	chr5	140744765/G//C	nonsynonymous	0	2
MAK	chr6	10796300/A//T	synonymous	0	2
TEAD3	chr6	35445093/A//G	nonsynonymous	0	2
ME1	chr6	84117563/G//A	nonsynonymous	0	2
SLC35D3	chr6	137245018/C//T	synonymous	0	2
ADGB	chr6	147067231/G//T	nonsynonymous	0	2
AEBP1	chr7	44153782/AGAG//A	nonsynonymous	0	2
ADCY1	chr7	45717589/G//A	nonsynonymous	0	2
CNTNAP2	chr7	147259256/T//G	nonsynonymous	0	2
B4GALT1	chr9	33113514/G//A	synonymous	0	2
SPATA31D1	chr9	84606779/C//G	nonsynonymous	0	2
COL27A1	chr9	117068826/G//A	synonymous	0	2
BRINP1	chr9	121976221/C//T	nonsynonymous	0	2
PRRC2B	chr9	134312072/C//T	nonsynonymous	0	2
STKLD1	chr9	136268101/C//T	synonymous	0	2
GJD4	chr10	35897414/T//C	nonsynonymous	0	2
SLC35C1	chr11	45832809/G//A	nonsynonymous	0	2
MPEG1	chr11	58980176/G//A	nonsynonymous	0	2
FAT3	chr11	92531907/C//G	nonsynonymous	0	2
NCOR2	chr12	124829261/C//A	nonsynonymous	0	2
TMEM132B	chr12	125900226/A//T	synonymous	0	2
FZD10	chr12	130648880/G//A	nonsynonymous	0	2
CHFR	chr12	133423650/G//A	nonsynonymous	0	2
COG6	chr13	40235011/G//A	nonsynonymous	0	2
GPR18	chr13	99907960/G//A	nonsynonymous	0	2
ATP11A	chr13	113473668/C//T	synonymous	0	2
TMEM255B	chr13	114503858/A//T	nonsynonymous	0	2
NYNRIN	chr14	24886019/C//T	synonymous	0	2
TMEM63C	chr14	77685925/G//A	synonymous	0	2
NRXN3	chr14	80130234/C//T	synonymous	1	1
TSHR	chr14	81610414/C//T	nonsynonymous	0	2
BEGAIN	chr14	101004410/C//T	nonsynonymous	0	2
AHNAK2	chr14	105408233/C//T	nonsynonymous	0	2
ITPKA	chr15	41794213/G//A	synonymous	0	2
JMJD7-PLA2G4B	chr15	42132384/C//T	nonsynonymous	0	2
TLN2	chr15	62985084/G//A	nonsynonymous	0	2
SALL1	chr16	51174874/G//A	nonsynonymous	0	2
PLA2G15	chr16	68289802/G//A	synonymous	0	2
SPIRE2	chr16	89936544/G//A	nonsynonymous	0	2
SLC16A11	chr17	6945853/G//A	synonymous	0	2
CDH2	chr18	25573526/C//T	nonsynonymous	0	2
ONECUT3	chr19	1754717/T//A	synonymous	0	2
CLEC4M	chr19	7831683/C//T	nonsynonymous	0	2
MUC16	chr19	9069343/G//T	nonsynonymous	0	2
TMEM221	chr19	17547373/C//T	nonsynonymous	0	2
HCK	chr20	30689254/G//A	nonsynonymous	0	2
SYCP2	chr20	58449037/A//C	synonymous	0	2
DSCAM	chr21	41560972/C//A	synonymous	0	2
RFPL2	chr22	32590383/G//C	nonsynonymous	0	2
CELSR1	chr22	46777907/C//T	synonymous	0	2
PLCXD1	chrX	215903/G//A	synonymous	0	2
FAM120C	chrX	54160429/C//T	nonsynonymous	0	2
CXorf40B	chrX	149101841/C//T	synonymous	0	2
KRTAP5-1	chr11	1606177/G//A	synonymous	1	1
EXOSC10	chr1	11148191/G//A	nonsynonymous	0	2

PDIK1L	chr1	26448961/C//G	nonsynonymous	0	2
RAD54L	chr1	46725713/G//A	nonsynonymous	0	2
BEND5	chr1	49202110/G//A	synonymous	0	2
CYR61	chr1	86047865/C//T	nonsynonymous	0	2
NTRK1	chr1	156845316/G//A	synonymous	0	2
PGBD5	chr1	230468761/C//T	nonsynonymous	0	2
OR2C3	chr1	247695442/G//A	synonymous	0	2
OR11L1	chr1	248004552/C//T	nonsynonymous	0	2
TRAPPC12	chr2	3391892/C//T	synonymous	0	2
SLC5A7	chr2	108622549/C//A	synonymous	0	2
NEB	chr2	152541388/G//A	synonymous	0	2
TTN	chr2	179465997/G//A	nonsynonymous	0	2
UNC80	chr2	210737560/C//T	nonsynonymous	0	2
COL6A3	chr2	238249315/C//T	synonymous	0	2
CADPS	chr3	62739268/G//A	nonsynonymous	0	2
VGLL3	chr3	87039905/C//T	synonymous	0	2
ABI3BP	chr3	100567677/C//T	nonsynonymous	0	2
KALRN	chr3	124281775/C//T	nonsynonymous	0	2
HEG1	chr3	124731591/G//A	synonymous	0	2
RNF168	chr3	196198821/G//A	nonsynonymous	0	2
EPHA5	chr4	66201723/C//A	nonsynonymous	0	2
FBXW7	chr4	153249384/C//T	nonsynonymous	0	2
FBXW7	chr4	153249504/C//A	nonsynonymous	0	2
WDR17	chr4	177056255/C//T	synonymous	0	2
IRX4	chr5	1882101/C//T	nonsynonymous	0	2
APC	chr5	112173531/C//A	nonsynonymous	0	2
PCDHA4	chr5	140188931/C//T	nonsynonymous	0	2
PCDHB12	chr5	140590002/C//T	nonsynonymous	0	2
PCDHGA1	chr5	140712483/C//T	synonymous	0	2
PCDHGA12	chr5	140810848/G//T	synonymous	0	2
TCOF1	chr5	149771158/G//A	nonsynonymous	0	2
FAM196B	chr5	169309942/C//T	nonsynonymous	0	2
GFPT2	chr5	179755287/C//T	nonsynonymous	0	2
TFAP2A	chr6	10410285/G//A	nonsynonymous	0	2
TREM1	chr6	41250249/C//T	nonsynonymous	0	2
RIMS1	chr6	73110298/G//T	nonsynonymous	0	2
COL12A1	chr6	75853014/C//T	nonsynonymous	0	2
GLI3	chr7	42004661/G//A	nonsynonymous	0	2
TRIM56	chr7	100732070/T//C	nonsynonymous	0	2
SND1	chr7	127729551/G//A	nonsynonymous	0	2
HTR5A	chr7	154876000/G//A	nonsynonymous	0	2
DLGAP2	chr8	1626444/G//A	nonsynonymous	0	2
OPLAH	chr8	145114792/G//A	synonymous	0	2
ACO1	chr9	32420939/C//T	nonsynonymous	0	2
USP20	chr9	132631613/G//A	nonsynonymous	0	2
CUBN	chr10	16949566/G//A	nonsynonymous	0	2
C10orf10	chr10	45473072/T//A	nonsynonymous	0	2
C10orf71	chr10	50533357/C//T	nonsynonymous	0	2
PRF1	chr10	72360448/C//T	nonsynonymous	0	2
PDZD8	chr10	119044307/G//C	nonsynonymous	0	2
CYP2E1	chr10	135345657/G//A	nonsynonymous	0	2
HRAS	chr11	533907/G//A	nonsynonymous	0	2
DNHD1	chr11	6580224/C//T	nonsynonymous	0	2
LRTOMT	chr11	71819857/G//A	synonymous	0	2
FAT3	chr11	92534532/G//T	nonsynonymous	0	2
ARHGAP32	chr11	128842397/A//G	nonsynonymous	0	2
C12orf40	chr12	40114849/C//T	synonymous	0	2
DBX2	chr12	45444763/C//T	synonymous	0	2
KMT2D	chr12	49440049/C//T	nonsynonymous	0	2
CERS5	chr12	50524462/G//T	nonsynonymous	0	2
MFSD5	chr12	53647879/G//T	synonymous	0	2
TBX3	chr12	115114117/C//T	nonsynonymous	0	2
MMP17	chr12	132325304/C//T	synonymous	0	2
SACS	chr13	23910620/C//T	synonymous	0	2
CKAP2	chr13	53049067/G//A	nonsynonymous	0	2
NALCN	chr13	101881824/G//A	nonsynonymous	0	2
OCA2	chr15	28116374/C//T	nonsynonymous	0	2
	chr15	50578233/C//T	synonymous	0	2
USP50	chr15	50835990/G//A	synonymous	0	2
IFT140	chr16	1637986/C//T	nonsynonymous	0	2

DNAH9	chr17	11806178/G//A	nonsynonymous	0	2
ZNF207	chr17	30694972/C//T	nonsynonymous	0	2
GPR179	chr17	36491542/G//A	synonymous	0	2
SOST	chr17	41832768/G//A	nonsynonymous	0	2
ANKRD24	chr19	4216762/G//A	synonymous	0	2
LONP1	chr19	5705931/G//C	nonsynonymous	0	2
DNM2	chr19	10930670/G//A	synonymous	0	2
CC2D1A	chr19	14029610/C//T	nonsynonymous	0	2
OR10H2	chr19	15839090/G//A	synonymous	0	2
USHBP1	chr19	17373566/G//T	nonsynonymous	0	2
ZNF91	chr19	23544842/C//A	nonsynonymous	0	2
PRODH2	chr19	36303732/C//T	nonsynonymous	0	2
SPTBN4	chr19	41012284/C//A	nonsynonymous	0	2
CEACAM7	chr19	42191081/C//T	nonsynonymous	0	2
GYS1	chr19	49472811/G//A	nonsynonymous	0	2
RCN3	chr19	50031913/C//T	nonsynonymous	0	2
ZNF816	chr19	53454186/C//T	nonsynonymous	0	2
LILRB5	chr19	54756839/G//A	nonsynonymous	0	2
ATRN	chr20	3619532/G//A	nonsynonymous	0	2
PAX1	chr20	21695297/C//T	synonymous	0	2
KCNK15	chr20	43374711/T//C	nonsynonymous	0	2
	chr20	60074496/C//T	synonymous	0	2
UCKL1	chr20	62571772/A//T	nonsynonymous	0	2
ELFN2	chr22	37769636/C//T	nonsynonymous	0	2
NPTXR	chr22	39224324/A//G	nonsynonymous	0	2
HCCS	chrX	11130237/G//A	synonymous	0	2
MAP7D2	chrX	20082881/G//A	nonsynonymous	0	2
RBM10	chrX	47045987/G//A	nonsynonymous	0	2
MAGED1	chrX	51637376/C//T	synonymous	0	2
FGD1	chrX	54496521/G//A	synonymous	0	2
PFKFB1	chrX	54978378/C//T	nonsynonymous	0	2
AMER1	chrX	63411567/C//A	nonsynonymous	0	2
NHSL2	chrX	71357066/C//T	nonsynonymous	0	2
PCDH11X	chrX	91873660/C//T	synonymous	0	2
HTR2C	chrX	114141278/C//T	nonsynonymous	0	2
XPNPEP2	chrX	128880287/T//G	nonsynonymous	0	2
FLNA	chrX	153587695/C//T	nonsynonymous	0	2
PIK3CA	chr3	178921553/T//A	nonsynonymous	2	0
RS1	chrX	18674876/A//C	nonsynonymous	1	1
NPHP4	chr1	5947423/A//G	nonsynonymous	0	2
H6PD	chr1	9324542/G//T	nonsynonymous	0	2
SF3B4	chr1	149897784/G//C	nonsynonymous	0	2
ATP8B2	chr1	154313419/G//T	nonsynonymous	0	2
RGS8	chr1	182635145/G//A	nonsynonymous	0	2
HMCN1	chr1	186056576/G//A	synonymous	0	2
REN	chr1	204125866/C//T	nonsynonymous	0	2
C4BPA	chr1	207307809/G//A	nonsynonymous	0	2
USH2A	chr1	216496942/G//A	nonsynonymous	0	2
SPATA17-AS1	chr1	217955487/A//T	synonymous	0	2
RYR2	chr1	237713944/C//T	nonsynonymous	0	2
CNST	chr1	246754806/C//A	synonymous	0	2
IAH1	chr2	9616143/G//A	nonsynonymous	0	2
HAAO	chr2	42995017/C//T	nonsynonymous	0	2
DUSP11	chr2	74007031/T//G	nonsynonymous	0	2
ZAP70	chr2	98354366/C//T	synonymous	0	2
GLI2	chr2	121746380/G//A	nonsynonymous	0	2
CACNB4	chr2	152727124/G//A	nonsynonymous	0	2
FASTKD1	chr2	170394599/ATT//A	nonsynonymous	0	2
TTN	chr2	179422105/G//A	nonsynonymous	0	2
TTN	chr2	179574287/A//G	synonymous	0	2
TTN	chr2	179640851/C//T	nonsynonymous	0	2
CFAP65	chr2	219884406/C//T	nonsynonymous	0	2
ZNF660	chr3	44636677/C//A	nonsynonymous	0	2
NBEAL2	chr3	47047263/A//G	nonsynonymous	0	2
CEP70	chr3	138291733/G//C	nonsynonymous	0	2
NLGN1	chr3	173993181/T//A	synonymous	0	2
YEATS2	chr3	183493795/G//C	nonsynonymous	0	2
ZDHHC19	chr3	195936284/C//T	nonsynonymous	0	2
ZNF721	chr4	437293/A//G	synonymous	1	1
PDE6B	chr4	619760/G//A	synonymous	0	2

LOC650293	chr4	8951552/A//T	nonsynonymous	0	2
BOD1L1	chr4	13601693/G//A	synonymous	0	2
DSPP	chr4	88533913/G//A	nonsynonymous	0	2
KIAA1109	chr4	123260422/C//T	nonsynonymous	0	2
SPOCK3	chr4	168155253/C//T	synonymous	0	2
SEMA5A	chr5	9136646/C//T	synonymous	0	2
NLN	chr5	65054607/C//G	nonsynonymous	0	2
APC	chr5	112175746/T//TG	nonsynonymous	0	2
ANKHD1	chr5	139876644/C//T	nonsynonymous	0	2
PCDHB6	chr5	140531787/G//A	nonsynonymous	0	2
CSF1R	chr5	149460527/G//A	nonsynonymous	0	2
FAM50B	chr6	3850900/C//T	synonymous	0	2
BTN3A2	chr6	26370611/G//A	synonymous	0	2
OR2B2	chr6	27879428/C//T	nonsynonymous	0	2
DNAH8	chr6	38919196/T//G	synonymous	0	2
KCNK16	chr6	39290200/C//T	synonymous	0	2
RNGTT	chr6	89673114/CTTG//C	nonsynonymous	0	2
ARFGEF3	chr6	138584412/G//A	nonsynonymous	0	2
INTS1	chr7	1526999/C//T	nonsynonymous	0	2
DNAH11	chr7	21583137/G//A	nonsynonymous	0	2
CAMK2B	chr7	44260447/T//TGTTGAC	nonsynonymous	0	2
ZNF804B	chr7	88964002/G//C	nonsynonymous	0	2
MUC12	chr7	100649810/C//T	synonymous	0	2
SSPO	chr7	149514738/G//T	synonymous	0	2
NRG1	chr8	32621346/C//T	nonsynonymous	0	2
IDO2	chr8	39840262/C//T	nonsynonymous	0	2
PENK	chr8	57354366/G//A	nonsynonymous	0	2
PI15	chr8	75737595/C//A	nonsynonymous	0	2
TMEM261	chr9	7798459/G//T	synonymous	0	2
GABBR2	chr9	101216341/C//T	synonymous	0	2
COL27A1	chr9	116930092/C//T	nonsynonymous	0	2
SARDH	chr9	136531892/C//T	nonsynonymous	0	2
C10orf71	chr10	50532116/G//A	nonsynonymous	0	2
GFRA1	chr10	117885010/G//A	synonymous	0	2
OR4A47	chr11	48510559/C//A	nonsynonymous	0	2
BIRC3	chr11	102195845/G//A	nonsynonymous	0	2
DYNC2H1	chr11	103026085/T//A	nonsynonymous	0	2
ATM	chr11	108165673/C//A	nonsynonymous	0	2
BCL9L	chr11	118772231/C//A	nonsynonymous	0	2
GRAMD1B	chr11	123479515/G//A	synonymous	0	2
B3GAT1	chr11	134253981/C//T	nonsynonymous	0	2
PZP	chr12	9353540/T//C	synonymous	0	2
GRIN2B	chr12	13717003/G//A	nonsynonymous	0	2
C12orf40	chr12	40037672/T//A	nonsynonymous	0	2
UTP20	chr12	101711371/G//C	nonsynonymous	0	2
	chr13	78493652/G//A	synonymous	0	2
SPTLC2	chr14	78083012/T//A	synonymous	0	2
ATP10A	chr15	25981229/G//A	synonymous	0	2
TMOD2	chr15	52098677/G//A	nonsynonymous	0	2
RASGRF1	chr15	79254515/G//A	nonsynonymous	0	2
AGBL1	chr15	87097677/C//T	nonsynonymous	0	2
SV2B	chr15	91769480/C//T	synonymous	0	2
ANKS3	chr16	4755110/G//C	nonsynonymous	0	2
CCDC189	chr16	30770500/C//T	synonymous	0	2
SNX20	chr16	50707972/A//C	nonsynonymous	0	2
AARS	chr16	70298892/G//A	synonymous	0	2
APRT	chr16	88876125/GAGA//G	nonsynonymous	0	2
MYO1C	chr17	1386947/C//A	nonsynonymous	0	2
PLXDC1	chr17	37226124/C//T	synonymous	0	2
UBE2O	chr17	74395854/G//A	nonsynonymous	0	2
CXXC1	chr18	47810462/G//A	synonymous	0	2
LRRC8E	chr19	7965443/G//A	nonsynonymous	0	2
ZNF560	chr19	9577924/C//T	nonsynonymous	0	2
ANO8	chr19	17434386/C//T	synonymous	0	2
ZNF493	chr19	21607687/A//T	nonsynonymous	0	2
KLC3	chr19	45848946/G//A	synonymous	0	2
GRIN2D	chr19	48908228/C//T	nonsynonymous	0	2
TMC4	chr19	54672341/G//T	nonsynonymous	0	2
ZNF135	chr19	58578975/A//G	nonsynonymous	0	2
ZNF837	chr19	58880716/C//A	synonymous	0	2

HSPA12B	chr20	3728978/C//T	nonsynonymous	0	2
ISM1	chr20	13260381/C//T	nonsynonymous	0	2
CSTL1	chr20	23421075/C//T	synonymous	0	2
DSCAM	chr21	41684166/C//T	nonsynonymous	0	2
MX2	chr21	42762532/C//T	nonsynonymous	0	2
SEZ6L	chr22	26688639/C//T	nonsynonymous	0	2
L1CAM	chrX	153128277/G//A	synonymous	0	2
HRH2	chr5	175110951/G//A	nonsynonymous	1	1
IL32	chr16	3119304/A//G	nonsynonymous	1	1
C1orf159	chr1	1025766/C//T	nonsynonymous	0	2
PRMT6	chr1	107599525/A//T	nonsynonymous	0	2
LCE3D	chr1	152552175/C//T	nonsynonymous	0	2
CD1D	chr1	158151984/A//T	nonsynonymous	0	2
IGSF8	chr1	160063885/G//A	synonymous	0	2
SELP	chr1	169582270/C//T	synonymous	0	2
RCOR3	chr1	211486934/C//T	nonsynonymous	0	2
CCDC85A	chr2	56420199/C//A	synonymous	0	2
CNTNAP5	chr2	125671818/G//T	nonsynonymous	0	2
GALNT13	chr2	155252622/C//T	nonsynonymous	0	2
TTN	chr2	179641726/C//A	nonsynonymous	0	2
KIF9	chr3	47282434/C//T	nonsynonymous	0	2
CHDH	chr3	53857672/A//C	nonsynonymous	0	2
CPOX	chr3	98309479/C//G	nonsynonymous	0	2
SPATA16	chr3	172737324/C//T	nonsynonymous	0	2
FAT4	chr4	126411117/G//A	synonymous	0	2
ZNF330	chr4	142143571/C//T	nonsynonymous	0	2
APC	chr5	112174956/C//A	nonsynonymous	0	2
PCDHA8	chr5	140222577/C//T	synonymous	0	2
FBXO38	chr5	147790333/G//A	synonymous	0	2
N4BP3	chr5	177547562/G//A	synonymous	0	2
ADAMTS2	chr5	178555111/C//T	synonymous	0	2
PPP1R18	chr6	30652228/C//T	nonsynonymous	0	2
TNXB	chr6	32020751/G//A	synonymous	0	2
ADGRB3	chr6	69703704/T//C	synonymous	0	2
DACT2	chr6	168708142/C//T	synonymous	0	2
CCDC126	chr7	23651027/C//A	nonsynonymous	0	2
CCM2	chr7	45113101/CAG//C	nonsynonymous	0	2
ABCA13	chr7	48391890/A//G	synonymous	0	2
WBSCR17	chr7	70597600/G//A	synonymous	0	2
MUC17	chr7	100682890/T//A	synonymous	0	2
ERICH5	chr8	99102250/C//T	synonymous	0	2
PTPRD	chr9	8499740/C//A	nonsynonymous	0	2
LMX1B	chr9	129458743/C//T	synonymous	0	2
MCM10	chr10	13212950/C//T	synonymous	0	2
ARMC3	chr10	23248494/C//T	synonymous	0	2
ABCC2	chr10	101591555/T//G	nonsynonymous	0	2
TCF7L2	chr10	114910883/G//A	nonsynonymous	0	2
GFRA1	chr10	117885029/G//A	nonsynonymous	0	2
MRVI1	chr11	10655556/G//A	synonymous	0	2
CD6	chr11	60780928/A//G	nonsynonymous	0	2
TKFC	chr11	61105457/C//T	synonymous	0	2
SLC22A11	chr11	64335131/C//T	synonymous	0	2
EHD1	chr11	64627508/C//T	nonsynonymous	0	2
CNTN5	chr11	99827597/C//T	nonsynonymous	0	2
DDI1	chr11	103907572/G//A	nonsynonymous	0	2
MPZL3	chr11	118106312/C//T	synonymous	0	2
ADAMTS8	chr11	130275813/G//A	synonymous	0	2
KDM5A	chr12	420115/C//T	nonsynonymous	0	2
C1R	chr12	7188341/C//A	nonsynonymous	0	2
DIP2B	chr12	51138473/G//A	nonsynonymous	0	2
OR6C6	chr12	55688654/G//A	synonymous	0	2
IRAK3	chr12	66583108/C//T	nonsynonymous	0	2
APAF1	chr12	99059446/G//A	synonymous	0	2
MAB21L1	chr13	36049381/G//A	nonsynonymous	0	2
SERPINE3	chr13	51915295/G//A	nonsynonymous	0	2
SLC7A8	chr14	23598870/G//A	nonsynonymous	0	2
SETD3	chr14	99879379/G//A	nonsynonymous	0	2
AHNAK2	chr14	105413319/G//A	synonymous	0	2
UBE3A	chr15	25620658/G//A	synonymous	0	2
NEO1	chr15	73590750/C//T	synonymous	0	2

PRR35	chr16	613540/G//A	synonymous	0	2
ZNF646	chr16	31087934/C//G	nonsynonymous	0	2
TRIM72	chr16	31230620/G//A	nonsynonymous	0	2
	chr16	67997963/T//C	synonymous	0	2
CLEC3A	chr16	78064664/G//T	nonsynonymous	0	2
TMEM132E	chr17	32964377/G//A	nonsynonymous	0	2
SLFN5	chr17	33591819/A//G	nonsynonymous	0	2
KRTAP16-1	chr17	39464337/G//A	nonsynonymous	0	2
KRT37	chr17	39580491/G//A	synonymous	0	2
G6PC3	chr17	42153393/G//A	synonymous	0	2
SLC39A11	chr17	71084892/G//T	synonymous	0	2
RNF213	chr17	78319794/C//T	synonymous	0	2
COLEC12	chr18	335067/G//A	synonymous	0	2
KDM4B	chr19	5040028/C//A	synonymous	0	2
TSSK6	chr19	19625690/GCACCTCGGGTGA//G	nonsynonymous	0	2
MYH14	chr19	50755899/G//A	nonsynonymous	0	2
ERVV-2	chr19	53554149/G//A	synonymous	0	2
SIGLEC1	chr20	3678674/G//A	synonymous	0	2
BTBD3	chr20	11900474/G//A	nonsynonymous	0	2
KCNS1	chr20	43727218/C//T	synonymous	0	2
PCDH11X	chrX	91133712/G//A	nonsynonymous	0	2
IGSF1	chrX	130409576/G//A	synonymous	0	2
CELSR2	chr1	109792751/T//C	nonsynonymous	1	1
SMAD4	chr18	48591919/G//A	nonsynonymous	0	2
OR13C2	chr9	107367132/G//A	synonymous	2	0
MADD	chr11	47345340/G//A	nonsynonymous	1	1
AGRN	chr1	977440/G//A	nonsynonymous	0	2
ARID1A	chr1	27097634/G//T	nonsynonymous	0	2
PTCH2	chr1	45288139/G//T	nonsynonymous	0	2
FLG	chr1	152282322/TC//T	nonsynonymous	0	2
FLG2	chr1	152326460/G//A	nonsynonymous	0	2
	chr1	197704663/A//C	synonymous	0	2
SUSD4	chr1	223396838/G//T	synonymous	0	2
NRXN1	chr2	50464010/C//T	nonsynonymous	0	2
REV1	chr2	100017692/C//G	synonymous	0	2
TTN	chr2	179592036/G//A	nonsynonymous	0	2
COL6A3	chr2	238280443/G//A	nonsynonymous	0	2
KIF1A	chr2	241710464/C//T	nonsynonymous	0	2
GHRL	chr3	10328487/G//A	nonsynonymous	0	2
CCDC174	chr3	14708373/C//T	nonsynonymous	0	2
RAD54L2	chr3	51667640/G//A	synonymous	0	2
ROBO1	chr3	78710387/G//C	nonsynonymous	0	2
VGLL3	chr3	87027847/G//A	nonsynonymous	0	2
COL6A6	chr3	130293185/C//T	synonymous	0	2
LIN54	chr4	83900144/G//A	nonsynonymous	0	2
GYPA	chr4	145040921/C//T	synonymous	0	2
RASGRF2	chr5	80388681/G//A	synonymous	0	2
SLCO6A1	chr5	101834473/G//A	nonsynonymous	0	2
HIST1H1E	chr6	26157169/C//T	nonsynonymous	0	2
GRIK2	chr6	102074264/A//C	nonsynonymous	0	2
PARK2	chr6	162683748/C//T	nonsynonymous	0	2
QKI	chr6	163991773/C//A	synonymous	0	2
DACT2	chr6	168710987/GTCCCCCATGC//G	nonsynonymous	0	2
MRM2	chr7	2281804/T//C	nonsynonymous	0	2
OSBPL3	chr7	24901368/G//T	synonymous	0	2
MUC17	chr7	100683527/G//A	nonsynonymous	0	2
SSPO	chr7	149500037/G//T	nonsynonymous	0	2
CSMD1	chr8	3072032/C//A	synonymous	0	2
AGPAT5	chr8	6582447/TC//T	nonsynonymous	0	2
MMP16	chr8	89198810/C//T	nonsynonymous	0	2
FAM78A	chr9	134151254/C//T	nonsynonymous	0	2
MYO3A	chr10	26491987/C//T	nonsynonymous	0	2
BICC1	chr10	60380613/A//C	nonsynonymous	0	2
PALD1	chr10	72285893/G//A	nonsynonymous	0	2
ASCC1	chr10	73862155/T//C	nonsynonymous	0	2
NEURL1	chr10	105349996/C//T	nonsynonymous	0	2
TRIM6-TRIM34	chr11	5664587/G//A	nonsynonymous	0	2
SLC22A8	chr11	62763294/T//C	synonymous	0	2
	chr11	84028117/C//T	synonymous	0	2
FAT3	chr11	92533549/G//A	nonsynonymous	0	2

OR8D4	chr11	123777175/C//A	nonsynonymous	0	2
DTX3	chr12	58001242/G//A	nonsynonymous	0	2
NAV3	chr12	78444704/C//T	nonsynonymous	0	2
NOS1	chr12	117749357/C//T	nonsynonymous	0	2
MED4	chr13	48654102/C//T	nonsynonymous	0	2
	chr14	58894871/G//C	synonymous	0	2
NTRK3	chr15	88476388/G//A	nonsynonymous	0	2
BFAR	chr16	14749020/G//A	nonsynonymous	0	2
PDILT	chr16	20376785/G//A	synonymous	0	2
ZNF423	chr16	49670812/G//A	nonsynonymous	0	2
ZFHX3	chr16	72991359/C//T	nonsynonymous	0	2
RTN4RL1	chr17	1840032/G//A	nonsynonymous	0	2
DNAH9	chr17	11573085/G//T	nonsynonymous	0	2
DNAH9	chr17	11573100/C//T	synonymous	0	2
ARHGAP44	chr17	12860007/A//G	nonsynonymous	0	2
ITGA2B	chr17	42460964/G//A	synonymous	0	2
ABCC3	chr17	48741216/G//A	synonymous	0	2
TSEN54	chr17	73518374/C//T	synonymous	0	2
TMEM200C	chr18	5891951/C//T	nonsynonymous	0	2
PIEZO2	chr18	10773615/C//T	synonymous	0	2
TMEM241	chr18	21017910/G//A	synonymous	0	2
SF3A2	chr19	2243494/G//A	nonsynonymous	0	2
FBN3	chr19	8130981/C//T	nonsynonymous	0	2
MUC16	chr19	9074728/C//T	nonsynonymous	0	2
CACNA1A	chr19	13428042/C//T	nonsynonymous	0	2
SLC7A9	chr19	33355156/G//A	synonymous	0	2
LGI4	chr19	35616141/G//A	nonsynonymous	0	2
	chr19	37488025/C//T	synonymous	0	2
CYP2S1	chr19	41699173/G//C	nonsynonymous	0	2
ZNF114	chr19	48790083/C//G	nonsynonymous	0	2
NUP62	chr19	50412516/C//A	synonymous	0	2
CD93	chr20	23066707/C//T	synonymous	0	2
HELZ2	chr20	62197136/C//T	synonymous	0	2
GRIK1-AS2	chr21	30968910/A//AC	synonymous	0	2
MICAL3	chr22	18324730/T//A	nonsynonymous	0	2
ASCC2	chr22	30221114/G//T	nonsynonymous	0	2
ASCC2	chr22	30221116/A//T	nonsynonymous	0	2
TTC38	chr22	46669938/G//A	nonsynonymous	0	2
PASD1	chrX	150828232/C//T	synonymous	0	2
FLNA	chrX	153588101/C//A	nonsynonymous	0	2
CAP1	chr1	40525058/G//T	nonsynonymous	0	2
CYR61	chr1	86048422/G//T	nonsynonymous	0	2
TDRKH	chr1	151748583/G//T	synonymous	0	2
OR6N1	chr1	158736265/C//G	nonsynonymous	0	2
KIFAP3	chr1	169993698/C//T	nonsynonymous	0	2
MAP10	chr1	232942380/G//T	nonsynonymous	0	2
RYR2	chr1	237617723/C//T	nonsynonymous	0	2
RYR2	chr1	237777877/C//G	nonsynonymous	0	2
DNAJC27	chr2	25179998/C//A	nonsynonymous	0	2
EMILIN1	chr2	27306296/G//A	synonymous	0	2
LTBP1	chr2	33359988/C//A	nonsynonymous	0	2
EGR4	chr2	73519207/G//T	nonsynonymous	0	2
MFSD9	chr2	103348821/G//T	synonymous	0	2
NCKAP5	chr2	133540350/G//A	nonsynonymous	0	2
FSIP2	chr2	186657368/G//A	synonymous	0	2
FSIP2	chr2	186671359/G//T	nonsynonymous	0	2
SLC4A3	chr2	220501562/T//G	nonsynonymous	0	2
KIF1A	chr2	241726704/G//A	synonymous	0	2
ZBTB38	chr3	141162485/G//T	nonsynonymous	0	2
PIK3CA	chr3	178947852/C//A	nonsynonymous	0	2
CHRD	chr3	184104513/C//T	synonymous	0	2
CNGA1	chr4	47939403/C//T	nonsynonymous	0	2
UGT2B11	chr4	70070229/C//A	nonsynonymous	0	2
ANKRD17	chr4	74008042/G//T	nonsynonymous	0	2
COQ2	chr4	84205700/G//C	nonsynonymous	0	2
C4orf17	chr4	100434315/G//A	nonsynonymous	0	2
TKTL2	chr4	164394012/G//A	nonsynonymous	0	2
APC	chr5	112175921/G//T	nonsynonymous	0	2
ISOC1	chr5	128442742/T//A	nonsynonymous	0	2
CDSN	chr6	31085263/C//T	synonymous	0	2

DDAH2	chr6	31695346/C//A	nonsynonymous	0	2
TNXB	chr6	32036591/G//A	synonymous	0	2
AGPAT1	chr6	32138359/G//T	nonsynonymous	0	2
ITPR3	chr6	33652576/C//T	synonymous	0	2
TDRD6	chr6	46658018/C//A	nonsynonymous	0	2
ADGRF5	chr6	46824491/C//T	synonymous	0	2
HMGCLL1	chr6	55300485/A//G	nonsynonymous	0	2
RIMS1	chr6	73110346/C//A	nonsynonymous	0	2
CD109	chr6	74405963/G//A	nonsynonymous	0	2
FILIP1	chr6	76023328/C//A	nonsynonymous	0	2
ADGRG6	chr6	142736162/C//A	nonsynonymous	0	2
CCM2	chr7	45077915/G//T	nonsynonymous	0	2
IFT22	chr7	100959808/C//A	nonsynonymous	0	2
LSM8	chr7	117825752/A//T	synonymous	0	2
CHRM2	chr7	136700653/G//T	synonymous	0	2
CRYGN	chr7	151133319/G//T	nonsynonymous	0	2
CSMD1	chr8	3076924/C//A	nonsynonymous	0	2
DPYSL2	chr8	26501512/G//T	nonsynonymous	0	2
PRKDC	chr8	48697850/A//C	nonsynonymous	0	2
C8orf76	chr8	124253558/C//A	nonsynonymous	0	2
PAPPA	chr9	118974045/T//A	nonsynonymous	0	2
OR1K1	chr9	125562424/C//A	nonsynonymous	0	2
NOC3L	chr10	96114824/G//T	nonsynonymous	0	2
SF3B2	chr11	65831130/G//T	nonsynonymous	0	2
FOLR3	chr11	71847014/C//A	nonsynonymous	0	2
KCNA1	chr12	5021845/A//G	nonsynonymous	0	2
KCNA5	chr12	5153556/G//A	synonymous	0	2
A2M	chr12	9232243/A//G	nonsynonymous	0	2
DERA	chr12	16185475/G//T	nonsynonymous	0	2
KRAS	chr12	25380275/T//G	nonsynonymous	0	2
KRT76	chr12	53171040/G//T	synonymous	0	2
MYBPC1	chr12	102061659/C//A	nonsynonymous	0	2
ATP8A2	chr13	26273417/T//C	nonsynonymous	0	2
RB1	chr13	48951059/C//A	nonsynonymous	0	2
ITGBL1	chr13	102345012/G//T	nonsynonymous	0	2
OR4K1	chr14	20404482/C//T	synonymous	0	2
MTHFD1	chr14	64896895/C//T	synonymous	0	2
DIO2	chr14	80669111/G//T	nonsynonymous	0	2
UNC79	chr14	94004455/G//T	nonsynonymous	0	2
DICER1	chr14	95598887/C//A	nonsynonymous	0	2
AHNAK2	chr14	105416060/C//A	nonsynonymous	0	2
BUB1B-PAK6	chr15	40568139/G//T	synonymous	0	2
MAP1A	chr15	43815956/G//A	nonsynonymous	0	2
WDR76	chr15	44143396/G//T	nonsynonymous	0	2
SPG11	chr15	44888379/G//A	nonsynonymous	0	2
SLC27A2	chr15	50521189/G//T	nonsynonymous	0	2
NTRK3	chr15	88669502/C//A	nonsynonymous	0	2
OR4F15	chr15	102359127/G//T	synonymous	0	2
PIGQ	chr16	630938/G//A	synonymous	0	2
ATP6V0C	chr16	2569368/G//A	nonsynonymous	0	2
AMDHD2	chr16	2580357/C//A	nonsynonymous	0	2
GDE1	chr16	19528503/C//A	nonsynonymous	0	2
LOC81691	chr16	20837172/G//T	synonymous	0	2
CHST4	chr16	71571419/C//A	nonsynonymous	0	2
ZFHX3	chr16	72832035/C//T	nonsynonymous	0	2
ZDHHC7	chr16	85010005/G//A	nonsynonymous	0	2
SPG7	chr16	89623414/G//T	nonsynonymous	0	2
USP6	chr17	5058871/C//A	nonsynonymous	0	2
TP53	chr17	7577581/A//G	nonsynonymous	0	2
DNAH9	chr17	11725340/T//C	synonymous	0	2
TMEM99	chr17	38991270/G//T	nonsynonymous	0	2
IMPA2	chr18	12009885/C//A	nonsynonymous	0	2
SMAD4	chr18	48604747/C//A	nonsynonymous	0	2
PSMD8	chr19	38870015/C//A	synonymous	0	2
NOVA2	chr19	46457136/C//A	nonsynonymous	0	2
KLK9	chr19	51506379/G//T	synonymous	0	2
RFPL4A	chr19	56274381/G//T	nonsynonymous	0	2
ANKEF1	chr20	10019289/G//A	nonsynonymous	0	2
PTPRT	chr20	40980857/G//T	nonsynonymous	0	2
KREMEN1	chr22	29563029/C//A	synonymous	0	2

NHS	chrX	17745861/C//A	nonsynonymous	0	2
KLHL34	chrX	21674199/C//A	nonsynonymous	0	2
ZNF630	chrX	47918944/C//A	nonsynonymous	0	2
TRO	chrX	54950965/G//T	nonsynonymous	0	2
CAPN6	chrX	110489802/G//C	synonymous	0	2
ARHGAP36	chrX	130217867/G//T	nonsynonymous	0	2
DFFB	chr1	3782394/G//T	nonsynonymous	0	2
RSBN1	chr1	114340278/C//T	nonsynonymous	0	2
FCRL4	chr1	157556004/G//A	synonymous	0	2
LHCGR	chr2	48936162/C//A	nonsynonymous	0	2
CSRNP3	chr2	166451600/T//A	nonsynonymous	0	2
CRYBA2	chr2	219857956/G//C	synonymous	0	2
CRTAP	chr3	33155928/AG//A	nonsynonymous	0	2
STAC	chr3	36526485/G//A	nonsynonymous	0	2
SCN5A	chr3	38627529/G//A	nonsynonymous	0	2
SLC6A20	chr3	45801399/C//T	nonsynonymous	0	2
SLC25A20	chr3	48921494/C//A	nonsynonymous	0	2
DOCK3	chr3	51418495/T//A	synonymous	0	2
CPNE4	chr3	131274365/G//A	synonymous	0	2
SPSB4	chr3	140785052/C//T	nonsynonymous	0	2
SST	chr3	187387985/C//T	nonsynonymous	0	2
C5orf42	chr5	37179461/A//T	nonsynonymous	0	2
EGFLAM	chr5	38463931/C//A	synonymous	0	2
APC	chr5	112175225/G//T	nonsynonymous	0	2
PCDHA7	chr5	140215350/C//T	nonsynonymous	0	2
PCDHB12	chr5	140588661/C//T	nonsynonymous	0	2
GPX3	chr5	150407628/C//T	synonymous	0	2
CANX	chr5	179155670/G//T	synonymous	0	2
LY6G6F	chr6	31675736/G//C	synonymous	0	2
DDX43	chr6	74115486/C//T	synonymous	0	2
ALDH8A1	chr6	135239564/C//T	nonsynonymous	0	2
PLEKHG1	chr6	151161857/G//T	nonsynonymous	0	2
GCK	chr7	44189585/C//G	nonsynonymous	0	2
FBXO24	chr7	100184248/C//T	synonymous	0	2
KCNB2	chr8	73848485/G//A	nonsynonymous	0	2
TG	chr8	133913768/C//T	nonsynonymous	0	2
MPDZ	chr9	13168518/C//T	nonsynonymous	0	2
DIRAS2	chr9	93375521/C//T	nonsynonymous	0	2
ZNF462	chr9	109691168/G//A	nonsynonymous	0	2
GOLGA2	chr9	131036249/C//G	nonsynonymous	0	2
SARDH	chr9	136559447/C//T	synonymous	0	2
BTG4	chr11	111365969/C//T	nonsynonymous	0	2
CD163	chr12	7639557/C//T	synonymous	0	2
TAS2R8	chr12	10959334/C//G	nonsynonymous	0	2
DBX2	chr12	45444518/C//T	nonsynonymous	0	2
MYL2	chr12	111351058/C//A	nonsynonymous	0	2
TPTE2	chr13	20041417/C//T	nonsynonymous	0	2
MTUS2	chr13	29598896/G//C	nonsynonymous	0	2
MAGEL2	chr15	23890185/G//A	nonsynonymous	0	2
MPG	chr16	129531/C//T	synonymous	0	2
LCAT	chr16	67976345/C//T	synonymous	0	2
TP53	chr17	7578535/T//C	nonsynonymous	0	2
GIP	chr17	47041769/C//A	nonsynonymous	0	2
CACNA1G	chr17	48668973/C//A	nonsynonymous	0	2
USH1G	chr17	72915647/G//A	synonymous	0	2
MAP2K2	chr19	4099263/G//A	synonymous	0	2
XAB2	chr19	7684695/T//C	nonsynonymous	0	2
EPOR	chr19	11494818/G//C	synonymous	0	2
NOTCH3	chr19	15285145/C//T	synonymous	0	2
ZNF570	chr19	37961269/C//T	nonsynonymous	0	2
RYR1	chr19	39063839/G//A	nonsynonymous	0	2
CPT1C	chr19	50209614/G//A	synonymous	0	2
ZSCAN1	chr19	58565043/G//A	nonsynonymous	0	2
ZCCHC3	chr20	278982/G//T	nonsynonymous	0	2
EPB41L1	chr20	34773139/G//C	nonsynonymous	0	2
CD40	chr20	44751359/C//T	nonsynonymous	0	2
TSHZ2	chr20	51873021/G//A	synonymous	0	2
KRTAP19-5	chr21	31874285/C//T	nonsynonymous	0	2
KRTAP6-2	chr21	31971054/C//T	nonsynonymous	0	2
CECR1	chr22	17690387/C//G	nonsynonymous	0	2

CACNA1I	chr22	40038854/A//C	nonsynonymous	0	2
LMF2	chr22	50944532/C//A	nonsynonymous	0	2
H2BFM	chrX	103294635/C//T	nonsynonymous	0	2
PNCK	chrX	152936787/G//A	synonymous	0	2
NPPB	chr1	11917717/G//T	nonsynonymous	0	2
TMEM39B	chr1	32538571/C//T	synonymous	0	2
FMN2	chr1	240255446/G//A	nonsynonymous	0	2
ZNF385D	chr3	21706379/G//A	nonsynonymous	0	2
NEK10	chr3	27387643/G//A	nonsynonymous	0	2
ITIH1	chr3	52812362/G//A	nonsynonymous	0	2
ROBO2	chr3	77657067/G//A	synonymous	0	2
ARHGAP31	chr3	119112351/A//G	nonsynonymous	0	2
SENP5	chr3	196612643/C//T	synonymous	0	2
ANKRD50	chr4	125590912/G//A	nonsynonymous	0	2
TENM3	chr4	183721521/G//A	synonymous	0	2
	chr5	133481490/T//G	synonymous	0	2
TIGD6	chr5	149374780/C//A	nonsynonymous	0	2
B3GAT2	chr6	71666080/A//G	nonsynonymous	0	2
PAPOLB	chr7	4900392/G//A	synonymous	0	2
PCLO	chr7	82784308/G//A	nonsynonymous	0	2
DNAJC9-AS1	chr10	75035372/G//A	synonymous	0	2
SLC18A2	chr10	119013636/G//T	nonsynonymous	0	2
TENM4	chr11	78423573/G//A	synonymous	0	2
CNTN5	chr11	100141892/G//T	nonsynonymous	0	2
USP5	chr12	6961398/C//T	nonsynonymous	0	2
HECTD4	chr12	112648043/C//T	nonsynonymous	0	2
FES	chr15	91434372/C//T	nonsynonymous	0	2
ZNF763	chr19	12089064/T//A	nonsynonymous	0	2
CRX	chr19	48337694/G//A	synonymous	0	2
LAIR2	chr19	55019407/C//T	synonymous	0	2
ZNF579	chr19	56090487/C//T	synonymous	0	2
BPIFB3	chr20	31643311/G//A	nonsynonymous	0	2
UPRT	chrX	74494248/G//A	synonymous	0	2
NRK	chrX	105132350/A//C	nonsynonymous	0	2
PRDM16	chr1	3342162/G//A	nonsynonymous	0	2
TRIM63	chr1	26385013/C//T	synonymous	0	2
NPR1	chr1	153659739/G//A	nonsynonymous	0	2
LRRC52	chr1	165532779/G//A	synonymous	0	2
NMNAT2	chr1	183253859/C//T	nonsynonymous	0	2
ESRRG	chr1	216741434/C//T	nonsynonymous	0	2
WDCP	chr2	24262278/G//A	synonymous	0	2
SP140L	chr2	231264940/A//G	synonymous	0	2
COL6A3	chr2	238233365/C//T	synonymous	0	2
MSL2	chr3	135870832/A//T	synonymous	0	2
VWA5B2	chr3	183951436/G//A	synonymous	0	2
SLIT2	chr4	20512691/A//G	nonsynonymous	0	2
CCKAR	chr4	26483527/G//A	synonymous	0	2
FRYL	chr4	48566035/G//A	nonsynonymous	0	2
RAPGEF2	chr4	160274738/C//T	synonymous	0	2
TERT	chr5	1293801/G//A	synonymous	0	2
APC	chr5	112154999/C//T	nonsynonymous	0	2
APC	chr5	112175562/CA//C	nonsynonymous	0	2
NRG2	chr5	139260516/C//T	nonsynonymous	0	2
PCDHB5	chr5	140515710/G//A	nonsynonymous	0	2
PCDHB9	chr5	140569047/C//T	nonsynonymous	0	2
PCDHB10	chr5	140574287/C//T	nonsynonymous	0	2
SAYSD1	chr6	39073378/G//A	synonymous	0	2
NCR2	chr6	41303906/C//T	nonsynonymous	0	2
GRIK2	chr6	102337558/G//T	nonsynonymous	0	2
	chr6	133789885/C//T	synonymous	0	2
EPM2A	chr6	145948724/G//A	nonsynonymous	0	2
LPA	chr6	160977156/C//T	nonsynonymous	0	2
TCTE3	chr6	170151581/C//T	synonymous	0	2
CYP2W1	chr7	1024867/G//A	nonsynonymous	0	2
RELN	chr7	103180687/G//A	nonsynonymous	0	2
PIK3CG	chr7	106509049/G//C	nonsynonymous	0	2
CTTNBP2	chr7	117431644/G//A	nonsynonymous	0	2
GRM8	chr7	126173252/C//T	synonymous	0	2
NEFM	chr8	24773204/C//T	synonymous	0	2
HGSNAT	chr8	43052992/G//A	synonymous	0	2

CHD7	chr8	61654747/G//A	synonymous	0	2
SLA	chr8	134052256/T//C	nonsynonymous	0	2
FAM205A	chr9	34726953/C//T	nonsynonymous	0	2
NOL8	chr9	95085779/G//A	nonsynonymous	0	2
TNC	chr9	117848697/C//T	nonsynonymous	0	2
CUBN	chr10	16970240/G//A	nonsynonymous	0	2
RASGEF1A	chr10	43692431/G//A	synonymous	0	2
PLCE1	chr10	95791314/G//A	nonsynonymous	0	2
STX3	chr11	59560901/C//T	nonsynonymous	0	2
CHD4	chr12	6682343/G//A	synonymous	0	2
KRT84	chr12	52772163/G//A	synonymous	0	2
TMTC2	chr12	83358846/C//T	nonsynonymous	0	2
CDK8	chr13	26828860/G//C	nonsynonymous	0	2
MAP1A	chr15	43819399/C//T	nonsynonymous	0	2
HDC	chr15	50546832/G//A	synonymous	0	2
WDR72	chr15	53994411/A//G	nonsynonymous	0	2
PLEKHO2	chr15	65157119/G//A	nonsynonymous	0	2
TLE3	chr15	70344749/C//T	nonsynonymous	0	2
KIF7	chr15	90190181/G//T	synonymous	0	2
WWP2	chr16	69971024/GCTGA//G	nonsynonymous	0	2
CA5A	chr16	87960471/C//T	nonsynonymous	0	2
TUBB3	chr16	90002107/C//T	synonymous	0	2
ZNF232	chr17	5012922/C//T	nonsynonymous	0	2
KRT28	chr17	38948719/T//A	nonsynonymous	0	2
DNAH17	chr17	76435185/C//A	nonsynonymous	0	2
LOXHD1	chr18	44140257/G//A	synonymous	0	2
OR7E24	chr19	9361848/G//A	synonymous	0	2
ZNF208	chr19	22154545/G//T	synonymous	0	2
ZNF541	chr19	48041483/G//T	synonymous	0	2
SLC23A2	chr20	4864370/T//A	nonsynonymous	0	2
RALGAPA2	chr20	20620458/G//A	nonsynonymous	0	2
BRWD1	chr21	40559209/T//C	nonsynonymous	0	2
PHKA2	chrX	18938299/G//A	nonsynonymous	0	2
KDM6A	chrX	44949182/T//G	synonymous	0	2
AR	chrX	66905872/G//A	nonsynonymous	0	2
P2RY4	chrX	69478701/G//A	synonymous	0	2
TEX13C	chrX	124454052/C//T	synonymous	0	2
FMR1	chrX	147011514/C//G	nonsynonymous	0	2
FLNC	chr7	128486357/G//A	nonsynonymous	1	1
SLC6A11	chr3	10857946/C//T	synonymous	2	0
WASHC2C	chr10	46242094/A//G	nonsynonymous	1	1
PLOD1	chr1	12030739/C//T	nonsynonymous	0	2
CELA2A	chr1	15793875/C//T	synonymous	0	2
DDI2	chr1	15978313/G//C	nonsynonymous	0	2
FCN3	chr1	27700948/G//A	synonymous	0	2
TXLNA	chr1	32659717/C//T	nonsynonymous	0	2
YARS	chr1	33246687/G//A	synonymous	0	2
RPS8	chr1	45243669/C//T	synonymous	0	2
ACOT11	chr1	55051557/C//T	synonymous	0	2
TTC4	chr1	55183239/T//C	nonsynonymous	0	2
C8A	chr1	57341830/G//A	nonsynonymous	0	2
LRIG2	chr1	113637023/C//T	nonsynonymous	0	2
ADAM30	chr1	120438227/G//C	nonsynonymous	0	2
TDRKH	chr1	151749074/G//A	synonymous	0	2
S100A7L2	chr1	153409544/C//T	nonsynonymous	0	2
INSRR	chr1	156815042/C//T	nonsynonymous	0	2
NTRK1	chr1	156834566/C//T	nonsynonymous	0	2
FCRL5	chr1	157514748/G//A	synonymous	0	2
CD1E	chr1	158325189/G//A	nonsynonymous	0	2
OR6Y1	chr1	158516941/C//T	nonsynonymous	0	2
OR10Z1	chr1	158576671/C//T	nonsynonymous	0	2
OR6K6	chr1	158724968/C//T	synonymous	0	2
FCER1A	chr1	159275991/A//C	nonsynonymous	0	2
LMX1A	chr1	165175156/G//A	synonymous	0	2
ADCY10	chr1	167815028/C//T	nonsynonymous	0	2
MROH9	chr1	170967498/C//T	nonsynonymous	0	2
PRRC2C	chr1	171492426/T//C	synonymous	0	2
SLC9C2	chr1	173523914/C//T	nonsynonymous	0	2
BRINP2	chr1	177247892/C//T	synonymous	0	2
TDRD5	chr1	179660070/G//A	nonsynonymous	0	2

CR2	chr1	207646175/A//G	synonymous	0	2
CR2	chr1	207651288/G//A	synonymous	0	2
DIEXF	chr1	210010361/C//T	synonymous	0	2
NSL1	chr1	212957795/G//A	synonymous	0	2
USH2A	chr1	215848027/G//A	nonsynonymous	0	2
DUSP10	chr1	221879780/A//C	nonsynonymous	0	2
PRSS38	chr1	228003916/G//A	nonsynonymous	0	2
OBSCN	chr1	228432252/C//T	nonsynonymous	0	2
RGS7	chr1	240979701/T//G	nonsynonymous	0	2
OR2T34	chr1	248737333/G//A	synonymous	0	2
ATP6V1C2	chr2	10866679/G//A	nonsynonymous	0	2
PLEK	chr2	68622942/G//A	synonymous	0	2
VWA3B	chr2	98804433/C//T	synonymous	0	2
RGPD4	chr2	108496546/C//T	nonsynonymous	0	2
ANAPC1	chr2	112621472/C//T	nonsynonymous	0	2
TMEM185B	chr2	120980377/G//A	nonsynonymous	0	2
SCN7A	chr2	167288867/C//T	synonymous	0	2
NOSTRIN	chr2	169659163/G//A	nonsynonymous	0	2
DNAH7	chr2	196753005/C//T	nonsynonymous	0	2
ZFAND2B	chr2	220072755/G//A	synonymous	0	2
IL17RE	chr3	9948451/C//T	nonsynonymous	0	2
IRAK2	chr3	10251277/C//T	synonymous	0	2
WDR48	chr3	39093488/G//A	synonymous	0	2
BSN	chr3	49690076/C//T	synonymous	0	2
DNAH12	chr3	57494116/C//T	nonsynonymous	0	2
VGLL3	chr3	86996222/A//G	synonymous	0	2
PARP14	chr3	122422678/G//A	synonymous	0	2
DNAJC13	chr3	132247132/A//G	nonsynonymous	0	2
TMEM108	chr3	133099017/C//T	synonymous	0	2
IGSF10	chr3	151160889/T//A	nonsynonymous	0	2
ATP11B	chr3	182559881/C//T	synonymous	0	2
P3H2	chr3	189681803/C//T	nonsynonymous	0	2
DRD5	chr4	9784373/C//T	synonymous	0	2
BST1	chr4	15716934/C//T	synonymous	0	2
KDR	chr4	55973929/C//T	nonsynonymous	0	2
PDCL2	chr4	56435957/C//T	nonsynonymous	0	2
DMP1	chr4	88584536/G//A	synonymous	0	2
STPG2	chr4	98902463/A//T	nonsynonymous	0	2
FSTL5	chr4	162459390/T//C	nonsynonymous	0	2
GLRA3	chr4	175649825/G//A	nonsynonymous	0	2
FAT1	chr4	187629251/T//C	synonymous	0	2
ZFP42	chr4	188924097/G//A	nonsynonymous	0	2
UGT3A2	chr5	36064457/G//A	synonymous	0	2
EGFLAM	chr5	38370519/G//A	nonsynonymous	0	2
CMYA5	chr5	79031244/C//T	nonsynonymous	0	2
SLC27A6	chr5	128302184/C//T	synonymous	0	2
PSD2	chr5	139189152/C//T	nonsynonymous	0	2
PCDHA8	chr5	140223200/C//T	nonsynonymous	0	2
PCDHA9	chr5	140228065/A//C	synonymous	0	2
PCDHA13	chr5	140264182/C//T	nonsynonymous	0	2
TTC1	chr5	159437572/G//T	nonsynonymous	0	2
GRM6	chr5	178410162/C//T	nonsynonymous	0	2
F13A1	chr6	6305779/A//G	synonymous	0	2
OR2B6	chr6	27925444/C//T	synonymous	0	2
EHMT2	chr6	31855418/G//A	synonymous	0	2
TREM1	chr6	41250262/G//A	nonsynonymous	0	2
AARS2	chr6	44272217/A//C	nonsynonymous	0	2
ENPP4	chr6	46108786/C//T	synonymous	0	2
DST	chr6	56392425/G//A	nonsynonymous	0	2
DST	chr6	56498960/A//T	synonymous	0	2
COL9A1	chr6	71012677/T//C	synonymous	0	2
SMAP1	chr6	71571319/C//T	synonymous	0	2
RFX6	chr6	117237395/G//A	nonsynonymous	0	2
RFX6	chr6	117237412/C//T	nonsynonymous	0	2
DAGLB	chr7	6461373/G//A	synonymous	0	2
ABCA13	chr7	48556372/G//C	synonymous	0	2
SEPT14	chr7	55914232/G//A	synonymous	0	2
ZNF117	chr7	64439855/G//A	nonsynonymous	0	2
TPST1	chr7	65706034/T//C	nonsynonymous	0	2
SEMA3E	chr7	82996945/T//C	nonsynonymous	0	2

SEMA3E	chr7	83021865/T//G	synonymous	0	2
ARPC1B	chr7	98992093/A//G	nonsynonymous	0	2
ZNF394	chr7	99097557/G//A	nonsynonymous	0	2
MBLAC1	chr7	99725234/G//A	synonymous	0	2
PVRIG	chr7	99818738/G//A	nonsynonymous	0	2
MUC17	chr7	100676134/C//T	synonymous	0	2
MUC17	chr7	100682015/A//G	nonsynonymous	0	2
RELN	chr7	103130334/G//A	synonymous	0	2
LAMB4	chr7	107718723/G//A	synonymous	0	2
C7orf66	chr7	108524089/G//A	nonsynonymous	0	2
KIAA1549	chr7	138583795/G//A	synonymous	0	2
KIAA1147	chr7	141385412/G//A	synonymous	0	2
MGAM	chr7	141724956/G//A	synonymous	0	2
MGAM	chr7	141764301/C//T	nonsynonymous	0	2
PRSS1	chr7	142459658/C//T	synonymous	0	2
EPHB6	chr7	142568774/G//A	synonymous	0	2
NOBOX	chr7	144094550/C//T	nonsynonymous	0	2
SLC4A2	chr7	150772569/C//T	nonsynonymous	0	2
PTPRN2	chr7	157959649/G//A	nonsynonymous	0	2
CSGALNACT1	chr8	19277882/G//A	synonymous	0	2
GSR	chr8	30538543/G//A	nonsynonymous	0	2
TEX15	chr8	30706440/G//A	nonsynonymous	0	2
CHD7	chr8	61778369/T//G	nonsynonymous	0	2
TRPA1	chr8	72935049/C//T	synonymous	0	2
KCNB2	chr8	73480497/G//A	synonymous	0	2
MATN2	chr8	99047886/A//T	nonsynonymous	0	2
EFR3A	chr8	132982763/T//C	synonymous	0	2
SLC45A4	chr8	142227249/C//T	nonsynonymous	0	2
LYPD2	chr8	143831696/G//A	synonymous	0	2
ZC3H3	chr8	144557752/G//A	synonymous	0	2
GLIS3	chr9	4117797/G//A	nonsynonymous	0	2
TYRP1	chr9	12694104/G//A	synonymous	0	2
TTC39B	chr9	15171991/G//A	synonymous	0	2
DDX58	chr9	32488094/T//C	nonsynonymous	0	2
APTX	chr9	33001630/G//A	synonymous	0	2
SPATA31A6	chr9	43627602/G//A	nonsynonymous	0	2
GDA	chr9	74865725/C//T	synonymous	0	2
CYLC2	chr9	105765505/G//T	nonsynonymous	0	2
ABCA1	chr9	107576392/A//C	synonymous	0	2
SVEP1	chr9	113168989/G//A	nonsynonymous	0	2
PAPPA	chr9	119065135/G//A	nonsynonymous	0	2
STRBP	chr9	125932183/C//T	nonsynonymous	0	2
USP20	chr9	132640649/G//A	synonymous	0	2
SLC39A12-AS1	chr10	18291941/C//T	synonymous	0	2
GAD2	chr10	26575294/C//T	synonymous	0	2
ANK3	chr10	61835456/G//A	nonsynonymous	0	2
CYP2C8	chr10	96796764/G//A	synonymous	0	2
CYP2C8	chr10	96827385/G//A	nonsynonymous	0	2
ADGRA1	chr10	134942208/C//G	synonymous	0	2
MUC5B	chr11	1264836/C//T	synonymous	0	2
OR52R1	chr11	4824767/G//T	nonsynonymous	0	2
OR51A4	chr11	4968294/T//A	nonsynonymous	0	2
OR51M1	chr11	5410987/C//T	nonsynonymous	0	2
OR51I2	chr11	5475487/G//A	nonsynonymous	0	2
OR56B4	chr11	6129689/C//T	synonymous	0	2
OVCH2	chr11	7718250/A//C	synonymous	0	2
OR5P2	chr11	7818388/G//A	synonymous	0	2
ANO3	chr11	26663444/T//C	synonymous	0	2
OR5L1	chr11	55579670/C//T	nonsynonymous	0	2
OR10AG1	chr11	55735873/A//T	nonsynonymous	0	2
OR5T2	chr11	56000410/C//T	synonymous	0	2
OR8H1	chr11	56057866/G//A	synonymous	0	2
DNAJB13	chr11	73670586/G//A	nonsynonymous	0	2
GRM5	chr11	88301131/G//A	nonsynonymous	0	2
CNTN5	chr11	100211289/G//A	nonsynonymous	0	2
TBRG1	chr11	124495721/A//T	nonsynonymous	0	2
ROBO4	chr11	124763877/C//T	synonymous	0	2
ETS1	chr11	128359363/G//A	synonymous	0	2
DDX11	chr12	31254840/C//T	nonsynonymous	0	2
LRRK2	chr12	40668707/A//T	nonsynonymous	0	2

MCRS1	chr12	49953259/G//A	nonsynonymous	0	2
NCKAP1L	chr12	54920357/C//T	synonymous	0	2
OR6C6	chr12	55688931/A//G	nonsynonymous	0	2
	chr12	56708420/A//G	synonymous	0	2
EEA1	chr12	93181662/G//A	nonsynonymous	0	2
HCFC2	chr12	104480655/T//A	nonsynonymous	0	2
SCARB1	chr12	125292481/G//A	synonymous	0	2
TMEM132B	chr12	126138713/G//A	synonymous	0	2
SACS	chr13	23911792/G//A	nonsynonymous	0	2
RNF17	chr13	25406036/G//A	nonsynonymous	0	2
MTUS2	chr13	29933467/G//A	nonsynonymous	0	2
KTN1	chr14	56107845/T//G	nonsynonymous	0	2
	chr14	76071894/A//G	synonymous	0	2
OR4M2	chr15	22368699/A//T	nonsynonymous	0	2
NPAP1	chr15	24922085/C//T	synonymous	0	2
NPAP1	chr15	24924372/C//T	nonsynonymous	0	2
KNL1	chr15	40954376/T//C	nonsynonymous	0	2
SLC30A4	chr15	45814199/G//A	synonymous	0	2
CTXN2	chr15	48493546/G//A	nonsynonymous	0	2
MYO9A	chr15	72189834/G//A	synonymous	0	2
CSPG4	chr15	75979765/G//A	nonsynonymous	0	2
CTSH	chr15	79221748/A//T	synonymous	0	2
BNC1	chr15	83926716/G//A	synonymous	0	2
OR4F15	chr15	102359249/A//G	nonsynonymous	0	2
TPSD1	chr16	1306582/C//T	nonsynonymous	0	2
MMP25	chr16	3100312/G//A	nonsynonymous	0	2
ZNF597	chr16	3493192/G//A	synonymous	0	2
MVP	chr16	29859246/C//T	nonsynonymous	0	2
ALDOA	chr16	30080264/A//T	nonsynonymous	0	2
ITGAM	chr16	31336577/C//T	synonymous	0	2
ZNF469	chr16	88497964/C//T	synonymous	0	2
ASGR1	chr17	7077599/C//T	nonsynonymous	0	2
NEURL4	chr17	7226074/G//A	nonsynonymous	0	2
ALOX15B	chr17	7951863/G//A	nonsynonymous	0	2
MYH2	chr17	10428353/G//A	synonymous	0	2
DNAH9	chr17	11513830/G//A	synonymous	0	2
KRT16	chr17	39768746/C//T	synonymous	0	2
AOC3	chr17	41004692/C//T	synonymous	0	2
XYLT2	chr17	48435893/T//G	nonsynonymous	0	2
TANC2	chr17	61497576/C//T	synonymous	0	2
FAM20A	chr17	66538840/G//A	nonsynonymous	0	2
RNF213	chr17	78333911/C//T	nonsynonymous	0	2
SLC14A2	chr18	43212350/G//A	nonsynonymous	0	2
MYO5B	chr18	47429135/G//A	synonymous	0	2
DOK6	chr18	67266712/G//A	synonymous	0	2
RTTN	chr18	67721486/G//A	nonsynonymous	0	2
MED16	chr19	873547/C//A	nonsynonymous	0	2
TMIGD2	chr19	4292675/G//A	nonsynonymous	0	2
MUC16	chr19	9046491/C//T	nonsynonymous	0	2
MUC16	chr19	9088160/G//A	nonsynonymous	0	2
OR7D4	chr19	9324972/G//A	nonsynonymous	0	2
ZNF846	chr19	9868788/G//A	nonsynonymous	0	2
CLEC17A	chr19	14707698/C//T	synonymous	0	2
OR7C1	chr19	14910425/G//A	nonsynonymous	0	2
OR10H2	chr19	15839661/G//A	nonsynonymous	0	2
OR10H1	chr19	15918812/G//A	synonymous	0	2
LRP3	chr19	33696243/C//T	synonymous	0	2
FFAR2	chr19	35941507/C//T	synonymous	0	2
WDR87	chr19	38379457/C//T	synonymous	0	2
CAPN12	chr19	39221478/C//T	synonymous	0	2
LGALS13	chr19	40095971/C//T	synonymous	0	2
FCGBP	chr19	40434171/C//T	nonsynonymous	0	2
PSG4	chr19	43699196/C//T	synonymous	0	2
PSG9	chr19	43762387/C//T	nonsynonymous	0	2
CEACAM16	chr19	45206647/C//T	synonymous	0	2
EXOC3L2	chr19	45719460/G//A	synonymous	0	2
FBXO46	chr19	46215810/G//A	nonsynonymous	0	2
FAM90A27P	chr19	53787293/G//A	synonymous	0	2
KIR3DL3	chr19	55237674/T//A	nonsynonymous	0	2
KIR3DL1	chr19	55329873/C//T	synonymous	0	2

NLRP2	chr19	55505734/C//T	nonsynonymous	0	2
GP6	chr19	55526472/G//A	synonymous	0	2
NLRP8	chr19	56466035/A//T	nonsynonymous	0	2
USP29	chr19	57641979/C//T	nonsynonymous	0	2
VPS16	chr20	2842528/T//A	nonsynonymous	0	2
FERMT1	chr20	6096576/G//A	synonymous	0	2
DNMT3B	chr20	31388691/C//G	nonsynonymous	0	2
PTPRT	chr20	40747110/C//T	nonsynonymous	0	2
PTPN1	chr20	49195089/G//A	nonsynonymous	0	2
CRYAA	chr21	44590630/C//T	nonsynonymous	0	2
KRTAP12-4	chr21	46074209/G//A	nonsynonymous	0	2
SEPT5	chr22	19709259/G//A	nonsynonymous	0	2
UPB1	chr22	24916348/C//T	synonymous	0	2
MYO18B	chr22	26317274/G//A	synonymous	0	2
PRR14L	chr22	32113223/C//T	nonsynonymous	0	2
TST	chr22	37414406/G//A	nonsynonymous	0	2
SMC1B	chr22	45757774/C//T	nonsynonymous	0	2
ZBED1	chrX	2408110/G//A	synonymous	0	2
MXRA5	chrX	3228960/C//T	synonymous	0	2
DCAF8L1	chrX	27998321/C//T	synonymous	0	2
ZNF81	chrX	47755298/C//T	nonsynonymous	0	2
IQSEC2	chrX	53264116/T//A	nonsynonymous	0	2
PAGE5	chrX	55246990/G//A	synonymous	0	2
FAAH2	chrX	57515301/C//T	nonsynonymous	0	2
MUM1L1	chrX	105450995/G//A	nonsynonymous	0	2
AMMECR1	chrX	109507750/A//T	nonsynonymous	0	2
TRPC5	chrX	111195560/G//A	nonsynonymous	0	2
TENM1	chrX	123518300/C//T	nonsynonymous	0	2
TENM1	chrX	123785942/C//T	synonymous	0	2
MAGEC1	chrX	140996446/G//A	nonsynonymous	0	2
MAGEC2	chrX	141290979/G//A	synonymous	0	2
F8	chrX	154194921/C//T	nonsynonymous	0	2
NLGN4Y	chrY	16952902/G//A	synonymous	0	2
CDC27	chr17	45214631/G//A	synonymous	0	2
CDC27	chr17	45214673/T//C	synonymous	0	2
PEX10	chr1	2338163/G//A	nonsynonymous	0	2
AGO4	chr1	36307291/T//A	synonymous	0	2
FSIP2	chr2	186658896/C//T	nonsynonymous	0	2
BTN2A2	chr6	26385354/G//A	nonsynonymous	0	2
FGD2	chr6	36990047/G//A	synonymous	0	2
TNS3	chr7	47409208/C//T	synonymous	0	2
WBSCR17	chr7	70853335/G//A	synonymous	0	2
ASB15	chr7	123269051/A//G	nonsynonymous	0	2
FLNC	chr7	128491632/G//A	nonsynonymous	0	2
DPYS	chr8	105441779/A//C	nonsynonymous	0	2
SAXO1	chr9	18928165/G//A	nonsynonymous	0	2
TRIM14	chr9	100857203/C//T	nonsynonymous	0	2
ANXA11	chr10	81928742/C//T	nonsynonymous	0	2
ENO4	chr10	118641155/C//T	nonsynonymous	0	2
RBMXL2	chr11	7110934/C//T	nonsynonymous	0	2
MYBPC3	chr11	47364678/G//A	synonymous	0	2
POU6F1	chr12	51590663/G//C	synonymous	0	2
TMBIM4	chr12	66539639/A//G	nonsynonymous	0	2
SLC10A2	chr13	103710740/G//T	synonymous	0	2
RASA3	chr13	114793321/G//T	nonsynonymous	0	2
HEATR4	chr14	73989454/C//T	nonsynonymous	0	2
FAM161B	chr14	74404384/G//A	nonsynonymous	0	2
TP53	chr17	7578526/C//A	nonsynonymous	0	2
MKS1	chr17	56283396/C//T	synonymous	0	2
RNF157	chr17	74157998/C//T	nonsynonymous	0	2
SPPL2B	chr19	2343274/G//A	nonsynonymous	0	2
CAMSAP3	chr19	7676731/C//T	nonsynonymous	0	2
CCL25	chr19	8121335/C//T	nonsynonymous	0	2
HAS1	chr19	52220435/C//T	synonymous	0	2
CST5	chr20	23856749/C//A	synonymous	0	2
ANOS1	chrX	8504983/C//A	nonsynonymous	0	2
CA14	chr1	150233925/C//T	synonymous	2	0
KCNT2	chr1	196397307/C//A	synonymous	2	0
KIF21B	chr1	200969696/G//A	nonsynonymous	2	0
EXOC8	chr1	231472400/G//A	synonymous	2	0

NRXN1	chr2	51254925/G//A	nonsynonymous	2	0
ZNF638	chr2	71650791/G//C	nonsynonymous	2	0
MAP4K4	chr2	102486182/G//A	synonymous	2	0
TTN	chr2	179460311/C//T	nonsynonymous	2	0
TTN	chr2	179588283/G//A	nonsynonymous	2	0
DUSP19	chr2	183943883/C//T	synonymous	2	0
ZNF502	chr3	44763580/G//A	nonsynonymous	2	0
ADAMTS9	chr3	64526795/C//A	nonsynonymous	2	0
LINC01205	chr3	109129037/C//T	synonymous	2	0
PARP14	chr3	122420480/A//T	nonsynonymous	2	0
PRR23B	chr3	138739094/A//G	nonsynonymous	2	0
CCDC96	chr4	7043936/G//T	nonsynonymous	2	0
SORCS2	chr4	7640138/G//T	nonsynonymous	2	0
ADGRL3	chr4	62897212/G//T	nonsynonymous	2	0
FAT4	chr4	126237907/C//T	nonsynonymous	2	0
PLEKHG4B	chr5	162951/G//A	nonsynonymous	2	0
APC	chr5	112174475/C//T	nonsynonymous	2	0
JARID2	chr6	15496357/C//G	synonymous	2	0
CDC5L	chr6	44376240/TC//T	nonsynonymous	2	0
ZFAND2A	chr7	1197351/C//A	nonsynonymous	2	0
EEPD1	chr7	36339009/C//A	synonymous	2	0
LRCH4	chr7	100183719/G//A	nonsynonymous	2	0
PIK3CG	chr7	106508186/C//T	synonymous	2	0
TMEM168	chr7	112407702/A//G	synonymous	2	0
CSMD3	chr8	113562942/C//T	nonsynonymous	2	0
SCRIB	chr8	144892714/C//T	nonsynonymous	2	0
ZNF517	chr8	146033665/G//A	nonsynonymous	2	0
DENND4C	chr9	19296161/G//A	nonsynonymous	2	0
ADARB2	chr10	1405862/C//T	synonymous	2	0
LOXL4	chr10	100021835/C//T	nonsynonymous	2	0
SORCS3	chr10	106916987/G//A	nonsynonymous	2	0
PNLIPRP1	chr10	118354337/G//T	synonymous	2	0
SMPD1	chr11	6415617/T//A	nonsynonymous	2	0
AHNAK	chr11	62297747/T//G	nonsynonymous	2	0
CLEC1A	chr12	10241808/G//A	synonymous	2	0
KLRC2	chr12	10584759/C//T	nonsynonymous	2	0
LRRK2	chr12	40629460/C//G	nonsynonymous	2	0
FRY	chr13	32785102/G//A	nonsynonymous	2	0
TRPC4	chr13	38357381/C//T	synonymous	2	0
CEP170B	chr14	105349447/C//T	nonsynonymous	2	0
HERC2	chr15	28459335/C//T	nonsynonymous	2	0
PDXDC1	chr16	15122810/C//T	nonsynonymous	2	0
NETO2	chr16	47117654/G//A	synonymous	2	0
AMFR	chr16	56423242/T//A	synonymous	2	0
ACD	chr16	67692120/G//T	synonymous	2	0
TUBB3	chr16	90001934/C//T	nonsynonymous	2	0
TTLL6	chr17	46847424/G//A	synonymous	2	0
BAHCC1	chr17	79423518/G//A	nonsynonymous	2	0
SEH1L	chr18	12982577/C//T	synonymous	2	0
CDH2	chr18	25570209/C//T	nonsynonymous	2	0
ADAMTS10	chr19	8668648/G//A	nonsynonymous	2	0
MUC16	chr19	9058740/C//T	nonsynonymous	2	0
COL5A3	chr19	10085063/G//A	nonsynonymous	2	0
COL5A3	chr19	10097252/C//T	nonsynonymous	2	0
MEF2B	chr19	19256620/C//A	nonsynonymous	2	0
PTPRT	chr20	41419986/C//T	nonsynonymous	2	0
OCSTAMP	chr20	45170467/G//A	nonsynonymous	2	0
NCAM2	chr21	22710768/C//T	nonsynonymous	2	0
AMER1	chrX	63410856/G//A	nonsynonymous	2	0
GPR61	chr1	110085839/C//T	synonymous	0	2
TP53BP2	chr1	223991129/ATT//A	nonsynonymous	0	2
TTC13	chr1	231044713/A//G	nonsynonymous	0	2
GCKR	chr2	27728609/C//G	nonsynonymous	0	2
NRXN1	chr2	50765703/C//T	nonsynonymous	0	2
DNAH6	chr2	84822874/G//A	synonymous	0	2
IL18R1	chr2	103013260/A//G	nonsynonymous	0	2
ABCB11	chr2	169783738/T//G	nonsynonymous	0	2
GPR155	chr2	175333714/T//C	nonsynonymous	0	2
HOXD4	chr2	177017341/C//G	nonsynonymous	0	2
FZD7	chr2	202899481/A//C	synonymous	0	2

CCDC14	chr3	123634231/G//T	nonsynonymous	0	2
CDH9	chr5	26902652/C//T	nonsynonymous	0	2
CDH9	chr5	26902665/T//G	nonsynonymous	0	2
SMPDL3A	chr6	123110440/C//T	synonymous	0	2
HIVEP2	chr6	143093216/T//C	nonsynonymous	0	2
TNRC18	chr7	5417165/G//A	synonymous	0	2
ZNF3	chr7	99669043/A//C	nonsynonymous	0	2
RUNX1T1	chr8	93029545/C//T	synonymous	0	2
FAM83H	chr8	144808504/T//C	nonsynonymous	0	2
GNA14	chr9	80039083/G//C	nonsynonymous	0	2
CALML3	chr10	5567131/T//C	nonsynonymous	0	2
	chr10	73466908/A//G	synonymous	0	2
	chr10	102768311/C//G	synonymous	0	2
NPS	chr10	129347809/G//T	nonsynonymous	0	2
E2F8	chr11	19258974/C//A	nonsynonymous	0	2
VWF	chr12	6122656/G//A	synonymous	0	2
ARID2	chr12	46215247/A//T	nonsynonymous	0	2
NBEA	chr13	35923332/A//G	synonymous	0	2
DLL4	chr15	41230310/A//G	synonymous	0	2
LIPC	chr15	58834056/A//G	nonsynonymous	0	2
TRIP4	chr15	64710919/G//A	synonymous	0	2
ITGAM	chr16	31335741/C//G	nonsynonymous	0	2
MEGF8	chr19	42857925/A//T	nonsynonymous	0	2
AAR2	chr20	34828240/C//T	synonymous	0	2
SOGA1	chr20	35438510/T//C	nonsynonymous	0	2
UMODL1	chr21	43529675/G//A	nonsynonymous	0	2
RLIM	chrX	73814221/T//C	nonsynonymous	0	2
CELA2A	chr1	15792640/G//A	nonsynonymous	0	2
SESN2	chr1	28598312/G//A	nonsynonymous	0	2
TMEM35B	chr1	35449460/C//A	nonsynonymous	0	2
PLD5	chr1	242277329/C//A	nonsynonymous	0	2
DNAH1	chr3	52379582/C//T	nonsynonymous	0	2
PDZRN3	chr3	73433871/C//T	nonsynonymous	0	2
PRR23C	chr3	138762920/G//A	synonymous	0	2
MAD2L1	chr4	120986940/C//T	nonsynonymous	0	2
CDH9	chr5	26881457/A//T	nonsynonymous	0	2
NEFL	chr8	24811218/G//A	nonsynonymous	0	2
FZD6	chr8	104336880/G//A	synonymous	0	2
KANK1	chr9	712005/C//T	synonymous	0	2
APBA1	chr9	72131897/C//T	nonsynonymous	0	2
PSD	chr10	104164434/G//A	nonsynonymous	0	2
PKP3	chr11	400597/G//A	synonymous	0	2
OR52B2	chr11	6191185/G//A	synonymous	0	2
MAP4K2	chr11	64559404/G//A	nonsynonymous	0	2
DNAJC14	chr12	56221415/T//G	nonsynonymous	0	2
SACS	chr13	23913385/A//C	nonsynonymous	0	2
NPAS3	chr14	34243738/T//C	nonsynonymous	0	2
PGBD4	chr15	34395020/C//T	synonymous	0	2
KRT222	chr17	38816346/G//A	synonymous	0	2
EPX	chr17	56272378/C//T	synonymous	0	2
SUPT4H1	chr17	56428830/C//T	nonsynonymous	0	2
ZNF607	chr19	38189301/T//C	nonsynonymous	0	2
SIGLEC8	chr19	51955858/G//T	synonymous	0	2
PEG3	chr19	57325802/A//C	nonsynonymous	0	2
MEI1	chr22	42177301/T//C	synonymous	0	2
NLGN4X	chrX	5811008/C//T	synonymous	0	2
FLJ44635	chrX	71380085/A//G	nonsynonymous	0	2
MUC12	chr7	100646613/A//G	nonsynonymous	1	1
UBR4	chr1	19475055/G//A	nonsynonymous	0	2
NRAS	chr1	115258747/C//T	nonsynonymous	0	2
BRINP3	chr1	190067206/G//A	nonsynonymous	0	2
RYR2	chr1	237941973/G//T	nonsynonymous	0	2
OTOF	chr2	26696867/G//A	nonsynonymous	0	2
FIGN	chr2	164467671/G//T	nonsynonymous	0	2
LRP2	chr2	170058138/G//A	nonsynonymous	0	2
SPATS2L	chr2	201342380/C//T	nonsynonymous	0	2
PARD3B	chr2	205978268/A//T	nonsynonymous	0	2
LRRFIP1	chr2	238668828/C//T	nonsynonymous	0	2
LHFPL4	chr3	9547661/C//T	synonymous	0	2
HDAC11	chr3	13545700/C//T	synonymous	0	2

DLEC1	chr3	38138681/C//T	synonymous	0	2
IQCF1	chr3	51928947/AAGGCCCTGAGTCCA(	nonsynonymous	0	2
ROBO2	chr3	77623761/C//T	nonsynonymous	0	2
OTOP1	chr4	4199549/G//A	nonsynonymous	0	2
HPGDS	chr4	95239083/A//G	nonsynonymous	0	2
NAA15	chr4	140282934/G//A	synonymous	0	2
RXFP3	chr5	33937730/C//T	synonymous	0	2
RICTOR	chr5	38954879/T//C	synonymous	0	2
PCDHA5	chr5	140202652/C//T	nonsynonymous	0	2
PCDHA13	chr5	140264048/G//C	nonsynonymous	0	2
PCDHB4	chr5	140503944/C//A	nonsynonymous	0	2
PLN	chr6	118880157/C//T	nonsynonymous	0	2
FNDC1	chr6	159657346/G//A	nonsynonymous	0	2
VPS41	chr7	38835199/A//G	nonsynonymous	0	2
VPS50	chr7	92940544/T//C	synonymous	0	2
PSMC2	chr7	103008511/G//A	synonymous	0	2
RELN	chr7	103294537/T//C	synonymous	0	2
SSPO	chr7	149511666/C//T	synonymous	0	2
PTPRN2	chr7	157959718/G//T	nonsynonymous	0	2
RALYL	chr8	85441584/G//A	nonsynonymous	0	2
RUNX1T1	chr8	92972726/C//T	nonsynonymous	0	2
EPPK1	chr8	144946137/G//C	nonsynonymous	0	2
TNC	chr9	117848461/C//T	nonsynonymous	0	2
CEL	chr9	135942036/G//A	nonsynonymous	0	2
DIP2C	chr10	373045/G//C	synonymous	0	2
FRMD4A	chr10	13698843/C//T	nonsynonymous	0	2
SH3PXD2A	chr10	105361825/G//C	synonymous	0	2
GRK5	chr10	121201572/C//T	synonymous	0	2
NKX6-2	chr10	134598483/C//A	nonsynonymous	0	2
MUC15	chr11	26587101/G//A	nonsynonymous	0	2
LTBP3	chr11	65306534/GGGCGGCGTC//G	synonymous	0	2
FGF3	chr11	69625251/C//T	nonsynonymous	0	2
POLD3	chr11	74329613/C//T	nonsynonymous	0	2
MTNR1B	chr11	92714845/C//T	synonymous	0	2
ELMOD1	chr11	107521069/C//T	nonsynonymous	0	2
OLR1	chr12	10319473/G//A	nonsynonymous	0	2
SOX5	chr12	23716202/C//T	nonsynonymous	0	2
PDZRN4	chr12	41967454/G//A	nonsynonymous	0	2
SOAT2	chr12	53509269/C//T	nonsynonymous	0	2
TRPV4	chr12	110232135/G//C	nonsynonymous	0	2
SCARB1	chr12	125284728/G//A	nonsynonymous	0	2
SHISA2	chr13	26624903/C//A	nonsynonymous	0	2
FLRT2	chr14	86088314/C//T	synonymous	0	2
GABRG3	chr15	27772697/C//T	synonymous	0	2
SLTM	chr15	59205706/C//T	nonsynonymous	0	2
CYP1A2	chr15	75042385/C//T	synonymous	0	2
IRX5	chr16	54966758/G//A	nonsynonymous	0	2
JPH3	chr16	87677988/C//T	synonymous	0	2
PIEZO1	chr16	88786135/C//T	synonymous	0	2
MFSD6L	chr17	8701955/G//A	nonsynonymous	0	2
RNF213	chr17	78313970/G//A	nonsynonymous	0	2
CCDC57	chr17	80159745/C//A	nonsynonymous	0	2
SLC25A23	chr19	6454630/C//T	synonymous	0	2
ZNF492	chr19	22847832/G//A	nonsynonymous	0	2
CEACAM21	chr19	42090784/G//A	nonsynonymous	0	2
SIGLEC1	chr20	3684736/C//A	nonsynonymous	0	2
SEL1L2	chr20	13866929/A//T	synonymous	0	2
SEMG1	chr20	43836216/C//T	nonsynonymous	0	2
PCIF1	chr20	44575770/G//A	nonsynonymous	0	2
KRTAP24-1	chr21	31655055/C//T	nonsynonymous	0	2
COL18A1	chr21	46929314/C//T	synonymous	0	2
ARVCF	chr22	19959493/G//A	synonymous	0	2
MAP7D3	chrX	135308130/G//A	nonsynonymous	0	2
ASPM	chr1	197112784/T//C	nonsynonymous	2	0
SPATA3	chr2	231861231/C//T	nonsynonymous	2	0
OR5H14	chr3	97869141/G//T	nonsynonymous	2	0
PCDHA8	chr5	140223065/C//T	nonsynonymous	2	0
STXBP5	chr6	147705975/A//G	synonymous	2	0
FAM120B	chr6	170632188/C//T	nonsynonymous	2	0
SRPK2	chr7	104766711/T//C	synonymous	2	0

ESYT2	chr7	158557420/T//G	nonsynonymous	2	0
TLR4	chr9	120476222/C//T	nonsynonymous	2	0
MYBPC1	chr12	102067281/C//G	nonsynonymous	2	0
MYBPC1	chr12	102067351/C//T	synonymous	2	0
CIT	chr12	120151997/C//A	synonymous	2	0
CIT	chr12	120152075/C//T	synonymous	2	0
CIT	chr12	120152080/C//T	nonsynonymous	2	0
CIT	chr12	120156153/C//T	synonymous	2	0
NDC80	chr18	2608742/C//T	nonsynonymous	2	0
CEP192	chr18	13018531/C//G	nonsynonymous	2	0
CHST9	chr18	24496723/A//G	nonsynonymous	2	0
LOXHD1	chr18	44057420/G//A	synonymous	2	0
PORCN	chrX	48372705/C//T	nonsynonymous	2	0
ASAP3	chr1	23759684/C//T	nonsynonymous	0	2
GRIK3	chr1	37324731/C//T	nonsynonymous	0	2
PATJ	chr1	62593737/G//A	nonsynonymous	0	2
NPR1	chr1	153661554/T//A	nonsynonymous	0	2
ATP8B2	chr1	154304153/C//T	nonsynonymous	0	2
CACNA1E	chr1	181765941/C//T	nonsynonymous	0	2
OBSCN	chr1	228528511/C//T	synonymous	0	2
RYR2	chr1	237586435/C//T	nonsynonymous	0	2
HEATR5B	chr2	37247902/G//A	nonsynonymous	0	2
UGGT1	chr2	128886756/G//A	synonymous	0	2
TTN	chr2	179579734/C//T	nonsynonymous	0	2
ITIH1	chr3	52825525/C//G	synonymous	0	2
PCDH10	chr4	134072546/C//A	synonymous	0	2
PCDH18	chr4	138451527/G//A	synonymous	0	2
MICB	chr6	31473408/C//T	nonsynonymous	0	2
MCM3	chr6	52133903/T//C	nonsynonymous	0	2
CSMD1	chr8	3267117/C//A	nonsynonymous	0	2
CACNA1B	chr9	140972618/C//T	nonsynonymous	0	2
CREM	chr10	35477252/C//T	synonymous	0	2
CTR9	chr11	10800351/G//A	nonsynonymous	0	2
SLC6A5	chr11	20673889/C//T	nonsynonymous	0	2
SLC22A12	chr11	64361032/G//A	nonsynonymous	0	2
RELT	chr11	73105561/G//A	synonymous	0	2
GRIK4	chr11	120837973/T//C	nonsynonymous	0	2
TMEM52B	chr12	10339095/C//T	nonsynonymous	0	2
H1FNT	chr12	48723366/G//A	nonsynonymous	0	2
METTL1	chr12	58163609/G//C	synonymous	0	2
MGAT4C	chr12	86373360/C//A	nonsynonymous	0	2
ANKS1B	chr12	99194813/C//A	nonsynonymous	0	2
COL4A2	chr13	111082263/A//G	nonsynonymous	0	2
INSM2	chr14	36003457/C//A	synonymous	0	2
TGFB3	chr14	76427303/C//G	nonsynonymous	0	2
UNC13C	chr15	54542602/C//T	synonymous	0	2
SCAMP5	chr15	75304135/C//T	synonymous	0	2
GAS7	chr17	9846527/G//A	synonymous	0	2
TTLL6	chr17	46865256/C//T	synonymous	0	2
AXIN2	chr17	63554326/T//C	nonsynonymous	0	2
CSNK1D	chr17	80207436/C//A	nonsynonymous	0	2
TMEM259	chr19	1011178/G//C	nonsynonymous	0	2
ZNF729	chr19	22498010/G//A	synonymous	0	2
PRR12	chr19	50100861/C//A	nonsynonymous	0	2
COL6A2	chr21	47552424/G//A	synonymous	0	2
SBF1	chr22	50885850/C//T	synonymous	0	2
ZNF41	chrX	47307035/A//C	nonsynonymous	0	2
CXorf56	chrX	118673642/C//T	synonymous	0	2
RAP1GAP	chr1	21934834/G//A	nonsynonymous	0	2
DMRTA2	chr1	50885051/C//G	synonymous	0	2
HIST2H2AB	chr1	149859170/C//T	synonymous	0	2
HIST2H2AB	chr1	149859172/C//T	nonsynonymous	0	2
BCAN	chr1	156622488/C//T	synonymous	0	2
LRRC52	chr1	165514161/G//A	synonymous	0	2
CRB1	chr1	197396761/G//A	nonsynonymous	0	2
GNPAT	chr1	231396396/G//A	synonymous	0	2
FMN2	chr1	240370270/G//A	nonsynonymous	0	2
HNRNPU	chr1	245022635/A//C	nonsynonymous	0	2
TEKT4	chr2	95541339/C//T	nonsynonymous	0	2
FBLN7	chr2	112944752/G//T	nonsynonymous	0	2

SLC35F5	chr2	114508060/A//G	nonsynonymous	0	2
THSD7B	chr2	137928507/C//T	synonymous	0	2
CERS6	chr2	169404125/G//A	nonsynonymous	0	2
KIF1A	chr2	241697895/G//A	nonsynonymous	0	2
TMEM43	chr3	14183314/C//T	synonymous	0	2
DCLK3	chr3	36779958/C//T	nonsynonymous	0	2
ITGA9	chr3	37550054/A//G	nonsynonymous	0	2
DENND6A	chr3	57678692/G//C	nonsynonymous	0	2
CLCN2	chr3	184071513/G//A	nonsynonymous	0	2
ATP8A1	chr4	42445623/G//A	synonymous	0	2
TACR3	chr4	104640933/G//A	synonymous	0	2
GRIA2	chr4	158262484/C//T	nonsynonymous	0	2
TLL1	chr4	166960527/A//G	nonsynonymous	0	2
POLK	chr5	74842927/G//T	nonsynonymous	0	2
RAPGEF6	chr5	130799835/A//G	synonymous	0	2
PCDHAC1	chr5	140307102/G//A	nonsynonymous	0	2
PCDHGA2	chr5	140718644/C//T	nonsynonymous	0	2
PCDHGA8	chr5	140774357/G//A	synonymous	0	2
GPRIN1	chr5	176025721/C//T	nonsynonymous	0	2
B3GAT2	chr6	71665713/C//T	synonymous	0	2
COL10A1	chr6	116442979/C//T	synonymous	0	2
DSE	chr6	116754714/G//C	nonsynonymous	0	2
ROS1	chr6	117725598/A//G	synonymous	0	2
TTLL2	chr6	167738715/G//A	synonymous	0	2
GLI3	chr7	42004686/C//T	nonsynonymous	0	2
ZNF479	chr7	57188092/T//C	nonsynonymous	0	2
ZAN	chr7	100390034/G//A	nonsynonymous	0	2
LRGUK	chr7	133884009/G//A	nonsynonymous	0	2
SSPO	chr7	149489292/C//T	nonsynonymous	0	2
YTHDF3	chr8	64099205/G//T	nonsynonymous	0	2
PKHD1L1	chr8	110457746/G//A	nonsynonymous	0	2
CPSF1	chr8	145619510/G//A	synonymous	0	2
SARDH	chr9	136573472/G//A	synonymous	0	2
FBXO18	chr10	5966282/G//A	nonsynonymous	0	2
KIAA1462	chr10	30318130/C//A	nonsynonymous	0	2
PANK1	chr10	91371689/G//T	synonymous	0	2
ITPRIP	chr10	106075620/C//T	nonsynonymous	0	2
BRSK2	chr11	1464831/G//A	nonsynonymous	0	2
ANO5	chr11	22272296/C//A	synonymous	0	2
LRTOMT	chr11	71819891/C//T	nonsynonymous	0	2
DYNC2H1	chr11	103027374/G//T	nonsynonymous	0	2
POU2F3	chr11	120117184/G//A	nonsynonymous	0	2
TECTA	chr11	120996079/C//A	synonymous	0	2
B3GAT1	chr11	134253894/C//T	nonsynonymous	0	2
CHD4	chr12	6702280/G//A	nonsynonymous	0	2
TMEM52B	chr12	10332173/G//A	synonymous	0	2
BCAT1	chr12	24995151/C//T	nonsynonymous	0	2
KIF21A	chr12	39730911/T//G	nonsynonymous	0	2
PUS7L	chr12	44124189/T//A	nonsynonymous	0	2
TMPRSS12	chr12	51237641/G//A	synonymous	0	2
NCKAP1L	chr12	54903701/C//T	nonsynonymous	0	2
NAV3	chr12	78512043/G//T	nonsynonymous	0	2
MYF6	chr12	81102787/G//A	synonymous	0	2
GALNT4	chr12	89918364/A//T	synonymous	0	2
UBE3B	chr12	109964182/T//A	nonsynonymous	0	2
UBE3B	chr12	109964184/C//G	nonsynonymous	0	2
OAS2	chr12	113444352/C//T	nonsynonymous	0	2
CFAP73	chr12	113593100/G//A	synonymous	0	2
NCOR2	chr12	124824955/C//T	synonymous	0	2
MTUS2	chr13	29600313/G//A	nonsynonymous	0	2
STOML3	chr13	39550715/C//T	nonsynonymous	0	2
SIPA1L1	chr14	72190597/C//A	nonsynonymous	0	2
SPTLC2	chr14	77978689/G//A	nonsynonymous	0	2
STON2	chr14	81743739/C//A	nonsynonymous	0	2
EML5	chr14	89206753/C//T	nonsynonymous	0	2
BAHD1	chr15	40758204/C//T	nonsynonymous	0	2
CHRNB4	chr15	78921579/C//T	synonymous	0	2
BNC1	chr15	83926317/C//T	synonymous	0	2
DECR2	chr16	461021/C//T	synonymous	0	2
TBL3	chr16	2024412/C//T	synonymous	0	2

DCUN1D3	chr16	20871597/CTT//C	nonsynonymous	0	2
MYLPF	chr16	30387520/C//T	nonsynonymous	0	2
CES1	chr16	55866969/G//A	synonymous	0	2
MT1E	chr16	56659717/G//C	synonymous	0	2
CNGB1	chr16	57993927/G//T	nonsynonymous	0	2
IRF8	chr16	85952175/T//C	nonsynonymous	0	2
CBFA2T3	chr16	88967911/C//T	nonsynonymous	0	2
LOXHD1	chr18	44102168/G//A	nonsynonymous	0	2
MBD1	chr18	47800143/G//A	nonsynonymous	0	2
CBLN2	chr18	70209125/C//T	nonsynonymous	0	2
PNPLA6	chr19	7600391/C//T	synonymous	0	2
OLFM2	chr19	9965354/G//T	nonsynonymous	0	2
CHERP	chr19	16631213/C//T	synonymous	0	2
TMEM59L	chr19	18727860/C//T	synonymous	0	2
SRRM5	chr19	44118015/G//A	nonsynonymous	0	2
BBC3	chr19	47729925/G//A	nonsynonymous	0	2
ZNF765	chr19	53911775/G//A	nonsynonymous	0	2
KIR3DL2	chr19	55378169/C//A	nonsynonymous	0	2
SOX12	chr20	307141/G//T	synonymous	0	2
SIGLEC1	chr20	3678574/C//G	nonsynonymous	0	2
BPIFB2	chr20	31603251/C//T	synonymous	0	2
BLCAP	chr20	36147288/C//T	synonymous	0	2
ZNF335	chr20	44586537/A//G	nonsynonymous	0	2
GNAS	chr20	57428567/C//A	nonsynonymous	0	2
TCF20	chr22	42607920/C//CT	nonsynonymous	0	2
STS	chrX	7268248/C//T	synonymous	0	2
NHS	chrX	17393902/G//A	nonsynonymous	0	2
RBM10	chrX	47038832/G//A	nonsynonymous	0	2
MTMR8	chrX	63551614/G//C	nonsynonymous	0	2
MAGEC1	chrX	140995993/C//G	nonsynonymous	0	2
MAGEA5	chrX	151284015/C//T	synonymous	0	2
AADACL3	chr1	12780908/C//T	nonsynonymous	0	2
HSPG2	chr1	22173979/C//T	nonsynonymous	0	2
DENND2C	chr1	115168392/C//G	nonsynonymous	0	2
JTB	chr1	153947137/T//C	synonymous	0	2
MNDA	chr1	158817521/A//C	nonsynonymous	0	2
CEP350	chr1	179983081/A//C	nonsynonymous	0	2
TP53BP2	chr1	223983855/G//C	nonsynonymous	0	2
CD207	chr2	71062848/G//A	nonsynonymous	0	2
LIMS1	chr2	109300538/C//T	synonymous	0	2
LY75-CD302	chr2	160706506/C//T	synonymous	0	2
LRP2	chr2	170009329/T//G	synonymous	0	2
TTN	chr2	179641109/G//C	nonsynonymous	0	2
COL5A2	chr2	189933587/C//T	synonymous	0	2
ITGA9	chr3	37583996/G//C	nonsynonymous	0	2
HYAL3	chr3	50331102/G//A	synonymous	0	2
EIF4A2	chr3	186506949/T//A	nonsynonymous	0	2
CWH43	chr4	48988363/C//T	synonymous	0	2
CCSER1	chr4	91229688/C//G	nonsynonymous	0	2
SEC24A	chr5	134039502/C//T	synonymous	0	2
EGR1	chr5	137803329/C//T	synonymous	0	2
LARP1	chr5	154181737/G//A	synonymous	0	2
DOCK2	chr5	169469134/A//G	nonsynonymous	0	2
EGFL8	chr6	32134489/G//A	nonsynonymous	0	2
CDK19	chr6	110942342/T//C	nonsynonymous	0	2
HDAC2	chr6	114274585/G//A	synonymous	0	2
ARL4A	chr7	12727831/C//T	synonymous	0	2
DNAH11	chr7	21923982/G//A	nonsynonymous	0	2
LRCH4	chr7	100174570/C//T	nonsynonymous	0	2
KIAA1549	chr7	138579169/G//T	synonymous	0	2
CNTNAP2	chr7	146997331/C//T	nonsynonymous	0	2
RP1L1	chr8	10470181/G//A	nonsynonymous	0	2
MRPL13	chr8	121408351/G//A	nonsynonymous	0	2
FAM135B	chr8	139164502/A//G	nonsynonymous	0	2
RIC1	chr9	5629361/C//G	nonsynonymous	0	2
ASPN	chr9	95228860/C//G	synonymous	0	2
ZNF25	chr10	38242118/C//G	nonsynonymous	0	2
PNLIPRP2	chr10	118385579/C//T	synonymous	0	2
RRM1	chr11	4156442/A//G	nonsynonymous	0	2
OR51G2	chr11	4936129/G//A	synonymous	0	2

WEE1	chr11	9595518/G//A	nonsynonymous	0	2
RRAS2	chr11	14316390/T//A	nonsynonymous	0	2
OR8H1	chr11	56058411/A//T	nonsynonymous	0	2
NRXN2	chr11	64415794/G//A	synonymous	0	2
LRP5	chr11	68216514/G//A	synonymous	0	2
DEFB108B	chr11	71548514/A//G	nonsynonymous	0	2
TIGAR	chr12	4446274/A//G	nonsynonymous	0	2
KLRG1	chr12	9162114/G//T	nonsynonymous	0	2
FMN1	chr15	33192235/T//C	nonsynonymous	0	2
LMF1	chr16	920817/C//T	nonsynonymous	0	2
CREBBP	chr16	3801752/C//G	nonsynonymous	0	2
ADAMTS18	chr16	77387786/G//T	synonymous	0	2
XAF1	chr17	6659334/T//G	synonymous	0	2
HOXB2	chr17	46620414/G//A	synonymous	0	2
WBP2	chr17	73843589/C//T	nonsynonymous	0	2
ASXL3	chr18	31323977/A//G	nonsynonymous	0	2
SERPINB11	chr18	61390453/C//A	nonsynonymous	0	2
HAS1	chr19	52217145/G//A	synonymous	0	2
SS18L1	chr20	60737958/G//A	nonsynonymous	0	2
ZC3H7B	chr22	41744093/G//A	nonsynonymous	0	2
ASB11	chrX	15315747/G//C	nonsynonymous	0	2
AMER1	chrX	63412600/T//C	synonymous	0	2
GJB1	chrX	70444389/G//A	nonsynonymous	0	2
DCAF12L1	chrX	125685239/A//C	synonymous	0	2
ITGA8	chr10	15559199/G//A	synonymous	2	0
THEM4	chr1	151849539/T//G	nonsynonymous	0	2
UBR3	chr2	170783230/A//G	nonsynonymous	0	2
GPR155	chr2	175337925/C//T	nonsynonymous	0	2
TTN	chr2	179434345/A//C	nonsynonymous	0	2
ATP13A4	chr3	193128778/C//T	nonsynonymous	0	2
TMEM150C	chr4	83411273/C//T	nonsynonymous	0	2
LOC100288152	chr5	475371/C//T	synonymous	0	2
ADGRV1	chr5	90040872/T//A	nonsynonymous	0	2
APC	chr5	112174905/GC//G	nonsynonymous	0	2
HLA-DRA	chr6	32411144/C//T	nonsynonymous	0	2
TAP2	chr6	32797287/C//A	nonsynonymous	0	2
DBF4	chr7	87537189/A//C	nonsynonymous	0	2
SETX	chr9	135203354/G//A	nonsynonymous	0	2
FBXL15	chr10	104180858/T//A	synonymous	0	2
LRP5	chr11	68192598/C//T	nonsynonymous	0	2
EMSY	chr11	76227234/G//A	nonsynonymous	0	2
ETFBKMT	chr12	31814952/G//A	nonsynonymous	0	2
DDN	chr12	49391638/C//T	nonsynonymous	0	2
PPP2R5C	chr14	102378789/T//C	synonymous	0	2
GOT2	chr16	58743459/C//A	synonymous	0	2
SCARF1	chr17	1538599/G//A	nonsynonymous	0	2
ATAD5	chr17	29187506/A//T	nonsynonymous	0	2
CRHR1	chr17	43906964/G//A	nonsynonymous	0	2
RNASEH2A	chr19	12924401/G//A	synonymous	0	2
ZSCAN1	chr19	58565347/C//T	synonymous	0	2
CSTL1	chr20	23425414/A//C	nonsynonymous	0	2
JPH2	chr20	42815301/G//A	synonymous	0	2
ZNF831	chr20	57768129/C//T	synonymous	0	2
VSIG1	chrX	107310317/C//T	nonsynonymous	0	2
ADGRG4	chrX	135475738/TAGTA//T	synonymous	0	2
ZNF91	chr19	23543393/C//T	synonymous	1	1
	chr5	58295378/A//G	synonymous	1	1
ACP6	chr1	147120168/G//C	synonymous	0	2
MSH6	chr2	48026865/A//C	nonsynonymous	0	2
IMPG2	chr3	100951717/T//C	synonymous	0	2
ITGB5	chr3	124492625/A//T	nonsynonymous	0	2
TMEM14EP	chr3	152058325/C//G	synonymous	0	2
ZSCAN25	chr7	99227024/A//G	nonsynonymous	0	2
PNPLA7	chr9	140361788/C//T	nonsynonymous	0	2
ITGB1	chr10	33200888/T//C	nonsynonymous	0	2
HCFC2	chr12	104489320/A//G	nonsynonymous	0	2
DACH1	chr13	72063208/G//C	nonsynonymous	0	2
MINK1	chr17	4791066/A//G	nonsynonymous	0	2
ADORA2B	chr17	15878541/G//A	nonsynonymous	0	2
SLC16A2	chrX	73744207/C//T	nonsynonymous	0	2

MUC5B	chr11	1269835/G//A	nonsynonymous	1	1
SLC2A5	chr1	9100037/G//A	nonsynonymous	0	2
CSMD2	chr1	34071032/C//A	nonsynonymous	0	2
TMEM53	chr1	45120287/G//A	nonsynonymous	0	2
MKNK1	chr1	47059780/C//G	synonymous	0	2
NPR1	chr1	153659829/G//A	nonsynonymous	0	2
FCRL4	chr1	157545354/T//G	nonsynonymous	0	2
TPO	chr2	1457562/C//A	synonymous	0	2
SOX11	chr2	5833402/C//A	synonymous	0	2
APOB	chr2	21234034/C//T	synonymous	0	2
ALK	chr2	29436871/T//A	nonsynonymous	0	2
COX7A2L	chr2	42580398/T//A	nonsynonymous	0	2
NRXN1	chr2	50779958/C//T	nonsynonymous	0	2
SLC4A10	chr2	162813669/C//T	synonymous	0	2
SCN3A	chr2	165952159/C//G	nonsynonymous	0	2
CWC22	chr2	180830642/C//A	nonsynonymous	0	2
ZNF804A	chr2	185801540/C//G	nonsynonymous	0	2
DNAH7	chr2	196682523/C//A	nonsynonymous	0	2
PLCL1	chr2	198949860/A//T	nonsynonymous	0	2
GRM7	chr3	7620980/C//T	nonsynonymous	0	2
PRRT3	chr3	9990781/C//G	synonymous	0	2
WNT5A	chr3	55513444/C//A	nonsynonymous	0	2
CASR	chr3	121994736/G//A	synonymous	0	2
PIK3R4	chr3	130425975/T//A	nonsynonymous	0	2
PIK3CB	chr3	138403578/C//T	nonsynonymous	0	2
HPS3	chr3	148872972/G//A	synonymous	0	2
PCDH7	chr4	30725886/T//G	nonsynonymous	0	2
GABRA2	chr4	46307639/G//T	nonsynonymous	0	2
KIT	chr4	55598083/C//T	synonymous	0	2
PDLIM5	chr4	95561456/G//A	nonsynonymous	0	2
MTTP	chr4	100530028/A//G	nonsynonymous	0	2
UGT3A2	chr5	36039725/G//A	nonsynonymous	0	2
ANKHD1	chr5	139876627/A//G	nonsynonymous	0	2
TAPBP	chr6	33272828/T//A	nonsynonymous	0	2
KCNK16	chr6	39284616/C//T	synonymous	0	2
RIPPLY2	chr6	84567190/A//C	synonymous	0	2
CLVS2	chr6	123332274/T//C	synonymous	0	2
PLEKHG1	chr6	151152350/C//T	synonymous	0	2
FNDC1	chr6	159654059/C//T	nonsynonymous	0	2
TMEM184A	chr7	1587403/G//A	synonymous	0	2
BBS9	chr7	33644866/A//C	synonymous	0	2
GLI3	chr7	42116391/G//A	nonsynonymous	0	2
STYXL1	chr7	75633169/C//A	nonsynonymous	0	2
MAGI2	chr7	77649177/C//A	nonsynonymous	0	2
SLC35B4	chr7	133985017/A//T	synonymous	0	2
ZNF212	chr7	148951454/G//A	nonsynonymous	0	2
ZDHHC2	chr8	17014363/G//A	nonsynonymous	0	2
ZBTB10	chr8	81411760/G//A	nonsynonymous	0	2
ATAD2	chr8	124408638/G//T	synonymous	0	2
ADGRB1	chr8	143623483/C//T	synonymous	0	2
VLDLR	chr9	2648762/A//AATG	nonsynonymous	0	2
DENND4C	chr9	19298088/C//G	nonsynonymous	0	2
PHF2	chr9	96392283/G//A	nonsynonymous	0	2
INVS	chr9	103054690/A//G	synonymous	0	2
DNAJC25	chr9	114409525/A//G	nonsynonymous	0	2
CNTRL	chr9	123920255/T//G	synonymous	0	2
CDH23	chr10	73567085/G//A	nonsynonymous	0	2
TLL2	chr10	98156991/G//A	nonsynonymous	0	2
CRTAC1	chr10	99644052/G//A	nonsynonymous	0	2
OR56A5	chr11	5989467/G//T	synonymous	0	2
RBMXL2	chr11	7110710/G//A	nonsynonymous	0	2
SAC3D1	chr11	64811905/C//T	synonymous	0	2
PITPNM1	chr11	67266278/C//T	nonsynonymous	0	2
COLCA2	chr11	111179125/A//AC	nonsynonymous	0	2
TMPRSS4	chr11	117975524/G//T	nonsynonymous	0	2
MFRP	chr11	119216505/G//A	synonymous	0	2
CDON	chr11	125889541/G//A	nonsynonymous	0	2
HOXC12	chr12	54349055/C//T	synonymous	0	2
ERBB3	chr12	56493467/A//G	nonsynonymous	0	2
APOF	chr12	56755430/G//T	nonsynonymous	0	2

ATP6V1D	chr14	67812546/G//C	nonsynonymous	0	2
PDCD7	chr15	65421421/G//A	nonsynonymous	0	2
CLCN7	chr16	1501651/C//T	nonsynonymous	0	2
CRAMP1	chr16	1720713/T//C	nonsynonymous	0	2
UBN1	chr16	4924674/C//A	nonsynonymous	0	2
RBFOX1	chr16	7568246/C//T	nonsynonymous	0	2
HERPUD1	chr16	56977104/G//A	nonsynonymous	0	2
TP53	chr17	7577556/C//A	nonsynonymous	0	2
CCDC144NL	chr17	20769896/G//T	nonsynonymous	0	2
IGF2BP1	chr17	47115724/G//A	nonsynonymous	0	2
COL1A1	chr17	48278906/G//A	synonymous	0	2
KDM4B	chr19	5135544/C//T	synonymous	0	2
F2RL3	chr19	17000917/C//T	nonsynonymous	0	2
ZNF429	chr19	21720592/C//T	synonymous	0	2
JAG1	chr20	10654282/C//T	synonymous	0	2
OSER1	chr20	42826029/T//C	nonsynonymous	0	2
ARFGEF2	chr20	47626798/G//A	nonsynonymous	0	2
KLHL34	chrX	21675315/C//A	nonsynonymous	0	2
PHKA1	chrX	71856166/G//C	synonymous	0	2
PCDH9	chr13	67799594/G//T	synonymous	0	2
PIGT	chr20	44047976/G//C	nonsynonymous	0	2
RSPH1	chr21	43913111/C//T	nonsynonymous	0	2
SPAST	chr2	32379522/C//A	nonsynonymous	0	2
CXCR4	chr2	136873174/T//C	synonymous	0	2
BCL6	chr3	187440347/G//A	nonsynonymous	0	2
PRDM9	chr5	23509680/C//A	nonsynonymous	0	2
CAPSL	chr5	35910537/T//G	nonsynonymous	0	2
CUX1	chr7	101821870/G//A	nonsynonymous	0	2
CPA4	chr7	129938672/C//T	synonymous	0	2
HTRA4	chr8	38832000/C//T	nonsynonymous	0	2
POTEA	chr8	43159856/T//A	nonsynonymous	0	2
BTBD16	chr10	124091981/A//G	nonsynonymous	0	2
ERN2	chr16	23716404/C//T	synonymous	0	2
CLTC	chr17	57743971/G//A	nonsynonymous	0	2
ANKRD30B	chr18	14799236/G//A	synonymous	0	2
PLPPR3	chr19	813060/G//A	nonsynonymous	0	2
KLK6	chr19	51462454/G//A	nonsynonymous	0	2
SUN2	chr22	39138431/G//A	nonsynonymous	0	2
ELK1	chrX	47500745/A//G	synonymous	0	2
PDE6B	chr4	629753/G//A	nonsynonymous	0	2
KLF3	chr4	38690317/A//T	nonsynonymous	0	2
HADH	chr4	108955528/G//A	synonymous	0	2
OXCT1	chr5	41870549/G//A	synonymous	0	2
FER	chr5	108516573/A//G	nonsynonymous	0	2
APC	chr5	112175207/G//T	nonsynonymous	0	2
CACNA2D1	chr7	81601133/T//C	nonsynonymous	0	2
CCKBR	chr11	6291485/G//A	nonsynonymous	0	2
LPCAT4	chr15	34652309/T//C	synonymous	0	2
TELO2	chr16	1552070/G//A	nonsynonymous	0	2
MVP	chr16	29845144/G//T	nonsynonymous	0	2
NUDC	chr1	27268066/A//G	nonsynonymous	0	2
ZBTB8B	chr1	32936869/C//T	nonsynonymous	0	2
ADORA3	chr1	112043018/C//T	nonsynonymous	0	2
CD58	chr1	117086952/C//T	synonymous	0	2
FLG	chr1	152286419/C//A	nonsynonymous	0	2
PROX1	chr1	214171387/C//T	synonymous	0	2
SNAP47	chr1	227935417/C//T	nonsynonymous	0	2
NBAS	chr2	15608557/C//T	nonsynonymous	0	2
KLHL29	chr2	23785133/G//A	nonsynonymous	0	2
ADCY3	chr2	25141311/C//T	synonymous	0	2
ADGRF3	chr2	26541791/G//A	nonsynonymous	0	2
LRP1B	chr2	141215226/A//G	synonymous	0	2
UNC80	chr2	210769562/T//A	nonsynonymous	0	2
FAM198A	chr3	43074964/C//T	synonymous	0	2
PRSS42	chr3	46875371/C//T	nonsynonymous	0	2
GLYCTK	chr3	52324719/G//C	nonsynonymous	0	2
FAM3D	chr3	58622103/G//A	nonsynonymous	0	2
MINA	chr3	97666283/T//C	synonymous	0	2
IMPG2	chr3	100948296/G//A	synonymous	0	2
PARP9	chr3	122274384/G//T	nonsynonymous	0	2

EPHB1	chr3	134960061/C//T	synonymous	0	2
NPFFR2	chr4	73013287/C//T	nonsynonymous	0	2
PDHA2	chr4	96762158/G//A	nonsynonymous	0	2
TBC1D9	chr4	141543544/G//T	nonsynonymous	0	2
TENM3	chr4	183713754/C//T	synonymous	0	2
SRD5A1	chr5	6652042/C//T	synonymous	0	2
TRIO	chr5	14316767/G//A	nonsynonymous	0	2
YTHDC2	chr5	112901657/C//G	nonsynonymous	0	2
	chr5	114632120/T//G	synonymous	0	2
PCDHA2	chr5	140176640/C//T	synonymous	0	2
	chr5	146728358/C//T	synonymous	0	2
HK3	chr5	176314293/C//T	nonsynonymous	0	2
ADAMTS2	chr5	178562961/C//T	synonymous	0	2
PRL	chr6	22297215/C//T	synonymous	0	2
POM121L2	chr6	27279113/G//A	synonymous	0	2
DHX16	chr6	30624192/G//A	synonymous	0	2
TBC1D22B	chr6	37250092/C//T	nonsynonymous	0	2
KCNK17	chr6	39271757/C//T	nonsynonymous	0	2
RSPH9	chr6	43612811/G//T	synonymous	0	2
RIMS1	chr6	72960935/G//A	synonymous	0	2
ADGB	chr6	147106810/C//T	nonsynonymous	0	2
AMZ1	chr7	2740104/G//A	nonsynonymous	0	2
WIPF3	chr7	29928974/G//A	synonymous	0	2
AMPH	chr7	38543261/G//T	nonsynonymous	0	2
PKD1L1	chr7	47874739/G//T	nonsynonymous	0	2
GNAI1	chr7	79764428/C//A	synonymous	0	2
SAMD9	chr7	92735052/G//T	nonsynonymous	0	2
PENK	chr8	57354307/A//G	nonsynonymous	0	2
UBR5	chr8	103282298/C//A	nonsynonymous	0	2
PKHD1L1	chr8	110445349/G//A	nonsynonymous	0	2
LHX6	chr9	124979499/C//T	nonsynonymous	0	2
TLX1	chr10	102894092/G//A	synonymous	0	2
FANK1	chr10	127697794/G//A	nonsynonymous	0	2
OR52N1	chr11	5809405/C//T	synonymous	0	2
TRIM66	chr11	8670079/G//A	synonymous	0	2
ABTB2	chr11	34218942/G//A	nonsynonymous	0	2
CHST1	chr11	45672089/C//T	nonsynonymous	0	2
OR4A5	chr11	51412094/A//G	nonsynonymous	0	2
SLC43A3	chr11	57182516/C//T	nonsynonymous	0	2
DDB1	chr11	61097512/G//A	nonsynonymous	0	2
KDM2A	chr11	67021716/G//A	nonsynonymous	0	2
FAT3	chr11	92615929/G//A	nonsynonymous	0	2
FAT3	chr11	92616282/C//T	synonymous	0	2
ETV6	chr12	12038862/C//T	synonymous	0	2
KMT2D	chr12	49432374/C//T	nonsynonymous	0	2
MBD6	chr12	57918174/C//T	nonsynonymous	0	2
CUX2	chr12	111758427/G//A	nonsynonymous	0	2
HECTD4	chr12	112638577/G//A	nonsynonymous	0	2
SRRM4	chr12	119591395/G//A	synonymous	0	2
HOMEZ	chr14	23746402/A//G	synonymous	0	2
FANCM	chr14	45636243/C//T	nonsynonymous	0	2
ESRRB	chr14	76948385/G//A	nonsynonymous	0	2
C14orf177	chr14	99183533/G//A	synonymous	0	2
CDC42BPB	chr14	103452870/C//T	nonsynonymous	0	2
KIF26A	chr14	104643443/C//T	nonsynonymous	0	2
ADAMTS7	chr15	79056391/C//T	synonymous	0	2
TM2D3	chr15	102187102/C//A	nonsynonymous	0	2
PKD1	chr16	2162830/C//T	synonymous	0	2
ANKS3	chr16	4777146/C//T	nonsynonymous	0	2
ACSM2A	chr16	20494386/A//G	nonsynonymous	0	2
HIC1	chr17	1961343/G//A	synonymous	0	2
KRTAP9-8	chr17	39394729/C//A	synonymous	0	2
KRT31	chr17	39551196/C//T	nonsynonymous	0	2
ITGA3	chr17	48158698/C//T	nonsynonymous	0	2
KIF19	chr17	72344009/G//A	nonsynonymous	0	2
ANKRD24	chr19	4207949/C//A	synonymous	0	2
ADAMTS10	chr19	8665945/C//T	nonsynonymous	0	2
HAPLN4	chr19	19369541/C//T	nonsynonymous	0	2
PSG8	chr19	43268101/C//G	nonsynonymous	0	2
CCDC9	chr19	47774873/C//T	nonsynonymous	0	2

ZNF551	chr19	58199242/C//T	synonymous	0	2
NCOA6	chr20	33338036/C//T	synonymous	0	2
SALL4	chr20	50405552/G//A	nonsynonymous	0	2
NELFCD	chr20	57568575/C//G	nonsynonymous	0	2
LAMA5	chr20	60888729/G//A	synonymous	0	2
TMPRSS15	chr21	19744536/C//T	nonsynonymous	0	2
G6PD	chrX	153761236/G//A	synonymous	0	2
SNX20	chr16	50707501/C//T	nonsynonymous	0	2
CHD5	chr1	6196810/C//T	nonsynonymous	0	2
TTN	chr2	179404285/C//T	nonsynonymous	0	2
RAB17	chr2	238484117/T//C	nonsynonymous	0	2
RACK1	chr5	180664719/C//T	nonsynonymous	0	2
CUL9	chr6	43152296/G//A	nonsynonymous	0	2
COBL	chr7	51092803/C//T	synonymous	0	2
DYNC1I1	chr7	95668627/G//A	nonsynonymous	0	2
ZNF800	chr7	127017360/A//C	nonsynonymous	0	2
APBB1	chr11	6424589/GTTCC//G	nonsynonymous	0	2
ZNF839	chr14	102793047/C//T	synonymous	0	2
ADAMTS7	chr15	79054835/C//T	nonsynonymous	0	2
SPATA8	chr15	97326860/G//T	synonymous	0	2
NARFL	chr16	780891/G//A	nonsynonymous	0	2
BRD7	chr16	50353064/T//A	synonymous	0	2
BRD7	chr16	50357579/A//C	nonsynonymous	0	2
CBFA2T3	chr16	88947724/G//A	synonymous	0	2
SPDYE4	chr17	8661684/G//A	nonsynonymous	0	2
ZNF444	chr19	56671341/G//T	nonsynonymous	0	2
IGSF21	chr1	18692039/G//A	nonsynonymous	0	2
IGSF21	chr1	18692064/C//T	synonymous	0	2
UBR4	chr1	19441439/T//C	nonsynonymous	0	2
LRRC41	chr1	46751567/C//T	nonsynonymous	0	2
WDR47	chr1	109517338/G//A	nonsynonymous	0	2
ALX3	chr1	110613093/G//A	synonymous	0	2
AMPD1	chr1	115220069/G//A	nonsynonymous	0	2
CD101	chr1	117561057/C//A	nonsynonymous	0	2
BRINP3	chr1	190067182/G//T	nonsynonymous	0	2
B3GALT2	chr1	193150445/C//G	nonsynonymous	0	2
KDM5B	chr1	202742324/T//G	synonymous	0	2
USH2A	chr1	215987195/C//T	nonsynonymous	0	2
ESRRG	chr1	216850601/C//A	nonsynonymous	0	2
TPO	chr2	1507848/G//A	nonsynonymous	0	2
PPP1R21	chr2	48698330/A//T	synonymous	0	2
MBD5	chr2	149225950/A//G	synonymous	0	2
PKP4	chr2	159499208/A//T	nonsynonymous	0	2
FSIP2	chr2	186669608/A//G	nonsynonymous	0	2
TNS1	chr2	218750822/G//A	nonsynonymous	0	2
EPHA3	chr3	89445005/C//T	nonsynonymous	0	2
GPR15	chr3	98251720/G//T	nonsynonymous	0	2
ARHGAP31	chr3	119121047/C//T	nonsynonymous	0	2
MED12L	chr3	150883648/G//A	nonsynonymous	0	2
KCNAB1	chr3	155838523/C//T	synonymous	0	2
LEKR1	chr3	156763371/C//T	synonymous	0	2
FRYL	chr4	48530060/T//C	synonymous	0	2
EPHA5	chr4	66535467/C//T	synonymous	0	2
UNC5C	chr4	96090536/A//G	nonsynonymous	0	2
FAT4	chr4	126371717/A//T	synonymous	0	2
	chr4	155411154/C//A	synonymous	0	2
RWDD4	chr4	184562624/A//G	nonsynonymous	0	2
F11	chr4	187197491/C//T	synonymous	0	2
LIFR	chr5	38484884/G//A	nonsynonymous	0	2
HCN1	chr5	45645392/G//T	nonsynonymous	0	2
BDP1	chr5	70806438/G//C	nonsynonymous	0	2
APC	chr5	112174177/TA//T	nonsynonymous	0	2
PCDHA3	chr5	140182318/G//A	synonymous	0	2
PCDHA9	chr5	140228407/C//T	synonymous	0	2
PCDHA10	chr5	140237259/G//A	synonymous	0	2
PCDHB6	chr5	140529815/C//A	synonymous	0	2
SLC6A7	chr5	149581903/G//A	synonymous	0	2
CLK4	chr5	178045718/C//T	nonsynonymous	0	2
FLT4	chr5	180048002/C//T	nonsynonymous	0	2
CUL7	chr6	43011249/G//A	nonsynonymous	0	2

DSE	chr6	116757926/G//A	synonymous	0	2
FAM162B	chr6	117086602/G//A	synonymous	0	2
SYNE1	chr6	152497562/C//T	nonsynonymous	0	2
SYNE1	chr6	152697579/G//A	synonymous	0	2
DPY19L1	chr7	34989453/A//C	nonsynonymous	0	2
GLI3	chr7	42065984/G//A	synonymous	0	2
ABCA13	chr7	48559764/T//G	nonsynonymous	0	2
CAV1	chr7	116199133/C//T	nonsynonymous	0	2
PTPRZ1	chr7	121513546/G//A	synonymous	0	2
AGBL3	chr7	134800209/G//T	nonsynonymous	0	2
OR2A12	chr7	143792935/C//T	synonymous	0	2
HTR5A	chr7	154863255/G//T	nonsynonymous	0	2
TEX15	chr8	30703039/C//A	nonsynonymous	0	2
COL14A1	chr8	121357691/C//G	nonsynonymous	0	2
TRAPPC9	chr8	141231563/C//T	nonsynonymous	0	2
ZNF7	chr8	146068289/C//A	nonsynonymous	0	2
APTX	chr9	32987592/C//T	nonsynonymous	0	2
TUBAL3	chr10	5435603/G//A	synonymous	0	2
SH2D4B	chr10	82363362/AGAG//A	nonsynonymous	0	2
CCDC172	chr10	118084602/G//T	nonsynonymous	0	2
VENTX	chr10	135053512/C//T	nonsynonymous	0	2
NDUFV1	chr11	67379444/G//A	nonsynonymous	0	2
P2RY2	chr11	72945859/G//A	nonsynonymous	0	2
FGF23	chr12	4479673/G//A	nonsynonymous	0	2
KCNA1	chr12	5020842/G//A	nonsynonymous	0	2
C1S	chr12	7177603/G//A	nonsynonymous	0	2
DDX47	chr12	12974984/C//T	synonymous	0	2
TMEM117	chr12	44605125/C//A	nonsynonymous	0	2
OR6C75	chr12	55758935/G//T	nonsynonymous	0	2
LRRIQ1	chr12	85521745/G//T	nonsynonymous	0	2
ANKS1B	chr12	100219128/C//T	synonymous	0	2
CHFR	chr12	133438211/C//A	nonsynonymous	0	2
DCLK1	chr13	36413245/G//A	synonymous	0	2
SLITRK5	chr13	88327927/G//A	nonsynonymous	0	2
VRTN	chr14	74823784/C//T	nonsynonymous	0	2
LTBP2	chr14	74970002/C//T	nonsynonymous	0	2
DYNC1H1	chr14	102502890/C//T	nonsynonymous	0	2
MGA	chr15	42028600/C//T	nonsynonymous	0	2
IGDCC3	chr15	65622066/G//A	synonymous	0	2
DPP8	chr15	65744385/G//A	nonsynonymous	0	2
FES	chr15	91428690/C//T	nonsynonymous	0	2
TARSL2	chr15	102198059/G//A	nonsynonymous	0	2
AXIN1	chr16	396719/T//C	nonsynonymous	0	2
MAPK8IP3	chr16	1797267/G//A	nonsynonymous	0	2
ZSCAN10	chr16	3140023/G//A	nonsynonymous	0	2
PLK1	chr16	23700947/G//A	nonsynonymous	0	2
IL21R	chr16	27460570/C//T	nonsynonymous	0	2
PRMT7	chr16	68373447/G//A	nonsynonymous	0	2
SSH2	chr17	28256967/G//A	nonsynonymous	0	2
SLFN11	chr17	33680060/C//T	nonsynonymous	0	2
PIGW	chr17	34894465/A//T	nonsynonymous	0	2
PHOSPHO1	chr17	47304107/G//A	synonymous	0	2
DNAH17	chr17	76462822/G//A	nonsynonymous	0	2
CBX4	chr17	77808437/A//ACC	nonsynonymous	0	2
MIB1	chr18	19438593/G//A	nonsynonymous	0	2
NETO1	chr18	70416243/G//T	synonymous	0	2
TSHZ1	chr18	72999127/G//A	nonsynonymous	0	2
IL27RA	chr19	14142779/C//T	nonsynonymous	0	2
RAB3A	chr19	18313455/G//A	synonymous	0	2
CHST8	chr19	34180099/G//A	synonymous	0	2
RBM42	chr19	36125212/C//T	nonsynonymous	0	2
EPN1	chr19	56196963/C//T	nonsynonymous	0	2
ZNF586	chr19	58281225/C//T	synonymous	0	2
ZNF8	chr19	58806380/C//T	synonymous	0	2
CST9L	chr20	23549134/G//T	synonymous	0	2
ZNF341	chr20	32379218/C//T	synonymous	0	2
PTPRT	chr20	40739064/C//T	nonsynonymous	0	2
PKNOX1	chr21	44438310/G//A	synonymous	0	2
	chr22	24226126/T//C	synonymous	0	2
SEC14L6	chr22	30925140/C//T	nonsynonymous	0	2

TCN2	chr22	31022498/T//C	nonsynonymous	0	2
MOV10L1	chr22	50555696/G//A	nonsynonymous	0	2
SBF1	chr22	50903000/G//A	synonymous	0	2
FAM9B	chrX	8995925/C//T	nonsynonymous	0	2
SHROOM2	chrX	9863072/C//T	nonsynonymous	0	2
TCEANC	chrX	13681651/C//A	nonsynonymous	0	2
NLGN3	chrX	70367694/C//T	nonsynonymous	0	2
OGT	chrX	70787871/A//T	synonymous	0	2
ARMCX5-GPRASP2	chrX	101970352/C//A	synonymous	0	2
AMOT	chrX	112024138/C//T	nonsynonymous	0	2
DCAF12L2	chrX	125298905/G//A	nonsynonymous	0	2
DCAF12L2	chrX	125299462/C//A	nonsynonymous	0	2
MAGEC2	chrX	141291741/G//A	synonymous	0	2
OPN1LW	chrX	153416302/C//T	nonsynonymous	0	2
AURKAIP1	chr1	1309158/G//A	synonymous	1	1
LRRC4C	chr11	40137642/C//T	synonymous	1	1
ZNF208	chr19	22155847/C//T	synonymous	1	1
KRTAP10-1	chr21	45959195/G//A	nonsynonymous	1	1
MUC17	chr7	100677606/G//T	nonsynonymous	1	1
DENND1B	chr1	197576309/A//T	synonymous	0	2
	chr18	51729257/A//T	synonymous	1	1
TP53	chr17	7578437/G//A	nonsynonymous	2	0
ZNF100	chr19	21909767/C//T	nonsynonymous	1	1
CORT	chr1	10510240/G//A	synonymous	0	2
MAD2L2	chr1	11740666/G//A	synonymous	0	2
PRAMEF1	chr1	12855771/C//T	nonsynonymous	0	2
PADI6	chr1	17721460/G//A	nonsynonymous	0	2
WASF2	chr1	27744798/G//A	nonsynonymous	0	2
ZMYM4	chr1	35852842/G//A	nonsynonymous	0	2
DMAP1	chr1	44684871/C//T	synonymous	0	2
ZYG11B	chr1	53279314/T//C	nonsynonymous	0	2
S1PR1	chr1	101705353/G//A	synonymous	0	2
S1PR1	chr1	101705554/C//A	synonymous	0	2
SLC6A17	chr1	110714803/T//C	synonymous	0	2
AMPD1	chr1	115220643/C//T	nonsynonymous	0	2
FLG	chr1	152281866/C//T	synonymous	0	2
C1orf68	chr1	152692474/T//C	synonymous	0	2
SPTA1	chr1	158592788/C//A	nonsynonymous	0	2
VANGL2	chr1	160385613/C//T	synonymous	0	2
OLFML2B	chr1	161970098/C//T	nonsynonymous	0	2
SELP	chr1	169582887/C//T	nonsynonymous	0	2
DHX9	chr1	182827934/C//T	nonsynonymous	0	2
RGL1	chr1	183881267/G//T	nonsynonymous	0	2
TRMT1L	chr1	185109148/T//C	nonsynonymous	0	2
KIF21B	chr1	200961481/G//A	nonsynonymous	0	2
HHIPL2	chr1	222717195/C//T	nonsynonymous	0	2
TRIM67	chr1	231351191/G//A	synonymous	0	2
RYR2	chr1	237713957/C//T	synonymous	0	2
GREM2	chr1	240656232/G//A	synonymous	0	2
RGS7	chr1	240977013/C//T	synonymous	0	2
DNMT3A	chr2	25536876/G//A	synonymous	0	2
GALNT14	chr2	31361028/G//A	synonymous	0	2
MTHFD2	chr2	74425827/G//T	nonsynonymous	0	2
CNTNAP5	chr2	125192210/G//T	nonsynonymous	0	2
LCT	chr2	136566318/G//A	nonsynonymous	0	2
LOC100507600	chr2	136579779/G//T	synonymous	0	2
SCN1A	chr2	166848284/G//A	nonsynonymous	0	2
	chr2	188343388/C//T	synonymous	0	2
DNAH7	chr2	196636445/C//T	nonsynonymous	0	2
B3GNT7	chr2	232262715/G//T	nonsynonymous	0	2
NCL	chr2	232321417/T//C	nonsynonymous	0	2
ESPNL	chr2	239039954/G//A	nonsynonymous	0	2
KLHL30	chr2	239059766/C//T	synonymous	0	2
MKRN2	chr3	12613749/C//T	synonymous	0	2
UBE2E2	chr3	23631313/C//T	synonymous	0	2
EOMES	chr3	27763030/G//A	synonymous	0	2
GLB1	chr3	33114035/C//T	nonsynonymous	0	2
SCN5A	chr3	38628912/C//T	synonymous	0	2
PTPRG	chr3	62248483/G//A	nonsynonymous	0	2
ROBO2	chr3	77629269/G//A	nonsynonymous	0	2

CHRD	chr3	184104393/G//T	synonymous	0	2
PIGG	chr4	517656/G//A	nonsynonymous	0	2
FGFRL1	chr4	1016268/G//T	synonymous	0	2
C1QTNF7	chr4	15437568/C//T	synonymous	0	2
FAM184B	chr4	17710912/G//A	nonsynonymous	0	2
SLIT2	chr4	20259563/G//T	nonsynonymous	0	2
PCDH7	chr4	30725822/A//G	synonymous	0	2
TMPRSS11A	chr4	68789839/GACTT//G	synonymous	0	2
TMPRSS11B	chr4	69111344/C//T	synonymous	0	2
NUDT9	chr4	88370379/G//C	nonsynonymous	0	2
SPARCL1	chr4	88415280/C//A	nonsynonymous	0	2
FAT4	chr4	126367473/C//G	nonsynonymous	0	2
FSTL5	chr4	162307369/C//A	nonsynonymous	0	2
FSTL5	chr4	162380374/G//T	nonsynonymous	0	2
ADAMTS16	chr5	5186351/C//T	nonsynonymous	0	2
PRDM9	chr5	23522805/C//A	nonsynonymous	0	2
CDH9	chr5	26881404/C//T	synonymous	0	2
NIPBL	chr5	36985548/G//A	nonsynonymous	0	2
PCDHB11	chr5	140580101/C//T	nonsynonymous	0	2
	chr5	140871326/G//A	synonymous	0	2
PPP2R2B	chr5	146030186/G//T	nonsynonymous	0	2
PDGFRB	chr5	149512491/G//A	nonsynonymous	0	2
STC2	chr5	172744903/C//T	nonsynonymous	0	2
RREB1	chr6	7231817/G//A	nonsynonymous	0	2
MYLIP	chr6	16144037/G//A	nonsynonymous	0	2
SLC17A1	chr6	25820128/A//C	nonsynonymous	0	2
HIST1H1C	chr6	26056236/CCTTCTTGGG//C	nonsynonymous	0	2
OR10C1	chr6	29408456/G//A	nonsynonymous	0	2
ANKS1A	chr6	34985431/C//T	synonymous	0	2
PTCRA	chr6	42890794/G//A	nonsynonymous	0	2
KCNQ5	chr6	73843246/C//T	synonymous	0	2
FRK	chr6	116263645/G//A	nonsynonymous	0	2
THEMIS	chr6	128134623/T//G	nonsynonymous	0	2
LPA	chr6	160968972/G//T	synonymous	0	2
PARK2	chr6	162864355/T//G	nonsynonymous	0	2
ELFN1	chr7	1786477/G//A	nonsynonymous	0	2
FSCN1	chr7	5645038/G//C	nonsynonymous	0	2
	chr7	19035740/C//A	synonymous	0	2
ELMO1	chr7	37250986/C//T	synonymous	0	2
KRIT1	chr7	91842667/G//A	nonsynonymous	0	2
TFR2	chr7	100238383/G//T	synonymous	0	2
ATXN7L1	chr7	105278909/G//C	nonsynonymous	0	2
SSPO	chr7	149487409/G//A	nonsynonymous	0	2
CSMD1	chr8	2967742/G//A	synonymous	0	2
INTS10	chr8	19687979/C//T	synonymous	0	2
INTS9	chr8	28633383/T//C	nonsynonymous	0	2
WRN	chr8	30958372/G//A	synonymous	0	2
PXDNL	chr8	52321807/C//T	nonsynonymous	0	2
DNAJC5B	chr8	66989015/C//T	synonymous	0	2
CPA6	chr8	68658258/C//T	nonsynonymous	0	2
PREX2	chr8	68992691/C//T	synonymous	0	2
KCNB2	chr8	73480500/G//T	nonsynonymous	0	2
MMP16	chr8	89180161/C//T	nonsynonymous	0	2
CSMD3	chr8	113657457/G//T	synonymous	0	2
COL22A1	chr8	139890287/G//A	nonsynonymous	0	2
SCRIB	chr8	144893402/G//C	synonymous	0	2
SMARCA2	chr9	2058328/T//G	nonsynonymous	0	2
ADAMTSL1	chr9	18777224/C//T	synonymous	0	2
TLE4	chr9	82188702/G//A	nonsynonymous	0	2
OR13C2	chr9	107367720/C//A	synonymous	0	2
OR13C9	chr9	107380012/T//C	synonymous	0	2
ASTN2	chr9	119977007/C//T	synonymous	0	2
TSC1	chr9	135797350/C//T	synonymous	0	2
DIP2C	chr10	391025/G//A	nonsynonymous	0	2
PTCHD3	chr10	27702459/C//T	nonsynonymous	0	2
CDH23	chr10	73563112/G//A	nonsynonymous	0	2
SH2D4B	chr10	82298025/G//A	synonymous	0	2
CFAP46	chr10	134659596/C//T	nonsynonymous	0	2
DGKZ	chr11	46400767/C//G	nonsynonymous	0	2
LRFN4	chr11	66625986/C//T	synonymous	0	2

DSCAML1	chr11	117335856/G//A	nonsynonymous	0	2
OR8G1	chr11	124120779/G//A	synonymous	0	2
APOBEC1	chr12	7802129/C//T	synonymous	0	2
CLEC4C	chr12	7883439/G//A	nonsynonymous	0	2
CASC1	chr12	25261549/G//T	nonsynonymous	0	2
AQP2	chr12	50349335/C//T	nonsynonymous	0	2
SLC26A10	chr12	58014797/C//T	synonymous	0	2
OS9	chr12	58111969/C//T	nonsynonymous	0	2
NAV3	chr12	78592429/C//G	nonsynonymous	0	2
ULK1	chr12	132405878/C//T	nonsynonymous	0	2
CCDC169	chr13	36871810/C//T	nonsynonymous	0	2
SPRY2	chr13	80911780/C//A	nonsynonymous	0	2
MYO16	chr13	109793030/G//C	synonymous	0	2
TEP1	chr14	20848416/G//A	nonsynonymous	0	2
RNASE11	chr14	21051942/C//T	synonymous	0	2
RNF31	chr14	24626515/G//A	nonsynonymous	0	2
DACT1	chr14	59113522/G//A	synonymous	0	2
TMEM63C	chr14	77705948/G//A	nonsynonymous	0	2
VRK1	chr14	97326889/T//A	synonymous	0	2
INF2	chr14	105167948/G//T	synonymous	0	2
FAM227B	chr15	49868986/C//T	synonymous	0	2
CORO2B	chr15	69018260/C//T	nonsynonymous	0	2
HBQ1	chr16	230747/G//A	nonsynonymous	0	2
PRR35	chr16	615404/C//T	synonymous	0	2
ADCY9	chr16	4164025/G//A	synonymous	0	2
SMG1	chr16	18823286/G//T	nonsynonymous	0	2
GPR139	chr16	20084944/C//T	synonymous	0	2
IRX6	chr16	55362789/G//A	nonsynonymous	0	2
ZNF319	chr16	58031577/G//A	nonsynonymous	0	2
CDH11	chr16	64981515/G//A	synonymous	0	2
CDH1	chr16	68849586/G//T	nonsynonymous	0	2
HYDIN	chr16	71094403/T//G	synonymous	0	2
CNTNAP4	chr16	76350307/C//A	synonymous	0	2
ADAMTS18	chr16	77327095/C//T	nonsynonymous	0	2
FOXF1	chr16	86545145/G//A	nonsynonymous	0	2
TUBB3	chr16	90001883/G//A	nonsynonymous	0	2
PLD2	chr17	4726139/C//A	nonsynonymous	0	2
TP53	chr17	7577067/T//A	nonsynonymous	0	2
TP53	chr17	7577069/C//A	nonsynonymous	0	2
NTN1	chr17	9143098/C//A	nonsynonymous	0	2
MYH1	chr17	10416212/G//T	synonymous	0	2
NOS2	chr17	26125721/C//T	synonymous	0	2
ARHGAP23	chr17	36614486/G//A	synonymous	0	2
KCNH6	chr17	61613154/C//T	nonsynonymous	0	2
KCNH6	chr17	61615880/G//A	nonsynonymous	0	2
CD300C	chr17	72537643/C//T	synonymous	0	2
RNF157	chr17	74141389/C//T	nonsynonymous	0	2
MTCL1	chr18	8784555/C//T	nonsynonymous	0	2
SERPINB2	chr18	61569672/G//A	nonsynonymous	0	2
DSEL	chr18	65179052/A//T	nonsynonymous	0	2
ELANE	chr19	852341/C//T	nonsynonymous	0	2
ABCA7	chr19	1045159/C//A	synonymous	0	2
ATCAY	chr19	3918819/G//A	synonymous	0	2
ZNF558	chr19	8933397/C//T	synonymous	0	2
B3GNT3	chr19	17919126/C//T	synonymous	0	2
FFAR3	chr19	35850481/C//T	nonsynonymous	0	2
ZNF569	chr19	37903536/G//A	nonsynonymous	0	2
LRRC4B	chr19	51021608/C//T	synonymous	0	2
SYT3	chr19	51128655/G//A	synonymous	0	2
BIRC8	chr19	53793576/C//T	nonsynonymous	0	2
AURKC	chr19	57744038/G//A	nonsynonymous	0	2
ADAM33	chr20	3654269/C//T	synonymous	0	2
REM1	chr20	30072136/G//A	nonsynonymous	0	2
TOP1	chr20	39690075/C//G	nonsynonymous	0	2
GNAS	chr20	57429235/C//T	synonymous	0	2
KIAA1671	chr22	25436780/A//T	nonsynonymous	0	2
SELENOM	chr22	31501230/C//T	synonymous	0	2
LARGE1	chr22	33778016/G//A	synonymous	0	2
FBLN1	chr22	45970514/C//T	synonymous	0	2
ARHGAP6	chrX	11682692/G//A	nonsynonymous	0	2

WNK3	chrX	54263455/G//A	nonsynonymous	0	2
MAGEC2	chrX	141291745/C//T	nonsynonymous	0	2
GPR50	chrX	150348705/C//T	nonsynonymous	0	2
FBN2	chr5	127873165/C//T	synonymous	0	2
VTCN1	chr1	117699488/A//C	nonsynonymous	0	2
MNDA	chr1	158811949/G//A	synonymous	0	2
SELP	chr1	169582277/A//G	nonsynonymous	0	2
CEP170	chr1	243319649/G//T	nonsynonymous	0	2
ITSN2	chr2	24426535/C//T	nonsynonymous	0	2
DHX57	chr2	39053107/C//G	nonsynonymous	0	2
STEAP3	chr2	120005592/C//T	nonsynonymous	0	2
GALNT13	chr2	155102354/T//A	nonsynonymous	0	2
TTN	chr2	179454763/G//A	synonymous	0	2
SCN11A	chr3	38908847/C//T	nonsynonymous	0	2
POC1A	chr3	52159166/G//A	nonsynonymous	0	2
CACNA2D3	chr3	54922012/C//G	nonsynonymous	0	2
ROBO2	chr3	77147379/CT//C	nonsynonymous	0	2
DZIP3	chr3	108394727/C//T	synonymous	0	2
DGKG	chr3	185986694/C//T	nonsynonymous	0	2
CPZ	chr4	8603110/G//T	nonsynonymous	0	2
CPZ	chr4	8603121/C//A	synonymous	0	2
ADGRL3	chr4	62936417/G//A	nonsynonymous	0	2
CDH9	chr5	26881405/G//A	nonsynonymous	0	2
ITGA1	chr5	52214594/T//C	nonsynonymous	0	2
APC	chr5	112173665/A//T	nonsynonymous	0	2
FBN2	chr5	127624892/G//C	nonsynonymous	0	2
PCDHGB1	chr5	140729957/C//T	nonsynonymous	0	2
FOXQ1	chr6	1312852/G//A	synonymous	0	2
RREB1	chr6	7232039/G//A	nonsynonymous	0	2
ADGRF5	chr6	46826169/C//T	synonymous	0	2
FAM135A	chr6	71190660/A//C	nonsynonymous	0	2
LAMA4	chr6	112460430/G//A	synonymous	0	2
PACRG	chr6	163235315/T//A	nonsynonymous	0	2
BMPER	chr7	34085913/C//T	synonymous	0	2
CALCR	chr7	93091405/A//G	nonsynonymous	0	2
ZNF746	chr7	149171593/G//A	nonsynonymous	0	2
ADAM2	chr8	39624709/G//T	nonsynonymous	0	2
DCAF4L2	chr8	88885044/G//A	nonsynonymous	0	2
OSGIN2	chr8	90937251/G//A	nonsynonymous	0	2
DPYS	chr8	105440236/C//T	nonsynonymous	0	2
PRUNE2	chr9	79325235/C//T	nonsynonymous	0	2
POLR3A	chr10	79741953/CGTGTG//C	nonsynonymous	0	2
IFIT1	chr10	91163378/T//G	nonsynonymous	0	2
SMC3	chr10	112340744/G//A	nonsynonymous	0	2
PHRF1	chr11	608092/T//C	nonsynonymous	0	2
CAPRIN1	chr11	34074027/G//C	synonymous	0	2
INTS5	chr11	62415327/G//A	nonsynonymous	0	2
SPTBN2	chr11	66453435/G//A	synonymous	0	2
TRIM77	chr11	89447424/G//A	nonsynonymous	0	2
DYNC2H1	chr11	103048526/G//A	nonsynonymous	0	2
NCAM1	chr11	113126682/G//A	nonsynonymous	0	2
OR8D1	chr11	124179758/C//T	nonsynonymous	0	2
SLCO1B1	chr12	21327592/T//C	nonsynonymous	0	2
IL22	chr12	68647128/G//A	nonsynonymous	0	2
NAV3	chr12	78512039/C//A	nonsynonymous	0	2
ANKS1B	chr12	99640356/G//A	synonymous	0	2
OAS2	chr12	113448328/G//A	synonymous	0	2
PLD4	chr14	105395274/G//A	synonymous	0	2
PIEZO1	chr16	88807934/G//A	synonymous	0	2
TP53	chr17	7578553/T//C	nonsynonymous	0	2
MARCH10	chr17	60814491/T//A	nonsynonymous	0	2
CYB561	chr17	61511684/G//A	synonymous	0	2
TEX2	chr17	62291095/G//A	synonymous	0	2
SLC25A10	chr17	79686879/G//A	nonsynonymous	0	2
ZNF750	chr17	80789903/G//A	nonsynonymous	0	2
ATP8B3	chr19	1800401/G//A	synonymous	0	2
CYP4F22	chr19	15640598/G//A	nonsynonymous	0	2
SIRT2	chr19	39371522/C//A	nonsynonymous	0	2
NDUFAF5	chr20	13765714/G//T	synonymous	0	2
NHSL2	chrX	71358496/C//A	synonymous	0	2

GPR174	chrX	78427441/C//A	nonsynonymous	0	2
TBC1D8B	chrX	106117198/C//A	synonymous	0	2
SAGE1	chrX	134988657/G//A	nonsynonymous	0	2
GCN1	chr12	120598014/T//C	nonsynonymous	1	1
GPR153	chr1	6314624/C//A	nonsynonymous	0	2
HCN1	chr5	45262467/C//T	synonymous	1	1
ZNF98	chr19	22574657/T//C	synonymous	0	2
NCMAP	chr1	24921953/C//T	nonsynonymous	0	2
CSF1	chr1	110466732/G//T	nonsynonymous	0	2
FLG	chr1	152281983/G//A	synonymous	0	2
GAD1	chr2	171693364/A//G	synonymous	0	2
TTN	chr2	179451486/T//C	nonsynonymous	0	2
UNC80	chr2	210769633/C//A	nonsynonymous	0	2
C3orf14	chr3	62319060/G//A	nonsynonymous	0	2
PDIA5	chr3	122865102/C//T	nonsynonymous	0	2
CHST13	chr3	126261099/C//T	nonsynonymous	0	2
OPA1	chr3	193364896/A//C	nonsynonymous	0	2
QDPR	chr4	17513680/T//C	synonymous	0	2
ANAPC4	chr4	25419908/G//A	synonymous	0	2
CYP2U1	chr4	108871406/C//T	nonsynonymous	0	2
TRAPPC11	chr4	184629722/G//A	nonsynonymous	0	2
ANKH	chr5	14758657/C//T	nonsynonymous	0	2
CDH18	chr5	19612625/C//T	synonymous	0	2
MRPS30	chr5	44815150/G//A	nonsynonymous	0	2
CDKL3	chr5	133640227/C//T	nonsynonymous	0	2
CXCL14	chr5	134914198/G//A	synonymous	0	2
PCDHB7	chr5	140553541/C//T	synonymous	0	2
	chr5	141233955/C//T	synonymous	0	2
ADAMTS2	chr5	178585776/G//A	synonymous	0	2
	chr6	30313237/G//A	synonymous	0	2
PTCRA	chr6	42883872/C//T	synonymous	0	2
ASCC3	chr6	101100621/T//A	nonsynonymous	0	2
MAP3K5	chr6	137041639/G//A	synonymous	0	2
PLG	chr6	161134093/G//A	synonymous	0	2
ABCA13	chr7	48494747/C//G	nonsynonymous	0	2
TMEM248	chr7	66413557/A//G	nonsynonymous	0	2
CALN1	chr7	71252855/C//T	nonsynonymous	0	2
ACHE	chr7	100490410/G//C	synonymous	0	2
LAMB4	chr7	107744978/C//T	synonymous	0	2
KCND2	chr7	119915600/G//A	nonsynonymous	0	2
TMEM178B	chr7	140912480/C//T	nonsynonymous	0	2
MTERF3	chr8	97258097/T//G	nonsynonymous	0	2
SHB	chr9	37974800/A//T	nonsynonymous	0	2
SEMA4D	chr9	92002361/C//T	nonsynonymous	0	2
TRIM32	chr9	119461850/T//G	nonsynonymous	0	2
STXBP1	chr9	130438109/G//T	nonsynonymous	0	2
UAP1L1	chr9	139977184/C//T	synonymous	0	2
ADARB2	chr10	1405321/C//T	nonsynonymous	0	2
KIF5B	chr10	32310167/G//C	nonsynonymous	0	2
WDR11	chr10	122610974/C//T	synonymous	0	2
OSBPL5	chr11	3123503/C//A	nonsynonymous	0	2
OR52J3	chr11	5068422/C//T	nonsynonymous	0	2
CABP4	chr11	67225897/C//T	nonsynonymous	0	2
OR8S1	chr12	48921822/C//T	nonsynonymous	0	2
KMT2D	chr12	49418622/C//G	nonsynonymous	0	2
TNS2	chr12	53452896/C//T	nonsynonymous	0	2
ZFC3H1	chr12	72020049/G//A	synonymous	0	2
GIT2	chr12	110383069/C//T	nonsynonymous	0	2
DDX54	chr12	113616826/G//A	synonymous	0	2
SLC8B1	chr12	113737492/C//T	synonymous	0	2
TNFRSF19	chr13	24190175/G//T	nonsynonymous	0	2
PTPN21	chr14	88945510/C//T	synonymous	0	2
SEZ6L2	chr16	29909230/G//T	nonsynonymous	0	2
AARS	chr16	70302247/C//T	nonsynonymous	0	2
PKD1L2	chr16	81211572/G//T	synonymous	0	2
PLD2	chr17	4714232/G//A	synonymous	0	2
TP53	chr17	7577018/C//T	nonsynonymous	0	2
KRT14	chr17	39742607/T//G	nonsynonymous	0	2
RNFT1	chr17	58034650/C//T	nonsynonymous	0	2
SOX9	chr17	70119820/CA//C	nonsynonymous	0	2

NPTX1	chr17	78444762/G//A	nonsynonymous	0	2
TBCD	chr17	80888453/C//T	nonsynonymous	0	2
FHOD3	chr18	34191951/G//A	nonsynonymous	0	2
SMAD2	chr18	45374990/T//C	nonsynonymous	0	2
CACTIN	chr19	3612045/T//C	nonsynonymous	0	2
INSR	chr19	7163143/T//A	nonsynonymous	0	2
NPHS1	chr19	36339073/G//A	synonymous	0	2
ZNF571	chr19	38055647/C//T	synonymous	0	2
NUP62	chr19	50412686/T//A	nonsynonymous	0	2
SLC23A2	chr20	4893541/G//A	synonymous	0	2
CD93	chr20	23066694/C//T	nonsynonymous	0	2
PIGU	chr20	33148709/G//A	synonymous	0	2
JPH2	chr20	42744802/CG//C	nonsynonymous	0	2
SEMG2	chr20	43851878/A//T	nonsynonymous	0	2
GNAS	chr20	57430121/C//T	nonsynonymous	0	2
KRTAP8-1	chr21	32185398/G//A	synonymous	0	2
PATZ1	chr22	31723224/C//T	nonsynonymous	0	2
DDX17	chr22	38882427/C//T	nonsynonymous	0	2
MGAT3	chr22	39883845/C//T	nonsynonymous	0	2
FANCB	chrX	14877361/C//T	synonymous	0	2
MAGEB18	chrX	26157258/G//T	nonsynonymous	0	2
FTHL17	chrX	31090100/C//T	synonymous	0	2
BHLHB9	chrX	102004690/C//T	nonsynonymous	0	2
TRPC5	chrX	111019945/G//T	nonsynonymous	0	2
CDC27	chr17	45219315/T//G	synonymous	0	2
CPTP	chr1	1262892/G//A	nonsynonymous	0	2
GPR157	chr1	9164633/C//T	nonsynonymous	0	2
SELENON	chr1	26140643/A//G	nonsynonymous	0	2
ADGRB2	chr1	32221809/C//T	nonsynonymous	0	2
GPX7	chr1	53074130/G//A	synonymous	0	2
GJA5	chr1	147230995/G//A	nonsynonymous	0	2
OR6K6	chr1	158724764/C//T	synonymous	0	2
LHX4-AS1	chr1	180243175/T//C	synonymous	0	2
NLRP3	chr1	247607422/G//A	nonsynonymous	0	2
NRXN1	chr2	50850732/G//A	nonsynonymous	0	2
CTNNA2	chr2	80816441/C//A	nonsynonymous	0	2
GPR39	chr2	133402920/C//G	nonsynonymous	0	2
DNAJC10	chr2	183582817/G//A	nonsynonymous	0	2
MAP2	chr2	210560164/A//T	synonymous	0	2
BARD1	chr2	215617252/A//C	nonsynonymous	0	2
CCR4	chr3	32995826/C//G	nonsynonymous	0	2
SLC6A20	chr3	45807096/C//T	synonymous	0	2
PLXNB1	chr3	48451704/C//T	nonsynonymous	0	2
GOLGB1	chr3	121441213/T//A	nonsynonymous	0	2
	chr3	158300601/A//G	synonymous	0	2
TNK2	chr3	195609019/C//T	nonsynonymous	0	2
TRMT44	chr4	8469935/C//T	nonsynonymous	0	2
BTC	chr4	75695348/C//T	nonsynonymous	0	2
KIAA1109	chr4	123161112/G//C	nonsynonymous	0	2
NIPBL	chr5	37044530/A//G	nonsynonymous	0	2
ANKRD31	chr5	74464905/G//A	nonsynonymous	0	2
VCAN	chr5	82786281/G//A	synonymous	0	2
NR3C1	chr5	142658907/G//A	synonymous	0	2
PDGFRB	chr5	149513451/C//T	nonsynonymous	0	2
IL12B	chr5	158747472/A//G	nonsynonymous	0	2
GABRB2	chr5	160721231/G//A	nonsynonymous	0	2
MAML1	chr5	179201752/C//T	synonymous	0	2
OR14J1	chr6	29274618/G//A	nonsynonymous	0	2
OR2H2	chr6	29556084/C//T	synonymous	0	2
TREML2	chr6	41166018/G//A	nonsynonymous	0	2
KHDC1L	chr6	73935118/G//A	nonsynonymous	0	2
SMPD2	chr6	109764886/C//T	synonymous	0	2
PLG	chr6	161155095/C//T	synonymous	0	2
SDK1	chr7	4119221/G//A	synonymous	0	2
COBL	chr7	51096287/G//C	nonsynonymous	0	2
ZKSCAN1	chr7	99621462/G//A	synonymous	0	2
COL26A1	chr7	101199070/C//A	synonymous	0	2
WNT2	chr7	116955205/C//T	nonsynonymous	0	2
SSPO	chr7	149500107/G//A	nonsynonymous	0	2
ADAMDEC1	chr8	24251598/G//A	nonsynonymous	0	2

UNC5D	chr8	35093412/G//A	synonymous	0	2
ADAM18	chr8	39525666/C//T	synonymous	0	2
SFRP1	chr8	41166280/G//T	nonsynonymous	0	2
MYBL1	chr8	67479194/G//A	nonsynonymous	0	2
DCSTAMP	chr8	105367322/G//A	nonsynonymous	0	2
CSMD3	chr8	113529403/C//T	nonsynonymous	0	2
ARC	chr8	143695471/G//T	synonymous	0	2
C5	chr9	123745027/G//A	nonsynonymous	0	2
MUC5B	chr11	1267641/C//T	synonymous	0	2
FLRT1	chr11	63884335/C//T	nonsynonymous	0	2
DDI1	chr11	103908577/G//A	nonsynonymous	0	2
OR10G4	chr11	123886320/A//G	synonymous	0	2
KCNA6	chr12	4920191/C//T	synonymous	0	2
TMEM52B	chr12	10332174/A//G	nonsynonymous	0	2
ADAMTS20	chr12	43944807/C//T	nonsynonymous	0	2
KRT5	chr12	52908784/C//T	nonsynonymous	0	2
MSRB3	chr12	65722322/TACACACATCA//T	nonsynonymous	0	2
PPFIA2	chr12	81777975/C//A	nonsynonymous	0	2
LRRIQ1	chr12	85626496/G//T	nonsynonymous	0	2
USP44	chr12	95922718/A//G	synonymous	0	2
RPH3A	chr12	113303233/G//A	nonsynonymous	0	2
TMEM132D	chr12	129563125/A//G	nonsynonymous	0	2
MTUS2	chr13	29855918/C//T	nonsynonymous	0	2
PCDH17	chr13	58207227/G//A	nonsynonymous	0	2
CCDC168	chr13	103383005/T//C	nonsynonymous	0	2
OR4Q3	chr14	20216343/G//A	nonsynonymous	0	2
CLEC14A	chr14	38725024/G//A	synonymous	0	2
SIX4	chr14	61190701/G//A	nonsynonymous	0	2
MAP1A	chr15	43820928/C//T	synonymous	0	2
RNF111	chr15	59368258/C//T	nonsynonymous	0	2
ACAN	chr15	89385018/A//G	nonsynonymous	0	2
MSLN	chr16	815000/G//A	synonymous	0	2
FLYWCH1	chr16	2988367/C//T	nonsynonymous	0	2
TBX6	chr16	30100485/C//T	nonsynonymous	0	2
CDH8	chr16	61747818/G//A	synonymous	0	2
CES3	chr16	66997759/G//A	nonsynonymous	0	2
EDC4	chr16	67918118/C//A	synonymous	0	2
FOXL1	chr16	86612368/C//T	synonymous	0	2
MYBBP1A	chr17	4446037/C//T	nonsynonymous	0	2
TM4SF5	chr17	4686251/C//T	synonymous	0	2
TXNDC17	chr17	6544424/A//T	nonsynonymous	0	2
TP53	chr17	7577132/CT//C	nonsynonymous	0	2
TP53	chr17	7578526/C//T	nonsynonymous	0	2
MYO15A	chr17	18023041/C//T	synonymous	0	2
UNK	chr17	73808347/C//T	nonsynonymous	0	2
ALKBH7	chr19	6374905/G//A	nonsynonymous	0	2
C19orf47	chr19	40829971/G//A	nonsynonymous	0	2
ERCC2	chr19	45867057/C//T	synonymous	0	2
SHANK1	chr19	51171407/G//A	synonymous	0	2
LOC100129083	chr19	51919951/G//A	nonsynonymous	0	2
FCAR	chr19	55385722/C//T	synonymous	0	2
ZNF814	chr19	58385055/C//T	nonsynonymous	0	2
TSHZ2	chr20	51871611/G//C	nonsynonymous	0	2
ZNF831	chr20	57766515/G//A	synonymous	0	2
CDH4	chr20	60448937/C//T	nonsynonymous	0	2
RTEL1	chr20	62290757/T//C	nonsynonymous	0	2
ADAMTS5	chr21	28327141/C//A	nonsynonymous	0	2
PCDH11X	chrX	91090769/C//A	nonsynonymous	0	2
MAGEA12	chrX	151900718/G//A	nonsynonymous	0	2
ERBB4	chr2	212289025/T//A	synonymous	0	2
MYO10	chr5	16699658/C//T	nonsynonymous	0	2
LVRN	chr5	115348077/C//A	nonsynonymous	0	2
LRRC28	chr15	99828068/C//G	synonymous	0	2
AGO4	chr1	36306804/C//T	nonsynonymous	0	2
GRIK3	chr1	37271757/G//A	synonymous	0	2
PRPF3	chr1	150297545/G//A	nonsynonymous	0	2
ASTN1	chr1	176992584/C//T	nonsynonymous	0	2
QSOX1	chr1	180166416/G//A	synonymous	0	2
OBSCN	chr1	228471428/G//A	nonsynonymous	0	2
OSR1	chr2	19553398/G//T	nonsynonymous	0	2

FBXO41	chr2	73486085/G//A	synonymous	0	2
FER1L5	chr2	97357669/C//T	synonymous	0	2
THSD7B	chr2	137917796/G//A	synonymous	0	2
OSBPL6	chr2	179226532/C//T	nonsynonymous	0	2
TTN	chr2	179599535/A//C	nonsynonymous	0	2
IQCF1	chr3	51929179/C//T	synonymous	0	2
PPM1M	chr3	52282387/A//C	nonsynonymous	0	2
EPHA6	chr3	97311565/C//T	synonymous	0	2
GRAMD1C	chr3	113649666/C//T	nonsynonymous	0	2
SEMA5B	chr3	122632822/G//A	nonsynonymous	0	2
XRN1	chr3	142151562/C//T	nonsynonymous	0	2
C1QTNF7	chr4	15443893/G//T	nonsynonymous	0	2
FREM3	chr4	144621238/G//A	synonymous	0	2
FBXW7	chr4	153249400/G//A	nonsynonymous	0	2
IL31RA	chr5	55179013/G//A	nonsynonymous	0	2
ARHGEF28	chr5	73072424/C//T	nonsynonymous	0	2
APC	chr5	112175174/G//T	nonsynonymous	0	2
JADE2	chr5	133896552/G//A	nonsynonymous	0	2
HIST1H1B	chr6	27834842/G//T	nonsynonymous	0	2
COL21A1	chr6	55925754/C//T	nonsynonymous	0	2
C6orf163	chr6	88054832/A//T	synonymous	0	2
SLC22A16	chr6	110763562/C//T	synonymous	0	2
REV3L	chr6	111650871/T//C	nonsynonymous	0	2
FNDC1	chr6	159653499/G//A	nonsynonymous	0	2
SEMA3E	chr7	83014750/C//A	nonsynonymous	0	2
ZSCAN21	chr7	99654851/G//GAGGC	nonsynonymous	0	2
HR	chr8	21984772/G//A	nonsynonymous	0	2
PLEC	chr8	144991734/G//A	synonymous	0	2
PALM2-AKAP2	chr9	112900755/G//A	synonymous	0	2
FAM129B	chr9	130270468/G//A	synonymous	0	2
AKR1C1	chr10	5008116/G//A	nonsynonymous	0	2
C10orf10	chr10	45473043/C//A	nonsynonymous	0	2
	chr10	126716328/T//C	synonymous	0	2
CFAP46	chr10	134754546/C//T	nonsynonymous	0	2
MUC5B	chr11	1253691/C//T	nonsynonymous	0	2
MRGPRX4	chr11	18194994/A//G	nonsynonymous	0	2
ROBO3	chr11	124749773/G//A	nonsynonymous	0	2
TAS2R9	chr12	10961912/C//T	nonsynonymous	0	2
SRRM4	chr12	119540111/G//C	nonsynonymous	0	2
OASL	chr12	121469346/T//C	nonsynonymous	0	2
CLIP1	chr12	122861951/G//A	synonymous	0	2
USPL1	chr13	31232294/G//T	nonsynonymous	0	2
SETDB2-PHF11	chr13	50092165/T//G	nonsynonymous	0	2
PCDH17	chr13	58208945/C//T	synonymous	0	2
SLC10A1	chr14	70263684/G//A	synonymous	0	2
IRF2BPL	chr14	77491733/C//T	synonymous	0	2
STON2	chr14	81744376/C//T	nonsynonymous	0	2
SERPINA6	chr14	94780856/C//T	nonsynonymous	0	2
INF2	chr14	105167941/C//T	nonsynonymous	0	2
PGPEP1L	chr15	99512804/C//T	nonsynonymous	0	2
PRR35	chr16	615081/C//A	nonsynonymous	0	2
ZNF689	chr16	30616199/G//A	nonsynonymous	0	2
USB1	chr16	58052921/C//T	nonsynonymous	0	2
CDH11	chr16	64984749/G//A	synonymous	0	2
C16orf46	chr16	81095024/C//T	synonymous	0	2
VTN	chr17	26697154/C//T	synonymous	0	2
ERBB2	chr17	37879658/G//A	nonsynonymous	0	2
SEPT4	chr17	56606580/T//G	synonymous	0	2
AATK	chr17	79101475/G//A	synonymous	0	2
SAFB	chr19	5649943/C//T	synonymous	0	2
USE1	chr19	17330546/G//A	nonsynonymous	0	2
ELL	chr19	18576252/T//C	nonsynonymous	0	2
RYR1	chr19	38990456/C//T	synonymous	0	2
JPH2	chr20	42744632/C//T	synonymous	0	2
CBR3	chr21	37518545/C//T	nonsynonymous	0	2
SUMO3	chr21	46229000/C//T	nonsynonymous	0	2
IL2RB	chr22	37533656/G//A	nonsynonymous	0	2
SYP	chrX	49054218/G//C	synonymous	0	2
TAF1	chrX	70626576/G//C	nonsynonymous	0	2
FLNA	chrX	153590653/G//A	synonymous	0	2

OSBPL9	chr1	52211296/A//C	nonsynonymous	0	2
PKN2	chr1	89250492/A//T	nonsynonymous	0	2
SPTA1	chr1	158639506/C//T	nonsynonymous	0	2
FAM110C	chr2	45506/T//C	nonsynonymous	0	2
APOB	chr2	21230354/G//T	nonsynonymous	0	2
TTN	chr2	179516186/C//A	nonsynonymous	0	2
PTH2R	chr2	209358240/C//A	nonsynonymous	0	2
FGFR3	chr4	1805511/G//A	synonymous	0	2
TARS	chr5	33457506/AG//A	nonsynonymous	0	2
APC	chr5	112176017/G//GCA	nonsynonymous	0	2
PCDHA6	chr5	140208455/C//A	nonsynonymous	0	2
PCDHGA7	chr5	140764170/C//A	synonymous	0	2
NKAPL	chr6	28227806/TAA//T	nonsynonymous	0	2
HSPA1L	chr6	31778688/G//T	nonsynonymous	0	2
GPR6	chr6	110300760/G//A	nonsynonymous	0	2
FAM20C	chr7	295968/G//A	nonsynonymous	0	2
SNX8	chr7	2294711/C//T	nonsynonymous	0	2
STRA8	chr7	134925410/C//T	nonsynonymous	0	2
DGKI	chr7	137080402/C//A	nonsynonymous	0	2
CHRNA6	chr8	42611803/G//A	nonsynonymous	0	2
CHD7	chr8	61763057/G//A	nonsynonymous	0	2
MTSS1	chr8	125565491/G//A	synonymous	0	2
TEK	chr9	27168535/G//A	nonsynonymous	0	2
OR13J1	chr9	35869820/C//T	synonymous	0	2
ODF2	chr9	131235251/C//T	nonsynonymous	0	2
ANO1	chr11	69934055/G//A	synonymous	0	2
PUS3	chr11	125763876/C//T	nonsynonymous	0	2
B4GALNT3	chr12	644398/A//G	nonsynonymous	0	2
SLC4A8	chr12	51857467/C//T	nonsynonymous	0	2
SLITRK5	chr13	88327743/G//A	nonsynonymous	0	2
UGGT2	chr13	96705547/G//C	nonsynonymous	0	2
ACIN1	chr14	23548850/G//GGGTT	nonsynonymous	0	2
ADAMTS7	chr15	79063592/C//T	synonymous	0	2
RASGRF1	chr15	79310133/C//T	synonymous	0	2
FLYWCH1	chr16	2983739/G//A	synonymous	0	2
ZNF213	chr16	3191321/C//T	synonymous	0	2
TTYH2	chr17	72246144/G//A	nonsynonymous	0	2
EVPL	chr17	74006367/G//C	synonymous	0	2
PCSK4	chr19	1483302/A//C	nonsynonymous	0	2
CAMSAP3	chr19	7682330/C//T	synonymous	0	2
SLC1A6	chr19	15073101/G//A	synonymous	0	2
MYO9B	chr19	17322521/C//T	nonsynonymous	0	2
PSG6	chr19	43411120/T//A	synonymous	0	2
LIG1	chr19	48639341/G//A	nonsynonymous	0	2
NLRP7	chr19	55435219/A//G	synonymous	0	2
MRGBP	chr20	61430987/C//T	nonsynonymous	0	2
OGFR	chr20	61444413/C//T	synonymous	0	2
IRS4	chrX	107978707/G//A	nonsynonymous	0	2
PADI3	chr1	17586115/G//A	synonymous	0	2
SH2D5	chr1	21048413/G//A	nonsynonymous	0	2
GRIK3	chr1	37271853/G//A	synonymous	0	2
DNAJC6	chr1	65852598/C//T	nonsynonymous	0	2
PROK1	chr1	110998872/C//T	nonsynonymous	0	2
BCAS2	chr1	115118330/A//C	nonsynonymous	0	2
SPAG17	chr1	118629341/G//A	synonymous	0	2
GJA8	chr1	147380408/G//A	nonsynonymous	0	2
LCE2B	chr1	152659659/C//A	synonymous	0	2
IQGAP3	chr1	156518002/G//A	nonsynonymous	0	2
KIF21B	chr1	200961451/G//T	synonymous	0	2
OBSCN	chr1	228495119/G//A	nonsynonymous	0	2
MAP3K21	chr1	233489647/C//A	nonsynonymous	0	2
EXO1	chr1	242023881/C//T	synonymous	0	2
OR2AK2	chr1	248129436/C//T	nonsynonymous	0	2
KCNF1	chr2	11053446/G//A	synonymous	0	2
MDH1	chr2	63831897/C//T	nonsynonymous	0	2
THSD7B	chr2	137814765/G//A	synonymous	0	2
TTN	chr2	179566963/G//T	nonsynonymous	0	2
COL4A4	chr2	227895207/G//C	nonsynonymous	0	2
COL4A3	chr2	228157983/C//A	nonsynonymous	0	2
ROBO2	chr3	77623684/G//A	nonsynonymous	0	2

LAMP3	chr3	182853518/G//T	nonsynonymous	0	2
HTR3D	chr3	183756305/G//A	nonsynonymous	0	2
FREM3	chr4	144617239/G//T	synonymous	0	2
IRX1	chr5	3599720/G//A	nonsynonymous	0	2
CDH10	chr5	24505268/C//T	nonsynonymous	0	2
FBN2	chr5	127648333/G//C	synonymous	0	2
FBN2	chr5	127653938/T//C	nonsynonymous	0	2
PCDHGB2	chr5	140741602/G//A	nonsynonymous	0	2
HMGXB3	chr5	149431497/C//T	synonymous	0	2
RNF44	chr5	175956762/G//A	synonymous	0	2
VARS	chr6	31747887/C//T	nonsynonymous	0	2
ABCC10	chr6	43415116/G//T	synonymous	0	2
GPR6	chr6	110300953/G//A	nonsynonymous	0	2
CEP85L	chr6	118791782/T//C	nonsynonymous	0	2
MYB	chr6	135517071/C//T	synonymous	0	2
SHPRH	chr6	146256076/G//A	nonsynonymous	0	2
PDE10A	chr6	165756944/A//C	nonsynonymous	0	2
SDK1	chr7	4056945/G//A	nonsynonymous	0	2
C7orf65	chr7	47698325/G//T	nonsynonymous	0	2
ABCA13	chr7	48412028/C//A	nonsynonymous	0	2
BRAF	chr7	140481397/C//A	nonsynonymous	0	2
PCM1	chr8	17843043/G//A	nonsynonymous	0	2
TMEM64	chr8	91643863/A//T	nonsynonymous	0	2
ADCY8	chr8	131848632/C//T	nonsynonymous	0	2
COL22A1	chr8	139618674/C//A	nonsynonymous	0	2
CNTNAP3B	chr9	43861144/C//T	nonsynonymous	0	2
TRPM3	chr9	73152290/C//T	nonsynonymous	0	2
IKBKAP	chr9	111653607/G//A	synonymous	0	2
CTNNAL1	chr9	111741604/G//A	nonsynonymous	0	2
OR1B1	chr9	125391444/C//T	nonsynonymous	0	2
CRAT	chr9	131862944/C//T	nonsynonymous	0	2
GJD4	chr10	35897350/C//T	synonymous	0	2
CTNNA3	chr10	67829254/CAT//C	synonymous	0	2
DOCK1	chr10	129224200/G//A	synonymous	0	2
CNGA4	chr11	6261521/C//A	nonsynonymous	0	2
SYT9	chr11	7324367/G//A	synonymous	0	2
CBL	chr11	119148487/G//A	nonsynonymous	0	2
NELL2	chr12	44915950/C//T	nonsynonymous	0	2
LACRT	chr12	55026054/G//A	nonsynonymous	0	2
SYCP3	chr12	102131736/A//T	synonymous	0	2
BTBD11	chr12	108051343/A//G	nonsynonymous	0	2
HCAR2	chr12	123187466/G//A	nonsynonymous	0	2
PCDH17	chr13	58207323/G//T	nonsynonymous	0	2
KLF5	chr13	73636643/G//A	synonymous	0	2
PNN	chr14	39649838/GAGGGTA//G	nonsynonymous	0	2
PNN	chr14	39649847/G//T	nonsynonymous	0	2
FAM179B	chr14	45433255/G//A	nonsynonymous	0	2
UNC79	chr14	94088460/C//T	synonymous	0	2
GABRA5	chr15	27159978/C//G	nonsynonymous	0	2
CSPG4	chr15	75968675/C//T	nonsynonymous	0	2
RHBDF1	chr16	111954/C//T	synonymous	0	2
NLRC3	chr16	3611718/C//T	nonsynonymous	0	2
SRL	chr16	4242321/G//T	nonsynonymous	0	2
USP7	chr16	9011001/TC//T	nonsynonymous	0	2
ABCC1	chr16	16130410/C//T	synonymous	0	2
ABCC12	chr16	48177854/G//A	nonsynonymous	0	2
SLC9A5	chr16	67286508/G//A	nonsynonymous	0	2
CLUH	chr17	2598824/C//T	synonymous	0	2
TP53	chr17	7578442/T//C	nonsynonymous	0	2
NTN1	chr17	8925978/G//T	synonymous	0	2
MYH3	chr17	10543325/G//T	synonymous	0	2
KIF2B	chr17	51900435/C//T	nonsynonymous	0	2
BAHCC1	chr17	79425420/G//A	synonymous	0	2
FAAP100	chr17	79517864/G//A	nonsynonymous	0	2
ZNF516	chr18	74091424/A//C	synonymous	0	2
CNN2	chr19	1037670/A//G	nonsynonymous	0	2
SEMA6B	chr19	4544353/C//T	nonsynonymous	0	2
PODNL1	chr19	14049119/C//G	synonymous	0	2
PKN1	chr19	14552077/G//C	nonsynonymous	0	2
ZNF507	chr19	32847568/C//A	nonsynonymous	0	2

FAM187B	chr19	35715865/C//T	nonsynonymous	0	2
ZNF585A	chr19	37642418/T//C	synonymous	0	2
ATP1A3	chr19	42472968/G//T	synonymous	0	2
ERF	chr19	42753668/G//A	nonsynonymous	0	2
SNPH	chr20	1285771/G//A	synonymous	0	2
REM1	chr20	30072073/G//A	nonsynonymous	0	2
L3MBTL1	chr20	42143373/C//T	synonymous	0	2
NTSR1	chr20	61340774/C//A	nonsynonymous	0	2
PCNT	chr21	47851820/A//G	synonymous	0	2
	chr22	30079052/A//T	synonymous	0	2
PLXNB2	chr22	50722998/G//A	synonymous	0	2
NYX	chrX	41333793/G//A	nonsynonymous	0	2
TRPC5OS	chrX	111145461/C//A	synonymous	0	2
ZNF732	chr4	265845/C//T	synonymous	0	2
OR1K1	chr9	125562968/G//C	synonymous	0	2
BAGE3	chr21	11049617/C//G	nonsynonymous	1	1
ST3GAL3	chr1	44364864/C//T	synonymous	0	2
UHMK1	chr1	162469867/TC//T	nonsynonymous	0	2
HADHA	chr2	26461987/C//G	nonsynonymous	0	2
CRIM1	chr2	36749369/G//A	nonsynonymous	0	2
TEKT4	chr2	95537665/C//T	nonsynonymous	0	2
AMMECR1L	chr2	128627067/G//A	nonsynonymous	0	2
HSPD1	chr2	198352673/T//G	nonsynonymous	0	2
TMEM198	chr2	220412567/G//A	nonsynonymous	0	2
TOPAZ1	chr3	44283835/C//T	nonsynonymous	0	2
SETD2	chr3	47162997/T//G	nonsynonymous	0	2
GOLGB1	chr3	121414975/T//C	synonymous	0	2
ITGB5	chr3	124540214/G//A	synonymous	0	2
RHO	chr3	129251251/G//A	nonsynonymous	0	2
TRIM42	chr3	140401985/C//T	synonymous	0	2
LPP	chr3	188590548/C//T	synonymous	0	2
NRROS	chr3	196388567/C//T	nonsynonymous	0	2
PROM1	chr4	16010694/G//A	synonymous	0	2
ATP8A1	chr4	42618043/T//C	synonymous	0	2
UBA6	chr4	68489850/T//A	nonsynonymous	0	2
WDFY3	chr4	85731344/C//G	nonsynonymous	0	2
PPP3CA	chr4	102001731/T//C	nonsynonymous	0	2
PDZD2	chr5	32069671/T//C	synonymous	0	2
IL6ST	chr5	55259225/T//C	synonymous	0	2
PCDHA12	chr5	140255547/G//A	nonsynonymous	0	2
PCDHGA9	chr5	140784372/C//T	nonsynonymous	0	2
BMP6	chr6	7727818/C//T	synonymous	0	2
TRIM27	chr6	28876581/T//A	synonymous	0	2
CDSN	chr6	31085253/G//A	nonsynonymous	0	2
FNDC1	chr6	159653528/G//A	nonsynonymous	0	2
MRPL18	chr6	160212039/A//G	synonymous	0	2
	chr7	21468433/A//C	synonymous	0	2
FGFR1	chr8	38287438/C//G	synonymous	0	2
CSMD3	chr8	113308209/T//C	nonsynonymous	0	2
LAMC3	chr9	133911650/C//T	nonsynonymous	0	2
DHX32	chr10	127527615/G//T	nonsynonymous	0	2
OR5D13	chr11	55541620/G//A	nonsynonymous	0	2
TENM4	chr11	78381121/C//T	nonsynonymous	0	2
NCAPD3	chr11	134080199/T//G	nonsynonymous	0	2
GSG1	chr12	13241739/G//A	nonsynonymous	0	2
PRICKLE1	chr12	42853796/C//T	nonsynonymous	0	2
DBX2	chr12	45429831/C//T	nonsynonymous	0	2
CIT	chr12	120148110/C//A	nonsynonymous	0	2
GPR183	chr13	99947874/C//T	nonsynonymous	0	2
CDT1	chr16	88873536/G//A	synonymous	0	2
ERBB2	chr17	37868208/C//T	nonsynonymous	0	2
AXIN2	chr17	63554552/C//CCAA	nonsynonymous	0	2
SOX9	chr17	70117860/A//T	nonsynonymous	0	2
EBI3	chr19	4231214/C//T	nonsynonymous	0	2
CHAF1B	chr21	37783872/T//C	nonsynonymous	0	2
AMY2B	chr1	104114762/C//T	nonsynonymous	0	2
FLG	chr1	152277189/G//A	synonymous	0	2
OBSCN	chr1	228482738/G//A	nonsynonymous	0	2
NID1	chr1	236145059/C//T	nonsynonymous	0	2
MTR	chr1	237054459/G//A	nonsynonymous	0	2

APOB	chr2	21247929/G//A	nonsynonymous	0	2
DRC1	chr2	26644195/A//G	nonsynonymous	0	2
TCF23	chr2	27373194/G//A	synonymous	0	2
RASGRP3	chr2	33747142/G//T	synonymous	0	2
KCNIP3	chr2	96049004/G//A	nonsynonymous	0	2
CFAP221	chr2	120404625/C//T	nonsynonymous	0	2
XIRP2	chr2	168103596/T//G	synonymous	0	2
TTN	chr2	179596443/C//A	nonsynonymous	0	2
NEUROD1	chr2	182543157/C//T	nonsynonymous	0	2
ECEL1	chr2	233350643/C//T	nonsynonymous	0	2
CNTN4	chr3	2613143/G//A	synonymous	0	2
ZBBX	chr3	167051664/T//C	nonsynonymous	0	2
GP5	chr3	194118986/G//A	nonsynonymous	0	2
EVC	chr4	5754776/C//T	nonsynonymous	0	2
GBA3	chr4	22820477/A//T	synonymous	0	2
GABRG1	chr4	46060534/C//T	nonsynonymous	0	2
GLRA3	chr4	175749941/G//A	nonsynonymous	0	2
MAP3K1	chr5	56181795/C//T	nonsynonymous	0	2
PIK3R1	chr5	67589569/TGTAGGGAAAAAATTA	nonsynonymous	0	2
APC	chr5	112174455/T//TAATAG	nonsynonymous	0	2
PCDHGA3	chr5	140723769/C//T	nonsynonymous	0	2
	chr5	146728306/G//A	synonymous	0	2
GEMIN5	chr5	154278010/T//C	nonsynonymous	0	2
PANK3	chr5	168006289/G//A	synonymous	0	2
FAM193B	chr5	176966040/C//T	nonsynonymous	0	2
TUBB2B	chr6	3226841/A//T	nonsynonymous	0	2
HIST1H2BG	chr6	26216777/C//T	nonsynonymous	0	2
ZNF391	chr6	27368870/C//T	nonsynonymous	0	2
SLC44A4	chr6	31833313/T//A	nonsynonymous	0	2
C2	chr6	31896515/G//A	nonsynonymous	0	2
SPATS1	chr6	44329580/G//A	nonsynonymous	0	2
ANKRD6	chr6	90326298/C//T	synonymous	0	2
TXLNB	chr6	139597966/C//T	nonsynonymous	0	2
CREB5	chr7	28610075/C//T	synonymous	0	2
KMT2C	chr7	151841864/T//A	synonymous	0	2
SNAI2	chr8	49832591/G//A	synonymous	0	2
PXDNL	chr8	52258454/G//A	nonsynonymous	0	2
	chr8	120608106/G//A	synonymous	0	2
ZC3H3	chr8	144618587/G//A	synonymous	0	2
ACO1	chr9	32408522/G//A	nonsynonymous	0	2
GNE	chr9	36222986/G//T	nonsynonymous	0	2
APBA1	chr9	72047577/G//A	nonsynonymous	0	2
TMEM38B	chr9	108467985/T//C	synonymous	0	2
OBP2B	chr9	136083867/C//T	synonymous	0	2
LHX3	chr9	139091555/G//GCA	nonsynonymous	0	2
C9orf142	chr9	139887110/G//A	synonymous	0	2
DIP2C	chr10	408466/C//T	nonsynonymous	0	2
OPTN	chr10	13166004/G//A	nonsynonymous	0	2
DNMBP	chr10	101648613/C//T	nonsynonymous	0	2
OR51Q1	chr11	5443900/C//T	nonsynonymous	0	2
OR9Q2	chr11	57958876/T//C	nonsynonymous	0	2
ARAP1	chr11	72423585/C//T	nonsynonymous	0	2
SORL1	chr11	121429350/G//C	nonsynonymous	0	2
CD163L1	chr12	7531838/C//T	nonsynonymous	0	2
TAS2R8	chr12	10959439/G//C	nonsynonymous	0	2
SLC5A8	chr12	101551054/G//A	synonymous	0	2
AMER2	chr13	25744303/C//T	synonymous	0	2
FLT1	chr13	29012396/G//A	nonsynonymous	0	2
CTSG	chr14	25043468/G//A	nonsynonymous	0	2
FOXG1	chr14	29237831/C//T	nonsynonymous	0	2
TERB2	chr15	45249124/G//A	nonsynonymous	0	2
TLN2	chr15	63089545/G//A	nonsynonymous	0	2
CHTF18	chr16	845124/G//A	synonymous	0	2
IL4R	chr16	27374642/C//G	nonsynonymous	0	2
ADAMTS18	chr16	77353832/C//T	nonsynonymous	0	2
TMEM104	chr17	72791745/C//T	nonsynonymous	0	2
CBLN2	chr18	70205542/C//A	nonsynonymous	0	2
MUC16	chr19	8961996/C//A	nonsynonymous	0	2
	chr19	35646305/C//A	synonymous	0	2
PROSER3	chr19	36258697/G//A	synonymous	0	2

LTBP4	chr19	41128555/G//T	nonsynonymous	0	2
TPRX1	chr19	48306143/C//T	nonsynonymous	0	2
SMOX	chr20	4162600/G//A	nonsynonymous	0	2
SOGA1	chr20	35444460/C//T	nonsynonymous	0	2
ADAMTS5	chr21	28338138/G//A	synonymous	0	2
YBEY	chr21	47707032/C//T	nonsynonymous	0	2
SUV39H1	chrX	48559096/C//G	synonymous	0	2
PCDH11X	chrX	91134258/G//A	nonsynonymous	0	2
IRS4	chrX	107977567/C//T	nonsynonymous	0	2
TONSL	chr8	145661462/G//T	nonsynonymous	0	2
ZNF433	chr19	12125984/C//T	synonymous	0	2
ZNF208	chr19	22156830/C//T	nonsynonymous	0	2
BAGE3	chr21	11049621/G//A	nonsynonymous	1	1
BAGE4	chr21	11049623/T//C	nonsynonymous	1	1
BSDC1	chr1	32834053/C//T	nonsynonymous	0	2
TIE1	chr1	43770528/C//T	nonsynonymous	0	2
	chr1	51810762/G//A	synonymous	0	2
PATJ	chr1	62241004/C//T	nonsynonymous	0	2
BCL9	chr1	147096464/C//T	nonsynonymous	0	2
SLAMF7	chr1	160721156/G//C	nonsynonymous	0	2
SLC30A3	chr2	27480066/G//T	nonsynonymous	0	2
TCF7L1	chr2	85532525/G//A	nonsynonymous	0	2
LCT	chr2	136566512/C//A	synonymous	0	2
NEB	chr2	152554108/C//T	nonsynonymous	0	2
ALS2	chr2	202631982/C//T	nonsynonymous	0	2
NEPRO	chr3	112732803/G//T	nonsynonymous	0	2
CHST13	chr3	126261356/G//A	nonsynonymous	0	2
CLSTN2	chr3	140122484/G//A	synonymous	0	2
CLSTN2	chr3	140178443/A//T	nonsynonymous	0	2
TRPC1	chr3	142525039/C//T	nonsynonymous	0	2
AGTR1	chr3	148459160/A//G	nonsynonymous	0	2
EPHB3	chr3	184298752/C//T	synonymous	0	2
CFAP99	chr4	2461869/C//A	nonsynonymous	0	2
ENAM	chr4	71509557/G//C	nonsynonymous	0	2
PCDH10	chr4	134072148/G//T	nonsynonymous	0	2
MYOT	chr5	137222986/G//A	nonsynonymous	0	2
PCDHA6	chr5	140207950/C//T	nonsynonymous	0	2
SYCP2L	chr6	10887331/C//T	synonymous	0	2
HIST1H2AG	chr6	27100947/C//T	nonsynonymous	0	2
HIST1H2BM	chr6	27783149/C//T	nonsynonymous	0	2
RXRB	chr6	33168070/C//T	nonsynonymous	0	2
PHF1	chr6	33383094/G//A	nonsynonymous	0	2
SLC26A8	chr6	35965683/C//T	synonymous	0	2
LRRC73	chr6	43476632/G//A	nonsynonymous	0	2
CCNC	chr6	99998189/G//C	synonymous	0	2
LAMA4	chr6	112469484/C//A	nonsynonymous	0	2
FSCN1	chr7	5643546/C//T	synonymous	0	2
GRM3	chr7	86394750/G//C	nonsynonymous	0	2
RELN	chr7	103234872/C//T	nonsynonymous	0	2
FLNC	chr7	128486420/G//A	nonsynonymous	0	2
MPDZ	chr9	13168437/G//T	nonsynonymous	0	2
GALT	chr9	34646755/C//T	synonymous	0	2
POLR3A	chr10	79770276/G//A	nonsynonymous	0	2
OAT	chr10	126086536/A//C	nonsynonymous	0	2
ADAM12	chr10	127806744/T//A	nonsynonymous	0	2
KCNC1	chr11	17757916/G//A	nonsynonymous	0	2
SLC17A6	chr11	22360098/A//T	nonsynonymous	0	2
MTNR1B	chr11	92703054/G//A	nonsynonymous	0	2
KRT83	chr12	52708554/G//A	nonsynonymous	0	2
NOS1	chr12	117672378/G//A	nonsynonymous	0	2
MZT1	chr13	73301700/G//T	nonsynonymous	0	2
PARP2	chr14	20811833/C//T	synonymous	0	2
CCNB2	chr15	59397352/C//T	synonymous	0	2
HCN4	chr15	73624577/G//A	synonymous	0	2
CLK3	chr15	74908105/C//T	nonsynonymous	0	2
STARD5	chr15	81616490/G//A	synonymous	0	2
POLG	chr15	89876358/T//A	nonsynonymous	0	2
ITGAD	chr16	31437436/T//G	nonsynonymous	0	2
ADCY7	chr16	50343553/T//A	nonsynonymous	0	2
LOC100130950	chr17	5137997/G//A	synonymous	0	2

MMP28	chr17	34093887/G//A	nonsynonymous	0	2
FKBP10	chr17	39974510/C//T	synonymous	0	2
CD300LF	chr17	72700756/G//T	synonymous	0	2
ZBTB14	chr18	5293279/C//T	synonymous	0	2
PIEZO2	chr18	10761028/G//T	nonsynonymous	0	2
PNPLA6	chr19	7600813/C//T	nonsynonymous	0	2
FBN3	chr19	8212229/G//A	nonsynonymous	0	2
ZNF823	chr19	11833104/G//T	synonymous	0	2
HOOK2	chr19	12876942/G//A	nonsynonymous	0	2
HSPB6	chr19	36246550/C//T	nonsynonymous	0	2
FBXO27	chr19	39516137/G//A	nonsynonymous	0	2
PHLDB3	chr19	43979518/C//T	synonymous	0	2
RTN2	chr19	45992718/A//G	nonsynonymous	0	2
NLRP8	chr19	56473555/C//T	nonsynonymous	0	2
	chr20	3219627/G//A	synonymous	0	2
KAT14	chr20	18123341/C//T	nonsynonymous	0	2
TIAM1	chr21	32519227/T//A	nonsynonymous	0	2
SEC14L4	chr22	30891955/C//T	nonsynonymous	0	2
PIGA	chrX	15342835/T//C	nonsynonymous	0	2
IL1RAPL1	chrX	29301081/G//T	nonsynonymous	0	2
BEX1	chrX	102318048/C//T	nonsynonymous	0	2
INTS6L	chrX	134713896/A//G	nonsynonymous	0	2
ARHGAP4	chrX	153184440/C//T	nonsynonymous	0	2
MTOR	chr1	11184573/G//A	nonsynonymous	0	2
CSMD2	chr1	34180223/G//A	nonsynonymous	0	2
PODN	chr1	53544662/C//T	nonsynonymous	0	2
FLG	chr1	152278614/A//T	nonsynonymous	0	2
NPR1	chr1	153653059/C//T	synonymous	0	2
	chr1	174957810/C//A	synonymous	0	2
RGSL1	chr1	182520256/C//T	synonymous	0	2
OR2M5	chr1	248309327/G//A	nonsynonymous	0	2
OR2M3	chr1	248367070/G//A	nonsynonymous	0	2
KIDINS220	chr2	8926117/G//A	synonymous	0	2
EHBP1	chr2	63175875/G//A	nonsynonymous	0	2
THSD7B	chr2	138000059/G//A	nonsynonymous	0	2
TTN	chr2	179455956/G//A	nonsynonymous	0	2
ANKRD44	chr2	197943438/ACTGC//A	nonsynonymous	0	2
SPATA3	chr2	231861183/C//T	nonsynonymous	0	2
KIF1A	chr2	241661926/G//A	nonsynonymous	0	2
ZNF621	chr3	40567328/C//T	synonymous	0	2
LOC100132146	chr3	46658790/C//T	nonsynonymous	0	2
ATP6V1A	chr3	113499950/G//A	nonsynonymous	0	2
COL6A6	chr3	130305467/G//A	nonsynonymous	0	2
COL6A6	chr3	130311566/G//T	nonsynonymous	0	2
MED12L	chr3	151085967/G//T	nonsynonymous	0	2
ATP13A3	chr3	194170890/G//A	synonymous	0	2
ZFYVE28	chr4	2274921/C//T	nonsynonymous	0	2
AFAP1	chr4	7774601/G//A	synonymous	0	2
ATG12	chr5	115177248/A//G	nonsynonymous	0	2
PCDHB6	chr5	140530442/G//A	nonsynonymous	0	2
SPINK5	chr5	147493998/G//A	nonsynonymous	0	2
HSPA1A	chr6	31785438/C//T	synonymous	0	2
LRFN2	chr6	40399634/G//A	nonsynonymous	0	2
KLHDC3	chr6	42986824/C//T	synonymous	0	2
CRISP3	chr6	49696439/G//A	synonymous	0	2
PKHD1	chr6	51524087/C//A	nonsynonymous	0	2
RAET1G	chr6	150240331/G//A	nonsynonymous	0	2
FOXK1	chr7	4796676/C//T	nonsynonymous	0	2
RADIL	chr7	4874675/C//T	nonsynonymous	0	2
DNAH11	chr7	21599253/C//A	nonsynonymous	0	2
MYOM2	chr8	2005484/C//T	synonymous	0	2
VPS13B	chr8	100871666/G//A	nonsynonymous	0	2
TAF1L	chr9	32631935/G//A	nonsynonymous	0	2
PFKFB3	chr10	6259141/C//G	nonsynonymous	0	2
SFMBT2	chr10	7239527/C//T	nonsynonymous	0	2
TNNT3	chr11	1956136/G//A	nonsynonymous	0	2
EIF4G2	chr11	10827509/T//A	nonsynonymous	0	2
OR5AN1	chr11	59132537/A//G	nonsynonymous	0	2
TMEM132A	chr11	60701155/G//A	nonsynonymous	0	2
FLRT1	chr11	63883943/C//T	synonymous	0	2

NCAM1	chr11	113105804/G//A	synonymous	0	2
CEP164	chr11	117265175/G//A	nonsynonymous	0	2
XPO4	chr13	21395842/C//T	nonsynonymous	0	2
AHNAK2	chr14	105411897/G//A	synonymous	0	2
CATSPER2	chr15	43925084/C//T	synonymous	0	2
UNC13C	chr15	54305416/G//T	nonsynonymous	0	2
MIEF2	chr17	18167262/C//A	nonsynonymous	0	2
KRT12	chr17	39019979/G//A	synonymous	0	2
KRT23	chr17	39092747/C//T	nonsynonymous	0	2
KCNH4	chr17	40321665/C//T	nonsynonymous	0	2
SSTR2	chr17	71166235/C//T	synonymous	0	2
GALK1	chr17	73754580/G//A	synonymous	0	2
SMCHD1	chr18	2656092/C//T	synonymous	0	2
MUC16	chr19	9056490/G//A	nonsynonymous	0	2
ZNF333	chr19	14826225/G//A	synonymous	0	2
BRD4	chr19	15349565/G//A	nonsynonymous	0	2
F2RL3	chr19	17001255/G//A	synonymous	0	2
NUCB1	chr19	49422359/C//T	nonsynonymous	0	2
FPR1	chr19	52249323/G//A	nonsynonymous	0	2
PRPF31	chr19	54625280/G//A	nonsynonymous	0	2
TTI1	chr20	36627645/G//A	nonsynonymous	0	2
TRPM2	chr21	45819208/G//A	nonsynonymous	0	2
	chr21	47549065/C//T	synonymous	0	2
GNB1L	chr22	19776207/G//A	synonymous	0	2
DCAF8L2	chrX	27764983/C//A	synonymous	0	2
DMD	chrX	31838186/A//T	nonsynonymous	0	2
BCOR	chrX	39932471/G//T	nonsynonymous	0	2
SUV39H1	chrX	48564697/C//T	synonymous	0	2
IL2RG	chrX	70330763/G//A	nonsynonymous	0	2
TEX13A	chrX	104464446/G//T	synonymous	0	2
ADGRG4	chrX	135428487/A//G	synonymous	0	2
	chrX	140985378/G//C	synonymous	0	2
VMA21	chrX	150573447/G//A	nonsynonymous	0	2
DKC1	chrX	153996651/G//A	nonsynonymous	0	2
CCDC62	chr12	123276585/C//T	nonsynonymous	1	1
IPO4	chr14	24656649/C//T	nonsynonymous	2	0
B2M	chr15	45003780/ACT//A	nonsynonymous	2	0
SPOCD1	chr1	32259446/G//A	synonymous	0	2
THRAP3	chr1	36769666/A//G	synonymous	0	2
ERI3	chr1	44818519/C//T	synonymous	0	2
C1orf194	chr1	109650569/G//T	synonymous	0	2
FLG	chr1	152282957/G//A	nonsynonymous	0	2
NDUFS2	chr1	161173279/G//T	nonsynonymous	0	2
CR1	chr1	207679428/C//T	nonsynonymous	0	2
USP34	chr2	61441207/G//C	nonsynonymous	0	2
ALMS1	chr2	73677252/A//G	nonsynonymous	0	2
TFCP2L1	chr2	122004454/G//A	synonymous	0	2
LRP1B	chr2	141806738/T//A	nonsynonymous	0	2
TTN	chr2	179586849/G//T	nonsynonymous	0	2
STAT4	chr2	191927606/G//T	nonsynonymous	0	2
HTR2B	chr2	231973637/A//T	nonsynonymous	0	2
DOCK3	chr3	51102008/C//T	nonsynonymous	0	2
NPFFR2	chr4	72897735/C//T	synonymous	0	2
PKHD1	chr6	51890825/G//A	synonymous	0	2
GIMAP6	chr7	150325329/G//A	synonymous	0	2
CHD7	chr8	61775170/T//C	nonsynonymous	0	2
TRIM55	chr8	67039510/G//A	nonsynonymous	0	2
STMN2	chr8	80553707/T//C	synonymous	0	2
RBM20	chr10	112541506/G//A	nonsynonymous	0	2
PGGHG	chr11	290448/G//A	synonymous	0	2
MUC6	chr11	1016825/G//A	synonymous	0	2
OR51G2	chr11	4936134/G//A	synonymous	0	2
OR4D9	chr11	59282741/C//T	nonsynonymous	0	2
MYRF	chr11	61543834/G//T	nonsynonymous	0	2
FGF4	chr11	69588117/G//A	nonsynonymous	0	2
ANKS1B	chr12	99192714/G//A	nonsynonymous	0	2
AMFR	chr16	56436976/G//A	nonsynonymous	0	2
CES4A	chr16	67035231/G//A	nonsynonymous	0	2
SLFN12L	chr17	33802248/G//C	nonsynonymous	0	2
CWC25	chr17	36969097/T//A	nonsynonymous	0	2

DLGAP1	chr18	3742478/G//A	nonsynonymous	0	2
MTCL1	chr18	8720329/G//T	synonymous	0	2
MAPRE2	chr18	32681969/A//G	synonymous	0	2
SOCS6	chr18	67993111/G//T	nonsynonymous	0	2
LRRC8E	chr19	7965308/C//T	nonsynonymous	0	2
PDE4A	chr19	10574591/G//T	nonsynonymous	0	2
ZNF433	chr19	12127088/A//C	nonsynonymous	0	2
NLRP2	chr19	55481398/G//A	synonymous	0	2
XKR7	chr20	30584971/G//A	nonsynonymous	0	2
FHL1	chrX	135292128/C//T	synonymous	0	2
ARHGAP30	chr1	161039369/G//A	nonsynonymous	0	2
ATAD2B	chr2	24110706/T//C	nonsynonymous	0	2
CACNB4	chr2	152695685/C//T	nonsynonymous	0	2
KCNJ13	chr2	233632864/G//A	synonymous	0	2
REV3L	chr6	111696035/T//C	nonsynonymous	0	2
SYNE1	chr6	152779939/G//A	nonsynonymous	0	2
FBXL6	chr8	145579262/G//A	nonsynonymous	0	2
KLF9	chr9	73028188/G//A	nonsynonymous	0	2
USP5	chr12	6967622/T//C	nonsynonymous	0	2
IL23A	chr12	56733499/G//T	nonsynonymous	0	2
MRPL42	chr12	93895019/C//G	synonymous	0	2
	chr13	76195725/A//T	synonymous	0	2
ZFYVE26	chr14	68252866/T//C	nonsynonymous	0	2
ZFYVE1	chr14	73464830/T//C	nonsynonymous	0	2
ONECUT1	chr15	53049839/T//C	synonymous	0	2
ESRP2	chr16	68266723/G//T	synonymous	0	2
APOH	chr17	64208259/G//C	nonsynonymous	0	2
DGKK	chrX	50122626/G//A	nonsynonymous	0	2
POTEF	chr2	130877704/T//G	nonsynonymous	0	2
HRNR	chr1	152192293/C//T	synonymous	0	2
CEP104	chr1	3747752/T//C	nonsynonymous	0	2
CNKSR1	chr1	26515860/C//T	nonsynonymous	0	2
TAF1A	chr1	222734718/G//C	nonsynonymous	0	2
SCN9A	chr2	167128931/G//A	nonsynonymous	0	2
LRP2	chr2	170063767/G//T	synonymous	0	2
SCN10A	chr3	38739124/G//A	nonsynonymous	0	2
SCN10A	chr3	38830471/C//T	nonsynonymous	0	2
NISCH	chr3	52521374/C//T	synonymous	0	2
JAKMIP1	chr4	6064101/G//A	nonsynonymous	0	2
MEPE	chr4	88766977/T//C	synonymous	0	2
APC	chr5	112174144/T//G	nonsynonymous	0	2
PCDHA3	chr5	140182596/C//T	nonsynonymous	0	2
PCDHB16	chr5	140563551/G//A	nonsynonymous	0	2
MED7	chr5	156566017/C//T	synonymous	0	2
OR2B2	chr6	27879902/G//T	nonsynonymous	0	2
C6orf25	chr6	31691750/C//T	synonymous	0	2
RPS10-NUDT3	chr6	34392507/C//T	synonymous	0	2
LAMA4	chr6	112437107/C//T	nonsynonymous	0	2
UTRN	chr6	144835076/G//T	nonsynonymous	0	2
TTLL2	chr6	167754158/G//A	nonsynonymous	0	2
PAPOLB	chr7	4901388/C//T	synonymous	0	2
AGBL3	chr7	134719061/G//A	nonsynonymous	0	2
ZFHX4	chr8	77764394/C//T	nonsynonymous	0	2
TG	chr8	133899114/A//G	synonymous	0	2
COL22A1	chr8	139626111/G//A	nonsynonymous	0	2
KCNK9	chr8	140630787/C//T	nonsynonymous	0	2
LRRC24	chr8	145749933/C//T	synonymous	0	2
PTPRD	chr9	8404615/C//T	nonsynonymous	0	2
TRPM6	chr9	77354788/G//A	nonsynonymous	0	2
PRUNE2	chr9	79324251/C//A	nonsynonymous	0	2
TLL2	chr10	98146806/C//T	nonsynonymous	0	2
GFRA1	chr10	117849314/C//G	nonsynonymous	0	2
CHST15	chr10	125771945/C//T	nonsynonymous	0	2
BAD	chr11	64051848/G//A	synonymous	0	2
FAT3	chr11	92532289/C//T	nonsynonymous	0	2
SLC6A12	chr12	308071/C//T	synonymous	0	2
ARID2	chr12	46233112/A//T	nonsynonymous	0	2
CCER1	chr12	91348460/G//A	synonymous	0	2
TMEM132C	chr12	129190945/G//A	synonymous	0	2
P2RX5-TAX1BP3	chr17	3582877/G//A	synonymous	0	2

DNAH9	chr17	11572805/T//A	nonsynonymous	0	2
NFATC1	chr18	77227587/C//T	synonymous	0	2
HCN2	chr19	613304/C//T	synonymous	0	2
CYP4F22	chr19	15648408/G//A	nonsynonymous	0	2
NDUFA13	chr19	19638564/C//T	synonymous	0	2
	chr19	36277510/G//A	synonymous	0	2
LRRC4B	chr19	51021383/G//A	synonymous	0	2
SLC17A9	chr20	61594112/G//A	synonymous	0	2
BAIAP2L2	chr22	38485118/G//A	nonsynonymous	0	2
COL4A6	chrX	107449792/C//T	nonsynonymous	0	2
AMOT	chrX	112033819/C//T	nonsynonymous	0	2
MAGEA6	chrX	151870313/C//A	synonymous	0	2
CDC27	chr17	45234416/A//G	synonymous	0	2
OR2T33	chr1	248436809/G//A	nonsynonymous	0	2
OR2T33	chr1	248436811/G//C	synonymous	0	2
ZNF678	chr1	227843225/G//T	nonsynonymous	1	1
VWA1	chr1	1372301/C//T	synonymous	0	2
RLF	chr1	40703484/G//A	nonsynonymous	0	2
STIL	chr1	47770581/G//A	synonymous	0	2
KCND3	chr1	112525070/G//A	synonymous	0	2
CELF3	chr1	151681524/C//T	nonsynonymous	0	2
MEX3A	chr1	156046736/C//T	nonsynonymous	0	2
NES	chr1	156642952/C//T	nonsynonymous	0	2
OR10J3	chr1	159284326/C//CAG	nonsynonymous	0	2
ERLEC1	chr2	54028682/C//G	nonsynonymous	0	2
ZNF638	chr2	71592732/A//T	nonsynonymous	0	2
KLF7	chr2	207988838/G//A	synonymous	0	2
UNC80	chr2	210698894/T//A	nonsynonymous	0	2
GPBAR1	chr2	219128027/C//T	nonsynonymous	0	2
CHPF	chr2	220405256/G//A	nonsynonymous	0	2
SP140	chr2	231109759/G//T	nonsynonymous	0	2
CRBN	chr3	3192468/A//T	synonymous	0	2
LRRN1	chr3	3888295/A//G	nonsynonymous	0	2
DAG1	chr3	49568841/C//T	synonymous	0	2
PLXND1	chr3	129289738/C//T	nonsynonymous	0	2
COL6A5	chr3	130124863/G//T	nonsynonymous	0	2
EIF4G1	chr3	184039835/G//C	nonsynonymous	0	2
PCDH7	chr4	30724715/G//A	synonymous	0	2
KIAA1211	chr4	57180528/C//T	nonsynonymous	0	2
UGT2B7	chr4	69962369/A//G	nonsynonymous	0	2
OTUD4	chr4	146059506/T//C	synonymous	0	2
ADAMTS16	chr5	5235263/C//T	nonsynonymous	0	2
	chr5	67584573/C//T	synonymous	0	2
PCDHB12	chr5	140590068/G//A	nonsynonymous	0	2
SH3TC2	chr5	148407374/G//A	nonsynonymous	0	2
CSNK1A1	chr5	148885135/T//C	nonsynonymous	0	2
EBF1	chr5	158250178/G//A	synonymous	0	2
POM121L2	chr6	27279595/T//C	nonsynonymous	0	2
COL9A1	chr6	70942343/G//A	nonsynonymous	0	2
HBS1L	chr6	135371765/A//G	nonsynonymous	0	2
MAP3K4	chr6	161470370/C//T	nonsynonymous	0	2
	chr7	50560546/G//A	synonymous	0	2
CPA1	chr7	130021978/G//A	synonymous	0	2
DLGAP2	chr8	1497287/G//C	nonsynonymous	0	2
XKR6	chr8	10782308/C//T	nonsynonymous	0	2
SPIDR	chr8	48614334/C//G	nonsynonymous	0	2
MVB12B	chr9	129154466/G//A	synonymous	0	2
TNKS2	chr10	93579041/G//C	nonsynonymous	0	2
EXOSC1	chr10	99205716/G//T	synonymous	0	2
CYP2E1	chr10	135347330/C//T	nonsynonymous	0	2
IFITM10	chr11	1769016/C//T	nonsynonymous	0	2
FAM160A2	chr11	6243709/A//G	nonsynonymous	0	2
SAA4	chr11	18253070/G//A	synonymous	0	2
DCDC5	chr11	30914468/G//A	nonsynonymous	0	2
OR5D18	chr11	55587824/C//T	nonsynonymous	0	2
FAT3	chr11	92088223/G//T	nonsynonymous	0	2
ATM	chr11	108186549/G//T	nonsynonymous	0	2
SPA17	chr11	124564229/G//A	nonsynonymous	0	2
A2ML1	chr12	9009766/T//C	nonsynonymous	0	2
ABCD2	chr12	39947836/G//C	nonsynonymous	0	2

MVK	chr12	110034316/C//T	synonymous	0	2
TMEM132C	chr12	129153985/C//T	synonymous	0	2
EP400	chr12	132476772/G//A	nonsynonymous	0	2
SLC7A1	chr13	30091337/C//T	nonsynonymous	0	2
ZC3H13	chr13	46554110/C//G	nonsynonymous	0	2
RBM23	chr14	23374865/A//G	synonymous	0	2
SYT16	chr14	62550996/C//T	nonsynonymous	0	2
RTL1	chr14	101349752/C//T	synonymous	0	2
ITPRIPL2	chr16	19126865/G//A	nonsynonymous	0	2
ZNF469	chr16	88504587/C//T	nonsynonymous	0	2
NCOR1	chr17	15978998/G//A	nonsynonymous	0	2
SEZ6	chr17	27308787/C//T	nonsynonymous	0	2
MAP2K6	chr17	67522793/C//T	nonsynonymous	0	2
TSEN54	chr17	73513673/G//T	synonymous	0	2
SNAPC2	chr19	7987488/G//A	nonsynonymous	0	2
IL27RA	chr19	14163015/G//A	synonymous	0	2
ZNF506	chr19	19906386/C//T	nonsynonymous	0	2
C19orf47	chr19	40842279/C//T	nonsynonymous	0	2
MYT1	chr20	62839165/G//T	nonsynonymous	0	2
DSCAM	chr21	41446994/G//A	nonsynonymous	0	2
GRAP2	chr22	40343056/C//G	synonymous	0	2
ARSA	chr22	51065135/G//A	synonymous	0	2
GTPBP6	chrX	228205/C//T	nonsynonymous	0	2
MXRA5	chrX	3240691/G//T	nonsynonymous	0	2
CYP2A6	chr19	41349774/A//G	nonsynonymous	0	2
TSC1	chr9	135772717/G//T	synonymous	1	1
PLEKHM2	chr1	16060481/G//A	synonymous	0	2
UBR4	chr1	19504100/G//A	nonsynonymous	0	2
PQLC2	chr1	19651151/G//A	synonymous	0	2
CYP4B1	chr1	47276533/G//A	synonymous	0	2
DPYD	chr1	98039493/G//A	synonymous	0	2
RNPC3	chr1	104085918/C//T	synonymous	0	2
OR6K2	chr1	158669761/G//A	nonsynonymous	0	2
CACNA1S	chr1	201046260/A//C	synonymous	0	2
HIST3H3	chr1	228613003/C//T	synonymous	0	2
VN1R5	chr1	247420049/C//T	nonsynonymous	0	2
OR2AK2	chr1	248129043/G//A	nonsynonymous	0	2
VIT	chr2	37035794/G//A	synonymous	0	2
STON1-GTF2A1L	chr2	48872258/T//TGA	synonymous	0	2
TMEM127	chr2	96919488/C//T	synonymous	0	2
SCN1A	chr2	166848851/C//T	nonsynonymous	0	2
ITGAV	chr2	187534463/G//T	synonymous	0	2
FBXL2	chr3	33416825/C//T	nonsynonymous	0	2
LARS2	chr3	45557610/C//T	nonsynonymous	0	2
BOC	chr3	112998253/C//T	synonymous	0	2
LRRC66	chr4	52883346/T//C	nonsynonymous	0	2
CCNI2	chr5	132088655/C//T	nonsynonymous	0	2
PCDHA8	chr5	140222356/G//A	nonsynonymous	0	2
PCDHGC4	chr5	140864967/G//A	nonsynonymous	0	2
ANXA6	chr5	150502565/G//A	synonymous	0	2
GRM1	chr6	146755313/C//T	nonsynonymous	0	2
WBSCR17	chr7	70597632/G//A	synonymous	0	2
PPP1R9A	chr7	94916437/G//C	nonsynonymous	0	2
MUC12	chr7	100645731/G//A	nonsynonymous	0	2
	chr7	101916640/G//A	synonymous	0	2
SSPO	chr7	149490536/C//T	synonymous	0	2
CSMD1	chr8	2819985/T//C	synonymous	0	2
	chr8	32505800/G//A	synonymous	0	2
SLA	chr8	134050937/G//A	synonymous	0	2
TMEM215	chr9	32784629/G//A	nonsynonymous	0	2
NOL8	chr9	95080904/C//T	nonsynonymous	0	2
RPL12	chr9	130213057/C//G	nonsynonymous	0	2
STXBP1	chr9	130438960/C//T	synonymous	0	2
CFAP157	chr9	130471944/C//T	synonymous	0	2
FAM166A	chr9	140138248/G//C	synonymous	0	2
IDI2	chr10	1070629/C//T	nonsynonymous	0	2
PCDH15	chr10	55591209/A//T	synonymous	0	2
PCDH15	chr10	55591256/A//T	nonsynonymous	0	2
CALHM2	chr10	105209293/C//T	nonsynonymous	0	2
ADAM12	chr10	127753439/G//A	synonymous	0	2

DCDC1	chr11	31312220/T//C	nonsynonymous	0	2
OR4B1	chr11	48238977/G//T	nonsynonymous	0	2
ZFP91	chr11	58378459/A//T	nonsynonymous	0	2
CCDC83	chr11	85593563/G//A	nonsynonymous	0	2
ATM	chr11	108224583/C//T	nonsynonymous	0	2
DSCAML1	chr11	117376243/T//C	nonsynonymous	0	2
ABCG4	chr11	119031669/G//A	synonymous	0	2
OVCH1	chr12	29630406/C//T	nonsynonymous	0	2
PRICKLE1	chr12	42858944/CCT//C	nonsynonymous	0	2
OR6C65	chr12	55794795/C//A	synonymous	0	2
ZFC3H1	chr12	72027136/C//T	nonsynonymous	0	2
VEZT	chr12	95660352/G//A	synonymous	0	2
ACACB	chr12	109704198/C//T	synonymous	0	2
HECTD4	chr12	112614503/C//A	nonsynonymous	0	2
SRRM4	chr12	119583478/G//A	nonsynonymous	0	2
PCDH17	chr13	58299162/T//G	nonsynonymous	0	2
CCDC168	chr13	103391600/T//C	nonsynonymous	0	2
TUBGCP3	chr13	113209243/G//A	synonymous	0	2
NPAS3	chr14	34145574/C//T	nonsynonymous	0	2
CHRNA7	chr15	32460293/C//T	synonymous	0	2
TICRR	chr15	90167851/G//A	nonsynonymous	0	2
KIF7	chr15	90185440/C//A	nonsynonymous	0	2
HMOX2	chr16	4558191/G//A	nonsynonymous	0	2
MMP15	chr16	58076239/C//T	synonymous	0	2
ALDOC	chr17	26901752/C//T	nonsynonymous	0	2
SDK2	chr17	71431640/C//T	nonsynonymous	0	2
ZNF699	chr19	9408062/T//A	nonsynonymous	0	2
ATP4A	chr19	36050068/C//T	nonsynonymous	0	2
OVOL2	chr20	18022244/A//T	nonsynonymous	0	2
TP53TG5	chr20	44003875/C//T	nonsynonymous	0	2
HELZ2	chr20	62197474/C//T	nonsynonymous	0	2
ASPHD2	chr22	26839195/G//A	synonymous	0	2
C22orf23	chr22	38347483/G//C	nonsynonymous	0	2
OPHN1	chrX	67454376/G//A	nonsynonymous	0	2
PIK3CA	chr3	178927980/T//C	nonsynonymous	1	1
DMRT2	chr9	1053820/A//C	nonsynonymous	0	2
RTEL1	chr20	62293280/C//T	nonsynonymous	0	2
MUC12	chr7	100646938/C//G	nonsynonymous	0	2
KRTAP9-4	chr17	39406104/T//G	synonymous	0	2
ATP1A2	chr1	160097481/C//T	synonymous	0	2
TRIB2	chr2	12880934/G//A	synonymous	0	2
SH3BP5	chr3	15300453/G//A	synonymous	0	2
FASTKD3	chr5	7867835/G//A	nonsynonymous	0	2
ANKH	chr5	14716853/A//C	nonsynonymous	0	2
CAPSL	chr5	35921153/C//T	nonsynonymous	0	2
PCDHB7	chr5	140554097/G//A	nonsynonymous	0	2
ADAMTS2	chr5	178608112/G//A	synonymous	0	2
PCLO	chr7	82581607/C//T	nonsynonymous	0	2
ANK3	chr10	61844457/G//A	nonsynonymous	0	2
CCAR1	chr10	70509310/C//T	nonsynonymous	0	2
MTA2	chr11	62363224/C//T	synonymous	0	2
BSX	chr11	122848369/C//T	synonymous	0	2
PPM1E	chr17	57060270/C//G	synonymous	0	2
SOX9	chr17	70118890/C//A	nonsynonymous	0	2
ZNF521	chr18	22806812/G//A	nonsynonymous	0	2
ZNF418	chr19	58439004/G//C	nonsynonymous	0	2
WISP2	chr20	43355826/G//A	nonsynonymous	0	2
ZNF831	chr20	57767002/G//A	nonsynonymous	0	2
KIAA1644	chr22	44681488/C//T	nonsynonymous	0	2
CLCN4	chrX	10174547/C//A	synonymous	0	2
ARID1A	chr1	27105601/G//GACCCAGGACAGA(	nonsynonymous	0	2
ARID1A	chr1	27105970/A//G	nonsynonymous	0	2
SLC35D1	chr1	67516120/G//A	nonsynonymous	0	2
HS2ST1	chr1	87570287/A//T	nonsynonymous	0	2
ZNF644	chr1	91406333/T//A	nonsynonymous	0	2
PRMT6	chr1	107600343/G//T	nonsynonymous	0	2
OTUD7B	chr1	149916870/C//G	nonsynonymous	0	2
BNIPL	chr1	151016067/C//A	synonymous	0	2
ZNF687	chr1	151259041/C//G	nonsynonymous	0	2
SPRR2D	chr1	153012487/G//A	synonymous	0	2

CD5L	chr1	157803114/G//A	nonsynonymous	0	2
PRRC2C	chr1	171506526/T//C	synonymous	0	2
SEC16B	chr1	177930817/C//T	nonsynonymous	0	2
LAMC1	chr1	183077481/A//G	nonsynonymous	0	2
BRINP3	chr1	190067013/G//C	synonymous	0	2
KDM5B	chr1	202701071/A//T	synonymous	0	2
HHIPL2	chr1	222716878/C//A	nonsynonymous	0	2
TLR5	chr1	223284172/A//T	nonsynonymous	0	2
CDC42BPA	chr1	227261594/T//A	nonsynonymous	0	2
RYR2	chr1	237889609/G//T	nonsynonymous	0	2
NLRP3	chr1	247597425/C//T	nonsynonymous	0	2
OR2T1	chr1	248569427/T//C	synonymous	0	2
OR2T1	chr1	248569712/A//G	synonymous	0	2
SDC1	chr2	20402614/G//A	synonymous	0	2
FSHR	chr2	49190888/C//G	nonsynonymous	0	2
NRXN1	chr2	51149809/C//G	nonsynonymous	0	2
CCDC88A	chr2	55544911/G//A	nonsynonymous	0	2
PUS10	chr2	61175277/C//T	nonsynonymous	0	2
DYSF	chr2	71892374/C//A	nonsynonymous	0	2
ANKRD36	chr2	97877417/C//G	nonsynonymous	0	2
UXS1	chr2	106739477/G//A	synonymous	0	2
ACMSD	chr2	135619550/C//G	nonsynonymous	0	2
LRP1B	chr2	141739815/C//A	nonsynonymous	0	2
SCN1A	chr2	166894610/T//C	synonymous	0	2
NFE2L2	chr2	178098944/C//G	nonsynonymous	0	2
TTN	chr2	179483201/G//T	nonsynonymous	0	2
ABCB6	chr2	220074542/C//T	synonymous	0	2
DOCK10	chr2	225666640/G//T	synonymous	0	2
TWIST2	chr2	239757169/C//T	nonsynonymous	0	2
CHL1	chr3	425528/G//T	nonsynonymous	0	2
NUP210	chr3	13417919/C//G	nonsynonymous	0	2
GPR15	chr3	98251419/C//T	nonsynonymous	0	2
KALRN	chr3	124437811/C//G	nonsynonymous	0	2
ALDH1L1	chr3	125849077/C//T	synonymous	0	2
TRPC1	chr3	142443488/C//G	nonsynonymous	0	2
NLGN1	chr3	173998359/C//G	nonsynonymous	0	2
CCDC39	chr3	180364876/C//G	nonsynonymous	0	2
AFAP1	chr4	7873749/A//G	synonymous	0	2
KIAA1109	chr4	123095795/A//T	nonsynonymous	0	2
GPM6A	chr4	176556026/T//A	synonymous	0	2
IRF2	chr4	185339327/G//GTGCTTAACTTTG[illegible]	nonsynonymous	0	2
LRRC14B	chr5	195330/C//T	synonymous	0	2
DNAH5	chr5	13727761/G//A	nonsynonymous	0	2
C5orf42	chr5	37120365/T//C	nonsynonymous	0	2
FGF10	chr5	44310536/T//C	nonsynonymous	0	2
ENC1	chr5	73930728/C//A	nonsynonymous	0	2
DMGDH	chr5	78359526/C//A	synonymous	0	2
LRRTM2	chr5	138209756/C//T	nonsynonymous	0	2
PCDHA5	chr5	140201654/G//C	nonsynonymous	0	2
PCDHA12	chr5	140257229/A//T	synonymous	0	2
PCDHGA2	chr5	140719343/G//T	nonsynonymous	0	2
FAT2	chr5	150923673/AATCC//A	nonsynonymous	0	2
SGCD	chr5	156016263/C//G	nonsynonymous	0	2
NEURL1B	chr5	172097297/G//A	nonsynonymous	0	2
KIF13A	chr6	17850576/G//A	nonsynonymous	0	2
SLC17A2	chr6	25921623/C//A	nonsynonymous	0	2
B3GALT4	chr6	33245316/G//A	synonymous	0	2
SCUBE3	chr6	35182162/G//C	synonymous	0	2
ADGRF5	chr6	46846090/C//A	nonsynonymous	0	2
ADGRB3	chr6	70071191/C//A	nonsynonymous	0	2
ZNF292	chr6	87926095/G//T	nonsynonymous	0	2
ZNF292	chr6	87968755/A//T	nonsynonymous	0	2
HACE1	chr6	105192060/G//C	nonsynonymous	0	2
MAN1A1	chr6	119510982/C//A	nonsynonymous	0	2
TBC1D32	chr6	121481167/C//A	nonsynonymous	0	2
GJA1	chr6	121768065/G//T	synonymous	0	2
ECHDC1	chr6	127611343/G//A	synonymous	0	2
	chr6	135518099/G//A	synonymous	0	2
AKAP12	chr6	151672372/C//T	nonsynonymous	0	2
DNAAF5	chr7	796442/C//T	synonymous	0	2

ABCB5	chr7	20782651/C//T	nonsynonymous	0	2
HOXA3	chr7	27147985/G//A	nonsynonymous	0	2
PSMA2	chr7	42964290/C//G	nonsynonymous	0	2
ADCY1	chr7	45719357/G//A	nonsynonymous	0	2
ZNF736	chr7	63809190/G//A	nonsynonymous	0	2
CRCP	chr7	65610434/G//C	nonsynonymous	0	2
BAZ1B	chr7	72879800/C//T	nonsynonymous	0	2
DNAJC30	chr7	73097423/C//T	nonsynonymous	0	2
SEMA3E	chr7	83022004/C//A	nonsynonymous	0	2
MUC12	chr7	100635924/G//C	nonsynonymous	0	2
MUC12	chr7	100639173/G//T	nonsynonymous	0	2
DENND2A	chr7	140301681/C//A	nonsynonymous	0	2
MGAM	chr7	141721445/T//A	nonsynonymous	0	2
CNTNAP2	chr7	146805445/GTGTTCTGAC//G	synonymous	0	2
GIMAP7	chr7	150217384/G//T	nonsynonymous	0	2
WDR60	chr7	158716333/C//T	synonymous	0	2
FER1L6	chr8	124989684/G//A	nonsynonymous	0	2
GNAQ	chr9	80343579/C//T	nonsynonymous	0	2
MFSD14B	chr9	97216303/G//T	nonsynonymous	0	2
TRIM32	chr9	119460300/G//A	synonymous	0	2
TRAF1	chr9	123685978/C//A	synonymous	0	2
SPTAN1	chr9	131381207/G//C	nonsynonymous	0	2
PARD3	chr10	34606204/G//A	nonsynonymous	0	2
CHAT	chr10	50863203/G//T	nonsynonymous	0	2
PANK1	chr10	91344155/G//T	synonymous	0	2
DENND5A	chr11	9161193/C//G	synonymous	0	2
COMMD9	chr11	36300094/C//A	nonsynonymous	0	2
VWCE	chr11	61026625/T//C	nonsynonymous	0	2
EHBP1L1	chr11	65351795/C//T	synonymous	0	2
C11orf24	chr11	68030122/G//A	nonsynonymous	0	2
MSANTD4	chr11	105880290/C//A	nonsynonymous	0	2
DIXDC1	chr11	111863143/C//A	nonsynonymous	0	2
DDX6	chr11	118633971/T//C	nonsynonymous	0	2
FOXR1	chr11	118849839/G//A	synonymous	0	2
	chr12	9162198/C//T	synonymous	0	2
LRRK2	chr12	40681212/G//C	nonsynonymous	0	2
PDZRN4	chr12	41967501/A//T	nonsynonymous	0	2
PFKM	chr12	48528805/C//T	nonsynonymous	0	2
KRT73	chr12	53009113/C//G	nonsynonymous	0	2
LRP1	chr12	57605541/C//T	nonsynonymous	0	2
LRIG3	chr12	59266449/T//A	nonsynonymous	0	2
WIF1	chr12	65462568/T//C	nonsynonymous	0	2
TPH2	chr12	72335519/T//C	synonymous	0	2
SLC6A15	chr12	85279727/C//T	nonsynonymous	0	2
MYO1H	chr12	109831146/G//A	nonsynonymous	0	2
ATP2A2	chr12	110719673/G//T	nonsynonymous	0	2
TAOK3	chr12	118673480/T//C	synonymous	0	2
TMEM132D	chr12	130184843/G//A	synonymous	0	2
PRMT5	chr14	23392361/C//T	nonsynonymous	0	2
EIF2S1	chr14	67850094/A//C	nonsynonymous	0	2
APBA2	chr15	29346594/G//T	nonsynonymous	0	2
STRC	chr15	43896890/G//A	nonsynonymous	0	2
PLEKHO2	chr15	65157293/G//T	nonsynonymous	0	2
CCDC33	chr15	74623065/G//T	synonymous	0	2
SCAPER	chr15	76646320/C//T	nonsynonymous	0	2
SLC28A1	chr15	85478634/T//C	nonsynonymous	0	2
CLUAP1	chr16	3558304/A//T	nonsynonymous	0	2
VASN	chr16	4432309/C//T	synonymous	0	2
ZNF500	chr16	4802874/G//A	nonsynonymous	0	2
ACSM2B	chr16	20576092/T//G	nonsynonymous	0	2
KCTD13	chr16	29923187/G//C	nonsynonymous	0	2
ZNF646	chr16	31089024/T//A	nonsynonymous	0	2
BCKDK	chr16	31123520/C//T	synonymous	0	2
IRX3	chr16	54318187/G//C	synonymous	0	2
NUP93	chr16	56792525/C//T	synonymous	0	2
FANCA	chr16	89807252/A//T	nonsynonymous	0	2
SUPT6H	chr17	27011601/C//G	synonymous	0	2
GAS2L2	chr17	34073409/G//C	nonsynonymous	0	2
RAPGEFL1	chr17	38347691/G//A	nonsynonymous	0	2
NOG	chr17	54672271/G//C	nonsynonymous	0	2

BRIP1	chr17	59926496/T//C	synonymous	0	2
CCDC102B	chr18	66504364/C//A	nonsynonymous	0	2
APC2	chr19	1460800/G//A	nonsynonymous	0	2
ICAM4	chr19	10397957/C//T	nonsynonymous	0	2
CYP4F11	chr19	16038057/T//A	nonsynonymous	0	2
SLC27A1	chr19	17611395/G//GATCGCCC	nonsynonymous	0	2
KIAA1683	chr19	18380336/C//T	synonymous	0	2
ZNF100	chr19	21910152/T//A	nonsynonymous	0	2
ATP4A	chr19	36051416/G//C	synonymous	0	2
LRFN3	chr19	36435557/C//T	nonsynonymous	0	2
GLTSCR1	chr19	48198221/C//A	nonsynonymous	0	2
ETFB	chr19	51869586/C//A	synonymous	0	2
ZNF480	chr19	52825031/C//G	synonymous	0	2
ZNF480	chr19	52825979/C//T	synonymous	0	2
ZNF160	chr19	53572094/A//C	nonsynonymous	0	2
CACNG6	chr19	54515274/C//T	nonsynonymous	0	2
KIR2DL4	chr19	55325335/A//T	synonymous	0	2
NLRP4	chr19	56379095/C//T	nonsynonymous	0	2
ZFP28	chr19	57059217/C//G	nonsynonymous	0	2
ZNF256	chr19	58452652/T//A	nonsynonymous	0	2
TMPRSS15	chr21	19687493/A//C	nonsynonymous	0	2
DSCAM	chr21	41505937/C//A	nonsynonymous	0	2
	chr21	47966983/G//T	synonymous	0	2
SLC7A4	chr22	21384630/G//T	synonymous	0	2
MYO18B	chr22	26423196/T//C	nonsynonymous	0	2
HPS4	chr22	26860432/G//C	synonymous	0	2
CRYBA4	chr22	27021525/G//T	nonsynonymous	0	2
PPARA	chr22	46611128/C//T	synonymous	0	2
SSX1	chrX	48117228/G//T	nonsynonymous	0	2
STARD8	chrX	67943857/G//A	nonsynonymous	0	2
GABRA3	chrX	151358237/C//T	nonsynonymous	0	2
PRR36	chr19	7935904/C//T	synonymous	0	2
ZMYM6	chr1	35452689/A//T	synonymous	0	2
CDC14A	chr1	100819342/G//A	nonsynonymous	0	2
ATP1A2	chr1	160093812/A//C	nonsynonymous	0	2
COPA	chr1	160303427/G//A	nonsynonymous	0	2
IGFN1	chr1	201181634/C//A	nonsynonymous	0	2
LRP1B	chr2	142004815/C//G	nonsynonymous	0	2
SPEG	chr2	220346067/C//T	nonsynonymous	0	2
NMUR1	chr2	232393209/G//A	nonsynonymous	0	2
BTD	chr3	15686990/G//C	nonsynonymous	0	2
C3orf30	chr3	118865366/G//A	synonymous	0	2
TRH	chr3	129694770/G//A	synonymous	0	2
ZIC4	chr3	147108954/G//A	synonymous	0	2
DRD5	chr4	9784102/G//A	nonsynonymous	0	2
	chr5	134028852/G//A	synonymous	0	2
ADAMTS2	chr5	178585812/G//C	synonymous	0	2
FAM50B	chr6	3850366/G//A	synonymous	0	2
DAXX	chr6	33287481/G//A	nonsynonymous	0	2
BTBD9	chr6	38142862/C//T	nonsynonymous	0	2
PTPRK	chr6	128330321/C//A	nonsynonymous	0	2
MMP16	chr8	89198809/T//C	synonymous	0	2
PCSK5	chr9	78973641/T//G	nonsynonymous	0	2
COQ4	chr9	131094451/G//A	nonsynonymous	0	2
ANKRD16	chr10	5931047/G//C	nonsynonymous	0	2
KCNMA1	chr10	78709082/C//T	nonsynonymous	0	2
IFIT5	chr10	91177038/G//A	nonsynonymous	0	2
	chr10	100189321/G//A	synonymous	0	2
DMBT1	chr10	124336178/C//T	nonsynonymous	0	2
OR8H1	chr11	56058235/A//C	nonsynonymous	0	2
TMEM151A	chr11	66061833/G//A	nonsynonymous	0	2
LRRC32	chr11	76371084/C//T	nonsynonymous	0	2
CCDC15	chr11	124829104/G//T	nonsynonymous	0	2
FOXRED1	chr11	126142927/A//T	nonsynonymous	0	2
SMAD9	chr13	37453645/C//A	nonsynonymous	0	2
EPB42	chr15	43507442/A//G	nonsynonymous	0	2
HAPLN3	chr15	89424919/G//A	synonymous	0	2
ADAMTS17	chr15	100695429/G//A	synonymous	0	2
IFT140	chr16	1621437/A//G	synonymous	0	2
KRTAP2-4	chr17	39222021/C//T	nonsynonymous	0	2

HSF5	chr17	56565479/G//T	nonsynonymous	0	2
TTYH2	chr17	72218654/G//A	nonsynonymous	0	2
POTEC	chr18	14542786/C//T	nonsynonymous	0	2
NOTCH3	chr19	15281362/C//A	nonsynonymous	0	2
FOSB	chr19	45975949/G//A	synonymous	0	2
GPCPD1	chr20	5574004/C//T	nonsynonymous	0	2
BPIFB1	chr20	31876575/C//T	synonymous	0	2
TPST2	chr22	26937597/G//A	synonymous	0	2
TEF	chr22	41783512/G//A	synonymous	0	2
TRO	chrX	54957032/G//T	nonsynonymous	0	2
TRPC5OS	chrX	111145167/C//A	nonsynonymous	0	2
TENM1	chrX	123680906/A//G	synonymous	0	2
PDZD4	chrX	153072259/G//T	nonsynonymous	0	2
CACNA1E	chr1	181725204/G//A	nonsynonymous	0	2
RABIF	chr1	202858196/C//G	nonsynonymous	0	2
HHAT	chr1	210577873/G//C	synonymous	0	2
HHAT	chr1	210577884/G//A	nonsynonymous	0	2
LYPLAL1	chr1	219352561/A//G	nonsynonymous	0	2
OR1C1	chr1	247921569/G//A	nonsynonymous	0	2
TCF23	chr2	27372140/G//T	nonsynonymous	0	2
BCL11A	chr2	60688585/C//T	nonsynonymous	0	2
TMEM131	chr2	98418376/A//G	synonymous	0	2
MCM6	chr2	136615535/G//A	nonsynonymous	0	2
ZEB2	chr2	145158873/T//C	nonsynonymous	0	2
PHOSPHO2-KLHL2	chr2	170592513/C//T	nonsynonymous	0	2
FAM171B	chr2	187627527/C//T	nonsynonymous	0	2
ATP2B2	chr3	10382299/C//T	nonsynonymous	0	2
EPHA3	chr3	89390994/C//T	nonsynonymous	0	2
TBC1D23	chr3	100009514/A//G	nonsynonymous	0	2
DNAJC13	chr3	132207301/G//C	synonymous	0	2
HLTF	chr3	148782618/T//C	synonymous	0	2
BCHE	chr3	165548715/G//T	nonsynonymous	0	2
DRD5	chr4	9783995/C//T	synonymous	0	2
AASDH	chr4	57250290/C//T	nonsynonymous	0	2
ADCY2	chr5	7709491/C//A	synonymous	0	2
IPO11	chr5	61833035/G//C	nonsynonymous	0	2
HTR1A	chr5	63257256/C//T	synonymous	0	2
RASGRF2	chr5	80419488/G//A	nonsynonymous	0	2
RAPGEF6	chr5	130766824/T//A	nonsynonymous	0	2
PCDHB2	chr5	140474741/C//A	nonsynonymous	0	2
ERGIC1	chr5	172353544/C//G	synonymous	0	2
HLA-DRB1	chr6	32548553/C//T	nonsynonymous	0	2
RIMS1	chr6	73110320/G//A	synonymous	0	2
MYO6	chr6	76624606/G//T	nonsynonymous	0	2
PHIP	chr6	79679812/G//A	synonymous	0	2
UBE2J1	chr6	90039567/C//T	nonsynonymous	0	2
FBXO30	chr6	146127135/A//T	nonsynonymous	0	2
GRM1	chr6	146350523/C//T	synonymous	0	2
SDK1	chr7	4304862/G//A	nonsynonymous	0	2
AMPH	chr7	38574566/ACTG//A	nonsynonymous	0	2
IGFBP3	chr7	45956889/G//A	nonsynonymous	0	2
IKZF1	chr7	50455075/C//T	nonsynonymous	0	2
ADRA1A	chr8	26721942/G//A	nonsynonymous	0	2
TEX15	chr8	30702140/T//C	nonsynonymous	0	2
SPATA31D1	chr9	84605873/C//G	nonsynonymous	0	2
ZNF883	chr9	115759644/T//C	nonsynonymous	0	2
NET1	chr10	5498724/G//A	nonsynonymous	0	2
CUBN	chr10	16878276/C//T	nonsynonymous	0	2
	chr10	55568933/G//C	synonymous	0	2
MMP26	chr11	5010966/G//A	nonsynonymous	0	2
TRIM3	chr11	6477532/C//T	nonsynonymous	0	2
TTC17	chr11	43425627/C//G	nonsynonymous	0	2
TENM4	chr11	78383335/C//T	nonsynonymous	0	2
ARHGEF25	chr12	58009799/C//T	synonymous	0	2
LRRC10	chr12	70004478/G//A	synonymous	0	2
RASSF9	chr12	86199651/C//T	nonsynonymous	0	2
SACS	chr13	23912440/G//A	nonsynonymous	0	2
	chr13	33017007/A//T	synonymous	0	2
PCDH8	chr13	53420657/G//A	nonsynonymous	0	2
RBM23	chr14	23375531/G//A	nonsynonymous	0	2

GOLGA5	chr14	93273121/G//A	synonymous	0	2
C15orf65	chr15	55710805/T//A	synonymous	0	2
CSPG4	chr15	75979962/G//A	synonymous	0	2
FOXL1	chr16	86612665/C//T	synonymous	0	2
MYH1	chr17	10399369/G//A	synonymous	0	2
LIG3	chr17	33323178/C//T	synonymous	0	2
WNK4	chr17	40947112/G//A	synonymous	0	2
PTPRS	chr19	5222888/G//T	nonsynonymous	0	2
COL5A3	chr19	10102709/C//A	nonsynonymous	0	2
RHPN2	chr19	33481570/T//G	synonymous	0	2
HIPK4	chr19	40886406/C//T	nonsynonymous	0	2
PLEKHA4	chr19	49364680/C//T	nonsynonymous	0	2
BRSK1	chr19	55805499/C//T	synonymous	0	2
GINS1	chr20	25388437/G//T	synonymous	0	2
SLC5A3	chr21	35469024/C//G	nonsynonymous	0	2
DSCAM	chr21	41684283/G//A	nonsynonymous	0	2
TAB3	chrX	30870896/G//A	nonsynonymous	0	2
IL1RAPL2	chrX	104984676/G//A	nonsynonymous	0	2
LRCH2	chrX	114357746/C//T	nonsynonymous	0	2
ENOX2	chrX	129813701/C//T	nonsynonymous	0	2
KLHL21	chr1	6662343/C//T	nonsynonymous	0	2
PAX7	chr1	19018316/C//T	nonsynonymous	0	2
FAM46B	chr1	27332410/C//T	synonymous	0	2
TFAP2E	chr1	36060042/C//T	nonsynonymous	0	2
SSX2IP	chr1	85121602/T//G	nonsynonymous	0	2
PLPPR4	chr1	99772097/G//A	nonsynonymous	0	2
COL11A1	chr1	103385866/C//A	nonsynonymous	0	2
HRNR	chr1	152191000/T//G	synonymous	0	2
TP53BP2	chr1	223983550/C//T	synonymous	0	2
TARBP1	chr1	234565962/T//A	nonsynonymous	0	2
PRR30	chr2	27360213/G//A	nonsynonymous	0	2
NRBP1	chr2	27664736/C//T	synonymous	0	2
EGR4	chr2	73520484/G//T	synonymous	0	2
NPAS2	chr2	101565821/C//T	synonymous	0	2
IWS1	chr2	128263135/T//C	nonsynonymous	0	2
NGEF	chr2	233756165/G//A	nonsynonymous	0	2
FANCD2	chr3	10107162/G//T	nonsynonymous	0	2
PLCL2	chr3	17051564/G//T	nonsynonymous	0	2
IQCF6	chr3	51812781/C//T	nonsynonymous	0	2
LRIG1	chr3	66463428/G//A	nonsynonymous	0	2
ALDH1L1	chr3	125854473/G//T	synonymous	0	2
STAG1	chr3	136342065/G//A	nonsynonymous	0	2
PCOLCE2	chr3	142539743/T//G	nonsynonymous	0	2
ARHGEF26	chr3	153840779/CGAA//C	nonsynonymous	0	2
C3orf80	chr3	159943551/G//A	synonymous	0	2
UNC5C	chr4	96166179/A//T	nonsynonymous	0	2
ZDHHC11	chr5	825275/T//G	synonymous	0	2
ADAMTS16	chr5	5319237/A//G	nonsynonymous	0	2
FER	chr5	108207871/A//T	nonsynonymous	0	2
APC	chr5	112170863/G//C	nonsynonymous	0	2
RANBP17	chr5	170336775/T//G	synonymous	0	2
TUBB2B	chr6	3225036/C//T	synonymous	0	2
DDR1	chr6	30859898/G//T	nonsynonymous	0	2
EYA4	chr6	133783809/A//C	nonsynonymous	0	2
TRA2A	chr7	23555997/T//C	synonymous	0	2
POU6F2	chr7	39491228/G//A	nonsynonymous	0	2
PTPRZ1	chr7	121652219/C//T	nonsynonymous	0	2
PAXIP1	chr7	154735869/T//G	nonsynonymous	0	2
AGPAT5	chr8	6614704/A//T	nonsynonymous	0	2
SGCZ	chr8	13947906/G//A	synonymous	0	2
SCARA3	chr8	27516511/C//T	nonsynonymous	0	2
DPY19L4	chr8	95800154/G//T	nonsynonymous	0	2
ADCY8	chr8	131916248/C//T	nonsynonymous	0	2
GPT	chr8	145731400/C//T	nonsynonymous	0	2
CCDC171	chr9	15920344/C//T	nonsynonymous	0	2
RUSC2	chr9	35547005/C//T	nonsynonymous	0	2
TNC	chr9	117783543/C//T	nonsynonymous	0	2
ABO	chr9	136131623/C//T	synonymous	0	2
GPR158	chr10	25886785/C//T	nonsynonymous	0	2
RET	chr10	43598033/A//T	nonsynonymous	0	2

SLIT1	chr10	98803205/C//T	nonsynonymous	0	2
OR52K2	chr11	4471063/T//G	nonsynonymous	0	2
STK33	chr11	8478918/C//T	nonsynonymous	0	2
KIF18A	chr11	28083996/C//A	nonsynonymous	0	2
MMP13	chr11	102825296/G//A	synonymous	0	2
OR6C74	chr12	55641682/C//T	nonsynonymous	0	2
KCNC2	chr12	75601181/C//T	nonsynonymous	0	2
FGD6	chr12	95603024/A//G	nonsynonymous	0	2
DNAH10	chr12	124289591/A//G	synonymous	0	2
CCDC168	chr13	103399818/A//T	nonsynonymous	0	2
RNASE10	chr14	20979164/C//A	nonsynonymous	0	2
POLG	chr15	89864365/C//T	nonsynonymous	0	2
BLM	chr15	91334019/C//G	nonsynonymous	0	2
HAGHL	chr16	779377/C//T	synonymous	0	2
ABCA3	chr16	2347759/C//T	synonymous	0	2
VWA3A	chr16	22161155/C//T	nonsynonymous	0	2
MT4	chr16	56601692/A//T	nonsynonymous	0	2
MMP15	chr16	58075691/C//T	nonsynonymous	0	2
SPDYE4	chr17	8661682/G//A	nonsynonymous	0	2
MYO15A	chr17	18022899/C//T	nonsynonymous	0	2
CDR2L	chr17	72999691/C//G	nonsynonymous	0	2
LRRC30	chr18	7231425/G//A	nonsynonymous	0	2
ANKRD12	chr18	9257562/G//A	nonsynonymous	0	2
DSG3	chr18	29044263/A//C	nonsynonymous	0	2
PRR36	chr19	7938477/G//A	nonsynonymous	0	2
MAP2K7	chr19	7975375/G//A	nonsynonymous	0	2
CRTC1	chr19	18870996/G//A	nonsynonymous	0	2
LIN7B	chr19	49618159/C//T	nonsynonymous	0	2
LILRA1	chr19	55106724/G//A	nonsynonymous	0	2
ZNF667	chr19	56973772/G//T	synonymous	0	2
PROKR2	chr20	5282748/G//T	nonsynonymous	0	2
TSSK2	chr22	19119027/G//A	nonsynonymous	0	2
NLGN4X	chrX	5810849/A//T	synonymous	0	2
ASB11	chrX	15307658/C//A	nonsynonymous	0	2
BRWD3	chrX	80049264/G//A	nonsynonymous	0	2
TEX13B	chrX	107225343/A//C	synonymous	0	2
NPHP4	chr1	5937242/C//T	nonsynonymous	0	2
CAMTA1	chr1	7737741/C//A	synonymous	0	2
DISP3	chr1	11561566/C//A	nonsynonymous	0	2
FBLIM1	chr1	16101295/C//T	synonymous	0	2
ALDH4A1	chr1	19208250/C//A	synonymous	0	2
ALDH4A1	chr1	19208347/G//A	nonsynonymous	0	2
PINK1	chr1	20964476/A//G	nonsynonymous	0	2
KIF17	chr1	21031094/G//T	synonymous	0	2
SLC9A1	chr1	27480705/G//A	nonsynonymous	0	2
IFI6	chr1	27994825/C//T	synonymous	0	2
RAB42	chr1	28920511/C//T	nonsynonymous	0	2
HDAC1	chr1	32768278/C//T	nonsynonymous	0	2
ZMYM6	chr1	35476197/C//A	nonsynonymous	0	2
ZC3H12A	chr1	37949109/C//T	nonsynonymous	0	2
EIF2B3	chr1	45316624/C//A	nonsynonymous	0	2
TRABD2B	chr1	48460088/G//A	nonsynonymous	0	2
ACOT11	chr1	55070012/C//G	synonymous	0	2
PCSK9	chr1	55524212/G//A	synonymous	0	2
DAB1	chr1	57480925/C//A	nonsynonymous	0	2
DAB1	chr1	57610966/G//T	synonymous	0	2
C1orf87	chr1	60503678/G//C	synonymous	0	2
INSL5	chr1	67266822/A//G	nonsynonymous	0	2
C1orf141	chr1	67559071/T//C	nonsynonymous	0	2
C1orf141	chr1	67559104/T//A	nonsynonymous	0	2
LRRC7	chr1	70300505/C//T	synonymous	0	2
ERICH3	chr1	75055471/C//A	nonsynonymous	0	2
ERICH3	chr1	75065541/G//A	nonsynonymous	0	2
CLCA2	chr1	86891142/C//A	nonsynonymous	0	2
ABCA4	chr1	94463655/C//A	nonsynonymous	0	2
VAV3	chr1	108145715/C//A	nonsynonymous	0	2
SLC25A24	chr1	108728453/C//A	nonsynonymous	0	2
KCNC4	chr1	110775540/C//T	synonymous	0	2
DENND2C	chr1	115130484/C//G	nonsynonymous	0	2
SPTA1	chr1	158596683/C//A	nonsynonymous	0	2

PCP4L1	chr1	161228741/C//T	synonymous	0	2
FCGR2B	chr1	161641416/C//A	nonsynonymous	0	2
SUCO	chr1	172558660/A//G	nonsynonymous	0	2
TNN	chr1	175066710/G//T	nonsynonymous	0	2
PAPPA2	chr1	176659272/G//A	nonsynonymous	0	2
BRINP3	chr1	190250828/G//T	nonsynonymous	0	2
CFHR2	chr1	196920047/G//A	nonsynonymous	0	2
KIF21B	chr1	200957978/G//A	nonsynonymous	0	2
PLXNA2	chr1	208390188/G//A	synonymous	0	2
PLXNA2	chr1	208390500/G//A	synonymous	0	2
FLVCR1	chr1	213068638/A//C	synonymous	0	2
TGFB2	chr1	218520201/C//T	nonsynonymous	0	2
MARK1	chr1	220823970/G//A	synonymous	0	2
CCDC185	chr1	223568024/G//A	nonsynonymous	0	2
CDC42BPA	chr1	227504799/C//G	nonsynonymous	0	2
NID1	chr1	236187393/C//A	nonsynonymous	0	2
NID1	chr1	236189373/G//T	nonsynonymous	0	2
RYR2	chr1	237711810/G//C	nonsynonymous	0	2
RYR2	chr1	237872271/C//G	nonsynonymous	0	2
CEP170	chr1	243349617/C//T	nonsynonymous	0	2
ZBTB18	chr1	244218142/G//T	nonsynonymous	0	2
OR2L2	chr1	248202201/C//T	nonsynonymous	0	2
TPO	chr2	1499877/C//A	nonsynonymous	0	2
MYT1L	chr2	1893147/T//A	nonsynonymous	0	2
RRM2	chr2	10269445/G//T	nonsynonymous	0	2
ITSN2	chr2	24507633/T//A	nonsynonymous	0	2
GAREM2	chr2	26399320/G//T	nonsynonymous	0	2
SLC5A6	chr2	27428936/G//A	synonymous	0	2
CAD	chr2	27460408/C//T	nonsynonymous	0	2
C2orf16	chr2	27803860/C//A	nonsynonymous	0	2
PLB1	chr2	28719012/G//T	nonsynonymous	0	2
TTC27	chr2	32983494/C//T	nonsynonymous	0	2
VIT	chr2	37035654/C//A	nonsynonymous	0	2
SLC3A1	chr2	44507966/G//T	nonsynonymous	0	2
TSPYL6	chr2	54482822/G//A	nonsynonymous	0	2
ARHGAP25	chr2	69049767/G//C	nonsynonymous	0	2
NFU1	chr2	69627624/C//G	nonsynonymous	0	2
ATP6V1B1	chr2	71186145/C//T	synonymous	0	2
EXOC6B	chr2	72707805/G//A	synonymous	0	2
ALMS1	chr2	73680192/G//T	nonsynonymous	0	2
ACTG2	chr2	74136174/C//A	synonymous	0	2
REG3G	chr2	79254213/T//A	synonymous	0	2
CD8A	chr2	87013091/A//T	synonymous	0	2
KRCC1	chr2	88327507/G//A	synonymous	0	2
EIF2AK3	chr2	88874656/C//A	nonsynonymous	0	2
TGFBRAP1	chr2	105924177/G//A	synonymous	0	2
RANBP2	chr2	109399042/T//C	synonymous	0	2
RALB	chr2	121043495/A//C	nonsynonymous	0	2
CNTNAP5	chr2	125521714/C//G	synonymous	0	2
GPR17	chr2	128408808/C//A	nonsynonymous	0	2
THSD7B	chr2	138169224/C//A	nonsynonymous	0	2
LRP1B	chr2	141004680/G//T	nonsynonymous	0	2
KYNU	chr2	143715202/C//A	synonymous	0	2
NEB	chr2	152522012/G//A	synonymous	0	2
TANC1	chr2	160050986/G//C	synonymous	0	2
XIRP2	chr2	168114653/G//T	synonymous	0	2
TTN	chr2	179402297/T//C	nonsynonymous	0	2
TTN	chr2	179549401/G//A	nonsynonymous	0	2
RAPH1	chr2	204322304/C//T	synonymous	0	2
LOC200726	chr2	207513444/T//A	nonsynonymous	0	2
METTL21A	chr2	208489072/T//A	nonsynonymous	0	2
VWC2L	chr2	215301413/G//C	nonsynonymous	0	2
WNT6	chr2	219738418/C//G	nonsynonymous	0	2
CDK5R2	chr2	219824473/C//A	synonymous	0	2
SPEG	chr2	220336622/G//T	nonsynonymous	0	2
SCG2	chr2	224463552/T//A	nonsynonymous	0	2
FAM124B	chr2	225244611/G//T	nonsynonymous	0	2
DOCK10	chr2	225796334/C//A	nonsynonymous	0	2
COL4A3	chr2	228137736/A//T	synonymous	0	2
INPP5D	chr2	233925096/C//A	synonymous	0	2

SH3BP4	chr2	235951082/A//G	nonsynonymous	0	2
SH3BP4	chr2	235961238/G//T	synonymous	0	2
IQCA1	chr2	237276892/C//A	nonsynonymous	0	2
PDCD1	chr2	242794788/C//T	nonsynonymous	0	2
KCNH8	chr3	19295140/C//G	synonymous	0	2
KCNH8	chr3	19322786/C//A	nonsynonymous	0	2
EFHB	chr3	19975181/C//T	synonymous	0	2
PDCD6IP	chr3	33853613/A//T	nonsynonymous	0	2
DNAH1	chr3	52422631/C//T	synonymous	0	2
STAB1	chr3	52554547/G//T	nonsynonymous	0	2
ITIH4	chr3	52852141/C//G	nonsynonymous	0	2
CACNA1D	chr3	53839080/C//T	nonsynonymous	0	2
WNT5A	chr3	55508502/C//T	nonsynonymous	0	2
LMOD3	chr3	69168498/A//G	synonymous	0	2
BBX	chr3	107429400/G//A	synonymous	0	2
POLQ	chr3	121207300/C//A	nonsynonymous	0	2
UROC1	chr3	126216947/G//A	nonsynonymous	0	2
	chr3	126220685/C//A	synonymous	0	2
COL6A6	chr3	130284164/C//A	nonsynonymous	0	2
RASA2	chr3	141292002/C//T	nonsynonymous	0	2
SLC9A9	chr3	143515708/A//T	nonsynonymous	0	2
PLOD2	chr3	145788891/G//A	nonsynonymous	0	2
ZIC4	chr3	147109029/C//T	synonymous	0	2
ZIC1	chr3	147128730/G//T	synonymous	0	2
HPS3	chr3	148857855/G//C	nonsynonymous	0	2
TSC22D2	chr3	150127997/AAAGCTCG//A	nonsynonymous	0	2
KCNAB1	chr3	156170720/G//A	nonsynonymous	0	2
MLF1	chr3	158289059/C//T	synonymous	0	2
SI	chr3	164786976/C//T	nonsynonymous	0	2
LRRC31	chr3	169557760/G//A	synonymous	0	2
SLC2A2	chr3	170732358/G//C	nonsynonymous	0	2
PEX5L	chr3	179526153/G//A	synonymous	0	2
LPP	chr3	188426059/G//T	nonsynonymous	0	2
NRROS	chr3	196386837/G//T	nonsynonymous	0	2
ZFYVE28	chr4	2306969/G//T	nonsynonymous	0	2
KDR	chr4	55946139/G//T	nonsynonymous	0	2
CENPC	chr4	68384034/C//A	nonsynonymous	0	2
GC	chr4	72635114/C//T	nonsynonymous	0	2
ANTXR2	chr4	80899213/C//A	nonsynonymous	0	2
SNCA	chr4	90743430/G//T	synonymous	0	2
CENPE	chr4	104070370/T//C	nonsynonymous	0	2
ENPEP	chr4	111412263/G//T	synonymous	0	2
ANK2	chr4	114279022/C//A	nonsynonymous	0	2
PRDM5	chr4	121698358/T//C	nonsynonymous	0	2
RNF150	chr4	141888947/C//T	nonsynonymous	0	2
C4orf45	chr4	159814831/G//T	synonymous	0	2
DDX60	chr4	169195105/C//A	nonsynonymous	0	2
EXOC3	chr5	446373/G//T	nonsynonymous	0	2
SLC6A3	chr5	1422073/C//T	nonsynonymous	0	2
IRX4	chr5	1882119/G//C	nonsynonymous	0	2
CTNND2	chr5	11082943/G//T	synonymous	0	2
CDH10	chr5	24509698/T//A	synonymous	0	2
AGXT2	chr5	34998834/C//G	nonsynonymous	0	2
C7	chr5	40937765/T//G	nonsynonymous	0	2
MROH2B	chr5	41057265/C//A	nonsynonymous	0	2
FBXO4	chr5	41925363/G//T	synonymous	0	2
GHR	chr5	42719221/G//T	nonsynonymous	0	2
NNT	chr5	43624218/A//G	nonsynonymous	0	2
FGF10	chr5	44388542/C//A	nonsynonymous	0	2
FGF10	chr5	44388698/C//A	nonsynonymous	0	2
MCIDAS	chr5	54523034/C//A	synonymous	0	2
MAP3K1	chr5	56171105/G//T	nonsynonymous	0	2
MAST4	chr5	66460988/G//C	nonsynonymous	0	2
ADGRV1	chr5	90049518/C//T	nonsynonymous	0	2
KCNN2	chr5	113740349/T//A	nonsynonymous	0	2
KCNN2	chr5	113831851/C//A	nonsynonymous	0	2
PCDHA6	chr5	140208457/G//T	nonsynonymous	0	2
PCDHA7	chr5	140215836/G//A	nonsynonymous	0	2
PCDHA11	chr5	140250991/T//A	nonsynonymous	0	2
PCDHB10	chr5	140572514/C//G	nonsynonymous	0	2

PCDHB12	chr5	140588582/G//T	nonsynonymous	0	2
PCDHGA6	chr5	140754865/G//A	synonymous	0	2
ABLIM3	chr5	148622082/C//A	synonymous	0	2
CCDC69	chr5	150563079/G//C	nonsynonymous	0	2
SLC36A2	chr5	150704892/C//A	nonsynonymous	0	2
SPARC	chr5	151051208/C//A	nonsynonymous	0	2
GRIA1	chr5	153026721/G//T	nonsynonymous	0	2
ATP10B	chr5	160047592/G//T	synonymous	0	2
	chr5	162930366/T//G	synonymous	0	2
TENM2	chr5	166712041/C//A	synonymous	0	2
SLIT3	chr5	168187871/TC//T	nonsynonymous	0	2
FOXI1	chr5	169532986/C//T	nonsynonymous	0	2
BOD1	chr5	173040196/C//A	nonsynonymous	0	2
ZNF879	chr5	178459595/A//T	nonsynonymous	0	2
OR2Y1	chr5	180166123/C//A	nonsynonymous	0	2
RREB1	chr6	7211139/C//T	synonymous	0	2
GCNT2	chr6	10626777/G//A	synonymous	0	2
CD83	chr6	14131826/G//T	nonsynonymous	0	2
HIST1H3I	chr6	27839874/C//G	nonsynonymous	0	2
GTF2H4	chr6	30879860/G//T	nonsynonymous	0	2
TNXB	chr6	32035631/G//T	synonymous	0	2
TAP2	chr6	32805705/C//A	synonymous	0	2
	chr6	42903269/G//T	synonymous	0	2
TDRD6	chr6	46658034/G//C	nonsynonymous	0	2
HMGCLL1	chr6	55304234/T//A	nonsynonymous	0	2
BMP5	chr6	55739636/C//A	nonsynonymous	0	2
KIAA1586	chr6	56917505/A//G	nonsynonymous	0	2
EYS	chr6	64574244/C//T	nonsynonymous	0	2
TTK	chr6	80732156/G//T	nonsynonymous	0	2
IBTK	chr6	82904329/AT//A	nonsynonymous	0	2
TRDN	chr6	123759228/G//T	nonsynonymous	0	2
MED23	chr6	131931258/T//C	synonymous	0	2
TAAR6	chr6	132891983/G//C	nonsynonymous	0	2
TAAR6	chr6	132892191/G//T	nonsynonymous	0	2
	chr6	154331664/C//A	synonymous	0	2
OPRM1	chr6	154412236/C//A	nonsynonymous	0	2
TIAM2	chr6	155503448/G//T	synonymous	0	2
IGF2R	chr6	160448258/C//T	nonsynonymous	0	2
WDR27	chr6	169857546/C//A	synonymous	0	2
WIPI2	chr7	5256241/G//T	synonymous	0	2
NXPH1	chr7	8791355/G//T	nonsynonymous	0	2
SFRP4	chr7	37951805/G//T	nonsynonymous	0	2
NPC1L1	chr7	44574045/C//A	nonsynonymous	0	2
IGFBP3	chr7	45954541/G//T	nonsynonymous	0	2
IKZF1	chr7	50444497/G//T	synonymous	0	2
ZNF117	chr7	64438479/T//A	synonymous	0	2
CACNA2D1	chr7	81614020/A//T	nonsynonymous	0	2
DBF4	chr7	87517355/G//T	nonsynonymous	0	2
ZNF804B	chr7	88965173/G//T	synonymous	0	2
FZD1	chr7	90895884/C//T	synonymous	0	2
CDK6	chr7	92462615/C//T	nonsynonymous	0	2
PEG10	chr7	94293493/G//A	nonsynonymous	0	2
LAMB1	chr7	107593974/C//A	nonsynonymous	0	2
LAMB1	chr7	107595983/T//A	nonsynonymous	0	2
PNPLA8	chr7	108131890/C//T	synonymous	0	2
PPP1R3A	chr7	113519322/C//A	nonsynonymous	0	2
CFTR	chr7	117149111/C//T	nonsynonymous	0	2
CPED1	chr7	120906303/C//A	nonsynonymous	0	2
NDUFA5	chr7	123197466/G//A	nonsynonymous	0	2
GCC1	chr7	127224713/A//T	nonsynonymous	0	2
FSCN3	chr7	127238495/C//A	nonsynonymous	0	2
MKLN1	chr7	131149075/C//G	nonsynonymous	0	2
CALD1	chr7	134618183/G//T	nonsynonymous	0	2
SVOPL	chr7	138363762/G//C	nonsynonymous	0	2
ATP6V0A4	chr7	138444520/C//T	nonsynonymous	0	2
KEL	chr7	142643395/G//T	nonsynonymous	0	2
TAS2R41	chr7	143175796/T//G	synonymous	0	2
CTAGE6	chr7	143453530/A//T	nonsynonymous	0	2
ZNF282	chr7	148904487/G//T	nonsynonymous	0	2
ZNF777	chr7	149153037/C//A	nonsynonymous	0	2

CRYGN	chr7	151133409/G//T	nonsynonymous	0	2
DLGAP2	chr8	1497461/A//T	nonsynonymous	0	2
PXDNL	chr8	52321030/C//A	nonsynonymous	0	2
SOX17	chr8	55370986/C//A	synonymous	0	2
FAM110B	chr8	59059611/G//T	synonymous	0	2
ZFHX4	chr8	77616941/A//T	synonymous	0	2
ZFHX4	chr8	77617383/A//T	nonsynonymous	0	2
ZFHX4	chr8	77768511/G//T	nonsynonymous	0	2
RALYL	chr8	85686813/G//C	nonsynonymous	0	2
NECAB1	chr8	91953055/G//C	nonsynonymous	0	2
POP1	chr8	99158853/G//T	nonsynonymous	0	2
	chr8	100519370/G//T	synonymous	0	2
RIMS2	chr8	104897695/C//A	nonsynonymous	0	2
RIMS2	chr8	104940115/C//A	nonsynonymous	0	2
DCSTAMP	chr8	105367169/G//T	nonsynonymous	0	2
DPYS	chr8	105405165/G//T	synonymous	0	2
ZFPM2	chr8	106814844/C//A	nonsynonymous	0	2
CSMD3	chr8	113301724/C//A	synonymous	0	2
CSMD3	chr8	113303794/T//A	synonymous	0	2
	chr8	118159081/A//G	synonymous	0	2
FAM135B	chr8	139153480/G//A	synonymous	0	2
FAM135B	chr8	139190820/G//T	synonymous	0	2
ARC	chr8	143694490/G//T	synonymous	0	2
SPATC1	chr8	145094871/C//T	synonymous	0	2
RFX3	chr9	3248079/G//A	nonsynonymous	0	2
EQTN	chr9	27297025/G//T	nonsynonymous	0	2
TOPORS	chr9	32543353/T//G	synonymous	0	2
SIGMAR1	chr9	34637764/C//T	synonymous	0	2
FANCG	chr9	35077335/A//G	nonsynonymous	0	2
CCDC180	chr9	100092802/C//A	nonsynonymous	0	2
ANKS6	chr9	101530418/G//C	nonsynonymous	0	2
GALNT12	chr9	101599362/C//T	nonsynonymous	0	2
COL15A1	chr9	101818657/C//A	nonsynonymous	0	2
GRIN3A	chr9	104499681/G//GA	nonsynonymous	0	2
OR13C2	chr9	107367884/G//C	nonsynonymous	0	2
KLF4	chr9	110249963/G//T	nonsynonymous	0	2
FKBP15	chr9	115936807/C//A	nonsynonymous	0	2
RNF183	chr9	116060154/G//T	nonsynonymous	0	2
TRAF1	chr9	123673639/GC//G	nonsynonymous	0	2
OR1B1	chr9	125390986/C//A	nonsynonymous	0	2
OR1L6	chr9	125513016/A//C	nonsynonymous	0	2
SCAI	chr9	127765424/T//A	nonsynonymous	0	2
ODF2	chr9	131218481/C//T	synonymous	0	2
USP20	chr9	132640649/G//T	synonymous	0	2
GPR107	chr9	132816191/G//T	synonymous	0	2
SETX	chr9	135221833/C//A	nonsynonymous	0	2
DPP7	chr9	140009143/G//A	nonsynonymous	0	2
NDOR1	chr9	140110544/A//T	nonsynonymous	0	2
ADARB2	chr10	1779252/C//A	nonsynonymous	0	2
AKR1C4	chr10	5254658/G//T	nonsynonymous	0	2
FBXO18	chr10	5960348/TG//T	nonsynonymous	0	2
ITGA8	chr10	15634295/C//A	synonymous	0	2
ITGA8	chr10	15688860/G//T	nonsynonymous	0	2
CACNB2	chr10	18429770/C//A	synonymous	0	2
ARMC3	chr10	23270571/C//A	synonymous	0	2
KIAA1217	chr10	24508829/G//C	synonymous	0	2
FZD8	chr10	35930233/A//C	nonsynonymous	0	2
ANKRD30A	chr10	37441000/C//A	nonsynonymous	0	2
RBP3	chr10	48388623/G//T	nonsynonymous	0	2
GDF10	chr10	48429113/C//A	nonsynonymous	0	2
FRMPD2	chr10	49450351/C//A	nonsynonymous	0	2
WDFY4	chr10	50178193/G//T	nonsynonymous	0	2
VSTM4	chr10	50315706/C//A	nonsynonymous	0	2
	chr10	55566570/C//A	synonymous	0	2
	chr10	55569257/C//A	synonymous	0	2
PCDH15	chr10	56138617/C//T	synonymous	0	2
PCDH15	chr10	56287564/T//A	synonymous	0	2
ANK3	chr10	61829710/T//A	synonymous	0	2
ZNF365	chr10	64159602/A//T	synonymous	0	2
PALD1	chr10	72291046/G//C	nonsynonymous	0	2

ADAMTS14	chr10	72493723/G//A	nonsynonymous	0	2
UNC5B	chr10	73056465/G//T	nonsynonymous	0	2
PSAP	chr10	73581696/C//A	synonymous	0	2
NDST2	chr10	75568185/C//G	synonymous	0	2
SH2D4B	chr10	82348465/G//T	nonsynonymous	0	2
DNTT	chr10	98079048/C//A	nonsynonymous	0	2
FGF8	chr10	103534923/G//A	synonymous	0	2
LDB1	chr10	103871191/C//A	nonsynonymous	0	2
CFAP58	chr10	106128266/T//C	nonsynonymous	0	2
ADD3	chr10	111892126/A//T	nonsynonymous	0	2
KCNK18	chr10	118969488/G//T	nonsynonymous	0	2
PDZD8	chr10	119044080/T//C	nonsynonymous	0	2
CPXM2	chr10	125506476/T//A	nonsynonymous	0	2
ADAM8	chr10	135082998/C//T	synonymous	0	2
TUBGCP2	chr10	135106161/C//G	synonymous	0	2
OR51Q1	chr11	5443416/A//C	synonymous	0	2
OR52N1	chr11	5810018/G//T	nonsynonymous	0	2
FAM160A2	chr11	6245755/C//A	synonymous	0	2
OR2AG1	chr11	6806580/A//G	synonymous	0	2
OR10A2	chr11	6891858/G//A	synonymous	0	2
INSC	chr11	15267544/C//A	nonsynonymous	0	2
SLC5A12	chr11	26725449/C//A	nonsynonymous	0	2
FSHB	chr11	30255312/C//A	nonsynonymous	0	2
DCDC5	chr11	30914433/C//A	nonsynonymous	0	2
C11orf91	chr11	33720062/T//C	nonsynonymous	0	2
CAT	chr11	34475412/C//G	nonsynonymous	0	2
API5	chr11	43333621/C//T	synonymous	0	2
PEX16	chr11	45932473/C//A	nonsynonymous	0	2
CKAP5	chr11	46772100/C//A	nonsynonymous	0	2
LRP4	chr11	46907690/C//A	nonsynonymous	0	2
TRIM48	chr11	55032767/G//T	nonsynonymous	0	2
OR4A16	chr11	55110769/C//A	synonymous	0	2
OR5D18	chr11	55587111/G//T	synonymous	0	2
OR5D18	chr11	55587840/C//T	synonymous	0	2
OR8H2	chr11	55873044/C//A	nonsynonymous	0	2
OR8J3	chr11	55904280/C//A	nonsynonymous	0	2
OR8H1	chr11	56058116/G//A	synonymous	0	2
OR5M11	chr11	56310718/C//A	nonsynonymous	0	2
APLNR	chr11	57003765/C//G	nonsynonymous	0	2
OR9Q2	chr11	57958836/C//A	nonsynonymous	0	2
MS4A12	chr11	60264955/C//G	nonsynonymous	0	2
CCDC86	chr11	60617675/C//T	synonymous	0	2
AHNAK	chr11	62297270/C//A	nonsynonymous	0	2
SLC22A10	chr11	63072289/G//C	nonsynonymous	0	2
LTBP3	chr11	65321327/C//T	nonsynonymous	0	2
TSGA10IP	chr11	65713257/G//T	synonymous	0	2
TSGA10IP	chr11	65715305/C//T	synonymous	0	2
ZDHHC24	chr11	66311292/C//G	nonsynonymous	0	2
LRP5	chr11	68177608/G//T	nonsynonymous	0	2
IGHMBP2	chr11	68671331/C//T	synonymous	0	2
SHANK2	chr11	70332580/A//T	nonsynonymous	0	2
NEU3	chr11	74717570/T//C	synonymous	0	2
DLG2	chr11	83180305/C//A	nonsynonymous	0	2
NAALAD2	chr11	89867893/T//C	synonymous	0	2
FAT3	chr11	92577145/C//A	nonsynonymous	0	2
NCAM1	chr11	113075227/G//T	nonsynonymous	0	2
APOC3	chr11	116701532/G//T	nonsynonymous	0	2
DSCAML1	chr11	117301550/C//T	nonsynonymous	0	2
OR6T1	chr11	123813724/G//T	synonymous	0	2
OR10G8	chr11	123901084/G//T	nonsynonymous	0	2
ROBO4	chr11	124763758/G//T	nonsynonymous	0	2
B3GAT1	chr11	134253842/A//T	nonsynonymous	0	2
DYRK4	chr12	4705775/T//A	nonsynonymous	0	2
NOP2	chr12	6666224/G//A	nonsynonymous	0	2
ATN1	chr12	7046673/C//T	nonsynonymous	0	2
C1S	chr12	7177277/G//T	synonymous	0	2
CD163	chr12	7649684/C//A	nonsynonymous	0	2
PZP	chr12	9310394/A//G	nonsynonymous	0	2
PTPRO	chr12	15661660/C//A	nonsynonymous	0	2
RERGL	chr12	18234243/C//G	nonsynonymous	0	2

SOX5	chr12	23999038/A//G	synonymous	0	2
KRAS	chr12	25398284/C//G	nonsynonymous	0	2
SMCO2	chr12	27648646/A//T	nonsynonymous	0	2
FAR2	chr12	29423542/C//A	nonsynonymous	0	2
OVCH1-AS1	chr12	29614763/T//A	synonymous	0	2
DDX11	chr12	31249932/G//T	synonymous	0	2
H3F3C	chr12	31944562/C//A	synonymous	0	2
PDZRN4	chr12	41946460/G//T	nonsynonymous	0	2
KANSL2	chr12	49054239/C//A	synonymous	0	2
KMT2D	chr12	49427980/T//A	nonsynonymous	0	2
KMT2D	chr12	49427983/C//G	nonsynonymous	0	2
PRPH	chr12	49689154/G//A	synonymous	0	2
KRT4	chr12	53202199/T//C	nonsynonymous	0	2
RDH5	chr12	56117734/G//A	nonsynonymous	0	2
PAN2	chr12	56720416/G//C	nonsynonymous	0	2
MYO1A	chr12	57435079/G//T	synonymous	0	2
MBD6	chr12	57920471/G//C	nonsynonymous	0	2
AVIL	chr12	58197140/G//A	nonsynonymous	0	2
LGR5	chr12	71834080/C//G	synonymous	0	2
PTPRQ	chr12	81028730/A//T	synonymous	0	2
MYF6	chr12	81102701/C//A	nonsynonymous	0	2
EPYC	chr12	91365624/T//A	nonsynonymous	0	2
NTN4	chr12	96104404/C//A	nonsynonymous	0	2
CCDC38	chr12	96300201/T//C	synonymous	0	2
ASCL4	chr12	108169074/C//A	nonsynonymous	0	2
HECTD4	chr12	112622917/C//A	nonsynonymous	0	2
PRKAB1	chr12	120118099/A//T	nonsynonymous	0	2
TMEM132C	chr12	129180502/C//T	nonsynonymous	0	2
TMEM132C	chr12	129190373/C//G	nonsynonymous	0	2
GALNT9	chr12	132685807/C//G	nonsynonymous	0	2
PXMP2	chr12	133272600/A//T	nonsynonymous	0	2
USPL1	chr13	31232649/C//G	nonsynonymous	0	2
MAB21L1	chr13	36049869/A//T	nonsynonymous	0	2
SMAD9	chr13	37453486/T//C	nonsynonymous	0	2
FREM2	chr13	39435703/G//A	synonymous	0	2
PCDH8	chr13	53420228/G//A	nonsynonymous	0	2
PCDH20	chr13	61989265/G//C	nonsynonymous	0	2
TBC1D4	chr13	75866402/T//C	nonsynonymous	0	2
SLITRK5	chr13	88329816/G//T	nonsynonymous	0	2
NALCN	chr13	101763564/G//T	nonsynonymous	0	2
OR4M1	chr14	20248525/C//A	nonsynonymous	0	2
OR11H6	chr14	20692358/T//A	nonsynonymous	0	2
RNASE13	chr14	21502366/C//T	nonsynonymous	0	2
LRFN5	chr14	42356105/C//A	nonsynonymous	0	2
LRFN5	chr14	42356586/G//T	nonsynonymous	0	2
FSCB	chr14	44975685/C//T	nonsynonymous	0	2
FAM179B	chr14	45431933/C//T	synonymous	0	2
RPL10L	chr14	47120593/C//A	nonsynonymous	0	2
C14orf105	chr14	57938199/C//A	nonsynonymous	0	2
JDP2	chr14	75904753/G//T	nonsynonymous	0	2
TTLL5	chr14	76129455/G//T	synonymous	0	2
NRXN3	chr14	79175805/A//G	synonymous	0	2
CEP128	chr14	81372391/A//G	nonsynonymous	0	2
BCL11B	chr14	99641428/C//T	nonsynonymous	0	2
KIF26A	chr14	104618695/G//A	nonsynonymous	0	2
RYR3	chr15	33988429/G//T	nonsynonymous	0	2
GJD2	chr15	35046676/G//T	nonsynonymous	0	2
DUOXA2	chr15	45406664/C//G	synonymous	0	2
PLEKHO2	chr15	65157332/G//T	nonsynonymous	0	2
SCAPER	chr15	77067357/T//C	nonsynonymous	0	2
NTRK3	chr15	88679227/C//T	synonymous	0	2
VPS33B	chr15	91550237/C//A	nonsynonymous	0	2
SV2B	chr15	91809855/A//T	nonsynonymous	0	2
ADAMTS17	chr15	100636585/C//T	nonsynonymous	0	2
PDILT	chr16	20380957/A//T	nonsynonymous	0	2
ACSM2B	chr16	20552055/G//T	nonsynonymous	0	2
ATXN2L	chr16	28847619/G//T	synonymous	0	2
QPRT	chr16	29708560/C//T	nonsynonymous	0	2
HEATR3	chr16	50128648/A//G	nonsynonymous	0	2
NUP93	chr16	56839516/A//T	nonsynonymous	0	2

NLRC5	chr16	57073765/G//T	synonymous	0	2
CDH8	chr16	61935328/G//A	nonsynonymous	0	2
CHST5	chr16	75563836/G//A	synonymous	0	2
PKD1L2	chr16	81155247/C//A	nonsynonymous	0	2
GSE1	chr16	85667744/G//C	synonymous	0	2
CPNE7	chr16	89642448/T//C	nonsynonymous	0	2
ZNF276	chr16	89805514/G//C	synonymous	0	2
NCBP3	chr17	3716423/T//G	nonsynonymous	0	2
FAM64A	chr17	6352718/C//A	nonsynonymous	0	2
PHF23	chr17	7139283/C//A	nonsynonymous	0	2
MYH2	chr17	10448808/A//G	synonymous	0	2
KIAA0100	chr17	26951342/G//C	nonsynonymous	0	2
TBC1D29	chr17	28890256/A//T	nonsynonymous	0	2
CSF3	chr17	38171999/C//G	synonymous	0	2
TMEM99	chr17	38990868/A//G	nonsynonymous	0	2
DBF4B	chr17	42827984/G//T	nonsynonymous	0	2
PLEKHM1	chr17	43517549/T//A	nonsynonymous	0	2
SNX11	chr17	46196368/G//A	nonsynonymous	0	2
CACNA1G	chr17	48685333/G//A	nonsynonymous	0	2
SPAG9	chr17	49073994/C//G	nonsynonymous	0	2
SCN4A	chr17	62018923/G//A	synonymous	0	2
TEX2	chr17	62290017/C//A	nonsynonymous	0	2
TEX2	chr17	62290449/T//A	nonsynonymous	0	2
ARSG	chr17	66339925/G//T	synonymous	0	2
ABCA9	chr17	67031905/T//A	nonsynonymous	0	2
KIF19	chr17	72349725/G//A	synonymous	0	2
CASKIN2	chr17	73509865/C//G	synonymous	0	2
ITGB4	chr17	73749980/G//A	nonsynonymous	0	2
AFMID	chr17	76202015/TCC//T	synonymous	0	2
CEP295NL	chr17	76887851/G//A	synonymous	0	2
MTCL1	chr18	8783739/G//T	nonsynonymous	0	2
ROCK1	chr18	18629847/T//C	synonymous	0	2
ATP5A1	chr18	43671693/C//A	nonsynonymous	0	2
LIPG	chr18	47095862/C//A	nonsynonymous	0	2
ALPK2	chr18	56204754/G//T	nonsynonymous	0	2
ZADH2	chr18	72913384/T//C	nonsynonymous	0	2
C2CD4C	chr19	407837/C//T	synonymous	0	2
NMRK2	chr19	3936644/A//C	nonsynonymous	0	2
EVI5L	chr19	7927313/G//C	nonsynonymous	0	2
MUC16	chr19	8993550/G//A	synonymous	0	2
PPAN	chr19	10216927/T//C	synonymous	0	2
ZNF564	chr19	12638579/G//A	nonsynonymous	0	2
ZNF564	chr19	12638670/G//A	synonymous	0	2
CPAMD8	chr19	17111307/C//A	nonsynonymous	0	2
SUGP2	chr19	19115058/G//A	nonsynonymous	0	2
ZNF729	chr19	22499007/C//G	nonsynonymous	0	2
ZNF254	chr19	24309549/A//G	synonymous	0	2
ZNF536	chr19	30935088/A//T	nonsynonymous	0	2
TSHZ3	chr19	31770505/G//T	nonsynonymous	0	2
CHST8	chr19	34262897/G//T	nonsynonymous	0	2
ACP7	chr19	39589258/C//A	nonsynonymous	0	2
ACP7	chr19	39589260/G//A	nonsynonymous	0	2
FCGBP	chr19	40411831/C//T	nonsynonymous	0	2
FCGBP	chr19	40421397/G//A	nonsynonymous	0	2
ERICH4	chr19	41949215/C//G	synonymous	0	2
CIC	chr19	42794881/C//T	nonsynonymous	0	2
PSG9	chr19	43763006/C//A	synonymous	0	2
PSG9	chr19	43773581/C//A	nonsynonymous	0	2
IRGC	chr19	44223086/C//G	nonsynonymous	0	2
ZNF180	chr19	45004268/G//A	nonsynonymous	0	2
CCDC61	chr19	46521582/C//T	synonymous	0	2
PNMAL1	chr19	46973425/C//A	nonsynonymous	0	2
ELSPBP1	chr19	48523018/C//T	nonsynonymous	0	2
DKKL1	chr19	49878082/C//A	synonymous	0	2
POLD1	chr19	50918719/C//A	nonsynonymous	0	2
	chr19	51890430/G//T	synonymous	0	2
SIGLEC8	chr19	51961708/T//A	synonymous	0	2
ZNF836	chr19	52663827/C//G	nonsynonymous	0	2
ZNF534	chr19	52942089/T//C	nonsynonymous	0	2
ZNF578	chr19	53005138/G//T	nonsynonymous	0	2

CACNG6	chr19	54496179/G//A	synonymous	0	2
LILRA2	chr19	55086011/C//A	nonsynonymous	0	2
KIR2DL3	chr19	55253721/C//A	synonymous	0	2
KIR2DL3	chr19	55255397/C//A	synonymous	0	2
KIR2DL4	chr19	55325198/G//A	nonsynonymous	0	2
NLRP7	chr19	55450452/G//C	nonsynonymous	0	2
NLRP7	chr19	55452869/T//G	nonsynonymous	0	2
SYT5	chr19	55687383/T//C	nonsynonymous	0	2
FAM71E2	chr19	55871277/G//C	nonsynonymous	0	2
ZNF134	chr19	58132761/G//T	nonsynonymous	0	2
A1BG	chr19	58863053/G//T	nonsynonymous	0	2
A1BG	chr19	58863863/C//T	synonymous	0	2
ZNF497	chr19	58868292/C//A	nonsynonymous	0	2
NOP56	chr20	2634013/TG//T	nonsynonymous	0	2
RASSF2	chr20	4764908/G//A	synonymous	0	2
SLC23A2	chr20	4883165/C//T	nonsynonymous	0	2
PLCB4	chr20	9353023/C//A	nonsynonymous	0	2
PLCB4	chr20	9453496/T//A	nonsynonymous	0	2
PAX1	chr20	21689870/C//A	nonsynonymous	0	2
THBD	chr20	23028401/G//A	synonymous	0	2
DNMT3B	chr20	31386297/G//T	nonsynonymous	0	2
BPIFB1	chr20	31876555/C//A	nonsynonymous	0	2
C20orf173	chr20	34116899/A//T	nonsynonymous	0	2
PTPRT	chr20	40747057/C//A	nonsynonymous	0	2
TP53TG5	chr20	44006822/G//T	synonymous	0	2
PTPN1	chr20	49196310/C//T	nonsynonymous	0	2
MOCS3	chr20	49576379/G//T	nonsynonymous	0	2
ZFP64	chr20	50769190/G//A	nonsynonymous	0	2
CASS4	chr20	55027341/A//T	nonsynonymous	0	2
RTFDC1	chr20	55048380/G//T	synonymous	0	2
FAM209B	chr20	55108517/G//T	nonsynonymous	0	2
KCNQ2	chr20	62076612/G//T	synonymous	0	2
HELZ2	chr20	62199859/C//A	nonsynonymous	0	2
GABPA	chr21	27124544/T//C	synonymous	0	2
LTN1	chr21	30307431/C//A	synonymous	0	2
MAP3K7CL	chr21	30547127/C//A	synonymous	0	2
KRTAP15-1	chr21	31812887/C//A	nonsynonymous	0	2
KRTAP15-1	chr21	31812982/C//A	nonsynonymous	0	2
KRTAP19-1	chr21	31852401/G//A	nonsynonymous	0	2
TIAM1	chr21	32502637/C//A	synonymous	0	2
TIAM1	chr21	32585723/T//A	synonymous	0	2
DOPEY2	chr21	37618530/CTCT//C	nonsynonymous	0	2
TMPRSS3	chr21	43804100/C//A	nonsynonymous	0	2
RRP1B	chr21	45107994/T//C	nonsynonymous	0	2
PCNT	chr21	47831371/C//G	nonsynonymous	0	2
DIP2A	chr21	47953566/G//T	synonymous	0	2
MICAL3	chr22	18368739/C//G	nonsynonymous	0	2
SLC7A4	chr22	21385934/G//T	nonsynonymous	0	2
CCDC116	chr22	21990806/G//A	nonsynonymous	0	2
CYTH4	chr22	37699294/G//A	nonsynonymous	0	2
SHANK3	chr22	51160006/G//A	nonsynonymous	0	2
MXRA5	chrX	3248742/G//T	nonsynonymous	0	2
SMS	chrX	22003344/A//C	synonymous	0	2
ARX	chrX	25033871/C//A	synonymous	0	2
NR0B1	chrX	30326574/G//T	nonsynonymous	0	2
CITED1	chrX	71521690/C//A	nonsynonymous	0	2
GPR174	chrX	78426828/C//G	nonsynonymous	0	2
CENPI	chrX	100364979/G//T	nonsynonymous	0	2
TAF7L	chrX	100537432/A//C	nonsynonymous	0	2
COL4A5	chrX	107930803/T//C	synonymous	0	2
ALG13	chrX	110925450/C//T	synonymous	0	2
ALG13	chrX	110964908/A//T	synonymous	0	2
ALG13	chrX	111003103/C//G	nonsynonymous	0	2
ZCCHC16	chrX	111698456/A//T	nonsynonymous	0	2
LHFPL1	chrX	111874594/C//A	synonymous	0	2
WDR44	chrX	117526989/C//T	nonsynonymous	0	2
C1GALT1C1	chrX	119760853/C//G	nonsynonymous	0	2
DCAF12L1	chrX	125685755/C//A	synonymous	0	2
SMARCA1	chrX	128582421/C//A	nonsynonymous	0	2
XPNPEP2	chrX	128886152/G//T	nonsynonymous	0	2

ELF4	chrX	129208592/G//C	nonsynonymous	0	2
IGSF1	chrX	130411024/G//C	nonsynonymous	0	2
SPANXN4	chrX	142121903/G//T	nonsynonymous	0	2
SLITRK2	chrX	144906192/G//T	nonsynonymous	0	2
AFF2	chrX	147967499/C//A	nonsynonymous	0	2
MAGEA4	chrX	151093074/A//T	nonsynonymous	0	2
GABRA3	chrX	151376513/C//G	nonsynonymous	0	2
GABRQ	chrX	151818903/G//T	nonsynonymous	0	2
BGN	chrX	152771986/G//C	nonsynonymous	0	2
ARHGEF19	chr1	16535181/C//T	synonymous	0	2
ARID1A	chr1	27097790/C//T	nonsynonymous	0	2
DMRTA2	chr1	50884545/G//A	nonsynonymous	0	2
ZNHIT6	chr1	86171813/G//A	nonsynonymous	0	2
CHI3L2	chr1	111773482/C//T	synonymous	0	2
SPTA1	chr1	158641928/C//T	nonsynonymous	0	2
FCRLA	chr1	161681736/C//T	nonsynonymous	0	2
SEC16B	chr1	177934261/G//A	nonsynonymous	0	2
CR1	chr1	207791470/C//T	nonsynonymous	0	2
MARK1	chr1	220826579/G//A	nonsynonymous	0	2
TRIM17	chr1	228596425/G//A	nonsynonymous	0	2
FMN2	chr1	240497520/G//T	nonsynonymous	0	2
OR2W3	chr1	248058988/G//A	nonsynonymous	0	2
ZAP70	chr2	98351723/G//A	nonsynonymous	0	2
LCT	chr2	136566554/C//T	synonymous	0	2
C3orf20	chr3	14768435/G//A	nonsynonymous	0	2
SRPRB	chr3	133538480/G//A	nonsynonymous	0	2
FGF12	chr3	191861766/G//A	synonymous	0	2
CRMP1	chr4	5851121/G//A	nonsynonymous	0	2
NWD2	chr4	37445533/T//C	synonymous	0	2
LIMCH1	chr4	41664882/G//A	nonsynonymous	0	2
ADGRL3	chr4	62761553/G//T	nonsynonymous	0	2
FAT4	chr4	126411115/G//A	nonsynonymous	0	2
DCHS2	chr4	155219573/T//C	nonsynonymous	0	2
LIFR	chr5	38482253/G//C	nonsynonymous	0	2
MAP1B	chr5	71495815/G//A	synonymous	0	2
APC	chr5	112175273/C//T	nonsynonymous	0	2
PCDHA1	chr5	140167228/C//T	synonymous	0	2
C1QTNF2	chr5	159776163/G//A	synonymous	0	2
AARS2	chr6	44274757/C//T	nonsynonymous	0	2
RMND1	chr6	151766445/C//T	nonsynonymous	0	2
SYNE1	chr6	152469350/C//T	nonsynonymous	0	2
FOXK1	chr7	4802185/G//C	synonymous	0	2
CSMD1	chr8	3047526/G//A	nonsynonymous	0	2
MTMR7	chr8	17218683/C//T	synonymous	0	2
LOXL2	chr8	23185989/G//A	synonymous	0	2
DPYSL2	chr8	26501103/G//T	nonsynonymous	0	2
MRPS28	chr8	80831229/GTTC//G	nonsynonymous	0	2
CNBD1	chr8	88298809/C//T	nonsynonymous	0	2
TG	chr8	133899412/A//T	nonsynonymous	0	2
ADGRB1	chr8	143545763/C//T	synonymous	0	2
APBA1	chr9	72131497/G//A	synonymous	0	2
NAA35	chr9	88591601/G//A	nonsynonymous	0	2
BRINP1	chr9	122000972/C//T	nonsynonymous	0	2
	chr9	139757345/G//A	synonymous	0	2
ABCA2	chr9	139903022/G//A	nonsynonymous	0	2
ADARB2	chr10	1405391/C//T	synonymous	0	2
PITRM1	chr10	3214939/C//A	nonsynonymous	0	2
CXCL12	chr10	44868765/G//A	synonymous	0	2
CNNM1	chr10	101089496/G//A	nonsynonymous	0	2
ADAM8	chr10	135086353/G//T	nonsynonymous	0	2
E2F8	chr11	19258962/C//T	nonsynonymous	0	2
LGR4	chr11	27406830/G//T	nonsynonymous	0	2
LRRC55	chr11	56949887/G//A	nonsynonymous	0	2
NRXN2	chr11	64374714/G//A	nonsynonymous	0	2
MEN1	chr11	64577568/G//A	nonsynonymous	0	2
NUMA1	chr11	71719756/C//T	nonsynonymous	0	2
ELMOD1	chr11	107521111/C//A	nonsynonymous	0	2
CRACR2A	chr12	3757653/C//T	synonymous	0	2
GCN1	chr12	120575762/G//A	synonymous	0	2
SUPT20H	chr13	37614754/CAGG//C	nonsynonymous	0	2

ZC3H13	chr13	46539502/C//T	nonsynonymous	0	2
HEATR5A	chr14	31828141/G//A	nonsynonymous	0	2
SNX22	chr15	64445847/TC//T	synonymous	0	2
CREBBP	chr16	3843598/T//C	synonymous	0	2
BRD7	chr16	50357588/C//T	synonymous	0	2
SALL1	chr16	51175647/G//A	synonymous	0	2
CLEC3A	chr16	78064478/A//G	nonsynonymous	0	2
GGT6	chr17	4461592/G//C	nonsynonymous	0	2
BCL6B	chr17	6929887/G//A	nonsynonymous	0	2
CTC1	chr17	8133649/G//A	nonsynonymous	0	2
GPRC5C	chr17	72443319/G//T	synonymous	0	2
TMEM200C	chr18	5891793/C//T	synonymous	0	2
SMAD2	chr18	45374935/G//A	nonsynonymous	0	2
MKNK2	chr19	2042842/C//T	nonsynonymous	0	2
OR7G2	chr19	9213395/C//T	synonymous	0	2
SLC4A11	chr20	3209334/T//C	nonsynonymous	0	2
CENPB	chr20	3765987/C//T	nonsynonymous	0	2
JAG1	chr20	10620460/C//T	nonsynonymous	0	2
PLAGL2	chr20	30789847/C//T	synonymous	0	2
	chr20	50701266/C//T	synonymous	0	2
ATP5J	chr21	27097661/C//A	nonsynonymous	0	2
MYO18B	chr22	26422905/G//A	nonsynonymous	0	2
SEZ6L	chr22	26706747/C//T	synonymous	0	2
CSF2RB	chr22	37322096/C//T	nonsynonymous	0	2
RIBC2	chr22	45818219/C//T	synonymous	0	2
ZRSR2	chrX	15841226/G//A	nonsynonymous	0	2
KDM5C	chrX	53222475/G//A	nonsynonymous	0	2
PASD1	chrX	150791486/C//A	synonymous	0	2
SIGLEC10	chr19	51920196/G//T	nonsynonymous	1	1
ACAP3	chr1	1235203/G//A	synonymous	0	2
PRDM16	chr1	3329104/C//T	synonymous	0	2
DCLRE1B	chr1	114449646/A//G	nonsynonymous	0	2
SV2A	chr1	149882190/C//T	nonsynonymous	0	2
ASTN1	chr1	177001649/G//A	nonsynonymous	0	2
GREB1	chr2	11702707/C//T	synonymous	0	2
	chr2	50282102/G//A	synonymous	0	2
NEB	chr2	152584315/A//ATGCTGGCTGTGC	nonsynonymous	0	2
SGPP2	chr2	223423168/G//A	nonsynonymous	0	2
DZIP3	chr3	108380816/T//C	synonymous	0	2
COL6A5	chr3	130110250/G//A	nonsynonymous	0	2
PIK3R4	chr3	130463786/T//C	nonsynonymous	0	2
MME	chr3	154861260/C//A	nonsynonymous	0	2
VWA5B2	chr3	183956545/G//A	nonsynonymous	0	2
DGKQ	chr4	954474/C//T	synonymous	0	2
PPP2R2C	chr4	6331065/G//A	nonsynonymous	0	2
ETNPPL	chr4	109670527/T//A	nonsynonymous	0	2
ZNF622	chr5	16465616/C//A	synonymous	0	2
IL7R	chr5	35867514/A//T	nonsynonymous	0	2
PAPD4	chr5	78937020/G//A	nonsynonymous	0	2
PCDHA7	chr5	140215522/C//T	synonymous	0	2
PCDHAC2	chr5	140347709/G//A	nonsynonymous	0	2
PCDHGB6	chr5	140790074/T//C	nonsynonymous	0	2
TENM2	chr5	167645211/C//T	nonsynonymous	0	2
FGFR4	chr5	176519746/G//A	nonsynonymous	0	2
C6orf10	chr6	32261696/C//T	nonsynonymous	0	2
MDGA1	chr6	37616903/C//T	synonymous	0	2
TIAM2	chr6	155450852/G//A	synonymous	0	2
HGF	chr7	81372780/C//T	nonsynonymous	0	2
DOCK4	chr7	111509677/G//A	nonsynonymous	0	2
CSMD1	chr8	3045415/C//T	nonsynonymous	0	2
SPIN1	chr9	91077434/C//T	nonsynonymous	0	2
TTC16	chr9	130479679/C//T	synonymous	0	2
ZEB1	chr10	31809845/G//T	nonsynonymous	0	2
PPIF	chr10	81111308/C//T	synonymous	0	2
AHNAK	chr11	62297794/G//A	synonymous	0	2
CD163	chr12	7636108/T//A	nonsynonymous	0	2
APOBEC1	chr12	7805421/C//A	nonsynonymous	0	2
AQP2	chr12	50344571/C//A	synonymous	0	2
TNS2	chr12	53453037/C//T	nonsynonymous	0	2
PTPRB	chr12	70948999/C//T	nonsynonymous	0	2

MLNR	chr13	49796404/C//T	nonsynonymous	0	2
FANCM	chr14	45628283/TTA//T	nonsynonymous	0	2
CREBBP	chr16	3781903/C//G	nonsynonymous	0	2
MYH11	chr16	15820824/TGGCCTG//T	nonsynonymous	0	2
FBXL19	chr16	30958241/C//T	synonymous	0	2
IRX6	chr16	55362731/A//G	nonsynonymous	0	2
PLCG2	chr16	81939031/C//T	synonymous	0	2
MYO5B	chr18	47431095/C//T	nonsynonymous	0	2
ADGRE1	chr19	6937658/C//T	nonsynonymous	0	2
FBN3	chr19	8152978/G//A	synonymous	0	2
ZNF560	chr19	9577933/G//A	nonsynonymous	0	2
ICAM3	chr19	10446447/G//A	synonymous	0	2
ACP7	chr19	39589642/G//A	nonsynonymous	0	2
ZNF155	chr19	44501401/C//A	nonsynonymous	0	2
	chr19	49464247/G//A	synonymous	0	2
OCSTAMP	chr20	45174689/G//T	synonymous	0	2
FNDC11	chr20	62187762/C//T	nonsynonymous	0	2
PPM1F	chr22	22285597/G//A	nonsynonymous	0	2
IL1RAPL1	chrX	29973828/G//A	nonsynonymous	0	2
KLHL4	chrX	86869549/T//A	nonsynonymous	0	2
RGAG1	chrX	109697441/G//C	nonsynonymous	0	2
CAMTA1	chr1	7723800/C//T	nonsynonymous	0	2
IFI44L	chr1	79093956/C//T	nonsynonymous	0	2
PLXNA2	chr1	208390159/C//T	nonsynonymous	0	2
SPRTN	chr1	231488837/T//G	synonymous	0	2
MEIS1	chr2	66739344/A//C	nonsynonymous	0	2
DYSF	chr2	71755471/C//T	synonymous	0	2
SCN7A	chr2	167263067/G//A	nonsynonymous	0	2
LRP2	chr2	170044681/G//A	nonsynonymous	0	2
TTN	chr2	179639685/A//G	synonymous	0	2
TTLL4	chr2	219611877/C//T	nonsynonymous	0	2
LHFPL4	chr3	9594226/C//T	nonsynonymous	0	2
SLC6A11	chr3	10916701/G//A	nonsynonymous	0	2
DCBLD2	chr3	98518560/C//A	nonsynonymous	0	2
COPG1	chr3	128979179/T//G	nonsynonymous	0	2
CEP70	chr3	138216922/T//A	nonsynonymous	0	2
MCF2L2	chr3	182897473/C//A	nonsynonymous	0	2
TMPRSS11F	chr4	68930433/C//T	nonsynonymous	0	2
ENAM	chr4	71495230/C//T	nonsynonymous	0	2
PDLIM5	chr4	95496909/C//T	nonsynonymous	0	2
ADH4	chr4	100048388/G//T	synonymous	0	2
UGT3A1	chr5	35955903/C//T	nonsynonymous	0	2
PITX1	chr5	134364597/C//T	nonsynonymous	0	2
FLT4	chr5	180047286/G//A	nonsynonymous	0	2
GRIK2	chr6	102134198/C//T	synonymous	0	2
FOXO3	chr6	108985058/C//T	nonsynonymous	0	2
AKAP12	chr6	151670781/G//C	nonsynonymous	0	2
IDO2	chr8	39840287/C//T	synonymous	0	2
FAM83H	chr8	144809386/C//T	nonsynonymous	0	2
SPATA31D1	chr9	84609179/G//A	nonsynonymous	0	2
TNC	chr9	117848337/C//A	nonsynonymous	0	2
	chr9	130529487/C//T	synonymous	0	2
PTGES	chr9	132501843/C//T	synonymous	0	2
CAMSAP1	chr9	138713625/T//A	nonsynonymous	0	2
CYP2C9	chr10	96748635/C//T	synonymous	0	2
TCF7L2	chr10	114920402/G//A	nonsynonymous	0	2
SSRP1	chr11	57100235/C//G	nonsynonymous	0	2
ZP1	chr11	60640892/G//A	nonsynonymous	0	2
SIPA1	chr11	65408752/C//T	synonymous	0	2
NDUFV1	chr11	67374452/A//C	synonymous	0	2
HIKESHI	chr11	86013450/C//T	synonymous	0	2
OPCML	chr11	132527154/G//A	synonymous	0	2
FKBP4	chr12	2910433/G//A	nonsynonymous	0	2
GLI1	chr12	57858986/G//A	nonsynonymous	0	2
ZC3H13	chr13	46539484/C//T	nonsynonymous	0	2
ANKRD9	chr14	102974154/G//A	nonsynonymous	0	2
AHNAK2	chr14	105409226/C//T	nonsynonymous	0	2
TP53	chr17	7577139/G//C	nonsynonymous	0	2
PIK3R5	chr17	8791712/G//A	synonymous	0	2
TRAF4	chr17	27074841/TCCCCCATCCCTA//T	nonsynonymous	0	2

GPR179	chr17	36489255/C//T	nonsynonymous	0	2
TCAP	chr17	37821645/G//A	synonymous	0	2
TMC6	chr17	76121807/C//T	nonsynonymous	0	2
TSHZ3	chr19	31767470/C//A	nonsynonymous	0	2
B9D2	chr19	41860701/G//A	synonymous	0	2
RSPH6A	chr19	46313870/C//T	synonymous	0	2
EMC10	chr19	50983941/G//A	nonsynonymous	0	2
CFAP61	chr20	20257861/C//T	nonsynonymous	0	2
CDH4	chr20	60448849/C//T	nonsynonymous	0	2
KDM6A	chrX	44929133/G//T	nonsynonymous	0	2
DACH2	chrX	85769337/C//T	nonsynonymous	0	2
PLP1	chrX	103041545/G//A	nonsynonymous	0	2
LRRC7	chr1	70486713/T//C	synonymous	0	2
HRNR	chr1	152191793/C//A	nonsynonymous	0	2
CD5L	chr1	157804297/C//A	nonsynonymous	0	2
QSOX1	chr1	180166009/G//T	nonsynonymous	0	2
DHX9	chr1	182827993/G//T	nonsynonymous	0	2
CNTNAP5	chr2	124999880/C//T	synonymous	0	2
ITGA4	chr2	182350694/C//T	synonymous	0	2
KCNJ13	chr2	233633251/G//C	nonsynonymous	0	2
RAF1	chr3	12645699/G//A	nonsynonymous	0	2
ZKSCAN7	chr3	44598526/T//C	synonymous	0	2
BSN	chr3	49691542/C//A	nonsynonymous	0	2
ADAMTS9	chr3	64672386/G//A	nonsynonymous	0	2
SHOX2	chr3	157817721/G//T	synonymous	0	2
PAK2	chr3	196530018/T//A	nonsynonymous	0	2
PRDM5	chr4	121631494/C//T	synonymous	0	2
KIAA1109	chr4	123128331/A//G	nonsynonymous	0	2
HELT	chr4	185941627/G//A	nonsynonymous	0	2
CWC27	chr5	64267525/T//G	synonymous	0	2
APC	chr5	112170864/T//C	nonsynonymous	0	2
DAAM2	chr6	39869681/C//A	nonsynonymous	0	2
TBX18	chr6	85457773/C//T	synonymous	0	2
RTN4IP1	chr6	107076875/C//T	nonsynonymous	0	2
STK31	chr7	23871924/C//T	nonsynonymous	0	2
ARF5	chr7	127228594/G//A	synonymous	0	2
NOBOX	chr7	144098453/C//A	nonsynonymous	0	2
LOXL2	chr8	23225536/G//T	nonsynonymous	0	2
DMRT3	chr9	990483/C//T	synonymous	0	2
IKBKAP	chr9	111641032/T//A	nonsynonymous	0	2
ARHGAP21	chr10	24884718/G//A	nonsynonymous	0	2
PTCHD3	chr10	27702276/C//T	nonsynonymous	0	2
FAS	chr10	90767508/T//G	nonsynonymous	0	2
CFAP43	chr10	105942151/T//C	synonymous	0	2
TCF7L2	chr10	114920404/G//T	nonsynonymous	0	2
OR5P3	chr11	7847244/G//T	synonymous	0	2
OR4A5	chr11	51412067/A//T	nonsynonymous	0	2
OR5L1	chr11	55578986/T//C	nonsynonymous	0	2
OR5B2	chr11	58190376/C//T	nonsynonymous	0	2
HSPA8	chr11	122928630/G//T	synonymous	0	2
DERA	chr12	16109906/C//T	nonsynonymous	0	2
GALNT6	chr12	51754523/G//A	synonymous	0	2
KRT78	chr12	53241867/C//T	synonymous	0	2
TMEM132D	chr12	129563223/AG//A	nonsynonymous	0	2
BRCA2	chr13	32910553/T//G	nonsynonymous	0	2
MYO16	chr13	109792839/G//A	nonsynonymous	0	2
OR4M1	chr14	20249096/G//T	synonymous	0	2
FAM179B	chr14	45475282/G//C	nonsynonymous	0	2
CACNA1H	chr16	1263776/G//T	synonymous	0	2
MYH11	chr16	15876241/C//T	nonsynonymous	0	2
RNF40	chr16	30783454/G//A	synonymous	0	2
PIEZO1	chr16	88800780/G//A	nonsynonymous	0	2
INPP5K	chr17	1399652/G//A	synonymous	0	2
MYH4	chr17	10369637/C//T	nonsynonymous	0	2
MYO18A	chr17	27423853/A//G	synonymous	0	2
HOXB6	chr17	46675286/G//A	nonsynonymous	0	2
APC2	chr19	1453491/G//A	synonymous	0	2
ADAMTS10	chr19	8650462/C//T	nonsynonymous	0	2
FUT1	chr19	49253669/C//T	synonymous	0	2
LRRC4B	chr19	51022293/G//A	nonsynonymous	0	2

KMT5C	chr19	55854165/G//T	nonsynonymous	0	2
PEG3	chr19	57327460/A//G	nonsynonymous	0	2
STK4	chr20	43629826/TCTGGCACGATGGTT(	nonsynonymous	0	2
PCK1	chr20	56139658/G//A	nonsynonymous	0	2
COL9A3	chr20	61461755/G//A	nonsynonymous	0	2
TIAM1	chr21	32624094/G//A	nonsynonymous	0	2
SPECC1L	chr22	24709364/C//G	nonsynonymous	0	2
PASD1	chrX	150832640/C//A	nonsynonymous	0	2
ECE1	chr1	21551775/C//T	nonsynonymous	0	2
C8A	chr1	57373778/G//A	nonsynonymous	0	2
MCL1	chr1	150551905/TCGCCCTCCCGGGC(	nonsynonymous	0	2
IGFN1	chr1	201175370/C//T	nonsynonymous	0	2
ATP2B4	chr1	203678532/G//A	nonsynonymous	0	2
PROX1	chr1	214209168/T//A	synonymous	0	2
OBSCN	chr1	228474735/T//A	nonsynonymous	0	2
ALLC	chr2	3744999/A//G	nonsynonymous	0	2
DYSF	chr2	71913688/C//T	synonymous	0	2
FER1L5	chr2	97368466/C//T	synonymous	0	2
LONRF2	chr2	100900912/C//T	nonsynonymous	0	2
SCTR	chr2	120252125/C//T	nonsynonymous	0	2
TTN	chr2	179584079/G//A	nonsynonymous	0	2
HDAC4	chr2	240056298/G//A	synonymous	0	2
PDCD6IP	chr3	33885677/G//A	nonsynonymous	0	2
DNAH1	chr3	52430754/C//T	nonsynonymous	0	2
CCDC80	chr3	112357368/C//T	nonsynonymous	0	2
ZBBX	chr3	167086345/C//T	nonsynonymous	0	2
TBL1XR1	chr3	176768329/C//T	nonsynonymous	0	2
ECE2	chr3	184008894/G//A	nonsynonymous	0	2
FDCSP	chr4	71099843/G//A	nonsynonymous	0	2
HSD17B11	chr4	88261668/A//C	synonymous	0	2
CTNND2	chr5	11082938/G//A	synonymous	0	2
TRIO	chr5	14399169/G//A	nonsynonymous	0	2
RAPGEF6	chr5	130766721/A//C	nonsynonymous	0	2
PCDHA12	chr5	140255780/G//A	synonymous	0	2
LARP1	chr5	154181859/A//G	nonsynonymous	0	2
HCG4	chr6	29760561/T//C	synonymous	0	2
KLC4	chr6	43038532/C//T	nonsynonymous	0	2
SYNE1	chr6	152652399/C//T	nonsynonymous	0	2
TCTE3	chr6	170140249/G//A	synonymous	0	2
AIMP2	chr7	6048934/T//G	synonymous	0	2
FAM126A	chr7	22985434/G//A	nonsynonymous	0	2
NOD1	chr7	30491333/C//T	nonsynonymous	0	2
ADAM22	chr7	87743914/C//T	synonymous	0	2
CPED1	chr7	120884298/C//T	nonsynonymous	0	2
DLGAP2	chr8	1497054/C//T	synonymous	0	2
CSMD1	chr8	3057245/C//T	nonsynonymous	0	2
GFRA2	chr8	21632502/G//A	nonsynonymous	0	2
UBAP2	chr9	33956082/C//T	synonymous	0	2
AKR1E2	chr10	4889406/C//A	nonsynonymous	0	2
ITIH5	chr10	7621738/G//A	synonymous	0	2
OR51G1	chr11	4945161/C//T	nonsynonymous	0	2
AMBRA1	chr11	46430124/G//A	synonymous	0	2
OR4C13	chr11	49974970/G//T	synonymous	0	2
OR6Q1	chr11	57798846/C//T	nonsynonymous	0	2
GDPD5	chr11	75160109/G//A	synonymous	0	2
KIRREL3	chr11	126391225/G//A	nonsynonymous	0	2
ADAMTS20	chr12	43819382/C//T	nonsynonymous	0	2
C12orf42	chr12	103700000/C//T	nonsynonymous	0	2
MYL2	chr12	111358330/C//T	nonsynonymous	0	2
GJA3	chr13	20716272/C//T	nonsynonymous	0	2
LATS2	chr13	21557735/C//T	nonsynonymous	0	2
RNF17	chr13	25418809/G//T	nonsynonymous	0	2
SPERT	chr13	46287865/C//T	synonymous	0	2
CPB2	chr13	46656636/G//A	synonymous	0	2
COL4A1	chr13	110823045/G//A	synonymous	0	2
SFTA3	chr14	36946213/G//A	nonsynonymous	0	2
LRRC74A	chr14	77336556/G//A	synonymous	0	2
SEPT12	chr16	4835998/AG//A	nonsynonymous	0	2
AXIN2	chr17	63554657/C//T	nonsynonymous	0	2
CARD14	chr17	78171953/C//T	synonymous	0	2

CBLN2	chr18	70205461/C//T	nonsynonymous	0	2
THEG	chr19	374015/C//T	synonymous	0	2
DNMT1	chr19	10270744/G//C	synonymous	0	2
TMEM221	chr19	17547644/C//A	nonsynonymous	0	2
ZNF526	chr19	42729489/C//T	nonsynonymous	0	2
FPR1	chr19	52249881/G//A	nonsynonymous	0	2
PPP2R1A	chr19	52715982/C//T	nonsynonymous	0	2
BIRC8	chr19	53793270/G//A	nonsynonymous	0	2
GZF1	chr20	23345225/AGT//A	nonsynonymous	0	2
LAMA5	chr20	60899182/C//T	nonsynonymous	0	2
MICAL3	chr22	18300834/G//T	nonsynonymous	0	2
MGAT3	chr22	39884331/G//A	nonsynonymous	0	2
SSX3	chrX	48214650/G//A	nonsynonymous	0	2
NRK	chrX	105066794/G//A	synonymous	0	2
FAM213B	chr1	2520877/G//A	synonymous	0	2
SH2D5	chr1	21049310/G//A	nonsynonymous	0	2
C1QB	chr1	22987524/G//A	nonsynonymous	0	2
GNL2	chr1	38056437/C//A	nonsynonymous	0	2
PODN	chr1	53546439/G//A	nonsynonymous	0	2
PRMT6	chr1	107600225/C//A	nonsynonymous	0	2
CACNA1E	chr1	181731751/C//T	synonymous	0	2
RYR2	chr1	237955613/A//C	nonsynonymous	0	2
KIF26B	chr1	245849809/C//T	nonsynonymous	0	2
ZNF513	chr2	27600703/C//A	synonymous	0	2
STON1	chr2	48808268/C//A	nonsynonymous	0	2
NRXN1	chr2	50850512/C//A	synonymous	0	2
SFXN5	chr2	73226130/G//A	nonsynonymous	0	2
MAP3K19	chr2	135749117/C//T	nonsynonymous	0	2
ARHGAP15	chr2	144381721/C//T	synonymous	0	2
STAT4	chr2	191929525/C//A	synonymous	0	2
MST1	chr3	49723305/G//A	nonsynonymous	0	2
PARP9	chr3	122274665/A//G	nonsynonymous	0	2
UMPS	chr3	124449214/T//C	synonymous	0	2
GRXCR1	chr4	43032468/C//T	nonsynonymous	0	2
PCDH10	chr4	134072693/G//A	synonymous	0	2
HMGCR	chr5	74655296/T//G	nonsynonymous	0	2
PCDHA11	chr5	140250554/C//T	synonymous	0	2
PCDHAC2	chr5	140346777/C//T	synonymous	0	2
FAT2	chr5	150911165/C//T	nonsynonymous	0	2
ZSCAN12	chr6	28366007/C//A	nonsynonymous	0	2
BACH2	chr6	90660863/G//A	nonsynonymous	0	2
FYN	chr6	111995778/G//A	synonymous	0	2
ROS1	chr6	117718079/T//A	nonsynonymous	0	2
KIAA0408	chr6	127765360/C//T	nonsynonymous	0	2
SDK1	chr7	4249744/G//A	nonsynonymous	0	2
AOAH	chr7	36580001/G//T	synonymous	0	2
GLI3	chr7	42004777/C//T	synonymous	0	2
ZNF789	chr7	99084545/T//A	nonsynonymous	0	2
NUB1	chr7	151065011/A//T	nonsynonymous	0	2
LYN	chr8	56922678/G//T	synonymous	0	2
KLHL38	chr8	124658060/G//A	synonymous	0	2
MTSS1	chr8	125568569/G//T	nonsynonymous	0	2
NRAP	chr10	115406711/C//T	nonsynonymous	0	2
EIF3A	chr10	120801889/G//A	nonsynonymous	0	2
PANX3	chr11	124487339/G//A	nonsynonymous	0	2
IQSEC3	chr12	247858/C//T	synonymous	0	2
A2ML1	chr12	9010551/G//A	synonymous	0	2
SLC38A1	chr12	46602843/C//G	nonsynonymous	0	2
KMT5A	chr12	123892134/G//A	nonsynonymous	0	2
FZD10	chr12	130647943/C//G	nonsynonymous	0	2
TRPC4	chr13	38266260/T//C	synonymous	0	2
TGDS	chr13	95246124/G//A	nonsynonymous	0	2
MYO16	chr13	109753151/T//C	synonymous	0	2
MBIP	chr14	36768231/A//G	synonymous	0	2
DCAF4	chr14	73422355/G//A	nonsynonymous	0	2
ABCA3	chr16	2331096/C//T	nonsynonymous	0	2
PPL	chr16	4953882/C//T	synonymous	0	2
PDILT	chr16	20386176/C//T	nonsynonymous	0	2
DNAH3	chr16	20946714/G//A	nonsynonymous	0	2
SH2B1	chr16	28880552/G//A	nonsynonymous	0	2

CX3CL1	chr16	57416074/C//T	synonymous	0	2
HOXB2	chr17	46620402/G//A	synonymous	0	2
WFIKKN2	chr17	48917544/G//C	nonsynonymous	0	2
FOXJ1	chr17	74134149/C//T	nonsynonymous	0	2
FASN	chr17	80043361/G//T	nonsynonymous	0	2
ADAMTS10	chr19	8650468/G//A	nonsynonymous	0	2
SMARCA4	chr19	11144072/C//A	nonsynonymous	0	2
NOTCH3	chr19	15290912/G//A	nonsynonymous	0	2
LRRC25	chr19	18507178/C//T	nonsynonymous	0	2
FFAR1	chr19	35843150/G//A	synonymous	0	2
ZNF222	chr19	44536820/T//G	nonsynonymous	0	2
BPIFB6	chr20	31625457/G//A	synonymous	0	2
WDR4	chr21	44270219/C//A	synonymous	0	2
SRPX	chrX	38079975/C//CGCA	nonsynonymous	0	2
USP11	chrX	47100800/A//G	nonsynonymous	0	2
TESK2	chr1	45887414/G//A	synonymous	0	2
DCAF8	chr1	160206935/G//A	nonsynonymous	0	2
HEATR5B	chr2	37306396/G//A	nonsynonymous	0	2
PUS10	chr2	61172166/T//C	nonsynonymous	0	2
CFAP65	chr2	219867731/C//T	synonymous	0	2
ACTR8	chr3	53902802/C//T	nonsynonymous	0	2
MAGI1	chr3	65350364/C//T	synonymous	0	2
USF3	chr3	113392204/GTGTCTCCCGGTTTT	nonsynonymous	0	2
NMD3	chr3	160967265/C//T	synonymous	0	2
NCEH1	chr3	172351279/C//G	nonsynonymous	0	2
NLGN1	chr3	173993172/A//C	nonsynonymous	0	2
UGT2B11	chr4	70079712/A//T	synonymous	0	2
ARHGAP10	chr4	148796175/C//T	nonsynonymous	0	2
DNAH5	chr5	13862716/C//T	synonymous	0	2
IL7R	chr5	35871196/C//A	synonymous	0	2
PARP8	chr5	50057714/A//G	synonymous	0	2
FBN2	chr5	127674754/C//G	nonsynonymous	0	2
KDM3B	chr5	137761134/A//G	nonsynonymous	0	2
PCDHA7	chr5	140215249/G//A	synonymous	0	2
SLIT3	chr5	168137998/G//A	nonsynonymous	0	2
HTR1E	chr6	87725612/C//T	nonsynonymous	0	2
GJA10	chr6	90605509/G//GACATC	nonsynonymous	0	2
EPHA7	chr6	93965748/T//A	nonsynonymous	0	2
OSTM1	chr6	108395521/CAG//C	nonsynonymous	0	2
UTRN	chr6	144860503/C//G	nonsynonymous	0	2
GINM1	chr6	149900993/A//C	nonsynonymous	0	2
CCDC129	chr7	31617627/G//A	nonsynonymous	0	2
CFTR	chr7	117180285/G//A	nonsynonymous	0	2
WRN	chr8	30921904/T//C	synonymous	0	2
TMEM74	chr8	109796964/G//A	nonsynonymous	0	2
ADGRB1	chr8	143604078/C//T	synonymous	0	2
RECQL4	chr8	145742130/G//A	nonsynonymous	0	2
CEL	chr9	135942291/C//A	nonsynonymous	0	2
SEC16A	chr9	139370315/G//A	nonsynonymous	0	2
MRGPRE	chr11	3249856/G//A	synonymous	0	2
APBB1	chr11	6424583/C//T	nonsynonymous	0	2
FAT3	chr11	92086160/G//T	synonymous	0	2
BSX	chr11	122852256/C//T	nonsynonymous	0	2
GRAMD1B	chr11	123489466/C//T	nonsynonymous	0	2
CBX5	chr12	54651349/C//T	nonsynonymous	0	2
PROSER1	chr13	39588297/G//T	synonymous	0	2
F7	chr13	113770069/G//A	nonsynonymous	0	2
MOK	chr14	102717163/G//C	nonsynonymous	0	2
FBN1	chr15	48707932/C//T	nonsynonymous	0	2
CCDC154	chr16	1486005/G//A	nonsynonymous	0	2
SEC14L5	chr16	5064997/C//T	synonymous	0	2
CCDC113	chr16	58314676/A//T	synonymous	0	2
VEZF1	chr17	56060147/T//C	nonsynonymous	0	2
UNC13D	chr17	73831953/G//A	nonsynonymous	0	2
RTTN	chr18	67742760/A//T	nonsynonymous	0	2
ZSWIM4	chr19	13928047/TCCA//T	nonsynonymous	0	2
KLHL26	chr19	18779306/G//A	nonsynonymous	0	2
PROKR2	chr20	5294763/G//A	nonsynonymous	0	2
DZANK1	chr20	18414375/G//A	nonsynonymous	0	2
FNDC11	chr20	62187657/G//A	nonsynonymous	0	2

TPTE	chr21	10934035/T//G	synonymous	0	2
RWDD2B	chr21	30391617/T//C	synonymous	0	2
MX2	chr21	42754364/G//A	nonsynonymous	0	2
RFPL3	chr22	32754420/G//A	nonsynonymous	0	2
MSL3	chrX	11783633/C//T	nonsynonymous	0	2
MBTPS2	chrX	21871489/C//T	synonymous	0	2
PPP1R3F	chrX	49126925/A//G	nonsynonymous	0	2
ARHGAP36	chrX	130217871/C//T	synonymous	0	2
SPANXN3	chrX	142596998/A//C	synonymous	0	2
PTGFRN	chr1	117527548/C//T	nonsynonymous	0	2
MSTO1	chr1	155582731/C//A	nonsynonymous	0	2
OR2T6	chr1	248551152/G//T	nonsynonymous	0	2
KIDINS220	chr2	8874786/G//A	nonsynonymous	0	2
GCFC2	chr2	75900601/C//G	nonsynonymous	0	2
TCF7L1	chr2	85532424/C//T	nonsynonymous	0	2
NEB	chr2	152411476/C//T	nonsynonymous	0	2
LRP2	chr2	170003430/G//A	synonymous	0	2
OTOS	chr2	241078536/C//T	synonymous	0	2
PARP14	chr3	122437079/G//T	nonsynonymous	0	2
CFAP99	chr4	2452697/T//A	synonymous	0	2
ANAPC4	chr4	25415267/A//G	nonsynonymous	0	2
PDGFRA	chr4	55152093/A//T	nonsynonymous	0	2
TNXB	chr6	32023660/C//T	nonsynonymous	0	2
RBAK-RBAKDN	chr7	5096949/G//T	synonymous	0	2
	chr7	18201897/C//T	synonymous	0	2
POU6F2	chr7	39503937/G//A	synonymous	0	2
PKHD1L1	chr8	110476857/G//C	nonsynonymous	0	2
TSC1	chr9	135782182/ATCACTTAGAGTGAC	nonsynonymous	0	2
SLC17A6	chr11	22363122/G//A	synonymous	0	2
APIP	chr11	34912080/G//A	nonsynonymous	0	2
YPEL4	chr11	57414250/A//G	synonymous	0	2
ZDHHC5	chr11	57466446/A//G	nonsynonymous	0	2
MAP3K11	chr11	65365989/G//T	nonsynonymous	0	2
LRRK2	chr12	40618982/A//G	nonsynonymous	0	2
TSFM	chr12	58189959/G//A	nonsynonymous	0	2
NUDT21	chr16	56485007/G//A	synonymous	0	2
ZNF385C	chr17	40180813/C//T	nonsynonymous	0	2
DSC3	chr18	28611146/T//C	synonymous	0	2
GRAMD1A	chr19	35500336/A//T	nonsynonymous	0	2
LILRA3	chr19	54803751/G//A	nonsynonymous	0	2
AP1B1	chr22	29727508/G//C	nonsynonymous	0	2
ZNF676	chr19	22363610/A//G	synonymous	1	1
ARHGEF16	chr1	3396094/C//T	nonsynonymous	0	2
LAPTM5	chr1	31230568/G//A	nonsynonymous	0	2
GNL2	chr1	38042024/C//T	synonymous	0	2
MROH7	chr1	55158132/G//A	nonsynonymous	0	2
	chr1	154131494/C//T	synonymous	0	2
ARHGEF11	chr1	156915924/G//A	synonymous	0	2
TNR	chr1	175335134/C//T	nonsynonymous	0	2
HMCN1	chr1	186089174/C//A	synonymous	0	2
CFH	chr1	196697476/C//T	nonsynonymous	0	2
SOX13	chr1	204093952/G//A	nonsynonymous	0	2
CD55	chr1	207510701/C//A	nonsynonymous	0	2
RCOR3	chr1	211433487/G//A	synonymous	0	2
HHIPL2	chr1	222717155/A//G	nonsynonymous	0	2
TLR5	chr1	223286273/C//T	nonsynonymous	0	2
SOX11	chr2	5833832/G//A	nonsynonymous	0	2
GREB1	chr2	11774317/G//A	synonymous	0	2
DNAJC27	chr2	25180016/G//A	nonsynonymous	0	2
C2orf71	chr2	29293988/C//T	nonsynonymous	0	2
CYP1B1	chr2	38301932/G//A	synonymous	0	2
EML6	chr2	55094009/A//C	nonsynonymous	0	2
EML6	chr2	55130533/A//G	synonymous	0	2
DCTN1	chr2	74605336/C//T	nonsynonymous	0	2
CTNNA2	chr2	80136861/G//A	nonsynonymous	0	2
RETSAT	chr2	85573121/G//A	nonsynonymous	0	2
ZAP70	chr2	98351754/C//T	nonsynonymous	0	2
ZAP70	chr2	98354255/C//T	synonymous	0	2
VWA3B	chr2	98737854/C//T	nonsynonymous	0	2
SULT1C2	chr2	108921879/G//A	synonymous	0	2

PSD4	chr2	113956750/C//T	nonsynonymous	0	2
LRP1B	chr2	141986816/G//A	synonymous	0	2
SCN1A	chr2	166897971/C//A	nonsynonymous	0	2
LRP2	chr2	170011077/C//T	nonsynonymous	0	2
TTN	chr2	179605646/C//G	nonsynonymous	0	2
MDH1B	chr2	207619823/G//A	nonsynonymous	0	2
SLC4A7	chr3	27477943/C//T	synonymous	0	2
DCLK3	chr3	36778833/A//G	nonsynonymous	0	2
BSN	chr3	49693617/AC//A	nonsynonymous	0	2
OR5K4	chr3	98073013/C//T	nonsynonymous	0	2
ADPRH	chr3	119305168/C//T	nonsynonymous	0	2
PIK3CA	chr3	178916941/GAAA//G	nonsynonymous	0	2
DGKQ	chr4	961354/C//T	nonsynonymous	0	2
STIM2	chr4	27024157/G//A	nonsynonymous	0	2
ARHGAP24	chr4	86921886/G//A	synonymous	0	2
ABCE1	chr4	146025614/A//T	nonsynonymous	0	2
FGG	chr4	155530878/G//A	synonymous	0	2
FAM218A	chr4	165878415/C//T	nonsynonymous	0	2
KLHL2	chr4	166239042/G//A	synonymous	0	2
TLL1	chr4	167020440/C//T	nonsynonymous	0	2
ADGRV1	chr5	89968532/G//T	nonsynonymous	0	2
CDO1	chr5	115151945/G//A	synonymous	0	2
PCDHA2	chr5	140176028/G//A	synonymous	0	2
PCDHGA1	chr5	140711963/G//C	nonsynonymous	0	2
RARS	chr5	167919821/ATCAG//A	nonsynonymous	0	2
	chr5	176917504/C//T	synonymous	0	2
FLT4	chr5	180030261/C//T	synonymous	0	2
HIST1H2AM	chr6	27860732/G//A	synonymous	0	2
ZSCAN23	chr6	28403896/G//A	nonsynonymous	0	2
ADGRF4	chr6	47682880/C//G	nonsynonymous	0	2
ZNF451	chr6	57018666/G//A	nonsynonymous	0	2
EPHA7	chr6	93982096/G//A	nonsynonymous	0	2
MAS1	chr6	160328810/G//A	nonsynonymous	0	2
THSD7A	chr7	11447009/C//A	nonsynonymous	0	2
MYO1G	chr7	45010636/G//A	nonsynonymous	0	2
KCND2	chr7	120385981/C//T	nonsynonymous	0	2
ZNF800	chr7	127014530/C//G	nonsynonymous	0	2
TNPO3	chr7	128622312/C//T	nonsynonymous	0	2
DENND2A	chr7	140301451/G//A	synonymous	0	2
OR2F1	chr7	143657178/G//A	nonsynonymous	0	2
PXDNL	chr8	52320945/G//A	nonsynonymous	0	2
CYP7B1	chr8	65711080/A//G	nonsynonymous	0	2
GML	chr8	143921917/C//T	nonsynonymous	0	2
IFNE	chr9	21481267/G//T	nonsynonymous	0	2
TAF1L	chr9	32631823/C//T	nonsynonymous	0	2
SHC3	chr9	91656987/C//T	synonymous	0	2
C9orf84	chr9	114484839/G//A	nonsynonymous	0	2
TOR2A	chr9	130494917/C//T	nonsynonymous	0	2
DBH	chr9	136513028/C//T	nonsynonymous	0	2
SOHLH1	chr9	138590296/C//T	nonsynonymous	0	2
ITIH5	chr10	7621854/G//A	nonsynonymous	0	2
GPR158	chr10	25887039/G//A	synonymous	0	2
PTCHD3	chr10	27703184/T//A	synonymous	0	2
GDF2	chr10	48413661/C//T	nonsynonymous	0	2
NPFFR1	chr10	72014923/G//A	synonymous	0	2
PYROXD2	chr10	100147036/A//T	synonymous	0	2
TCF7L2	chr10	114920401/C//T	nonsynonymous	0	2
KCNK18	chr10	118969094/G//A	nonsynonymous	0	2
SEC23IP	chr10	121658296/G//A	nonsynonymous	0	2
DPYSL4	chr10	134006332/G//T	nonsynonymous	0	2
MRVI1	chr11	10715175/C//T	synonymous	0	2
OR8J1	chr11	56128395/A//G	nonsynonymous	0	2
OR8U8	chr11	56143923/C//A	nonsynonymous	0	2
NDUFV1	chr11	67376110/G//A	synonymous	0	2
GUCY1A2	chr11	106558338/C//T	synonymous	0	2
GUCY1A2	chr11	106810562/C//A	nonsynonymous	0	2
ATM	chr11	108170615/GAGT//G	synonymous	0	2
TMEM225	chr11	123755951/C//T	nonsynonymous	0	2
ABCD2	chr12	40013370/T//A	nonsynonymous	0	2
COL2A1	chr12	48372442/C//T	nonsynonymous	0	2

EIF4B	chr12	53415611/C//T	nonsynonymous	0	2
LHX5	chr12	113906191/C//T	nonsynonymous	0	2
MSI1	chr12	120789172/G//A	synonymous	0	2
RIMBP2	chr12	130921759/G//A	synonymous	0	2
MAB21L1	chr13	36050056/A//C	nonsynonymous	0	2
NALCN	chr13	101881877/T//C	nonsynonymous	0	2
KCNH5	chr14	63447858/G//T	nonsynonymous	0	2
VASH1	chr14	77242499/C//T	synonymous	0	2
AQR	chr15	35252994/T//C	nonsynonymous	0	2
ADAMTSL3	chr15	84582093/C//T	synonymous	0	2
PAGR1	chr16	29828254/T//C	synonymous	0	2
	chr16	47497822/C//T	synonymous	0	2
CNGB1	chr16	57937766/C//G	nonsynonymous	0	2
CDH13	chr16	83828646/C//T	synonymous	0	2
CYBA	chr16	88713185/CAT//C	nonsynonymous	0	2
TUBB3	chr16	90001447/G//A	synonymous	0	2
PSMD11	chr17	30781579/G//T	nonsynonymous	0	2
SMG8	chr17	57289739/A//G	synonymous	0	2
CEP295NL	chr17	76887123/T//C	nonsynonymous	0	2
CTDP1	chr18	77473087/G//A	nonsynonymous	0	2
PSPN	chr19	6375712/C//A	nonsynonymous	0	2
OR1I1	chr19	15198417/C//A	nonsynonymous	0	2
BORCS8-MEF2B	chr19	19256573/G//A	nonsynonymous	0	2
FXYD5	chr19	35655083/C//T	nonsynonymous	0	2
LIN37	chr19	36239657/C//A	nonsynonymous	0	2
CLIP3	chr19	36518084/G//A	nonsynonymous	0	2
ZNF224	chr19	44612386/G//A	synonymous	0	2
ZNF347	chr19	53643536/C//T	synonymous	0	2
ZSCAN18	chr19	58598713/G//A	synonymous	0	2
BPIFB4	chr20	31671576/C//T	synonymous	0	2
BPIFB4	chr20	31680266/G//A	synonymous	0	2
PHF21B	chr22	45279020/C//T	synonymous	0	2
TUBGCP6	chr22	50658874/G//A	nonsynonymous	0	2
CTPS2	chrX	16608902/G//T	synonymous	0	2
ARX	chrX	25022894/G//A	nonsynonymous	0	2
WNK3	chrX	54328237/A//T	nonsynonymous	0	2
POU3F4	chrX	82764336/G//T	nonsynonymous	0	2
BHLHB9	chrX	102004391/G//A	synonymous	0	2
IRS4	chrX	107978988/C//T	nonsynonymous	0	2
ATP2B3	chrX	152821531/C//T	nonsynonymous	0	2
GALR1	chr18	74962466/C//T	synonymous	2	0
IRF2BP2	chr1	234744250/G//A	synonymous	1	1
MROH7	chr1	55144943/G//C	nonsynonymous	0	2
LRRIQ3	chr1	74507600/C//A	nonsynonymous	0	2
OLFM3	chr1	102270255/G//T	synonymous	0	2
OR10K1	chr1	158435933/C//T	synonymous	0	2
BRINP3	chr1	190067310/G//C	synonymous	0	2
OR2T33	chr1	248436532/G//A	synonymous	0	2
OR2T11	chr1	248789580/G//C	nonsynonymous	0	2
REV1	chr2	100024506/T//TA	nonsynonymous	0	2
ARPC4-TTLL3	chr3	9839339/A//G	synonymous	0	2
RASSF1	chr3	50369518/T//C	nonsynonymous	0	2
XRN1	chr3	142141670/T//C	synonymous	0	2
PPP3CA	chr4	102030182/C//T	nonsynonymous	0	2
IRX4	chr5	1880923/G//A	nonsynonymous	0	2
ADAMTS12	chr5	33576343/G//A	nonsynonymous	0	2
TNIP1	chr5	150436429/C//T	synonymous	0	2
SLC17A5	chr6	74304813/C//T	nonsynonymous	0	2
ARFGEF3	chr6	138655292/T//C	nonsynonymous	0	2
MIOS	chr7	7625309/A//T	nonsynonymous	0	2
EGFR	chr7	55266451/T//C	nonsynonymous	0	2
ZNF736	chr7	63808639/T//G	nonsynonymous	0	2
ACTR3B	chr7	152549283/G//T	nonsynonymous	0	2
KIAA1456	chr8	12879282/T//G	nonsynonymous	0	2
ADAM32	chr8	39103689/A//G	synonymous	0	2
OXR1	chr8	107726063/G//T	nonsynonymous	0	2
NUTM2G	chr9	99697649/G//C	nonsynonymous	0	2
AIP	chr11	67257620/C//CA	nonsynonymous	0	2
AMOTL1	chr11	94501548/C//T	synonymous	0	2
KMT2D	chr12	49438268/C//G	nonsynonymous	0	2

SACS	chr13	23913313/C//G	nonsynonymous	0	2
SCAPER	chr15	77092661/C//T	nonsynonymous	0	2
LONP2	chr16	48381552/C//T	synonymous	0	2
TP53	chr17	7578492/C//T	nonsynonymous	0	2
RARA	chr17	38511593/G//A	nonsynonymous	0	2
RGL3	chr19	11517152/C//G	synonymous	0	2
LCA5L	chr21	40777886/G//A	synonymous	0	2
DSCAM	chr21	41455850/C//A	nonsynonymous	0	2
DSCAM	chr21	41455853/C//A	nonsynonymous	0	2
MAP7D2	chrX	20071036/C//A	synonymous	0	2
MAOA	chrX	43587499/TTGTGGTATGTGAAGC	nonsynonymous	0	2
BCAP31	chrX	152988699/T//A	synonymous	0	2
ABCD1	chrX	152990727/G//A	synonymous	0	2
TIE1	chr1	43770628/C//T	synonymous	0	2
DNAJC6	chr1	65867445/A//G	synonymous	0	2
CLCA4	chr1	87041231/G//T	nonsynonymous	0	2
NECTIN4	chr1	161047448/G//C	synonymous	0	2
CHRM3	chr1	240071053/C//T	nonsynonymous	0	2
GREB1	chr2	11706619/C//T	synonymous	0	2
DHX57	chr2	39095433/C//T	nonsynonymous	0	2
CNTNAP5	chr2	125192161/G//T	nonsynonymous	0	2
FASTKD1	chr2	170411764/C//T	nonsynonymous	0	2
KLHL40	chr3	42727870/G//A	nonsynonymous	0	2
ITIH4	chr3	52861129/TCC//T	nonsynonymous	0	2
ABLIM2	chr4	8108336/C//T	synonymous	0	2
DCHS2	chr4	155219414/G//A	nonsynonymous	0	2
DNAH5	chr5	13737379/G//A	nonsynonymous	0	2
PCDHA6	chr5	140208144/C//T	synonymous	0	2
PCDHA10	chr5	140236992/G//A	synonymous	0	2
PCDHGA2	chr5	140718683/G//A	nonsynonymous	0	2
OR2Y1	chr5	180166600/G//A	synonymous	0	2
IP6K3	chr6	33694691/G//A	synonymous	0	2
MYB	chr6	135511445/G//A	nonsynonymous	0	2
SCAF8	chr6	155116245/A//T	nonsynonymous	0	2
TIAM2	chr6	155500543/A//G	nonsynonymous	0	2
RNASET2	chr6	167343147/C//T	nonsynonymous	0	2
TNRC18	chr7	5430246/C//A	synonymous	0	2
NPC1L1	chr7	44560710/C//T	synonymous	0	2
SLC26A4	chr7	107314635/G//A	nonsynonymous	0	2
PTPRZ1	chr7	121651353/G//A	synonymous	0	2
NUGGC	chr8	27888854/G//A	nonsynonymous	0	2
KCNB2	chr8	73480323/G//A	synonymous	0	2
KCNS2	chr8	99441272/C//T	synonymous	0	2
TOPORS-AS1	chr9	32552391/C//T	synonymous	0	2
OR2K2	chr9	114090031/G//T	nonsynonymous	0	2
AKR1C3	chr10	5136635/G//A	synonymous	0	2
HOGA1	chr10	99371385/G//A	nonsynonymous	0	2
NRAP	chr10	115365628/C//T	nonsynonymous	0	2
DOCK1	chr10	129207588/T//G	nonsynonymous	0	2
SLC17A6	chr11	22363157/C//T	nonsynonymous	0	2
BRCA2	chr13	32910728/G//T	nonsynonymous	0	2
TDRD3	chr13	61057927/A//G	nonsynonymous	0	2
ELMSAN1	chr14	74206008/G//A	nonsynonymous	0	2
ZNF609	chr15	64792307/C//T	nonsynonymous	0	2
SIN3A	chr15	75688801/GGATTGTTGCCA//G	nonsynonymous	0	2
NGRN	chr15	90814937/G//T	nonsynonymous	0	2
SALL1	chr16	51175457/C//T	nonsynonymous	0	2
ADAD2	chr16	84230315/C//T	nonsynonymous	0	2
ARID3A	chr19	932570/C//G	nonsynonymous	0	2
MUC16	chr19	9067143/G//A	nonsynonymous	0	2
HAMP	chr19	35775942/G//A	synonymous	0	2
CEACAM21	chr19	42085829/G//A	nonsynonymous	0	2
CDH4	chr20	60348080/G//A	nonsynonymous	0	2
XKR3	chr22	17265164/C//T	nonsynonymous	0	2
NHS	chrX	17750068/C//T	synonymous	0	2
POLA1	chrX	24833182/A//G	nonsynonymous	0	2
TSPYL2	chrX	53115441/G//A	nonsynonymous	0	2
ACTRT1	chrX	127186075/G//A	synonymous	0	2
ARHGAP4	chrX	153175255/C//T	nonsynonymous	0	2
RCC2	chr1	17742976/C//T	synonymous	0	2

WNT4	chr1	22446647/C//T	nonsynonymous	0	2
DAB1	chr1	57480729/G//A	nonsynonymous	0	2
TSPAN2	chr1	115615583/C//T	nonsynonymous	0	2
CHRM3	chr1	240071655/C//T	nonsynonymous	0	2
DDX1	chr2	15767213/TG//T	nonsynonymous	0	2
RAD51AP2	chr2	17696607/G//A	nonsynonymous	0	2
EHD3	chr2	31489172/C//T	nonsynonymous	0	2
LRP1B	chr2	141460055/T//C	nonsynonymous	0	2
SPATA3	chr2	231860926/C//T	synonymous	0	2
ARPC4-TTLL3	chr3	9854657/T//A	nonsynonymous	0	2
ZNF35	chr3	44700929/G//A	synonymous	0	2
CELSR3	chr3	48691065/G//A	nonsynonymous	0	2
USP19	chr3	49147889/G//A	nonsynonymous	0	2
ZBED2	chr3	111313039/G//A	nonsynonymous	0	2
DCHS2	chr4	155160409/G//A	synonymous	0	2
SLC1A3	chr5	36684074/G//A	synonymous	0	2
PLCXD3	chr5	41382421/C//T	nonsynonymous	0	2
APC	chr5	112175513/G//T	nonsynonymous	0	2
KCTD16	chr5	143853471/C//T	nonsynonymous	0	2
PEX6	chr6	42934337/G//A	nonsynonymous	0	2
IGF2BP3	chr7	23390960/G//A	nonsynonymous	0	2
PIK3CG	chr7	106508556/C//T	nonsynonymous	0	2
CLCN1	chr7	143013285/C//T	synonymous	0	2
RP1	chr8	55542638/G//A	nonsynonymous	0	2
PLPP7	chr9	134165701/G//A	nonsynonymous	0	2
ARMC4	chr10	28276429/G//A	nonsynonymous	0	2
OGDHL	chr10	50966551/G//A	nonsynonymous	0	2
SORCS3	chr10	106849589/C//T	nonsynonymous	0	2
VEGFB	chr11	64003379/ACAGCTGGTGCCCAG	nonsynonymous	0	2
TRMT112	chr11	64084783/T//TACGCGCC	nonsynonymous	0	2
TAS2R8	chr12	10959421/G//A	synonymous	0	2
GAS2L3	chr12	101017610/A//G	nonsynonymous	0	2
PXN	chr12	120651777/G//A	synonymous	0	2
DCLK1	chr13	36700265/C//T	nonsynonymous	0	2
SLITRK5	chr13	88329424/C//T	nonsynonymous	0	2
DOCK9	chr13	99461718/CT//C	nonsynonymous	0	2
FLRT2	chr14	86088894/C//T	nonsynonymous	0	2
PPP4R4	chr14	94696962/G//A	synonymous	0	2
ZNF106	chr15	42710039/G//A	synonymous	0	2
	chr15	74327359/G//A	synonymous	0	2
HBA1	chr16	227320/C//T	synonymous	0	2
BAIAP3	chr16	1391404/C//T	synonymous	0	2
TIMM22	chr17	900411/G//A	nonsynonymous	0	2
TP53	chr17	7577069/CG//C	nonsynonymous	0	2
PRKCA	chr17	64728922/G//T	nonsynonymous	0	2
ABCA8	chr17	66914238/C//T	nonsynonymous	0	2
CDR2L	chr17	73000280/C//T	synonymous	0	2
MGAT5B	chr17	74922813/G//A	nonsynonymous	0	2
SCGB2B2	chr19	35085245/G//A	synonymous	0	2
SPTBN4	chr19	41077993/C//T	nonsynonymous	0	2
TMEM86B	chr19	55738428/G//A	synonymous	0	2
NLRP8	chr19	56465927/C//T	nonsynonymous	0	2
PRAMEF4	chr1	12941754/A//G	nonsynonymous	0	2
PODN	chr1	53544159/C//T	nonsynonymous	0	2
SEC16B	chr1	177930821/G//C	nonsynonymous	0	2
LYST	chr1	235969126/G//A	nonsynonymous	0	2
RYR2	chr1	237670101/G//A	nonsynonymous	0	2
GCKR	chr2	27728609/C//T	nonsynonymous	0	2
DPP4	chr2	162873630/C//T	nonsynonymous	0	2
CCDC150	chr2	197597272/G//A	nonsynonymous	0	2
IGFBP2	chr2	217525370/G//A	nonsynonymous	0	2
DOCK10	chr2	225714255/T//A	nonsynonymous	0	2
SEPT2	chr2	242263652/C//A	nonsynonymous	0	2
RTP5	chr2	242815307/C//T	nonsynonymous	0	2
LZTFL1	chr3	45870094/G//A	nonsynonymous	0	2
ALCAM	chr3	105260478/G//A	nonsynonymous	0	2
PIK3CA	chr3	178916943/AAAG//A	nonsynonymous	0	2
PIK3CA	chr3	178916948/T//G	nonsynonymous	0	2
ANK2	chr4	114279509/C//T	synonymous	0	2
ADGRV1	chr5	89921058/G//A	nonsynonymous	0	2

APC	chr5	112173992/C//T	nonsynonymous	0	2
APC	chr5	112175675/A//AAG	nonsynonymous	0	2
JAKMIP2	chr5	147040637/G//A	synonymous	0	2
LARP1	chr5	154193795/A//G	synonymous	0	2
GRM6	chr5	178408783/C//T	nonsynonymous	0	2
MRNIP	chr5	179264799/C//T	synonymous	0	2
HLA-DMB	chr6	32904946/T//TA	nonsynonymous	0	2
HLA-DPA1	chr6	33037006/T//C	nonsynonymous	0	2
MUT	chr6	49403271/G//T	synonymous	0	2
FLNC	chr7	128490982/G//A	nonsynonymous	0	2
TNKS	chr8	9634257/C//G	synonymous	0	2
DOK2	chr8	21771059/C//T	synonymous	0	2
CA8	chr8	61192305/C//T	nonsynonymous	0	2
ZFHX4	chr8	77617228/G//A	nonsynonymous	0	2
FRMPD1	chr9	37744862/C//T	nonsynonymous	0	2
OMD	chr9	95177734/A//G	synonymous	0	2
EXD3	chr9	140218211/C//T	nonsynonymous	0	2
SFMBT2	chr10	7214536/C//T	nonsynonymous	0	2
PSD	chr10	104176766/C//T	synonymous	0	2
ELP4	chr11	31805046/G//A	nonsynonymous	0	2
TUBGCP3	chr13	113174192/C//T	synonymous	0	2
AHNAK2	chr14	105418127/C//T	nonsynonymous	0	2
AHNAK2	chr14	105418134/A//G	synonymous	0	2
AQP9	chr15	58467155/G//A	nonsynonymous	0	2
THSD4	chr15	72050263/G//C	nonsynonymous	0	2
GLYR1	chr16	4895119/G//A	synonymous	0	2
SLC13A2	chr17	26800818/C//T	nonsynonymous	0	2
JUP	chr17	39927915/C//T	synonymous	0	2
ZNF407	chr18	72347541/A//G	synonymous	0	2
STAP2	chr19	4328719/C//A	synonymous	0	2
MUC16	chr19	9074840/C//A	nonsynonymous	0	2
PODNL1	chr19	14044066/C//T	nonsynonymous	0	2
CYP4F2	chr19	16008282/C//T	nonsynonymous	0	2
RASGRP4	chr19	38903659/G//A	nonsynonymous	0	2
TRPM4	chr19	49686029/C//A	synonymous	0	2
CPXM1	chr20	2775076/C//T	synonymous	0	2
CDH4	chr20	60503249/G//T	synonymous	0	2
COL18A1	chr21	46876032/C//A	synonymous	0	2
COL6A2	chr21	47551899/C//T	synonymous	0	2
OTC	chrX	38240676/C//T	nonsynonymous	0	2
SLC38A5	chrX	48317972/G//A	nonsynonymous	0	2
WNK3	chrX	54275461/T//C	nonsynonymous	0	2
AMER1	chrX	63412110/G//A	nonsynonymous	0	2
TKTL1	chrX	153539304/C//T	synonymous	0	2
CD1B	chr1	158300741/C//A	nonsynonymous	0	2
LAMC1	chr1	183072705/G//T	nonsynonymous	0	2
NID1	chr1	236205338/C//T	nonsynonymous	0	2
MYT1L	chr2	1914021/C//T	nonsynonymous	0	2
FN1	chr2	216252994/C//T	nonsynonymous	0	2
TNP1	chr2	217724626/G//A	synonymous	0	2
VIPR1	chr3	42577836/G//A	synonymous	0	2
DCBLD2	chr3	98531213/C//T	nonsynonymous	0	2
KALRN	chr3	124053186/G//A	synonymous	0	2
FRYL	chr4	48597670/TC//T	nonsynonymous	0	2
FAT4	chr4	126328050/G//A	nonsynonymous	0	2
F2R	chr5	76028624/CTCA//C	nonsynonymous	0	2
PCDHGB5	chr5	140779518/C//T	synonymous	0	2
JARID2	chr6	15497178/C//A	synonymous	0	2
ZSCAN9	chr6	28200820/G//A	nonsynonymous	0	2
DPCR1	chr6	30916512/C//T	nonsynonymous	0	2
ANK1	chr8	41577302/G//A	synonymous	0	2
DENND4C	chr9	19346630/A//T	nonsynonymous	0	2
PAPPA	chr9	119129992/G//A	nonsynonymous	0	2
MKX	chr10	28024186/G//A	nonsynonymous	0	2
CDH23	chr10	73567093/C//T	synonymous	0	2
SORCS1	chr10	108337060/C//G	synonymous	0	2
CFAP46	chr10	134726304/G//A	nonsynonymous	0	2
CYP2R1	chr11	14913646/G//A	nonsynonymous	0	2
MRGPRX1	chr11	18955931/C//T	nonsynonymous	0	2
OR5AP2	chr11	56409905/A//G	nonsynonymous	0	2

LPCAT3	chr12	7092651/G//T	synonymous	0	2
AICDA	chr12	8758007/G//A	synonymous	0	2
RAB3IP	chr12	70150317/C//T	synonymous	0	2
TMEM119	chr12	108986148/C//T	synonymous	0	2
PDX1	chr13	28494180/G//A	synonymous	0	2
VWA8	chr13	42303699/C//A	nonsynonymous	0	2
PCDH9	chr13	67801376/C//A	synonymous	0	2
SCEL	chr13	78211272/G//T	nonsynonymous	0	2
PTPN21	chr14	88936292/T//G	nonsynonymous	0	2
CIITA	chr16	11016291/C//T	synonymous	0	2
SALL1	chr16	51171083/G//A	synonymous	0	2
TP53	chr17	7577569/A//C	nonsynonymous	0	2
BCAS3	chr17	59001836/G//A	synonymous	0	2
AXIN2	chr17	63554549/C//T	nonsynonymous	0	2
RNF213	chr17	78319853/C//T	nonsynonymous	0	2
ZNF532	chr18	56586308/G//A	synonymous	0	2
ZNF792	chr19	35449593/G//A	nonsynonymous	0	2
DMKN	chr19	36000827/C//T	nonsynonymous	0	2
CYP2A7	chr19	41384824/A//G	synonymous	0	2
TRPM4	chr19	49692259/C//T	nonsynonymous	0	2
ZIM2	chr19	57286602/G//A	synonymous	0	2
ZCCHC3	chr20	278500/G//T	synonymous	0	2
ATRN	chr20	3528079/C//T	nonsynonymous	0	2
NCOA6	chr20	33303070/T//G	synonymous	0	2
CECR2	chr22	18022564/G//A	nonsynonymous	0	2
DGCR8	chr22	20074043/A//C	nonsynonymous	0	2
TTC28	chr22	28378157/C//T	synonymous	0	2
SHROOM2	chrX	9863363/G//A	nonsynonymous	0	2
MBTPS2	chrX	21869659/G//T	synonymous	0	2
TAF1	chrX	70602614/A//G	nonsynonymous	0	2
	chr1	215179191/C//T	synonymous	0	2
ZFP36L2	chr2	43452649/G//A	synonymous	0	2
ADAM23	chr2	207457443/C//T	synonymous	0	2
MTMR14	chr3	9743627/A//G	synonymous	0	2
ZIC4	chr3	147108775/T//C	nonsynonymous	0	2
FAT4	chr4	126373546/G//A	nonsynonymous	0	2
APC	chr5	112175675/AAG//A	nonsynonymous	0	2
ZNF862	chr7	149558454/C//T	synonymous	0	2
RBM33	chr7	155538160/G//A	nonsynonymous	0	2
LOXL2	chr8	23225607/G//A	synonymous	0	2
TAF1L	chr9	32634655/T//G	nonsynonymous	0	2
ABCA1	chr9	107583748/G//A	synonymous	0	2
C1RL	chr12	7261801/C//A	synonymous	0	2
ZKSCAN2	chr16	25251416/G//A	synonymous	0	2
ARHGAP23	chr17	36622816/C//T	nonsynonymous	0	2
KCNH4	chr17	40318419/G//A	nonsynonymous	0	2
RGS9	chr17	63200383/C//T	synonymous	0	2
CACNG5	chr17	64880644/G//A	nonsynonymous	0	2
DNAH17	chr17	76455195/C//T	nonsynonymous	0	2
ZNF99	chr19	22940177/G//T	nonsynonymous	0	2
KRTAP10-10	chr21	46057676/C//A	nonsynonymous	0	2
MAGEB2	chrX	30237623/A//C	nonsynonymous	0	2
RP1	chr8	55534012/C//T	synonymous	1	1
TMEM201	chr1	9655960/G//A	synonymous	0	2
SPTA1	chr1	158615044/C//G	nonsynonymous	0	2
TNR	chr1	175304930/C//T	nonsynonymous	0	2
CEP170	chr1	243289612/A//C	synonymous	0	2
C1orf100	chr1	244538768/A//T	nonsynonymous	0	2
ZNF385B	chr2	180383268/C//T	nonsynonymous	0	2
MARCH4	chr2	217234856/C//T	nonsynonymous	0	2
ARPC2	chr2	219118734/T//C	synonymous	0	2
OBSL1	chr2	220432668/G//A	nonsynonymous	0	2
TOPAZ1	chr3	44286486/C//T	nonsynonymous	0	2
CACNA2D2	chr3	50417431/C//T	synonymous	0	2
TRIM59	chr3	160156557/A//T	nonsynonymous	0	2
CCKAR	chr4	26490886/G//A	synonymous	0	2
ADAMTS3	chr4	73181644/A//G	synonymous	0	2
SPATA5	chr4	124235157/C//A	nonsynonymous	0	2
KIAA0825	chr5	93732217/G//T	synonymous	0	2
APC	chr5	112128209/C//T	nonsynonymous	0	2

FBN2	chr5	127873312/C//T	synonymous	0	2
PCDHA6	chr5	140207808/C//T	synonymous	0	2
GLRA1	chr5	151202502/C//T	nonsynonymous	0	2
ZBED9	chr6	28554306/T//C	synonymous	0	2
GLTSCR1L	chr6	42832599/C//T	synonymous	0	2
SYNE1	chr6	152469354/C//T	nonsynonymous	0	2
PDE10A	chr6	165848745/G//T	nonsynonymous	0	2
VSTM2A	chr7	54612486/C//T	synonymous	0	2
ARF5	chr7	127230160/T//A	nonsynonymous	0	2
EPHB6	chr7	142561718/C//A	synonymous	0	2
SSPO	chr7	149512311/G//A	nonsynonymous	0	2
RHOBTB2	chr8	22864364/C//T	synonymous	0	2
ZFHX4	chr8	77767937/C//T	nonsynonymous	0	2
MSANTD3-TMEFF1	chr9	103338824/G//A	nonsynonymous	0	2
ACTL7B	chr9	111617947/G//A	synonymous	0	2
CERCAM	chr9	131186866/G//A	nonsynonymous	0	2
SFMBT2	chr10	7214470/G//A	nonsynonymous	0	2
SLF2	chr10	102709849/G//A	nonsynonymous	0	2
TCF7L2	chr10	114905777/C//T	nonsynonymous	0	2
TRIM66	chr11	8643339/G//A	synonymous	0	2
SPON1	chr11	14156968/C//T	nonsynonymous	0	2
ALX4	chr11	44289129/C//T	nonsynonymous	0	2
FMNL3	chr12	50100886/C//T	nonsynonymous	0	2
PTPRR	chr12	71286509/C//G	nonsynonymous	0	2
PTPRQ	chr12	80878331/G//T	nonsynonymous	0	2
PITPNM2	chr12	123489914/G//A	synonymous	0	2
B3GLCT	chr13	31858802/A//G	nonsynonymous	0	2
DCLK1	chr13	36445444/T//G	nonsynonymous	0	2
SMAD9	chr13	37441502/G//A	nonsynonymous	0	2
FAM124A	chr13	51825615/G//A	nonsynonymous	0	2
TMEM121	chr14	105995821/A//G	nonsynonymous	0	2
	chr15	60907163/A//T	synonymous	0	2
NUBP2	chr16	1838575/G//A	nonsynonymous	0	2
ABCA3	chr16	2327601/C//T	synonymous	0	2
MEFV	chr16	3306490/A//G	nonsynonymous	0	2
ERCC4	chr16	14014102/T//G	nonsynonymous	0	2
PDILT	chr16	20380898/G//A	synonymous	0	2
ZNF646	chr16	31090868/C//G	nonsynonymous	0	2
DHODH	chr16	72046000/G//A	nonsynonymous	0	2
PLCG2	chr16	81939006/A//G	nonsynonymous	0	2
TP53	chr17	7579311/C//T	nonsynonymous	0	2
CDK12	chr17	37649018/G//A	nonsynonymous	0	2
TBKBP1	chr17	45776807/C//T	synonymous	0	2
APCDD1	chr18	10485553/G//A	nonsynonymous	0	2
ANKRD30B	chr18	14754979/G//T	nonsynonymous	0	2
SMAD4	chr18	48604665/G//A	nonsynonymous	0	2
MKNK2	chr19	2041097/C//T	nonsynonymous	0	2
EEF2	chr19	3977881/C//T	nonsynonymous	0	2
PNPLA6	chr19	7600405/T//A	synonymous	0	2
FCGBP	chr19	40434049/C//A	nonsynonymous	0	2
C5AR1	chr19	47823623/C//T	nonsynonymous	0	2
PCSK2	chr20	17462728/G//A	synonymous	0	2
KCNS1	chr20	43727125/G//A	synonymous	0	2
	chr20	44036908/A//T	synonymous	0	2
HELZ2	chr20	62196629/C//T	synonymous	0	2
BCOR	chrX	39933535/G//A	nonsynonymous	0	2
FUNDC1	chrX	44397786/C//T	nonsynonymous	0	2
BMP15	chrX	50654046/G//T	nonsynonymous	0	2
EFNB1	chrX	68058522/G//C	nonsynonymous	0	2
BEX4	chrX	102471104/C//T	nonsynonymous	0	2
FAM122C	chrX	133988149/G//A	synonymous	0	2
INTS6L	chrX	134679422/C//A	synonymous	0	2
BRS3	chrX	135572359/G//T	nonsynonymous	0	2
HSD3B1	chr1	120056807/G//T	nonsynonymous	0	2
DCST2	chr1	155003693/C//T	nonsynonymous	0	2
TNR	chr1	175304903/C//T	nonsynonymous	0	2
PAPPA2	chr1	176564216/G//A	synonymous	0	2
KIAA1614	chr1	180885999/C//A	nonsynonymous	0	2
ASPM	chr1	197108983/G//T	nonsynonymous	0	2
RRP15	chr1	218478377/A//C	nonsynonymous	0	2

RAD51AP2	chr2	17698997/G//A	nonsynonymous	0	2
WDR33	chr2	128463863/T//C	synonymous	0	2
ITGB6	chr2	160982890/C//T	nonsynonymous	0	2
MTTP	chr4	100543904/G//A	nonsynonymous	0	2
PCDH10	chr4	134072359/C//T	nonsynonymous	0	2
MARCH1	chr4	164775228/GTGT//G	nonsynonymous	0	2
CFAP97	chr4	186111386/T//C	nonsynonymous	0	2
F2R	chr5	76028964/G//T	nonsynonymous	0	2
PRR7	chr5	176881943/C//T	synonymous	0	2
HIVEP1	chr6	12125319/G//A	nonsynonymous	0	2
VARS	chr6	31750907/G//A	synonymous	0	2
WDR46	chr6	33248649/G//A	nonsynonymous	0	2
ZBTB9	chr6	33424049/G//A	nonsynonymous	0	2
AKAP12	chr6	151672878/GAAAGCTTTGAA//G	nonsynonymous	0	2
SDK1	chr7	4247868/C//T	synonymous	0	2
TBX20	chr7	35289718/C//T	synonymous	0	2
COPG2	chr7	130298471/G//A	nonsynonymous	0	2
OR6V1	chr7	142749857/C//A	synonymous	0	2
KCNH2	chr7	150655559/G//A	synonymous	0	2
FAM92A	chr8	94722048/G//A	nonsynonymous	0	2
KCNQ3	chr8	133182600/C//T	nonsynonymous	0	2
MAF1	chr8	145161524/C//G	nonsynonymous	0	2
MAF1	chr8	145162048/C//A	synonymous	0	2
KANK1	chr9	711574/G//A	nonsynonymous	0	2
C9orf57	chr9	74675503/G//A	synonymous	0	2
COL5A1	chr9	137716468/A//T	nonsynonymous	0	2
FCN1	chr9	137801580/C//T	synonymous	0	2
CH17-360D5.1	chr10	47086894/C//T	synonymous	0	2
RTKN2	chr10	63958157/T//G	nonsynonymous	0	2
CCDC73	chr11	32739646/C//T	synonymous	0	2
OR5A2	chr11	59189643/G//A	nonsynonymous	0	2
MMP13	chr11	102826158/C//A	nonsynonymous	0	2
FXYD6-FXYD2	chr11	117713494/C//T	synonymous	0	2
MCAM	chr11	119182271/C//T	nonsynonymous	0	2
ARID2	chr12	46243962/C//T	nonsynonymous	0	2
RBMS2	chr12	56962768/G//T	nonsynonymous	0	2
MVK	chr12	110029088/A//G	nonsynonymous	0	2
NBEA	chr13	35806753/G//T	nonsynonymous	0	2
	chr13	36424850/G//T	synonymous	0	2
CARMIL3	chr14	24523913/T//C	synonymous	0	2
	chr14	59003744/G//C	synonymous	0	2
CATSPERB	chr14	92171041/C//T	synonymous	0	2
CLCN7	chr16	1499026/C//T	nonsynonymous	0	2
SLC5A11	chr16	24886984/G//A	nonsynonymous	0	2
ORC6	chr16	46731661/G//A	synonymous	0	2
DNAH2	chr17	7736020/T//A	nonsynonymous	0	2
NCOR1	chr17	16042434/G//A	nonsynonymous	0	2
KLHL11	chr17	40021672/T//G	synonymous	0	2
PLEKHM1	chr17	43531376/C//T	synonymous	0	2
TNRC6C	chr17	76045127/G//C	synonymous	0	2
CCDC40	chr17	78055379/G//A	nonsynonymous	0	2
DUS1L	chr17	80015934/T//G	synonymous	0	2
SS18	chr18	23619415/T//C	nonsynonymous	0	2
ELP2	chr18	33709951/C//T	nonsynonymous	0	2
CASP14	chr19	15166913/C//A	synonymous	0	2
CCDC97	chr19	41822462/C//T	nonsynonymous	0	2
IRGC	chr19	44222974/C//T	synonymous	0	2
A1BG	chr19	58861785/C//T	synonymous	0	2
SNTA1	chr20	32000520/G//A	nonsynonymous	0	2
LAMA5	chr20	60909319/C//T	nonsynonymous	0	2
MAGEB18	chrX	26157931/G//A	nonsynonymous	0	2
TBX22	chrX	79281262/G//T	nonsynonymous	0	2
BRWD3	chrX	79955473/T//C	nonsynonymous	0	2
PLEKHN1	chr1	908308/C//T	nonsynonymous	0	2
KCND3	chr1	112524471/C//T	nonsynonymous	0	2
RPRD2	chr1	150443408/G//A	nonsynonymous	0	2
ADAMTSL4	chr1	150529979/G//A	nonsynonymous	0	2
LRRC52	chr1	165532892/C//T	nonsynonymous	0	2
SPATA17	chr1	217955580/G//T	nonsynonymous	0	2
SNTG2	chr2	1161247/G//A	nonsynonymous	0	2

C2orf16	chr2	27802456/G//A	nonsynonymous	0	2
IMMT	chr2	86389145/G//A	nonsynonymous	0	2
MAL	chr2	95715410/G//A	nonsynonymous	0	2
LRP1B	chr2	141201978/C//T	synonymous	0	2
XIRP2	chr2	168102520/A//C	nonsynonymous	0	2
TTN	chr2	179640232/C//T	nonsynonymous	0	2
MAP2	chr2	210545499/T//A	synonymous	0	2
DOCK3	chr3	51395491/C//T	nonsynonymous	0	2
FBXO40	chr3	121340468/G//A	synonymous	0	2
NPHP3	chr3	132405120/G//A	nonsynonymous	0	2
MME	chr3	154832866/C//T	nonsynonymous	0	2
SKIL	chr3	170078733/T//A	nonsynonymous	0	2
EVC2	chr4	5624508/G//A	nonsynonymous	0	2
ADGRL3	chr4	62936266/C//A	synonymous	0	2
MAPK10	chr4	87028508/G//T	synonymous	0	2
GRIA2	chr4	158284166/C//T	synonymous	0	2
CYP4V2	chr4	187130015/TCAGGG//T	nonsynonymous	0	2
DROSHA	chr5	31483660/C//A	nonsynonymous	0	2
THBS4	chr5	79363850/G//A	nonsynonymous	0	2
ACOT12	chr5	80681619/C//T	nonsynonymous	0	2
MEGF10	chr5	126676292/G//A	nonsynonymous	0	2
BRD8	chr5	137495853/C//T	nonsynonymous	0	2
LARP1	chr5	154193792/G//T	synonymous	0	2
GRK6	chr5	176867760/C//T	synonymous	0	2
DST	chr6	56499286/G//T	nonsynonymous	0	2
HIVEP2	chr6	143074721/G//A	synonymous	0	2
PAPOLB	chr7	4901458/C//T	synonymous	0	2
ZNF680	chr7	63982102/G//T	nonsynonymous	0	2
COL1A2	chr7	94057787/C//T	nonsynonymous	0	2
RP1	chr8	55540003/G//T	synonymous	0	2
CYP7A1	chr8	59409732/G//A	synonymous	0	2
TRPA1	chr8	72966070/G//A	nonsynonymous	0	2
RIC1	chr9	5763741/C//A	nonsynonymous	0	2
TEK	chr9	27190654/C//T	synonymous	0	2
IKBKAP	chr9	111653498/C//T	nonsynonymous	0	2
PBX3	chr9	128722969/A//G	nonsynonymous	0	2
PPP1R26	chr9	138379556/C//T	nonsynonymous	0	2
CSGALNACT2	chr10	43678784/C//T	nonsynonymous	0	2
C10orf71	chr10	50534395/G//A	nonsynonymous	0	2
OR51I1	chr11	5462039/G//T	synonymous	0	2
OR4C45	chr11	48367133/C//T	nonsynonymous	0	2
OR4D11	chr11	59271520/G//A	nonsynonymous	0	2
CEP164	chr11	117265117/C//T	nonsynonymous	0	2
FGF23	chr12	4479778/C//A	nonsynonymous	0	2
GPD1	chr12	50498531/TGTG//T	nonsynonymous	0	2
STAB2	chr12	104156142/C//T	nonsynonymous	0	2
TBX3	chr12	115112282/C//A	synonymous	0	2
ZC3H13	chr13	46541939/G//A	nonsynonymous	0	2
UGGT2	chr13	96508528/G//A	nonsynonymous	0	2
HS6ST3	chr13	97484922/C//T	nonsynonymous	0	2
FANCM	chr14	45665742/A//G	nonsynonymous	0	2
PAPLN	chr14	73719483/G//A	nonsynonymous	0	2
TSHR	chr14	81610843/T//C	synonymous	0	2
UNC13C	chr15	54847643/G//T	nonsynonymous	0	2
SRRM2	chr16	2816561/C//T	nonsynonymous	0	2
ZDHHC1	chr16	67428979/G//A	nonsynonymous	0	2
TP53	chr17	7577609/C//A	nonsynonymous	0	2
DNAH2	chr17	7661952/C//T	nonsynonymous	0	2
DNAH2	chr17	7662780/G//A	nonsynonymous	0	2
MFSD6L	chr17	8701083/C//A	nonsynonymous	0	2
B4GALNT2	chr17	47230234/G//T	synonymous	0	2
HSF5	chr17	56557283/G//T	nonsynonymous	0	2
DNAI2	chr17	72278019/G//A	synonymous	0	2
DNAH17	chr17	76556973/T//C	nonsynonymous	0	2
CCDC102B	chr18	66721381/C//A	synonymous	0	2
RHPN2	chr19	33535174/C//T	nonsynonymous	0	2
SIPA1L3	chr19	38643527/G//T	nonsynonymous	0	2
IFNL1	chr19	39788648/G//A	synonymous	0	2
HNRNPUL1	chr19	41770664/C//T	nonsynonymous	0	2
PRR19	chr19	42814069/C//A	synonymous	0	2

KLK14	chr19	51581261/C//G	synonymous	0	2
RALGAPB	chr20	37146584/A//T	nonsynonymous	0	2
TFAP2C	chr20	55208518/G//A	synonymous	0	2
DIDO1	chr20	61513648/C//T	synonymous	0	2
ADAMTS5	chr21	28337976/C//T	synonymous	0	2
TANGO2	chr22	20049139/C//T	nonsynonymous	0	2
TTC28	chr22	28378457/G//A	nonsynonymous	0	2
CSF2RB	chr22	37334323/C//T	nonsynonymous	0	2
FBLN1	chr22	45943038/C//T	synonymous	0	2
FRMPD4	chrX	12735911/T//C	nonsynonymous	0	2
KIAA1210	chrX	118222394/G//T	synonymous	0	2
DCAF12L1	chrX	125686452/G//A	nonsynonymous	0	2
PADI1	chr1	17563856/C//G	nonsynonymous	0	2
NRXN1	chr2	51255159/G//A	nonsynonymous	0	2
OBSL1	chr2	220431600/C//T	nonsynonymous	0	2
DAW1	chr2	228736411/G//A	synonymous	0	2
IMPDH2	chr3	49065958/G//A	nonsynonymous	0	2
KDR	chr4	55948806/C//A	synonymous	0	2
TLR3	chr4	187004649/A//G	synonymous	0	2
NIM1K	chr5	43277343/C//T	synonymous	0	2
APC	chr5	112175525/G//T	nonsynonymous	0	2
AHI1	chr6	135749776/G//A	nonsynonymous	0	2
POLD2	chr7	44154493/G//A	nonsynonymous	0	2
SMO	chr7	128848687/G//A	nonsynonymous	0	2
EBF2	chr8	25766064/A//T	nonsynonymous	0	2
OSR2	chr8	99961843/C//T	synonymous	0	2
TG	chr8	133885337/G//A	nonsynonymous	0	2
COL22A1	chr8	139890150/G//A	synonymous	0	2
SPATA31E1	chr9	90502636/G//A	synonymous	0	2
OR1N1	chr9	125288840/C//T	nonsynonymous	0	2
NOTCH1	chr9	139407553/G//A	nonsynonymous	0	2
LRRTM3	chr10	68857603/G//A	synonymous	0	2
GRID1	chr10	87966238/C//T	nonsynonymous	0	2
PDE3B	chr11	14666067/G//A	nonsynonymous	0	2
KMT2A	chr11	118373478/A//G	nonsynonymous	0	2
ASUN	chr12	27064156/G//T	nonsynonymous	0	2
ARPC3	chr12	110873938/G//T	nonsynonymous	0	2
TUBA3C	chr13	19753683/G//A	synonymous	0	2
GPHB5	chr14	63779783/A//G	synonymous	0	2
EVL	chr14	100608082/G//C	nonsynonymous	0	2
SMAD6	chr15	67073514/G//A	nonsynonymous	0	2
POLG	chr15	89876408/C//T	nonsynonymous	0	2
NLRC3	chr16	3613509/C//T	nonsynonymous	0	2
ITPRIPL2	chr16	19127324/G//A	nonsynonymous	0	2
IRX6	chr16	55362612/A//C	nonsynonymous	0	2
COG4	chr16	70557324/T//C	synonymous	0	2
FOXF1	chr16	86544853/C//A	synonymous	0	2
PIK3R5	chr17	8792007/G//A	nonsynonymous	0	2
KSR1	chr17	25910106/C//T	nonsynonymous	0	2
ANKFN1	chr17	54526456/C//T	synonymous	0	2
SERPINB12	chr18	61228356/G//A	synonymous	0	2
NFATC1	chr18	77171334/C//T	synonymous	0	2
LPAR2	chr19	19738008/C//T	nonsynonymous	0	2
TSKS	chr19	50251422/C//T	nonsynonymous	0	2
ZNF814	chr19	58385448/C//T	nonsynonymous	0	2
ZNF337	chr20	25656283/C//T	synonymous	0	2
DLGAP4	chr20	35060532/C//T	nonsynonymous	0	2
PTPRT	chr20	40747036/G//C	synonymous	0	2
WWC3	chrX	10102561/G//A	synonymous	0	2
MAGEB6	chrX	26212383/T//A	nonsynonymous	0	2
WDR13	chrX	48457313/C//T	nonsynonymous	0	2
PCSK1N	chrX	48689731/C//T	nonsynonymous	0	2
SLC7A3	chrX	70149560/C//T	synonymous	0	2
MAGEC3	chrX	140985546/C//T	synonymous	0	2
SPANXN1	chrX	144337246/A//G	nonsynonymous	0	2
MUC6	chr11	1018169/C//G	synonymous	1	1
ZNF540	chr19	38090521/T//C	synonymous	1	1
CDC27	chr17	45234713/T//G	synonymous	0	2
SAMD11	chr1	879438/G//A	nonsynonymous	0	2
C1orf158	chr1	12820783/T//G	nonsynonymous	0	2

BEST4	chr1	45250796/TAGA//T	nonsynonymous	0	2
WLS	chr1	68611546/G//T	synonymous	0	2
CGN	chr1	151491544/C//T	synonymous	0	2
ADCY10	chr1	167792340/C//A	synonymous	0	2
MRPS14	chr1	174992588/T//A	synonymous	0	2
LAMB3	chr1	209807883/G//T	nonsynonymous	0	2
TRAF3IP3	chr1	209948949/C//T	synonymous	0	2
OBSCN	chr1	228481947/G//A	synonymous	0	2
SLC35F3	chr1	234367172/G//A	nonsynonymous	0	2
NID1	chr1	236192973/C//T	nonsynonymous	0	2
RIF1	chr2	152320972/A//G	synonymous	0	2
CYTIP	chr2	158272481/G//A	nonsynonymous	0	2
ATF2	chr2	175962237/T//A	nonsynonymous	0	2
CCDC141	chr2	179742706/A//T	synonymous	0	2
CHPF	chr2	220404662/C//T	nonsynonymous	0	2
ALPP	chr2	233246432/G//T	nonsynonymous	0	2
SAG	chr2	234229317/G//A	nonsynonymous	0	2
PPP1R7	chr2	242097242/G//T	nonsynonymous	0	2
CDCP1	chr3	45152273/T//TTAAAC	nonsynonymous	0	2
CACNA1D	chr3	53757544/G//A	nonsynonymous	0	2
BOC	chr3	112997066/C//T	nonsynonymous	0	2
CASR	chr3	122003306/C//T	synonymous	0	2
TFRC	chr3	195782058/C//T	nonsynonymous	0	2
	chr4	5894638/C//T	synonymous	0	2
PDGFRA	chr4	55161324/C//T	nonsynonymous	0	2
ABCG2	chr4	89052252/G//T	synonymous	0	2
GRID2	chr4	94032033/C//T	nonsynonymous	0	2
ANKRD50	chr4	125592031/A//G	synonymous	0	2
FREM3	chr4	144620940/G//A	nonsynonymous	0	2
FBXW7	chr4	153247204/C//A	nonsynonymous	0	2
FSTL5	chr4	162376242/C//G	synonymous	0	2
TRAPPC11	chr4	184628011/T//A	nonsynonymous	0	2
ADCY2	chr5	7727287/C//T	nonsynonymous	0	2
NIM1K	chr5	43246015/G//A	synonymous	0	2
ANKRD31	chr5	74400601/C//G	nonsynonymous	0	2
APC	chr5	112176964/G//A	synonymous	0	2
PCDHGB6	chr5	140789134/G//A	synonymous	0	2
ADAMTS2	chr5	178555035/C//T	nonsynonymous	0	2
DNAH8	chr6	38840417/G//A	nonsynonymous	0	2
PTCHD4	chr6	47846408/G//A	synonymous	0	2
FNDC1	chr6	159653533/C//T	synonymous	0	2
SDK1	chr7	4169715/G//A	nonsynonymous	0	2
ZNF853	chr7	6662431/C//T	synonymous	0	2
GTF2IRD1	chr7	73973268/G//A	nonsynonymous	0	2
GAL3ST4	chr7	99757738/C//T	nonsynonymous	0	2
PCOLCE	chr7	100201623/C//A	nonsynonymous	0	2
NOS3	chr7	150698351/C//T	synonymous	0	2
DPP6	chr7	154667804/C//T	nonsynonymous	0	2
PTPRN2	chr7	158282442/C//T	nonsynonymous	0	2
CSMD1	chr8	3072103/T//G	nonsynonymous	0	2
SPIDR	chr8	48639798/C//A	nonsynonymous	0	2
VCPIP1	chr8	67577110/G//A	nonsynonymous	0	2
SBSPON	chr8	73993446/G//A	nonsynonymous	0	2
FBXO10	chr9	37516025/C//T	nonsynonymous	0	2
TJP2	chr9	71852871/G//C	nonsynonymous	0	2
FANCC	chr9	97897640/T//C	synonymous	0	2
ZNF462	chr9	109692894/C//T	nonsynonymous	0	2
NUP214	chr9	134070655/C//T	nonsynonymous	0	2
ADAMTS13	chr9	136297766/C//T	nonsynonymous	0	2
MAP3K8	chr10	30736779/T//G	synonymous	0	2
PARD3	chr10	34558680/G//A	synonymous	0	2
C10orf71	chr10	50534222/C//T	nonsynonymous	0	2
P4HA1	chr10	74828736/T//A	nonsynonymous	0	2
BMPR1A	chr10	88672027/T//G	synonymous	0	2
EXOC6	chr10	94675507/C//A	synonymous	0	2
SFRP5	chr10	99527528/C//T	nonsynonymous	0	2
OR52L1	chr11	6007488/T//C	nonsynonymous	0	2
DGKZ	chr11	46394565/G//A	nonsynonymous	0	2
OR5D13	chr11	55541545/G//A	nonsynonymous	0	2
FIBP	chr11	65653098/G//A	synonymous	0	2

FGF19	chr11	69514314/C//T	nonsynonymous	0	2
ANO1	chr11	69962565/G//A	nonsynonymous	0	2
POU2F3	chr11	120176347/G//A	synonymous	0	2
TECTA	chr11	120983847/G//A	nonsynonymous	0	2
ST3GAL4	chr11	126278027/C//T	synonymous	0	2
ADAMTS8	chr11	130289058/C//T	nonsynonymous	0	2
CLEC4A	chr12	8288182/G//T	nonsynonymous	0	2
PZP	chr12	9346733/C//T	synonymous	0	2
C2CD5	chr12	22697103/T//C	synonymous	0	2
ARL1	chr12	101801517/G//T	synonymous	0	2
RPH3A	chr12	113327879/G//A	synonymous	0	2
RNFT2	chr12	117287160/T//C	synonymous	0	2
TRPC4	chr13	38320332/G//A	synonymous	0	2
CYSLTR2	chr13	49281668/C//T	nonsynonymous	0	2
SCEL	chr13	78146321/G//T	nonsynonymous	0	2
SLITRK1	chr13	84453819/G//A	synonymous	0	2
MYO16	chr13	109550367/G//A	nonsynonymous	0	2
LTB4R2	chr14	24780795/G//A	nonsynonymous	0	2
GEMIN2	chr14	39594200/G//T	nonsynonymous	0	2
SYNE2	chr14	64444652/G//A	synonymous	0	2
UNC79	chr14	94139775/G//A	nonsynonymous	0	2
SLC24A5	chr15	48414111/C//T	nonsynonymous	0	2
CYP1A2	chr15	75042757/C//T	synonymous	0	2
BNC1	chr15	83933220/G//A	synonymous	0	2
ACAN	chr15	89400777/C//A	nonsynonymous	0	2
ZNF598	chr16	2052252/C//T	nonsynonymous	0	2
PKD1L2	chr16	81190533/G//A	synonymous	0	2
ADAD2	chr16	84229828/G//A	nonsynonymous	0	2
MYOCD	chr17	12666404/G//A	nonsynonymous	0	2
AP2B1	chr17	33935314/G//A	nonsynonymous	0	2
MAPT	chr17	44060900/C//T	nonsynonymous	0	2
DNAH17	chr17	76533475/C//T	nonsynonymous	0	2
MTCL1	chr18	8813127/G//A	nonsynonymous	0	2
MIB1	chr18	19359498/A//G	nonsynonymous	0	2
CELF4	chr18	34854357/G//A	nonsynonymous	0	2
SERPINB7	chr18	61468118/G//T	nonsynonymous	0	2
BSG	chr19	581598/C//T	synonymous	0	2
SAFB	chr19	5664112/C//T	nonsynonymous	0	2
KRI1	chr19	10670606/G//A	synonymous	0	2
MAP1S	chr19	17837565/G//A	nonsynonymous	0	2
ZNF91	chr19	23545427/C//T	synonymous	0	2
CLIP3	chr19	36517504/C//T	synonymous	0	2
TIMM50	chr19	39980373/C//T	synonymous	0	2
MBOAT7	chr19	54692357/C//A	synonymous	0	2
XKR7	chr20	30584778/G//A	nonsynonymous	0	2
PHF20	chr20	34389505/G//A	nonsynonymous	0	2
TPD52L2	chr20	62521257/G//A	nonsynonymous	0	2
LKAAEAR1	chr20	62715013/C//T	nonsynonymous	0	2
MYT1	chr20	62836442/G//A	synonymous	0	2
USP25	chr21	17250799/C//T	synonymous	0	2
GABPA	chr21	27124343/G//A	synonymous	0	2
MICAL3	chr22	18300441/G//A	synonymous	0	2
MED15	chr22	20937518/G//A	synonymous	0	2
HORMAD2	chr22	30514974/G//T	synonymous	0	2
EP300	chr22	41542766/A//G	nonsynonymous	0	2
CASK	chrX	41379671/G//A	synonymous	0	2
MAGEC1	chrX	140995937/C//T	nonsynonymous	0	2
PDZD4	chrX	153069675/C//T	synonymous	0	2
GDI1	chrX	153670996/G//A	nonsynonymous	0	2
ELAVL4	chr1	50661335/C//T	nonsynonymous	0	2
NTNG1	chr1	107937929/C//A	synonymous	0	2
USH2A	chr1	216019297/A//G	nonsynonymous	0	2
RYR2	chr1	237754065/G//A	synonymous	0	2
PSMD2	chr3	184019363/C//A	synonymous	0	2
MUC4	chr3	195516765/G//A	synonymous	0	2
IL21	chr4	123542048/C//T	nonsynonymous	0	2
DNAH5	chr5	13817688/C//T	synonymous	0	2
IQGAP2	chr5	75996995/G//C	nonsynonymous	0	2
GPX3	chr5	150406496/G//A	nonsynonymous	0	2
TCP11	chr6	35103896/A//C	nonsynonymous	0	2

TRDN	chr6	123588882/G//C	nonsynonymous	0	2
CHST12	chr7	2472574/C//T	synonymous	0	2
HDAC9	chr7	18669028/G//C	nonsynonymous	0	2
GRM3	chr7	86415654/G//A	synonymous	0	2
ACHE	chr7	100491069/G//T	nonsynonymous	0	2
FIS1	chr7	100887397/A//G	synonymous	0	2
TBXAS1	chr7	139653218/C//T	nonsynonymous	0	2
SLC20A2	chr8	42302276/G//A	synonymous	0	2
PXDNL	chr8	52320916/T//A	nonsynonymous	0	2
SLCO5A1	chr8	70674113/G//A	synonymous	0	2
TMEM67	chr8	94794688/T//C	synonymous	0	2
TMOD1	chr9	100328214/C//T	nonsynonymous	0	2
GRIN3A	chr9	104433256/G//A	nonsynonymous	0	2
ASB6	chr9	132400631/A//G	nonsynonymous	0	2
RPL7A	chr9	136218179/C//T	synonymous	0	2
CACNA1B	chr9	141016110/C//T	nonsynonymous	0	2
UCMA	chr10	13276307/G//A	synonymous	0	2
HNRNPH3	chr10	70097718/T//C	synonymous	0	2
MAPK8IP1	chr11	45926798/G//A	synonymous	0	2
OR4C16	chr11	55339950/C//T	nonsynonymous	0	2
OR5T3	chr11	56020212/C//A	nonsynonymous	0	2
DRAP1	chr11	65686838/C//T	synonymous	0	2
SHANK2	chr11	70331532/G//A	synonymous	0	2
DGAT2	chr11	75509332/C//T	synonymous	0	2
ATM	chr11	108100022/C//G	synonymous	0	2
C11orf87	chr11	109294937/C//T	nonsynonymous	0	2
TRPV4	chr12	110230452/C//T	synonymous	0	2
ZNF10	chr12	133732200/A//G	nonsynonymous	0	2
FLT3	chr13	28644740/G//T	nonsynonymous	0	2
FREM2	chr13	39433471/T//G	nonsynonymous	0	2
RYR3	chr15	33928584/G//A	nonsynonymous	0	2
FAM227B	chr15	49833942/G//A	nonsynonymous	0	2
TNFAIP8L3	chr15	51350185/C//T	nonsynonymous	0	2
SLX4	chr16	3639704/C//G	nonsynonymous	0	2
SLX4	chr16	3639707/A//G	nonsynonymous	0	2
DPEP2	chr16	68024026/G//A	synonymous	0	2
TBC1D28	chr17	18542007/C//T	nonsynonymous	0	2
TEPSIN	chr17	79212886/A//G	synonymous	0	2
DPP9-AS1	chr19	4683327/C//T	nonsynonymous	0	2
SLC25A41	chr19	6430156/G//A	nonsynonymous	0	2
GZF1	chr20	23346053/G//A	nonsynonymous	0	2
RRP1B	chr21	45108001/C//T	synonymous	0	2
P2RY10	chrX	78216570/C//A	nonsynonymous	0	2
TEX13B	chrX	107225121/G//A	synonymous	0	2
TTC34	chr1	2704168/G//A	nonsynonymous	0	2
ALDH4A1	chr1	19202875/G//A	synonymous	0	2
HNRNPR	chr1	23637528/G//A	nonsynonymous	0	2
BCL9	chr1	147091036/C//T	nonsynonymous	0	2
SELP	chr1	169580777/C//T	nonsynonymous	0	2
SEC16B	chr1	177927431/G//A	nonsynonymous	0	2
C4BPA	chr1	207297306/C//T	synonymous	0	2
APOB	chr2	21228460/C//T	synonymous	0	2
SCN2A	chr2	166188041/C//T	nonsynonymous	0	2
CALCRL	chr2	188217027/G//A	synonymous	0	2
NBEAL1	chr2	203922136/T//A	nonsynonymous	0	2
KLHL40	chr3	42727514/C//T	nonsynonymous	0	2
BSN	chr3	49689855/C//A	nonsynonymous	0	2
FOXP1	chr3	71015056/G//C	nonsynonymous	0	2
CCDC149	chr4	24833204/T//C	nonsynonymous	0	2
CTNND2	chr5	11022979/C//T	synonymous	0	2
CDH12	chr5	21751762/A//T	synonymous	0	2
SKIV2L2	chr5	54645429/A//C	synonymous	0	2
SLC12A2	chr5	127484508/T//A	nonsynonymous	0	2
PCDHA6	chr5	140209658/C//T	nonsynonymous	0	2
PCDHA10	chr5	140235823/C//T	nonsynonymous	0	2
DST	chr6	56504092/G//A	nonsynonymous	0	2
PNISR	chr6	99862548/A//C	synonymous	0	2
SYNE1	chr6	152694259/C//T	synonymous	0	2
TIAM2	chr6	155451018/G//A	nonsynonymous	0	2
MYL10	chr7	101256809/G//T	nonsynonymous	0	2

LMBR1	chr7	156521374/C//A	synonymous	0	2
MROH6	chr8	144651882/C//G	synonymous	0	2
TTC39B	chr9	15182379/A//C	nonsynonymous	0	2
RNF20	chr9	104297738/A//T	synonymous	0	2
GSN	chr9	124065184/C//T	synonymous	0	2
KCNT1	chr9	138648724/C//T	synonymous	0	2
AP2A2	chr11	1009380/G//A	nonsynonymous	0	2
LMO1	chr11	8251849/G//A	synonymous	0	2
ZDHHC5	chr11	57466726/C//T	synonymous	0	2
DTX3	chr12	58001002/G//A	nonsynonymous	0	2
GAS2L3	chr12	101018303/G//T	nonsynonymous	0	2
STAB2	chr12	104049365/G//A	synonymous	0	2
DACH1	chr13	72053354/C//G	nonsynonymous	0	2
SLC15A1	chr13	99360965/G//A	nonsynonymous	0	2
OR4N2	chr14	20295838/G//A	synonymous	0	2
HERC2	chr15	28505917/C//T	synonymous	0	2
BUB1B-PAK6	chr15	40564422/C//A	synonymous	0	2
JMJD7-PLA2G4B	chr15	42132385/G//A	synonymous	0	2
ANKS4B	chr16	21261257/C//T	nonsynonymous	0	2
KIAA0556	chr16	27751487/C//T	synonymous	0	2
CNOT1	chr16	58555154/G//C	nonsynonymous	0	2
JPH3	chr16	87678059/G//A	nonsynonymous	0	2
PITPNM3	chr17	6373651/C//T	nonsynonymous	0	2
TP53	chr17	7579312/C//T	synonymous	0	2
CENPV	chr17	16253293/A//T	nonsynonymous	0	2
CCT6B	chr17	33269517/C//T	nonsynonymous	0	2
ABCA6	chr17	67082780/T//C	nonsynonymous	0	2
TMPRSS9	chr19	2405505/C//T	synonymous	0	2
MUC16	chr19	9061629/C//T	nonsynonymous	0	2
WIZ	chr19	15558930/G//T	synonymous	0	2
ELL	chr19	18572626/G//T	nonsynonymous	0	2
CILP2	chr19	19654099/C//T	synonymous	0	2
UBA2	chr19	34951452/C//T	synonymous	0	2
ZNF225	chr19	44636885/A//G	synonymous	0	2
FPR2	chr19	52272072/G//A	nonsynonymous	0	2
DEFB128	chr20	168744/T//G	nonsynonymous	0	2
MRPS26	chr20	3026773/T//TGG	nonsynonymous	0	2
POTED	chr21	14982506/C//T	synonymous	0	2
MYO18B	chr22	26423239/C//T	synonymous	0	2
LARGE1	chr22	33780238/C//T	nonsynonymous	0	2
CERK	chr22	47108093/G//A	synonymous	0	2
KLHL34	chrX	21674416/G//A	synonymous	0	2
ABCB7	chrX	74289296/G//A	synonymous	0	2
COL4A5	chrX	107850057/G//A	nonsynonymous	0	2
MIER1	chr1	67437399/T//A	nonsynonymous	0	2
SPTA1	chr1	158641934/C//T	nonsynonymous	0	2
CADM3	chr1	159162405/G//A	synonymous	0	2
PRRC2C	chr1	171510110/C//T	nonsynonymous	0	2
KCNS3	chr2	18112885/G//A	nonsynonymous	0	2
MARCO	chr2	119735446/G//A	nonsynonymous	0	2
LRP2	chr2	170044631/G//A	synonymous	0	2
COL6A3	chr2	238283455/G//A	synonymous	0	2
CAPN10	chr2	241535800/GC//G	nonsynonymous	0	2
PTH1R	chr3	46945102/C//T	nonsynonymous	0	2
C3orf62	chr3	49314135/C//T	synonymous	0	2
GNAI2	chr3	50295131/G//A	synonymous	0	2
TLR9	chr3	52257944/G//A	synonymous	0	2
EPHA3	chr3	89259600/C//T	synonymous	0	2
LOC101929106	chr3	186915250/G//A	synonymous	0	2
LRBA	chr4	151511990/G//A	nonsynonymous	0	2
GRM1	chr6	146755554/G//A	synonymous	0	2
AFDN	chr6	168366435/A//C	nonsynonymous	0	2
SCRN1	chr7	29976312/C//A	nonsynonymous	0	2
GLI3	chr7	42005805/G//A	nonsynonymous	0	2
ALKBH4	chr7	102098332/C//T	nonsynonymous	0	2
AASS	chr7	121741475/G//A	synonymous	0	2
RPS20	chr8	56985782/G//A	nonsynonymous	0	2
ANKRD18A	chr9	38615690/G//A	synonymous	0	2
LAMC3	chr9	133942460/G//A	nonsynonymous	0	2
OGDHL	chr10	50943247/C//A	synonymous	0	2

CTNNA3	chr10	68139057/C//A	nonsynonymous	0	2
FAM160B1	chr10	116595383/G//A	nonsynonymous	0	2
IFITM10	chr11	1756635/C//A	nonsynonymous	0	2
PTPMT1	chr11	47593062/G//A	nonsynonymous	0	2
ATM	chr11	108124595/A//G	synonymous	0	2
SORL1	chr11	121458770/C//T	nonsynonymous	0	2
SLCO1B1	chr12	21353611/GACATATTTTTTAC//G	synonymous	0	2
KMT2D	chr12	49431709/C//T	nonsynonymous	0	2
TNS2	chr12	53452368/C//T	nonsynonymous	0	2
LRP1	chr12	57573135/G//A	nonsynonymous	0	2
HECTD4	chr12	112610547/C//T	nonsynonymous	0	2
MED13L	chr12	116452987/T//A	nonsynonymous	0	2
MSI1	chr12	120795616/C//T	synonymous	0	2
TUBA3C	chr13	19747960/G//A	synonymous	0	2
RNASE12	chr14	21058725/C//T	nonsynonymous	0	2
ABHD12B	chr14	51344792/A//T	nonsynonymous	0	2
TEX9	chr15	56657655/G//A	nonsynonymous	0	2
ADAMTS7	chr15	79092787/C//T	nonsynonymous	0	2
SYNM	chr15	99653860/G//A	nonsynonymous	0	2
ADGRG1	chr16	57693415/G//A	synonymous	0	2
PHLPP2	chr16	71683330/G//A	synonymous	0	2
SOX15	chr17	7493053/G//A	synonymous	0	2
TP53	chr17	7577565/T//G	nonsynonymous	0	2
ERBB2	chr17	37866626/G//A	nonsynonymous	0	2
MTMR4	chr17	56573372/G//A	nonsynonymous	0	2
DUS1L	chr17	80020801/C//T	nonsynonymous	0	2
CSNK1D	chr17	80210409/A//G	nonsynonymous	0	2
ZBTB7A	chr19	4054578/T//G	nonsynonymous	0	2
TUBB4A	chr19	6495666/G//A	nonsynonymous	0	2
MUC16	chr19	8971814/C//T	nonsynonymous	0	2
TSSK6	chr19	19625855/C//T	nonsynonymous	0	2
FUT2	chr19	49206784/C//T	nonsynonymous	0	2
LRRC4B	chr19	51020907/G//A	nonsynonymous	0	2
NLRP4	chr19	56370309/C//T	nonsynonymous	0	2
CST1	chr20	23731307/C//T	nonsynonymous	0	2
ELMO2	chr20	45008900/T//TTATAGACCGGAGA	nonsynonymous	0	2
ELFN2	chr22	37771475/C//T	nonsynonymous	0	2
CACNA1I	chr22	40080504/G//A	nonsynonymous	0	2
NHS	chrX	17744925/G//A	nonsynonymous	0	2
PINK1-AS	chr1	20975352/G//A	synonymous	0	2
MFSD2A	chr1	40434383/C//T	nonsynonymous	0	2
SYT6	chr1	114640405/G//A	nonsynonymous	0	2
SUCO	chr1	172520757/C//A	nonsynonymous	0	2
DHX9	chr1	182847315/C//A	synonymous	0	2
ARID4B	chr1	235384944/G//A	nonsynonymous	0	2
RASGRP3	chr2	33783393/G//A	synonymous	0	2
SPTBN1	chr2	54858309/A//G	nonsynonymous	0	2
PRPF40A	chr2	153529138/C//G	nonsynonymous	0	2
LNPK	chr2	176794717/C//T	nonsynonymous	0	2
TOP2B	chr3	25668727/C//T	nonsynonymous	0	2
ERC2	chr3	56468542/C//T	nonsynonymous	0	2
PIK3CA	chr3	178927405/G//A	nonsynonymous	0	2
VWA5B2	chr3	183955098/C//T	synonymous	0	2
NAT8L	chr4	2062814/G//A	nonsynonymous	0	2
ABHD18	chr4	128905493/G//C	nonsynonymous	0	2
FBXL7	chr5	15936846/C//T	nonsynonymous	0	2
ADAMTS12	chr5	33576233/G//T	nonsynonymous	0	2
DMXL1	chr5	118573026/G//C	nonsynonymous	0	2
PCDHB15	chr5	140626476/G//A	nonsynonymous	0	2
FAM196B	chr5	169291376/C//T	synonymous	0	2
ZNF454	chr5	178391652/T//A	synonymous	0	2
TBC1D9B	chr5	179301933/C//T	nonsynonymous	0	2
RNF182	chr6	13977817/C//T	nonsynonymous	0	2
ALDH5A1	chr6	24528372/G//A	nonsynonymous	0	2
TFEB	chr6	41658537/G//T	nonsynonymous	0	2
TTBK1	chr6	43230694/C//T	nonsynonymous	0	2
AK9	chr6	109886053/A//T	synonymous	0	2
FUCA2	chr6	143825256/G//T	synonymous	0	2
SYNE1	chr6	152485341/G//T	nonsynonymous	0	2
SEMA3E	chr7	83032029/G//A	synonymous	0	2

TRIM24	chr7	138210038/C//G	nonsynonymous	0	2
EPHA1	chr7	143098560/C//T	nonsynonymous	0	2
PCM1	chr8	17808196/T//G	nonsynonymous	0	2
ADCY8	chr8	131916264/C//T	synonymous	0	2
AK8	chr9	135601126/G//A	synonymous	0	2
COL5A1	chr9	137622232/G//A	nonsynonymous	0	2
FBXW5	chr9	139837245/C//T	nonsynonymous	0	2
WDFY4	chr10	50025362/G//A	nonsynonymous	0	2
SH3PXD2A	chr10	105363170/C//T	nonsynonymous	0	2
MYBPC3	chr11	47353651/TGCCTCGCCCTGTAA(	nonsynonymous	0	2
DDX10	chr11	108547830/C//T	nonsynonymous	0	2
FZD10	chr12	130649126/C//A	nonsynonymous	0	2
GALNT9	chr12	132834388/G//A	nonsynonymous	0	2
PABPC3	chr13	25670547/A//G	nonsynonymous	0	2
KCNH5	chr14	63416962/C//T	nonsynonymous	0	2
AHNAK2	chr14	105408167/C//T	nonsynonymous	0	2
PACS2	chr14	105843233/C//T	synonymous	0	2
EIF2AK4	chr15	40259780/G//C	nonsynonymous	0	2
CFAP161	chr15	81440257/G//A	nonsynonymous	0	2
ADAMTSL3	chr15	84611716/C//T	nonsynonymous	0	2
TICRR	chr15	90144726/G//T	nonsynonymous	0	2
PLCG2	chr16	81888168/G//A	nonsynonymous	0	2
GUCY2D	chr17	7909860/C//T	synonymous	0	2
COL1A1	chr17	48262919/C//T	nonsynonymous	0	2
MPO	chr17	56356997/C//T	synonymous	0	2
GREB1L	chr18	19030983/G//C	nonsynonymous	0	2
DSG3	chr18	29041264/G//T	nonsynonymous	0	2
PTPRS	chr19	5208035/C//T	synonymous	0	2
SLC6A16	chr19	49812267/G//A	synonymous	0	2
SHANK1	chr19	51207687/C//T	synonymous	0	2
NLRP4	chr19	56369355/C//T	nonsynonymous	0	2
EBF4	chr20	2736394/C//T	synonymous	0	2
SIGLEC1	chr20	3679928/G//A	synonymous	0	2
DSCAM	chr21	41710041/G//A	synonymous	0	2
CELSR1	chr22	46806455/G//A	synonymous	0	2
FAM47A	chrX	34149076/G//A	synonymous	0	2
ITIH6	chrX	54785263/C//T	nonsynonymous	0	2
IL13RA2	chrX	114244226/T//A	nonsynonymous	0	2
SLC12A2	chr5	127419934/G//T	synonymous	0	2
TAS1R2	chr1	19166642/G//A	synonymous	0	2
PLA2G2D	chr1	20441992/G//A	synonymous	0	2
RPS6KA1	chr1	26873448/G//T	nonsynonymous	0	2
PSRC1	chr1	109823599/C//T	nonsynonymous	0	2
LCE2B	chr1	152659570/G//A	nonsynonymous	0	2
DENND4B	chr1	153902804/C//T	nonsynonymous	0	2
MEF2D	chr1	156450646/C//T	nonsynonymous	0	2
F5	chr1	169529958/G//A	synonymous	0	2
GREB1	chr2	11773069/G//A	nonsynonymous	0	2
TTN	chr2	179574580/C//T	nonsynonymous	0	2
B3GNT7	chr2	232262990/C//T	nonsynonymous	0	2
ATP2B2	chr3	10387254/G//A	synonymous	0	2
PTH1R	chr3	46944925/C//T	nonsynonymous	0	2
ZNF589	chr3	48282661/G//T	synonymous	0	2
GOLIM4	chr3	167758622/T//C	synonymous	0	2
EPHA5	chr4	66217147/C//T	nonsynonymous	0	2
FSTL5	chr4	162376247/G//A	nonsynonymous	0	2
SLCO6A1	chr5	101795418/G//A	synonymous	0	2
IL17B	chr5	148756510/G//A	nonsynonymous	0	2
CANX	chr5	179155704/C//T	synonymous	0	2
GPX5	chr6	28499621/A//G	nonsynonymous	0	2
CARD11	chr7	2956983/C//T	nonsynonymous	0	2
ZPBP	chr7	50097646/G//A	synonymous	0	2
SHH	chr7	155604641/C//T	nonsynonymous	0	2
LEPROTL1	chr8	29963261/G//A	nonsynonymous	0	2
PKHD1L1	chr8	110439239/G//A	nonsynonymous	0	2
TAF2	chr8	120744276/T//C	nonsynonymous	0	2
DAB2IP	chr9	124536676/C//G	synonymous	0	2
RPL12	chr9	130213658/G//A	synonymous	0	2
ITIH2	chr10	7791191/C//T	nonsynonymous	0	2
SEC24C	chr10	75525213/C//T	nonsynonymous	0	2

INPP5F	chr10	121586580/C//T	nonsynonymous	0	2
OR52L1	chr11	6007675/G//A	synonymous	0	2
FAT3	chr11	92534204/G//A	synonymous	0	2
SUOX	chr12	56398366/G//A	nonsynonymous	0	2
PARP4	chr13	25020846/A//T	synonymous	0	2
AKAP6	chr14	33201657/T//C	synonymous	0	2
ESRRB	chr14	76957987/C//T	nonsynonymous	0	2
RCOR1	chr14	103180847/G//A	nonsynonymous	0	2
KCTD5	chr16	2732553/G//A	nonsynonymous	0	2
KRT33A	chr17	39506939/G//A	synonymous	0	2
CASKIN2	chr17	73500913/C//G	nonsynonymous	0	2
CHST9	chr18	24722731/G//A	nonsynonymous	0	2
RTTN	chr18	67860633/G//T	nonsynonymous	0	2
SCAMP4	chr19	1923078/G//T	synonymous	0	2
UNC13A	chr19	17759354/A//C	nonsynonymous	0	2
DACT3	chr19	47151800/G//A	nonsynonymous	0	2
KRTAP26-1	chr21	31692123/T//G	nonsynonymous	0	2
LSS	chr21	47616130/A//T	nonsynonymous	0	2
LRRIQ3	chr1	74621436/A//C	nonsynonymous	0	2
PTGFRN	chr1	117527385/C//T	nonsynonymous	0	2
HMCN1	chr1	186055376/A//G	nonsynonymous	0	2
CRB1	chr1	197390123/T//G	synonymous	0	2
RGS7	chr1	241262053/C//T	nonsynonymous	0	2
TRIM58	chr1	248028085/C//T	nonsynonymous	0	2
C2orf16	chr2	27800019/G//C	nonsynonymous	0	2
ALMS1	chr2	73675086/T//G	synonymous	0	2
DCTN1	chr2	74593117/G//A	nonsynonymous	0	2
TTN	chr2	179433497/G//C	nonsynonymous	0	2
TTN	chr2	179435825/G//A	nonsynonymous	0	2
ITGAV	chr2	187523864/A//T	nonsynonymous	0	2
ITGAV	chr2	187523871/A//C	nonsynonymous	0	2
COL4A4	chr2	227954648/G//A	synonymous	0	2
IQCA1	chr2	237240012/T//A	nonsynonymous	0	2
GRM7	chr3	7456740/T//A	nonsynonymous	0	2
CMTM8	chr3	32409387/C//G	synonymous	0	2
ITIH3	chr3	52830583/C//A	synonymous	0	2
CLSTN2	chr3	140282886/C//T	nonsynonymous	0	2
TFRC	chr3	195800982/A//T	nonsynonymous	0	2
AMTN	chr4	71398221/G//C	synonymous	0	2
ZNF366	chr5	71756620/T//C	nonsynonymous	0	2
APC	chr5	112174368/A//G	nonsynonymous	0	2
DTWD2	chr5	118183880/C//G	nonsynonymous	0	2
SLC22A5	chr5	131724685/A//T	nonsynonymous	0	2
PCDHGB7	chr5	140799203/G//A	nonsynonymous	0	2
NR3C1	chr5	142779628/A//G	synonymous	0	2
MAS1L	chr6	29455513/G//A	nonsynonymous	0	2
ZBTB12	chr6	31867915/C//T	nonsynonymous	0	2
AGPAT1	chr6	32137096/C//T	nonsynonymous	0	2
TRERF1	chr6	42196326/C//T	synonymous	0	2
HTR1E	chr6	87725542/C//T	nonsynonymous	0	2
RPS6KA2	chr6	166912105/T//C	nonsynonymous	0	2
AFDN	chr6	168281175/C//G	nonsynonymous	0	2
RAC1	chr7	6442061/A//G	nonsynonymous	0	2
SRI	chr7	87838768/C//T	nonsynonymous	0	2
GIGYF1	chr7	100285988/G//A	synonymous	0	2
RIMS2	chr8	104898096/T//G	synonymous	0	2
PCSK5	chr9	78953292/C//A	nonsynonymous	0	2
C9orf170	chr9	89771487/G//T	nonsynonymous	0	2
TTC16	chr9	130487166/C//T	nonsynonymous	0	2
ZNF239	chr10	44052784/C//T	synonymous	0	2
PSAP	chr10	73578875/A//T	synonymous	0	2
PTEN	chr10	89685281/C//A	nonsynonymous	0	2
P2RY2	chr11	72945588/C//T	synonymous	0	2
HEPHL1	chr11	93806533/G//A	nonsynonymous	0	2
TTC12	chr11	113235672/G//C	nonsynonymous	0	2
DRD2	chr11	113287668/C//T	nonsynonymous	0	2
KCNH3	chr12	49942696/C//T	nonsynonymous	0	2
LRP1	chr12	57559637/C//T	synonymous	0	2
MDM1	chr12	68707296/G//C	nonsynonymous	0	2
KCNC2	chr12	75444245/T//A	nonsynonymous	0	2

EEA1	chr12	93169745/T//G	synonymous	0	2
ASCL1	chr12	103352569/G//A	nonsynonymous	0	2
HCFC2	chr12	104458288/C//G	synonymous	0	2
EPSTI1	chr13	43462531/G//A	nonsynonymous	0	2
PCDH17	chr13	58207224/G//T	nonsynonymous	0	2
CCDC168	chr13	103391809/C//G	nonsynonymous	0	2
MCF2L	chr13	113751232/C//T	synonymous	0	2
VCPKMT	chr14	50579441/T//C	synonymous	0	2
HERC2	chr15	28356964/T//C	nonsynonymous	0	2
MAP1A	chr15	43814768/C//A	nonsynonymous	0	2
ASB7	chr15	101152555/C//T	nonsynonymous	0	2
N4BP1	chr16	48595715/C//A	nonsynonymous	0	2
CDH5	chr16	66426126/G//A	nonsynonymous	0	2
SLC12A4	chr16	67979715/C//T	synonymous	0	2
NXN	chr17	722749/C//A	nonsynonymous	0	2
TP53	chr17	7577559/G//C	nonsynonymous	0	2
NR1D1	chr17	38256315/A//T	nonsynonymous	0	2
ACBD4	chr17	43216515/G//A	nonsynonymous	0	2
PLEKHM1	chr17	43552494/C//T	nonsynonymous	0	2
SPPL2C	chr17	43924136/T//A	nonsynonymous	0	2
CACNG5	chr17	64873407/T//C	synonymous	0	2
SALL3	chr18	76753806/C//T	synonymous	0	2
PPAN-P2RY11	chr19	10225208/C//T	nonsynonymous	0	2
EPHX3	chr19	15338577/C//T	synonymous	0	2
ZNF85	chr19	21132145/C//A	synonymous	0	2
GYS1	chr19	49473855/C//T	nonsynonymous	0	2
GYS1	chr19	49488804/C//T	nonsynonymous	0	2
SSC5D	chr19	56005278/C//T	synonymous	0	2
BPIFB3	chr20	31644469/C//T	synonymous	0	2
PCIF1	chr20	44576376/C//T	synonymous	0	2
ADAMTS1	chr21	28212796/G//T	nonsynonymous	0	2
MED15	chr22	20929408/G//A	synonymous	0	2
MTMR8	chrX	63557167/C//G	nonsynonymous	0	2
TBX22	chrX	79281201/A//G	synonymous	0	2
MCF2	chrX	138679654/C//A	nonsynonymous	0	2
WDR63	chr1	85592280/C//T	synonymous	0	2
COL24A1	chr1	86361567/C//A	nonsynonymous	0	2
NRAS	chr1	115258748/C//A	nonsynonymous	0	2
AQP10	chr1	154296135/C//T	nonsynonymous	0	2
OR6K3	chr1	158687177/G//T	nonsynonymous	0	2
NCSTN	chr1	160314608/G//A	nonsynonymous	0	2
KCNT2	chr1	196398790/C//T	nonsynonymous	0	2
TIA1	chr2	70475545/G//A	synonymous	0	2
	chr2	127818194/G//A	synonymous	0	2
PDE11A	chr2	178576605/G//C	nonsynonymous	0	2
TTN	chr2	179582456/C//T	nonsynonymous	0	2
LRRC3B	chr3	26751568/C//T	synonymous	0	2
PIK3CA	chr3	178916890/C//T	nonsynonymous	1	1
PALLD	chr4	169837147/A//G	nonsynonymous	0	2
IRX1	chr5	3600770/T//A	nonsynonymous	0	2
PRDM9	chr5	23521146/G//A	synonymous	0	2
CDH10	chr5	24537633/G//A	nonsynonymous	0	2
IL7R	chr5	35876230/G//A	nonsynonymous	0	2
FCHO2	chr5	72264372/T//C	synonymous	0	2
IQGAP2	chr5	75979032/A//G	synonymous	0	2
	chr5	96062532/A//G	synonymous	0	2
APC	chr5	112116592/C//T	nonsynonymous	0	2
PCDHAC1	chr5	140307011/C//T	synonymous	0	2
PCDHGB1	chr5	140730646/G//C	nonsynonymous	0	2
PCDHGA6	chr5	140753980/C//T	synonymous	0	2
HMP19	chr5	173534535/C//T	synonymous	0	2
MGAT4B	chr5	179225987/G//T	nonsynonymous	0	2
ZNF655	chr7	99169919/C//T	nonsynonymous	0	2
COL22A1	chr8	139856384/C//T	nonsynonymous	0	2
OR1B1	chr9	125391523/G//A	nonsynonymous	0	2
SVIL	chr10	29788191/C//T	nonsynonymous	0	2
KNDC1	chr10	135012256/G//A	synonymous	0	2
OR4S2	chr11	55419095/G//T	nonsynonymous	0	2
PPP1CA	chr11	67166217/G//A	synonymous	0	2
NARS2	chr11	78204159/T//C	nonsynonymous	0	2

MMP12	chr11	102738782/G//A	synonymous	0	2
KRT4	chr12	53201627/C//T	nonsynonymous	0	2
KRT18	chr12	53346031/G//A	synonymous	0	2
SPRYD4	chr12	56862997/G//GAC	nonsynonymous	0	2
PABPC3	chr13	25670780/C//T	synonymous	0	2
TNFSF11	chr13	43175105/G//A	nonsynonymous	0	2
TRIP11	chr14	92436047/C//T	synonymous	0	2
APBA2	chr15	29393892/C//T	nonsynonymous	0	2
TYRO3	chr15	41870493/C//T	synonymous	0	2
CACNA1H	chr16	1252143/G//A	nonsynonymous	0	2
SRRM2	chr16	2816859/C//T	synonymous	0	2
NLRC5	chr16	57080538/T//C	nonsynonymous	0	2
DHODH	chr16	72055167/G//A	nonsynonymous	0	2
DBNDD1	chr16	90075763/G//A	nonsynonymous	0	2
CD300LB	chr17	72522034/G//A	nonsynonymous	0	2
BAHCC1	chr17	79409742/G//T	nonsynonymous	0	2
SMARCA4	chr19	11107001/G//A	nonsynonymous	0	2
FAM71E2	chr19	55869791/C//T	synonymous	0	2
ZNF135	chr19	58573033/G//A	nonsynonymous	0	2
ID1	chr20	30193900/C//G	synonymous	0	2
PI4KA	chr22	21105965/G//A	synonymous	0	2
TBL1X	chrX	9673116/G//A	nonsynonymous	0	2
FAM47A	chrX	34149053/C//T	nonsynonymous	0	2
AMER1	chrX	63411857/AT//A	nonsynonymous	0	2
CXorf57	chrX	105905472/C//G	nonsynonymous	0	2
CDC27	chr17	45214682/A//G	synonymous	0	2
CDC27	chr17	45249306/T//C	synonymous	0	2
CNN2	chr19	1037718/G//T	nonsynonymous	1	1
CNN2	chr19	1037731/C//T	synonymous	1	1
CELSR2	chr1	109806825/C//T	synonymous	0	2
CFAP45	chr1	159846370/C//T	nonsynonymous	0	2
NHLH1	chr1	160340950/C//A	synonymous	0	2
TBC1D8	chr2	101624566/T//A	nonsynonymous	0	2
EFCC1	chr3	128757682/G//A	synonymous	0	2
AGTR1	chr3	148458855/T//C	synonymous	0	2
SELENOT	chr3	150340945/C//T	nonsynonymous	0	2
UTS2B	chr3	190993079/G//A	nonsynonymous	0	2
COL25A1	chr4	110223192/G//A	synonymous	0	2
CDH12	chr5	21854778/A//G	nonsynonymous	0	2
ELOVL7	chr5	60050429/G//A	synonymous	0	2
LHFPL2	chr5	77806035/A//G	nonsynonymous	0	2
PCDHGA11	chr5	140802666/G//A	synonymous	0	2
PTCHD4	chr6	47846507/G//A	synonymous	0	2
GINM1	chr6	149903738/A//C	nonsynonymous	0	2
GRID2IP	chr7	6590937/C//T	nonsynonymous	0	2
ABCB8	chr7	150739194/C//T	synonymous	0	2
FAM135B	chr8	139180201/C//T	nonsynonymous	0	2
KDM4C	chr9	6849705/G//A	synonymous	0	2
FRMPD1	chr9	37740243/C//G	nonsynonymous	0	2
SPATA31C2	chr9	90745960/C//A	nonsynonymous	0	2
EBF3	chr10	131640527/C//T	nonsynonymous	0	2
CYB5R2	chr11	7690492/A//C	nonsynonymous	0	2
ALKBH8	chr11	107420502/C//T	nonsynonymous	0	2
UCHL3	chr13	76143602/C//T	nonsynonymous	0	2
ACSM5	chr16	20451694/C//T	nonsynonymous	0	2
ZP2	chr16	21210898/G//T	nonsynonymous	0	2
LOXHD1	chr18	44184136/C//A	nonsynonymous	0	2
MYO1F	chr19	8604901/C//T	nonsynonymous	0	2
VSTM2B	chr19	30020879/C//T	nonsynonymous	0	2
BCAS1	chr20	52561412/G//C	synonymous	0	2
OSBP2	chr22	31289934/A//T	nonsynonymous	0	2
PRR14L	chr22	32108310/G//A	nonsynonymous	0	2
ATP7A	chrX	77245039/C//T	synonymous	0	2
CAMTA1	chr1	7797372/C//T	nonsynonymous	0	2
MTOR	chr1	11174416/G//A	nonsynonymous	0	2
CROCC	chr1	17281249/G//A	synonymous	0	2
ARID1A	chr1	27087516/C//T	nonsynonymous	0	2
CCDC18	chr1	93701915/G//A	synonymous	0	2
CHRNB2	chr1	154544383/C//T	nonsynonymous	0	2
APCS	chr1	159557776/G//T	nonsynonymous	0	2

C4BPA	chr1	207288790/C//T	nonsynonymous	0	2
TRAF5	chr1	211534505/G//A	nonsynonymous	0	2
HEATR5B	chr2	37310477/A//G	synonymous	0	2
MAP4K4	chr2	102486762/C//G	nonsynonymous	0	2
GYPC	chr2	127453576/G//A	nonsynonymous	0	2
CCDC74B	chr2	130897828/C//T	nonsynonymous	0	2
WDR12	chr2	203764258/C//T	nonsynonymous	0	2
KIAA1143	chr3	44803099/G//A	synonymous	0	2
CACNA2D2	chr3	50413083/A//G	synonymous	0	2
ROBO2	chr3	77147197/C//T	nonsynonymous	0	2
IFT57	chr3	107910402/G//A	nonsynonymous	0	2
BCHE	chr3	165491137/G//T	synonymous	0	2
ZBBX	chr3	167035292/G//A	synonymous	0	2
ZNF732	chr4	264929/G//C	nonsynonymous	0	2
ENAM	chr4	71509947/G//T	nonsynonymous	0	2
HERC5	chr4	89391766/G//A	nonsynonymous	0	2
FAT4	chr4	126373819/G//A	nonsynonymous	0	2
FAT4	chr4	126412778/T//A	nonsynonymous	0	2
ADAMTS16	chr5	5303830/G//A	nonsynonymous	0	2
TRIO	chr5	14304606/G//A	nonsynonymous	0	2
LIFR	chr5	38506646/C//T	synonymous	0	2
HCN1	chr5	45262694/A//C	nonsynonymous	0	2
APC	chr5	112175231/AG//A	nonsynonymous	0	2
PCDHB2	chr5	140475774/G//T	nonsynonymous	0	2
PCDHB2	chr5	140476594/C//T	synonymous	0	2
AGER	chr6	32149150/G//A	nonsynonymous	0	2
GTPBP2	chr6	43589794/T//C	synonymous	0	2
MCM3	chr6	52132742/T//C	nonsynonymous	0	2
TIAM2	chr6	155500564/C//T	nonsynonymous	0	2
ZNF107	chr7	64167325/C//A	nonsynonymous	0	2
BRAF	chr7	140453193/T//A	nonsynonymous	0	2
SNAI2	chr8	49833898/C//T	synonymous	0	2
RPS20	chr8	56985664/G//A	synonymous	0	2
PSKH2	chr8	87081758/C//T	nonsynonymous	0	2
CSMD3	chr8	113347667/C//T	synonymous	0	2
DENND3	chr8	142161840/C//T	synonymous	0	2
FAM205A	chr9	34723338/C//T	nonsynonymous	0	2
GABBR2	chr9	101156497/G//A	synonymous	0	2
OR10A3	chr11	7960939/G//C	synonymous	0	2
NAV2	chr11	20127136/G//A	nonsynonymous	0	2
CXCR5	chr11	118765145/G//A	nonsynonymous	0	2
CASC1	chr12	25272197/C//T	synonymous	0	2
KRAS	chr12	25378561/G//A	nonsynonymous	0	2
WNT10B	chr12	49360032/C//T	nonsynonymous	0	2
NAV3	chr12	78400656/G//T	nonsynonymous	0	2
ATP2B1	chr12	90015397/T//C	nonsynonymous	0	2
AMDHD1	chr12	96346520/C//T	nonsynonymous	0	2
EP400	chr12	132464291/C//T	nonsynonymous	0	2
SLITRK1	chr13	84454159/G//A	nonsynonymous	0	2
RPL10L	chr14	47120869/C//T	nonsynonymous	0	2
NRDE2	chr14	90770394/T//C	nonsynonymous	0	2
EML1	chr14	100387183/C//T	synonymous	0	2
TBL3	chr16	2024079/G//C	nonsynonymous	0	2
DCUN1D3	chr16	20873571/C//T	nonsynonymous	0	2
IGSF6	chr16	21652814/G//A	synonymous	0	2
	chr16	27414458/C//T	synonymous	0	2
SRCAP	chr16	30735763/C//T	nonsynonymous	0	2
PKD1L3	chr16	71981428/C//A	nonsynonymous	0	2
TP53	chr17	7578493/C//T	nonsynonymous	0	2
NOG	chr17	54672334/C//T	synonymous	0	2
SOX9	chr17	70117888/C//T	nonsynonymous	0	2
MUC16	chr19	9072560/G//T	synonymous	0	2
PNKP	chr19	50365820/TTC//T	nonsynonymous	0	2
PCSK2	chr20	17434451/G//A	nonsynonymous	0	2
PYGB	chr20	25259713/C//A	nonsynonymous	0	2
BPIFB3	chr20	31644455/G//A	nonsynonymous	0	2
PHACTR3	chr20	58318189/G//A	nonsynonymous	0	2
CXorf57	chrX	105876238/C//T	nonsynonymous	0	2
MTOR	chr1	11190804/C//T	nonsynonymous	0	2
PTPRU	chr1	29649988/C//T	nonsynonymous	0	2

CCDC30	chr1	43119549/G//T	nonsynonymous	0	2
RAB3B	chr1	52385726/C//T	nonsynonymous	0	2
USH2A	chr1	216373317/T//G	nonsynonymous	0	2
	chr1	228547794/C//T	synonymous	0	2
DNMT3A	chr2	25467072/C//T	nonsynonymous	0	2
SLC8A1	chr2	40366572/G//A	synonymous	0	2
FER1L5	chr2	97364418/G//A	nonsynonymous	0	2
RANBP2	chr2	109384577/G//C	nonsynonymous	0	2
KCNH7	chr2	163241412/G//A	synonymous	0	2
LRP2	chr2	170145603/C//T	synonymous	0	2
TTN	chr2	179599566/G//A	nonsynonymous	0	2
	chr2	179611639/C//T	synonymous	0	2
ERBB4	chr2	212812202/T//A	nonsynonymous	0	2
ABCA12	chr2	215820002/T//C	nonsynonymous	0	2
EPHA4	chr2	222291194/C//T	nonsynonymous	0	2
KIF1A	chr2	241697789/C//T	nonsynonymous	0	2
SLC10A6	chr4	87749282/C//T	nonsynonymous	0	2
ARFIP1	chr4	153809339/T//C	synonymous	0	2
CTNND2	chr5	11346540/C//T	synonymous	0	2
DNAH5	chr5	13717534/G//A	nonsynonymous	0	2
DNAH5	chr5	13839564/G//C	nonsynonymous	0	2
APC	chr5	112155040/AAGT//A	nonsynonymous	0	2
APC	chr5	112173968/G//T	nonsynonymous	0	2
ARL14EPL	chr5	115389771/G//A	nonsynonymous	0	2
KIF20A	chr5	137521396/G//A	nonsynonymous	0	2
PCDHGA8	chr5	140774290/C//T	nonsynonymous	0	2
	chr5	172660466/C//A	synonymous	0	2
GRM6	chr5	178408780/G//A	nonsynonymous	0	2
TNXB	chr6	32016215/G//T	nonsynonymous	0	2
GPR31	chr6	167570915/C//T	synonymous	0	2
NPC1L1	chr7	44556399/T//C	nonsynonymous	0	2
ZNF107	chr7	64166886/G//A	synonymous	0	2
SSPO	chr7	149474914/G//A	nonsynonymous	0	2
FGFR1	chr8	38273515/C//T	nonsynonymous	0	2
C8orf89	chr8	74153697/G//A	nonsynonymous	0	2
ABRA	chr8	107782026/G//A	synonymous	0	2
ADCY8	chr8	131922058/G//A	synonymous	0	2
LHX3	chr9	139092585/C//A	nonsynonymous	0	2
RPP30	chr10	92654596/G//A	nonsynonymous	0	2
KRTAP5-3	chr11	1628975/C//T	nonsynonymous	0	2
HBB	chr11	5248007/TC//T	nonsynonymous	0	2
HBB	chr11	5248009/C//A	nonsynonymous	0	2
CCDC34	chr11	27360530/C//T	synonymous	0	2
SLCO1B1	chr12	21325669/G//A	nonsynonymous	0	2
ARNTL2	chr12	27571069/C//T	synonymous	0	2
HOXC11	chr12	54367314/G//A	nonsynonymous	0	2
CDK4	chr12	58143021/G//A	nonsynonymous	0	2
PLXNC1	chr12	94641708/C//T	synonymous	0	2
SLC41A2	chr12	105255075/T//C	nonsynonymous	0	2
PCCA	chr13	100953799/A//G	nonsynonymous	0	2
CHAMP1	chr13	115090859/C//G	nonsynonymous	0	2
SYNE2	chr14	64554536/A//G	nonsynonymous	0	2
SYNE3	chr14	95899734/G//A	nonsynonymous	0	2
TLN2	chr15	63055872/C//T	nonsynonymous	0	2
NEO1	chr15	73593699/C//T	synonymous	0	2
RAB40C	chr16	677687/C//T	synonymous	0	2
HYDIN	chr16	70937972/G//T	synonymous	0	2
PRPF8	chr17	1584915/C//T	synonymous	0	2
TP53	chr17	7578403/C//A	nonsynonymous	0	2
SETBP1	chr18	42531573/G//C	synonymous	0	2
MED16	chr19	891134/G//A	synonymous	0	2
C3	chr19	6697381/C//T	nonsynonymous	0	2
ZNF560	chr19	9577403/A//G	synonymous	0	2
FIZ1	chr19	56104205/C//T	nonsynonymous	0	2
NAA20	chr20	20007498/C//T	synonymous	0	2
CSF2RB	chr22	37319384/G//A	nonsynonymous	0	2
EFHC2	chrX	44101415/T//A	nonsynonymous	0	2
GUCY2F	chrX	108628457/C//G	nonsynonymous	0	2
ALG13	chrX	110961403/C//T	nonsynonymous	0	2
PRR32	chrX	125955050/C//A	synonymous	0	2

EXTL1	chr1	26357056/AGTGGAGAAGGTCAT(	nonsynonymous	0	2
KTI12	chr1	52498620/G//A	nonsynonymous	0	2
PYHIN1	chr1	158913731/T//C	nonsynonymous	0	2
MRPL55	chr1	228295398/G//A	nonsynonymous	0	2
NYAP2	chr2	226447267/G//A	synonymous	0	2
ARL8B	chr3	5164236/A//G	nonsynonymous	0	2
WFS1	chr4	6303707/G//A	nonsynonymous	0	2
DNAH5	chr5	13771075/G//T	nonsynonymous	0	2
PCDHA11	chr5	140248958/C//T	synonymous	0	2
MUC17	chr7	100681310/T//G	nonsynonymous	0	2
CNBD1	chr8	87878734/C//A	nonsynonymous	0	2
PKD2L1	chr10	102057297/G//A	synonymous	0	2
MUC6	chr11	1017596/T//G	nonsynonymous	0	2
UBQLN3	chr11	5529277/C//T	synonymous	0	2
LATS2	chr13	21562902/G//A	synonymous	0	2
PPP1R36	chr14	65054003/G//A	nonsynonymous	0	2
THSD4	chr15	72063436/C//T	nonsynonymous	0	2
IGF1R	chr15	99434645/C//T	synonymous	0	2
ZFHX3	chr16	72827200/C//T	synonymous	0	2
TP53	chr17	7578280/G//A	nonsynonymous	0	2
PIAS4	chr19	4028729/C//T	synonymous	0	2
TUBB4A	chr19	6495439/C//T	synonymous	0	2
CST2	chr20	23807165/G//A	nonsynonymous	0	2
ARHGAP40	chr20	37266370/G//T	synonymous	0	2
TXNRD2	chr22	19882966/C//T	nonsynonymous	0	2
RRP7A	chr22	42910123/C//T	nonsynonymous	0	2
MAGEB3	chrX	30254946/C//T	nonsynonymous	0	2
EIF4G3	chr1	21183906/T//C	nonsynonymous	0	2
SPTA1	chr1	158615071/T//C	synonymous	0	2
SELP	chr1	169562858/G//A	nonsynonymous	0	2
ZNF2	chr2	95847186/C//T	nonsynonymous	0	2
TTN	chr2	179430920/G//A	nonsynonymous	0	2
ZNF804A	chr2	185802272/A//G	nonsynonymous	0	2
SLC16A14	chr2	230911062/G//A	synonymous	0	2
HRH1	chr3	11300877/G//A	nonsynonymous	0	2
STXBP5L	chr3	120973858/A//G	nonsynonymous	0	2
DNAH5	chr5	13810276/G//A	nonsynonymous	0	2
APC	chr5	112173299/A//T	nonsynonymous	0	2
REEP2	chr5	137780550/C//T	synonymous	0	2
PCDHA12	chr5	140257073/C//T	synonymous	0	2
NOP16	chr5	175811323/C//T	nonsynonymous	0	2
DBN1	chr5	176894484/C//T	nonsynonymous	0	2
TUBB2A	chr6	3154768/C//T	nonsynonymous	0	2
HIVEP2	chr6	143094845/T//C	nonsynonymous	0	2
SCRN1	chr7	29980495/G//T	synonymous	0	2
PXDNL	chr8	52321298/G//A	synonymous	0	2
FBXO43	chr8	101153061/T//G	nonsynonymous	0	2
FBXO43	chr8	101153070/T//C	nonsynonymous	0	2
S1PR3	chr9	91617087/C//T	synonymous	0	2
NFIL3	chr9	94171857/T//C	nonsynonymous	0	2
ZNF618	chr9	116750814/C//T	synonymous	0	2
SFMBT2	chr10	7213976/C//T	nonsynonymous	0	2
CELF2	chr10	11259384/G//A	synonymous	0	2
GAD2	chr10	26559562/C//T	synonymous	0	2
KNDC1	chr10	135015124/C//A	nonsynonymous	0	2
MUC5B	chr11	1263797/C//G	nonsynonymous	0	2
UBQLN3	chr11	5530063/A//C	nonsynonymous	0	2
OR56A3	chr11	5969113/G//T	nonsynonymous	0	2
EXPH5	chr11	108380424/C//T	nonsynonymous	0	2
OR9K2	chr12	55524135/G//A	nonsynonymous	0	2
SHMT2	chr12	57627809/C//T	nonsynonymous	0	2
SACS	chr13	23909025/C//T	nonsynonymous	0	2
HSPH1	chr13	31711307/T//A	synonymous	0	2
LRCH1	chr13	47286716/C//T	synonymous	0	2
SIN3A	chr15	75705361/G//A	nonsynonymous	0	2
KIF7	chr15	90191935/C//T	nonsynonymous	0	2
FURIN	chr15	91418815/C//T	synonymous	0	2
AMDHD2	chr16	2578553/C//T	synonymous	0	2
TP53	chr17	7577547/C//A	nonsynonymous	0	2
SMARCE1	chr17	38801843/G//A	synonymous	0	2

FBF1	chr17	73906853/A//G	nonsynonymous	0	2
FASN	chr17	80044989/G//A	nonsynonymous	0	2
IZUMO4	chr19	2097444/G//A	nonsynonymous	0	2
TLE6	chr19	2995011/G//A	synonymous	0	2
ZFR2	chr19	3810779/C//T	nonsynonymous	0	2
PGLYRP2	chr19	15586552/C//G	nonsynonymous	0	2
USHBP1	chr19	17361222/C//T	nonsynonymous	0	2
ATP1A3	chr19	42490359/G//A	nonsynonymous	0	2
DIDO1	chr20	61512103/C//T	synonymous	0	2
FTCD	chr21	47558513/G//A	nonsynonymous	0	2
SDF2L1	chr22	21997210/C//A	synonymous	0	2
PRAME	chr22	22892383/C//A	nonsynonymous	0	2
ITIH6	chrX	54777579/C//T	nonsynonymous	0	2
DCAF12L2	chrX	125298954/C//A	nonsynonymous	0	2
MAMLD1	chrX	149680307/G//A	synonymous	0	2
PSEN2	chr1	227081727/C//T	synonymous	0	1
OBSCN	chr1	228528254/G//A	synonymous	0	1
LRP1B	chr2	141812733/G//A	nonsynonymous	0	1
NEB	chr2	152586136/G//A	nonsynonymous	0	1
G6PC2	chr2	169764299/G//A	nonsynonymous	0	1
HDAC4	chr2	239975273/C//A	nonsynonymous	0	1
SEC22A	chr3	122928222/T//C	nonsynonymous	0	1
IGSF10	chr3	151165674/G//A	nonsynonymous	0	1
ACTL6A	chr3	179287636/A//G	nonsynonymous	0	1
LIFR	chr5	38481718/A//G	synonymous	0	1
PCDHA7	chr5	140216232/G//A	nonsynonymous	0	1
NMUR2	chr5	151784383/C//A	nonsynonymous	0	1
PFDN6	chr6	33258189/C//G	synonymous	0	1
IFNGR1	chr6	137519225/G//A	synonymous	0	1
WBSCR17	chr7	71142212/C//A	nonsynonymous	0	1
DPP6	chr7	154681011/G//A	synonymous	0	1
CCDC166	chr8	144789995/G//A	synonymous	0	1
LOC100128593	chr9	139642803/G//A	synonymous	0	1
NRP1	chr10	33469287/G//A	nonsynonymous	0	1
MUC5B	chr11	1269741/C//G	synonymous	0	1
SLC22A8	chr11	62763203/C//T	nonsynonymous	0	1
MAP3K11	chr11	65366953/C//A	synonymous	0	1
FAT3	chr11	92534306/C//A	synonymous	0	1
APOA5	chr11	116661270/A//G	synonymous	0	1
UBASH3B	chr11	122647787/G//A	nonsynonymous	0	1
WNT10B	chr12	49359825/A//G	synonymous	0	1
SVOP	chr12	109372444/G//A	synonymous	0	1
SLITRK5	chr13	88328265/C//T	nonsynonymous	0	1
CCDC168	chr13	103395705/T//A	nonsynonymous	0	1
	chr14	24836123/G//C	synonymous	0	1
RALGAPA1	chr14	36017715/T//C	synonymous	0	1
RYR3	chr15	34021138/G//A	nonsynonymous	0	1
POLG	chr15	89864150/C//T	nonsynonymous	0	1
NFATC2IP	chr16	28962559/C//G	nonsynonymous	0	1
FUS	chr16	31202389/G//A	nonsynonymous	0	1
STAC2	chr17	37381740/C//T	nonsynonymous	0	1
SMAD4	chr18	48604722/G//C	nonsynonymous	0	1
ZNF14	chr19	19822629/G//T	synonymous	0	1
ASXL1	chr20	31023270/A//AT	nonsynonymous	0	1
EMILIN3	chr20	39990789/C//T	nonsynonymous	0	1
KRTAP10-3	chr21	45977975/G//A	synonymous	0	1
HPS4	chr22	26879765/C//T	synonymous	0	1
ABCB7	chrX	74295352/G//A	nonsynonymous	0	1
TTC13	chr1	231067545/G//C	synonymous	0	1
USP53	chr4	120212345/G//A	synonymous	0	1
PCDHB2	chr5	140474438/T//G	nonsynonymous	0	1
ABCC10	chr6	43412666/G//C	nonsynonymous	0	1
CNR1	chr6	88854928/G//A	synonymous	0	1
SP4	chr7	21468417/G//A	synonymous	0	1
ENTPD4	chr8	23294634/C//T	nonsynonymous	0	1
VPS13A	chr9	79954834/G//T	synonymous	0	1
KIAA0368	chr9	114135524/A//C	synonymous	0	1
OR5D16	chr11	55606482/G//A	synonymous	0	1
SENP1	chr12	48491856/T//C	nonsynonymous	0	1
CLDN9	chr16	3064074/G//C	synonymous	0	1

KRTAP4-2	chr17	39334186/A//G	synonymous	0	1
MRC2	chr17	60769840/G//A	synonymous	0	1
ZNF407	chr18	72345941/A//G	nonsynonymous	0	1
ZNF559	chr19	9452431/G//C	nonsynonymous	0	1
ZNF729	chr19	22498764/A//G	nonsynonymous	0	1
	chr19	40903992/G//A	synonymous	0	1
CXorf36	chrX	45059975/G//T	nonsynonymous	0	1
ZBTB17	chr1	16274878/T//C	nonsynonymous	1	0
ZSCAN20	chr1	33960621/C//T	nonsynonymous	1	0
KIAA0319L	chr1	35900476/G//A	synonymous	1	0
ZFP69	chr1	40945115/G//A	nonsynonymous	1	0
SLFNL1	chr1	41483014/C//T	synonymous	1	0
OSBPL9	chr1	52242576/G//T	synonymous	1	0
SNX7	chr1	99225611/G//T	nonsynonymous	1	0
OLFML2B	chr1	161987210/GCCCCA//G	nonsynonymous	1	0
CFH	chr1	196646684/A//G	nonsynonymous	1	0
FAM84A	chr2	14774371/C//T	nonsynonymous	1	0
APOB	chr2	21237377/T//C	nonsynonymous	1	0
FAM179A	chr2	29274661/T//C	nonsynonymous	1	0
LRP1B	chr2	141083397/G//T	nonsynonymous	1	0
TANC1	chr2	160075763/G//C	synonymous	1	0
DNAH7	chr2	196729006/A//G	nonsynonymous	1	0
MARCH4	chr2	217123959/G//C	synonymous	1	0
CHL1	chr3	401981/G//A	nonsynonymous	1	0
EXOSC7	chr3	45046796/C//T	nonsynonymous	1	0
CADPS	chr3	62518545/C//A	nonsynonymous	1	0
UBA3	chr3	69111006/G//A	nonsynonymous	1	0
PARP14	chr3	122432731/C//T	nonsynonymous	1	0
DZIP1L	chr3	137813808/C//T	nonsynonymous	1	0
VWA5B2	chr3	183957713/G//C	nonsynonymous	1	0
NUDT9	chr4	88375539/T//C	nonsynonymous	1	0
FAT1	chr4	187549345/T//A	nonsynonymous	1	0
CDH10	chr5	24511534/C//T	nonsynonymous	1	0
ANKHD1	chr5	139865214/A//T	nonsynonymous	1	0
NOP16	chr5	175815488/T//C	nonsynonymous	1	0
ZFP2	chr5	178358703/A//G	nonsynonymous	1	0
PKHD1	chr6	51941108/G//A	synonymous	1	0
KLHL31	chr6	53519033/G//C	nonsynonymous	1	0
TINAG	chr6	54219323/T//C	nonsynonymous	1	0
HDAC2	chr6	114262214/T//A	synonymous	1	0
DSE	chr6	116720628/C//T	nonsynonymous	1	0
EEPD1	chr7	36324333/C//T	synonymous	1	0
EGFR	chr7	55241707/G//A	nonsynonymous	1	0
EGFR	chr7	55249005/G//T	nonsynonymous	1	0
CAPZA2	chr7	116557787/A//G	nonsynonymous	1	0
FLNC	chr7	128483276/C//T	synonymous	1	0
CSMD1	chr8	3165982/G//A	synonymous	1	0
PROSC	chr8	37632969/T//A	nonsynonymous	1	0
MTERF3	chr8	97269291/T//C	nonsynonymous	1	0
ZNF251	chr8	145948076/A//AG	nonsynonymous	1	0
ADAMTSL1	chr9	18706827/C//T	synonymous	1	0
ANK3	chr10	61965627/T//C	nonsynonymous	1	0
CFAP46	chr10	134664793/G//A	nonsynonymous	1	0
TRPM5	chr11	2436631/A//G	nonsynonymous	1	0
RAG1	chr11	36595433/G//C	nonsynonymous	1	0
PKNOX2	chr11	125299970/C//T	synonymous	1	0
PHB2	chr12	7079764/C//A	synonymous	1	0
OVCH1	chr12	29640670/T//A	nonsynonymous	1	0
KRT74	chr12	52966327/C//T	nonsynonymous	1	0
RPL10L	chr14	47120750/C//A	nonsynonymous	1	0
SIX4	chr14	61190487/C//A	synonymous	1	0
DYNC1H1	chr14	102500443/C//T	nonsynonymous	1	0
MYO9A	chr15	72154872/T//C	nonsynonymous	1	0
IGF1R	chr15	99482454/A//G	nonsynonymous	1	0
	chr16	30000921/A//T	synonymous	1	0
NOD2	chr16	50741792/G//A	synonymous	1	0
CHD9	chr16	53191341/G//A	nonsynonymous	1	0
MYH2	chr17	10438620/G//T	synonymous	1	0
TANC2	chr17	61466019/A//G	synonymous	1	0
WDR7	chr18	54606595/T//A	synonymous	1	0

POLRMT	chr19	619660/C//T	nonsynonymous	1	0
ZNF98	chr19	22574507/C//T	nonsynonymous	1	0
ZNF99	chr19	22940819/C//T	nonsynonymous	1	0
GEMIN7	chr19	45593803/G//A	synonymous	1	0
PCBP3	chr21	47333890/C//T	nonsynonymous	1	0
CYLC1	chrX	83128482/G//T	nonsynonymous	1	0
TSPAN6	chrX	99890621/G//A	synonymous	1	0
NRK	chrX	105167201/G//A	nonsynonymous	1	0
COL4A5	chrX	107811865/C//T	nonsynonymous	1	0
CSMD2	chr1	34164507/G//T	synonymous	1	0
	chr3	42947777/T//C	synonymous	1	0
FRAS1	chr4	79321907/T//C	nonsynonymous	1	0
DPCR1	chr6	30919042/T//A	nonsynonymous	1	0
ZNF680	chr7	63982032/C//T	nonsynonymous	1	0
HK1	chr10	71136767/T//C	nonsynonymous	1	0
CEP55	chr10	95262948/A//G	nonsynonymous	1	0
TPCN2	chr11	68848953/A//G	nonsynonymous	1	0
AHNAK2	chr14	105411924/T//C	synonymous	1	0
AHNAK2	chr14	105411935/A//G	synonymous	1	0
MRC2	chr17	60767046/C//T	synonymous	1	0
C5AR1	chr19	47823842/C//T	nonsynonymous	1	0
ZNF628	chr19	55993019/C//T	synonymous	1	0
ZNF141	chr4	367169/G//A	nonsynonymous	1	0
ZNF141	chr4	367201/C//A	synonymous	1	0
ZNF141	chr4	367206/C//G	nonsynonymous	1	0
GAS6	chr13	114529991/G//A	synonymous	1	0
KRTAP10-5	chr21	45999964/G//T	synonymous	1	0
HRH1	chr3	11301096/C//T	nonsynonymous	0	1
AGTR1	chr3	148459850/A//T	nonsynonymous	0	1
AGGF1	chr5	76349784/C//A	synonymous	0	1
ZSCAN12	chr6	28366147/G//C	nonsynonymous	0	1
PILRA	chr7	99971731/C//T	synonymous	0	1
DDX10	chr11	108535968/A//T	nonsynonymous	0	1
SMARCC2	chr12	56558290/T//C	nonsynonymous	0	1
CREBBP	chr16	3830753/C//A	synonymous	0	1
KIR2DS4	chr19	55344151/C//T	synonymous	0	1
SLC25A14	chrX	129483282/C//T	synonymous	0	1
SLITRK4	chrX	142717857/T//C	synonymous	0	1
CROCC	chr1	17280760/A//C	nonsynonymous	1	0
ARID1A	chr1	27107087/GGGCT//G	nonsynonymous	1	0
ARID1A	chr1	27107093/CCCGCG//C	nonsynonymous	1	0
SZT2	chr1	43912737/C//G	nonsynonymous	1	0
UROD	chr1	45480627/G//T	nonsynonymous	1	0
ZCCHC11	chr1	52924026/AAC//A	nonsynonymous	1	0
SHC1	chr1	154942572/G//C	nonsynonymous	1	0
FCRL5	chr1	157488568/G//T	nonsynonymous	1	0
ATP1A4	chr1	160143513/A//T	synonymous	1	0
CACNA1E	chr1	181724510/C//A	synonymous	1	0
CACNA1E	chr1	181741358/C//T	synonymous	1	0
NAV1	chr1	201751574/G//A	nonsynonymous	1	0
ANGEL2	chr1	213170538/A//T	nonsynonymous	1	0
GTF3C2	chr2	27552303/T//G	nonsynonymous	1	0
EML6	chr2	55184953/C//T	nonsynonymous	1	0
MRPL19	chr2	75881922/T//C	nonsynonymous	1	0
GCFC2	chr2	75900599/ATCT//A	nonsynonymous	1	0
GCFC2	chr2	75900602/T//A	nonsynonymous	1	0
FABP1	chr2	88422601/A//G	nonsynonymous	1	0
ZNF804A	chr2	185798333/C//T	nonsynonymous	1	0
KANSL1L	chr2	210896258/A//C	nonsynonymous	1	0
SP110	chr2	231042250/A//C	synonymous	1	0
NSUN3	chr3	93813134/A//G	nonsynonymous	1	0
GUCY1A3	chr4	156625091/G//T	nonsynonymous	1	0
TENM3	chr4	183601037/T//C	synonymous	1	0
PCDHA8	chr5	140220990/C//A	nonsynonymous	1	0
PCDHB7	chr5	140553130/C//T	synonymous	1	0
RMND5B	chr5	177571073/C//G	nonsynonymous	1	0
MICA	chr6	31378326/A//G	nonsynonymous	1	0
LY6G6F	chr6	31675548/C//T	synonymous	1	0
COL21A1	chr6	55925822/C//A	nonsynonymous	1	0
DST	chr6	56380313/T//C	nonsynonymous	1	0

AKIRIN2	chr6	88387630/TAG//T	nonsynonymous	1	0
ASCC3	chr6	100964182/C//A	nonsynonymous	1	0
DDO	chr6	110714120/C//T	nonsynonymous	1	0
TUBE1	chr6	112405419/C//T	synonymous	1	0
C7orf65	chr7	47698664/T//C	synonymous	1	0
ZNF679	chr7	63727002/A//C	nonsynonymous	1	0
MUC17	chr7	100678029/G//T	nonsynonymous	1	0
SPIDR	chr8	48196640/G//A	synonymous	1	0
ATAD2	chr8	124357307/C//T	synonymous	1	0
ODF2	chr9	131256862/A//G	nonsynonymous	1	0
SMC3	chr10	112362968/G//A	nonsynonymous	1	0
CYP2E1	chr10	135341064/G//A	synonymous	1	0
CNGA4	chr11	6261799/G//A	nonsynonymous	1	0
CYB5R2	chr11	7690950/T//G	nonsynonymous	1	0
OR5M8	chr11	56258015/T//C	nonsynonymous	1	0
OR1S2	chr11	57971539/G//T	nonsynonymous	1	0
CCDC88B	chr11	64120974/T//C	synonymous	1	0
RBM4B	chr11	66436740/C//A	nonsynonymous	1	0
TECTA	chr11	121036011/G//T	nonsynonymous	1	0
GALNT8	chr12	4870212/T//C	nonsynonymous	1	0
DENND5B	chr12	31648795/G//C	nonsynonymous	1	0
NR4A1	chr12	52448993/C//T	synonymous	1	0
ITGB7	chr12	53590529/G//A	nonsynonymous	1	0
ACSS3	chr12	81647122/G//T	nonsynonymous	1	0
STAB2	chr12	104149253/C//T	synonymous	1	0
HECTD4	chr12	112601067/G//A	nonsynonymous	1	0
PAN3	chr13	28748505/T//C	nonsynonymous	1	0
DNAJC3	chr13	96443237/C//T	nonsynonymous	1	0
CDH24	chr14	23518864/A//G	synonymous	1	0
PLEKHG3	chr14	65210089/G//T	nonsynonymous	1	0
NUDT14	chr14	105643034/G//A	synonymous	1	0
CYFIP1	chr15	22947069/A//T	nonsynonymous	1	0
SLC12A6	chr15	34531307/C//T	nonsynonymous	1	0
GCNT3	chr15	59911563/G//T	nonsynonymous	1	0
CILP	chr15	65490909/C//T	nonsynonymous	1	0
AXIN1	chr16	338238/T//TG	nonsynonymous	1	0
NOD2	chr16	50746078/G//A	synonymous	1	0
HYDIN	chr16	70926329/C//A	nonsynonymous	1	0
FAM64A	chr17	6348729/TACTG//T	synonymous	1	0
PIGW	chr17	34894458/T//A	nonsynonymous	1	0
ZNF709	chr19	12575176/T//C	synonymous	1	0
ZNF709	chr19	12575188/G//A	synonymous	1	0
ZNF709	chr19	12575197/G//A	synonymous	1	0
ZNF709	chr19	12575212/T//C	synonymous	1	0
ZNF99	chr19	22952103/C//T	synonymous	1	0
RELB	chr19	45532212/G//A	nonsynonymous	1	0
S100B	chr21	48022200/A//G	synonymous	1	0
EFCAB6	chr22	43924715/G//A	synonymous	1	0
CERK	chr22	47085968/G//C	nonsynonymous	1	0
CSF2RA	chrX	1428376/C//A	synonymous	1	0
SLC25A6	chrX	1506277/C//T	nonsynonymous	1	0
MXRA5	chrX	3242042/C//T	nonsynonymous	1	0
RGN	chrX	46952345/G//A	synonymous	1	0
COL4A5	chrX	107867484/G//T	nonsynonymous	1	0
	chr1	52499523/T//C	synonymous	0	1
TCTEX1D1	chr1	67243077/T//C	synonymous	0	1
FBN2	chr5	127710398/C//T	nonsynonymous	0	1
URGCP-MRPS24	chr7	43927020/T//A	nonsynonymous	0	1
PRLHR	chr10	120353828/G//A	nonsynonymous	0	1
LTBR	chr12	6494299/G//A	nonsynonymous	0	1
DNAJC15	chr13	43681416/G//A	synonymous	0	1
ZNF681	chr19	23927648/C//A	nonsynonymous	0	1
LTBP4	chr19	41115429/C//A	synonymous	0	1
CHD6	chr20	40049342/T//C	nonsynonymous	0	1
CDH4	chr20	60419770/C//T	nonsynonymous	0	1
MEGF6	chr1	3428150/C//T	nonsynonymous	0	1
TMEM131	chr2	98388801/G//C	synonymous	0	1
VWA3B	chr2	98887138/T//TG	synonymous	0	1
SAP130	chr2	128757930/G//A	nonsynonymous	0	1
ZEB2	chr2	145154083/C//T	nonsynonymous	0	1

ACADL	chr2	211085530/A//G	synonymous	0	1
SLC19A3	chr2	228566973/A//C	nonsynonymous	0	1
OR6B2	chr2	240968930/C//T	nonsynonymous	0	1
CLRN1	chr3	150645667/C//G	synonymous	0	1
MECOM	chr3	168845744/A//G	nonsynonymous	0	1
BMP3	chr4	81974645/T//G	synonymous	0	1
FGA	chr4	155507878/C//T	nonsynonymous	0	1
RNF180	chr5	63509426/G//T	synonymous	0	1
NR2F1	chr5	92924069/G//A	nonsynonymous	0	1
PCDHB11	chr5	140581749/G//T	synonymous	0	1
MUC22	chr6	30995635/C//T	synonymous	0	1
KCNK16	chr6	39285555/G//A	synonymous	0	1
CRISP1	chr6	49825093/C//A	nonsynonymous	0	1
MDN1	chr6	90362784/T//C	nonsynonymous	0	1
HECW1	chr7	43540360/C//T	nonsynonymous	0	1
COL1A2	chr7	94027077/A//G	synonymous	0	1
FOXP2	chr7	114294066/T//C	nonsynonymous	0	1
DLC1	chr8	12946153/C//G	nonsynonymous	0	1
GFRA2	chr8	21608125/C//T	nonsynonymous	0	1
SCARA5	chr8	27779217/G//A	nonsynonymous	0	1
BRF2	chr8	37702136/C//T	nonsynonymous	0	1
PKHD1L1	chr8	110509264/T//G	nonsynonymous	0	1
CSMD3	chr8	113301594/C//T	nonsynonymous	0	1
NOL8	chr9	95080971/A//G	synonymous	0	1
ZRANB1	chr10	126673556/G//A	nonsynonymous	0	1
A2M-AS1	chr12	9220458/A//AC	synonymous	0	1
ITPR2	chr12	26809309/G//A	nonsynonymous	0	1
NCKAP1L	chr12	54910704/G//A	synonymous	0	1
TPH2	chr12	72338219/C//T	nonsynonymous	0	1
TCTN2	chr12	124181001/A//G	nonsynonymous	0	1
TEX26	chr13	31543103/C//T	nonsynonymous	0	1
	chr14	96998645/T//G	synonymous	0	1
GCOM1	chr15	58001163/A//T	nonsynonymous	0	1
TPSAB1	chr16	1291439/G//A	nonsynonymous	0	1
ANKRD30B	chr18	14763745/C//T	nonsynonymous	0	1
CAMSAP3	chr19	7675772/C//T	nonsynonymous	0	1
DOPEY2	chr21	37618898/C//A	synonymous	0	1
ZBED4	chr22	50278618/G//A	synonymous	0	1
ACTRT2	chr1	2938364/C//T	synonymous	1	0
TP73	chr1	3647538/G//A	nonsynonymous	1	0
SELENON	chr1	26138212/C//T	nonsynonymous	1	0
SMG5	chr1	156220714/C//T	nonsynonymous	1	0
PYHIN1	chr1	158908254/A//G	synonymous	1	0
NCSTN	chr1	160314587/T//A	nonsynonymous	1	0
OLFML2B	chr1	161954010/C//T	nonsynonymous	1	0
REN	chr1	204131165/G//A	synonymous	1	0
SPRTN	chr1	231488794/C//T	nonsynonymous	1	0
SNTG2	chr2	1168851/C//T	synonymous	1	0
FEZ2	chr2	36818080/A//G	nonsynonymous	1	0
LRP1B	chr2	141116494/G//T	nonsynonymous	1	0
LRP1B	chr2	141299446/C//T	nonsynonymous	1	0
LRP2	chr2	170101368/G//A	nonsynonymous	1	0
PLCL1	chr2	198949948/G//A	synonymous	1	0
TNS1	chr2	218713295/C//T	nonsynonymous	1	0
CHL1	chr3	383694/G//A	nonsynonymous	1	0
RARB	chr3	25502710/G//T	nonsynonymous	1	0
DNAH12	chr3	57414099/T//A	nonsynonymous	1	0
ARF4	chr3	57569724/C//T	synonymous	1	0
ARHGAP31	chr3	119120876/G//A	nonsynonymous	1	0
XRN1	chr3	142066080/T//A	nonsynonymous	1	0
	chr4	6037757/G//A	synonymous	1	0
MANBA	chr4	103586012/G//C	synonymous	1	0
EGF	chr4	110929386/G//T	nonsynonymous	1	0
ANK2	chr4	114223940/C//A	nonsynonymous	1	0
APC	chr5	112175251/GAGCGAAGTTCC//G	nonsynonymous	1	0
CEP120	chr5	122751791/T//C	synonymous	1	0
PCDHA3	chr5	140182465/C//T	synonymous	1	0
PCDHA3	chr5	140182651/C//T	synonymous	1	0
ANKS1A	chr6	34985338/C//T	synonymous	1	0
USP45	chr6	99936581/A//T	synonymous	1	0

ARMC2	chr6	109294741/A//G	synonymous	1	0
RSPH3	chr6	159421010/C//T	synonymous	1	0
PRKAR1B	chr7	635794/G//A	nonsynonymous	1	0
RADIL	chr7	4855901/A//G	nonsynonymous	1	0
ZNF138	chr7	64292279/A//G	nonsynonymous	1	0
STEAP4	chr7	87913400/G//A	nonsynonymous	1	0
RELN	chr7	103124179/C//T	nonsynonymous	1	0
GEM	chr8	95272598/C//T	nonsynonymous	1	0
TRHR	chr8	110100448/A//G	nonsynonymous	1	0
CCDC180	chr9	100111446/G//A	nonsynonymous	1	0
RET	chr10	43595916/G//T	nonsynonymous	1	0
PCDH15	chr10	55849814/G//T	synonymous	1	0
PTEN	chr10	89692940/C//T	nonsynonymous	1	0
ANKRD1	chr10	92672647/C//A	nonsynonymous	1	0
EBF3	chr10	131666103/G//A	synonymous	1	0
MUC2	chr11	1101101/C//A	nonsynonymous	1	0
OR5L2	chr11	55595115/C//A	nonsynonymous	1	0
OR5B2	chr11	58190150/C//A	synonymous	1	0
CLPB	chr11	72018239/T//C	synonymous	1	0
KLRC1	chr12	10603633/C//T	synonymous	1	0
TBC1D15	chr12	72291684/A//G	synonymous	1	0
FBXW8	chr12	117462049/G//A	nonsynonymous	1	0
CIT	chr12	120156617/G//T	nonsynonymous	1	0
DYNC1H1	chr14	102471157/C//T	nonsynonymous	1	0
DNAH3	chr16	21063049/C//T	nonsynonymous	1	0
IRX5	chr16	54966694/G//A	synonymous	1	0
UTP6	chr17	30202347/G//T	nonsynonymous	1	0
KRT34	chr17	39538435/C//T	nonsynonymous	1	0
FSCN2	chr17	79502166/C//A	nonsynonymous	1	0
CXXC1	chr18	47810129/C//T	synonymous	1	0
ZNF91	chr19	23544261/T//G	nonsynonymous	1	0
TSHZ3	chr19	31770258/G//A	synonymous	1	0
CCDC114	chr19	48821986/G//A	nonsynonymous	1	0
ZNF614	chr19	52519922/C//T	nonsynonymous	1	0
PEG3	chr19	57328804/C//T	nonsynonymous	1	0
ZCCHC3	chr20	279539/A//G	synonymous	1	0
LZTR1	chr22	21344712/A//G	nonsynonymous	1	0
MTFP1	chr22	30823324/T//C	nonsynonymous	1	0
CLCN5	chrX	49853429/A//G	synonymous	1	0
GPR173	chrX	53106133/G//A	synonymous	1	0
TENM1	chrX	123785896/G//T	nonsynonymous	1	0
VPS13D	chr1	12326959/C//G	nonsynonymous	1	0
CD48	chr1	160654968/G//A	nonsynonymous	1	0
F5	chr1	169528488/G//A	synonymous	1	0
PRRC2C	chr1	171514610/T//C	synonymous	1	0
KIAA1614	chr1	180897590/T//C	synonymous	1	0
ADSS	chr1	244600961/T//C	synonymous	1	0
ATP6V1C2	chr2	10924461/C//A	synonymous	1	0
NCOA1	chr2	24991317/A//G	synonymous	1	0
SULT1C3	chr2	108868909/C//T	synonymous	1	0
NEUROD1	chr2	182543123/C//G	nonsynonymous	1	0
KANSL1L	chr2	210888764/TCTTGA//T	nonsynonymous	1	0
ADAMTS9	chr3	64601739/T//C	nonsynonymous	1	0
SLCO2A1	chr3	133698403/G//A	synonymous	1	0
SMC4	chr3	160150140/G//A	nonsynonymous	1	0
CLDN16	chr3	190106143/G//A	nonsynonymous	1	0
GBA3	chr4	22749310/G//A	synonymous	1	0
CCDC149	chr4	24854805/G//C	synonymous	1	0
SHISA3	chr4	42403166/C//T	nonsynonymous	1	0
ATP8A1	chr4	42487542/A//T	synonymous	1	0
FAT4	chr4	126389756/T//G	nonsynonymous	1	0
SLC25A4	chr4	186066034/C//G	synonymous	1	0
FAT1	chr4	187539695/G//GA	nonsynonymous	1	0
RANBP3L	chr5	36257108/G//A	nonsynonymous	1	0
C5orf42	chr5	37245883/T//C	nonsynonymous	1	0
DAB2	chr5	39376159/AGATTTGGTAACTGG//	nonsynonymous	1	0
PCDHGB6	chr5	140789798/G//A	nonsynonymous	1	0
HIST1H3D	chr6	26197391/CGCTCTTTCG//C	nonsynonymous	1	0
HIST1H3D	chr6	26197402/GCAGC//G	nonsynonymous	1	0
MDN1	chr6	90411669/G//A	nonsynonymous	1	0

GRIK2	chr6	102266354/T//C	nonsynonymous	1	0
INTS1	chr7	1516460/C//G	nonsynonymous	1	0
DNAH11	chr7	21727014/C//T	synonymous	1	0
KMT2E	chr7	104747667/G//C	nonsynonymous	1	0
	chr7	139056169/C//T	synonymous	1	0
EFR3A	chr8	133008712/A//G	nonsynonymous	1	0
ADGRB1	chr8	143545805/C//G	synonymous	1	0
PIGO	chr9	35089078/C//G	synonymous	1	0
HNRNPF	chr10	43883204/G//T	synonymous	1	0
RUFY2	chr10	70136708/C//T	synonymous	1	0
USP54	chr10	75296196/C//A	nonsynonymous	1	0
MUC5B	chr11	1272534/A//C	synonymous	1	0
TRIM6-TRIM34	chr11	5618072/G//C	synonymous	1	0
DCHS1	chr11	6661461/C//T	nonsynonymous	1	0
OR5B21	chr11	58274803/G//T	nonsynonymous	1	0
AHNAK	chr11	62296388/G//A	nonsynonymous	1	0
FLRT1	chr11	63884881/C//T	nonsynonymous	1	0
INPPL1	chr11	71948459/A//G	synonymous	1	0
EMSY	chr11	76237635/A//G	nonsynonymous	1	0
CREBZF	chr11	85375309/T//C	nonsynonymous	1	0
RNF26	chr11	119206355/G//A	nonsynonymous	1	0
CCT2	chr12	69983422/A//AT	nonsynonymous	1	0
CCDC60	chr12	119966552/G//A	nonsynonymous	1	0
EP400	chr12	132535190/T//A	nonsynonymous	1	0
NAXD	chr13	111274643/A//T	nonsynonymous	1	0
DTD2	chr14	31917547/G//C	nonsynonymous	1	0
TXNDC16	chr14	52957632/T//A	nonsynonymous	1	0
ATP10A	chr15	25963550/C//T	synonymous	1	0
HERC2	chr15	28493826/G//C	nonsynonymous	1	0
DNAJA3	chr16	4492286/A//T	synonymous	1	0
NDE1	chr16	15818335/ACT//A	synonymous	1	0
C16orf62	chr16	19621637/C//G	synonymous	1	0
ITGAX	chr16	31392265/C//T	synonymous	1	0
TP53	chr17	7577545/T//C	nonsynonymous	1	0
ALOXE3	chr17	8014823/C//A	nonsynonymous	1	0
CASC3	chr17	38324469/C//G	nonsynonymous	1	0
MED13	chr17	60042423/C//T	synonymous	1	0
ZNF417	chr19	58427833/C//A	synonymous	1	0
EDEM2	chr20	33711692/C//G	nonsynonymous	1	0
UBE2L3	chr22	21975877/A//G	synonymous	1	0
HCCS	chrX	11136643/A//G	nonsynonymous	1	0
TOP2A	chr17	38557328/A//AAAAG	nonsynonymous	1	0
	chr20	44444029/G//T	synonymous	1	0
DNMT3A	chr2	25505353/G//A	synonymous	0	1
MYO15A	chr17	18034656/G//T	nonsynonymous	0	1
	chr19	51890495/C//T	synonymous	0	1
ZNF480	chr19	52825062/T//C	nonsynonymous	0	1
GLIS1	chr1	54060643/G//A	synonymous	0	1
ZNF695	chr1	247171227/G//T	synonymous	0	1
HERC5	chr4	89400539/A//G	nonsynonymous	0	1
SEMA3C	chr7	80387707/C//T	nonsynonymous	0	1
ADAM18	chr8	39495205/C//T	synonymous	0	1
TATDN1	chr8	125516556/T//C	synonymous	0	1
DBX1	chr11	20177902/G//T	nonsynonymous	0	1
FEN1	chr11	61563538/T//C	synonymous	0	1
ZNF714	chr19	21300905/T//C	nonsynonymous	0	1
AP1B1	chr22	29759007/G//C	synonymous	0	1
ZCCHC11	chr1	52903933/T//C	synonymous	0	1
MFSD14A	chr1	100533730/G//A	nonsynonymous	0	1
TNR	chr1	175335069/G//A	synonymous	0	1
NBAS	chr2	15614187/T//C	synonymous	0	1
MGAT4A	chr2	99294830/G//A	nonsynonymous	0	1
BAZ2B	chr2	160252273/C//T	synonymous	0	1
TTN	chr2	179474247/C//A	nonsynonymous	0	1
KCNH8	chr3	19498240/A//ATC	synonymous	0	1
KCNH8	chr3	19498255/T//G	synonymous	0	1
EHHADH	chr3	184971805/G//A	synonymous	0	1
SFRP2	chr4	154709962/A//G	nonsynonymous	0	1
MARCH1	chr4	164507068/C//T	nonsynonymous	0	1
NEIL3	chr4	178274649/C//G	nonsynonymous	0	1

PCDHA11	chr5	140250917/G//A	synonymous	0	1
PCDHB7	chr5	140553939/C//T	nonsynonymous	0	1
DIAPH1	chr5	140962837/C//T	nonsynonymous	0	1
FAT2	chr5	150947110/A//C	synonymous	0	1
NSD1	chr5	176638793/C//T	synonymous	0	1
MAS1L	chr6	29454693/G//A	synonymous	0	1
UHRF1BP1	chr6	34759954/A//G	synonymous	0	1
ULBP2	chr6	150267513/C//T	nonsynonymous	0	1
CSMD1	chr8	3047554/C//T	nonsynonymous	0	1
PLEKHA2	chr8	38809812/C//T	synonymous	0	1
RGS22	chr8	101083656/G//C	nonsynonymous	0	1
TLE1	chr9	84302310/T//C	nonsynonymous	0	1
KYAT1	chr9	131597829/G//T	nonsynonymous	0	1
AGPAT2	chr9	139572003/A//C	nonsynonymous	0	1
	chr10	55566321/T//C	synonymous	0	1
MYBPC3	chr11	47364615/G//A	synonymous	0	1
FOXR1	chr11	118851387/G//A	nonsynonymous	0	1
SORL1	chr11	121340819/C//G	nonsynonymous	0	1
A2ML1	chr12	9016454/T//C	synonymous	0	1
DIP2B	chr12	51097946/C//T	synonymous	0	1
MBD6	chr12	57920994/C//T	nonsynonymous	0	1
CLEC14A	chr14	38724907/G//A	synonymous	0	1
GEMIN4	chr17	650986/C//T	synonymous	0	1
LAMA1	chr18	7042183/C//G	nonsynonymous	0	1
POTEC	chr18	14542908/C//T	nonsynonymous	0	1
ZNF823	chr19	11832565/A//T	nonsynonymous	0	1
PSMC4	chr19	40480666/G//A	nonsynonymous	0	1
SFI1	chr22	32014387/C//A	synonymous	0	1
DFFB	chr1	3800085/G//A	nonsynonymous	0	1
ALPL	chr1	21904093/C//G	synonymous	0	1
	chr1	26872416/C//T	synonymous	0	1
ZMYM4	chr1	35864590/A//C	nonsynonymous	0	1
ST3GAL3	chr1	44202013/C//T	nonsynonymous	0	1
TNR	chr1	175292483/C//T	synonymous	0	1
KIF26B	chr1	245849618/C//T	synonymous	0	1
ZNF124	chr1	247320365/A//T	nonsynonymous	0	1
PLB1	chr2	28718985/G//A	nonsynonymous	0	1
ANKRD36	chr2	97845505/T//A	nonsynonymous	0	1
ITGA4	chr2	182360535/G//A	nonsynonymous	0	1
IGFBP5	chr2	217543680/T//C	nonsynonymous	0	1
OBSL1	chr2	220422557/C//T	nonsynonymous	0	1
COL4A3	chr2	228168613/C//T	nonsynonymous	0	1
LARS2	chr3	45435950/C//T	nonsynonymous	0	1
USP19	chr3	49151435/C//T	nonsynonymous	0	1
SFMBT1	chr3	52941684/C//T	nonsynonymous	0	1
NFKBIZ	chr3	101570923/G//T	synonymous	0	1
UGT2B28	chr4	70146795/T//C	nonsynonymous	0	1
ANXA5	chr4	122604636/AC//A	synonymous	0	1
FAT4	chr4	126411861/C//T	synonymous	0	1
LARP1B	chr4	129127674/A//C	nonsynonymous	0	1
POU4F2	chr4	147561756/C//T	synonymous	0	1
C5orf42	chr5	37142470/A//G	synonymous	0	1
IQGAP2	chr5	75936934/T//A	synonymous	0	1
FAM114A2	chr5	153407722/G//A	nonsynonymous	0	1
SLC17A3	chr6	25850350/C//T	nonsynonymous	0	1
HIST1H1D	chr6	26234566/T//C	nonsynonymous	0	1
NOTCH4	chr6	32170051/G//A	nonsynonymous	0	1
INTS1	chr7	1529250/G//A	nonsynonymous	0	1
CADPS2	chr7	122130280/G//C	nonsynonymous	0	1
RP1	chr8	55539725/G//A	nonsynonymous	0	1
TRPA1	chr8	72969150/A//T	nonsynonymous	0	1
FBXO43	chr8	101146202/T//C	nonsynonymous	0	1
TONSL	chr8	145661594/C//T	nonsynonymous	0	1
PTPRD	chr9	8636794/C//T	nonsynonymous	0	1
GABBR2	chr9	101340307/G//A	synonymous	0	1
PCBD1	chr10	72643682/A//C	synonymous	0	1
OR5B12	chr11	58207494/A//G	nonsynonymous	0	1
HSPB2	chr11	111783500/G//C	synonymous	0	1
OR10G4	chr11	123886380/C//T	synonymous	0	1
ADAMTS15	chr11	130343086/C//T	synonymous	0	1

SLCO1B3	chr12	21011428/G//A	synonymous	0	1
ARID2	chr12	46287446/C//A	synonymous	0	1
RNFT2	chr12	117178224/C//T	synonymous	0	1
OGFOD2	chr12	123461215/G//A	synonymous	0	1
KBTBD7	chr13	41767850/C//T	nonsynonymous	0	1
BCL2L2	chr14	23778144/A//G	synonymous	0	1
ATG14	chr14	55878516/C//A	nonsynonymous	0	1
SECISBP2L	chr15	49319555/G//T	synonymous	0	1
USP8	chr15	50773694/A//T	nonsynonymous	0	1
TTC23	chr15	99762036/C//T	nonsynonymous	0	1
C16orf59	chr16	2510948/T//C	nonsynonymous	0	1
MMP15	chr16	58077135/G//A	nonsynonymous	0	1
	chr17	8110694/A//G	synonymous	0	1
COX10	chr17	14095332/CTTG//C	nonsynonymous	0	1
FLII	chr17	18157911/G//C	nonsynonymous	0	1
SARM1	chr17	26715534/T//C	synonymous	0	1
SYT4	chr18	40850419/C//T	nonsynonymous	0	1
DOCK6	chr19	11348720/C//T	nonsynonymous	0	1
ZNF99	chr19	22941969/T//C	nonsynonymous	0	1
GRIK5	chr19	42526118/C//T	nonsynonymous	0	1
PLEKHA4	chr19	49357492/G//A	synonymous	0	1
ZNF417	chr19	58420699/C//A	nonsynonymous	0	1
DSCAM	chr21	42080552/G//A	synonymous	0	1
RNF215	chr22	30780470/G//T	nonsynonymous	0	1
KIAA2022	chrX	73960381/G//T	synonymous	0	1
DHRS3	chr1	12638881/T//C	nonsynonymous	1	0
KAZN	chr1	15382629/C//A	synonymous	1	0
INPP5B	chr1	38338649/T//C	nonsynonymous	1	0
SZT2	chr1	43891246/G//C	nonsynonymous	1	0
RAD54L	chr1	46726931/A//G	nonsynonymous	1	0
ORC1	chr1	52851616/C//T	synonymous	1	0
JUN	chr1	59247792/G//A	synonymous	1	0
	chr1	144931697/A//G	synonymous	1	0
FLG	chr1	152283628/T//G	nonsynonymous	1	0
PMVK	chr1	154909061/G//C	synonymous	1	0
ADAM15	chr1	155026945/CACCAGAGCACCCC(	nonsynonymous	1	0
FCRL5	chr1	157504555/C//A	nonsynonymous	1	0
SLC9C2	chr1	173517622/A//G	nonsynonymous	1	0
PROX1	chr1	214170702/C//T	nonsynonymous	1	0
USH2A	chr1	216371894/T//C	nonsynonymous	1	0
OBSCN	chr1	228466868/C//T	synonymous	1	0
ID2	chr2	8822540/A//G	nonsynonymous	1	0
SF3B6	chr2	24290573/A//G	synonymous	1	0
ARHGAP25	chr2	69053179/G//T	nonsynonymous	1	0
TMEM182	chr2	103431399/T//C	nonsynonymous	1	0
NPHP1	chr2	110905561/T//C	nonsynonymous	1	0
GLI2	chr2	121729597/G//T	nonsynonymous	1	0
ZRANB3	chr2	135975169/T//C	synonymous	1	0
RIF1	chr2	152273140/C//T	nonsynonymous	1	0
FAP	chr2	163045647/C//G	nonsynonymous	1	0
DNAH7	chr2	196671513/G//A	nonsynonymous	1	0
CTNNB1	chr3	41266124/A//G	nonsynonymous	1	0
IQCF5	chr3	51909587/C//T	synonymous	1	0
DNAH12	chr3	57386152/T//C	nonsynonymous	1	0
DNAH12	chr3	57394246/T//A	nonsynonymous	1	0
PRICKLE2	chr3	64085564/G//A	synonymous	1	0
MYH15	chr3	108100292/A//G	synonymous	1	0
CCDC80	chr3	112324595/T//G	nonsynonymous	1	0
NNT	chr5	43644783/A//G	nonsynonymous	1	0
WDR41	chr5	76732220/C//T	nonsynonymous	1	0
PCDHB8	chr5	140558930/A//C	nonsynonymous	1	0
KIF4B	chr5	154395644/G//A	nonsynonymous	1	0
LYRM4	chr6	5109731/G//C	synonymous	1	0
MRPS18A	chr6	43648826/T//G	synonymous	1	0
TCTE1	chr6	44253968/G//C	synonymous	1	0
KHDRBS2	chr6	62604653/G//A	nonsynonymous	1	0
PHF3	chr6	64422653/A//T	synonymous	1	0
EPHA7	chr6	93964405/G//T	nonsynonymous	1	0
EPHA7	chr6	93969203/A//G	synonymous	1	0
MMS22L	chr6	97634380/T//C	synonymous	1	0

MMS22L	chr6	97679308/T//C	nonsynonymous	1	0
STK31	chr7	23872011/T//C	synonymous	1	0
GLI3	chr7	42007345/G//T	synonymous	1	0
ZNF679	chr7	63727016/C//G	nonsynonymous	1	0
SAMD9L	chr7	92761755/G//A	nonsynonymous	1	0
SAMD9L	chr7	92764961/T//C	synonymous	1	0
RELN	chr7	103137060/C//T	nonsynonymous	1	0
PRAG1	chr8	8235458/C//T	nonsynonymous	1	0
NEIL2	chr8	11643766/A//G	nonsynonymous	1	0
NEFL	chr8	24813924/C//A	nonsynonymous	1	0
MCM4	chr8	48883893/T//G	synonymous	1	0
PXDNL	chr8	52387558/G//A	nonsynonymous	1	0
RP1	chr8	55541955/G//T	nonsynonymous	1	0
PKHD1L1	chr8	110477038/A//T	synonymous	1	0
CSMD3	chr8	113301671/T//C	nonsynonymous	1	0
RANBP6	chr9	6012568/T//C	nonsynonymous	1	0
MPDZ	chr9	13217183/T//A	nonsynonymous	1	0
TLE1	chr9	84205888/A//T	nonsynonymous	1	0
C9orf84	chr9	114464392/T//G	nonsynonymous	1	0
FBXW5	chr9	139836814/G//A	synonymous	1	0
MEIG1	chr10	15014678/G//A	synonymous	1	0
YME1L1	chr10	27437850/C//G	nonsynonymous	1	0
ARMC4	chr10	28229517/C//T	nonsynonymous	1	0
MPP7	chr10	28408599/T//C	synonymous	1	0
NODAL	chr10	72195393/G//A	synonymous	1	0
IDE	chr10	94214155/G//A	synonymous	1	0
BAG3	chr10	121436267/G//A	nonsynonymous	1	0
SEC23IP	chr10	121658348/G//T	nonsynonymous	1	0
HPX	chr11	6462189/G//C	nonsynonymous	1	0
OR5M1	chr11	56380091/A//T	nonsynonymous	1	0
PATL1	chr11	59410402/G//C	nonsynonymous	1	0
AHNAK	chr11	62293365/G//T	nonsynonymous	1	0
ATL3	chr11	63419981/T//C	nonsynonymous	1	0
MIR6751	chr11	64897449/G//T	synonymous	1	0
GAL3ST3	chr11	65811050/G//A	nonsynonymous	1	0
KMT5B	chr11	67925191/C//G	nonsynonymous	1	0
PPFIA2	chr12	81734880/A//G	synonymous	1	0
ZNF664-FAM101A	chr12	124799047/C//T	synonymous	1	0
FLT1	chr13	28880896/T//C	nonsynonymous	1	0
ALOX5AP	chr13	31338161/T//C	nonsynonymous	1	0
NBEA	chr13	36229790/G//T	nonsynonymous	1	0
NBEA	chr13	36245141/A//T	synonymous	1	0
POSTN	chr13	38151891/A//G	synonymous	1	0
CLDN10	chr13	96205110/A//G	nonsynonymous	1	0
ADCY4	chr14	24802150/C//A	synonymous	1	0
MAGEL2	chr15	23891051/C//T	synonymous	1	0
C16orf96	chr16	4626149/T//G	synonymous	1	0
RABEP2	chr16	28935775/C//A	nonsynonymous	1	0
NLRC5	chr16	57093425/C//G	nonsynonymous	1	0
TMED6	chr16	69383498/G//C	nonsynonymous	1	0
HP	chr16	72088516/C//T	synonymous	1	0
CLUH	chr17	2605359/G//A	synonymous	1	0
MYH13	chr17	10222301/G//T	nonsynonymous	1	0
RARA	chr17	38508733/C//T	nonsynonymous	1	0
EFTUD2	chr17	42927668/C//T	synonymous	1	0
MYOM1	chr18	3188938/A//G	synonymous	1	0
LAMA1	chr18	6999991/C//A	nonsynonymous	1	0
LAMA1	chr18	7050897/A//T	nonsynonymous	1	0
MIER2	chr19	306679/G//A	synonymous	1	0
CFD	chr19	863245/G//A	synonymous	1	0
MUC16	chr19	9046344/G//T	nonsynonymous	1	0
MUC16	chr19	9070992/A//C	nonsynonymous	1	0
ZNF493	chr19	21607315/A//G	nonsynonymous	1	0
ACTN4	chr19	39195593/AAAGAT//A	nonsynonymous	1	0
LILRA4	chr19	54845018/T//G	nonsynonymous	1	0
SIRPD	chr20	1532397/T//C	nonsynonymous	1	0
PAX1	chr20	21695172/G//T	nonsynonymous	1	0
KIAA1755	chr20	36870146/G//A	synonymous	1	0
GRIK1	chr21	31027151/A//G	synonymous	1	0
MAPK1	chr22	22160240/A//T	nonsynonymous	1	0

MORC2	chr22	31330841/C//T	nonsynonymous	1	0
MEI1	chr22	42159235/G//A	synonymous	1	0
CITED1	chrX	71521697/A//G	nonsynonymous	1	0
CSTF2	chrX	100081706/T//A	synonymous	1	0
FMR1	chrX	147024813/G//A	nonsynonymous	1	0
OPN1LW	chrX	153421881/T//C	nonsynonymous	1	0
TAZ	chrX	153648414/C//G	nonsynonymous	1	0
SH3BP4	chr2	235951172/T//G	nonsynonymous	1	0
ZNF621	chr3	40574185/G//T	nonsynonymous	1	0
CADPS	chr3	62556616/A//C	synonymous	1	0
ZNF717	chr3	75786256/T//A	nonsynonymous	1	0
ZNF717	chr3	75786302/G//A	synonymous	1	0
ABI3BP	chr3	100583677/C//G	nonsynonymous	1	0
ABCC5	chr3	183670989/T//C	nonsynonymous	1	0
MMRN1	chr4	90856925/A//G	synonymous	1	0
EXOSC9	chr4	122724131/T//C	nonsynonymous	1	0
NPY5R	chr4	164272750/G//C	nonsynonymous	1	0
ADGRV1	chr5	90098646/C//T	nonsynonymous	1	0
LIX1	chr5	96443110/T//C	nonsynonymous	1	0
TEX43	chr5	125967482/C//T	nonsynonymous	1	0
FBN2	chr5	127697411/C//G	synonymous	1	0
ARAP3	chr5	141035825/C//G	nonsynonymous	1	0
ZBTB22	chr6	33283741/T//C	nonsynonymous	1	0
TBC1D22B	chr6	37259052/C//T	nonsynonymous	1	0
HNRNPA2B1	chr7	26240275/G//C	synonymous	1	0
ANLN	chr7	36483401/A//G	nonsynonymous	1	0
GLI3	chr7	42011928/T//C	synonymous	1	0
SRRT	chr7	100482572/G//A	nonsynonymous	1	0
GPR22	chr7	107115542/T//A	nonsynonymous	1	0
IFNK	chr9	27524701/G//C	nonsynonymous	1	0
EPB41L4B	chr9	111965968/A//G	nonsynonymous	1	0
CNTRL	chr9	123907515/T//A	synonymous	1	0
GPR158	chr10	25465104/G//T	nonsynonymous	1	0
UNC5B	chr10	73053609/C//A	synonymous	1	0
PHRF1	chr11	608430/C//T	nonsynonymous	1	0
DNHD1	chr11	6567241/T//C	nonsynonymous	1	0
RPS6KA4	chr11	64132915/C//T	nonsynonymous	1	0
GRM5	chr11	88337985/A//G	nonsynonymous	1	0
FRY	chr13	32676093/T//G	synonymous	1	0
FANCM	chr14	45624579/A//G	nonsynonymous	1	0
TDRD9	chr14	104473035/C//T	synonymous	1	0
PLA2G4E	chr15	42276652/G//T	synonymous	1	0
XYLT1	chr16	17235232/A//G	synonymous	1	0
MAP2K3	chr17	21202188/A//G	nonsynonymous	1	0
MRPL58	chr17	73008929/C//A	nonsynonymous	1	0
ZNF791	chr19	12739060/T//A	synonymous	1	0
TM6SF2	chr19	19381926/C//T	nonsynonymous	1	0
RYR1	chr19	38956963/C//A	synonymous	1	0
LTBP4	chr19	41133668/C//G	synonymous	1	0
OPA3	chr19	46056900/C//T	nonsynonymous	1	0
TULP2	chr19	49398635/G//A	nonsynonymous	1	0
PJA1	chrX	68382310/G//A	nonsynonymous	1	0
MED12	chrX	70349672/T//C	synonymous	1	0
DIAPH2	chrX	96167520/T//C	nonsynonymous	1	0
CSDE1	chr1	115272942/C//A	synonymous	0	1
OR2T5	chr1	248652011/A//T	nonsynonymous	0	1
OR2T5	chr1	248652023/G//A	nonsynonymous	0	1
HOXD1	chr2	177053948/T//A	nonsynonymous	0	1
OSGEPL1	chr2	190626202/A//G	synonymous	0	1
CEP63	chr3	134226050/T//G	nonsynonymous	0	1
CYTL1	chr4	5018892/T//C	synonymous	0	1
KDM1B	chr6	18197348/C//T	nonsynonymous	0	1
MUC22	chr6	30997806/C//G	nonsynonymous	0	1
MED23	chr6	131910626/A//G	synonymous	0	1
DYNC1H1	chr14	102452454/A//G	nonsynonymous	0	1
GRIN2A	chr16	9857813/C//A	nonsynonymous	0	1
NIPSNAP1	chr22	29954906/T//C	nonsynonymous	0	1
PER3	chr1	7887336/G//C	nonsynonymous	0	1
CX3CR1	chr3	39307718/T//C	nonsynonymous	0	1
OR5M1	chr11	56380203/T//C	nonsynonymous	0	1

CCNB1IP1	chr14	20779740/G//A	nonsynonymous	0	1
ZNF568	chr19	37441333/C//A	synonymous	0	1
TTLL10	chr1	1115467/C//T	nonsynonymous	1	0
ZBTB48	chr1	6647264/G//C	nonsynonymous	1	0
PADI2	chr1	17395715/C//A	nonsynonymous	1	0
UBR4	chr1	19489045/C//T	nonsynonymous	1	0
ADGRB2	chr1	32209913/A//G	nonsynonymous	1	0
CYP4A11	chr1	47403750/G//A	synonymous	1	0
ACOT11	chr1	55050405/C//A	nonsynonymous	1	0
ODF2L	chr1	86820526/T//C	nonsynonymous	1	0
HS2ST1	chr1	87570357/A//G	nonsynonymous	1	0
AMPD1	chr1	115238129/G//A	synonymous	1	0
PYGO2	chr1	154932153/C//T	nonsynonymous	1	0
NTRK1	chr1	156843452/C//T	nonsynonymous	1	0
DUSP27	chr1	167095682/C//T	synonymous	1	0
AVPR1B	chr1	206231124/T//C	synonymous	1	0
GREM2	chr1	240656784/C//T	synonymous	1	0
OR2G2	chr1	247752431/C//T	nonsynonymous	1	0
OR2G2	chr1	247752496/C//G	nonsynonymous	1	0
SNTG2	chr2	1079328/A//G	nonsynonymous	1	0
TPO	chr2	1520732/A//C	nonsynonymous	1	0
ADAM17	chr2	9637285/A//C	nonsynonymous	1	0
EFR3B	chr2	25351187/A//G	synonymous	1	0
CTNNA2	chr2	80136803/C//T	synonymous	1	0
SNRNP200	chr2	96957579/G//T	nonsynonymous	1	0
EPB41L5	chr2	120776787/A//G	nonsynonymous	1	0
THSD7B	chr2	138425360/T//C	synonymous	1	0
LRP1B	chr2	141533724/G//A	nonsynonymous	1	0
CCDC148	chr2	159201796/T//G	nonsynonymous	1	0
PLA2R1	chr2	160807955/T//C	nonsynonymous	1	0
RAPGEF4	chr2	173853468/A//G	synonymous	1	0
TTN	chr2	179414470/C//T	nonsynonymous	1	0
DNAH7	chr2	196753625/G//A	synonymous	1	0
MAP2	chr2	210561660/A//G	synonymous	1	0
	chr2	210569332/A//T	synonymous	1	0
AAMP	chr2	219134229/C//T	synonymous	1	0
COL4A4	chr2	227872117/G//A	nonsynonymous	1	0
HDLBP	chr2	242194476/T//A	nonsynonymous	1	0
NBEAL2	chr3	47049106/A//G	nonsynonymous	1	0
SETD2	chr3	47163990/T//C	synonymous	1	0
AMT	chr3	49457659/A//G	synonymous	1	0
PARP3	chr3	51978812/A//C	synonymous	1	0
FAM208A	chr3	56707752/T//C	synonymous	1	0
OR5AC2	chr3	97806645/T//C	nonsynonymous	1	0
CMSS1	chr3	99879320/C//A	nonsynonymous	1	0
ABI3BP	chr3	100513829/C//A	nonsynonymous	1	0
PIK3CB	chr3	138431047/T//C	synonymous	1	0
XRN1	chr3	142133009/C//T	nonsynonymous	1	0
U2SURP	chr3	142740327/A//G	nonsynonymous	1	0
CCNL1	chr3	156878012/C//T	synonymous	1	0
PCGF3	chr4	755200/A//C	nonsynonymous	1	0
BOD1L1	chr4	13589328/T//G	nonsynonymous	1	0
BOD1L1	chr4	13589346/T//G	nonsynonymous	1	0
LGI2	chr4	25005179/C//T	nonsynonymous	1	0
PCDH7	chr4	30725353/A//G	nonsynonymous	1	0
ATP8A1	chr4	42629055/T//A	nonsynonymous	1	0
GABRA2	chr4	46334668/G//A	synonymous	1	0
ATP10D	chr4	47574206/G//C	nonsynonymous	1	0
LRRC66	chr4	52860714/T//C	nonsynonymous	1	0
TMPRSS11D	chr4	68688041/A//T	synonymous	1	0
AMTN	chr4	71390682/A//G	synonymous	1	0
PLA2G12A	chr4	110639914/T//C	synonymous	1	0
	chr4	168041020/T//C	synonymous	1	0
CCDC110	chr4	186379510/A//G	nonsynonymous	1	0
CDH9	chr5	26902727/T//C	nonsynonymous	1	0
BDP1	chr5	70754572/C//T	nonsynonymous	1	0
VCAN	chr5	82817834/G//A	nonsynonymous	1	0
ARSK	chr5	94901828/G//T	nonsynonymous	1	0
LNPEP	chr5	96320863/A//T	nonsynonymous	1	0
PAM	chr5	102345459/G//T	nonsynonymous	1	0

PRR16	chr5	120022017/C//T	synonymous	1	0
MEGF10	chr5	126781158/T//C	nonsynonymous	1	0
SOWAHA	chr5	132150612/G//A	synonymous	1	0
HSPA9	chr5	137891798/T//C	synonymous	1	0
PSD2	chr5	139189332/G//A	nonsynonymous	1	0
PCDHB1	chr5	140431977/G//A	nonsynonymous	1	0
PCDHB5	chr5	140515332/T//G	nonsynonymous	1	0
PCDHB12	chr5	140588654/C//A	nonsynonymous	1	0
C1QTNF2	chr5	159781938/G//A	synonymous	1	0
DOCK2	chr5	169468131/C//A	synonymous	1	0
LCP2	chr5	169679462/T//A	nonsynonymous	1	0
HIVEP1	chr6	12123066/A//G	nonsynonymous	1	0
HCG4	chr6	29760311/C//A	synonymous	1	0
HCG4	chr6	29760518/C//A	synonymous	1	0
COL11A2	chr6	33145242/C//A	nonsynonymous	1	0
CPNE5	chr6	36712824/C//A	nonsynonymous	1	0
OPN5	chr6	47779418/C//A	nonsynonymous	1	0
MTFR2	chr6	136554516/T//C	nonsynonymous	1	0
SYNJ2	chr6	158507940/C//T	nonsynonymous	1	0
AGR2	chr7	16841013/A//T	synonymous	1	0
PCLO	chr7	82582241/C//A	nonsynonymous	1	0
SEMA3E	chr7	83119476/A//T	nonsynonymous	1	0
ADAM22	chr7	87792341/A//G	nonsynonymous	1	0
SAMD9L	chr7	92760874/G//C	nonsynonymous	1	0
SAMD9L	chr7	92765159/T//C	synonymous	1	0
ZAN	chr7	100395027/C//CTAG	nonsynonymous	1	0
MUC12	chr7	100648786/A//G	nonsynonymous	1	0
SRPK2	chr7	104782822/A//G	synonymous	1	0
GPR85	chr7	112724266/G//T	nonsynonymous	1	0
MGAM	chr7	141796157/A//G	nonsynonymous	1	0
ZNF467	chr7	149466217/C//A	nonsynonymous	1	0
CSMD1	chr8	3047554/C//A	nonsynonymous	1	0
CSMD1	chr8	3047593/G//C	nonsynonymous	1	0
MTUS1	chr8	17510934/C//T	synonymous	1	0
MOS	chr8	57025616/G//T	nonsynonymous	1	0
PRDM14	chr8	70978732/T//C	synonymous	1	0
RMDN1	chr8	87492552/C//T	nonsynonymous	1	0
RIMS2	chr8	104930668/G//A	nonsynonymous	1	0
MED30	chr8	118540915/C//A	nonsynonymous	1	0
EFR3A	chr8	132991138/A//G	synonymous	1	0
WISP1	chr8	134237712/C//T	synonymous	1	0
COL22A1	chr8	139610990/G//A	nonsynonymous	1	0
GPT	chr8	145729750/G//A	synonymous	1	0
SMARCA2	chr9	2029006/A//G	synonymous	1	0
JAK2	chr9	5054865/G//A	nonsynonymous	1	0
ADAMTSL1	chr9	18776928/G//T	nonsynonymous	1	0
TRPM6	chr9	77359055/G//A	nonsynonymous	1	0
TRPM6	chr9	77411773/T//G	nonsynonymous	1	0
VPS13A	chr9	79792630/G//T	nonsynonymous	1	0
ZNF510	chr9	99522421/T//A	nonsynonymous	1	0
LCN10	chr9	139633999/C//T	nonsynonymous	1	0
FAM208B	chr10	5790906/C//T	nonsynonymous	1	0
PRPF18	chr10	13658445/C//G	synonymous	1	0
NEBL	chr10	21185962/T//C	synonymous	1	0
PARD3	chr10	34625126/C//A	nonsynonymous	1	0
WDFY4	chr10	49933991/G//A	synonymous	1	0
STOX1	chr10	70646129/C//G	nonsynonymous	1	0
HTR7	chr10	92509161/T//C	nonsynonymous	1	0
HTR7	chr10	92617103/A//T	nonsynonymous	1	0
SLK	chr10	105763010/A//G	nonsynonymous	1	0
MUC6	chr11	1017691/C//G	nonsynonymous	1	0
HPX	chr11	6462177/C//G	nonsynonymous	1	0
OR2AG2	chr11	6790155/A//T	nonsynonymous	1	0
PPFIBP2	chr11	7674305/C//T	synonymous	1	0
OR5I1	chr11	55703386/A//T	nonsynonymous	1	0
OR4D9	chr11	59283296/G//A	nonsynonymous	1	0
MS4A14	chr11	60184320/G//A	nonsynonymous	1	0
UBXN1	chr11	62444314/G//T	nonsynonymous	1	0
PAAF1	chr11	73620561/GTGGTTCTTCTA//G	nonsynonymous	1	0
TENM4	chr11	78482154/C//G	nonsynonymous	1	0

ALKBH8	chr11	107393142/T//C	synonymous	1	0
ATM	chr11	108183157/G//T	nonsynonymous	1	0
ANKK1	chr11	113268050/C//T	nonsynonymous	1	0
USP2	chr11	119228856/T//A	synonymous	1	0
OR6M1	chr11	123676331/T//C	nonsynonymous	1	0
TAPBPL	chr12	6566726/C//CT	nonsynonymous	1	0
SPSB2	chr12	6981789/T//C	nonsynonymous	1	0
TAS2R31	chr12	11183722/A//C	nonsynonymous	1	0
GUCY2C	chr12	14775088/C//T	nonsynonymous	1	0
OR10A7	chr12	55615226/C//A	nonsynonymous	1	0
OR6C76	chr12	55820677/T//C	nonsynonymous	1	0
PPP1CC	chr12	111157952/A//G	synonymous	1	0
NOS1	chr12	117768223/T//C	nonsynonymous	1	0
RNF10	chr12	121001319/A//G	nonsynonymous	1	0
HCAR2	chr12	123187512/A//G	synonymous	1	0
RNF17	chr13	25439050/C//T	nonsynonymous	1	0
SLITRK1	chr13	84453725/C//A	nonsynonymous	1	0
SLITRK1	chr13	84454863/C//A	nonsynonymous	1	0
NPAS3	chr14	34263253/A//G	synonymous	1	0
ATG14	chr14	55836534/C//T	nonsynonymous	1	0
EXOC5	chr14	57676458/T//C	nonsynonymous	1	0
LTBP2	chr14	74983598/G//A	synonymous	1	0
NRXN3	chr14	79175990/G//A	nonsynonymous	1	0
DIO2	chr14	80669141/A//G	nonsynonymous	1	0
RORA	chr15	60792102/T//C	nonsynonymous	1	0
	chr15	72521976/G//A	synonymous	1	0
ABHD2	chr15	89738612/G//T	nonsynonymous	1	0
SULT1A1	chr16	28620214/T//C	synonymous	1	0
BRD7	chr16	50357576/A//C	nonsynonymous	1	0
RSPRY1	chr16	57238678/C//T	synonymous	1	0
HYDIN	chr16	71103167/T//C	synonymous	1	0
MYH2	chr17	10436622/G//A	synonymous	1	0
NARR	chr17	27044539/G//A	nonsynonymous	1	0
LHX1	chr17	35300206/C//A	synonymous	1	0
PLEKHH3	chr17	40825724/C//G	nonsynonymous	1	0
RUNDC3A	chr17	42390822/C//T	nonsynonymous	1	0
GFAP	chr17	42989040/C//G	synonymous	1	0
USP32	chr17	58348713/T//C	nonsynonymous	1	0
APOH	chr17	64216729/T//C	nonsynonymous	1	0
EPG5	chr18	43496514/C//T	synonymous	1	0
DCC	chr18	50918115/G//C	nonsynonymous	1	0
CHAF1A	chr19	4423354/A//G	nonsynonymous	1	0
INSR	chr19	7119582/C//A	synonymous	1	0
OR7A5	chr19	14938194/G//T	nonsynonymous	1	0
CYP4F11	chr19	16038291/C//T	nonsynonymous	1	0
B3GNT3	chr19	17923047/T//A	synonymous	1	0
ZNF492	chr19	22847644/G//A	synonymous	1	0
WDR87	chr19	38384894/A//G	synonymous	1	0
RYR1	chr19	38976446/C//T	synonymous	1	0
RYR1	chr19	38987167/G//A	nonsynonymous	1	0
CCDC155	chr19	49900953/G//C	nonsynonymous	1	0
ZNF649	chr19	52394965/T//C	nonsynonymous	1	0
ZNF880	chr19	52887531/T//A	nonsynonymous	1	0
NLRP4	chr19	56392946/A//T	nonsynonymous	1	0
ZSCAN18	chr19	58596045/C//G	synonymous	1	0
DEFB125	chr20	76658/C//T	nonsynonymous	1	0
CFAP61	chr20	20168646/G//T	nonsynonymous	1	0
CFAP61	chr20	20269464/G//A	nonsynonymous	1	0
POFUT1	chr20	30816144/A//G	synonymous	1	0
ARHGAP40	chr20	37258182/G//T	nonsynonymous	1	0
SLC9A8	chr20	48467365/C//G	nonsynonymous	1	0
DSCAM	chr21	42080397/T//C	nonsynonymous	1	0
P2RX6	chr22	21370241/C//G	nonsynonymous	1	0
TTC28	chr22	28559231/C//A	nonsynonymous	1	0
HUWE1	chrX	53579632/C//T	nonsynonymous	1	0
MAGEC2	chrX	141291506/C//A	nonsynonymous	1	0
AFF2	chrX	148044330/C//A	nonsynonymous	1	0
XPR1	chr1	180805748/G//A	nonsynonymous	0	1
TTN	chr2	179586761/C//T	synonymous	0	1
PCDHA3	chr5	140182767/C//T	nonsynonymous	0	1

PIGO	chr9	35092715/G//C	nonsynonymous	0	1
ADAMTS13	chr9	136310819/G//T	nonsynonymous	0	1
DYNC1H1	chr14	102500356/G//T	nonsynonymous	0	1
ZNF91	chr19	23542561/T//C	nonsynonymous	0	1
ZNF254	chr19	24310353/C//T	synonymous	0	1
PLXNB3	chrX	153040756/C//T	nonsynonymous	0	1
PRDM16	chr1	3347635/G//T	nonsynonymous	0	1
CEP85	chr1	26597578/C//T	nonsynonymous	0	1
CLCC1	chr1	109477428/T//C	nonsynonymous	0	1
NES	chr1	156645063/G//A	nonsynonymous	0	1
TOR1AIP1	chr1	179851726/G//A	nonsynonymous	0	1
EFEMP1	chr2	56144989/T//C	nonsynonymous	0	1
PLEKHM3	chr2	208795817/C//A	synonymous	0	1
MUC4	chr3	195501057/G//A	nonsynonymous	0	1
DCHS2	chr4	155253722/A//T	nonsynonymous	0	1
TTC37	chr5	94872524/A//G	nonsynonymous	0	1
SLCO6A1	chr5	101834355/C//T	nonsynonymous	0	1
PCDHA2	chr5	140175344/C//T	synonymous	0	1
PCLO	chr7	82585022/T//C	synonymous	0	1
GAS2	chr11	22707301/T//G	nonsynonymous	0	1
FOLH1	chr11	49194908/C//T	nonsynonymous	0	1
FOXR1	chr11	118850340/C//T	synonymous	0	1
OPCML	chr11	132527153/C//T	nonsynonymous	0	1
PDZRN4	chr12	41582283/C//A	nonsynonymous	0	1
TSPAN31	chr12	58140385/A//G	nonsynonymous	0	1
PKD1L3	chr16	72012159/T//C	nonsynonymous	0	1
SLFN12L	chr17	33802334/C//T	nonsynonymous	0	1
SLFN14	chr17	33879846/C//A	nonsynonymous	0	1
AZU1	chr19	827877/G//A	nonsynonymous	0	1
CLEC4M	chr19	7831670/G//A	nonsynonymous	0	1
CEACAM4	chr19	42132216/A//C	synonymous	0	1
LRRN4	chr20	6022756/T//A	nonsynonymous	0	1
DZANK1	chr20	18446024/C//A	synonymous	0	1
JADE3	chrX	46918164/C//A	synonymous	0	1
POU3F4	chrX	82763752/C//T	synonymous	0	1
B3GALT6	chr1	1168576/G//A	synonymous	1	0
MEGF6	chr1	3410666/C//A	synonymous	1	0
HNRNPCL2	chr1	13183534/A//G	synonymous	1	0
SYF2	chr1	25558947/T//C	nonsynonymous	1	0
COL16A1	chr1	32138199/G//T	synonymous	1	0
FAM167B	chr1	32713237/G//T	nonsynonymous	1	0
BSDC1	chr1	32849489/TC//T	nonsynonymous	1	0
ZSCAN20	chr1	33960288/A//G	nonsynonymous	1	0
CSMD2	chr1	34291330/G//A	nonsynonymous	1	0
COL24A1	chr1	86361738/C//A	nonsynonymous	1	0
CNN3	chr1	95368702/G//C	nonsynonymous	1	0
KCNA2	chr1	111147341/T//A	nonsynonymous	1	0
WDR3	chr1	118495019/A//G	nonsynonymous	1	0
SPAG17	chr1	118628611/G//T	nonsynonymous	1	0
TCHHL1	chr1	152059721/G//A	nonsynonymous	1	0
SPRR3	chr1	152975573/A//T	nonsynonymous	1	0
LRRC71	chr1	156894307/G//A	synonymous	1	0
ATP1A2	chr1	160106081/C//A	synonymous	1	0
DDR2	chr1	162735830/C//A	nonsynonymous	1	0
F5	chr1	169519201/A//G	synonymous	1	0
CENPL	chr1	173772481/T//C	nonsynonymous	1	0
TDRD5	chr1	179604848/A//T	nonsynonymous	1	0
XPR1	chr1	180794373/C//T	nonsynonymous	1	0
CAMSAP2	chr1	200818733/G//T	nonsynonymous	1	0
TMEM9	chr1	201104876/C//A	nonsynonymous	1	0
CAMK1G	chr1	209776562/C//A	synonymous	1	0
NEK2	chr1	211848764/C//A	nonsynonymous	1	0
FAM71A	chr1	212799866/T//C	synonymous	1	0
CENPF	chr1	214837081/C//T	nonsynonymous	1	0
KCNK2	chr1	215408381/G//C	nonsynonymous	1	0
USH2A	chr1	216595632/A//G	nonsynonymous	1	0
MIA3	chr1	222802551/G//T	synonymous	1	0
ENAH	chr1	225707084/C//A	synonymous	1	0
GJC2	chr1	228346780/G//T	synonymous	1	0
OBSCN	chr1	228402078/G//A	nonsynonymous	1	0

ZNF496	chr1	247464483/C//T	nonsynonymous	1	0
OR2L3	chr1	248224016/C//T	synonymous	1	0
TPO	chr2	1491590/C//A	synonymous	1	0
PXDN	chr2	1652828/G//T	synonymous	1	0
APOB	chr2	21236338/C//A	nonsynonymous	1	0
PRR30	chr2	27360134/G//T	nonsynonymous	1	0
GALNT14	chr2	31215831/C//A	nonsynonymous	1	0
GPR75	chr2	54081915/T//A	synonymous	1	0
CLHC1	chr2	55436968/C//A	nonsynonymous	1	0
NAT8B	chr2	73928402/C//A	nonsynonymous	1	0
ASTL	chr2	96799702/A//G	nonsynonymous	1	0
CNNM3	chr2	97494360/C//A	nonsynonymous	1	0
THSD7B	chr2	138169422/C//A	nonsynonymous	1	0
GALNT13	chr2	155115538/C//G	nonsynonymous	1	0
GALNT5	chr2	158140951/A//T	nonsynonymous	1	0
SCN2A	chr2	166246078/T//A	nonsynonymous	1	0
TTN	chr2	179594202/C//A	synonymous	1	0
ZNF804A	chr2	185801972/C//A	nonsynonymous	1	0
PGAP1	chr2	197767457/G//A	nonsynonymous	1	0
BOLL	chr2	198593260/A//G	synonymous	1	0
	chr2	198650709/G//T	synonymous	1	0
NBEAL1	chr2	204067468/A//T	nonsynonymous	1	0
IHH	chr2	219922284/C//A	nonsynonymous	1	0
NYAP2	chr2	226447297/C//A	synonymous	1	0
SPHKAP	chr2	228886510/C//A	nonsynonymous	1	0
SNED1	chr2	242005004/C//A	synonymous	1	0
GAL3ST2	chr2	242743699/C//A	synonymous	1	0
FANCD2	chr3	10130162/G//T	nonsynonymous	1	0
FBLN2	chr3	13659600/G//T	nonsynonymous	1	0
CLEC3B	chr3	45077295/C//G	nonsynonymous	1	0
RTP3	chr3	46541939/G//T	nonsynonymous	1	0
ATRIP	chr3	48501972/G//T	nonsynonymous	1	0
DOCK3	chr3	51376364/A//T	nonsynonymous	1	0
ERC2	chr3	56468430/C//T	synonymous	1	0
PRICKLE2	chr3	64133312/C//G	nonsynonymous	1	0
CNTN3	chr3	74383939/C//A	nonsynonymous	1	0
CNTN3	chr3	74384027/C//A	nonsynonymous	1	0
HTR1F	chr3	88040781/C//A	synonymous	1	0
EPHA6	chr3	96706732/C//A	nonsynonymous	1	0
ABI3BP	chr3	100568939/G//T	nonsynonymous	1	0
SEMA5B	chr3	122630811/C//T	nonsynonymous	1	0
SEMA5B	chr3	122632854/G//T	synonymous	1	0
KALRN	chr3	123813742/C//G	nonsynonymous	1	0
COL6A6	chr3	130281926/G//T	nonsynonymous	1	0
ATP2C1	chr3	130718450/T//C	nonsynonymous	1	0
TF	chr3	133485155/C//T	nonsynonymous	1	0
FOXL2	chr3	138664355/G//T	synonymous	1	0
FOXL2NB	chr3	138669304/C//T	nonsynonymous	1	0
MED12L	chr3	151129089/G//C	nonsynonymous	1	0
SLC7A14	chr3	170219111/C//A	nonsynonymous	1	0
TNIK	chr3	170906583/C//A	nonsynonymous	1	0
B3GNT5	chr3	182988663/A//T	synonymous	1	0
MCF2L2	chr3	183056683/G//T	nonsynonymous	1	0
CHRD	chr3	184099522/C//T	nonsynonymous	1	0
APOD	chr3	195300841/T//C	nonsynonymous	1	0
JAKMIP1	chr4	6107341/C//A	synonymous	1	0
BOD1L1	chr4	13606405/C//G	nonsynonymous	1	0
ADGRA3	chr4	22446639/C//A	nonsynonymous	1	0
DCAF4L1	chr4	41984902/A//T	nonsynonymous	1	0
UGT2B15	chr4	69513029/G//T	synonymous	1	0
DSPP	chr4	88533335/G//T	nonsynonymous	1	0
ADH6	chr4	100131284/G//T	nonsynonymous	1	0
ETNPPL	chr4	109675784/C//A	nonsynonymous	1	0
ANK2	chr4	114279225/G//T	nonsynonymous	1	0
UGT8	chr4	115597216/G//T	nonsynonymous	1	0
BBS12	chr4	123663923/G//C	synonymous	1	0
GATB	chr4	152592353/C//T	synonymous	1	0
FGA	chr4	155507433/G//T	nonsynonymous	1	0
NPY2R	chr4	156135819/G//C	nonsynonymous	1	0
GRIA2	chr4	158257666/C//A	synonymous	1	0

PLEKHG4B	chr5	156333/C//G	synonymous	1	0
CDH18	chr5	19747262/G//T	nonsynonymous	1	0
ADAMTS12	chr5	33576198/G//T	synonymous	1	0
ADAMTS12	chr5	33576611/G//C	nonsynonymous	1	0
MROH2B	chr5	41065442/T//C	nonsynonymous	1	0
HCN1	chr5	45396723/G//A	synonymous	1	0
ZSWIM6	chr5	60837770/G//T	synonymous	1	0
HTR1A	chr5	63256861/G//T	nonsynonymous	1	0
MAP1B	chr5	71491674/C//T	nonsynonymous	1	0
VCAN	chr5	82833085/C//A	synonymous	1	0
PJA2	chr5	108680412/G//A	nonsynonymous	1	0
WDR36	chr5	110462709/A//T	synonymous	1	0
TRIM36	chr5	114472700/T//A	nonsynonymous	1	0
CDO1	chr5	115152025/C//A	nonsynonymous	1	0
DMXL1	chr5	118506843/A//G	synonymous	1	0
FTMT	chr5	121188080/G//T	nonsynonymous	1	0
TXNDC15	chr5	134210133/G//T	nonsynonymous	1	0
KDM3B	chr5	137721930/G//A	nonsynonymous	1	0
KDM3B	chr5	137727794/G//T	nonsynonymous	1	0
HSPA9	chr5	137895563/T//A	nonsynonymous	1	0
CXXC5	chr5	139060567/G//C	nonsynonymous	1	0
NRG2	chr5	139235313/C//A	nonsynonymous	1	0
PCDHB14	chr5	140603519/G//T	nonsynonymous	1	0
PCDHGA3	chr5	140724198/C//A	nonsynonymous	1	0
PCDHGA8	chr5	140773365/G//A	nonsynonymous	1	0
PCDHGA8	chr5	140773978/C//A	nonsynonymous	1	0
FAM71B	chr5	156589774/G//T	nonsynonymous	1	0
TENM2	chr5	167653146/C//A	nonsynonymous	1	0
TENM2	chr5	167675203/C//A	nonsynonymous	1	0
DOCK2	chr5	169081448/C//A	nonsynonymous	1	0
DOCK2	chr5	169108886/G//T	synonymous	1	0
DOCK2	chr5	169435558/C//G	nonsynonymous	1	0
DOCK2	chr5	169472834/C//A	synonymous	1	0
SH3PXD2B	chr5	171765885/T//C	nonsynonymous	1	0
CDHR2	chr5	176002745/T//A	nonsynonymous	1	0
GPRIN1	chr5	176026433/C//A	nonsynonymous	1	0
HK3	chr5	176317818/C//T	synonymous	1	0
ZNF879	chr5	178460490/G//C	nonsynonymous	1	0
ADAMTS2	chr5	178770995/C//T	nonsynonymous	1	0
OR12D3	chr6	29343016/C//A	nonsynonymous	1	0
HLA-G	chr6	29796354/C//A	nonsynonymous	1	0
MUC22	chr6	31000298/C//A	synonymous	1	0
UHRF1BP1	chr6	34827112/G//A	synonymous	1	0
TFEB	chr6	41652628/C//A	synonymous	1	0
FRS3	chr6	41740673/C//A	nonsynonymous	1	0
XPO5	chr6	43517301/C//A	synonymous	1	0
AARS2	chr6	44270880/C//A	synonymous	1	0
TDRD6	chr6	46658964/C//T	synonymous	1	0
ADGRF5	chr6	46826584/C//A	nonsynonymous	1	0
OPN5	chr6	47759558/A//G	nonsynonymous	1	0
IL17A	chr6	52054027/G//T	synonymous	1	0
GSTA3	chr6	52764805/G//T	nonsynonymous	1	0
WISP3	chr6	112375615/C//A	synonymous	1	0
DSE	chr6	116720731/G//T	nonsynonymous	1	0
TRDN	chr6	123786098/G//C	nonsynonymous	1	0
TAAR6	chr6	132892429/A//T	nonsynonymous	1	0
VNN1	chr6	133004345/G//T	synonymous	1	0
MTFR2	chr6	136562750/C//A	nonsynonymous	1	0
THBS2	chr6	169632298/G//C	synonymous	1	0
THBS2	chr6	169648685/C//T	nonsynonymous	1	0
ELFN1	chr7	1785692/C//A	nonsynonymous	1	0
HDAC9	chr7	18868847/C//A	synonymous	1	0
RAPGEF5	chr7	22200155/G//A	nonsynonymous	1	0
NEUROD6	chr7	31378020/C//A	nonsynonymous	1	0
NPSR1	chr7	34698068/C//A	nonsynonymous	1	0
TBX20	chr7	35288377/C//A	nonsynonymous	1	0
TBX20	chr7	35288392/C//A	nonsynonymous	1	0
AOAH	chr7	36671674/C//A	synonymous	1	0
VPS41	chr7	38937685/C//A	synonymous	1	0
MYL7	chr7	44180002/T//A	nonsynonymous	1	0

CAMK2B	chr7	44260498/G//T	synonymous	1	0
ADCY1	chr7	45744109/A//G	synonymous	1	0
C7orf72	chr7	50198773/G//C	synonymous	1	0
GRB10	chr7	50672070/C//A	nonsynonymous	1	0
POM121L12	chr7	53103614/C//A	nonsynonymous	1	0
EGFR	chr7	55248998/A//ATGGCCAGCG	nonsynonymous	1	0
PCLO	chr7	82451939/C//A	nonsynonymous	1	0
GATAD1	chr7	92085804/G//C	nonsynonymous	1	0
COL1A2	chr7	94039106/C//A	synonymous	1	0
LMTK2	chr7	97822091/C//T	nonsynonymous	1	0
ZAN	chr7	100385618/G//T	nonsynonymous	1	0
PPP1R3A	chr7	113519710/T//A	synonymous	1	0
FOXP2	chr7	114304481/C//A	nonsynonymous	1	0
CPED1	chr7	120765938/G//A	nonsynonymous	1	0
DPP6	chr7	154664333/G//T	nonsynonymous	1	0
DLGAP2	chr8	1616573/C//T	nonsynonymous	1	0
CSMD1	chr8	2796150/A//T	nonsynonymous	1	0
CSMD1	chr8	3059189/A//T	nonsynonymous	1	0
CSMD1	chr8	3072084/G//T	nonsynonymous	1	0
MSR1	chr8	16012654/C//A	nonsynonymous	1	0
CLVS1	chr8	62212477/A//G	nonsynonymous	1	0
MSC-AS1	chr8	72755767/A//G	synonymous	1	0
ZFHX4	chr8	77618916/T//A	synonymous	1	0
ZFHX4	chr8	77767187/G//T	nonsynonymous	1	0
CNGB3	chr8	87588156/G//T	nonsynonymous	1	0
NECAB1	chr8	91836996/T//C	nonsynonymous	1	0
SLC26A7	chr8	92307752/G//C	synonymous	1	0
ERICH5	chr8	99101434/A//G	synonymous	1	0
UBR5	chr8	103316283/G//A	nonsynonymous	1	0
TMEM74	chr8	109796802/A//G	nonsynonymous	1	0
CSMD3	chr8	113842019/C//A	nonsynonymous	1	0
TRPS1	chr8	116426388/G//T	nonsynonymous	1	0
SLC30A8	chr8	118174072/G//T	nonsynonymous	1	0
FER1L6	chr8	125022918/C//A	nonsynonymous	1	0
FER1L6	chr8	125088524/G//T	nonsynonymous	1	0
PLEC	chr8	144998364/C//A	synonymous	1	0
KCNV2	chr9	2718387/C//T	synonymous	1	0
TEK	chr9	27185489/G//C	nonsynonymous	1	0
KIF24	chr9	34306440/C//A	nonsynonymous	1	0
TRPM3	chr9	73736142/G//T	synonymous	1	0
PRUNE2	chr9	79320709/C//G	nonsynonymous	1	0
SPATA31E1	chr9	90502985/C//A	nonsynonymous	1	0
WNK2	chr9	96054798/C//A	nonsynonymous	1	0
NUTM2F	chr9	97084497/G//T	nonsynonymous	1	0
PTCH1	chr9	98221907/G//C	nonsynonymous	1	0
COL15A1	chr9	101778409/G//T	synonymous	1	0
ACTL7A	chr9	111624863/C//A	synonymous	1	0
ASTN2	chr9	119976935/G//C	nonsynonymous	1	0
BRINP1	chr9	122075437/G//A	nonsynonymous	1	0
OR1L8	chr9	125330043/G//A	synonymous	1	0
LRRC8A	chr9	131669842/G//T	synonymous	1	0
GBGT1	chr9	136029461/G//T	synonymous	1	0
SLC2A6	chr9	136338284/G//T	nonsynonymous	1	0
COL5A1	chr9	137619128/T//C	nonsynonymous	1	0
ITIH5	chr10	7605124/C//A	synonymous	1	0
GATA3	chr10	8097754/G//C	nonsynonymous	1	0
TMEM72	chr10	45430574/C//G	nonsynonymous	1	0
ARHGAP22	chr10	49791062/C//A	nonsynonymous	1	0
KCNMA1	chr10	78839244/T//A	nonsynonymous	1	0
PPP1R3C	chr10	93389963/G//T	synonymous	1	0
DNMBP	chr10	101636846/C//G	synonymous	1	0
PDCD11	chr10	105182750/G//T	nonsynonymous	1	0
PNLIPRP3	chr10	118196365/C//A	synonymous	1	0
RGS10	chr10	121286823/A//G	nonsynonymous	1	0
ADAM12	chr10	127786997/C//A	synonymous	1	0
STIM1	chr11	4091332/C//T	synonymous	1	0
OR52K2	chr11	4471089/C//A	nonsynonymous	1	0
OR2AG2	chr11	6789744/A//T	nonsynonymous	1	0
OVCH2	chr11	7723358/C//T	nonsynonymous	1	0
TUB	chr11	8122512/T//C	nonsynonymous	1	0

USH1C	chr11	17547964/G//T	nonsynonymous	1	0
LUZP2	chr11	24927546/T//A	nonsynonymous	1	0
CAT	chr11	34489905/A//T	nonsynonymous	1	0
APLNR	chr11	57003672/C//A	synonymous	1	0
OR4D9	chr11	59282539/G//T	nonsynonymous	1	0
GIF	chr11	59609965/G//T	synonymous	1	0
MS4A2	chr11	59857230/C//A	nonsynonymous	1	0
MYRF	chr11	61541451/C//A	synonymous	1	0
ATG2A	chr11	64665380/G//C	nonsynonymous	1	0
FGF3	chr11	69625346/G//T	synonymous	1	0
C2CD3	chr11	73795993/C//T	synonymous	1	0
TYR	chr11	88911653/T//C	nonsynonymous	1	0
C11orf88	chr11	111386248/C//A	nonsynonymous	1	0
DIXDC1	chr11	111863237/C//G	nonsynonymous	1	0
ANKK1	chr11	113267975/T//A	nonsynonymous	1	0
CEP164	chr11	117233148/A//T	nonsynonymous	1	0
RNF26	chr11	119206828/G//T	synonymous	1	0
C11orf63	chr11	122774743/A//C	nonsynonymous	1	0
C11orf63	chr11	122805772/A//T	nonsynonymous	1	0
ZNF202	chr11	123601558/C//A	nonsynonymous	1	0
OR10S1	chr11	123847817/G//A	synonymous	1	0
ROBO4	chr11	124764231/G//T	nonsynonymous	1	0
KCNJ5	chr11	128781541/G//T	nonsynonymous	1	0
ADAMTS15	chr11	130319347/G//T	nonsynonymous	1	0
DCP1B	chr12	2058395/C//A	nonsynonymous	1	0
VWF	chr12	6131201/C//A	nonsynonymous	1	0
CDKN1B	chr12	12871184/G//T	synonymous	1	0
SLCO1A2	chr12	21459918/C//A	nonsynonymous	1	0
FAR2	chr12	29423556/G//T	nonsynonymous	1	0
OVCH1	chr12	29580528/A//G	synonymous	1	0
H3F3C	chr12	31944542/A//T	synonymous	1	0
ARID2	chr12	46245192/C//T	nonsynonymous	1	0
KRT6A	chr12	52882211/G//C	nonsynonymous	1	0
MON2	chr12	62974125/C//T	nonsynonymous	1	0
OTOGL	chr12	80661089/C//A	nonsynonymous	1	0
PTPRQ	chr12	80936104/G//T	nonsynonymous	1	0
MYF5	chr12	81110965/G//C	synonymous	1	0
AMDHD1	chr12	96359480/G//A	nonsynonymous	1	0
PAH	chr12	103232978/C//T	nonsynonymous	1	0
WASHC4	chr12	105501516/G//A	synonymous	1	0
ASCL4	chr12	108169355/C//A	nonsynonymous	1	0
NOS1	chr12	117665296/G//T	nonsynonymous	1	0
SLC15A4	chr12	129283880/C//A	synonymous	1	0
RIMBP2	chr12	130921734/G//T	nonsynonymous	1	0
SLC46A3	chr13	29278236/C//A	nonsynonymous	1	0
SOHLH2	chr13	36747888/G//A	nonsynonymous	1	0
LACC1	chr13	44462933/G//T	synonymous	1	0
GTF2F2	chr13	45857588/G//T	nonsynonymous	1	0
PCDH8	chr13	53421120/C//A	synonymous	1	0
SLITRK1	chr13	84454647/G//T	nonsynonymous	1	0
NALCN	chr13	101844397/C//A	nonsynonymous	1	0
OR11G2	chr14	20666187/T//A	synonymous	1	0
OR11H6	chr14	20692552/C//A	synonymous	1	0
KLHL33	chr14	20898240/G//T	nonsynonymous	1	0
SLC22A17	chr14	23815903/C//A	nonsynonymous	1	0
JPH4	chr14	24040150/C//A	nonsynonymous	1	0
NFATC4	chr14	24838754/G//T	nonsynonymous	1	0
RALGAPA1	chr14	36190936/C//A	nonsynonymous	1	0
KCNH5	chr14	63174550/G//T	synonymous	1	0
RIN3	chr14	93125603/G//T	synonymous	1	0
UNC79	chr14	94158105/T//A	nonsynonymous	1	0
AHNAK2	chr14	105417672/C//A	nonsynonymous	1	0
APBA2	chr15	29393893/G//A	nonsynonymous	1	0
TRPM1	chr15	31323263/G//T	nonsynonymous	1	0
TCF12	chr15	57526270/C//T	nonsynonymous	1	0
CILP	chr15	65489210/G//C	synonymous	1	0
ADAMTS7	chr15	79066539/G//T	nonsynonymous	1	0
AP3B2	chr15	83328734/T//A	synonymous	1	0
IQGAP1	chr15	90996362/A//G	nonsynonymous	1	0
BLM	chr15	91354534/A//G	nonsynonymous	1	0

CASKIN1	chr16	2235383/G//T	nonsynonymous	1	0
ANKS4B	chr16	21261756/G//A	nonsynonymous	1	0
IL21R	chr16	27457321/C//G	synonymous	1	0
SPN	chr16	29675497/C//A	nonsynonymous	1	0
ITGAD	chr16	31424208/C//T	nonsynonymous	1	0
SNX20	chr16	50711300/A//G	synonymous	1	0
IRX6	chr16	55360410/A//T	nonsynonymous	1	0
PRMT7	chr16	68349877/G//T	synonymous	1	0
PLCG2	chr16	81927380/G//T	nonsynonymous	1	0
GSG2	chr17	3627592/G//T	synonymous	1	0
MINK1	chr17	4795446/G//A	synonymous	1	0
GAS7	chr17	9830049/C//A	nonsynonymous	1	0
MYH8	chr17	10301821/T//A	nonsynonymous	1	0
MYH3	chr17	10544395/T//C	synonymous	1	0
DNAH9	chr17	11535997/C//G	nonsynonymous	1	0
TRPV2	chr17	16332275/C//G	nonsynonymous	1	0
DRC3	chr17	17891393/G//A	nonsynonymous	1	0
ZNF286B	chr17	18565401/C//A	nonsynonymous	1	0
MTRNR2L1	chr17	22023531/G//T	synonymous	1	0
PIPOX	chr17	27371958/C//A	synonymous	1	0
KRT23	chr17	39092544/G//T	synonymous	1	0
GHDC	chr17	40344570/G//A	nonsynonymous	1	0
CNTNAP1	chr17	40842952/A//G	nonsynonymous	1	0
RAMP2	chr17	40914638/G//T	nonsynonymous	1	0
CCDC43	chr17	42767139/G//T	synonymous	1	0
MAPT	chr17	44051821/C//A	synonymous	1	0
KANSL1	chr17	44171997/C//A	nonsynonymous	1	0
KIF2B	chr17	51901193/G//T	nonsynonymous	1	0
AKAP1	chr17	55191953/G//C	nonsynonymous	1	0
MTMR4	chr17	56572961/C//A	nonsynonymous	1	0
SMG8	chr17	57288473/A//T	nonsynonymous	1	0
MED13	chr17	60038953/G//T	nonsynonymous	1	0
RPTOR	chr17	78820305/A//T	synonymous	1	0
NDC80	chr18	2573036/G//A	nonsynonymous	1	0
ANKRD30B	chr18	14799227/C//A	synonymous	1	0
GALNT1	chr18	33289771/G//T	synonymous	1	0
ATP8B1	chr18	55328516/C//A	nonsynonymous	1	0
THEG	chr19	362382/G//T	nonsynonymous	1	0
MISP	chr19	757525/C//T	synonymous	1	0
ATCAY	chr19	3924661/T//C	synonymous	1	0
FUT6	chr19	5832095/A//T	nonsynonymous	1	0
ACTL9	chr19	8807816/G//T	nonsynonymous	1	0
MUC16	chr19	9004911/G//T	nonsynonymous	1	0
MUC16	chr19	9046298/G//T	nonsynonymous	1	0
OR1M1	chr19	9204092/C//G	nonsynonymous	1	0
ZNF699	chr19	9407093/G//A	synonymous	1	0
MRPL4	chr19	10363327/G//A	synonymous	1	0
MRPL4	chr19	10363330/C//T	synonymous	1	0
ZNF763	chr19	12089637/C//T	nonsynonymous	1	0
C19orf43	chr19	12845180/C//G	nonsynonymous	1	0
AKAP8	chr19	15483039/G//A	synonymous	1	0
ZNF429	chr19	21720133/T//A	nonsynonymous	1	0
ZNF91	chr19	23542898/A//G	synonymous	1	0
TSHZ3	chr19	31769962/G//T	nonsynonymous	1	0
APLP1	chr19	36362844/A//T	synonymous	1	0
LRFN3	chr19	36430514/G//T	nonsynonymous	1	0
RYR1	chr19	38958415/G//T	nonsynonymous	1	0
RYR1	chr19	38987525/C//A	nonsynonymous	1	0
PPP1R13L	chr19	45900309/C//A	nonsynonymous	1	0
HIF3A	chr19	46823803/C//A	nonsynonymous	1	0
PLEKHA4	chr19	49362878/G//T	synonymous	1	0
SCAF1	chr19	50156939/G//T	nonsynonymous	1	0
SYT3	chr19	51128491/G//T	nonsynonymous	1	0
SYT3	chr19	51140583/G//T	nonsynonymous	1	0
KLK8	chr19	51501014/G//T	nonsynonymous	1	0
SIGLECL1	chr19	51768706/C//A	nonsynonymous	1	0
SIGLEC6	chr19	52034144/C//A	nonsynonymous	1	0
ERVV-2	chr19	53554186/G//A	synonymous	1	0
DPRX	chr19	54140044/C//A	synonymous	1	0
MBOAT7	chr19	54684757/C//A	nonsynonymous	1	0

LILRB5	chr19	54760477/G//T	nonsynonymous	1	0
BRSK1	chr19	55814091/G//T	nonsynonymous	1	0
TGM3	chr20	2312900/C//T	nonsynonymous	1	0
LZTS3	chr20	3146925/G//A	nonsynonymous	1	0
ACSS2	chr20	33470606/G//T	nonsynonymous	1	0
SAMHD1	chr20	35580166/T//A	synonymous	1	0
SEMG1	chr20	43836863/G//T	nonsynonymous	1	0
ZNF335	chr20	44580854/G//A	nonsynonymous	1	0
PREX1	chr20	47317331/G//A	nonsynonymous	1	0
KCNB1	chr20	47990987/C//A	nonsynonymous	1	0
NFATC2	chr20	50140068/C//A	nonsynonymous	1	0
PCK1	chr20	56136600/A//G	nonsynonymous	1	0
DIDO1	chr20	61512589/C//A	synonymous	1	0
BIRC7	chr20	61867557/A//T	nonsynonymous	1	0
COL20A1	chr20	61959838/C//T	nonsynonymous	1	0
ZGPAT	chr20	62365043/G//T	nonsynonymous	1	0
TIAM1	chr21	32582473/C//A	nonsynonymous	1	0
SON	chr21	34925936/A//G	nonsynonymous	1	0
PFKL	chr21	45738454/G//T	synonymous	1	0
COL6A2	chr21	47538593/G//C	synonymous	1	0
AIFM3	chr22	21331055/G//A	synonymous	1	0
SLC7A4	chr22	21384612/G//T	synonymous	1	0
MYO18B	chr22	26228924/C//A	nonsynonymous	1	0
SEZ6L	chr22	26688690/C//A	nonsynonymous	1	0
TOM1	chr22	35723381/G//T	nonsynonymous	1	0
CACNA1I	chr22	40055701/G//A	nonsynonymous	1	0
EFCAB6	chr22	44168906/C//A	nonsynonymous	1	0
PHF21B	chr22	45291935/A//T	nonsynonymous	1	0
ARHGAP6	chrX	11206969/C//G	nonsynonymous	1	0
GRPR	chrX	16170562/G//A	nonsynonymous	1	0
SSX3	chrX	48214690/A//C	synonymous	1	0
SLC38A5	chrX	48325429/G//C	nonsynonymous	1	0
HEPH	chrX	65393529/C//A	nonsynonymous	1	0
HEPH	chrX	65478712/G//T	nonsynonymous	1	0
OTUD6A	chrX	69282407/A//G	nonsynonymous	1	0
PLP1	chrX	103042768/G//T	synonymous	1	0
COL4A5	chrX	107824257/T//A	synonymous	1	0
GUCY2F	chrX	108625425/G//A	synonymous	1	0
WDR44	chrX	117576266/A//T	synonymous	1	0
CXorf56	chrX	118699328/G//A	synonymous	1	0
SH2D1A	chrX	123505280/T//A	synonymous	1	0
SPANXN1	chrX	144337272/G//T	nonsynonymous	1	0
RENBP	chrX	153208443/G//T	nonsynonymous	1	0
PLEKHN1	chr1	908396/C//T	synonymous	1	0
ZSCAN20	chr1	33958975/C//T	nonsynonymous	1	0
TESK2	chr1	45810729/C//T	nonsynonymous	1	0
DOCK7	chr1	62958451/CCTT//C	nonsynonymous	1	0
WDR77	chr1	111985985/C//T	synonymous	1	0
MAGI3	chr1	114193810/C//T	nonsynonymous	1	0
ARHGAP30	chr1	161022528/C//T	nonsynonymous	1	0
NFASC	chr1	204985634/G//A	synonymous	1	0
CENPF	chr1	214814779/G//A	nonsynonymous	1	0
ALMS1	chr2	73651768/G//A	synonymous	1	0
RFX8	chr2	102029526/C//T	nonsynonymous	1	0
DPP10	chr2	116525941/A//G	synonymous	1	0
NRP2	chr2	206628626/G//A	nonsynonymous	1	0
OR6B3	chr2	240984556/C//T	nonsynonymous	1	0
COPS9	chr2	241070449/C//T	synonymous	1	0
PLXNB1	chr3	48461058/C//G	nonsynonymous	1	0
HTR3C	chr3	183778014/G//A	nonsynonymous	1	0
GBA3	chr4	22749270/G//A	nonsynonymous	1	0
CDH9	chr5	26881414/G//A	nonsynonymous	1	0
DHFR	chr5	79933820/G//A	nonsynonymous	1	0
NRG2	chr5	139231286/G//A	nonsynonymous	1	0
CD14	chr5	140012308/C//T	synonymous	1	0
ABLIM3	chr5	148620240/G//T	synonymous	1	0
CAMK2A	chr5	149630277/C//T	nonsynonymous	1	0
PHACTR1	chr6	13287430/A//T	synonymous	1	0
KIF13A	chr6	17852181/C//A	synonymous	1	0
RIMS1	chr6	72806843/G//A	nonsynonymous	1	0

FAM20C	chr7	286403/C//G	nonsynonymous	1	0
ABCB1	chr7	87138606/G//A	synonymous	1	0
POT1	chr7	124510981/C//T	nonsynonymous	1	0
HR	chr8	21977371/G//A	nonsynonymous	1	0
DCAF4L2	chr8	88886173/G//A	synonymous	1	0
ACTL7B	chr9	111617548/G//A	synonymous	1	0
EHF	chr11	34668200/C//G	synonymous	1	0
OR5D13	chr11	55541762/G//A	synonymous	1	0
SART1	chr11	65743897/G//A	nonsynonymous	1	0
CCDC89	chr11	85396627/A//G	nonsynonymous	1	0
NCAPD3	chr11	134029786/C//T	nonsynonymous	1	0
CD163	chr12	7640095/C//G	nonsynonymous	1	0
BICD1	chr12	32487463/C//T	nonsynonymous	1	0
TRPC4	chr13	38237650/C//A	nonsynonymous	1	0
TMCO3	chr13	114154294/G//T	nonsynonymous	1	0
HIF1A	chr14	62203755/GAACCTGATGCTTT//G	nonsynonymous	1	0
PLEKHD1	chr14	69994592/C//T	nonsynonymous	1	0
MAPKBP1	chr15	42105522/G//A	synonymous	1	0
SLC24A1	chr15	65917840/C//T	synonymous	1	0
CHST6	chr16	75513265/C//T	synonymous	1	0
ADAD2	chr16	84224938/G//A	synonymous	1	0
OR1I1	chr19	15198246/G//A	nonsynonymous	1	0
ATP13A1	chr19	19758447/C//T	synonymous	1	0
TMC2	chr20	2616588/C//A	nonsynonymous	1	0
GFRA4	chr20	3644023/C//T	synonymous	1	0
MYT1	chr20	62830228/A//G	nonsynonymous	1	0
TTC3	chr21	38558036/A//G	nonsynonymous	1	0
CACNA1I	chr22	40075108/G//A	synonymous	1	0
MAGEC3	chrX	140969248/T//A	nonsynonymous	1	0
RERE	chr1	8418472/C//CCT	nonsynonymous	0	1
LYPLA2	chr1	24121029/T//C	nonsynonymous	0	1
TNP1	chr2	217724771/A//C	synonymous	0	1
HJURP	chr2	234746195/A//G	synonymous	0	1
NME6	chr3	48342942/G//C	synonymous	0	1
TMCO6	chr5	140023477/T//C	nonsynonymous	0	1
NAALADL1	chr11	64825889/C//G	synonymous	0	1
ACP7	chr19	39600831/C//G	synonymous	0	1
L1CAM	chrX	153128826/T//C	nonsynonymous	0	1
CAMTA1	chr1	7725220/C//T	synonymous	1	0
ARHGEF10L	chr1	17964464/C//T	nonsynonymous	1	0
PAX7	chr1	18960951/C//T	synonymous	1	0
MEF2D	chr1	156438646/T//C	synonymous	1	0
PEAR1	chr1	156882292/C//T	nonsynonymous	1	0
KCNJ9	chr1	160057361/C//A	nonsynonymous	1	0
PAPPA2	chr1	176708793/C//A	nonsynonymous	1	0
DUSP10	chr1	221875658/C//A	synonymous	1	0
ACTA1	chr1	229568112/G//T	nonsynonymous	1	0
CTNNA2	chr2	80646724/G//T	nonsynonymous	1	0
SUCLG1	chr2	84668213/T//C	nonsynonymous	1	0
ACOXL	chr2	111542370/C//T	nonsynonymous	1	0
POTEE	chr2	132021711/G//T	nonsynonymous	1	0
LRP1B	chr2	141208157/G//C	nonsynonymous	1	0
LRP1B	chr2	141208166/C//A	nonsynonymous	1	0
LRP1B	chr2	141819639/G//C	nonsynonymous	1	0
KIF5C	chr2	149838075/G//C	synonymous	1	0
HOXD13	chr2	176958334/A//G	nonsynonymous	1	0
SH3BP4	chr2	235950254/G//C	nonsynonymous	1	0
RNPEPL1	chr2	241516028/G//T	synonymous	1	0
DHX30	chr3	47890762/C//A	nonsynonymous	1	0
CCDC71	chr3	49200313/C//A	synonymous	1	0
STXBP5L	chr3	120959337/A//T	synonymous	1	0
PHC3	chr3	169840435/G//T	nonsynonymous	1	0
NAT8L	chr4	2062822/C//G	synonymous	1	0
SH3BP2	chr4	2834139/G//T	nonsynonymous	1	0
CEP135	chr4	56823443/C//T	nonsynonymous	1	0
SLC4A4	chr4	72363407/A//T	nonsynonymous	1	0
BBS12	chr4	123664128/G//T	nonsynonymous	1	0
ADCY2	chr5	7757635/G//T	nonsynonymous	1	0
DNAH5	chr5	13928227/G//C	nonsynonymous	1	0
MAP1B	chr5	71494436/G//T	nonsynonymous	1	0

ACOT12	chr5	80639651/C//T	nonsynonymous	1	0
LYSMD3	chr5	89814801/TGAATG//T	nonsynonymous	1	0
DMXL1	chr5	118485976/A//G	nonsynonymous	1	0
TRPC7	chr5	135602026/T//A	synonymous	1	0
PCDHGB1	chr5	140732203/C//A	nonsynonymous	1	0
PDGFRB	chr5	149499694/G//T	synonymous	1	0
GABRB2	chr5	160721114/C//T	nonsynonymous	1	0
EHMT2	chr6	31855349/C//A	synonymous	1	0
ATF6B	chr6	32086562/C//G	nonsynonymous	1	0
LEMD2	chr6	33746132/G//A	nonsynonymous	1	0
ELOVL4	chr6	80629134/C//T	synonymous	1	0
GRM1	chr6	146673505/G//T	nonsynonymous	1	0
TFR2	chr7	100218706/G//T	nonsynonymous	1	0
PAX4	chr7	127255572/C//A	nonsynonymous	1	0
CSMD1	chr8	2808787/A//T	nonsynonymous	1	0
CSMD1	chr8	2815228/G//T	synonymous	1	0
ADAM2	chr8	39624777/G//A	synonymous	1	0
KIAA1429	chr8	95531327/T//C	nonsynonymous	1	0
TRAPPC9	chr8	140743439/C//A	synonymous	1	0
ADGRB1	chr8	143558473/A//T	nonsynonymous	1	0
ZNF510	chr9	99538394/C//A	synonymous	1	0
PFKFB3	chr10	6263484/C//T	synonymous	1	0
BMS1	chr10	43287968/T//G	nonsynonymous	1	0
KLLN	chr10	89622259/A//G	synonymous	1	0
OR8I2	chr11	55860851/T//A	nonsynonymous	1	0
NDUFV1	chr11	67377937/G//A	nonsynonymous	1	0
ARHGEF17	chr11	73076867/G//T	nonsynonymous	1	0
LIPT2	chr11	74203189/G//A	synonymous	1	0
MTNR1B	chr11	92703061/G//A	nonsynonymous	1	0
MAML2	chr11	95825383/C//T	synonymous	1	0
ARHGAP20	chr11	110485361/T//C	nonsynonymous	1	0
DRD2	chr11	113295295/C//T	nonsynonymous	1	0
MCAM	chr11	119182818/G//A	synonymous	1	0
FGF23	chr12	4479659/G//T	synonymous	1	0
CD163L1	chr12	7585240/A//G	nonsynonymous	1	0
CD163	chr12	7655108/T//A	nonsynonymous	1	0
RPL41	chr12	56510999/C//T	synonymous	1	0
STAB2	chr12	104111547/G//T	nonsynonymous	1	0
WSCD2	chr12	108589739/G//C	nonsynonymous	1	0
NAA16	chr13	41905445/A//G	nonsynonymous	1	0
HTR2A	chr13	47469783/G//T	nonsynonymous	1	0
CPNE6	chr14	24544456/C//T	nonsynonymous	1	0
NKX2-1	chr14	36986962/G//C	nonsynonymous	1	0
LRFN5	chr14	42356819/G//T	nonsynonymous	1	0
ISM2	chr14	77942224/C//A	nonsynonymous	1	0
BEGAIN	chr14	101006857/G//T	nonsynonymous	1	0
AHNAK2	chr14	105416158/G//A	nonsynonymous	1	0
ACAN	chr15	89402100/C//G	nonsynonymous	1	0
SLC5A2	chr16	31494478/A//G	synonymous	1	0
MMP2	chr16	55523623/T//A	nonsynonymous	1	0
CDH8	chr16	61689523/A//C	nonsynonymous	1	0
CDH8	chr16	61851400/G//T	synonymous	1	0
ACAP1	chr17	7246804/G//T	nonsynonymous	1	0
NCOR1	chr17	15995323/T//C	nonsynonymous	1	0
SP2	chr17	45994154/G//A	synonymous	1	0
SCN4A	chr17	62045673/T//A	nonsynonymous	1	0
DNAH17	chr17	76565481/G//C	nonsynonymous	1	0
LAMA1	chr18	6977742/C//T	nonsynonymous	1	0
SYT4	chr18	40853745/G//T	nonsynonymous	1	0
VSTM2B	chr19	30054894/C//A	synonymous	1	0
GRAMD1A	chr19	35505221/C//T	synonymous	1	0
PEG3	chr19	57329187/G//A	synonymous	1	0
ZIK1	chr19	58102221/G//T	nonsynonymous	1	0
KIZ	chr20	21143084/A//T	synonymous	1	0
FAM209B	chr20	55108549/A//T	nonsynonymous	1	0
ABHD16B	chr20	62493371/C//G	nonsynonymous	1	0
OPRL1	chr20	62730150/T//C	nonsynonymous	1	0
CLDN8	chr21	31588150/C//A	nonsynonymous	1	0
MIS18A	chr21	33651353/C//A	synonymous	1	0
CECR2	chr22	18027934/A//G	nonsynonymous	1	0

TMEM211	chr22	25331336/G//T	synonymous	1	0
PRR14L	chr22	32112031/T//C	synonymous	1	0
SLC5A4	chr22	32614516/G//A	synonymous	1	0
NPTXR	chr22	39219240/C//A	nonsynonymous	1	0
DMD	chrX	32563298/C//A	nonsynonymous	1	0
PFKFB1	chrX	54975592/G//T	synonymous	1	0
RBMXL3	chrX	114424397/C//T	synonymous	1	0
SLC25A5	chrX	118603690/C//G	nonsynonymous	1	0
ENOX2	chrX	129765514/C//A	nonsynonymous	1	0
ENOX2	chrX	129837150/G//T	nonsynonymous	1	0
PNMA5	chrX	152159095/C//T	nonsynonymous	1	0
KLHL21	chr1	6653411/G//A	synonymous	0	1
DISP3	chr1	11561859/C//T	synonymous	0	1
CTRC	chr1	15769913/G//A	nonsynonymous	0	1
HSPG2	chr1	22156076/G//A	nonsynonymous	0	1
OPRD1	chr1	29189436/C//T	nonsynonymous	0	1
AGO1	chr1	36379482/C//A	nonsynonymous	0	1
RNF115	chr1	145663244/C//T	nonsynonymous	0	1
NIT1	chr1	161089667/G//T	nonsynonymous	0	1
BRINP2	chr1	177247890/C//T	nonsynonymous	0	1
CEP350	chr1	180044227/C//T	nonsynonymous	0	1
FAM129A	chr1	184764744/C//T	synonymous	0	1
C1orf106	chr1	200881115/C//T	synonymous	0	1
SYT2	chr1	202572237/C//T	nonsynonymous	0	1
PIGR	chr1	207110911/G//A	nonsynonymous	0	1
ABHD1	chr2	27352719/C//T	nonsynonymous	0	1
TANC1	chr2	160032874/C//T	nonsynonymous	0	1
TNS1	chr2	218683029/C//T	synonymous	0	1
CTDSP1	chr2	219267992/G//A	nonsynonymous	0	1
SPEG	chr2	220333930/C//T	synonymous	0	1
HJURP	chr2	234749829/C//T	nonsynonymous	0	1
ATP2B2	chr3	10491116/G//A	nonsynonymous	0	1
FEZF2	chr3	62358581/C//T	synonymous	0	1
IGSF11	chr3	118623521/T//C	synonymous	0	1
HCLS1	chr3	121354594/G//A	nonsynonymous	0	1
ADCY5	chr3	123015053/C//T	nonsynonymous	0	1
MYL5	chr4	674346/C//T	nonsynonymous	0	1
ZFYVE28	chr4	2273051/C//T	nonsynonymous	0	1
KLHL5	chr4	39064182/G//T	nonsynonymous	0	1
NPFFR2	chr4	72897598/C//A	synonymous	0	1
ANKRD17	chr4	74013069/C//T	nonsynonymous	0	1
SHROOM3	chr4	77662799/C//T	nonsynonymous	0	1
BASP1	chr5	17275508/C//T	synonymous	0	1
C6	chr5	41199935/C//T	nonsynonymous	0	1
ZNF131	chr5	43174797/G//A	synonymous	0	1
NNT	chr5	43655960/G//A	nonsynonymous	0	1
LVRN	chr5	115298868/C//T	nonsynonymous	0	1
SLC35A4	chr5	139946808/C//T	synonymous	0	1
PCDHA5	chr5	140203245/G//A	nonsynonymous	0	1
RNF145	chr5	158630518/C//T	synonymous	0	1
INTS1	chr7	1524958/C//T	nonsynonymous	0	1
CARD11	chr7	2968323/G//A	nonsynonymous	0	1
PKD1L1	chr7	47917128/C//G	nonsynonymous	0	1
TRRAP	chr7	98565244/G//A	nonsynonymous	0	1
CUX1	chr7	101870830/C//T	nonsynonymous	0	1
OR2A25	chr7	143771320/G//A	nonsynonymous	0	1
AOC1	chr7	150555109/C//T	synonymous	0	1
KMT2C	chr7	151882712/C//T	nonsynonymous	0	1
ESYT2	chr7	158534354/G//A	synonymous	0	1
SOX7	chr8	10583195/A//G	synonymous	0	1
SLC18A1	chr8	20022428/G//A	synonymous	0	1
AZIN1	chr8	103840911/G//A	synonymous	0	1
ABRA	chr8	107781767/G//A	nonsynonymous	0	1
COL22A1	chr8	139642994/A//T	synonymous	0	1
TMEM249	chr8	145577491/C//T	nonsynonymous	0	1
FAM166B	chr9	35563207/C//T	nonsynonymous	0	1
FBXO10	chr9	37521678/G//A	synonymous	0	1
FANCC	chr9	97934405/C//T	nonsynonymous	0	1
OR13C5	chr9	107361178/TGTTA//T	nonsynonymous	0	1
UGCG	chr9	114695145/C//T	synonymous	0	1

NRP1	chr10	33619675/T//C	nonsynonymous	0	1
PARD3	chr10	34806021/C//A	nonsynonymous	0	1
C10orf71	chr10	50535004/A//G	synonymous	0	1
WASHC2A	chr10	51827904/T//C	nonsynonymous	0	1
FAM149B1	chr10	74994678/G//A	nonsynonymous	0	1
FAM35A	chr10	88930227/T//C	synonymous	0	1
PRAP1	chr10	135165868/G//A	nonsynonymous	0	1
WEE1	chr11	9595597/G//A	synonymous	0	1
ADM	chr11	10328079/G//A	nonsynonymous	0	1
EFEMP2	chr11	65635367/G//A	nonsynonymous	0	1
ALDH3B2	chr11	67433148/C//T	synonymous	0	1
PHOX2A	chr11	71952251/C//T	synonymous	0	1
RELT	chr11	73100204/G//A	nonsynonymous	0	1
MTNR1B	chr11	92702947/G//A	nonsynonymous	0	1
CEP126	chr11	101833161/G//T	nonsynonymous	0	1
SYT10	chr12	33538176/C//T	synonymous	0	1
KMT2D	chr12	49426107/G//T	synonymous	0	1
FGD6	chr12	95488477/G//T	synonymous	0	1
SELPLG	chr12	109017064/C//T	synonymous	0	1
SRRM4	chr12	119594313/C//T	nonsynonymous	0	1
CIT	chr12	120271915/C//T	nonsynonymous	0	1
ARL11	chr13	50205022/C//A	nonsynonymous	0	1
PCDH20	chr13	61987661/T//C	nonsynonymous	0	1
IPO5	chr13	98662271/G//T	synonymous	0	1
TEP1	chr14	20871929/G//A	nonsynonymous	0	1
OR4N4	chr15	22382965/C//T	nonsynonymous	0	1
VPS18	chr15	41191152/G//A	nonsynonymous	0	1
RAB27A	chr15	55497817/A//G	nonsynonymous	0	1
KIF7	chr15	90185494/G//A	synonymous	0	1
ASB7	chr15	101169752/G//A	nonsynonymous	0	1
SMG1	chr16	18851023/C//T	synonymous	0	1
KNOP1	chr16	19721901/C//T	nonsynonymous	0	1
NETO2	chr16	47163190/C//T	synonymous	0	1
CHD9	chr16	53189928/A//G	synonymous	0	1
PDPR	chr16	70177545/C//T	nonsynonymous	0	1
HYDIN	chr16	70972554/G//A	nonsynonymous	0	1
EFNB3	chr17	7612843/C//T	synonymous	0	1
CNTROB	chr17	7843066/A//G	nonsynonymous	0	1
TOM1L2	chr17	17772690/T//C	nonsynonymous	0	1
TAOK1	chr17	27869642/C//T	nonsynonymous	0	1
TMEM132E	chr17	32964987/C//T	synonymous	0	1
ARHGAP23	chr17	36614383/G//A	nonsynonymous	0	1
RUNDC1	chr17	41143096/A//G	nonsynonymous	0	1
TBX2	chr17	59481775/C//T	synonymous	0	1
RHBDF2	chr17	74468110/C//T	nonsynonymous	0	1
ME2	chr18	48473498/C//T	nonsynonymous	0	1
C2CD4C	chr19	407917/C//T	nonsynonymous	0	1
TLE2	chr19	3025079/G//A	nonsynonymous	0	1
ZNF699	chr19	9407257/C//T	nonsynonymous	0	1
BRD4	chr19	15376189/G//A	synonymous	0	1
SIN3B	chr19	16980660/G//A	synonymous	0	1
UPF1	chr19	18966840/G//A	nonsynonymous	0	1
NR2C2AP	chr19	19312402/C//A	synonymous	0	1
FXYD7	chr19	35642224/C//T	synonymous	0	1
CIC	chr19	42791751/C//T	nonsynonymous	0	1
PTGIR	chr19	47127207/G//A	synonymous	0	1
KCNJ14	chr19	48965354/G//A	nonsynonymous	0	1
TARM1	chr19	54573238/G//A	synonymous	0	1
EPB41L1	chr20	34783271/G//A	synonymous	0	1
PLCG1	chr20	39794859/C//T	nonsynonymous	0	1
EYA2	chr20	45717964/C//T	nonsynonymous	0	1
UCKL1	chr20	62572001/G//A	synonymous	0	1
RGS19	chr20	62705590/G//A	synonymous	0	1
ADAMTS1	chr21	28213357/G//A	synonymous	0	1
EMID1	chr22	29630174/G//A	nonsynonymous	0	1
AP1B1	chr22	29745272/G//A	nonsynonymous	0	1
RS1	chrX	18674837/G//A	synonymous	0	1
SYTL5	chrX	37967952/G//T	nonsynonymous	0	1
TEX13B	chrX	107224511/A//G	synonymous	0	1
TRPC5	chrX	111090334/C//T	synonymous	0	1

ZNF280C	chrX	129354448/C//T	nonsynonymous	0	1
MAMLD1	chrX	149680664/C//A	synonymous	0	1
	chrX	152110326/G//A	synonymous	0	1
RAB42	chr1	28920641/A//G	synonymous	0	1
COL16A1	chr1	32118412/C//T	nonsynonymous	0	1
MIER1	chr1	67452181/G//A	synonymous	0	1
CLCA4	chr1	87038389/C//T	nonsynonymous	0	1
RNF115	chr1	145663260/C//T	synonymous	0	1
GJA8	chr1	147380435/C//T	nonsynonymous	0	1
UBAP2L	chr1	154242830/A//G	synonymous	0	1
PEAR1	chr1	156878118/G//T	synonymous	0	1
SRGAP2	chr1	206626635/C//A	nonsynonymous	0	1
LEFTY1	chr1	226074626/G//A	nonsynonymous	0	1
C1orf101	chr1	244715729/G//A	synonymous	0	1
OR2T1	chr1	248570297/G//T	nonsynonymous	0	1
TPO	chr2	1457473/C//T	nonsynonymous	0	1
APOB	chr2	21242657/G//A	synonymous	0	1
	chr2	49003525/G//T	synonymous	0	1
AFTPH	chr2	64779295/G//A	synonymous	0	1
ASTL	chr2	96801128/C//T	nonsynonymous	0	1
LIMS2	chr2	128414974/C//T	synonymous	0	1
GALNT13	chr2	154801015/G//A	nonsynonymous	0	1
TTN	chr2	179436634/C//T	nonsynonymous	0	1
SPEG	chr2	220337690/G//A	nonsynonymous	0	1
ITPR1	chr3	4704852/C//T	nonsynonymous	0	1
EAF1	chr3	15477915/C//G	nonsynonymous	0	1
CCDC14	chr3	123680114/C//T	synonymous	0	1
TF	chr3	133473429/C//T	nonsynonymous	0	1
ZIC4	chr3	147108806/G//A	nonsynonymous	0	1
HGFAC	chr4	3446406/G//A	nonsynonymous	0	1
RXFP3	chr5	33937316/C//T	synonymous	0	1
	chr5	33951631/G//A	synonymous	0	1
NR3C1	chr5	142779874/G//A	synonymous	0	1
CAGE1	chr6	7370254/A//C	synonymous	0	1
RNF39	chr6	30038955/C//T	nonsynonymous	0	1
MUC22	chr6	30994341/C//T	nonsynonymous	0	1
UHRF1BP1	chr6	34835294/C//T	nonsynonymous	0	1
SCUBE3	chr6	35201032/C//T	synonymous	0	1
DNAH8	chr6	38905882/G//A	nonsynonymous	0	1
PRPH2	chr6	42690035/C//T	nonsynonymous	0	1
DST	chr6	56334718/C//T	nonsynonymous	0	1
GLI3	chr7	42088202/G//T	synonymous	0	1
CACNA2D1	chr7	81614032/A//C	synonymous	0	1
SEMA3D	chr7	84628897/G//A	synonymous	0	1
WDR91	chr7	134871810/G//C	nonsynonymous	0	1
SNTG1	chr8	51351160/G//A	nonsynonymous	0	1
PXDNL	chr8	52232532/G//T	synonymous	0	1
TRPA1	chr8	72970024/C//T	nonsynonymous	0	1
NECAB1	chr8	91804101/C//T	synonymous	0	1
ATAD2	chr8	124333356/C//T	synonymous	0	1
ADCY8	chr8	132052065/C//T	nonsynonymous	0	1
KCNQ3	chr8	133141799/G//T	synonymous	0	1
PSCA	chr8	143763566/G//A	synonymous	0	1
SPATA31C1	chr9	90538057/C//T	nonsynonymous	0	1
OR13C3	chr9	107298417/A//G	synonymous	0	1
NR5A1	chr9	127262992/C//T	nonsynonymous	0	1
C9orf163	chr9	139379279/C//CT	nonsynonymous	0	1
NOTCH1	chr9	139403459/C//T	nonsynonymous	0	1
GRIN1	chr9	140034068/G//A	nonsynonymous	0	1
PNPLA7	chr9	140374832/G//A	nonsynonymous	0	1
NPFFR1	chr10	72020460/C//T	nonsynonymous	0	1
DLG5	chr10	79570925/G//A	nonsynonymous	0	1
PTPN5	chr11	18765564/A//T	nonsynonymous	0	1
OR4C13	chr11	49974856/T//C	synonymous	0	1
OR5D16	chr11	55606581/G//A	synonymous	0	1
USP28	chr11	113675474/C//G	nonsynonymous	0	1
AICDA	chr12	8757845/G//A	synonymous	0	1
ST8SIA1	chr12	22487124/C//T	nonsynonymous	0	1
FIGNL2	chr12	52215965/G//A	nonsynonymous	0	1
SLC46A3	chr13	29286819/A//C	nonsynonymous	0	1

SPERT	chr13	46287980/A//G	nonsynonymous	0	1
KLHL1	chr13	70549838/G//C	synonymous	0	1
	chr13	113851461/C//A	synonymous	0	1
FLRT2	chr14	86089389/G//A	nonsynonymous	0	1
GSC	chr14	95235458/A//C	nonsynonymous	0	1
INO80	chr15	41372071/C//T	nonsynonymous	0	1
WHAMM	chr15	83482020/G//T	nonsynonymous	0	1
C15orf40	chr15	83679094/G//T	nonsynonymous	0	1
RPL3L	chr16	2003968/C//T	nonsynonymous	0	1
GPR139	chr16	20043133/G//A	nonsynonymous	0	1
PRMT7	chr16	68358715/G//A	nonsynonymous	0	1
DHRS7C	chr17	9680578/C//T	nonsynonymous	0	1
GHDC	chr17	40342245/C//T	synonymous	0	1
FAM171A2	chr17	42433918/C//A	nonsynonymous	0	1
SLC25A41	chr19	6427341/A//G	synonymous	0	1
ACTL9	chr19	8807842/C//T	nonsynonymous	0	1
MUC16	chr19	9088670/C//A	nonsynonymous	0	1
IFNL3	chr19	39734324/C//T	nonsynonymous	0	1
TNNT1	chr19	55652273/C//T	nonsynonymous	0	1
CFAP61	chr20	20278851/G//A	synonymous	0	1
MYBL2	chr20	42311463/G//T	nonsynonymous	0	1
FBLN1	chr22	45938127/G//T	nonsynonymous	0	1
PDZD4	chrX	153068716/GCA//G	synonymous	0	1
NBPF1	chr1	16913612/G//A	synonymous	0	1
RYR2	chr1	237774218/C//T	nonsynonymous	0	1
TSGA10	chr2	99697815/T//C	synonymous	0	1
THG1L	chr5	157160035/G//A	nonsynonymous	0	1
DMRT3	chr9	990875/C//T	nonsynonymous	0	1
	chr10	125780757/G//C	synonymous	0	1
MHRT	chr14	23886562/A//G	synonymous	0	1
MIA2	chr14	39717107/A//G	synonymous	0	1
MDGA2	chr14	47351270/G//T	nonsynonymous	0	1
MYO18B	chr22	26291213/C//T	nonsynonymous	0	1
HPCA	chr1	33354520/G//A	synonymous	0	1
	chr1	158986489/A//T	synonymous	0	1
PPIG	chr2	170487292/G//A	nonsynonymous	0	1
BARD1	chr2	215593725/T//C	nonsynonymous	0	1
TIAM2	chr6	155485552/C//T	nonsynonymous	0	1
ZNHIT2	chr11	64884573/G//T	nonsynonymous	0	1
RIN3	chr14	93118767/T//TCA	nonsynonymous	0	1
PSMD3	chr17	38153963/G//C	synonymous	0	1
PSMD12	chr17	65336909/C//A	synonymous	0	1
KIAA1683	chr19	18377273/C//CG	nonsynonymous	0	1
LOC101927051	chr22	38038813/C//T	synonymous	0	1
TXLNG	chrX	16850848/CAGAG//C	nonsynonymous	0	1
APEX2	chrX	55029513/C//A	synonymous	0	1
CROCC	chr1	17249172/C//T	synonymous	1	0
CEP85	chr1	26581980/C//T	nonsynonymous	1	0
SLC35D1	chr1	67507988/G//C	synonymous	1	0
LMNA	chr1	156106194/C//A	synonymous	1	0
METTL18	chr1	169762049/C//A	nonsynonymous	1	0
CCDC88A	chr2	55544941/G//A	nonsynonymous	1	0
	chr2	97637887/C//T	synonymous	1	0
ITM2C	chr2	231729678/G//A	synonymous	1	0
DGKD	chr2	234346121/C//T	synonymous	1	0
OR5K1	chr3	98188595/A//G	nonsynonymous	1	0
RHO	chr3	129251463/C//A	nonsynonymous	1	0
MECOM	chr3	168834474/GCTTATGA//G	nonsynonymous	1	0
ZNF732	chr4	264820/G//A	synonymous	1	0
PLEKHG4B	chr5	171207/C//T	nonsynonymous	1	0
	chr5	73218954/C//T	synonymous	1	0
ADGRV1	chr5	90020924/A//G	nonsynonymous	1	0
PCDHGA1	chr5	140712533/C//T	nonsynonymous	1	0
PCDHGB3	chr5	140751844/C//T	nonsynonymous	1	0
HIST1H2AL	chr6	27833349/GACA//G	nonsynonymous	1	0
CARD11	chr7	2946340/G//A	nonsynonymous	1	0
SCIN	chr7	12675752/G//GTCT	nonsynonymous	1	0
SBDS	chr7	66456200/C//T	nonsynonymous	1	0
ZNF804B	chr7	88965493/G//A	nonsynonymous	1	0
PPP1R9A	chr7	94540327/A//G	nonsynonymous	1	0

GNA14	chr9	80046298/C//T	nonsynonymous	1	0
GTF3C4	chr9	135554781/C//T	nonsynonymous	1	0
MYO3A	chr10	26315314/C//A	nonsynonymous	1	0
PTEN	chr10	89692904/C//G	nonsynonymous	1	0
PLPP4	chr10	122334667/C//T	nonsynonymous	1	0
LUZP2	chr11	25100270/C//A	synonymous	1	0
GUCY1A2	chr11	106558445/G//A	nonsynonymous	1	0
SRPRA	chr11	126135214/G//A	synonymous	1	0
LRP1	chr12	57593182/C//T	synonymous	1	0
MYF6	chr12	81101930/G//T	synonymous	1	0
C15orf41	chr15	37001439/G//C	nonsynonymous	1	0
BUB1B	chr15	40457324/C//T	nonsynonymous	1	0
PYGO1	chr15	55839304/C//T	synonymous	1	0
LIPC	chr15	58838165/G//A	nonsynonymous	1	0
DNAH3	chr16	21136562/C//T	synonymous	1	0
GTF3C1	chr16	27549121/G//A	nonsynonymous	1	0
POLR2C	chr16	57503204/C//T	nonsynonymous	1	0
TCF25	chr16	89965043/C//A	nonsynonymous	1	0
STAT5B	chr17	40353861/G//A	synonymous	1	0
SGSH	chr17	78188487/C//T	nonsynonymous	1	0
CELF4	chr18	34853075/G//T	nonsynonymous	1	0
ELOA2	chr18	44561092/C//T	nonsynonymous	1	0
POLRMT	chr19	619646/C//T	synonymous	1	0
SLC25A41	chr19	6426523/G//A	synonymous	1	0
FBN3	chr19	8206610/C//T	nonsynonymous	1	0
RGL3	chr19	11517430/C//T	nonsynonymous	1	0
PIK3R2	chr19	18266589/A//C	synonymous	1	0
FOXA2	chr20	22563679/GCC//G	nonsynonymous	1	0
CD93	chr20	23065533/C//A	nonsynonymous	1	0
MYH7B	chr20	33578925/G//A	nonsynonymous	1	0
CEP250	chr20	34060581/G//T	nonsynonymous	1	0
NCAM2	chr21	22664456/C//A	nonsynonymous	1	0
TIAM1	chr21	32526628/C//T	synonymous	1	0
PRDM15	chr21	43279720/A//G	nonsynonymous	1	0
RNF185	chr22	31591554/C//A	nonsynonymous	1	0
SUN2	chr22	39132218/G//A	synonymous	1	0
MIEF1	chr22	39910225/C//G	nonsynonymous	1	0
EP300	chr22	41573057/TCTGCAAGCAGCTCA[illegible]	nonsynonymous	1	0
MXRA5	chrX	3235405/G//A	nonsynonymous	1	0
FTHL17	chrX	31089535/C//T	nonsynonymous	1	0
ACSL4	chrX	108926505/T//C	nonsynonymous	1	0
PLXNB3	chrX	153036313/G//A	nonsynonymous	1	0
F8	chrX	154157903/G//T	nonsynonymous	1	0
DNAH1	chr3	52383391/C//T	synonymous	0	1
CHST13	chr3	126260696/G//C	nonsynonymous	0	1
NDST1	chr5	149900838/C//T	nonsynonymous	0	1
CARD11	chr7	2951847/C//T	nonsynonymous	0	1
WASL	chr7	123332486/T//C	nonsynonymous	0	1
CTSB	chr8	11703251/G//T	nonsynonymous	0	1
CSGALNACT1	chr8	19362728/G//T	synonymous	0	1
TMEM64	chr8	91657642/G//A	synonymous	0	1
HSPA14	chr10	14893286/C//T	nonsynonymous	0	1
OR8K3	chr11	56085881/G//T	nonsynonymous	0	1
JAML	chr11	118076602/C//CGCGAG	nonsynonymous	0	1
BSX	chr11	122848365/C//T	nonsynonymous	0	1
PAPLN	chr14	73719482/C//T	nonsynonymous	0	1
HS3ST3A1	chr17	13503956/C//A	nonsynonymous	0	1
POLR2E	chr19	1091849/T//C	nonsynonymous	0	1
ZNF708	chr19	21476223/C//T	synonymous	0	1
PTPRT	chr20	40979344/C//G	nonsynonymous	0	1
SPRED2	chr2	65540895/C//T	nonsynonymous	0	1
HSPD1	chr2	198352607/T//C	nonsynonymous	0	1
HSPD1	chr2	198352609/C//T	synonymous	0	1
CFAP44	chr3	113118844/G//C	nonsynonymous	0	1
COL6A6	chr3	130313144/G//A	nonsynonymous	0	1
EYS	chr6	65707569/T//A	nonsynonymous	0	1
DOPEY1	chr6	83847340/G//A	synonymous	0	1
QRSL1	chr6	107097034/C//G	nonsynonymous	0	1
SLC18B1	chr6	133091444/C//A	nonsynonymous	0	1
GLI3	chr7	42005935/G//A	synonymous	0	1

ACHE	chr7	100491656/G//A	synonymous	0	1
HR	chr8	21976718/T//C	nonsynonymous	0	1
SMARCA2	chr9	2039818/A//G	synonymous	0	1
UCN3	chr10	5415985/C//A	nonsynonymous	0	1
TET1	chr10	70333031/A//G	synonymous	0	1
PYROXD2	chr10	100174826/C//T	nonsynonymous	0	1
SLC18A2	chr10	119003828/G//C	synonymous	0	1
IGSF22	chr11	18737033/T//C	nonsynonymous	0	1
AMBRA1	chr11	46564512/G//C	nonsynonymous	0	1
RTN4RL2	chr11	57243799/C//T	synonymous	0	1
B3GAT1	chr11	134254012/G//A	synonymous	0	1
TNFRSF1A	chr12	6442966/C//T	nonsynonymous	0	1
ANO6	chr12	45815015/G//A	synonymous	0	1
ELF1	chr13	41515390/A//G	nonsynonymous	0	1
LACC1	chr13	44456359/A//G	nonsynonymous	0	1
COL4A2	chr13	111144444/G//A	nonsynonymous	0	1
	chr15	45409642/G//A	synonymous	0	1
BBS4	chr15	73004634/C//T	nonsynonymous	0	1
IQGAP1	chr15	91026607/G//C	nonsynonymous	0	1
CBX8	chr17	77768429/AGCAT//A	synonymous	0	1
CBX8	chr17	77768432/AT//A	synonymous	0	1
ZNF708	chr19	21476979/C//T	synonymous	0	1
ZNF208	chr19	22155615/C//G	nonsynonymous	0	1
ZNF607	chr19	38200664/G//C	synonymous	0	1
SIPA1L3	chr19	38633345/A//G	synonymous	0	1
PSG1	chr19	43376047/T//G	nonsynonymous	0	1
NLRP9	chr19	56244180/C//A	synonymous	0	1
CPXM1	chr20	2779123/C//T	nonsynonymous	0	1
SPTLC3	chr20	13145399/A//G	nonsynonymous	0	1
SYCP2	chr20	58440643/A//T	synonymous	0	1
ZNRF3	chr22	29446905/G//T	nonsynonymous	0	1
GOLGB1	chr3	121410049/T//A	nonsynonymous	1	0
SSPO	chr7	149486718/T//TG	nonsynonymous	1	0
YME1L1	chr10	27423801/T//A	nonsynonymous	1	0
SHISA9	chr16	13329129/C//T	nonsynonymous	1	0
HRC	chr19	49655307/C//T	synonymous	1	0
BIRC7	chr20	61869312/G//A	nonsynonymous	1	0
IQSEC2	chrX	53284028/C//T	nonsynonymous	1	0
ARID1A	chr1	27106177/TC//T	nonsynonymous	1	0
AHDC1	chr1	27876459/G//A	nonsynonymous	1	0
TAF12	chr1	28929974/C//A	synonymous	1	0
USP24	chr1	55611703/A//C	nonsynonymous	1	0
OLFM3	chr1	102270326/A//G	nonsynonymous	1	0
TROVE2	chr1	193038643/G//A	synonymous	1	0
ELF3	chr1	201981520/A//G	nonsynonymous	1	0
PLEKHA6	chr1	204236633/GGAC//G	nonsynonymous	1	0
TRIM54	chr2	27505591/A//C	synonymous	1	0
SOCS5	chr2	46986818/G//A	nonsynonymous	1	0
FER1L5	chr2	97370431/T//A	synonymous	1	0
TBC1D8	chr2	101624402/A//C	nonsynonymous	1	0
SULT1C4	chr2	109002830/T//C	nonsynonymous	1	0
NCKAP5	chr2	133541098/C//T	nonsynonymous	1	0
ANKRD44	chr2	197990573/G//A	synonymous	1	0
UGT1A3	chr2	234638367/A//G	nonsynonymous	1	0
XYLB	chr3	38438587/C//A	nonsynonymous	1	0
ULK4	chr3	41938339/G//A	nonsynonymous	1	0
HTT	chr4	3235072/G//C	synonymous	1	0
BLOC1S4	chr4	6717936/C//G	synonymous	1	0
NUP54	chr4	77069526/A//G	nonsynonymous	1	0
TIGD4	chr4	153690731/T//C	nonsynonymous	1	0
CDH18	chr5	19571863/G//T	nonsynonymous	1	0
GOLPH3	chr5	32126313/C//T	synonymous	1	0
CCDC112	chr5	114604607/A//G	nonsynonymous	1	0
WNT8A	chr5	137424682/T//A	nonsynonymous	1	0
NDUFA2	chr5	140027150/T//G	nonsynonymous	1	0
LCP2	chr5	169677886/C//A	nonsynonymous	1	0
EYS	chr6	64431050/A//G	synonymous	1	0
GPC2	chr7	99769474/C//T	synonymous	1	0
FBXO24	chr7	100189359/T//A	nonsynonymous	1	0
LRWD1	chr7	102112658/C//T	synonymous	1	0

SSPO	chr7	149504000/C//T	nonsynonymous	1	0
CHMP7	chr8	23104362/A//C	nonsynonymous	1	0
PCMTD1	chr8	52733173/C//T	nonsynonymous	1	0
RUNX1T1	chr8	93026943/T//C	nonsynonymous	1	0
SDC2	chr8	97605773/A//T	synonymous	1	0
RFX3	chr9	3266221/A//G	nonsynonymous	1	0
IFNA1	chr9	21440665/G//C	synonymous	1	0
NTRK2	chr9	87342641/G//A	nonsynonymous	1	0
IFIT1	chr10	91162822/A//C	nonsynonymous	1	0
FRA10AC1	chr10	95458057/C//T	nonsynonymous	1	0
MRVI1	chr11	10647853/G//A	nonsynonymous	1	0
NUCB2	chr11	17316952/G//A	nonsynonymous	1	0
E2F8	chr11	19246893/C//A	nonsynonymous	1	0
OR4A47	chr11	48510624/T//A	nonsynonymous	1	0
CEP295	chr11	93431708/G//A	synonymous	1	0
DYNC2H1	chr11	102988533/T//C	nonsynonymous	1	0
ABCG4	chr11	119025563/T//A	synonymous	1	0
VSIG2	chr11	124619712/C//T	nonsynonymous	1	0
ADAMTS8	chr11	130275876/G//A	synonymous	1	0
ITPR2	chr12	26492400/T//C	nonsynonymous	1	0
MAP3K12	chr12	53879230/T//C	nonsynonymous	1	0
FRS2	chr12	69968697/A//C	synonymous	1	0
TMTC3	chr12	88589406/C//G	nonsynonymous	1	0
CDK17	chr12	96676384/A//C	synonymous	1	0
SOS2	chr14	50626736/T//C	nonsynonymous	1	0
PTGDR	chr14	52734783/T//C	nonsynonymous	1	0
OCA2	chr15	28202801/G//A	nonsynonymous	1	0
TRPM7	chr15	50884697/T//A	synonymous	1	0
SLTM	chr15	59225608/T//C	nonsynonymous	1	0
MEGF11	chr15	66209171/G//C	nonsynonymous	1	0
PARP6	chr15	72548885/G//A	nonsynonymous	1	0
	chr15	101454856/T//C	synonymous	1	0
FAHD1	chr16	1877259/T//C	nonsynonymous	1	0
RABEP1	chr17	5289508/A//C	synonymous	1	0
CDK12	chr17	37682569/G//A	nonsynonymous	1	0
RNF213	chr17	78354771/C//T	nonsynonymous	1	0
SWSAP1	chr19	11486385/G//T	nonsynonymous	1	0
ZNF91	chr19	23543978/A//G	synonymous	1	0
ZNF681	chr19	23926644/C//A	nonsynonymous	1	0
CYP2F1	chr19	41622503/C//A	nonsynonymous	1	0
MEGF8	chr19	42848939/A//G	nonsynonymous	1	0
PSG3	chr19	43237162/G//T	nonsynonymous	1	0
NLRP5	chr19	56538643/G//A	synonymous	1	0
ZNF749	chr19	57953265/T//A	nonsynonymous	1	0
RBCK1	chr20	409643/G//T	nonsynonymous	1	0
DEFB121	chr20	29993974/G//A	synonymous	1	0
SNTA1	chr20	32005646/G//A	nonsynonymous	1	0
PLTP	chr20	44538268/G//A	synonymous	1	0
KRTAP19-3	chr21	31863987/A//G	synonymous	1	0
TCP10L	chr21	33954592/A//T	nonsynonymous	1	0
	chr21	37444697/G//T	synonymous	1	0
DDTL	chr22	24313596/GGAT//G	synonymous	1	0
DEPDC5	chr22	32180797/C//G	synonymous	1	0
ATP7A	chrX	77298207/T//C	nonsynonymous	1	0
LCE2D	chr1	152636595/A//C	nonsynonymous	0	1
DCST2	chr1	155002711/T//C	synonymous	0	1
SULT6B1	chr2	37402363/C//A	nonsynonymous	0	1
OLA1	chr2	175087815/C//A	nonsynonymous	0	1
DAAM2	chr6	39836633/G//T	nonsynonymous	0	1
MUC17	chr7	100676265/T//G	nonsynonymous	0	1
DPP6	chr7	154263926/G//T	nonsynonymous	0	1
TULP3	chr12	3046899/A//G	synonymous	0	1
ZNF493	chr19	21606567/G//T	nonsynonymous	0	1
ZNF493	chr19	21606579/G//A	nonsynonymous	0	1
ZNF493	chr19	21606587/C//T	nonsynonymous	0	1
ZNF493	chr19	21606594/A//G	nonsynonymous	0	1
KCNG1	chr20	49620953/C//T	nonsynonymous	0	1
ACAP3	chr1	1235356/G//A	synonymous	1	0
NT5C1A	chr1	40124856/A//G	synonymous	1	0
RLF	chr1	40704503/A//C	nonsynonymous	1	0

PATJ	chr1	62594608/T//C	synonymous	1	0
PTGFRN	chr1	117527323/C//G	nonsynonymous	1	0
BLZF1	chr1	169347631/G//C	nonsynonymous	1	0
TMCC2	chr1	205238494/C//T	synonymous	1	0
IL10	chr1	206944411/G//A	synonymous	1	0
NID1	chr1	236141141/G//C	synonymous	1	0
DHX57	chr2	39046214/G//C	nonsynonymous	1	0
TTN	chr2	179444376/C//T	synonymous	1	0
ZNF142	chr2	219509184/T//A	synonymous	1	0
NYAP2	chr2	226491836/A//C	nonsynonymous	1	0
VILL	chr3	38035915/A//G	nonsynonymous	1	0
PHLDB2	chr3	111651212/A//G	nonsynonymous	1	0
ZBTB38	chr3	141164720/G//A	nonsynonymous	1	0
SAMD7	chr3	169644376/G//A	nonsynonymous	1	0
TBC1D19	chr4	26585857/G//C	nonsynonymous	1	0
CLPTM1L	chr5	1338031/C//T	synonymous	1	0
AMACR	chr5	33989473/C//T	nonsynonymous	1	0
MCTP1	chr5	94259719/G//C	nonsynonymous	1	0
PCDHB6	chr5	140530453/C//A	synonymous	1	0
ARHGEF37	chr5	149008460/C//G	nonsynonymous	1	0
ZFP62	chr5	180278047/A//C	nonsynonymous	1	0
SLC35A1	chr6	88221233/A//G	nonsynonymous	1	0
CYP2W1	chr7	1027174/C//T	synonymous	1	0
HNRNPA2B1	chr7	26233248/C//G	nonsynonymous	1	0
GHRHR	chr7	31010759/T//G	nonsynonymous	1	0
AMPH	chr7	38471815/G//A	synonymous	1	0
GSAP	chr7	76943803/A//T	nonsynonymous	1	0
NUP205	chr7	135307609/G//C	nonsynonymous	1	0
TTC26	chr7	138818574/G//T	synonymous	1	0
KMT2C	chr7	151878301/G//A	nonsynonymous	1	0
FZD3	chr8	28420463/A//G	nonsynonymous	1	0
SLC25A32	chr8	104413864/G//T	nonsynonymous	1	0
EPPK1	chr8	144942235/G//A	synonymous	1	0
SLC1A1	chr9	4585449/C//G	nonsynonymous	1	0
ZBTB26	chr9	125681594/T//A	nonsynonymous	1	0
PRRC2B	chr9	134357930/C//G	nonsynonymous	1	0
DHTKD1	chr10	12160855/A//G	nonsynonymous	1	0
DDX50	chr10	70673023/T//C	nonsynonymous	1	0
TACC2	chr10	123843331/C//T	nonsynonymous	1	0
ANO3	chr11	26619968/G//T	nonsynonymous	1	0
FIBIN	chr11	27016612/C//G	nonsynonymous	1	0
LMO2	chr11	33881105/C//T	nonsynonymous	1	0
CKAP5	chr11	46811714/G//T	nonsynonymous	1	0
MTMR2	chr11	95578126/TCGATGTCCAAAACTT	nonsynonymous	1	0
IGSF9B	chr11	133791255/T//C	nonsynonymous	1	0
METTL25	chr12	82850590/T//C	synonymous	1	0
RPL10L	chr14	47120477/C//T	nonsynonymous	1	0
MAP3K9	chr14	71197427/C//T	synonymous	1	0
TJP1	chr15	30025440/G//A	nonsynonymous	1	0
TJP1	chr15	30025442/G//A	nonsynonymous	1	0
GABPB1	chr15	50570713/C//A	synonymous	1	0
ANKRD34C	chr15	79585908/C//T	synonymous	1	0
ECI1	chr16	2296858/A//G	nonsynonymous	1	0
MYO18A	chr17	27417564/C//T	nonsynonymous	1	0
TAF15	chr17	34151147/G//A	nonsynonymous	1	0
DSC2	chr18	28649083/C//G	nonsynonymous	1	0
PIGN	chr18	59713164/G//A	synonymous	1	0
CNN1	chr19	11660540/C//G	nonsynonymous	1	0
BRD4	chr19	15366994/C//T	synonymous	1	0
PDE4C	chr19	18343948/G//T	synonymous	1	0
CATSPERG	chr19	38834239/G//C	nonsynonymous	1	0
LTBP4	chr19	41119382/C//T	nonsynonymous	1	0
PRMT1	chr19	50180558/G//C	synonymous	1	0
ZNF665	chr19	53678798/G//A	synonymous	1	0
SIGLEC1	chr20	3687764/C//A	nonsynonymous	1	0
ZFP64	chr20	50776688/A//G	nonsynonymous	1	0
POTED	chr21	15013735/A//G	nonsynonymous	1	0
DDX53	chrX	23020019/A//G	synonymous	1	0
XK	chrX	37553670/A//G	nonsynonymous	1	0
USP11	chrX	47104080/G//C	nonsynonymous	1	0

IGSF1	chrX	130420665/G//A	synonymous	1	0
SLC9A6	chrX	135095108/G//C	nonsynonymous	1	0
RAB39B	chrX	154490399/C//G	nonsynonymous	1	0
KANSL3	chr2	97302850/C//T	nonsynonymous	0	1
BSN	chr3	49700826/G//A	synonymous	0	1
SPATA6L	chr9	4629172/T//A	synonymous	0	1
TMEM159	chr16	21190854/T//C	nonsynonymous	0	1
PLCD3	chr17	43195689/C//T	nonsynonymous	0	1
DDX42	chr17	61889348/G//C	nonsynonymous	0	1
ZNF493	chr19	21606544/C//T	synonymous	0	1
CEP104	chr1	3746354/C//T	nonsynonymous	1	0
AJAP1	chr1	4772361/G//A	nonsynonymous	1	0
NPHP4	chr1	5925300/C//T	nonsynonymous	1	0
TNFRSF9	chr1	7993277/G//A	synonymous	1	0
KAZN	chr1	15441066/C//T	nonsynonymous	1	0
SRSF10	chr1	24297734/T//C	synonymous	1	0
BSDC1	chr1	32852428/C//T	synonymous	1	0
TRIM62	chr1	33612990/G//A	nonsynonymous	1	0
ZMYM1	chr1	35570335/T//G	nonsynonymous	1	0
SCMH1	chr1	41625486/A//C	synonymous	1	0
PPCS	chr1	42925492/G//A	synonymous	1	0
STIL	chr1	47765628/T//G	nonsynonymous	1	0
TRABD2B	chr1	48267183/C//T	nonsynonymous	1	0
ABCA4	chr1	94495043/C//A	synonymous	1	0
VAV3	chr1	108185236/A//G	synonymous	1	0
CREB3L4	chr1	153941008/C//T	nonsynonymous	1	0
FCRL2	chr1	157736705/C//T	nonsynonymous	1	0
CRP	chr1	159683783/A//C	nonsynonymous	1	0
RXRG	chr1	165389229/C//A	nonsynonymous	1	0
RXRG	chr1	165398062/T//G	nonsynonymous	1	0
TNR	chr1	175372551/C//T	nonsynonymous	1	0
SEC16B	chr1	177908830/T//C	synonymous	1	0
AXDND1	chr1	179460838/A//C	synonymous	1	0
HMCN1	chr1	186026487/A//C	nonsynonymous	1	0
ZBED6	chr1	203766686/A//C	synonymous	1	0
ZBED6	chr1	203766880/A//C	nonsynonymous	1	0
PLEKHA6	chr1	204226699/C//T	nonsynonymous	1	0
PPP1R15B	chr1	204380372/G//A	synonymous	1	0
RASSF5	chr1	206680917/C//G	synonymous	1	0
DISP1	chr1	223178441/T//C	synonymous	1	0
DNAH14	chr1	225347105/A//C	nonsynonymous	1	0
ENAH	chr1	225700608/A//C	synonymous	1	0
TRIM17	chr1	228596410/A//C	nonsynonymous	1	0
RYR2	chr1	237824227/C//T	nonsynonymous	1	0
NLRP3	chr1	247599375/G//A	nonsynonymous	1	0
SH3YL1	chr2	218777/T//G	synonymous	1	0
MYT1L	chr2	1926189/T//G	nonsynonymous	1	0
AGBL5	chr2	27279532/A//C	synonymous	1	0
C2orf71	chr2	29294944/T//C	synonymous	1	0
C2orf71	chr2	29296991/T//G	nonsynonymous	1	0
SRBD1	chr2	45616498/A//C	nonsynonymous	1	0
ADD2	chr2	70910888/C//T	synonymous	1	0
CYP26B1	chr2	72360318/C//T	nonsynonymous	1	0
TLX2	chr2	74742016/C//T	nonsynonymous	1	0
DNAH6	chr2	84897519/A//T	nonsynonymous	1	0
C2orf68	chr2	85839024/A//G	synonymous	1	0
SMYD1	chr2	88405988/A//T	nonsynonymous	1	0
IL18R1	chr2	102998119/T//G	nonsynonymous	1	0
NCK2	chr2	106471636/G//A	nonsynonymous	1	0
DDX18	chr2	118582533/T//G	synonymous	1	0
MYO7B	chr2	128338393/T//C	nonsynonymous	1	0
R3HDM1	chr2	136393651/T//C	synonymous	1	0
CCDC148	chr2	159165952/T//C	nonsynonymous	1	0
HOXD4	chr2	177017392/C//A	nonsynonymous	1	0
TTN	chr2	179438362/C//A	nonsynonymous	1	0
TTN	chr2	179462342/C//T	nonsynonymous	1	0
CCDC141	chr2	179721049/A//C	nonsynonymous	1	0
UBE2E3	chr2	181846912/A//C	nonsynonymous	1	0
FSIP2	chr2	186657485/T//G	nonsynonymous	1	0
ITGAV	chr2	187466775/C//T	synonymous	1	0

DIRC1	chr2	189599379/T//G	nonsynonymous	1	0
PMS1	chr2	190728866/G//A	nonsynonymous	1	0
HECW2	chr2	197184123/A//G	synonymous	1	0
SCG2	chr2	224462218/C//T	nonsynonymous	1	0
IRS1	chr2	227660885/C//T	nonsynonymous	1	0
FANCD2	chr3	10081473/C//T	synonymous	1	0
ATG7	chr3	11340213/T//G	nonsynonymous	1	0
RAF1	chr3	12641896/T//C	nonsynonymous	1	0
LRRFIP2	chr3	37107769/T//G	synonymous	1	0
SLC22A14	chr3	38354563/G//A	nonsynonymous	1	0
ACVR2B	chr3	38521253/T//C	nonsynonymous	1	0
MYRIP	chr3	39942341/G//A	nonsynonymous	1	0
ULK4	chr3	41759295/T//G	nonsynonymous	1	0
ZMYND10	chr3	50379354/T//A	nonsynonymous	1	0
GRM2	chr3	51747011/A//G	nonsynonymous	1	0
DNAH12	chr3	57394029/T//G	nonsynonymous	1	0
CADPS	chr3	62543155/G//A	nonsynonymous	1	0
ZNF717	chr3	75787231/T//G	nonsynonymous	1	0
ADGRG7	chr3	100365553/T//G	nonsynonymous	1	0
SLC9C1	chr3	111993740/T//C	synonymous	1	0
POLQ	chr3	121264564/G//A	nonsynonymous	1	0
COL6A5	chr3	130148429/G//A	nonsynonymous	1	0
CEP70	chr3	138248235/A//G	synonymous	1	0
FAIM	chr3	138341054/T//G	nonsynonymous	1	0
EIF2A	chr3	150264581/C//T	synonymous	1	0
AADAC	chr3	151546016/T//G	synonymous	1	0
SPATA16	chr3	172834969/A//G	nonsynonymous	1	0
MAP3K13	chr3	185198203/T//C	synonymous	1	0
SENP2	chr3	185329464/T//G	nonsynonymous	1	0
ZNF595	chr4	53310/C//T	synonymous	1	0
LCORL	chr4	17885717/A//C	nonsynonymous	1	0
KIT	chr4	55599298/T//G	nonsynonymous	1	0
UBA6	chr4	68492134/T//A	nonsynonymous	1	0
UGT2B15	chr4	69535906/G//A	nonsynonymous	1	0
CNOT6L	chr4	78695831/G//A	nonsynonymous	1	0
MAPK10	chr4	87080444/C//T	synonymous	1	0
ABCG2	chr4	89013444/A//C	nonsynonymous	1	0
	chr4	106833380/A//C	synonymous	1	0
CFI	chr4	110687738/A//C	nonsynonymous	1	0
TRAM1L1	chr4	118005652/C//T	nonsynonymous	1	0
QRFPR	chr4	122257997/C//T	nonsynonymous	1	0
TRPC3	chr4	122872662/C//T	synonymous	1	0
SPATA5	chr4	123854599/A//C	nonsynonymous	1	0
SCLT1	chr4	129869644/T//G	synonymous	1	0
FBXW7	chr4	153253743/CTTACCCTCT//C	nonsynonymous	1	0
TLR3	chr4	187003641/T//C	synonymous	1	0
CTNND2	chr5	10988315/T//G	nonsynonymous	1	0
DROSHA	chr5	31529209/A//G	synonymous	1	0
PRLR	chr5	35066154/A//G	synonymous	1	0
PRLR	chr5	35070347/T//A	nonsynonymous	1	0
IPO11	chr5	61747654/A//C	nonsynonymous	1	0
IQGAP2	chr5	75969868/T//A	synonymous	1	0
VCAN	chr5	82786137/C//T	synonymous	1	0
ERAP2	chr5	96237330/C//T	nonsynonymous	1	0
NUDT12	chr5	102894981/T//C	nonsynonymous	1	0
COMMD10	chr5	115423281/G//A	synonymous	1	0
DMXL1	chr5	118468944/A//G	nonsynonymous	1	0
ADAMTS19	chr5	128958007/T//G	nonsynonymous	1	0
DCANP1	chr5	134782781/T//C	synonymous	1	0
PCDHB8	chr5	140559742/C//T	synonymous	1	0
PCDHGB1	chr5	140730281/G//A	nonsynonymous	1	0
PCDHGA5	chr5	140744632/C//T	synonymous	1	0
PCDH1	chr5	141244104/C//T	nonsynonymous	1	0
SH3RF2	chr5	145427343/T//G	synonymous	1	0
PPARGC1B	chr5	149214875/T//C	nonsynonymous	1	0
HAVCR2	chr5	156533664/A//C	nonsynonymous	1	0
CDHR2	chr5	176008491/G//T	nonsynonymous	1	0
BTN3A3	chr6	26448673/A//C	synonymous	1	0
TRIM39	chr6	30309774/G//A	nonsynonymous	1	0
LTB	chr6	31549353/G//A	nonsynonymous	1	0

CFB	chr6	31916652/CA//C	nonsynonymous	1	0
TNXB	chr6	32011551/C//T	synonymous	1	0
COL11A2	chr6	33147257/A//G	nonsynonymous	1	0
PI16	chr6	36926964/G//A	nonsynonymous	1	0
DNAH8	chr6	38691120/T//G	nonsynonymous	1	0
DNAH8	chr6	38854694/G//T	nonsynonymous	1	0
SRF	chr6	43146835/G//A	nonsynonymous	1	0
ABCC10	chr6	43417245/A//C	nonsynonymous	1	0
CD2AP	chr6	47573976/G//A	nonsynonymous	1	0
COL12A1	chr6	75899084/A//C	nonsynonymous	1	0
LAMA4	chr6	112462115/T//G	nonsynonymous	1	0
FAM184A	chr6	119296260/G//A	synonymous	1	0
SMPDL3A	chr6	123130254/T//G	nonsynonymous	1	0
THEMIS	chr6	128134644/G//A	nonsynonymous	1	0
LAMA2	chr6	129573434/T//A	nonsynonymous	1	0
	chr6	134498900/T//C	synonymous	1	0
AGPAT4	chr6	161570290/G//A	synonymous	1	0
AGPAT4	chr6	161575251/T//G	nonsynonymous	1	0
GNA12	chr7	2834656/G//A	nonsynonymous	1	0
WIPI2	chr7	5256293/G//A	synonymous	1	0
USP42	chr7	6189360/A//G	synonymous	1	0
ABCA13	chr7	48280594/C//T	nonsynonymous	1	0
ZNF727	chr7	63537907/C//T	synonymous	1	0
RHBDD2	chr7	75508356/G//A	synonymous	1	0
PCLO	chr7	82455931/A//C	nonsynonymous	1	0
BAIAP2L1	chr7	97923404/G//A	nonsynonymous	1	0
MUC17	chr7	100680574/T//G	synonymous	1	0
CDHR3	chr7	105673030/G//A	nonsynonymous	1	0
SLC26A4	chr7	107355940/C//T	synonymous	1	0
FOXP2	chr7	114302182/A//G	synonymous	1	0
CFTR	chr7	117232014/A//T	nonsynonymous	1	0
MEST	chr7	130144793/C//T	nonsynonymous	1	0
OR9A2	chr7	142724096/T//C	nonsynonymous	1	0
PIP	chr7	142829240/A//T	nonsynonymous	1	0
ESYT2	chr7	158534396/T//C	synonymous	1	0
XKR4	chr8	56270411/A//G	nonsynonymous	1	0
LOC101929415	chr8	57358592/A//C	synonymous	1	0
ZFHX4	chr8	77765507/T//C	nonsynonymous	1	0
POP1	chr8	99148759/A//C	nonsynonymous	1	0
TRPS1	chr8	116599498/T//C	synonymous	1	0
TAF2	chr8	120809934/T//G	nonsynonymous	1	0
DENND3	chr8	142170832/A//C	nonsynonymous	1	0
SPATC1	chr8	145095094/A//G	nonsynonymous	1	0
SPATC1	chr8	145101649/C//T	nonsynonymous	1	0
SCRT1	chr8	145557134/C//T	nonsynonymous	1	0
C8orf82	chr8	145752962/G//A	nonsynonymous	1	0
RLN1	chr9	5335500/A//G	synonymous	1	0
MPDZ	chr9	13137949/A//C	synonymous	1	0
ADAMTSL1	chr9	18817110/A//G	nonsynonymous	1	0
TMEM215	chr9	32784771/T//C	nonsynonymous	1	0
TRPM3	chr9	73151277/A//G	synonymous	1	0
ZNF484	chr9	95610229/T//G	nonsynonymous	1	0
HSD17B3	chr9	99007623/T//C	synonymous	1	0
CORO2A	chr9	100888982/T//G	nonsynonymous	1	0
SVEP1	chr9	113213589/G//A	nonsynonymous	1	0
DNAJC25	chr9	114412197/T//G	nonsynonymous	1	0
RGS3	chr9	116359311/C//T	synonymous	1	0
POMT1	chr9	134396857/A//G	nonsynonymous	1	0
QSOX2	chr9	139107119/T//C	nonsynonymous	1	0
ITIH2	chr10	7772099/A//G	synonymous	1	0
DCLRE1C	chr10	14976415/ATAT//A	nonsynonymous	1	0
ENKUR	chr10	25273759/T//C	nonsynonymous	1	0
GAD2	chr10	26505588/C//T	synonymous	1	0
BMS1	chr10	43279985/A//G	nonsynonymous	1	0
CDH23	chr10	73562805/A//C	nonsynonymous	1	0
SPOCK2	chr10	73828052/A//G	nonsynonymous	1	0
ECD	chr10	74897757/T//G	synonymous	1	0
KAT6B	chr10	76744857/A//C	nonsynonymous	1	0
DLG5	chr10	79588632/AGACTTACCGCAAC//A	nonsynonymous	1	0
LRIT1	chr10	85991664/A//G	synonymous	1	0

HTR7	chr10	92503358/C//T	nonsynonymous	1	0
PPP1R3C	chr10	93390320/T//C	synonymous	1	0
BTAF1	chr10	93716974/T//A	synonymous	1	0
PSD	chr10	104176552/G//T	nonsynonymous	1	0
ZDHHC6	chr10	114205100/G//A	nonsynonymous	1	0
NRAP	chr10	115357780/A//G	nonsynonymous	1	0
ATRNL1	chr10	116881494/C//T	nonsynonymous	1	0
CCDC172	chr10	118100276/T//G	nonsynonymous	1	0
EMX2	chr10	119305166/T//G	nonsynonymous	1	0
NPS	chr10	129350730/G//A	nonsynonymous	1	0
ODF3	chr11	200034/C//T	synonymous	1	0
DNHD1	chr11	6560457/C//T	synonymous	1	0
STK33	chr11	8496460/A//C	synonymous	1	0
SBF2	chr11	9802059/T//C	nonsynonymous	1	0
ADM	chr11	10327219/T//C	synonymous	1	0
PLEKHA7	chr11	16863170/T//C	nonsynonymous	1	0
BDNF	chr11	27679477/T//A	nonsynonymous	1	0
HIPK3	chr11	33361086/T//G	synonymous	1	0
PAMR1	chr11	35492234/G//A	synonymous	1	0
FJX1	chr11	35641283/T//G	nonsynonymous	1	0
NUP160	chr11	47830007/A//C	synonymous	1	0
OR4C16	chr11	55340499/T//C	nonsynonymous	1	0
OR5L1	chr11	55579417/C//T	nonsynonymous	1	0
TMX2-CTNND1	chr11	57575861/G//A	nonsynonymous	1	0
OR5B17	chr11	58126404/T//G	nonsynonymous	1	0
	chr11	62259254/T//A	synonymous	1	0
CCDC88B	chr11	64120599/C//T	nonsynonymous	1	0
CCND1	chr11	69456028/C//T	synonymous	1	0
CLPB	chr11	72040784/T//G	nonsynonymous	1	0
NARS2	chr11	78154699/A//C	synonymous	1	0
MAML2	chr11	95712475/T//C	synonymous	1	0
PGR	chr11	100996782/A//C	nonsynonymous	1	0
MMP10	chr11	102647342/C//T	nonsynonymous	1	0
DYNC2H1	chr11	103106430/T//A	synonymous	1	0
APOA4	chr11	116692595/G//A	nonsynonymous	1	0
ATP5L	chr11	118272379/C//T	synonymous	1	0
HINFP	chr11	118997721/T//C	nonsynonymous	1	0
TECTA	chr11	121016795/G//A	nonsynonymous	1	0
TECTA	chr11	121037332/A//G	nonsynonymous	1	0
ZNF202	chr11	123597340/A//G	nonsynonymous	1	0
VWA5A	chr11	124013271/C//T	nonsynonymous	1	0
STT3A	chr11	125483068/C//T	nonsynonymous	1	0
	chr12	974423/A//C	synonymous	1	0
KCNA6	chr12	4920129/T//G	nonsynonymous	1	0
TAS2R7	chr12	10954293/T//G	synonymous	1	0
ATF7IP	chr12	14609557/C//T	synonymous	1	0
ART4	chr12	14993862/A//C	nonsynonymous	1	0
PLCZ1	chr12	18847846/T//G	synonymous	1	0
ITPR2	chr12	26592163/C//T	synonymous	1	0
KMT2D	chr12	49435769/C//T	nonsynonymous	1	0
KMT2D	chr12	49440390/A//T	nonsynonymous	1	0
RACGAP1	chr12	50386066/G//A	nonsynonymous	1	0
ANKRD33	chr12	52284486/G//A	synonymous	1	0
KRT75	chr12	52827011/T//G	nonsynonymous	1	0
KRT77	chr12	53086281/C//T	nonsynonymous	1	0
B4GALNT1	chr12	58021577/T//C	nonsynonymous	1	0
OS9	chr12	58109716/G//T	nonsynonymous	1	0
AGAP2	chr12	58125223/A//C	nonsynonymous	1	0
TBC1D30	chr12	65269392/T//C	nonsynonymous	1	0
PTPRQ	chr12	80899880/A//T	nonsynonymous	1	0
PTPRQ	chr12	80928719/C//T	nonsynonymous	1	0
TMTC2	chr12	83251012/A//G	nonsynonymous	1	0
PLXNC1	chr12	94641715/T//G	nonsynonymous	1	0
METAP2	chr12	95877030/A//C	nonsynonymous	1	0
TCHP	chr12	110340970/T//C	nonsynonymous	1	0
HECTD4	chr12	112664493/T//C	synonymous	1	0
P2RX7	chr12	121615145/T//A	nonsynonymous	1	0
NCOR2	chr12	124904573/T//C	nonsynonymous	1	0
GOLGA3	chr12	133381543/C//T	synonymous	1	0
SLC46A3	chr13	29275525/A//C	synonymous	1	0

CCNA1	chr13	37016808/T//C	synonymous	1	0
PROSER1	chr13	39585556/C//A	synonymous	1	0
NEK5	chr13	52639378/A//C	synonymous	1	0
MYCBP2	chr13	77632438/T//G	nonsynonymous	1	0
TUBGCP3	chr13	113209310/T//G	nonsynonymous	1	0
PROZ	chr13	113826243/A//G	nonsynonymous	1	0
TEP1	chr14	20845918/A//T	synonymous	1	0
TEP1	chr14	20848166/A//C	nonsynonymous	1	0
CHD8	chr14	21871295/C//T	nonsynonymous	1	0
CEBPE	chr14	23586802/C//T	nonsynonymous	1	0
PTGDR	chr14	52734787/T//C	synonymous	1	0
KIAA0586	chr14	58932588/T//G	nonsynonymous	1	0
GPR135	chr14	59931113/T//C	nonsynonymous	1	0
PLEKHH1	chr14	68052682/T//G	synonymous	1	0
PAPLN	chr14	73720567/C//T	synonymous	1	0
ENTPD5	chr14	74440675/T//C	nonsynonymous	1	0
KCNK13	chr14	90651353/A//T	synonymous	1	0
CATSPERB	chr14	92157898/A//G	nonsynonymous	1	0
SLC25A29	chr14	100765225/G//A	synonymous	1	0
DYNC1H1	chr14	102486300/G//A	nonsynonymous	1	0
TEX22	chr14	105878016/C//T	nonsynonymous	1	0
TMEM121	chr14	105995383/A//T	nonsynonymous	1	0
MGA	chr15	42052603/G//A	nonsynonymous	1	0
TP53BP1	chr15	43701129/C//T	nonsynonymous	1	0
DMXL2	chr15	51772760/A//C	nonsynonymous	1	0
AQP9	chr15	58467149/C//T	synonymous	1	0
AQP9	chr15	58467221/G//A	nonsynonymous	1	0
TLN2	chr15	63111820/G//A	nonsynonymous	1	0
THAP10	chr15	71184397/T//G	nonsynonymous	1	0
HYKK	chr15	78825590/A//C	nonsynonymous	1	0
SRRM2	chr16	2813049/T//G	synonymous	1	0
ZNF597	chr16	3486869/A//C	nonsynonymous	1	0
NLRC3	chr16	3613358/G//A	nonsynonymous	1	0
TMEM114	chr16	8619840/C//T	nonsynonymous	1	0
PMM2	chr16	8900241/G//A	synonymous	1	0
MYH11	chr16	15818194/A//G	nonsynonymous	1	0
CNGB1	chr16	57993918/G//C	nonsynonymous	1	0
CDYL2	chr16	80646565/G//T	synonymous	1	0
CENPBD1	chr16	90037903/A//G	nonsynonymous	1	0
NLRP1	chr17	5442808/T//C	nonsynonymous	1	0
KIAA0753	chr17	6531639/T//G	nonsynonymous	1	0
DNAH2	chr17	7733994/A//C	nonsynonymous	1	0
ALOX15B	chr17	7948594/T//A	synonymous	1	0
SLC47A1	chr17	19459356/T//C	nonsynonymous	1	0
GAS2L2	chr17	34073407/G//A	nonsynonymous	1	0
TAF15	chr17	34163131/C//T	synonymous	1	0
PCGF2	chr17	36894799/C//T	nonsynonymous	1	0
TUBG2	chr17	40818167/C//T	synonymous	1	0
SCRN2	chr17	45916189/T//A	nonsynonymous	1	0
MRPS23	chr17	55917131/G//A	synonymous	1	0
SDK2	chr17	71361417/T//C	nonsynonymous	1	0
SDK2	chr17	71398180/T//C	nonsynonymous	1	0
ITGB4	chr17	73752532/C//T	synonymous	1	0
JMJD6	chr17	74716544/G//A	synonymous	1	0
DNAH17	chr17	76437252/T//C	nonsynonymous	1	0
RIOK3	chr18	21059315/G//A	nonsynonymous	1	0
SLC39A6	chr18	33702226/G//A	nonsynonymous	1	0
ATP8B1	chr18	55319947/T//G	nonsynonymous	1	0
ZNF516	chr18	74092087/A//G	synonymous	1	0
C2CD4C	chr19	408054/A//G	nonsynonymous	1	0
ZFR2	chr19	3816721/G//A	nonsynonymous	1	0
PLIN4	chr19	4510758/T//C	nonsynonymous	1	0
KLHL26	chr19	18780067/C//T	synonymous	1	0
ZNF85	chr19	21132486/A//C	nonsynonymous	1	0
ZNF790	chr19	37310758/A//C	nonsynonymous	1	0
SARS2	chr19	39421431/T//G	synonymous	1	0
ZNF226	chr19	44681724/T//G	nonsynonymous	1	0
FBXO46	chr19	46215501/C//T	nonsynonymous	1	0
IGFL1	chr19	46734121/T//C	synonymous	1	0
SULT2A1	chr19	48385457/A//G	nonsynonymous	1	0

KLK6	chr19	51466699/T//A	nonsynonymous	1	0
ZNF577	chr19	52376939/T//G	synonymous	1	0
ZNF701	chr19	53085669/G//A	synonymous	1	0
LILRB4	chr19	55179530/G//T	synonymous	1	0
NLRP2	chr19	55497535/C//T	nonsynonymous	1	0
PPP1R12C	chr19	55606841/G//T	nonsynonymous	1	0
IL11	chr19	55880193/T//C	nonsynonymous	1	0
SLC27A5	chr19	59012697/G//A	synonymous	1	0
TRMT6	chr20	5927078/T//C	nonsynonymous	1	0
BFSP1	chr20	17474896/G//A	synonymous	1	0
INSM1	chr20	20350274/G//A	nonsynonymous	1	0
RALGAPA2	chr20	20621440/A//C	nonsynonymous	1	0
CNBD2	chr20	34563889/G//A	nonsynonymous	1	0
CHD6	chr20	40122261/T//A	nonsynonymous	1	0
TTPAL	chr20	43113046/T//C	nonsynonymous	1	0
DBNDD2	chr20	44035149/G//A	nonsynonymous	1	0
WFDC8	chr20	44184438/T//G	nonsynonymous	1	0
MMP9	chr20	44639224/C//T	synonymous	1	0
SULF2	chr20	46318989/C//T	synonymous	1	0
KCNG1	chr20	49626158/T//C	nonsynonymous	1	0
CDH26	chr20	58562546/C//A	synonymous	1	0
LIME1	chr20	62368882/C//T	synonymous	1	0
NCAM2	chr21	22746201/G//A	nonsynonymous	1	0
USP16	chr21	30422481/T//G	nonsynonymous	1	0
KRTAP8-1	chr21	32185373/T//C	nonsynonymous	1	0
TTC3-AS1	chr21	38560729/T//G	synonymous	1	0
ICOSLG	chr21	45655425/C//T	nonsynonymous	1	0
THOC5	chr22	29914991/T//G	synonymous	1	0
PES1	chr22	30987801/T//G	nonsynonymous	1	0
OSBP2	chr22	31266438/G//A	synonymous	1	0
APOL6	chr22	36054828/A//G	nonsynonymous	1	0
CBX6	chr22	39267500/T//G	nonsynonymous	1	0
FAM47B	chrX	34962126/G//A	nonsynonymous	1	0
RGN	chrX	46940685/C//A	nonsynonymous	1	0
PCSK1N	chrX	48690711/T//C	nonsynonymous	1	0
BMP15	chrX	50658994/T//G	nonsynonymous	1	0
PABPC5	chrX	90690938/C//T	nonsynonymous	1	0
TEX13B	chrX	107224403/G//T	synonymous	1	0
CHRDL1	chrX	109919473/T//G	nonsynonymous	1	0
LHFPL1	chrX	111874465/T//G	synonymous	1	0
RAB33A	chrX	129318486/C//T	synonymous	1	0
ARHGAP36	chrX	130219957/A//T	nonsynonymous	1	0
MAGEA1	chrX	152482536/C//T	nonsynonymous	1	0
C1QC	chr1	22974270/C//A	synonymous	1	0
GSTM1	chr1	110235893/G//A	synonymous	1	0
LRRC71	chr1	156902322/G//T	synonymous	1	0
FCRL3	chr1	157650789/C//A	nonsynonymous	1	0
CADM3	chr1	159166723/C//G	synonymous	1	0
PPOX	chr1	161140567/G//A	synonymous	1	0
ADCY10	chr1	167874258/C//A	nonsynonymous	1	0
DARS2	chr1	173808617/C//A	nonsynonymous	1	0
CACNA1E	chr1	181724484/A//T	nonsynonymous	1	0
RYR2	chr1	237619975/G//C	nonsynonymous	1	0
RYR2	chr1	237831224/G//T	nonsynonymous	1	0
ZBTB18	chr1	244218403/G//T	nonsynonymous	1	0
OR2W5	chr1	247655051/C//A	nonsynonymous	1	0
OR11L1	chr1	248004763/C//A	nonsynonymous	1	0
MAT2A	chr2	85769400/G//A	synonymous	1	0
LY75-CD302	chr2	160741801/C//G	nonsynonymous	1	0
GPR155	chr2	175337730/C//T	nonsynonymous	1	0
ABCA12	chr2	215917261/A//T	nonsynonymous	1	0
ABCA12	chr2	215917263/G//T	nonsynonymous	1	0
EIF4G1	chr3	184039328/C//T	nonsynonymous	1	0
FRYL	chr4	48567639/C//G	nonsynonymous	1	0
FRYL	chr4	48567647/G//A	synonymous	1	0
SEMA5A	chr5	9066732/C//A	synonymous	1	0
IGFBP3	chr7	45956183/G//T	synonymous	1	0
POLR2J	chr7	102119347/G//A	synonymous	1	0
ZNF596	chr8	193783/A//G	synonymous	1	0
DENND3	chr8	142151419/T//C	nonsynonymous	1	0

SFXN2	chr10	104491523/G//C	nonsynonymous	1	0
KCNH5	chr14	63447928/T//C	nonsynonymous	1	0
AREL1	chr14	75151298/C//G	nonsynonymous	1	0
CHTF18	chr16	845128/G//C	synonymous	1	0
ZNF75A	chr16	3367315/A//G	nonsynonymous	1	0
NDE1	chr16	15771723/G//A	synonymous	1	0
KDM8	chr16	27221820/G//T	nonsynonymous	1	0
ASPHD1	chr16	29917172/G//T	nonsynonymous	1	0
HYDIN	chr16	70884466/G//C	nonsynonymous	1	0
FOXL1	chr16	86612256/C//A	synonymous	1	0
CCDC47	chr17	61824197/C//G	synonymous	1	0
USH1G	chr17	72919028/C//A	synonymous	1	0
KHSRP	chr19	6415670/G//C	nonsynonymous	1	0
CC2D1A	chr19	14038711/G//C	synonymous	1	0
ZNF85	chr19	21132175/C//T	synonymous	1	0
BPIFC	chr22	32813103/AATGG//A	nonsynonymous	1	0
MSL3	chrX	11790811/G//T	nonsynonymous	1	0
MAGEB2	chrX	30237457/C//G	nonsynonymous	1	0
LHFPL1	chrX	111874810/C//A	nonsynonymous	1	0
CSAG1	chrX	151904489/C//A	synonymous	1	0
LIN28A	chr1	26737351/G//C	synonymous	0	1
TRPA1	chr8	72981375/G//T	nonsynonymous	0	1
VDAC2	chr10	76973835/G//C	synonymous	0	1
KRTAP5-9	chr11	71259676/C//T	synonymous	0	1
PTPRM	chr18	8387120/G//A	synonymous	0	1
MFSD12	chr19	3548214/G//C	synonymous	0	1
CACNA1A	chr19	13470535/T//C	nonsynonymous	0	1
ZNF335	chr20	44594349/G//C	synonymous	0	1
LRRIQ3	chr1	74507578/T//C	nonsynonymous	0	1
OR10R2	chr1	158450142/C//T	nonsynonymous	0	1
NBAS	chr2	15514798/C//T	nonsynonymous	0	1
ZFP36L2	chr2	43453448/T//C	nonsynonymous	0	1
STEAP3	chr2	120005382/G//A	nonsynonymous	0	1
ALCAM	chr3	105266240/C//A	nonsynonymous	0	1
DNAJC13	chr3	132235592/C//A	nonsynonymous	0	1
LDB2	chr4	16760830/G//A	synonymous	0	1
DNAH5	chr5	13824421/G//A	nonsynonymous	0	1
TUSC3	chr8	15480637/C//T	nonsynonymous	0	1
HGSNAT	chr8	43054688/AAAG//A	nonsynonymous	0	1
ADARB2	chr10	1245965/C//T	nonsynonymous	0	1
REP15	chr12	27849621/A//G	synonymous	0	1
PDZRN4	chr12	41966716/C//G	nonsynonymous	0	1
KRT5	chr12	52910513/C//A	synonymous	0	1
CALCOCO1	chr12	54108408/T//C	nonsynonymous	0	1
GCN1	chr12	120587792/C//T	synonymous	0	1
ATP6V0A2	chr12	124228862/C//T	nonsynonymous	0	1
TMEM132C	chr12	128899980/G//A	synonymous	0	1
EP400	chr12	132490745/C//T	synonymous	0	1
PAPD5	chr16	50251527/G//A	nonsynonymous	0	1
ADCYAP1	chr18	908344/G//A	synonymous	0	1
ZIC3	chrX	136649891/C//A	synonymous	0	1
MAP3K6	chr1	27690535/G//A	nonsynonymous	1	0
KPNA6	chr1	32620212/G//T	nonsynonymous	1	0
DMBX1	chr1	46976778/G//A	nonsynonymous	1	0
HRNR	chr1	152191848/C//A	nonsynonymous	1	0
SLC27A3	chr1	153750309/C//T	nonsynonymous	1	0
DCST1	chr1	155007173/CAGA//C	nonsynonymous	1	0
SMG5	chr1	156235701/G//A	nonsynonymous	1	0
	chr1	182775597/C//T	synonymous	1	0
ASPM	chr1	197115463/G//A	synonymous	1	0
CTSE	chr1	206331215/C//T	nonsynonymous	1	0
USH2A	chr1	216497016/G//C	synonymous	1	0
OBSCN	chr1	228404405/T//A	nonsynonymous	1	0
DISC1	chr1	231830151/C//T	nonsynonymous	1	0
PCNX2	chr1	233363026/C//T	nonsynonymous	1	0
OR2M2	chr1	248343299/G//A	synonymous	1	0
ADCY3	chr2	25057656/C//T	synonymous	1	0
KCNK3	chr2	26951326/C//T	nonsynonymous	1	0
CHAC2	chr2	54001675/A//C	synonymous	1	0
RFX8	chr2	102029503/G//A	nonsynonymous	1	0

ORC2	chr2	201796156/A//T	nonsynonymous	1	0
FZD7	chr2	202900122/G//A	nonsynonymous	1	0
NRP2	chr2	206608199/C//T	nonsynonymous	1	0
DNER	chr2	230453149/C//T	nonsynonymous	1	0
COL6A3	chr2	238280774/G//A	nonsynonymous	1	0
MLH1	chr3	37092014/G//A	nonsynonymous	1	0
KLHL40	chr3	42727780/G//A	nonsynonymous	1	0
SCAP	chr3	47456081/G//A	nonsynonymous	1	0
DOCK3	chr3	51367632/T//A	nonsynonymous	1	0
STAB1	chr3	52555658/C//T	synonymous	1	0
ROBO1	chr3	78987805/C//A	nonsynonymous	1	0
SENP7	chr3	101117864/G//A	nonsynonymous	1	0
NFKBIZ	chr3	101576208/C//T	nonsynonymous	1	0
PLXND1	chr3	129324809/C//T	nonsynonymous	1	0
PLS1	chr3	142395004/G//C	nonsynonymous	1	0
GPR171	chr3	150917221/A//T	synonymous	1	0
IGSF10	chr3	151164666/T//C	nonsynonymous	1	0
PCDH7	chr4	30723062/C//A	synonymous	1	0
PPP3CA	chr4	101947109/G//A	synonymous	1	0
ANK2	chr4	114257148/G//A	nonsynonymous	1	0
SYNPO2	chr4	119952612/G//T	nonsynonymous	1	0
	chr4	122103898/G//A	synonymous	1	0
SLC45A2	chr5	33944808/G//A	nonsynonymous	1	0
OXCT1	chr5	41862853/C//T	nonsynonymous	1	0
MAST4	chr5	66459542/G//A	nonsynonymous	1	0
ADGRV1	chr5	89938502/C//T	synonymous	1	0
GPLD1	chr6	24448431/G//A	synonymous	1	0
UNC5CL	chr6	40998408/C//T	synonymous	1	0
TMEM151B	chr6	44243538/G//A	synonymous	1	0
KHDRBS2	chr6	62995819/G//A	nonsynonymous	1	0
RIMS1	chr6	73110409/G//A	nonsynonymous	1	0
FAM46A	chr6	82461740/C//T	nonsynonymous	1	0
CD164	chr6	109703584/T//G	synonymous	1	0
RPF2	chr6	111306282/G//A	nonsynonymous	1	0
FRK	chr6	116381344/C//T	nonsynonymous	1	0
C6orf58	chr6	127899823/T//G	synonymous	1	0
SLC2A12	chr6	134349932/T//C	nonsynonymous	1	0
FBXO30	chr6	146125797/C//T	nonsynonymous	1	0
NPC1L1	chr7	44579949/G//A	synonymous	1	0
RELN	chr7	103389945/A//G	nonsynonymous	1	0
LHFPL3	chr7	104377280/A//T	nonsynonymous	1	0
PRSS1	chr7	142459785/G//A	nonsynonymous	1	0
DLC1	chr8	13357342/T//C	nonsynonymous	1	0
NEFM	chr8	24771385/G//A	nonsynonymous	1	0
ADAM9	chr8	38934808/G//A	nonsynonymous	1	0
CHD7	chr8	61748802/C//T	nonsynonymous	1	0
CNBD1	chr8	88298763/T//A	synonymous	1	0
KIAA1429	chr8	95518786/A//G	nonsynonymous	1	0
UBR5	chr8	103310700/G//A	nonsynonymous	1	0
ARC	chr8	143694892/G//A	synonymous	1	0
CCDC166	chr8	144790006/C//T	nonsynonymous	1	0
SCRT1	chr8	145557658/G//A	nonsynonymous	1	0
KIAA1161	chr9	34371539/C//T	nonsynonymous	1	0
TLN1	chr9	35704085/G//A	nonsynonymous	1	0
ITGA8	chr10	15648327/T//G	nonsynonymous	1	0
	chr10	21462794/G//T	synonymous	1	0
KIAA1462	chr10	30315575/G//A	nonsynonymous	1	0
SIRT1	chr10	69676110/T//C	synonymous	1	0
SRGN	chr10	70863861/G//T	nonsynonymous	1	0
TLL2	chr10	98155015/T//C	nonsynonymous	1	0
TLL2	chr10	98180733/G//A	synonymous	1	0
PKD2L1	chr10	102054363/C//T	nonsynonymous	1	0
VWA2	chr10	116032663/CTG//C	nonsynonymous	1	0
SHTN1	chr10	118645924/G//A	synonymous	1	0
MKI67	chr10	129906957/C//T	synonymous	1	0
MUC5B	chr11	1261106/T//C	nonsynonymous	1	0
RASGRP2	chr11	64508433/C//T	nonsynonymous	1	0
ATF7IP	chr12	14613698/CCATCTGG//C	nonsynonymous	1	0
CNTN1	chr12	41414150/G//A	nonsynonymous	1	0
MARS	chr12	57892216/C//T	nonsynonymous	1	0

OTOGL	chr12	80722869/C//T	nonsynonymous	1	0
VEZT	chr12	95645777/C//G	nonsynonymous	1	0
ACACB	chr12	109703292/G//A	synonymous	1	0
MTMR6	chr13	25831889/T//A	synonymous	1	0
FRY	chr13	32745389/C//T	synonymous	1	0
FRY	chr13	32745391/A//T	nonsynonymous	1	0
NRXN3	chr14	79175719/C//T	nonsynonymous	1	0
SECISBP2L	chr15	49329905/A//C	nonsynonymous	1	0
ZNF609	chr15	64966371/C//T	nonsynonymous	1	0
RHBDF1	chr16	112593/G//A	nonsynonymous	1	0
TELO2	chr16	1547388/C//T	nonsynonymous	1	0
BICDL2	chr16	3085444/G//T	synonymous	1	0
LOC81691	chr16	20838444/C//T	nonsynonymous	1	0
CORO1A	chr16	30198730/C//T	nonsynonymous	1	0
DCTPP1	chr16	30435691/G//T	synonymous	1	0
ALOX12	chr17	6900337/G//A	nonsynonymous	1	0
SLC46A1	chr17	26732283/G//A	synonymous	1	0
ARHGAP27	chr17	43473163/C//T	synonymous	1	0
LOXHD1	chr18	44181306/G//A	synonymous	1	0
CSNK1G2	chr19	1980366/C//T	synonymous	1	0
RGL3	chr19	11508124/G//T	synonymous	1	0
RTBDN	chr19	12936599/C//T	nonsynonymous	1	0
MAU2	chr19	19453581/C//T	synonymous	1	0
ZNF676	chr19	22363448/A//T	synonymous	1	0
KCTD15	chr19	34292194/C//T	synonymous	1	0
SIPA1L3	chr19	38696757/G//A	synonymous	1	0
LGALS4	chr19	39299421/A//G	nonsynonymous	1	0
ZFP36	chr19	39899240/T//C	synonymous	1	0
TGFB1	chr19	41850657/C//T	nonsynonymous	1	0
SYMPK	chr19	46351096/C//T	nonsynonymous	1	0
PPP1R15A	chr19	49377112/A//G	nonsynonymous	1	0
IL4I1	chr19	50394668/C//T	synonymous	1	0
ADNP	chr20	49509223/G//A	synonymous	1	0
TCEA2	chr20	62701931/G//A	synonymous	1	0
TXNRD2	chr22	19867719/C//T	nonsynonymous	1	0
RHBDD3	chr22	29659912/C//A	synonymous	1	0
CARD10	chr22	37887405/C//T	nonsynonymous	1	0
PLA2G6	chr22	38512196/A//G	nonsynonymous	1	0
MKL1	chr22	40814523/A//G	nonsynonymous	1	0
CYB5R3	chr22	43023685/G//A	nonsynonymous	1	0
CSF2RA	chrX	1413250/G//A	nonsynonymous	1	0
AKAP17A	chrX	1719668/C//T	synonymous	1	0
ELK1	chrX	47497393/G//A	synonymous	1	0
AMER1	chrX	63411798/G//A	nonsynonymous	1	0
ZC3H12B	chrX	64722262/A//G	nonsynonymous	1	0
	chrX	65242605/T//G	synonymous	1	0
TGIF2LX	chrX	89177810/A//C	nonsynonymous	1	0
IL1RAPL2	chrX	104440380/G//A	nonsynonymous	1	0
RIPPLY1	chrX	106144031/T//C	nonsynonymous	1	0
TEX13C	chrX	124454244/G//A	nonsynonymous	1	0
IGSF1	chrX	130410108/T//G	nonsynonymous	1	0
FGF13	chrX	137715110/C//T	synonymous	1	0
ATP11C	chrX	138845544/G//T	nonsynonymous	1	0
SSR4	chrX	153060020/C//T	synonymous	1	0
SPOCD1	chr1	32258877/A//T	nonsynonymous	1	0
MACF1	chr1	39913742/A//C	nonsynonymous	1	0
WLS	chr1	68591905/C//CCTCCT	synonymous	1	0
SWT1	chr1	185151113/G//A	synonymous	1	0
SWT1	chr1	185240469/A//T	nonsynonymous	1	0
PLA2G4A	chr1	186916083/G//A	synonymous	1	0
LYPLAL1	chr1	219366572/A//C	nonsynonymous	1	0
RYR2	chr1	237693773/G//A	nonsynonymous	1	0
TMEM178A	chr2	39944209/C//T	nonsynonymous	1	0
SMYD1	chr2	88402588/A//T	nonsynonymous	1	0
FABP1	chr2	88427580/C//A	synonymous	1	0
ADRA2B	chr2	96781763/C//T	synonymous	1	0
TFCP2L1	chr2	121991681/T//C	nonsynonymous	1	0
R3HDM1	chr2	136467790/A//C	nonsynonymous	1	0
KIF5C	chr2	149864549/A//T	nonsynonymous	1	0
GALNT5	chr2	158115048/G//A	nonsynonymous	1	0

KCNH7	chr2	163228456/T//G	nonsynonymous	1	0
SPEG	chr2	220342687/C//T	synonymous	1	0
TRIP12	chr2	230723907/G//T	nonsynonymous	1	0
KIAA1143	chr3	44802993/C//G	nonsynonymous	1	0
SETD2	chr3	47098913/G//A	nonsynonymous	1	0
GPR62	chr3	51990578/C//T	nonsynonymous	1	0
OR5K4	chr3	98073061/C//T	nonsynonymous	1	0
PLXNA1	chr3	126749203/G//T	nonsynonymous	1	0
CPNE4	chr3	131261462/G//T	nonsynonymous	1	0
MED12L	chr3	150883738/T//A	nonsynonymous	1	0
SMCO1	chr3	196234948/T//C	nonsynonymous	1	0
FAM193A	chr4	2701815/A//T	nonsynonymous	1	0
NSG1	chr4	4418973/C//T	synonymous	1	0
N4BP2	chr4	40121965/T//G	nonsynonymous	1	0
MTTP	chr4	100496001/T//A	synonymous	1	0
FHDC1	chr4	153896911/A//G	nonsynonymous	1	0
MTNR1A	chr4	187455633/A//G	nonsynonymous	1	0
IL7R	chr5	35873736/T//G	nonsynonymous	1	0
SREK1	chr5	65458311/G//A	nonsynonymous	1	0
VCAN	chr5	82816528/A//G	synonymous	1	0
IL13	chr5	131993869/C//T	synonymous	1	0
PCDHAC1	chr5	140307087/A//G	nonsynonymous	1	0
CYFIP2	chr5	156742108/T//C	synonymous	1	0
CAGE1	chr6	7387208/A//G	synonymous	1	0
OR10C1	chr6	29408515/C//T	synonymous	1	0
PPP1R10	chr6	30569403/T//A	nonsynonymous	1	0
ADGRB3	chr6	69653736/G//A	nonsynonymous	1	0
KCNQ5	chr6	73904449/C//T	nonsynonymous	1	0
SASH1	chr6	148711287/C//G	nonsynonymous	1	0
PDE1C	chr7	31913008/C//T	nonsynonymous	1	0
ZPBP	chr7	50022971/C//T	nonsynonymous	1	0
HTRA4	chr8	38834250/G//C	nonsynonymous	1	0
PENK	chr8	57358471/G//A	synonymous	1	0
RBM12B	chr8	94747664/T//C	synonymous	1	0
SDC2	chr8	97620641/A//G	nonsynonymous	1	0
MTBP	chr8	121458666/T//G	synonymous	1	0
NDRG1	chr8	134292489/C//A	nonsynonymous	1	0
RFX3	chr9	3262966/T//A	nonsynonymous	1	0
	chr9	21994138/C//T	synonymous	1	0
VCP	chr9	35066793/C//A	synonymous	1	0
NTRK2	chr9	87563472/T//A	nonsynonymous	1	0
ZNF782	chr9	99581918/A//G	synonymous	1	0
C9orf84	chr9	114490159/G//A	nonsynonymous	1	0
HERC4	chr10	69785324/A//T	nonsynonymous	1	0
MUC6	chr11	1015931/C//G	synonymous	1	0
SYT13	chr11	45307594/C//A	nonsynonymous	1	0
RNASEH2C	chr11	65486735/A//C	synonymous	1	0
TMTC1	chr12	29709864/C//G	nonsynonymous	1	0
RAPGEF3	chr12	48143552/C//T	nonsynonymous	1	0
KRT6B	chr12	52842665/G//C	nonsynonymous	1	0
OR6C2	chr12	55846002/A//C	nonsynonymous	1	0
PPFIA2	chr12	81675223/C//A	nonsynonymous	1	0
MLNR	chr13	49795287/C//T	nonsynonymous	1	0
PCDH20	chr13	61986963/G//A	synonymous	1	0
FBXL3	chr13	77581404/GA//G	nonsynonymous	1	0
C14orf28	chr14	45369698/A//G	synonymous	1	0
ARID4A	chr14	58817925/A//G	synonymous	1	0
DYNC1H1	chr14	102500720/GGCAGGCCA//G	nonsynonymous	1	0
AHNAK2	chr14	105418765/A//C	nonsynonymous	1	0
GALK2	chr15	49620332/G//A	synonymous	1	0
ABHD17C	chr15	81046805/C//T	synonymous	1	0
CACNA1H	chr16	1255202/G//A	nonsynonymous	1	0
	chr16	7743333/G//T	synonymous	1	0
TNRC6A	chr16	24802056/T//G	nonsynonymous	1	0
ZNF668	chr16	31075771/C//A	nonsynonymous	1	0
BRD7	chr16	50354641/T//G	nonsynonymous	1	0
HOXB3	chr17	46627978/C//A	synonymous	1	0
DCAF7	chr17	61627962/C//G	synonymous	1	0
FN3KRP	chr17	80674699/G//C	nonsynonymous	1	0
NETO1	chr18	70417644/G//A	synonymous	1	0

WDR83	chr19	12781614/C//T	nonsynonymous	1	0
ZNF569	chr19	37903571/G//A	synonymous	1	0
RASGRP4	chr19	38916755/C//T	synonymous	1	0
FKRP	chr19	47259744/C//G	nonsynonymous	1	0
RUVBL2	chr19	49510569/G//T	nonsynonymous	1	0
VSIG10L	chr19	51844525/C//A	nonsynonymous	1	0
GINS1	chr20	25397736/T//A	synonymous	1	0
E2F1	chr20	32266007/C//T	nonsynonymous	1	0
OSBPL2	chr20	60854364/C//T	nonsynonymous	1	0
NCAM2	chr21	22746308/A//G	synonymous	1	0
IFNAR1	chr21	34713414/A//G	nonsynonymous	1	0
FANCB	chrX	14871260/C//T	synonymous	1	0
BEND2	chrX	18198725/T//C	nonsynonymous	1	0
DCAF8L2	chrX	27765619/C//T	nonsynonymous	1	0
TENM1	chrX	123517991/C//G	nonsynonymous	1	0
SRRM1	chr1	24973166/G//C	nonsynonymous	1	0
PHACTR4	chr1	28793031/C//G	nonsynonymous	1	0
TRNAU1AP	chr1	28891255/G//A	nonsynonymous	1	0
GRIK3	chr1	37315930/G//C	synonymous	1	0
GRIK3	chr1	37324849/C//T	nonsynonymous	1	0
SLC6A9	chr1	44474146/C//T	nonsynonymous	1	0
PTGER3	chr1	71512884/G//T	nonsynonymous	1	0
GSTM4	chr1	110203836/A//G	nonsynonymous	1	0
TNFAIP8L2	chr1	151131185/C//T	synonymous	1	0
SHC1	chr1	154942861/G//A	nonsynonymous	1	0
IQGAP3	chr1	156498731/C//T	synonymous	1	0
CFAP45	chr1	159847181/C//G	nonsynonymous	1	0
FCGR3B	chr1	161601001/C//A	synonymous	1	0
TEDDM1	chr1	182369476/C//G	nonsynonymous	1	0
LGR6	chr1	202287707/C//T	nonsynonymous	1	0
ACBD3	chr1	226352573/G//A	synonymous	1	0
OBSCN	chr1	228401898/C//G	nonsynonymous	1	0
KIDINS220	chr2	8877070/T//C	synonymous	1	0
GREB1	chr2	11773008/A//C	synonymous	1	0
STON1	chr2	48809445/A//G	nonsynonymous	1	0
DYSF	chr2	71816779/C//A	nonsynonymous	1	0
EIF5B	chr2	100013254/A//G	nonsynonymous	1	0
NCKAP5	chr2	133540750/C//T	nonsynonymous	1	0
NCKAP5	chr2	133541662/C//G	nonsynonymous	1	0
NCKAP5	chr2	133541717/C//G	nonsynonymous	1	0
NOSTRIN	chr2	169711966/G//C	nonsynonymous	1	0
GPR155	chr2	175346504/T//C	nonsynonymous	1	0
ANKRD44	chr2	198011781/C//T	nonsynonymous	1	0
MPP4	chr2	202546280/C//G	nonsynonymous	1	0
IKZF2	chr2	214012489/C//G	nonsynonymous	1	0
NYAP2	chr2	226446806/G//A	nonsynonymous	1	0
DGKD	chr2	234358003/C//T	synonymous	1	0
TGFBR2	chr3	30648460/C//T	nonsynonymous	1	0
GLB1	chr3	33058248/C//A	nonsynonymous	1	0
SLC6A20	chr3	45812753/G//C	nonsynonymous	1	0
BAP1	chr3	52438535/G//C	nonsynonymous	1	0
MLF1	chr3	158317802/G//A	nonsynonymous	1	0
MLF1	chr3	158317826/G//A	nonsynonymous	1	0
KCNMB2	chr3	178560503/C//T	synonymous	1	0
GNB4	chr3	179131510/T//C	nonsynonymous	1	0
HES1	chr3	193854434/G//A	nonsynonymous	1	0
ZFYVE28	chr4	2306215/C//T	nonsynonymous	1	0
LYAR	chr4	4276301/C//G	nonsynonymous	1	0
MUC7	chr4	71339777/G//A	synonymous	1	0
BMP2K	chr4	79792148/G//A	synonymous	1	0
MEPE	chr4	88767356/G//A	nonsynonymous	1	0
MEPE	chr4	88767581/G//A	nonsynonymous	1	0
FBXW7	chr4	153247366/C//G	nonsynonymous	1	0
FASTKD3	chr5	7867559/A//G	nonsynonymous	1	0
PRKAA1	chr5	40762951/G//A	nonsynonymous	1	0
CENPH	chr5	68505608/G//C	nonsynonymous	1	0
SLC22A4	chr5	131630440/G//A	nonsynonymous	1	0
RXRB	chr6	33162601/G//A	nonsynonymous	1	0
WDR46	chr6	33254933/G//GC	nonsynonymous	1	0
C6orf1	chr6	34214856/C//T	synonymous	1	0

MTCH1	chr6	36949389/G//A	synonymous	1	0
CAPN11	chr6	44150963/A//G	nonsynonymous	1	0
KHDRBS2	chr6	62995888/G//C	synonymous	1	0
PHF3	chr6	64413542/C//G	synonymous	1	0
PEX7	chr6	137147517/C//T	synonymous	1	0
HOXA2	chr7	27141739/G//C	synonymous	1	0
TAX1BP1	chr7	27831732/C//T	synonymous	1	0
PDE1C	chr7	31793081/G//T	nonsynonymous	1	0
NME8	chr7	37934089/A//T	nonsynonymous	1	0
POM121L12	chr7	53104256/G//C	synonymous	1	0
PCLO	chr7	82585054/C//A	nonsynonymous	1	0
CROT	chr7	87011387/C//T	synonymous	1	0
TRIM56	chr7	100731925/C//T	synonymous	1	0
POT1	chr7	124537229/G//C	synonymous	1	0
ADCK2	chr7	140374537/G//C	nonsynonymous	1	0
RHOBTB2	chr8	22863632/C//G	synonymous	1	0
UNC5D	chr8	35406896/T//C	nonsynonymous	1	0
SLC20A2	chr8	42287734/C//T	synonymous	1	0
RP1	chr8	55533600/G//A	nonsynonymous	1	0
RRM2B	chr8	103225048/C//T	nonsynonymous	1	0
RIC1	chr9	5762534/A//T	synonymous	1	0
DAPK1	chr9	90321209/G//A	nonsynonymous	1	0
SH3GLB2	chr9	131777154/C//A	nonsynonymous	1	0
SETX	chr9	135171391/C//T	nonsynonymous	1	0
SVIL	chr10	29818745/T//C	nonsynonymous	1	0
BMPR1A	chr10	88679109/A//G	nonsynonymous	1	0
SLF2	chr10	102689692/T//C	synonymous	1	0
HSPA12A	chr10	118443409/G//C	nonsynonymous	1	0
VAX1	chr10	118893684/C//A	synonymous	1	0
EDRF1	chr10	127434298/C//T	synonymous	1	0
ARHGAP1	chr11	46701004/G//A	nonsynonymous	1	0
PELI3	chr11	66243306/G//A	nonsynonymous	1	0
SIK2	chr11	111591362/C//T	synonymous	1	0
OR10G7	chr11	123909311/T//C	nonsynonymous	1	0
ARHGAP32	chr11	128843276/G//C	nonsynonymous	1	0
JAM3	chr11	134014156/G//A	nonsynonymous	1	0
GLB1L2	chr11	134238660/G//T	nonsynonymous	1	0
	chr12	977794/G//T	synonymous	1	0
DIP2B	chr12	51112541/T//C	synonymous	1	0
TBC1D30	chr12	65264496/G//T	nonsynonymous	1	0
GRIP1	chr12	67072646/C//A	synonymous	1	0
ANKS1B	chr12	99640634/A//G	nonsynonymous	1	0
GNPTAB	chr12	102174023/A//T	nonsynonymous	1	0
TMEM132C	chr12	129100751/C//G	nonsynonymous	1	0
PCDH20	chr13	61989195/G//A	nonsynonymous	1	0
CCNB1IP1	chr14	20779574/C//G	synonymous	1	0
SLC7A8	chr14	23598961/G//C	synonymous	1	0
BCL2L2-PABPN1	chr14	23793256/A//G	nonsynonymous	1	0
MYH7	chr14	23884470/T//A	nonsynonymous	1	0
ATP6V1D	chr14	67817358/G//C	nonsynonymous	1	0
SMOC1	chr14	70418979/G//A	nonsynonymous	1	0
GOLGA5	chr14	93275790/G//A	synonymous	1	0
ASPG	chr14	104571795/C//T	synonymous	1	0
INF2	chr14	105167964/G//A	nonsynonymous	1	0
TJP1	chr15	30058552/G//A	nonsynonymous	1	0
BAHD1	chr15	40757624/G//C	nonsynonymous	1	0
DMXL2	chr15	51839541/T//C	nonsynonymous	1	0
VWA9	chr15	65877204/G//A	synonymous	1	0
CYP1A2	chr15	75042130/C//G	synonymous	1	0
	chr15	88522620/C//T	synonymous	1	0
MCTP2	chr15	94841792/G//A	nonsynonymous	1	0
SRCAP	chr16	30749083/C//T	synonymous	1	0
CMTM2	chr16	66613997/C//G	nonsynonymous	1	0
ZNF821	chr16	71894380/T//C	synonymous	1	0
COTL1	chr16	84623758/G//A	nonsynonymous	1	0
IGF2BP1	chr17	47126713/G//A	nonsynonymous	1	0
TSPOAP1	chr17	56388201/G//C	nonsynonymous	1	0
RNF43	chr17	56448361/C//A	nonsynonymous	1	0
HEATR6	chr17	58151235/C//T	nonsynonymous	1	0
RNF157	chr17	74163136/C//A	nonsynonymous	1	0

OXLD1	chr17	79632518/C//A	nonsynonymous	1	0
PPP4R1	chr18	9549327/C//T	nonsynonymous	1	0
BSG	chr19	577824/G//C	nonsynonymous	1	0
C19orf35	chr19	2275759/C//G	nonsynonymous	1	0
MUC16	chr19	9064690/A//G	nonsynonymous	1	0
ZNF266	chr19	9524719/C//T	synonymous	1	0
EPS15L1	chr19	16472589/C//A	nonsynonymous	1	0
LTBP4	chr19	41112402/C//G	nonsynonymous	1	0
PLAUR	chr19	44153021/G//A	synonymous	1	0
GLTSCR1	chr19	48197973/G//T	nonsynonymous	1	0
ZSCAN5A	chr19	56735186/G//A	synonymous	1	0
FASTKD5	chr20	3128084/G//A	synonymous	1	0
RIN2	chr20	19945654/C//G	nonsynonymous	1	0
RBL1	chr20	35651206/G//C	synonymous	1	0
WFDC3	chr20	44416566/G//T	nonsynonymous	1	0
ZNFX1	chr20	47865468/C//T	nonsynonymous	1	0
TMEM189-UBE2V1	chr20	48744682/C//G	nonsynonymous	1	0
CCT8	chr21	30433612/G//A	nonsynonymous	1	0
VPREB1	chr22	22599567/C//G	nonsynonymous	1	0
NEFH	chr22	29885108/G//T	nonsynonymous	1	0
	chr22	38155476/A//T	synonymous	1	0
FAM227A	chr22	39032603/C//T	nonsynonymous	1	0
ENTHD1	chr22	40140040/G//A	synonymous	1	0
CERK	chr22	47103854/T//A	nonsynonymous	1	0
PLXNB2	chr22	50725658/G//A	synonymous	1	0
PRPS2	chrX	12817374/G//C	nonsynonymous	1	0
ZFX	chrX	24228302/T//C	synonymous	1	0
SHROOM4	chrX	50376942/C//G	nonsynonymous	1	0
CENPI	chrX	100382211/C//G	nonsynonymous	1	0
RAB40A	chrX	102755419/C//G	nonsynonymous	1	0
POLR1B	chr2	113300010/G//C	synonymous	1	0
OR5K1	chr3	98189141/T//C	nonsynonymous	1	0
PCDHB16	chr5	140563574/A//G	synonymous	1	0
AHNAK	chr11	62296098/A//G	synonymous	1	0
TMEM241	chr18	21017767/A//G	nonsynonymous	1	0
SERBP1	chr1	67895862/GCTT//G	nonsynonymous	0	1
BAZ2B	chr2	160294939/C//T	nonsynonymous	0	1
ZNF662	chr3	42956779/A//G	nonsynonymous	0	1
STXBP5L	chr3	120764365/T//G	synonymous	0	1
GMPS	chr3	155654229/T//G	nonsynonymous	0	1
CCKAR	chr4	26483554/G//A	synonymous	0	1
PROB1	chr5	138728758/C//G	synonymous	0	1
HIPK3	chr11	33308847/T//C	nonsynonymous	0	1
CUX2	chr12	111772390/C//T	synonymous	0	1
WDR66	chr12	122359412/G//A	synonymous	0	1
GPR12	chr13	27333859/C//T	nonsynonymous	0	1
FLT3	chr13	28597488/G//A	nonsynonymous	0	1
NBEA	chr13	35630144/A//T	synonymous	0	1
BTBD7	chr14	93712622/T//G	nonsynonymous	0	1
NEDD4L	chr18	56008313/G//A	nonsynonymous	0	1
REXO1	chr19	1816713/C//T	nonsynonymous	0	1
AP2A1	chr19	50304309/G//T	nonsynonymous	0	1
KRTAP10-11	chr21	46066655/C//T	nonsynonymous	0	1
FOXRED2	chr22	36902196/C//T	nonsynonymous	0	1
BRD1	chr22	50216641/G//A	nonsynonymous	0	1
HDX	chrX	83723705/C//A	nonsynonymous	0	1
AJAP1	chr1	4772468/T//G	nonsynonymous	0	1
MASP2	chr1	11090239/C//T	nonsynonymous	0	1
ORC1	chr1	52854133/G//A	nonsynonymous	0	1
NRAS	chr1	115256529/T//A	nonsynonymous	0	1
FLG	chr1	152284341/A//G	synonymous	0	1
CLK2	chr1	155238138/C//G	nonsynonymous	0	1
OR6P1	chr1	158532554/T//C	nonsynonymous	0	1
PYHIN1	chr1	158908242/G//A	synonymous	0	1
F5	chr1	169519237/C//T	nonsynonymous	0	1
TNN	chr1	175048512/G//A	synonymous	0	1
PAPPA2	chr1	176709217/T//G	nonsynonymous	0	1
LMOD1	chr1	201869656/C//T	nonsynonymous	0	1
LGR6	chr1	202266710/C//T	synonymous	0	1
TLR5	chr1	223285735/T//C	synonymous	0	1

COQ8A	chr1	227153010/T//G	nonsynonymous	0	1
KCNS3	chr2	18113742/A//C	synonymous	0	1
C1GALT1C1L	chr2	43902572/C//T	nonsynonymous	0	1
SERTAD2	chr2	64863684/T//C	nonsynonymous	0	1
PLEK	chr2	68620372/G//T	nonsynonymous	0	1
SNRPG	chr2	70515204/A//G	nonsynonymous	0	1
PAIP2B	chr2	71417053/C//A	synonymous	0	1
RPIA	chr2	89036166/T//A	synonymous	0	1
GLI2	chr2	121736103/T//C	nonsynonymous	0	1
THSD7B	chr2	137928415/C//T	nonsynonymous	0	1
ZEB2	chr2	145147113/C//G	nonsynonymous	0	1
ERBB4	chr2	212426640/G//C	synonymous	0	1
PTPRN	chr2	220174086/C//T	synonymous	0	1
CHPF	chr2	220404338/C//G	nonsynonymous	0	1
HTR2B	chr2	231973849/C//T	synonymous	0	1
GBX2	chr2	237074550/T//C	synonymous	0	1
AGXT	chr2	241812439/G//A	nonsynonymous	0	1
BRK1	chr3	10157335/G//A	synonymous	0	1
TOP2B	chr3	25665193/A//G	nonsynonymous	0	1
PHLDB2	chr3	111632285/G//A	synonymous	0	1
H1FOO	chr3	129262086/G//A	nonsynonymous	0	1
SI	chr3	164739065/T//G	nonsynonymous	0	1
MUC4	chr3	195512982/G//C	synonymous	0	1
ABLIM2	chr4	7968660/G//A	synonymous	0	1
ATP8A1	chr4	42416704/C//T	nonsynonymous	0	1
SLC4A4	chr4	72332167/T//C	synonymous	0	1
LAMTOR3	chr4	100808515/G//T	synonymous	0	1
CLGN	chr4	141320019/G//T	synonymous	0	1
DCHS2	chr4	155155671/T//C	synonymous	0	1
SEMA5A	chr5	9197321/A//C	nonsynonymous	0	1
ANKRD33B	chr5	10618448/G//A	nonsynonymous	0	1
PDE6A	chr5	149274859/A//G	synonymous	0	1
HMP19	chr5	173473876/G//A	nonsynonymous	0	1
HK3	chr5	176310821/A//G	nonsynonymous	0	1
GMPR	chr6	16279125/A//T	synonymous	0	1
ENPP4	chr6	46107400/A//C	nonsynonymous	0	1
ANKRD6	chr6	90326335/A//T	nonsynonymous	0	1
PTPRK	chr6	128294223/C//T	nonsynonymous	0	1
UTRN	chr6	145157070/C//A	nonsynonymous	0	1
SYNE1	chr6	152532625/C//A	synonymous	0	1
CCDC129	chr7	31682778/G//T	nonsynonymous	0	1
AKAP9	chr7	91718731/C//T	synonymous	0	1
SSPO	chr7	149494361/T//G	nonsynonymous	0	1
	chr8	10411536/A//G	synonymous	0	1
PIWIL2	chr8	22140549/C//T	nonsynonymous	0	1
DOCK5	chr8	25132925/C//G	nonsynonymous	0	1
SOX17	chr8	55371890/C//A	nonsynonymous	0	1
RAB2A	chr8	61504424/G//C	nonsynonymous	0	1
MSC	chr8	72754928/C//A	nonsynonymous	0	1
ZFHX4	chr8	77766530/T//G	nonsynonymous	0	1
LRRC14	chr8	145745758/G//A	nonsynonymous	0	1
SMC2	chr9	106860814/C//T	nonsynonymous	0	1
KCNT1	chr9	138670570/G//A	synonymous	0	1
NMT2	chr10	15161351/A//T	nonsynonymous	0	1
PARD3	chr10	34637002/A//G	synonymous	0	1
ERCC6	chr10	50678262/G//A	synonymous	0	1
TCERG1L	chr10	132944928/T//G	synonymous	0	1
TRIM6-TRIM34	chr11	5626619/G//A	nonsynonymous	0	1
DNHD1	chr11	6565367/G//A	synonymous	0	1
DNHD1	chr11	6579727/A//C	nonsynonymous	0	1
DNHD1	chr11	6589893/A//C	nonsynonymous	0	1
NAT10	chr11	34145931/A//G	nonsynonymous	0	1
OR5B2	chr11	58190240/A//G	synonymous	0	1
B4GAT1	chr11	66114981/G//T	nonsynonymous	0	1
NAALAD2	chr11	89916117/G//T	synonymous	0	1
POU2F3	chr11	120173073/C//G	nonsynonymous	0	1
TECTA	chr11	120984420/C//T	synonymous	0	1
RHNO1	chr12	2997238/C//A	synonymous	0	1
FGF23	chr12	4479590/C//A	synonymous	0	1
KCNA1	chr12	5021148/G//T	nonsynonymous	0	1

PTPRO	chr12	15702109/T//A	nonsynonymous	0	1
CNTN1	chr12	41327362/A//C	nonsynonymous	0	1
NTN4	chr12	96180806/C//T	nonsynonymous	0	1
NCOR2	chr12	124810039/C//T	nonsynonymous	0	1
SIAH3	chr13	46357672/C//T	nonsynonymous	0	1
NEK5	chr13	52678655/C//G	nonsynonymous	0	1
TEP1	chr14	20852773/C//G	synonymous	0	1
TEP1	chr14	20876433/G//C	nonsynonymous	0	1
OR4E2	chr14	22133978/C//T	nonsynonymous	0	1
HEATR5A	chr14	31792965/CCTT//C	nonsynonymous	0	1
CDKL1	chr14	50862550/C//G	nonsynonymous	0	1
ATG14	chr14	55852764/G//C	nonsynonymous	0	1
GALNT16	chr14	69799865/C//G	nonsynonymous	0	1
AK7	chr14	96871210/A//G	synonymous	0	1
	chr14	102606531/G//A	synonymous	0	1
GCOM1	chr15	58004258/C//T	nonsynonymous	0	1
DENND4A	chr15	66044774/T//G	nonsynonymous	0	1
TLE3	chr15	70350553/G//C	nonsynonymous	0	1
CEMIP	chr15	81225691/G//A	nonsynonymous	0	1
SRCAP	chr16	30734433/T//G	nonsynonymous	0	1
FBXL19	chr16	30941859/C//T	nonsynonymous	0	1
ATP6V0D1	chr16	67487558/G//C	nonsynonymous	0	1
TMEM132E	chr17	32965126/G//A	nonsynonymous	0	1
KRT13	chr17	39659270/G//A	synonymous	0	1
MMD	chr17	53471754/A//C	nonsynonymous	0	1
CCDC47	chr17	61843417/G//A	nonsynonymous	0	1
GPR142	chr17	72366808/G//C	nonsynonymous	0	1
ZACN	chr17	74076386/A//G	nonsynonymous	0	1
ELANE	chr19	852977/G//A	nonsynonymous	0	1
ZNRF4	chr19	5456372/G//C	nonsynonymous	0	1
TNFSF14	chr19	6665234/G//A	synonymous	0	1
MUC16	chr19	9014592/T//G	nonsynonymous	0	1
OR7A5	chr19	14938830/G//A	nonsynonymous	0	1
PPM1N	chr19	46001838/C//T	synonymous	0	1
NCR1	chr19	55424060/C//A	nonsynonymous	0	1
ZNF274	chr19	58721330/T//G	nonsynonymous	0	1
BPIFB2	chr20	31609146/C//T	synonymous	0	1
SALL4	chr20	50407527/T//A	nonsynonymous	0	1
ITGB2	chr21	46314973/T//A	nonsynonymous	0	1
CLTCL1	chr22	19230413/T//A	nonsynonymous	0	1
CENPM	chr22	42335163/A//G	nonsynonymous	0	1
DCAF8L1	chrX	27999353/C//T	synonymous	0	1
CHRDL1	chrX	109963159/G//T	synonymous	0	1
TBC1D4	chr13	76055813/G//A	nonsynonymous	0	1
MIB1	chr18	19378037/T//C	synonymous	0	1
CATSPERD	chr19	5720779/A//G	nonsynonymous	0	1
LRP3	chr19	33697160/G//A	nonsynonymous	0	1
EMP3	chr19	48833734/C//T	synonymous	0	1
DCST2	chr1	155005168/T//A	synonymous	1	0
GLI2	chr2	121746035/G//A	nonsynonymous	1	0
MSTN	chr2	190922144/T//C	nonsynonymous	1	0
RBM44	chr2	238738143/C//A	nonsynonymous	1	0
MUC4	chr3	195513345/A//C	synonymous	1	0
TRPA1	chr8	72945944/C//A	nonsynonymous	1	0
TMEM38B	chr9	108536368/G//T	synonymous	1	0
USP6NL	chr10	11567400/C//A	nonsynonymous	1	0
HELLS	chr10	96336475/T//C	synonymous	1	0
ERC1	chr12	1136990/A//G	synonymous	1	0
AMN1	chr12	31850691/C//T	nonsynonymous	1	0
HPD	chr12	122295293/A//G	nonsynonymous	1	0
C1QTNF8	chr16	1143931/G//T	nonsynonymous	1	0
PANX2	chr22	50615940/G//T	nonsynonymous	1	0
HIVEP3	chr1	42047370/C//T	synonymous	0	1
PYGO2	chr1	154931962/C//CCA	nonsynonymous	0	1
TPO	chr2	1481359/G//A	nonsynonymous	0	1
ALK	chr2	30143189/C//T	nonsynonymous	0	1
DNAH7	chr2	196729628/G//A	nonsynonymous	0	1
SH3BP4	chr2	235951463/G//A	nonsynonymous	0	1
GPR27	chr3	71803695/C//G	nonsynonymous	0	1
SULT1E1	chr4	70707807/T//C	nonsynonymous	0	1

CDKL3	chr5	133634358/C//A	nonsynonymous	0	1
LCP2	chr5	169693840/A//G	synonymous	0	1
DNAAF5	chr7	766531/G//A	synonymous	0	1
SDK1	chr7	4247766/G//A	synonymous	0	1
C7orf72	chr7	50191089/G//A	nonsynonymous	0	1
UNC5D	chr8	35648045/A//G	synonymous	0	1
FAM135B	chr8	139144962/G//A	synonymous	0	1
DIRAS2	chr9	93376084/C//T	nonsynonymous	0	1
KIAA1217	chr10	24813284/AC//A	nonsynonymous	0	1
PLPP4	chr10	122216878/G//A	synonymous	0	1
MYOD1	chr11	17741687/C//T	nonsynonymous	0	1
HHIPL1	chr14	100125805/C//T	nonsynonymous	0	1
AKT1	chr14	105246551/C//T	nonsynonymous	0	1
FAM227B	chr15	49800421/T//C	synonymous	0	1
RIT2	chr18	40323472/C//A	nonsynonymous	0	1
LOXHD1	chr18	44171911/G//A	nonsynonymous	0	1
PRR22	chr19	5783194/A//T	nonsynonymous	0	1
ZNF844	chr19	12187112/A//G	nonsynonymous	0	1
ZNF432	chr19	52538316/C//T	nonsynonymous	0	1
PRKCG	chr19	54395793/G//A	synonymous	0	1
NLRP13	chr19	56424494/G//A	nonsynonymous	0	1
HIC2	chr22	21800028/C//T	nonsynonymous	0	1
ELFN2	chr22	37769180/C//T	nonsynonymous	0	1
MAPK12	chr22	50693806/G//A	synonymous	0	1
ACOT11	chr1	55050334/G//A	nonsynonymous	0	1
ZNF678	chr1	227843384/G//A	nonsynonymous	0	1
RYR2	chr1	237711896/T//A	synonymous	0	1
PLD5	chr1	242687627/G//A	synonymous	0	1
SCN5A	chr3	38618222/G//A	synonymous	0	1
TREX1	chr3	48509005/G//A	synonymous	0	1
TXNRD3	chr3	126349772/A//G	nonsynonymous	0	1
ABCF3	chr3	183904014/A//G	nonsynonymous	0	1
MCTP1	chr5	94620278/A//C	nonsynonymous	0	1
PCDHA6	chr5	140207736/C//T	synonymous	0	1
SIM1	chr6	100897265/C//A	nonsynonymous	0	1
GOPC	chr6	117898621/A//G	nonsynonymous	0	1
SYTL3	chr6	159139232/G//A	nonsynonymous	0	1
SLC39A12	chr10	18242199/C//T	synonymous	0	1
UNC5B	chr10	73050771/G//A	nonsynonymous	0	1
BEST1	chr11	61727361/C//T	synonymous	0	1
DYRK2	chr12	68043728/G//C	synonymous	0	1
STRC	chr15	43892860/C//T	nonsynonymous	0	1
CACNA1H	chr16	1260814/G//A	nonsynonymous	0	1
SHISA9	chr16	12996110/G//A	synonymous	0	1
ACSM2B	chr16	20557723/T//A	nonsynonymous	0	1
SF3B3	chr16	70566519/T//C	synonymous	0	1
TMEM100	chr17	53798125/T//A	nonsynonymous	0	1
OLIG1	chr21	34442945/C//A	synonymous	0	1
BAIAP2L2	chr22	38494065/C//T	synonymous	0	1
NOX1	chrX	100125762/G//A	synonymous	0	1
RGAG1	chrX	109694569/A//G	nonsynonymous	0	1
MTOR	chr1	11300434/G//A	nonsynonymous	1	0
RSG1	chr1	16558642/G//A	synonymous	1	0
HIVEP3	chr1	42047553/G//T	synonymous	1	0
LRRC41	chr1	46751322/G//A	nonsynonymous	1	0
PLPP3	chr1	57002669/G//A	synonymous	1	0
FLG	chr1	152283029/C//T	nonsynonymous	1	0
HCN3	chr1	155258058/G//A	nonsynonymous	1	0
TGFB2	chr1	218578621/C//T	nonsynonymous	1	0
EPRS	chr1	220206946/TAAAGG//T	nonsynonymous	1	0
PRSS38	chr1	228033871/G//A	nonsynonymous	1	0
KIF26B	chr1	245530662/C//CA	nonsynonymous	1	0
GCKR	chr2	27746408/C//A	synonymous	1	0
PLB1	chr2	28788148/C//A	nonsynonymous	1	0
LBH	chr2	30457350/C//T	nonsynonymous	1	0
CDC42EP3	chr2	37873277/T//C	nonsynonymous	1	0
HK2	chr2	75107448/G//A	nonsynonymous	1	0
SFTPB	chr2	85890558/C//T	nonsynonymous	1	0
SFTPB	chr2	85892764/C//T	nonsynonymous	1	0
FAHD2B	chr2	97749714/C//T	nonsynonymous	1	0

ITGAV	chr2	187532395/T//C	synonymous	1	0
UNC80	chr2	210681643/G//A	nonsynonymous	1	0
ERBB4	chr2	212566803/T//C	nonsynonymous	1	0
ERBB4	chr2	212570063/C//T	nonsynonymous	1	0
EFHD1	chr2	233527654/C//T	nonsynonymous	1	0
CNTN4	chr3	2861218/G//A	nonsynonymous	1	0
TMEM43	chr3	14172437/G//A	nonsynonymous	1	0
CTNNB1	chr3	41275300/A//G	nonsynonymous	1	0
KLHDC8B	chr3	49212285/G//A	nonsynonymous	1	0
BSN	chr3	49688471/G//A	nonsynonymous	1	0
STAB1	chr3	52540709/G//A	nonsynonymous	1	0
CADPS	chr3	62860386/G//A	nonsynonymous	1	0
FAM19A4	chr3	68782292/G//A	synonymous	1	0
OTOL1	chr3	161214726/T//C	nonsynonymous	1	0
PEX5L	chr3	179525600/C//T	nonsynonymous	1	0
CLRN2	chr4	17524630/G//A	nonsynonymous	1	0
POLR2B	chr4	57897157/A//G	synonymous	1	0
UGT2A2	chr4	70455274/C//T	nonsynonymous	1	0
ZDHHC11	chr5	843723/C//A	nonsynonymous	1	0
ZDHHC11	chr5	843815/C//T	synonymous	1	0
ERAP2	chr5	96222388/T//C	synonymous	1	0
SLCO6A1	chr5	101816044/G//A	synonymous	1	0
CEP120	chr5	122748148/T//C	synonymous	1	0
PRRC2A	chr6	31592275/G//A	nonsynonymous	1	0
EGFL8	chr6	32134691/C//G	nonsynonymous	1	0
BYSL	chr6	41895235/G//A	nonsynonymous	1	0
SERINC1	chr6	122792936/C//G	synonymous	1	0
SLC35D3	chr6	137245340/G//A	nonsynonymous	1	0
VWDE	chr7	12417482/G//A	nonsynonymous	1	0
ZNF680	chr7	64004737/C//T	nonsynonymous	1	0
AUTS2	chr7	70227938/G//A	synonymous	1	0
FKBP6	chr7	72743431/C//T	nonsynonymous	1	0
ABCB4	chr7	87031527/A//G	nonsynonymous	1	0
MUC17	chr7	100682625/T//C	nonsynonymous	1	0
FLNC	chr7	128492997/C//T	synonymous	1	0
AGBL3	chr7	134718909/C//T	synonymous	1	0
	chr7	137151673/C//T	synonymous	1	0
KIAA1549	chr7	138546161/C//T	synonymous	1	0
CNTNAP2	chr7	147336321/C//A	nonsynonymous	1	0
ARHGEF10	chr8	1900959/G//A	synonymous	1	0
DKK4	chr8	42231801/T//C	synonymous	1	0
CYP7A1	chr8	59409193/G//A	nonsynonymous	1	0
ZFHX4	chr8	77619880/G//C	nonsynonymous	1	0
ZFHX4	chr8	77763460/C//T	nonsynonymous	1	0
DCAF4L2	chr8	88886244/G//A	synonymous	1	0
JRK	chr8	143740392/C//T	synonymous	1	0
SMARCA2	chr9	2081976/C//G	nonsynonymous	1	0
IFNA5	chr9	21305264/T//C	synonymous	1	0
TLN1	chr9	35717362/C//T	nonsynonymous	1	0
HABP4	chr9	99220765/C//T	nonsynonymous	1	0
OR13C5	chr9	107361216/G//A	nonsynonymous	1	0
OR1J2	chr9	125273995/C//A	nonsynonymous	1	0
BARHL1	chr9	135458451/G//A	synonymous	1	0
CACNA1B	chr9	140907687/C//T	nonsynonymous	1	0
TIAL1	chr10	121336654/C//A	nonsynonymous	1	0
OR51I1	chr11	5462460/C//T	synonymous	1	0
FAM160A2	chr11	6244908/G//C	nonsynonymous	1	0
RBMXL2	chr11	7110641/C//T	nonsynonymous	1	0
SLC15A3	chr11	60718796/C//T	synonymous	1	0
RTN3	chr11	63486826/C//T	synonymous	1	0
PDE2A	chr11	72290042/G//A	nonsynonymous	1	0
SYTL2	chr11	85468769/T//A	synonymous	1	0
SCN3B	chr11	123509026/T//A	nonsynonymous	1	0
OR10G4	chr11	123886957/C//T	nonsynonymous	1	0
NFRKB	chr11	129739590/G//A	synonymous	1	0
CACNA1C	chr12	2595411/A//C	nonsynonymous	1	0
KRT6C	chr12	52864970/G//A	synonymous	1	0
POLR3B	chr12	106821012/T//G	nonsynonymous	1	0
OR4N2	chr14	20296101/G//A	nonsynonymous	1	0
SLC7A8	chr14	23607214/A//G	nonsynonymous	1	0

ZFHX2	chr14	23991694/G//A	nonsynonymous	1	0
DACT1	chr14	59113321/C//T	synonymous	1	0
ERH	chr14	69847243/C//A	synonymous	1	0
ADAM20	chr14	70990766/C//T	nonsynonymous	1	0
TSHR	chr14	81610191/G//A	nonsynonymous	1	0
EML5	chr14	89161739/C//T	nonsynonymous	1	0
CHRFAM7A	chr15	30659641/C//T	nonsynonymous	1	0
C15orf41	chr15	36989592/G//T	nonsynonymous	1	0
THSD4	chr15	71507432/C//T	nonsynonymous	1	0
CHTF18	chr16	845792/C//T	synonymous	1	0
KIAA0556	chr16	27752153/C//A	synonymous	1	0
EXOC3L1	chr16	67221630/G//C	nonsynonymous	1	0
CDT1	chr16	88873746/G//A	nonsynonymous	1	0
CLUH	chr17	2607687/AG//A	nonsynonymous	1	0
ALOX15	chr17	4542813/C//T	synonymous	1	0
RNF112	chr17	19319607/G//A	synonymous	1	0
KRTAP4-6	chr17	39296225/C//T	nonsynonymous	1	0
MPO	chr17	56355377/C//T	nonsynonymous	1	0
TGIF1	chr18	3457742/T//TC	nonsynonymous	1	0
PIEZO2	chr18	10744143/C//T	nonsynonymous	1	0
MC5R	chr18	13826685/G//A	synonymous	1	0
SHC2	chr19	436408/G//A	synonymous	1	0
MUC16	chr19	9072181/G//T	nonsynonymous	1	0
ZNF429	chr19	21719394/A//T	nonsynonymous	1	0
FFAR1	chr19	35842809/A//T	nonsynonymous	1	0
PRR19	chr19	42814947/A//G	synonymous	1	0
RUVBL2	chr19	49507610/A//G	nonsynonymous	1	0
MYBPC2	chr19	50958370/CT//C	nonsynonymous	1	0
SIRPA	chr20	1918249/C//T	synonymous	1	0
DSTN	chr20	17587904/T//C	synonymous	1	0
NCOA6	chr20	33370078/A//C	nonsynonymous	1	0
PABPC1L	chr20	43545422/G//A	nonsynonymous	1	0
TP53TG5	chr20	44003892/G//A	synonymous	1	0
CTCFL	chr20	56083709/G//T	nonsynonymous	1	0
KRTAP8-1	chr21	32185530/G//A	synonymous	1	0
DSCAM	chr21	41725556/C//T	nonsynonymous	1	0
CCDC157	chr22	30768224/G//A	synonymous	1	0
SLC25A17	chr22	41173230/A//G	nonsynonymous	1	0
NIPAL3	chr1	24795521/T//G	nonsynonymous	1	0
ZNF691	chr1	43316884/A//T	synonymous	1	0
RGL1	chr1	183895277/A//T	nonsynonymous	1	0
IGFN1	chr1	201180808/G//A	nonsynonymous	1	0
ZC3H11A	chr1	203818931/G//C	nonsynonymous	1	0
KCNH1	chr1	210856674/T//C	nonsynonymous	1	0
YWHAQ	chr2	9731565/T//A	nonsynonymous	1	0
SLC4A1AP	chr2	27888009/A//G	nonsynonymous	1	0
PSMD1	chr2	232035379/G//A	nonsynonymous	1	0
TMEM45A	chr3	100275773/G//T	synonymous	1	0
ZGRF1	chr4	113539924/T//C	nonsynonymous	1	0
FAT4	chr4	126238403/G//C	nonsynonymous	1	0
MDN1	chr6	90402180/G//C	synonymous	1	0
	chr7	50560685/A//T	synonymous	1	0
TSGA13	chr7	130356591/A//G	nonsynonymous	1	0
NUP205	chr7	135277883/G//A	synonymous	1	0
IDO1	chr8	39781020/G//A	nonsynonymous	1	0
CKS2	chr9	91926142/A//G	synonymous	1	0
SORCS1	chr10	108521360/T//C	synonymous	1	0
B4GALNT4	chr11	376945/G//A	nonsynonymous	1	0
KRTAP5-4	chr11	1642937/T//C	synonymous	1	0
OR52N4	chr11	5776584/C//T	nonsynonymous	1	0
B3GAT3	chr11	62384462/C//G	nonsynonymous	1	0
SORL1	chr11	121476119/G//T	nonsynonymous	1	0
CLEC12A	chr12	10137646/A//G	synonymous	1	0
BORCS5	chr12	12588589/C//T	nonsynonymous	1	0
AVPR1A	chr12	63543907/C//T	nonsynonymous	1	0
ACACB	chr12	109637216/T//G	nonsynonymous	1	0
PLA2G1B	chr12	120763765/G//C	nonsynonymous	1	0
MAB21L1	chr13	36050224/C//T	nonsynonymous	1	0
RNASE2	chr14	21424286/A//C	nonsynonymous	1	0
MYH7	chr14	23900833/G//T	synonymous	1	0

C14orf105	chr14	57938165/C//G	nonsynonymous	1	0
CIPC	chr14	77580486/A//T	nonsynonymous	1	0
HERC2	chr15	28362228/C//A	nonsynonymous	1	0
FAM174B	chr15	93198737/G//C	synonymous	1	0
GSPT1	chr16	11981479/CTCTTGTGGCCAGGG(	nonsynonymous	1	0
EDC4	chr16	67914478/G//A	nonsynonymous	1	0
TAT	chr16	71602127/C//T	nonsynonymous	1	0
ZNF469	chr16	88494622/C//T	synonymous	1	0
PIGS	chr17	26881391/C//T	synonymous	1	0
IMPA2	chr18	12030540/C//T	synonymous	1	0
DCC	chr18	50592522/T//C	nonsynonymous	1	0
IZUMO4	chr19	2097022/CAAG//C	nonsynonymous	1	0
ITPKC	chr19	41235244/C//T	nonsynonymous	1	0
ZNF766	chr19	52793565/T//C	nonsynonymous	1	0
SOGA1	chr20	35444182/C//A	nonsynonymous	1	0
MTIF2	chr2	55489531/T//C	synonymous	0	1
HACE1	chr6	105232049/C//T	nonsynonymous	0	1
EYA4	chr6	133789852/G//A	nonsynonymous	0	1
MUC12	chr7	100646729/C//A	synonymous	0	1
BICD2	chr9	95481847/C//T	synonymous	0	1
FAM222A	chr12	110205821/G//T	nonsynonymous	0	1
ABCC12	chr16	48174690/G//A	nonsynonymous	0	1
ZNF429	chr19	21720109/T//A	synonymous	0	1
ZNF208	chr19	22154475/T//C	nonsynonymous	0	1
NCOA3	chr20	46279845/G//A	synonymous	0	1
KCNQ2	chr20	62076064/C//T	nonsynonymous	0	1
ARFGAP3	chr22	43193568/A//G	synonymous	0	1
KLHL21	chr1	6662647/C//T	synonymous	0	1
OR2M5	chr1	248309108/G//A	nonsynonymous	0	1
CHL1	chr3	382469/T//C	synonymous	0	1
ZIC4	chr3	147109032/G//A	synonymous	0	1
ZNF479	chr7	57188201/G//A	synonymous	0	1
NUSAP1	chr15	41672446/T//A	synonymous	0	1
TPSD1	chr16	1306897/A//C	synonymous	0	1
ABCC1	chr16	16230488/G//A	nonsynonymous	0	1
TMEM88	chr17	7758795/G//A	synonymous	0	1
PNMAL2	chr19	46997064/G//A	synonymous	0	1
CACNA1I	chr22	40042724/T//G	nonsynonymous	0	1
RERE	chr1	8420835/T//C	nonsynonymous	1	0
DISP3	chr1	11589860/C//G	synonymous	1	0
PLEKHM2	chr1	16059199/C//T	synonymous	1	0
EMC1	chr1	19566346/T//C	nonsynonymous	1	0
HSPG2	chr1	22168589/C//T	synonymous	1	0
ADGRB2	chr1	32222875/G//T	synonymous	1	0
MACF1	chr1	39913738/C//CT	nonsynonymous	1	0
ELAVL4	chr1	50659594/A//G	synonymous	1	0
IL23R	chr1	67685284/T//C	nonsynonymous	1	0
HHLA3	chr1	70820691/A//T	synonymous	1	0
LPAR3	chr1	85279765/T//C	nonsynonymous	1	0
SH3GLB1	chr1	87200833/T//C	synonymous	1	0
MYBPHL	chr1	109838868/G//T	synonymous	1	0
CHIA	chr1	111863043/C//T	synonymous	1	0
DPM3	chr1	155112388/T//C	synonymous	1	0
DUSP27	chr1	167096121/A//G	nonsynonymous	1	0
XCL2	chr1	168513203/G//A	synonymous	1	0
SELE	chr1	169701773/A//G	nonsynonymous	1	0
METTL11B	chr1	170115238/C//G	synonymous	1	0
PRRX1	chr1	170695538/T//C	nonsynonymous	1	0
ABL2	chr1	179100616/T//A	nonsynonymous	1	0
ZBTB41	chr1	197128476/T//A	synonymous	1	0
MYBPH	chr1	203143694/G//T	synonymous	1	0
PLEKHA6	chr1	204198072/T//C	nonsynonymous	1	0
SIPA1L2	chr1	232539282/T//G	nonsynonymous	1	0
HEATR1	chr1	236722353/T//A	nonsynonymous	1	0
MYT1L	chr2	1926897/G//A	nonsynonymous	1	0
TRIB2	chr2	12858505/C//T	synonymous	1	0
XDH	chr2	31605932/C//T	nonsynonymous	1	0
USP34	chr2	61430340/A//C	nonsynonymous	1	0
USP34	chr2	61508331/A//T	nonsynonymous	1	0
CD8B	chr2	87080156/T//C	nonsynonymous	1	0

FAHD2A	chr2	96078265/G//T	nonsynonymous	1	0
NEB	chr2	152359394/C//G	nonsynonymous	1	0
SLC4A10	chr2	162661003/A//C	synonymous	1	0
SP3	chr2	174820795/G//A	nonsynonymous	1	0
TTN	chr2	179427848/C//G	nonsynonymous	1	0
	chr2	179611286/C//A	synonymous	1	0
FAM126B	chr2	201881890/G//A	synonymous	1	0
STK36	chr2	219544405/A//C	nonsynonymous	1	0
INHA	chr2	220439806/G//A	nonsynonymous	1	0
CCDC140	chr2	223168633/A//G	synonymous	1	0
AGXT	chr2	241812394/A//T	nonsynonymous	1	0
HDLBP	chr2	242178175/T//C	nonsynonymous	1	0
RTP5	chr2	242813861/C//A	synonymous	1	0
OXTR	chr3	8794826/A//G	nonsynonymous	1	0
MKRN2	chr3	12616509/A//C	synonymous	1	0
OSBPL10	chr3	31703495/T//G	synonymous	1	0
CBLB	chr3	105464755/T//C	synonymous	1	0
ACPP	chr3	132075733/G//A	synonymous	1	0
UBA5	chr3	132394127/T//C	nonsynonymous	1	0
PIK3CB	chr3	138409898/T//C	synonymous	1	0
AADAC	chr3	151542572/A//G	nonsynonymous	1	0
CCNL1	chr3	156870949/T//C	synonymous	1	0
PIK3CA	chr3	178938820/T//G	nonsynonymous	1	0
ST6GAL1	chr3	186793555/A//G	synonymous	1	0
HTT	chr4	3216850/TG//T	nonsynonymous	1	0
LGI2	chr4	25005259/A//G	synonymous	1	0
KCTD8	chr4	44176818/A//T	nonsynonymous	1	0
GABRA4	chr4	46976331/T//A	nonsynonymous	1	0
GABRB1	chr4	47408742/C//A	synonymous	1	0
G3BP2	chr4	76580970/T//C	nonsynonymous	1	0
INTS12	chr4	106616701/T//C	nonsynonymous	1	0
CASP6	chr4	110612159/G//A	nonsynonymous	1	0
NDNF	chr4	121958433/C//A	synonymous	1	0
KIAA1109	chr4	123130472/G//A	nonsynonymous	1	0
FAT4	chr4	126237623/A//G	synonymous	1	0
USP38	chr4	144107113/G//C	nonsynonymous	1	0
DCHS2	chr4	155219570/C//A	nonsynonymous	1	0
TRIML1	chr4	189068532/G//A	synonymous	1	0
PLEKHG4B	chr5	163580/C//T	synonymous	1	0
MYO10	chr5	16704788/G//A	nonsynonymous	1	0
CDH18	chr5	19483592/G//A	nonsynonymous	1	0
ADAMTS12	chr5	33577021/C//A	nonsynonymous	1	0
FYB	chr5	39134971/G//T	nonsynonymous	1	0
PDE4D	chr5	58270553/C//T	nonsynonymous	1	0
ERBIN	chr5	65349449/A//G	nonsynonymous	1	0
ZFYVE16	chr5	79734549/A//G	nonsynonymous	1	0
NUDT12	chr5	102895068/C//A	nonsynonymous	1	0
FBXL21	chr5	135276980/T//G	nonsynonymous	1	0
MATR3	chr5	138661182/A//G	synonymous	1	0
PCDHA10	chr5	140235829/G//A	nonsynonymous	1	0
LARS	chr5	145522462/T//C	nonsynonymous	1	0
SAP30L	chr5	153830666/A//G	nonsynonymous	1	0
HIST1H2AI	chr6	27776373/G//A	nonsynonymous	1	0
PKHD1	chr6	51918830/A//T	synonymous	1	0
MLIP	chr6	54095706/A//G	synonymous	1	0
COL12A1	chr6	75862129/C//T	nonsynonymous	1	0
PHIP	chr6	79724813/ATCGTATTT//A	nonsynonymous	1	0
ASCC3	chr6	101037865/G//A	synonymous	1	0
DCBLD1	chr6	117841116/A//G	synonymous	1	0
OLIG3	chr6	137814321/C//A	synonymous	1	0
PHACTR2	chr6	144086809/G//T	nonsynonymous	1	0
UTRN	chr6	144774965/A//G	synonymous	1	0
SHPRH	chr6	146275947/C//T	nonsynonymous	1	0
LATS1	chr6	150023144/T//C	nonsynonymous	1	0
SYNE1	chr6	152651876/C//T	synonymous	1	0
ARID1B	chr6	157502151/G//A	nonsynonymous	1	0
KIF25	chr6	168440840/G//A	nonsynonymous	1	0
CYTH3	chr7	6210833/C//T	nonsynonymous	1	0
KDELR2	chr7	6502790/C//T	synonymous	1	0
ZNF853	chr7	6662206/G//A	synonymous	1	0

ITGB8	chr7	20444437/A//C	nonsynonymous	1	0
ABCB5	chr7	20785017/G//T	nonsynonymous	1	0
NT5C3A	chr7	33057175/T//C	nonsynonymous	1	0
AOAH	chr7	36657899/A//T	synonymous	1	0
	chr7	56146063/A//G	synonymous	1	0
ZNF479	chr7	57199995/T//G	nonsynonymous	1	0
ZNF107	chr7	64167518/G//A	nonsynonymous	1	0
ELN	chr7	73477515/G//C	nonsynonymous	1	0
	chr7	77325692/C//A	synonymous	1	0
SEMA3A	chr7	83739816/G//T	nonsynonymous	1	0
ASB4	chr7	95167057/G//A	nonsynonymous	1	0
PDK4	chr7	95221871/G//A	nonsynonymous	1	0
MUC12	chr7	100646679/C//A	nonsynonymous	1	0
LRRN3	chr7	110763252/T//G	nonsynonymous	1	0
KCND2	chr7	119915139/C//T	synonymous	1	0
PTPRZ1	chr7	121653170/C//A	nonsynonymous	1	0
PTPRZ1	chr7	121684568/T//C	synonymous	1	0
OR9A2	chr7	142723365/G//T	synonymous	1	0
NOBOX	chr7	144101686/A//G	nonsynonymous	1	0
FASTK	chr7	150777875/A//G	synonymous	1	0
KMT2C	chr7	151893062/T//A	nonsynonymous	1	0
WRN	chr8	31024662/C//A	synonymous	1	0
KCNU1	chr8	36788626/T//G	nonsynonymous	1	0
ZFHX4	chr8	77618721/C//T	nonsynonymous	1	0
C8orf59	chr8	86127149/C//G	nonsynonymous	1	0
WWP1	chr8	87392998/A//G	synonymous	1	0
PDP1	chr8	94934602/T//A	nonsynonymous	1	0
COL14A1	chr8	121353588/C//T	nonsynonymous	1	0
TG	chr8	134034345/T//C	nonsynonymous	1	0
PLEC	chr8	144994909/G//A	nonsynonymous	1	0
FREM1	chr9	14842306/G//T	synonymous	1	0
FAM205A	chr9	34725101/A//G	synonymous	1	0
PHF24	chr9	34971615/C//A	nonsynonymous	1	0
TRIM14	chr9	100854209/G//A	nonsynonymous	1	0
PAPPA	chr9	119115116/A//C	nonsynonymous	1	0
GSN	chr9	124091570/A//AT	nonsynonymous	1	0
PSMB7	chr9	127174706/T//C	nonsynonymous	1	0
SNAPC4	chr9	139272778/C//A	synonymous	1	0
C8G	chr9	139841272/CGAGAGACGACCCC/	synonymous	1	0
SYT15	chr10	46959958/G//A	synonymous	1	0
GPRIN2	chr10	46999580/C//T	synonymous	1	0
SEC24C	chr10	75529726/G//T	nonsynonymous	1	0
IFIT3	chr10	91098590/A//G	nonsynonymous	1	0
HPS6	chr10	103827404/C//T	nonsynonymous	1	0
PPP2R2D	chr10	133761295/G//T	nonsynonymous	1	0
TH	chr11	2188190/A//T	synonymous	1	0
OR52B6	chr11	5602809/G//A	nonsynonymous	1	0
TRIM66	chr11	8662298/T//C	nonsynonymous	1	0
LGR4	chr11	27389585/G//A	synonymous	1	0
KCNA4	chr11	30034301/C//G	synonymous	1	0
FSHB	chr11	30255173/G//A	synonymous	1	0
CAPRIN1	chr11	34104392/T//G	nonsynonymous	1	0
CAPRIN1	chr11	34104398/GAGCAA//G	nonsynonymous	1	0
OR8J3	chr11	55904369/C//T	nonsynonymous	1	0
OR10W1	chr11	58034431/G//C	nonsynonymous	1	0
OSBP	chr11	59377855/T//C	synonymous	1	0
PC	chr11	66633744/T//C	nonsynonymous	1	0
MAML2	chr11	95718774/T//C	synonymous	1	0
CASP1	chr11	104900424/G//T	nonsynonymous	1	0
ZBTB16	chr11	114057727/G//T	nonsynonymous	1	0
ABCG4	chr11	119029322/T//G	nonsynonymous	1	0
ETS1	chr11	128332254/C//T	synonymous	1	0
NFRKB	chr11	129739762/G//C	nonsynonymous	1	0
NELL2	chr12	45173751/G//T	synonymous	1	0
ARID2	chr12	46215239/G//A	nonsynonymous	1	0
METTL7B	chr12	56075563/C//G	nonsynonymous	1	0
USP15	chr12	62696638/A//T	nonsynonymous	1	0
E2F7	chr12	77423657/T//C	nonsynonymous	1	0
NAV3	chr12	78531101/G//A	nonsynonymous	1	0
ACSS3	chr12	81647185/G//A	nonsynonymous	1	0

MTUS2	chr13	29608132/G//A	synonymous	1	0
ALOX5AP	chr13	31318290/C//T	nonsynonymous	1	0
STARD13	chr13	33685917/C//T	nonsynonymous	1	0
COG6	chr13	40254151/G//T	synonymous	1	0
TRIM13	chr13	50586410/CTGATTTG//C	nonsynonymous	1	0
ING1	chr13	111372314/A//G	synonymous	1	0
ATP11AUN	chr13	113333779/G//A	nonsynonymous	1	0
OR4K17	chr14	20586490/A//G	nonsynonymous	1	0
PRMT5	chr14	23392330/A//G	nonsynonymous	1	0
ADCY4	chr14	24795286/T//C	nonsynonymous	1	0
	chr14	24836150/T//A	synonymous	1	0
RPL10L	chr14	47120838/A//G	synonymous	1	0
EXOC5	chr14	57700583/C//T	nonsynonymous	1	0
GPHN	chr14	67576911/A//G	nonsynonymous	1	0
AREL1	chr14	75143399/T//A	nonsynonymous	1	0
YLPM1	chr14	75296002/C//A	nonsynonymous	1	0
FLVCR2	chr14	76088484/C//T	synonymous	1	0
TECPR2	chr14	102916209/G//A	synonymous	1	0
NUTM1	chr15	34649615/C//A	synonymous	1	0
MGA	chr15	42058322/C//G	nonsynonymous	1	0
TTBK2	chr15	43075699/A//T	nonsynonymous	1	0
NR2E3	chr15	72110057/C//A	synonymous	1	0
LINGO1	chr15	77907742/G//A	synonymous	1	0
AXIN1	chr16	396701/C//A	nonsynonymous	1	0
CHTF18	chr16	839359/G//A	nonsynonymous	1	0
TMC5	chr16	19474624/A//T	nonsynonymous	1	0
TMC5	chr16	19481007/G//A	nonsynonymous	1	0
IRX6	chr16	55363194/T//C	nonsynonymous	1	0
CES1	chr16	55846808/T//C	synonymous	1	0
CNOT1	chr16	58572083/CTGCTGCATATTAACC	nonsynonymous	1	0
PRPF8	chr17	1561564/G//T	nonsynonymous	1	0
ANKFY1	chr17	4098475/G//C	synonymous	1	0
MYBBP1A	chr17	4445767/G//A	nonsynonymous	1	0
PIK3R6	chr17	8722414/C//A	nonsynonymous	1	0
MIEF2	chr17	18166460/G//A	nonsynonymous	1	0
ITGA3	chr17	48149412/C//T	nonsynonymous	1	0
ABCC3	chr17	48750941/C//T	nonsynonymous	1	0
MRPL58	chr17	73017032/C//T	nonsynonymous	1	0
DNAH17	chr17	76433845/C//T	nonsynonymous	1	0
FASN	chr17	80041677/T//A	nonsynonymous	1	0
EMILIN2	chr18	2906882/G//T	nonsynonymous	1	0
SLC14A2	chr18	43262357/C//A	nonsynonymous	1	0
C18orf54	chr18	51904564/G//T	nonsynonymous	1	0
CDH19	chr18	64172185/G//C	nonsynonymous	1	0
NETO1	chr18	70534561/T//G	synonymous	1	0
BTBD2	chr19	1986890/A//G	nonsynonymous	1	0
MFSD12	chr19	3546099/A//G	nonsynonymous	1	0
XAB2	chr19	7684701/T//C	nonsynonymous	1	0
KANK3	chr19	8399566/G//A	nonsynonymous	1	0
ZNF562	chr19	9763883/T//G	synonymous	1	0
SMARCA4	chr19	11118636/A//C	nonsynonymous	1	0
ADGRL1	chr19	14261661/C//T	synonymous	1	0
CPAMD8	chr19	17003920/T//A	nonsynonymous	1	0
MPV17L2	chr19	18304161/G//A	nonsynonymous	1	0
ZNF91	chr19	23544243/G//C	nonsynonymous	1	0
ZNF790	chr19	37310406/T//C	synonymous	1	0
ARHGEF1	chr19	42402706/A//C	nonsynonymous	1	0
IGFL3	chr19	46623521/C//T	synonymous	1	0
LHB	chr19	49519484/G//T	synonymous	1	0
KIR2DL3	chr19	55255249/A//C	nonsynonymous	1	0
KIR3DL1	chr19	55340910/C//A	synonymous	1	0
PLCB4	chr20	9353031/G//A	nonsynonymous	1	0
OTOR	chr20	16730650/A//G	nonsynonymous	1	0
	chr20	25059434/C//T	synonymous	1	0
ACTR5	chr20	37377146/C//A	nonsynonymous	1	0
EPPIN	chr20	44170712/C//T	synonymous	1	0
DIDO1	chr20	61525473/G//C	nonsynonymous	1	0
CHRNA4	chr20	61981681/G//A	nonsynonymous	1	0
KRTAP19-5	chr21	31874308/C//A	nonsynonymous	1	0
PDE9A	chr21	44180955/T//C	synonymous	1	0

APOBEC3G	chr22	39474935/A//G	nonsynonymous	1	0
MID1	chrX	10427814/A//G	nonsynonymous	1	0
GPR173	chrX	53105924/G//A	nonsynonymous	1	0
RAP1GAP	chr1	21926095/C//T	synonymous	1	0
TGFBR3	chr1	92177963/A//T	nonsynonymous	1	0
PPM1J	chr1	113253206/CAT//C	nonsynonymous	1	0
HRNR	chr1	152191036/G//C	nonsynonymous	1	0
CD1D	chr1	158150868/C//T	synonymous	1	0
TNN	chr1	175105000/C//T	nonsynonymous	1	0
HHIPL2	chr1	222717148/G//A	synonymous	1	0
KIF26B	chr1	245849839/A//G	nonsynonymous	1	0
DNAJC5G	chr2	27500791/G//A	nonsynonymous	1	0
LYG1	chr2	99907840/G//A	nonsynonymous	1	0
LRP1B	chr2	141819833/G//T	nonsynonymous	1	0
ACVR2A	chr2	148674858/C//T	nonsynonymous	1	0
SCN2A	chr2	166245293/G//A	synonymous	1	0
SP3	chr2	174820146/G//C	nonsynonymous	1	0
GTF3C3	chr2	197637794/G//C	nonsynonymous	1	0
TGFBR2	chr3	30713868/T//C	nonsynonymous	1	0
ALCAM	chr3	105266044/G//A	nonsynonymous	1	0
DDIT4L	chr4	101111145/A//G	synonymous	1	0
	chr4	155411350/G//A	synonymous	1	0
GALNTL6	chr4	173730640/C//T	synonymous	1	0
IRX1	chr5	3600357/G//A	nonsynonymous	1	0
ADGRV1	chr5	90087079/G//A	synonymous	1	0
PCDHA1	chr5	140166590/G//A	nonsynonymous	1	0
PCDHA2	chr5	140175828/G//A	nonsynonymous	1	0
ZNF879	chr5	178460035/C//T	synonymous	1	0
SLC44A4	chr6	31844858/C//T	synonymous	1	0
HLA-DRA	chr6	32411192/G//A	nonsynonymous	1	0
COL19A1	chr6	70637867/C//T	synonymous	1	0
KCNQ5	chr6	73332284/C//T	nonsynonymous	1	0
SYNE1	chr6	152623032/G//T	nonsynonymous	1	0
ELFN1	chr7	1784761/G//A	nonsynonymous	1	0
ABCB1	chr7	87145863/G//C	nonsynonymous	1	0
SND1	chr7	127714722/G//A	nonsynonymous	1	0
PRKDC	chr8	48775012/G//A	synonymous	1	0
EIF3E	chr8	109214012/C//T	synonymous	1	0
GABBR2	chr9	101258693/A//G	nonsynonymous	1	0
EPB41L4B	chr9	111962597/C//T	nonsynonymous	1	0
ZNF483	chr9	114304274/C//T	synonymous	1	0
ADARB2	chr10	1405582/G//A	nonsynonymous	1	0
ANKRD30A	chr10	37490212/C//A	nonsynonymous	1	0
AP3M1	chr10	75888956/C//T	nonsynonymous	1	0
CYP2C9	chr10	96707529/T//C	synonymous	1	0
CHRM4	chr11	46406942/C//T	nonsynonymous	1	0
TENM4	chr11	78383362/T//G	nonsynonymous	1	0
ACVR1B	chr12	52385715/C//T	nonsynonymous	1	0
ANKS1B	chr12	99223050/C//T	nonsynonymous	1	0
VPS33A	chr12	122716852/T//C	nonsynonymous	1	0
CCDC169-SOHLH2	chr13	36827942/G//A	nonsynonymous	1	0
PROZ	chr13	113814357/G//A	nonsynonymous	1	0
AHNAK2	chr14	105413431/G//A	nonsynonymous	1	0
CPPED1	chr16	12798612/C//T	nonsynonymous	1	0
CACNG3	chr16	24268054/G//A	synonymous	1	0
HYDIN	chr16	70928403/G//A	nonsynonymous	1	0
ASGR1	chr17	7076867/C//T	synonymous	1	0
KCNAB3	chr17	7829392/T//C	nonsynonymous	1	0
USP43	chr17	9549164/C//T	nonsynonymous	1	0
MYOCD	chr17	12656357/A//G	synonymous	1	0
ZNF345	chr19	37368165/C//T	nonsynonymous	1	0
ASPDH	chr19	51015728/C//T	nonsynonymous	1	0
ZIM3	chr19	57648246/C//T	nonsynonymous	1	0
NFATC2	chr20	50133423/C//T	nonsynonymous	1	0
TMEM50B	chr21	34839420/C//T	nonsynonymous	1	0
COL18A1	chr21	46893834/G//A	nonsynonymous	1	0
CCT8L2	chr22	17072750/G//T	nonsynonymous	1	0
ZNF74	chr22	20760049/C//T	synonymous	1	0
TCEAL7	chrX	102586377/G//A	nonsynonymous	0	1
NCOA1	chr2	24964684/G//A	nonsynonymous	0	1

UPP2	chr2	158962710/A//G	synonymous	0	1
SCN5A	chr3	38639246/C//T	nonsynonymous	0	1
	chr3	120320116/G//A	synonymous	0	1
EIF2AK1	chr7	6064327/C//T	nonsynonymous	0	1
ADCY1	chr7	45632384/C//T	synonymous	0	1
SEMA3A	chr7	83592562/A//G	nonsynonymous	0	1
DAPK1	chr9	90301509/G//T	synonymous	0	1
ABL1	chr9	133760290/G//A	synonymous	0	1
MYOD1	chr11	17741935/G//A	synonymous	0	1
P2RY6	chr11	73007645/C//A	nonsynonymous	0	1
WDR66	chr12	122372196/G//A	nonsynonymous	0	1
DCLK1	chr13	36413299/G//A	synonymous	0	1
SEMA6D	chr15	48055259/C//A	nonsynonymous	0	1
LINGO1	chr15	77924660/C//A	synonymous	0	1
CDRT1	chr17	15498181/A//G	synonymous	0	1
ABCA10	chr17	67152056/G//A	nonsynonymous	0	1
CCDC40	chr17	78039323/C//T	nonsynonymous	0	1
	chr18	53177850/C//A	synonymous	0	1
PTBP1	chr19	806496/C//A	synonymous	0	1
CHERP	chr19	16640558/T//C	nonsynonymous	0	1
NWD1	chr19	16908672/C//T	nonsynonymous	0	1
VPS16	chr20	2843343/A//G	nonsynonymous	0	1
SLC13A3	chr20	45194969/C//T	nonsynonymous	0	1
GNAS	chr20	57429282/C//T	nonsynonymous	0	1
ITSN1	chr21	35122472/C//T	nonsynonymous	0	1
EFCAB6	chr22	44004440/C//T	nonsynonymous	0	1
C1orf112	chr1	169772447/T//C	synonymous	0	1
ASPM	chr1	197053560/T//G	synonymous	0	1
GAD1	chr2	171716307/G//A	nonsynonymous	0	1
OR5K4	chr3	98073598/A//T	nonsynonymous	0	1
PROM1	chr4	15993865/C//G	synonymous	0	1
GRXCR1	chr4	42964930/T//G	nonsynonymous	0	1
FAT4	chr4	126372924/G//T	nonsynonymous	0	1
TCTE1	chr6	44250201/C//A	nonsynonymous	0	1
ESYT2	chr7	158536268/G//C	synonymous	0	1
VCPIP1	chr8	67578208/G//A	nonsynonymous	0	1
CA3	chr8	86351091/C//T	synonymous	0	1
DMRTA1	chr9	22447686/C//A	nonsynonymous	0	1
GLYATL2	chr11	58604803/A//C	nonsynonymous	0	1
ACTR10	chr14	58666924/T//C	synonymous	0	1
TGM5	chr15	43552656/G//C	nonsynonymous	0	1
ZNF441	chr19	11892301/T//A	synonymous	0	1
ZNF626	chr19	20808090/C//T	nonsynonymous	0	1
MAG	chr19	35804233/T//G	nonsynonymous	0	1
SNX21	chr20	44469833/C//T	nonsynonymous	0	1
DSCAM	chr21	41385160/G//A	nonsynonymous	0	1
NLGN4X	chrX	5821363/G//A	synonymous	0	1
GOSR2	chr17	45016000/G//A	nonsynonymous	1	0
SLC6A9	chr1	44468651/G//A	synonymous	0	1
TTC22	chr1	55253451/C//G	synonymous	0	1
NEGR1	chr1	71873119/G//T	synonymous	0	1
CNN3	chr1	95369022/A//G	nonsynonymous	0	1
CNN3	chr1	95369027/T//G	nonsynonymous	0	1
OVGP1	chr1	111966175/A//C	nonsynonymous	0	1
POLR3GL	chr1	145456556/T//C	synonymous	0	1
PRPF3	chr1	150297379/A//G	synonymous	0	1
MCL1	chr1	150551489/T//G	nonsynonymous	0	1
FAM63A	chr1	150974744/T//G	nonsynonymous	0	1
LCE1C	chr1	152777810/C//T	nonsynonymous	0	1
TTC24	chr1	156553164/T//C	synonymous	0	1
ITLN2	chr1	160914970/C//T	nonsynonymous	0	1
NECTIN4	chr1	161047389/G//A	nonsynonymous	0	1
B3GALT2	chr1	193149682/C//G	nonsynonymous	0	1
KCNT2	chr1	196227479/C//T	nonsynonymous	0	1
TNNI1	chr1	201380612/C//T	nonsynonymous	0	1
GPR37L1	chr1	202097037/C//G	nonsynonymous	0	1
	chr1	207647068/T//A	synonymous	0	1
LAMB3	chr1	209796462/G//A	synonymous	0	1
DNAH14	chr1	225380529/A//G	synonymous	0	1
LGALS8	chr1	236711334/G//C	nonsynonymous	0	1

ZBTB18	chr1	244217391/C//T	synonymous	0	1
CPSF3	chr2	9595791/A//G	nonsynonymous	0	1
FSHR	chr2	49190145/G//T	synonymous	0	1
XPO1	chr2	61719336/A//G	synonymous	0	1
MDH1	chr2	63831910/AGATGTCAACCATGC(	nonsynonymous	0	1
UGP2	chr2	64109643/A//G	nonsynonymous	0	1
SPRED2	chr2	65540606/G//T	synonymous	0	1
NAT8	chr2	73868065/A//G	synonymous	0	1
M1AP	chr2	74802564/C//A	nonsynonymous	0	1
IL36RN	chr2	113820246/T//A	nonsynonymous	0	1
GLI2	chr2	121740424/G//A	nonsynonymous	0	1
CNTNAP5	chr2	125262123/A//C	nonsynonymous	0	1
LRP1B	chr2	141747096/T//C	synonymous	0	1
EVX2	chr2	176948149/T//C	nonsynonymous	0	1
SESTD1	chr2	180036872/T//C	nonsynonymous	0	1
SLC39A10	chr2	196578267/G//T	nonsynonymous	0	1
ZDBF2	chr2	207174720/T//C	nonsynonymous	0	1
NYAP2	chr2	226446713/A//T	nonsynonymous	0	1
PER2	chr2	239185818/G//C	nonsynonymous	0	1
CNTN4	chr3	2942394/A//G	nonsynonymous	0	1
ITPR1	chr3	4715942/T//C	nonsynonymous	0	1
COL7A1	chr3	48619778/C//T	nonsynonymous	0	1
ALAS1	chr3	52238813/A//G	nonsynonymous	0	1
OR5H14	chr3	97868282/T//C	nonsynonymous	0	1
MORC1	chr3	108682402/C//A	nonsynonymous	0	1
TMEM39A	chr3	119166009/G//A	nonsynonymous	0	1
CDV3	chr3	133306883/A//C	nonsynonymous	0	1
PCCB	chr3	135969227/G//A	nonsynonymous	0	1
PARM1	chr4	75938176/C//T	synonymous	0	1
PAQR3	chr4	79860197/T//C	nonsynonymous	0	1
PPM1K	chr4	89189435/G//A	synonymous	0	1
GIMD1	chr4	107288544/T//A	nonsynonymous	0	1
KIAA1109	chr4	123132101/A//G	nonsynonymous	0	1
NEIL3	chr4	178274584/A//G	nonsynonymous	0	1
MYO10	chr5	16783559/T//C	nonsynonymous	0	1
POU5F2	chr5	93076379/C//G	synonymous	0	1
CEP120	chr5	122726952/T//G	nonsynonymous	0	1
P4HA2	chr5	131544986/A//G	nonsynonymous	0	1
EGR1	chr5	137802935/TGGGCAC//T	nonsynonymous	0	1
PCDHA5	chr5	140201924/G//T	synonymous	0	1
PCDHGA9	chr5	140783648/G//A	nonsynonymous	0	1
NEURL1B	chr5	172113924/C//A	nonsynonymous	0	1
UIMC1	chr5	176335648/T//C	nonsynonymous	0	1
KIF6	chr6	39554139/A//G	synonymous	0	1
NT5DC1	chr6	116559361/T//C	synonymous	0	1
DSE	chr6	116756855/A//G	synonymous	0	1
MED23	chr6	131915454/T//C	nonsynonymous	0	1
PLEKHG1	chr6	151140857/C//T	nonsynonymous	0	1
SYNE1	chr6	152589249/T//A	nonsynonymous	0	1
SYNE1	chr6	152605274/C//T	nonsynonymous	0	1
GTF2I	chr7	74103545/C//A	nonsynonymous	0	1
AKAP9	chr7	91631315/T//C	nonsynonymous	0	1
AKAP9	chr7	91631686/G//T	nonsynonymous	0	1
PNPLA8	chr7	108155949/T//C	synonymous	0	1
KCND2	chr7	120382567/T//G	nonsynonymous	0	1
PPP1R3B	chr8	8998625/G//A	synonymous	0	1
BMP1	chr8	22067193/C//T	synonymous	0	1
SCARA3	chr8	27516840/T//G	nonsynonymous	0	1
WHSC1L1	chr8	38189101/C//T	nonsynonymous	0	1
TACC1	chr8	38704209/A//G	synonymous	0	1
RUNX1T1	chr8	92972502/T//A	nonsynonymous	0	1
CSMD3	chr8	113395876/A//G	nonsynonymous	0	1
FBXO10	chr9	37541515/G//A	nonsynonymous	0	1
C9orf84	chr9	114518636/A//G	synonymous	0	1
CNTRL	chr9	123852559/A//C	nonsynonymous	0	1
MVB12B	chr9	129157855/G//C	nonsynonymous	0	1
STXBP1	chr9	130440726/G//A	nonsynonymous	0	1
NUP214	chr9	134003760/A//T	nonsynonymous	0	1
ADARB2	chr10	1405598/G//A	synonymous	0	1
PTCHD3	chr10	27702722/C//T	nonsynonymous	0	1

HNRNPF	chr10	43883256/T//A	nonsynonymous	0	1
WDFY4	chr10	50178223/C//A	nonsynonymous	0	1
IPMK	chr10	59956282/T//C	nonsynonymous	0	1
MARCH5	chr10	94100524/G//C	nonsynonymous	0	1
TWNK	chr10	102753231/T//C	synonymous	0	1
PNLIPRP2	chr10	118389533/T//C	synonymous	0	1
STIM1	chr11	4080549/T//G	nonsynonymous	0	1
CHRM4	chr11	46407567/G//T	nonsynonymous	0	1
OR4B1	chr11	48238811/C//A	synonymous	0	1
ZNHIT2	chr11	64884416/A//T	nonsynonymous	0	1
PCNX3	chr11	65386027/G//A	synonymous	0	1
SNX32	chr11	65617471/C//T	synonymous	0	1
P2RY6	chr11	73007987/C//T	nonsynonymous	0	1
NDUFC2	chr11	77780913/T//C	synonymous	0	1
NPAT	chr11	108031685/A//C	nonsynonymous	0	1
ANKK1	chr11	113270877/T//C	nonsynonymous	0	1
ZW10	chr11	113618296/G//A	nonsynonymous	0	1
KMT2A	chr11	118375389/C//G	nonsynonymous	0	1
USP2	chr11	119227465/G//T	synonymous	0	1
TAS2R14	chr12	11091650/T//A	nonsynonymous	0	1
GRIN2B	chr12	14018891/G//A	synonymous	0	1
ABCC9	chr12	22065941/A//T	synonymous	0	1
ITPR2	chr12	26810988/T//C	synonymous	0	1
CAPRIN2	chr12	30886650/C//T	synonymous	0	1
PDZRN4	chr12	41946547/T//C	synonymous	0	1
KMT2D	chr12	49431603/GTGTGCCCACTGCTA(	nonsynonymous	0	1
LRP1	chr12	57548002/A//C	nonsynonymous	0	1
SLC6A15	chr12	85285643/C//A	nonsynonymous	0	1
RFX4	chr12	107155328/A//G	synonymous	0	1
PXN	chr12	120650213/G//A	synonymous	0	1
BCL7A	chr12	122473296/G//A	synonymous	0	1
ATP12A	chr13	25265349/T//C	synonymous	0	1
AKAP11	chr13	42875389/T//G	nonsynonymous	0	1
THSD1	chr13	52951857/C//A	nonsynonymous	0	1
PCDH8	chr13	53418649/G//C	synonymous	0	1
HEATR4	chr14	73976045/C//T	nonsynonymous	0	1
SLTM	chr15	59205695/G//T	synonymous	0	1
GLCE	chr15	69560957/G//T	nonsynonymous	0	1
AXIN1	chr16	396763/G//T	nonsynonymous	0	1
CDR2	chr16	22360605/GAGGTCAT//G	nonsynonymous	0	1
NLRC5	chr16	57089415/G//C	nonsynonymous	0	1
CDH1	chr16	68845623/A//G	nonsynonymous	0	1
NCBP3	chr17	3729422/C//T	nonsynonymous	0	1
ENO3	chr17	4860301/A//G	nonsynonymous	0	1
PER1	chr17	8046759/G//C	nonsynonymous	0	1
SYNRG	chr17	35969356/T//C	synonymous	0	1
GHDC	chr17	40344303/C//T	nonsynonymous	0	1
HSF5	chr17	56536272/C//A	nonsynonymous	0	1
MRC2	chr17	60755949/A//T	nonsynonymous	0	1
SMARCD2	chr17	61911059/T//C	nonsynonymous	0	1
FASN	chr17	80042164/AGGCCCTTGGCAG//A	nonsynonymous	0	1
ANKRD12	chr18	9254363/T//A	nonsynonymous	0	1
	chr18	47793937/A//C	synonymous	0	1
SOCS6	chr18	67992714/C//G	nonsynonymous	0	1
MUC16	chr19	8976852/A//G	nonsynonymous	0	1
S1PR2	chr19	10334909/C//T	nonsynonymous	0	1
CD22	chr19	35832264/G//T	nonsynonymous	0	1
ZNF420	chr19	37618988/T//A	nonsynonymous	0	1
FCGBP	chr19	40430515/G//A	synonymous	0	1
PSG1	chr19	43383826/T//C	synonymous	0	1
	chr19	50985156/C//T	synonymous	0	1
SIGLEC7	chr19	51645997/G//A	nonsynonymous	0	1
SIGLEC10	chr19	51919408/C//T	synonymous	0	1
RDH13	chr19	55574430/G//GGCGTCCA	synonymous	0	1
GGT7	chr20	33440236/C//G	nonsynonymous	0	1
EIF6	chr20	33867456/T//C	nonsynonymous	0	1
KRTAP15-1	chr21	31812786/C//A	synonymous	0	1
URB1	chr21	33692938/C//T	nonsynonymous	0	1
COL18A1	chr21	46907378/C//T	synonymous	0	1
COL6A2	chr21	47531377/C//T	synonymous	0	1

IL17RA	chr22	17590288/C//T	nonsynonymous	0	1
AIFM3	chr22	21333960/G//C	nonsynonymous	0	1
INPP5J	chr22	31530083/G//A	nonsynonymous	0	1
SLC5A4	chr22	32621797/G//A	synonymous	0	1
CSF2RB	chr22	37334020/G//A	nonsynonymous	0	1
PLXNB2	chr22	50719802/C//CA	nonsynonymous	0	1
XAGE3	chrX	52891672/A//G	synonymous	0	1
RNF103	chr2	86850964/G//A	synonymous	0	1
SOX14	chr3	137484242/G//A	nonsynonymous	0	1
ADGRL3	chr4	62903477/G//A	nonsynonymous	0	1
C1QTNF2	chr5	159776485/G//A	nonsynonymous	0	1
MGAT1	chr5	180219379/C//T	nonsynonymous	0	1
FGD2	chr6	36995888/G//A	synonymous	0	1
HTR1B	chr6	78172108/G//A	nonsynonymous	0	1
TPD52L1	chr6	125550289/G//A	nonsynonymous	0	1
VNN1	chr6	133004380/C//A	nonsynonymous	0	1
SCARA5	chr8	27779322/G//A	nonsynonymous	0	1
TRAPPC9	chr8	141297751/G//A	nonsynonymous	0	1
TLN1	chr9	35704070/G//A	nonsynonymous	0	1
TRPM6	chr9	77427326/C//T	nonsynonymous	0	1
SUSD1	chr9	114886681/C//T	nonsynonymous	0	1
MUC5B	chr11	1270557/C//T	synonymous	0	1
KCNA4	chr11	30032744/A//G	synonymous	0	1
MAPK8IP1	chr11	45921897/G//T	nonsynonymous	0	1
LRP5	chr11	68171070/C//T	synonymous	0	1
NXPE2	chr11	114568959/G//C	nonsynonymous	0	1
RIMKLB	chr12	8902672/G//A	synonymous	0	1
C12orf49	chr12	117160940/T//C	nonsynonymous	0	1
DHRS4L2	chr14	24459534/GGAAGGC//G	nonsynonymous	0	1
RTL1	chr14	101348991/G//A	nonsynonymous	0	1
NDN	chr15	23931940/C//T	nonsynonymous	0	1
PDIA3	chr15	44038837/A//G	nonsynonymous	0	1
PRTG	chr15	55912900/C//A	nonsynonymous	0	1
PRTG	chr15	55930816/C//T	nonsynonymous	0	1
MFSD6L	chr17	8701134/C//T	synonymous	0	1
NMT1	chr17	43174608/G//A	nonsynonymous	0	1
ACSF2	chr17	48548428/G//A	nonsynonymous	0	1
MVB12A	chr19	17530890/C//T	synonymous	0	1
ZCCHC3	chr20	278893/G//A	synonymous	0	1
SLC7A3	chrX	70147847/G//A	nonsynonymous	0	1
PADI3	chr1	17607298/G//A	synonymous	1	0
MROH7	chr1	55139784/C//T	synonymous	1	0
AMPD1	chr1	115231253/C//T	synonymous	1	0
CTSK	chr1	150776511/G//A	nonsynonymous	1	0
CACNA1E	chr1	181727217/C//T	synonymous	1	0
RYR2	chr1	237994867/T//C	nonsynonymous	1	0
RGS7	chr1	241146429/C//G	nonsynonymous	1	0
GREB1	chr2	11758702/C//T	nonsynonymous	1	0
LAPTM4A	chr2	20240691/C//T	nonsynonymous	1	0
ASTL	chr2	96801125/T//G	nonsynonymous	1	0
SLC9A4	chr2	103120148/A//G	nonsynonymous	1	0
	chr2	219137525/C//T	synonymous	1	0
CCDC13	chr3	42751260/G//T	nonsynonymous	1	0
KLHL6	chr3	183273168/C//T	nonsynonymous	1	0
EVC2	chr4	5564548/G//T	synonymous	1	0
HTRA3	chr4	8293200/C//T	nonsynonymous	1	0
PLEKHG4B	chr5	161985/G//A	nonsynonymous	1	0
CTNND2	chr5	11159743/G//A	nonsynonymous	1	0
MAP3K1	chr5	56177572/T//G	nonsynonymous	1	0
TMEM174	chr5	72469396/C//T	nonsynonymous	1	0
APC	chr5	112175423/C//T	nonsynonymous	1	0
FSTL4	chr5	132534717/C//T	synonymous	1	0
PCDHGA4	chr5	140735077/A//G	nonsynonymous	1	0
TCOF1	chr5	149776261/A//T	nonsynonymous	1	0
VWC2	chr7	49815448/C//T	synonymous	1	0
GTF2IRD1	chr7	74005218/C//T	synonymous	1	0
RBM48	chr7	92163855/G//A	synonymous	1	0
MUC17	chr7	100682566/G//A	nonsynonymous	1	0
ATP6V0A4	chr7	138453545/G//A	nonsynonymous	1	0
ZNF703	chr8	37554809/C//T	synonymous	1	0

FAM110B	chr8	59059391/C//T	nonsynonymous	1	0
	chr8	69552680/G//T	synonymous	1	0
PSKH2	chr8	87060713/G//A	nonsynonymous	1	0
TRPS1	chr8	116599328/T//A	nonsynonymous	1	0
EIF3H	chr8	117738365/G//T	nonsynonymous	1	0
COL22A1	chr8	139601521/C//T	nonsynonymous	1	0
ALDH1B1	chr9	38395949/G//A	synonymous	1	0
C9orf170	chr9	89771571/C//T	synonymous	1	0
PTCHD3	chr10	27692282/C//A	nonsynonymous	1	0
CTNNA3	chr10	69299304/G//A	nonsynonymous	1	0
CNNM1	chr10	101089521/G//A	nonsynonymous	1	0
PIK3C2A	chr11	17143769/G//A	nonsynonymous	1	0
OR9G1	chr11	56468125/G//A	nonsynonymous	1	0
CDC42BPG	chr11	64602556/T//C	nonsynonymous	1	0
MAJIN	chr11	64722229/C//T	synonymous	1	0
CADM1	chr11	115099990/G//T	synonymous	1	0
GUCY2C	chr12	14834387/G//A	synonymous	1	0
GRIP1	chr12	66839124/C//T	nonsynonymous	1	0
CMKLR1	chr12	108685994/C//T	nonsynonymous	1	0
HPD	chr12	122294503/T//C	nonsynonymous	1	0
KTN1	chr14	56083231/A//C	synonymous	1	0
DYNC1H1	chr14	102504901/G//A	synonymous	1	0
CEP170B	chr14	105349814/C//T	synonymous	1	0
CHRNA3	chr15	78888945/C//G	synonymous	1	0
WDR90	chr16	707778/G//A	synonymous	1	0
MYH11	chr16	15854419/T//C	nonsynonymous	1	0
GPRC5B	chr16	19884093/C//T	synonymous	1	0
SPN	chr16	29675259/C//T	synonymous	1	0
TOX3	chr16	52473133/G//A	synonymous	1	0
ATP6V0A1	chr17	40630581/G//A	nonsynonymous	1	0
SUPT4H1	chr17	56428769/A//C	nonsynonymous	1	0
TBC1D16	chr17	77914563/G//A	synonymous	1	0
CETN1	chr18	580790/C//T	nonsynonymous	1	0
MUC16	chr19	8974072/C//T	nonsynonymous	1	0
ZSWIM4	chr19	13919899/C//T	nonsynonymous	1	0
TSHZ3	chr19	31769340/C//T	synonymous	1	0
NPHS1	chr19	36339605/C//T	synonymous	1	0
RYR1	chr19	38980792/G//A	nonsynonymous	1	0
PIH1D1	chr19	49952828/C//T	nonsynonymous	1	0
RDH13	chr19	55560098/G//A	nonsynonymous	1	0
ZNF667	chr19	56953456/T//C	nonsynonymous	1	0
SNAP25	chr20	10277627/G//T	synonymous	1	0
DZANK1	chr20	18446028/A//C	synonymous	1	0
TNFRSF14	chr1	2493255/G//A	nonsynonymous	0	1
XIRP2	chr2	168099530/G//A	nonsynonymous	0	1
	chr2	223065773/C//T	synonymous	0	1
ALDH1L1	chr3	125850246/C//A	nonsynonymous	0	1
TFDP2	chr3	141671423/C//A	nonsynonymous	0	1
ZFP57	chr6	29644688/T//C	synonymous	0	1
RORA	chr15	60803748/T//C	nonsynonymous	0	1
TNS4	chr17	38634836/G//A	nonsynonymous	0	1
EPX	chr17	56274319/G//A	nonsynonymous	0	1
DOK6	chr18	67508553/G//C	nonsynonymous	0	1
PNMAL2	chr19	46997605/C//T	nonsynonymous	0	1
EHD2	chr19	48239731/A//G	nonsynonymous	0	1
FLG	chr1	152277218/G//A	nonsynonymous	0	1
SLAMF6	chr1	160456950/T//C	nonsynonymous	0	1
ANTXR1	chr2	69409775/T//G	nonsynonymous	0	1
RALB	chr2	121050771/G//T	nonsynonymous	0	1
NEB	chr2	152512986/G//T	synonymous	0	1
FN1	chr2	216286974/G//T	synonymous	0	1
EIF4E2	chr2	233431791/G//T	nonsynonymous	0	1
CPZ	chr4	8621052/C//A	nonsynonymous	0	1
DHX15	chr4	24557884/G//T	nonsynonymous	0	1
ADH1C	chr4	100266117/C//A	nonsynonymous	0	1
FAT1	chr4	187539681/C//G	nonsynonymous	0	1
GRIA1	chr5	153078591/C//A	nonsynonymous	0	1
SLC17A1	chr6	25811903/G//T	synonymous	0	1
AK9	chr6	109954527/C//A	nonsynonymous	0	1
MAP7	chr6	136732758/C//A	nonsynonymous	0	1

GRM8	chr7	126173195/G//T	synonymous	0	1
KMT2C	chr7	151873360/C//A	nonsynonymous	0	1
PINX1	chr8	10622973/C//A	nonsynonymous	0	1
DLC1	chr8	12943827/G//T	nonsynonymous	0	1
CISD1	chr10	60028986/C//A	synonymous	0	1
ACCSL	chr11	44075057/G//T	nonsynonymous	0	1
KBTBD7	chr13	41767341/A//G	synonymous	0	1
MNAT1	chr14	61346534/G//T	nonsynonymous	0	1
CDH3	chr16	68716280/A//T	nonsynonymous	0	1
NGFR	chr17	47587773/G//A	nonsynonymous	0	1
SERINC3	chr20	43142601/G//T	synonymous	0	1
EPHA2	chr1	16461529/A//G	nonsynonymous	0	1
CDC14A	chr1	100963701/C//A	synonymous	0	1
NOTCH2	chr1	120469211/C//A	nonsynonymous	0	1
NUP210L	chr1	154002435/C//A	nonsynonymous	0	1
CD1E	chr1	158325818/C//T	nonsynonymous	0	1
ACTR1B	chr2	98273309/C//T	synonymous	0	1
SCN3A	chr2	166032822/C//A	nonsynonymous	0	1
XIRP2	chr2	168107782/G//T	nonsynonymous	0	1
ANKAR	chr2	190592631/C//A	nonsynonymous	0	1
ACTR8	chr3	53907070/C//A	nonsynonymous	0	1
MAATS1	chr3	119459479/G//T	synonymous	0	1
COL6A5	chr3	130116479/G//T	synonymous	0	1
EPHA5	chr4	66467879/G//T	synonymous	0	1
TTC29	chr4	147628224/T//C	synonymous	0	1
KIAA0825	chr5	93856361/T//A	nonsynonymous	0	1
SH3TC2	chr5	148384435/G//T	nonsynonymous	0	1
RBM22	chr5	150072465/G//T	nonsynonymous	0	1
SKIV2L	chr6	31935596/C//A	synonymous	0	1
RING1	chr6	33179196/G//T	synonymous	0	1
STXBP5	chr6	147680367/G//T	nonsynonymous	0	1
C8orf74	chr8	10532308/G//T	nonsynonymous	0	1
TRIM66	chr11	8641076/G//T	nonsynonymous	0	1
UBASH3B	chr11	122669676/A//G	nonsynonymous	0	1
C3AR1	chr12	8211721/G//C	nonsynonymous	0	1
ABCC9	chr12	22086768/C//A	nonsynonymous	0	1
XPOT	chr12	64819193/G//T	nonsynonymous	0	1
AACS	chr12	125561121/C//A	nonsynonymous	0	1
CLYBL	chr13	100425254/C//A	nonsynonymous	0	1
GCNT3	chr15	59911193/G//T	nonsynonymous	0	1
NDE1	chr16	15781280/C//A	synonymous	0	1
SULT1A1	chr16	28619674/G//T	nonsynonymous	0	1
ENO3	chr17	4858823/G//T	synonymous	0	1
MRPS23	chr17	55927420/C//T	synonymous	0	1
PPM1E	chr17	56833475/C//A	synonymous	0	1
ALPK2	chr18	56165323/C//G	nonsynonymous	0	1
ZNF426	chr19	9639029/C//A	synonymous	0	1
CACNA1A	chr19	13373652/G//T	synonymous	0	1
ZNF99	chr19	22940394/A//T	nonsynonymous	0	1
EXOSC5	chr19	41895700/G//A	synonymous	0	1
KLK7	chr19	51487256/C//G	synonymous	0	1
TBC1D20	chr20	422563/C//A	synonymous	0	1
ADNP	chr20	49508115/G//T	nonsynonymous	0	1
PPIL2	chr22	22051288/C//A	synonymous	0	1
MIEF1	chr22	39908251/C//T	nonsynonymous	0	1
ACO2	chr22	41916215/C//A	nonsynonymous	0	1
HUWE1	chrX	53561098/G//T	nonsynonymous	0	1
HUWE1	chrX	53595708/C//A	synonymous	0	1
MAGEE1	chrX	75648911/A//G	synonymous	0	1
RAB3GAP1	chr2	135920551/G//A	synonymous	0	1
ASIC5	chr4	156757953/C//T	nonsynonymous	0	1
OVCH2	chr11	7726202/ACT//A	nonsynonymous	0	1
MMP19	chr12	56236612/C//A	nonsynonymous	0	1
CEACAM5	chr19	42212588/A//G	synonymous	0	1
SPO11	chr20	55910962/C//T	nonsynonymous	0	1
DSCAM	chr21	42080523/C//T	nonsynonymous	0	1
PHKA2	chrX	18926918/C//G	nonsynonymous	0	1
KAZN	chr1	15429883/A//G	nonsynonymous	0	1
KLF17	chr1	44595176/C//T	nonsynonymous	0	1
	chr1	154832233/C//T	synonymous	0	1

CD5L	chr1	157805922/C//G	nonsynonymous	0	1
SLAMF7	chr1	160719819/C//T	synonymous	0	1
CACNA1E	chr1	181548207/G//A	nonsynonymous	0	1
TGFBRAP1	chr2	105912951/G//A	synonymous	0	1
LRP2	chr2	170099968/C//T	synonymous	0	1
HOXD11	chr2	176973790/T//G	nonsynonymous	0	1
DAW1	chr2	228786284/A//G	synonymous	0	1
FAM208A	chr3	56716768/G//A	synonymous	0	1
MAGI1	chr3	65425603/T//C	synonymous	0	1
ROBO2	chr3	77526559/C//A	synonymous	0	1
CCDC80	chr3	112358646/C//T	nonsynonymous	0	1
P3H2	chr3	189706770/C//T	nonsynonymous	0	1
EDIL3	chr5	83239326/T//C	nonsynonymous	0	1
PRRC1	chr5	126874762/C//T	nonsynonymous	0	1
FAT2	chr5	150945441/C//T	nonsynonymous	0	1
MSX2	chr5	174151854/G//A	synonymous	0	1
LRFN2	chr6	40400194/C//T	nonsynonymous	0	1
ARMC2	chr6	109282801/G//A	nonsynonymous	0	1
FAM20C	chr7	193557/G//A	nonsynonymous	0	1
ABCA13	chr7	48413990/C//G	nonsynonymous	0	1
BAIAP2L1	chr7	97937061/G//A	nonsynonymous	0	1
MUC17	chr7	100676968/G//A	synonymous	0	1
MUC17	chr7	100676972/T//G	nonsynonymous	0	1
MUC17	chr7	100676975/A//G	nonsynonymous	0	1
HILPDA	chr7	128097383/G//A	nonsynonymous	0	1
TNKS	chr8	9590821/A//G	nonsynonymous	0	1
COL14A1	chr8	121298173/T//A	synonymous	0	1
C10orf10	chr10	45473359/G//A	synonymous	0	1
SLF2	chr10	102676985/A//G	synonymous	0	1
CYP2E1	chr10	135352289/C//T	nonsynonymous	0	1
	chr11	74429886/C//T	synonymous	0	1
FAT3	chr11	92088168/G//T	nonsynonymous	0	1
CRYAB	chr11	111779627/G//A	nonsynonymous	0	1
ATN1	chr12	7045903/G//A	synonymous	0	1
CLEC12A	chr12	10131668/G//T	synonymous	0	1
GUCY2C	chr12	14769648/G//A	synonymous	0	1
CCDC175	chr14	60018075/G//T	nonsynonymous	0	1
PCNX4	chr14	60592418/G//T	synonymous	0	1
OCA2	chr15	28228486/C//T	synonymous	0	1
RASGRF1	chr15	79341880/G//A	synonymous	0	1
PLIN1	chr15	90213364/C//T	nonsynonymous	0	1
HIRIP3	chr16	30005902/A//G	synonymous	0	1
KAT8	chr16	31131803/C//T	nonsynonymous	0	1
P2RX1	chr17	3801167/G//A	nonsynonymous	0	1
MYH4	chr17	10353795/G//A	nonsynonymous	0	1
MPRIP	chr17	17061749/G//A	synonymous	0	1
RAB11B	chr19	8464941/G//A	nonsynonymous	0	1
PDE4A	chr19	10531603/G//A	nonsynonymous	0	1
PSG6	chr19	43411745/A//T	nonsynonymous	0	1
PSG7	chr19	43430635/G//A	nonsynonymous	0	1
PNMAL2	chr19	46998632/C//T	nonsynonymous	0	1
NLRP2	chr19	55494076/G//A	nonsynonymous	0	1
ANKEF1	chr20	10032366/C//T	nonsynonymous	0	1
ADAMTS5	chr21	28302341/C//T	nonsynonymous	0	1
ITSN1	chr21	35186250/G//A	synonymous	0	1
HIRA	chr22	19373085/C//T	nonsynonymous	0	1
PI4KA	chr22	21174187/T//C	synonymous	0	1
ZNF280A	chr22	22868586/G//A	nonsynonymous	0	1
ADSL	chr22	40742532/C//T	synonymous	0	1
PDHA1	chrX	19375842/G//A	synonymous	0	1
MAGEB6	chrX	26212489/G//T	nonsynonymous	0	1
MED14	chrX	40522239/C//G	nonsynonymous	0	1
MAGIX	chrX	49020184/G//A	nonsynonymous	0	1
PRICKLE3	chrX	49035641/G//A	nonsynonymous	0	1
TBC1D8B	chrX	106065296/T//A	nonsynonymous	0	1
CT47B1	chrX	120009199/G//A	nonsynonymous	0	1
GPC3	chrX	133119415/G//A	nonsynonymous	0	1
MAP7D3	chrX	135313889/G//A	synonymous	0	1
GPR50	chrX	150348322/G//A	nonsynonymous	0	1
COL16A1	chr1	32134409/C//T	synonymous	0	1

SLC5A9	chr1	48713124/T//C	nonsynonymous	0	1
AMIGO1	chr1	110051285/G//A	nonsynonymous	0	1
CACNA1E	chr1	181768048/C//T	synonymous	0	1
LRP2	chr2	170135978/C//T	nonsynonymous	0	1
TTN	chr2	179427828/G//A	synonymous	0	1
TTN	chr2	179458769/G//A	nonsynonymous	0	1
PRSS56	chr2	233386821/G//A	nonsynonymous	0	1
ESPNL	chr2	239040113/G//A	nonsynonymous	0	1
CFAP100	chr3	126135307/G//A	nonsynonymous	0	1
PARL	chr3	183547352/G//A	synonymous	0	1
FBXL7	chr5	15936822/C//T	nonsynonymous	0	1
CAMK4	chr5	110818625/G//A	nonsynonymous	0	1
TRIM41	chr5	180661938/A//G	synonymous	0	1
FOXC1	chr6	1612242/C//T	nonsynonymous	0	1
TULP1	chr6	35480432/G//A	nonsynonymous	0	1
DPYSL4	chr10	134006288/G//A	synonymous	0	1
VWCE	chr11	61026449/T//C	nonsynonymous	0	1
ACSS3	chr12	81471893/G//A	synonymous	0	1
CCDC60	chr12	119926627/C//A	nonsynonymous	0	1
TMEM132C	chr12	128900065/G//A	nonsynonymous	0	1
DDX51	chr12	132624721/G//A	nonsynonymous	0	1
METTL21C	chr13	103346751/G//A	nonsynonymous	0	1
CCDC168	chr13	103400485/A//G	synonymous	0	1
ANKRD30B	chr18	14791416/T//C	nonsynonymous	0	1
ZNF521	chr18	22806591/C//A	nonsynonymous	0	1
CDH19	chr18	64211317/G//C	nonsynonymous	0	1
DOCK6	chr19	11313372/G//A	nonsynonymous	0	1
SLC8A2	chr19	47969386/C//T	nonsynonymous	0	1
ZNF587B	chr19	58355773/G//A	synonymous	0	1
WAS	chrX	48544528/G//A	synonymous	0	1
RGAG4	chrX	71350506/G//A	synonymous	0	1
LAGE3	chrX	153707216/G//A	synonymous	0	1
CELSR2	chr1	109812752/C//T	synonymous	1	0
SWT1	chr1	185159711/GA//G	nonsynonymous	1	0
IFT172	chr2	27707969/C//G	nonsynonymous	1	0
PUS10	chr2	61239025/T//C	nonsynonymous	1	0
SCN9A	chr2	167055257/T//A	synonymous	1	0
KALRN	chr3	123983517/A//C	nonsynonymous	1	0
SLC22A23	chr6	3273153/G//A	synonymous	1	0
OPRK1	chr8	54141897/G//T	nonsynonymous	1	0
CHD7	chr8	61768708/C//T	nonsynonymous	1	0
KIAA1217	chr10	24762383/G//A	nonsynonymous	1	0
LUZP2	chr11	25004714/T//G	nonsynonymous	1	0
RARRES3	chr11	63312108/C//A	nonsynonymous	1	0
ARHGAP32	chr11	128843082/G//C	nonsynonymous	1	0
ADAMTS20	chr12	43826131/A//C	nonsynonymous	1	0
SFSWAP	chr12	132195931/G//A	nonsynonymous	1	0
TUBA3C	chr13	19748256/T//C	nonsynonymous	1	0
EDNRB	chr13	78492569/G//A	nonsynonymous	1	0
FES	chr15	91436548/C//T	nonsynonymous	1	0
SBNO2	chr19	1122282/C//T	nonsynonymous	1	0
FPR2	chr19	52272632/C//T	nonsynonymous	1	0
NEFH	chr22	29876449/T//C	synonymous	1	0
USP11	chrX	47103002/G//A	nonsynonymous	1	0
TFE3	chrX	48895939/C//T	nonsynonymous	1	0
RPS6KA6	chrX	83374897/A//C	nonsynonymous	1	0
SAGE1	chrX	134994053/A//G	nonsynonymous	1	0
CCDC24	chr1	44461299/G//A	synonymous	1	0
TNR	chr1	175335178/C//T	nonsynonymous	1	0
ASTN1	chr1	176903419/G//A	nonsynonymous	1	0
TGFB2	chr1	218519986/G//GT	synonymous	1	0
TTN	chr2	179574482/G//C	nonsynonymous	1	0
	chr2	179611568/C//G	synonymous	1	0
FSIP2	chr2	186657726/C//G	nonsynonymous	1	0
ARHGEF3	chr3	56763545/C//T	nonsynonymous	1	0
LRIG1	chr3	66501994/G//A	synonymous	1	0
TNIP2	chr4	2749631/CCT//C	nonsynonymous	1	0
GRXCR1	chr4	42895362/C//T	nonsynonymous	1	0
PCDH10	chr4	134073249/C//T	nonsynonymous	1	0
FREM3	chr4	144617149/C//G	nonsynonymous	1	0

IRX4	chr5	1878714/G//A	nonsynonymous	1	0
FAM172A	chr5	92956636/T//C	synonymous	1	0
PCDHGB2	chr5	140741673/G//C	synonymous	1	0
KIAA0141	chr5	141309253/C//T	synonymous	1	0
BCKDHB	chr6	80910723/T//G	nonsynonymous	1	0
BACH2	chr6	90642386/G//A	nonsynonymous	1	0
FIG4	chr6	110064889/G//A	nonsynonymous	1	0
ANLN	chr7	36456702/C//T	synonymous	1	0
GLI3	chr7	42187878/C//T	nonsynonymous	1	0
HECW1	chr7	43484524/G//A	nonsynonymous	1	0
PTPRZ1	chr7	121652025/G//A	synonymous	1	0
PNMA2	chr8	26365313/G//A	nonsynonymous	1	0
ANK1	chr8	41575639/G//A	synonymous	1	0
TLN1	chr9	35711339/G//A	nonsynonymous	1	0
RECK	chr9	36063805/G//A	synonymous	1	0
PTGES	chr9	132502055/G//A	synonymous	1	0
GRID1	chr10	87966159/C//T	nonsynonymous	1	0
TEX36	chr10	127350448/C//T	synonymous	1	0
	chr11	10482912/A//C	synonymous	1	0
AHNAK	chr11	62294220/C//T	nonsynonymous	1	0
RPS6KA4	chr11	64128707/G//A	synonymous	1	0
PPM1H	chr12	63195861/C//T	nonsynonymous	1	0
PPM1H	chr12	63195862/GTGACGCCACCA//G	nonsynonymous	1	0
TRPC4	chr13	38211357/G//A	nonsynonymous	1	0
SLITRK5	chr13	88328216/T//A	nonsynonymous	1	0
F7	chr13	113772980/G//A	synonymous	1	0
RASGRF1	chr15	79296172/G//A	synonymous	1	0
PEX11A	chr15	90233895/G//A	synonymous	1	0
ZNF668	chr16	31073213/C//T	nonsynonymous	1	0
TP53	chr17	7579377/G//A	nonsynonymous	1	0
TMC6	chr17	76113915/G//A	synonymous	1	0
L3MBTL4	chr18	6263972/C//T	nonsynonymous	1	0
DPP9-AS1	chr19	4684914/G//A	synonymous	1	0
FBN3	chr19	8174242/G//A	nonsynonymous	1	0
CHST8	chr19	34263492/G//A	nonsynonymous	1	0
SYCN	chr19	39694822/C//T	nonsynonymous	1	0
ARHGEF1	chr19	42406450/C//T	nonsynonymous	1	0
PPFIA3	chr19	49637110/G//C	nonsynonymous	1	0
GPR32	chr19	51274358/C//T	synonymous	1	0
GP6	chr19	55543882/G//A	nonsynonymous	1	0
SHISA7	chr19	55952038/C//T	nonsynonymous	1	0
HAO1	chr20	7894946/C//T	nonsynonymous	1	0
ZHX3	chr20	39813844/G//A	synonymous	1	0
WISP2	chr20	43348691/G//A	nonsynonymous	1	0
GGA1	chr22	38004855/G//A	synonymous	1	0
CPXCR1	chrX	88009231/T//C	synonymous	1	0
PDZD4	chrX	153069584/C//T	nonsynonymous	1	0
RNF223	chr1	1007863/G//C	synonymous	0	1
CEP104	chr1	3731955/C//T	synonymous	0	1
USH2A	chr1	215990450/G//A	synonymous	0	1
NMI	chr2	152138493/G//A	nonsynonymous	0	1
LY75-CD302	chr2	160738683/G//A	nonsynonymous	0	1
SLC41A3	chr3	125786813/C//T	nonsynonymous	0	1
IGSF10	chr3	151161457/C//T	nonsynonymous	0	1
BCHE	chr3	165548795/G//A	synonymous	0	1
FYTTD1	chr3	197476796/C//A	synonymous	0	1
SEMA6A	chr5	115803401/G//A	nonsynonymous	0	1
DGKB	chr7	14653001/A//G	nonsynonymous	0	1
C8orf34	chr8	69243520/G//A	synonymous	0	1
RAD54B	chr8	95404120/T//G	nonsynonymous	0	1
TMEM262	chr11	64856464/T//G	nonsynonymous	0	1
CEP290	chr12	88483023/C//T	nonsynonymous	0	1
DRAM1	chr12	102313933/C//T	nonsynonymous	0	1
SHF	chr15	45467553/C//A	nonsynonymous	0	1
RABEP1	chr17	5241350/A//G	nonsynonymous	0	1
KRTAP4-7	chr17	39240819/C//G	nonsynonymous	0	1
DEFB127	chr20	139618/G//T	nonsynonymous	0	1
KRTAP10-6	chr21	46012345/G//A	synonymous	0	1
EFHC2	chrX	44037749/G//T	nonsynonymous	0	1
MTMR8	chrX	63488686/C//A	nonsynonymous	0	1

SAMD11	chr1	878174/A//C	nonsynonymous	0	1
ADAMTSL4	chr1	150525993/C//T	nonsynonymous	0	1
CACNA1S	chr1	201021722/AGTT//A	nonsynonymous	0	1
OR2T1	chr1	248569793/C//A	synonymous	0	1
PGBD2	chr1	249211252/A//G	nonsynonymous	0	1
MYT1L	chr2	1927015/G//A	nonsynonymous	0	1
DCTN1	chr2	74592702/C//A	nonsynonymous	0	1
REG3G	chr2	79254176/G//C	nonsynonymous	0	1
TMEM87B	chr2	112813198/G//C	synonymous	0	1
NEB	chr2	152521103/C//A	nonsynonymous	0	1
ALPI	chr2	233322567/C//T	synonymous	0	1
KCNJ13	chr2	233641209/A//G	synonymous	0	1
SLC6A11	chr3	10885979/C//T	nonsynonymous	0	1
LRTM1	chr3	54952775/G//T	nonsynonymous	0	1
CADM2	chr3	85961601/G//A	nonsynonymous	0	1
KALRN	chr3	124385304/G//A	synonymous	0	1
PIK3CA	chr3	178952085/A//G	nonsynonymous	0	1
ZAR1	chr4	48493209/G//T	nonsynonymous	0	1
NPFFR2	chr4	73013024/A//T	nonsynonymous	0	1
TTC29	chr4	147724712/C//A	nonsynonymous	0	1
SLC9A3	chr5	482797/C//T	nonsynonymous	0	1
UTP15	chr5	72875849/G//A	nonsynonymous	0	1
HIST1H2AC	chr6	26124432/C//T	synonymous	0	1
GLP1R	chr6	39046774/T//G	nonsynonymous	0	1
GRM1	chr6	146755118/G//T	nonsynonymous	0	1
TMEM184A	chr7	1588272/G//C	nonsynonymous	0	1
AP5Z1	chr7	4830418/C//T	nonsynonymous	0	1
HOXA3	chr7	27147987/C//T	synonymous	0	1
PRKAG2	chr7	151478366/G//T	nonsynonymous	0	1
ZFHX4	chr8	77765934/C//A	synonymous	0	1
LRRC14	chr8	145745317/C//G	nonsynonymous	0	1
TTC39B	chr9	15189759/C//T	synonymous	0	1
C9orf43	chr9	116183406/G//A	synonymous	0	1
	chr10	55566684/A//G	synonymous	0	1
TRPM5	chr11	2443546/T//G	synonymous	0	1
LRRC4C	chr11	40136068/T//A	nonsynonymous	0	1
OR5AS1	chr11	55797932/T//C	nonsynonymous	0	1
SLCO1B3	chr12	21033799/G//T	nonsynonymous	0	1
KRT78	chr12	53233099/G//T	nonsynonymous	0	1
ATP5B	chr12	57033869/G//C	nonsynonymous	0	1
KSR2	chr12	117923406/A//T	synonymous	0	1
KATNAL1	chr13	30784483/T//C	nonsynonymous	0	1
PSME2	chr14	24614682/G//A	synonymous	0	1
NAA30	chr14	57857785/G//A	nonsynonymous	0	1
TRPM7	chr15	50853865/A//T	synonymous	0	1
CFAP52	chr17	9511466/G//A	nonsynonymous	0	1
ANKRD30B	chr18	14848935/C//T	synonymous	0	1
ATP13A1	chr19	19762609/T//C	synonymous	0	1
ZNF90	chr19	20229661/T//C	nonsynonymous	0	1
ZNF460	chr19	57802631/G//A	nonsynonymous	0	1
ZNF134	chr19	58131535/G//A	nonsynonymous	0	1
RTFDC1	chr20	55088407/C//G	nonsynonymous	0	1
	chr20	57415215/C//T	synonymous	0	1
TIAM1	chr21	32493018/G//A	nonsynonymous	0	1
CCT8L2	chr22	17073058/G//T	nonsynonymous	0	1
MRPL20	chr1	1342552/C//G	nonsynonymous	1	0
NPHP4	chr1	5987724/G//A	nonsynonymous	1	0
SPEN	chr1	16256039/C//T	nonsynonymous	1	0
SPEN	chr1	16261225/C//T	synonymous	1	0
CROCC	chr1	17297983/G//C	nonsynonymous	1	0
PADI1	chr1	17552618/C//G	nonsynonymous	1	0
HSPG2	chr1	22222460/C//G	nonsynonymous	1	0
RCAN3	chr1	24861734/G//A	synonymous	1	0
RPS6KA1	chr1	26878163/C//T	synonymous	1	0
SPOCD1	chr1	32265436/G//C	nonsynonymous	1	0
EPHA10	chr1	38192766/C//A	synonymous	1	0
SZT2	chr1	43896169/G//A	nonsynonymous	1	0
DPH2	chr1	44437938/C//G	nonsynonymous	1	0
TESK2	chr1	45810473/G//T	synonymous	1	0
GPBP1L1	chr1	46120949/T//C	nonsynonymous	1	0

DMRTA2	chr1	50887206/C//T	nonsynonymous	1	0
USP24	chr1	55573005/C//G	nonsynonymous	1	0
KANK4	chr1	62740567/G//C	nonsynonymous	1	0
GBP5	chr1	89732235/C//T	nonsynonymous	1	0
C1orf146	chr1	92709821/G//A	nonsynonymous	1	0
PTBP2	chr1	97250744/G//T	nonsynonymous	1	0
COL11A1	chr1	103488391/A//T	nonsynonymous	1	0
DCLRE1B	chr1	114454079/C//T	synonymous	1	0
C1orf68	chr1	152692587/C//A	nonsynonymous	1	0
S100A7L2	chr1	153410694/C//A	nonsynonymous	1	0
DENND4B	chr1	153913442/T//G	nonsynonymous	1	0
DCST1	chr1	155014067/G//A	synonymous	1	0
GON4L	chr1	155790453/G//A	synonymous	1	0
GON4L	chr1	155796689/G//T	synonymous	1	0
ETV3	chr1	157094855/G//A	synonymous	1	0
CD1D	chr1	158152889/C//A	nonsynonymous	1	0
PIGM	chr1	160000555/C//T	synonymous	1	0
NECTIN4	chr1	161047318/G//A	nonsynonymous	1	0
KIFAP3	chr1	169947249/C//T	nonsynonymous	1	0
SLC9C2	chr1	173502751/G//A	synonymous	1	0
AXDND1	chr1	179497499/G//C	nonsynonymous	1	0
HMCN1	chr1	185946970/C//T	nonsynonymous	1	0
ZBTB41	chr1	197168703/C//A	nonsynonymous	1	0
TMEM183A	chr1	202985249/G//A	nonsynonymous	1	0
LRRN2	chr1	204587181/G//A	nonsynonymous	1	0
PTPN14	chr1	214558098/T//C	nonsynonymous	1	0
USH2A	chr1	216497566/C//T	nonsynonymous	1	0
HHIPL2	chr1	222717268/G//A	synonymous	1	0
OBSCN	chr1	228523518/G//T	nonsynonymous	1	0
OBSCN	chr1	228528507/AGC//A	nonsynonymous	1	0
TARBP1	chr1	234565430/T//G	synonymous	1	0
KMO	chr1	241729895/C//T	synonymous	1	0
OR2W3	chr1	248059652/T//C	nonsynonymous	1	0
ID2	chr2	8822196/G//A	synonymous	1	0
NCOA1	chr2	24930098/G//C	nonsynonymous	1	0
TCF23	chr2	27372140/G//A	nonsynonymous	1	0
ZNF512	chr2	27823590/G//A	nonsynonymous	1	0
BIRC6	chr2	32774440/C//G	nonsynonymous	1	0
RASGRP3	chr2	33783357/G//C	nonsynonymous	1	0
FAM98A	chr2	33811695/C//A	synonymous	1	0
STRN	chr2	37105099/C//T	nonsynonymous	1	0
MSH2	chr2	47637271/C//G	synonymous	1	0
VPS54	chr2	64139739/G//A	nonsynonymous	1	0
MXD1	chr2	70162564/G//C	nonsynonymous	1	0
LRRTM1	chr2	80530279/G//T	synonymous	1	0
TRABD2A	chr2	85097470/C//A	nonsynonymous	1	0
LONRF2	chr2	100900817/G//A	synonymous	1	0
PTPN4	chr2	120684184/G//A	nonsynonymous	1	0
TFCP2L1	chr2	121989404/C//T	nonsynonymous	1	0
SMPD4	chr2	130930912/C//T	synonymous	1	0
NCKAP5	chr2	133541623/C//T	nonsynonymous	1	0
TMEM163	chr2	135308176/G//T	nonsynonymous	1	0
GPD2	chr2	157352623/G//A	nonsynonymous	1	0
SCN1A	chr2	166897755/T//C	nonsynonymous	1	0
TTN	chr2	179472948/C//A	nonsynonymous	1	0
MAP2	chr2	210560981/G//A	nonsynonymous	1	0
TRPM8	chr2	234869685/G//A	nonsynonymous	1	0
SRGAP3	chr3	9166502/T//C	nonsynonymous	1	0
CMTM6	chr3	32533348/C//T	nonsynonymous	1	0
CCDC13	chr3	42787432/G//A	nonsynonymous	1	0
ALS2CL	chr3	46724796/C//T	synonymous	1	0
SEMA3B	chr3	50311211/C//T	nonsynonymous	1	0
BAP1	chr3	52436285/G//C	synonymous	1	0
BAP1	chr3	52436330/G//A	nonsynonymous	1	0
DNAH12	chr3	57475322/C//G	nonsynonymous	1	0
CD96	chr3	111368541/C//A	synonymous	1	0
KALRN	chr3	124157739/G//T	nonsynonymous	1	0
TRIM42	chr3	140401444/G//A	nonsynonymous	1	0
SPSB4	chr3	140785227/C//T	nonsynonymous	1	0
MECOM	chr3	168845832/GCGATATTGC//G	nonsynonymous	1	0

TNIK	chr3	170856176/G//C	synonymous	1	0
FNDC3B	chr3	172060887/A//G	synonymous	1	0
PEX5L	chr3	179597763/C//G	nonsynonymous	1	0
CLDN16	chr3	190106082/C//T	synonymous	1	0
UTS2B	chr3	190995875/C//G	nonsynonymous	1	0
PAK2	chr3	196534776/G//A	nonsynonymous	1	0
LIMCH1	chr4	41652586/C//T	synonymous	1	0
SGCB	chr4	52894910/C//A	nonsynonymous	1	0
CENPC	chr4	68385122/G//C	nonsynonymous	1	0
SLC4A4	chr4	72399967/G//A	nonsynonymous	1	0
TKTL2	chr4	164393443/C//A	nonsynonymous	1	0
SPOCK3	chr4	167921542/G//C	nonsynonymous	1	0
TENM3	chr4	183717888/C//G	nonsynonymous	1	0
IRF2	chr4	185340693/C//T	nonsynonymous	1	0
SLC9A3	chr5	482785/C//T	nonsynonymous	1	0
SLC6A18	chr5	1240703/G//C	synonymous	1	0
SLC6A18	chr5	1244753/G//T	synonymous	1	0
ICE1	chr5	5462631/G//A	nonsynonymous	1	0
DNAH5	chr5	13721120/T//C	nonsynonymous	1	0
MTMR12	chr5	32263251/C//T	synonymous	1	0
RAI14	chr5	34811141/G//T	nonsynonymous	1	0
SLC1A3	chr5	36608615/G//C	nonsynonymous	1	0
WDR70	chr5	37479947/A//G	nonsynonymous	1	0
TRIM23	chr5	64907537/G//A	nonsynonymous	1	0
HEXB	chr5	73992547/C//G	nonsynonymous	1	0
RFESD	chr5	94991989/C//G	nonsynonymous	1	0
SLCO4C1	chr5	101597682/G//C	nonsynonymous	1	0
SLCO6A1	chr5	101726715/C//A	nonsynonymous	1	0
WDR36	chr5	110428030/G//T	nonsynonymous	1	0
TGFBI	chr5	135391485/G//T	synonymous	1	0
NDUFA2	chr5	140027146/C//A	nonsynonymous	1	0
PCDHA6	chr5	140207821/G//A	nonsynonymous	1	0
PCDHAC1	chr5	140307589/C//G	nonsynonymous	1	0
ARAP3	chr5	141033958/C//A	synonymous	1	0
SH3RF2	chr5	145393384/G//C	synonymous	1	0
PDE6A	chr5	149278933/C//T	synonymous	1	0
FAM71B	chr5	156592810/G//A	nonsynonymous	1	0
STK10	chr5	171554431/G//C	synonymous	1	0
PHYKPL	chr5	177651723/G//A	nonsynonymous	1	0
BTNL8	chr5	180338553/A//G	nonsynonymous	1	0
F13A1	chr6	6305588/G//A	synonymous	1	0
CAP2	chr6	17463309/G//C	synonymous	1	0
HIST1H2BN	chr6	27806547/G//A	synonymous	1	0
VARS2	chr6	30893361/C//T	synonymous	1	0
TBC1D22B	chr6	37259034/C//G	nonsynonymous	1	0
CMTR1	chr6	37442319/G//A	nonsynonymous	1	0
FOXP4	chr6	41558001/C//G	synonymous	1	0
CUL9	chr6	43154098/G//A	nonsynonymous	1	0
TTBK1	chr6	43214412/C//T	nonsynonymous	1	0
DST	chr6	56366327/G//C	nonsynonymous	1	0
PTP4A1	chr6	64286822/A//G	nonsynonymous	1	0
ADGRB3	chr6	70049379/T//A	nonsynonymous	1	0
OSTM1	chr6	108395659/C//G	nonsynonymous	1	0
CLVS2	chr6	123377142/G//A	synonymous	1	0
AKAP12	chr6	151627011/G//C	nonsynonymous	1	0
ARID1B	chr6	157517446/G//A	nonsynonymous	1	0
DAGLB	chr7	6472552/G//A	synonymous	1	0
AHR	chr7	17379014/C//G	nonsynonymous	1	0
ABCB5	chr7	20767983/C//T	synonymous	1	0
FAM188B	chr7	30890096/G//A	nonsynonymous	1	0
NME8	chr7	37916441/G//C	nonsynonymous	1	0
AMPH	chr7	38431513/C//T	nonsynonymous	1	0
POLM	chr7	44113777/G//A	nonsynonymous	1	0
YKT6	chr7	44240771/G//C	nonsynonymous	1	0
DDC	chr7	50531103/A//C	synonymous	1	0
EIF4H	chr7	73604602/A//C	nonsynonymous	1	0
FGL2	chr7	76828832/C//T	synonymous	1	0
PPP1R9A	chr7	94897931/C//T	nonsynonymous	1	0
SYPL1	chr7	105752658/C//G	nonsynonymous	1	0
DGKI	chr7	137237260/T//A	nonsynonymous	1	0

DENND2A	chr7	140302044/C//T	nonsynonymous	1	0
DLGAP2	chr8	1624730/G//A	nonsynonymous	1	0
DLC1	chr8	13356861/G//T	synonymous	1	0
DPYSL2	chr8	26439542/G//A	nonsynonymous	1	0
KIF13B	chr8	28928189/C//T	synonymous	1	0
IDO2	chr8	39836595/C//A	nonsynonymous	1	0
C8orf46	chr8	67428153/G//A	nonsynonymous	1	0
PUF60	chr8	144904075/G//C	nonsynonymous	1	0
SMARCA2	chr9	2104044/A//T	nonsynonymous	1	0
SMARCA2	chr9	2104046/C//G	nonsynonymous	1	0
SMARCA2	chr9	2104048/TA//T	nonsynonymous	1	0
ERMP1	chr9	5832756/G//A	nonsynonymous	1	0
ADAMTSL1	chr9	18826437/G//A	nonsynonymous	1	0
TAF1L	chr9	32633458/C//T	nonsynonymous	1	0
TAF1L	chr9	32634335/G//A	nonsynonymous	1	0
CDK20	chr9	90582395/G//C	synonymous	1	0
INVS	chr9	103015410/G//C	nonsynonymous	1	0
SMC2	chr9	106864732/G//A	nonsynonymous	1	0
NIPSNAP3B	chr9	107528692/G//C	nonsynonymous	1	0
EPB41L4B	chr9	111936884/C//T	nonsynonymous	1	0
TRIM32	chr9	119461152/C//G	synonymous	1	0
GSN	chr9	124074734/G//C	nonsynonymous	1	0
CFAP157	chr9	130476161/G//A	nonsynonymous	1	0
VAV2	chr9	136654418/C//T	synonymous	1	0
ITIH5	chr10	7621750/G//A	synonymous	1	0
FRMD4A	chr10	14372081/G//T	synonymous	1	0
MYO3A	chr10	26310551/C//T	synonymous	1	0
C10orf71	chr10	50530719/C//G	synonymous	1	0
CDH23	chr10	73573101/C//A	nonsynonymous	1	0
OIT3	chr10	74684188/G//T	nonsynonymous	1	0
DLG5	chr10	79581809/G//C	nonsynonymous	1	0
RBP4	chr10	95351861/C//T	nonsynonymous	1	0
SORBS1	chr10	97097042/G//C	nonsynonymous	1	0
PSD	chr10	104171502/C//T	nonsynonymous	1	0
ATRNL1	chr10	116919928/G//C	synonymous	1	0
SYT9	chr11	7324416/C//A	nonsynonymous	1	0
NLRP10	chr11	7982074/G//C	nonsynonymous	1	0
	chr11	17409622/C//G	synonymous	1	0
OR8K5	chr11	55927026/G//C	synonymous	1	0
P2RX3	chr11	57106083/G//C	nonsynonymous	1	0
DAGLA	chr11	61503230/C//T	nonsynonymous	1	0
ZNHIT2	chr11	64884774/C//T	nonsynonymous	1	0
XRRA1	chr11	74562136/C//A	nonsynonymous	1	0
ME3	chr11	86198404/C//G	nonsynonymous	1	0
CEP295	chr11	93432770/G//A	synonymous	1	0
ANKK1	chr11	113258676/G//T	nonsynonymous	1	0
PHLDB1	chr11	118498017/C//G	synonymous	1	0
MCAM	chr11	119183111/C//T	nonsynonymous	1	0
NANOGNB	chr12	7926531/T//A	synonymous	1	0
	chr12	16747187/C//T	synonymous	1	0
GYS2	chr12	21728859/C//A	nonsynonymous	1	0
C12orf40	chr12	40114708/C//T	synonymous	1	0
ADAMTS20	chr12	43846406/C//G	nonsynonymous	1	0
ADAMTS20	chr12	43862441/G//C	synonymous	1	0
KCNH3	chr12	49944005/C//T	nonsynonymous	1	0
	chr12	51695910/G//A	synonymous	1	0
NCKAP1L	chr12	54925595/G//C	nonsynonymous	1	0
OR6C6	chr12	55688576/C//G	nonsynonymous	1	0
PTPRB	chr12	70953371/T//C	nonsynonymous	1	0
TPH2	chr12	72388307/C//G	nonsynonymous	1	0
PTPRQ	chr12	80981994/G//T	nonsynonymous	1	0
TMTC2	chr12	83324329/G//C	synonymous	1	0
VEZT	chr12	95611552/A//T	synonymous	1	0
ANKS1B	chr12	100048949/C//T	nonsynonymous	1	0
MYBPC1	chr12	102061589/G//C	nonsynonymous	1	0
STAB2	chr12	104064519/G//T	nonsynonymous	1	0
SVOP	chr12	109372378/C//T	synonymous	1	0
ACACB	chr12	109617856/G//A	nonsynonymous	1	0
NOS1	chr12	117768355/G//T	nonsynonymous	1	0
NCOR2	chr12	124829341/C//G	nonsynonymous	1	0

TMEM132B	chr12	125811205/T//A	synonymous	1	0
TUBA3C	chr13	19751629/G//T	nonsynonymous	1	0
FLT3	chr13	28631597/C//A	nonsynonymous	1	0
USPL1	chr13	31211949/G//A	nonsynonymous	1	0
TRIM13	chr13	50586434/C//T	nonsynonymous	1	0
WDFY2	chr13	52277747/G//A	nonsynonymous	1	0
TBC1D4	chr13	75936709/G//C	nonsynonymous	1	0
MYCBP2	chr13	77667359/A//G	synonymous	1	0
RPGRIP1	chr14	21756151/G//T	nonsynonymous	1	0
ARHGAP5	chr14	32561803/A//G	nonsynonymous	1	0
INSM2	chr14	36004501/C//G	nonsynonymous	1	0
BMP4	chr14	54418769/C//A	nonsynonymous	1	0
RTN1	chr14	60070604/G//A	synonymous	1	0
SGPP1	chr14	64194075/G//C	synonymous	1	0
HSPA2	chr14	65008818/C//G	nonsynonymous	1	0
ELMSAN1	chr14	74192785/C//A	nonsynonymous	1	0
YLPM1	chr14	75266097/G//T	nonsynonymous	1	0
SEL1L	chr14	81993311/G//A	synonymous	1	0
MTMR10	chr15	31235118/G//A	nonsynonymous	1	0
NUTM1	chr15	34649246/C//T	nonsynonymous	1	0
INO80	chr15	41313318/G//A	synonymous	1	0
TP53BP1	chr15	43738643/C//T	nonsynonymous	1	0
CEP152	chr15	49081119/G//A	nonsynonymous	1	0
SHC4	chr15	49217114/C//G	nonsynonymous	1	0
DMXL2	chr15	51799381/G//C	nonsynonymous	1	0
MNS1	chr15	56757190/C//T	synonymous	1	0
HERC1	chr15	63987061/C//G	nonsynonymous	1	0
PEAK1	chr15	77472801/C//G	nonsynonymous	1	0
VPS33B	chr15	91550210/C//T	nonsynonymous	1	0
PGPEP1L	chr15	99514273/G//A	nonsynonymous	1	0
RNF151	chr16	2018556/C//T	nonsynonymous	1	0
CREBBP	chr16	3900983/T//C	nonsynonymous	1	0
MKL2	chr16	14351988/G//A	nonsynonymous	1	0
DCUN1D3	chr16	20871385/G//A	synonymous	1	0
STX1B	chr16	31008001/C//G	synonymous	1	0
PSMB10	chr16	67970115/C//T	synonymous	1	0
TLDC1	chr16	84520388/G//C	synonymous	1	0
FOXC2	chr16	86601504/C//G	nonsynonymous	1	0
ANKRD11	chr16	89347088/G//A	synonymous	1	0
CHMP1A	chr16	89712414/G//A	synonymous	1	0
TP53	chr17	7577580/T//C	nonsynonymous	1	0
MFSD6L	chr17	8700909/G//C	synonymous	1	0
MYH1	chr17	10399420/C//T	synonymous	1	0
DNAH9	chr17	11713684/C//G	nonsynonymous	1	0
UBB	chr17	16285668/G//C	synonymous	1	0
GAS2L2	chr17	34074917/T//G	synonymous	1	0
ACACA	chr17	35597396/C//T	synonymous	1	0
KRT36	chr17	39643837/G//A	synonymous	1	0
CRHR1-IT1-CRHR1	chr17	43907504/C//T	nonsynonymous	1	0
DDX42	chr17	61864566/C//G	nonsynonymous	1	0
KPNA2	chr17	66038383/G//T	nonsynonymous	1	0
KCNJ16	chr17	68128754/C//T	nonsynonymous	1	0
JMJD6	chr17	74717909/C//A	synonymous	1	0
BAHCC1	chr17	79410341/G//T	nonsynonymous	1	0
THOC1	chr18	214879/A//G	nonsynonymous	1	0
METTL4	chr18	2567147/G//C	nonsynonymous	1	0
EPB41L3	chr18	5416267/C//T	synonymous	1	0
NPC1	chr18	21124325/G//C	nonsynonymous	1	0
DSC2	chr18	28648974/C//A	synonymous	1	0
DSG1	chr18	28919908/C//A	nonsynonymous	1	0
DTNA	chr18	32418063/C//A	nonsynonymous	1	0
CELF4	chr18	35145406/C//T	nonsynonymous	1	0
CDH20	chr18	59195273/T//A	nonsynonymous	1	0
SERPINB12	chr18	61232681/G//A	nonsynonymous	1	0
CDH7	chr18	63430135/G//T	synonymous	1	0
CCDC102B	chr18	66504517/C//A	nonsynonymous	1	0
NETO1	chr18	70461607/G//A	nonsynonymous	1	0
	chr18	72515917/C//G	synonymous	1	0
ZNF556	chr19	2877456/G//C	nonsynonymous	1	0
TJP3	chr19	3747998/C//T	synonymous	1	0

LONP1	chr19	5719727/G//C	nonsynonymous	1	0
ARHGEF18	chr19	7535055/C//T	synonymous	1	0
C19orf45	chr19	7566083/C//G	synonymous	1	0
ZNF564	chr19	12638467/G//A	nonsynonymous	1	0
ZNF490	chr19	12721450/G//C	synonymous	1	0
PKN1	chr19	14574716/C//G	synonymous	1	0
CYP4F12	chr19	15806826/C//G	nonsynonymous	1	0
MYO9B	chr19	17321599/G//A	nonsynonymous	1	0
PIK3R2	chr19	18278074/C//T	nonsynonymous	1	0
LRP3	chr19	33697178/C//T	nonsynonymous	1	0
USF2	chr19	35760872/C//G	nonsynonymous	1	0
FFAR1	chr19	35842963/G//T	nonsynonymous	1	0
RBM42	chr19	36128086/C//G	nonsynonymous	1	0
POLR2I	chr19	36605737/C//G	nonsynonymous	1	0
HKR1	chr19	37853512/C//T	nonsynonymous	1	0
EIF3K	chr19	39125632/G//C	nonsynonymous	1	0
ZNF526	chr19	42729156/G//C	nonsynonymous	1	0
CIC	chr19	42794881/C//G	nonsynonymous	1	0
ZNF155	chr19	44500277/C//T	nonsynonymous	1	0
SYMPK	chr19	46338380/G//A	nonsynonymous	1	0
ZNF497	chr19	58867517/G//A	synonymous	1	0
ZNF497	chr19	58868237/G//A	synonymous	1	0
PCNA	chr20	5100319/C//G	synonymous	1	0
CDK5RAP1	chr20	31954740/G//A	synonymous	1	0
SRC	chr20	36026096/C//G	synonymous	1	0
KIAA1755	chr20	36841902/C//T	nonsynonymous	1	0
KIAA1755	chr20	36856595/C//G	nonsynonymous	1	0
TTPAL	chr20	43117912/C//A	synonymous	1	0
DNTTIP1	chr20	44433984/C//T	synonymous	1	0
ZMYND8	chr20	45976607/G//A	synonymous	1	0
PREX1	chr20	47276499/G//T	nonsynonymous	1	0
SLC17A9	chr20	61598069/G//T	nonsynonymous	1	0
COL20A1	chr20	61944141/C//T	synonymous	1	0
NCAM2	chr21	22652925/G//T	nonsynonymous	1	0
TTC3	chr21	38573738/C//T	synonymous	1	0
ZBTB21	chr21	43411101/G//A	nonsynonymous	1	0
TRPM2	chr21	45855102/T//A	nonsynonymous	1	0
ZNF280A	chr22	22868851/G//T	synonymous	1	0
NEFH	chr22	29886138/G//C	nonsynonymous	1	0
SEC14L3	chr22	30857333/C//G	nonsynonymous	1	0
KCNJ4	chr22	38823441/C//A	nonsynonymous	1	0
CELSR1	chr22	46931990/C//T	nonsynonymous	1	0
GRAMD4	chr22	47070582/C//T	synonymous	1	0
PPP6R2	chr22	50857359/C//G	nonsynonymous	1	0
STS	chrX	7171243/G//A	nonsynonymous	1	0
DMD	chrX	31496480/C//T	nonsynonymous	1	0
BCOR	chrX	39930385/C//G	nonsynonymous	1	0
UBA1	chrX	47058662/A//G	synonymous	1	0
GPR173	chrX	53105836/G//C	synonymous	1	0
DIAPH2	chrX	96369772/C//G	nonsynonymous	1	0
H2BFWT	chrX	103268009/T//C	nonsynonymous	1	0
CXorf66	chrX	139038159/C//T	nonsynonymous	1	0
FAM50A	chrX	153677643/G//C	synonymous	1	0
COL3A1	chr2	189872824/G//A	nonsynonymous	0	1
ANKAR	chr2	190560987/T//C	nonsynonymous	0	1
OTOL1	chr3	161221011/G//A	nonsynonymous	0	1
GYPA	chr4	145038032/G//A	nonsynonymous	0	1
ATP10B	chr5	160039910/C//T	synonymous	0	1
FAM71F1	chr7	128371177/C//T	synonymous	0	1
TRIM49B	chr11	49057845/C//T	synonymous	0	1
KANSL2	chr12	49065687/G//A	nonsynonymous	0	1
SYT1	chr12	79679661/G//A	synonymous	0	1
FNDC3A	chr13	49742767/T//C	synonymous	0	1
C16orf62	chr16	19693720/A//G	synonymous	0	1
CDH8	chr16	61761126/G//A	synonymous	0	1
KRT13	chr17	39659342/C//T	synonymous	0	1
ZNF708	chr19	21477189/T//C	synonymous	0	1
LILRA2	chr19	55085760/C//T	synonymous	0	1
BRSK1	chr19	55814164/C//T	synonymous	0	1
RBBP9	chr20	18477719/C//T	synonymous	0	1

TAB1	chr22	39811633/C//T	nonsynonymous	0	1
SLITRK4	chrX	142718857/G//A	nonsynonymous	0	1
PLCH2	chr1	2428368/T//C	nonsynonymous	0	1
CHD5	chr1	6181185/G//A	nonsynonymous	0	1
ARHGEF10L	chr1	17964397/G//A	nonsynonymous	0	1
SLC30A2	chr1	26365741/C//T	synonymous	0	1
OPRD1	chr1	29189248/G//T	synonymous	0	1
TMEM125	chr1	43739056/G//A	synonymous	0	1
ZFYVE9	chr1	52704941/C//T	nonsynonymous	0	1
GLIS1	chr1	54060134/C//T	nonsynonymous	0	1
L1TD1	chr1	62675709/T//C	synonymous	0	1
DPYD	chr1	97915746/G//A	nonsynonymous	0	1
TSPAN2	chr1	115600176/C//T	synonymous	0	1
CD101	chr1	117559832/T//C	nonsynonymous	0	1
GBA	chr1	155206170/C//G	nonsynonymous	0	1
RGSL1	chr1	182443666/G//A	nonsynonymous	0	1
SNTG2	chr2	1320038/A//G	nonsynonymous	0	1
TPO	chr2	1507752/C//T	nonsynonymous	0	1
DTNB	chr2	25655874/G//A	synonymous	0	1
AGBL5	chr2	27279641/G//A	nonsynonymous	0	1
XDH	chr2	31596802/G//A	synonymous	0	1
LTBP1	chr2	33590543/T//G	nonsynonymous	0	1
SRBD1	chr2	45647000/G//A	synonymous	0	1
EML6	chr2	55074705/G//A	nonsynonymous	0	1
ACTR2	chr2	65473721/G//A	nonsynonymous	0	1
KANSL3	chr2	97267886/G//A	nonsynonymous	0	1
VWA3B	chr2	98928456/C//T	synonymous	0	1
ST6GAL2	chr2	107459888/C//T	synonymous	0	1
POLR1B	chr2	113309425/G//A	nonsynonymous	0	1
MARCO	chr2	119752009/G//A	synonymous	0	1
INHBB	chr2	121107494/C//T	synonymous	0	1
GLI2	chr2	121554883/G//C	synonymous	0	1
SMPD4	chr2	130910154/C//T	nonsynonymous	0	1
AMER3	chr2	131521549/C//T	nonsynonymous	0	1
SCN1A	chr2	166850913/C//T	nonsynonymous	0	1
XIRP2	chr2	168100329/G//T	nonsynonymous	0	1
TTN	chr2	179489342/C//T	nonsynonymous	0	1
TTN	chr2	179578733/T//A	synonymous	0	1
	chr2	179614551/C//T	synonymous	0	1
	chr2	179614778/C//T	synonymous	0	1
UBE2E3	chr2	181927682/A//G	synonymous	0	1
ZNF804A	chr2	185803675/C//T	synonymous	0	1
FSIP2	chr2	186668103/G//A	synonymous	0	1
DNAH7	chr2	196682511/C//T	nonsynonymous	0	1
DNAH7	chr2	196824997/C//T	nonsynonymous	0	1
PLCL1	chr2	198948531/G//A	nonsynonymous	0	1
FAM117B	chr2	203622149/C//T	nonsynonymous	0	1
ZDBF2	chr2	207169608/C//T	nonsynonymous	0	1
PTPRN	chr2	220167031/T//C	synonymous	0	1
COL4A4	chr2	227924234/C//T	nonsynonymous	0	1
COL4A4	chr2	227966607/C//T	nonsynonymous	0	1
SLC16A14	chr2	230923873/G//A	nonsynonymous	0	1
SP110	chr2	231037580/G//A	synonymous	0	1
UGT1A3	chr2	234638302/C//T	nonsynonymous	0	1
ITPR1	chr3	4735247/T//A	nonsynonymous	0	1
GRM7	chr3	7782124/G//A	synonymous	0	1
BRPF1	chr3	9785404/C//T	synonymous	0	1
MLH1	chr3	37090106/T//A	synonymous	0	1
CX3CR1	chr3	39307580/G//T	synonymous	0	1
ZNF660	chr3	44636445/G//A	nonsynonymous	0	1
DUSP7	chr3	52084895/T//C	nonsynonymous	0	1
ERC2	chr3	56044569/C//T	nonsynonymous	0	1
DNAH12	chr3	57327840/C//T	nonsynonymous	0	1
FAM19A1	chr3	68593678/G//A	synonymous	0	1
OR5H6	chr3	97983134/C//T	synonymous	0	1
MYH15	chr3	108218295/C//T	synonymous	0	1
KALRN	chr3	124385899/C//T	nonsynonymous	0	1
KALRN	chr3	124413289/G//A	nonsynonymous	0	1
TF	chr3	133472531/C//T	synonymous	0	1
IGSF10	chr3	151164158/G//A	nonsynonymous	0	1

WDR49	chr3	167196602/C//T	synonymous	0	1
GHSR	chr3	172163253/C//G	nonsynonymous	0	1
HTT	chr4	3136260/G//T	nonsynonymous	0	1
GPR78	chr4	8582779/G//A	nonsynonymous	0	1
RHOH	chr4	40244970/G//A	synonymous	0	1
NIPAL1	chr4	48038100/G//A	nonsynonymous	0	1
UGT2B28	chr4	70146860/G//A	nonsynonymous	0	1
UGT2B4	chr4	70346551/C//T	nonsynonymous	0	1
ENAM	chr4	71507988/A//C	nonsynonymous	0	1
FRAS1	chr4	79440574/C//T	synonymous	0	1
FRAS1	chr4	79462330/G//A	synonymous	0	1
GK2	chr4	80327860/G//A	nonsynonymous	0	1
STPG2	chr4	98893466/C//T	nonsynonymous	0	1
TACR3	chr4	104511147/G//A	nonsynonymous	0	1
FAT4	chr4	126373145/C//T	synonymous	0	1
LRBA	chr4	151207169/G//A	nonsynonymous	0	1
DCHS2	chr4	155225899/C//T	nonsynonymous	0	1
GLRB	chr4	158057999/C//T	nonsynonymous	0	1
NPY1R	chr4	164246500/G//A	synonymous	0	1
WDR17	chr4	177083218/C//T	synonymous	0	1
TENM3	chr4	183673180/C//T	nonsynonymous	0	1
PRIMPOL	chr4	185580515/G//A	nonsynonymous	0	1
ACSL1	chr4	185678832/A//G	nonsynonymous	0	1
SORBS2	chr4	186544118/T//C	nonsynonymous	0	1
TRIML1	chr4	189068145/C//T	synonymous	0	1
TRIP13	chr5	908566/C//G	nonsynonymous	0	1
TERT	chr5	1254627/G//A	synonymous	0	1
RAI14	chr5	34824414/C//T	nonsynonymous	0	1
EGFLAM	chr5	38409148/G//A	nonsynonymous	0	1
C7	chr5	40959612/A//T	nonsynonymous	0	1
PLCXD3	chr5	41382624/G//A	nonsynonymous	0	1
SNX18	chr5	53815498/A//T	synonymous	0	1
DMGDH	chr5	78328564/G//A	nonsynonymous	0	1
ANKRD34B	chr5	79855227/C//T	synonymous	0	1
RASGRF2	chr5	80388761/G//A	nonsynonymous	0	1
ADGRV1	chr5	90008209/C//T	nonsynonymous	0	1
NREP	chr5	111066608/G//A	synonymous	0	1
FAM170A	chr5	118965335/G//A	synonymous	0	1
CDKL3	chr5	133648042/C//T	synonymous	0	1
TRPC7	chr5	135567150/G//A	synonymous	0	1
PCDHA8	chr5	140222859/G//A	synonymous	0	1
PCDHA10	chr5	140235951/G//A	synonymous	0	1
PCDHGA1	chr5	140711414/G//A	nonsynonymous	0	1
SPRY4	chr5	141693909/G//A	synonymous	0	1
SYNPO	chr5	150028681/C//T	nonsynonymous	0	1
GPX3	chr5	150407685/G//A	synonymous	0	1
SLC36A2	chr5	150722526/C//T	nonsynonymous	0	1
NMUR2	chr5	151771983/C//T	synonymous	0	1
TIMD4	chr5	156378573/G//A	nonsynonymous	0	1
ZNF354A	chr5	178152459/G//A	synonymous	0	1
MAML1	chr5	179201473/C//T	synonymous	0	1
SYCP2L	chr6	10924851/C//T	nonsynonymous	0	1
SYCP2L	chr6	10928651/C//T	nonsynonymous	0	1
OR2H1	chr6	29430333/C//T	nonsynonymous	0	1
NEU1	chr6	31829180/G//A	nonsynonymous	0	1
CUL9	chr6	43153904/G//A	nonsynonymous	0	1
COL21A1	chr6	56033060/G//A	synonymous	0	1
DDX43	chr6	74110056/G//A	nonsynonymous	0	1
SCML4	chr6	108067916/C//T	nonsynonymous	0	1
AK9	chr6	109885451/G//A	nonsynonymous	0	1
TMEM200A	chr6	130763029/G//A	nonsynonymous	0	1
	chr6	134213066/C//G	synonymous	0	1
ARID1B	chr6	157405868/G//A	nonsynonymous	0	1
ZDHHC14	chr6	157963612/G//A	synonymous	0	1
SYNJ2	chr6	158497676/G//A	nonsynonymous	0	1
TMEM181	chr6	158957722/A//C	nonsynonymous	0	1
GET4	chr7	927147/C//T	synonymous	0	1
DNAH11	chr7	21818642/G//A	nonsynonymous	0	1
ADCYAP1R1	chr7	31126109/C//T	nonsynonymous	0	1
KBTBD2	chr7	32910246/G//A	nonsynonymous	0	1

ADCY1	chr7	45662327/C//T	synonymous	0	1
ADCY1	chr7	45743249/C//T	synonymous	0	1
SAMD9	chr7	92734014/G//A	nonsynonymous	0	1
CALCR	chr7	93098063/G//A	nonsynonymous	0	1
CYP3A4	chr7	99358551/C//T	nonsynonymous	0	1
SLC26A3	chr7	107414384/G//A	nonsynonymous	0	1
PRSS58	chr7	141952127/C//T	nonsynonymous	0	1
BLK	chr8	11406632/G//A	nonsynonymous	0	1
FGL1	chr8	17743049/G//A	synonymous	0	1
	chr8	32505611/G//A	synonymous	0	1
KAT6A	chr8	41834550/A//C	nonsynonymous	0	1
RP1	chr8	55533742/G//A	synonymous	0	1
RP1	chr8	55533769/G//A	synonymous	0	1
XKR4	chr8	56435915/G//A	nonsynonymous	0	1
PDE7A	chr8	66635783/G//A	synonymous	0	1
KCNB2	chr8	73849846/C//T	synonymous	0	1
ZFHX4	chr8	77766710/C//T	nonsynonymous	0	1
ZFPM2	chr8	106813886/C//T	nonsynonymous	0	1
MTBP	chr8	121514750/C//T	nonsynonymous	0	1
POU5F1B	chr8	128428104/C//T	synonymous	0	1
PHF20L1	chr8	133829667/C//T	nonsynonymous	0	1
PTPRD	chr9	8375953/C//T	synonymous	0	1
TEK	chr9	27203112/C//T	nonsynonymous	0	1
RECK	chr9	36087870/C//T	nonsynonymous	0	1
GRIN3A	chr9	104499726/G//A	nonsynonymous	0	1
ZNF462	chr9	109689872/C//T	nonsynonymous	0	1
TXNDC8	chr9	113100108/C//T	synonymous	0	1
MUSK	chr9	113496561/C//T	nonsynonymous	0	1
COL27A1	chr9	116930567/C//T	synonymous	0	1
PTGS1	chr9	125154632/C//A	nonsynonymous	0	1
DENND1A	chr9	126429324/A//G	nonsynonymous	0	1
OLFML2A	chr9	127572289/G//A	synonymous	0	1
SOHLH1	chr9	138585425/C//T	synonymous	0	1
HKDC1	chr10	70987081/T//G	nonsynonymous	0	1
ZFYVE27	chr10	99510220/C//T	nonsynonymous	0	1
PSD	chr10	104176568/G//A	synonymous	0	1
PLEKHS1	chr10	115528614/C//T	nonsynonymous	0	1
HTRA1	chr10	124273755/C//T	synonymous	0	1
CHST15	chr10	125804253/C//T	synonymous	0	1
MKI67	chr10	129905780/G//A	synonymous	0	1
SLC22A18	chr11	2924650/C//T	synonymous	0	1
ART5	chr11	3660251/C//T	nonsynonymous	0	1
OR52J3	chr11	5067800/C//T	synonymous	0	1
NLRP10	chr11	7981578/C//T	synonymous	0	1
RPL27A	chr11	8704302/CG//C	synonymous	0	1
DGKZ	chr11	46397632/T//A	synonymous	0	1
OR4C46	chr11	51515461/C//T	synonymous	0	1
SLC43A1	chr11	57256440/G//A	nonsynonymous	0	1
OR10Q1	chr11	57996108/C//T	synonymous	0	1
GLYATL2	chr11	58601929/C//T	nonsynonymous	0	1
LGALS12	chr11	63283733/C//T	synonymous	0	1
SART1	chr11	65733659/C//T	synonymous	0	1
GAL3ST3	chr11	65811027/G//A	synonymous	0	1
LRFN4	chr11	66627606/C//T	synonymous	0	1
SHANK2	chr11	70653177/G//A	synonymous	0	1
P2RY2	chr11	72945667/G//T	nonsynonymous	0	1
INTS4	chr11	77692623/C//T	nonsynonymous	0	1
SLC6A12	chr12	305982/G//A	nonsynonymous	0	1
PRMT8	chr12	3659116/T//C	synonymous	0	1
CRACR2A	chr12	3782690/T//C	nonsynonymous	0	1
GRIN2B	chr12	13716857/G//A	synonymous	0	1
GRIN2B	chr12	13769599/A//T	synonymous	0	1
PTPRO	chr12	15734642/G//A	synonymous	0	1
CASC1	chr12	25311453/C//T	nonsynonymous	0	1
PDZRN4	chr12	41587911/G//A	nonsynonymous	0	1
AMIGO2	chr12	47471999/A//G	nonsynonymous	0	1
AMIGO2	chr12	47472073/G//A	nonsynonymous	0	1
KMT2D	chr12	49435127/G//A	synonymous	0	1
FAM186A	chr12	50745280/C//T	nonsynonymous	0	1
TFCP2	chr12	51566103/C//A	nonsynonymous	0	1

BEST3	chr12	70049329/G//A	synonymous	0	1
PHLDA1	chr12	76424287/T//A	synonymous	0	1
PTPRQ	chr12	80839335/G//A	nonsynonymous	0	1
RASSF9	chr12	86198958/C//T	nonsynonymous	0	1
TMPO	chr12	98927753/A//C	nonsynonymous	0	1
STAB2	chr12	104126961/G//A	nonsynonymous	0	1
ISCU	chr12	108961025/C//T	synonymous	0	1
FAM109A	chr12	111800556/G//A	nonsynonymous	0	1
OAS2	chr12	113424940/G//A	nonsynonymous	0	1
KSR2	chr12	117992969/C//T	synonymous	0	1
HNF1A	chr12	121416602/G//A	nonsynonymous	0	1
FLT3	chr13	28636066/G//A	synonymous	0	1
KLHL1	chr13	70413255/C//T	nonsynonymous	0	1
COL4A1	chr13	110826993/G//A	nonsynonymous	0	1
GRK1	chr13	114321926/C//T	synonymous	0	1
JPH4	chr14	24040285/G//A	nonsynonymous	0	1
DLL4	chr15	41228477/G//A	nonsynonymous	0	1
JMJD7	chr15	42128991/C//T	nonsynonymous	0	1
LRRC57	chr15	42840614/G//A	nonsynonymous	0	1
SORD	chr15	45353299/A//T	synonymous	0	1
MAPK6	chr15	52339162/A//G	nonsynonymous	0	1
MAP2K5	chr15	67842423/A//T	synonymous	0	1
NOX5	chr15	69348989/G//A	nonsynonymous	0	1
PPCDC	chr15	75320597/C//T	synonymous	0	1
SCAPER	chr15	77057343/G//A	nonsynonymous	0	1
CHRNB4	chr15	78917469/G//A	synonymous	0	1
DET1	chr15	89070909/G//A	nonsynonymous	0	1
IDH2	chr15	90628316/G//T	nonsynonymous	0	1
SLCO3A1	chr15	92669283/C//T	synonymous	0	1
GRIN2A	chr16	9892190/T//A	nonsynonymous	0	1
MKL2	chr16	14340836/G//A	synonymous	0	1
XYLT1	chr16	17211721/A//T	nonsynonymous	0	1
SYT17	chr16	19195159/C//T	nonsynonymous	0	1
C17orf107	chr17	4805148/C//T	synonymous	0	1
USP6	chr17	5072213/G//A	nonsynonymous	0	1
PFAS	chr17	8157579/C//T	nonsynonymous	0	1
DNAH9	chr17	11671886/C//T	synonymous	0	1
DNAH9	chr17	11790213/G//A	synonymous	0	1
FLCN	chr17	17131337/G//A	nonsynonymous	0	1
MYO15A	chr17	18049396/C//T	nonsynonymous	0	1
MIEF2	chr17	18168135/C//T	synonymous	0	1
FAM83G	chr17	18881680/G//A	synonymous	0	1
NUFIP2	chr17	27621090/G//A	synonymous	0	1
ARHGAP23	chr17	36623042/C//T	nonsynonymous	0	1
KRT17	chr17	39775939/C//T	synonymous	0	1
CNP	chr17	40120746/G//A	nonsynonymous	0	1
FAM134C	chr17	40735557/G//A	nonsynonymous	0	1
KIF18B	chr17	43003834/G//A	synonymous	0	1
SCN4A	chr17	62018761/G//A	synonymous	0	1
AXIN2	chr17	63533737/G//A	nonsynonymous	0	1
	chr17	65882347/C//T	synonymous	0	1
GRIN2C	chr17	72848489/C//T	nonsynonymous	0	1
PIK3C3	chr18	39584455/C//T	nonsynonymous	0	1
RIT2	chr18	40695401/T//A	synonymous	0	1
ELOA2	chr18	44560659/C//T	nonsynonymous	0	1
SERPINB12	chr18	61233843/G//A	nonsynonymous	0	1
SERPINB3	chr18	61326713/C//T	nonsynonymous	0	1
SHD	chr19	4290474/C//T	synonymous	0	1
CHAF1A	chr19	4433433/C//T	nonsynonymous	0	1
ACSBG2	chr19	6183050/G//A	synonymous	0	1
MUC16	chr19	9047872/C//T	synonymous	0	1
MUC16	chr19	9066569/G//A	synonymous	0	1
MUC16	chr19	9076530/C//A	nonsynonymous	0	1
MUC16	chr19	9087450/G//A	synonymous	0	1
PRKACA	chr19	14208406/C//T	synonymous	0	1
SYDE1	chr19	15224595/C//T	nonsynonymous	0	1
NOTCH3	chr19	15271884/G//A	synonymous	0	1
CYP4F3	chr19	15757879/G//A	nonsynonymous	0	1
	chr19	15791160/G//A	synonymous	0	1
EPS15L1	chr19	16497011/G//A	nonsynonymous	0	1

COMP	chr19	18893907/G//A	synonymous	0	1
ZNF90	chr19	20228644/C//T	nonsynonymous	0	1
ZNF99	chr19	22940859/T//A	nonsynonymous	0	1
FAM187B	chr19	35716061/G//A	synonymous	0	1
ATP4A	chr19	36046336/G//A	synonymous	0	1
THAP8	chr19	36526359/G//A	nonsynonymous	0	1
ZNF260	chr19	37005847/G//A	synonymous	0	1
CATSPERG	chr19	38855605/G//A	synonymous	0	1
LRFN1	chr19	39805113/G//A	synonymous	0	1
CYP2A7	chr19	41381556/C//T	synonymous	0	1
CEACAM19	chr19	45179640/C//T	synonymous	0	1
EML2-AS1	chr19	46145533/C//T	synonymous	0	1
TRPM4	chr19	49685858/C//T	synonymous	0	1
ZNF534	chr19	52941227/T//A	nonsynonymous	0	1
VN1R2	chr19	53762045/C//T	synonymous	0	1
LILRA5	chr19	54818745/G//A	nonsynonymous	0	1
LILRA1	chr19	55112178/G//A	nonsynonymous	0	1
ZNF628	chr19	55995769/G//A	synonymous	0	1
CCDC106	chr19	56162764/C//T	synonymous	0	1
ZSCAN5A	chr19	56735050/G//A	nonsynonymous	0	1
ZNF582	chr19	56901453/G//A	nonsynonymous	0	1
ZIM2	chr19	57286515/C//T	synonymous	0	1
PEG3	chr19	57335807/C//T	nonsynonymous	0	1
ZNF584	chr19	58928499/G//A	nonsynonymous	0	1
	chr20	29976852/C//T	synonymous	0	1
SULF2	chr20	46292868/T//G	synonymous	0	1
PREX1	chr20	47307632/C//G	nonsynonymous	0	1
ARFGEF2	chr20	47649641/C//T	synonymous	0	1
DPM1	chr20	49575082/G//A	synonymous	0	1
ZNF831	chr20	57767889/G//A	synonymous	0	1
GRIK1	chr21	31015210/G//A	nonsynonymous	0	1
KRTAP15-1	chr21	31812704/G//A	nonsynonymous	0	1
TSPEAR	chr21	45947200/C//T	nonsynonymous	0	1
VPREB1	chr22	22599717/G//A	nonsynonymous	0	1
RAB36	chr22	23498182/C//T	synonymous	0	1
CRYBB3	chr22	25597363/G//A	synonymous	0	1
EMID1	chr22	29639464/G//A	nonsynonymous	0	1
GAL3ST1	chr22	30953312/G//C	nonsynonymous	0	1
INPP5J	chr22	31522937/C//T	synonymous	0	1
KLHDC7B	chr22	50986860/G//A	nonsynonymous	0	1
MAGEE2	chrX	75003804/A//G	synonymous	0	1
ITPKB	chr1	226827281/T//C	nonsynonymous	0	1
DNAH7	chr2	196849429/C//T	nonsynonymous	0	1
CYP8B1	chr3	42916000/C//T	nonsynonymous	0	1
ZNF717	chr3	75787159/C//T	nonsynonymous	0	1
PHF3	chr6	64412430/AGTGGAAC//A	nonsynonymous	0	1
ZNF92	chr7	64864074/C//T	synonymous	0	1
NYAP1	chr7	100084509/G//A	nonsynonymous	0	1
TFR2	chr7	100218639/C//G	synonymous	0	1
SLC39A12	chr10	18266997/G//A	nonsynonymous	0	1
GABRG3	chr15	27772774/C//T	nonsynonymous	0	1
SPIRE2	chr16	89916910/C//T	nonsynonymous	0	1
SLC43A2	chr17	1486571/C//T	nonsynonymous	0	1
TP53	chr17	7577609/C//T	nonsynonymous	0	1
MAP2K3	chr17	21207835/G//A	synonymous	0	1
ABCA7	chr19	1054323/C//T	nonsynonymous	0	1
DEDD2	chr19	42721059/C//G	nonsynonymous	0	1
JAKMIP2	chr5	146971254/A//T	synonymous	0	1
BMT2	chr7	112461978/T//C	nonsynonymous	0	1
CCDC166	chr8	144789931/G//A	nonsynonymous	0	1
PKN3	chr9	131468131/C//T	synonymous	0	1
ZFYVE26	chr14	68249846/G//GCCA	nonsynonymous	0	1
MYO5A	chr15	52708452/C//G	nonsynonymous	0	1
ZNF709	chr19	12575035/C//T	synonymous	0	1
AJAP1	chr1	4834599/C//T	synonymous	1	0
ZC3H12A	chr1	37948856/G//A	nonsynonymous	1	0
OR6Y1	chr1	158517563/G//T	synonymous	1	0
NUF2	chr1	163310172/A//G	nonsynonymous	1	0
TNN	chr1	175046943/G//A	nonsynonymous	1	0
BRINP3	chr1	190067657/G//A	nonsynonymous	1	0

USP34	chr2	61633162/C//G	nonsynonymous	1	0
TTC31	chr2	74720154/C//T	nonsynonymous	1	0
CLK1	chr2	201726007/C//A	nonsynonymous	1	0
GPR1	chr2	207041079/C//A	nonsynonymous	1	0
ABCA12	chr2	215876306/C//A	nonsynonymous	1	0
PTPRN	chr2	220173972/C//T	nonsynonymous	1	0
TGM4	chr3	44916130/G//T	synonymous	1	0
CCR3	chr3	46306706/C//T	synonymous	1	0
COL7A1	chr3	48621334/G//A	nonsynonymous	1	0
ROBO1	chr3	78667190/G//A	nonsynonymous	1	0
JAKMIP1	chr4	6086661/C//T	nonsynonymous	1	0
MAD2L1	chr4	120986839/C//A	nonsynonymous	1	0
TENM3	chr4	183267959/C//A	nonsynonymous	1	0
WWC2	chr4	184166609/C//T	nonsynonymous	1	0
ADCY2	chr5	7707861/C//T	synonymous	1	0
KCNN2	chr5	113740487/C//A	nonsynonymous	1	0
PCDHAC2	chr5	140346543/G//A	synonymous	1	0
PCDHB12	chr5	140590010/G//A	nonsynonymous	1	0
PCDHGA8	chr5	140772387/G//A	nonsynonymous	1	0
IBTK	chr6	82925884/C//G	nonsynonymous	1	0
CALCR	chr7	93125209/C//A	synonymous	1	0
TECPR1	chr7	97874258/C//T	nonsynonymous	1	0
RBM28	chr7	127979361/G//A	nonsynonymous	1	0
ZC3HAV1	chr7	138764890/G//T	nonsynonymous	1	0
GIMAP6	chr7	150325337/C//T	nonsynonymous	1	0
TNKS	chr8	9634208/C//T	nonsynonymous	1	0
PAG1	chr8	81897534/G//A	nonsynonymous	1	0
CNBD1	chr8	88296971/G//A	nonsynonymous	1	0
CPQ	chr8	97797533/C//T	synonymous	1	0
UNC13B	chr9	35378415/GGGA//G	nonsynonymous	1	0
PAX6	chr11	31824257/G//T	nonsynonymous	1	0
OR5D16	chr11	55606627/T//A	nonsynonymous	1	0
OR8U8	chr11	56143450/C//A	synonymous	1	0
HTR2A	chr13	47409736/C//T	nonsynonymous	1	0
CMA1	chr14	24974804/C//T	nonsynonymous	1	0
ESRRB	chr14	76928919/C//T	synonymous	1	0
PPP1R14D	chr15	41120881/T//C	synonymous	1	0
FEM1B	chr15	68570711/G//A	synonymous	1	0
KLHL25	chr15	86312269/G//A	nonsynonymous	1	0
C16orf96	chr16	4638190/C//A	nonsynonymous	1	0
BCAS3	chr17	59024616/A//G	nonsynonymous	1	0
DYRK1B	chr19	40317921/G//T	synonymous	1	0
SLC8A2	chr19	47960223/T//C	nonsynonymous	1	0
NUCB1	chr19	49414466/A//C	nonsynonymous	1	0
TARM1	chr19	54578145/C//G	nonsynonymous	1	0
RRP1	chr21	45219481/G//C	nonsynonymous	1	0
PLXNB2	chr22	50726369/C//A	nonsynonymous	1	0
CT47B1	chrX	120008869/G//A	nonsynonymous	1	0
RYR2	chr1	237954819/C//T	synonymous	0	1
CDCA7	chr2	174232418/TCTA//T	synonymous	0	1
TTN	chr2	179576050/G//A	nonsynonymous	0	1
POU4F2	chr4	147561500/A//C	nonsynonymous	0	1
FAXDC2	chr5	154202034/C//A	nonsynonymous	0	1
ELOVL2	chr6	10983985/A//G	synonymous	0	1
CDC40	chr6	110501698/G//A	synonymous	0	1
UTRN	chr6	144872220/T//A	nonsynonymous	0	1
PMS2	chr7	6026860/G//T	synonymous	0	1
DPP6	chr7	154561192/G//A	nonsynonymous	0	1
NAT1	chr8	18080283/A//G	nonsynonymous	0	1
CEBPD	chr8	48650359/G//A	synonymous	0	1
ARHGAP39	chr8	145771019/C//T	nonsynonymous	0	1
	chr9	116356436/G//A	synonymous	0	1
ENKUR	chr10	25304929/C//T	synonymous	0	1
SVIL	chr10	29821810/C//T	nonsynonymous	0	1
LSP1	chr11	1901336/G//A	nonsynonymous	0	1
OR52W1	chr11	6221235/T//A	nonsynonymous	0	1
MMP13	chr11	102822868/G//A	synonymous	0	1
SLC2A3	chr12	8083105/T//A	nonsynonymous	0	1
C1QL4	chr12	49726804/A//G	synonymous	0	1
CAND1	chr12	67675775/A//T	nonsynonymous	0	1

TBX3	chr12	115114119/T//C	synonymous	0	1
ARRB2	chr17	4621220/G//A	nonsynonymous	0	1
ZNF208	chr19	22154249/A//G	nonsynonymous	0	1
CD93	chr20	23066178/C//A	nonsynonymous	0	1
	chr20	39802757/G//A	synonymous	0	1
DIP2A	chr21	47957169/A//G	nonsynonymous	0	1
PPM1F	chr22	22287803/C//T	nonsynonymous	0	1
MN1	chr22	28195002/C//T	synonymous	0	1
SEC14L4	chr22	30891602/G//T	nonsynonymous	0	1
ZNF182	chrX	47835963/T//G	nonsynonymous	0	1
FAM132A	chr1	1179386/C//A	nonsynonymous	1	0
SCNN1D	chr1	1225868/T//C	nonsynonymous	1	0
ARHGEF16	chr1	3383907/G//A	synonymous	1	0
KCNAB2	chr1	6156688/G//T	synonymous	1	0
KLHL21	chr1	6659234/C//G	nonsynonymous	1	0
SLC2A5	chr1	9098566/C//A	nonsynonymous	1	0
EXOSC10	chr1	11137685/C//T	synonymous	1	0
MTOR	chr1	11194529/A//G	synonymous	1	0
DISP3	chr1	11561097/G//A	synonymous	1	0
KIAA2013	chr1	11985748/G//A	nonsynonymous	1	0
	chr1	19952003/G//A	synonymous	1	0
RAP1GAP	chr1	21940493/C//T	synonymous	1	0
ZBTB40	chr1	22817041/A//G	synonymous	1	0
EPHB2	chr1	23191387/C//T	nonsynonymous	1	0
AHDC1	chr1	27876919/C//T	nonsynonymous	1	0
OPRD1	chr1	29189458/G//T	nonsynonymous	1	0
MATN1	chr1	31189101/G//T	nonsynonymous	1	0
ZMYM1	chr1	35579238/C//T	nonsynonymous	1	0
CDCA8	chr1	38158272/C//T	synonymous	1	0
EPHA10	chr1	38192873/C//A	nonsynonymous	1	0
MACF1	chr1	39893807/C//T	synonymous	1	0
HIVEP3	chr1	41976647/C//G	synonymous	1	0
KDM4A	chr1	44156519/G//T	nonsynonymous	1	0
CCDC24	chr1	44458319/G//A	synonymous	1	0
C1orf228	chr1	45190814/G//T	nonsynonymous	1	0
MAST2	chr1	46497987/C//T	nonsynonymous	1	0
SSBP3	chr1	54871722/C//T	synonymous	1	0
PARS2	chr1	55224490/C//T	synonymous	1	0
C8A	chr1	57373626/C//T	synonymous	1	0
KANK4	chr1	62728944/C//A	nonsynonymous	1	0
RPE65	chr1	68904875/C//T	nonsynonymous	1	0
ERICH3	chr1	75036945/C//G	nonsynonymous	1	0
SLC44A5	chr1	75684230/T//G	nonsynonymous	1	0
SYDE2	chr1	85666410/G//C	synonymous	1	0
DDAH1	chr1	85790518/C//G	nonsynonymous	1	0
COL24A1	chr1	86591119/G//T	synonymous	1	0
ZNF644	chr1	91404519/C//T	nonsynonymous	1	0
CD101	chr1	117554544/C//T	nonsynonymous	1	0
GJA8	chr1	147380416/G//A	nonsynonymous	1	0
FLG	chr1	152276848/T//C	nonsynonymous	1	0
ATP8B2	chr1	154321492/C//T	synonymous	1	0
ZBTB7B	chr1	154987160/G//C	synonymous	1	0
ZBTB7B	chr1	154987446/G//C	nonsynonymous	1	0
BCAN	chr1	156616884/G//T	nonsynonymous	1	0
FCRL5	chr1	157494075/C//T	nonsynonymous	1	0
OR6Y1	chr1	158517412/T//A	nonsynonymous	1	0
SPTA1	chr1	158590229/G//T	nonsynonymous	1	0
OR6K2	chr1	158670205/T//G	nonsynonymous	1	0
OR6K3	chr1	158687452/C//A	nonsynonymous	1	0
SLAMF6	chr1	160466034/G//C	nonsynonymous	1	0
HSPA6	chr1	161494885/T//C	nonsynonymous	1	0
ADCY10	chr1	167793989/G//A	synonymous	1	0
TBX19	chr1	168278108/G//A	nonsynonymous	1	0
SELP	chr1	169578873/G//T	nonsynonymous	1	0
FMO3	chr1	171085371/G//T	nonsynonymous	1	0
FMO3	chr1	171086425/G//A	nonsynonymous	1	0
FMO3	chr1	171086501/G//A	synonymous	1	0
PAPPA2	chr1	176564136/G//A	nonsynonymous	1	0
CACNA1E	chr1	181680125/G//A	nonsynonymous	1	0
RHOU	chr1	228879380/C//G	nonsynonymous	1	0

OR2W5	chr1	247655163/C//A	nonsynonymous	1	0
OR2W3	chr1	248058946/C//A	synonymous	1	0
C2orf16	chr2	27800241/G//A	nonsynonymous	1	0
C2orf16	chr2	27800409/G//A	nonsynonymous	1	0
C2orf16	chr2	27800754/G//C	nonsynonymous	1	0
C2orf16	chr2	27800813/G//T	nonsynonymous	1	0
	chr2	27851333/G//C	synonymous	1	0
TTC27	chr2	32891832/C//G	synonymous	1	0
LTBP1	chr2	33586558/G//A	nonsynonymous	1	0
FSHR	chr2	49190942/C//A	nonsynonymous	1	0
EHBP1	chr2	63091982/G//C	nonsynonymous	1	0
AAK1	chr2	69748088/G//T	nonsynonymous	1	0
DYSF	chr2	71791180/C//T	synonymous	1	0
SFXN5	chr2	73172030/C//T	synonymous	1	0
ATOH8	chr2	85981929/C//T	nonsynonymous	1	0
TEX37	chr2	88828811/G//T	nonsynonymous	1	0
INPP4A	chr2	99172190/G//A	nonsynonymous	1	0
EIF5B	chr2	99980269/G//C	nonsynonymous	1	0
TMEM182	chr2	103414356/G//A	synonymous	1	0
MERTK	chr2	112725811/C//G	nonsynonymous	1	0
PSD4	chr2	113940771/C//T	synonymous	1	0
TNFAIP6	chr2	152222682/C//T	synonymous	1	0
NEB	chr2	152500642/T//C	nonsynonymous	1	0
BBS5	chr2	170350334/A//G	synonymous	1	0
TTN	chr2	179500287/C//T	nonsynonymous	1	0
COL3A1	chr2	189875015/G//A	nonsynonymous	1	0
HECW2	chr2	197297950/G//A	synonymous	1	0
PGAP1	chr2	197738442/T//C	synonymous	1	0
AOX1	chr2	201527577/G//C	nonsynonymous	1	0
ZDBF2	chr2	207169719/G//T	nonsynonymous	1	0
UNC80	chr2	210640747/G//A	synonymous	1	0
ACSL3	chr2	223783839/C//T	nonsynonymous	1	0
SLC19A3	chr2	228552941/T//G	nonsynonymous	1	0
PRSS56	chr2	233386552/C//T	nonsynonymous	1	0
UGT1A3	chr2	234638543/C//T	synonymous	1	0
HDAC4	chr2	240005892/G//A	nonsynonymous	1	0
CNTN6	chr3	1427489/T//C	synonymous	1	0
CNOT10	chr3	32726880/G//C	synonymous	1	0
UBP1	chr3	33444402/G//C	synonymous	1	0
KIF15	chr3	44852426/A//G	nonsynonymous	1	0
CACNA2D2	chr3	50405594/C//G	nonsynonymous	1	0
DNAH12	chr3	57431952/C//T	nonsynonymous	1	0
OR5AC2	chr3	97806690/T//G	nonsynonymous	1	0
ABI3BP	chr3	100585772/G//A	synonymous	1	0
DZIP3	chr3	108409708/C//T	synonymous	1	0
MORC1	chr3	108778716/T//A	nonsynonymous	1	0
USF3	chr3	113379494/C//T	synonymous	1	0
DIRC2	chr3	122545776/C//G	synonymous	1	0
MBD4	chr3	129151452/G//A	synonymous	1	0
COL6A5	chr3	130110423/G//C	nonsynonymous	1	0
PLSCR2	chr3	146173191/C//G	nonsynonymous	1	0
ZIC1	chr3	147130417/G//A	synonymous	1	0
PTX3	chr3	157160444/G//T	nonsynonymous	1	0
IFT80	chr3	160025526/C//T	nonsynonymous	1	0
VWA5B2	chr3	183959568/G//A	synonymous	1	0
CLCN2	chr3	184070114/G//A	synonymous	1	0
TNK2	chr3	195611833/C//T	synonymous	1	0
	chr3	196669250/G//C	synonymous	1	0
ZFYVE28	chr4	2272555/G//A	nonsynonymous	1	0
MAN2B2	chr4	6590784/G//A	nonsynonymous	1	0
TBCK	chr4	107183324/C//T	synonymous	1	0
ANK2	chr4	114186118/G//A	synonymous	1	0
KIAA1109	chr4	123195581/C//T	nonsynonymous	1	0
PCDH10	chr4	134072901/C//A	nonsynonymous	1	0
FREM3	chr4	144617001/G//T	nonsynonymous	1	0
TLR2	chr4	154625872/A//G	nonsynonymous	1	0
TKTL2	chr4	164394508/C//A	nonsynonymous	1	0
TMA16	chr4	164436581/G//T	nonsynonymous	1	0
HMGB2	chr4	174253275/C//G	nonsynonymous	1	0
ICE1	chr5	5462233/C//T	nonsynonymous	1	0

MED10	chr5	6378600/C//T	synonymous	1	0
TRIO	chr5	14498701/G//C	nonsynonymous	1	0
MROH2B	chr5	41000359/C//A	nonsynonymous	1	0
FBXO4	chr5	41930004/C//G	nonsynonymous	1	0
GRAMD3	chr5	125819198/A//C	nonsynonymous	1	0
LRRTM2	chr5	138210132/A//T	nonsynonymous	1	0
ANKHD1	chr5	139838217/A//G	nonsynonymous	1	0
GABRA1	chr5	161324263/C//A	synonymous	1	0
KIAA0319	chr6	24559354/C//A	nonsynonymous	1	0
	chr6	28198257/GT//G	synonymous	1	0
OR12D2	chr6	29365385/G//T	synonymous	1	0
HCG4	chr6	29759784/T//C	synonymous	1	0
PPP1R10	chr6	30577613/A//T	nonsynonymous	1	0
LY6G5B	chr6	31640068/G//A	synonymous	1	0
C6orf10	chr6	32335738/G//C	synonymous	1	0
TAP2	chr6	32805897/T//C	synonymous	1	0
UHRF1BP1	chr6	34804053/G//T	nonsynonymous	1	0
CCDC167	chr6	37467685/G//A	synonymous	1	0
KCNK16	chr6	39290140/G//T	synonymous	1	0
TDRD6	chr6	46661036/A//T	nonsynonymous	1	0
GFRAL	chr6	55223906/C//A	nonsynonymous	1	0
DST	chr6	56463388/T//C	synonymous	1	0
EYS	chr6	65301559/C//G	nonsynonymous	1	0
ADGRB3	chr6	69640507/G//T	nonsynonymous	1	0
COL12A1	chr6	75806970/G//A	nonsynonymous	1	0
IBTK	chr6	82906088/T//G	nonsynonymous	1	0
ZNF292	chr6	87969468/G//T	nonsynonymous	1	0
GABRR1	chr6	89899919/A//T	nonsynonymous	1	0
RRAGD	chr6	90082230/A//G	nonsynonymous	1	0
AK9	chr6	109980517/C//T	nonsynonymous	1	0
HS3ST5	chr6	114378975/C//T	nonsynonymous	1	0
PKIB	chr6	123038945/G//A	synonymous	1	0
SOGA3	chr6	127837673/C//T	synonymous	1	0
MOXD1	chr6	132722341/G//A	synonymous	1	0
REPS1	chr6	139247574/C//T	synonymous	1	0
HECA	chr6	139487803/G//T	synonymous	1	0
ADGB	chr6	146993453/T//G	nonsynonymous	1	0
TULP4	chr6	158922987/C//G	synonymous	1	0
FNDC1	chr6	159688918/A//G	synonymous	1	0
ELFN1	chr7	1785053/G//A	nonsynonymous	1	0
RADIL	chr7	4839315/C//G	nonsynonymous	1	0
WIPI2	chr7	5232787/G//T	nonsynonymous	1	0
WIPI2	chr7	5270512/C//T	synonymous	1	0
ACTB	chr7	5569032/C//T	nonsynonymous	1	0
GRID2IP	chr7	6590823/A//T	nonsynonymous	1	0
THSD7A	chr7	11676395/A//T	synonymous	1	0
VWDE	chr7	12379958/G//T	nonsynonymous	1	0
SCIN	chr7	12664670/G//A	synonymous	1	0
TRA2A	chr7	23545814/T//C	nonsynonymous	1	0
TBX20	chr7	35288387/C//G	synonymous	1	0
HECW1	chr7	43485109/G//T	nonsynonymous	1	0
EGFR	chr7	55273185/C//G	nonsynonymous	1	0
SUMF2	chr7	56147230/C//T	nonsynonymous	1	0
PTPN12	chr7	77256211/C//G	synonymous	1	0
PCLO	chr7	82579872/C//T	synonymous	1	0
PCLO	chr7	82584547/G//A	nonsynonymous	1	0
SEMA3E	chr7	83021900/C//A	synonymous	1	0
SEMA3D	chr7	84727263/C//G	nonsynonymous	1	0
DLX6	chr7	96637029/C//A	synonymous	1	0
DLX5	chr7	96653821/C//T	nonsynonymous	1	0
BAIAP2L1	chr7	97935801/C//T	synonymous	1	0
ZNF394	chr7	99091493/C//A	nonsynonymous	1	0
ZNF394	chr7	99091913/C//T	nonsynonymous	1	0
ZNF394	chr7	99092228/C//G	nonsynonymous	1	0
ZNF394	chr7	99092237/C//T	nonsynonymous	1	0
STAG3	chr7	99811671/C//T	synonymous	1	0
FBXO24	chr7	100189337/C//T	nonsynonymous	1	0
NAMPT	chr7	105891624/C//G	nonsynonymous	1	0
FAM71F2	chr7	128317840/G//A	synonymous	1	0
CPA4	chr7	129946665/C//T	synonymous	1	0

LRGUK	chr7	133827880/A//G	nonsynonymous	1	0
WDR60	chr7	158662594/C//T	nonsynonymous	1	0
CSMD1	chr8	3072125/G//T	synonymous	1	0
ZNF705G	chr8	7215671/G//A	nonsynonymous	1	0
VPS37A	chr8	17137765/G//T	nonsynonymous	1	0
NAT2	chr8	18257584/T//C	nonsynonymous	1	0
POLR3D	chr8	22107969/G//T	nonsynonymous	1	0
RHOBTB2	chr8	22863521/C//G	synonymous	1	0
TRIM35	chr8	27146728/G//C	synonymous	1	0
PTK2B	chr8	27255180/G//T	nonsynonymous	1	0
ZNF703	chr8	37555125/G//T	nonsynonymous	1	0
GOLGA7	chr8	41348336/T//A	synonymous	1	0
SLC20A2	chr8	42294700/C//T	nonsynonymous	1	0
CLVS1	chr8	62370901/C//A	nonsynonymous	1	0
C8orf44	chr8	67592100/C//A	nonsynonymous	1	0
PRDM14	chr8	70982062/G//A	nonsynonymous	1	0
	chr8	85097210/C//G	synonymous	1	0
RALYL	chr8	85785527/G//A	nonsynonymous	1	0
CDH17	chr8	95164199/G//A	nonsynonymous	1	0
RAD54B	chr8	95423415/C//A	nonsynonymous	1	0
PKHD1L1	chr8	110464394/A//T	nonsynonymous	1	0
CSMD3	chr8	113249445/T//C	nonsynonymous	1	0
AARD	chr8	117954894/C//T	nonsynonymous	1	0
FAM83A	chr8	124206337/G//A	nonsynonymous	1	0
FBXO32	chr8	124546968/A//C	nonsynonymous	1	0
SQLE	chr8	126021442/C//T	nonsynonymous	1	0
FAM135B	chr8	139163519/T//C	nonsynonymous	1	0
TOP1MT	chr8	144391641/G//A	synonymous	1	0
MROH6	chr8	144651884/C//A	nonsynonymous	1	0
PLEC	chr8	144994074/G//A	synonymous	1	0
PLEC	chr8	144995739/G//A	synonymous	1	0
PLEC	chr8	144995893/G//A	nonsynonymous	1	0
FOXD4	chr9	116940/C//T	nonsynonymous	1	0
GLDC	chr9	6553428/G//A	synonymous	1	0
PTPRD	chr9	8340361/C//A	nonsynonymous	1	0
HAUS6	chr9	19102636/G//A	nonsynonymous	1	0
FOCAD	chr9	20789352/C//G	synonymous	1	0
	chr9	21974583/G//A	synonymous	1	0
DNAI1	chr9	34512430/C//A	synonymous	1	0
SPAG8	chr9	35810968/C//G	synonymous	1	0
TRPM3	chr9	73478031/A//G	synonymous	1	0
PRUNE2	chr9	79325251/G//A	nonsynonymous	1	0
FRMD3	chr9	85913706/C//T	nonsynonymous	1	0
CDK20	chr9	90585703/T//A	nonsynonymous	1	0
AUH	chr9	93979572/T//C	nonsynonymous	1	0
FRRS1L	chr9	111903663/C//G	nonsynonymous	1	0
PKN3	chr9	131468097/A//T	nonsynonymous	1	0
PRRC2B	chr9	134308065/G//A	synonymous	1	0
PRRC2B	chr9	134363470/C//T	nonsynonymous	1	0
DDX31	chr9	135545417/C//G	nonsynonymous	1	0
SLC2A6	chr9	136339215/C//T	synonymous	1	0
SLC2A6	chr9	136342335/C//T	nonsynonymous	1	0
CAMSAP1	chr9	138703218/G//C	nonsynonymous	1	0
SEC16A	chr9	139350150/G//C	synonymous	1	0
ZMYND19	chr9	140477587/G//A	nonsynonymous	1	0
CACNA1B	chr9	140880876/C//A	nonsynonymous	1	0
FAM170B	chr10	50339892/G//T	nonsynonymous	1	0
C10orf71	chr10	50530816/C//A	nonsynonymous	1	0
C10orf71	chr10	50530830/C//A	synonymous	1	0
PCDH15	chr10	55582167/A//G	synonymous	1	0
PCDH15	chr10	55826601/G//T	nonsynonymous	1	0
TBATA	chr10	72538356/C//A	nonsynonymous	1	0
CDH23	chr10	73539144/G//C	nonsynonymous	1	0
NDST2	chr10	75567268/G//A	synonymous	1	0
CCSER2	chr10	86131500/C//A	nonsynonymous	1	0
PTEN	chr10	89717615/C//T	nonsynonymous	1	0
HTR7	chr10	92502206/T//G	synonymous	1	0
TNKS2	chr10	93601930/A//T	nonsynonymous	1	0
LGI1	chr10	95553074/G//C	nonsynonymous	1	0
MMS19	chr10	99228139/G//A	nonsynonymous	1	0

PNLIPRP3	chr10	118231366/A//G	nonsynonymous	1	0
GPR26	chr10	125434427/C//G	nonsynonymous	1	0
ANO9	chr11	431698/T//C	nonsynonymous	1	0
TRPM5	chr11	2434743/A//C	nonsynonymous	1	0
NAP1L4	chr11	2992765/C//G	nonsynonymous	1	0
OR51G2	chr11	4936516/G//A	synonymous	1	0
OR52J3	chr11	5068334/C//T	synonymous	1	0
OR52D1	chr11	5510505/T//A	nonsynonymous	1	0
MRGPRX4	chr11	18194787/G//A	synonymous	1	0
HIPK3	chr11	33308138/A//G	nonsynonymous	1	0
TRIM44	chr11	35747683/C//G	nonsynonymous	1	0
MADD	chr11	47311504/G//T	nonsynonymous	1	0
OR5D18	chr11	55587794/G//T	nonsynonymous	1	0
OR5F1	chr11	55761660/C//A	nonsynonymous	1	0
OR8H2	chr11	55873438/G//A	nonsynonymous	1	0
OR9G4	chr11	56510496/C//G	nonsynonymous	1	0
	chr11	61313652/C//G	synonymous	1	0
ROM1	chr11	62382135/G//A	nonsynonymous	1	0
GANAB	chr11	62397707/C//A	nonsynonymous	1	0
SLC22A6	chr11	62747212/G//T	nonsynonymous	1	0
SLC22A9	chr11	63141134/G//T	synonymous	1	0
NRXN2	chr11	64374593/G//T	synonymous	1	0
MEN1	chr11	64575503/C//T	nonsynonymous	1	0
CDC42BPG	chr11	64594187/G//C	nonsynonymous	1	0
LTBP3	chr11	65321727/G//A	synonymous	1	0
	chr11	65479815/C//T	synonymous	1	0
DRAP1	chr11	65686987/G//C	nonsynonymous	1	0
DRAP1	chr11	65687256/G//A	synonymous	1	0
GPR152	chr11	67219059/C//A	synonymous	1	0
MYO7A	chr11	76868003/G//T	nonsynonymous	1	0
FAT3	chr11	92258000/G//T	nonsynonymous	1	0
GUCY1A2	chr11	106888851/G//T	synonymous	1	0
ATM	chr11	108098602/G//A	nonsynonymous	1	0
CADM1	chr11	115047300/G//T	nonsynonymous	1	0
BCL9L	chr11	118772862/C//T	synonymous	1	0
BCL9L	chr11	118772879/C//T	nonsynonymous	1	0
OR10G4	chr11	123886959/G//T	synonymous	1	0
OR8D2	chr11	124189167/C//A	nonsynonymous	1	0
	chr11	126276146/G//T	synonymous	1	0
KIRREL3	chr11	126432769/C//A	nonsynonymous	1	0
CACNA1C	chr12	2764391/C//A	nonsynonymous	1	0
VWF	chr12	6153620/C//T	synonymous	1	0
VAMP1	chr12	6575058/C//G	nonsynonymous	1	0
MLF2	chr12	6858111/C//T	synonymous	1	0
	chr12	6925068/G//A	synonymous	1	0
A2M	chr12	9260184/T//C	nonsynonymous	1	0
ITPR2	chr12	26628328/A//G	synonymous	1	0
ADAMTS20	chr12	43944991/G//A	synonymous	1	0
OR10AD1	chr12	48596553/T//C	nonsynonymous	1	0
SCN8A	chr12	52139810/C//G	nonsynonymous	1	0
ACVR1B	chr12	52385756/C//T	synonymous	1	0
TNS2	chr12	53454263/C//G	nonsynonymous	1	0
TIMELESS	chr12	56818592/G//C	nonsynonymous	1	0
USP15	chr12	62715244/G//C	nonsynonymous	1	0
CAPS2	chr12	75685575/C//A	nonsynonymous	1	0
HSP90B1	chr12	104341494/C//T	synonymous	1	0
ALDH1L2	chr12	105418260/A//G	synonymous	1	0
SPPL3	chr12	121204063/C//A	synonymous	1	0
SETD1B	chr12	122248307/G//A	nonsynonymous	1	0
ULK1	chr12	132397757/G//A	nonsynonymous	1	0
CENPJ	chr13	25473709/C//T	nonsynonymous	1	0
PCDH9	chr13	67802323/C//T	nonsynonymous	1	0
MZT1	chr13	73301712/G//A	nonsynonymous	1	0
SOX21	chr13	95363411/C//A	synonymous	1	0
CCDC168	chr13	103387959/T//C	nonsynonymous	1	0
CCDC168	chr13	103389437/C//A	nonsynonymous	1	0
TUBGCP3	chr13	113208413/C//A	synonymous	1	0
EDDM3A	chr14	21216139/G//C	nonsynonymous	1	0
SLC39A2	chr14	21469324/C//T	synonymous	1	0
OR4E2	chr14	22133659/TC//T	nonsynonymous	1	0

CEBPE	chr14	23588197/T//A	nonsynonymous	1	0
MYH6	chr14	23866429/G//C	nonsynonymous	1	0
EGLN3	chr14	34419631/C//T	nonsynonymous	1	0
NEMF	chr14	50300193/G//T	nonsynonymous	1	0
SYNE2	chr14	64518507/G//C	nonsynonymous	1	0
ZBTB25	chr14	64954476/C//G	nonsynonymous	1	0
PLEKHH1	chr14	68044918/T//A	nonsynonymous	1	0
GALNT16	chr14	69795176/G//C	nonsynonymous	1	0
LTBP2	chr14	74970642/G//T	nonsynonymous	1	0
AREL1	chr14	75136712/C//A	nonsynonymous	1	0
YLPM1	chr14	75276398/G//T	nonsynonymous	1	0
TMEM63C	chr14	77715633/G//T	nonsynonymous	1	0
CEP128	chr14	81371163/C//T	nonsynonymous	1	0
TTC7B	chr14	91007590/C//G	synonymous	1	0
UNC79	chr14	94088662/G//T	nonsynonymous	1	0
BDKRB1	chr14	96730361/C//T	synonymous	1	0
XRCC3	chr14	104173386/G//A	synonymous	1	0
AKT1	chr14	105242094/G//C	synonymous	1	0
CDCA4	chr14	105477522/G//C	synonymous	1	0
TMEM121	chr14	105995871/G//C	nonsynonymous	1	0
GABRG3	chr15	27778007/G//A	nonsynonymous	1	0
SRP14	chr15	40328521/A//G	synonymous	1	0
GANC	chr15	42566705/T//C	nonsynonymous	1	0
STARD9	chr15	42984093/G//C	nonsynonymous	1	0
WDR72	chr15	54005060/G//A	nonsynonymous	1	0
UNC13C	chr15	54556551/C//A	nonsynonymous	1	0
UNC13C	chr15	54919042/A//T	nonsynonymous	1	0
SAXO2	chr15	82564106/T//A	synonymous	1	0
RHCG	chr15	90021178/C//A	nonsynonymous	1	0
TICRR	chr15	90119385/G//A	nonsynonymous	1	0
CRTC3-AS1	chr15	91184298/G//T	synonymous	1	0
ADCY9	chr16	4016403/C//T	nonsynonymous	1	0
TEKT5	chr16	10729649/C//G	nonsynonymous	1	0
TEKT5	chr16	10729730/C//G	nonsynonymous	1	0
GP2	chr16	20335462/C//A	nonsynonymous	1	0
ERN2	chr16	23724565/C//G	synonymous	1	0
GTF3C1	chr16	27503952/G//A	nonsynonymous	1	0
PHKG2	chr16	30767938/G//C	nonsynonymous	1	0
ZNF646	chr16	31091190/T//C	nonsynonymous	1	0
	chr16	31476490/C//T	synonymous	1	0
MYLK3	chr16	46766141/C//A	nonsynonymous	1	0
CHD9	chr16	53281210/G//A	nonsynonymous	1	0
RBL2	chr16	53496570/G//C	synonymous	1	0
NLRC5	chr16	57111659/G//T	nonsynonymous	1	0
CNOT1	chr16	58585122/C//T	nonsynonymous	1	0
CTCF	chr16	67644949/C//G	nonsynonymous	1	0
WDR59	chr16	74926460/C//G	nonsynonymous	1	0
VAT1L	chr16	77896747/C//A	nonsynonymous	1	0
JPH3	chr16	87678560/G//T	nonsynonymous	1	0
ZZEF1	chr17	4007973/G//A	synonymous	1	0
TP53	chr17	7578461/C//A	nonsynonymous	1	0
CNTROB	chr17	7843483/G//A	nonsynonymous	1	0
MYO15A	chr17	18022509/C//T	nonsynonymous	1	0
ALDH3A1	chr17	19642911/G//C	nonsynonymous	1	0
LGALS9B	chr17	20354898/C//T	nonsynonymous	1	0
SARM1	chr17	26708775/C//T	nonsynonymous	1	0
NF1	chr17	29587418/A//G	nonsynonymous	1	0
EVI2A	chr17	29645568/G//C	nonsynonymous	1	0
EVI2A	chr17	29645572/G//A	synonymous	1	0
NF1	chr17	29654724/C//T	nonsynonymous	1	0
CDK5R1	chr17	30815538/G//T	synonymous	1	0
MMP28	chr17	34095305/C//T	nonsynonymous	1	0
KRT38	chr17	39596456/G//T	nonsynonymous	1	0
TMEM92	chr17	48356248/C//A	nonsynonymous	1	0
PTRH2	chr17	57775303/G//A	nonsynonymous	1	0
CACNG4	chr17	65026770/G//C	nonsynonymous	1	0
UNC13D	chr17	73824173/G//A	synonymous	1	0
PRCD	chr17	74536649/C//T	nonsynonymous	1	0
RNF213	chr17	78311731/G//C	nonsynonymous	1	0
ENDOV	chr17	78389521/C//G	nonsynonymous	1	0

CEP76	chr18	12673455/C//T	nonsynonymous	1	0
SEH1L	chr18	12948110/C//T	synonymous	1	0
DSC1	chr18	28719849/G//C	nonsynonymous	1	0
DSG2	chr18	29118910/G//T	synonymous	1	0
SLC39A6	chr18	33694390/C//T	nonsynonymous	1	0
LOXHD1	chr18	44087641/G//T	nonsynonymous	1	0
DCC	chr18	50923746/G//T	synonymous	1	0
DCC	chr18	50985717/G//A	nonsynonymous	1	0
SERPINB5	chr18	61151660/C//T	synonymous	1	0
SOCS6	chr18	67992285/C//T	synonymous	1	0
ZNF236	chr18	74583674/C//G	nonsynonymous	1	0
PRTN3	chr19	843546/G//A	synonymous	1	0
PRTN3	chr19	843922/G//A	nonsynonymous	1	0
DOT1L	chr19	2180709/C//G	synonymous	1	0
C19orf35	chr19	2276358/C//G	nonsynonymous	1	0
C19orf35	chr19	2276386/C//T	nonsynonymous	1	0
TMPRSS9	chr19	2408554/C//T	nonsynonymous	1	0
PNPLA6	chr19	7620510/G//C	nonsynonymous	1	0
MUC16	chr19	9007823/C//A	nonsynonymous	1	0
MUC16	chr19	9082632/C//A	nonsynonymous	1	0
OR7D4	chr19	9325343/G//T	synonymous	1	0
ZNF491	chr19	11917384/G//T	nonsynonymous	1	0
JUNB	chr19	12903478/A//G	nonsynonymous	1	0
IL27RA	chr19	14163014/C//A	nonsynonymous	1	0
BRD4	chr19	15375222/G//A	nonsynonymous	1	0
BRD4	chr19	15375245/G//C	nonsynonymous	1	0
BRD4	chr19	15376368/G//A	nonsynonymous	1	0
MYO9B	chr19	17256266/C//G	nonsynonymous	1	0
KCNN1	chr19	18084632/C//T	synonymous	1	0
MAST3	chr19	18235458/G//C	nonsynonymous	1	0
MAU2	chr19	19431858/G//C	nonsynonymous	1	0
ZNF253	chr19	20003149/G//T	nonsynonymous	1	0
ZNF257	chr19	22255730/C//G	synonymous	1	0
ZNF98	chr19	22574378/A//G	synonymous	1	0
TSHZ3	chr19	31767750/G//C	synonymous	1	0
ATP4A	chr19	36046622/G//A	synonymous	1	0
ZNF585B	chr19	37677661/G//A	nonsynonymous	1	0
EID2B	chr19	40023261/G//C	nonsynonymous	1	0
LGALS13	chr19	40097990/C//A	synonymous	1	0
CEACAM3	chr19	42315244/G//C	nonsynonymous	1	0
	chr19	44896475/G//C	synonymous	1	0
CLASRP	chr19	45567278/C//T	nonsynonymous	1	0
CLASRP	chr19	45567673/C//T	synonymous	1	0
CCDC9	chr19	47768149/C//A	nonsynonymous	1	0
DHX34	chr19	47861273/G//T	nonsynonymous	1	0
EHD2	chr19	48244378/G//C	nonsynonymous	1	0
NUCB1	chr19	49425736/G//T	synonymous	1	0
HRC	chr19	49657090/T//A	nonsynonymous	1	0
SPIB	chr19	50926966/G//T	synonymous	1	0
FAM71E1	chr19	50970959/C//G	nonsynonymous	1	0
	chr19	50985126/C//T	synonymous	1	0
PPP2R1A	chr19	52719823/G//A	synonymous	1	0
LAIR2	chr19	55019361/G//A	nonsynonymous	1	0
BRSK1	chr19	55805584/C//G	nonsynonymous	1	0
ZNF865	chr19	56127194/C//G	nonsynonymous	1	0
USP29	chr19	57642565/A//T	nonsynonymous	1	0
ZSCAN22	chr19	58846324/C//A	nonsynonymous	1	0
SLC52A3	chr20	744340/G//T	nonsynonymous	1	0
UBOX5	chr20	3090963/G//A	synonymous	1	0
SIGLEC1	chr20	3677868/C//A	synonymous	1	0
PROKR2	chr20	5294809/G//T	synonymous	1	0
MCM8	chr20	5965591/C//G	nonsynonymous	1	0
NECAB3	chr20	32247332/C//T	nonsynonymous	1	0
RBL1	chr20	35668613/G//A	nonsynonymous	1	0
PTPRT	chr20	40735470/C//G	nonsynonymous	1	0
SNX21	chr20	44462966/G//T	nonsynonymous	1	0
PMEPA1	chr20	56227302/C//T	nonsynonymous	1	0
KRTAP19-6	chr21	31914008/G//T	nonsynonymous	1	0
PWP2	chr21	45545952/G//C	nonsynonymous	1	0
COL18A1	chr21	46930135/G//T	nonsynonymous	1	0

THAP7	chr22	21354209/A//G	nonsynonymous	1	0
SPECC1L	chr22	24718114/G//T	nonsynonymous	1	0
SGSM1	chr22	25243638/G//T	synonymous	1	0
GTPBP1	chr22	39120351/G//T	nonsynonymous	1	0
SUN2	chr22	39132377/C//T	synonymous	1	0
ENTHD1	chr22	40140264/G//T	nonsynonymous	1	0
FAM118A	chr22	45728463/A//G	nonsynonymous	1	0
CELSR1	chr22	46761239/C//T	nonsynonymous	1	0
CERK	chr22	47086028/C//T	nonsynonymous	1	0
NLGN4X	chrX	6069169/G//C	nonsynonymous	1	0
VSIG4	chrX	65252485/G//C	nonsynonymous	1	0
HEPH	chrX	65411995/G//T	nonsynonymous	1	0
NONO	chrX	70510482/G//T	synonymous	1	0
MAGEE1	chrX	75649625/A//T	synonymous	1	0
ZCCHC5	chrX	77913882/C//A	synonymous	1	0
SH3BGRL	chrX	80457597/C//G	synonymous	1	0
TCEAL6	chrX	101395922/C//A	nonsynonymous	1	0
TRPC5	chrX	111095610/G//A	synonymous	1	0
ZCCHC16	chrX	111698128/C//A	nonsynonymous	1	0
MCF2	chrX	138708799/G//T	nonsynonymous	1	0
HCFC1	chrX	153225812/G//A	nonsynonymous	1	0
MECP2	chrX	153296179/T//C	nonsynonymous	1	0
VAMP7	chrX	155169418/G//T	nonsynonymous	1	0
UTY	chrY	15448202/C//G	nonsynonymous	1	0
TTN	chr2	179516838/T//G	nonsynonymous	0	1
HSPE1-MOB4	chr2	198404875/C//A	nonsynonymous	0	1
	chr5	76987358/T//C	synonymous	0	1
C6orf223	chr6	43968343/T//A	synonymous	0	1
ZNF627	chr19	11708414/A//G	synonymous	0	1
ACTN2	chr1	236898996/C//G	nonsynonymous	0	1
STEAP3	chr2	120005350/G//A	synonymous	0	1
SDPR	chr2	192700549/C//T	synonymous	0	1
RBMS3	chr3	29804395/T//C	synonymous	0	1
IRX4	chr5	1878831/G//A	nonsynonymous	0	1
GRM1	chr6	146719926/G//A	nonsynonymous	0	1
ADGB	chr6	147062272/T//C	synonymous	0	1
KRTAP5-7	chr11	71238418/T//C	synonymous	0	1
ELF1	chr13	41515375/G//T	nonsynonymous	0	1
FSCB	chr14	44975650/C//A	nonsynonymous	0	1
	chr14	76525620/C//T	synonymous	0	1
CDK5RAP3	chr17	46058832/C//T	synonymous	0	1
FBLN1	chr22	45923784/G//A	nonsynonymous	0	1
CLCN4	chrX	10176538/C//T	nonsynonymous	0	1
CDX4	chrX	72667577/C//T	nonsynonymous	0	1
FGGY	chr1	59844482/C//G	nonsynonymous	0	1
NRXN1	chr2	50318544/T//G	nonsynonymous	0	1
IWS1	chr2	128253649/G//A	synonymous	0	1
FIP1L1	chr4	54257307/G//T	nonsynonymous	0	1
PDZD2	chr5	32093017/G//T	nonsynonymous	0	1
TTK	chr6	80751855/G//A	nonsynonymous	0	1
BRAT1	chr7	2578199/A//C	nonsynonymous	0	1
DDX58	chr9	32492399/A//G	synonymous	0	1
GAL3ST3	chr11	65810612/G//T	nonsynonymous	0	1
P4HA3	chr11	73978275/C//T	synonymous	0	1
CNTN5	chr11	99827542/C//A	synonymous	0	1
NALCN	chr13	101890123/G//A	nonsynonymous	0	1
BEAN1	chr16	66514563/C//A	nonsynonymous	0	1
PIK3R6	chr17	8732065/G//A	nonsynonymous	0	1
NCOR1	chr17	16055279/A//G	nonsynonymous	0	1
SLC27A1	chr19	17612122/C//A	synonymous	0	1
ZNF845	chr19	53856097/G//A	synonymous	0	1
NLRP12	chr19	54314024/C//T	nonsynonymous	0	1
TTC3	chr21	38512955/T//TG	nonsynonymous	0	1
RGAG1	chrX	109698430/C//A	nonsynonymous	0	1
TAS1R2	chr1	19175994/G//T	synonymous	1	0
UBR4	chr1	19415267/T//A	nonsynonymous	1	0
PQLC2	chr1	19653800/G//T	nonsynonymous	1	0
C1orf234	chr1	23337381/C//T	synonymous	1	0
GPN2	chr1	27216603/C//T	synonymous	1	0
TGFBR3	chr1	92187590/C//A	nonsynonymous	1	0

SLC16A4	chr1	110906499/A//G	synonymous	1	0
FCRL2	chr1	157739788/C//T	nonsynonymous	1	0
LRRC52	chr1	165532993/G//C	nonsynonymous	1	0
RGS18	chr1	192128387/C//A	nonsynonymous	1	0
IRF6	chr1	209963889/A//T	synonymous	1	0
USH2A	chr1	216420117/C//G	synonymous	1	0
PXDN	chr2	1668756/G//A	nonsynonymous	1	0
MAP4K3	chr2	39553298/T//C	synonymous	1	0
PSME4	chr2	54152713/C//A	nonsynonymous	1	0
OTX1	chr2	63281174/C//G	synonymous	1	0
ZNF638	chr2	71654391/A//G	nonsynonymous	1	0
DNAH6	chr2	84864357/G//A	synonymous	1	0
EN1	chr2	119600683/G//A	nonsynonymous	1	0
PTPN4	chr2	120704089/T//C	synonymous	1	0
CXCR4	chr2	136872530/C//A	nonsynonymous	1	0
MARCH7	chr2	160608984/C//A	nonsynonymous	1	0
TBR1	chr2	162273225/G//A	nonsynonymous	1	0
SLC4A10	chr2	162762239/T//C	synonymous	1	0
TTN	chr2	179594215/G//T	nonsynonymous	1	0
CD28	chr2	204599609/C//A	nonsynonymous	1	0
PARD3B	chr2	206023514/G//T	synonymous	1	0
UNC80	chr2	210683944/AC//A	nonsynonymous	1	0
DOCK10	chr2	225639731/G//A	synonymous	1	0
IQSEC1	chr3	12983224/G//A	synonymous	1	0
CYP8B1	chr3	42916843/C//A	nonsynonymous	1	0
STAB1	chr3	52556576/C//T	nonsynonymous	1	0
DNAH12	chr3	57509595/T//C	nonsynonymous	1	0
BTLA	chr3	112185044/T//G	nonsynonymous	1	0
ARHGAP31	chr3	119134770/G//T	nonsynonymous	1	0
RYK	chr3	133896895/CAT//C	nonsynonymous	1	0
CLSTN2	chr3	140285027/G//T	nonsynonymous	1	0
HTR3D	chr3	183750728/A//G	nonsynonymous	1	0
ZNF718	chr4	155880/C//A	nonsynonymous	1	0
ZNF721	chr4	436217/C//T	nonsynonymous	1	0
SLIT2	chr4	20530600/T//A	nonsynonymous	1	0
PCDH7	chr4	30724248/G//T	nonsynonymous	1	0
EPHA5	chr4	66356264/A//C	nonsynonymous	1	0
CENPC	chr4	68374716/T//A	nonsynonymous	1	0
TMPRSS11B	chr4	69093792/T//C	nonsynonymous	1	0
ENAM	chr4	71503558/G//T	nonsynonymous	1	0
BMPR1B	chr4	96036848/C//G	nonsynonymous	1	0
FGG	chr4	155531221/T//C	nonsynonymous	1	0
TLL1	chr4	166929161/T//A	nonsynonymous	1	0
FAM149A	chr4	187077194/A//T	nonsynonymous	1	0
CDH18	chr5	19473350/A//C	synonymous	1	0
C9	chr5	39341753/C//T	nonsynonymous	1	0
HAPLN1	chr5	82969250/G//T	nonsynonymous	1	0
ADGRV1	chr5	90040901/T//C	nonsynonymous	1	0
PCDHA11	chr5	140249967/C//A	synonymous	1	0
PCDHB1	chr5	140431237/C//A	nonsynonymous	1	0
PCDHB5	chr5	140515745/A//T	nonsynonymous	1	0
FAM114A2	chr5	153374519/G//A	nonsynonymous	1	0
TIMD4	chr5	156378738/G//T	nonsynonymous	1	0
GCM2	chr6	10874768/G//T	synonymous	1	0
TDP2	chr6	24667031/A//G	synonymous	1	0
TRIM40	chr6	30114824/G//A	synonymous	1	0
COL11A2	chr6	33151951/G//A	nonsynonymous	1	0
DNAH8	chr6	38821058/G//C	nonsynonymous	1	0
RHAG	chr6	49585879/C//G	nonsynonymous	1	0
HMGCLL1	chr6	55364076/G//GT	nonsynonymous	1	0
SPACA1	chr6	88768436/G//A	nonsynonymous	1	0
FUT9	chr6	96651553/C//A	nonsynonymous	1	0
MCHR2	chr6	100382373/A//G	nonsynonymous	1	0
CEP85L	chr6	118803087/G//C	nonsynonymous	1	0
ADGB	chr6	147103239/A//T	nonsynonymous	1	0
PLEKHG1	chr6	151152926/G//A	synonymous	1	0
HOXA7	chr7	27195942/C//A	nonsynonymous	1	0
ELMO1	chr7	37172821/TGGCA//T	nonsynonymous	1	0
CDK13	chr7	40085593/A//G	nonsynonymous	1	0
ZNF804B	chr7	88966283/T//G	synonymous	1	0

TMEM130	chr7	98445672/G//T	synonymous	1	0
MUC17	chr7	100681922/G//T	nonsynonymous	1	0
SND1	chr7	127292423/C//A	synonymous	1	0
DGKI	chr7	137076061/C//A	nonsynonymous	1	0
OR6B1	chr7	143701942/C//G	nonsynonymous	1	0
WDR60	chr7	158719137/G//T	nonsynonymous	1	0
CHD7	chr8	61763075/C//A	synonymous	1	0
DCSTAMP	chr8	105367193/T//C	nonsynonymous	1	0
PKHD1L1	chr8	110457826/T//A	nonsynonymous	1	0
FAM135B	chr8	139209851/A//T	nonsynonymous	1	0
LY6K	chr8	143784532/G//T	nonsynonymous	1	0
	chr8	144671206/C//A	synonymous	1	0
CDKN2A	chr9	21971017/G//A	nonsynonymous	1	0
ZNF462	chr9	109688639/G//C	nonsynonymous	1	0
BARHL1	chr9	135464779/C//A	nonsynonymous	1	0
TSC1	chr9	135801105/C//A	nonsynonymous	1	0
ABCA2	chr9	139906796/C//A	nonsynonymous	1	0
SPAG6	chr10	22678174/G//A	nonsynonymous	1	0
GPR158	chr10	25510031/G//C	nonsynonymous	1	0
HNRNPF	chr10	43882712/T//C	synonymous	1	0
ALOX5	chr10	45939200/G//T	nonsynonymous	1	0
	chr10	55570347/C//T	synonymous	1	0
	chr10	79814481/A//C	synonymous	1	0
TAF5	chr10	105137996/C//A	nonsynonymous	1	0
FRG2B	chr10	135438858/C//A	nonsynonymous	1	0
OR56A4	chr11	6024071/G//A	nonsynonymous	1	0
OR52B2	chr11	6190634/C//A	nonsynonymous	1	0
FIBIN	chr11	27016550/C//A	nonsynonymous	1	0
KIAA1549L	chr11	33566485/TAA//T	nonsynonymous	1	0
SLC1A2	chr11	35338940/G//T	synonymous	1	0
OR5R1	chr11	56185229/G//C	synonymous	1	0
TCN1	chr11	59629059/A//G	nonsynonymous	1	0
MS4A3	chr11	59828646/G//A	nonsynonymous	1	0
MTMR2	chr11	95574855/T//TG	nonsynonymous	1	0
TRPC6	chr11	101342020/G//A	nonsynonymous	1	0
TMPRSS5	chr11	113563894/C//A	nonsynonymous	1	0
OR10G8	chr11	123900829/A//C	nonsynonymous	1	0
PATE3	chr11	125659285/G//T	synonymous	1	0
KCNA6	chr12	4920569/G//T	synonymous	1	0
KMT2D	chr12	49426354/T//C	nonsynonymous	1	0
KRT74	chr12	52962018/G//A	synonymous	1	0
MON2	chr12	62981861/G//T	nonsynonymous	1	0
KCNMB4	chr12	70824339/C//T	nonsynonymous	1	0
AACS	chr12	125609287/A//C	synonymous	1	0
CHFR	chr12	133428309/C//T	nonsynonymous	1	0
SKA3	chr13	21742311/C//A	nonsynonymous	1	0
MTUS2	chr13	29598877/G//C	nonsynonymous	1	0
KLHL1	chr13	70681571/A//G	synonymous	1	0
GZMB	chr14	25100291/T//C	nonsynonymous	1	0
GPR33	chr14	31953075/C//T	nonsynonymous	1	0
ANGEL1	chr14	77255689/C//G	nonsynonymous	1	0
PPP4R4	chr14	94640904/C//A	synonymous	1	0
PPP1R13B	chr14	104206346/C//G	nonsynonymous	1	0
CYFIP1	chr15	22969157/C//G	synonymous	1	0
RYR3	chr15	33936626/C//A	nonsynonymous	1	0
SHC4	chr15	49149902/T//C	nonsynonymous	1	0
CEMIP	chr15	81234184/G//A	synonymous	1	0
NTRK3	chr15	88476420/G//A	synonymous	1	0
FAM169B	chr15	98982906/C//T	nonsynonymous	1	0
PTX4	chr16	1536479/A//T	nonsynonymous	1	0
RPL3L	chr16	1997279/C//A	nonsynonymous	1	0
SRRM2	chr16	2820678/G//GA	nonsynonymous	1	0
CDIP1	chr16	4562627/C//T	synonymous	1	0
GRIN2A	chr16	9862919/C//A	nonsynonymous	1	0
ABCC1	chr16	16150143/G//T	synonymous	1	0
GP2	chr16	20334304/A//T	synonymous	1	0
ACSM1	chr16	20638641/G//A	synonymous	1	0
CLN3	chr16	28488850/C//A	nonsynonymous	1	0
ZNF469	chr16	88501863/C//G	nonsynonymous	1	0
TP53	chr17	7578490/A//C	nonsynonymous	1	0

DHRS7B	chr17	21087744/A//G	nonsynonymous	1	0
CRLF3	chr17	29123220/T//G	nonsynonymous	1	0
ATXN7L3	chr17	42275391/T//G	synonymous	1	0
IGF2BP1	chr17	47122363/C//T	nonsynonymous	1	0
ENPP7	chr17	77708843/G//A	nonsynonymous	1	0
NOL4	chr18	31538256/C//A	nonsynonymous	1	0
CCDC102B	chr18	66541921/G//T	nonsynonymous	1	0
EVI5L	chr19	7911409/G//A	synonymous	1	0
MUC16	chr19	9017003/C//A	nonsynonymous	1	0
ICAM1	chr19	10395145/A//G	nonsynonymous	1	0
PRKCSH	chr19	11548753/C//T	synonymous	1	0
MRI1	chr19	13875781/G//A	nonsynonymous	1	0
RAB8A	chr19	16232590/G//A	synonymous	1	0
TMEM38A	chr19	16797155/G//T	nonsynonymous	1	0
CEP89	chr19	33430789/C//T	nonsynonymous	1	0
PDCD2L	chr19	34895662/C//T	nonsynonymous	1	0
ATP1A3	chr19	42492166/C//A	synonymous	1	0
PSG11	chr19	43530583/C//A	synonymous	1	0
DMWD	chr19	46288981/G//A	synonymous	1	0
PRR12	chr19	50124811/A//G	nonsynonymous	1	0
MYBPC2	chr19	50938469/A//G	nonsynonymous	1	0
CEACAM18	chr19	51983625/C//A	nonsynonymous	1	0
PEG3	chr19	57327547/T//A	nonsynonymous	1	0
ZNF772	chr19	57988758/T//A	synonymous	1	0
ZNF419	chr19	58005010/G//A	nonsynonymous	1	0
RBCK1	chr20	401662/C//T	nonsynonymous	1	0
PAK5	chr20	9560813/C//A	nonsynonymous	1	0
MACROD2	chr20	15866458/T//A	nonsynonymous	1	0
DTD1	chr20	18576673/G//A	nonsynonymous	1	0
C20orf78	chr20	18810827/G//T	synonymous	1	0
ZNF341	chr20	32369109/T//C	synonymous	1	0
ZNFX1	chr20	47869275/A//G	synonymous	1	0
KRTAP13-2	chr21	31744537/C//T	synonymous	1	0
P2RX6	chr22	21381027/C//T	synonymous	1	0
ANKRD54	chr22	38228970/T//C	nonsynonymous	1	0
ZC3H7B	chr22	41752341/G//A	synonymous	1	0
SMS	chrX	21997031/A//G	nonsynonymous	1	0
DMD	chrX	32360321/C//A	nonsynonymous	1	0
FAM133A	chrX	92965053/G//C	nonsynonymous	1	0
MORC4	chrX	106186060/G//T	nonsynonymous	1	0
MAGEC3	chrX	140983132/T//G	nonsynonymous	1	0
LCE1E	chr1	152759950/A//G	nonsynonymous	0	1
SCN3A	chr2	166003539/C//A	nonsynonymous	0	1
ZDBF2	chr2	207173138/G//A	nonsynonymous	0	1
PECR	chr2	216946472/G//C	synonymous	0	1
CATIP	chr2	219221813/T//C	synonymous	0	1
MINA	chr3	97673296/G//A	nonsynonymous	0	1
TF	chr3	133485261/C//T	synonymous	0	1
PRR23C	chr3	138763069/C//T	nonsynonymous	0	1
PCOLCE2	chr3	142537195/T//A	nonsynonymous	0	1
FAM193A	chr4	2695335/C//A	nonsynonymous	0	1
GRID2	chr4	94128609/G//A	nonsynonymous	0	1
PCDHAC2	chr5	140348518/A//G	nonsynonymous	0	1
PCDHB15	chr5	140627399/C//T	synonymous	0	1
GRM6	chr5	178413475/C//T	nonsynonymous	0	1
UTRN	chr6	144844161/G//T	nonsynonymous	0	1
CSMD1	chr8	2820916/G//A	synonymous	0	1
COL15A1	chr9	101706315/C//T	synonymous	0	1
ANK3	chr10	61835684/TTAA//T	nonsynonymous	0	1
LUZP2	chr11	24753667/T//A	nonsynonymous	0	1
NRXN3	chr14	79454485/G//A	nonsynonymous	0	1
AQR	chr15	35253019/T//C	synonymous	0	1
ZNF205	chr16	3170156/C//T	nonsynonymous	0	1
RNF40	chr16	30783423/G//A	nonsynonymous	0	1
ZNF799	chr19	12502412/G//A	nonsynonymous	0	1
ZNF93	chr19	20044889/C//A	synonymous	0	1
ZNF600	chr19	53270784/G//T	nonsynonymous	0	1
TMC4	chr19	54667551/G//A	synonymous	0	1
KRTAP19-4	chr21	31869296/C//T	nonsynonymous	0	1
RRP1B	chr21	45107760/C//T	nonsynonymous	0	1

GGT1	chr22	25007132/A//G	synonymous	0	1
NR1I3	chr1	161200707/G//A	synonymous	0	1
PATE4	chr11	125708198/C//A	synonymous	0	1
FOXO1	chr13	41133688/G//A	nonsynonymous	0	1
HERC2	chr15	28356991/T//G	nonsynonymous	0	1
FADS6	chr17	72889685/G//A	synonymous	0	1
TRIM28	chr19	59056419/G//A	nonsynonymous	0	1
MOB1A	chr2	74405956/C//G	synonymous	0	1
LOC102724428	chr21	44837551/G//A	synonymous	0	1
GRAMD4	chr22	47022753/C//T	synonymous	0	1
PCDHB8	chr5	140558317/C//G	synonymous	0	1
MMEL1	chr1	2541230/C//T	synonymous	0	1
AHDC1	chr1	27874481/C//T	synonymous	0	1
PHACTR4	chr1	28800071/C//T	synonymous	0	1
KHDRBS1	chr1	32508212/A//G	nonsynonymous	0	1
ROR1	chr1	64608187/A//C	nonsynonymous	0	1
HIPK1	chr1	114483921/C//T	nonsynonymous	0	1
IGSF3	chr1	117142835/G//A	nonsynonymous	0	1
ARHGEF2	chr1	155938954/G//A	synonymous	0	1
ETV3	chr1	157095674/C//T	synonymous	0	1
COPA	chr1	160305051/C//T	nonsynonymous	0	1
PAPPA2	chr1	176563716/C//T	nonsynonymous	0	1
PXDN	chr2	1670017/G//A	synonymous	0	1
SRBD1	chr2	45620101/C//T	nonsynonymous	0	1
AAK1	chr2	69732795/G//A	synonymous	0	1
ST6GAL2	chr2	107459657/C//T	synonymous	0	1
AMER3	chr2	131520745/T//C	nonsynonymous	0	1
FASTKD1	chr2	170428416/G//A	nonsynonymous	0	1
PDK1	chr2	173457728/G//C	nonsynonymous	0	1
TTN	chr2	179649066/C//T	nonsynonymous	0	1
CCDC36	chr3	49294248/G//A	nonsynonymous	0	1
SI	chr3	164777683/T//C	synonymous	0	1
ADGRL3	chr4	62598506/C//A	nonsynonymous	0	1
EPHA5	chr4	66230819/G//A	nonsynonymous	0	1
	chr4	71665917/C//A	synonymous	0	1
PITX2	chr4	111539468/G//A	nonsynonymous	0	1
ANK2	chr4	114199051/T//G	nonsynonymous	0	1
FBXL7	chr5	15928350/C//T	nonsynonymous	0	1
CD180	chr5	66479727/G//A	nonsynonymous	0	1
PCDHA13	chr5	140263638/C//T	synonymous	0	1
PCDHGB2	chr5	140741694/C//T	synonymous	0	1
HIST1H3I	chr6	27840069/G//A	nonsynonymous	0	1
ZNF311	chr6	28963337/C//T	nonsynonymous	0	1
MTCH1	chr6	36953902/G//T	synonymous	0	1
	chr6	56481129/G//A	synonymous	0	1
FBXL4	chr6	99365519/G//C	nonsynonymous	0	1
COL10A1	chr6	116442924/C//T	nonsynonymous	0	1
LPA	chr6	160978540/G//A	synonymous	0	1
AFDN	chr6	168315993/G//A	synonymous	0	1
ZNF736	chr7	63809448/G//A	nonsynonymous	0	1
TRRAP	chr7	98535281/A//T	nonsynonymous	0	1
GPC2	chr7	99773327/C//G	nonsynonymous	0	1
GIMAP1	chr7	150417172/C//T	nonsynonymous	0	1
EXTL3	chr8	28573891/C//T	synonymous	0	1
C9orf66	chr9	215209/G//A	nonsynonymous	0	1
CNTLN	chr9	17394657/TAC//T	nonsynonymous	0	1
TRPM3	chr9	73152239/G//A	nonsynonymous	0	1
	chr9	74477530/A//T	synonymous	0	1
SHC3	chr9	91657028/C//T	nonsynonymous	0	1
NOL8	chr9	95077787/G//A	nonsynonymous	0	1
COL27A1	chr9	116931433/G//C	nonsynonymous	0	1
KIF11	chr10	94381149/C//T	nonsynonymous	0	1
ATRNL1	chr10	116919910/C//T	synonymous	0	1
IFITM2	chr11	309199/C//G	synonymous	0	1
B4GALNT4	chr11	380410/G//A	nonsynonymous	0	1
OR4C16	chr11	55340288/G//T	nonsynonymous	0	1
PYGM	chr11	64514805/G//A	nonsynonymous	0	1
OR10G8	chr11	123900645/C//A	nonsynonymous	0	1
PLXNC1	chr12	94673392/C//A	nonsynonymous	0	1
LTA4H	chr12	96429326/C//T	synonymous	0	1

TMTC4	chr13	101264658/T//C	synonymous	0	1
AHNAK2	chr14	105418199/C//G	nonsynonymous	0	1
CTDSPL2	chr15	44776443/A//C	nonsynonymous	0	1
RASGRF1	chr15	79382616/G//A	synonymous	0	1
TNFRSF12A	chr16	3070438/G//A	nonsynonymous	0	1
SLC12A3	chr16	56918064/C//T	synonymous	0	1
CCDC113	chr16	58293780/G//A	nonsynonymous	0	1
PHLPP2	chr16	71689260/C//G	nonsynonymous	0	1
VTN	chr17	26694992/G//A	synonymous	0	1
CCL7	chr17	32598748/C//A	nonsynonymous	0	1
KRT33A	chr17	39504881/C//T	nonsynonymous	0	1
ATP6V0A1	chr17	40622210/C//A	nonsynonymous	0	1
KIF2B	chr17	51900267/G//A	synonymous	0	1
KIF2B	chr17	51900872/C//T	nonsynonymous	0	1
CA4	chr17	58235127/C//T	nonsynonymous	0	1
RHBDF2	chr17	74473778/G//A	synonymous	0	1
DSEL	chr18	65178219/C//T	synonymous	0	1
C19orf12	chr19	30193733/G//A	synonymous	0	1
LTBP4	chr19	41132965/C//T	synonymous	0	1
IGLON5	chr19	51830117/C//T	nonsynonymous	0	1
ZNF528	chr19	52919895/G//T	nonsynonymous	0	1
LAMA5	chr20	60893632/G//A	nonsynonymous	0	1
DGCR2	chr22	19026298/G//C	synonymous	0	1
SGSM1	chr22	25270520/C//T	nonsynonymous	0	1
SH3BGRL	chrX	80533877/C//T	synonymous	0	1
HTR2C	chrX	113965848/G//A	nonsynonymous	0	1
AADACL4	chr1	12711251/G//A	nonsynonymous	1	0
DDOST	chr1	20987697/G//T	nonsynonymous	1	0
HECTD3	chr1	45469408/C//T	nonsynonymous	1	0
KCND3	chr1	112525257/G//A	nonsynonymous	1	0
	chr1	155658228/G//A	synonymous	1	0
DHX9	chr1	182852461/A//G	synonymous	1	0
USH2A	chr1	216260092/C//T	synonymous	1	0
MSH6	chr2	48026269/A//C	synonymous	1	0
CLK1	chr2	201726566/G//A	nonsynonymous	1	0
RAPH1	chr2	204354447/C//T	nonsynonymous	1	0
MDH1B	chr2	207604353/T//A	nonsynonymous	1	0
	chr2	238647952/G//A	synonymous	1	0
STAC	chr3	36570364/T//G	nonsynonymous	1	0
MCM2	chr3	127338106/G//C	synonymous	1	0
AHSG	chr3	186335024/C//A	nonsynonymous	1	0
MASP1	chr3	186937921/C//G	nonsynonymous	1	0
CCDC50	chr3	191087779/C//T	synonymous	1	0
LYAR	chr4	4276203/G//T	synonymous	1	0
ADH1A	chr4	100203628/C//T	nonsynonymous	1	0
MCUB	chr4	110606494/TACA//T	nonsynonymous	1	0
ANK2	chr4	114153375/A//G	nonsynonymous	1	0
LARP1B	chr4	129131169/A//T	nonsynonymous	1	0
FBXW7	chr4	153332826/G//A	nonsynonymous	1	0
APC	chr5	112175095/AAT//A	nonsynonymous	1	0
SLC4A9	chr5	139743402/C//T	nonsynonymous	1	0
PCDHB1	chr5	140431468/C//T	nonsynonymous	1	0
STK32A	chr5	146730662/G//A	synonymous	1	0
FAT2	chr5	150922622/G//A	nonsynonymous	1	0
ZNF391	chr6	27368827/T//C	synonymous	1	0
MCHR2	chr6	100395746/C//A	nonsynonymous	1	0
SEPT7	chr7	35923586/G//C	nonsynonymous	1	0
HGF	chr7	81399245/C//T	nonsynonymous	1	0
EZH2	chr7	148504759/T//A	nonsynonymous	1	0
ZNF398	chr7	148876317/C//T	synonymous	1	0
GSDMC	chr8	130789650/G//A	nonsynonymous	1	0
IFNW1	chr9	21141555/G//A	synonymous	1	0
KIF27	chr9	86530211/A//G	nonsynonymous	1	0
AFAP1L2	chr10	116055783/T//G	synonymous	1	0
DPYSL4	chr10	134014433/C//T	synonymous	1	0
PAX6	chr11	31815600/G//C	nonsynonymous	1	0
OR4C11	chr11	55371464/C//T	nonsynonymous	1	0
FGF4	chr11	69588116/C//T	synonymous	1	0
TENM4	chr11	78440584/G//C	nonsynonymous	1	0
STT3A	chr11	125478028/G//T	nonsynonymous	1	0

CHD4	chr12	6711326/G//A	nonsynonymous	1	0
TMEM132B	chr12	126138519/C//T	nonsynonymous	1	0
SACS	chr13	23908639/G//C	nonsynonymous	1	0
PARP4	chr13	25058885/C//A	nonsynonymous	1	0
DCLK1	chr13	36428695/G//A	nonsynonymous	1	0
MYO16	chr13	109318373/C//T	synonymous	1	0
NDRG2	chr14	21486174/G//C	nonsynonymous	1	0
ANPEP	chr15	90347621/C//A	nonsynonymous	1	0
USP31	chr16	23080221/T//A	nonsynonymous	1	0
PALB2	chr16	23619229/G//A	synonymous	1	0
IL21R	chr16	27455948/T//G	nonsynonymous	1	0
MYH8	chr17	10318675/G//A	synonymous	1	0
FZD2	chr17	42635766/C//G	nonsynonymous	1	0
NOG	chr17	54672083/C//T	nonsynonymous	1	0
MPO	chr17	56355502/G//A	nonsynonymous	1	0
TANC2	chr17	61473124/C//T	synonymous	1	0
CASKIN2	chr17	73500919/C//A	nonsynonymous	1	0
DNAH17	chr17	76486909/G//A	synonymous	1	0
GAA	chr17	78086718/C//T	synonymous	1	0
EPB41L3	chr18	5419724/G//A	nonsynonymous	1	0
DNM2	chr19	10887882/G//A	synonymous	1	0
ZNF709	chr19	12575517/A//T	nonsynonymous	1	0
PRKACA	chr19	14208621/G//A	synonymous	1	0
GATAD2A	chr19	19609311/C//A	synonymous	1	0
ZNF208	chr19	22155691/A//G	synonymous	1	0
NUDT19	chr19	33200188/CCTCT//C	nonsynonymous	1	0
FBXO17	chr19	39435697/C//T	nonsynonymous	1	0
SUPT5H	chr19	39965358/G//A	synonymous	1	0
CEACAM21	chr19	42085788/T//A	nonsynonymous	1	0
SRRM5	chr19	44116502/C//T	nonsynonymous	1	0
EPS8L1	chr19	55592678/G//T	nonsynonymous	1	0
KIAA1755	chr20	36874362/C//T	nonsynonymous	1	0
RBPJL	chr20	43940894/G//C	nonsynonymous	1	0
COL9A3	chr20	61468445/A//G	synonymous	1	0
ELFN2	chr22	37770993/G//A	synonymous	1	0
NHS	chrX	17745463/T//C	synonymous	1	0
KIAA2022	chrX	73959338/A//G	synonymous	1	0
TENM1	chrX	123518560/T//G	nonsynonymous	1	0
DCAF12L2	chrX	125298944/C//T	nonsynonymous	1	0
CAMSAP2	chr1	200822405/G//A	nonsynonymous	0	1
PSD4	chr2	113953346/G//A	nonsynonymous	0	1
MAML3	chr4	140646965/G//T	synonymous	0	1
ERBIN	chr5	65349668/G//A	nonsynonymous	0	1
	chr6	43399821/G//A	synonymous	0	1
VENTX	chr10	135053603/C//T	synonymous	0	1
LRRC4C	chr11	40136424/A//G	synonymous	0	1
GLT1D1	chr12	129442144/G//A	nonsynonymous	0	1
TBL3	chr16	2026949/G//A	nonsynonymous	0	1
ZFR2	chr19	3827588/C//T	nonsynonymous	0	1
CASS4	chr20	55033453/C//T	nonsynonymous	0	1
PBDC1	chrX	75392990/G//T	nonsynonymous	0	1
UEVLD	chr11	18568435/G//A	nonsynonymous	1	0
NCOR1	chr17	15960927/G//A	nonsynonymous	1	0
BCL9	chr1	147091043/G//T	nonsynonymous	0	1
GPR161	chr1	168065851/G//A	nonsynonymous	0	1
EFCAB2	chr1	245246963/G//C	nonsynonymous	0	1
XCR1	chr3	46063022/C//T	nonsynonymous	0	1
ZNF717	chr3	75787194/C//T	nonsynonymous	0	1
ITGB5	chr3	124567148/T//C	synonymous	0	1
TRIM42	chr3	140419756/G//C	nonsynonymous	0	1
APOD	chr3	195295950/C//A	nonsynonymous	0	1
APC	chr5	112174833/T//G	nonsynonymous	0	1
	chr7	34917833/G//T	synonymous	0	1
CLDN4	chr7	73245608/C//T	nonsynonymous	0	1
CHID1	chr11	902274/G//A	synonymous	0	1
SLC43A1	chr11	57252562/G//A	synonymous	0	1
NBEA	chr13	36229880/A//G	nonsynonymous	0	1
FREM2	chr13	39446912/C//T	nonsynonymous	0	1
RNASE11	chr14	21052358/A//C	synonymous	0	1
ATG2B	chr14	96798938/G//C	nonsynonymous	0	1

TDRD9	chr14	104491992/C//T	nonsynonymous	0	1
ZNF594	chr17	5086092/G//T	nonsynonymous	0	1
TP53	chr17	7578475/G//A	nonsynonymous	0	1
PIPOX	chr17	27381673/C//T	synonymous	0	1
ESCO1	chr18	19154044/G//A	nonsynonymous	0	1
ZNF20	chr19	12243769/C//T	nonsynonymous	0	1
RSPH6A	chr19	46308136/G//A	nonsynonymous	0	1
CCDC61	chr19	46520028/C//G	nonsynonymous	0	1
YWHAB	chr20	43532699/A//C	nonsynonymous	0	1
ZBP1	chr20	56188364/C//G	nonsynonymous	0	1
SLC25A17	chr22	41195070/C//A	nonsynonymous	0	1
TBL1X	chrX	9679803/T//C	nonsynonymous	0	1
OTUD6A	chrX	69283143/G//C	nonsynonymous	0	1
PLXNB3	chrX	153033043/G//A	nonsynonymous	0	1
AGO4	chr1	36297040/G//A	synonymous	0	1
BTF3L4	chr1	52530527/C//T	nonsynonymous	0	1
OR2T1	chr1	248569520/T//A	synonymous	0	1
SCN3A	chr2	166032716/A//G	synonymous	0	1
GBX2	chr2	237076454/A//G	nonsynonymous	0	1
PCDH7	chr4	30724207/G//A	nonsynonymous	0	1
SH3D19	chr4	152053536/C//A	nonsynonymous	0	1
CD180	chr5	66479864/C//T	synonymous	0	1
VCAN	chr5	82816946/C//A	nonsynonymous	0	1
FAM81B	chr5	94784045/C//G	nonsynonymous	0	1
MCC	chr5	112437487/A//T	synonymous	0	1
TRIM31	chr6	30071922/A//C	nonsynonymous	0	1
C6orf226	chr6	42858380/G//A	synonymous	0	1
COL12A1	chr6	75865571/C//T	nonsynonymous	0	1
MMRN2	chr10	88702351/A//C	synonymous	0	1
DHRS4L2	chr14	24458201/G//C	nonsynonymous	0	1
TIGD7	chr16	3350459/A//T	synonymous	0	1
SLC6A2	chr16	55729208/C//T	synonymous	0	1
RYR1	chr19	38942419/G//C	nonsynonymous	0	1
NUDT10	chrX	51075841/A//G	synonymous	0	1
GPR50	chrX	150348483/G//A	nonsynonymous	0	1
PADI6	chr1	17720860/T//C	nonsynonymous	1	0
NKAIN1	chr1	31656799/C//A	synonymous	1	0
STK40	chr1	36819977/A//T	nonsynonymous	1	0
GBP3	chr1	89476668/T//C	synonymous	1	0
ABCD3	chr1	94955523/C//T	synonymous	1	0
	chr1	100950069/T//A	synonymous	1	0
FLG	chr1	152280094/T//A	nonsynonymous	1	0
BCAN	chr1	156627487/T//C	nonsynonymous	1	0
DUSP23	chr1	159751082/C//T	synonymous	1	0
PIGM	chr1	160001056/G//C	synonymous	1	0
MAEL	chr1	166974631/C//A	nonsynonymous	1	0
XCL1	chr1	168549415/T//A	nonsynonymous	1	0
CFH	chr1	196648809/A//G	nonsynonymous	1	0
OSR1	chr2	19552915/G//C	nonsynonymous	1	0
PLB1	chr2	28828759/A//G	nonsynonymous	1	0
VIT	chr2	36994354/G//T	nonsynonymous	1	0
CTNNA2	chr2	80085237/G//A	nonsynonymous	1	0
MARCO	chr2	119731958/G//A	synonymous	1	0
HECW2	chr2	197087019/A//C	nonsynonymous	1	0
	chr2	202027946/A//G	synonymous	1	0
CXCR2	chr2	219000428/C//A	nonsynonymous	1	0
DNER	chr2	230312058/G//A	nonsynonymous	1	0
ING5	chr2	242651426/G//A	nonsynonymous	1	0
KAT2B	chr3	20178532/C//CTTT	nonsynonymous	1	0
PLCD1	chr3	38052782/T//C	nonsynonymous	1	0
TMIE	chr3	46742983/G//T	synonymous	1	0
PLXND1	chr3	129284220/G//T	nonsynonymous	1	0
GMPS	chr3	155652818/A//C	nonsynonymous	1	0
FNDC3B	chr3	171969142/G//A	nonsynonymous	1	0
SOD3	chr4	24801260/C//T	synonymous	1	0
KLHL5	chr4	39064521/A//T	synonymous	1	0
FIP1L1	chr4	54245266/G//C	nonsynonymous	1	0
ALB	chr4	74282003/C//T	nonsynonymous	1	0
ANXA3	chr4	79525498/A//G	nonsynonymous	1	0
WDFY3	chr4	85609382/T//C	nonsynonymous	1	0

FBXW7	chr4	153244219/T//C	synonymous	1	0
MUC22	chr6	30993985/G//C	synonymous	1	0
SPDEF	chr6	34511800/C//T	nonsynonymous	1	0
PKHD1	chr6	51747908/C//G	nonsynonymous	1	0
POPDC3	chr6	105607581/C//T	synonymous	1	0
SASH1	chr6	148865843/G//T	synonymous	1	0
LMTK2	chr7	97823423/G//T	nonsynonymous	1	0
TRIM24	chr7	138269649/C//T	nonsynonymous	1	0
PARP12	chr7	139726085/G//A	synonymous	1	0
OR9A4	chr7	141619594/C//T	nonsynonymous	1	0
KCNU1	chr8	36675170/T//A	nonsynonymous	1	0
ZFPM2	chr8	106814002/A//G	synonymous	1	0
COL22A1	chr8	139833607/A//T	synonymous	1	0
SLC39A4	chr8	145640240/A//T	nonsynonymous	1	0
FNBP1	chr9	132652692/C//G	synonymous	1	0
NOTCH1	chr9	139400097/G//A	synonymous	1	0
PHYHIPL	chr10	60994215/C//A	nonsynonymous	1	0
MYOF	chr10	95069887/A//C	nonsynonymous	1	0
DKK3	chr11	11990022/C//G	nonsynonymous	1	0
IGSF22	chr11	18738367/G//A	nonsynonymous	1	0
ANO3	chr11	26353806/G//T	synonymous	1	0
TRAF6	chr11	36522977/A//G	nonsynonymous	1	0
MS4A4A	chr11	60064720/G//T	nonsynonymous	1	0
MAML2	chr11	95712793/A//G	synonymous	1	0
APOA1-AS	chr11	116706953/C//T	synonymous	1	0
SC5D	chr11	121177145/A//G	nonsynonymous	1	0
EPS8	chr12	15822733/C//A	synonymous	1	0
KRT76	chr12	53164871/G//T	nonsynonymous	1	0
MMP19	chr12	56233528/G//A	synonymous	1	0
TSC22D1	chr13	45147536/A//T	nonsynonymous	1	0
MYCBP2	chr13	77817263/G//T	nonsynonymous	1	0
OR4K13	chr14	20502600/G//A	synonymous	1	0
VRK1	chr14	97322495/A//G	nonsynonymous	1	0
DIO3	chr14	102028060/G//T	nonsynonymous	1	0
ATP10A	chr15	25932898/G//A	synonymous	1	0
GJD2	chr15	35044867/C//A	nonsynonymous	1	0
ITGA11	chr15	68695322/G//T	synonymous	1	0
PRMT7	chr16	68380124/C//G	nonsynonymous	1	0
SERPINF1	chr17	1680813/G//T	synonymous	1	0
MYH2	chr17	10432652/C//A	nonsynonymous	1	0
TANC2	chr17	61345101/C//A	synonymous	1	0
AQP4	chr18	24442424/T//G	nonsynonymous	1	0
TSHZ1	chr18	72999886/G//T	nonsynonymous	1	0
EEF2	chr19	3977959/T//C	nonsynonymous	1	0
PTPRS	chr19	5245831/G//A	nonsynonymous	1	0
CAMSAP3	chr19	7676670/A//G	nonsynonymous	1	0
	chr19	7831118/C//G	synonymous	1	0
ADGRL1	chr19	14288480/C//A	synonymous	1	0
GDF15	chr19	18499616/C//T	synonymous	1	0
ZNF429	chr19	21720038/G//A	nonsynonymous	1	0
SIRT2	chr19	39384156/G//A	nonsynonymous	1	0
RPS16	chr19	39924385/A//C	nonsynonymous	1	0
OPA3	chr19	46056968/T//A	nonsynonymous	1	0
CALM3	chr19	47112409/G//C	nonsynonymous	1	0
ZNF415	chr19	53612963/G//A	nonsynonymous	1	0
NLRP8	chr19	56466692/C//A	nonsynonymous	1	0
MTG2	chr20	60770908/A//T	synonymous	1	0
ADAMTS1	chr21	28212733/C//T	synonymous	1	0
TIAM1	chr21	32624382/G//A	nonsynonymous	1	0
DSCAM	chr21	41719740/G//T	nonsynonymous	1	0
SF3A1	chr22	30735013/T//C	nonsynonymous	1	0
RASD2	chr22	35948196/ACC//A	synonymous	1	0
HUWE1	chrX	53588796/A//G	synonymous	1	0
KCNJ9	chr1	160053797/A//C	synonymous	0	1
TXNDC9	chr2	99938574/T//C	nonsynonymous	0	1
FAIM	chr3	138341028/A//G	nonsynonymous	0	1
LRIT3	chr4	110791778/A//G	nonsynonymous	0	1
ADAMTS16	chr5	5303867/C//T	synonymous	0	1
TRIM41	chr5	180651221/G//A	synonymous	0	1
SCIN	chr7	12680135/T//A	nonsynonymous	0	1

ZNF273	chr7	64388845/G//A	nonsynonymous	0	1
ZNF804B	chr7	88847471/T//G	nonsynonymous	0	1
RAD21	chr8	117869521/C//A	nonsynonymous	0	1
SLA	chr8	134114789/C//A	synonymous	0	1
TOR4A	chr9	140173611/T//C	nonsynonymous	0	1
ADRB1	chr10	115804459/T//C	nonsynonymous	0	1
CCDC86	chr11	60609552/C//A	synonymous	0	1
RAB39A	chr11	107832683/G//A	nonsynonymous	0	1
CACNA1C	chr12	2783786/C//G	nonsynonymous	0	1
TM9SF2	chr13	100214940/T//G	synonymous	0	1
CYFIP1	chr15	22999526/C//T	nonsynonymous	0	1
ASPHD1	chr16	29912823/A//G	synonymous	0	1
MT4	chr16	56601692/A//G	nonsynonymous	0	1
FANCA	chr16	89857913/G//A	synonymous	0	1
CENPV	chr17	16253298/T//C	synonymous	0	1
KRTAP1-3	chr17	39190640/C//A	nonsynonymous	0	1
KRTAP4-11	chr17	39274364/T//G	nonsynonymous	0	1
USHBP1	chr19	17370450/C//T	nonsynonymous	0	1
PPP2R1A	chr19	52716213/G//A	synonymous	0	1
CST8	chr20	23473648/C//A	nonsynonymous	0	1
BRWD1	chr21	40641853/C//T	nonsynonymous	0	1
TTC28	chr22	28378314/G//A	synonymous	0	1
IRS4	chrX	107977199/G//T	nonsynonymous	0	1
ACVR2A	chr2	148672879/G//A	synonymous	0	1
JMY	chr5	78610494/C//T	nonsynonymous	0	1
	chr5	148897408/T//G	synonymous	0	1
SYNCRIP	chr6	86325067/T//G	nonsynonymous	0	1
ELN	chr7	73466304/G//A	nonsynonymous	0	1
CPQ	chr8	98155433/T//C	synonymous	0	1
COL27A1	chr9	117052349/A//G	synonymous	0	1
COL13A1	chr10	71640267/C//G	synonymous	0	1
DYNC2H1	chr11	103049827/A//G	nonsynonymous	0	1
DYNC2H1	chr11	103049842/T//C	nonsynonymous	0	1
GLT1D1	chr12	129383793/T//G	synonymous	0	1
CTDSPL2	chr15	44783186/C//T	nonsynonymous	0	1
SLC12A4	chr16	67979865/C//G	nonsynonymous	0	1
ALDH3A1	chr17	19646652/T//G	nonsynonymous	0	1
C18orf8	chr18	21111671/T//G	synonymous	0	1
PAXBP1	chr21	34110601/G//A	synonymous	0	1
CABIN1	chr22	24515573/G//A	nonsynonymous	0	1
RBM10	chrX	47038751/TG//T	nonsynonymous	0	1
TENM1	chrX	124029835/T//C	nonsynonymous	0	1
ALDH9A1	chr1	165664638/T//C	synonymous	1	0
CFHR1	chr1	196797356/C//A	nonsynonymous	1	0
ZP4	chr1	238048784/G//C	nonsynonymous	1	0
STARD7	chr2	96853022/T//G	nonsynonymous	1	0
TTN	chr2	179604692/T//G	nonsynonymous	1	0
TRIP12	chr2	230683204/T//A	nonsynonymous	1	0
TRANK1	chr3	36873129/G//A	nonsynonymous	1	0
ABHD14A	chr3	52015014/T//C	nonsynonymous	1	0
WNT5A	chr3	55508594/C//T	nonsynonymous	1	0
APC	chr5	112175266/AGT//A	nonsynonymous	1	0
PCDHGB6	chr5	140789709/G//A	nonsynonymous	1	0
HIST1H2BJ	chr6	27100381/T//A	nonsynonymous	1	0
MOG	chr6	29638498/C//T	nonsynonymous	1	0
TUBB	chr6	30691933/T//C	nonsynonymous	1	0
GPR63	chr6	97246998/C//T	nonsynonymous	1	0
TIAM2	chr6	155485673/G//A	nonsynonymous	1	0
T	chr6	166580128/G//A	synonymous	1	0
SLC20A2	chr8	42287753/G//A	nonsynonymous	1	0
FRMPD1	chr9	37737218/G//A	synonymous	1	0
CIZ1	chr9	130932334/C//T	nonsynonymous	1	0
EHMT1	chr9	140605438/AG//A	nonsynonymous	1	0
EPS8L2	chr11	724785/C//T	nonsynonymous	1	0
DCDC1	chr11	31327282/C//T	nonsynonymous	1	0
CAPN1	chr11	64951014/C//T	nonsynonymous	1	0
NLRX1	chr11	119050423/C//T	nonsynonymous	1	0
ITPR2	chr12	26731707/G//A	synonymous	1	0
OR7D2	chr19	9297229/G//A	nonsynonymous	1	0
LDLR	chr19	11227644/G//T	synonymous	1	0

ZNF709	chr19	12575060/G//A	nonsynonymous	1	0
PSG3	chr19	43228185/G//T	synonymous	1	0
MYPOP	chr19	46393982/G//C	nonsynonymous	1	0
RFPL4A	chr19	56273285/A//G	nonsynonymous	1	0
CHMP4B	chr20	32399313/T//C	synonymous	1	0
MICALL1	chr22	38327935/C//T	nonsynonymous	1	0
NLGN4X	chrX	5811586/T//C	nonsynonymous	1	0
DMD	chrX	31140005/C//T	synonymous	1	0
PASD1	chrX	150817153/T//C	synonymous	1	0
TNFRSF14	chr1	2492099/C//T	nonsynonymous	0	1
FAM229A	chr1	32826930/T//C	synonymous	0	1
MKNK1	chr1	47048932/C//T	nonsynonymous	0	1
ZFYVE9	chr1	52704972/C//A	nonsynonymous	0	1
ARHGAP29	chr1	94639604/C//T	nonsynonymous	0	1
KCND3	chr1	112524533/G//A	synonymous	0	1
ATP1A1	chr1	116932947/G//A	nonsynonymous	0	1
HORMAD1	chr1	150672638/T//A	nonsynonymous	0	1
TCHH	chr1	152081608/A//C	nonsynonymous	0	1
ATP8B2	chr1	154316887/C//T	synonymous	0	1
SMG7	chr1	183520021/G//C	nonsynonymous	0	1
RGL1	chr1	183876153/C//G	nonsynonymous	0	1
KMO	chr1	241724031/A//C	nonsynonymous	0	1
IL1R1	chr2	102792849/G//A	nonsynonymous	0	1
ST6GAL2	chr2	107459634/G//A	nonsynonymous	0	1
ZEB2	chr2	145157570/A//G	nonsynonymous	0	1
ATF2	chr2	175986250/G//T	nonsynonymous	0	1
UBE2E3	chr2	181927645/G//A	synonymous	0	1
TM4SF20	chr2	228228351/T//G	synonymous	0	1
SCN10A	chr3	38760228/G//A	synonymous	0	1
TRAK1	chr3	42265286/C//T	synonymous	0	1
FRG2C	chr3	75714707/A//G	nonsynonymous	0	1
ROBO1	chr3	78688903/T//C	nonsynonymous	0	1
OR5K4	chr3	98073186/A//G	synonymous	0	1
MORC1	chr3	108751608/G//A	synonymous	0	1
KY	chr3	134329177/G//A	synonymous	0	1
JAKMIP1	chr4	6082016/C//T	synonymous	0	1
LGI2	chr4	25032164/C//T	nonsynonymous	0	1
ADGRL3	chr4	62599246/A//G	nonsynonymous	0	1
WDFY3	chr4	85657456/C//T	nonsynonymous	0	1
ADH4	chr4	100047803/G//A	nonsynonymous	0	1
PAPSS1	chr4	108622350/C//T	nonsynonymous	0	1
ABCE1	chr4	146044699/G//A	synonymous	0	1
LOC100130744	chr5	14712970/G//A	synonymous	0	1
TENM2	chr5	167674683/C//T	nonsynonymous	0	1
GPX5	chr6	28501748/C//T	nonsynonymous	0	1
SYNGAP1	chr6	33410682/C//T	nonsynonymous	0	1
COL12A1	chr6	75841698/G//A	synonymous	0	1
SNAP91	chr6	84265899/C//T	synonymous	0	1
CEP162	chr6	84879151/C//T	nonsynonymous	0	1
INTS1	chr7	1538761/G//A	synonymous	0	1
DNAH11	chr7	21600793/G//C	synonymous	0	1
PDE1C	chr7	31904610/G//A	synonymous	0	1
PSMA2	chr7	42964371/G//C	nonsynonymous	0	1
ZNF716	chr7	57529806/A//G	synonymous	0	1
TMEM120A	chr7	75616443/A//T	synonymous	0	1
SMO	chr7	128850838/G//C	nonsynonymous	0	1
XKR4	chr8	56436305/C//T	nonsynonymous	0	1
ZFHX4	chr8	77768162/C//T	nonsynonymous	0	1
CPSF1	chr8	145626174/C//T	nonsynonymous	0	1
FOCAD	chr9	20885192/G//A	nonsynonymous	0	1
PALM2-AKAP2	chr9	112900761/C//A	nonsynonymous	0	1
STOM	chr9	124115534/G//T	nonsynonymous	0	1
RPL35	chr9	127623798/T//C	nonsynonymous	0	1
SETX	chr9	135202959/A//G	synonymous	0	1
RALGDS	chr9	135977392/G//C	nonsynonymous	0	1
LARP4B	chr10	910123/C//G	nonsynonymous	0	1
ANKRD30A	chr10	37430724/A//G	nonsynonymous	0	1
CH17-360D5.1	chr10	47087611/G//A	synonymous	0	1
ZMIZ1	chr10	81060645/C//T	synonymous	0	1
FRA10AC1	chr10	95443831/T//G	nonsynonymous	0	1

GPAM	chr10	113928224/C//T	nonsynonymous	0	1
ZRANB1	chr10	126631316/C//G	nonsynonymous	0	1
	chr10	126716179/C//A	synonymous	0	1
PIDD1	chr11	802350/C//T	nonsynonymous	0	1
SBF2	chr11	9810655/C//G	nonsynonymous	0	1
TSPAN9	chr12	3390479/C//T	nonsynonymous	0	1
SLCO1B7	chr12	21174450/T//G	nonsynonymous	0	1
PDZRN4	chr12	41966268/C//T	nonsynonymous	0	1
SLC4A8	chr12	51856203/C//G	nonsynonymous	0	1
BEST3	chr12	70049581/G//A	synonymous	0	1
ZFC3H1	chr12	72025951/G//C	nonsynonymous	0	1
UTP20	chr12	101750706/T//G	nonsynonymous	0	1
POSTN	chr13	38162117/G//A	nonsynonymous	0	1
LRFN5	chr14	42357120/C//T	nonsynonymous	0	1
ESR2	chr14	64727364/G//A	nonsynonymous	0	1
BTBD6	chr14	105716419/CTAGAGGA//C	nonsynonymous	0	1
BTBD6	chr14	105716429/TTGCCAAC//T	nonsynonymous	0	1
OR4N4	chr15	22383341/G//A	nonsynonymous	0	1
RYR3	chr15	34094075/C//G	nonsynonymous	0	1
UNC13C	chr15	54786890/G//A	nonsynonymous	0	1
NMB	chr15	85200529/C//T	nonsynonymous	0	1
MYH11	chr16	15851692/C//T	nonsynonymous	0	1
ERN2	chr16	23724460/G//A	nonsynonymous	0	1
SEZ6L2	chr16	29891281/G//A	nonsynonymous	0	1
ADAMTS18	chr16	77359805/G//A	nonsynonymous	0	1
PRPF8	chr17	1580469/G//C	synonymous	0	1
ABCA9	chr17	66980313/C//T	synonymous	0	1
SLC39A11	chr17	70644965/G//A	synonymous	0	1
MTCL1	chr18	8784582/C//T	nonsynonymous	0	1
MUC16	chr19	9074748/T//C	nonsynonymous	0	1
CYP4F8	chr19	15734038/C//T	synonymous	0	1
HAPLN4	chr19	19368878/G//A	synonymous	0	1
ZNF208	chr19	22156279/A//G	synonymous	0	1
NUCB1	chr19	49409095/G//A	nonsynonymous	0	1
SIGLEC7	chr19	51645694/G//A	nonsynonymous	0	1
KIR3DL1	chr19	55333096/T//C	synonymous	0	1
	chr19	57742423/C//T	synonymous	0	1
CENPB	chr20	3765547/G//A	synonymous	0	1
CST8	chr20	23473638/G//A	nonsynonymous	0	1
OGFR	chr20	61440970/C//T	nonsynonymous	0	1
KCNQ2	chr20	62046388/G//A	nonsynonymous	0	1
KRTAP7-1	chr21	32201924/G//C	nonsynonymous	0	1
URB1	chr21	33735517/G//A	nonsynonymous	0	1
UBASH3A	chr21	43833258/C//T	synonymous	0	1
COL6A1	chr21	47406935/C//T	synonymous	0	1
COL6A1	chr21	47421199/G//A	nonsynonymous	0	1
RGL4	chr22	24034602/G//C	nonsynonymous	0	1
APOBEC3F	chr22	39448694/C//T	synonymous	0	1
WNK3	chrX	54360023/C//A	nonsynonymous	0	1
FGD1	chrX	54475683/G//A	nonsynonymous	0	1
MAMLD1	chrX	149638791/G//T	nonsynonymous	0	1
CCDC27	chr1	3669010/A//C	synonymous	1	0
ZBTB48	chr1	6640743/A//T	nonsynonymous	1	0
KIF17	chr1	21036260/C//A	nonsynonymous	1	0
SLC9A1	chr1	27428988/C//T	nonsynonymous	1	0
EPB41	chr1	29314409/G//C	nonsynonymous	1	0
EPHA10	chr1	38227621/G//T	nonsynonymous	1	0
EXO5	chr1	40980323/A//C	nonsynonymous	1	0
KIF2C	chr1	45213331/A//T	nonsynonymous	1	0
GLIS1	chr1	54060606/G//A	synonymous	1	0
C8A	chr1	57383351/T//C	nonsynonymous	1	0
CLCA2	chr1	86894233/G//C	nonsynonymous	1	0
STXBP3	chr1	109336276/A//G	synonymous	1	0
DCST1	chr1	155013975/A//T	nonsynonymous	1	0
OR10K1	chr1	158435420/G//T	synonymous	1	0
SLAMF8	chr1	159799930/G//T	nonsynonymous	1	0
SLAMF1	chr1	160607098/G//T	nonsynonymous	1	0
TNR	chr1	175375559/G//A	synonymous	1	0
RFWD2	chr1	176085819/T//A	nonsynonymous	1	0
RGSL1	chr1	182517879/C//G	nonsynonymous	1	0

HMCN1	chr1	186072734/C//A	synonymous	1	0
TMEM9	chr1	201104861/T//G	nonsynonymous	1	0
PPFIA4	chr1	203033055/G//T	nonsynonymous	1	0
HHIPL2	chr1	222705401/C//A	nonsynonymous	1	0
OBSCN	chr1	228401914/G//T	nonsynonymous	1	0
OBSCN	chr1	228506637/C//T	synonymous	1	0
RYR2	chr1	237765331/C//A	nonsynonymous	1	0
ZP4	chr1	238050862/C//T	nonsynonymous	1	0
OR2T12	chr1	248458084/G//C	nonsynonymous	1	0
OR2T6	chr1	248551360/G//T	nonsynonymous	1	0
OR2G6	chr1	248685323/G//T	nonsynonymous	1	0
NBAS	chr2	15555788/T//C	nonsynonymous	1	0
PFN4	chr2	24345326/T//C	nonsynonymous	1	0
ITSN2	chr2	24533419/T//C	synonymous	1	0
DNMT3A	chr2	25463523/C//A	nonsynonymous	1	0
DNMT3A	chr2	25466857/C//G	synonymous	1	0
C2orf73	chr2	54570921/G//A	nonsynonymous	1	0
EML6	chr2	55077234/T//C	synonymous	1	0
EML6	chr2	55155882/A//C	nonsynonymous	1	0
REG1B	chr2	79313551/A//C	nonsynonymous	1	0
AFF3	chr2	100209956/C//A	nonsynonymous	1	0
CFAP221	chr2	120413990/G//T	nonsynonymous	1	0
GLI2	chr2	121747822/G//A	nonsynonymous	1	0
CNTNAP5	chr2	125521556/C//A	nonsynonymous	1	0
CNTNAP5	chr2	125671823/G//T	nonsynonymous	1	0
KYNU	chr2	143712411/G//T	nonsynonymous	1	0
RIF1	chr2	152321666/G//C	nonsynonymous	1	0
NEB	chr2	152534686/C//A	nonsynonymous	1	0
CCDC173	chr2	170507014/C//G	nonsynonymous	1	0
TTN	chr2	179485462/G//A	nonsynonymous	1	0
HECW2	chr2	197298155/G//C	synonymous	1	0
FN1	chr2	216286816/C//T	nonsynonymous	1	0
DES	chr2	220290413/G//T	nonsynonymous	1	0
SPHKAP	chr2	228860413/G//T	synonymous	1	0
SP100	chr2	231311547/A//T	nonsynonymous	1	0
KLHL30	chr2	239057695/T//C	nonsynonymous	1	0
GRM7	chr3	6903538/G//C	nonsynonymous	1	0
MAPKAPK3	chr3	50681855/T//C	nonsynonymous	1	0
TRMT10C	chr3	101284616/C//A	nonsynonymous	1	0
TIGIT	chr3	114012958/C//T	nonsynonymous	1	0
SEMA5B	chr3	122647469/C//A	synonymous	1	0
KALRN	chr3	124418737/C//T	nonsynonymous	1	0
CCNL1	chr3	156867178/C//T	synonymous	1	0
IFT80	chr3	160095215/T//C	synonymous	1	0
MECOM	chr3	168840416/C//G	nonsynonymous	1	0
NCEH1	chr3	172353761/A//G	nonsynonymous	1	0
NCEH1	chr3	172353767/C//G	nonsynonymous	1	0
ABCC5	chr3	183667822/C//A	synonymous	1	0
DGKG	chr3	185969560/G//T	nonsynonymous	1	0
ZNF141	chr4	366896/G//C	nonsynonymous	1	0
NKX3-2	chr4	13544069/CG//C	nonsynonymous	1	0
GBA3	chr4	22749488/C//T	nonsynonymous	1	0
BEND4	chr4	42119656/C//T	nonsynonymous	1	0
GABRB1	chr4	47163420/A//G	nonsynonymous	1	0
PCDH10	chr4	134111320/T//A	synonymous	1	0
RAPGEF2	chr4	160263098/A//G	nonsynonymous	1	0
	chr4	186559228/C//A	synonymous	1	0
ADAMTS16	chr5	5146343/C//A	synonymous	1	0
CDH9	chr5	26889971/C//T	nonsynonymous	1	0
C9	chr5	39306822/A//T	nonsynonymous	1	0
NNT	chr5	43704430/C//T	nonsynonymous	1	0
GPBP1	chr5	56527134/G//A	nonsynonymous	1	0
AP3B1	chr5	77396788/T//C	nonsynonymous	1	0
PCDHA6	chr5	140209902/C//T	synonymous	1	0
PCDHB12	chr5	140590526/G//T	nonsynonymous	1	0
PCDHB15	chr5	140626806/G//C	nonsynonymous	1	0
GRIA1	chr5	153078550/G//C	nonsynonymous	1	0
FLT4	chr5	180056404/G//T	synonymous	1	0
FOXQ1	chr6	1312836/G//A	synonymous	1	0
POM121L2	chr6	27278964/T//A	nonsynonymous	1	0

TRIM39-RPP21	chr6	30314265/C//T	nonsynonymous	1	0
HLA-B	chr6	31324586/C//T	synonymous	1	0
TBC1D22B	chr6	37259112/G//A	nonsynonymous	1	0
PRPH2	chr6	42689487/C//T	synonymous	1	0
DEFB112	chr6	50011494/T//A	nonsynonymous	1	0
COL21A1	chr6	56035861/C//A	nonsynonymous	1	0
COL12A1	chr6	75831029/C//A	nonsynonymous	1	0
TBX18	chr6	85454021/C//T	nonsynonymous	1	0
ZNF292	chr6	87969667/G//T	nonsynonymous	1	0
POU3F2	chr6	99283908/C//T	nonsynonymous	1	0
TRDN	chr6	123824904/C//A	nonsynonymous	1	0
HBS1L	chr6	135315006/T//C	nonsynonymous	1	0
SYNE1	chr6	152469233/T//C	nonsynonymous	1	0
TAGAP	chr6	159457357/G//T	synonymous	1	0
PARK2	chr6	162864343/T//C	nonsynonymous	1	0
WDR27	chr6	170068258/T//C	nonsynonymous	1	0
CARD11	chr7	2976663/C//T	synonymous	1	0
SOSTDC1	chr7	16505400/G//C	synonymous	1	0
GLI3	chr7	42006210/C//A	nonsynonymous	1	0
OGDH	chr7	44735710/G//A	synonymous	1	0
TBRG4	chr7	45148473/T//C	nonsynonymous	1	0
CYP3A4	chr7	99361483/T//A	nonsynonymous	1	0
PILRB	chr7	99957091/G//T	nonsynonymous	1	0
TSC22D4	chr7	100075364/G//T	nonsynonymous	1	0
CDHR3	chr7	105636787/C//T	nonsynonymous	1	0
LAMB4	chr7	107746318/T//C	nonsynonymous	1	0
ZNF277	chr7	111981073/C//A	nonsynonymous	1	0
ASB15	chr7	123269038/T//C	synonymous	1	0
TRPV5	chr7	142630456/T//G	nonsynonymous	1	0
C7orf33	chr7	148312423/G//A	nonsynonymous	1	0
DPP6	chr7	154564595/G//T	nonsynonymous	1	0
ZNF596	chr8	192976/T//C	synonymous	1	0
PRAG1	chr8	8185318/G//T	nonsynonymous	1	0
WRN	chr8	30916685/G//A	nonsynonymous	1	0
NKX6-3	chr8	41503937/C//A	synonymous	1	0
WWP1	chr8	87447875/C//T	nonsynonymous	1	0
LRRC69	chr8	92201837/G//A	nonsynonymous	1	0
KIAA1429	chr8	95521950/T//C	nonsynonymous	1	0
DCSTAMP	chr8	105361005/C//T	synonymous	1	0
CSMD3	chr8	113237030/G//C	synonymous	1	0
ENPP2	chr8	120569881/T//A	synonymous	1	0
ARC	chr8	143694723/G//T	nonsynonymous	1	0
MAF1	chr8	145160556/C//T	synonymous	1	0
IFNE	chr9	21481352/A//G	synonymous	1	0
FRMPD1	chr9	37708396/G//C	nonsynonymous	1	0
PRUNE2	chr9	79322543/C//A	nonsynonymous	1	0
TLE1	chr9	84300771/C//A	nonsynonymous	1	0
SPIN1	chr9	91077454/C//T	nonsynonymous	1	0
HSD17B3	chr9	99015139/C//T	nonsynonymous	1	0
RABGAP1	chr9	125860043/G//C	synonymous	1	0
ZMYND19	chr9	140481441/C//A	nonsynonymous	1	0
ARMC3	chr10	23248029/A//G	nonsynonymous	1	0
GPR158	chr10	25888205/A//G	synonymous	1	0
PGBD3	chr10	50723370/T//G	synonymous	1	0
JMJD1C	chr10	64960406/C//A	nonsynonymous	1	0
STOX1	chr10	70652343/A//G	nonsynonymous	1	0
MSS51	chr10	75187453/C//T	nonsynonymous	1	0
PPIF	chr10	81112084/A//T	nonsynonymous	1	0
NRG3	chr10	83635804/C//A	nonsynonymous	1	0
FGFBP3	chr10	93668747/C//G	synonymous	1	0
CEP55	chr10	95287948/A//G	synonymous	1	0
PDE6C	chr10	95380670/T//C	nonsynonymous	1	0
SORCS3	chr10	106907418/C//A	nonsynonymous	1	0
ADAM12	chr10	127797169/A//T	nonsynonymous	1	0
RASSF7	chr11	562612/G//A	nonsynonymous	1	0
PDDC1	chr11	772447/C//T	nonsynonymous	1	0
OR52K1	chr11	4510574/G//T	nonsynonymous	1	0
OR51G1	chr11	4945085/G//T	nonsynonymous	1	0
OR56A3	chr11	5968608/C//A	nonsynonymous	1	0
CCKBR	chr11	6290998/G//A	nonsynonymous	1	0

TAF10	chr11	6632128/G//T	synonymous	1	0
GAS2	chr11	22759284/G//T	nonsynonymous	1	0
KIF18A	chr11	28090864/C//A	nonsynonymous	1	0
CD82	chr11	44636848/G//T	synonymous	1	0
PTPRJ	chr11	48161299/C//A	nonsynonymous	1	0
OR4A15	chr11	55135859/G//T	nonsynonymous	1	0
OR4S2	chr11	55419186/G//A	synonymous	1	0
OR5D18	chr11	55587408/A//T	nonsynonymous	1	0
OR5AP2	chr11	56409913/C//G	nonsynonymous	1	0
OR5B17	chr11	58126440/T//A	nonsynonymous	1	0
PACS1	chr11	65998332/G//C	nonsynonymous	1	0
USP28	chr11	113683190/C//A	nonsynonymous	1	0
CADM1	chr11	115088653/C//T	synonymous	1	0
DSCAML1	chr11	117332320/G//T	synonymous	1	0
KDM5A	chr12	394646/C//A	nonsynonymous	1	0
C12orf40	chr12	40076470/T//C	synonymous	1	0
C12orf40	chr12	40076606/G//T	nonsynonymous	1	0
NELL2	chr12	45108493/A//G	synonymous	1	0
KMT2D	chr12	49439843/C//A	synonymous	1	0
NEUROD4	chr12	55420319/G//T	nonsynonymous	1	0
AGAP2	chr12	58123542/G//A	nonsynonymous	1	0
MON2	chr12	62926222/G//T	nonsynonymous	1	0
PTPRQ	chr12	81062862/T//G	nonsynonymous	1	0
WSCD2	chr12	108604025/G//T	nonsynonymous	1	0
DDX54	chr12	113603774/C//G	nonsynonymous	1	0
GCN1	chr12	120582806/G//C	nonsynonymous	1	0
HNF1A	chr12	121426784/C//A	synonymous	1	0
P2RX7	chr12	121593889/C//T	nonsynonymous	1	0
ANAPC5	chr12	121746496/T//C	nonsynonymous	1	0
DNAH10	chr12	124403382/T//C	synonymous	1	0
RIMBP2	chr12	130907084/G//T	nonsynonymous	1	0
POLE	chr12	133202351/C//A	synonymous	1	0
TUBA3C	chr13	19751605/G//T	nonsynonymous	1	0
CCDC169-SOHLH2	chr13	36801502/C//A	nonsynonymous	1	0
RB1	chr13	48934254/G//T	nonsynonymous	1	0
CCDC168	chr13	103383558/T//C	nonsynonymous	1	0
CCDC168	chr13	103384484/T//A	nonsynonymous	1	0
CCDC168	chr13	103392414/C//A	nonsynonymous	1	0
KDELC1	chr13	103445751/A//G	synonymous	1	0
ATP11A	chr13	113481091/G//T	nonsynonymous	1	0
TGM1	chr14	24724726/G//C	synonymous	1	0
SOS2	chr14	50585478/C//T	nonsynonymous	1	0
DLGAP5	chr14	55618520/A//T	nonsynonymous	1	0
AREL1	chr14	75139593/T//C	nonsynonymous	1	0
POMT2	chr14	77765851/G//A	synonymous	1	0
SERPINA10	chr14	94754894/T//C	nonsynonymous	1	0
SERPINA1	chr14	94849205/G//A	nonsynonymous	1	0
SETD3	chr14	99865307/C//T	synonymous	1	0
BRF1	chr14	105707631/C//T	nonsynonymous	1	0
TMEM121	chr14	105995375/C//T	synonymous	1	0
TJP1	chr15	30008992/C//A	nonsynonymous	1	0
MEIS2	chr15	37388528/C//T	nonsynonymous	1	0
SH3GL3	chr15	84287216/T//C	synonymous	1	0
LINS1	chr15	101120475/C//A	nonsynonymous	1	0
OR4F6	chr15	102346650/A//T	nonsynonymous	1	0
ZNF205	chr16	3169581/G//A	nonsynonymous	1	0
GSPT1	chr16	11981553/T//A	synonymous	1	0
ERI2	chr16	20809677/G//A	nonsynonymous	1	0
ITGAM	chr16	31341637/C//T	synonymous	1	0
ZNF267	chr16	31885291/G//T	nonsynonymous	1	0
CHD9	chr16	53262954/A//T	nonsynonymous	1	0
SLC6A2	chr16	55734137/G//C	nonsynonymous	1	0
NDRG4	chr16	58540830/C//G	nonsynonymous	1	0
B3GNT9	chr16	67184389/C//G	synonymous	1	0
	chr16	75301866/C//A	synonymous	1	0
ZNF469	chr16	88496192/G//C	nonsynonymous	1	0
GAS8	chr16	90103775/G//T	nonsynonymous	1	0
SMYD4	chr17	1703217/C//T	nonsynonymous	1	0
CAMKK1	chr17	3773092/G//A	synonymous	1	0
MYOCD	chr17	12655975/C//A	nonsynonymous	1	0

PIPOX	chr17	27370247/G//A	nonsynonymous	1	0
TMEM132E	chr17	32959848/G//T	nonsynonymous	1	0
ACLY	chr17	40039389/T//C	nonsynonymous	1	0
ACLY	chr17	40062806/C//A	nonsynonymous	1	0
EFTUD2	chr17	42960467/G//A	synonymous	1	0
ABI3	chr17	47299492/C//T	nonsynonymous	1	0
EFCAB3	chr17	60484387/A//G	nonsynonymous	1	0
CDH2	chr18	25565717/G//T	nonsynonymous	1	0
LOXHD1	chr18	44069044/G//T	synonymous	1	0
TLE6	chr19	2987052/C//G	nonsynonymous	1	0
SH2D3A	chr19	6754916/G//A	nonsynonymous	1	0
ADAMTS10	chr19	8666008/C//A	nonsynonymous	1	0
OR1M1	chr19	9204223/G//T	nonsynonymous	1	0
ZNF317	chr19	9271156/G//C	nonsynonymous	1	0
S1PR2	chr19	10335411/C//T	synonymous	1	0
HSH2D	chr19	16268148/G//T	nonsynonymous	1	0
IL12RB1	chr19	18170743/T//A	synonymous	1	0
ZNF208	chr19	22155945/G//T	nonsynonymous	1	0
ZNF676	chr19	22363018/C//A	nonsynonymous	1	0
SPTBN4	chr19	41029415/T//C	synonymous	1	0
LTBP4	chr19	41111456/G//C	synonymous	1	0
ATP1A3	chr19	42490091/G//T	synonymous	1	0
PSG9	chr19	43766236/A//G	nonsynonymous	1	0
QPCTL	chr19	46196785/G//A	nonsynonymous	1	0
PGLYRP1	chr19	46522894/C//G	nonsynonymous	1	0
PRKD2	chr19	47195026/C//T	synonymous	1	0
NUP62	chr19	50411560/A//G	nonsynonymous	1	0
SIGLEC12	chr19	52004887/A//T	nonsynonymous	1	0
NLRP2	chr19	55493625/G//A	nonsynonymous	1	0
ZNF304	chr19	57868842/A//G	synonymous	1	0
GPCPD1	chr20	5559220/C//A	nonsynonymous	1	0
PAK5	chr20	9523252/C//A	nonsynonymous	1	0
PDRG1	chr20	30533666/A//C	nonsynonymous	1	0
CDK5RAP1	chr20	31960495/C//G	nonsynonymous	1	0
TOX2	chr20	42680053/G//A	synonymous	1	0
SALL4	chr20	50407914/C//G	nonsynonymous	1	0
CASS4	chr20	55027898/C//A	nonsynonymous	1	0
BIRC7	chr20	61870843/C//A	synonymous	1	0
GRIK1	chr21	31023576/G//T	nonsynonymous	1	0
KRTAP12-1	chr21	46101836/G//T	nonsynonymous	1	0
SEC14L4	chr22	30890909/T//C	nonsynonymous	1	0
GAL3ST1	chr22	30951143/T//A	nonsynonymous	1	0
EFCAB6	chr22	44028111/T//C	synonymous	1	0
DDX53	chrX	23018643/A//G	nonsynonymous	1	0
DDX53	chrX	23018896/G//C	nonsynonymous	1	0
AKAP4	chrX	49955570/G//T	synonymous	1	0
ARMCX1	chrX	100808746/C//T	nonsynonymous	1	0
CT83	chrX	115594047/G//A	synonymous	1	0
PER3	chr1	7844810/G//A	synonymous	0	1
EFCAB14-AS1	chr1	47152395/C//T	synonymous	0	1
WLS	chr1	68614325/G//A	nonsynonymous	0	1
NRAS	chr1	115251176/A//T	nonsynonymous	0	1
SV2A	chr1	149884831/A//C	nonsynonymous	0	1
RFX5	chr1	151316199/G//A	nonsynonymous	0	1
UAP1	chr1	162560144/G//C	nonsynonymous	0	1
FMO4	chr1	171303720/C//A	nonsynonymous	0	1
PAPPA2	chr1	176564100/T//G	nonsynonymous	0	1
C4BPA	chr1	207314561/G//A	nonsynonymous	0	1
SERTAD4	chr1	210415483/A//G	nonsynonymous	0	1
MARK1	chr1	220835207/A//C	nonsynonymous	0	1
MIA3	chr1	222801754/A//T	nonsynonymous	0	1
KCNK1	chr1	233807097/A//C	nonsynonymous	0	1
RBM34	chr1	235323818/T//TTAC	synonymous	0	1
FMN2	chr1	240371827/C//T	nonsynonymous	0	1
FAM228B	chr2	24384470/A//G	nonsynonymous	0	1
THUMPD2	chr2	39988537/A//C	synonymous	0	1
INSIG2	chr2	118854299/C//T	nonsynonymous	0	1
GPR17	chr2	128409305/G//A	synonymous	0	1
LRP1B	chr2	141356217/C//T	nonsynonymous	0	1
	chr2	152448582/A//G	synonymous	0	1

FMNL2	chr2	153486345/T//G	nonsynonymous	0	1
TTN	chr2	179498694/C//A	nonsynonymous	0	1
ZNF804A	chr2	185801900/A//C	nonsynonymous	0	1
COQ10B	chr2	198324736/G//A	nonsynonymous	0	1
COL4A4	chr2	227920748/G//A	nonsynonymous	0	1
SH3BP4	chr2	235962240/T//C	nonsynonymous	0	1
WNT7A	chr3	13896033/C//T	nonsynonymous	0	1
LOC105377102	chr3	57312115/T//G	synonymous	0	1
DRD3	chr3	113849961/T//G	synonymous	0	1
AGTR1	chr3	148458970/G//A	nonsynonymous	0	1
SORCS2	chr4	7684507/A//C	nonsynonymous	0	1
GSTCD	chr4	106650592/C//G	synonymous	0	1
SPOCK3	chr4	167675888/C//G	synonymous	0	1
ADAMTS12	chr5	33549457/A//T	nonsynonymous	0	1
MRPS30	chr5	44815406/G//A	synonymous	0	1
APC	chr5	112128142/G//C	nonsynonymous	0	1
UBE2D2	chr5	138941330/C//T	synonymous	0	1
PPP2R2B	chr5	146414779/A//G	synonymous	0	1
UNC5A	chr5	176304256/C//T	nonsynonymous	0	1
	chr5	176917415/A//G	synonymous	0	1
GFPT2	chr5	179762895/C//T	synonymous	0	1
MUC22	chr6	30993987/C//G	nonsynonymous	0	1
COL12A1	chr6	75844552/T//C	nonsynonymous	0	1
IBTK	chr6	82930431/C//G	nonsynonymous	0	1
NMBR	chr6	142409490/C//T	synonymous	0	1
UTRN	chr6	144837479/A//C	nonsynonymous	0	1
AKAP12	chr6	151672611/G//A	nonsynonymous	0	1
LPA	chr6	161011999/G//A	nonsynonymous	0	1
DNAH11	chr7	21599414/A//C	synonymous	0	1
ELMO1	chr7	37298780/G//C	synonymous	0	1
	chr7	44198722/C//T	synonymous	0	1
KIAA1324L	chr7	86568191/G//T	synonymous	0	1
AGBL3	chr7	134800342/C//A	nonsynonymous	0	1
KMT2C	chr7	151842372/C//T	synonymous	0	1
RAB11FIP1	chr8	37730615/AAGGAAGAGG//A	nonsynonymous	0	1
CNBD1	chr8	88365992/C//T	synonymous	0	1
RAD54B	chr8	95470624/T//A	nonsynonymous	0	1
HAS2	chr8	122641070/C//T	nonsynonymous	0	1
DMRT3	chr9	990616/G//T	nonsynonymous	0	1
MPDZ	chr9	13113003/A//C	synonymous	0	1
MPDZ	chr9	13222420/G//T	nonsynonymous	0	1
SPATA31E1	chr9	90500960/G//A	nonsynonymous	0	1
CTNNAL1	chr9	111741629/G//A	nonsynonymous	0	1
ITIH2	chr10	7774439/C//T	nonsynonymous	0	1
ZNF33A	chr10	38344460/G//C	nonsynonymous	0	1
IPMK	chr10	59997561/G//A	nonsynonymous	0	1
HOGA1	chr10	99361627/C//T	synonymous	0	1
HPSE2	chr10	100481555/C//T	nonsynonymous	0	1
BBIP1	chr10	112660288/A//C	synonymous	0	1
EBF3	chr10	131760427/T//G	synonymous	0	1
CYP2E1	chr10	135345184/A//G	nonsynonymous	0	1
CHRNA10	chr11	3687482/G//A	nonsynonymous	0	1
PC	chr11	66616565/C//T	synonymous	0	1
DLG2	chr11	83676428/T//G	nonsynonymous	0	1
SESN3	chr11	94910997/T//G	nonsynonymous	0	1
DYNC2H1	chr11	103027315/T//C	nonsynonymous	0	1
DDI1	chr11	103907477/C//T	synonymous	0	1
ALG9	chr11	111731303/C//A	nonsynonymous	0	1
OVCH1	chr12	29649172/T//G	nonsynonymous	0	1
PDZRN4	chr12	41903633/T//C	nonsynonymous	0	1
MRPS31	chr13	41340972/G//T	nonsynonymous	0	1
DNAJC3	chr13	96438294/C//T	nonsynonymous	0	1
TAT	chr16	71607434/A//C	synonymous	0	1
CNTNAP4	chr16	76482789/G//A	nonsynonymous	0	1
CNTNAP4	chr16	76555983/G//C	nonsynonymous	0	1
DNAH2	chr17	7683470/T//C	synonymous	0	1
DDX52	chr17	35988675/G//C	nonsynonymous	0	1
KRT27	chr17	38936568/T//C	nonsynonymous	0	1
CD300A	chr17	72469704/G//A	nonsynonymous	0	1
TGIF1	chr18	3457679/C//T	nonsynonymous	0	1

ADAMTSL5	chr19	1506176/C//T	synonymous	0	1
ZNF676	chr19	22363207/C//T	nonsynonymous	0	1
KIAA0355	chr19	34819054/A//T	nonsynonymous	0	1
NFKBID	chr19	36387703/C//T	synonymous	0	1
LILRA2	chr19	55085309/C//T	synonymous	0	1
LILRA2	chr19	55085345/C//T	synonymous	0	1
KIF16B	chr20	16360383/T//A	nonsynonymous	0	1
C20orf78	chr20	18790746/C//T	nonsynonymous	0	1
APMAP	chr20	24954344/G//A	nonsynonymous	0	1
BPIFB2	chr20	31609157/C//T	synonymous	0	1
CTNNBL1	chr20	36406063/G//A	synonymous	0	1
ZMYND8	chr20	45850020/G//A	nonsynonymous	0	1
SYCP2	chr20	58456073/C//T	nonsynonymous	0	1
CDH4	chr20	60504690/C//T	nonsynonymous	0	1
TEX33	chr22	37387220/C//A	nonsynonymous	0	1
BAIAP2L2	chr22	38484909/C//T	nonsynonymous	0	1
TMEM27	chrX	15682848/A//G	synonymous	0	1
KCNH1	chr1	211192426/C//T	nonsynonymous	0	1
C7	chr5	40909614/G//C	synonymous	0	1
DBN1	chr5	176885149/C//T	synonymous	0	1
VARS	chr6	31745355/G//A	synonymous	0	1
ATM	chr11	108216623/ACTT//A	nonsynonymous	0	1
PLIN4	chr19	4511575/C//G	synonymous	0	1
CD52	chr1	26644535/C//T	synonymous	0	1
ZSCAN20	chr1	33961016/C//A	nonsynonymous	0	1
MROH7	chr1	55139709/G//T	synonymous	0	1
PRPF3	chr1	150305648/C//A	nonsynonymous	0	1
RUSC1	chr1	155295653/A//G	nonsynonymous	0	1
KCNJ9	chr1	160054338/TCTC//T	nonsynonymous	0	1
LMX1A	chr1	165322441/C//A	nonsynonymous	0	1
LRRC52	chr1	165532959/G//A	synonymous	0	1
ASTN1	chr1	176983962/G//A	synonymous	0	1
OBSCN	chr1	228528950/TGAA//T	nonsynonymous	0	1
VRK2	chr2	58373454/G//T	nonsynonymous	0	1
TACR1	chr2	75425746/G//T	nonsynonymous	0	1
NEB	chr2	152466344/C//A	nonsynonymous	0	1
NEB	chr2	152527602/A//G	nonsynonymous	0	1
HIBCH	chr2	191077780/T//C	nonsynonymous	0	1
ABCA12	chr2	215872531/T//C	nonsynonymous	0	1
PTPRN	chr2	220168485/G//A	nonsynonymous	0	1
EIF4E2	chr2	233431864/G//A	nonsynonymous	0	1
UBA7	chr3	49847996/C//T	nonsynonymous	0	1
SEMA3G	chr3	52475350/GAGA//G	nonsynonymous	0	1
PBRM1	chr3	52610695/G//A	nonsynonymous	0	1
ZNF654	chr3	88188681/G//A	nonsynonymous	0	1
SPICE1	chr3	113225453/CT//C	nonsynonymous	0	1
PLXNA1	chr3	126723491/C//T	nonsynonymous	0	1
MCM2	chr3	127340009/C//T	nonsynonymous	0	1
LRRC34	chr3	169513364/CCTT//C	nonsynonymous	0	1
RTP1	chr3	186915345/G//C	synonymous	0	1
	chr4	5894524/C//T	synonymous	0	1
TLR3	chr4	187004187/C//T	synonymous	0	1
APC	chr5	112175097/TA//T	nonsynonymous	0	1
LVRN	chr5	115361714/A//T	nonsynonymous	0	1
EDN1	chr6	12292689/TAAAG//T	nonsynonymous	0	1
SLC17A5	chr6	74363647/A//G	synonymous	0	1
FILIP1	chr6	76124433/T//C	nonsynonymous	0	1
HTR1E	chr6	87725621/C//T	nonsynonymous	0	1
HTR1E	chr6	87725844/G//A	synonymous	0	1
CFAP206	chr6	88125559/C//T	nonsynonymous	0	1
PM20D2	chr6	89856287/G//A	nonsynonymous	0	1
ADGB	chr6	146993375/G//C	nonsynonymous	0	1
NOX3	chr6	155750119/C//T	synonymous	0	1
COBL	chr7	51111153/C//T	nonsynonymous	0	1
ZNF727	chr7	63538770/G//A	nonsynonymous	0	1
CLIP2	chr7	73778611/TGGA//T	nonsynonymous	0	1
OR2AE1	chr7	99473990/T//A	nonsynonymous	0	1
RELN	chr7	103206762/A//G	synonymous	0	1
KMT2C	chr7	151853423/A//G	synonymous	0	1
CSMD3	chr8	113562966/T//C	nonsynonymous	0	1

CDK5RAP2	chr9	123165286/G//A	nonsynonymous	0	1
TTF1	chr9	135266140/ATCT//A	nonsynonymous	0	1
NDOR1	chr9	140108792/CAG//C	nonsynonymous	0	1
CUBN	chr10	16882464/T//A	synonymous	0	1
CASC10	chr10	21785840/G//A	synonymous	0	1
C10orf71	chr10	50533669/C//A	nonsynonymous	0	1
CCNJ	chr10	97816519/T//TA	nonsynonymous	0	1
NRIP3	chr11	9009762/C//T	nonsynonymous	0	1
ATG13	chr11	46670710/A//G	nonsynonymous	0	1
BTBD18	chr11	57518682/CCTT//C	synonymous	0	1
KMT2A	chr11	118344015/C//T	nonsynonymous	0	1
ARHGEF12	chr11	120295075/A//C	synonymous	0	1
FOXM1	chr12	2975690/GGAA//G	synonymous	0	1
TAS2R50	chr12	11139209/GAAGT//G	nonsynonymous	0	1
TMTC1	chr12	29904634/G//A	synonymous	0	1
SLC25A3	chr12	98991753/ACT//A	nonsynonymous	0	1
MAPKAPK5	chr12	112306614/C//T	synonymous	0	1
GJA3	chr13	20717032/C//T	synonymous	0	1
TRPC4	chr13	38229402/C//A	nonsynonymous	0	1
COG6	chr13	40230001/G//A	synonymous	0	1
CCDC168	chr13	103391055/T//C	nonsynonymous	0	1
OR10G3	chr14	22038380/G//A	nonsynonymous	0	1
BCL2L2-PABPN1	chr14	23777028/G//T	nonsynonymous	0	1
SYNE2	chr14	64687196/C//G	synonymous	0	1
TDRD9	chr14	104501374/G//A	nonsynonymous	0	1
GABRG3	chr15	27773121/A//G	nonsynonymous	0	1
SEMA6D	chr15	48063247/T//C	synonymous	0	1
MYO5A	chr15	52656824/T//C	nonsynonymous	0	1
RHOT2	chr16	722370/G//A	nonsynonymous	0	1
MMP25	chr16	3100249/C//T	synonymous	0	1
KCTD19	chr16	67328477/GCTTA//G	nonsynonymous	0	1
GPR179	chr17	36482529/CCTT//C	nonsynonymous	0	1
FAM134C	chr17	40761321/G//A	nonsynonymous	0	1
EPX	chr17	56276569/G//T	synonymous	0	1
CLTC	chr17	57768030/AAAG//A	nonsynonymous	0	1
GGA3	chr17	73237555/T//C	nonsynonymous	0	1
RHBDF2	chr17	74471161/G//A	nonsynonymous	0	1
PIGU	chr20	33264825/G//C	nonsynonymous	0	1
EMILIN3	chr20	39991219/ACACT//A	nonsynonymous	0	1
CDH22	chr20	44841656/T//C	nonsynonymous	0	1
COL20A1	chr20	61943889/C//T	nonsynonymous	0	1
TMPRSS15	chr21	19770649/A//G	synonymous	0	1
IL10RB	chr21	34649060/T//G	nonsynonymous	0	1
IL10RB	chr21	34649062/A//T	synonymous	0	1
	chrX	131842512/A//C	synonymous	0	1
EVI5	chr1	93170107/A//G	synonymous	1	0
TUFT1	chr1	151547482/G//C	nonsynonymous	1	0
THEM5	chr1	151820687/G//A	synonymous	1	0
HDGF	chr1	156713499/C//G	nonsynonymous	1	0
AOX1	chr2	201527611/C//T	synonymous	1	0
SFMBT1	chr3	52939283/C//G	nonsynonymous	1	0
NIT2	chr3	100074154/T//C	synonymous	1	0
	chr4	122148570/C//A	synonymous	1	0
PCDHA5	chr5	140202808/C//T	nonsynonymous	1	0
COL28A1	chr7	7529063/G//A	nonsynonymous	1	0
ZNF92	chr7	64864377/T//A	nonsynonymous	1	0
SLC26A5	chr7	103017274/T//C	synonymous	1	0
GIMAP8	chr7	150171469/T//A	nonsynonymous	1	0
NEBL	chr10	21076214/G//T	nonsynonymous	1	0
PDE3A	chr12	20799806/G//A	synonymous	1	0
METTL7B	chr12	56075897/A//T	nonsynonymous	1	0
MYBPC1	chr12	102069131/C//T	synonymous	1	0
MYBPC1	chr12	102071165/C//T	synonymous	1	0
DDX54	chr12	113618768/C//CTTT	nonsynonymous	1	0
PAX9	chr14	37132303/G//A	nonsynonymous	1	0
PRKCB	chr16	24135288/G//A	nonsynonymous	1	0
PRKCB	chr16	24135292/G//A	nonsynonymous	1	0
RNF157	chr17	74158640/T//A	nonsynonymous	1	0
CBLN2	chr18	70205557/G//T	nonsynonymous	1	0
CYP4F2	chr19	16003209/G//T	nonsynonymous	1	0

FPGT-TNNI3K	chr1	75009635/G//A	nonsynonymous	0	1
ACTA1	chr1	229568022/G//A	nonsynonymous	0	1
OR2M4	chr1	248402692/T//A	synonymous	0	1
USP39	chr2	85843229/G//T	synonymous	0	1
HOXD10	chr2	176983761/GCAC//G	nonsynonymous	0	1
TTN	chr2	179498143/C//T	nonsynonymous	0	1
SNED1	chr2	241992622/C//T	synonymous	0	1
RTP1	chr3	186915535/C//A	nonsynonymous	0	1
HSPA9	chr5	137903256/G//T	synonymous	0	1
FIG4	chr6	110059570/G//A	nonsynonymous	0	1
RPA3	chr7	7677540/A//C	nonsynonymous	0	1
AMPH	chr7	38515010/C//A	nonsynonymous	0	1
ZNF3	chr7	99669695/T//TTTTCAGACTGACT	nonsynonymous	0	1
MUC17	chr7	100683521/G//A	nonsynonymous	0	1
PENK	chr8	57354184/C//T	nonsynonymous	0	1
TSPYL5	chr8	98289971/G//A	synonymous	0	1
LRP12	chr8	105510312/G//T	synonymous	0	1
PPP1R3C	chr10	93390459/G//T	nonsynonymous	0	1
MMS19	chr10	99220700/C//T	synonymous	0	1
ADAM8	chr10	135085450/G//T	nonsynonymous	0	1
NT5DC3	chr12	104171660/C//G	nonsynonymous	0	1
ATP7B	chr13	52515275/A//C	nonsynonymous	0	1
	chr16	66582929/A//G	synonymous	0	1
ZFHX3	chr16	72993436/G//A	synonymous	0	1
USP10	chr16	84778593/A//T	nonsynonymous	0	1
CCDC144NL	chr17	20769899/G//T	nonsynonymous	0	1
CCDC144NL	chr17	20769904/G//A	synonymous	0	1
ABCA10	chr17	67160237/G//C	nonsynonymous	0	1
C18orf8	chr18	21109621/T//C	nonsynonymous	0	1
MBD3	chr19	1581165/C//G	nonsynonymous	0	1
ZNF208	chr19	22154522/C//T	nonsynonymous	0	1
CEACAM5	chr19	42223790/ATGCCTGTGAGGAAT(	nonsynonymous	0	1
ZNF835	chr19	57175312/T//C	nonsynonymous	0	1
BPIFA1	chr20	31829869/C//G	nonsynonymous	0	1
IL1RAPL1	chrX	29973480/A//G	nonsynonymous	0	1
ANAPC4	chr4	25408830/T//C	synonymous	0	1
NAALADL1	chr11	64825678/T//C	nonsynonymous	0	1
ULK3	chr15	75130094/T//C	nonsynonymous	0	1
ZNF626	chr19	20807474/G//T	synonymous	0	1
ADGRB2	chr1	32203115/C//A	nonsynonymous	0	1
	chr1	33476387/C//T	synonymous	0	1
	chr1	33476396/G//A	synonymous	0	1
	chr1	33476404/T//G	synonymous	0	1
SERBP1	chr1	67889969/A//G	synonymous	0	1
TTN	chr2	179396885/G//A	synonymous	0	1
DMGDH	chr5	78365412/C//T	nonsynonymous	0	1
MUC17	chr7	100683233/A//G	nonsynonymous	0	1
MUC17	chr7	100683240/G//C	nonsynonymous	0	1
MUC17	chr7	100684389/A//C	nonsynonymous	0	1
MEST	chr7	130138309/T//C	nonsynonymous	0	1
KIAA1549	chr7	138601659/C//T	nonsynonymous	0	1
MMP16	chr8	89209517/C//T	nonsynonymous	0	1
OR1L8	chr9	125330145/T//C	synonymous	0	1
LCN1	chr9	138416725/C//T	synonymous	0	1
PALD1	chr10	72324237/G//A	nonsynonymous	0	1
ALG8	chr11	77815396/G//A	synonymous	0	1
TMEM132C	chr12	128899647/C//T	synonymous	0	1
CHFR	chr12	133425249/G//A	synonymous	0	1
CYFIP1	chr15	22925862/C//G	nonsynonymous	0	1
FBN1	chr15	48776038/G//A	nonsynonymous	0	1
TP53	chr17	7578253/C//A	nonsynonymous	0	1
C18orf32	chr18	47010042/G//T	nonsynonymous	0	1
ZNF493	chr19	21607030/C//A	synonymous	0	1
GAPDHS	chr19	36029295/C//T	nonsynonymous	0	1
FCGBP	chr19	40434072/G//A	nonsynonymous	0	1
TRPM4	chr19	49693561/C//T	nonsynonymous	0	1
ZNF776	chr19	58265741/T//TG	nonsynonymous	0	1
ASIP	chr20	32850684/G//T	nonsynonymous	0	1
RGS19	chr20	62707990/G//A	nonsynonymous	0	1
COL6A2	chr21	47546454/A//G	synonymous	0	1

NUDT10	chrX	51075748/G//C	synonymous	0	1
NUDT10	chrX	51075755/A//G	synonymous	0	1
NHSL2	chrX	71359952/G//A	nonsynonymous	0	1
SCNN1D	chr1	1223181/C//T	nonsynonymous	0	1
WDTC1	chr1	27632878/C//T	synonymous	0	1
S1PR1	chr1	101705240/C//T	nonsynonymous	0	1
PRPF3	chr1	150312945/A//G	nonsynonymous	0	1
OR6Y1	chr1	158517100/G//A	nonsynonymous	0	1
SPTA1	chr1	158582712/C//A	synonymous	0	1
TP53BP2	chr1	224009051/G//GATAC	nonsynonymous	0	1
DNAH14	chr1	225418830/C//T	synonymous	0	1
DNAH14	chr1	225418856/C//T	synonymous	0	1
MYT1L	chr2	1914112/C//T	nonsynonymous	0	1
OTOF	chr2	26712121/C//T	nonsynonymous	0	1
DYSF	chr2	71755516/C//T	synonymous	0	1
ZNF514	chr2	95818911/T//G	nonsynonymous	0	1
SH3RF3	chr2	110049067/C//T	nonsynonymous	0	1
PTPRN	chr2	220161043/C//T	nonsynonymous	0	1
NYAP2	chr2	226447359/C//T	nonsynonymous	0	1
ALPPL2	chr2	233273244/C//A	nonsynonymous	0	1
HRH1	chr3	11300836/G//T	nonsynonymous	0	1
ULK4	chr3	41795958/C//T	nonsynonymous	0	1
FLNB	chr3	58110223/T//C	nonsynonymous	0	1
KY	chr3	134323032/C//A	nonsynonymous	0	1
SKIL	chr3	170078221/G//C	synonymous	0	1
NAALADL2	chr3	174951783/A//T	nonsynonymous	0	1
DNAJB11	chr3	186302347/A//C	synonymous	0	1
ZFYVE28	chr4	2341196/C//T	nonsynonymous	0	1
SLIT2	chr4	20255355/G//A	synonymous	0	1
COMMD8	chr4	47465645/G//A	synonymous	0	1
RNF150	chr4	141832422/G//A	synonymous	0	1
TRIO	chr5	14369609/G//A	nonsynonymous	0	1
SLC38A9	chr5	54922354/C//T	nonsynonymous	0	1
ARHGEF28	chr5	73207312/G//A	synonymous	0	1
APC	chr5	112175480/G//T	nonsynonymous	0	1
PCDHB13	chr5	140595723/G//A	synonymous	0	1
PCDHGB3	chr5	140750854/C//T	nonsynonymous	0	1
HDAC3	chr5	141005808/G//A	synonymous	0	1
KIF13A	chr6	17783899/T//G	synonymous	0	1
HLA-DRA	chr6	32410347/C//T	nonsynonymous	0	1
FOXP4	chr6	41559001/A//G	nonsynonymous	0	1
ZNF292	chr6	87969545/A//G	synonymous	0	1
AKAP12	chr6	151673991/T//C	nonsynonymous	0	1
CCDC129	chr7	31614355/C//T	synonymous	0	1
PDE1C	chr7	31890277/T//A	nonsynonymous	0	1
COL1A2	chr7	94057162/G//A	nonsynonymous	0	1
CCDC136	chr7	128457907/G//A	synonymous	0	1
HTR5A	chr7	154862972/C//T	synonymous	0	1
DLGAP2	chr8	1616768/G//A	nonsynonymous	0	1
HR	chr8	21976581/G//A	synonymous	0	1
NRG1	chr8	32617751/C//T	synonymous	0	1
RBM12B	chr8	94747853/T//G	nonsynonymous	0	1
ADGRB1	chr8	143614743/C//T	synonymous	0	1
ZFP41	chr8	144332401/G//A	nonsynonymous	0	1
PLEC	chr8	144998262/C//T	synonymous	0	1
KIAA2026	chr9	5944921/T//A	nonsynonymous	0	1
NOL8	chr9	95078250/C//G	nonsynonymous	0	1
CCDC180	chr9	100111480/A//C	nonsynonymous	0	1
COL15A1	chr9	101777835/G//A	nonsynonymous	0	1
TNC	chr9	117849524/G//A	synonymous	0	1
TSC1	chr9	135772018/T//TC	nonsynonymous	0	1
CACNA1B	chr9	140919436/G//T	nonsynonymous	0	1
ITPRIP	chr10	106074893/G//A	nonsynonymous	0	1
CACUL1	chr10	120445584/C//T	synonymous	0	1
IKZF5	chr10	124753870/C//T	nonsynonymous	0	1
MUC2	chr11	1086062/G//C	nonsynonymous	0	1
NAV2	chr11	20005744/C//T	nonsynonymous	0	1
PCNX3	chr11	65385924/C//T	nonsynonymous	0	1
SLCO1C1	chr12	20858984/C//T	nonsynonymous	0	1
FZD10	chr12	130647807/G//A	nonsynonymous	0	1

GALNT9	chr12	132688190/C//T	nonsynonymous	0	1
GJA3	chr13	20716950/C//T	nonsynonymous	0	1
WDR89	chr14	64065773/G//T	nonsynonymous	0	1
DCAF5	chr14	69521868/C//T	nonsynonymous	0	1
ABCD4	chr14	74763130/C//T	nonsynonymous	0	1
KCNK10	chr14	88652306/C//T	nonsynonymous	0	1
AHNAK2	chr14	105407965/G//A	nonsynonymous	0	1
ATP10A	chr15	25959144/G//A	nonsynonymous	0	1
RYR3	chr15	34152846/C//T	nonsynonymous	0	1
ACTC1	chr15	35084689/C//T	nonsynonymous	0	1
DUOXA1	chr15	45415067/G//A	nonsynonymous	0	1
PIGB	chr15	55647094/A//T	nonsynonymous	0	1
SYNM	chr15	99670458/C//T	synonymous	0	1
ABCA3	chr16	2329026/G//A	nonsynonymous	0	1
TP53	chr17	7579349/A//G	nonsynonymous	0	1
KRT33A	chr17	39506777/G//A	synonymous	0	1
SDK2	chr17	71410804/G//A	synonymous	0	1
MIR6785	chr17	73494642/C//T	synonymous	0	1
	chr19	3852565/C//G	synonymous	0	1
ZBTB7A	chr19	4054557/G//T	nonsynonymous	0	1
TIMM44	chr19	7997740/C//T	nonsynonymous	0	1
PPFIA3	chr19	49636350/C//T	synonymous	0	1
TSKS	chr19	50266451/G//A	synonymous	0	1
PPP6R1	chr19	55752908/G//A	synonymous	0	1
HSPBP1	chr19	55789129/A//G	nonsynonymous	0	1
ZNF418	chr19	58441821/C//A	nonsynonymous	0	1
CTSA	chr20	44520671/GT//G	synonymous	0	1
OCSTAMP	chr20	45170434/C//T	nonsynonymous	0	1
CDH26	chr20	58558000/G//A	nonsynonymous	0	1
ABHD16B	chr20	62493892/CCT//C	nonsynonymous	0	1
TPTE	chr21	10916424/G//T	nonsynonymous	0	1
KRTAP10-6	chr21	46011459/A//T	nonsynonymous	0	1
KRTAP10-10	chr21	46057634/T//C	synonymous	0	1
IL17RA	chr22	17590339/G//A	nonsynonymous	0	1
GABRA3	chrX	151376560/C//T	nonsynonymous	0	1
IL9R	chrX	155232691/G//A	synonymous	0	1
C1orf127	chr1	11015069/G//A	nonsynonymous	0	1
MTOR	chr1	11291472/C//T	nonsynonymous	0	1
NUDC	chr1	27269428/C//T	nonsynonymous	0	1
CSMD2	chr1	34042990/G//A	synonymous	0	1
ADGRL2	chr1	82445558/T//G	nonsynonymous	0	1
ATP1A1	chr1	116940595/G//GA	nonsynonymous	0	1
CD2	chr1	117311137/G//A	nonsynonymous	0	1
PDE4DIP	chr1	144917819/C//T	synonymous	0	1
SNX27	chr1	151665508/C//T	nonsynonymous	0	1
FLG	chr1	152279766/C//T	synonymous	0	1
MROH9	chr1	170952642/G//T	nonsynonymous	0	1
LMOD1	chr1	201868770/G//A	synonymous	0	1
PTPN7	chr1	202127427/C//T	nonsynonymous	0	1
PPP1R12B	chr1	202462309/C//G	nonsynonymous	0	1
SYT2	chr1	202571524/A//T	nonsynonymous	0	1
PIK3C2B	chr1	204438146/G//A	nonsynonymous	0	1
NFASC	chr1	204956671/A//G	synonymous	0	1
CAPN9	chr1	230898463/G//A	nonsynonymous	0	1
OPN3	chr1	241757901/C//A	nonsynonymous	0	1
OPN3	chr1	241757914/C//T	nonsynonymous	0	1
LYPD8	chr1	248902813/C//T	synonymous	0	1
PXDN	chr2	1647152/C//T	nonsynonymous	0	1
GREB1	chr2	11725913/G//A	synonymous	0	1
EFR3B	chr2	25344633/C//T	nonsynonymous	0	1
STON1	chr2	48822492/C//T	synonymous	0	1
CD207	chr2	71061146/G//T	synonymous	0	1
TLX2	chr2	74743192/G//A	nonsynonymous	0	1
NPHP1	chr2	110959012/T//C	synonymous	0	1
FIGN	chr2	164466711/C//G	nonsynonymous	0	1
XIRP2	chr2	168105787/C//T	nonsynonymous	0	1
RAPGEF4	chr2	173916484/C//T	nonsynonymous	0	1
TTN	chr2	179588222/G//T	nonsynonymous	0	1
ZNF804A	chr2	185800665/C//A	nonsynonymous	0	1
SDPR	chr2	192701184/C//T	nonsynonymous	0	1

SF3B1	chr2	198268383/G//A	nonsynonymous	0	1
FAM124B	chr2	225266035/C//G	nonsynonymous	0	1
UBE2E2	chr3	23631309/G//A	nonsynonymous	0	1
ARPP21	chr3	35780803/T//G	synonymous	0	1
SETD2	chr3	47147574/G//A	synonymous	0	1
IMPDH2	chr3	49065725/T//G	nonsynonymous	0	1
MAGI1	chr3	65425594/C//A	nonsynonymous	0	1
LRIG1	chr3	66455621/C//G	nonsynonymous	0	1
MITF	chr3	70014217/G//A	nonsynonymous	0	1
FRG2C	chr3	75714950/C//A	nonsynonymous	0	1
FRG2C	chr3	75714980/C//A	synonymous	0	1
DTX3L	chr3	122288808/TC//T	nonsynonymous	0	1
PLXNA1	chr3	126707962/G//A	nonsynonymous	0	1
CLDN18	chr3	137749822/G//A	nonsynonymous	0	1
CHST2	chr3	142840662/G//A	nonsynonymous	0	1
BCHE	chr3	165548605/T//G	nonsynonymous	0	1
EPHB3	chr3	184299294/AC//A	synonymous	0	1
MUC4	chr3	195477791/G//A	synonymous	0	1
ZFYVE28	chr4	2272571/G//A	nonsynonymous	0	1
	chr4	6043906/G//T	synonymous	0	1
NWD2	chr4	37445570/G//A	nonsynonymous	0	1
DCAF4L1	chr4	41985017/G//A	synonymous	0	1
KDR	chr4	55968597/G//A	nonsynonymous	0	1
UNC5C	chr4	96469992/C//T	nonsynonymous	0	1
PCDH10	chr4	134072608/G//A	nonsynonymous	0	1
PCDH10	chr4	134073220/G//A	nonsynonymous	0	1
FREM3	chr4	144619718/T//C	nonsynonymous	0	1
SH3D19	chr4	152048847/C//G	nonsynonymous	0	1
AADAT	chr4	171010904/G//A	synonymous	0	1
TRIML1	chr4	189063516/A//G	synonymous	0	1
APC	chr5	112175324/G//T	nonsynonymous	0	1
PCDHA7	chr5	140215612/C//T	synonymous	0	1
PCDHB5	chr5	140515898/C//T	synonymous	0	1
PCDHB7	chr5	140554747/A//C	synonymous	0	1
CCDC69	chr5	150562922/C//T	synonymous	0	1
HIST1H4B	chr6	26027476/G//A	nonsynonymous	0	1
TRIM10	chr6	30122008/C//T	nonsynonymous	0	1
C2	chr6	31911201/C//A	nonsynonymous	0	1
PHF1	chr6	33382788/G//A	nonsynonymous	0	1
ADGRB3	chr6	69943287/G//T	nonsynonymous	0	1
FAXC	chr6	99771429/C//T	synonymous	0	1
AKAP12	chr6	151670824/C//T	nonsynonymous	0	1
SYNE1	chr6	152652719/G//A	synonymous	0	1
LPA	chr6	160968970/C//G	nonsynonymous	0	1
GRID2IP	chr7	6590860/G//A	nonsynonymous	0	1
MET	chr7	116339238/G//A	nonsynonymous	0	1
RBM28	chr7	127983793/G//A	nonsynonymous	0	1
TMEM213	chr7	138487844/C//T	synonymous	0	1
TMEM178B	chr7	140912470/G//A	synonymous	0	1
CSMD1	chr8	3165968/C//T	nonsynonymous	0	1
BLK	chr8	11406628/A//G	nonsynonymous	0	1
USP17L2	chr8	11995059/G//A	nonsynonymous	0	1
MSR1	chr8	15978003/G//A	synonymous	0	1
STMN4	chr8	27094329/C//T	synonymous	0	1
PRKDC	chr8	48771530/C//T	synonymous	0	1
NPBWR1	chr8	53852577/C//A	nonsynonymous	0	1
	chr8	85097141/T//C	synonymous	0	1
MMP16	chr8	89179901/C//T	nonsynonymous	0	1
TSPYL5	chr8	98289940/C//T	nonsynonymous	0	1
ZFP41	chr8	144332656/G//A	synonymous	0	1
CCDC171	chr9	15744625/C//T	nonsynonymous	0	1
UBAP1	chr9	34241696/T//C	nonsynonymous	0	1
CD72	chr9	35618230/C//T	nonsynonymous	0	1
C9orf64	chr9	86570455/T//C	synonymous	0	1
ZNF782	chr9	99581108/G//A	synonymous	0	1
ZFP37	chr9	115806161/C//A	nonsynonymous	0	1
SFMBT2	chr10	7409746/C//T	nonsynonymous	0	1
ARMC3	chr10	23290920/G//A	nonsynonymous	0	1
ZEB1	chr10	31810508/A//T	nonsynonymous	0	1
WDFY4	chr10	50108272/G//A	nonsynonymous	0	1

MYPN	chr10	69959257/G//A	nonsynonymous	0	1
RBM20	chr10	112583354/G//T	nonsynonymous	0	1
TCERG1L	chr10	133107543/G//C	nonsynonymous	0	1
OR51D1	chr11	4661424/G//A	nonsynonymous	0	1
OR51G1	chr11	4944909/C//T	nonsynonymous	0	1
PRKCDBP	chr11	6340592/C//T	nonsynonymous	0	1
DCHS1	chr11	6661827/G//A	nonsynonymous	0	1
SBF2	chr11	10051373/G//T	nonsynonymous	0	1
ADM	chr11	10327269/C//T	synonymous	0	1
BTBD10	chr11	13435172/C//T	nonsynonymous	0	1
PRMT3	chr11	20515739/C//G	nonsynonymous	0	1
KIAA1549L	chr11	33631349/C//T	nonsynonymous	0	1
LRRC55	chr11	56954811/A//G	nonsynonymous	0	1
SLC22A9	chr11	63177310/G//A	synonymous	0	1
DYNC2H1	chr11	103006456/C//T	nonsynonymous	0	1
GRIA4	chr11	105774539/G//A	nonsynonymous	0	1
FGF23	chr12	4479711/G//A	nonsynonymous	0	1
KCNA5	chr12	5153592/C//T	synonymous	0	1
CLEC12A	chr12	10131592/G//A	nonsynonymous	0	1
ITPR2	chr12	26839532/G//A	nonsynonymous	0	1
DENND5B	chr12	31633096/G//A	nonsynonymous	0	1
ADAMTS20	chr12	43895992/G//T	nonsynonymous	0	1
DIP2B	chr12	51135262/G//A	nonsynonymous	0	1
TARBP2	chr12	53898584/C//T	nonsynonymous	0	1
TESPA1	chr12	55356244/G//A	nonsynonymous	0	1
USP15	chr12	62778051/G//C	nonsynonymous	0	1
CLIP1	chr12	122825735/G//A	synonymous	0	1
TNFRSF19	chr13	24200860/C//T	nonsynonymous	0	1
WASF3	chr13	27216411/C//T	nonsynonymous	0	1
CSNK1A1L	chr13	37679574/G//A	synonymous	0	1
	chr13	45911548/T//A	synonymous	0	1
COL4A2	chr13	111125291/G//A	nonsynonymous	0	1
OR4K15	chr14	20443944/C//T	synonymous	0	1
RAB2B	chr14	21930566/G//A	synonymous	0	1
MIS18BP1	chr14	45711497/T//G	nonsynonymous	0	1
MAP3K9	chr14	71275841/A//AGCG	nonsynonymous	0	1
MAP3K9	chr14	71275847/G//GGC	nonsynonymous	0	1
SPTBN5	chr15	42160355/C//T	nonsynonymous	0	1
ZNF106	chr15	42731621/C//T	nonsynonymous	0	1
PYGO1	chr15	55838402/T//C	nonsynonymous	0	1
IGDCC4	chr15	65688147/C//T	nonsynonymous	0	1
EFL1	chr15	82521441/T//C	nonsynonymous	0	1
KIF7	chr15	90192286/G//A	nonsynonymous	0	1
CASKIN1	chr16	2239249/C//T	nonsynonymous	0	1
FOXC2	chr16	86601863/G//A	nonsynonymous	0	1
FOXL1	chr16	86612374/G//A	synonymous	0	1
TP53	chr17	7577570/C//A	nonsynonymous	0	1
MAPK7	chr17	19283207/C//T	synonymous	0	1
RHBDL3	chr17	30648078/G//A	nonsynonymous	0	1
KRT12	chr17	39023165/C//G	nonsynonymous	0	1
KRTAP17-1	chr17	39471556/G//A	synonymous	0	1
B4GALNT2	chr17	47241524/C//T	nonsynonymous	0	1
TMEM200C	chr18	5891154/C//A	synonymous	0	1
MTCL1	chr18	8825566/G//C	nonsynonymous	0	1
PIEZO2	chr18	10759788/G//T	synonymous	0	1
CDH2	chr18	25591863/G//A	nonsynonymous	0	1
SMAD4	chr18	48593530/T//C	synonymous	0	1
STARD6	chr18	51858190/C//T	nonsynonymous	0	1
KCNG2	chr18	77659375/C//T	synonymous	0	1
PTPRS	chr19	5223298/G//A	synonymous	0	1
S1PR5	chr19	10625298/C//T	synonymous	0	1
TSHZ3	chr19	31767972/C//T	synonymous	0	1
	chr19	34302791/A//G	synonymous	0	1
CBLC	chr19	45284578/C//T	synonymous	0	1
VSIG10L	chr19	51837453/C//T	nonsynonymous	0	1
KIR3DL1	chr19	55333029/A//G	nonsynonymous	0	1
KCNQ2	chr20	62038051/C//T	synonymous	0	1
AP1B1	chr22	29736694/G//A	nonsynonymous	0	1
TBL1X	chrX	9656230/G//A	synonymous	0	1
ZRSR2	chrX	15836766/G//A	nonsynonymous	0	1

SSX3	chrX	48209429/C//A	nonsynonymous	0	1
CACNA1F	chrX	49071665/G//A	nonsynonymous	0	1
CCNB3	chrX	50090672/C//T	synonymous	0	1
IGSF1	chrX	130419222/G//A	nonsynonymous	0	1
FLNA	chrX	153587681/G//A	synonymous	0	1
PRDM16	chr1	3328111/T//C	synonymous	0	1
ERICH3	chr1	75038849/T//C	nonsynonymous	0	1
ADAR	chr1	154562723/T//A	synonymous	0	1
KLF7	chr2	207945886/G//A	synonymous	0	1
CHRD	chr3	184099086/T//G	nonsynonymous	0	1
ETFDH	chr4	159620273/T//C	synonymous	0	1
F12	chr5	176831638/C//T	nonsynonymous	0	1
DPCR1	chr6	30918017/G//C	nonsynonymous	0	1
DPCR1	chr6	30918020/G//A	synonymous	0	1
RECQL4	chr8	145738082/G//A	nonsynonymous	0	1
FAM208B	chr10	5805045/T//C	synonymous	0	1
PPRC1	chr10	103900431/C//G	nonsynonymous	0	1
LRP4	chr11	46911611/T//C	nonsynonymous	0	1
CEP295	chr11	93430558/G//T	nonsynonymous	0	1
FRY	chr13	32869430/C//A	nonsynonymous	0	1
KRTAP10-8	chr21	46032704/T//C	synonymous	0	1
ARHGEF6	chrX	135750333/G//C	synonymous	0	1
MIB2	chr1	1560204/C//T	nonsynonymous	0	1
HMGCL	chr1	24134657/G//T	nonsynonymous	0	1
VANGL1	chr1	116227966/G//C	nonsynonymous	0	1
IGSF3	chr1	117156796/C//T	synonymous	0	1
HIST2H2BF	chr1	149783907/T//A	synonymous	0	1
MPZ	chr1	161275569/G//A	synonymous	0	1
GREB1	chr2	11720839/C//T	nonsynonymous	0	1
AGBL5	chr2	27282095/C//T	nonsynonymous	0	1
GALNT14	chr2	31165136/C//T	nonsynonymous	0	1
SIX3	chr2	45169522/C//T	synonymous	0	1
QARS	chr3	49139136/C//T	nonsynonymous	0	1
XRN1	chr3	142137350/ATAC//A	nonsynonymous	0	1
KIAA0232	chr4	6865370/C//T	synonymous	0	1
RAB28	chr4	13378248/T//G	nonsynonymous	0	1
ADGRL3	chr4	62936159/A//G	nonsynonymous	0	1
TDO2	chr4	156837016/G//A	nonsynonymous	0	1
IL7R	chr5	35876479/G//C	nonsynonymous	0	1
RICTOR	chr5	38945012/C//T	synonymous	0	1
IPO11	chr5	61822020/C//T	nonsynonymous	0	1
HNRNPAB	chr5	177637232/C//T	nonsynonymous	0	1
SYCP2L	chr6	10956394/A//G	synonymous	0	1
RGL2	chr6	33261072/C//T	nonsynonymous	0	1
EYS	chr6	65301401/G//A	synonymous	0	1
FAXC	chr6	99796976/G//A	synonymous	0	1
ABCB5	chr7	20721273/C//T	nonsynonymous	0	1
NACAD	chr7	45120762/A//T	nonsynonymous	0	1
GRM3	chr7	86416012/G//A	nonsynonymous	0	1
ADCK5	chr8	145603132/G//A	synonymous	0	1
C9orf170	chr9	89771645/A//G	nonsynonymous	0	1
GOLGA2	chr9	131038214/C//T	synonymous	0	1
HBB	chr11	5248295/C//A	synonymous	0	1
CHST1	chr11	45671853/C//T	synonymous	0	1
SLC22A12	chr11	64368987/G//A	synonymous	0	1
NRXN2	chr11	64480524/G//A	synonymous	0	1
STRAP	chr12	16035611/C//T	synonymous	0	1
GYS2	chr12	21692213/C//T	synonymous	0	1
TUBA1B	chr12	49522666/C//T	nonsynonymous	0	1
SOAT2	chr12	53517620/A//G	nonsynonymous	0	1
PPFIA2	chr12	81741421/A//C	nonsynonymous	0	1
SLITRK5	chr13	88328471/C//T	synonymous	0	1
IRS2	chr13	110436459/C//T	nonsynonymous	0	1
AK7	chr14	96909081/C//T	synonymous	0	1
KLC1	chr14	104123917/TG//T	nonsynonymous	0	1
CALML4	chr15	68491996/A//G	nonsynonymous	0	1
ZNF768	chr16	30537217/C//G	nonsynonymous	0	1
SLC9A5	chr16	67300126/C//G	nonsynonymous	0	1
	chr17	7121584/A//G	synonymous	0	1
TP53	chr17	7577124/C//CTT	nonsynonymous	0	1

NCOR1	chr17	16004608/C//G	synonymous	0	1
HOXB3	chr17	46627821/G//A	nonsynonymous	0	1
CD300A	chr17	72469865/C//A	synonymous	0	1
NT5C	chr17	73126588/C//T	nonsynonymous	0	1
CDH2	chr18	25582977/G//T	nonsynonymous	0	1
ST8SIA3	chr18	55024422/C//T	nonsynonymous	0	1
CHAF1A	chr19	4442261/A//G	nonsynonymous	0	1
ZNF700	chr19	12058103/C//G	nonsynonymous	0	1
TEAD2	chr19	49858459/G//A	nonsynonymous	0	1
ZNF583	chr19	56935224/G//C	nonsynonymous	0	1
ZNF835	chr19	57176313/G//A	nonsynonymous	0	1
ABHD12	chr20	25288635/G//A	synonymous	0	1
TPX2	chr20	30363766/C//A	nonsynonymous	0	1
NCOA6	chr20	33329429/G//C	nonsynonymous	0	1
MYH7B	chr20	33576054/C//A	nonsynonymous	0	1
KRTAP10-9	chr21	46047670/A//G	synonymous	0	1
DRICH1	chr22	23967083/T//C	synonymous	0	1
CSDC2	chr22	41967840/C//G	synonymous	0	1
MAMLD1	chrX	149639020/C//T	nonsynonymous	0	1
STARD7	chr2	96852993/A//G	nonsynonymous	0	1
SPSB2	chr12	6981758/T//C	nonsynonymous	0	1
MAD2L2	chr1	11735727/G//A	synonymous	0	1
KLHDC7A	chr1	18808363/T//C	synonymous	0	1
ALPL	chr1	21887209/C//A	nonsynonymous	0	1
ARID1A	chr1	27106536/G//A	nonsynonymous	0	1
CSMD2	chr1	34128578/C//T	synonymous	0	1
DENND2C	chr1	115143437/T//G	synonymous	0	1
ATP1A1	chr1	116940535/G//A	nonsynonymous	0	1
CD1C	chr1	158262140/T//C	nonsynonymous	0	1
OR10K2	chr1	158390331/C//A	nonsynonymous	0	1
LY9	chr1	160793466/C//T	synonymous	0	1
RXRG	chr1	165376098/C//A	nonsynonymous	0	1
FMO3	chr1	171077224/A//T	synonymous	0	1
PAPPA2	chr1	176564001/G//C	nonsynonymous	0	1
F13B	chr1	197031053/T//C	synonymous	0	1
ADORA1	chr1	203134525/G//A	nonsynonymous	0	1
RASSF5	chr1	206711531/G//A	nonsynonymous	0	1
	chr1	215179287/G//T	synonymous	0	1
USH2A	chr1	216262483/T//G	nonsynonymous	0	1
ESRRG	chr1	216850659/G//A	synonymous	0	1
DNAH14	chr1	225548376/T//A	synonymous	0	1
ACTN2	chr1	236917258/C//T	synonymous	0	1
RYR2	chr1	237580416/A//C	synonymous	0	1
WDR64	chr1	241964434/G//A	nonsynonymous	0	1
EFR3B	chr2	25361930/C//T	synonymous	0	1
SRBD1	chr2	45645611/A//G	synonymous	0	1
FER1L5	chr2	97327433/A//T	nonsynonymous	0	1
ZAP70	chr2	98354343/G//A	nonsynonymous	0	1
SH3RF3	chr2	110036026/C//T	nonsynonymous	0	1
MARCO	chr2	119735509/C//A	nonsynonymous	0	1
HS6ST1	chr2	129025861/G//A	nonsynonymous	0	1
LRP1B	chr2	141474353/A//C	nonsynonymous	0	1
SCN3A	chr2	166010980/C//A	nonsynonymous	0	1
XIRP2	chr2	167760150/C//G	nonsynonymous	0	1
LRP2	chr2	170148849/G//C	nonsynonymous	0	1
TTN	chr2	179411203/G//C	nonsynonymous	0	1
TTN	chr2	179517245/C//A	nonsynonymous	0	1
TTN	chr2	179604859/C//T	synonymous	0	1
CTLA4	chr2	204735510/C//T	nonsynonymous	0	1
ABCA12	chr2	215901721/T//A	nonsynonymous	0	1
OBSL1	chr2	220422558/G//A	synonymous	0	1
SNED1	chr2	242009413/C//T	nonsynonymous	0	1
CHL1	chr3	367653/C//A	nonsynonymous	0	1
CHL1	chr3	407651/T//A	nonsynonymous	0	1
TADA3	chr3	9831547/T//A	nonsynonymous	0	1
IL17RE	chr3	9957221/C//T	synonymous	0	1
KCNH8	chr3	19554722/A//T	nonsynonymous	0	1
KCNH8	chr3	19554726/G//T	nonsynonymous	0	1
UBE2E1	chr3	23932001/C//T	synonymous	0	1
KIF9	chr3	47277620/A//G	synonymous	0	1

ROBO2	chr3	77614097/C//T	synonymous	0	1
HTR1F	chr3	88040708/C//A	nonsynonymous	0	1
OR5K4	chr3	98073425/C//A	nonsynonymous	0	1
CD200R1	chr3	112647756/T//G	nonsynonymous	0	1
GPR156	chr3	119886815/G//T	nonsynonymous	0	1
EPHB1	chr3	134851558/G//A	nonsynonymous	0	1
ANKUB1	chr3	149485255/C//T	synonymous	0	1
WFS1	chr4	6302839/T//A	nonsynonymous	0	1
CC2D2A	chr4	15511774/G//A	nonsynonymous	0	1
RBPJ	chr4	26430416/A//G	synonymous	0	1
ARHGAP24	chr4	86893204/C//G	nonsynonymous	0	1
ZGRF1	chr4	113506819/C//G	nonsynonymous	0	1
ANK2	chr4	114120177/C//A	nonsynonymous	0	1
ANK2	chr4	114262959/C//A	nonsynonymous	0	1
PCDH10	chr4	134073712/A//C	nonsynonymous	0	1
POU4F2	chr4	147561590/A//G	nonsynonymous	0	1
DCLK2	chr4	151168779/C//A	synonymous	0	1
FBXW7	chr4	153245404/G//C	nonsynonymous	0	1
GALNTL6	chr4	173269747/A//C	nonsynonymous	0	1
DNAH5	chr5	13753353/T//A	nonsynonymous	0	1
NPR3	chr5	32712350/G//A	synonymous	0	1
ESM1	chr5	54275199/G//A	synonymous	0	1
IQGAP2	chr5	75950778/T//A	nonsynonymous	0	1
APC	chr5	112155032/A//T	nonsynonymous	0	1
FTMT	chr5	121187824/C//A	synonymous	0	1
PCDHB15	chr5	140626994/C//T	synonymous	0	1
PCDHGA10	chr5	140793243/C//T	synonymous	0	1
TIGD6	chr5	149375015/C//T	synonymous	0	1
MRPL22	chr5	154339607/T//A	nonsynonymous	0	1
GABRB2	chr5	160721398/T//C	nonsynonymous	0	1
DOCK2	chr5	169445988/C//A	nonsynonymous	0	1
GMDS	chr6	1742775/T//C	nonsynonymous	0	1
HLA-A	chr6	29911321/G//T	nonsynonymous	0	1
UNC5CL	chr6	41002489/C//G	nonsynonymous	0	1
DEFB113	chr6	49936451/G//A	nonsynonymous	0	1
EYS	chr6	65301875/T//G	nonsynonymous	0	1
COL12A1	chr6	75847298/G//T	synonymous	0	1
EPHA7	chr6	94120608/G//T	nonsynonymous	0	1
ASCC3	chr6	101127562/T//G	nonsynonymous	0	1
SYNE1	chr6	152473245/G//A	nonsynonymous	0	1
PLG	chr6	161155096/G//A	nonsynonymous	0	1
AMPH	chr7	38574608/G//A	nonsynonymous	0	1
ADCY1	chr7	45743024/G//A	nonsynonymous	0	1
HEPACAM2	chr7	92848743/A//G	nonsynonymous	0	1
COL1A2	chr7	94033901/G//T	nonsynonymous	0	1
FBXL13	chr7	102665681/C//T	synonymous	0	1
CADPS2	chr7	122261662/G//A	nonsynonymous	0	1
GRM8	chr7	126086408/T//G	nonsynonymous	0	1
KEL	chr7	142658568/C//T	synonymous	0	1
ZNF862	chr7	149547424/T//A	nonsynonymous	0	1
TMUB1	chr7	150778577/G//A	synonymous	0	1
AGAP3	chr7	150815406/C//T	synonymous	0	1
CSMD1	chr8	2820133/G//A	synonymous	0	1
UNC5D	chr8	35608327/G//T	nonsynonymous	0	1
SFRP1	chr8	41166478/C//A	synonymous	0	1
PRKDC	chr8	48824969/C//T	nonsynonymous	0	1
PXDNL	chr8	52505201/T//G	nonsynonymous	0	1
ATP6V1H	chr8	54628532/G//A	nonsynonymous	0	1
ASPH	chr8	62479779/T//C	synonymous	0	1
C8orf46	chr8	67417720/G//A	synonymous	0	1
FER1L6	chr8	124992839/G//A	nonsynonymous	0	1
FAM135B	chr8	139255226/A//C	nonsynonymous	0	1
CCDC166	chr8	144789984/C//T	nonsynonymous	0	1
NOL6	chr9	33472321/G//A	synonymous	0	1
RNF20	chr9	104323504/C//T	nonsynonymous	0	1
C9orf50	chr9	132382719/G//A	synonymous	0	1
COL5A1	chr9	137591774/C//A	nonsynonymous	0	1
TRAF2	chr9	139811067/G//A	synonymous	0	1
SFMBT2	chr10	7205784/C//A	nonsynonymous	0	1
ITIH2	chr10	7763613/C//T	nonsynonymous	0	1

ST8SIA6	chr10	17365142/G//A	nonsynonymous	0	1
CHAT	chr10	50822475/G//A	synonymous	0	1
	chr10	55583193/G//C	synonymous	0	1
TFAM	chr10	60154773/G//A	nonsynonymous	0	1
NOLC1	chr10	103921417/A//G	nonsynonymous	0	1
GFRA1	chr10	118029084/C//A	nonsynonymous	0	1
KNDC1	chr10	135020825/C//T	nonsynonymous	0	1
OR51L1	chr11	5021063/T//G	nonsynonymous	0	1
OR52A1	chr11	5172828/C//A	nonsynonymous	0	1
AMPD3	chr11	10503746/C//T	nonsynonymous	0	1
OR10AG1	chr11	55735262/A//C	synonymous	0	1
FAT3	chr11	92088309/C//T	nonsynonymous	0	1
MTNR1B	chr11	92715424/G//C	synonymous	0	1
DSCAML1	chr11	117307909/C//T	nonsynonymous	0	1
IGSF9B	chr11	133790944/C//T	synonymous	0	1
KRAS	chr12	25398285/C//G	nonsynonymous	0	1
LRRK2	chr12	40702389/A//T	synonymous	0	1
NELL2	chr12	45108499/G//A	synonymous	0	1
AMIGO2	chr12	47472836/C//G	synonymous	0	1
NT5DC3	chr12	104187022/G//A	synonymous	0	1
BTBD11	chr12	107914351/C//T	nonsynonymous	0	1
CUX2	chr12	111748069/G//T	nonsynonymous	0	1
KDM2B	chr12	121880605/G//A	nonsynonymous	0	1
HIP1R	chr12	123343709/C//T	nonsynonymous	0	1
FZD10	chr12	130648815/G//A	nonsynonymous	0	1
PIWIL1	chr12	130840137/G//T	nonsynonymous	0	1
SACS	chr13	23929780/T//G	nonsynonymous	0	1
CCNA1	chr13	37012228/C//T	nonsynonymous	0	1
THSD1	chr13	52952261/C//A	nonsynonymous	0	1
	chr13	78493594/A//C	synonymous	0	1
MCF2L	chr13	113742087/G//A	nonsynonymous	0	1
ATP4B	chr13	114307232/C//T	nonsynonymous	0	1
MYH7	chr14	23884312/C//T	synonymous	0	1
RTN1	chr14	60194232/G//A	synonymous	0	1
SIX6	chr14	60976504/C//G	nonsynonymous	0	1
TSHR	chr14	81609501/G//T	nonsynonymous	0	1
STON2	chr14	81862474/G//A	nonsynonymous	0	1
RYR3	chr15	33938601/C//T	nonsynonymous	0	1
OIP5	chr15	41605468/T//G	synonymous	0	1
MAP1A	chr15	43814174/G//A	nonsynonymous	0	1
SMAD3	chr15	67479795/C//T	nonsynonymous	0	1
SPESP1	chr15	69223037/G//A	synonymous	0	1
ZNF423	chr16	49671686/G//A	synonymous	0	1
PAPD5	chr16	50261799/C//T	nonsynonymous	0	1
NFAT5	chr16	69726407/G//A	synonymous	0	1
LDHD	chr16	75149198/C//T	synonymous	0	1
PKD1L2	chr16	81134713/G//A	synonymous	0	1
PAFAH1B1	chr17	2570317/C//A	nonsynonymous	0	1
TP53	chr17	7577520/A//C	nonsynonymous	0	1
ACACA	chr17	35687345/C//T	synonymous	0	1
GPR179	chr17	36486308/G//A	synonymous	0	1
TUBD1	chr17	57955630/G//A	synonymous	0	1
MGAT5B	chr17	74902155/G//A	nonsynonymous	0	1
NETO1	chr18	70417579/A//G	nonsynonymous	0	1
STXBP2	chr19	7703933/G//A	nonsynonymous	0	1
MUC16	chr19	9058790/A//T	synonymous	0	1
DOCK6	chr19	11327936/G//A	nonsynonymous	0	1
ANGPTL8	chr19	11350332/T//G	nonsynonymous	0	1
ZNF844	chr19	12186726/G//C	nonsynonymous	0	1
ZNF20	chr19	12244163/C//A	nonsynonymous	0	1
DNASE2	chr19	12986962/C//T	nonsynonymous	0	1
CACNA1A	chr19	13394176/G//A	nonsynonymous	0	1
USHBP1	chr19	17369094/T//G	nonsynonymous	0	1
MAP1S	chr19	17838795/C//T	nonsynonymous	0	1
HAPLN4	chr19	19368852/G//A	nonsynonymous	0	1
ZNF536	chr19	30934917/C//T	nonsynonymous	0	1
RHPN2	chr19	33481468/G//A	synonymous	0	1
WTIP	chr19	34973365/C//T	synonymous	0	1
LRFN3	chr19	36435648/G//A	synonymous	0	1
SELENOV	chr19	40006100/G//A	nonsynonymous	0	1

CNTD2	chr19	40729317/G//A	nonsynonymous	0	1
SPTBN4	chr19	40996147/C//T	nonsynonymous	0	1
SPHK2	chr19	49129596/G//T	nonsynonymous	0	1
NTN5	chr19	49173642/G//T	nonsynonymous	0	1
VSIG10L	chr19	51841274/C//T	nonsynonymous	0	1
ZNF611	chr19	53209907/T//C	nonsynonymous	0	1
ZNF816	chr19	53453169/G//C	nonsynonymous	0	1
VPS16	chr20	2842500/C//T	nonsynonymous	0	1
LBP	chr20	36997684/G//T	nonsynonymous	0	1
PABPC1L	chr20	43566719/C//T	nonsynonymous	0	1
	chr21	35209291/G//C	synonymous	0	1
RUNX1	chr21	36164821/C//T	nonsynonymous	0	1
DSCAM	chr21	41684059/C//A	nonsynonymous	0	1
APOBEC3G	chr22	39475008/G//A	nonsynonymous	0	1
TAB1	chr22	39813809/G//A	nonsynonymous	0	1
PKDREJ	chr22	46657020/G//C	nonsynonymous	0	1
DMD	chrX	31792079/T//A	nonsynonymous	0	1
HUWE1	chrX	53622245/C//T	synonymous	0	1
PJA1	chrX	68382826/C//A	nonsynonymous	0	1
TEX13B	chrX	107225289/C//A	nonsynonymous	0	1
KIAA1210	chrX	118220565/T//A	nonsynonymous	0	1
KIAA1210	chrX	118223438/C//T	synonymous	0	1
PANK4	chr1	2458010/G//T	nonsynonymous	1	0
GLMP	chr1	156264693/C//G	nonsynonymous	1	0
BRINP2	chr1	177199032/C//A	nonsynonymous	1	0
CACNA1S	chr1	201047035/G//A	nonsynonymous	1	0
OBSCN	chr1	228495974/G//A	nonsynonymous	1	0
ALK	chr2	29917871/C//T	nonsynonymous	1	0
RTN4	chr2	55253892/G//A	nonsynonymous	1	0
POLR1A	chr2	86332948/C//G	synonymous	1	0
THSD7B	chr2	138400105/G//T	nonsynonymous	1	0
TADA3	chr3	9821952/C//A	synonymous	1	0
TGM4	chr3	44948677/T//C	nonsynonymous	1	0
CCR3	chr3	46307474/G//A	synonymous	1	0
PRSS46	chr3	46775651/G//A	nonsynonymous	1	0
HTT	chr4	3205840/C//T	nonsynonymous	1	0
PRDM8	chr4	81123543/C//T	synonymous	1	0
TDO2	chr4	156838615/C//A	nonsynonymous	1	0
SLC36A2	chr5	150722499/T//G	synonymous	1	0
RANBP17	chr5	170610431/C//A	nonsynonymous	1	0
LOC100129636	chr6	29044208/G//A	synonymous	1	0
LST1	chr6	31555071/G//C	synonymous	1	0
CYP39A1	chr6	46563742/C//A	synonymous	1	0
	chr6	56482136/T//G	synonymous	1	0
PNISR	chr6	99848395/C//G	synonymous	1	0
TAAR5	chr6	132910335/G//A	nonsynonymous	1	0
TNRC18	chr7	5427992/T//G	nonsynonymous	1	0
THSD7A	chr7	11441461/G//A	synonymous	1	0
CHN2	chr7	29394262/C//T	nonsynonymous	1	0
PPP1R17	chr7	31746967/G//T	synonymous	1	0
PKD1L1	chr7	47944794/T//C	nonsynonymous	1	0
PEX1	chr7	92120639/A//T	nonsynonymous	1	0
DNAJC2	chr7	102964073/C//A	nonsynonymous	1	0
EFCAB10	chr7	105205609/T//TG	synonymous	1	0
ATP6V1H	chr8	54723717/T//C	synonymous	1	0
LACTB2	chr8	71581325/G//C	nonsynonymous	1	0
LAPTM4B	chr8	98831390/A//C	nonsynonymous	1	0
ANP32B	chr9	100774709/G//A	nonsynonymous	1	0
OR1Q1	chr9	125377624/G//T	nonsynonymous	1	0
MVB12B	chr9	129102902/A//G	nonsynonymous	1	0
SAPCD2	chr9	139959140/C//T	nonsynonymous	1	0
PNPLA7	chr9	140392676/G//C	synonymous	1	0
CALML5	chr10	5541148/A//G	nonsynonymous	1	0
VSTM4	chr10	50315995/A//C	nonsynonymous	1	0
ANK3	chr10	61822912/T//A	nonsynonymous	1	0
HHEX	chr10	94452138/C//A	synonymous	1	0
COX15	chr10	101478178/G//C	nonsynonymous	1	0
CPXM2	chr10	125601920/G//T	synonymous	1	0
FSHB	chr11	30255167/C//A	synonymous	1	0
OR4C46	chr11	51515417/A//T	nonsynonymous	1	0

SSRP1	chr11	57099876/A//G	synonymous	1	0
SYVN1	chr11	64898270/GCCGCTGGAA//G	nonsynonymous	1	0
SHANK2	chr11	70333519/G//C	nonsynonymous	1	0
KDM5A	chr12	420128/C//A	nonsynonymous	1	0
A2M	chr12	9262457/C//G	synonymous	1	0
LRP6	chr12	12274312/T//C	synonymous	1	0
	chr12	16757897/G//A	synonymous	1	0
OR6C70	chr12	55863565/G//A	nonsynonymous	1	0
POC1B	chr12	89918934/G//A	synonymous	1	0
DIABLO	chr12	122702952/A//G	synonymous	1	0
POLE	chr12	133202856/G//C	nonsynonymous	1	0
ANKLE2	chr12	133313503/G//T	nonsynonymous	1	0
RXFP2	chr13	32356838/T//A	nonsynonymous	1	0
SLITRK5	chr13	88329811/C//A	nonsynonymous	1	0
STARD9	chr15	42979501/C//G	nonsynonymous	1	0
TMC7	chr16	19049350/C//T	nonsynonymous	1	0
NLRC5	chr16	57056185/G//C	nonsynonymous	1	0
CNGB1	chr16	57921976/C//T	nonsynonymous	1	0
PLCG2	chr16	81891927/G//A	nonsynonymous	1	0
COASY	chr17	40717746/A//C	nonsynonymous	1	0
DHX8	chr17	41584964/G//T	nonsynonymous	1	0
EPN3	chr17	48614006/A//G	nonsynonymous	1	0
TSPAN10	chr17	79612227/C//G	synonymous	1	0
DSC1	chr18	28742522/G//A	synonymous	1	0
SETBP1	chr18	42532265/G//T	nonsynonymous	1	0
SERPINB2	chr18	61570162/C//G	nonsynonymous	1	0
DSEL	chr18	65181902/T//A	synonymous	1	0
	chr19	36001410/G//T	synonymous	1	0
SPTBN4	chr19	41063070/G//A	nonsynonymous	1	0
GYS1	chr19	49473097/G//T	nonsynonymous	1	0
ZNF813	chr19	53995084/A//C	nonsynonymous	1	0
ZNF773	chr19	58018506/A//G	nonsynonymous	1	0
ZNF417	chr19	58419841/G//T	synonymous	1	0
ZNF417	chr19	58419874/T//C	synonymous	1	0
	chr20	35414974/T//C	synonymous	1	0
NCOA5	chr20	44708043/T//C	synonymous	1	0
SALL4	chr20	50408326/G//A	synonymous	1	0
NTSR1	chr20	61391627/G//C	synonymous	1	0
C21orf59	chr21	33984548/AACCATGGCGGTCCC	nonsynonymous	1	0
TOB2	chr22	41832941/C//T	nonsynonymous	1	0
MAGEB4	chrX	30260774/C//G	nonsynonymous	1	0
CFAP47	chrX	35969974/A//G	nonsynonymous	1	0
TRPC5	chrX	111155645/T//G	nonsynonymous	1	0
BCORL1	chrX	129190186/G//A	synonymous	1	0
CPSF3L	chr1	1249718/G//A	nonsynonymous	0	1
CTPS1	chr1	41456915/G//A	nonsynonymous	0	1
ZNF678	chr1	227842469/A//T	nonsynonymous	0	1
CPOX	chr3	98312301/C//T	synonymous	0	1
CEP63	chr3	134256054/G//A	nonsynonymous	0	1
APC	chr5	112162917/CT//C	nonsynonymous	0	1
TCP11	chr6	35089892/A//G	nonsynonymous	0	1
EYS	chr6	65098712/G//A	synonymous	0	1
PTPRK	chr6	128302368/G//T	nonsynonymous	0	1
DNAH11	chr7	21784642/C//T	nonsynonymous	0	1
OR56A5	chr11	5989028/C//A	nonsynonymous	0	1
MPPED2	chr11	30439146/G//T	nonsynonymous	0	1
TRIM64C	chr11	49078797/T//G	nonsynonymous	0	1
FAT3	chr11	92533838/C//T	synonymous	0	1
CEP57	chr11	95546241/G//A	synonymous	0	1
USP5	chr12	6966014/C//T	nonsynonymous	0	1
ARHGEF7	chr13	111767996/C//T	synonymous	0	1
SYNE2	chr14	64469455/A//G	nonsynonymous	0	1
DYNC1H1	chr14	102476243/T//C	nonsynonymous	0	1
EXOC3L4	chr14	103574836/G//A	nonsynonymous	0	1
RUNDC3A	chr17	42390005/G//A	synonymous	0	1
MRPL38	chr17	73895028/G//A	nonsynonymous	0	1
TNFAIP8L1	chr19	4652078/T//C	nonsynonymous	0	1
TSPAN16	chr19	11409609/T//C	nonsynonymous	0	1
NAPA	chr19	47995335/G//A	synonymous	0	1
SIGLEC6	chr19	52033213/G//C	synonymous	0	1

CTSS	chr1	150724280/C//T	nonsynonymous	0	1
VANGL2	chr1	160385686/C//T	nonsynonymous	0	1
SLC26A9	chr1	205895684/C//T	nonsynonymous	0	1
OBSCN	chr1	228482667/G//T	nonsynonymous	0	1
APOB	chr2	21224728/G//A	synonymous	0	1
FEZ2	chr2	36818146/T//A	nonsynonymous	0	1
SATB2	chr2	200137235/T//C	nonsynonymous	0	1
NRP2	chr2	206659485/C//T	synonymous	0	1
CPS1	chr2	211525284/G//T	nonsynonymous	0	1
	chr3	97367184/A//G	synonymous	0	1
CHST2	chr3	142840545/G//A	nonsynonymous	0	1
SHOX2	chr3	157816005/G//C	synonymous	0	1
TRIO	chr5	14358394/G//C	nonsynonymous	0	1
TRERF1	chr6	42227245/G//C	nonsynonymous	0	1
BCLAF1	chr6	136599399/G//T	nonsynonymous	0	1
EPHB6	chr7	142564330/C//T	synonymous	0	1
EFCAB1	chr8	49647736/C//T	synonymous	0	1
MTDH	chr8	98673327/G//C	nonsynonymous	0	1
KANK1	chr9	712620/A//C	synonymous	0	1
CRAT	chr9	131857899/C//T	synonymous	0	1
GOT1	chr10	101163281/G//A	nonsynonymous	0	1
	chr11	31824419/G//A	synonymous	0	1
SMTNL1	chr11	57310324/C//T	nonsynonymous	0	1
PTPRQ	chr12	81046548/T//A	nonsynonymous	0	1
ING1	chr13	111367738/T//G	synonymous	0	1
BFAR	chr16	14748998/C//T	synonymous	0	1
NCOR1	chr17	15971361/G//C	nonsynonymous	0	1
ADAP2	chr17	29283470/C//T	nonsynonymous	0	1
PTPRM	chr18	7906587/T//C	synonymous	0	1
ELAVL1	chr19	8038751/A//C	synonymous	0	1
KLC3	chr19	45848934/TG//T	nonsynonymous	0	1
ZXDB	chrX	57618472/G//C	synonymous	0	1
IRS4	chrX	107978461/G//A	nonsynonymous	0	1
SPANXN1	chrX	144337254/G//A	nonsynonymous	0	1
MROH9	chr1	170955772/C//G	nonsynonymous	0	1
PXDN	chr2	1667376/C//A	nonsynonymous	0	1
TTC31	chr2	74710237/A//G	synonymous	0	1
UBR3	chr2	170937194/G//C	nonsynonymous	0	1
KBTBD12	chr3	127641985/A//G	synonymous	0	1
KLHL5	chr4	39098384/A//G	nonsynonymous	0	1
DDX39B	chr6	31508245/G//A	nonsynonymous	0	1
TOMM6	chr6	41755403/T//C	synonymous	0	1
ASZ1	chr7	117023052/T//C	nonsynonymous	0	1
ERI1	chr8	8860616/G//A	nonsynonymous	0	1
WWP1	chr8	87439967/G//A	nonsynonymous	0	1
RAD54B	chr8	95412698/G//A	synonymous	0	1
IFNA14	chr9	21239700/C//T	nonsynonymous	0	1
GRIN3A	chr9	104500150/A//G	nonsynonymous	0	1
CBL	chr11	119156088/C//T	nonsynonymous	0	1
CHD3	chr17	7806315/A//G	nonsynonymous	0	1
AP2B1	chr17	33998903/A//T	nonsynonymous	0	1
ADARB1	chr21	46645524/G//A	synonymous	0	1
FOXD3	chr1	63789981/C//T	nonsynonymous	0	1
TTN	chr2	179582010/G//T	nonsynonymous	0	1
TOP2B	chr3	25705730/A//AC	nonsynonymous	0	1
ST3GAL6	chr3	98503879/A//G	nonsynonymous	0	1
ADAM2	chr8	39679151/C//A	nonsynonymous	0	1
ZNF408	chr11	46724304/T//C	synonymous	0	1
CSRP2	chr12	77257081/G//A	synonymous	0	1
PTGER2	chr14	52781651/G//T	nonsynonymous	0	1
NOXRED1	chr14	77860856/C//A	synonymous	0	1
LOXHD1	chr18	44172522/A//G	nonsynonymous	0	1
MRO	chr18	48325712/G//A	synonymous	0	1
SLC2A7	chr1	9063367/A//G	nonsynonymous	1	0
	chr1	10364222/C//T	synonymous	1	0
TESK2	chr1	45813330/G//A	nonsynonymous	1	0
COL11A1	chr1	103428215/A//G	synonymous	1	0
TRIM33	chr1	114968100/G//C	nonsynonymous	1	0
TOR1AIP1	chr1	179887116/T//C	synonymous	1	0
CFHR2	chr1	196928171/G//C	nonsynonymous	1	0

KIF14	chr1	200586864/C//T	nonsynonymous	1	0
THUMPD2	chr2	39995585/G//T	nonsynonymous	1	0
LRP1B	chr2	141812688/T//TGCATGACCC	nonsynonymous	1	0
ZEB2	chr2	145157244/T//C	nonsynonymous	1	0
ABCA12	chr2	215862491/C//G	nonsynonymous	1	0
NBEAL2	chr3	47048739/G//C	synonymous	1	0
FAT1	chr4	187542871/T//G	nonsynonymous	1	0
RP1	chr8	55541514/C//G	nonsynonymous	1	0
CCDC166	chr8	144790077/C//T	nonsynonymous	1	0
MURC	chr9	103340578/G//A	synonymous	1	0
KYAT1	chr9	131597934/C//T	nonsynonymous	1	0
ABCA2	chr9	139909224/G//A	synonymous	1	0
LRRC27	chr10	134165210/G//A	synonymous	1	0
OR52A1	chr11	5172757/G//A	synonymous	1	0
DGKZ	chr11	46388098/A//G	nonsynonymous	1	0
VWCE	chr11	61026214/G//T	nonsynonymous	1	0
LTBP3	chr11	65314008/G//T	nonsynonymous	1	0
SPTBN2	chr11	66472646/G//C	nonsynonymous	1	0
FCHSD2	chr11	72794733/A//T	synonymous	1	0
TRIM77	chr11	89443791/A//T	nonsynonymous	1	0
	chr11	131240661/G//A	synonymous	1	0
VWF	chr12	6132880/C//A	nonsynonymous	1	0
C3AR1	chr12	8211947/C//T	nonsynonymous	1	0
PABPC3	chr13	25671106/A//G	nonsynonymous	1	0
FOXA1	chr14	38061458/C//T	synonymous	1	0
DRC7	chr16	57735938/G//A	nonsynonymous	1	0
TP53	chr17	7579439/GC//G	nonsynonymous	1	0
ALOX12B	chr17	7976064/G//A	synonymous	1	0
QRICH2	chr17	74288961/T//A	nonsynonymous	1	0
	chr18	690748/G//T	synonymous	1	0
ANKRD24	chr19	4217339/A//C	synonymous	1	0
FEM1A	chr19	4793055/G//A	nonsynonymous	1	0
CCNE1	chr19	30311669/C//T	nonsynonymous	1	0
SIGLEC10	chr19	51920166/G//T	nonsynonymous	1	0
ZNF816	chr19	53453811/T//C	nonsynonymous	1	0
LILRB2	chr19	54778533/C//T	synonymous	1	0
DNTTIP1	chr20	44424012/G//A	nonsynonymous	1	0
KRTAP10-6	chr21	46012279/G//A	synonymous	1	0
CNTN2	chr1	205042928/G//A	synonymous	1	0
HLA-C	chr6	31239006/G//T	nonsynonymous	1	0
ZNF92	chr7	64864109/T//G	nonsynonymous	1	0
BRCA1	chr17	41228556/T//C	nonsynonymous	1	0
DDX42	chr17	61886190/A//G	nonsynonymous	1	0
FCRL3	chr1	157650499/T//G	nonsynonymous	0	1
FMO3	chr1	171083389/T//G	nonsynonymous	0	1
DENND1B	chr1	197480861/A//G	synonymous	0	1
ZNF281	chr1	200376637/G//A	nonsynonymous	0	1
HSD11B1	chr1	209879232/G//A	synonymous	0	1
ZP4	chr1	238054027/A//C	synonymous	0	1
FMN2	chr1	240371923/T//C	synonymous	0	1
FMN2	chr1	240497208/T//C	nonsynonymous	0	1
KIF26B	chr1	245851399/G//A	nonsynonymous	0	1
HEATR5B	chr2	37255878/C//G	nonsynonymous	0	1
DHX57	chr2	39088395/C//T	nonsynonymous	0	1
PSME4	chr2	54159145/A//C	synonymous	0	1
NEB	chr2	152423720/C//A	nonsynonymous	0	1
	chr2	166020347/A//G	synonymous	0	1
SCN1A	chr2	166848771/T//G	nonsynonymous	0	1
	chr2	179610825/T//G	synonymous	0	1
SP140	chr2	231112630/G//C	nonsynonymous	0	1
	chr3	384687/A//G	synonymous	0	1
PSMD6	chr3	64008042/G//C	synonymous	0	1
PRICKLE2	chr3	64148769/C//G	nonsynonymous	0	1
CPA3	chr3	148601406/C//A	nonsynonymous	0	1
SOX2	chr3	181430210/GCGGCGGCAACTCC	nonsynonymous	0	1
GABRB1	chr4	47163459/C//A	nonsynonymous	0	1
WDFY3	chr4	85715820/A//T	synonymous	0	1
ADGRV1	chr5	89948189/G//C	nonsynonymous	0	1
SH3TC2	chr5	148431729/A//G	nonsynonymous	0	1
FAM71B	chr5	156590428/G//T	nonsynonymous	0	1

RNF145	chr5	158585737/C//A	nonsynonymous	0	1
TDRD6	chr6	46656182/C//T	nonsynonymous	0	1
DNAH11	chr7	21599376/T//C	nonsynonymous	0	1
HECW1	chr7	43485066/C//T	synonymous	0	1
DGKI	chr7	137170128/G//T	nonsynonymous	0	1
SVOPL	chr7	138281172/C//T	nonsynonymous	0	1
SSPO	chr7	149503904/C//T	nonsynonymous	0	1
BLK	chr8	11412370/G//A	synonymous	0	1
PXDNL	chr8	52287285/G//A	synonymous	0	1
CSPP1	chr8	67998281/A//G	nonsynonymous	0	1
ZFHX4	chr8	77766282/C//T	synonymous	0	1
RIMS2	chr8	104955133/A//G	nonsynonymous	0	1
VLDLR	chr9	2647544/C//T	nonsynonymous	0	1
WDFY4	chr10	50165260/C//T	synonymous	0	1
DMBT1	chr10	124353101/G//A	synonymous	0	1
DCDC1	chr11	31349702/T//C	synonymous	0	1
DDB2	chr11	47238556/C//A	nonsynonymous	0	1
OR4C46	chr11	51515421/T//G	nonsynonymous	0	1
AHNAK	chr11	62287766/T//C	nonsynonymous	0	1
KLRB1	chr12	9747961/G//C	nonsynonymous	0	1
LDHB	chr12	21795029/A//C	nonsynonymous	0	1
MPHOSPH9	chr12	123641410/A//T	nonsynonymous	0	1
GTF3A	chr13	27998802/G//A	synonymous	0	1
MAB21L1	chr13	36049417/G//T	synonymous	0	1
NBEA	chr13	36202224/G//C	nonsynonymous	0	1
OR4K15	chr14	20443837/A//C	nonsynonymous	0	1
ADAM21	chr14	70924301/T//A	nonsynonymous	0	1
SPRED1	chr15	38643710/G//C	nonsynonymous	0	1
C15orf52	chr15	40627653/A//G	synonymous	0	1
HDC	chr15	50535210/G//T	synonymous	0	1
GP2	chr16	20330949/T//A	nonsynonymous	0	1
SRCAP	chr16	30720956/C//G	nonsynonymous	0	1
CES1	chr16	55854390/A//C	nonsynonymous	0	1
JUP	chr17	39912005/C//A	nonsynonymous	0	1
MRC2	chr17	60749511/C//G	nonsynonymous	0	1
RYR1	chr19	39052022/C//T	synonymous	0	1
NLRP5	chr19	56561901/G//C	synonymous	0	1
TASP1	chr20	13604101/C//T	nonsynonymous	0	1
PTPRT	chr20	41400106/T//G	nonsynonymous	0	1
SEMG2	chr20	43851235/A//C	nonsynonymous	0	1
PCBP3	chr21	47269966/T//C	synonymous	0	1
TSSK2	chr22	19119970/T//C	nonsynonymous	0	1
SFI1	chr22	32012989/G//C	nonsynonymous	0	1
PPEF1	chrX	18751900/A//C	nonsynonymous	0	1
KDM6A	chrX	44918338/G//A	nonsynonymous	0	1
GRIPAP1	chrX	48849996/C//G	synonymous	0	1
TRO	chrX	54953538/A//C	nonsynonymous	0	1
AWAT1	chrX	69458119/A//G	nonsynonymous	0	1
LRCH2	chrX	114361445/A//C	nonsynonymous	0	1
TENM1	chrX	124029830/C//A	nonsynonymous	0	1
ADGRG4	chrX	135470021/T//A	synonymous	0	1
ARHGEF6	chrX	135750147/G//C	synonymous	0	1
PRKCZ	chr1	2116433/C//T	synonymous	0	1
CASZ1	chr1	10720214/C//T	synonymous	0	1
CROCC	chr1	17257875/G//A	synonymous	0	1
CSMD2	chr1	34192255/G//A	synonymous	0	1
PDE4B	chr1	66723359/T//A	nonsynonymous	0	1
ANP32E	chr1	150208146/G//T	synonymous	0	1
DENND4B	chr1	153909208/T//C	nonsynonymous	0	1
OR6Y1	chr1	158517832/A//T	nonsynonymous	0	1
OR10X1	chr1	158549540/G//C	synonymous	0	1
VANGL2	chr1	160388931/C//T	nonsynonymous	0	1
TNN	chr1	175066648/G//A	nonsynonymous	0	1
TNR	chr1	175375396/C//T	nonsynonymous	0	1
RYR2	chr1	237666611/C//T	nonsynonymous	0	1
SOX11	chr2	5833483/C//T	synonymous	0	1
TEX37	chr2	88829078/A//G	synonymous	0	1
CCDC93	chr2	118732437/C//T	nonsynonymous	0	1
MGAT5	chr2	135119934/G//C	nonsynonymous	0	1
TTN	chr2	179549431/G//T	nonsynonymous	0	1

ZNF804A	chr2	185800753/C//A	nonsynonymous	0	1
TMEFF2	chr2	192818466/C//T	nonsynonymous	0	1
GPR55	chr2	231774771/G//A	nonsynonymous	0	1
B3GNT7	chr2	232263023/G//A	nonsynonymous	0	1
ITPR1	chr3	4722294/C//T	nonsynonymous	0	1
DOCK3	chr3	51265479/C//A	nonsynonymous	0	1
TF	chr3	133478162/G//A	nonsynonymous	0	1
NWD2	chr4	37447889/G//A	nonsynonymous	0	1
PDGFRA	chr4	55144540/A//G	nonsynonymous	0	1
TRPC3	chr4	122854128/G//A	synonymous	0	1
ABCE1	chr4	146030403/T//C	nonsynonymous	0	1
FAT1	chr4	187540638/G//C	nonsynonymous	0	1
HTR1A	chr5	63257077/A//T	nonsynonymous	0	1
MEGF10	chr5	126792936/C//T	nonsynonymous	0	1
ARHGAP26	chr5	142283133/C//A	nonsynonymous	0	1
FAM193B	chr5	176952051/C//G	nonsynonymous	0	1
CAP2	chr6	17507405/C//T	synonymous	0	1
DDX39B	chr6	31498649/C//T	nonsynonymous	0	1
PNPLA1	chr6	36262156/T//A	nonsynonymous	0	1
SRSF12	chr6	89808392/A//G	nonsynonymous	0	1
ANKRD6	chr6	90333675/A//T	nonsynonymous	0	1
ARMC2	chr6	109258205/C//G	synonymous	0	1
SYNE1	chr6	152804279/C//T	nonsynonymous	0	1
ARID1B	chr6	157528716/G//A	synonymous	0	1
THBS2	chr6	169620303/G//A	synonymous	0	1
SOSTDC1	chr7	16505207/T//C	synonymous	0	1
PDE1C	chr7	31855647/C//T	synonymous	0	1
AEBP1	chr7	44149705/C//T	synonymous	0	1
ZNF736	chr7	63796713/G//A	nonsynonymous	0	1
TBL2	chr7	72985185/G//A	synonymous	0	1
POR	chr7	75583397/G//A	synonymous	0	1
MAGI2	chr7	77708256/A//G	synonymous	0	1
AKAP9	chr7	91708438/A//G	nonsynonymous	0	1
SGCE	chr7	94285433/G//A	synonymous	0	1
PEG10	chr7	94293791/G//T	nonsynonymous	0	1
GPR22	chr7	107114494/T//C	synonymous	0	1
KRBA1	chr7	149430564/C//T	nonsynonymous	0	1
CYHR1	chr8	145689968/G//A	synonymous	0	1
FREM1	chr9	14868828/C//A	nonsynonymous	0	1
BICD2	chr9	95480947/C//A	nonsynonymous	0	1
COL15A1	chr9	101802811/C//T	synonymous	0	1
ZNF462	chr9	109686504/C//A	nonsynonymous	0	1
GLE1	chr9	131277825/G//A	synonymous	0	1
OLFM1	chr9	137998685/C//G	nonsynonymous	0	1
GJD4	chr10	35897459/G//A	nonsynonymous	0	1
HBG2	chr11	5274533/T//G	nonsynonymous	0	1
MRGPRX1	chr11	18955511/A//G	nonsynonymous	0	1
OR4S1	chr11	48327866/T//A	nonsynonymous	0	1
CDC42BPG	chr11	64602033/G//A	nonsynonymous	0	1
ZFPL1	chr11	64855507/G//A	nonsynonymous	0	1
UBASH3B	chr11	122526634/C//A	synonymous	0	1
EI24	chr11	125453595/A//T	synonymous	0	1
CHD4	chr12	6691871/G//C	nonsynonymous	0	1
EPS8	chr12	15823847/C//T	synonymous	0	1
LRRK2	chr12	40728922/C//A	nonsynonymous	0	1
COPZ1	chr12	54737046/C//G	nonsynonymous	0	1
TRHDE	chr12	73046878/C//A	nonsynonymous	0	1
CMKLR1	chr12	108685821/C//T	nonsynonymous	0	1
SRRM4	chr12	119568586/C//T	nonsynonymous	0	1
SOHLH2	chr13	36767830/A//G	synonymous	0	1
HTR2A	chr13	47409051/A//G	nonsynonymous	0	1
TBC1D4	chr13	75898454/G//T	nonsynonymous	0	1
SLITRK6	chr13	86368219/T//A	nonsynonymous	0	1
SLITRK6	chr13	86369344/G//A	nonsynonymous	0	1
IRS2	chr13	110435221/T//C	synonymous	0	1
SOX1	chr13	112722295/G//A	nonsynonymous	0	1
POTEG	chr14	19553556/G//A	nonsynonymous	0	1
LRP10	chr14	23346314/C//T	nonsynonymous	0	1
DLGAP5	chr14	55655725/C//A	nonsynonymous	0	1
C14orf39	chr14	60951662/T//G	synonymous	0	1

EIF2B2	chr14	75473387/C//G	synonymous	0	1
NRXN3	chr14	80158612/G//A	synonymous	0	1
PKM	chr15	72492851/C//T	nonsynonymous	0	1
ADAMTSL3	chr15	84582090/G//A	synonymous	0	1
CLUAP1	chr16	3556415/G//A	nonsynonymous	0	1
GSPT1	chr16	11971412/A//G	nonsynonymous	0	1
PAPD5	chr16	50186916/C//A	nonsynonymous	0	1
SPNS2	chr17	4436706/G//A	synonymous	0	1
CSHL1	chr17	61988667/C//T	synonymous	0	1
ABCA8	chr17	66915620/G//T	synonymous	0	1
DNAH17	chr17	76475198/G//C	nonsynonymous	0	1
BAHCC1	chr17	79426527/C//T	synonymous	0	1
CBLN2	chr18	70209248/G//A	nonsynonymous	0	1
HDGFRP2	chr19	4494222/G//A	nonsynonymous	0	1
SAFB	chr19	5661648/G//A	nonsynonymous	0	1
TSHZ3	chr19	31768675/C//T	nonsynonymous	0	1
PSMC4	chr19	40478074/G//A	nonsynonymous	0	1
PSG1	chr19	43372462/C//T	nonsynonymous	0	1
HAO1	chr20	7864263/G//T	nonsynonymous	0	1
HCK	chr20	30681743/T//C	synonymous	0	1
SLC32A1	chr20	37357130/T//A	nonsynonymous	0	1
GNAS	chr20	57429128/G//A	nonsynonymous	0	1
CDH4	chr20	60448790/A//G	nonsynonymous	0	1
DIP2A	chr21	47957204/G//A	synonymous	0	1
MKL1	chr22	40816477/C//T	nonsynonymous	0	1
TRABD	chr22	50632768/G//C	nonsynonymous	0	1
SCML1	chrX	17768154/T//G	nonsynonymous	0	1
SMS	chrX	21997083/AGG//A	nonsynonymous	0	1
MTRNR2L10	chrX	55208360/C//T	nonsynonymous	0	1
CAPN6	chrX	110496310/G//A	synonymous	0	1
MAP7D3	chrX	135313132/A//G	synonymous	0	1
GNL1	chr6	30524043/CCG//C	synonymous	1	0
ATXN3	chr14	92537354/C//G	nonsynonymous	1	0
ZNF726	chr19	24115797/A//T	synonymous	1	0
KPTN	chr19	47986463/A//G	nonsynonymous	1	0
ZBTB17	chr1	16272253/A//T	synonymous	1	0
PATJ	chr1	62483535/A//G	nonsynonymous	1	0
NTNG1	chr1	107867537/C//T	nonsynonymous	1	0
ATP1A2	chr1	160106108/G//A	synonymous	1	0
CCDC142	chr2	74702189/A//AG	nonsynonymous	1	0
TMEM131	chr2	98412846/T//C	nonsynonymous	1	0
KIF5C	chr2	149806950/C//A	synonymous	1	0
NFE2L2	chr2	178098965/T//A	nonsynonymous	1	0
TTN	chr2	179569463/C//G	nonsynonymous	1	0
SATB2	chr2	200320707/G//A	synonymous	1	0
FN1	chr2	216271962/G//C	synonymous	1	0
PER2	chr2	239186564/G//A	nonsynonymous	1	0
IL17RD	chr3	57139962/T//A	nonsynonymous	1	0
ATP13A5	chr3	193039582/G//T	synonymous	1	0
CPN2	chr3	194062814/A//G	synonymous	1	0
TNIP2	chr4	2743855/T//C	synonymous	1	0
KIT	chr4	55593602/G//A	synonymous	1	0
SELENOP	chr5	42808417/G//A	synonymous	1	0
MAST4	chr5	66460411/G//A	nonsynonymous	1	0
CDK7	chr5	68531284/A//G	synonymous	1	0
GRIA1	chr5	153144132/G//A	synonymous	1	0
GFPT2	chr5	179745866/G//C	synonymous	1	0
TUBB2A	chr6	3155948/G//T	nonsynonymous	1	0
FAM83B	chr6	54735002/ATGAATGGACATT//A	synonymous	1	0
DST	chr6	56433383/T//G	nonsynonymous	1	0
LGSN	chr6	63990729/G//C	nonsynonymous	1	0
BACH2	chr6	90642375/A//C	nonsynonymous	1	0
SOGA3	chr6	127797356/G//A	synonymous	1	0
MACC1	chr7	20197843/A//G	nonsynonymous	1	0
SLC12A9	chr7	100457527/A//G	nonsynonymous	1	0
WDR34	chr9	131397011/G//C	nonsynonymous	1	0
SEC31B	chr10	102255156/G//A	nonsynonymous	1	0
C10orf95	chr10	104210700/C//CG	nonsynonymous	1	0
CTBP2	chr10	126686672/G//A	synonymous	1	0
	chr11	3062265/C//T	synonymous	1	0

OVCH2	chr11	7718314/C//T	nonsynonymous	1	0
LDHA	chr11	18421044/C//A	nonsynonymous	1	0
KCNA4	chr11	30033399/A//C	nonsynonymous	1	0
DNM1L	chr12	32895531/A//G	nonsynonymous	1	0
SLC38A4	chr12	47186907/TA//T	synonymous	1	0
XPOT	chr12	64803758/C//T	synonymous	1	0
HECTD4	chr12	112613591/C//T	nonsynonymous	1	0
NYNRIN	chr14	24884321/C//G	synonymous	1	0
AHNAK2	chr14	105415279/T//C	nonsynonymous	1	0
AHNAK2	chr14	105415281/G//C	synonymous	1	0
RYR3	chr15	34150071/G//A	nonsynonymous	1	0
PKM	chr15	72494854/G//A	synonymous	1	0
SAXO2	chr15	82574607/C//A	nonsynonymous	1	0
IGF1R	chr15	99500580/C//T	nonsynonymous	1	0
UNKL	chr16	1420252/C//T	synonymous	1	0
ZC3H7A	chr16	11845315/T//C	nonsynonymous	1	0
DHX38	chr16	72142239/C//G	nonsynonymous	1	0
GAS8-AS1	chr16	90095838/A//G	synonymous	1	0
TLCD1	chr17	27051637/A//T	nonsynonymous	1	0
BRCA1	chr17	41209153/C//G	nonsynonymous	1	0
MMD	chr17	53488660/G//C	nonsynonymous	1	0
MTCL1	chr18	8784096/G//A	nonsynonymous	1	0
MUC16	chr19	9063751/C//T	nonsynonymous	1	0
KRI1	chr19	10665806/A//C	nonsynonymous	1	0
PLPPR2	chr19	11475288/G//C	synonymous	1	0
ZNF526	chr19	42729030/C//T	nonsynonymous	1	0
ADAM33	chr20	3662619/G//A	synonymous	1	0
NINL	chr20	25434140/G//A	nonsynonymous	1	0
ZNF337	chr20	25655979/T//C	nonsynonymous	1	0
MXRA5	chrX	3239617/G//A	nonsynonymous	1	0
KLHL34	chrX	21675450/A//T	nonsynonymous	1	0
DUSP9	chrX	152915467/G//A	nonsynonymous	1	0
AGMAT	chr1	15911364/G//A	synonymous	1	0
CLSPN	chr1	36212457/G//C	nonsynonymous	1	0
CSF3R	chr1	36937740/G//T	nonsynonymous	1	0
HPCAL4	chr1	40148316/C//T	synonymous	1	0
CACHD1	chr1	65117915/A//T	nonsynonymous	1	0
LRRC7	chr1	70478661/G//A	nonsynonymous	1	0
NEXN	chr1	78401558/G//C	synonymous	1	0
BTBD8	chr1	92573536/G//T	nonsynonymous	1	0
PALMD	chr1	100155068/A//T	nonsynonymous	1	0
TSHB	chr1	115576615/C//A	nonsynonymous	1	0
TARS2	chr1	150468996/G//A	synonymous	1	0
NPR1	chr1	153655941/C//A	nonsynonymous	1	0
TPM3	chr1	154143148/T//C	nonsynonymous	1	0
FCGR3A	chr1	161514606/A//C	nonsynonymous	1	0
RGS8	chr1	182641960/G//C	synonymous	1	0
LAMC2	chr1	183197751/C//T	nonsynonymous	1	0
TPR	chr1	186324573/T//C	nonsynonymous	1	0
PLEKHA6	chr1	204210820/G//C	synonymous	1	0
VN1R5	chr1	247419973/T//C	synonymous	1	0
YWHAQ	chr2	9770300/G//A	synonymous	1	0
GREB1	chr2	11728987/C//T	synonymous	1	0
WDCP	chr2	24262091/G//C	nonsynonymous	1	0
ERLEC1	chr2	54028705/G//A	nonsynonymous	1	0
TGFA	chr2	70680399/C//T	synonymous	1	0
DCTN1	chr2	74605138/G//A	nonsynonymous	1	0
CTNNA2	chr2	80136827/C//T	synonymous	1	0
ARID5A	chr2	97216870/A//G	nonsynonymous	1	0
UGGT1	chr2	128903374/C//G	synonymous	1	0
XIRP2	chr2	168104191/G//C	nonsynonymous	1	0
GORASP2	chr2	171807839/C//G	nonsynonymous	1	0
GPR155	chr2	175346374/T//C	nonsynonymous	1	0
NFE2L2	chr2	178098973/C//G	nonsynonymous	1	0
TTN	chr2	179406036/T//A	nonsynonymous	1	0
COL3A1	chr2	189851860/C//A	nonsynonymous	1	0
HIBCH	chr2	191161620/C//T	synonymous	1	0
SMARCAL1	chr2	217332742/C//T	synonymous	1	0
CFAP65	chr2	219892540/C//A	nonsynonymous	1	0
SPEG	chr2	220346397/T//G	nonsynonymous	1	0

OBSL1	chr2	220422286/G//T	nonsynonymous	1	0
ACSL3	chr2	223781119/T//A	nonsynonymous	1	0
COL4A3	chr2	228155479/G//T	nonsynonymous	1	0
DAW1	chr2	228786132/A//T	nonsynonymous	1	0
SP140L	chr2	231191947/C//G	synonymous	1	0
ACKR3	chr2	237489344/C//T	nonsynonymous	1	0
PASK	chr2	242082415/C//T	synonymous	1	0
ZCWPW2	chr3	28454717/A//G	nonsynonymous	1	0
HHATL	chr3	42734298/T//A	nonsynonymous	1	0
SMARCC1	chr3	47703896/T//C	nonsynonymous	1	0
ZNF589	chr3	48310279/C//G	synonymous	1	0
IMPDH2	chr3	49065918/C//G	synonymous	1	0
OR5AC2	chr3	97806690/T//C	nonsynonymous	1	0
TMEM45A	chr3	100287738/C//G	nonsynonymous	1	0
ADGRG7	chr3	100387900/G//C	nonsynonymous	1	0
MYH15	chr3	108163486/C//A	nonsynonymous	1	0
POGLUT1	chr3	119209542/C//G	synonymous	1	0
MAATS1	chr3	119456274/G//T	nonsynonymous	1	0
LRRC58	chr3	120050193/G//A	nonsynonymous	1	0
HCLS1	chr3	121351997/G//A	nonsynonymous	1	0
LINC01565	chr3	128292561/C//T	synonymous	1	0
	chr3	132086560/T//C	synonymous	1	0
TF	chr3	133467323/G//A	synonymous	1	0
BCHE	chr3	165548790/C//G	nonsynonymous	1	0
NAALADL2	chr3	175184961/G//A	nonsynonymous	1	0
PIK3CA	chr3	178952074/G//C	nonsynonymous	1	0
CHRD	chr3	184104472/G//A	nonsynonymous	1	0
IQCG	chr3	197616555/G//A	nonsynonymous	1	0
ZNF595	chr4	87169/T//A	nonsynonymous	1	0
TXK	chr4	48106911/GA//G	synonymous	1	0
UGT2B4	chr4	70355242/A//C	nonsynonymous	1	0
SULT1B1	chr4	70592755/C//G	synonymous	1	0
CABS1	chr4	71200803/A//C	nonsynonymous	1	0
SEC31A	chr4	83765578/G//GAC	nonsynonymous	1	0
RAP1GDS1	chr4	99363214/A//T	nonsynonymous	1	0
ZGRF1	chr4	113509055/T//C	nonsynonymous	1	0
PRDM5	chr4	121737672/G//T	nonsynonymous	1	0
HMGB2	chr4	174254661/T//C	nonsynonymous	1	0
ADAM29	chr4	175898400/G//T	nonsynonymous	1	0
SLC25A4	chr4	186066254/G//A	nonsynonymous	1	0
GHR	chr5	42719379/G//T	nonsynonymous	1	0
IQGAP2	chr5	75699191/G//C	synonymous	1	0
FTMT	chr5	121187819/C//G	nonsynonymous	1	0
RAD50	chr5	131925511/C//G	nonsynonymous	1	0
PCDHB9	chr5	140567805/G//T	nonsynonymous	1	0
FAT2	chr5	150911499/CCGGCA//C	nonsynonymous	1	0
CYFIP2	chr5	156787289/G//T	nonsynonymous	1	0
CDHR2	chr5	176005447/C//G	synonymous	1	0
PPP1R3G	chr6	5086563/G//T	nonsynonymous	1	0
SYCP2L	chr6	10894436/A//G	nonsynonymous	1	0
ZNF184	chr6	27419657/G//T	nonsynonymous	1	0
TAP2	chr6	32800441/C//G	nonsynonymous	1	0
UHRF1BP1	chr6	34827325/G//T	nonsynonymous	1	0
SLC25A27	chr6	46620956/G//T	nonsynonymous	1	0
COL9A1	chr6	71004216/A//T	nonsynonymous	1	0
PNRC1	chr6	89793532/C//G	nonsynonymous	1	0
HSF2	chr6	122743351/C//T	synonymous	1	0
ARFGEF3	chr6	138584734/C//T	nonsynonymous	1	0
STXBP5	chr6	147684656/G//A	nonsynonymous	1	0
SYNE1	chr6	152737903/G//A	nonsynonymous	1	0
IGF2R	chr6	160482960/G//A	synonymous	1	0
STK31	chr7	23871758/G//A	nonsynonymous	1	0
STK17A	chr7	43622953/C//G	synonymous	1	0
IKZF1	chr7	50467949/A//G	nonsynonymous	1	0
CACNA2D1	chr7	81689745/G//A	nonsynonymous	1	0
PCLO	chr7	82584446/A//G	synonymous	1	0
SEMA3E	chr7	83022001/G//C	nonsynonymous	1	0
DYNC1I1	chr7	95614255/G//C	nonsynonymous	1	0
PCOLCE-AS1	chr7	100187294/G//T	synonymous	1	0
MUC12	chr7	100635220/C//T	nonsynonymous	1	0

PMPCB	chr7	102949402/C//T	nonsynonymous	1	0
TMEM209	chr7	129841684/C//T	synonymous	1	0
TRPV5	chr7	142625311/G//A	nonsynonymous	1	0
PRSS55	chr8	10383256/A//G	synonymous	1	0
C8orf86	chr8	38386148/G//T	nonsynonymous	1	0
C8orf86	chr8	38386158/C//G	synonymous	1	0
RP1	chr8	55542442/G//C	nonsynonymous	1	0
	chr8	93107453/A//T	synonymous	1	0
PKHD1L1	chr8	110441625/G//A	synonymous	1	0
CSMD3	chr8	113516053/A//G	synonymous	1	0
FRMPD1	chr9	37745224/A//C	nonsynonymous	1	0
TRPM3	chr9	73736147/T//C	nonsynonymous	1	0
RASEF	chr9	85677596/T//C	nonsynonymous	1	0
TRIM32	chr9	119461904/G//C	nonsynonymous	1	0
OLFML2A	chr9	127561585/C//A	synonymous	1	0
LRSAM1	chr9	130258290/G//A	synonymous	1	0
FAM129B	chr9	130271353/C//A	nonsynonymous	1	0
FAM188A	chr10	15831256/C//G	nonsynonymous	1	0
GPR158	chr10	25878011/C//G	nonsynonymous	1	0
ITGB1	chr10	33211612/G//C	nonsynonymous	1	0
PCDH15	chr10	55583117/G//C	nonsynonymous	1	0
GRID1	chr10	87966397/G//C	nonsynonymous	1	0
CYP26C1	chr10	94828248/G//A	nonsynonymous	1	0
ABCC2	chr10	101610461/C//A	nonsynonymous	1	0
ERLIN1	chr10	101935806/T//C	nonsynonymous	1	0
PITX3	chr10	103990410/C//G	nonsynonymous	1	0
SH3PXD2A	chr10	105386868/G//C	nonsynonymous	1	0
CCDC172	chr10	118084569/C//G	nonsynonymous	1	0
PNLIP	chr10	118318726/T//A	nonsynonymous	1	0
PLEKHA1	chr10	124166086/C//G	synonymous	1	0
DNHD1	chr11	6549937/A//C	nonsynonymous	1	0
ABCC8	chr11	17428330/G//A	synonymous	1	0
FANCF	chr11	22646888/G//T	nonsynonymous	1	0
OVOL1	chr11	65561716/G//A	nonsynonymous	1	0
C11orf86	chr11	66742996/G//C	nonsynonymous	1	0
C2CD3	chr11	73820158/A//G	nonsynonymous	1	0
FOLH1B	chr11	89413830/A//G	nonsynonymous	1	0
TRIM77	chr11	89450612/T//A	nonsynonymous	1	0
USP28	chr11	113688563/A//T	synonymous	1	0
CADM1	chr11	115080343/G//T	synonymous	1	0
TECTA	chr11	121023726/C//G	synonymous	1	0
FGF23	chr12	4488761/T//C	synonymous	1	0
CDKN1B	chr12	12871149/G//T	nonsynonymous	1	0
EPS8	chr12	15835901/T//C	synonymous	1	0
DBX2	chr12	45444751/C//T	synonymous	1	0
KMT2D	chr12	49426270/G//T	nonsynonymous	1	0
POU6F1	chr12	51585527/C//G	nonsynonymous	1	0
MFSD5	chr12	53646729/C//G	nonsynonymous	1	0
OR6C70	chr12	55863201/G//C	nonsynonymous	1	0
MIP	chr12	56845061/C//A	synonymous	1	0
SRGAP1	chr12	64437329/A//C	nonsynonymous	1	0
ZFC3H1	chr12	72025913/C//G	nonsynonymous	1	0
TMEM132C	chr12	129190037/G//A	nonsynonymous	1	0
FNDC3A	chr13	49749536/A//T	synonymous	1	0
KLHL1	chr13	70293531/T//C	nonsynonymous	1	0
ABCC4	chr13	95724056/GTGCTTCTTTTT//G	nonsynonymous	1	0
EFS	chr14	23834235/G//C	synonymous	1	0
PSME2	chr14	24612705/G//A	synonymous	1	0
RALGAPA1	chr14	36147299/C//A	nonsynonymous	1	0
TBPL2	chr14	55907242/C//G	nonsynonymous	1	0
SPTB	chr14	65271724/A//T	nonsynonymous	1	0
C14orf1	chr14	76117842/GGAA//G	synonymous	1	0
SPTLC2	chr14	78043230/T//C	nonsynonymous	1	0
PPP4R3A	chr14	91928438/C//T	nonsynonymous	1	0
MKRN3	chr15	23811712/C//T	synonymous	1	0
MAP1A	chr15	43821137/G//C	nonsynonymous	1	0
ATP8B4	chr15	50154444/G//T	nonsynonymous	1	0
RFX7	chr15	56390279/C//T	synonymous	1	0
	chr15	67665847/G//A	synonymous	1	0
CSPG4	chr15	75983014/G//C	nonsynonymous	1	0

CRAMP1	chr16	1723904/G//A	nonsynonymous	1	0
ELOB	chr16	2822044/C//T	nonsynonymous	1	0
CCP110	chr16	19547609/A//T	synonymous	1	0
SRCAP	chr16	30735164/G//A	synonymous	1	0
SALL1	chr16	51171420/C//T	nonsynonymous	1	0
TPPP3	chr16	67424925/C//T	nonsynonymous	1	0
ESRP2	chr16	68265073/G//A	synonymous	1	0
HYDIN	chr16	70906055/C//A	nonsynonymous	1	0
CDYL2	chr16	80718975/G//C	nonsynonymous	1	0
FBXO31	chr16	87369787/C//T	nonsynonymous	1	0
ANKFY1	chr17	4074136/C//T	nonsynonymous	1	0
C17orf107	chr17	4805130/G//A	synonymous	1	0
POLR2A	chr17	7401049/G//A	synonymous	1	0
MYH4	chr17	10366429/G//A	synonymous	1	0
NF1	chr17	29496952/C//G	nonsynonymous	1	0
C17orf50	chr17	34091073/G//C	nonsynonymous	1	0
KRT25	chr17	38906791/G//A	nonsynonymous	1	0
KLHL10	chr17	40004265/C//T	synonymous	1	0
CHAD	chr17	48545832/G//A	nonsynonymous	1	0
EPN3	chr17	48616632/G//C	nonsynonymous	1	0
ERN1	chr17	62130266/G//C	nonsynonymous	1	0
ERN1	chr17	62130278/G//C	nonsynonymous	1	0
ABCA6	chr17	67092423/T//C	synonymous	1	0
ABCA6	chr17	67092434/T//C	nonsynonymous	1	0
USP14	chr18	210474/A//G	synonymous	1	0
MTCL1	chr18	8783697/C//G	nonsynonymous	1	0
ZNF521	chr18	22804644/G//A	synonymous	1	0
ZNF521	chr18	22804647/C//A	nonsynonymous	1	0
SOCS6	chr18	67993270/G//C	nonsynonymous	1	0
TMIGD2	chr19	4292691/G//T	nonsynonymous	1	0
PLIN5	chr19	4534063/C//T	synonymous	1	0
TRIP10	chr19	6751159/C//G	synonymous	1	0
MUC16	chr19	9073852/G//T	nonsynonymous	1	0
ICAM1	chr19	10395209/G//A	synonymous	1	0
LDLR	chr19	11227573/C//A	nonsynonymous	1	0
OR10H2	chr19	15839630/C//G	nonsynonymous	1	0
BABAM1	chr19	17382409/A//G	nonsynonymous	1	0
DDA1	chr19	17424889/G//A	nonsynonymous	1	0
KLHL26	chr19	18779580/C//T	nonsynonymous	1	0
PDCD5	chr19	33078225/C//T	synonymous	1	0
B3GNT8	chr19	41932536/G//T	nonsynonymous	1	0
TMEM145	chr19	42827751/C//T	nonsynonymous	1	0
PSG7	chr19	43439924/A//G	synonymous	1	0
CD3EAP	chr19	45911557/C//A	nonsynonymous	1	0
NTN5	chr19	49167063/C//G	nonsynonymous	1	0
PPFIA3	chr19	49636324/C//G	nonsynonymous	1	0
TRPM4	chr19	49691973/G//C	nonsynonymous	1	0
PNKP	chr19	50364953/C//G	nonsynonymous	1	0
ZNF766	chr19	52793492/C//G	nonsynonymous	1	0
ZNF611	chr19	53208318/G//A	nonsynonymous	1	0
ZNF835	chr19	57175103/C//T	synonymous	1	0
ZNF256	chr19	58455348/C//G	synonymous	1	0
KAT14	chr20	18143353/C//G	nonsynonymous	1	0
PREX1	chr20	47253165/C//G	nonsynonymous	1	0
BMP7	chr20	55746009/G//A	synonymous	1	0
PHACTR3	chr20	58415419/C//T	synonymous	1	0
CABLES2	chr20	60966347/G//C	nonsynonymous	1	0
TCFL5	chr20	61473367/TGTGCAG//T	nonsynonymous	1	0
CHAF1B	chr21	37785511/C//T	nonsynonymous	1	0
PCNT	chr21	47831296/G//A	nonsynonymous	1	0
CECR2	chr22	18021615/C//T	nonsynonymous	1	0
SNAP29	chr22	21213488/C//G	nonsynonymous	1	0
MICALL1	chr22	38318186/C//T	synonymous	1	0
RPL3	chr22	39710697/G//T	nonsynonymous	1	0
RPS19BP1	chr22	39928758/C//T	synonymous	1	0
PHKA2	chrX	18924697/G//A	nonsynonymous	1	0
MED14	chrX	40522245/G//C	nonsynonymous	1	0
SLITRK4	chrX	142718488/A//G	nonsynonymous	1	0
PTPN22	chr1	114397664/C//T	nonsynonymous	0	1
GDF7	chr2	20870897/C//T	synonymous	0	1

DNAH6	chr2	84811376/T//A	nonsynonymous	0	1
IPCEF1	chr6	154533903/C//A	nonsynonymous	0	1
OSBPL3	chr7	24843993/C//T	synonymous	0	1
NUCB2	chr11	17352482/C//G	nonsynonymous	0	1
MTNR1B	chr11	92703025/C//T	nonsynonymous	0	1
KRT4	chr12	53207759/A//T	synonymous	0	1
ARHGEF40	chr14	21549107/G//A	nonsynonymous	0	1
ADCY9	chr16	4163864/C//T	nonsynonymous	0	1
ZNF714	chr19	21300853/C//T	synonymous	0	1
ZNF208	chr19	22153917/T//C	synonymous	0	1
MED29	chr19	39888400/A//G	synonymous	0	1
IGLON5	chr19	51831093/G//A	nonsynonymous	0	1
XRCC6	chr22	42046836/A//G	nonsynonymous	0	1
MXRA5	chrX	3228449/C//T	nonsynonymous	0	1
DCAF12L1	chrX	125685851/C//A	synonymous	0	1
ARHGEF6	chrX	135764105/G//A	nonsynonymous	0	1
MAST4	chr5	66459566/G//A	nonsynonymous	1	0
BAZ1A	chr14	35269495/T//A	nonsynonymous	1	0
SYNE2	chr14	64488666/A//G	nonsynonymous	1	0
BTBD7	chr14	93761226/T//C	nonsynonymous	1	0
PPP1R13B	chr14	104209054/G//A	synonymous	1	0
AHNAK2	chr14	105405542/G//A	nonsynonymous	1	0
SMAD6	chr15	66996033/C//T	nonsynonymous	1	0
LOC100506388	chr17	182928/C//A	synonymous	1	0
UNC45B	chr17	33503014/A//G	synonymous	1	0
RBBP8	chr18	20548821/A//G	nonsynonymous	1	0
OR7C1	chr19	14910531/G//C	nonsynonymous	1	0
ZBTB40	chr1	22852920/G//A	synonymous	0	1
ZNHIT6	chr1	86171980/T//C	nonsynonymous	0	1
DPYD	chr1	97981309/G//T	synonymous	0	1
GLUL	chr1	182354907/G//T	synonymous	0	1
RPS6KC1	chr1	213303064/C//T	nonsynonymous	0	1
GDF7	chr2	20870478/G//T	nonsynonymous	0	1
PLB1	chr2	28812545/G//T	nonsynonymous	0	1
PHOSPHO2	chr2	170557486/A//G	nonsynonymous	0	1
TTN	chr2	179605569/A//T	nonsynonymous	0	1
CRYGC	chr2	208993033/C//T	nonsynonymous	0	1
SCN10A	chr3	38739123/C//T	nonsynonymous	0	1
KALRN	chr3	124380731/C//T	nonsynonymous	0	1
GATA2	chr3	128205115/G//A	nonsynonymous	0	1
SLC7A14	chr3	170198349/G//T	synonymous	0	1
BST1	chr4	15704955/G//A	nonsynonymous	0	1
KLF3	chr4	38690369/C//T	nonsynonymous	0	1
RASGEF1B	chr4	82355053/A//T	nonsynonymous	0	1
LARP1B	chr4	129028426/T//G	nonsynonymous	0	1
PCDH10	chr4	134071517/C//T	synonymous	0	1
SPOCK3	chr4	167983675/C//T	nonsynonymous	0	1
VEGFC	chr4	177649053/A//G	nonsynonymous	0	1
FBN2	chr5	127686594/G//A	synonymous	0	1
BRD8	chr5	137503684/C//A	nonsynonymous	0	1
PCDHAC1	chr5	140307474/G//A	nonsynonymous	0	1
TENM2	chr5	167645618/G//T	nonsynonymous	0	1
JARID2	chr6	15507592/C//G	synonymous	0	1
EHMT2	chr6	31852191/G//A	nonsynonymous	0	1
SOBP	chr6	107955548/G//A	synonymous	0	1
	chr6	128330421/G//T	synonymous	0	1
MTHFD1L	chr6	151247339/T//C	synonymous	0	1
LPA	chr6	160958890/G//T	nonsynonymous	0	1
BBS9	chr7	33297025/A//G	synonymous	0	1
ABCA13	chr7	48626793/C//T	nonsynonymous	0	1
TECPR1	chr7	97854396/C//A	synonymous	0	1
ZAN	chr7	100356153/G//A	nonsynonymous	0	1
IFNA6	chr9	21350872/A//G	synonymous	0	1
TAF1L	chr9	32631814/C//T	nonsynonymous	0	1
OR13D1	chr9	107457487/G//C	nonsynonymous	0	1
OLFML2A	chr9	127561615/C//T	nonsynonymous	0	1
KIAA1462	chr10	30318048/C//T	synonymous	0	1
INA	chr10	105036950/C//T	synonymous	0	1
JAKMIP3	chr10	133980544/T//G	synonymous	0	1
SPON1	chr11	14284464/C//T	nonsynonymous	0	1

NAV2	chr11	19954790/G//A	nonsynonymous	0	1
MS4A1	chr11	60230542/T//C	nonsynonymous	0	1
SRSF8	chr11	94800918/A//C	synonymous	0	1
ATM	chr11	108203625/G//T	nonsynonymous	0	1
ITFG2	chr12	2932038/C//T	nonsynonymous	0	1
ACSM4	chr12	7477149/T//A	synonymous	0	1
PHC1	chr12	9085283/G//T	synonymous	0	1
KLRC2	chr12	10583820/C//T	nonsynonymous	0	1
BICD1	chr12	32481245/G//A	nonsynonymous	0	1
DNM1L	chr12	32832340/T//C	nonsynonymous	0	1
TUBA1A	chr12	49582843/G//T	synonymous	0	1
LRP1	chr12	57599465/C//G	synonymous	0	1
LHX5	chr12	113901367/G//A	synonymous	0	1
SHISA2	chr13	26621109/G//A	nonsynonymous	0	1
VSX2	chr14	74706361/G//A	nonsynonymous	0	1
DIO3	chr14	102028652/C//T	synonymous	0	1
CEP152	chr15	49060527/T//C	nonsynonymous	0	1
NTRK3	chr15	88472649/C//T	nonsynonymous	0	1
WDR90	chr16	705326/G//A	nonsynonymous	0	1
MSRB1	chr16	1993167/G//A	synonymous	0	1
CCP110	chr16	19548388/G//T	nonsynonymous	0	1
DNAH3	chr16	21060982/C//T	nonsynonymous	0	1
PRMT7	chr16	68382273/A//T	nonsynonymous	0	1
ABCA10	chr17	67146120/C//G	synonymous	0	1
LOXHD1	chr18	44121805/C//T	nonsynonymous	0	1
ZNF532	chr18	56587348/C//T	nonsynonymous	0	1
TSHZ3	chr19	31768624/T//G	nonsynonymous	0	1
TSHZ3	chr19	31768640/G//T	nonsynonymous	0	1
CD22	chr19	35837695/G//A	synonymous	0	1
HAUS5	chr19	36109316/G//A	synonymous	0	1
CLIP3	chr19	36507118/G//A	synonymous	0	1
SPRED3	chr19	38880956/G//A	nonsynonymous	0	1
PLEKHA4	chr19	49348816/G//A	nonsynonymous	0	1
NLRP5	chr19	56511154/G//A	nonsynonymous	0	1
NKX2-4	chr20	21376793/C//T	nonsynonymous	0	1
ZBTB46	chr20	62407156/G//A	nonsynonymous	0	1
TOP3B	chr22	22326297/G//A	synonymous	0	1
MEI1	chr22	42189884/G//C	nonsynonymous	0	1
FBLN1	chr22	45944486/G//A	synonymous	0	1
IL3RA	chrX	1471272/C//T	synonymous	0	1
USP9X	chrX	41025363/C//T	nonsynonymous	0	1
BCORL1	chrX	129148993/G//T	nonsynonymous	0	1
GPR50	chrX	150349664/C//A	nonsynonymous	0	1
PIK3CD	chr1	9784478/C//T	nonsynonymous	0	1
ASB17	chr1	76397784/C//T	nonsynonymous	0	1
IRF6	chr1	209964203/G//A	nonsynonymous	0	1
DNAH6	chr2	84804474/T//C	nonsynonymous	0	1
RMND5A	chr2	86968045/T//A	synonymous	0	1
TTN	chr2	179575433/G//A	nonsynonymous	0	1
BRPF1	chr3	9784744/C//T	synonymous	0	1
KBTBD12	chr3	127642773/G//C	nonsynonymous	0	1
	chr3	178984599/C//T	synonymous	0	1
LGI2	chr4	25014073/G//A	nonsynonymous	0	1
PLK4	chr4	128802215/G//A	synonymous	0	1
ADAMTS16	chr5	5182287/C//A	nonsynonymous	0	1
APC	chr5	112111354/G//T	nonsynonymous	0	1
SEMA6A	chr5	115840540/C//T	nonsynonymous	0	1
TXNDC15	chr5	134223822/A//C	nonsynonymous	0	1
HIST1H4L	chr6	27841253/G//A	synonymous	0	1
TAAR2	chr6	132938394/G//T	nonsynonymous	0	1
SYNE1	chr6	152725390/C//G	synonymous	0	1
MAGI2	chr7	78636403/C//G	synonymous	0	1
CSMD1	chr8	3611512/G//A	nonsynonymous	0	1
UBXN8	chr8	30612264/C//T	nonsynonymous	0	1
CCDC107	chr9	35658340/G//A	synonymous	0	1
PNLIPRP1	chr10	118360611/G//A	nonsynonymous	0	1
FOLR3	chr11	71850797/C//T	synonymous	0	1
TRHDE	chr12	73056901/G//A	nonsynonymous	0	1
SDS	chr12	113836598/C//T	nonsynonymous	0	1
KLHL1	chr13	70535512/T//C	nonsynonymous	0	1

SLITRK6	chr13	86369681/G//C	synonymous	0	1
TMTC4	chr13	101278382/T//C	nonsynonymous	0	1
FAM177A1	chr14	35550459/A//T	synonymous	0	1
	chr14	79746837/C//CT	synonymous	0	1
SHC4	chr15	49127200/C//A	nonsynonymous	0	1
HS3ST4	chr16	26147192/C//T	nonsynonymous	0	1
ALDH3A1	chr17	19641771/G//A	synonymous	0	1
HOXB3	chr17	46629809/C//T	nonsynonymous	0	1
LOXHD1	chr18	44057729/C//T	synonymous	0	1
ZNF433	chr19	12125995/C//T	nonsynonymous	0	1
CILP2	chr19	19654863/C//T	synonymous	0	1
TTC9B	chr19	40724038/G//A	nonsynonymous	0	1
SAMD10	chr20	62608427/G//A	synonymous	0	1
DSCAM	chr21	41710235/G//A	nonsynonymous	0	1
SHANK3	chr22	51169172/T//C	nonsynonymous	0	1
NUDC	chr1	27272169/T//C	synonymous	0	1
IGSF3	chr1	117146268/A//G	synonymous	0	1
TBCE	chr1	235599161/T//G	synonymous	0	1
THSD7B	chr2	137814006/T//A	nonsynonymous	0	1
ALPI	chr2	233321977/G//T	nonsynonymous	0	1
PCDH10	chr4	134084409/G//A	synonymous	0	1
IRX1	chr5	3599510/C//T	nonsynonymous	0	1
TMED4	chr7	44620760/G//C	nonsynonymous	0	1
ZNF705G	chr8	7215812/C//T	nonsynonymous	0	1
HAUS6	chr9	19082998/A//C	nonsynonymous	0	1
PDCD4	chr10	112635836/A//G	synonymous	0	1
CLEC4A	chr12	8276483/G//A	synonymous	0	1
MAP1A	chr15	43821123/G//C	synonymous	0	1
MAP1A	chr15	43821125/C//G	nonsynonymous	0	1
POLG	chr15	89876861/C//T	nonsynonymous	0	1
SRRM2	chr16	2819213/C//T	nonsynonymous	0	1
KIFC3	chr16	57803585/G//A	synonymous	0	1
DVL2	chr17	7137551/C//A	nonsynonymous	0	1
MED1	chr17	37566602/AG//A	nonsynonymous	0	1
SGSH	chr17	78185988/G//A	synonymous	0	1
FAM71E2	chr19	55870520/A//T	synonymous	0	1
ZSCAN22	chr19	58850710/G//A	synonymous	0	1
ADGRB2	chr1	32196442/G//A	nonsynonymous	0	1
HCN3	chr1	155258051/C//T	nonsynonymous	0	1
UBQLN4	chr1	156021547/G//A	synonymous	0	1
BRINP2	chr1	177198978/C//T	synonymous	0	1
ALMS1	chr2	73827844/G//A	nonsynonymous	0	1
COL6A3	chr2	238277378/C//A	synonymous	0	1
TWIST2	chr2	239757059/G//A	nonsynonymous	0	1
PRRT3	chr3	9991145/T//C	nonsynonymous	0	1
ADGRA3	chr4	22389403/C//T	synonymous	0	1
NPFFR2	chr4	73003776/G//A	synonymous	0	1
USO1	chr4	76725353/A//G	nonsynonymous	0	1
SEPT11	chr4	77949872/A//G	synonymous	0	1
PRDM9	chr5	23510135/A//T	nonsynonymous	0	1
C7	chr5	40972656/G//T	nonsynonymous	0	1
ISL1	chr5	50685738/T//C	nonsynonymous	0	1
MCTP1	chr5	94253682/G//T	synonymous	0	1
CAMK4	chr5	110819898/G//A	nonsynonymous	0	1
APC	chr5	112175145/A//AT	nonsynonymous	0	1
PCDHB11	chr5	140581615/G//A	synonymous	0	1
HIST1H2BD	chr6	26158611/G//C	nonsynonymous	0	1
LPA	chr6	161085267/C//T	synonymous	0	1
LPA	chr6	161085295/A//G	synonymous	0	1
TNRC18	chr7	5372346/C//T	synonymous	0	1
ZNF479	chr7	57188200/C//A	nonsynonymous	0	1
KCP	chr7	128531004/G//T	nonsynonymous	0	1
OR2A5	chr7	143748195/G//A	nonsynonymous	0	1
DLGAP2	chr8	1496882/G//A	nonsynonymous	0	1
FGFR1	chr8	38274873/G//A	synonymous	0	1
C9orf47	chr9	91605803/C//T	synonymous	0	1
DENND1A	chr9	126319981/A//G	synonymous	0	1
FGF8	chr10	103530314/C//T	synonymous	0	1
SMNDC1	chr10	112058411/A//G	synonymous	0	1
RIC8A	chr11	208728/C//T	synonymous	0	1

HPD	chr12	122277638/A//G	nonsynonymous	0	1
GSC	chr14	95234970/C//CA	nonsynonymous	0	1
MAGEL2	chr15	23892671/G//A	synonymous	0	1
ZNF106	chr15	42742213/T//C	nonsynonymous	0	1
TRPM7	chr15	50875281/C//A	synonymous	0	1
TMC3	chr15	81651776/A//C	nonsynonymous	0	1
TUFM	chr16	28857638/CAGA//C	synonymous	0	1
JPH3	chr16	87636782/C//T	synonymous	0	1
TEKT1	chr17	6719159/T//C	nonsynonymous	0	1
DBF4B	chr17	42828503/C//T	nonsynonymous	0	1
ADCYAP1	chr18	909533/G//A	nonsynonymous	0	1
C18orf8	chr18	21083555/G//A	synonymous	0	1
GPX4	chr19	1104121/A//G	nonsynonymous	0	1
DCAF15	chr19	14071820/C//T	synonymous	0	1
	chr19	41066381/C//T	synonymous	0	1
	chr20	48732132/G//A	synonymous	0	1
CDH4	chr20	60485505/G//A	nonsynonymous	0	1
C21orf62	chr21	34166740/C//T	synonymous	0	1
TRPM2	chr21	45846968/G//A	synonymous	0	1
RNF215	chr22	30783097/T//C	nonsynonymous	0	1
TNRC6B	chr22	40673147/G//T	nonsynonymous	0	1
TAF1	chrX	70596914/AGGAAGCAACACAGG	nonsynonymous	0	1
GPR88	chr1	101005074/C//T	synonymous	0	1
DENND2D	chr1	111734882/C//T	synonymous	0	1
LRRTM4	chr2	76975878/G//A	synonymous	0	1
EIF5B	chr2	99953958/A//G	synonymous	0	1
PDE11A	chr2	178704994/G//A	nonsynonymous	0	1
FAM171B	chr2	187618753/C//T	nonsynonymous	0	1
COL3A1	chr2	189859318/C//T	nonsynonymous	0	1
GBX2	chr2	237074648/C//T	nonsynonymous	0	1
CCNL1	chr3	156877986/G//T	synonymous	0	1
C4orf22	chr4	81256976/C//T	synonymous	0	1
LMNB1	chr5	126141282/C//T	nonsynonymous	0	1
FOXI1	chr5	169535487/T//C	nonsynonymous	0	1
SYNE1	chr6	152804264/C//T	nonsynonymous	0	1
ADAMDEC1	chr8	24256552/A//C	nonsynonymous	0	1
PURG	chr8	30890254/G//A	synonymous	0	1
WRN	chr8	30922424/G//A	synonymous	0	1
UBR5	chr8	103424470/T//C	synonymous	0	1
PSMA1	chr11	14536002/A//G	nonsynonymous	0	1
RAG1	chr11	36596373/C//T	nonsynonymous	0	1
FAT3	chr11	92531160/G//A	nonsynonymous	0	1
PDE3A	chr12	20833032/A//T	nonsynonymous	0	1
DPY19L2	chr12	63954278/G//T	synonymous	0	1
PNP	chr14	20944576/G//A	nonsynonymous	0	1
DUOXA2	chr15	45408760/G//A	synonymous	0	1
TPSD1	chr16	1306973/A//G	nonsynonymous	0	1
DDX19B	chr16	70333255/T//C	nonsynonymous	0	1
PTPRS	chr19	5245794/C//T	synonymous	0	1
ZNF430	chr19	21240831/T//A	synonymous	0	1
ZNF135	chr19	58570640/G//A	synonymous	0	1
	chr2	27592873/G//A	synonymous	0	1
C2orf54	chr2	241831142/G//A	nonsynonymous	0	1
TMEM158	chr3	45267352/G//C	synonymous	0	1
DCUN1D1	chr3	182683473/T//C	synonymous	0	1
IGF2BP2	chr3	185410517/A//G	nonsynonymous	0	1
MUC17	chr7	100680571/C//T	synonymous	0	1
MUC17	chr7	100680587/A//G	nonsynonymous	0	1
KCNB2	chr8	73480147/C//T	nonsynonymous	0	1
ZC3H3	chr8	144557739/G//A	nonsynonymous	0	1
SPATA31C1	chr9	90534206/T//C	nonsynonymous	0	1
MRPL51	chr12	6602313/T//TCCCC	synonymous	0	1
ACTR10	chr14	58701118/T//C	nonsynonymous	0	1
LIPC	chr15	58834078/T//C	nonsynonymous	0	1
C16orf58	chr16	31504842/A//G	nonsynonymous	0	1
ZNF652	chr17	47375931/G//C	nonsynonymous	0	1
APLP1	chr19	36365454/C//T	nonsynonymous	0	1
ZNF850	chr19	37240387/C//T	nonsynonymous	0	1
ZNF665	chr19	53668200/T//C	nonsynonymous	0	1
MCM3AP	chr21	47704043/T//C	synonymous	0	1

DCAF8L1	chrX	27998721/T//C	nonsynonymous	0	1
UBA1	chrX	47062165/A//G	synonymous	0	1
NHSL2	chrX	71363224/G//T	nonsynonymous	0	1
THOC2	chrX	122765652/T//C	nonsynonymous	0	1
SAMD11	chr1	878199/C//T	nonsynonymous	0	1
PLEKHN1	chr1	909351/G//A	nonsynonymous	0	1
CCNL2	chr1	1333703/A//G	nonsynonymous	0	1
PRKCZ	chr1	2082319/C//T	nonsynonymous	0	1
MORN1	chr1	2268202/G//A	nonsynonymous	0	1
TNFRSF9	chr1	7980847/T//C	synonymous	0	1
UBE4B	chr1	10155612/G//A	nonsynonymous	0	1
OTUD3	chr1	20224037/G//A	nonsynonymous	0	1
OTUD3	chr1	20231407/C//T	synonymous	0	1
MYOM3	chr1	24417349/A//G	nonsynonymous	0	1
SRRM1	chr1	24995972/G//A	nonsynonymous	0	1
CNKSR1	chr1	26508877/G//A	nonsynonymous	0	1
ARID1A	chr1	27057801/G//A	synonymous	0	1
OPRD1	chr1	29138971/G//A	nonsynonymous	0	1
COL16A1	chr1	32131521/G//A	nonsynonymous	0	1
	chr1	38227015/T//A	synonymous	0	1
RIMKLA	chr1	42880572/C//T	nonsynonymous	0	1
ZMYND12	chr1	42915598/C//G	nonsynonymous	0	1
SZT2	chr1	43906230/G//A	synonymous	0	1
KDM4A	chr1	44160566/G//A	nonsynonymous	0	1
CCDC24	chr1	44457614/C//T	nonsynonymous	0	1
SLC6A9	chr1	44482838/C//T	synonymous	0	1
CYP4B1	chr1	47276601/A//C	nonsynonymous	0	1
ACOT11	chr1	55069616/G//A	synonymous	0	1
MROH7	chr1	55138759/C//A	synonymous	0	1
TACSTD2	chr1	59042681/C//A	nonsynonymous	0	1
LRRIQ3	chr1	74492601/T//A	nonsynonymous	0	1
ODF2L	chr1	86841988/A//G	synonymous	0	1
BARHL2	chr1	91182512/C//T	nonsynonymous	0	1
ATP1A1	chr1	116916128/G//A	synonymous	0	1
TBX15	chr1	119441645/C//A	synonymous	0	1
CHD1L	chr1	146757979/G//A	nonsynonymous	0	1
GJA8	chr1	147380139/C//T	synonymous	0	1
MTMR11	chr1	149901110/G//A	nonsynonymous	0	1
ANXA9	chr1	150956814/GCT//G	nonsynonymous	0	1
TNFAIP8L2	chr1	151131667/C//T	nonsynonymous	0	1
LCE5A	chr1	152484135/C//T	nonsynonymous	0	1
CRTC2	chr1	153923811/G//A	synonymous	0	1
DCST2	chr1	154996948/C//T	nonsynonymous	0	1
TTC24	chr1	156554729/G//A	nonsynonymous	0	1
NES	chr1	156646783/C//T	nonsynonymous	0	1
INSRR	chr1	156815188/G//A	nonsynonymous	0	1
SPTA1	chr1	158651438/C//T	nonsynonymous	0	1
PYHIN1	chr1	158946527/A//G	synonymous	0	1
CFAP45	chr1	159842312/C//T	nonsynonymous	0	1
COPA	chr1	160260457/G//A	nonsynonymous	0	1
NHLH1	chr1	160340709/G//A	nonsynonymous	0	1
CD48	chr1	160654748/C//T	nonsynonymous	0	1
HSPA6	chr1	161496265/G//A	nonsynonymous	0	1
NUF2	chr1	163317624/G//A	synonymous	0	1
SUCO	chr1	172558041/A//G	synonymous	0	1
RC3H1	chr1	173933300/A//G	nonsynonymous	0	1
BRINP2	chr1	177249704/C//T	synonymous	0	1
QSOX1	chr1	180153164/C//T	nonsynonymous	0	1
ZNF648	chr1	182025929/C//T	nonsynonymous	0	1
LAMC1	chr1	183105726/C//T	synonymous	0	1
NMNAT2	chr1	183253929/T//C	synonymous	0	1
NCF2	chr1	183556087/T//C	nonsynonymous	0	1
TRMT1L	chr1	185112528/G//A	nonsynonymous	0	1
HMCN1	chr1	185878496/C//T	nonsynonymous	0	1
HMCN1	chr1	186057797/C//T	nonsynonymous	0	1
HMCN1	chr1	186123088/G//T	synonymous	0	1
KIF21B	chr1	200972715/G//A	nonsynonymous	0	1
PKP1	chr1	201289496/G//A	nonsynonymous	0	1
PKP1	chr1	201293684/C//T	synonymous	0	1
PPP1R12B	chr1	202385972/G//A	nonsynonymous	0	1

PIK3C2B	chr1	204418312/C//T	nonsynonymous	0	1
DSTYK	chr1	205116802/C//A	nonsynonymous	0	1
PLXNA2	chr1	208215630/T//A	nonsynonymous	0	1
SPATA17	chr1	217856639/C//A	nonsynonymous	0	1
	chr1	222922755/C//T	synonymous	0	1
PARP1	chr1	226573298/G//A	synonymous	0	1
CDC42BPA	chr1	227181944/C//T	synonymous	0	1
MAP10	chr1	232941955/G//A	nonsynonymous	0	1
PCNX2	chr1	233121865/T//C	synonymous	0	1
ARID4B	chr1	235377278/T//C	synonymous	0	1
FMN2	chr1	240255915/C//T	nonsynonymous	0	1
KIF26B	chr1	245530503/A//G	nonsynonymous	0	1
KIF26B	chr1	245851576/C//T	nonsynonymous	0	1
OR2L8	chr1	248112665/G//A	nonsynonymous	0	1
TPO	chr2	1481195/G//A	nonsynonymous	0	1
COLEC11	chr2	3691338/C//T	nonsynonymous	0	1
RHOB	chr2	20647177/G//A	synonymous	0	1
ASXL2	chr2	25991735/C//T	synonymous	0	1
OTOF	chr2	26684696/C//A	nonsynonymous	0	1
TMEM214	chr2	27263686/C//T	nonsynonymous	0	1
AGBL5	chr2	27281318/G//A	synonymous	0	1
ABHD1	chr2	27353142/T//C	synonymous	0	1
NLRC4	chr2	32475512/A//G	nonsynonymous	0	1
STRN	chr2	37152349/G//T	synonymous	0	1
ATL2	chr2	38525643/C//T	synonymous	0	1
PRKCE	chr2	46203692/G//A	synonymous	0	1
MCFD2	chr2	47168943/G//C	synonymous	0	1
MSH6	chr2	48027332/C//T	nonsynonymous	0	1
STON1	chr2	48809633/T//G	nonsynonymous	0	1
RTKN	chr2	74657762/G//A	synonymous	0	1
CD8B	chr2	87073853/G//A	synonymous	0	1
VWA3B	chr2	98920243/C//A	nonsynonymous	0	1
AFF3	chr2	100209937/C//A	nonsynonymous	0	1
IL18RAP	chr2	103053711/G//A	nonsynonymous	0	1
IL18RAP	chr2	103067368/C//T	nonsynonymous	0	1
SLC9A2	chr2	103281757/A//G	nonsynonymous	0	1
SLC20A1	chr2	113418089/T//G	nonsynonymous	0	1
CNTNAP5	chr2	125504946/G//A	nonsynonymous	0	1
POTEF	chr2	130832934/T//A	nonsynonymous	0	1
SMPD4	chr2	130910926/T//C	nonsynonymous	0	1
LRP1B	chr2	141625226/A//G	synonymous	0	1
EPC2	chr2	149528665/G//T	nonsynonymous	0	1
NEB	chr2	152432838/G//A	nonsynonymous	0	1
NEB	chr2	152534548/A//G	nonsynonymous	0	1
CACNB4	chr2	152728958/T//C	nonsynonymous	0	1
TANC1	chr2	160087093/G//A	nonsynonymous	0	1
LY75-CD302	chr2	160732144/G//A	synonymous	0	1
ITGB6	chr2	160982913/A//G	synonymous	0	1
CSRNP3	chr2	166514422/C//T	synonymous	0	1
SCN1A	chr2	166903389/G//A	nonsynonymous	0	1
SP5	chr2	171573116/G//A	synonymous	0	1
DCAF17	chr2	172325452/G//A	nonsynonymous	0	1
	chr2	173686678/G//A	synonymous	0	1
MTX2	chr2	177134382/G//A	nonsynonymous	0	1
PRKRA	chr2	179308038/C//A	nonsynonymous	0	1
TTN	chr2	179396115/G//A	nonsynonymous	0	1
TTN	chr2	179476842/G//A	nonsynonymous	0	1
PDE1A	chr2	183070725/G//A	nonsynonymous	0	1
ZNF804A	chr2	185463618/G//A	synonymous	0	1
FSIP2	chr2	186669432/T//G	synonymous	0	1
FAM171B	chr2	187559062/A//ACAG	nonsynonymous	0	1
COL5A2	chr2	189904278/A//G	synonymous	0	1
HIBCH	chr2	191152343/A//C	nonsynonymous	0	1
HECW2	chr2	197086952/C//T	nonsynonymous	0	1
GTF3C3	chr2	197636573/C//T	nonsynonymous	0	1
MAIP1	chr2	200820625/C//A	nonsynonymous	0	1
FZD5	chr2	208632393/C//T	synonymous	0	1
IKZF2	chr2	213914467/C//T	nonsynonymous	0	1
IGFBP5	chr2	217543673/C//T	nonsynonymous	0	1
TNS1	chr2	218749836/G//A	nonsynonymous	0	1

CATIP-AS1	chr2	219232312/A//G	nonsynonymous	0	1
CTDSP1	chr2	219267856/C//T	synonymous	0	1
STK16	chr2	220112915/T//A	nonsynonymous	0	1
EPHA4	chr2	222290844/A//G	synonymous	0	1
WDFY1	chr2	224770664/C//A	nonsynonymous	0	1
SLC19A3	chr2	228564253/T//C	nonsynonymous	0	1
GIGYF2	chr2	233710455/G//A	nonsynonymous	0	1
TRPM8	chr2	234871975/C//T	nonsynonymous	0	1
OR6B3	chr2	240985360/C//T	nonsynonymous	0	1
ANKMY1	chr2	241465688/G//A	synonymous	0	1
TRNT1	chr3	3188208/G//A	nonsynonymous	0	1
LRRN1	chr3	3887170/A//G	nonsynonymous	0	1
ITPR1	chr3	4824376/C//T	nonsynonymous	0	1
EDEM1	chr3	5229604/G//A	synonymous	0	1
WNT7A	chr3	13860616/C//T	nonsynonymous	0	1
PP2D1	chr3	20043179/C//T	nonsynonymous	0	1
XIRP1	chr3	39229709/C//T	nonsynonymous	0	1
	chr3	46305920/T//C	synonymous	0	1
NBEAL2	chr3	47035448/G//A	nonsynonymous	0	1
SCAP	chr3	47459938/G//A	nonsynonymous	0	1
SMARCC1	chr3	47651714/T//C	nonsynonymous	0	1
PLXNB1	chr3	48450804/G//A	nonsynonymous	0	1
PLXNB1	chr3	48453975/C//T	nonsynonymous	0	1
RBM6	chr3	50112685/C//T	synonymous	0	1
CYB561D2	chr3	50390809/C//T	synonymous	0	1
ZNF654	chr3	88189029/T//C	nonsynonymous	0	1
ABHD10	chr3	111697936/G//A	nonsynonymous	0	1
RABL3	chr3	120428633/C//T	nonsynonymous	0	1
EFCAB12	chr3	129127507/T//C	synonymous	0	1
ALG1L2	chr3	129800761/A//G	synonymous	0	1
MFSD1	chr3	158546867/A//G	synonymous	0	1
MYNN	chr3	169497342/T//A	synonymous	0	1
EIF5A2	chr3	170612207/T//C	nonsynonymous	0	1
USP13	chr3	179501916/G//A	nonsynonymous	0	1
PSMD2	chr3	184023889/T//C	nonsynonymous	0	1
MAP3K13	chr3	185191267/C//A	synonymous	0	1
GP5	chr3	194118248/G//A	nonsynonymous	0	1
CTBP1	chr4	1207295/G//A	nonsynonymous	0	1
CCDC96	chr4	7043169/G//A	synonymous	0	1
SLIT2	chr4	20535220/G//T	nonsynonymous	0	1
RFC1	chr4	39329302/G//T	nonsynonymous	0	1
SCFD2	chr4	54218838/T//C	nonsynonymous	0	1
KDR	chr4	55980401/C//T	synonymous	0	1
DCK	chr4	71889425/T//C	nonsynonymous	0	1
C4orf26	chr4	76489380/G//A	nonsynonymous	0	1
TACR3	chr4	104510910/T//A	nonsynonymous	0	1
FABP2	chr4	120240802/C//CATAATA	synonymous	0	1
PCDH18	chr4	138449888/G//A	synonymous	0	1
KIAA0922	chr4	154544214/G//A	nonsynonymous	0	1
PLEKHG4B	chr5	163461/G//A	nonsynonymous	0	1
PLEKHG4B	chr5	169489/G//A	nonsynonymous	0	1
ADAMTS16	chr5	5235166/C//T	synonymous	0	1
PDZD2	chr5	32090300/G//A	nonsynonymous	0	1
RXFP3	chr5	33937352/C//T	synonymous	0	1
NUP155	chr5	37342749/C//A	nonsynonymous	0	1
DEPDC1B	chr5	59982947/C//T	nonsynonymous	0	1
TNPO1	chr5	72201176/G//A	nonsynonymous	0	1
AGGF1	chr5	76330241/A//G	nonsynonymous	0	1
ACOT12	chr5	80641755/A//T	nonsynonymous	0	1
MCC	chr5	112363143/CTT//C	nonsynonymous	0	1
KCNN2	chr5	113698604/C//T	synonymous	0	1
SLC22A4	chr5	131630667/A//T	nonsynonymous	0	1
TMEM173	chr5	138860370/C//T	synonymous	0	1
PCDHGC4	chr5	140865385/C//T	synonymous	0	1
PCDHGC5	chr5	140869930/C//T	nonsynonymous	0	1
SH3RF2	chr5	145428746/C//T	synonymous	0	1
ARHGEF37	chr5	149001459/C//T	nonsynonymous	0	1
PPARGC1B	chr5	149216359/G//A	nonsynonymous	0	1
CSF1R	chr5	149435891/T//A	nonsynonymous	0	1
CAMK2A	chr5	149602213/C//A	synonymous	0	1

SYNPO	chr5	150027890/C//T	nonsynonymous	0	1
GRIA1	chr5	153190672/G//A	nonsynonymous	0	1
GEMIN5	chr5	154291403/C//T	nonsynonymous	0	1
ADAM19	chr5	156929996/A//C	synonymous	0	1
TENM2	chr5	167627028/C//T	nonsynonymous	0	1
DOCK2	chr5	169483771/C//A	nonsynonymous	0	1
HIVEP1	chr6	12122491/G//T	nonsynonymous	0	1
HIST1H4E	chr6	26204873/A//G	nonsynonymous	0	1
BTN3A1	chr6	26409796/G//A	nonsynonymous	0	1
HIST1H2AL	chr6	27833416/A//G	nonsynonymous	0	1
ZSCAN31	chr6	28297163/G//A	nonsynonymous	0	1
GABBR1	chr6	29574685/G//T	synonymous	0	1
TRIM15	chr6	30140183/C//T	synonymous	0	1
MSH5	chr6	31730334/C//A	synonymous	0	1
SKIV2L	chr6	31929106/C//A	synonymous	0	1
TNXB	chr6	32037339/C//T	nonsynonymous	0	1
PPT2	chr6	32130376/G//A	nonsynonymous	0	1
TMEM217	chr6	37183047/C//T	nonsynonymous	0	1
TRERF1	chr6	42196321/G//A	nonsynonymous	0	1
PTK7	chr6	43109906/A//T	synonymous	0	1
ZNF318	chr6	43307593/G//T	synonymous	0	1
CDC5L	chr6	44358054/C//A	nonsynonymous	0	1
KHDRBS2	chr6	62390821/C//A	synonymous	0	1
UBE3D	chr6	83748171/G//A	nonsynonymous	0	1
PRSS35	chr6	84233731/A//G	nonsynonymous	0	1
TBX18	chr6	85473784/C//T	nonsynonymous	0	1
ZNF292	chr6	87965690/T//C	synonymous	0	1
CFAP206	chr6	88125476/A//G	nonsynonymous	0	1
PNRC1	chr6	89791084/G//A	synonymous	0	1
MDN1	chr6	90398365/G//T	nonsynonymous	0	1
PRDM13	chr6	100062179/C//T	synonymous	0	1
ASCC3	chr6	101248174/A//G	nonsynonymous	0	1
LACE1	chr6	108668225/T//C	nonsynonymous	0	1
CDC40	chr6	110534291/C//T	synonymous	0	1
FAM162B	chr6	117086676/C//T	nonsynonymous	0	1
MCM9	chr6	119137260/A//G	nonsynonymous	0	1
GRM1	chr6	146350563/C//T	synonymous	0	1
SYNE1	chr6	152748950/G//T	nonsynonymous	0	1
FNDC1	chr6	159667980/G//A	nonsynonymous	0	1
PLG	chr6	161157944/G//T	nonsynonymous	0	1
FAM20C	chr7	300057/C//T	synonymous	0	1
ADAP1	chr7	943769/G//A	synonymous	0	1
TTYH3	chr7	2698055/G//A	synonymous	0	1
AP5Z1	chr7	4815378/A//G	nonsynonymous	0	1
RADIL	chr7	4854934/G//A	nonsynonymous	0	1
FSCN1	chr7	5645141/C//T	synonymous	0	1
MPP6	chr7	24718758/C//T	nonsynonymous	0	1
EVX1	chr7	27284929/C//T	synonymous	0	1
EVX1	chr7	27285725/G//A	nonsynonymous	0	1
NOD1	chr7	30492448/A//C	synonymous	0	1
CRHR2	chr7	30693147/G//A	nonsynonymous	0	1
ELMO1	chr7	36917634/G//A	synonymous	0	1
AMPH	chr7	38671029/C//A	synonymous	0	1
ZMIZ2	chr7	44800102/G//A	nonsynonymous	0	1
SUN3	chr7	48046787/G//T	nonsynonymous	0	1
PHKG1	chr7	56149727/G//A	synonymous	0	1
TRIM50	chr7	72730677/G//T	nonsynonymous	0	1
POR	chr7	75615146/C//T	nonsynonymous	0	1
MAGI2	chr7	77797242/G//A	synonymous	0	1
ABCB4	chr7	87042961/A//G	nonsynonymous	0	1
SLC25A40	chr7	87488015/T//C	nonsynonymous	0	1
	chr7	94833007/G//T	synonymous	0	1
ZNF394	chr7	99091416/G//A	synonymous	0	1
ACTL6B	chr7	100244443/C//T	synonymous	0	1
SH2B2	chr7	101944121/C//T	nonsynonymous	0	1
ALKBH4	chr7	102098235/T//G	nonsynonymous	0	1
RELN	chr7	103276917/T//G	nonsynonymous	0	1
DOCK4	chr7	111629193/C//T	nonsynonymous	0	1
CFTR	chr7	117304805/G//T	nonsynonymous	0	1
SPAM1	chr7	123593838/C//A	nonsynonymous	0	1

FSCN3	chr7	127236036/C//T	nonsynonymous	0	1
SND1	chr7	127732214/G//T	synonymous	0	1
ATP6V0A4	chr7	138434041/C//T	nonsynonymous	0	1
PARP12	chr7	139727072/G//A	synonymous	0	1
RAB19	chr7	140125841/G//A	nonsynonymous	0	1
ZNF786	chr7	148769135/G//A	synonymous	0	1
ZNF783	chr7	148979241/G//A	nonsynonymous	0	1
ABCB8	chr7	150732754/C//T	synonymous	0	1
ASIC3	chr7	150748266/G//C	nonsynonymous	0	1
AGAP3	chr7	150814491/G//A	nonsynonymous	0	1
AGAP3	chr7	150820980/G//A	synonymous	0	1
ERICH1	chr8	665927/C//T	nonsynonymous	0	1
CLN8	chr8	1719331/C//T	synonymous	0	1
KBTBD11	chr8	1950591/C//T	synonymous	0	1
CSMD1	chr8	2944682/C//T	nonsynonymous	0	1
RP1L1	chr8	10470609/G//A	synonymous	0	1
TNFRSF10D	chr8	22995716/C//T	nonsynonymous	0	1
NEFM	chr8	24772369/G//A	nonsynonymous	0	1
CDCA2	chr8	25346186/C//T	nonsynonymous	0	1
KIF13B	chr8	28929225/G//A	synonymous	0	1
LEPROTL1	chr8	29963280/G//A	nonsynonymous	0	1
HTRA4	chr8	38831993/G//A	nonsynonymous	0	1
NKAIN3	chr8	63492148/G//A	synonymous	0	1
NCOA2	chr8	71036301/A//G	nonsynonymous	0	1
KIAA1429	chr8	95556105/T//C	nonsynonymous	0	1
ODF1	chr8	103572755/C//T	synonymous	0	1
CTHRC1	chr8	104383929/CCTCCTGCTG//C	nonsynonymous	0	1
	chr8	105010412/G//T	synonymous	0	1
FAM91A1	chr8	124787446/G//T	nonsynonymous	0	1
ZNF572	chr8	125989904/A//G	nonsynonymous	0	1
NSMCE2	chr8	126369992/C//T	synonymous	0	1
PHF20L1	chr8	133848922/C//T	nonsynonymous	0	1
SCRIB	chr8	144875164/A//G	synonymous	0	1
SCRIB	chr8	144887474/C//T	synonymous	0	1
PUF60	chr8	144906524/C//T	nonsynonymous	0	1
PARP10	chr8	145051796/G//A	synonymous	0	1
LRRC24	chr8	145748646/T//C	nonsynonymous	0	1
ZNF517	chr8	146033433/C//T	nonsynonymous	0	1
COMMD5	chr8	146076122/C//T	nonsynonymous	0	1
DOCK8	chr9	439289/C//T	synonymous	0	1
VLDLR	chr9	2643294/G//A	nonsynonymous	0	1
KIAA2026	chr9	5968121/G//A	nonsynonymous	0	1
ELAVL2	chr9	23692366/A//G	synonymous	0	1
SPATA31A6	chr9	43627415/G//A	synonymous	0	1
TRPM6	chr9	77365604/C//A	nonsynonymous	0	1
TLE1	chr9	84205934/G//A	synonymous	0	1
DAPK1	chr9	90321943/G//A	synonymous	0	1
DAPK1	chr9	90322041/A//G	nonsynonymous	0	1
IPPK	chr9	95400291/C//T	nonsynonymous	0	1
ZNF169	chr9	97062405/G//A	nonsynonymous	0	1
XPA	chr9	100449380/G//C	nonsynonymous	0	1
TBC1D2	chr9	100961717/G//A	synonymous	0	1
ACTL7A	chr9	111625335/G//A	nonsynonymous	0	1
PALM2-AKAP2	chr9	112899423/T//C	synonymous	0	1
PAPPA	chr9	119033623/G//A	nonsynonymous	0	1
OLFML2A	chr9	127572206/C//T	nonsynonymous	0	1
SPTAN1	chr9	131394454/G//A	nonsynonymous	0	1
GTF3C4	chr9	135553865/G//A	nonsynonymous	0	1
NOTCH1	chr9	139417422/C//T	nonsynonymous	0	1
NOTCH1	chr9	139417596/C//T	nonsynonymous	0	1
EXD3	chr9	140243564/G//A	nonsynonymous	0	1
PNPLA7	chr9	140391642/G//A	synonymous	0	1
CACNA1B	chr9	140941438/G//A	nonsynonymous	0	1
PRKCQ	chr10	6483996/G//A	synonymous	0	1
FAM171A1	chr10	15256185/C//T	nonsynonymous	0	1
FAM171A1	chr10	15325874/T//C	synonymous	0	1
VIM	chr10	17277333/G//A	nonsynonymous	0	1
ZEB1	chr10	31810281/G//T	nonsynonymous	0	1
	chr10	49632560/A//G	synonymous	0	1
	chr10	55566748/A//G	synonymous	0	1

SIRT1	chr10	69648669/A//T	nonsynonymous	0	1
RUFY2	chr10	70166932/G//T	synonymous	0	1
SUPV3L1	chr10	70968481/A//C	nonsynonymous	0	1
TYSND1	chr10	71905392/G//C	synonymous	0	1
RGR	chr10	86008724/G//T	nonsynonymous	0	1
RGR	chr10	86018506/G//A	synonymous	0	1
OPN4	chr10	88419077/C//T	synonymous	0	1
IFIT1	chr10	91162860/G//T	nonsynonymous	0	1
ALDH18A1	chr10	97380874/C//T	nonsynonymous	0	1
FGF8	chr10	103534502/G//A	synonymous	0	1
GBF1	chr10	104128144/GCT//G	nonsynonymous	0	1
CALHM3	chr10	105238777/G//A	nonsynonymous	0	1
SORCS3	chr10	106401130/G//A	synonymous	0	1
NRAP	chr10	115423113/G//A	synonymous	0	1
TRUB1	chr10	116698102/A//C	synonymous	0	1
TRUB1	chr10	116698105/G//T	nonsynonymous	0	1
SHTN1	chr10	118764561/C//T	synonymous	0	1
PDZD8	chr10	119134384/C//T	nonsynonymous	0	1
EIF3A	chr10	120809460/G//A	synonymous	0	1
BUB3	chr10	124920065/C//T	nonsynonymous	0	1
BCCIP	chr10	127524676/G//A	nonsynonymous	0	1
DOCK1	chr10	129237356/A//G	nonsynonymous	0	1
MKI67	chr10	129908758/G//A	nonsynonymous	0	1
DPYSL4	chr10	134008415/C//T	nonsynonymous	0	1
DPYSL4	chr10	134010549/C//T	nonsynonymous	0	1
CYP2E1	chr10	135345128/G//A	nonsynonymous	0	1
EPS8L2	chr11	720751/G//T	synonymous	0	1
LSP1	chr11	1901345/G//A	nonsynonymous	0	1
CHRNA10	chr11	3688759/C//T	nonsynonymous	0	1
APBB1	chr11	6432517/C//T	nonsynonymous	0	1
SCUBE2	chr11	9087461/C//T	nonsynonymous	0	1
MYOD1	chr11	17741650/C//T	synonymous	0	1
LMO2	chr11	33880858/C//T	synonymous	0	1
API5	chr11	43342458/C//T	nonsynonymous	0	1
ACCS	chr11	44105394/G//A	synonymous	0	1
KBTBD4	chr11	47600430/A//T	synonymous	0	1
LRRC55	chr11	56955012/G//A	synonymous	0	1
PATL1	chr11	59426365/G//A	nonsynonymous	0	1
TMEM109	chr11	60689554/C//T	nonsynonymous	0	1
CPSF7	chr11	61187409/G//A	nonsynonymous	0	1
LRRN4CL	chr11	62455697/G//A	nonsynonymous	0	1
STIP1	chr11	63971000/G//A	nonsynonymous	0	1
KCNK4-TEX40	chr11	64065728/G//A	synonymous	0	1
CDC42BPG	chr11	64606350/C//T	nonsynonymous	0	1
NAALADL1	chr11	64824854/G//A	nonsynonymous	0	1
PITPNM1	chr11	67261435/C//T	nonsynonymous	0	1
NUMA1	chr11	71725554/G//A	nonsynonymous	0	1
RSF1	chr11	77383222/G//A	nonsynonymous	0	1
CCDC89	chr11	85396236/C//T	nonsynonymous	0	1
CCDC83	chr11	85630527/T//A	nonsynonymous	0	1
TYR	chr11	88911551/A//G	nonsynonymous	0	1
CEP126	chr11	101832467/T//C	synonymous	0	1
RDX	chr11	110128835/A//T	nonsynonymous	0	1
NCAM1	chr11	113078059/C//T	synonymous	0	1
ZW10	chr11	113639667/C//T	nonsynonymous	0	1
ZBTB16	chr11	114121208/C//T	synonymous	0	1
DSCAML1	chr11	117302334/A//G	nonsynonymous	0	1
C2CD2L	chr11	118981304/C//T	nonsynonymous	0	1
GRAMD1B	chr11	123493225/G//A	synonymous	0	1
SRPRA	chr11	126134389/C//T	nonsynonymous	0	1
FKBP4	chr12	2906347/G//T	nonsynonymous	0	1
CHD4	chr12	6687043/G//A	nonsynonymous	0	1
HEBP1	chr12	13128371/T//G	nonsynonymous	0	1
CAPZA3	chr12	18891993/G//A	nonsynonymous	0	1
PDE3A	chr12	20803540/T//C	synonymous	0	1
SLCO1B1	chr12	21353476/T//A	nonsynonymous	0	1
RASSF8	chr12	26217518/A//G	nonsynonymous	0	1
ITPR2	chr12	26775278/G//A	synonymous	0	1
ADAMTS20	chr12	43748048/A//C	synonymous	0	1
KMT2D	chr12	49443503/G//A	nonsynonymous	0	1

NCKAP5L	chr12	50190161/G//A	synonymous	0	1
GPD1	chr12	50497867/G//A	nonsynonymous	0	1
CERS5	chr12	50536906/G//A	nonsynonymous	0	1
TARBP2	chr12	53899446/G//A	nonsynonymous	0	1
SUOX	chr12	56397780/C//T	synonymous	0	1
CAND1	chr12	67703684/A//T	nonsynonymous	0	1
CAPS2	chr12	75678746/G//A	nonsynonymous	0	1
TMTC2	chr12	83250836/C//T	nonsynonymous	0	1
PARPBP	chr12	102517728/G//A	nonsynonymous	0	1
WSCD2	chr12	108641899/C//T	nonsynonymous	0	1
UBE3B	chr12	109921677/A//G	nonsynonymous	0	1
SH2B3	chr12	111856065/G//A	nonsynonymous	0	1
ATXN2	chr12	111908477/G//A	nonsynonymous	0	1
TPCN1	chr12	113726017/G//A	nonsynonymous	0	1
GCN1	chr12	120572451/A//G	nonsynonymous	0	1
C12orf43	chr12	121448948/A//G	synonymous	0	1
P2RX7	chr12	121622316/G//A	nonsynonymous	0	1
MORN3	chr12	122107322/C//A	nonsynonymous	0	1
WDR66	chr12	122441592/C//T	synonymous	0	1
CLIP1	chr12	122757390/T//C	synonymous	0	1
SBNO1	chr12	123815060/C//T	synonymous	0	1
DNAH10	chr12	124270304/C//T	synonymous	0	1
FBRSL1	chr12	133159750/C//T	nonsynonymous	0	1
GOLGA3	chr12	133360756/C//A	nonsynonymous	0	1
ATP12A	chr13	25265211/G//A	synonymous	0	1
WASF3	chr13	27255363/G//A	nonsynonymous	0	1
GTF3A	chr13	28009569/G//T	nonsynonymous	0	1
FRY	chr13	32752579/G//A	synonymous	0	1
POSTN	chr13	38144729/G//A	nonsynonymous	0	1
DGKH	chr13	42803314/C//T	nonsynonymous	0	1
ZC3H13	chr13	46542110/G//A	nonsynonymous	0	1
VPS36	chr13	52992189/C//T	synonymous	0	1
DNAJC3	chr13	96443193/G//A	nonsynonymous	0	1
MYO16	chr13	109793153/G//A	synonymous	0	1
TUBGCP3	chr13	113174261/G//A	nonsynonymous	0	1
TUBGCP3	chr13	113212536/C//T	synonymous	0	1
ADPRHL1	chr13	114077287/G//A	synonymous	0	1
SUPT16H	chr14	21831393/C//A	nonsynonymous	0	1
MMP14	chr14	23312593/C//T	synonymous	0	1
LRP10	chr14	23345585/A//G	synonymous	0	1
SLC22A17	chr14	23817755/G//A	nonsynonymous	0	1
HEATR5A	chr14	31782236/G//A	nonsynonymous	0	1
MBIP	chr14	36784005/G//A	synonymous	0	1
FOXA1	chr14	38061208/G//A	nonsynonymous	0	1
CLEC14A	chr14	38725058/C//G	nonsynonymous	0	1
FANCM	chr14	45654560/T//G	synonymous	0	1
	chr14	50789458/C//T	synonymous	0	1
CGRRF1	chr14	55005089/G//A	synonymous	0	1
TMEM260	chr14	57052475/G//A	synonymous	0	1
ARID4A	chr14	58796296/C//T	nonsynonymous	0	1
RTN1	chr14	60069777/A//G	synonymous	0	1
PLEKHD1	chr14	69995141/C//T	synonymous	0	1
PCNX1	chr14	71444742/G//A	nonsynonymous	0	1
LTBP2	chr14	74969592/C//T	nonsynonymous	0	1
CPSF2	chr14	92622936/C//A	nonsynonymous	0	1
GOLGA5	chr14	93299517/A//C	synonymous	0	1
UNC79	chr14	94069669/G//A	nonsynonymous	0	1
SERPINA5	chr14	95056484/T//C	synonymous	0	1
DICER1	chr14	95574784/A//G	synonymous	0	1
CEP170B	chr14	105342672/G//T	nonsynonymous	0	1
AHNAK2	chr14	105416320/A//G	nonsynonymous	0	1
GPR132	chr14	105518274/G//A	nonsynonymous	0	1
GABRA5	chr15	27188433/G//A	nonsynonymous	0	1
NSMCE3	chr15	29561737/T//C	nonsynonymous	0	1
FAM98B	chr15	38776806/A//T	synonymous	0	1
MGA	chr15	41988912/T//C	synonymous	0	1
ZNF106	chr15	42742512/C//T	nonsynonymous	0	1
STARD9	chr15	43011022/C//T	nonsynonymous	0	1
FBN1	chr15	48729576/G//A	nonsynonymous	0	1
FBN1	chr15	48777571/C//T	nonsynonymous	0	1

LEO1	chr15	52230255/A//G	synonymous	0	1
RFX7	chr15	56388616/G//A	nonsynonymous	0	1
LDHAL6B	chr15	59499582/G//A	nonsynonymous	0	1
TLN2	chr15	63009863/A//G	nonsynonymous	0	1
HERC1	chr15	63970259/C//T	synonymous	0	1
HERC1	chr15	64019934/G//A	synonymous	0	1
KBTBD13	chr15	65369649/G//A	nonsynonymous	0	1
UBAP1L	chr15	65394744/C//T	synonymous	0	1
MEGF11	chr15	66191105/G//A	nonsynonymous	0	1
EDC3	chr15	74925040/G//A	synonymous	0	1
SIN3A	chr15	75694229/C//T	nonsynonymous	0	1
ARNT2	chr15	80845031/A//G	synonymous	0	1
BTBD1	chr15	83735814/T//C	synonymous	0	1
WFIKKN1	chr16	683074/CAG//C	nonsynonymous	0	1
PRR25	chr16	863752/C//T	nonsynonymous	0	1
TPSB2	chr16	1279460/G//T	synonymous	0	1
CRAMP1	chr16	1682272/C//T	synonymous	0	1
MAPK8IP3	chr16	1818983/C//T	synonymous	0	1
TRAF7	chr16	2225837/C//A	synonymous	0	1
E4F1	chr16	2284731/G//A	nonsynonymous	0	1
AMDHD2	chr16	2578385/A//G	synonymous	0	1
ZSCAN10	chr16	3139035/G//A	synonymous	0	1
CIITA	chr16	10989169/G//T	nonsynonymous	0	1
XYLT1	chr16	17294417/G//A	synonymous	0	1
SYT17	chr16	19184096/C//T	synonymous	0	1
GP2	chr16	20325987/C//T	nonsynonymous	0	1
TNRC6A	chr16	24829938/C//T	nonsynonymous	0	1
CCDC189	chr16	30771047/T//C	nonsynonymous	0	1
ABCC11	chr16	48234378/C//A	nonsynonymous	0	1
CDH16	chr16	66946249/C//T	nonsynonymous	0	1
RRAD	chr16	66959009/C//T	nonsynonymous	0	1
ESRP2	chr16	68267742/C//T	nonsynonymous	0	1
PHLPP2	chr16	71715672/C//T	nonsynonymous	0	1
TMEM231	chr16	75575345/C//T	nonsynonymous	0	1
GSE1	chr16	85690916/A//C	nonsynonymous	0	1
SLC7A5	chr16	87873326/C//T	synonymous	0	1
ANKRD11	chr16	89346901/C//T	nonsynonymous	0	1
RPH3AL	chr17	97041/C//T	synonymous	0	1
CLUH	chr17	2595924/C//T	nonsynonymous	0	1
P2RX1	chr17	3802999/C//T	synonymous	0	1
NLGN2	chr17	7320485/C//T	synonymous	0	1
NLGN2	chr17	7320545/G//A	synonymous	0	1
DNAH2	chr17	7722533/C//T	nonsynonymous	0	1
	chr17	8025353/C//T	synonymous	0	1
MYH10	chr17	8439232/T//C	synonymous	0	1
PIRT	chr17	10728805/C//T	nonsynonymous	0	1
FLII	chr17	18149949/G//A	synonymous	0	1
TAOK1	chr17	27870038/T//C	nonsynonymous	0	1
SLC6A4	chr17	28537597/C//T	nonsynonymous	0	1
TEFM	chr17	29226143/C//T	synonymous	0	1
SYNRG	chr17	35913996/T//G	nonsynonymous	0	1
SYNRG	chr17	35969448/G//A	synonymous	0	1
CDK12	chr17	37627158/G//C	nonsynonymous	0	1
WIPF2	chr17	38430198/C//T	nonsynonymous	0	1
KRT15	chr17	39674848/C//T	nonsynonymous	0	1
WNK4	chr17	40937386/G//A	synonymous	0	1
BRCA1	chr17	41251786/C//A	synonymous	0	1
ARHGAP27	chr17	43481033/G//A	synonymous	0	1
FAM117A	chr17	47793619/G//T	synonymous	0	1
CACNA1G	chr17	48667850/G//A	nonsynonymous	0	1
PCTP	chr17	53852214/C//T	synonymous	0	1
EPX	chr17	56271153/G//A	nonsynonymous	0	1
TUBD1	chr17	57955556/T//C	nonsynonymous	0	1
TBX4	chr17	59533889/C//T	nonsynonymous	0	1
STRADA	chr17	61790815/C//T	nonsynonymous	0	1
ERN1	chr17	62135251/C//T	nonsynonymous	0	1
AXIN2	chr17	63533488/T//C	nonsynonymous	0	1
EXOC7	chr17	74090654/T//A	nonsynonymous	0	1
RNF157	chr17	74141315/C//T	synonymous	0	1
CCDC40	chr17	78058708/G//A	nonsynonymous	0	1

SLC16A3	chr17	80195340/G//A	nonsynonymous	0	1
ANKRD30B	chr18	14852512/A//T	synonymous	0	1
DSG1	chr18	28906935/T//C	synonymous	0	1
CNDP2	chr18	72168698/A//G	synonymous	0	1
ATP9B	chr18	77134003/C//T	nonsynonymous	0	1
NFATC1	chr18	77246347/G//A	nonsynonymous	0	1
PARD6G	chr18	77918073/C//T	nonsynonymous	0	1
BSG	chr19	580444/C//T	nonsynonymous	0	1
MED16	chr19	871987/G//A	synonymous	0	1
ABCA7	chr19	1047222/G//C	nonsynonymous	0	1
MEX3D	chr19	1555805/G//A	synonymous	0	1
DIRAS1	chr19	2717669/G//A	nonsynonymous	0	1
CHAF1A	chr19	4422572/C//T	nonsynonymous	0	1
NRTN	chr19	5828223/G//A	synonymous	0	1
PEX11G	chr19	7543211/C//T	synonymous	0	1
CD209	chr19	7812374/T//C	synonymous	0	1
OR7E24	chr19	9361754/T//C	nonsynonymous	0	1
PPAN	chr19	10221240/C//A	synonymous	0	1
S1PR2	chr19	10335094/C//T	nonsynonymous	0	1
ICAM1	chr19	10394255/G//A	nonsynonymous	0	1
TYK2	chr19	10465191/G//A	synonymous	0	1
CDC37	chr19	10506606/T//C	nonsynonymous	0	1
KANK2	chr19	11303802/C//T	nonsynonymous	0	1
RGL3	chr19	11512916/A//G	nonsynonymous	0	1
DHPS	chr19	12788210/T//A	nonsynonymous	0	1
DHPS	chr19	12791072/C//T	nonsynonymous	0	1
HOOK2	chr19	12878668/C//T	nonsynonymous	0	1
JUNB	chr19	12902436/G//A	synonymous	0	1
FARSA	chr19	13035326/G//A	nonsynonymous	0	1
NACC1	chr19	13246278/A//G	nonsynonymous	0	1
RFX1	chr19	14074087/G//A	synonymous	0	1
PKN1	chr19	14580619/G//A	nonsynonymous	0	1
ZNF333	chr19	14830122/G//A	synonymous	0	1
KCNN1	chr19	18092643/G//A	synonymous	0	1
GMIP	chr19	19744695/G//A	nonsynonymous	0	1
ZNF91	chr19	23543531/T//A	synonymous	0	1
ARHGAP33	chr19	36271872/C//T	nonsynonymous	0	1
ACTN4	chr19	39212332/C//T	synonymous	0	1
SPTBN4	chr19	40996001/G//A	nonsynonymous	0	1
MEGF8	chr19	42848196/G//A	nonsynonymous	0	1
APOE	chr19	45412298/G//A	nonsynonymous	0	1
PPP1R37	chr19	45646858/G//A	synonymous	0	1
PPM1N	chr19	46002284/C//T	nonsynonymous	0	1
QPCTL	chr19	46206267/G//A	nonsynonymous	0	1
PRKD2	chr19	47207834/C//T	nonsynonymous	0	1
ARHGAP35	chr19	47423198/G//T	nonsynonymous	0	1
NTN5	chr19	49165166/C//T	nonsynonymous	0	1
FLT3LG	chr19	49977874/G//T	synonymous	0	1
CPT1C	chr19	50204577/C//A	nonsynonymous	0	1
ZNF473	chr19	50548081/C//T	synonymous	0	1
POLD1	chr19	50912789/C//T	synonymous	0	1
SHANK1	chr19	51171129/G//A	nonsynonymous	0	1
EPS8L1	chr19	55598206/G//A	synonymous	0	1
PPP1R12C	chr19	55602640/G//A	synonymous	0	1
ZNF134	chr19	58132398/T//C	nonsynonymous	0	1
ZNF837	chr19	58879247/C//T	nonsynonymous	0	1
MZF1	chr19	59080905/G//A	nonsynonymous	0	1
RBCK1	chr20	398229/G//A	nonsynonymous	0	1
IDH3B	chr20	2641196/C//T	nonsynonymous	0	1
FASTKD5	chr20	3128656/C//T	nonsynonymous	0	1
LZTS3	chr20	3145465/C//T	nonsynonymous	0	1
ESF1	chr20	13763615/G//A	nonsynonymous	0	1
NINL	chr20	25484647/C//T	nonsynonymous	0	1
SNTA1	chr20	32031304/G//T	synonymous	0	1
MMP24	chr20	33839819/C//A	synonymous	0	1
DSN1	chr20	35381173/G//A	synonymous	0	1
TGM2	chr20	36766591/G//T	synonymous	0	1
PPP1R16B	chr20	37546998/G//A	nonsynonymous	0	1
JPH2	chr20	42744506/C//T	synonymous	0	1
ZNF334	chr20	45131009/A//G	synonymous	0	1

PREX1	chr20	47364404/C//T	nonsynonymous	0	1
SNAI1	chr20	48604570/G//A	nonsynonymous	0	1
TFAP2C	chr20	55206341/G//A	synonymous	0	1
SPO11	chr20	55904875/C//T	synonymous	0	1
GNAS	chr20	57428326/C//T	synonymous	0	1
LAMA5	chr20	60891033/G//A	nonsynonymous	0	1
DIDO1	chr20	61542401/C//T	synonymous	0	1
COL20A1	chr20	61941114/G//A	synonymous	0	1
SOX18	chr20	62679779/G//A	nonsynonymous	0	1
LTN1	chr21	30329802/A//G	synonymous	0	1
GRIK1	chr21	30971233/G//A	nonsynonymous	0	1
SON	chr21	34918513/G//A	synonymous	0	1
	chr21	36265220/A//G	synonymous	0	1
TFF2	chr21	43771051/C//T	nonsynonymous	0	1
UBASH3A	chr21	43838653/C//T	synonymous	0	1
SLC37A1	chr21	43963540/C//T	synonymous	0	1
CBSL	chr21	44474040/C//T	nonsynonymous	0	1
RRP1B	chr21	45107928/C//T	nonsynonymous	0	1
AIRE	chr21	45713744/G//A	nonsynonymous	0	1
MICAL3	chr22	18368670/C//T	nonsynonymous	0	1
TRMT2A	chr22	20100968/G//A	synonymous	0	1
KLHL22	chr22	20796627/G//T	synonymous	0	1
AIFM3	chr22	21329977/G//A	synonymous	0	1
HIC2	chr22	21800692/G//A	nonsynonymous	0	1
CCDC116	chr22	21989363/C//A	synonymous	0	1
ZNF280B	chr22	22843423/C//T	nonsynonymous	0	1
MYO18B	chr22	26388399/G//A	nonsynonymous	0	1
GAS2L1	chr22	29707952/C//T	nonsynonymous	0	1
LARGE1	chr22	34000543/G//A	nonsynonymous	0	1
SSTR3	chr22	37603678/G//A	synonymous	0	1
JOSD1	chr22	39095928/G//A	nonsynonymous	0	1
CHADL	chr22	41633559/C//T	nonsynonymous	0	1
TCF20	chr22	42607828/G//A	nonsynonymous	0	1
PRR5-ARHGAP8	chr22	45182492/G//A	synonymous	0	1
PHF21B	chr22	45312312/C//T	nonsynonymous	0	1
CELSR1	chr22	46931191/G//T	nonsynonymous	0	1
PLXNB2	chr22	50728676/C//T	nonsynonymous	0	1
PPP2R3B	chrX	347448/G//A	synonymous	0	1
MAGEB2	chrX	30236744/G//A	nonsynonymous	0	1
DDX3X	chrX	41204467/G//A	nonsynonymous	0	1
GATA1	chrX	48650883/C//T	synonymous	0	1
TRO	chrX	54957244/T//C	nonsynonymous	0	1
PHKA1	chrX	71864297/C//A	nonsynonymous	0	1
KIAA2022	chrX	73961577/T//C	nonsynonymous	0	1
BRWD3	chrX	79984249/A//G	nonsynonymous	0	1
RHOXF1	chrX	119249408/A//G	nonsynonymous	0	1
ZNF75D	chrX	134421629/T//G	nonsynonymous	0	1
BRS3	chrX	135570611/G//T	nonsynonymous	0	1
MAMLD1	chrX	149638916/A//C	synonymous	0	1
PASD1	chrX	150840104/G//A	synonymous	0	1
UTY	chrY	15417973/C//A	nonsynonymous	0	1
KIF17	chr1	21009263/C//T	synonymous	0	1
HEYL	chr1	40092321/G//A	nonsynonymous	0	1
NTRK1	chr1	156849867/G//A	nonsynonymous	0	1
NCSTN	chr1	160321968/G//A	nonsynonymous	0	1
FASTKD2	chr2	207652704/T//C	synonymous	0	1
ANO10	chr3	43591341/C//G	nonsynonymous	0	1
PCLO	chr7	82390770/T//A	nonsynonymous	0	1
C8orf74	chr8	10557965/C//T	nonsynonymous	0	1
CNTRL	chr9	123870180/A//G	synonymous	0	1
RTF1	chr15	41709342/C//T	nonsynonymous	0	1
GTF3C1	chr16	27506538/C//A	nonsynonymous	0	1
NOB1	chr16	69776309/C//G	nonsynonymous	0	1
	chr17	80904962/T//C	synonymous	0	1
ME2	chr18	48439194/A//G	nonsynonymous	0	1
	chr20	50179158/T//C	synonymous	0	1
TRMT2B	chrX	100297217/C//A	nonsynonymous	0	1
FLNA	chrX	153588741/G//A	nonsynonymous	0	1
LRRC47	chr1	3703599/C//T	synonymous	0	1
MTOR	chr1	11169361/C//G	nonsynonymous	0	1

UBR4	chr1	19482877/G//A	nonsynonymous	0	1
THRAP3	chr1	36769555/A//T	nonsynonymous	0	1
CSF3R	chr1	36934832/C//T	nonsynonymous	0	1
SLC5A9	chr1	48695052/A//G	nonsynonymous	0	1
WDR47	chr1	109545026/T//A	nonsynonymous	0	1
CELSR2	chr1	109804222/G//A	synonymous	0	1
OR10X1	chr1	158549238/A//G	nonsynonymous	0	1
SELP	chr1	169572304/A//T	synonymous	0	1
C1orf112	chr1	169820997/C//T	nonsynonymous	0	1
PAPPA2	chr1	176738902/C//T	nonsynonymous	0	1
NEK7	chr1	198262082/G//A	synonymous	0	1
LRRN2	chr1	204587549/G//A	synonymous	0	1
CNIH3	chr1	224918258/T//G	nonsynonymous	0	1
ARF1	chr1	228285714/A//G	nonsynonymous	0	1
GREM2	chr1	240656228/G//A	synonymous	0	1
TMEM247	chr2	46707760/G//A	nonsynonymous	0	1
BMP10	chr2	69093273/C//T	synonymous	0	1
MOGS	chr2	74692319/G//C	nonsynonymous	0	1
RGPD3	chr2	107040985/T//C	synonymous	0	1
NXPH2	chr2	139537756/C//T	synonymous	0	1
LRP1B	chr2	141004675/T//G	nonsynonymous	0	1
LRP1B	chr2	141473567/A//G	nonsynonymous	0	1
LRP1B	chr2	141665551/G//C	nonsynonymous	0	1
XIRP2	chr2	168106537/A//C	nonsynonymous	0	1
ZNF804A	chr2	185803858/G//A	synonymous	0	1
COL3A1	chr2	189875411/T//C	nonsynonymous	0	1
ALS2CR12	chr2	202153371/T//G	synonymous	0	1
ALS2CR11	chr2	202352441/A//G	nonsynonymous	0	1
CHL1	chr3	425526/G//T	nonsynonymous	0	1
SETMAR	chr3	4355071/G//A	nonsynonymous	0	1
NEK10	chr3	27343282/G//C	nonsynonymous	0	1
TRAIP	chr3	49879364/A//C	nonsynonymous	0	1
CEP97	chr3	101474304/C//A	synonymous	0	1
SIDT1	chr3	113335006/A//C	nonsynonymous	0	1
TMEM39A	chr3	119151018/A//C	nonsynonymous	0	1
LINC01565	chr3	128292265/A//G	synonymous	0	1
COL6A6	chr3	130380702/C//T	nonsynonymous	0	1
CCNL1	chr3	156877237/T//A	nonsynonymous	0	1
RNF212	chr4	1075206/C//A	nonsynonymous	0	1
PPARGC1A	chr4	23797471/C//T	nonsynonymous	0	1
LRRC66	chr4	52861782/A//C	nonsynonymous	0	1
SGCB	chr4	52890046/C//T	synonymous	0	1
WDFY3	chr4	85715821/G//A	nonsynonymous	0	1
NDST3	chr4	119161715/T//A	nonsynonymous	0	1
SEC24D	chr4	119745770/C//T	synonymous	0	1
GUCY1B3	chr4	156723524/T//G	synonymous	0	1
DNAH5	chr5	13901621/C//T	nonsynonymous	0	1
CMYA5	chr5	79030594/A//C	nonsynonymous	0	1
SLC27A6	chr5	128326109/G//T	synonymous	0	1
PCDHA3	chr5	140181496/C//T	synonymous	0	1
STK10	chr5	171533645/G//A	nonsynonymous	0	1
OR12D2	chr6	29365080/G//A	nonsynonymous	0	1
LGSN	chr6	63990218/G//C	nonsynonymous	0	1
NKAIN2	chr6	125112532/A//C	nonsynonymous	0	1
HINT3	chr6	126296015/T//C	nonsynonymous	0	1
RSPH3	chr6	159421015/G//A	synonymous	0	1
VWDE	chr7	12376686/T//G	nonsynonymous	0	1
SKAP2	chr7	26904037/G//C	synonymous	0	1
MYL7	chr7	44179386/C//A	nonsynonymous	0	1
ABCA13	chr7	48315615/A//T	nonsynonymous	0	1
ABCA13	chr7	48378049/T//C	nonsynonymous	0	1
CYP3A5	chr7	99247847/T//G	nonsynonymous	0	1
MCM7	chr7	99695536/C//T	synonymous	0	1
TAF6	chr7	99711291/G//A	synonymous	0	1
SNAI2	chr8	49832510/G//A	synonymous	0	1
SULF1	chr8	70540097/C//A	nonsynonymous	0	1
PRDM14	chr8	70981551/G//A	nonsynonymous	0	1
OTUD6B	chr8	92092954/TTGAGGTAAGTTTGTA	nonsynonymous	0	1
ADCY8	chr8	132051710/G//C	synonymous	0	1
ARC	chr8	143695314/G//A	nonsynonymous	0	1

EEF1D	chr8	144669019/T//C	synonymous	0	1
ARHGAP39	chr8	145772787/C//A	nonsynonymous	0	1
ACO1	chr9	32408615/A//G	nonsynonymous	0	1
ZNF189	chr9	104171713/C//G	nonsynonymous	0	1
KLF4	chr9	110249544/C//T	synonymous	0	1
OR1K1	chr9	125562933/T//G	nonsynonymous	0	1
ABL1	chr9	133761044/G//A	nonsynonymous	0	1
WDFY4	chr10	49984922/G//C	nonsynonymous	0	1
WDFY4	chr10	49985007/G//C	nonsynonymous	0	1
C10orf71	chr10	50531657/C//A	nonsynonymous	0	1
ARL3	chr10	104436669/G//A	synonymous	0	1
FAM175B	chr10	126490438/T//C	nonsynonymous	0	1
OR51V1	chr11	5221722/T//A	nonsynonymous	0	1
TUB	chr11	8120325/A//C	nonsynonymous	0	1
OR4C13	chr11	49974877/T//A	nonsynonymous	0	1
SHANK2	chr11	70653176/C//T	nonsynonymous	0	1
SLCO2B1	chr11	74915475/A//G	synonymous	0	1
PAFAH1B2	chr11	117038186/C//A	nonsynonymous	0	1
SORL1	chr11	121429469/C//T	nonsynonymous	0	1
OR10S1	chr11	123847696/TGAA//T	nonsynonymous	0	1
PZP	chr12	9310401/C//A	nonsynonymous	0	1
PIK3C2G	chr12	18715683/T//G	nonsynonymous	0	1
AMN1	chr12	31850315/G//A	nonsynonymous	0	1
NELL2	chr12	45105162/G//T	nonsynonymous	0	1
KMT2D	chr12	49447055/C//A	nonsynonymous	0	1
AMHR2	chr12	53825073/A//G	nonsynonymous	0	1
RASSF9	chr12	86199033/T//G	nonsynonymous	0	1
TMCC3	chr12	94965451/T//G	nonsynonymous	0	1
CAMKK2	chr12	121711939/C//T	nonsynonymous	0	1
PITPNM2	chr12	123485680/C//T	nonsynonymous	0	1
MPHOSPH9	chr12	123678990/G//A	synonymous	0	1
POLE	chr12	133201305/T//A	nonsynonymous	0	1
RNF17	chr13	25435416/T//A	nonsynonymous	0	1
NBEA	chr13	36006465/G//A	nonsynonymous	0	1
GPR183	chr13	99948240/C//A	nonsynonymous	0	1
ITGBL1	chr13	102231737/G//A	nonsynonymous	0	1
IRS2	chr13	110434954/G//A	synonymous	0	1
RNF31	chr14	24617291/T//G	nonsynonymous	0	1
FAM177A1	chr14	35515734/C//T	synonymous	0	1
ERO1A	chr14	53133093/T//G	nonsynonymous	0	1
AREL1	chr14	75139812/G//T	nonsynonymous	0	1
YLPM1	chr14	75245248/G//T	nonsynonymous	0	1
ATG2B	chr14	96798984/C//T	nonsynonymous	0	1
ATG2B	chr14	96798993/C//G	nonsynonymous	0	1
CYFIP1	chr15	22960813/A//G	nonsynonymous	0	1
ZSCAN29	chr15	43656313/C//T	nonsynonymous	0	1
FBN1	chr15	48703440/G//A	nonsynonymous	0	1
UNC13C	chr15	54306141/T//G	synonymous	0	1
CCDC78	chr16	774980/C//T	synonymous	0	1
USP7	chr16	9014215/C//T	nonsynonymous	0	1
CDH11	chr16	64984688/A//C	nonsynonymous	0	1
PHLPP2	chr16	71686829/G//A	nonsynonymous	0	1
CYBA	chr16	88709701/G//C	synonymous	0	1
NLRP1	chr17	5436279/C//T	synonymous	0	1
MYO15A	chr17	18023636/G//A	nonsynonymous	0	1
TBX4	chr17	59534026/G//A	nonsynonymous	0	1
SCN4A	chr17	62019236/C//T	nonsynonymous	0	1
TMEM105	chr17	79287759/C//G	nonsynonymous	0	1
TXNDC2	chr18	9886658/A//T	nonsynonymous	0	1
OSBPL1A	chr18	21921577/C//A	nonsynonymous	0	1
LOXHD1	chr18	44229200/G//A	nonsynonymous	0	1
ADGRE2	chr19	14862338/T//G	nonsynonymous	0	1
HSH2D	chr19	16268205/G//A	synonymous	0	1
AP1M1	chr19	16319964/C//T	synonymous	0	1
LGALS14	chr19	40195160/G//C	synonymous	0	1
MEGF8	chr19	42875551/G//A	nonsynonymous	0	1
PSG11	chr19	43529202/A//C	nonsynonymous	0	1
HAS1	chr19	52222852/G//C	synonymous	0	1
RALGAPA2	chr20	20486184/A//C	synonymous	0	1
CST8	chr20	23472335/C//T	nonsynonymous	0	1

NCOA5	chr20	44708080/G//T	synonymous	0	1
SALL4	chr20	50408075/T//A	nonsynonymous	0	1
TPTE	chr21	10943025/G//C	synonymous	0	1
RSPH1	chr21	43913192/G//C	synonymous	0	1
LZTR1	chr22	21337331/A//T	synonymous	0	1
SH3BP1	chr22	38039722/C//T	nonsynonymous	0	1
C22orf23	chr22	38347484/ATGTGGCGCTGCTG//A	nonsynonymous	0	1
KDM5C	chrX	53230858/C//T	synonymous	0	1
ZDHHC15	chrX	74670630/A//G	synonymous	0	1
USP26	chrX	132161785/A//T	nonsynonymous	0	1
CDC14B	chr9	99272029/T//C	synonymous	0	1
KREMEN2	chr16	3018055/C//T	synonymous	0	1
CLEC18B	chr16	74451990/A//G	synonymous	0	1
CDC27	chr17	45214643/A//C	synonymous	0	1
CDC27	chr17	45214648/G//C	nonsynonymous	0	1
CDC27	chr17	45214651/T//G	nonsynonymous	0	1
CDC27	chr17	45214654/C//T	nonsynonymous	0	1
ZNF83	chr19	53116867/G//A	synonymous	0	1
TTLL10	chr1	1132059/T//C	nonsynonymous	0	1
EXOSC10	chr1	11147946/T//C	nonsynonymous	0	1
POU2F1	chr1	167381461/A//G	synonymous	0	1
	chr1	247150512/G//A	synonymous	0	1
SLC16A14	chr2	230914479/G//A	nonsynonymous	0	1
CAND2	chr3	12856892/C//A	nonsynonymous	0	1
	chr4	38054802/T//C	synonymous	0	1
SEC24B	chr4	110402912/AGAG//A	nonsynonymous	0	1
ADAMTS19	chr5	128797427/T//C	nonsynonymous	0	1
NEUROG1	chr5	134871211/C//T	nonsynonymous	0	1
PCDHGC5	chr5	140869419/C//A	synonymous	0	1
FREM1	chr9	14797646/A//T	synonymous	0	1
MUC5B	chr11	1264742/G//C	nonsynonymous	0	1
TNKS1BP1	chr11	57080868/C//G	nonsynonymous	0	1
AHNAK2	chr14	105411957/G//A	synonymous	0	1
VPS4B	chr18	61089516/A//G	synonymous	0	1
DNMT1	chr19	10262449/G//T	nonsynonymous	0	1
MAST1	chr19	12984488/C//T	nonsynonymous	0	1
ZNF208	chr19	22156143/A//G	nonsynonymous	0	1
GSPT2	chrX	51488362/C//G	nonsynonymous	0	1
NONO	chrX	70516465/A//G	nonsynonymous	0	1
TGIF2LX	chrX	89177249/A//G	synonymous	0	1
THOC2	chrX	122802038/T//C	nonsynonymous	0	1
CASR	chr3	122002833/C//T	nonsynonymous	1	0
VEGFC	chr4	177609015/C//A	synonymous	1	0
ZNF622	chr5	16463367/C//G	nonsynonymous	1	0
THSD7A	chr7	11630234/G//T	nonsynonymous	1	0
ZNF273	chr7	64389263/T//C	synonymous	1	0
SAMD9	chr7	92733853/C//A	nonsynonymous	1	0
DLX6	chr7	96635585/C//A	nonsynonymous	1	0
TAF6	chr7	99705993/C//A	synonymous	1	0
SLC12A9	chr7	100459499/G//T	synonymous	1	0
MUC17	chr7	100676260/G//A	nonsynonymous	1	0
MUC17	chr7	100676262/C//T	nonsynonymous	1	0
MUC17	chr7	100682639/T//C	nonsynonymous	1	0
GCC1	chr7	127224254/G//A	nonsynonymous	1	0
FSCN3	chr7	127236496/C//G	nonsynonymous	1	0
DCSTAMP	chr8	105361626/T//C	synonymous	1	0
WDYHV1	chr8	124440257/G//T	nonsynonymous	1	0
ZNF7	chr8	146066742/T//C	nonsynonymous	1	0
STARD9	chr15	43008875/G//C	nonsynonymous	1	0
CA12	chr15	63637706/G//C	nonsynonymous	1	0
	chr15	65992923/C//A	synonymous	1	0
CACNA1G	chr17	48652912/C//A	synonymous	1	0
RASIP1	chr19	49232760/A//AC	nonsynonymous	1	0
PPFIA3	chr19	49632690/G//T	nonsynonymous	1	0
LZTR1	chr22	21346649/T//C	synonymous	1	0
MCF2	chrX	138724739/A//G	synonymous	1	0
PLCH2	chr1	2428283/G//C	nonsynonymous	0	1
OR2AK2	chr1	248128832/A//G	nonsynonymous	0	1
SP140	chr2	231115734/G//T	nonsynonymous	0	1
ABI3BP	chr3	100569519/G//A	nonsynonymous	0	1

LRRC15	chr3	194081215/G//A	synonymous	0	1
ARHGAP24	chr4	86491743/C//T	nonsynonymous	0	1
SEC24B	chr4	110448573/C//G	nonsynonymous	0	1
KIAA1109	chr4	123166278/A//G	nonsynonymous	0	1
SLC25A2	chr5	140683333/T//A	nonsynonymous	0	1
TENM2	chr5	167645219/C//T	synonymous	0	1
CEP162	chr6	84884484/A//G	nonsynonymous	0	1
CTTNBP2	chr7	117407125/G//A	synonymous	0	1
GTF3C4	chr9	135546293/T//C	nonsynonymous	0	1
VAV2	chr9	136635554/A//T	nonsynonymous	0	1
CAMK1D	chr10	12870807/C//T	nonsynonymous	0	1
CDH23	chr10	73491904/C//T	synonymous	0	1
NRAP	chr10	115368195/A//C	nonsynonymous	0	1
LRRC55	chr11	56954716/C//T	synonymous	0	1
RSF1	chr11	77451933/T//G	nonsynonymous	0	1
TAGLN	chr11	117074140/G//A	nonsynonymous	0	1
	chr11	128804929/C//G	synonymous	0	1
FAIM2	chr12	50264148/C//G	synonymous	0	1
LOC283710	chr15	31522990/A//G	synonymous	0	1
MAPKBP1	chr15	42117624/G//A	nonsynonymous	0	1
CNDP2	chr18	72180911/T//C	nonsynonymous	0	1
TSHZ1	chr18	72998791/C//T	nonsynonymous	0	1
PCSK4	chr19	1490397/C//T	synonymous	0	1
ZNF266	chr19	9523870/T//TA	synonymous	0	1
DOCK6	chr19	11332540/C//T	synonymous	0	1
ALKBH6	chr19	36501839/T//C	nonsynonymous	0	1
PLCB4	chr20	9389753/G//T	nonsynonymous	0	1
RNF220	chr1	45101728/T//C	synonymous	0	1
PGLYRP3	chr1	153270488/A//G	nonsynonymous	0	1
LMLN	chr3	197687156/T//C	nonsynonymous	0	1
FAT4	chr4	126389938/A//G	synonymous	0	1
MCUR1	chr6	13792139/T//C	nonsynonymous	0	1
AGPAT5	chr8	6588351/A//G	synonymous	0	1
LY96	chr8	74917041/A//G	synonymous	0	1
HDHD3	chr9	116136099/A//C	nonsynonymous	0	1
TSPAN18	chr11	44950703/A//G	nonsynonymous	0	1
BTBD18	chr11	57511734/T//C	nonsynonymous	0	1
ABCD2	chr12	39947686/A//G	synonymous	0	1
SBNO1	chr12	123834868/A//G	nonsynonymous	0	1
STX4	chr16	31045861/A//G	synonymous	0	1
SCARF1	chr17	1538482/A//G	nonsynonymous	0	1
SPATA20	chr17	48628085/G//A	nonsynonymous	0	1
MAP3K3	chr17	61771097/A//G	nonsynonymous	0	1
RNF213	chr17	78263537/A//G	nonsynonymous	0	1
CYP4F3	chr19	15770027/A//T	synonymous	0	1
CFAP61	chr20	20232263/T//C	synonymous	0	1
GABPA	chr21	27117611/A//G	synonymous	0	1
KRTAP10-5	chr21	45999963/A//T	nonsynonymous	0	1
A4GALT	chr22	43089941/T//C	nonsynonymous	0	1
KLHL15	chrX	24024389/T//C	nonsynonymous	0	1
GNB1	chr1	1756910/C//T	synonymous	0	1
OR10Z1	chr1	158576955/T//G	nonsynonymous	0	1
EXOC8	chr1	231471992/G//A	synonymous	0	1
CCDC141	chr2	179702381/C//T	nonsynonymous	0	1
PGAP1	chr2	197729782/G//C	nonsynonymous	0	1
OBSL1	chr2	220427387/G//A	nonsynonymous	0	1
ALG1L2	chr3	129810972/C//T	nonsynonymous	0	1
RASGEF1B	chr4	82368684/G//A	nonsynonymous	0	1
KDM3B	chr5	137727034/C//T	synonymous	0	1
ICK	chr6	52874346/G//A	synonymous	0	1
EPHA7	chr6	93956542/A//C	nonsynonymous	0	1
ESR1	chr6	152265445/C//T	nonsynonymous	0	1
TRRAP	chr7	98610000/G//A	synonymous	0	1
PENK	chr8	57354070/A//C	nonsynonymous	0	1
C8orf87	chr8	94179040/G//T	synonymous	0	1
ADGRB1	chr8	143546219/G//A	synonymous	0	1
PFKP	chr10	3174577/C//T	synonymous	0	1
FAM149B1	chr10	74937633/T//G	nonsynonymous	0	1
FGFR2	chr10	123256169/C//G	nonsynonymous	0	1
OVCH2	chr11	7726152/A//T	nonsynonymous	0	1

PAX6	chr11	31823185/C//A	nonsynonymous	0	1
GRAMD1B	chr11	123464880/T//A	synonymous	0	1
KRT84	chr12	52779085/C//A	synonymous	0	1
PTPRQ	chr12	80899949/A//C	nonsynonymous	0	1
DMXL2	chr15	51828855/G//A	nonsynonymous	0	1
WDR72	chr15	53815505/C//A	nonsynonymous	0	1
ZSCAN10	chr16	3139914/G//A	synonymous	0	1
MGRN1	chr16	4714719/G//T	nonsynonymous	0	1
CSNK1D	chr17	80206849/G//A	nonsynonymous	0	1
DSC1	chr18	28711608/G//T	synonymous	0	1
TRMT1	chr19	13221039/C//T	nonsynonymous	0	1
ZNF208	chr19	22154503/T//C	synonymous	0	1
SULT2B1	chr19	49094891/G//A	nonsynonymous	0	1
C19orf84	chr19	51893761/T//C	synonymous	0	1
ZNF836	chr19	52660639/C//T	synonymous	0	1
MGAT3	chr22	39883471/C//T	nonsynonymous	0	1
GRIPAP1	chrX	48839501/G//A	nonsynonymous	0	1
L1CAM	chrX	153134029/G//A	synonymous	0	1
CTNNA2	chr2	80136928/G//A	synonymous	0	1
CCNT2	chr2	135712242/C//T	synonymous	0	1
BAP1	chr3	52436267/C//T	synonymous	0	1
SMC4	chr3	160130142/A//G	nonsynonymous	0	1
AFF1	chr4	88029402/TCAGA//T	nonsynonymous	0	1
GHR	chr5	42719249/C//A	nonsynonymous	0	1
RNF5	chr6	32148107/A//G	synonymous	0	1
ADGRB3	chr6	69666056/G//T	nonsynonymous	0	1
WIPI2	chr7	5270618/T//C	synonymous	0	1
MUC17	chr7	100684790/G//A	nonsynonymous	0	1
VPS13B	chr8	100829981/A//G	nonsynonymous	0	1
SLC38A2	chr12	46757700/TACTC//T	synonymous	0	1
FGD6	chr12	95603492/G//C	nonsynonymous	0	1
KIAA0556	chr16	27763232/G//A	nonsynonymous	0	1
TEN1	chr17	73996244/G//A	synonymous	0	1
LOXHD1	chr18	44146331/G//A	nonsynonymous	0	1
S1PR4	chr19	3179191/T//C	nonsynonymous	0	1
ADAMTS10	chr19	8650345/A//G	nonsynonymous	0	1
ZBTB45	chr19	59025341/A//G	synonymous	0	1
SS18L1	chr20	60738567/G//A	nonsynonymous	0	1
RPS6KA3	chrX	20183018/T//C	nonsynonymous	0	1
ARHGEF10L	chr1	17934289/G//A	synonymous	0	1
C1orf94	chr1	34663360/G//C	synonymous	0	1
SLC1A7	chr1	53569209/G//T	nonsynonymous	0	1
PDE4DIP	chr1	144856900/C//T	synonymous	0	1
SCAMP3	chr1	155226201/A//C	nonsynonymous	0	1
NPHS2	chr1	179530429/C//T	nonsynonymous	0	1
NCF2	chr1	183542323/C//T	synonymous	0	1
EIF2D	chr1	206776391/C//T	nonsynonymous	0	1
FMN2	chr1	240255801/C//T	nonsynonymous	0	1
TPO	chr2	1507741/G//T	nonsynonymous	0	1
PXDN	chr2	1648512/C//G	synonymous	0	1
ITSN2	chr2	24521629/G//A	nonsynonymous	0	1
CLIP4	chr2	29404441/G//A	synonymous	0	1
CWC22	chr2	180838512/G//T	nonsynonymous	0	1
CREB1	chr2	208461769/A//G	nonsynonymous	0	1
SPAG16	chr2	214794853/A//T	nonsynonymous	0	1
IGFBP5	chr2	217541438/T//A	synonymous	0	1
EPHA4	chr2	222301844/T//C	nonsynonymous	0	1
RBP1	chr3	139258420/C//G	nonsynonymous	0	1
ARHGEF26	chr3	153912502/A//C	nonsynonymous	0	1
TMEM175	chr4	945016/A//G	synonymous	0	1
SH3TC1	chr4	8218826/G//A	synonymous	0	1
CEP135	chr4	56865746/C//G	nonsynonymous	0	1
GRIA2	chr4	158284007/C//T	synonymous	0	1
ADAMTS12	chr5	33596160/G//A	nonsynonymous	0	1
ZFP62	chr5	180277836/G//T	nonsynonymous	0	1
BTN2A1	chr6	26468702/C//G	synonymous	0	1
GPX5	chr6	28501782/T//C	synonymous	0	1
MRPS18B	chr6	30593463/T//C	synonymous	0	1
CD109	chr6	74440269/A//G	nonsynonymous	0	1
LCA5	chr6	80223150/C//A	nonsynonymous	0	1

SYNE1	chr6	152652849/C//T	nonsynonymous	0	1
DNAH11	chr7	21939024/G//A	nonsynonymous	0	1
STK31	chr7	23823196/C//A	synonymous	0	1
ASB15	chr7	123269198/C//G	nonsynonymous	0	1
MYBL1	chr8	67477030/C//T	nonsynonymous	0	1
ZNF16	chr8	146157094/C//T	nonsynonymous	0	1
ARMC3	chr10	23326314/C//A	nonsynonymous	0	1
EGR2	chr10	64572891/G//T	synonymous	0	1
JMJD1C	chr10	64973993/A//C	nonsynonymous	0	1
SLIT1	chr10	98763926/T//C	nonsynonymous	0	1
R3HCC1L	chr10	99968930/T//C	synonymous	0	1
ASRGL1	chr11	62124620/A//C	synonymous	0	1
VEGFB	chr11	64005838/C//T	synonymous	0	1
TMTC1	chr12	29673632/C//A	nonsynonymous	0	1
KIF21A	chr12	39740293/C//A	nonsynonymous	0	1
SSH1	chr12	109186370/A//C	nonsynonymous	0	1
CIT	chr12	120152003/G//A	synonymous	0	1
RPLP0	chr12	120638605/A//G	synonymous	0	1
GSX1	chr13	28368072/C//T	nonsynonymous	0	1
BIVM-ERCC5	chr13	103528250/C//T	synonymous	0	1
PCNX1	chr14	71500737/A//C	nonsynonymous	0	1
RTF1	chr15	41750023/G//A	nonsynonymous	0	1
UNC45A	chr15	91479641/A//C	nonsynonymous	0	1
CDIP1	chr16	4562631/A//G	nonsynonymous	0	1
CES2	chr16	66972112/T//C	synonymous	0	1
WNT3	chr17	44845917/G//T	nonsynonymous	0	1
COL1A1	chr17	48272129/G//A	nonsynonymous	0	1
RNF213	chr17	78321349/T//C	nonsynonymous	0	1
ADGRE1	chr19	6887606/A//G	synonymous	0	1
OLFM2	chr19	9968104/A//C	nonsynonymous	0	1
CCDC105	chr19	15121827/C//T	nonsynonymous	0	1
NOTCH3	chr19	15276255/C//T	synonymous	0	1
ZNF676	chr19	22362888/C//G	nonsynonymous	0	1
ZNF676	chr19	22362898/C//A	nonsynonymous	0	1
ZNF223	chr19	44564724/G//T	synonymous	0	1
C5AR1	chr19	47823135/G//A	nonsynonymous	0	1
ZNF480	chr19	52825419/C//G	nonsynonymous	0	1
RALGAPB	chr20	37191223/G//C	nonsynonymous	0	1
APOBEC3C	chr22	39414309/GATA//G	nonsynonymous	0	1
MEI1	chr22	42154467/G//C	nonsynonymous	0	1
MOV10L1	chr22	50580514/G//C	nonsynonymous	0	1
USP51	chrX	55513535/A//G	nonsynonymous	0	1
DGAT2L6	chrX	69419128/T//C	synonymous	0	1
ATRX	chrX	76855247/C//G	nonsynonymous	0	1
LRCH2	chrX	114414073/C//T	nonsynonymous	0	1
FMR1	chrX	147011561/G//T	nonsynonymous	0	1
MAGEA10	chrX	151303608/T//C	nonsynonymous	0	1
ACAP3	chr1	1229464/T//C	synonymous	0	1
AURKAIP1	chr1	1309159/G//T	synonymous	0	1
ILDR2	chr1	166927263/C//T	nonsynonymous	0	1
MAP3K2	chr2	128087571/T//C	nonsynonymous	0	1
KCNH7	chr2	163374576/C//T	nonsynonymous	0	1
CHRNA1	chr2	175612935/G//A	nonsynonymous	0	1
C2orf54	chr2	241829520/C//T	nonsynonymous	0	1
SETD2	chr3	47108598/C//T	nonsynonymous	0	1
STAB1	chr3	52544193/TCTC//T	nonsynonymous	0	1
PDGFC	chr4	157693833/C//T	synonymous	0	1
CDH6	chr5	31317954/C//T	nonsynonymous	0	1
PCDHB13	chr5	140594626/G//A	nonsynonymous	0	1
SNAP91	chr6	84366469/C//A	synonymous	0	1
ADCY1	chr7	45701752/G//A	nonsynonymous	0	1
TRPV5	chr7	142627457/G//A	synonymous	0	1
DNAJC25	chr9	114393692/G//C	nonsynonymous	0	1
ITIH5	chr10	7605017/T//G	synonymous	0	1
FAM170B	chr10	50341994/C//T	synonymous	0	1
TCF7L2	chr10	114912149/C//T	nonsynonymous	0	1
MUC5B	chr11	1272570/T//A	synonymous	0	1
CAPN1	chr11	64950983/C//G	nonsynonymous	0	1
P4HA3	chr11	73979206/C//A	synonymous	0	1
KMT2D	chr12	49426280/A//T	nonsynonymous	0	1

PPFIA2	chr12	81751992/G//A	nonsynonymous	0	1
RALGAPA1	chr14	36278108/T//C	synonymous	0	1
TSHR	chr14	81609934/C//T	nonsynonymous	0	1
BCL11B	chr14	99640811/T//C	nonsynonymous	0	1
MAN2A2	chr15	91449992/G//T	nonsynonymous	0	1
TP53	chr17	7578381/TGAGCAGCGCTCATGG	nonsynonymous	0	1
DNAH2	chr17	7680960/A//G	synonymous	0	1
TOM1L1	chr17	52978229/G//T	nonsynonymous	0	1
LGALS3BP	chr17	76972097/A//G	nonsynonymous	0	1
RNF213	chr17	78311721/A//G	nonsynonymous	0	1
SMCHD1	chr18	2694612/G//A	nonsynonymous	0	1
SMAD7	chr18	46448268/A//G	nonsynonymous	0	1
NR2F6	chr19	17343260/G//A	synonymous	0	1
HKR1	chr19	37835677/G//A	nonsynonymous	0	1
HRC	chr19	49657345/T//C	nonsynonymous	0	1
A1BG	chr19	58861853/A//G	nonsynonymous	0	1
LBP	chr20	36982695/G//T	nonsynonymous	0	1
EYA2	chr20	45702829/C//G	nonsynonymous	0	1
DIDO1	chr20	61525207/G//A	nonsynonymous	0	1
CACNG2	chr22	36960800/G//A	synonymous	0	1
PLCXD1	chrX	209787/A//G	nonsynonymous	0	1
CSF2RA	chrX	1407734/G//A	synonymous	0	1
CSF1	chr1	110465892/C//T	nonsynonymous	0	1
ARNT	chr1	150849036/G//C	nonsynonymous	0	1
GRM7	chr3	6903418/G//A	nonsynonymous	0	1
PTPN23	chr3	47448087/C//T	synonymous	0	1
C3orf22	chr3	126272281/A//G	synonymous	0	1
GPR149	chr3	154055972/G//T	nonsynonymous	0	1
SI	chr3	164712053/A//G	synonymous	0	1
MSX1	chr4	4864599/C//T	nonsynonymous	0	1
GRID2	chr4	94344060/T//G	nonsynonymous	0	1
APC	chr5	112128206/TC//T	nonsynonymous	0	1
PCDHGA5	chr5	140745223/A//C	synonymous	0	1
MELK	chr9	36633188/C//T	synonymous	0	1
SCAI	chr9	127733651/G//A	nonsynonymous	0	1
RNF169	chr11	74460312/G//A	synonymous	0	1
RILPL1	chr12	124017805/G//A	synonymous	0	1
SPG11	chr15	44876252/C//T	nonsynonymous	0	1
SLC43A2	chr17	1531170/G//A	synonymous	0	1
SLC26A11	chr17	78199707/A//G	synonymous	0	1
ANO8	chr19	17435562/C//T	nonsynonymous	0	1
FCGBP	chr19	40430514/C//T	nonsynonymous	0	1
ZIM3	chr19	57646336/C//T	nonsynonymous	0	1
ZNF530	chr19	58117835/A//T	nonsynonymous	0	1
AAR2	chr20	34828241/G//A	nonsynonymous	0	1
PAXBP1	chr21	34123526/T//G	nonsynonymous	0	1
IGSF1	chrX	130408613/C//T	nonsynonymous	0	1
MCF2	chrX	138669937/T//A	nonsynonymous	0	1
KCNH7	chr2	163374251/T//C	nonsynonymous	0	1
DHX36	chr3	154042007/C//T	nonsynonymous	0	1
DCTN4	chr5	150110638/T//C	nonsynonymous	0	1
VARS2	chr6	30882929/T//C	synonymous	0	1
MICAL1	chr6	109775394/A//T	synonymous	0	1
PTPRK	chr6	128330369/G//A	nonsynonymous	0	1
HOXA1	chr7	27135437/T//C	nonsynonymous	0	1
AASS	chr7	121756788/A//C	nonsynonymous	0	1
ZNF425	chr7	148802010/G//A	nonsynonymous	0	1
IPPK	chr9	95396747/G//A	nonsynonymous	0	1
XPA	chr9	100459581/C//A	synonymous	0	1
FZD8	chr10	35930179/T//C	nonsynonymous	0	1
TALDO1	chr11	747450/G//A	synonymous	0	1
NUP98	chr11	3793076/C//T	nonsynonymous	0	1
LRP5	chr11	68115681/C//T	nonsynonymous	0	1
NLRX1	chr11	119045358/A//G	nonsynonymous	0	1
VWF	chr12	6172164/C//T	nonsynonymous	0	1
HECTD4	chr12	112623041/C//T	synonymous	0	1
ATP6V0A2	chr12	124203253/G//A	synonymous	0	1
AHNAK2	chr14	105409721/C//T	nonsynonymous	0	1
PRTG	chr15	55929504/T//TG	nonsynonymous	0	1
SCAPER	chr15	77021000/G//A	nonsynonymous	0	1

ZFHX3	chr16	72831900/G//A	synonymous	0	1
PIEZO1	chr16	88783576/T//C	nonsynonymous	0	1
RNF135	chr17	29298258/G//T	nonsynonymous	0	1
HDAC5	chr17	42160935/T//C	nonsynonymous	0	1
TBC1D16	chr17	77984383/A//G	nonsynonymous	0	1
CBLN2	chr18	70209237/G//A	synonymous	0	1
KRTAP10-1	chr21	45959724/A//G	nonsynonymous	0	1
SHOX	chrX	591627/C//T	synonymous	0	1
ESPN	chr1	6511917/T//C	nonsynonymous	0	1
FLG	chr1	152282662/C//T	nonsynonymous	0	1
DENND4B	chr1	153914749/C//G	nonsynonymous	0	1
LYST	chr1	235969483/CAAGCCTATAA//C	nonsynonymous	0	1
TRIM58	chr1	248039736/G//T	nonsynonymous	0	1
OR2L3	chr1	248224618/C//G	nonsynonymous	0	1
ZNF385D	chr3	21552504/C//T	synonymous	0	1
ARIH2	chr3	49006000/G//A	nonsynonymous	0	1
PRR27	chr4	71024177/A//G	nonsynonymous	0	1
MRPL1	chr4	78830471/A//G	nonsynonymous	0	1
CYP21A2	chr6	31975246/A//G	nonsynonymous	0	1
FAM184A	chr6	119345743/T//G	nonsynonymous	0	1
CUX1	chr7	101740713/C//G	nonsynonymous	0	1
C7orf55-LUC7L2	chr7	139094332/G//A	synonymous	0	1
RSPO2	chr8	109001362/G//A	nonsynonymous	0	1
PLEC	chr8	145007382/G//A	synonymous	0	1
CNTNAP3B	chr9	43861150/G//A	nonsynonymous	0	1
TLE1	chr9	84208155/T//C	nonsynonymous	0	1
PPP1R32	chr11	61249390/G//A	nonsynonymous	0	1
SRRM4	chr12	119568591/G//A	synonymous	0	1
CARS2	chr13	111335443/A//G	nonsynonymous	0	1
RPS6KL1	chr14	75376355/C//T	synonymous	0	1
AHNAK2	chr14	105417837/A//G	synonymous	0	1
HERC2	chr15	28463760/A//G	nonsynonymous	0	1
KLHL25	chr15	86312695/T//C	nonsynonymous	0	1
ENPP7	chr17	77709384/G//A	synonymous	0	1
LMNB2	chr19	2431858/T//C	nonsynonymous	0	1
LMTK3	chr19	49004271/A//G	nonsynonymous	0	1
L3MBTL1	chr20	42143284/A//C	nonsynonymous	0	1
ADAMTS1	chr21	28212236/G//A	nonsynonymous	0	1
TIAM1	chr21	32595738/G//T	nonsynonymous	0	1
HDX	chrX	83591858/G//T	nonsynonymous	0	1
PRAMEF1	chr1	12855721/G//A	nonsynonymous	0	1
FAM46B	chr1	27332618/C//T	synonymous	0	1
ATXN7L2	chr1	110030371/T//C	synonymous	0	1
C1orf198	chr1	230979385/C//A	nonsynonymous	0	1
EIF2AK3	chr2	88870428/T//A	synonymous	0	1
CCDC141	chr2	179701781/G//A	nonsynonymous	0	1
FN1	chr2	216259331/G//T	nonsynonymous	0	1
SEPT2	chr2	242277146/C//T	nonsynonymous	0	1
NBEAL2	chr3	47041434/C//G	nonsynonymous	0	1
ATP13A3	chr3	194175013/C//T	nonsynonymous	0	1
UNC5C	chr4	96199504/T//G	nonsynonymous	0	1
PCDHA12	chr5	140257256/G//A	synonymous	0	1
PGK2	chr6	49753902/T//A	synonymous	0	1
SOBP	chr6	107955326/G//A	synonymous	0	1
SF3B5	chr6	144416555/G//A	nonsynonymous	0	1
PCLO	chr7	82586093/G//T	synonymous	0	1
ZAN	chr7	100331777/C//G	synonymous	0	1
MCPH1	chr8	6299584/T//G	synonymous	0	1
GLI4	chr8	144358341/A//G	synonymous	0	1
WNK2	chr9	96051273/A//G	nonsynonymous	0	1
SDHAF2	chr11	61205189/C//A	synonymous	0	1
MYO16	chr13	109535423/T//A	nonsynonymous	0	1
ZNF839	chr14	102792579/G//T	synonymous	0	1
RPLP1	chr15	69745341/C//T	synonymous	0	1
	chr15	74327709/C//T	synonymous	0	1
RAB40C	chr16	677575/CAG//C	nonsynonymous	0	1
CRISPLD2	chr16	84883102/C//T	synonymous	0	1
HOXB3	chr17	46627936/G//A	synonymous	0	1
LOXHD1	chr18	44157825/G//A	synonymous	0	1
PNPLA6	chr19	7621540/G//A	nonsynonymous	0	1

LDLR	chr19	11200178/G//A	synonymous	0	1
PSG6	chr19	43420585/G//C	nonsynonymous	0	1
CKM	chr19	45810055/ACTT//A	nonsynonymous	0	1
TMC4	chr19	54664092/AAGACTT//A	nonsynonymous	0	1
NLRP8	chr19	56487554/T//C	nonsynonymous	0	1
EYA2	chr20	45633652/C//T	nonsynonymous	0	1
IDS	chrX	148579841/T//A	synonymous	0	1
TMIGD3	chr1	112031612/G//T	nonsynonymous	0	1
ROBO2	chr3	77693915/C//A	nonsynonymous	0	1
SEC61A1	chr3	127779466/G//A	nonsynonymous	0	1
KIAA0232	chr4	6882650/T//C	nonsynonymous	0	1
GUF1	chr4	44684392/C//T	synonymous	0	1
MMRN1	chr4	90856821/A//G	nonsynonymous	0	1
ZGRF1	chr4	113540373/T//C	synonymous	0	1
SETD7	chr4	140439129/A//G	nonsynonymous	0	1
CLDN22	chr4	184240951/C//A	nonsynonymous	0	1
FAM71B	chr5	156592669/G//A	nonsynonymous	0	1
RP1	chr8	55533727/G//T	synonymous	0	1
RP1	chr8	55542929/ACT//A	synonymous	0	1
C8orf89	chr8	74171568/T//C	synonymous	0	1
TOR2A	chr9	130494553/G//A	synonymous	0	1
ITIH5	chr10	7683943/G//A	synonymous	0	1
AGBL2	chr11	47701525/C//T	synonymous	0	1
SSTR1	chr14	38679346/G//A	nonsynonymous	0	1
UNC13C	chr15	54435818/C//G	nonsynonymous	0	1
CSK	chr15	75091012/C//G	synonymous	0	1
ZNF232	chr17	5009519/A//G	nonsynonymous	0	1
CLEC10A	chr17	6982153/A//G	synonymous	0	1
MRC2	chr17	60751524/G//T	nonsynonymous	0	1
ZNF844	chr19	12187206/G//A	nonsynonymous	0	1
NWD1	chr19	16842067/A//G	nonsynonymous	0	1
BAGE3	chr21	11049596/C//T	nonsynonymous	0	1
PI4KA	chr22	21066824/C//A	nonsynonymous	0	1
GTPBP6	chrX	228217/C//T	nonsynonymous	0	1
KCNE5	chrX	108868228/G//A	nonsynonymous	0	1
CXorf56	chrX	118675352/G//T	nonsynonymous	0	1
ABCD1	chrX	152991032/G//A	nonsynonymous	0	1
AGO1	chr1	36367669/C//T	nonsynonymous	0	1
OR2W3	chr1	248058989/C//T	nonsynonymous	0	1
KLHL30	chr2	239049843/G//A	nonsynonymous	0	1
ROBO2	chr3	77611855/T//G	synonymous	0	1
U2SURP	chr3	142735740/G//A	nonsynonymous	0	1
VEPH1	chr3	156978959/T//A	nonsynonymous	0	1
PI4K2B	chr4	25235844/A//G	nonsynonymous	0	1
MAPK10	chr4	86938432/G//A	synonymous	0	1
MMRN1	chr4	90857387/C//G	synonymous	0	1
SLC6A3	chr5	1403172/T//C	synonymous	0	1
APC	chr5	112173668/CAA//C	nonsynonymous	0	1
PPIC	chr5	122364464/C//T	synonymous	0	1
PCDHGB5	chr5	140779334/T//C	nonsynonymous	0	1
KIF4B	chr5	154393956/G//T	nonsynonymous	0	1
NIPAL4	chr5	156887257/C//T	nonsynonymous	0	1
MXD3	chr5	176738873/G//A	synonymous	0	1
ADAMTS2	chr5	178699962/C//T	nonsynonymous	0	1
MBOAT1	chr6	20124776/G//A	nonsynonymous	0	1
STK31	chr7	23775208/A//G	nonsynonymous	0	1
CA1	chr8	86240910/A//G	synonymous	0	1
FAM120AOS	chr9	96214617/G//C	synonymous	0	1
PYROXD2	chr10	100152753/A//G	synonymous	0	1
ACER3	chr11	76726116/G//A	nonsynonymous	0	1
ARHGAP20	chr11	110501477/A//G	nonsynonymous	0	1
RTL1	chr14	101349773/G//A	synonymous	0	1
EXOC3L4	chr14	103574772/G//A	nonsynonymous	0	1
TDRD9	chr14	104433138/G//A	synonymous	0	1
DHODH	chr16	72057510/C//T	nonsynonymous	0	1
ANKFY1	chr17	4088189/G//T	synonymous	0	1
EPG5	chr18	43440124/G//A	synonymous	0	1
ZNF30	chr19	35435001/C//T	synonymous	0	1
ZNF772	chr19	57985332/G//A	synonymous	0	1
NDUFAF5	chr20	13797787/C//T	synonymous	0	1

RALGAPB	chr20	37137768/C//A	synonymous	0	1
PCNT	chr21	47744105/C//T	synonymous	0	1
MYO18B	chr22	26164433/G//A	nonsynonymous	0	1
CELA3A	chr1	22333860/T//C	synonymous	0	1
FANCD2	chr3	10122775/TTATTA//T	nonsynonymous	0	1
NBEAL2	chr3	47042645/G//A	nonsynonymous	0	1
DDX60L	chr4	169292905/G//A	nonsynonymous	0	1
BHMT	chr5	78421907/A//G	nonsynonymous	0	1
SKP1	chr5	133509644/A//G	nonsynonymous	0	1
NME5	chr5	137464275/A//G	synonymous	0	1
GRIA1	chr5	153026518/C//T	nonsynonymous	0	1
PRPF4B	chr6	4021638/C//A	synonymous	0	1
SLCO5A1	chr8	70585189/T//C	nonsynonymous	0	1
PLEC	chr8	144999791/T//C	nonsynonymous	0	1
PLAA	chr9	26913896/G//A	synonymous	0	1
	chr9	124062408/A//G	synonymous	0	1
MKI67	chr10	129910533/T//C	synonymous	0	1
PHLDB1	chr11	118516165/C//T	synonymous	0	1
KIRREL3	chr11	126391239/C//T	nonsynonymous	0	1
CACNA1C	chr12	2714265/C//T	synonymous	0	1
SLITRK5	chr13	88329425/G//A	synonymous	0	1
HECTD1	chr14	31570023/A//G	synonymous	0	1
SAMD15	chr14	77845345/C//T	synonymous	0	1
RYR3	chr15	34040685/C//T	nonsynonymous	0	1
SLC5A11	chr16	24918033/A//G	synonymous	0	1
RCVRN	chr17	9804370/G//A	synonymous	0	1
EPB41L3	chr18	5410605/C//T	nonsynonymous	0	1
ANKRD24	chr19	4200118/T//C	nonsynonymous	0	1
OR7D4	chr19	9325369/C//T	nonsynonymous	0	1
ZNF101	chr19	19790270/C//T	nonsynonymous	0	1
ZNF536	chr19	31039694/G//A	synonymous	0	1
PEG3	chr19	57335875/C//T	nonsynonymous	0	1
AGRN	chr1	981871/C//T	synonymous	1	0
B3GALT6	chr1	1168066/G//A	synonymous	1	0
B3GALT6	chr1	1168074/T//C	nonsynonymous	1	0
VWA1	chr1	1374475/C//T	nonsynonymous	1	0
ATAD3C	chr1	1396281/G//A	nonsynonymous	1	0
SLC35E2B	chr1	1602978/G//A	nonsynonymous	1	0
AJAP1	chr1	4832475/C//T	synonymous	1	0
CA6	chr1	9017227/G//A	nonsynonymous	1	0
H6PD	chr1	9307049/C//T	nonsynonymous	1	0
CLSTN1	chr1	9801240/C//T	synonymous	1	0
DISP3	chr1	11579424/T//C	synonymous	1	0
MAD2L2	chr1	11735211/C//T	synonymous	1	0
DNAJC16	chr1	15870904/T//C	synonymous	1	0
PADI1	chr1	17550162/G//A	nonsynonymous	1	0
NBPF3	chr1	21799981/G//A	synonymous	1	0
CELA3B	chr1	22304903/G//A	nonsynonymous	1	0
EPHA8	chr1	22922613/G//A	nonsynonymous	1	0
AUNIP	chr1	26163988/CCTTT//C	nonsynonymous	1	0
	chr1	26872423/G//A	synonymous	1	0
GPATCH3	chr1	27224193/T//C	nonsynonymous	1	0
AHDC1	chr1	27874822/G//A	nonsynonymous	1	0
COL16A1	chr1	32122666/C//T	nonsynonymous	1	0
RBBP4	chr1	33150106/A//G	synonymous	1	0
ZSCAN20	chr1	33945009/C//T	synonymous	1	0
CSMD2	chr1	34401390/G//A	nonsynonymous	1	0
ZMYM1	chr1	35578622/T//C	synonymous	1	0
SH3D21	chr1	36785375/G//A	nonsynonymous	1	0
MACF1	chr1	39903506/C//T	nonsynonymous	1	0
KCNQ4	chr1	41289794/G//A	nonsynonymous	1	0
HIVEP3	chr1	42046399/T//A	nonsynonymous	1	0
ZNF691	chr1	43317082/G//A	synonymous	1	0
MPL	chr1	43812266/C//T	synonymous	1	0
C1orf228	chr1	45190334/G//A	synonymous	1	0
IPP	chr1	46165795/C//T	nonsynonymous	1	0
POMGNT1	chr1	46657790/T//C	nonsynonymous	1	0
RAD54L	chr1	46743519/C//T	nonsynonymous	1	0
FAAH	chr1	46872016/G//A	synonymous	1	0
DMRTA2	chr1	50887031/C//T	nonsynonymous	1	0

CC2D1B	chr1	52823496/A//G	synonymous	1	0
SLC1A7	chr1	53558467/C//T	synonymous	1	0
DIO1	chr1	54359956/G//A	nonsynonymous	1	0
L1TD1	chr1	62675694/A//G	synonymous	1	0
DOCK7	chr1	62941773/TAG//T	nonsynonymous	1	0
LRRC7	chr1	70505050/C//T	synonymous	1	0
ANKRD13C	chr1	70781186/C//T	nonsynonymous	1	0
CLCA2	chr1	86921118/G//T	nonsynonymous	1	0
BCAR3	chr1	94048000/AAGG//A	nonsynonymous	1	0
DPYD	chr1	98293695/G//A	nonsynonymous	1	0
VCAM1	chr1	101198237/CA//C	nonsynonymous	1	0
COL11A1	chr1	103444449/A//G	synonymous	1	0
AMPD2	chr1	110170884/G//A	synonymous	1	0
KCNC4	chr1	110766299/C//T	synonymous	1	0
IGSF3	chr1	117208901/C//T	synonymous	1	0
PTGFRN	chr1	117527334/G//A	nonsynonymous	1	0
PDE4DIP	chr1	144866673/G//A	nonsynonymous	1	0
GJA5	chr1	147231087/G//A	nonsynonymous	1	0
FCGR1A	chr1	149760138/G//A	nonsynonymous	1	0
CTSK	chr1	150778432/T//G	nonsynonymous	1	0
PI4KB	chr1	151266968/C//T	nonsynonymous	1	0
CELF3	chr1	151680350/C//T	nonsynonymous	1	0
FLG	chr1	152278610/A//T	nonsynonymous	1	0
FLG	chr1	152281795/C//T	nonsynonymous	1	0
S100A16	chr1	153580156/G//A	nonsynonymous	1	0
ADAR	chr1	154562843/G//T	synonymous	1	0
ASH1L	chr1	155330147/G//A	nonsynonymous	1	0
GON4L	chr1	155721649/C//T	nonsynonymous	1	0
SEMA4A	chr1	156130310/G//A	synonymous	1	0
CD1D	chr1	158152697/C//A	nonsynonymous	1	0
OR6K3	chr1	158687028/T//C	nonsynonymous	1	0
TADA1	chr1	166829551/C//T	synonymous	1	0
ADCY10	chr1	167791382/AAG//A	synonymous	1	0
F5	chr1	169511621/C//T	nonsynonymous	1	0
PRRC2C	chr1	171501770/C//T	nonsynonymous	1	0
SUCO	chr1	172558328/CTGT//C	nonsynonymous	1	0
ASTN1	chr1	176913089/G//A	nonsynonymous	1	0
LAMC2	chr1	183204870/G//A	synonymous	1	0
APOBEC4	chr1	183616825/TTTC//T	nonsynonymous	1	0
ASPM	chr1	197060089/T//A	nonsynonymous	1	0
SOX13	chr1	204095006/A//G	nonsynonymous	1	0
GOLT1A	chr1	204183081/A//G	synonymous	1	0
CNTN2	chr1	205034377/G//A	nonsynonymous	1	0
ELK4	chr1	205592918/C//T	synonymous	1	0
AVPR1B	chr1	206224329/C//T	synonymous	1	0
RASSF5	chr1	206757860/A//T	nonsynonymous	1	0
C4BPA	chr1	207288791/G//A	nonsynonymous	1	0
IRF6	chr1	209961873/G//A	synonymous	1	0
NSL1	chr1	212911820/TTTC//T	nonsynonymous	1	0
	chr1	216348525/G//A	synonymous	1	0
CAPN2	chr1	223951883/C//T	nonsynonymous	1	0
RAB4A	chr1	229433239/T//A	nonsynonymous	1	0
LYST	chr1	235922762/T//G	synonymous	1	0
ACTN2	chr1	236914816/C//T	nonsynonymous	1	0
ZP4	chr1	238050848/A//T	nonsynonymous	1	0
RGS7	chr1	241100003/T//A	nonsynonymous	1	0
EXO1	chr1	242042551/G//T	nonsynonymous	1	0
KIF26B	chr1	245530163/G//A	nonsynonymous	1	0
ZNF124	chr1	247320049/C//T	nonsynonymous	1	0
ZNF496	chr1	247463849/C//T	nonsynonymous	1	0
OR6F1	chr1	247875316/C//A	nonsynonymous	1	0
SH3BP5L	chr1	249106231/G//A	synonymous	1	0
PXDN	chr2	1647234/G//A	synonymous	1	0
	chr2	1796046/C//T	synonymous	1	0
KIDINS220	chr2	8871451/T//C	nonsynonymous	1	0
ASAP2	chr2	9498960/T//C	nonsynonymous	1	0
YWHAQ	chr2	9770391/C//T	nonsynonymous	1	0
WDR35	chr2	20160311/T//G	synonymous	1	0
PUM2	chr2	20455850/T//C	nonsynonymous	1	0
IFT172	chr2	27699557/T//C	synonymous	1	0

PLB1	chr2	28820884/C//T	synonymous	1	0
EHD3	chr2	31457713/A//G	nonsynonymous	1	0
BIRC6	chr2	32832696/C//T	nonsynonymous	1	0
RASGRP3	chr2	33780611/T//C	nonsynonymous	1	0
THADA	chr2	43520054/G//A	synonymous	1	0
STON1-GTF2A1L	chr2	48848415/T//C	nonsynonymous	1	0
NRXN1	chr2	50724736/A//C	nonsynonymous	1	0
TSPYL6	chr2	54482768/G//A	nonsynonymous	1	0
TSPYL6	chr2	54483379/C//T	synonymous	1	0
SPTBN1	chr2	54856197/GTTC//G	nonsynonymous	1	0
SPTBN1	chr2	54883115/G//A	nonsynonymous	1	0
EML6	chr2	55181179/C//T	nonsynonymous	1	0
FAM161A	chr2	62067586/ACT//A	nonsynonymous	1	0
ETAA1	chr2	67630506/A//T	nonsynonymous	1	0
CLEC4F	chr2	71044114/C//T	synonymous	1	0
VAX2	chr2	71160418/G//A	synonymous	1	0
SFXN5	chr2	73298860/C//T	synonymous	1	0
TET3	chr2	74274855/G//A	nonsynonymous	1	0
TET3	chr2	74320727/G//A	synonymous	1	0
LOXL3	chr2	74776642/C//T	synonymous	1	0
LRRTM4	chr2	77746710/A//C	nonsynonymous	1	0
POLR1A	chr2	86254541/AGCT//A	synonymous	1	0
RNF103	chr2	86831237/C//A	nonsynonymous	1	0
RMND5A	chr2	86998712/A//T	nonsynonymous	1	0
GPAT2	chr2	96688935/G//A	nonsynonymous	1	0
SLC9A2	chr2	103324702/GAAGA//G	nonsynonymous	1	0
SLC5A7	chr2	108604648/G//A	nonsynonymous	1	0
GCC2	chr2	109124124/CTATT//C	synonymous	1	0
RANBP2	chr2	109383727/T//C	synonymous	1	0
CKAP2L	chr2	113522198/T//G	synonymous	1	0
INSIG2	chr2	118854234/G//T	synonymous	1	0
INSIG2	chr2	118854301/C//T	nonsynonymous	1	0
STEAP3	chr2	120003349/C//T	nonsynonymous	1	0
CFAP221	chr2	120362373/G//A	synonymous	1	0
MYO7B	chr2	128347805/G//T	nonsynonymous	1	0
LIMS2	chr2	128397861/A//G	synonymous	1	0
WDR33	chr2	128466334/C//T	nonsynonymous	1	0
RAB6C	chr2	130738148/G//A	nonsynonymous	1	0
GPR39	chr2	133402692/TG//T	nonsynonymous	1	0
ZRANB3	chr2	136103170/T//G	nonsynonymous	1	0
LRP1B	chr2	141283486/C//T	synonymous	1	0
MBD5	chr2	149247747/ACTGTGG//A	nonsynonymous	1	0
GALNT13	chr2	154996845/T//C	synonymous	1	0
KCNJ3	chr2	155711572/G//C	nonsynonymous	1	0
ACVR1C	chr2	158406720/C//T	nonsynonymous	1	0
WDSUB1	chr2	160105008/C//T	nonsynonymous	1	0
MARCH7	chr2	160605043/C//G	synonymous	1	0
TANK	chr2	162091890/C//T	nonsynonymous	1	0
TBR1	chr2	162279931/G//A	synonymous	1	0
SCN2A	chr2	166237705/G//A	nonsynonymous	1	0
SCN1A	chr2	166894292/G//A	synonymous	1	0
SCN7A	chr2	167284444/G//A	nonsynonymous	1	0
XIRP2	chr2	168067299/G//A	nonsynonymous	1	0
STK39	chr2	168997201/G//A	synonymous	1	0
KLHL41	chr2	170377509/CAT//C	nonsynonymous	1	0
WIPF1	chr2	175436410/G//A	nonsynonymous	1	0
CHRNA1	chr2	175614840/A//G	nonsynonymous	1	0
HOXD9	chr2	176987598/C//T	synonymous	1	0
HOXD3	chr2	177036623/C//T	nonsynonymous	1	0
TTN	chr2	179404558/A//G	nonsynonymous	1	0
TTN	chr2	179425019/C//T	nonsynonymous	1	0
TTN	chr2	179426155/G//A	nonsynonymous	1	0
TTN	chr2	179427827/C//T	nonsynonymous	1	0
TTN	chr2	179481856/C//T	nonsynonymous	1	0
TTN	chr2	179495927/C//T	synonymous	1	0
TTN	chr2	179593733/G//A	synonymous	1	0
TTN	chr2	179658219/G//T	nonsynonymous	1	0
DNAJC10	chr2	183623615/A//G	synonymous	1	0
SDPR	chr2	192701125/T//C	nonsynonymous	1	0
SLC39A10	chr2	196581534/G//A	nonsynonymous	1	0

DNAH7	chr2	196729464/ATTG//A	nonsynonymous	1	0
C2orf69	chr2	200776332/T//C	synonymous	1	0
SPATS2L	chr2	201342423/C//G	nonsynonymous	1	0
NBEAL1	chr2	204075830/G//T	nonsynonymous	1	0
	chr2	206608305/G//A	synonymous	1	0
MDH1B	chr2	207621736/G//A	nonsynonymous	1	0
PLEKHM3	chr2	208795674/G//A	nonsynonymous	1	0
CRYGB	chr2	209007370/A//G	synonymous	1	0
PIKFYVE	chr2	209191033/T//A	nonsynonymous	1	0
ERBB4	chr2	212578340/C//G	nonsynonymous	1	0
XRCC5	chr2	216986893/G//A	synonymous	1	0
TNS1	chr2	218683225/G//A	nonsynonymous	1	0
NYAP2	chr2	226447618/C//T	synonymous	1	0
COL4A3	chr2	228167820/G//C	nonsynonymous	1	0
MFF	chr2	228212087/G//C	nonsynonymous	1	0
USP40	chr2	234465631/C//T	nonsynonymous	1	0
ACKR3	chr2	237489345/G//A	synonymous	1	0
ESPNL	chr2	239036341/C//A	nonsynonymous	1	0
SNED1	chr2	241988107/C//T	synonymous	1	0
HDLBP	chr2	242186201/G//A	nonsynonymous	1	0
SSUH2	chr3	8671412/C//A	nonsynonymous	1	0
SRGAP3	chr3	9146411/C//T	nonsynonymous	1	0
CIDEC	chr3	9908698/G//A	synonymous	1	0
FANCD2	chr3	10084326/A//C	synonymous	1	0
TOP2B	chr3	25677436/C//A	nonsynonymous	1	0
DCLK3	chr3	36779904/G//A	nonsynonymous	1	0
ULK4	chr3	41973348/T//A	nonsynonymous	1	0
TGM4	chr3	44932115/T//G	nonsynonymous	1	0
CCR3	chr3	46306741/C//A	nonsynonymous	1	0
ALS2CL	chr3	46725332/C//T	synonymous	1	0
SCAP	chr3	47459927/C//T	nonsynonymous	1	0
ELP6	chr3	47539884/C//T	synonymous	1	0
CDHR4	chr3	49833072/GA//G	nonsynonymous	1	0
MST1R	chr3	49924952/G//A	nonsynonymous	1	0
RBM6	chr3	50005308/A//G	synonymous	1	0
RBM6	chr3	50005421/G//A	nonsynonymous	1	0
WDR82	chr3	52295490/A//G	nonsynonymous	1	0
FEZF2	chr3	62355871/C//T	nonsynonymous	1	0
CNTN3	chr3	74350837/G//A	nonsynonymous	1	0
ROBO1	chr3	78706415/C//T	nonsynonymous	1	0
POU1F1	chr3	87313475/CTT//C	nonsynonymous	1	0
TRAT1	chr3	108568047/A//C	nonsynonymous	1	0
DRD3	chr3	113850098/C//T	synonymous	1	0
NR1I2	chr3	119536019/C//T	nonsynonymous	1	0
GPR156	chr3	119887219/T//C	nonsynonymous	1	0
PDIA5	chr3	122842941/C//T	nonsynonymous	1	0
KALRN	chr3	124385375/G//A	nonsynonymous	1	0
OSBPL11	chr3	125249357/T//G	synonymous	1	0
MCM2	chr3	127339589/C//T	nonsynonymous	1	0
RHO	chr3	129249765/C//T	synonymous	1	0
ACAD11	chr3	132294684/G//A	nonsynonymous	1	0
ZIC1	chr3	147128453/G//A	nonsynonymous	1	0
HLTF	chr3	148750058/G//A	synonymous	1	0
PFN2	chr3	149683983/C//T	synonymous	1	0
P2RY14	chr3	150931793/G//A	synonymous	1	0
IQCJ-SCHIP1	chr3	159583963/TGA//T	nonsynonymous	1	0
NLGN1	chr3	173997257/A//G	nonsynonymous	1	0
CCDC39	chr3	180332761/G//T	nonsynonymous	1	0
YEATS2	chr3	183524790/CAAG//C	nonsynonymous	1	0
DGKG	chr3	185997644/T//C	nonsynonymous	1	0
CLDN1	chr3	190026113/T//C	nonsynonymous	1	0
GMNC	chr3	190573409/G//A	nonsynonymous	1	0
	chr3	196746640/A//T	synonymous	1	0
	chr3	196910748/G//A	synonymous	1	0
FYTTD1	chr3	197476735/G//A	synonymous	1	0
IQCG	chr3	197665551/G//A	nonsynonymous	1	0
POLN	chr4	2200401/C//T	nonsynonymous	1	0
NSG1	chr4	4393312/G//T	nonsynonymous	1	0
HTRA3	chr4	8293178/G//A	nonsynonymous	1	0
CPZ	chr4	8603208/G//A	synonymous	1	0

BOD1L1	chr4	13604564/C//T	synonymous	1	0
CC2D2A	chr4	15589532/A//G	nonsynonymous	1	0
LAP3	chr4	17585107/G//A	synonymous	1	0
FAM184B	chr4	17707453/T//C	synonymous	1	0
PI4K2B	chr4	25256828/G//A	nonsynonymous	1	0
LIMCH1	chr4	41615555/C//T	nonsynonymous	1	0
PHOX2B	chr4	41749534/C//T	synonymous	1	0
TXK	chr4	48091781/G//A	nonsynonymous	1	0
LRRC66	chr4	52861404/G//A	nonsynonymous	1	0
TMPRSS11A	chr4	68777063/G//A	synonymous	1	0
CSN2	chr4	70823054/T//G	nonsynonymous	1	0
CABS1	chr4	71201008/C//T	synonymous	1	0
THAP9	chr4	83829088/C//T	nonsynonymous	1	0
WDFY3	chr4	85738542/T//G	synonymous	1	0
ABCG2	chr4	89034639/A//T	nonsynonymous	1	0
TBCK	chr4	107154789/A//T	nonsynonymous	1	0
KIAA1109	chr4	123267786/G//T	nonsynonymous	1	0
ANKRD50	chr4	125590284/C//T	nonsynonymous	1	0
SMAD1	chr4	146479027/C//T	synonymous	1	0
DCLK2	chr4	151160952/C//T	nonsynonymous	1	0
FGB	chr4	155487066/G//A	nonsynonymous	1	0
SPOCK3	chr4	167921589/G//T	nonsynonymous	1	0
ACSL1	chr4	185724511/G//A	nonsynonymous	1	0
FAM149A	chr4	187088380/G//A	nonsynonymous	1	0
TRIML2	chr4	189012873/C//T	nonsynonymous	1	0
FRG1	chr4	190883087/G//T	nonsynonymous	1	0
LRRC14B	chr5	192284/G//A	nonsynonymous	1	0
SEMA5A	chr5	9119183/G//A	nonsynonymous	1	0
ANKRD33B	chr5	10618520/G//A	nonsynonymous	1	0
CDH10	chr5	24498555/A//T	nonsynonymous	1	0
ZFR	chr5	32444742/A//G	nonsynonymous	1	0
ITGA2	chr5	52386383/C//T	nonsynonymous	1	0
MAP3K1	chr5	56178237/A//T	synonymous	1	0
GPBP1	chr5	56542967/A//G	synonymous	1	0
MARVELD2	chr5	68715229/G//T	nonsynonymous	1	0
NAIP	chr5	70308603/G//A	nonsynonymous	1	0
MCCC2	chr5	70895518/G//A	nonsynonymous	1	0
PPIP5K2	chr5	102494271/T//A	nonsynonymous	1	0
CXCL14	chr5	134914483/C//T	nonsynonymous	1	0
SPATA24	chr5	138737518/G//T	synonymous	1	0
PCDHA4	chr5	140188813/C//T	nonsynonymous	1	0
TAF7	chr5	140699293/C//T	nonsynonymous	1	0
ABLIM3	chr5	148620265/T//A	nonsynonymous	1	0
AFAP1L1	chr5	148709328/G//A	nonsynonymous	1	0
SLC36A1	chr5	150858891/T//C	nonsynonymous	1	0
FAT2	chr5	150923897/T//C	nonsynonymous	1	0
GEMIN5	chr5	154296712/G//C	nonsynonymous	1	0
KIF4B	chr5	154394866/T//C	nonsynonymous	1	0
ADAM19	chr5	156908868/A//G	synonymous	1	0
ADAM19	chr5	156915406/G//T	nonsynonymous	1	0
SLIT3	chr5	168216615/T//G	synonymous	1	0
LCP2	chr5	169675759/C//T	nonsynonymous	1	0
RANBP17	chr5	170610211/A//T	nonsynonymous	1	0
SH3PXD2B	chr5	171765654/G//A	nonsynonymous	1	0
SLC22A23	chr6	3273552/G//A	nonsynonymous	1	0
FARS2	chr6	5404837/G//A	synonymous	1	0
FARS2	chr6	5771583/C//T	nonsynonymous	1	0
NEDD9	chr6	11213645/G//T	nonsynonymous	1	0
NUP153	chr6	17632936/A//G	synonymous	1	0
CDKAL1	chr6	20548906/C//T	nonsynonymous	1	0
NRSN1	chr6	24146260/C//T	synonymous	1	0
GPLD1	chr6	24429141/A//G	synonymous	1	0
CARMIL1	chr6	25551146/T//G	synonymous	1	0
HIST1H2BA	chr6	25727357/G//A	nonsynonymous	1	0
SLC17A2	chr6	25914865/T//A	nonsynonymous	1	0
HIST1H2BH	chr6	26252186/T//A	nonsynonymous	1	0
ZSCAN26	chr6	28239934/C//G	nonsynonymous	1	0
GABBR1	chr6	29574685/G//A	nonsynonymous	1	0
GABBR1	chr6	29588948/G//A	nonsynonymous	1	0
ZFP57	chr6	29643227/C//T	synonymous	1	0

CFB	chr6	31915247/C//T	nonsynonymous	1	0
PHF1	chr6	33382066/A//T	nonsynonymous	1	0
ITPR3	chr6	33626528/ACT//A	nonsynonymous	1	0
UHRF1BP1	chr6	34823441/C//T	nonsynonymous	1	0
ANKS1A	chr6	34952864/G//A	nonsynonymous	1	0
TCP11	chr6	35086154/G//A	synonymous	1	0
ZNF76	chr6	35255628/C//T	synonymous	1	0
BRPF3	chr6	36198206/G//A	synonymous	1	0
DNAH8	chr6	38791392/T//C	nonsynonymous	1	0
UNC5CL	chr6	41001860/T//C	nonsynonymous	1	0
UBR2	chr6	42637910/C//T	nonsynonymous	1	0
CUL7	chr6	43010919/C//T	nonsynonymous	1	0
CUL7	chr6	43016165/C//T	synonymous	1	0
POLH	chr6	43581581/A//C	nonsynonymous	1	0
MUT	chr6	49425735/G//A	nonsynonymous	1	0
PKHD1	chr6	51613426/G//A	synonymous	1	0
GSTA4	chr6	52859023/G//A	nonsynonymous	1	0
TINAG	chr6	54185435/C//T	synonymous	1	0
BMP5	chr6	55639039/G//A	nonsynonymous	1	0
RIMS1	chr6	72945331/C//T	nonsynonymous	1	0
CD109	chr6	74517822/A//T	nonsynonymous	1	0
PGM3	chr6	83889614/T//C	nonsynonymous	1	0
CEP162	chr6	84904743/A//G	nonsynonymous	1	0
LOC101929057	chr6	90397923/A//G	synonymous	1	0
MDN1	chr6	90432740/A//G	synonymous	1	0
MDN1	chr6	90466033/A//C	nonsynonymous	1	0
MAP3K7	chr6	91261906/T//C	synonymous	1	0
MCHR2	chr6	100403974/T//C	nonsynonymous	1	0
SCML4	chr6	108067978/G//A	synonymous	1	0
ARMC2	chr6	109274324/G//A	nonsynonymous	1	0
REV3L	chr6	111654417/C//T	nonsynonymous	1	0
HS3ST5	chr6	114378576/G//A	nonsynonymous	1	0
RSPH4A	chr6	116938163/A//C	nonsynonymous	1	0
RNF217	chr6	125379190/C//T	nonsynonymous	1	0
NCOA7	chr6	126236555/G//T	nonsynonymous	1	0
MTFR2	chr6	136560720/C//T	synonymous	1	0
PCMT1	chr6	150123393/G//A	nonsynonymous	1	0
SYNE1	chr6	152809633/TTC//T	nonsynonymous	1	0
OPRM1	chr6	154412543/G//A	nonsynonymous	1	0
TFB1M	chr6	155606337/T//C	synonymous	1	0
ARID1B	chr6	157511253/C//T	synonymous	1	0
SOD2	chr6	160105957/C//G	nonsynonymous	1	0
IGF2R	chr6	160445699/G//A	synonymous	1	0
RPS6KA2	chr6	166844003/G//A	nonsynonymous	1	0
THBS2	chr6	169634937/G//A	nonsynonymous	1	0
DNAAF5	chr7	810139/C//T	synonymous	1	0
MAD1L1	chr7	1997278/G//A	nonsynonymous	1	0
	chr7	2552899/A//G	synonymous	1	0
SDK1	chr7	4014070/C//T	synonymous	1	0
TNRC18	chr7	5352793/T//C	nonsynonymous	1	0
CCZ1B	chr7	6862938/G//A	synonymous	1	0
THSD7A	chr7	11521477/C//A	nonsynonymous	1	0
BZW2	chr7	16705063/A//T	synonymous	1	0
HDAC9	chr7	18767220/G//A	synonymous	1	0
HOXA13	chr7	27237694/A//G	synonymous	1	0
AMPH	chr7	38424397/CCTT//C	synonymous	1	0
HECW1	chr7	43484439/G//A	synonymous	1	0
HECW1	chr7	43484745/C//T	synonymous	1	0
NACAD	chr7	45120841/G//A	nonsynonymous	1	0
ABCA13	chr7	48315537/A//T	nonsynonymous	1	0
ABCA13	chr7	48315623/A//C	nonsynonymous	1	0
ZPBP	chr7	50097705/C//A	nonsynonymous	1	0
C7orf72	chr7	50198670/C//T	nonsynonymous	1	0
GRB10	chr7	50671770/G//T	nonsynonymous	1	0
PHKG1	chr7	56155352/C//T	synonymous	1	0
ZNF680	chr7	63982619/A//T	nonsynonymous	1	0
ZNF273	chr7	64388485/A//T	nonsynonymous	1	0
AUTS2	chr7	70255623/C//T	nonsynonymous	1	0
SSC4D	chr7	76019569/C//T	nonsynonymous	1	0
UPK3B	chr7	76144547/G//T	synonymous	1	0

RSBN1L	chr7	77408061/A//G	nonsynonymous	1	0
KIAA1324L	chr7	86521059/A//T	nonsynonymous	1	0
TMEM243	chr7	86825978/G//T	nonsynonymous	1	0
ADAM22	chr7	87774511/C//T	synonymous	1	0
AKAP9	chr7	91632259/C//G	nonsynonymous	1	0
AKAP9	chr7	91671391/C//CAG	nonsynonymous	1	0
AKAP9	chr7	91671391/CAG//C	nonsynonymous	1	0
DLX6	chr7	96639226/C//T	nonsynonymous	1	0
BHLHA15	chr7	97841714/G//A	synonymous	1	0
TECPR1	chr7	97866237/GAA//G	synonymous	1	0
BAIAP2L1	chr7	97941417/TTC//T	nonsynonymous	1	0
PTCD1	chr7	99021415/G//T	nonsynonymous	1	0
ZNF789	chr7	99084964/G//A	synonymous	1	0
ZNF394	chr7	99096363/CCTT//C	nonsynonymous	1	0
GATS	chr7	99821630/C//A	nonsynonymous	1	0
MUC12	chr7	100635818/T//C	synonymous	1	0
FBXL13	chr7	102572379/G//T	nonsynonymous	1	0
FBXL13	chr7	102667935/A//T	nonsynonymous	1	0
DNAJC2	chr7	102960240/C//A	nonsynonymous	1	0
LAMB1	chr7	107569989/T//G	nonsynonymous	1	0
LAMB1	chr7	107580772/C//T	synonymous	1	0
FOXP2	chr7	114066640/G//A	nonsynonymous	1	0
MET	chr7	116340103/T//G	nonsynonymous	1	0
MET	chr7	116380016/C//T	nonsynonymous	1	0
CTTNBP2	chr7	117431448/T//G	nonsynonymous	1	0
KCND2	chr7	119914673/C//T	synonymous	1	0
AASS	chr7	121716586/G//A	nonsynonymous	1	0
RNF148	chr7	122342863/A//G	synonymous	1	0
GRM8	chr7	126086295/C//A	nonsynonymous	1	0
UBE2H	chr7	129519401/A//C	synonymous	1	0
KLHDC10	chr7	129770502/G//A	synonymous	1	0
CPA4	chr7	129938592/A//C	nonsynonymous	1	0
ATP6V0A4	chr7	138437551/C//T	nonsynonymous	1	0
DENND2A	chr7	140221739/G//A	nonsynonymous	1	0
TAS2R38	chr7	141672670/G//A	nonsynonymous	1	0
EPHB6	chr7	142567571/G//A	nonsynonymous	1	0
EPHA1	chr7	143098621/G//A	synonymous	1	0
ZNF467	chr7	149462454/G//A	synonymous	1	0
SSPO	chr7	149477491/C//T	nonsynonymous	1	0
GIMAP1	chr7	150417581/C//T	synonymous	1	0
ATG9B	chr7	150721080/G//T	nonsynonymous	1	0
CDK5	chr7	150751560/C//T	synonymous	1	0
	chr7	150759818/T//C	synonymous	1	0
PAXIP1-AS2	chr7	154738364/C//T	synonymous	1	0
PAXIP1	chr7	154775046/A//C	synonymous	1	0
CSMD1	chr8	3265502/C//T	nonsynonymous	1	0
SPAG11B	chr8	7308410/G//A	synonymous	1	0
PPP1R3B	chr8	8998461/C//T	nonsynonymous	1	0
XKR6	chr8	10755924/G//A	synonymous	1	0
SGCZ	chr8	13948034/C//A	nonsynonymous	1	0
MTMR7	chr8	17218640/T//C	nonsynonymous	1	0
CCAR2	chr8	22471637/G//A	nonsynonymous	1	0
CCAR2	chr8	22473325/C//A	nonsynonymous	1	0
LOXL2	chr8	23167207/G//A	synonymous	1	0
NKX3-1	chr8	23538913/G//A	nonsynonymous	1	0
ADAM28	chr8	24151625/CGAG//C	synonymous	1	0
PTK2B	chr8	27289827/G//A	synonymous	1	0
INTS9	chr8	28717005/C//T	nonsynonymous	1	0
GTF2E2	chr8	30436510/T//C	synonymous	1	0
TTI2	chr8	33367316/G//T	nonsynonymous	1	0
IDO2	chr8	39847357/G//A	nonsynonymous	1	0
POLB	chr8	42229091/A//C	synonymous	1	0
PRKDC	chr8	48839861/T//C	nonsynonymous	1	0
PXDNL	chr8	52336239/G//A	nonsynonymous	1	0
ST18	chr8	53079447/G//A	nonsynonymous	1	0
ST18	chr8	53084645/G//A	nonsynonymous	1	0
LYPLA1	chr8	54965240/A//G	nonsynonymous	1	0
RRS1	chr8	67342012/G//A	nonsynonymous	1	0
ZFHX4	chr8	77763378/A//G	synonymous	1	0
ZFHX4	chr8	77763388/A//T	nonsynonymous	1	0

CA3	chr8	86358426/G//A	nonsynonymous	1	0
UBR5	chr8	103274241/G//T	nonsynonymous	1	0
PKHD1L1	chr8	110456058/C//T	nonsynonymous	1	0
COLEC10	chr8	120101977/C//T	synonymous	1	0
COL14A1	chr8	121256213/C//T	synonymous	1	0
ATAD2	chr8	124382161/A//T	nonsynonymous	1	0
NDRG1	chr8	134270645/T//C	synonymous	1	0
TRAPPC9	chr8	140743452/G//A	nonsynonymous	1	0
AGO2	chr8	141569591/G//A	synonymous	1	0
PTK2	chr8	141900634/G//A	synonymous	1	0
ADGRB1	chr8	143545613/A//ACTG	nonsynonymous	1	0
ADGRB1	chr8	143562726/G//T	synonymous	1	0
ZC3H3	chr8	144618480/G//A	nonsynonymous	1	0
EPPK1	chr8	144942425/G//A	nonsynonymous	1	0
PLEC	chr8	145007393/G//A	nonsynonymous	1	0
GPT	chr8	145732422/T//C	synonymous	1	0
KCNV2	chr9	2729544/C//T	synonymous	1	0
GLIS3	chr9	3932477/GAGAA//G	synonymous	1	0
UBAP1	chr9	34241382/T//C	nonsynonymous	1	0
ARID3C	chr9	34621507/G//A	nonsynonymous	1	0
RUSC2	chr9	35548488/C//T	nonsynonymous	1	0
FAM189A2	chr9	72006723/C//A	synonymous	1	0
VPS13A	chr9	79980443/A//G	nonsynonymous	1	0
BICD2	chr9	95482664/G//A	nonsynonymous	1	0
FBP1	chr9	97367752/CTCT//C	nonsynonymous	1	0
LOC158434	chr9	98876971/G//T	synonymous	1	0
ALG2	chr9	101980646/ACT//A	nonsynonymous	1	0
ABCA1	chr9	107576738/C//T	nonsynonymous	1	0
SVEP1	chr9	113173602/C//T	nonsynonymous	1	0
OR2K2	chr9	114090169/G//A	nonsynonymous	1	0
AKNA	chr9	117124045/G//A	nonsynonymous	1	0
WHRN	chr9	117266881/C//T	synonymous	1	0
PSMD5	chr9	123589114/CCTT//C	nonsynonymous	1	0
TRAF1	chr9	123675814/C//T	nonsynonymous	1	0
RAB14	chr9	123943776/A//G	synonymous	1	0
RC3H2	chr9	125620999/C//T	synonymous	1	0
CIZ1	chr9	130929386/T//C	nonsynonymous	1	0
SLC27A4	chr9	131105522/C//T	synonymous	1	0
ABL1	chr9	133748333/C//T	nonsynonymous	1	0
DBH	chr9	136507554/C//A	nonsynonymous	1	0
COL5A1	chr9	137646164/G//A	nonsynonymous	1	0
NOTCH1	chr9	139390545/C//T	nonsynonymous	1	0
NOTCH1	chr9	139401345/C//T	nonsynonymous	1	0
STPG3	chr9	140147634/C//T	synonymous	1	0
NELFB	chr9	140157598/C//T	nonsynonymous	1	0
IDI2	chr10	1068645/C//T	synonymous	1	0
CALML3	chr10	5567348/C//T	synonymous	1	0
KIN	chr10	7804456/C//T	nonsynonymous	1	0
GATA3	chr10	8100385/C//T	nonsynonymous	1	0
MCM10	chr10	13214463/G//A	nonsynonymous	1	0
RSU1	chr10	16796963/G//A	nonsynonymous	1	0
PLXDC2	chr10	20106038/C//T	synonymous	1	0
SPAG6	chr10	22634526/G//A	synonymous	1	0
THNSL1	chr10	25313813/G//A	nonsynonymous	1	0
SVIL	chr10	29759374/C//T	nonsynonymous	1	0
ZNF33A	chr10	38345239/C//T	synonymous	1	0
	chr10	38404289/C//A	synonymous	1	0
TMEM72	chr10	45429081/C//T	synonymous	1	0
FRMPD2	chr10	49395262/CAG//C	nonsynonymous	1	0
VSTM4	chr10	50285340/G//A	synonymous	1	0
ERCC6	chr10	50669425/C//G	nonsynonymous	1	0
DKK1	chr10	54074727/C//T	synonymous	1	0
ARID5B	chr10	63700111/C//A	nonsynonymous	1	0
JMJD1C	chr10	64936171/C//T	nonsynonymous	1	0
HK1	chr10	71146123/C//T	synonymous	1	0
TACR2	chr10	71164812/G//A	nonsynonymous	1	0
AIFM2	chr10	71877583/C//T	nonsynonymous	1	0
EIF4EBP2	chr10	72179787/T//A	nonsynonymous	1	0
DNAJB12	chr10	74097988/T//C	nonsynonymous	1	0
MRPS16	chr10	75012240/T//C	nonsynonymous	1	0

ZSWIM8	chr10	75552600/G//T	nonsynonymous	1	0
VCL	chr10	75857078/T//A	synonymous	1	0
SH2D4B	chr10	82330038/C//T	nonsynonymous	1	0
CCSER2	chr10	86130961/C//T	synonymous	1	0
MMRN2	chr10	88696451/G//A	synonymous	1	0
EXOC6	chr10	94816756/C//T	nonsynonymous	1	0
MYOF	chr10	95191232/C//T	nonsynonymous	1	0
SORBS1	chr10	97158877/C//A	nonsynonymous	1	0
HOGA1	chr10	99371343/C//T	nonsynonymous	1	0
DNMBP	chr10	101646266/G//A	nonsynonymous	1	0
POLL	chr10	103339476/G//A	nonsynonymous	1	0
ELOVL3	chr10	103988225/C//T	synonymous	1	0
GBF1	chr10	104128503/G//A	nonsynonymous	1	0
NFKB2	chr10	104160959/C//T	synonymous	1	0
INA	chr10	105037661/C//T	synonymous	1	0
SH3PXD2A	chr10	105362551/G//A	synonymous	1	0
SH3PXD2A	chr10	105428364/A//G	nonsynonymous	1	0
ZDHHC6	chr10	114201968/GTACA//G	nonsynonymous	1	0
DCLRE1A	chr10	115605438/A//G	synonymous	1	0
TRUB1	chr10	116698259/G//A	nonsynonymous	1	0
ATRNL1	chr10	117059645/C//T	synonymous	1	0
CPXM2	chr10	125528096/C//A	synonymous	1	0
METTL10	chr10	126454007/C//T	synonymous	1	0
FAM196A	chr10	128973879/G//T	nonsynonymous	1	0
KNDC1	chr10	135012703/G//A	synonymous	1	0
TUBGCP2	chr10	135096700/C//T	nonsynonymous	1	0
CYP2E1	chr10	135352468/A//C	nonsynonymous	1	0
LMNTD2	chr11	555766/C//T	synonymous	1	0
MUC6	chr11	1016181/G//A	nonsynonymous	1	0
MUC6	chr11	1027416/C//T	nonsynonymous	1	0
MUC5B	chr11	1260191/G//A	nonsynonymous	1	0
MRPL23	chr11	1977505/C//T	nonsynonymous	1	0
TRPM5	chr11	2432869/C//T	nonsynonymous	1	0
OSBPL5	chr11	3111907/C//T	nonsynonymous	1	0
OSBPL5	chr11	3114838/G//A	nonsynonymous	1	0
TRIM68	chr11	4621807/T//A	nonsynonymous	1	0
TRIM68	chr11	4621826/C//A	nonsynonymous	1	0
FAM160A2	chr11	6244992/G//A	nonsynonymous	1	0
TIMM10B	chr11	6502733/G//A	synonymous	1	0
DNHD1	chr11	6579307/C//T	nonsynonymous	1	0
DCHS1	chr11	6651093/G//A	synonymous	1	0
DENND5A	chr11	9200512/T//A	nonsynonymous	1	0
KCNA4	chr11	30033223/C//T	nonsynonymous	1	0
RAG1	chr11	36595034/G//A	synonymous	1	0
ACCSL	chr11	44077820/T//C	nonsynonymous	1	0
ACCSL	chr11	44081396/C//T	nonsynonymous	1	0
EXT2	chr11	44265712/A//G	nonsynonymous	1	0
LARGE2	chr11	45949735/G//A	nonsynonymous	1	0
CREB3L1	chr11	46341846/C//T	synonymous	1	0
AMBRA1	chr11	46564956/G//A	synonymous	1	0
SPI1	chr11	47399980/C//T	synonymous	1	0
C1QTNF4	chr11	47611937/G//A	synonymous	1	0
SSRP1	chr11	57100236/G//A	nonsynonymous	1	0
RTN4RL2	chr11	57244377/A//G	nonsynonymous	1	0
UBE2L6	chr11	57319894/C//T	synonymous	1	0
OR9Q2	chr11	57958188/G//A	nonsynonymous	1	0
ZP1	chr11	60642695/G//A	nonsynonymous	1	0
TMEM109	chr11	60688363/G//A	synonymous	1	0
TKFC	chr11	61106630/G//A	nonsynonymous	1	0
FEN1	chr11	61563190/C//T	synonymous	1	0
RTN3	chr11	63487703/G//T	nonsynonymous	1	0
TRPT1	chr11	63992065/G//A	nonsynonymous	1	0
NRXN2	chr11	64398008/G//A	nonsynonymous	1	0
ATG2A	chr11	64665390/C//T	nonsynonymous	1	0
CAPN1	chr11	64955904/G//A	nonsynonymous	1	0
LTBP3	chr11	65306813/C//T	nonsynonymous	1	0
PCNX3	chr11	65402067/T//C	nonsynonymous	1	0
EFEMP2	chr11	65635430/C//T	nonsynonymous	1	0
CATSPER1	chr11	65792928/G//A	nonsynonymous	1	0
CTSF	chr11	66331417/G//A	nonsynonymous	1	0

SPTBN2	chr11	66478070/G//A	synonymous	1	0
RAD9A	chr11	67161081/G//A	nonsynonymous	1	0
CARNS1	chr11	67191750/G//A	nonsynonymous	1	0
RPS6KB2	chr11	67202501/C//T	nonsynonymous	1	0
RPS6KB2	chr11	67202561/C//T	nonsynonymous	1	0
CORO1B	chr11	67206223/C//T	synonymous	1	0
RNF121	chr11	71701702/A//T	nonsynonymous	1	0
INPPL1	chr11	71946411/C//T	nonsynonymous	1	0
PGM2L1	chr11	74058250/A//G	synonymous	1	0
RNF169	chr11	74547252/A//G	nonsynonymous	1	0
SLCO2B1	chr11	74880777/G//A	nonsynonymous	1	0
MYO7A	chr11	76910723/C//T	nonsynonymous	1	0
	chr11	85689112/C//T	synonymous	1	0
ME3	chr11	86158135/G//A	nonsynonymous	1	0
NOX4	chr11	89106601/T//C	synonymous	1	0
FAT3	chr11	92590382/G//A	nonsynonymous	1	0
MRE11	chr11	94209558/C//T	nonsynonymous	1	0
RDX	chr11	110143339/G//A	synonymous	1	0
C11orf52	chr11	111796699/G//T	nonsynonymous	1	0
TTC12	chr11	113194114/G//A	nonsynonymous	1	0
ANKK1	chr11	113270139/G//A	nonsynonymous	1	0
CCDC84	chr11	118869767/G//A	nonsynonymous	1	0
OR8D1	chr11	124179867/T//A	nonsynonymous	1	0
TBRG1	chr11	124496402/C//T	nonsynonymous	1	0
HYLS1	chr11	125769890/G//C	nonsynonymous	1	0
ARHGAP32	chr11	128842779/T//C	nonsynonymous	1	0
BARX2	chr11	129306798/G//A	nonsynonymous	1	0
CACNA1C	chr12	2566843/G//A	nonsynonymous	1	0
ANO2	chr12	5685129/T//A	nonsynonymous	1	0
CD163	chr12	7633810/C//T	nonsynonymous	1	0
GDF3	chr12	7842887/A//C	nonsynonymous	1	0
A2M	chr12	9220331/A//G	synonymous	1	0
DUSP16	chr12	12630318/G//A	nonsynonymous	1	0
GRIN2B	chr12	13716975/T//C	nonsynonymous	1	0
RERG	chr12	15262301/A//T	nonsynonymous	1	0
SLCO1B1	chr12	21392016/A//G	nonsynonymous	1	0
ITPR2	chr12	26492303/G//T	synonymous	1	0
ITPR2	chr12	26540523/A//G	synonymous	1	0
SLC38A1	chr12	46591833/G//A	nonsynonymous	1	0
TMEM106C	chr12	48358117/A//C	nonsynonymous	1	0
WNT1	chr12	49374262/G//A	synonymous	1	0
KMT2D	chr12	49420034/G//A	nonsynonymous	1	0
TUBA1B	chr12	49521828/T//G	nonsynonymous	1	0
METTL7A	chr12	51319005/C//A	nonsynonymous	1	0
SCN8A	chr12	52096685/A//T	nonsynonymous	1	0
ACVR1B	chr12	52374782/G//A	nonsynonymous	1	0
KRT6C	chr12	52863037/C//T	nonsynonymous	1	0
KRT6C	chr12	52865898/T//A	nonsynonymous	1	0
TNS2	chr12	53450839/C//T	synonymous	1	0
C12orf10	chr12	53699803/C//T	nonsynonymous	1	0
MAP3K12	chr12	53876390/G//T	nonsynonymous	1	0
OR6C3	chr12	55726169/A//G	nonsynonymous	1	0
ZC3H10	chr12	56514380/G//A	nonsynonymous	1	0
SMARCC2	chr12	56559297/C//T	nonsynonymous	1	0
TIMELESS	chr12	56822668/G//A	nonsynonymous	1	0
GPR182	chr12	57389445/G//A	nonsynonymous	1	0
LRP1	chr12	57589752/C//T	synonymous	1	0
LRP1	chr12	57602927/C//T	synonymous	1	0
CTDSP2	chr12	58223341/G//A	nonsynonymous	1	0
E2F7	chr12	77423610/CCTTGGGCATGA//C	nonsynonymous	1	0
NAV3	chr12	78512077/G//A	nonsynonymous	1	0
OTOGL	chr12	80647292/A//G	synonymous	1	0
ACSS3	chr12	81472059/C//T	synonymous	1	0
TMTC3	chr12	88588912/A//G	nonsynonymous	1	0
NR2C1	chr12	95452105/C//T	synonymous	1	0
CDK17	chr12	96679828/C//T	nonsynonymous	1	0
GAS2L3	chr12	101005850/G//A	nonsynonymous	1	0
STAB2	chr12	104025461/G//T	nonsynonymous	1	0
MVK	chr12	110019275/C//T	synonymous	1	0
FAM222A	chr12	110206569/G//A	nonsynonymous	1	0

RASAL1	chr12	113565611/G//A	synonymous	1	0
RASAL1	chr12	113565892/C//T	nonsynonymous	1	0
KDM2B	chr12	121879044/C//A	synonymous	1	0
TMEM120B	chr12	122150739/G//A	synonymous	1	0
LRRC43	chr12	122669280/CCTT//C	nonsynonymous	1	0
LRRC43	chr12	122674895/G//A	nonsynonymous	1	0
PITPNM2	chr12	123480003/G//A	nonsynonymous	1	0
NCOR2	chr12	124829476/T//C	nonsynonymous	1	0
SACS	chr13	23915737/A//C	nonsynonymous	1	0
DCLK1	chr13	36367516/A//T	nonsynonymous	1	0
SMAD9	chr13	37453735/T//C	nonsynonymous	1	0
FAM216B	chr13	43362907/G//A	nonsynonymous	1	0
SIAH3	chr13	46357845/G//A	synonymous	1	0
TBC1D4	chr13	75936553/C//T	nonsynonymous	1	0
IRS2	chr13	110435247/T//C	nonsynonymous	1	0
MCF2L	chr13	113750949/C//T	nonsynonymous	1	0
TEP1	chr14	20864043/G//T	synonymous	1	0
CHD8	chr14	21859717/T//A	nonsynonymous	1	0
SLC7A8	chr14	23598893/C//T	nonsynonymous	1	0
NPAS3	chr14	34269589/G//A	synonymous	1	0
SEC23A	chr14	39545272/C//T	nonsynonymous	1	0
DHRS7	chr14	60619659/G//A	nonsynonymous	1	0
MAX	chr14	65569181/A//G	synonymous	1	0
DCAF5	chr14	69522003/C//T	nonsynonymous	1	0
EXD2	chr14	69704341/A//G	nonsynonymous	1	0
SIPA1L1	chr14	72138170/C//T	nonsynonymous	1	0
RPS6KL1	chr14	75373831/CAG//C	synonymous	1	0
IRF2BPL	chr14	77492076/G//A	nonsynonymous	1	0
UNC79	chr14	94088440/C//G	nonsynonymous	1	0
BDKRB1	chr14	96730711/C//T	nonsynonymous	1	0
BCL11B	chr14	99641745/C//T	synonymous	1	0
BEGAIN	chr14	101005071/G//A	synonymous	1	0
HSP90AA1	chr14	102549948/A//G	nonsynonymous	1	0
MAGEL2	chr15	23890249/G//A	nonsynonymous	1	0
LOC283710	chr15	31515380/G//T	synonymous	1	0
ACTC1	chr15	35085681/G//A	synonymous	1	0
PLA2G4E	chr15	42289349/C//A	nonsynonymous	1	0
ZNF106	chr15	42717214/G//A	nonsynonymous	1	0
DUOX1	chr15	45440497/C//T	synonymous	1	0
COPS2	chr15	49429384/A//T	nonsynonymous	1	0
UNC13C	chr15	54916051/A//G	synonymous	1	0
TLN2	chr15	62990954/C//G	nonsynonymous	1	0
TLE3	chr15	70349854/C//T	nonsynonymous	1	0
PARP6	chr15	72552975/T//C	synonymous	1	0
EDC3	chr15	74963814/G//A	nonsynonymous	1	0
ADAMTS7	chr15	79083560/A//C	synonymous	1	0
CTSH	chr15	79215337/G//A	synonymous	1	0
RASGRF1	chr15	79265671/G//A	synonymous	1	0
TMED3	chr15	79606219/G//A	nonsynonymous	1	0
WFIKKN1	chr16	683118/G//A	nonsynonymous	1	0
FBXL16	chr16	744667/C//G	nonsynonymous	1	0
CCDC78	chr16	774456/G//A	synonymous	1	0
CACNA1H	chr16	1254199/C//T	nonsynonymous	1	0
CACNA1H	chr16	1270066/C//T	nonsynonymous	1	0
CLCN7	chr16	1510911/G//A	synonymous	1	0
ZNF598	chr16	2050440/G//A	nonsynonymous	1	0
SLC9A3R2	chr16	2088026/C//T	synonymous	1	0
TSC2	chr16	2138594/T//G	nonsynonymous	1	0
SRRM2	chr16	2820429/C//T	nonsynonymous	1	0
CREBBP	chr16	3779009/C//A	nonsynonymous	1	0
NUDT16L1	chr16	4743811/C//T	synonymous	1	0
ABAT	chr16	8862092/G//A	nonsynonymous	1	0
TEKT5	chr16	10788719/A//G	synonymous	1	0
CIITA	chr16	11004047/C//T	nonsynonymous	1	0
CPPED1	chr16	12798692/G//A	synonymous	1	0
NDE1	chr16	15818080/C//T	nonsynonymous	1	0
XYLT1	chr16	17202750/G//A	synonymous	1	0
ARL6IP1	chr16	18810016/CACTT//C	synonymous	1	0
DNAH3	chr16	21098318/G//A	nonsynonymous	1	0
SCNN1B	chr16	23360039/G//A	nonsynonymous	1	0

PALB2	chr16	23637666/G//A	nonsynonymous	1	0
ERN2	chr16	23713589/G//T	nonsynonymous	1	0
ZKSCAN2	chr16	25258678/C//T	nonsynonymous	1	0
IL21R	chr16	27441411/G//A	nonsynonymous	1	0
ATP2A1	chr16	28912037/G//A	nonsynonymous	1	0
KCTD13	chr16	29922307/GCTT//G	nonsynonymous	1	0
TAOK2	chr16	29993080/G//A	synonymous	1	0
TBX6	chr16	30097630/C//T	synonymous	1	0
CORO1A	chr16	30198700/CAGG//C	nonsynonymous	1	0
ITGAL	chr16	30533122/A//G	synonymous	1	0
ZNF646	chr16	31091876/A//C	nonsynonymous	1	0
N4BP1	chr16	48595366/TTC//T	nonsynonymous	1	0
PAPD5	chr16	50263126/C//A	nonsynonymous	1	0
NKD1	chr16	50667212/A//G	synonymous	1	0
SNX20	chr16	50707502/G//A	nonsynonymous	1	0
IRX5	chr16	54966711/G//T	nonsynonymous	1	0
SLC12A3	chr16	56914061/T//G	nonsynonymous	1	0
ADGRG3	chr16	57702231/GCTC//G	synonymous	1	0
KIFC3	chr16	57803598/C//T	nonsynonymous	1	0
	chr16	58045005/G//T	synonymous	1	0
MMP15	chr16	58075586/C//T	nonsynonymous	1	0
C16orf70	chr16	67144124/C//T	nonsynonymous	1	0
ESRP2	chr16	68265914/C//T	nonsynonymous	1	0
PLA2G15	chr16	68289862/C//T	synonymous	1	0
MTSS1L	chr16	70698083/C//T	nonsynonymous	1	0
CMTR2	chr16	71318208/G//C	nonsynonymous	1	0
TAT	chr16	71602163/G//A	nonsynonymous	1	0
ZFHX3	chr16	72984509/G//A	synonymous	1	0
LDHD	chr16	75148559/CAG//C	nonsynonymous	1	0
CHST5	chr16	75563212/G//A	synonymous	1	0
CNTNAP4	chr16	76461484/G//A	nonsynonymous	1	0
ADAD2	chr16	84229054/T//A	nonsynonymous	1	0
KIAA0513	chr16	85100714/G//A	nonsynonymous	1	0
COX4I1	chr16	85838590/C//T	nonsynonymous	1	0
MVD	chr16	88721602/G//A	synonymous	1	0
MVD	chr16	88722528/G//A	synonymous	1	0
CBFA2T3	chr16	88951498/C//T	nonsynonymous	1	0
SPG7	chr16	89614493/C//T	synonymous	1	0
DPEP1	chr16	89702753/C//T	nonsynonymous	1	0
SPIRE2	chr16	89929937/C//T	synonymous	1	0
CTNS	chr17	3560069/G//A	nonsynonymous	1	0
SPNS3	chr17	4352565/C//T	nonsynonymous	1	0
ARRB2	chr17	4619476/G//T	nonsynonymous	1	0
ENO3	chr17	4859365/G//A	nonsynonymous	1	0
NUP88	chr17	5290776/G//A	nonsynonymous	1	0
BCL6B	chr17	6930348/G//A	nonsynonymous	1	0
ZBTB4	chr17	7366280/C//T	nonsynonymous	1	0
POLR2A	chr17	7414827/G//A	nonsynonymous	1	0
TP53	chr17	7577106/G//T	nonsynonymous	1	0
KDM6B	chr17	7752560/G//A	nonsynonymous	1	0
TMEM88	chr17	7758850/G//A	nonsynonymous	1	0
CNTROB	chr17	7852767/G//A	nonsynonymous	1	0
CCDC42	chr17	8644901/C//T	nonsynonymous	1	0
FAM83G	chr17	18881426/C//T	nonsynonymous	1	0
SPECC1	chr17	20149379/G//A	nonsynonymous	1	0
FAM222B	chr17	27086837/G//A	nonsynonymous	1	0
LIG3	chr17	33325268/C//T	nonsynonymous	1	0
SRCIN1	chr17	36714554/G//A	nonsynonymous	1	0
MLLT6	chr17	36873780/C//A	nonsynonymous	1	0
RPL23	chr17	37006725/T//G	nonsynonymous	1	0
ERBB2	chr17	37880988/G//A	nonsynonymous	1	0
MED24	chr17	38183216/A//G	synonymous	1	0
KRT26	chr17	38928009/G//A	synonymous	1	0
KRT28	chr17	38954552/C//T	nonsynonymous	1	0
TMEM99	chr17	38990756/GGA//G	synonymous	1	0
KRT12	chr17	39019784/G//A	nonsynonymous	1	0
KRTAP16-1	chr17	39465179/G//T	nonsynonymous	1	0
KRT38	chr17	39593735/G//A	nonsynonymous	1	0
KRT14	chr17	39742999/G//A	nonsynonymous	1	0
KRT16	chr17	39766102/C//T	synonymous	1	0

KRT16	chr17	39768832/C//T	nonsynonymous	1	0
ETV4	chr17	41606588/C//T	nonsynonymous	1	0
FAM171A2	chr17	42436994/G//A	nonsynonymous	1	0
EFTUD2	chr17	42962639/G//A	nonsynonymous	1	0
NMT1	chr17	43180413/C//T	nonsynonymous	1	0
KPNB1	chr17	45747218/G//A	nonsynonymous	1	0
SKAP1	chr17	46257560/TTTC//T	nonsynonymous	1	0
HOXB7	chr17	46688163/G//A	nonsynonymous	1	0
TTLL6	chr17	46847200/A//G	nonsynonymous	1	0
CALCOCO2	chr17	46933448/T//C	synonymous	1	0
ZNF652	chr17	47375724/C//T	synonymous	1	0
NGFR	chr17	47590273/G//A	nonsynonymous	1	0
FAM117A	chr17	47810082/G//A	nonsynonymous	1	0
ACSF2	chr17	48540901/T//A	nonsynonymous	1	0
RSAD1	chr17	48559597/C//T	nonsynonymous	1	0
SPATA20	chr17	48628420/C//A	nonsynonymous	1	0
ABCC3	chr17	48734136/G//A	nonsynonymous	1	0
ANKRD40	chr17	48777241/ATCT//A	nonsynonymous	1	0
STXBP4	chr17	53158490/G//C	nonsynonymous	1	0
METTL2A	chr17	60501252/G//T	synonymous	1	0
SMURF2	chr17	62543856/G//T	nonsynonymous	1	0
CACNG5	chr17	64875117/T//C	nonsynonymous	1	0
ABCA9	chr17	66980276/C//T	nonsynonymous	1	0
ABCA10	chr17	67187331/G//A	nonsynonymous	1	0
ABCA5	chr17	67252413/G//A	nonsynonymous	1	0
DNAI2	chr17	72308360/G//A	synonymous	1	0
CDR2L	chr17	72999773/C//T	synonymous	1	0
MRPL58	chr17	73015818/C//T	nonsynonymous	1	0
RHBDF2	chr17	74471178/C//T	synonymous	1	0
TMC6	chr17	76115097/G//A	synonymous	1	0
TMC6	chr17	76116846/C//T	nonsynonymous	1	0
CBX8	chr17	77768438/CTT//C	nonsynonymous	1	0
RNF213	chr17	78318781/C//T	nonsynonymous	1	0
CEP131	chr17	79173559/C//T	nonsynonymous	1	0
TEPSIN	chr17	79207302/G//A	synonymous	1	0
SLC38A10	chr17	79249934/G//A	synonymous	1	0
CCDC57	chr17	80159757/T//A	nonsynonymous	1	0
CETN1	chr18	580357/G//A	synonymous	1	0
CETN1	chr18	580369/G//A	synonymous	1	0
TUBB6	chr18	12308790/A//G	synonymous	1	0
MC2R	chr18	13884764/C//T	nonsynonymous	1	0
DSG1	chr18	28935186/A//G	synonymous	1	0
GAREM1	chr18	29848495/G//A	nonsynonymous	1	0
CCDC178	chr18	30936289/A//G	nonsynonymous	1	0
INO80C	chr18	33048616/CG//C	nonsynonymous	1	0
ELP2	chr18	33738797/G//C	nonsynonymous	1	0
FHOD3	chr18	34205551/C//T	synonymous	1	0
FHOD3	chr18	34335224/C//T	nonsynonymous	1	0
EPG5	chr18	43447720/G//T	synonymous	1	0
SERPINB3	chr18	61322907/C//T	nonsynonymous	1	0
ZNF516	chr18	74091620/C//T	nonsynonymous	1	0
POLRMT	chr19	617326/G//A	synonymous	1	0
CSNK1G2	chr19	1969838/G//A	nonsynonymous	1	0
MOB3A	chr19	2076952/C//T	nonsynonymous	1	0
AP3D1	chr19	2114786/G//T	nonsynonymous	1	0
PLEKHJ1	chr19	2233843/G//A	nonsynonymous	1	0
AMH	chr19	2250865/G//A	nonsynonymous	1	0
C19orf35	chr19	2279068/G//A	nonsynonymous	1	0
TMPRSS9	chr19	2422173/G//A	nonsynonymous	1	0
ZNF57	chr19	2917068/C//T	nonsynonymous	1	0
TLE6	chr19	2994074/C//T	nonsynonymous	1	0
DAPK3	chr19	3959469/G//T	nonsynonymous	1	0
ZBTB7A	chr19	4054972/C//T	nonsynonymous	1	0
PTPRS	chr19	5286176/G//A	synonymous	1	0
SAFB2	chr19	5592866/C//T	nonsynonymous	1	0
ZNF557	chr19	7082930/G//C	nonsynonymous	1	0
ZNF557	chr19	7082995/G//T	nonsynonymous	1	0
ARHGEF18	chr19	7521296/A//C	synonymous	1	0
CLEC4G	chr19	7794324/C//T	synonymous	1	0
MAP2K7	chr19	7975600/T//C	nonsynonymous	1	0

TIMM44	chr19	7997819/T//C	nonsynonymous	1	0
FBN3	chr19	8196139/C//T	nonsynonymous	1	0
HNRNPM	chr19	8553704/G//A	nonsynonymous	1	0
PRAM1	chr19	8564192/G//A	nonsynonymous	1	0
MUC16	chr19	9070823/G//A	synonymous	1	0
ZNF177	chr19	9491671/C//T	nonsynonymous	1	0
DNMT1	chr19	10249170/C//A	nonsynonymous	1	0
KANK2	chr19	11286574/G//A	synonymous	1	0
DOCK6	chr19	11352717/G//A	synonymous	1	0
RGL3	chr19	11517431/G//A	synonymous	1	0
CNN1	chr19	11657660/G//A	nonsynonymous	1	0
ZNF563	chr19	12429897/G//A	synonymous	1	0
ZNF799	chr19	12501888/T//C	nonsynonymous	1	0
ZNF799	chr19	12502261/C//T	synonymous	1	0
C19orf43	chr19	12841773/C//T	synonymous	1	0
MRI1	chr19	13879811/C//T	nonsynonymous	1	0
PODNL1	chr19	14043709/C//T	nonsynonymous	1	0
PODNL1	chr19	14046601/C//T	nonsynonymous	1	0
SYDE1	chr19	15221401/C//T	nonsynonymous	1	0
WIZ	chr19	15538174/G//A	nonsynonymous	1	0
OR10H1	chr19	15918479/G//A	synonymous	1	0
CYP4F2	chr19	16000324/C//T	nonsynonymous	1	0
SMIM7	chr19	16764883/C//T	nonsynonymous	1	0
CPAMD8	chr19	17038901/G//A	synonymous	1	0
BABAM1	chr19	17382421/C//A	nonsynonymous	1	0
NXNL1	chr19	17566739/C//T	nonsynonymous	1	0
PGLS	chr19	17631789/G//A	nonsynonymous	1	0
ISYNA1	chr19	18545769/T//C	nonsynonymous	1	0
COMP	chr19	18895107/CTG//C	nonsynonymous	1	0
RFXANK	chr19	19309497/G//A	nonsynonymous	1	0
ZNF429	chr19	21719602/A//T	nonsynonymous	1	0
ZNF91	chr19	23545374/T//C	nonsynonymous	1	0
POP4	chr19	30102763/C//T	synonymous	1	0
GPATCH1	chr19	33603414/C//T	nonsynonymous	1	0
GRAMD1A	chr19	35500994/A//G	nonsynonymous	1	0
GRAMD1A	chr19	35506762/C//A	synonymous	1	0
FFAR2	chr19	35940962/C//T	nonsynonymous	1	0
KMT2B	chr19	36212289/G//A	synonymous	1	0
KMT2B	chr19	36219720/CTA//C	nonsynonymous	1	0
U2AF1L4	chr19	36234972/G//A	synonymous	1	0
NPHS1	chr19	36342558/C//T	synonymous	1	0
ZNF585B	chr19	37677399/A//T	nonsynonymous	1	0
CAPN12	chr19	39230703/G//A	synonymous	1	0
RINL	chr19	39361545/C//A	nonsynonymous	1	0
PAK4	chr19	39667346/C//T	synonymous	1	0
ZNF780B	chr19	40541063/C//T	nonsynonymous	1	0
CYP2A7	chr19	41387975/G//C	synonymous	1	0
GRIK5	chr19	42509946/C//T	nonsynonymous	1	0
CLPTM1	chr19	45480633/G//A	nonsynonymous	1	0
PPP1R37	chr19	45648175/C//T	nonsynonymous	1	0
NKPD1	chr19	45655705/G//A	nonsynonymous	1	0
RUVBL2	chr19	49514566/C//T	nonsynonymous	1	0
TRPM4	chr19	49661040/T//A	synonymous	1	0
RRAS	chr19	50139926/AGTC//A	nonsynonymous	1	0
NUP62	chr19	50412230/C//T	nonsynonymous	1	0
KCNC3	chr19	50826961/G//A	nonsynonymous	1	0
MYBPC2	chr19	50949234/G//A	nonsynonymous	1	0
ZNF614	chr19	52520008/A//G	synonymous	1	0
ZNF611	chr19	53219136/C//T	synonymous	1	0
ZNF331	chr19	54080005/A//G	nonsynonymous	1	0
NLRP12	chr19	54313858/C//T	nonsynonymous	1	0
LILRB2	chr19	54778647/C//T	nonsynonymous	1	0
LENG9	chr19	54974156/T//C	nonsynonymous	1	0
ZNF628	chr19	55994419/G//A	nonsynonymous	1	0
SSC5D	chr19	56002261/C//T	nonsynonymous	1	0
SBK3	chr19	56052682/C//T	nonsynonymous	1	0
NLRP13	chr19	56443371/C//A	nonsynonymous	1	0
ZNF444	chr19	56658390/C//T	nonsynonymous	1	0
ZNF583	chr19	56934369/C//T	synonymous	1	0
ZNF835	chr19	57175082/C//T	synonymous	1	0

ZNF419	chr19	58005429/C//T	nonsynonymous	1	0
ZNF416	chr19	58083987/G//A	nonsynonymous	1	0
ZNF418	chr19	58439152/C//T	nonsynonymous	1	0
ZNF135	chr19	58579099/G//A	nonsynonymous	1	0
ZNF324B	chr19	58967509/G//A	nonsynonymous	1	0
TRIM28	chr19	59061102/A//G	nonsynonymous	1	0
CHMP2A	chr19	59063477/TCTC//T	nonsynonymous	1	0
UBE2M	chr19	59069703/ACTC//A	nonsynonymous	1	0
TRIB3	chr20	372216/C//T	nonsynonymous	1	0
ANGPT4	chr20	853512/T//C	synonymous	1	0
TMC2	chr20	2597946/C//T	synonymous	1	0
MRPS26	chr20	3028480/G//A	nonsynonymous	1	0
ITPA	chr20	3194024/C//T	nonsynonymous	1	0
ADAM33	chr20	3655217/G//T	synonymous	1	0
SIGLEC1	chr20	3672664/G//A	nonsynonymous	1	0
HSPA12B	chr20	3732828/G//T	synonymous	1	0
ADRA1D	chr20	4228580/C//T	nonsynonymous	1	0
C20orf196	chr20	5843777/C//T	nonsynonymous	1	0
LRRN4	chr20	6022512/TCTC//T	nonsynonymous	1	0
MACROD2	chr20	16030540/G//T	synonymous	1	0
SNRPB2	chr20	16721665/G//A	synonymous	1	0
XRN2	chr20	21321480/A//G	nonsynonymous	1	0
THBD	chr20	23028959/C//T	nonsynonymous	1	0
GZF1	chr20	23351050/A//T	nonsynonymous	1	0
CST2	chr20	23804668/G//A	nonsynonymous	1	0
PYGB	chr20	25273163/C//T	synonymous	1	0
PYGB	chr20	25277023/CAAG//C	nonsynonymous	1	0
REM1	chr20	30070124/G//T	nonsynonymous	1	0
DNMT3B	chr20	31375124/CAG//C	nonsynonymous	1	0
BPIFA3	chr20	31814763/G//A	nonsynonymous	1	0
RALY	chr20	32664890/AGCGGTG//A	nonsynonymous	1	0
AHCY	chr20	32868904/T//C	nonsynonymous	1	0
MMP24	chr20	33842556/C//T	synonymous	1	0
CPNE1	chr20	34220286/T//C	nonsynonymous	1	0
ROMO1	chr20	34288723/C//T	synonymous	1	0
DLGAP4	chr20	35155500/CAG//C	synonymous	1	0
	chr20	35414971/G//A	synonymous	1	0
MANBAL	chr20	35929811/G//A	nonsynonymous	1	0
PPP1R16B	chr20	37464624/C//T	nonsynonymous	1	0
PLCG1	chr20	39801484/C//A	synonymous	1	0
PTPRT	chr20	40747137/G//A	nonsynonymous	1	0
SERINC3	chr20	43135617/C//T	nonsynonymous	1	0
SLC13A3	chr20	45192149/C//T	synonymous	1	0
PREX1	chr20	47273667/C//T	synonymous	1	0
ARFGEF2	chr20	47626942/G//T	nonsynonymous	1	0
KCNG1	chr20	49626651/C//T	synonymous	1	0
ZBP1	chr20	56179473/C//T	synonymous	1	0
STX16	chr20	57251333/G//A	nonsynonymous	1	0
LAMA5	chr20	60912868/C//T	nonsynonymous	1	0
SLC17A9	chr20	61594022/G//A	nonsynonymous	1	0
HELZ2	chr20	62197125/G//A	nonsynonymous	1	0
ZNF512B	chr20	62591275/C//T	nonsynonymous	1	0
BTG3	chr21	18981355/T//C	synonymous	1	0
BACH1	chr21	30693737/C//T	nonsynonymous	1	0
KRTAP13-2	chr21	31744546/T//A	synonymous	1	0
KRTAP20-3	chr21	32015371/T//A	synonymous	1	0
IFNGR2	chr21	34787307/C//T	synonymous	1	0
RUNX1	chr21	36253003/G//A	nonsynonymous	1	0
HLCS	chr21	38126601/C//T	synonymous	1	0
DSCR3	chr21	38600558/G//A	synonymous	1	0
WDR4	chr21	44279795/C//T	nonsynonymous	1	0
AIRE	chr21	45707415/C//T	nonsynonymous	1	0
TRPM2	chr21	45837892/C//T	nonsynonymous	1	0
KRTAP10-1	chr21	45959727/C//T	nonsynonymous	1	0
COL6A1	chr21	47414095/G//A	synonymous	1	0
PCNT	chr21	47809165/G//T	nonsynonymous	1	0
CECR1	chr22	17684517/C//T	nonsynonymous	1	0
ATP6V1E1	chr22	18077351/G//T	nonsynonymous	1	0
	chr22	18347656/T//C	synonymous	1	0
DGCR14	chr22	19121797/G//A	nonsynonymous	1	0

CLDN5	chr22	19511514/G//A	synonymous	1	0
DGCR6L	chr22	20303645/T//C	synonymous	1	0
SCARF2	chr22	20784741/AC//A	nonsynonymous	1	0
CCDC116	chr22	21988654/G//A	nonsynonymous	1	0
TOP3B	chr22	22318660/G//A	nonsynonymous	1	0
RSPH14	chr22	23401659/A//G	nonsynonymous	1	0
CRYBB3	chr22	25603057/G//A	nonsynonymous	1	0
GAL3ST1	chr22	30951135/G//A	synonymous	1	0
SMTN	chr22	31491563/C//T	nonsynonymous	1	0
MKL1	chr22	40814898/C//T	nonsynonymous	1	0
ACO2	chr22	41924512/C//T	synonymous	1	0
TTLL1	chr22	43464472/G//A	synonymous	1	0
MPPED1	chr22	43820980/C//T	synonymous	1	0
MPPED1	chr22	43901551/C//T	synonymous	1	0
FBLN1	chr22	45944507/G//A	nonsynonymous	1	0
FBLN1	chr22	45996293/C//T	synonymous	1	0
ZBED4	chr22	50279963/A//G	nonsynonymous	1	0
ZBED4	chr22	50280174/G//A	nonsynonymous	1	0
PLXNB2	chr22	50715280/ATCT//A	nonsynonymous	1	0
AKAP17A	chrX	1720304/C//T	synonymous	1	0
ARSE	chrX	2861212/C//A	nonsynonymous	1	0
SHROOM2	chrX	9912752/C//T	synonymous	1	0
MSL3	chrX	11781060/C//T	synonymous	1	0
PDHA1	chrX	19372623/T//C	synonymous	1	0
CNKSR2	chrX	21450833/A//G	nonsynonymous	1	0
KLHL34	chrX	21675110/G//A	nonsynonymous	1	0
POLA1	chrX	24766500/C//T	nonsynonymous	1	0
USP9X	chrX	41084056/T//G	nonsynonymous	1	0
GPR34	chrX	41555109/G//A	nonsynonymous	1	0
GPR34	chrX	41555509/G//A	nonsynonymous	1	0
KDM6A	chrX	44929411/CACAA//C	nonsynonymous	1	0
CCDC120	chrX	48924858/G//A	nonsynonymous	1	0
DGAT2L6	chrX	69421880/C//T	nonsynonymous	1	0
SLC7A3	chrX	70148046/C//T	nonsynonymous	1	0
GCNA	chrX	70823781/G//C	nonsynonymous	1	0
BRWD3	chrX	79938099/G//A	nonsynonymous	1	0
SATL1	chrX	84362545/C//T	nonsynonymous	1	0
SYTL4	chrX	99943435/C//T	synonymous	1	0
RAB40AL	chrX	102192261/C//T	synonymous	1	0
RAB40AL	chrX	102192301/C//T	synonymous	1	0
	chrX	107018602/G//A	synonymous	1	0
DOCK11	chrX	117727239/T//A	nonsynonymous	1	0
ATP1B4	chrX	119504684/G//A	nonsynonymous	1	0
CT47B1	chrX	120008923/G//A	nonsynonymous	1	0
TENM1	chrX	123554504/C//T	nonsynonymous	1	0
SLITRK2	chrX	144905707/C//A	synonymous	1	0
MTMR1	chrX	149867750/G//T	nonsynonymous	1	0
PNMA3	chrX	152226290/G//A	nonsynonymous	1	0
PDZD4	chrX	153073810/C//T	synonymous	1	0
AGRN	chr1	989220/G//A	synonymous	1	0
SDF4	chr1	1152775/T//G	synonymous	1	0
ACAP3	chr1	1231196/T//C	nonsynonymous	1	0
TMEM52	chr1	1850663/C//T	synonymous	1	0
SKI	chr1	2238048/G//A	synonymous	1	0
FAM213B	chr1	2522398/G//A	synonymous	1	0
ARHGEF16	chr1	3379949/A//G	nonsynonymous	1	0
MEGF6	chr1	3414992/G//T	nonsynonymous	1	0
WRAP73	chr1	3548111/G//A	nonsynonymous	1	0
TNFRSF25	chr1	6524611/C//T	nonsynonymous	1	0
SLC2A7	chr1	9078354/C//T	nonsynonymous	1	0
SLC2A5	chr1	9107757/G//A	synonymous	1	0
PRDM2	chr1	14142916/G//A	synonymous	1	0
CROCC	chr1	17266568/C//T	synonymous	1	0
KLHDC7A	chr1	18809401/G//A	synonymous	1	0
SH2D5	chr1	21048348/C//T	synonymous	1	0
LUZP1	chr1	23420706/G//A	nonsynonymous	1	0
AIM1L	chr1	26672915/G//A	synonymous	1	0
EYA3	chr1	28339675/G//A	nonsynonymous	1	0
COL16A1	chr1	32149309/T//A	nonsynonymous	1	0
YARS	chr1	33263372/C//T	nonsynonymous	1	0

PHC2	chr1	33820473/G//A	nonsynonymous	1	0
CSMD2	chr1	34043056/T//C	synonymous	1	0
CLSPN	chr1	36226719/C//T	nonsynonymous	1	0
MACF1	chr1	39816482/G//A	nonsynonymous	1	0
PABPC4	chr1	40029354/G//A	nonsynonymous	1	0
TIE1	chr1	43787300/C//T	nonsynonymous	1	0
SZT2	chr1	43892784/C//T	nonsynonymous	1	0
HYI	chr1	43919347/G//A	nonsynonymous	1	0
MOB3C	chr1	47075273/G//A	synonymous	1	0
CYP4A22	chr1	47610279/A//G	nonsynonymous	1	0
PLPP3	chr1	56977797/G//A	nonsynonymous	1	0
KANK4	chr1	62728894/G//A	synonymous	1	0
LRRC7	chr1	70504950/C//T	nonsynonymous	1	0
FPGT-TNNI3K	chr1	74808751/A//G	synonymous	1	0
ERICH3	chr1	75039054/C//T	synonymous	1	0
DPYD	chr1	97915735/G//T	synonymous	1	0
NTNG1	chr1	107867019/C//T	nonsynonymous	1	0
WDR3	chr1	118492373/G//A	nonsynonymous	1	0
HMGCS2	chr1	120307195/G//A	synonymous	1	0
BCL9	chr1	147096063/C//T	nonsynonymous	1	0
LCE4A	chr1	152681817/G//T	nonsynonymous	1	0
LCE6A	chr1	152816139/G//T	nonsynonymous	1	0
S100A7A	chr1	153390684/T//C	synonymous	1	0
DCST2	chr1	155006064/C//T	synonymous	1	0
NES	chr1	156640832/C//A	nonsynonymous	1	0
INSRR	chr1	156810756/G//C	nonsynonymous	1	0
IGSF9	chr1	159897269/T//C	nonsynonymous	1	0
ATF6	chr1	161789463/G//A	nonsynonymous	1	0
	chr1	170993552/G//A	synonymous	1	0
FMO2	chr1	171173187/C//A	nonsynonymous	1	0
CENPL	chr1	173772301/C//T	nonsynonymous	1	0
TNR	chr1	175299294/C//T	nonsynonymous	1	0
PAPPA2	chr1	176564651/C//T	synonymous	1	0
CACNA1E	chr1	181767775/G//A	synonymous	1	0
CRB1	chr1	197326074/G//A	nonsynonymous	1	0
PRELP	chr1	203453234/G//A	nonsynonymous	1	0
INTS7	chr1	212118170/TCAGA//T	nonsynonymous	1	0
EPHX1	chr1	226027607/G//A	nonsynonymous	1	0
OBSCN	chr1	228437778/G//A	synonymous	1	0
NUP133	chr1	229623219/C//T	nonsynonymous	1	0
SPRTN	chr1	231474135/T//C	synonymous	1	0
SPRTN	chr1	231488936/T//A	nonsynonymous	1	0
LYST	chr1	235926125/G//A	nonsynonymous	1	0
NID1	chr1	236142389/G//A	synonymous	1	0
AKT3	chr1	243716141/A//G	synonymous	1	0
ZNF692	chr1	249144986/G//T	synonymous	1	0
ASAP2	chr2	9531330/G//A	synonymous	1	0
GREB1	chr2	11733078/G//A	nonsynonymous	1	0
KCNS3	chr2	18112750/T//C	nonsynonymous	1	0
CENPO	chr2	25039667/C//T	synonymous	1	0
DPYSL5	chr2	27150167/C//T	nonsynonymous	1	0
ZFP36L2	chr2	43452670/CGCCGCGCT//C	nonsynonymous	1	0
SLC3A1	chr2	44531406/G//A	nonsynonymous	1	0
EPAS1	chr2	46605076/C//T	synonymous	1	0
PSME4	chr2	54114505/T//C	synonymous	1	0
SPRED2	chr2	65571892/G//A	synonymous	1	0
GKN1	chr2	69207131/C//T	synonymous	1	0
ANXA4	chr2	70008698/A//G	nonsynonymous	1	0
AUP1	chr2	74755401/G//A	synonymous	1	0
REG3A	chr2	79386576/C//T	synonymous	1	0
EIF2AK3	chr2	88885388/G//A	nonsynonymous	1	0
ARID5A	chr2	97215998/C//T	nonsynonymous	1	0
FER1L5	chr2	97359382/C//A	nonsynonymous	1	0
ACTR1B	chr2	98277102/G//A	nonsynonymous	1	0
ZAP70	chr2	98340514/G//A	synonymous	1	0
RPL31	chr2	101620642/C//T	nonsynonymous	1	0
PAX8	chr2	114035992/C//T	synonymous	1	0
RABL2A	chr2	114392711/C//T	synonymous	1	0
DPP10	chr2	116594084/C//T	synonymous	1	0
DPP10	chr2	116599789/C//A	synonymous	1	0

CLASP1	chr2	122182758/G//A	synonymous	1	0
GPR17	chr2	128409228/G//A	nonsynonymous	1	0
PTPN18	chr2	131130760/G//A	nonsynonymous	1	0
NCKAP5	chr2	133542260/T//G	nonsynonymous	1	0
MMADHC	chr2	150432267/CCAAA//C	nonsynonymous	1	0
NEB	chr2	152536281/G//A	nonsynonymous	1	0
ERMN	chr2	158177848/G//A	nonsynonymous	1	0
PLA2R1	chr2	160798643/T//C	nonsynonymous	1	0
ABCB11	chr2	169836414/G//A	nonsynonymous	1	0
HOXD10	chr2	176982075/G//T	nonsynonymous	1	0
PRKRA	chr2	179296971/G//A	synonymous	1	0
TTN	chr2	179659805/C//T	synonymous	1	0
ANKRD44	chr2	198011816/C//T	nonsynonymous	1	0
C2orf69	chr2	200776337/C//T	nonsynonymous	1	0
CASP10	chr2	202074219/G//A	nonsynonymous	1	0
ALS2CR11	chr2	202352432/C//T	nonsynonymous	1	0
ALS2CR11	chr2	202483904/G//A	synonymous	1	0
NBEAL1	chr2	203942536/T//G	nonsynonymous	1	0
MARCH4	chr2	217234862/C//T	nonsynonymous	1	0
PLCD4	chr2	219499283/G//A	nonsynonymous	1	0
BCS1L	chr2	219526204/G//T	nonsynonymous	1	0
ABCB6	chr2	220083308/TGAA//T	nonsynonymous	1	0
SPEG	chr2	220313218/G//A	synonymous	1	0
SGPP2	chr2	223423149/G//A	synonymous	1	0
CCL20	chr2	228680224/A//T	nonsynonymous	1	0
CAB39	chr2	231682475/G//A	nonsynonymous	1	0
NEU2	chr2	233899595/C//T	nonsynonymous	1	0
INPP5D	chr2	234085947/C//A	nonsynonymous	1	0
COL6A3	chr2	238268795/C//T	nonsynonymous	1	0
HDAC4	chr2	239988469/G//A	synonymous	1	0
ING5	chr2	242648665/G//A	synonymous	1	0
CHL1	chr3	403423/G//A	nonsynonymous	1	0
CNTN6	chr3	1427482/G//A	nonsynonymous	1	0
ITPR1	chr3	4752022/C//T	nonsynonymous	1	0
GRM7	chr3	7620225/C//A	synonymous	1	0
SRGAP3	chr3	9166509/G//A	nonsynonymous	1	0
ATP2B2	chr3	10370571/G//A	nonsynonymous	1	0
C3orf20	chr3	14801459/C//A	nonsynonymous	1	0
RBSN	chr3	15115637/C//G	nonsynonymous	1	0
TOP2B	chr3	25665185/G//T	nonsynonymous	1	0
CMTM7	chr3	32483464/C//T	nonsynonymous	1	0
SLC22A14	chr3	38357177/G//A	nonsynonymous	1	0
CTNNB1	chr3	41266136/T//C	nonsynonymous	1	0
ULK4	chr3	41723277/G//A	nonsynonymous	1	0
TRAK1	chr3	42242320/C//T	nonsynonymous	1	0
SNRK	chr3	43381981/G//A	nonsynonymous	1	0
TOPAZ1	chr3	44283618/C//T	nonsynonymous	1	0
CCR5	chr3	46414567/C//T	synonymous	1	0
TMIE	chr3	46747287/C//T	nonsynonymous	1	0
PRSS50	chr3	46759307/C//T	nonsynonymous	1	0
SETD2	chr3	47147573/C//T	nonsynonymous	1	0
PTPN23	chr3	47449380/TGAG//T	nonsynonymous	1	0
BSN	chr3	49693165/G//A	nonsynonymous	1	0
MST1	chr3	49725324/C//T	nonsynonymous	1	0
AMIGO3	chr3	49755784/T//C	nonsynonymous	1	0
UBA7	chr3	49845499/C//T	nonsynonymous	1	0
MANF	chr3	51422837/A//G	nonsynonymous	1	0
DCAF1	chr3	51457518/C//T	nonsynonymous	1	0
PCBP4	chr3	51994635/G//A	synonymous	1	0
ALAS1	chr3	52237952/G//A	synonymous	1	0
PHF7	chr3	52456888/G//A	nonsynonymous	1	0
NISCH	chr3	52525953/G//A	nonsynonymous	1	0
NT5DC2	chr3	52558701/G//A	nonsynonymous	1	0
NT5DC2	chr3	52563158/C//T	synonymous	1	0
CACNA1D	chr3	53766861/A//G	synonymous	1	0
FLNB	chr3	58108948/C//T	synonymous	1	0
ROBO1	chr3	78666908/G//A	nonsynonymous	1	0
DCBLD2	chr3	98568405/T//C	synonymous	1	0
USF3	chr3	113378424/T//A	nonsynonymous	1	0
GPR156	chr3	119886465/A//G	nonsynonymous	1	0

ILDR1	chr3	121707170/C//T	synonymous	1	0
PARP14	chr3	122418965/T//C	nonsynonymous	1	0
MUC13	chr3	124635234/A//G	synonymous	1	0
PLXNA1	chr3	126730914/T//C	synonymous	1	0
DNAJB8	chr3	128181453/G//A	synonymous	1	0
RPN1	chr3	128369394/G//T	nonsynonymous	1	0
H1FOO	chr3	129266245/G//A	nonsynonymous	1	0
P2RY14	chr3	150931533/A//G	nonsynonymous	1	0
RSRC1	chr3	157839961/G//A	nonsynonymous	1	0
GFM1	chr3	158364662/C//T	synonymous	1	0
SLITRK3	chr3	164906038/G//A	nonsynonymous	1	0
PIK3CA	chr3	178952007/A//G	nonsynonymous	1	0
TTC14	chr3	180322086/A//G	nonsynonymous	1	0
	chr3	183951081/A//G	synonymous	1	0
PIGG	chr4	517607/G//A	synonymous	1	0
CRIPAK	chr4	1389637/G//A	synonymous	1	0
FGFR3	chr4	1803556/C//A	synonymous	1	0
CFAP99	chr4	2438661/C//T	nonsynonymous	1	0
CFAP99	chr4	2461810/G//A	nonsynonymous	1	0
MFSD10	chr4	2934340/C//T	nonsynonymous	1	0
BOD1L1	chr4	13603250/T//C	synonymous	1	0
CLRN2	chr4	17528510/C//T	synonymous	1	0
CCDC149	chr4	24810405/G//A	nonsynonymous	1	0
UGDH	chr4	39501788/G//T	nonsynonymous	1	0
PHOX2B	chr4	41749414/A//T	synonymous	1	0
UBA6	chr4	68488568/C//T	nonsynonymous	1	0
UBA6	chr4	68530933/C//G	nonsynonymous	1	0
TMPRSS11D	chr4	68698915/G//A	synonymous	1	0
ODAM	chr4	71068035/A//T	nonsynonymous	1	0
AMTN	chr4	71396954/A//G	nonsynonymous	1	0
COX18	chr4	73935133/C//T	synonymous	1	0
SHROOM3	chr4	77675533/G//T	nonsynonymous	1	0
SEPT11	chr4	77871062/C//A	synonymous	1	0
COPS4	chr4	83978544/C//T	nonsynonymous	1	0
GRID2	chr4	94159544/G//C	nonsynonymous	1	0
ARHGEF38	chr4	106580459/C//T	synonymous	1	0
SEC24D	chr4	119661900/G//A	nonsynonymous	1	0
MAML3	chr4	140811552/A//G	synonymous	1	0
TTC29	chr4	147824706/G//A	synonymous	1	0
MAB21L2	chr4	151505150/C//T	synonymous	1	0
FHDC1	chr4	153896654/A//G	synonymous	1	0
KIAA0922	chr4	154512317/T//C	nonsynonymous	1	0
UBE2QL1	chr5	6449286/C//A	nonsynonymous	1	0
SEMA5A	chr5	9224811/C//T	synonymous	1	0
TRIO	chr5	14462967/T//A	nonsynonymous	1	0
OTULIN	chr5	14690341/G//A	nonsynonymous	1	0
CDH10	chr5	24535360/C//T	nonsynonymous	1	0
GDNF	chr5	37815999/C//CA	nonsynonymous	1	0
EGFLAM	chr5	38407070/C//T	synonymous	1	0
HCN1	chr5	45695953/G//A	synonymous	1	0
MIER3	chr5	56224635/T//C	nonsynonymous	1	0
PDE4D	chr5	59189534/G//C	synonymous	1	0
IPO11	chr5	61887492/T//C	nonsynonymous	1	0
SLCO6A1	chr5	101724475/A//G	nonsynonymous	1	0
TSLP	chr5	110411737/C//T	nonsynonymous	1	0
PRDM6	chr5	122426245/G//A	nonsynonymous	1	0
FBN2	chr5	127595357/G//A	synonymous	1	0
KDM3B	chr5	137756622/T//G	nonsynonymous	1	0
PCDHA5	chr5	140202710/C//T	synonymous	1	0
PCDHA7	chr5	140215715/C//T	nonsynonymous	1	0
PCDHA11	chr5	140248642/T//C	synonymous	1	0
PCDHA12	chr5	140257286/C//T	synonymous	1	0
PCDHB7	chr5	140554409/G//A	nonsynonymous	1	0
PCDHB11	chr5	140579920/C//T	synonymous	1	0
PCDHGB4	chr5	140769185/C//T	synonymous	1	0
PCDHGA8	chr5	140772680/G//A	synonymous	1	0
PCDHGB6	chr5	140788613/C//T	nonsynonymous	1	0
PCDHGB6	chr5	140789295/C//T	nonsynonymous	1	0
C5orf46	chr5	147281224/G//A	synonymous	1	0
ARSI	chr5	149677184/C//T	nonsynonymous	1	0

ANXA6	chr5	150509025/G//A	synonymous	1	0
FAT2	chr5	150930198/T//C	nonsynonymous	1	0
NUDCD2	chr5	162883944/G//T	nonsynonymous	1	0
SLIT3	chr5	168100254/C//A	nonsynonymous	1	0
SLIT3	chr5	168151461/C//T	nonsynonymous	1	0
UNC5A	chr5	176295797/C//T	nonsynonymous	1	0
FGFR4	chr5	176524664/G//T	nonsynonymous	1	0
NSD1	chr5	176637095/A//G	synonymous	1	0
ZNF354C	chr5	178505998/A//G	nonsynonymous	1	0
RUFY1	chr5	179020630/T//C	nonsynonymous	1	0
MGAT4B	chr5	179225383/G//A	nonsynonymous	1	0
IRF4	chr6	393304/G//A	nonsynonymous	1	0
HUS1B	chr6	656746/G//A	nonsynonymous	1	0
RIOK1	chr6	7405163/A//C	synonymous	1	0
KIF13A	chr6	17826103/G//A	nonsynonymous	1	0
DCDC2	chr6	24301956/C//T	nonsynonymous	1	0
HIST1H3E	chr6	26225583/G//A	synonymous	1	0
ZNF311	chr6	28967299/A//C	nonsynonymous	1	0
GABBR1	chr6	29600151/C//T	synonymous	1	0
PSORS1C2	chr6	31105788/C//A	nonsynonymous	1	0
PSORS1C2	chr6	31105852/C//T	nonsynonymous	1	0
LTB	chr6	31548630/G//A	synonymous	1	0
BAG6	chr6	31616682/C//T	synonymous	1	0
CLIC1	chr6	31700145/T//C	nonsynonymous	1	0
EHMT2	chr6	31848026/G//A	synonymous	1	0
HSD17B8	chr6	33172677/A//G	nonsynonymous	1	0
RING1	chr6	33179725/C//T	synonymous	1	0
ITPR3	chr6	33654823/G//A	nonsynonymous	1	0
MTCH1	chr6	36945437/A//G	nonsynonymous	1	0
NFYA	chr6	41048612/C//T	synonymous	1	0
GCM1	chr6	52999058/G//A	nonsynonymous	1	0
GFRAL	chr6	55192316/C//T	synonymous	1	0
DST	chr6	56324972/G//A	synonymous	1	0
KIAA1586	chr6	56917664/T//C	nonsynonymous	1	0
KHDRBS2	chr6	62390854/G//T	synonymous	1	0
SMAP1	chr6	71567701/C//T	synonymous	1	0
KCNQ5	chr6	73332193/G//A	synonymous	1	0
KCNQ5	chr6	73904416/C//T	nonsynonymous	1	0
ZNF292	chr6	87970160/G//T	synonymous	1	0
MDN1	chr6	90466104/G//A	nonsynonymous	1	0
MAP3K7	chr6	91226119/C//T	synonymous	1	0
EPHA7	chr6	93982109/C//A	nonsynonymous	1	0
MICAL1	chr6	109765958/G//C	nonsynonymous	1	0
GTF3C6	chr6	111288798/C//T	synonymous	1	0
REV3L	chr6	111694065/G//A	synonymous	1	0
TCF21	chr6	134210497/G//GTCTC	synonymous	1	0
UTRN	chr6	145095468/T//A	nonsynonymous	1	0
FBXO30	chr6	146121277/G//A	nonsynonymous	1	0
FBXO30	chr6	146127488/G//A	synonymous	1	0
SHPRH	chr6	146262903/G//A	synonymous	1	0
PLEKHG1	chr6	151055123/C//T	synonymous	1	0
SYNE1	chr6	152527420/C//T	synonymous	1	0
SYNE1	chr6	152652494/G//A	synonymous	1	0
SERAC1	chr6	158549197/G//A	nonsynonymous	1	0
GTF2H5	chr6	158613095/C//A	nonsynonymous	1	0
PNLDC1	chr6	160221714/C//T	synonymous	1	0
C6orf118	chr6	165715178/G//A	synonymous	1	0
AFDN	chr6	168348530/C//T	synonymous	1	0
GNA12	chr7	2771274/G//A	synonymous	1	0
AP5Z1	chr7	4827933/G//A	synonymous	1	0
RBAK	chr7	5104163/A//G	nonsynonymous	1	0
FBXL18	chr7	5541567/G//A	synonymous	1	0
FSCN1	chr7	5643177/T//C	nonsynonymous	1	0
THSD7A	chr7	11509563/CCTT//C	nonsynonymous	1	0
HOXA9	chr7	27204717/G//T	synonymous	1	0
DPY19L1	chr7	34978931/G//A	nonsynonymous	1	0
ELMO1	chr7	37382350/G//A	synonymous	1	0
VPS41	chr7	38836354/G//A	synonymous	1	0
AEBP1	chr7	44151515/T//C	synonymous	1	0
ADCY1	chr7	45725723/C//T	nonsynonymous	1	0

AUTS2	chr7	70252422/G//T	synonymous	1	0
CLDN3	chr7	73184298/T//C	nonsynonymous	1	0
GRM3	chr7	86415798/C//T	synonymous	1	0
SAMD9L	chr7	92762645/G//A	synonymous	1	0
BAIAP2L1	chr7	97935802/G//A	nonsynonymous	1	0
STAG3	chr7	99808801/G//A	nonsynonymous	1	0
GNB2	chr7	100275168/C//T	synonymous	1	0
ZAN	chr7	100365467/G//A	nonsynonymous	1	0
CDHR3	chr7	105660944/C//T	synonymous	1	0
NAMPT	chr7	105902034/C//A	nonsynonymous	1	0
PIK3CG	chr7	106508067/C//T	nonsynonymous	1	0
PIK3CG	chr7	106523549/G//T	nonsynonymous	1	0
COG5	chr7	106876918/G//A	nonsynonymous	1	0
DOCK4	chr7	111430583/C//T	nonsynonymous	1	0
PTPRZ1	chr7	121695051/C//T	synonymous	1	0
TMEM229A	chr7	123672446/C//T	synonymous	1	0
FLNC	chr7	128477738/G//A	nonsynonymous	1	0
BPGM	chr7	134346364/G//A	nonsynonymous	1	0
KIAA1549	chr7	138546052/C//T	nonsynonymous	1	0
EPHB6	chr7	142566013/T//C	nonsynonymous	1	0
TRPV5	chr7	142626135/T//G	synonymous	1	0
OR2A25	chr7	143772135/C//T	synonymous	1	0
GIMAP7	chr7	150217478/G//A	nonsynonymous	1	0
SLC4A2	chr7	150771333/G//A	nonsynonymous	1	0
SLC4A2	chr7	150771522/G//A	nonsynonymous	1	0
FASTK	chr7	150775740/T//C	nonsynonymous	1	0
ABCF2	chr7	150921922/C//T	nonsynonymous	1	0
CHPF2	chr7	150935458/G//A	synonymous	1	0
ZNF596	chr8	196305/A//G	synonymous	1	0
CLDN23	chr8	8560335/G//A	nonsynonymous	1	0
MSRA	chr8	10159113/T//C	nonsynonymous	1	0
BLK	chr8	11400760/G//A	synonymous	1	0
FGF20	chr8	16859275/C//T	synonymous	1	0
PSD3	chr8	18793584/T//A	nonsynonymous	1	0
ATP6V1B2	chr8	20072417/G//T	nonsynonymous	1	0
TNFRSF10A	chr8	23054709/T//G	synonymous	1	0
LOXL2	chr8	23190928/C//A	nonsynonymous	1	0
FZD3	chr8	28360385/C//T	synonymous	1	0
UBXN8	chr8	30620812/G//A	nonsynonymous	1	0
UNC5D	chr8	35406823/C//T	synonymous	1	0
ZNF703	chr8	37555026/C//T	nonsynonymous	1	0
ADAM32	chr8	39080734/G//A	nonsynonymous	1	0
PRKDC	chr8	48691172/G//A	nonsynonymous	1	0
PRKDC	chr8	48769760/ATC//A	nonsynonymous	1	0
PXDNL	chr8	52321615/C//T	nonsynonymous	1	0
CYP7B1	chr8	65528557/A//G	nonsynonymous	1	0
CRISPLD1	chr8	75926256/C//T	nonsynonymous	1	0
PLEKHF2	chr8	96166751/G//A	nonsynonymous	1	0
MATN2	chr8	98943567/G//A	nonsynonymous	1	0
DCAF13	chr8	104432628/CA//C	nonsynonymous	1	0
PKHD1L1	chr8	110457292/C//T	nonsynonymous	1	0
ENPP2	chr8	120638874/G//A	nonsynonymous	1	0
ZHX2	chr8	123966105/C//T	synonymous	1	0
TMEM65	chr8	125339630/A//G	nonsynonymous	1	0
TG	chr8	133918958/C//T	synonymous	1	0
ADGRB1	chr8	143623568/A//G	nonsynonymous	1	0
ARC	chr8	143694798/G//T	nonsynonymous	1	0
ARC	chr8	143695069/G//A	synonymous	1	0
PARP10	chr8	145051805/G//A	synonymous	1	0
FBXL6	chr8	145579840/C//T	synonymous	1	0
RECQL4	chr8	145739084/G//A	synonymous	1	0
LRRC14	chr8	145746161/G//A	nonsynonymous	1	0
ZNF34	chr8	145998644/G//A	synonymous	1	0
PUM3	chr9	2837263/C//A	nonsynonymous	1	0
RIC1	chr9	5732441/A//G	synonymous	1	0
PTPRD	chr9	8500774/A//G	nonsynonymous	1	0
KLHL9	chr9	21333243/C//T	nonsynonymous	1	0
DNAI1	chr9	34514385/C//A	synonymous	1	0
TPM2	chr9	35685714/C//A	nonsynonymous	1	0
ZCCHC7	chr9	37304314/C//T	synonymous	1	0

	chr9	37592392/G//T	synonymous	1	0
TJP2	chr9	71869274/G//A	nonsynonymous	1	0
APBA1	chr9	72131117/C//T	nonsynonymous	1	0
APBA1	chr9	72131845/G//A	synonymous	1	0
TRPM3	chr9	73152232/G//A	nonsynonymous	1	0
VPS13A	chr9	79891080/G//A	nonsynonymous	1	0
RASEF	chr9	85622344/G//A	synonymous	1	0
DAPK1	chr9	90254311/C//T	nonsynonymous	1	0
SPATA31E1	chr9	90502800/G//A	nonsynonymous	1	0
C9orf47	chr9	91605924/G//T	nonsynonymous	1	0
	chr9	95477424/G//A	synonymous	1	0
PHF2	chr9	96439054/C//T	nonsynonymous	1	0
COL15A1	chr9	101824293/C//T	nonsynonymous	1	0
RNF20	chr9	104302910/C//CGA	nonsynonymous	1	0
PALM2-AKAP2	chr9	112542757/C//T	synonymous	1	0
KIAA0368	chr9	114140900/A//G	nonsynonymous	1	0
FKBP15	chr9	115938956/G//A	synonymous	1	0
COL27A1	chr9	117005815/G//A	nonsynonymous	1	0
PAPPA	chr9	118950361/C//T	synonymous	1	0
ASTN2	chr9	119976939/C//T	nonsynonymous	1	0
BRINP1	chr9	121930339/C//T	nonsynonymous	1	0
TRAF1	chr9	123675634/C//T	nonsynonymous	1	0
CNTRL	chr9	123930567/G//A	nonsynonymous	1	0
DAB2IP	chr9	124545883/G//A	synonymous	1	0
LHX2	chr9	126777612/G//A	nonsynonymous	1	0
ARPC5L	chr9	127636006/G//A	nonsynonymous	1	0
ZBTB43	chr9	129595364/C//T	synonymous	1	0
URM1	chr9	131152007/C//T	synonymous	1	0
DOLK	chr9	131708453/C//T	nonsynonymous	1	0
PTPA	chr9	131890299/C//T	synonymous	1	0
RAPGEF1	chr9	134455722/T//C	nonsynonymous	1	0
NTNG2	chr9	135073572/C//T	nonsynonymous	1	0
SARDH	chr9	136599036/T//C	nonsynonymous	1	0
OLFM1	chr9	138011815/G//A	nonsynonymous	1	0
KCNT1	chr9	138664620/G//A	nonsynonymous	1	0
SEC16A	chr9	139370287/G//A	nonsynonymous	1	0
NOTCH1	chr9	139401003/G//A	synonymous	1	0
NOTCH1	chr9	139401089/G//A	nonsynonymous	1	0
FBXW5	chr9	139835485/G//A	synonymous	1	0
C8G	chr9	139839792/C//T	nonsynonymous	1	0
NPDC1	chr9	139934854/A//G	nonsynonymous	1	0
ARRDC1	chr9	140509520/C//T	synonymous	1	0
LARP4B	chr10	858884/G//A	synonymous	1	0
LARP4B	chr10	859077/TG//T	nonsynonymous	1	0
PITRM1	chr10	3207620/C//T	nonsynonymous	1	0
TUBAL3	chr10	5437426/G//A	nonsynonymous	1	0
FBXO18	chr10	5953056/C//T	synonymous	1	0
ITIH5	chr10	7605068/G//A	nonsynonymous	1	0
UPF2	chr10	11963304/C//T	nonsynonymous	1	0
SEPHS1	chr10	13364974/C//T	synonymous	1	0
SUV39H2	chr10	14941597/G//A	synonymous	1	0
VIM	chr10	17271347/G//A	synonymous	1	0
MLLT10	chr10	21823613/G//A	nonsynonymous	1	0
ARHGAP21	chr10	24886483/C//T	nonsynonymous	1	0
ABI1	chr10	27149712/A//G	synonymous	1	0
PTCHD3	chr10	27702994/C//T	synonymous	1	0
ZNF438	chr10	31139161/C//T	nonsynonymous	1	0
RET	chr10	43612056/C//T	nonsynonymous	1	0
RBP3	chr10	48390151/C//T	nonsynonymous	1	0
H2AFY2	chr10	71851605/G//A	synonymous	1	0
PPA1	chr10	71978523/C//A	nonsynonymous	1	0
C10orf11	chr10	77818442/G//A	synonymous	1	0
KCNMA1	chr10	78709021/G//A	nonsynonymous	1	0
LRIT1	chr10	85991863/G//T	synonymous	1	0
IFIT5	chr10	91177797/ACTT//A	nonsynonymous	1	0
PLCE1	chr10	95892086/C//T	synonymous	1	0
HELLS	chr10	96350209/C//T	nonsynonymous	1	0
SLIT1	chr10	98761002/G//A	nonsynonymous	1	0
RRP12	chr10	99150504/G//T	nonsynonymous	1	0
ABCC2	chr10	101560169/G//A	nonsynonymous	1	0

GBF1	chr10	104019816/C//T	nonsynonymous	1	0
COL17A1	chr10	105821178/C//T	nonsynonymous	1	0
SORCS3	chr10	106926988/A//ATTG	synonymous	1	0
SORCS1	chr10	108924007/C//T	nonsynonymous	1	0
TRUB1	chr10	116731937/A//G	nonsynonymous	1	0
STK32C	chr10	134041593/C//T	nonsynonymous	1	0
TUBGCP2	chr10	135103461/G//A	synonymous	1	0
PKP3	chr11	397420/C//T	nonsynonymous	1	0
PKP3	chr11	404597/G//A	synonymous	1	0
AP2A2	chr11	994183/C//T	nonsynonymous	1	0
MUC5B	chr11	1281870/C//T	nonsynonymous	1	0
	chr11	1507749/G//A	synonymous	1	0
OR52I1	chr11	4615644/C//T	nonsynonymous	1	0
OR52N2	chr11	5841861/G//A	nonsynonymous	1	0
	chr11	6413410/A//G	synonymous	1	0
TRIM3	chr11	6478033/G//A	nonsynonymous	1	0
TRIM3	chr11	6478232/G//A	nonsynonymous	1	0
ZNF215	chr11	6977466/A//G	nonsynonymous	1	0
ZNF214	chr11	7021162/G//A	synonymous	1	0
TUB	chr11	8111175/C//T	synonymous	1	0
STK33	chr11	8413932/C//T	synonymous	1	0
AMPD3	chr11	10500123/C//T	nonsynonymous	1	0
MRVI1	chr11	10647725/G//A	nonsynonymous	1	0
MICAL2	chr11	12225989/T//G	nonsynonymous	1	0
MICAL2	chr11	12280095/CAA//C	nonsynonymous	1	0
CYP2R1	chr11	14900968/T//C	nonsynonymous	1	0
SOX6	chr11	15994483/C//T	nonsynonymous	1	0
LDHAL6A	chr11	18499264/G//T	nonsynonymous	1	0
MRGPRX1	chr11	18956155/G//A	synonymous	1	0
ZDHHC13	chr11	19194340/C//T	nonsynonymous	1	0
DEPDC7	chr11	33050316/C//A	nonsynonymous	1	0
APIP	chr11	34937823/G//A	synonymous	1	0
ALKBH3	chr11	43911301/A//T	nonsynonymous	1	0
PACSIN3	chr11	47204043/C//T	nonsynonymous	1	0
FOLH1	chr11	49196466/A//G	synonymous	1	0
OR4C13	chr11	49974299/G//A	nonsynonymous	1	0
OR8U8	chr11	56143125/C//T	nonsynonymous	1	0
VWCE	chr11	61042027/C//T	nonsynonymous	1	0
PPP1R32	chr11	61252271/C//T	nonsynonymous	1	0
METTL12	chr11	62434856/G//A	synonymous	1	0
FLRT1	chr11	63884457/G//A	nonsynonymous	1	0
ESRRA	chr11	64082287/G//A	nonsynonymous	1	0
ZFPL1	chr11	64855516/G//A	nonsynonymous	1	0
CAPN1	chr11	64978746/A//G	nonsynonymous	1	0
SNX32	chr11	65617637/C//T	nonsynonymous	1	0
LRFN4	chr11	66626391/C//T	synonymous	1	0
PITPNM1	chr11	67267252/C//T	nonsynonymous	1	0
ARHGEF17	chr11	73073129/G//A	synonymous	1	0
UCP2	chr11	73687954/C//T	nonsynonymous	1	0
LRRC32	chr11	76371063/C//T	nonsynonymous	1	0
TENM4	chr11	78381064/C//T	nonsynonymous	1	0
DLG2	chr11	83770417/C//T	nonsynonymous	1	0
TYR	chr11	88911457/C//T	synonymous	1	0
NAALAD2	chr11	89896599/T//C	nonsynonymous	1	0
FUT4	chr11	94277373/A//T	nonsynonymous	1	0
CNTN5	chr11	99786867/C//T	nonsynonymous	1	0
AASDHPPT	chr11	105965377/A//G	nonsynonymous	1	0
C11orf65	chr11	108277545/T//C	nonsynonymous	1	0
C11orf87	chr11	109294708/C//T	nonsynonymous	1	0
PPP2R1B	chr11	111631645/C//T	nonsynonymous	1	0
PLET1	chr11	112126197/C//T	synonymous	1	0
NCAM1	chr11	113076266/C//T	nonsynonymous	1	0
UBE4A	chr11	118244312/C//T	nonsynonymous	1	0
DDX6	chr11	118650364/A//G	nonsynonymous	1	0
BCL9L	chr11	118773232/C//T	nonsynonymous	1	0
USP2	chr11	119243468/C//A	nonsynonymous	1	0
HEPACAM	chr11	124794672/T//C	nonsynonymous	1	0
PKNOX2	chr11	125299986/G//A	nonsynonymous	1	0
CHEK1	chr11	125499144/C//T	nonsynonymous	1	0
GLB1L3	chr11	134151921/C//T	nonsynonymous	1	0

IQSEC3	chr12	248095/C//T	synonymous	1	0
	chr12	977855/G//A	synonymous	1	0
DCP1B	chr12	2058396/G//A	synonymous	1	0
CACNA1C	chr12	2705034/G//A	synonymous	1	0
VWF	chr12	6058291/G//A	nonsynonymous	1	0
CHD4	chr12	6680061/G//A	nonsynonymous	1	0
LPAR5	chr12	6730016/C//A	synonymous	1	0
DPPA3	chr12	7868009/G//A	nonsynonymous	1	0
FAM90A1	chr12	8377454/C//T	synonymous	1	0
KLRG1	chr12	9147747/A//G	nonsynonymous	1	0
TAS2R50	chr12	11139130/A//C	nonsynonymous	1	0
GRIN2B	chr12	13715819/C//A	nonsynonymous	1	0
ABCC9	chr12	22065878/A//G	synonymous	1	0
OVCH1	chr12	29596370/C//T	synonymous	1	0
KIAA1551	chr12	32134815/G//A	nonsynonymous	1	0
PKP2	chr12	33030886/C//T	nonsynonymous	1	0
ADAMTS20	chr12	43925860/G//A	synonymous	1	0
DBX2	chr12	45444525/C//T	synonymous	1	0
VDR	chr12	48251296/G//A	synonymous	1	0
VDR	chr12	48251318/T//C	nonsynonymous	1	0
KMT2D	chr12	49438647/G//A	nonsynonymous	1	0
PRPF40B	chr12	50027752/C//T	nonsynonymous	1	0
LARP4	chr12	50867293/A//G	synonymous	1	0
KRT71	chr12	52941744/G//A	nonsynonymous	1	0
KRT4	chr12	53207592/C//CCACCAGTGCCGA/	nonsynonymous	1	0
TNS2	chr12	53453144/C//T	synonymous	1	0
PCBP2	chr12	53874909/GTA//G	synonymous	1	0
NPFF	chr12	53900671/G//A	synonymous	1	0
HOXC10	chr12	54379463/G//A	synonymous	1	0
ITGA7	chr12	56089360/A//G	nonsynonymous	1	0
DGKA	chr12	56332316/C//T	nonsynonymous	1	0
LRP1	chr12	57600350/G//T	nonsynonymous	1	0
SLC16A7	chr12	60169201/C//T	synonymous	1	0
C12orf66	chr12	64609539/T//C	nonsynonymous	1	0
THAP2	chr12	72070679/C//T	nonsynonymous	1	0
TPH2	chr12	72372867/C//T	nonsynonymous	1	0
LUM	chr12	91498015/C//T	nonsynonymous	1	0
FGD6	chr12	95602654/G//A	synonymous	1	0
UTP20	chr12	101728224/A//C	nonsynonymous	1	0
STAB2	chr12	104157343/G//A	nonsynonymous	1	0
SVOP	chr12	109313446/C//T	synonymous	1	0
ACACB	chr12	109665250/C//T	synonymous	1	0
PPTC7	chr12	111020850/C//G	synonymous	1	0
CUX2	chr12	111779832/G//A	nonsynonymous	1	0
ACAD10	chr12	112185167/C//T	synonymous	1	0
HECTD4	chr12	112645735/G//A	nonsynonymous	1	0
RPH3A	chr12	113313513/G//A	nonsynonymous	1	0
OAS3	chr12	113405292/C//T	nonsynonymous	1	0
SLC8B1	chr12	113758716/C//T	synonymous	1	0
TBX5	chr12	114841611/C//T	synonymous	1	0
TBX3	chr12	115112047/C//T	nonsynonymous	1	0
NOS1	chr12	117768312/G//A	nonsynonymous	1	0
TAOK3	chr12	118598162/G//A	nonsynonymous	1	0
GCN1	chr12	120599816/C//T	nonsynonymous	1	0
RHOF	chr12	122217439/G//A	nonsynonymous	1	0
RSRC2	chr12	122990128/TTG//T	nonsynonymous	1	0
DNAH10	chr12	124323216/G//A	nonsynonymous	1	0
TMEM132C	chr12	128900017/C//T	nonsynonymous	1	0
GOLGA3	chr12	133373261/G//A	nonsynonymous	1	0
MRPL57	chr13	21750479/C//A	synonymous	1	0
NAA16	chr13	41943362/A//T	nonsynonymous	1	0
RGCC	chr13	42031789/G//A	synonymous	1	0
TSC22D1	chr13	45147467/C//T	nonsynonymous	1	0
TRIM13	chr13	50586337/C//G	nonsynonymous	1	0
KLHL1	chr13	70681704/C//G	nonsynonymous	1	0
PIBF1	chr13	73482668/G//T	nonsynonymous	1	0
RNF113B	chr13	98829292/G//A	nonsynonymous	1	0
FARP1	chr13	99093033/CG//C	nonsynonymous	1	0
NALCN	chr13	101742321/C//T	synonymous	1	0
MYO16	chr13	109318317/C//T	nonsynonymous	1	0

ADPRHL1	chr13	114077173/G//A	synonymous	1	0
TMCO3	chr13	114150011/C//T	nonsynonymous	1	0
TFDP1	chr13	114285960/C//T	nonsynonymous	1	0
OR4N2	chr14	20295777/C//T	nonsynonymous	1	0
SUPT16H	chr14	21825476/C//T	nonsynonymous	1	0
MYH7	chr14	23885391/C//T	nonsynonymous	1	0
MYH7	chr14	23899118/G//A	nonsynonymous	1	0
JPH4	chr14	24045579/G//A	nonsynonymous	1	0
DHRS4	chr14	24424359/A//G	nonsynonymous	1	0
DCAF11	chr14	24590580/G//A	nonsynonymous	1	0
HECTD1	chr14	31598407/G//A	synonymous	1	0
RALGAPA1	chr14	36097007/T//C	nonsynonymous	1	0
SEC23A	chr14	39554964/A//G	nonsynonymous	1	0
RPL10L	chr14	47120649/G//T	synonymous	1	0
SAV1	chr14	51132309/A//G	synonymous	1	0
NIN	chr14	51223733/C//T	nonsynonymous	1	0
TMEM260	chr14	57052566/G//A	nonsynonymous	1	0
NAA30	chr14	57866596/G//A	nonsynonymous	1	0
FAM71D	chr14	67669979/TCTC//T	nonsynonymous	1	0
ADAM20	chr14	70991442/C//T	synonymous	1	0
POMT2	chr14	77745130/G//A	synonymous	1	0
GSTZ1	chr14	77791259/G//A	nonsynonymous	1	0
TTC8	chr14	89336481/G//A	nonsynonymous	1	0
UNC79	chr14	94088466/C//T	synonymous	1	0
SLC25A47	chr14	100795149/C//A	synonymous	1	0
CDC42BPB	chr14	103440469/G//A	nonsynonymous	1	0
EIF5	chr14	103805657/G//A	nonsynonymous	1	0
BAG5	chr14	104026517/G//A	nonsynonymous	1	0
	chr14	104151375/T//C	synonymous	1	0
PPP1R13B	chr14	104216205/G//A	nonsynonymous	1	0
ADSSL1	chr14	105209481/C//T	synonymous	1	0
CEP170B	chr14	105350422/C//T	nonsynonymous	1	0
	chr15	26874149/A//AT	synonymous	1	0
NSMCE3	chr15	29560916/G//A	synonymous	1	0
FAN1	chr15	31200356/C//T	nonsynonymous	1	0
TRPM1	chr15	31352774/C//T	synonymous	1	0
THBS1	chr15	39882019/G//A	nonsynonymous	1	0
C15orf52	chr15	40631790/G//A	nonsynonymous	1	0
KNL1	chr15	40949602/A//T	nonsynonymous	1	0
EHD4	chr15	42211695/G//A	nonsynonymous	1	0
PLA2G4F	chr15	42446415/C//T	nonsynonymous	1	0
CDAN1	chr15	43020434/G//A	nonsynonymous	1	0
CGNL1	chr15	57731224/C//T	nonsynonymous	1	0
HERC1	chr15	63946430/C//T	nonsynonymous	1	0
CILP	chr15	65489551/C//T	nonsynonymous	1	0
TLE3	chr15	70358456/T//C	synonymous	1	0
	chr15	74501851/CA//C	synonymous	1	0
CLK3	chr15	74920957/C//T	nonsynonymous	1	0
COMMD4	chr15	75631682/C//T	nonsynonymous	1	0
MAN2C1	chr15	75655033/C//T	nonsynonymous	1	0
ANKRD34C	chr15	79586549/C//G	nonsynonymous	1	0
SLC28A1	chr15	85487883/G//A	nonsynonymous	1	0
PDE8A	chr15	85652353/C//T	nonsynonymous	1	0
MFGE8	chr15	89442687/C//T	nonsynonymous	1	0
CRTC3	chr15	91073394/C//A	synonymous	1	0
LINS1	chr15	101115224/T//C	nonsynonymous	1	0
RHBDF1	chr16	111928/G//A	nonsynonymous	1	0
LUC7L	chr16	240066/C//T	nonsynonymous	1	0
	chr16	319611/A//G	synonymous	1	0
BAIAP3	chr16	1392906/T//C	synonymous	1	0
IFT140	chr16	1573890/G//A	nonsynonymous	1	0
CCNF	chr16	2507097/C//T	synonymous	1	0
ZSCAN10	chr16	3142063/G//T	synonymous	1	0
NAA60	chr16	3533547/C//T	synonymous	1	0
CREBBP	chr16	3843460/C//T	synonymous	1	0
C16orf71	chr16	4787802/C//T	nonsynonymous	1	0
	chr16	10855356/C//T	synonymous	1	0
GSPT1	chr16	11988819/T//C	synonymous	1	0
GSPT1	chr16	11990538/G//A	nonsynonymous	1	0
SNX29	chr16	12662508/G//T	synonymous	1	0

SHISA9	chr16	13328918/G//T	nonsynonymous	1	0
NDE1	chr16	15785111/G//A	nonsynonymous	1	0
ABCC6	chr16	16248755/C//T	nonsynonymous	1	0
ERN2	chr16	23702482/C//T	nonsynonymous	1	0
ATP2A1	chr16	28898821/C//T	nonsynonymous	1	0
KCTD13	chr16	29918231/C//T	nonsynonymous	1	0
HIRIP3	chr16	30006878/C//A	nonsynonymous	1	0
FBRS	chr16	30680555/C//T	synonymous	1	0
SRCAP	chr16	30744736/G//A	nonsynonymous	1	0
ZNF668	chr16	31072779/A//G	synonymous	1	0
KAT8	chr16	31139397/C//A	synonymous	1	0
ITFG1	chr16	47195698/G//T	nonsynonymous	1	0
PHKB	chr16	47732398/G//A	nonsynonymous	1	0
N4BP1	chr16	48595606/C//A	nonsynonymous	1	0
SALL1	chr16	51175656/G//A	synonymous	1	0
AMFR	chr16	56396748/C//T	synonymous	1	0
SLC12A3	chr16	56916364/C//T	nonsynonymous	1	0
RSPRY1	chr16	57242022/G//T	nonsynonymous	1	0
GOT2	chr16	58743402/C//A	nonsynonymous	1	0
DYNC1LI2	chr16	66761662/C//T	nonsynonymous	1	0
ATP6V0D1	chr16	67472364/G//A	synonymous	1	0
ATP6V0D1	chr16	67472376/G//A	synonymous	1	0
CARMIL2	chr16	67691181/G//A	nonsynonymous	1	0
SLC7A6	chr16	68321751/T//C	nonsynonymous	1	0
CYB5B	chr16	69482040/G//A	nonsynonymous	1	0
WWOX	chr16	78458783/G//T	nonsynonymous	1	0
TAF1C	chr16	84215485/A//C	nonsynonymous	1	0
FBXO31	chr16	87367769/G//A	nonsynonymous	1	0
ZNF469	chr16	88505632/G//A	synonymous	1	0
CTU2	chr16	88781021/ACCT//A	nonsynonymous	1	0
MC1R	chr16	89986149/G//A	synonymous	1	0
CLUH	chr17	2593725/G//T	synonymous	1	0
CLUH	chr17	2605316/G//A	nonsynonymous	1	0
ATP2A3	chr17	3850810/G//A	nonsynonymous	1	0
ANKFY1	chr17	4075870/C//T	synonymous	1	0
SPNS2	chr17	4436596/G//A	nonsynonymous	1	0
SLC16A11	chr17	6945391/G//A	synonymous	1	0
CLDN7	chr17	7163799/A//G	nonsynonymous	1	0
C17orf74	chr17	7329693/G//A	nonsynonymous	1	0
SLC35G6	chr17	7385571/C//T	nonsynonymous	1	0
ATP1B2	chr17	7558878/G//A	nonsynonymous	1	0
TP53	chr17	7577139/G//A	nonsynonymous	1	0
WRAP53	chr17	7606652/G//A	nonsynonymous	1	0
PER1	chr17	8050655/G//A	synonymous	1	0
BORCS6	chr17	8092761/G//T	nonsynonymous	1	0
MYH13	chr17	10214508/G//A	nonsynonymous	1	0
MYH13	chr17	10247249/C//T	nonsynonymous	1	0
MYH3	chr17	10541679/C//T	nonsynonymous	1	0
MYH3	chr17	10541714/C//T	synonymous	1	0
MYH3	chr17	10542915/C//T	nonsynonymous	1	0
SCO1	chr17	10600618/A//C	synonymous	1	0
DNAH9	chr17	11645592/ACT//A	nonsynonymous	1	0
RAI1	chr17	17698713/G//T	synonymous	1	0
RAI1	chr17	17699465/C//T	nonsynonymous	1	0
SLC5A10	chr17	18922740/G//A	nonsynonymous	1	0
EPN2	chr17	19237285/G//A	synonymous	1	0
UNC119	chr17	26874353/G//A	nonsynonymous	1	0
SGK494	chr17	26938421/G//A	nonsynonymous	1	0
CRYBA1	chr17	27577319/G//A	nonsynonymous	1	0
C17orf75	chr17	30661534/A//G	synonymous	1	0
C17orf75	chr17	30669185/T//C	synonymous	1	0
TMEM132E	chr17	32953699/G//A	synonymous	1	0
TMEM132E	chr17	32955640/C//T	nonsynonymous	1	0
ARHGAP23	chr17	36623380/G//A	nonsynonymous	1	0
GSDMA	chr17	38122558/C//T	nonsynonymous	1	0
WIPF2	chr17	38421186/C//T	nonsynonymous	1	0
KRT23	chr17	39081703/G//A	nonsynonymous	1	0
KRT40	chr17	39135115/C//A	nonsynonymous	1	0
KRT33B	chr17	39521211/C//T	nonsynonymous	1	0
DHX58	chr17	40263421/G//A	nonsynonymous	1	0

NAGLU	chr17	40696205/CAAG//C	nonsynonymous	1	0
MLX	chr17	40723674/G//A	synonymous	1	0
AOC2	chr17	40997089/G//A	nonsynonymous	1	0
HDAC5	chr17	42162500/G//T	nonsynonymous	1	0
SPATA32	chr17	43331872/G//A	nonsynonymous	1	0
NPEPPS	chr17	45682757/A//G	nonsynonymous	1	0
TAC4	chr17	47918950/G//A	synonymous	1	0
EME1	chr17	48456881/C//T	synonymous	1	0
RSAD1	chr17	48562235/T//C	synonymous	1	0
CACNA1G	chr17	48683279/C//T	synonymous	1	0
VEZF1	chr17	56056607/C//T	synonymous	1	0
MKS1	chr17	56292126/C//T	nonsynonymous	1	0
LPO	chr17	56324994/T//C	nonsynonymous	1	0
USP32	chr17	58258825/T//C	nonsynonymous	1	0
SCN4A	chr17	62025364/G//A	synonymous	1	0
C17orf80	chr17	71232845/G//T	nonsynonymous	1	0
CEP131	chr17	79164443/G//A	nonsynonymous	1	0
CEP131	chr17	79167880/C//T	nonsynonymous	1	0
ASPSCR1	chr17	79966930/C//T	synonymous	1	0
GPS1	chr17	80012855/C//T	synonymous	1	0
RAB40B	chr17	80615972/A//G	nonsynonymous	1	0
EMILIN2	chr18	2913162/C//T	synonymous	1	0
EPB41L3	chr18	5396264/A//G	nonsynonymous	1	0
LAMA1	chr18	7117714/G//A	synonymous	1	0
PTPRM	chr18	8376517/C//T	synonymous	1	0
APCDD1	chr18	10487720/C//T	synonymous	1	0
ANKRD30B	chr18	14791477/T//C	synonymous	1	0
CDH2	chr18	25565119/G//A	nonsynonymous	1	0
GAREM1	chr18	29847792/G//A	synonymous	1	0
DTNA	chr18	32398248/G//A	nonsynonymous	1	0
ELOA2	chr18	44561289/G//A	nonsynonymous	1	0
ALPK2	chr18	56246909/C//T	nonsynonymous	1	0
PARD6G	chr18	77917975/C//T	synonymous	1	0
PTBP1	chr19	810702/G//A	nonsynonymous	1	0
ARID3A	chr19	964240/T//C	synonymous	1	0
C19orf71	chr19	3543894/G//A	synonymous	1	0
CACTIN	chr19	3612372/C//A	nonsynonymous	1	0
TJP3	chr19	3733900/G//A	synonymous	1	0
ANKRD24	chr19	4199727/C//A	synonymous	1	0
CHAF1A	chr19	4409651/G//A	synonymous	1	0
CD70	chr19	6586309/C//T	nonsynonymous	1	0
MUC16	chr19	9049911/G//A	nonsynonymous	1	0
MUC16	chr19	9074046/G//A	nonsynonymous	1	0
MUC16	chr19	9089228/C//A	nonsynonymous	1	0
OR1M1	chr19	9204102/T//C	nonsynonymous	1	0
MRPL4	chr19	10369182/G//A	nonsynonymous	1	0
CCDC151	chr19	11531472/A//G	synonymous	1	0
ELOF1	chr19	11665155/C//T	nonsynonymous	1	0
ZNF333	chr19	14829744/G//C	nonsynonymous	1	0
CYP4F3	chr19	15760857/G//A	nonsynonymous	1	0
F2RL3	chr19	17000886/G//A	synonymous	1	0
MYO9B	chr19	17278762/C//T	nonsynonymous	1	0
MVB12A	chr19	17534317/G//A	nonsynonymous	1	0
MAST3	chr19	18241518/C//A	synonymous	1	0
PDE4C	chr19	18322032/C//G	nonsynonymous	1	0
PGPEP1	chr19	18468322/G//A	nonsynonymous	1	0
CILP2	chr19	19655085/T//C	synonymous	1	0
ZNF90	chr19	20230112/T//C	synonymous	1	0
ZNF431	chr19	21365480/C//T	nonsynonymous	1	0
ZNF536	chr19	30934676/C//T	synonymous	1	0
KCTD15	chr19	34302423/C//T	nonsynonymous	1	0
ARHGAP33	chr19	36268753/G//A	synonymous	1	0
NFKBID	chr19	36386971/G//T	nonsynonymous	1	0
ZNF566	chr19	36939976/C//T	nonsynonymous	1	0
SYCN	chr19	39694735/C//T	nonsynonymous	1	0
LRFN1	chr19	39805798/G//A	nonsynonymous	1	0
FCGBP	chr19	40421528/G//A	nonsynonymous	1	0
ARHGEF1	chr19	42396432/G//A	nonsynonymous	1	0
CBLC	chr19	45293332/G//A	nonsynonymous	1	0
NECTIN2	chr19	45349844/T//C	nonsynonymous	1	0

PPP5C	chr19	46878954/G//A	nonsynonymous	1	0
C5AR1	chr19	47823651/G//A	nonsynonymous	1	0
SLC8A2	chr19	47960638/C//T	nonsynonymous	1	0
SNRNP70	chr19	49601737/G//A	nonsynonymous	1	0
NOSIP	chr19	50059077/G//A	synonymous	1	0
IRF3	chr19	50163053/G//A	nonsynonymous	1	0
IRF3	chr19	50166616/G//A	synonymous	1	0
AP2A1	chr19	50306448/T//C	nonsynonymous	1	0
MED25	chr19	50331796/C//T	synonymous	1	0
ACPT	chr19	51298205/G//T	nonsynonymous	1	0
CNOT3	chr19	54653310/G//A	synonymous	1	0
KIR2DS4	chr19	55344229/A//G	nonsynonymous	1	0
NLRP7	chr19	55453071/C//T	synonymous	1	0
SSC5D	chr19	56011570/C//T	nonsynonymous	1	0
SSC5D	chr19	56030241/G//A	nonsynonymous	1	0
FIZ1	chr19	56104952/G//A	nonsynonymous	1	0
ZNF784	chr19	56133340/C//T	nonsynonymous	1	0
NLRP4	chr19	56382262/C//T	synonymous	1	0
ZNF835	chr19	57175947/G//A	nonsynonymous	1	0
ZNF587B	chr19	58341914/G//A	synonymous	1	0
ZNF837	chr19	58879155/G//C	synonymous	1	0
ADRA1D	chr20	4228780/G//A	synonymous	1	0
JAG1	chr20	10620587/C//T	synonymous	1	0
GGTLC1	chr20	23966394/G//A	synonymous	1	0
BPIFB3	chr20	31643318/C//T	nonsynonymous	1	0
BPIFB4	chr20	31672739/G//A	nonsynonymous	1	0
SNTA1	chr20	32026691/G//A	nonsynonymous	1	0
MYH7B	chr20	33584569/T//C	synonymous	1	0
PLCG1	chr20	39766415/G//A	nonsynonymous	1	0
SEMG2	chr20	43851727/C//T	nonsynonymous	1	0
ZNF335	chr20	44599934/C//T	nonsynonymous	1	0
MMP9	chr20	44639830/C//A	nonsynonymous	1	0
ZNF334	chr20	45130548/G//A	nonsynonymous	1	0
SPATA2	chr20	48522331/C//T	nonsynonymous	1	0
KCNG1	chr20	49621062/G//A	synonymous	1	0
KCNG1	chr20	49626763/G//A	nonsynonymous	1	0
ZBP1	chr20	56186809/G//A	nonsynonymous	1	0
ZBP1	chr20	56186869/C//T	nonsynonymous	1	0
NELFCD	chr20	57564070/C//T	synonymous	1	0
CDH4	chr20	60348109/G//T	nonsynonymous	1	0
ADRM1	chr20	60883124/G//A	nonsynonymous	1	0
LAMA5	chr20	60886347/C//T	nonsynonymous	1	0
SLCO4A1	chr20	61288443/G//A	nonsynonymous	1	0
KCNQ2	chr20	62071069/A//G	synonymous	1	0
UCKL1	chr20	62572189/C//T	synonymous	1	0
LTN1	chr21	30357231/G//A	nonsynonymous	1	0
KRTAP13-1	chr21	31768653/C//A	synonymous	1	0
SCAF4	chr21	33057461/C//T	nonsynonymous	1	0
URB1	chr21	33721744/G//T	nonsynonymous	1	0
KCNJ6	chr21	39087292/G//A	synonymous	1	0
ETS2	chr21	40191697/G//A	synonymous	1	0
RIPK4	chr21	43161633/G//A	synonymous	1	0
ABCG1	chr21	43710300/C//T	synonymous	1	0
SLC37A1	chr21	43985955/G//A	nonsynonymous	1	0
PFKL	chr21	45726593/C//T	nonsynonymous	1	0
TSPEAR	chr21	45953608/C//T	nonsynonymous	1	0
COL6A1	chr21	47423939/C//T	synonymous	1	0
FTCD	chr21	47574209/G//A	nonsynonymous	1	0
DIP2A	chr21	47929239/G//A	nonsynonymous	1	0
DIP2A	chr21	47976917/G//A	synonymous	1	0
PRMT2	chr21	48069494/C//T	nonsynonymous	1	0
MICAL3	chr22	18378155/C//T	synonymous	1	0
ARVCF	chr22	19978191/G//A	nonsynonymous	1	0
ZDHHC8	chr22	20127115/C//T	nonsynonymous	1	0
ZDHHC8	chr22	20132832/G//C	nonsynonymous	1	0
MAPK1	chr22	22127163/T//A	nonsynonymous	1	0
SMARCB1	chr22	24175804/C//T	synonymous	1	0
SLC2A11	chr22	24210820/G//A	synonymous	1	0
MN1	chr22	28192923/G//T	nonsynonymous	1	0
TTC28	chr22	28394670/A//G	synonymous	1	0

PES1	chr22	30976629/C//A	nonsynonymous	1	0
RASD2	chr22	35947872/C//T	synonymous	1	0
IL2RB	chr22	37524301/C//A	synonymous	1	0
APOBEC3F	chr22	39448268/C//T	nonsynonymous	1	0
CACNA1I	chr22	40080499/G//A	nonsynonymous	1	0
TTLL1	chr22	43459882/C//T	synonymous	1	0
TTLL12	chr22	43564087/G//C	nonsynonymous	1	0
PKDREJ	chr22	46655196/T//C	nonsynonymous	1	0
MAP3K15	chrX	19443779/T//C	nonsynonymous	1	0
HDAC6	chrX	48681632/G//A	synonymous	1	0
PIM2	chrX	48775087/G//A	nonsynonymous	1	0
ITIH6	chrX	54783724/G//T	nonsynonymous	1	0
STARD8	chrX	67940161/G//A	nonsynonymous	1	0
ATRX	chrX	76939489/A//G	nonsynonymous	1	0
RPA4	chrX	96140039/G//A	nonsynonymous	1	0
ARMCX4	chrX	100749566/C//T	nonsynonymous	1	0
MUM1L1	chrX	105450853/C//T	synonymous	1	0
COL4A5	chrX	107842006/G//A	synonymous	1	0
DOCK11	chrX	117815215/C//T	synonymous	1	0
ELF4	chrX	129201398/A//C	nonsynonymous	1	0
L1CAM	chrX	153134085/C//T	nonsynonymous	1	0
TKTL1	chrX	153556271/C//T	nonsynonymous	1	0
LIN9	chr1	226453930/A//G	nonsynonymous	0	1
TET3	chr2	74326594/C//T	synonymous	0	1
HOXD1	chr2	177053577/C//T	synonymous	0	1
ATRIP	chr3	48498707/A//G	synonymous	0	1
LRBA	chr4	151814267/A//G	nonsynonymous	0	1
DBH	chr9	136501772/C//T	synonymous	0	1
MUC6	chr11	1016795/C//G	nonsynonymous	0	1
PITPNM1	chr11	67266256/A//G	nonsynonymous	0	1
UBC	chr12	125396833/G//A	synonymous	0	1
VASH1	chr14	77229481/A//G	synonymous	0	1
HSD3B7	chr16	30999326/C//T	nonsynonymous	0	1
ZNF14	chr19	19843919/A//G	synonymous	0	1
ZNF726	chr19	24115813/G//A	nonsynonymous	0	1
CELA2A	chr1	15783656/G//A	nonsynonymous	0	1
CSF1	chr1	110459975/A//G	nonsynonymous	0	1
PTGFRN	chr1	117529590/G//A	synonymous	0	1
RAB13	chr1	153957224/T//C	nonsynonymous	0	1
LMNA	chr1	156084775/G//T	synonymous	0	1
TMEM79	chr1	156255604/A//G	nonsynonymous	0	1
PEAR1	chr1	156883273/T//A	nonsynonymous	0	1
F13B	chr1	197031980/G//A	synonymous	0	1
DISC1	chr1	232172585/C//T	synonymous	0	1
ASPRV1	chr2	70188487/C//T	nonsynonymous	0	1
TRIM43	chr2	96260866/G//A	synonymous	0	1
GALNT5	chr2	158114910/G//T	nonsynonymous	0	1
TTN	chr2	179584922/G//C	nonsynonymous	0	1
KRBOX1	chr3	42983443/C//T	synonymous	0	1
USP4	chr3	49315781/CT//C	nonsynonymous	0	1
ATP13A4	chr3	193132381/C//T	nonsynonymous	0	1
RBM47	chr4	40440935/C//T	synonymous	0	1
SHROOM3	chr4	77660625/T//C	synonymous	0	1
SLC6A3	chr5	1409168/C//T	nonsynonymous	0	1
UGT3A1	chr5	35954342/T//C	nonsynonymous	0	1
PGGT1B	chr5	114548188/G//A	nonsynonymous	0	1
PCDHA1	chr5	140165983/G//A	synonymous	0	1
C6orf10	chr6	32337746/C//T	nonsynonymous	0	1
TCTE1	chr6	44255348/G//C	nonsynonymous	0	1
FAM83B	chr6	54791268/G//T	nonsynonymous	0	1
SYNE1	chr6	152719846/C//G	nonsynonymous	0	1
PVRIG	chr7	99818419/C//T	synonymous	0	1
SLC26A3	chr7	107430134/C//G	nonsynonymous	0	1
ZNF425	chr7	148801134/T//A	nonsynonymous	0	1
ZNF425	chr7	148801141/T//C	nonsynonymous	0	1
FAM120A	chr9	96318673/C//G	nonsynonymous	0	1
PRRX2	chr9	132482974/G//A	nonsynonymous	0	1
BICC1	chr10	60562879/C//T	synonymous	0	1
MFSD13A	chr10	104231068/C//T	nonsynonymous	0	1
PTDSS2	chr11	490545/G//T	nonsynonymous	0	1

RPS6KA4	chr11	64138105/C//T	synonymous	0	1
EHBP1L1	chr11	65343766/C//T	synonymous	0	1
TYR	chr11	88911176/C//T	nonsynonymous	0	1
PGR	chr11	100998499/C//T	nonsynonymous	0	1
CMAS	chr12	22214284/C//T	synonymous	0	1
HELB	chr12	66725214/C//T	nonsynonymous	0	1
DNAH10	chr12	124341628/G//A	nonsynonymous	0	1
PSMC6	chr14	53194225/G//A	nonsynonymous	0	1
EVL	chr14	100610098/T//C	synonymous	0	1
ZSCAN29	chr15	43656419/G//A	nonsynonymous	0	1
BCAR1	chr16	75263928/C//A	synonymous	0	1
AATF	chr17	35345911/C//G	nonsynonymous	0	1
MAP3K14-AS1	chr17	43344611/T//C	synonymous	0	1
MED13	chr17	60107208/A//G	synonymous	0	1
THOC1	chr18	214610/A//G	synonymous	0	1
LRRC8E	chr19	7963909/G//A	nonsynonymous	0	1
KRI1	chr19	10671697/T//C	synonymous	0	1
CARM1	chr19	11019855/T//C	nonsynonymous	0	1
CACNA1A	chr19	13616905/T//G	nonsynonymous	0	1
CC2D1A	chr19	14024029/A//G	nonsynonymous	0	1
HAUS5	chr19	36109600/A//T	nonsynonymous	0	1
PLD3	chr19	40883981/C//T	synonymous	0	1
CEACAM1	chr19	43032650/G//C	synonymous	0	1
PSG8	chr19	43262357/C//T	nonsynonymous	0	1
LHB	chr19	49519855/T//G	synonymous	0	1
STAU1	chr20	47734362/C//T	synonymous	0	1
BAGE4	chr21	11097536/C//A	synonymous	0	1
LPAR4	chrX	78010766/G//T	nonsynonymous	0	1
TCHH	chr1	152082986/GTTCC//G	nonsynonymous	0	1
APOB	chr2	21249777/T//A	synonymous	0	1
C2orf16	chr2	27805143/C//T	nonsynonymous	0	1
TTN-AS1	chr2	179470115/A//T	synonymous	0	1
SYN2	chr3	12187259/T//A	nonsynonymous	0	1
IMPG2	chr3	101010336/C//A	nonsynonymous	0	1
KALRN	chr3	124053180/C//T	synonymous	0	1
ANKRD31	chr5	74532547/C//G	synonymous	0	1
MUC22	chr6	30996697/T//C	synonymous	0	1
RSPO3	chr6	127469985/G//T	nonsynonymous	0	1
PAPOLB	chr7	4901208/G//A	synonymous	0	1
CDK13	chr7	40134059/G//T	nonsynonymous	0	1
ZNF479	chr7	57188030/T//G	nonsynonymous	0	1
SEMA3C	chr7	80374525/T//A	nonsynonymous	0	1
AKAP9	chr7	91707149/C//T	nonsynonymous	0	1
SSPO	chr7	149511855/G//A	synonymous	0	1
KMT2C	chr7	152007132/C//T	synonymous	0	1
SLC39A14	chr8	22273394/A//G	nonsynonymous	0	1
TM2D2	chr8	38851182/G//C	synonymous	0	1
SKIDA1	chr10	21804729/C//T	nonsynonymous	0	1
PACSIN3	chr11	47201022/A//G	nonsynonymous	0	1
DCUN1D5	chr11	102937214/A//T	nonsynonymous	0	1
WNK1	chr12	987460/C//A	nonsynonymous	0	1
ALDH2	chr12	112219776/G//A	nonsynonymous	0	1
EAPP	chr14	34985388/TA//T	synonymous	0	1
FRMD6	chr14	52194644/C//A	nonsynonymous	0	1
SV2B	chr15	91825001/T//G	nonsynonymous	0	1
SEPT1	chr16	30393464/C//T	nonsynonymous	0	1
SUPT6H	chr17	27003417/T//C	nonsynonymous	0	1
MILR1	chr17	62461659/G//C	nonsynonymous	0	1
TGFBR3L	chr19	7981159/G//A	nonsynonymous	0	1
PRAM1	chr19	8555772/T//A	nonsynonymous	0	1
ANO8	chr19	17441651/G//A	nonsynonymous	0	1
CNOT3	chr19	54647836/A//C	nonsynonymous	0	1
ZNF71	chr19	57133664/C//A	nonsynonymous	0	1
ZNF132	chr19	58946465/A//G	nonsynonymous	0	1
KAT14	chr20	18142571/C//T	nonsynonymous	0	1
SRSF6	chr20	42088466/A//T	synonymous	0	1
SOX18	chr20	62679525/G//A	synonymous	0	1
KRTAP10-9	chr21	46047295/A//G	synonymous	0	1
AIM1L	chr1	26671578/A//G	nonsynonymous	1	0
CSMD2	chr1	34006191/A//G	nonsynonymous	1	0

DENND2C	chr1	115166125/T//A	synonymous	1	0
PFDN2	chr1	161072096/T//G	nonsynonymous	1	0
SLC9C2	chr1	173517633/T//G	synonymous	1	0
RALGPS2	chr1	178861429/A//G	nonsynonymous	1	0
HMCN1	chr1	185988672/G//A	nonsynonymous	1	0
RAB3GAP2	chr1	220375699/C//T	nonsynonymous	1	0
ZEB2	chr2	145157522/C//A	nonsynonymous	1	0
LHFPL4	chr3	9543998/AAG//A	synonymous	1	0
LHFPL4	chr3	9594062/A//G	nonsynonymous	1	0
FANCD2OS	chr3	10146294/G//A	synonymous	1	0
CHDH	chr3	53853009/T//C	nonsynonymous	1	0
ROBO1	chr3	78706381/G//T	nonsynonymous	1	0
JAKMIP1	chr4	6083475/C//T	nonsynonymous	1	0
ENAM	chr4	71508491/C//T	nonsynonymous	1	0
MOG	chr6	29639192/T//C	synonymous	1	0
MDN1	chr6	90367948/C//T	nonsynonymous	1	0
PRPS1L1	chr7	18066440/G//A	synonymous	1	0
BMT2	chr7	112462378/C//G	nonsynonymous	1	0
OR2F2	chr7	143632955/T//C	synonymous	1	0
ST18	chr8	53084753/A//G	nonsynonymous	1	0
ZFHX4	chr8	77767054/A//G	nonsynonymous	1	0
ZNF572	chr8	125989602/T//G	nonsynonymous	1	0
RASEF	chr9	85615125/A//C	nonsynonymous	1	0
C9orf50	chr9	132375724/A//T	nonsynonymous	1	0
PRDM10	chr11	129782060/G//A	nonsynonymous	1	0
USP5	chr12	6968672/C//T	nonsynonymous	1	0
AVPR1A	chr12	63541267/C//G	nonsynonymous	1	0
BICDL1	chr12	120499580/A//G	nonsynonymous	1	0
SETDB2-PHF11	chr13	50092272/T//C	nonsynonymous	1	0
DOCK9	chr13	99538810/G//A	synonymous	1	0
ARHGEF40	chr14	21552086/G//A	synonymous	1	0
ACOT2	chr14	74036010/C//T	synonymous	1	0
XPO6	chr16	28222769/T//A	synonymous	1	0
RNF167	chr17	4844287/C//T	nonsynonymous	1	0
SGCA	chr17	48245825/C//A	nonsynonymous	1	0
TLK2	chr17	60613535/G//A	nonsynonymous	1	0
MYOM1	chr18	3189010/C//T	synonymous	1	0
ABCA7	chr19	1058047/T//C	nonsynonymous	1	0
PLIN5	chr19	4529803/G//A	nonsynonymous	1	0
CAMSAP3	chr19	7677865/C//T	nonsynonymous	1	0
ECSIT	chr19	11624808/A//G	nonsynonymous	1	0
NOTCH3	chr19	15302874/T//C	synonymous	1	0
PCED1A	chr20	2819713/C//G	nonsynonymous	1	0
KCNJ4	chr22	38823334/G//A	synonymous	1	0
MAGEB2	chrX	30237194/T//A	nonsynonymous	1	0
PLP1	chrX	103041508/C//T	synonymous	1	0
XPO1	chr2	61719589/T//C	nonsynonymous	0	1
GOLGA4	chr3	37402747/G//A	nonsynonymous	0	1
GPER1	chr7	1132429/C//T	synonymous	0	1
IGFBP1	chr7	45932783/GTA//G	synonymous	0	1
NFX1	chr9	33369947/A//G	nonsynonymous	0	1
CCDC34	chr11	27384459/C//T	nonsynonymous	0	1
DNAH10	chr12	124297910/G//A	nonsynonymous	0	1
LDHD	chr16	75148854/C//T	nonsynonymous	0	1
MYH4	chr17	10355577/C//T	nonsynonymous	0	1
RARA	chr17	38508583/A//G	nonsynonymous	0	1
FN3K	chr17	80696370/G//A	synonymous	0	1
ZNF714	chr19	21300262/T//C	synonymous	0	1
VSX1	chr20	25062450/C//T	nonsynonymous	0	1
POF1B	chrX	84561299/T//C	nonsynonymous	0	1
NXF2B	chrX	101623470/T//C	nonsynonymous	0	1
FLNA	chrX	153581656/C//T	synonymous	0	1
HDAC11	chr3	13525022/C//G	nonsynonymous	0	1
FIP1L1	chr4	54319247/CAG//C	nonsynonymous	0	1
GRK6	chr5	176863495/G//C	synonymous	0	1
NEU1	chr6	31827591/G//A	nonsynonymous	0	1
CDC40	chr6	110547399/C//T	nonsynonymous	0	1
ZNF680	chr7	63982167/A//T	nonsynonymous	0	1
NSUN5	chr7	72717849/C//T	synonymous	0	1
	chr9	71820073/A//G	synonymous	0	1

ENTPD1	chr10	97607225/G//T	nonsynonymous	0	1
NPAP1	chr15	24921934/C//A	nonsynonymous	0	1
GRB2	chr17	73316600/T//C	nonsynonymous	0	1
ATP8B1	chr18	55315606/T//C	synonymous	0	1
FAM199X	chrX	103411441/G//A	synonymous	0	1
PRAMEF11	chr1	12884937/C//G	nonsynonymous	0	1
IFI6	chr1	27994738/G//A	synonymous	0	1
UNC80	chr2	210745785/T//G	synonymous	0	1
VGLL3	chr3	87017802/G//T	nonsynonymous	0	1
HSD17B13	chr4	88244012/T//G	synonymous	0	1
MEPE	chr4	88766449/C//T	synonymous	0	1
DIAPH1	chr5	140896283/C//T	synonymous	0	1
PI16	chr6	36929682/G//A	nonsynonymous	0	1
PTPN12	chr7	77247853/T//C	synonymous	0	1
PTCD1	chr7	99017748/C//T	nonsynonymous	0	1
MUC12	chr7	100643255/C//G	nonsynonymous	0	1
RNF148	chr7	122342042/C//A	nonsynonymous	0	1
ATP6V1F	chr7	128502994/A//G	synonymous	0	1
GATA4	chr8	11566350/G//A	nonsynonymous	0	1
FGFR1	chr8	38287335/C//T	nonsynonymous	0	1
KDM4C	chr9	6849524/C//G	nonsynonymous	0	1
ERCC6L2	chr9	98643344/G//T	nonsynonymous	0	1
NOLC1	chr10	103919996/G//C	nonsynonymous	0	1
CAPN5	chr11	76826479/C//T	synonymous	0	1
TMEM225	chr11	123754797/A//C	nonsynonymous	0	1
OR8B8	chr11	124310561/A//G	nonsynonymous	0	1
HEBP1	chr12	13128297/T//C	nonsynonymous	0	1
DIP2B	chr12	51112580/A//G	synonymous	0	1
HIP1R	chr12	123339597/C//T	synonymous	0	1
	chr13	95098015/TCTGTTGAGACACAAA	synonymous	0	1
CHD8	chr14	21899176/T//C	synonymous	0	1
GEMIN2	chr14	39583584/G//T	nonsynonymous	0	1
BEGAIN	chr14	101004359/T//C	nonsynonymous	0	1
CCBE1	chr18	57105385/A//C	synonymous	0	1
NUCB1	chr19	49409068/A//G	nonsynonymous	0	1
GPCPD1	chr20	5554620/AAAG//A	synonymous	0	1
TXNRD2	chr22	19929310/A//G	nonsynonymous	0	1
DENND6B	chr22	50752254/G//A	nonsynonymous	0	1
SLC25A5	chrX	118605027/T//C	synonymous	0	1
MAGEC2	chrX	141290946/T//G	nonsynonymous	0	1
SLC10A3	chrX	153716374/A//G	synonymous	0	1
FLG2	chr1	152327700/T//C	synonymous	0	1
BSN	chr3	49700327/A//G	nonsynonymous	0	1
DNAH12	chr3	57431857/A//G	synonymous	0	1
	chr3	71247218/C//T	synonymous	0	1
SCLT1	chr4	129864157/T//G	nonsynonymous	0	1
WDR36	chr5	110446639/C//T	synonymous	0	1
APC	chr5	112174094/T//TA	nonsynonymous	0	1
ADAM32	chr8	39068746/T//C	nonsynonymous	0	1
ZNF79	chr9	130206895/G//A	nonsynonymous	0	1
KIF11	chr10	94405164/AAT//A	nonsynonymous	0	1
TCIRG1	chr11	67818055/T//C	nonsynonymous	0	1
MAGEL2	chr15	23891962/G//C	nonsynonymous	0	1
ACACA	chr17	35766436/G//A	synonymous	0	1
KAT7	chr17	47882742/C//T	nonsynonymous	0	1
CBX4	chr17	77812861/G//A	synonymous	0	1
TXNL4A	chr18	77748440/C//T	synonymous	0	1
IL12RB1	chr19	18191756/A//G	nonsynonymous	0	1
SLC7A4	chr22	21383687/A//G	nonsynonymous	0	1
ZNF674	chrX	46360667/A//G	synonymous	0	1
TFDP3	chrX	132351326/A//G	nonsynonymous	0	1
HRNR	chr1	152192365/G//A	synonymous	0	1
ATG7	chr3	11340183/C//T	nonsynonymous	0	1
CADM2	chr3	85932512/T//C	nonsynonymous	0	1
SLIT3	chr5	168727810/C//T	synonymous	0	1
CACNA2D1	chr7	81603852/G//A	synonymous	0	1
MUC17	chr7	100685758/G//T	nonsynonymous	0	1
KIAA1462	chr10	30316002/A//G	synonymous	0	1
ZW10	chr11	113608313/C//T	nonsynonymous	0	1
CADM1	chr11	115080317/G//A	nonsynonymous	0	1

ALG10B	chr12	38715051/A//G	synonymous	0	1
SETD1B	chr12	122242663/C//A	nonsynonymous	0	1
TNFRSF19	chr13	24233240/G//A	nonsynonymous	0	1
MAB21L1	chr13	36049789/G//A	nonsynonymous	0	1
MVP	chr16	29857256/A//G	synonymous	0	1
SPIRE1	chr18	12452291/A//G	nonsynonymous	0	1
SLC24A3	chr20	19566136/C//T	nonsynonymous	0	1
KCNB1	chr20	47990040/G//T	nonsynonymous	0	1
SSTR3	chr22	37603096/C//T	synonymous	0	1
CELSR1	chr22	46932140/C//T	nonsynonymous	0	1
DLG3	chrX	69670107/T//C	synonymous	0	1
PCDH11X	chrX	91133070/G//T	nonsynonymous	0	1
CHD5	chr1	6181203/G//A	nonsynonymous	0	1
HTR1D	chr1	23520502/T//C	nonsynonymous	0	1
RHCE	chr1	25735255/G//A	nonsynonymous	0	1
SLC9A1	chr1	27428546/G//A	synonymous	0	1
HDAC1	chr1	32797187/A//T	synonymous	0	1
FPGT-TNNI3K	chr1	74905180/C//A	nonsynonymous	0	1
ST6GALNAC5	chr1	77510110/C//T	synonymous	0	1
COL11A1	chr1	103488454/T//A	nonsynonymous	0	1
FCRL2	chr1	157739928/C//T	nonsynonymous	0	1
F5	chr1	169529840/C//T	nonsynonymous	0	1
C4BPA	chr1	207287635/G//A	synonymous	0	1
TRIM67	chr1	231299572/G//A	nonsynonymous	0	1
FAM98A	chr2	33817277/C//T	synonymous	0	1
	chr2	38523596/C//A	synonymous	0	1
ALMS1	chr2	73678185/C//G	nonsynonymous	0	1
EVA1A	chr2	75720328/C//A	synonymous	0	1
INPP4A	chr2	99156098/C//T	nonsynonymous	0	1
RANBP2	chr2	109399237/C//T	synonymous	0	1
CNTNAP5	chr2	125204473/C//T	nonsynonymous	0	1
NEB	chr2	152404243/G//A	nonsynonymous	0	1
TTN	chr2	179639731/A//G	nonsynonymous	0	1
ZNF804A	chr2	185801432/C//A	nonsynonymous	0	1
IDH1	chr2	209106742/C//T	nonsynonymous	0	1
PLXNB1	chr3	48462763/G//T	nonsynonymous	0	1
PHLDB2	chr3	111671473/A//C	nonsynonymous	0	1
PLXNA1	chr3	126752799/G//A	nonsynonymous	0	1
PRR23A	chr3	138724643/G//A	synonymous	0	1
TFRC	chr3	195785455/C//T	nonsynonymous	0	1
HTT	chr4	3174827/G//A	nonsynonymous	0	1
PRSS12	chr4	119239589/C//T	nonsynonymous	0	1
	chr4	155244315/A//G	synonymous	0	1
TENM3	chr4	183713468/C//T	synonymous	0	1
DCTD	chr4	183836213/T//C	nonsynonymous	0	1
GAPT	chr5	57790843/C//T	synonymous	0	1
THBS4	chr5	79355694/C//T	nonsynonymous	0	1
HAPLN1	chr5	82948404/G//T	nonsynonymous	0	1
PCDHB16	chr5	140564213/G//A	synonymous	0	1
DIAPH1	chr5	140896506/G//A	nonsynonymous	0	1
GABRG2	chr5	161530953/C//T	synonymous	0	1
GCM2	chr6	10874898/T//A	nonsynonymous	0	1
ZSCAN26	chr6	28239942/C//T	nonsynonymous	0	1
RIMS1	chr6	72892765/A//C	nonsynonymous	0	1
ROS1	chr6	117679098/T//C	synonymous	0	1
SYNE1	chr6	152749370/G//A	nonsynonymous	0	1
FRMD1	chr6	168462494/C//T	synonymous	0	1
DACT2	chr6	168709327/G//A	synonymous	0	1
MICALL2	chr7	1486367/TAAAGCCTCC//T	nonsynonymous	0	1
ABCB5	chr7	20725386/T//G	nonsynonymous	0	1
ELMO1	chr7	37298864/C//A	nonsynonymous	0	1
ZPBP	chr7	50070900/C//T	nonsynonymous	0	1
MUC17	chr7	100680286/T//G	nonsynonymous	0	1
CHRNB3	chr8	42585798/A//C	nonsynonymous	0	1
PRKDC	chr8	48794656/C//G	synonymous	0	1
PREX2	chr8	68981377/G//A	synonymous	0	1
CRISPLD1	chr8	75925185/C//A	nonsynonymous	0	1
FRMPD1	chr9	37745689/C//T	synonymous	0	1
ASPN	chr9	95219629/C//T	nonsynonymous	0	1
MVB12B	chr9	129243698/G//T	nonsynonymous	0	1

GRIN1	chr9	140052831/A//T	nonsynonymous	0	1
GRK5	chr10	121190924/C//T	nonsynonymous	0	1
MUC6	chr11	1027183/T//C	nonsynonymous	0	1
OR52E4	chr11	5906045/A//G	nonsynonymous	0	1
DCDC5	chr11	30921960/A//G	synonymous	0	1
CHST1	chr11	45671886/C//T	synonymous	0	1
FOLH1	chr11	49207382/G//T	nonsynonymous	0	1
SIPA1	chr11	65413711/T//C	nonsynonymous	0	1
ARAP1	chr11	72408985/G//A	nonsynonymous	0	1
FAT3	chr11	92086889/A//T	nonsynonymous	0	1
ATM	chr11	108181023/A//G	nonsynonymous	0	1
PATE2	chr11	125647313/A//G	synonymous	0	1
ITPR2	chr12	26809402/C//A	nonsynonymous	0	1
PDZRN4	chr12	41900422/G//A	synonymous	0	1
KRT5	chr12	52910594/C//T	synonymous	0	1
OR6C74	chr12	55641833/C//A	synonymous	0	1
OTOGL	chr12	80732968/G//A	nonsynonymous	0	1
NOS1	chr12	117723963/G//A	synonymous	0	1
MPHOSPH9	chr12	123687228/T//C	nonsynonymous	0	1
EP400	chr12	132514674/G//C	nonsynonymous	0	1
POSTN	chr13	38138685/C//A	nonsynonymous	0	1
COL4A1	chr13	110830520/C//T	synonymous	0	1
COL4A1	chr13	110959487/G//T	synonymous	0	1
TEP1	chr14	20839481/G//A	synonymous	0	1
MYH7	chr14	23899003/C//T	synonymous	0	1
ISM2	chr14	77941861/G//A	synonymous	0	1
GABRB3	chr15	27017587/C//T	nonsynonymous	0	1
MYO5A	chr15	52611515/G//A	nonsynonymous	0	1
SPSB3	chr16	1827200/G//T	nonsynonymous	0	1
CDH11	chr16	64984742/C//T	nonsynonymous	0	1
E2F4	chr16	67231782/C//A	nonsynonymous	0	1
CNTNAP4	chr16	76555045/T//G	nonsynonymous	0	1
C17orf105	chr17	41861215/G//A	nonsynonymous	0	1
ABCA9	chr17	67012417/T//C	nonsynonymous	0	1
RNF213	chr17	78272285/CCTT//C	nonsynonymous	0	1
DOT1L	chr19	2217079/G//A	nonsynonymous	0	1
OR1I1	chr19	15198492/G//A	nonsynonymous	0	1
ZNF257	chr19	22272145/C//T	synonymous	0	1
SYMPK	chr19	46332369/C//A	nonsynonymous	0	1
PRKD2	chr19	47177846/G//A	synonymous	0	1
ALDH16A1	chr19	49967449/C//G	nonsynonymous	0	1
RPS11	chr19	49999672/C//G	synonymous	0	1
ZNF28	chr19	53304131/C//A	nonsynonymous	0	1
SIGLEC1	chr20	3687191/C//T	nonsynonymous	0	1
GZF1	chr20	23346139/C//T	synonymous	0	1
KIAA1755	chr20	36869777/C//T	synonymous	0	1
LAMA5	chr20	60913540/G//A	synonymous	0	1
TIAM1	chr21	32497010/G//T	nonsynonymous	0	1
DACH2	chrX	85769390/G//A	synonymous	0	1
RNF113A	chrX	119004618/T//A	nonsynonymous	0	1
DCAF12L1	chrX	125685804/C//T	nonsynonymous	0	1
COLEC11	chr2	3691510/C//T	synonymous	0	1
ZDBF2	chr2	207171744/T//C	nonsynonymous	0	1
UGT2B11	chr4	70070357/T//C	synonymous	0	1
SLC6A3	chr5	1420692/C//A	nonsynonymous	0	1
CHRM2	chr7	136700632/T//C	synonymous	0	1
AQP7	chr9	33387004/T//C	synonymous	0	1
AQP7	chr9	33387047/T//G	nonsynonymous	0	1
OR8I2	chr11	55861510/C//A	nonsynonymous	0	1
DYNC2H1	chr11	103270434/G//A	nonsynonymous	0	1
OR10G2	chr14	22102374/T//C	nonsynonymous	0	1
OR10G2	chr14	22102439/C//G	nonsynonymous	0	1
HSP90AA1	chr14	102552665/A//G	synonymous	0	1
GNA15	chr19	3162985/G//A	nonsynonymous	0	1
C19orf44	chr19	16611599/A//G	synonymous	0	1
WDR87	chr19	38377031/C//A	nonsynonymous	0	1
NPEPL1	chr20	57287597/G//A	synonymous	0	1
KIAA1671	chr22	25424835/G//A	nonsynonymous	0	1
DEPDC5	chr22	32179919/G//A	nonsynonymous	0	1
GAB3	chrX	153906534/A//G	nonsynonymous	0	1

GPR88	chr1	101004688/G//A	nonsynonymous	0	1
KPRP	chr1	152732963/G//A	nonsynonymous	0	1
RGS5	chr1	163172659/A//G	synonymous	0	1
OR2T3	chr1	248636948/G//A	synonymous	0	1
IL17RE	chr3	9957496/A//G	synonymous	0	1
CCDC80	chr3	112328836/G//A	nonsynonymous	0	1
ALG1L2	chr3	129814998/C//G	nonsynonymous	0	1
FRG1	chr4	190883085/C//T	synonymous	0	1
TFAP2A	chr6	10398776/C//T	synonymous	0	1
POM121L2	chr6	27277166/G//C	synonymous	0	1
ZNF92	chr7	64864085/T//C	nonsynonymous	0	1
CPSF1	chr8	145620235/G//A	synonymous	0	1
ZNF517	chr8	146033687/C//T	synonymous	0	1
CNTFR	chr9	34552247/C//T	nonsynonymous	0	1
CCL21	chr9	34709543/C//G	nonsynonymous	0	1
IPPK	chr9	95410393/G//A	nonsynonymous	0	1
MUC5B	chr11	1267968/A//G	synonymous	0	1
MUC5B	chr11	1267971/C//A	synonymous	0	1
C12orf66	chr12	64588141/A//G	synonymous	0	1
METTL17	chr14	21462159/C//T	nonsynonymous	0	1
MDGA2	chr14	47315052/C//A	nonsynonymous	0	1
BRF1	chr14	105677604/G//A	synonymous	0	1
NLRP1	chr17	5436280/G//A	nonsynonymous	0	1
SAT2	chr17	7530288/C//G	nonsynonymous	0	1
NCOR1	chr17	16004573/T//C	nonsynonymous	0	1
TLCD1	chr17	27052976/T//C	nonsynonymous	0	1
ADAP2	chr17	29284982/C//T	synonymous	0	1
KAT2A	chr17	40266582/C//T	nonsynonymous	0	1
MC2R	chr18	13885045/C//T	nonsynonymous	0	1
POLRMT	chr19	622690/C//T	synonymous	0	1
PLIN4	chr19	4511108/G//A	nonsynonymous	0	1
NUDT19	chr19	33183295/C//T	synonymous	0	1
TSKS	chr19	50265373/G//A	nonsynonymous	0	1
KRTAP27-1	chr21	31709564/G//A	synonymous	0	1
NEFH	chr22	29879448/G//A	nonsynonymous	0	1
COL4A6	chrX	107431854/C//T	nonsynonymous	0	1
LRP8	chr1	53736893/T//C	synonymous	0	1
ABCB10	chr1	229654613/T//G	nonsynonymous	0	1
OR2L8	chr1	248112771/C//T	synonymous	0	1
PPM1G	chr2	27632276/A//G	nonsynonymous	0	1
SPTBN1	chr2	54876745/G//A	synonymous	0	1
ATG4B	chr2	242594053/C//T	nonsynonymous	0	1
ROBO2	chr3	77147399/G//A	nonsynonymous	0	1
	chr4	5894584/G//A	synonymous	0	1
SORCS2	chr4	7691261/G//A	nonsynonymous	0	1
GRSF1	chr4	71697397/A//G	synonymous	0	1
KIAA0922	chr4	154547368/G//A	nonsynonymous	0	1
LIX1	chr5	96478345/G//A	synonymous	0	1
IFT22	chr7	100959796/C//T	nonsynonymous	0	1
TNKS	chr8	9413559/C//T	nonsynonymous	0	1
STAB2	chr12	104118858/G//A	nonsynonymous	0	1
IMP3	chr15	75932199/G//A	nonsynonymous	0	1
ZNF823	chr19	11833116/A//T	synonymous	0	1
CD93	chr20	23065850/G//A	nonsynonymous	0	1
MYH9	chr22	36696955/T//G	nonsynonymous	0	1
HS6ST2	chrX	132092606/G//A	nonsynonymous	0	1
GBP4	chr1	89654416/T//A	nonsynonymous	0	1
ABCA4	chr1	94466437/T//A	nonsynonymous	0	1
WDR47	chr1	109553567/A//C	nonsynonymous	0	1
BOLA1	chr1	149871847/G//A	nonsynonymous	0	1
FLG2	chr1	152323864/T//A	nonsynonymous	0	1
TDRD10	chr1	154520087/T//C	synonymous	0	1
TPR	chr1	186313680/A//C	nonsynonymous	0	1
PTGS2	chr1	186648219/C//T	nonsynonymous	0	1
ZBTB41	chr1	197141337/A//AC	nonsynonymous	0	1
MAP3K21	chr1	233518453/C//G	nonsynonymous	0	1
OR2L8	chr1	248112899/T//C	nonsynonymous	0	1
NRXN1	chr2	50699589/C//G	nonsynonymous	0	1
WDPCP	chr2	63660892/T//A	nonsynonymous	0	1
AFTPH	chr2	64796313/G//A	synonymous	0	1

COX5B	chr2	98262589/G//T	nonsynonymous	0	1
	chr2	143746420/G//A	synonymous	0	1
PDK1	chr2	173431579/A//G	synonymous	0	1
ANKRD28	chr3	15711970/C//G	nonsynonymous	0	1
EOMES	chr3	27759208/C//T	nonsynonymous	0	1
SCN11A	chr3	38941557/G//C	nonsynonymous	0	1
CSPG5	chr3	47614265/G//A	synonymous	0	1
NCKIPSD	chr3	48716861/T//C	nonsynonymous	0	1
CACNA2D3	chr3	54537677/C//A	nonsynonymous	0	1
LRTM1	chr3	54959095/G//A	nonsynonymous	0	1
CADPS	chr3	62478032/C//T	synonymous	0	1
ADAMTS9	chr3	64592730/T//A	nonsynonymous	0	1
PHLDB2	chr3	111604216/G//A	nonsynonymous	0	1
WDR5B	chr3	122133657/C//T	nonsynonymous	0	1
HRASLS	chr3	192980753/T//G	synonymous	0	1
IDUA	chr4	998145/T//C	synonymous	0	1
BOD1L1	chr4	13616214/T//C	synonymous	0	1
FBXL5	chr4	15627073/C//G	nonsynonymous	0	1
WDR19	chr4	39278662/G//C	nonsynonymous	0	1
MOB1B	chr4	71844964/G//A	nonsynonymous	0	1
CXCL6	chr4	74702567/C//G	nonsynonymous	0	1
ANK2	chr4	114275381/G//A	synonymous	0	1
FAT4	chr4	126238020/C//T	nonsynonymous	0	1
FAT4	chr4	126241469/A//C	nonsynonymous	0	1
FHDC1	chr4	153895897/G//A	nonsynonymous	0	1
DDX60	chr4	169173813/C//CAT	synonymous	0	1
ADAM29	chr4	175897313/C//T	nonsynonymous	0	1
FAT1	chr4	187630570/G//A	nonsynonymous	0	1
CDH10	chr5	24488147/C//A	nonsynonymous	0	1
CDH9	chr5	26906907/G//A	synonymous	0	1
ADAMTS12	chr5	33596157/G//A	nonsynonymous	0	1
SPEF2	chr5	35806922/A//C	nonsynonymous	0	1
LIFR	chr5	38496574/C//T	nonsynonymous	0	1
GHR	chr5	42719120/G//A	nonsynonymous	0	1
ADAMTS6	chr5	64587290/G//A	nonsynonymous	0	1
DMGDH	chr5	78347156/T//A	nonsynonymous	0	1
DMGDH	chr5	78347158/C//A	nonsynonymous	0	1
ADGRV1	chr5	90059174/T//G	nonsynonymous	0	1
SLF1	chr5	94014552/C//G	nonsynonymous	0	1
SLCO4C1	chr5	101627086/A//AC	nonsynonymous	0	1
CDO1	chr5	115152120/G//A	synonymous	0	1
PCDHA9	chr5	140229441/C//A	nonsynonymous	0	1
PCDHGA12	chr5	140812292/G//A	nonsynonymous	0	1
FCHSD1	chr5	141025684/C//G	nonsynonymous	0	1
SPARC	chr5	151046042/C//T	nonsynonymous	0	1
DPCR1	chr6	30917791/C//A	nonsynonymous	0	1
ATF6B	chr6	32086743/A//G	nonsynonymous	0	1
MTO1	chr6	74189500/G//A	nonsynonymous	0	1
SENP6	chr6	76372942/G//T	nonsynonymous	0	1
HTR1E	chr6	87725443/G//A	nonsynonymous	0	1
SLC16A10	chr6	111498456/C//A	nonsynonymous	0	1
TSPYL1	chr6	116600332/C//A	nonsynonymous	0	1
KIAA0408	chr6	127767633/C//A	nonsynonymous	0	1
C6orf58	chr6	127911337/T//C	synonymous	0	1
AKAP7	chr6	131481312/C//G	nonsynonymous	0	1
EYA4	chr6	133844275/T//C	synonymous	0	1
LTV1	chr6	144184395/G//C	nonsynonymous	0	1
LPA	chr6	160953611/A//G	synonymous	0	1
C6orf118	chr6	165695136/T//G	nonsynonymous	0	1
C7orf50	chr7	1049661/C//T	nonsynonymous	0	1
TMEM184A	chr7	1586598/T//C	nonsynonymous	0	1
DGKB	chr7	14647119/A//C	nonsynonymous	0	1
MYO1G	chr7	45005870/C//T	nonsynonymous	0	1
FGL2	chr7	76826210/T//A	nonsynonymous	0	1
KIAA1324L	chr7	86688654/G//A	nonsynonymous	0	1
CYP3A43	chr7	99434170/GT//G	nonsynonymous	0	1
ZAN	chr7	100377093/C//T	nonsynonymous	0	1
CUX1	chr7	101844929/C//G	synonymous	0	1
LRRN3	chr7	110763472/G//A	nonsynonymous	0	1
MET	chr7	116340302/T//G	nonsynonymous	0	1

CADPS2	chr7	122526477/G//A	synonymous	0	1
CSMD1	chr8	2976038/C//T	nonsynonymous	0	1
PREX2	chr8	68972995/A//C	nonsynonymous	0	1
RIMS2	chr8	104709364/G//C	nonsynonymous	0	1
DEPTOR	chr8	120940662/G//T	nonsynonymous	0	1
TMEM215	chr9	32784266/G//A	nonsynonymous	0	1
NTRK2	chr9	87570408/C//T	synonymous	0	1
SHC3	chr9	91692780/G//C	nonsynonymous	0	1
CTNNAL1	chr9	111714531/C//T	nonsynonymous	0	1
ZNF79	chr9	130207317/G//A	synonymous	0	1
EHMT1	chr9	140712546/G//A	nonsynonymous	0	1
ADARB2	chr10	1405392/G//A	nonsynonymous	0	1
ITIH2	chr10	7776906/C//T	synonymous	0	1
CELF2	chr10	11207591/G//A	nonsynonymous	0	1
HTR7	chr10	92509094/G//A	nonsynonymous	0	1
PDE6C	chr10	95405734/C//A	nonsynonymous	0	1
CYP2C9	chr10	96708903/G//T	synonymous	0	1
SORCS1	chr10	108337210/G//A	nonsynonymous	0	1
TCF7L2	chr10	114910838/C//CAA	nonsynonymous	0	1
KMT5B	chr11	67934463/T//C	nonsynonymous	0	1
TPBGL	chr11	74953147/C//T	synonymous	0	1
GRM5	chr11	88242270/G//A	synonymous	0	1
FAT3	chr11	92087079/T//G	nonsynonymous	0	1
MTMR2	chr11	95621326/A//T	nonsynonymous	0	1
C11orf70	chr11	101937390/C//G	synonymous	0	1
KBTBD3	chr11	105929645/A//C	nonsynonymous	0	1
C11orf65	chr11	108253742/A//G	synonymous	0	1
TECTA	chr11	120998995/G//A	nonsynonymous	0	1
KCNJ1	chr11	128709878/G//C	nonsynonymous	0	1
KIF21A	chr12	39703391/A//G	nonsynonymous	0	1
PTPRQ	chr12	80935988/A//G	nonsynonymous	0	1
PAH	chr12	103237547/G//T	nonsynonymous	0	1
GPN3	chr12	110893657/C//T	nonsynonymous	0	1
HVCN1	chr12	111087191/T//G	synonymous	0	1
MYL2	chr12	111352042/C//T	synonymous	0	1
ACAD10	chr12	112150380/C//T	nonsynonymous	0	1
NOS1	chr12	117725946/G//A	nonsynonymous	0	1
	chr13	20012304/A//C	synonymous	0	1
ATP8A2	chr13	26535756/T//G	nonsynonymous	0	1
TM9SF2	chr13	100211615/A//G	nonsynonymous	0	1
OR4K1	chr14	20403822/A//G	synonymous	0	1
LRFN5	chr14	42356008/C//T	synonymous	0	1
SIX4	chr14	61180671/C//T	synonymous	0	1
PLEKHD1	chr14	69992748/C//A	nonsynonymous	0	1
KNL1	chr15	40914519/G//C	nonsynonymous	0	1
SEMA6D	chr15	48053199/A//C	nonsynonymous	0	1
TNFAIP8L3	chr15	51350195/G//T	nonsynonymous	0	1
CCDC33	chr15	74573129/G//A	nonsynonymous	0	1
CPEB1	chr15	83226687/G//A	synonymous	0	1
AXIN1	chr16	396863/C//A	nonsynonymous	0	1
COG7	chr16	23464232/C//T	synonymous	0	1
FBXL19	chr16	30937119/G//A	nonsynonymous	0	1
SETD1A	chr16	30977632/C//G	synonymous	0	1
CHST6	chr16	75513472/G//A	synonymous	0	1
FBXO31	chr16	87368936/G//A	nonsynonymous	0	1
ZBTB4	chr17	7369429/C//A	nonsynonymous	0	1
NTN1	chr17	9066156/C//T	nonsynonymous	0	1
CFAP52	chr17	9515710/C//G	nonsynonymous	0	1
RHBDL3	chr17	30648226/T//C	nonsynonymous	0	1
RHBDL3	chr17	30648232/T//C	nonsynonymous	0	1
LYZL6	chr17	34261790/G//A	synonymous	0	1
CD79B	chr17	62006571/G//A	synonymous	0	1
ABCA8	chr17	66933232/T//A	nonsynonymous	0	1
ABCA10	chr17	67190080/C//A	nonsynonymous	0	1
DNAH17	chr17	76497914/G//C	nonsynonymous	0	1
PTPRS	chr19	5239059/C//G	nonsynonymous	0	1
FBN3	chr19	8148241/C//T	nonsynonymous	0	1
MUC16	chr19	9073404/G//A	nonsynonymous	0	1
MUC16	chr19	9085788/T//C	synonymous	0	1
ZNF699	chr19	9408561/C//T	nonsynonymous	0	1

SAMD1	chr19	14199383/A//C	nonsynonymous	0	1
MYO9B	chr19	17267758/C//T	synonymous	0	1
ZNF536	chr19	31039147/C//T	nonsynonymous	0	1
UBA2	chr19	34941218/C//T	nonsynonymous	0	1
ARHGAP33	chr19	36269458/C//G	nonsynonymous	0	1
MAP4K1	chr19	39098655/G//A	nonsynonymous	0	1
EXOSC5	chr19	41895666/C//A	synonymous	0	1
CEACAM16	chr19	45206938/G//A	synonymous	0	1
ZNF175	chr19	52084988/CTCTAAAACTGAAGAA	synonymous	0	1
ZNF432	chr19	52544824/G//T	nonsynonymous	0	1
FAM90A27P	chr19	53786091/C//A	synonymous	0	1
RPL28	chr19	55903017/C//T	synonymous	0	1
SIGLEC1	chr20	3674930/T//C	nonsynonymous	0	1
RALGAPA2	chr20	20475824/A//T	synonymous	0	1
NINL	chr20	25457692/C//G	synonymous	0	1
MROH8	chr20	35807732/C//G	nonsynonymous	0	1
FITM2	chr20	42935651/G//A	nonsynonymous	0	1
ADARB1	chr21	46624592/G//A	nonsynonymous	0	1
CRYBB2	chr22	25627698/A//T	nonsynonymous	0	1
MYO18B	chr22	26164216/G//T	synonymous	0	1
APOL3	chr22	36537962/C//T	synonymous	0	1
PLXNB2	chr22	50716405/C//A	nonsynonymous	0	1
LANCL3	chrX	37534963/G//GTA	nonsynonymous	0	1
ALAS2	chrX	55050278/T//C	synonymous	0	1
	chrX	74670592/G//T	synonymous	0	1
TAS1R1	chr1	6636512/A//T	nonsynonymous	0	1
CASP9	chr1	15850603/G//A	synonymous	0	1
TCTEX1D4	chr1	45271809/G//A	nonsynonymous	0	1
AKNAD1	chr1	109359721/A//G	synonymous	0	1
RBM15	chr1	110882783/G//A	synonymous	0	1
FLG2	chr1	152327637/C//G	nonsynonymous	0	1
PAQR6	chr1	156215604/G//C	synonymous	0	1
DCAF6	chr1	168007647/G//C	nonsynonymous	0	1
USH2A	chr1	216173882/G//T	nonsynonymous	0	1
TGFB2	chr1	218614702/T//G	nonsynonymous	0	1
MARK1	chr1	220835511/A//C	synonymous	0	1
MRPL30	chr2	99804690/C//G	nonsynonymous	0	1
SH3RF3	chr2	110107065/A//G	nonsynonymous	0	1
CHRNA1	chr2	175624294/G//A	synonymous	0	1
CHST13	chr3	126261031/G//A	synonymous	0	1
MED12L	chr3	151083757/A//C	nonsynonymous	0	1
SST	chr3	187386931/G//C	nonsynonymous	0	1
EPHA5	chr4	66509078/T//C	synonymous	0	1
TMPRSS11BNL	chr4	69057023/T//C	synonymous	0	1
GC	chr4	72618269/G//T	nonsynonymous	0	1
PTPN13	chr4	87656895/T//C	nonsynonymous	0	1
COL25A1	chr4	109861803/C//T	nonsynonymous	0	1
KIAA1109	chr4	123171562/T//G	nonsynonymous	0	1
CYP4V2	chr4	187131762/C//T	synonymous	0	1
RGMB	chr5	98129068/C//T	synonymous	0	1
ADRA1B	chr5	159344847/T//C	nonsynonymous	0	1
POU5F1	chr6	31133754/G//T	nonsynonymous	0	1
AGER	chr6	32152006/T//C	synonymous	0	1
MCM3	chr6	52133910/A//C	nonsynonymous	0	1
UTRN	chr6	144869799/G//A	nonsynonymous	0	1
INHBA	chr7	41739915/T//C	nonsynonymous	0	1
TMEM178B	chr7	141170561/G//C	nonsynonymous	0	1
CSMD1	chr8	2910040/G//T	nonsynonymous	0	1
C8orf34	chr8	69380957/A//T	nonsynonymous	0	1
VPS13A	chr9	79972756/T//A	nonsynonymous	0	1
LRRC26	chr9	140063806/T//C	nonsynonymous	0	1
PFKP	chr10	3172133/C//T	synonymous	0	1
MYO3A	chr10	26491909/G//C	nonsynonymous	0	1
RGR	chr10	86018290/G//A	synonymous	0	1
OR10A6	chr11	7949754/A//C	synonymous	0	1
OR5B12	chr11	58207053/T//C	nonsynonymous	0	1
INTS5	chr11	62414893/C//A	nonsynonymous	0	1
C2CD3	chr11	73834038/C//T	nonsynonymous	0	1
DLG2	chr11	83674001/A//C	nonsynonymous	0	1
TRIM49	chr11	89531758/A//G	nonsynonymous	0	1

ROBO3	chr11	124740986/C//T	synonymous	0	1
C12orf40	chr12	40076501/T//A	nonsynonymous	0	1
FGD6	chr12	95478416/C//T	nonsynonymous	0	1
ANKS1B	chr12	99640070/T//C	nonsynonymous	0	1
CUX2	chr12	111758426/C//T	synonymous	0	1
WASF3	chr13	27256798/G//A	synonymous	0	1
PCDH8	chr13	53421233/A//G	nonsynonymous	0	1
CGRRF1	chr14	54976752/C//T	synonymous	0	1
CHURC1-FNTB	chr14	65381097/C//G	synonymous	0	1
UNC13C	chr15	54556597/A//C	synonymous	0	1
FBXL8	chr16	67197549/C//T	synonymous	0	1
CCL7	chr17	32598239/T//C	nonsynonymous	0	1
SAMD14	chr17	48193376/A//C	nonsynonymous	0	1
BCAS3	chr17	59469352/G//T	nonsynonymous	0	1
ANGPTL6	chr19	10204524/C//A	nonsynonymous	0	1
ZNF799	chr19	12501533/T//A	nonsynonymous	0	1
ZNF208	chr19	22157509/C//A	nonsynonymous	0	1
ZNF675	chr19	23836892/C//T	synonymous	0	1
ZNF254	chr19	24309169/A//C	synonymous	0	1
PRX	chr19	40902575/C//T	nonsynonymous	0	1
ZNF613	chr19	52448441/A//G	synonymous	0	1
KIZ	chr20	21112769/C//G	nonsynonymous	0	1
KCNQ2	chr20	62065222/C//T	nonsynonymous	0	1
APP	chr21	27284171/C//G	nonsynonymous	0	1
SLC19A1	chr21	46951781/G//A	synonymous	0	1
RFPL3	chr22	32754177/GT//G	nonsynonymous	0	1
TOM1	chr22	35734732/C//G	nonsynonymous	0	1
DMD	chrX	32404421/A//T	synonymous	0	1
SSX1	chrX	48117273/C//G	nonsynonymous	0	1
CACNA1F	chrX	49083115/C//A	nonsynonymous	0	1
MTRNR2L10	chrX	55208369/G//A	nonsynonymous	0	1
ATRX	chrX	76890106/C//A	nonsynonymous	0	1
CYSLTR1	chrX	77529090/A//C	nonsynonymous	0	1
	chrX	107018697/G//T	synonymous	0	1
AMELY	chrY	6736361/C//T	nonsynonymous	0	1
PER3	chr1	7886767/C//T	nonsynonymous	0	1
CSF3R	chr1	36938246/G//T	nonsynonymous	0	1
RLF	chr1	40627064/G//T	synonymous	0	1
CDKN2C	chr1	51439845/G//T	nonsynonymous	0	1
TTC39A	chr1	51771756/G//T	synonymous	0	1
IL23R	chr1	67666529/G//C	nonsynonymous	0	1
MCOLN3	chr1	85484819/C//A	nonsynonymous	0	1
SLC16A4	chr1	110919695/G//A	synonymous	0	1
LRIF1	chr1	111494810/T//C	synonymous	0	1
SYCP1	chr1	115520175/T//G	synonymous	0	1
	chr1	170994516/T//G	synonymous	0	1
SMG7	chr1	183511378/C//T	nonsynonymous	0	1
SRBD1	chr2	45773916/T//A	nonsynonymous	0	1
EML6	chr2	55040366/CAGCCTT//C	nonsynonymous	0	1
CCDC85A	chr2	56603050/C//A	nonsynonymous	0	1
DNAH6	chr2	84811319/G//C	nonsynonymous	0	1
RNF103	chr2	86831702/T//G	nonsynonymous	0	1
SMPD4	chr2	130914236/C//A	synonymous	0	1
SCN1A	chr2	166908496/G//T	nonsynonymous	0	1
XIRP2	chr2	168105810/A//G	synonymous	0	1
FRZB	chr2	183699520/C//A	synonymous	0	1
TNS1	chr2	218669221/G//A	synonymous	0	1
SPEG	chr2	220313389/C//T	synonymous	0	1
NGEF	chr2	233839551/G//T	nonsynonymous	0	1
TGFBR2	chr3	30713791/G//C	nonsynonymous	0	1
TGM4	chr3	44948474/C//T	nonsynonymous	0	1
GPR27	chr3	71803637/C//A	nonsynonymous	0	1
PDZRN3	chr3	73433708/G//T	nonsynonymous	0	1
ZNF717	chr3	75787218/T//C	nonsynonymous	0	1
HTR1F	chr3	88039863/C//A	synonymous	0	1
LRRC58	chr3	120067775/G//C	nonsynonymous	0	1
TSC22D2	chr3	150127116/C//G	synonymous	0	1
KLHL6	chr3	183225967/T//C	synonymous	0	1
PTPN13	chr4	87593630/TC//T	nonsynonymous	0	1
PPP3CA	chr4	102267975/G//C	synonymous	0	1

NAA15	chr4	140265403/C//T	nonsynonymous	0	1
ADAMTS16	chr5	5186209/G//T	nonsynonymous	0	1
UGT3A2	chr5	36052057/C//A	nonsynonymous	0	1
PARP8	chr5	50129857/C//G	nonsynonymous	0	1
PIK3R1	chr5	67589574/GGAAAAAATTACATGA	nonsynonymous	0	1
	chr6	35705074/G//A	synonymous	0	1
DNAH8	chr6	38858464/G//A	nonsynonymous	0	1
HSP90AB1	chr6	44218342/A//G	synonymous	0	1
DDX43	chr6	74111602/G//C	nonsynonymous	0	1
MANEA	chr6	96053987/C//T	synonymous	0	1
SCML4	chr6	108068024/G//A	nonsynonymous	0	1
LACE1	chr6	108616325/A//C	nonsynonymous	0	1
GPR6	chr6	110300310/G//C	synonymous	0	1
COL10A1	chr6	116442378/G//A	nonsynonymous	0	1
TCP10	chr6	167791530/G//C	nonsynonymous	0	1
SDK1	chr7	4260992/C//G	synonymous	0	1
DAGLB	chr7	6461424/C//T	synonymous	0	1
C7orf57	chr7	48086215/T//C	nonsynonymous	0	1
WBSCR17	chr7	71036376/C//A	nonsynonymous	0	1
RELN	chr7	103155697/G//T	nonsynonymous	0	1
FSCN3	chr7	127235689/G//T	nonsynonymous	0	1
TTC26	chr7	138818562/G//C	synonymous	0	1
CUL1	chr7	148457582/G//C	nonsynonymous	0	1
DNAJB6	chr7	157202641/G//A	nonsynonymous	0	1
CHD7	chr8	61754266/A//G	nonsynonymous	0	1
CHD7	chr8	61761133/C//T	nonsynonymous	0	1
SCRIB	chr8	144895008/G//A	nonsynonymous	0	1
GPR158	chr10	25886807/G//T	nonsynonymous	0	1
MAP3K8	chr10	30736861/A//G	nonsynonymous	0	1
PRKG1	chr10	53564414/T//A	nonsynonymous	0	1
PCDH15	chr10	56287643/G//C	synonymous	0	1
KCNMA1	chr10	78729766/G//A	nonsynonymous	0	1
MINPP1	chr10	89264575/G//T	synonymous	0	1
ZDHHC16	chr10	99213550/C//A	synonymous	0	1
AFAP1L2	chr10	116091655/C//G	synonymous	0	1
ATE1	chr10	123661984/C//A	nonsynonymous	0	1
AMPD3	chr11	10521628/CCCAGGTGACGGGGT	nonsynonymous	0	1
FLRT1	chr11	63883796/G//A	synonymous	0	1
SYVN1	chr11	64897536/C//A	nonsynonymous	0	1
DLG2	chr11	83173087/G//A	nonsynonymous	0	1
GPR83	chr11	94113814/G//A	nonsynonymous	0	1
CLMP	chr11	122945531/C//G	nonsynonymous	0	1
NCAPD3	chr11	134022796/C//T	synonymous	0	1
LRTM2	chr12	1943993/C//A	synonymous	0	1
GALNT8	chr12	4870122/A//T	nonsynonymous	0	1
TAS2R20	chr12	11149643/T//G	nonsynonymous	0	1
PYM1	chr12	56321588/C//A	synonymous	0	1
PPM1H	chr12	63328479/A//G	nonsynonymous	0	1
BBS10	chr12	76740364/C//T	synonymous	0	1
ACSS3	chr12	81472027/G//C	nonsynonymous	0	1
TMTC2	chr12	83290079/C//G	nonsynonymous	0	1
LRRIQ1	chr12	85531631/G//T	nonsynonymous	0	1
UTP20	chr12	101748719/G//T	synonymous	0	1
SDSL	chr12	113866247/A//G	nonsynonymous	0	1
SBNO1	chr12	123829949/C//T	nonsynonymous	0	1
DNAH10	chr12	124325871/G//C	nonsynonymous	0	1
KLHL1	chr13	70413144/T//G	nonsynonymous	0	1
GPC5	chr13	92345496/C//T	synonymous	0	1
GPR180	chr13	95264524/A//T	nonsynonymous	0	1
CCDC168	chr13	103395430/C//T	synonymous	0	1
IRS2	chr13	110435765/C//T	nonsynonymous	0	1
	chr14	35515656/C//A	synonymous	0	1
CLEC14A	chr14	38724597/C//T	nonsynonymous	0	1
KIAA0586	chr14	58938969/A//G	nonsynonymous	0	1
KLC1	chr14	104167582/G//C	synonymous	0	1
MTA1	chr14	105920636/TGTTAAAAGAAGGTA	nonsynonymous	0	1
ANPEP	chr15	90347789/G//T	nonsynonymous	0	1
GNPTG	chr16	1412233/C//A	nonsynonymous	0	1
CLCN7	chr16	1510851/G//A	synonymous	0	1
SPN	chr16	29675937/T//A	synonymous	0	1

HYDIN	chr16	71096235/A//T	nonsynonymous	0	1
TP53	chr17	7578405/GC//G	nonsynonymous	0	1
NTN1	chr17	8925798/G//A	synonymous	0	1
KIAA0100	chr17	26964079/C//T	synonymous	0	1
AARSD1	chr17	41103846/T//A	synonymous	0	1
PPY	chr17	42018835/G//T	nonsynonymous	0	1
DCAKD	chr17	43112154/T//C	nonsynonymous	0	1
COL1A1	chr17	48268813/G//C	synonymous	0	1
FOXK2	chr17	80543822/G//A	nonsynonymous	0	1
CABYR	chr18	21736331/C//A	nonsynonymous	0	1
PSTPIP2	chr18	43604637/C//A	nonsynonymous	0	1
SALL3	chr18	76754709/G//T	synonymous	0	1
VAV1	chr19	6854014/G//T	nonsynonymous	0	1
MUC16	chr19	9062872/T//C	nonsynonymous	0	1
SMARCA4	chr19	11123738/C//T	synonymous	0	1
HOOK2	chr19	12880903/G//C	synonymous	0	1
	chr19	12936804/T//A	synonymous	0	1
ADGRL1	chr19	14272368/C//T	synonymous	0	1
ILVBL	chr19	15226677/G//A	nonsynonymous	0	1
UPF1	chr19	18942937/G//T	synonymous	0	1
ZNF676	chr19	22375863/C//A	nonsynonymous	0	1
ZNF98	chr19	22574914/C//G	nonsynonymous	0	1
WDR62	chr19	36592920/C//T	synonymous	0	1
RYR1	chr19	38957043/G//C	synonymous	0	1
SUPT5H	chr19	39948308/G//T	synonymous	0	1
IRF2BP1	chr19	46387930/C//G	nonsynonymous	0	1
ZNF649	chr19	52394929/C//T	nonsynonymous	0	1
ZNF677	chr19	53741539/C//G	nonsynonymous	0	1
EPN1	chr19	56203244/G//T	nonsynonymous	0	1
AURKC	chr19	57744875/A//G	synonymous	0	1
FOXA2	chr20	22562499/T//A	nonsynonymous	0	1
BPIFB4	chr20	31671458/T//A	nonsynonymous	0	1
ARFGEF2	chr20	47628474/G//A	synonymous	0	1
SYCP2	chr20	58476774/G//A	synonymous	0	1
COL20A1	chr20	61950467/A//T	synonymous	0	1
APP	chr21	27394181/TGTG//T	nonsynonymous	0	1
TIAM1	chr21	32638475/T//C	nonsynonymous	0	1
KRTAP10-1	chr21	45959386/G//A	synonymous	0	1
GAB4	chr22	17488859/G//C	nonsynonymous	0	1
ASCC2	chr22	30198104/C//G	nonsynonymous	0	1
MTMR3	chr22	30416422/G//C	nonsynonymous	0	1
SEC14L3	chr22	30855999/G//T	synonymous	0	1
P2RY8	chrX	1584955/GGGTAGGTGAGATC//G	nonsynonymous	0	1
CFAP47	chrX	36091459/T//C	nonsynonymous	0	1
FUNDC1	chrX	44397766/T//C	nonsynonymous	0	1
STARD8	chrX	67938362/A//G	nonsynonymous	0	1
ZCCHC5	chrX	77913799/A//T	nonsynonymous	0	1
KLHL4	chrX	86890720/G//T	nonsynonymous	0	1
HS6ST2	chrX	132092392/C//G	nonsynonymous	0	1
KAZN	chr1	15429876/C//G	nonsynonymous	1	0
ASAP3	chr1	23782493/G//A	nonsynonymous	1	0
ATPIF1	chr1	28564419/A//G	nonsynonymous	1	0
KPNA6	chr1	32623912/G//T	nonsynonymous	1	0
USP1	chr1	62916297/T//C	nonsynonymous	1	0
WLS	chr1	68619231/T//C	nonsynonymous	1	0
CLCA2	chr1	86921100/A//ACAGCAAT	nonsynonymous	1	0
KCND3	chr1	112524680/C//T	synonymous	1	0
RHBG	chr1	156354353/C//A	synonymous	1	0
SPTA1	chr1	158626363/G//A	synonymous	1	0
ACKR1	chr1	159175253/G//A	synonymous	1	0
NUF2	chr1	163307804/T//C	synonymous	1	0
XCL2	chr1	168510224/T//G	nonsynonymous	1	0
SELE	chr1	169702130/A//C	nonsynonymous	1	0
TNR	chr1	175372637/C//T	synonymous	1	0
EDEM3	chr1	184718682/A//G	nonsynonymous	1	0
KIF21B	chr1	200968470/CA//C	synonymous	1	0
COLEC11	chr2	3652045/G//C	nonsynonymous	1	0
KLF11	chr2	10186482/C//A	nonsynonymous	1	0
GREB1	chr2	11696830/C//G	nonsynonymous	1	0
TRIB2	chr2	12880828/G//A	nonsynonymous	1	0

BCL11A	chr2	60688221/G//A	nonsynonymous	1	0
DYSF	chr2	71883367/C//T	nonsynonymous	1	0
MAP4K4	chr2	102481432/G//A	nonsynonymous	1	0
AMER3	chr2	131520234/C//A	synonymous	1	0
TTN	chr2	179593297/G//A	synonymous	1	0
TTN	chr2	179635145/C//A	nonsynonymous	1	0
C2orf88	chr2	191064757/G//A	synonymous	1	0
CUL3	chr2	225370767/G//A	nonsynonymous	1	0
ITPR1	chr3	4808288/G//T	nonsynonymous	1	0
LTF	chr3	46491995/C//T	nonsynonymous	1	0
PTPN23	chr3	47451029/G//GCGGC	nonsynonymous	1	0
PFKFB4	chr3	48563095/C//A	nonsynonymous	1	0
TBC1D23	chr3	99998573/T//C	nonsynonymous	1	0
IMPG2	chr3	100948239/A//C	nonsynonymous	1	0
IMPG2	chr3	100961632/G//A	synonymous	1	0
SEMA5B	chr3	122631758/C//T	nonsynonymous	1	0
ECE2	chr3	183994760/G//A	synonymous	1	0
ADAMTS16	chr5	5182228/C//T	synonymous	1	0
CTNND2	chr5	11397248/C//T	synonymous	1	0
LIFR	chr5	38523515/A//G	synonymous	1	0
PAIP1	chr5	43535629/T//C	synonymous	1	0
VCAN	chr5	82817214/C//A	nonsynonymous	1	0
EPB41L4A	chr5	111519775/C//A	nonsynonymous	1	0
FBN2	chr5	127625637/C//A	nonsynonymous	1	0
FBN2	chr5	127700375/C//G	synonymous	1	0
FSTL4	chr5	132648346/G//A	nonsynonymous	1	0
PSD2	chr5	139221974/G//A	nonsynonymous	1	0
PCDHB10	chr5	140572037/CA//C	synonymous	1	0
HIST1H2AH	chr6	27114865/G//A	synonymous	1	0
ABCF1	chr6	30550289/G//C	synonymous	1	0
COL11A2	chr6	33147254/C//T	nonsynonymous	1	0
GRM4	chr6	34100974/C//T	synonymous	1	0
NUP43	chr6	150067093/C//G	nonsynonymous	1	0
PARK2	chr6	163148804/C//T	synonymous	1	0
AMZ1	chr7	2748254/C//A	nonsynonymous	1	0
CCDC129	chr7	31617854/C//A	nonsynonymous	1	0
RFC2	chr7	73668649/G//A	nonsynonymous	1	0
POMZP3	chr7	76240779/C//T	synonymous	1	0
SEMA3A	chr7	83606502/G//A	nonsynonymous	1	0
LRRD1	chr7	91774285/G//A	nonsynonymous	1	0
ZAN	chr7	100336106/C//T	synonymous	1	0
LRRC61	chr7	150034397/A//C	synonymous	1	0
RB1CC1	chr8	53586528/C//T	synonymous	1	0
PENK	chr8	57354267/T//A	nonsynonymous	1	0
CYP7B1	chr8	65509153/G//A	synonymous	1	0
SLC7A13	chr8	87229781/G//A	nonsynonymous	1	0
SLC7A13	chr8	87242055/C//T	nonsynonymous	1	0
FBXO43	chr8	101152927/C//T	nonsynonymous	1	0
ATP6V1C1	chr8	104065051/G//A	nonsynonymous	1	0
NOV	chr8	120430340/G//A	nonsynonymous	1	0
TRPM6	chr9	77354770/C//T	nonsynonymous	1	0
ACTL7A	chr9	111625032/G//A	nonsynonymous	1	0
WDFY4	chr10	49939411/G//A	synonymous	1	0
WDFY4	chr10	50025361/C//T	synonymous	1	0
ADAMTS14	chr10	72434378/G//A	nonsynonymous	1	0
MUC6	chr11	1023653/C//T	nonsynonymous	1	0
MUC5B	chr11	1248622/G//C	nonsynonymous	1	0
MUC5B	chr11	1276480/G//C	nonsynonymous	1	0
PRKCDBP	chr11	6340514/G//A	nonsynonymous	1	0
MYBPC3	chr11	47353389/T//C	synonymous	1	0
IGHMBP2	chr11	68678981/A//G	synonymous	1	0
FAT3	chr11	92616284/T//G	nonsynonymous	1	0
FOXR1	chr11	118842525/G//A	synonymous	1	0
ETS1	chr11	128332460/T//C	synonymous	1	0
LRRK2	chr12	40758787/G//A	nonsynonymous	1	0
NAV3	chr12	78594291/G//T	nonsynonymous	1	0
APAF1	chr12	99056542/G//C	nonsynonymous	1	0
FBXO21	chr12	117612500/A//T	nonsynonymous	1	0
KDM2B	chr12	121947789/T//C	nonsynonymous	1	0
CENPJ	chr13	25486895/G//C	nonsynonymous	1	0

FREM2	chr13	39448724/G//T	nonsynonymous	1	0
VPS36	chr13	52997750/T//A	nonsynonymous	1	0
RNF219	chr13	79190883/C//T	nonsynonymous	1	0
CCDC168	chr13	103387740/G//T	nonsynonymous	1	0
MYO16	chr13	109779873/C//T	synonymous	1	0
RIN3	chr14	93119135/C//T	nonsynonymous	1	0
UNC79	chr14	93994980/A//T	nonsynonymous	1	0
FBN1	chr15	48722874/A//T	nonsynonymous	1	0
LIPC	chr15	58830528/G//A	synonymous	1	0
TLE3	chr15	70348685/C//T	nonsynonymous	1	0
PML	chr15	74337355/G//C	synonymous	1	0
PCSK6	chr15	101933442/C//T	nonsynonymous	1	0
LMF1	chr16	919948/G//A	nonsynonymous	1	0
LITAF	chr16	11650407/C//T	synonymous	1	0
E2F4	chr16	67226156/C//T	nonsynonymous	1	0
UTP4	chr16	69167365/G//A	nonsynonymous	1	0
ZNF469	chr16	88495363/C//A	synonymous	1	0
SMCR8	chr17	18219586/C//G	nonsynonymous	1	0
KIAA0100	chr17	26944322/A//G	synonymous	1	0
SLFN13	chr17	33772664/T//C	synonymous	1	0
DHX58	chr17	40257919/G//C	nonsynonymous	1	0
PSMC5	chr17	61908487/G//C	synonymous	1	0
BPTF	chr17	65920677/A//G	nonsynonymous	1	0
NPLOC4	chr17	79580474/A//G	nonsynonymous	1	0
SMAD4	chr18	48593533/G//T	nonsynonymous	1	0
PTPRS	chr19	5215339/C//T	nonsynonymous	1	0
PNPLA6	chr19	7605116/C//T	synonymous	1	0
MUC16	chr19	9076366/T//G	nonsynonymous	1	0
ZNF91	chr19	23542477/C//T	nonsynonymous	1	0
ZNF569	chr19	37903710/T//C	nonsynonymous	1	0
SIPA1L3	chr19	38573328/G//A	nonsynonymous	1	0
GPR4	chr19	46094222/C//A	synonymous	1	0
CCDC61	chr19	46506405/G//T	synonymous	1	0
GRWD1	chr19	48953982/G//A	nonsynonymous	1	0
NLRP13	chr19	56419313/CG//C	nonsynonymous	1	0
ACSS1	chr20	25004250/T//C	nonsynonymous	1	0
MYLK2	chr20	30421551/G//A	nonsynonymous	1	0
SLC32A1	chr20	37353531/G//T	nonsynonymous	1	0
ZHX3	chr20	39830767/C//A	nonsynonymous	1	0
PRPF6	chr20	62616366/G//A	nonsynonymous	1	0
C22orf31	chr22	29455047/G//A	nonsynonymous	1	0
CERK	chr22	47116047/C//A	nonsynonymous	1	0
RBMX	chrX	135961593/T//C	synonymous	1	0
SLITRK4	chrX	142716991/C//T	nonsynonymous	1	0
ZBTB40	chr1	22816680/C//T	nonsynonymous	0	1
NIPAL3	chr1	24771720/C//T	nonsynonymous	0	1
HIVEP3	chr1	42048132/C//G	nonsynonymous	0	1
TOE1	chr1	45806998/G//C	nonsynonymous	0	1
LRRC41	chr1	46745207/G//A	synonymous	0	1
ITGB3BP	chr1	63919611/C//G	nonsynonymous	0	1
CGN	chr1	151506506/G//A	nonsynonymous	0	1
GPR25	chr1	200842961/G//A	nonsynonymous	0	1
MIR3620	chr1	228284975/G//C	synonymous	0	1
RYR2	chr1	237881776/T//A	nonsynonymous	0	1
OSR1	chr2	19553320/G//A	nonsynonymous	0	1
USP34	chr2	61439008/C//G	nonsynonymous	0	1
FER1L5	chr2	97359669/C//T	nonsynonymous	0	1
ANKRD23	chr2	97505819/G//C	nonsynonymous	0	1
CLASP1	chr2	122216532/C//T	nonsynonymous	0	1
HECW2	chr2	197183624/C//G	nonsynonymous	0	1
ALPI	chr2	233321301/C//A	nonsynonymous	0	1
ZNF660	chr3	44636119/G//A	nonsynonymous	0	1
CTNND2	chr5	11565087/C//T	nonsynonymous	0	1
MYOZ3	chr5	150042509/C//A	synonymous	0	1
SLIT3	chr5	168216618/C//G	nonsynonymous	0	1
PRRC2A	chr6	31599524/G//A	nonsynonymous	0	1
FAM184A	chr6	119345733/T//C	synonymous	0	1
MYBL1	chr8	67507872/A//G	synonymous	0	1
MPDZ	chr9	13217243/T//C	synonymous	0	1
PSAT1	chr9	80943008/C//T	nonsynonymous	0	1

LOC100505478	chr9	117434068/G//C	synonymous	0	1
SET	chr9	131455933/C//T	nonsynonymous	0	1
MAMDC4	chr9	139749944/G//A	synonymous	0	1
PRTFDC1	chr10	25226229/G//C	nonsynonymous	0	1
CFAP46	chr10	134659655/G//T	nonsynonymous	0	1
SCT	chr11	626791/T//C	nonsynonymous	0	1
DCHS1	chr11	6648478/G//T	nonsynonymous	0	1
LUZP2	chr11	24759827/A//T	nonsynonymous	0	1
AHNAK	chr11	62288500/G//T	nonsynonymous	0	1
UCP3	chr11	73716830/A//T	synonymous	0	1
ARHGEF12	chr11	120348889/C//A	nonsynonymous	0	1
FLT3	chr13	28624352/C//A	nonsynonymous	0	1
SLC7A1	chr13	30097618/C//G	nonsynonymous	0	1
SPERT	chr13	46287373/C//T	synonymous	0	1
SETDB2-PHF11	chr13	50100564/T//C	nonsynonymous	0	1
BIVM-ERCC5	chr13	103524733/T//C	nonsynonymous	0	1
CGNL1	chr15	57731320/C//T	nonsynonymous	0	1
FASN	chr17	80046431/C//T	nonsynonymous	0	1
PTPN2	chr18	12794322/G//A	synonymous	0	1
RBBP8	chr18	20573441/G//C	nonsynonymous	0	1
PALM	chr19	746316/C//T	synonymous	0	1
PCSK4	chr19	1481622/C//G	synonymous	0	1
IL27RA	chr19	14157283/G//A	nonsynonymous	0	1
RYR1	chr19	38949964/C//T	synonymous	0	1
ZNF614	chr19	52519603/T//C	nonsynonymous	0	1
USP25	chr21	17197278/C//G	synonymous	0	1
SGSM1	chr22	25289403/C//T	synonymous	0	1
LMF2	chr22	50943390/G//A	synonymous	0	1
PCDH11X	chrX	91132676/C//A	nonsynonymous	0	1
SPANXN4	chrX	142122018/G//T	nonsynonymous	0	1
C1orf228	chr1	45190220/G//A	synonymous	0	1
CYP4A22	chr1	47603205/C//T	synonymous	0	1
GUK1	chr1	228336550/G//T	synonymous	0	1
GRHL1	chr2	10136459/G//A	nonsynonymous	0	1
ATAD2B	chr2	24098743/C//T	synonymous	0	1
XIRP2	chr2	168105216/C//T	synonymous	0	1
XIRP2	chr2	168115015/A//C	synonymous	0	1
TTN	chr2	179566951/C//T	nonsynonymous	0	1
CERKL	chr2	182413408/T//C	nonsynonymous	0	1
GLB1	chr3	33055712/G//A	synonymous	0	1
APC	chr5	112174446/CCAAA//C	nonsynonymous	0	1
DSP	chr6	7583247/G//A	nonsynonymous	0	1
ZNF318	chr6	43310552/T//TG	nonsynonymous	0	1
POM121L12	chr7	53103674/G//T	nonsynonymous	0	1
CADPS2	chr7	122303365/G//T	nonsynonymous	0	1
TRPA1	chr8	72981374/G//T	nonsynonymous	0	1
ZNF696	chr8	144378384/C//T	nonsynonymous	0	1
IFNA10	chr9	21207032/A//G	nonsynonymous	0	1
ADO	chr10	64565322/G//C	nonsynonymous	0	1
PTPMT1	chr11	47587472/G//C	nonsynonymous	0	1
DLAT	chr11	111904128/G//A	nonsynonymous	0	1
ROBO3	chr11	124746282/G//A	nonsynonymous	0	1
RECQL	chr12	21643151/G//A	nonsynonymous	0	1
WSCD2	chr12	108641993/G//A	nonsynonymous	0	1
OR5AU1	chr14	21623471/C//A	nonsynonymous	0	1
PTPN21	chr14	89016608/C//T	nonsynonymous	0	1
SHISA9	chr16	12996235/G//A	nonsynonymous	0	1
RSPRY1	chr16	57254765/T//G	synonymous	0	1
NOL3	chr16	67208366/G//A	synonymous	0	1
TNFSF12-TNFSF13	chr17	7463798/T//C	synonymous	0	1
TP53	chr17	7579389/G//A	nonsynonymous	0	1
AANAT	chr17	74465975/G//A	nonsynonymous	0	1
TMEM200C	chr18	5890968/C//A	nonsynonymous	0	1
ZNF626	chr19	20807139/G//T	nonsynonymous	0	1
VSTM2B	chr19	30019369/C//T	synonymous	0	1
CDH26	chr20	58560105/C//T	nonsynonymous	0	1
INPP5J	chr22	31518964/G//T	synonymous	0	1
MASP2	chr1	11087593/A//T	synonymous	1	0
AGTRAP	chr1	11807558/G//C	nonsynonymous	1	0
SLC2A1	chr1	43394892/T//C	nonsynonymous	1	0

DDX59	chr1	200613463/T//C	synonymous	1	0
PROX1	chr1	214170046/T//C	synonymous	1	0
WDR12	chr2	203759343/T//C	nonsynonymous	1	0
ATP2B2	chr3	10387792/G//A	nonsynonymous	1	0
ZBTB20	chr3	114058231/GAGA//G	nonsynonymous	1	0
FAM169A	chr5	74077730/T//A	nonsynonymous	1	0
CHD1	chr5	98234406/T//C	nonsynonymous	1	0
KIAA1549	chr7	138601515/C//T	nonsynonymous	1	0
C8orf86	chr8	38385932/T//C	nonsynonymous	1	0
PLCE1	chr10	96058431/G//A	synonymous	1	0
TAF6L	chr11	62554777/C//T	synonymous	1	0
MEN1	chr11	64575424/C//T	nonsynonymous	1	0
SLC13A2	chr17	26817530/C//T	nonsynonymous	1	0
LINGO3	chr19	2291614/G//A	synonymous	1	0
ZNF91	chr19	23543472/A//T	nonsynonymous	1	0
AKT2	chr19	40748451/C//A	nonsynonymous	1	0
OPRL1	chr20	62724092/G//A	nonsynonymous	1	0
CDK11B	chr1	1650787/T//C	nonsynonymous	0	1
CDK11B	chr1	1650797/A//G	nonsynonymous	0	1
CDK11B	chr1	1650801/T//C	synonymous	0	1
CDK11B	chr1	1650832/A//G	nonsynonymous	0	1
GABRD	chr1	1957086/G//A	nonsynonymous	0	1
HSPG2	chr1	22207275/A//G	synonymous	0	1
MR1	chr1	181019231/G//A	nonsynonymous	0	1
PPP1R12B	chr1	202396250/G//C	nonsynonymous	0	1
KCNH1	chr1	211093204/G//A	nonsynonymous	0	1
ADAM17	chr2	9676940/A//G	nonsynonymous	0	1
SRBD1	chr2	45645631/G//A	nonsynonymous	0	1
ARHGAP25	chr2	69002420/G//A	synonymous	0	1
CD207	chr2	71060088/C//A	nonsynonymous	0	1
NEB	chr2	152402887/A//G	nonsynonymous	0	1
TBR1	chr2	162280599/G//A	nonsynonymous	0	1
ABCA12	chr2	215809744/C//T	nonsynonymous	0	1
MREG	chr2	216809713/A//G	nonsynonymous	0	1
DNPEP	chr2	220251089/G//A	synonymous	0	1
HHLA2	chr3	108095384/G//A	nonsynonymous	0	1
TACR3	chr4	104640637/C//T	nonsynonymous	0	1
NIPBL	chr5	37048718/A//G	nonsynonymous	0	1
HTR1A	chr5	63257087/C//T	nonsynonymous	0	1
SERINC5	chr5	79441926/T//C	nonsynonymous	0	1
PCDHA7	chr5	140215525/G//A	synonymous	0	1
PCDHA8	chr5	140222472/G//A	synonymous	0	1
PCDHA10	chr5	140237454/G//A	synonymous	0	1
PCDHB4	chr5	140503064/C//T	nonsynonymous	0	1
CCT6A	chr7	56126095/A//G	nonsynonymous	0	1
AKAP9	chr7	91682104/G//A	nonsynonymous	0	1
MUC17	chr7	100680204/A//G	nonsynonymous	0	1
PODXL	chr7	131194344/G//A	nonsynonymous	0	1
ZDHHC2	chr8	17072741/T//A	synonymous	0	1
ASH2L	chr8	37986409/T//C	synonymous	0	1
TRPM3	chr9	73152199/G//A	nonsynonymous	0	1
KIF27	chr9	86518324/A//G	nonsynonymous	0	1
PALM2-AKAP2	chr9	112898561/G//T	nonsynonymous	0	1
ADAMTSL2	chr9	136433426/C//T	nonsynonymous	0	1
ABCA2	chr9	139902892/G//C	synonymous	0	1
CELF2	chr10	11370923/A//G	synonymous	0	1
RIC8A	chr11	211210/A//G	nonsynonymous	0	1
RAD9A	chr11	67160977/C//T	nonsynonymous	0	1
CEP126	chr11	101832479/G//T	nonsynonymous	0	1
NRIP2	chr12	2936359/G//A	synonymous	0	1
SOX5	chr12	23699322/C//A	nonsynonymous	0	1
LRRK2	chr12	40681230/A//C	nonsynonymous	0	1
GPR135	chr14	59930727/G//A	synonymous	0	1
CEP170B	chr14	105334833/C//T	nonsynonymous	0	1
LTK	chr15	41800415/G//C	synonymous	0	1
VPS39	chr15	42452920/T//C	synonymous	0	1
BLM	chr15	91347455/C//T	nonsynonymous	0	1
PRR25	chr16	857634/A//G	nonsynonymous	0	1
ZNF598	chr16	2048283/G//C	nonsynonymous	0	1
PRSS21	chr16	2868816/G//T	synonymous	0	1

TP53	chr17	7577520/A//G	nonsynonymous	0	1
EFTUD2	chr17	42942384/C//T	nonsynonymous	0	1
SGCA	chr17	48245074/G//A	nonsynonymous	0	1
TSPOAP1	chr17	56397923/C//T	nonsynonymous	0	1
CDH2	chr18	25532084/G//C	synonymous	0	1
DCC	chr18	50976983/G//T	nonsynonymous	0	1
ADNP2	chr18	77894047/A//G	nonsynonymous	0	1
ZNF585B	chr19	37677297/G//T	nonsynonymous	0	1
CENPB	chr20	3766584/C//T	nonsynonymous	0	1
PAK5	chr20	9561521/C//T	synonymous	0	1
TGM2	chr20	36767960/G//T	nonsynonymous	0	1
KRTAP27-1	chr21	31709459/C//A	synonymous	0	1
GAL3ST1	chr22	30951763/C//T	nonsynonymous	0	1
PRAMEF12	chr1	12835154/C//G	nonsynonymous	0	1
FMO4	chr1	171292308/C//A	nonsynonymous	0	1
DENND1B	chr1	197611844/G//A	synonymous	0	1
SOX13	chr1	204092268/C//G	nonsynonymous	0	1
DNAH14	chr1	225569113/G//T	nonsynonymous	0	1
THADA	chr2	43755127/C//A	nonsynonymous	0	1
GPR45	chr2	105858719/T//C	nonsynonymous	0	1
TTN	chr2	179412786/C//A	nonsynonymous	0	1
TTN	chr2	179634967/C//A	nonsynonymous	0	1
NAA50	chr3	113440545/A//G	synonymous	0	1
CHRD	chr3	184099066/GCAA//G	nonsynonymous	0	1
IQCG	chr3	197672522/C//T	synonymous	0	1
PPARGC1A	chr4	23826133/T//G	nonsynonymous	0	1
KIAA1211	chr4	57181047/G//A	nonsynonymous	0	1
TBC1D9	chr4	141600863/A//T	synonymous	0	1
DCHS2	chr4	155219563/G//C	nonsynonymous	0	1
PCDHA8	chr5	140221579/A//G	nonsynonymous	0	1
EYS	chr6	66205088/T//G	nonsynonymous	0	1
COL12A1	chr6	75801249/A//C	synonymous	0	1
COQ3	chr6	99825258/T//G	synonymous	0	1
TBPL1	chr6	134301402/A//C	synonymous	0	1
PDE7B	chr6	136268617/T//C	synonymous	0	1
MYCT1	chr6	153043287/A//T	nonsynonymous	0	1
DDC	chr7	50544318/T//G	synonymous	0	1
MAGI2	chr7	77762200/T//G	synonymous	0	1
MUC17	chr7	100685940/C//A	nonsynonymous	0	1
WNT2	chr7	116918022/T//G	synonymous	0	1
WEE2	chr7	141429417/C//G	nonsynonymous	0	1
ZNF703	chr8	37554909/G//A	nonsynonymous	0	1
ZNF703	chr8	37555631/T//C	synonymous	0	1
SYBU	chr8	110631127/C//A	nonsynonymous	0	1
UBAP2	chr9	33960876/C//T	nonsynonymous	0	1
GDA	chr9	74817499/G//A	nonsynonymous	0	1
AGTPBP1	chr9	88247786/A//C	nonsynonymous	0	1
OR1J2	chr9	125273400/T//G	nonsynonymous	0	1
GOLGA2	chr9	131020426/G//T	nonsynonymous	0	1
PPP1R26	chr9	138377413/G//T	nonsynonymous	0	1
CDH23	chr10	73574717/G//C	nonsynonymous	0	1
KCNQ1	chr11	2797192/A//C	nonsynonymous	0	1
	chr11	84996265/T//C	synonymous	0	1
SLC2A3	chr12	8078549/G//A	synonymous	0	1
KMT2D	chr12	49418658/C//A	nonsynonymous	0	1
KMT2D	chr12	49444157/C//T	nonsynonymous	0	1
CIT	chr12	120139444/A//C	nonsynonymous	0	1
RAB35	chr12	120535006/G//C	synonymous	0	1
EP400	chr12	132512807/A//C	synonymous	0	1
ZNF268	chr12	133779791/A//G	nonsynonymous	0	1
NYNRIN	chr14	24879039/C//T	nonsynonymous	0	1
INSM2	chr14	36004223/C//T	synonymous	0	1
SYNE2	chr14	64421439/A//G	nonsynonymous	0	1
CLPX	chr15	65477465/G//GC	nonsynonymous	0	1
PKD1	chr16	2161340/C//T	synonymous	0	1
CREBBP	chr16	3807810/C//T	synonymous	0	1
NF1	chr17	29546029/A//T	nonsynonymous	0	1
SLC4A1	chr17	42333152/A//C	synonymous	0	1
DAZAP1	chr19	1432644/A//G	nonsynonymous	0	1
ZC3H4	chr19	47584802/G//A	synonymous	0	1

RPL18	chr19	49118612/G//C	synonymous	0	1
TMEM86B	chr19	55738697/A//G	nonsynonymous	0	1
PRKX	chrX	3631276/C//T	nonsynonymous	0	1
HUWE1	chrX	53569489/G//C	nonsynonymous	0	1
RGAG1	chrX	109694237/C//A	nonsynonymous	0	1
TRPC5	chrX	111019549/G//C	nonsynonymous	0	1
FAM131C	chr1	16385178/G//A	synonymous	1	0
RWDD3	chr1	95712119/A//G	nonsynonymous	1	0
PIGC	chr1	172411009/C//A	nonsynonymous	1	0
OBSCN	chr1	228520597/C//T	nonsynonymous	1	0
NRXN1	chr2	50779731/G//A	nonsynonymous	1	0
LRP1B	chr2	141294186/A//T	nonsynonymous	1	0
KCNH8	chr3	19389246/G//A	synonymous	1	0
USP4	chr3	49323727/T//A	nonsynonymous	1	0
CASR	chr3	121975932/A//G	nonsynonymous	1	0
GHSR	chr3	172163134/G//A	synonymous	1	0
PIGX	chr3	196443721/T//C	synonymous	1	0
TSPAN5	chr4	99399908/G//A	synonymous	1	0
NDST3	chr4	119176902/T//C	synonymous	1	0
APC	chr5	112175696/C//T	nonsynonymous	1	0
PCDHA1	chr5	140167661/G//A	nonsynonymous	1	0
PCDHB4	chr5	140503423/G//A	nonsynonymous	1	0
PHYKPL	chr5	177658507/AAG//A	nonsynonymous	1	0
OR2J3	chr6	29079940/G//A	synonymous	1	0
EVX1	chr7	27285585/C//T	synonymous	1	0
EGFR	chr7	55259515/T//G	nonsynonymous	1	0
AKAP9	chr7	91630518/G//A	synonymous	1	0
NCAPG2	chr7	158449050/T//G	nonsynonymous	1	0
CSMD3	chr8	113349934/G//A	nonsynonymous	1	0
SLC34A3	chr9	140130574/G//A	synonymous	1	0
LIPJ	chr10	90351154/G//A	nonsynonymous	1	0
SLIT1	chr10	98824565/A//G	nonsynonymous	1	0
PPRC1	chr10	103901141/G//C	nonsynonymous	1	0
INSC	chr11	15267536/C//A	nonsynonymous	1	0
ABTB2	chr11	34219090/T//C	synonymous	1	0
OR5M1	chr11	56380231/A//C	nonsynonymous	1	0
CTTN	chr11	70267667/C//T	synonymous	1	0
AQP11	chr11	77320390/C//T	nonsynonymous	1	0
CEP164	chr11	117209303/A//G	nonsynonymous	1	0
SENP1	chr12	48459426/G//A	nonsynonymous	1	0
GPD1	chr12	50501914/G//GT	synonymous	1	0
ZNF84	chr12	133635381/C//T	nonsynonymous	1	0
AKAP11	chr13	42877556/T//A	synonymous	1	0
MYH7	chr14	23895255/G//A	nonsynonymous	1	0
XRCC3	chr14	104177401/C//G	synonymous	1	0
ANPEP	chr15	90349699/T//C	nonsynonymous	1	0
RPL3L	chr16	1995568/A//G	nonsynonymous	1	0
RANBP10	chr16	67765470/A//G	nonsynonymous	1	0
PHLPP2	chr16	71710482/G//A	nonsynonymous	1	0
ARHGEF15	chr17	8221739/G//A	nonsynonymous	1	0
SAP30BP	chr17	73702437/A//G	nonsynonymous	1	0
UNK	chr17	73812870/C//T	synonymous	1	0
ACAA2	chr18	47310300/G//A	nonsynonymous	1	0
AP3D1	chr19	2117371/G//A	synonymous	1	0
MUC16	chr19	9071760/G//T	nonsynonymous	1	0
ERICH3	chr1	75072397/T//G	nonsynonymous	0	1
AMY2B	chr1	104114231/T//A	nonsynonymous	0	1
ITGA10	chr1	145535814/C//T	nonsynonymous	0	1
PRRX1	chr1	170689027/C//T	synonymous	0	1
ASTN1	chr1	176992581/C//T	nonsynonymous	0	1
PTPRC	chr1	198665871/C//T	nonsynonymous	0	1
CHRM3	chr1	240071166/T//G	nonsynonymous	0	1
GREM2	chr1	240656109/C//T	synonymous	0	1
TAF1B	chr2	10074184/T//G	synonymous	0	1
GRHL1	chr2	10091847/G//T	synonymous	0	1
THADA	chr2	43804326/A//G	nonsynonymous	0	1
FSHR	chr2	49381425/G//A	synonymous	0	1
LRP1B	chr2	141031998/A//C	nonsynonymous	0	1
LRP2	chr2	170063759/T//A	synonymous	0	1
TTN	chr2	179641722/T//G	nonsynonymous	0	1

ZNF804A	chr2	185802205/A//C	nonsynonymous	0	1
HECW2	chr2	197189764/G//A	synonymous	0	1
PLCL1	chr2	198949664/A//T	nonsynonymous	0	1
NUP210	chr3	13372074/C//T	synonymous	0	1
MYRIP	chr3	40223746/A//C	nonsynonymous	0	1
FBXW12	chr3	48436036/G//A	nonsynonymous	0	1
RABL3	chr3	120428642/A//G	nonsynonymous	0	1
TMEM212	chr3	171561264/T//C	nonsynonymous	0	1
NLGN1	chr3	173997382/A//C	nonsynonymous	0	1
ATP5I	chr4	666231/A//G	synonymous	0	1
CCSER1	chr4	91230116/G//A	synonymous	0	1
SLC6A18	chr5	1246053/C//T	nonsynonymous	0	1
OR2B6	chr6	27925707/C//T	nonsynonymous	0	1
LEMD2	chr6	33746106/C//G	nonsynonymous	0	1
CUL9	chr6	43180922/C//T	nonsynonymous	0	1
TIAM2	chr6	155451528/T//C	nonsynonymous	0	1
THSD7A	chr7	11446622/G//T	nonsynonymous	0	1
ZCWPW1	chr7	100017306/G//A	nonsynonymous	0	1
POT1	chr7	124532400/T//G	nonsynonymous	0	1
CNTNAP2	chr7	145813996/G//A	nonsynonymous	0	1
DCAF13	chr8	104427572/T//C	synonymous	0	1
PLEC	chr8	145001444/C//T	synonymous	0	1
GLDC	chr9	6588660/C//T	synonymous	0	1
ITIH5	chr10	7608303/A//C	synonymous	0	1
NRXN2	chr11	64418887/C//T	nonsynonymous	0	1
DSCAML1	chr11	117389342/C//T	nonsynonymous	0	1
IQSEC3	chr12	274617/G//A	synonymous	0	1
CLEC4D	chr12	8667928/A//C	synonymous	0	1
SARNP	chr12	56151228/T//A	synonymous	0	1
EID3	chr12	104697876/G//A	nonsynonymous	0	1
ASCL4	chr12	108169082/A//G	synonymous	0	1
TUBA3C	chr13	19751632/T//G	nonsynonymous	0	1
RNF17	chr13	25442748/C//A	nonsynonymous	0	1
N4BP2L2	chr13	33095556/G//A	nonsynonymous	0	1
LPAR6	chr13	48985827/A//G	nonsynonymous	0	1
ABCC4	chr13	95887046/T//A	nonsynonymous	0	1
NALCN	chr13	101747983/A//C	nonsynonymous	0	1
DCAF4	chr14	73406514/G//A	nonsynonymous	0	1
VIPAS39	chr14	77901705/T//C	nonsynonymous	0	1
SLIRP	chr14	78183868/TC//T	nonsynonymous	0	1
	chr15	49448118/A//G	synonymous	0	1
ACSBG1	chr15	78474467/G//A	synonymous	0	1
KRTAP9-8	chr17	39394755/C//A	nonsynonymous	0	1
MLX	chr17	40723583/A//G	nonsynonymous	0	1
DCC	chr18	50278731/A//C	nonsynonymous	0	1
SALL3	chr18	76754379/C//T	synonymous	0	1
APC2	chr19	1460266/C//T	nonsynonymous	0	1
ECSIT	chr19	11624022/T//G	nonsynonymous	0	1
ZNF823	chr19	11833425/T//C	synonymous	0	1
PLEKHF1	chr19	30165133/C//T	synonymous	0	1
	chr19	40903966/G//A	synonymous	0	1
AURKC	chr19	57746736/A//C	nonsynonymous	0	1
IL3RA	chrX	1460714/C//T	nonsynonymous	0	1
SMPX	chrX	21755760/A//C	nonsynonymous	0	1
SLC9A7	chrX	46502788/C//T	nonsynonymous	0	1
OGT	chrX	70776893/C//T	nonsynonymous	0	1
PCDH11X	chrX	91090643/T//G	nonsynonymous	0	1
HTR2C	chrX	113965704/G//A	nonsynonymous	0	1
KLHL13	chrX	117079487/G//A	synonymous	0	1
TENM1	chrX	123518690/G//A	nonsynonymous	0	1
	chrX	131803197/G//A	synonymous	0	1
MAGEC3	chrX	140969216/T//G	synonymous	0	1
FMR1	chrX	147026492/C//A	nonsynonymous	0	1
SAMD11	chr1	861391/G//T	nonsynonymous	1	0
PRDM2	chr1	14108450/A//T	nonsynonymous	1	0
PADI6	chr1	17727866/G//A	nonsynonymous	1	0
SYNC	chr1	33168243/G//A	nonsynonymous	1	0
PODN	chr1	53542934/C//T	synonymous	1	0
WDR78	chr1	67306311/T//G	nonsynonymous	1	0
ERICH3	chr1	75078453/G//C	synonymous	1	0

LHX8	chr1	75606699/T//C	synonymous	1	0
WDR63	chr1	85570221/C//A	synonymous	1	0
CLCA4	chr1	87031141/A//G	synonymous	1	0
CCDC18	chr1	93671064/T//A	synonymous	1	0
OLFM3	chr1	102290644/T//C	nonsynonymous	1	0
RSBN1	chr1	114310929/C//T	nonsynonymous	1	0
SPAG17	chr1	118512746/G//C	synonymous	1	0
PRKAB2	chr1	146639484/C//T	nonsynonymous	1	0
NIT1	chr1	161089132/G//A	nonsynonymous	1	0
LMX1A	chr1	165322399/G//A	synonymous	1	0
IPO9	chr1	201842030/A//C	nonsynonymous	1	0
OBSCN	chr1	228522880/C//T	nonsynonymous	1	0
RYR2	chr1	237604682/G//A	nonsynonymous	1	0
RYR2	chr1	237774191/AAGGT//A	nonsynonymous	1	0
OR13G1	chr1	247836290/A//G	synonymous	1	0
ALK	chr2	29462567/C//G	nonsynonymous	1	0
CYP26B1	chr2	72371240/G//A	nonsynonymous	1	0
REG3G	chr2	79253961/A//C	synonymous	1	0
IL36A	chr2	113763452/G//T	nonsynonymous	1	0
GLI2	chr2	121747479/C//A	nonsynonymous	1	0
GALNT5	chr2	158157451/A//G	synonymous	1	0
TTN	chr2	179474878/G//C	nonsynonymous	1	0
DNAH7	chr2	196825050/T//C	nonsynonymous	1	0
RPL37A	chr2	217363579/G//C	synonymous	1	0
OSBPL10	chr3	32022448/G//A	nonsynonymous	1	0
ZMYND10	chr3	50380768/C//A	synonymous	1	0
ABHD14A-ACY1	chr3	52018099/G//A	nonsynonymous	1	0
PBRM1	chr3	52597433/C//T	nonsynonymous	1	0
IFT122	chr3	129195521/G//A	nonsynonymous	1	0
MCF2L2	chr3	183035994/G//A	synonymous	1	0
TNK2	chr3	195591056/C//T	nonsynonymous	1	0
WHSC1	chr4	1961285/C//G	nonsynonymous	1	0
PROM1	chr4	15982078/T//C	nonsynonymous	1	0
LNX1	chr4	54364904/G//C	nonsynonymous	1	0
FRAS1	chr4	79403122/C//T	synonymous	1	0
HERC6	chr4	89357009/G//A	nonsynonymous	1	0
LEF1	chr4	109088840/G//C	nonsynonymous	1	0
PLA2G12A	chr4	110650919/A//C	nonsynonymous	1	0
CDH12	chr5	21854880/G//C	synonymous	1	0
SPEF2	chr5	35727924/A//T	nonsynonymous	1	0
C6	chr5	41201726/G//T	nonsynonymous	1	0
ADAMTS19	chr5	129019824/T//C	synonymous	1	0
DDX46	chr5	134124246/C//CG	nonsynonymous	1	0
ECSCR	chr5	138837724/C//G	nonsynonymous	1	0
PCDHA8	chr5	140223265/G//A	nonsynonymous	1	0
PCDHA13	chr5	140261925/G//A	nonsynonymous	1	0
PCDHB11	chr5	140581500/G//C	nonsynonymous	1	0
FOXI1	chr5	169533147/C//T	synonymous	1	0
SLC26A8	chr6	35945132/G//A	synonymous	1	0
IBTK	chr6	82911146/C//T	nonsynonymous	1	0
LAMA2	chr6	129419363/C//T	nonsynonymous	1	0
MOXD1	chr6	132722526/G//T	nonsynonymous	1	0
TNRC18	chr7	5428772/C//A	nonsynonymous	1	0
THSD7A	chr7	11521407/C//T	synonymous	1	0
AMPH	chr7	38505847/G//T	synonymous	1	0
VPS41	chr7	38812198/G//A	nonsynonymous	1	0
SPDYE5	chr7	75130995/G//A	synonymous	1	0
AKAP9	chr7	91709358/A//C	nonsynonymous	1	0
MCM7	chr7	99694983/C//T	nonsynonymous	1	0
ACTL6B	chr7	100246358/G//T	nonsynonymous	1	0
SSPO	chr7	149486000/G//T	nonsynonymous	1	0
HR	chr8	21978433/T//C	synonymous	1	0
ADAMDEC1	chr8	24256928/C//T	nonsynonymous	1	0
UBXN8	chr8	30618400/T//C	synonymous	1	0
KCNB2	chr8	73480417/G//A	nonsynonymous	1	0
CALB1	chr8	91081456/C//T	nonsynonymous	1	0
ADCY8	chr8	132002695/C//T	nonsynonymous	1	0
RUSC2	chr9	35560740/C//T	nonsynonymous	1	0
PRUNE2	chr9	79320090/C//A	nonsynonymous	1	0
ZBTB6	chr9	125674140/C//A	nonsynonymous	1	0

NDOR1	chr9	140108752/G//A	synonymous	1	0
SFRP5	chr10	99531274/A//T	nonsynonymous	1	0
GSTO1	chr10	106019550/T//G	nonsynonymous	1	0
FGFR2	chr10	123239462/G//A	nonsynonymous	1	0
CD81	chr11	2417941/C//A	synonymous	1	0
ARFIP2	chr11	6498409/C//T	synonymous	1	0
SSRP1	chr11	57099257/G//A	nonsynonymous	1	0
RASGRP2	chr11	64507104/G//C	synonymous	1	0
BSX	chr11	122849968/C//A	nonsynonymous	1	0
SIAE	chr11	124543589/G//A	nonsynonymous	1	0
TMTC1	chr12	29904803/C//T	nonsynonymous	1	0
PRPF40B	chr12	50024333/T//A	synonymous	1	0
SOAT2	chr12	53509290/TGTGGGCCAG//T	nonsynonymous	1	0
AAAS	chr12	53701719/A//C	synonymous	1	0
ITGA7	chr12	56088586/T//A	synonymous	1	0
AVPR1A	chr12	63544123/C//T	nonsynonymous	1	0
FLT3	chr13	28626732/G//A	synonymous	1	0
PCDH17	chr13	58209215/G//A	synonymous	1	0
TNFSF13B	chr13	108959197/G//C	nonsynonymous	1	0
MYH7	chr14	23894973/C//A	nonsynonymous	1	0
AP1G2	chr14	24036440/G//A	synonymous	1	0
TSSK4	chr14	24676367/G//T	nonsynonymous	1	0
SIX4	chr14	61186701/G//C	synonymous	1	0
TRPM1	chr15	31360147/C//A	nonsynonymous	1	0
MYO5C	chr15	52497148/C//T	synonymous	1	0
TSC2	chr16	2106224/C//T	synonymous	1	0
TXNDC11	chr16	11773348/C//T	synonymous	1	0
POLR3E	chr16	22320356/T//G	nonsynonymous	1	0
RPGRIP1L	chr16	53686660/C//T	nonsynonymous	1	0
DHODH	chr16	72048400/G//C	nonsynonymous	1	0
CCL7	chr17	32598875/A//G	synonymous	1	0
	chr17	40726081/G//C	synonymous	1	0
HSF5	chr17	56540230/A//G	synonymous	1	0
HSF5	chr17	56557264/G//C	nonsynonymous	1	0
SCN4A	chr17	62043565/C//T	nonsynonymous	1	0
TMC8	chr17	76137064/G//A	synonymous	1	0
DNAH17	chr17	76488710/G//A	synonymous	1	0
EMILIN2	chr18	2892078/G//C	nonsynonymous	1	0
KIAA1468	chr18	59894675/C//G	nonsynonymous	1	0
ZNF516	chr18	74092160/C//T	nonsynonymous	1	0
ZNF585A	chr19	37644037/G//C	nonsynonymous	1	0
FCGBP	chr19	40412151/G//A	synonymous	1	0
PLA2G4C	chr19	48565308/G//A	nonsynonymous	1	0
SMIM17	chr19	57157174/G//T	nonsynonymous	1	0
DUSP15	chr20	30457385/A//T	nonsynonymous	1	0
CHD6	chr20	40044062/C//G	nonsynonymous	1	0
UQCR10	chr22	30165790/G//C	synonymous	1	0
RNF215	chr22	30775472/G//A	synonymous	1	0
DRG1	chr22	31830011/C//T	synonymous	1	0
ELFN2	chr22	37770945/G//A	synonymous	1	0
MICALL1	chr22	38317999/C//T	nonsynonymous	1	0
MAPK8IP2	chr22	51042070/A//C	nonsynonymous	1	0
SYTL5	chrX	37931380/ATGT//A	nonsynonymous	1	0
ATRX	chrX	76777815/T//C	nonsynonymous	1	0
TMEM257	chrX	144909390/C//A	nonsynonymous	1	0
TGIF2LY	chrY	3447882/C//A	synonymous	1	0
CTRC	chr1	15768948/C//T	nonsynonymous	0	1
PADI3	chr1	17609652/C//T	synonymous	0	1
GRIK3	chr1	37307343/G//A	synonymous	0	1
SZT2	chr1	43893034/T//A	synonymous	0	1
ACOT11	chr1	55073682/C//T	nonsynonymous	0	1
ATP1A4	chr1	160124948/C//T	synonymous	0	1
FMO3	chr1	171086555/A//C	nonsynonymous	0	1
PAPPA2	chr1	176526075/C//T	nonsynonymous	0	1
MYBPH	chr1	203137795/C//T	nonsynonymous	0	1
RBBP5	chr1	205070777/T//C	nonsynonymous	0	1
EXO1	chr1	242035540/G//T	nonsynonymous	0	1
KIF26B	chr1	245852001/G//A	nonsynonymous	0	1
UGGT1	chr2	128945048/C//G	nonsynonymous	0	1
ITPR1	chr3	4735251/G//A	synonymous	0	1

DYNC1LI1	chr3	32582626/G//A	nonsynonymous	0	1
CCDC36	chr3	49294687/T//C	nonsynonymous	0	1
SEMA3F	chr3	50212616/C//T	nonsynonymous	0	1
ABHD14A	chr3	52014422/G//A	synonymous	0	1
OR5AC2	chr3	97806608/G//A	nonsynonymous	0	1
DNAJB8	chr3	128181993/G//A	synonymous	0	1
EPHB1	chr3	134851646/G//A	nonsynonymous	0	1
EIF4G1	chr3	184035283/C//T	nonsynonymous	0	1
TRMT44	chr4	8469692/G//A	nonsynonymous	0	1
CFI	chr4	110670375/T//C	nonsynonymous	0	1
FRG1	chr4	190878567/G//C	nonsynonymous	0	1
FRG1	chr4	190878583/T//C	nonsynonymous	0	1
DNAH5	chr5	13752300/A//G	synonymous	0	1
CDH18	chr5	19838868/C//T	synonymous	0	1
EDIL3	chr5	83476288/T//C	nonsynonymous	0	1
APC	chr5	112174559/C//T	nonsynonymous	0	1
PCDHGA1	chr5	140711927/C//T	nonsynonymous	0	1
PCDHGB5	chr5	140778270/G//A	synonymous	0	1
FAT2	chr5	150948328/G//A	synonymous	0	1
FLT4	chr5	180057648/C//T	nonsynonymous	0	1
TRIM41	chr5	180651016/T//C	nonsynonymous	0	1
CAGE1	chr6	7373663/T//G	nonsynonymous	0	1
OR2B2	chr6	27879093/G//A	synonymous	0	1
ZBED9	chr6	28543508/C//T	nonsynonymous	0	1
COL21A1	chr6	56035494/C//T	nonsynonymous	0	1
EYS	chr6	65300131/G//A	nonsynonymous	0	1
NMBR	chr6	142399786/G//A	nonsynonymous	0	1
IFT22	chr7	100959700/C//T	synonymous	0	1
CSGALNACT1	chr8	19362817/G//A	nonsynonymous	0	1
PLEC	chr8	144998131/C//T	nonsynonymous	0	1
SARDH	chr9	136582462/G//A	nonsynonymous	0	1
OLFM1	chr9	137990330/C//T	nonsynonymous	0	1
DCHS1	chr11	6661913/G//A	nonsynonymous	0	1
ANO5	chr11	22284516/G//C	nonsynonymous	0	1
F2	chr11	46747438/C//A	nonsynonymous	0	1
MS4A6A	chr11	59942940/C//T	nonsynonymous	0	1
KDM5A	chr12	431588/T//C	synonymous	0	1
GNB3	chr12	6952177/C//T	nonsynonymous	0	1
PHC1	chr12	9092026/G//A	nonsynonymous	0	1
KMT2D	chr12	49435967/C//T	nonsynonymous	0	1
FZD10	chr12	130647800/G//A	nonsynonymous	0	1
	chr13	109282131/C//T	synonymous	0	1
FBXO33	chr14	39870376/C//T	synonymous	0	1
TDRD9	chr14	104422039/A//G	nonsynonymous	0	1
ATP10A	chr15	25932891/G//A	nonsynonymous	0	1
DISP2	chr15	40656103/C//T	nonsynonymous	0	1
SHC4	chr15	49127051/C//T	nonsynonymous	0	1
CYP11A1	chr15	74640284/C//T	nonsynonymous	0	1
RGS11	chr16	321199/A//T	nonsynonymous	0	1
ZNF263	chr16	3339607/G//T	synonymous	0	1
TIGD7	chr16	3350159/A//T	nonsynonymous	0	1
NOD2	chr16	50745613/G//A	synonymous	0	1
HYDIN	chr16	70883691/C//A	nonsynonymous	0	1
TP53	chr17	7579310/A//C	nonsynonymous	0	1
LRRC37B	chr17	30376310/A//G	nonsynonymous	0	1
KRT25	chr17	38906799/C//T	synonymous	0	1
KRT23	chr17	39092610/G//A	synonymous	0	1
NT5C3B	chr17	39985135/G//A	synonymous	0	1
PRPSAP1	chr17	74326675/T//G	nonsynonymous	0	1
ADAMTS10	chr19	8654233/C//T	nonsynonymous	0	1
SLC44A2	chr19	10747173/G//T	nonsynonymous	0	1
ZNF69	chr19	11998783/A//T	synonymous	0	1
ZNF536	chr19	30935611/G//A	nonsynonymous	0	1
CPT1C	chr19	50208001/G//A	nonsynonymous	0	1
NUP62	chr19	50412073/G//A	nonsynonymous	0	1
ZNF134	chr19	58132017/A//G	nonsynonymous	0	1
BFSP1	chr20	17474941/C//T	synonymous	0	1
SLC19A1	chr21	46951450/G//A	nonsynonymous	0	1
MTFP1	chr22	30823165/G//T	nonsynonymous	0	1
INPP5J	chr22	31522468/G//A	nonsynonymous	0	1

CYLC1	chrX	83128538/G//A	synonymous	0	1
DACH2	chrX	85906054/C//T	nonsynonymous	0	1
SLC35E2	chr1	1663913/G//C	nonsynonymous	0	1
WRAP73	chr1	3552499/TCTTCATAC//T	nonsynonymous	0	1
FAM131C	chr1	16384916/C//T	synonymous	0	1
ARID1A	chr1	27105519/A//AGG	nonsynonymous	0	1
SLC9A1	chr1	27428555/C//T	synonymous	0	1
EPHA10	chr1	38188716/C//T	nonsynonymous	0	1
MACF1	chr1	39550062/C//T	nonsynonymous	0	1
RLF	chr1	40705248/A//G	nonsynonymous	0	1
CDC14A	chr1	100905504/A//G	nonsynonymous	0	1
S1PR1	chr1	101704808/T//G	nonsynonymous	0	1
DDX20	chr1	112308549/A//C	synonymous	0	1
SELP	chr1	169586423/C//A	nonsynonymous	0	1
FMO2	chr1	171174569/T//C	nonsynonymous	0	1
TPR	chr1	186283112/A//G	synonymous	0	1
CFH	chr1	196642222/C//G	nonsynonymous	0	1
ZBTB41	chr1	197169098/T//C	nonsynonymous	0	1
EXOC8	chr1	231473467/C//G	nonsynonymous	0	1
OR2G2	chr1	247752442/A//C	nonsynonymous	0	1
APOB	chr2	21233293/A//G	synonymous	0	1
EML4	chr2	42396691/T//A	synonymous	0	1
EPCAM	chr2	47612303/A//G	nonsynonymous	0	1
ANKRD39	chr2	97514129/G//T	nonsynonymous	0	1
STAM2	chr2	153004806/T//C	nonsynonymous	0	1
PLA2R1	chr2	160833203/G//A	synonymous	0	1
FAP	chr2	163039918/A//G	synonymous	0	1
TTN	chr2	179647006/C//A	nonsynonymous	0	1
COL5A2	chr2	189916071/C//A	nonsynonymous	0	1
ANKRD44	chr2	197946442/T//C	nonsynonymous	0	1
DES	chr2	220290383/A//T	nonsynonymous	0	1
CTNNB1	chr3	41266097/G//T	nonsynonymous	0	1
COL7A1	chr3	48618031/C//T	nonsynonymous	0	1
CELSR3	chr3	48688892/C//T	nonsynonymous	0	1
QRICH1	chr3	49095091/TG//T	nonsynonymous	0	1
USP19	chr3	49152418/C//T	nonsynonymous	0	1
GLT8D1	chr3	52729011/C//G	nonsynonymous	0	1
TMEM39A	chr3	119176890/T//C	nonsynonymous	0	1
	chr3	119198852/A//G	synonymous	0	1
HEG1	chr3	124732119/T//C	synonymous	0	1
TXNRD3	chr3	126329898/G//C	nonsynonymous	0	1
SLC35G2	chr3	136574275/T//A	nonsynonymous	0	1
P3H2	chr3	189692438/T//C	nonsynonymous	0	1
SLC26A1	chr4	983497/G//A	synonymous	0	1
AFAP1	chr4	7802283/C//A	nonsynonymous	0	1
LAP3	chr4	17609065/C//T	synonymous	0	1
SLC34A2	chr4	25676209/C//T	synonymous	0	1
GABRA2	chr4	46252492/T//C	nonsynonymous	0	1
GYPA	chr4	145040861/A//G	synonymous	0	1
KLHL2	chr4	166234493/A//G	synonymous	0	1
SH3RF1	chr4	170051220/C//T	nonsynonymous	0	1
CDKN2AIP	chr4	184368521/G//T	nonsynonymous	0	1
TERT	chr5	1279531/G//A	nonsynonymous	0	1
SEMA5A	chr5	9197323/C//T	nonsynonymous	0	1
DNAH5	chr5	13844994/A//G	synonymous	0	1
CDH9	chr5	26881242/C//T	synonymous	0	1
CDH9	chr5	26916007/T//A	nonsynonymous	0	1
PDZD2	chr5	32000286/A//G	nonsynonymous	0	1
NIPBL	chr5	36985664/A//T	nonsynonymous	0	1
OSMR	chr5	38876479/A//G	synonymous	0	1
MOCS2	chr5	52405470/A//T	synonymous	0	1
MIER3	chr5	56246485/A//C	synonymous	0	1
CRHBP	chr5	76264653/C//G	nonsynonymous	0	1
KIAA0825	chr5	93800652/A//C	nonsynonymous	0	1
SLCO6A1	chr5	101726686/A//T	nonsynonymous	0	1
PPIP5K2	chr5	102515775/T//G	synonymous	0	1
PRRC1	chr5	126866083/A//AT	nonsynonymous	0	1
LEAP2	chr5	132209447/A//C	synonymous	0	1
TRPC7	chr5	135692447/T//C	nonsynonymous	0	1
HBEGF	chr5	139725660/G//A	nonsynonymous	0	1

SLC36A2	chr5	150696625/C//A	nonsynonymous	0	1
CCNJL	chr5	159682670/C//G	nonsynonymous	0	1
ZNF454	chr5	178392535/T//C	nonsynonymous	0	1
HNRNPH1	chr5	179045228/G//A	synonymous	0	1
POM121L2	chr6	27278478/C//A	nonsynonymous	0	1
HLA-DRB1	chr6	32548515/C//T	synonymous	0	1
PSMB8	chr6	32811680/G//T	nonsynonymous	0	1
CDKN1A	chr6	36651879/A//G	nonsynonymous	0	1
ASCC3	chr6	101073128/C//A	nonsynonymous	0	1
ARHGAP18	chr6	129905179/C//A	nonsynonymous	0	1
CARD11	chr7	2987326/G//A	nonsynonymous	0	1
PAPOLB	chr7	4901465/G//GCACGTCCCCCACC	synonymous	0	1
URGCP	chr7	43918522/TGGCGTATCAGGGGT(	nonsynonymous	0	1
PCLO	chr7	82784089/A//C	nonsynonymous	0	1
MBLAC1	chr7	99725815/A//T	nonsynonymous	0	1
SH2B2	chr7	101943811/G//A	nonsynonymous	0	1
KCNH2	chr7	150648732/G//T	synonymous	0	1
CLDN23	chr8	8560387/T//G	nonsynonymous	0	1
RP1L1	chr8	10469901/G//T	nonsynonymous	0	1
UNC5D	chr8	35648059/A//G	nonsynonymous	0	1
OSR2	chr8	99961829/G//T	nonsynonymous	0	1
FREM1	chr9	14851595/G//A	nonsynonymous	0	1
CDKN2A	chr9	21971209/T//G	nonsynonymous	0	1
IFNK	chr9	27524842/G//C	nonsynonymous	0	1
RECK	chr9	36122893/C//T	nonsynonymous	0	1
ALDOB	chr9	104189834/T//A	nonsynonymous	0	1
CHAT	chr10	50833526/A//T	nonsynonymous	0	1
KAT6B	chr10	76737145/A//G	nonsynonymous	0	1
NFKB2	chr10	104161574/GCAGCTGGGC//G	nonsynonymous	0	1
OR9G1	chr11	56468052/A//G	synonymous	0	1
UCP2	chr11	73688956/T//G	nonsynonymous	0	1
C2CD2L	chr11	118984801/T//A	synonymous	0	1
GRIN2B	chr12	13761695/C//T	nonsynonymous	0	1
ATF7IP	chr12	14589061/G//A	nonsynonymous	0	1
PLCZ1	chr12	18837150/A//G	nonsynonymous	0	1
ADAMTS20	chr12	43847704/A//T	synonymous	0	1
KMT2D	chr12	49435753/G//A	nonsynonymous	0	1
COQ10A	chr12	56663938/C//A	nonsynonymous	0	1
OTOGL	chr12	80714486/T//C	synonymous	0	1
CHST11	chr12	105150981/C//T	synonymous	0	1
CCDC60	chr12	119943079/C//A	nonsynonymous	0	1
PARP4	chr13	25033238/C//G	nonsynonymous	0	1
FBXL3	chr13	77581170/T//C	synonymous	0	1
NOVA1	chr14	27064662/T//A	nonsynonymous	0	1
SEC23A	chr14	39530981/G//C	nonsynonymous	0	1
EML5	chr14	89083211/A//G	synonymous	0	1
CDC42BPB	chr14	103410716/G//A	nonsynonymous	0	1
EIF5	chr14	103807418/A//G	synonymous	0	1
DMXL2	chr15	51830621/T//G	synonymous	0	1
TIPIN	chr15	66633482/A//C	synonymous	0	1
IQGAP1	chr15	91021124/A//G	synonymous	0	1
FURIN	chr15	91422015/A//G	nonsynonymous	0	1
ADAMTS17	chr15	100516258/G//A	nonsynonymous	0	1
TIGD7	chr16	3349433/G//A	synonymous	0	1
USP7	chr16	9004690/AAG//A	synonymous	0	1
GRIN2A	chr16	10032093/G//T	nonsynonymous	0	1
MYH11	chr16	15853436/A//G	synonymous	0	1
BCKDK	chr16	31123189/G//C	nonsynonymous	0	1
PRSS36	chr16	31161045/A//G	synonymous	0	1
C16orf58	chr16	31505058/T//C	nonsynonymous	0	1
ZNF469	chr16	88498853/C//A	nonsynonymous	0	1
RPH3AL	chr17	171126/T//C	nonsynonymous	0	1
NLRP1	chr17	5436704/T//C	nonsynonymous	0	1
C17orf74	chr17	7330663/T//A	nonsynonymous	0	1
MYH4	chr17	10350384/C//T	nonsynonymous	0	1
MYH4	chr17	10357022/T//C	nonsynonymous	0	1
MYH3	chr17	10535021/C//T	synonymous	0	1
DNAH9	chr17	11572723/C//T	nonsynonymous	0	1
SPECC1	chr17	20156825/C//T	nonsynonymous	0	1
ASIC2	chr17	32483621/G//T	synonymous	0	1

CDC6	chr17	38450313/G//C	synonymous	0	1
P3H4	chr17	39964258/T//A	nonsynonymous	0	1
VPS25	chr17	40925500/A//T	nonsynonymous	0	1
PHOSPHO1	chr17	47304096/G//A	synonymous	0	1
NXPH3	chr17	47656797/G//A	synonymous	0	1
ABCA5	chr17	67243654/G//A	synonymous	0	1
FBF1	chr17	73919384/T//C	nonsynonymous	0	1
NAPG	chr18	10526051/G//T	synonymous	0	1
PIAS2	chr18	44435378/C//A	nonsynonymous	0	1
ELAC1	chr18	48510938/G//A	synonymous	0	1
CNDP1	chr18	72201888/C//G	synonymous	0	1
JSRP1	chr19	2252324/C//T	synonymous	0	1
MRPL54	chr19	3762705/A//T	nonsynonymous	0	1
DPP9	chr19	4685768/T//A	nonsynonymous	0	1
MUC16	chr19	9089611/T//C	nonsynonymous	0	1
OR10H1	chr19	15918690/C//T	nonsynonymous	0	1
OR10H4	chr19	16060441/C//T	synonymous	0	1
GDF15	chr19	18499281/C//T	synonymous	0	1
CEBPA	chr19	33792218/C//A	synonymous	0	1
ZNF529	chr19	37038705/T//G	nonsynonymous	0	1
BCL3	chr19	45260921/T//C	nonsynonymous	0	1
RSPH6A	chr19	46318361/T//C	nonsynonymous	0	1
SAE1	chr19	47673111/G//A	nonsynonymous	0	1
ZNF444	chr19	56671207/C//T	synonymous	0	1
PLCB1	chr20	8630101/G//A	synonymous	0	1
SYNDIG1	chr20	24524124/G//A	nonsynonymous	0	1
UQCC1	chr20	33981915/C//A	nonsynonymous	0	1
	chr20	35090953/T//C	synonymous	0	1
	chr20	50714973/T//C	synonymous	0	1
VAPB	chr20	57015987/A//G	nonsynonymous	0	1
NPEPL1	chr20	57289713/C//T	nonsynonymous	0	1
HRH3	chr20	60791008/C//T	synonymous	0	1
COL20A1	chr20	61945231/A//G	synonymous	0	1
ADAMTS5	chr21	28338228/C//T	synonymous	0	1
CLDN8	chr21	31587732/G//C	nonsynonymous	0	1
KRTAP11-1	chr21	32253305/A//T	synonymous	0	1
HUNK	chr21	33346963/C//T	synonymous	0	1
PRR5-ARHGAP8	chr22	45110529/T//A	nonsynonymous	0	1
SHOX	chrX	591551/C//A	synonymous	0	1
GPR143	chrX	9711599/G//C	synonymous	0	1
MAGEB3	chrX	30254415/T//C	nonsynonymous	0	1
ERCC6L	chrX	71426614/A//G	nonsynonymous	0	1
NAP1L3	chrX	92928038/C//T	nonsynonymous	0	1
ACAP3	chr1	1235954/C//A	nonsynonymous	1	0
MIB2	chr1	1565891/G//T	nonsynonymous	1	0
GNB1	chr1	1756945/C//A	synonymous	1	0
GABRD	chr1	1961430/G//T	synonymous	1	0
	chr1	2129512/T//C	synonymous	1	0
CCDC27	chr1	3669346/C//G	nonsynonymous	1	0
CHD5	chr1	6185207/G//A	synonymous	1	0
CHD5	chr1	6211151/C//A	nonsynonymous	1	0
TAS1R1	chr1	6631169/G//T	nonsynonymous	1	0
CAMTA1	chr1	7721898/C//A	synonymous	1	0
CAMTA1	chr1	7725013/G//T	synonymous	1	0
ENO1	chr1	8926507/C//G	nonsynonymous	1	0
SLC2A5	chr1	9107753/G//C	nonsynonymous	1	0
KIF1B	chr1	10327445/A//G	nonsynonymous	1	0
MTOR	chr1	11205085/C//A	nonsynonymous	1	0
UBIAD1	chr1	11346189/G//T	synonymous	1	0
VPS13D	chr1	12439628/G//T	nonsynonymous	1	0
VPS13D	chr1	12446352/G//A	nonsynonymous	1	0
PRAMEF2	chr1	12920015/A//G	nonsynonymous	1	0
LRRC38	chr1	13839697/A//T	nonsynonymous	1	0
CTRC	chr1	15766827/C//A	synonymous	1	0
SPEN	chr1	16259386/G//T	synonymous	1	0
MFAP2	chr1	17302155/G//T	nonsynonymous	1	0
ACTL8	chr1	18153030/C//G	synonymous	1	0
TAS1R2	chr1	19181103/C//A	nonsynonymous	1	0
UBR4	chr1	19420517/C//A	synonymous	1	0
EMC1	chr1	19571414/C//A	nonsynonymous	1	0

AKR7A2	chr1	19633494/A//T	nonsynonymous	1	0
KIF17	chr1	21043961/G//T	synonymous	1	0
ECE1	chr1	21605735/C//T	nonsynonymous	1	0
USP48	chr1	22063126/G//C	synonymous	1	0
HSPG2	chr1	22204947/C//A	nonsynonymous	1	0
CELA3B	chr1	22310253/A//T	synonymous	1	0
MYOM3	chr1	24419500/A//T	nonsynonymous	1	0
GRHL3	chr1	24663166/A//T	nonsynonymous	1	0
GRHL3	chr1	24663628/C//A	nonsynonymous	1	0
GRHL3	chr1	24664207/C//T	synonymous	1	0
GPR3	chr1	27720645/G//T	nonsynonymous	1	0
THEMIS2	chr1	28209364/T//G	nonsynonymous	1	0
EYA3	chr1	28362171/G//A	nonsynonymous	1	0
GMEB1	chr1	29040740/C//T	nonsynonymous	1	0
MATN1	chr1	31194355/G//T	nonsynonymous	1	0
TMEM39B	chr1	32557504/TGAGGTG//T	nonsynonymous	1	0
CSMD2	chr1	34049336/A//T	synonymous	1	0
C1orf94	chr1	34666433/G//C	nonsynonymous	1	0
GJB5	chr1	35223627/C//A	nonsynonymous	1	0
DLGAP3	chr1	35365290/G//T	nonsynonymous	1	0
C1orf216	chr1	36181544/G//A	nonsynonymous	1	0
CLSPN	chr1	36209102/C//G	nonsynonymous	1	0
MFSD2A	chr1	40432276/A//T	nonsynonymous	1	0
RLF	chr1	40702002/C//T	nonsynonymous	1	0
SMAP2	chr1	40874407/A//T	nonsynonymous	1	0
HIVEP3	chr1	42048579/T//C	synonymous	1	0
ZNF691	chr1	43317307/C//T	synonymous	1	0
SZT2	chr1	43908593/G//T	nonsynonymous	1	0
DPH2	chr1	44436798/C//A	nonsynonymous	1	0
SLC6A9	chr1	44468176/C//A	synonymous	1	0
KIF2C	chr1	45223241/C//A	nonsynonymous	1	0
PTCH2	chr1	45293282/G//C	synonymous	1	0
EIF2B3	chr1	45345608/C//A	nonsynonymous	1	0
TESK2	chr1	45887468/C//A	nonsynonymous	1	0
NASP	chr1	46083822/A//G	synonymous	1	0
CCDC17	chr1	46088944/C//T	synonymous	1	0
CYP4B1	chr1	47282856/G//T	synonymous	1	0
AGBL4	chr1	49052792/C//T	nonsynonymous	1	0
KTI12	chr1	52499142/C//A	nonsynonymous	1	0
ZCCHC11	chr1	52962674/C//G	synonymous	1	0
ZYG11B	chr1	53287133/G//A	synonymous	1	0
SCP2	chr1	53440475/G//T	nonsynonymous	1	0
ACOT11	chr1	55070840/T//A	nonsynonymous	1	0
MROH7	chr1	55139712/G//T	synonymous	1	0
TMEM61	chr1	55457763/G//T	nonsynonymous	1	0
C1orf168	chr1	57209866/C//A	synonymous	1	0
DAB1	chr1	57476839/G//C	nonsynonymous	1	0
FGGY	chr1	60228215/G//C	nonsynonymous	1	0
HOOK1	chr1	60309173/C//T	nonsynonymous	1	0
ANGPTL3	chr1	63066810/G//A	nonsynonymous	1	0
DNAJC6	chr1	65855309/A//T	nonsynonymous	1	0
DNAJC6	chr1	65858430/G//T	nonsynonymous	1	0
DNAJC6	chr1	65860720/G//T	synonymous	1	0
PDE4B	chr1	66829252/G//T	nonsynonymous	1	0
RPE65	chr1	68895518/G//T	synonymous	1	0
PTGER3	chr1	71513147/C//A	synonymous	1	0
NEGR1	chr1	72163767/C//A	nonsynonymous	1	0
FPGT-TNNI3K	chr1	74834924/G//C	nonsynonymous	1	0
ST6GALNAC3	chr1	76877795/A//G	nonsynonymous	1	0
ZZZ3	chr1	78098988/C//A	nonsynonymous	1	0
FUBP1	chr1	78430900/T//G	nonsynonymous	1	0
ADGRL4	chr1	79470755/C//A	nonsynonymous	1	0
ADGRL2	chr1	82450383/C//T	synonymous	1	0
SSX2IP	chr1	85116068/T//C	synonymous	1	0
CLCA2	chr1	86890045/G//A	nonsynonymous	1	0
HS2ST1	chr1	87569200/G//T	nonsynonymous	1	0
PKN2	chr1	89236050/G//C	nonsynonymous	1	0
GBP4	chr1	89658756/G//A	synonymous	1	0
GBP5	chr1	89726344/C//T	synonymous	1	0
LRRC8D	chr1	90399906/G//C	nonsynonymous	1	0

GLMN	chr1	92713454/T//A	synonymous	1	0
MTF2	chr1	93594878/G//T	nonsynonymous	1	0
ARHGAP29	chr1	94674893/T//C	synonymous	1	0
SASS6	chr1	100584640/T//A	synonymous	1	0
COL11A1	chr1	103400630/C//A	nonsynonymous	1	0
COL11A1	chr1	103469999/C//A	nonsynonymous	1	0
AMY2B	chr1	104116534/G//A	nonsynonymous	1	0
VAV3	chr1	108247209/C//T	synonymous	1	0
NBPF4	chr1	108771772/G//T	nonsynonymous	1	0
CLCC1	chr1	109484102/G//A	nonsynonymous	1	0
GNAT2	chr1	110148698/C//G	nonsynonymous	1	0
EPS8L3	chr1	110295795/G//A	synonymous	1	0
SLC6A17	chr1	110741173/C//G	synonymous	1	0
PIFO	chr1	111889272/G//T	synonymous	1	0
FAM19A3	chr1	113264921/G//T	nonsynonymous	1	0
PHTF1	chr1	114249314/G//T	nonsynonymous	1	0
HIPK1	chr1	114483634/G//T	nonsynonymous	1	0
SYT6	chr1	114680185/T//A	nonsynonymous	1	0
CSDE1	chr1	115261252/C//A	nonsynonymous	1	0
CSDE1	chr1	115276439/C//A	nonsynonymous	1	0
CD101	chr1	117576700/G//A	nonsynonymous	1	0
TTF2	chr1	117617759/A//T	nonsynonymous	1	0
MAN1A2	chr1	118003134/G//A	nonsynonymous	1	0
WDR3	chr1	118496689/A//T	nonsynonymous	1	0
SPAG17	chr1	118642234/G//T	nonsynonymous	1	0
TBX15	chr1	119474381/C//G	synonymous	1	0
NBPF8	chr1	144618162/C//A	nonsynonymous	1	0
NBPF8	chr1	144828545/A//T	nonsynonymous	1	0
ANKRD34A	chr1	145473202/C//G	synonymous	1	0
PIAS3	chr1	145578475/C//A	synonymous	1	0
RNF115	chr1	145611213/C//G	synonymous	1	0
ENSA	chr1	150601985/G//A	synonymous	1	0
CTSS	chr1	150724348/C//T	nonsynonymous	1	0
SEMA6C	chr1	151115009/G//T	nonsynonymous	1	0
SNX27	chr1	151664947/G//C	nonsynonymous	1	0
TCHHL1	chr1	152057729/A//G	nonsynonymous	1	0
HRNR	chr1	152191725/G//T	nonsynonymous	1	0
FLG	chr1	152282080/C//G	nonsynonymous	1	0
FLG2	chr1	152326924/G//A	nonsynonymous	1	0
LCE3A	chr1	152595410/G//A	nonsynonymous	1	0
LCE2D	chr1	152636684/C//T	nonsynonymous	1	0
LELP1	chr1	153177226/G//T	nonsynonymous	1	0
	chr1	154131413/T//A	synonymous	1	0
CHRNB2	chr1	154544067/C//T	synonymous	1	0
KCNN3	chr1	154744636/G//A	synonymous	1	0
ASH1L	chr1	155429674/G//A	nonsynonymous	1	0
ASH1L	chr1	155449717/T//C	nonsynonymous	1	0
RIT1	chr1	155874548/G//C	nonsynonymous	1	0
IQGAP3	chr1	156498822/T//A	nonsynonymous	1	0
TTC24	chr1	156551243/G//T	nonsynonymous	1	0
TTC24	chr1	156551789/G//A	synonymous	1	0
TTC24	chr1	156554738/G//A	nonsynonymous	1	0
GPATCH4	chr1	156571225/G//A	nonsynonymous	1	0
PEAR1	chr1	156877988/A//T	nonsynonymous	1	0
PEAR1	chr1	156883668/G//T	nonsynonymous	1	0
FCRL5	chr1	157491068/G//C	nonsynonymous	1	0
FCRL2	chr1	157737060/C//A	nonsynonymous	1	0
FCRL1	chr1	157772370/G//A	nonsynonymous	1	0
CD1B	chr1	158299284/C//T	synonymous	1	0
CD1B	chr1	158300699/G//T	nonsynonymous	1	0
OR6P1	chr1	158532692/G//T	nonsynonymous	1	0
SPTA1	chr1	158624446/G//T	synonymous	1	0
SPTA1	chr1	158631132/G//A	synonymous	1	0
OR6K6	chr1	158725482/T//A	nonsynonymous	1	0
PYHIN1	chr1	158912034/G//T	nonsynonymous	1	0
AIM2	chr1	159035962/T//A	nonsynonymous	1	0
FCRL6	chr1	159778812/G//T	synonymous	1	0
KCNJ9	chr1	160054422/T//C	nonsynonymous	1	0
ATP1A4	chr1	160136392/G//T	synonymous	1	0
COPA	chr1	160305052/G//A	nonsynonymous	1	0

NCSTN	chr1	160324041/G//C	nonsynonymous	1	0
USP21	chr1	161132433/G//T	nonsynonymous	1	0
ADAMTS4	chr1	161163802/C//A	nonsynonymous	1	0
FCGR2B	chr1	161647135/G//A	nonsynonymous	1	0
OLFML2B	chr1	161967651/T//A	nonsynonymous	1	0
DDR2	chr1	162737130/G//T	nonsynonymous	1	0
CCDC190	chr1	162825067/G//T	nonsynonymous	1	0
RGS4	chr1	163044229/G//T	nonsynonymous	1	0
DUSP27	chr1	167095301/C//A	synonymous	1	0
SELP	chr1	169578823/C//T	nonsynonymous	1	0
	chr1	170010892/G//A	synonymous	1	0
PRRX1	chr1	170689014/G//T	nonsynonymous	1	0
	chr1	171227186/G//C	synonymous	1	0
FMO4	chr1	171303628/G//T	synonymous	1	0
RC3H1	chr1	173931097/G//A	synonymous	1	0
TNN	chr1	175052996/C//A	nonsynonymous	1	0
TNR	chr1	175362962/G//T	nonsynonymous	1	0
PAPPA2	chr1	176668287/C//A	nonsynonymous	1	0
BRINP2	chr1	177199214/G//T	nonsynonymous	1	0
SEC16B	chr1	177908869/G//C	nonsynonymous	1	0
	chr1	178490864/C//A	synonymous	1	0
	chr1	178490876/T//C	synonymous	1	0
ACBD6	chr1	180399365/T//A	synonymous	1	0
CACNA1E	chr1	181701874/G//T	synonymous	1	0
CACNA1E	chr1	181765872/C//T	nonsynonymous	1	0
HMCN1	chr1	186037077/C//T	nonsynonymous	1	0
HMCN1	chr1	186147581/G//C	nonsynonymous	1	0
HMCN1	chr1	186151308/G//A	nonsynonymous	1	0
RGS21	chr1	192316478/T//G	nonsynonymous	1	0
ASPM	chr1	197087109/C//A	nonsynonymous	1	0
CRB1	chr1	197390318/G//T	nonsynonymous	1	0
	chr1	197704924/C//T	synonymous	1	0
PTPRC	chr1	198721784/C//A	nonsynonymous	1	0
GPR25	chr1	200842836/G//C	nonsynonymous	1	0
CACNA1S	chr1	201042764/G//T	synonymous	1	0
IGFN1	chr1	201177070/G//A	nonsynonymous	1	0
IGFN1	chr1	201177909/G//A	synonymous	1	0
IGFN1	chr1	201195075/G//T	nonsynonymous	1	0
LGR6	chr1	202273741/G//T	synonymous	1	0
KDM5B	chr1	202710536/T//A	synonymous	1	0
SOX13	chr1	204092265/C//A	nonsynonymous	1	0
PIK3C2B	chr1	204438313/C//A	synonymous	1	0
NFASC	chr1	204966472/C//A	nonsynonymous	1	0
NFASC	chr1	204985616/C//A	nonsynonymous	1	0
IKBKE	chr1	206666663/C//T	nonsynonymous	1	0
	chr1	206730882/G//T	synonymous	1	0
PLXNA2	chr1	208215585/C//A	nonsynonymous	1	0
PLXNA2	chr1	208217876/C//T	nonsynonymous	1	0
HSD11B1	chr1	209907680/C//A	synonymous	1	0
KCNH1	chr1	210856884/C//G	synonymous	1	0
LPGAT1	chr1	211924390/C//A	nonsynonymous	1	0
PROX1	chr1	214170020/C//G	nonsynonymous	1	0
USH2A	chr1	216062137/C//A	nonsynonymous	1	0
EPRS	chr1	220180549/T//A	synonymous	1	0
RAB3GAP2	chr1	220356222/C//A	nonsynonymous	1	0
MARK1	chr1	220808778/G//T	nonsynonymous	1	0
MIA3	chr1	222802598/A//T	nonsynonymous	1	0
CNIH3	chr1	224922394/C//T	synonymous	1	0
CNIH3	chr1	224927090/G//A	synonymous	1	0
DNAH14	chr1	225239245/G//T	nonsynonymous	1	0
PYCR2	chr1	226109632/C//A	nonsynonymous	1	0
ZNF678	chr1	227834330/G//C	nonsynonymous	1	0
OBSCN	chr1	228412363/G//T	nonsynonymous	1	0
OBSCN	chr1	228469849/G//T	nonsynonymous	1	0
OBSCN	chr1	228525715/G//C	nonsynonymous	1	0
TRIM11	chr1	228582954/C//A	nonsynonymous	1	0
HIST3H3	chr1	228612704/G//T	nonsynonymous	1	0
MAP3K21	chr1	233514839/A//T	nonsynonymous	1	0
TARBP1	chr1	234603391/A//T	nonsynonymous	1	0
ACTN2	chr1	236911022/G//A	nonsynonymous	1	0

RYR2	chr1	237604687/C//G	nonsynonymous	1	0
RYR2	chr1	237656290/G//T	nonsynonymous	1	0
RYR2	chr1	237675019/G//T	nonsynonymous	1	0
RYR2	chr1	237693784/G//A	synonymous	1	0
RYR2	chr1	237729917/C//G	nonsynonymous	1	0
RYR2	chr1	237777432/G//T	synonymous	1	0
RYR2	chr1	237947123/C//A	synonymous	1	0
RYR2	chr1	237947478/A//T	nonsynonymous	1	0
RYR2	chr1	237948142/C//T	nonsynonymous	1	0
RYR2	chr1	237948251/T//A	synonymous	1	0
RYR2	chr1	237961330/C//A	synonymous	1	0
RYR2	chr1	237993857/A//T	nonsynonymous	1	0
ZP4	chr1	238048766/A//C	nonsynonymous	1	0
ZP4	chr1	238053955/G//C	synonymous	1	0
CHRM3	chr1	240070855/C//A	nonsynonymous	1	0
FMN2	chr1	240371908/C//A	nonsynonymous	1	0
FH	chr1	241665767/C//T	synonymous	1	0
WDR64	chr1	241932739/T//A	synonymous	1	0
KIF26B	chr1	245704135/C//A	synonymous	1	0
KIF26B	chr1	245850203/C//T	synonymous	1	0
ZNF669	chr1	247263875/A//T	nonsynonymous	1	0
VN1R5	chr1	247420311/C//A	nonsynonymous	1	0
OR2C3	chr1	247695143/C//A	nonsynonymous	1	0
OR2G2	chr1	247752220/C//G	nonsynonymous	1	0
OR2G2	chr1	247752232/G//T	nonsynonymous	1	0
OR2L8	chr1	248112472/G//T	nonsynonymous	1	0
OR2AK2	chr1	248129578/A//T	synonymous	1	0
OR2M5	chr1	248309236/A//T	nonsynonymous	1	0
OR2T12	chr1	248458616/C//A	nonsynonymous	1	0
OR14C36	chr1	248512343/C//A	synonymous	1	0
OR14C36	chr1	248512376/C//A	nonsynonymous	1	0
OR2T4	chr1	248525483/C//A	nonsynonymous	1	0
OR2T34	chr1	248737590/C//G	nonsynonymous	1	0
SH3BP5L	chr1	249107317/G//T	nonsynonymous	1	0
PXDN	chr2	1670260/T//A	nonsynonymous	1	0
	chr2	18768207/A//T	synonymous	1	0
APOB	chr2	21242715/C//A	nonsynonymous	1	0
APOB	chr2	21256307/G//C	nonsynonymous	1	0
WDCP	chr2	24262248/C//A	nonsynonymous	1	0
ADCY3	chr2	25064226/C//A	synonymous	1	0
OTOF	chr2	26693521/T//A	synonymous	1	0
PRR30	chr2	27360076/G//A	synonymous	1	0
IFT172	chr2	27706229/C//A	nonsynonymous	1	0
GCKR	chr2	27719811/C//A	nonsynonymous	1	0
GCKR	chr2	27722028/G//A	nonsynonymous	1	0
PLB1	chr2	28827543/G//T	nonsynonymous	1	0
PLB1	chr2	28865750/G//T	synonymous	1	0
VIT	chr2	37035650/C//A	nonsynonymous	1	0
DHX57	chr2	39088627/G//C	nonsynonymous	1	0
PRKCE	chr2	46412019/G//T	nonsynonymous	1	0
FSHR	chr2	49195848/C//A	nonsynonymous	1	0
FSHR	chr2	49216126/T//C	nonsynonymous	1	0
NRXN1	chr2	50149137/C//A	nonsynonymous	1	0
NRXN1	chr2	50280408/C//A	nonsynonymous	1	0
NRXN1	chr2	50724838/C//A	nonsynonymous	1	0
NRXN1	chr2	51255337/C//A	synonymous	1	0
MTIF2	chr2	55490790/G//C	nonsynonymous	1	0
CCDC88A	chr2	55562124/T//A	nonsynonymous	1	0
BCL11A	chr2	60687814/C//A	nonsynonymous	1	0
WDPCP	chr2	63631656/C//A	nonsynonymous	1	0
CD207	chr2	71058930/C//A	synonymous	1	0
ZNF638	chr2	71626697/G//T	nonsynonymous	1	0
EXOC6B	chr2	73053148/T//A	synonymous	1	0
WBP1	chr2	74685668/G//A	synonymous	1	0
REG3A	chr2	79385834/G//A	synonymous	1	0
CTNNA2	chr2	80101358/C//G	nonsynonymous	1	0
CTNNA2	chr2	80136775/T//A	nonsynonymous	1	0
DNAH6	chr2	84806759/G//A	nonsynonymous	1	0
DNAH6	chr2	84896602/G//T	nonsynonymous	1	0
EIF2AK3	chr2	88874870/T//C	nonsynonymous	1	0

ZNF514	chr2	95818951/C//A	synonymous	1	0
ZAP70	chr2	98354493/G//C	nonsynonymous	1	0
TMEM131	chr2	98378617/G//A	synonymous	1	0
CNGA3	chr2	99012521/C//G	nonsynonymous	1	0
AFF3	chr2	100210591/G//T	nonsynonymous	1	0
MAP4K4	chr2	102314998/A//T	nonsynonymous	1	0
MAP4K4	chr2	102315000/G//T	nonsynonymous	1	0
SLC9A4	chr2	103142737/G//C	nonsynonymous	1	0
SLC9A2	chr2	103281559/G//T	nonsynonymous	1	0
SULT1C4	chr2	109003886/T//C	nonsynonymous	1	0
RANBP2	chr2	109336035/G//A	synonymous	1	0
RANBP2	chr2	109336049/A//C	synonymous	1	0
RANBP2	chr2	109398836/T//C	nonsynonymous	1	0
SEPT10	chr2	110322096/T//A	nonsynonymous	1	0
FBLN7	chr2	112917415/G//T	synonymous	1	0
PSD4	chr2	113940630/G//T	synonymous	1	0
PSD4	chr2	113940977/A//G	nonsynonymous	1	0
CFAP221	chr2	120362858/C//T	nonsynonymous	1	0
CNTNAP5	chr2	125204369/C//T	nonsynonymous	1	0
CNTNAP5	chr2	125547491/C//A	nonsynonymous	1	0
MYO7B	chr2	128363480/T//C	nonsynonymous	1	0
GPR17	chr2	128408489/C//T	synonymous	1	0
CCDC74B	chr2	130897848/C//A	nonsynonymous	1	0
TUBA3E	chr2	130953849/A//T	nonsynonymous	1	0
PTPN18	chr2	131130495/G//T	synonymous	1	0
POTEE	chr2	132021417/G//T	nonsynonymous	1	0
TUBA3D	chr2	132236893/C//T	nonsynonymous	1	0
LCT	chr2	136567525/C//T	nonsynonymous	1	0
LCT	chr2	136570302/G//C	synonymous	1	0
LRP1B	chr2	141135751/C//T	nonsynonymous	1	0
LRP1B	chr2	141459401/C//T	nonsynonymous	1	0
LRP1B	chr2	141739744/C//T	nonsynonymous	1	0
LRP1B	chr2	141739804/G//T	nonsynonymous	1	0
ARHGAP15	chr2	144381777/G//A	nonsynonymous	1	0
KIF5C	chr2	149847569/C//T	nonsynonymous	1	0
NEB	chr2	152550854/C//G	nonsynonymous	1	0
FMNL2	chr2	153484896/G//A	nonsynonymous	1	0
GPD2	chr2	157426595/C//G	synonymous	1	0
CYTIP	chr2	158272636/G//A	synonymous	1	0
BAZ2B	chr2	160287388/G//C	nonsynonymous	1	0
LY75-CD302	chr2	160729091/T//C	nonsynonymous	1	0
TBR1	chr2	162280791/C//T	synonymous	1	0
SCN3A	chr2	165954039/A//T	synonymous	1	0
SCN3A	chr2	165994388/C//A	nonsynonymous	1	0
SCN1A	chr2	166848612/C//A	nonsynonymous	1	0
SCN1A	chr2	166892973/T//A	nonsynonymous	1	0
XIRP2	chr2	168099648/G//C	nonsynonymous	1	0
XIRP2	chr2	168100492/C//A	nonsynonymous	1	0
XIRP2	chr2	168106654/C//A	nonsynonymous	1	0
LRP2	chr2	170026237/C//A	nonsynonymous	1	0
LRP2	chr2	170134384/C//T	nonsynonymous	1	0
LRP2	chr2	170136881/C//T	nonsynonymous	1	0
BBS5	chr2	170350326/A//T	nonsynonymous	1	0
ATF2	chr2	175957885/T//G	nonsynonymous	1	0
HOXD9	chr2	176988014/C//A	nonsynonymous	1	0
HOXD8	chr2	176995172/C//A	synonymous	1	0
AGPS	chr2	178326618/C//T	synonymous	1	0
TTN	chr2	179422009/G//A	nonsynonymous	1	0
TTN	chr2	179434772/G//T	nonsynonymous	1	0
TTN	chr2	179454730/C//T	synonymous	1	0
TTN	chr2	179456539/G//C	nonsynonymous	1	0
TTN	chr2	179472135/T//G	nonsynonymous	1	0
TTN	chr2	179485016/G//T	nonsynonymous	1	0
TTN	chr2	179543147/C//A	nonsynonymous	1	0
TTN	chr2	179599501/C//A	synonymous	1	0
TTN	chr2	179605455/G//C	nonsynonymous	1	0
	chr2	179621125/C//T	synonymous	1	0
TTN	chr2	179638098/T//A	nonsynonymous	1	0
TTN	chr2	179640203/C//A	nonsynonymous	1	0
TTN	chr2	179659840/C//A	nonsynonymous	1	0

CCDC141	chr2	179825969/T//A	nonsynonymous	1	0
CWC22	chr2	180842970/G//A	synonymous	1	0
SSFA2	chr2	182792942/G//T	nonsynonymous	1	0
NCKAP1	chr2	183817611/T//TA	nonsynonymous	1	0
DUSP19	chr2	183943498/G//T	synonymous	1	0
FSIP2	chr2	186658414/G//T	nonsynonymous	1	0
FSIP2	chr2	186664547/C//A	nonsynonymous	1	0
FSIP2	chr2	186668269/G//T	nonsynonymous	1	0
MSTN	chr2	190922180/C//A	nonsynonymous	1	0
DNAH7	chr2	196698943/C//T	synonymous	1	0
CCDC150	chr2	197597303/G//T	synonymous	1	0
ANKRD44	chr2	197990648/C//A	synonymous	1	0
HSPE1-MOB4	chr2	198415298/C//T	synonymous	1	0
ALS2CR11	chr2	202483856/C//G	synonymous	1	0
MPP4	chr2	202545704/G//T	synonymous	1	0
ICOS	chr2	204821386/A//T	synonymous	1	0
ZDBF2	chr2	207171663/A//C	nonsynonymous	1	0
ZDBF2	chr2	207174438/G//C	nonsynonymous	1	0
ZDBF2	chr2	207175173/G//A	nonsynonymous	1	0
LOC200726	chr2	207509373/G//T	nonsynonymous	1	0
LOC200726	chr2	207513517/A//G	synonymous	1	0
UNC80	chr2	210658544/C//A	nonsynonymous	1	0
UNC80	chr2	210704155/G//T	nonsynonymous	1	0
UNC80	chr2	210786393/C//G	nonsynonymous	1	0
UNC80	chr2	210858989/G//T	nonsynonymous	1	0
ERBB4	chr2	212251773/C//T	nonsynonymous	1	0
IKZF2	chr2	213921652/C//T	nonsynonymous	1	0
ATIC	chr2	216209563/C//T	nonsynonymous	1	0
RPL37A	chr2	217363590/G//A	synonymous	1	0
CXCR1	chr2	219029453/C//A	nonsynonymous	1	0
CFAP65	chr2	219888102/G//C	nonsynonymous	1	0
DES	chr2	220285065/A//G	synonymous	1	0
SPEG	chr2	220348516/G//A	nonsynonymous	1	0
OBSL1	chr2	220423174/T//A	synonymous	1	0
SLC4A3	chr2	220494890/G//T	synonymous	1	0
CCDC140	chr2	223168989/C//T	nonsynonymous	1	0
SCG2	chr2	224462948/C//A	synonymous	1	0
DOCK10	chr2	225652109/G//T	synonymous	1	0
IRS1	chr2	227660616/C//G	nonsynonymous	1	0
COL4A4	chr2	227907880/C//G	nonsynonymous	1	0
SPHKAP	chr2	228855821/C//G	nonsynonymous	1	0
ECEL1	chr2	233349510/C//A	nonsynonymous	1	0
NGEF	chr2	233785032/G//T	nonsynonymous	1	0
INPP5D	chr2	233925260/G//T	nonsynonymous	1	0
DGKD	chr2	234350579/G//T	nonsynonymous	1	0
UGT1A7	chr2	234591296/C//G	nonsynonymous	1	0
SH3BP4	chr2	235950841/G//T	synonymous	1	0
COL6A3	chr2	238233359/A//T	synonymous	1	0
COL6A3	chr2	238249726/C//A	synonymous	1	0
COL6A3	chr2	238253060/G//A	nonsynonymous	1	0
COL6A3	chr2	238280890/C//T	nonsynonymous	1	0
LRRFIP1	chr2	238671955/A//T	synonymous	1	0
PER2	chr2	239161811/G//T	nonsynonymous	1	0
RNPEPL1	chr2	241517023/G//A	nonsynonymous	1	0
SNED1	chr2	242011244/G//C	synonymous	1	0
MTERF4	chr2	242039282/G//A	nonsynonymous	1	0
CHL1	chr3	386326/G//T	nonsynonymous	1	0
CNTN4	chr3	3095505/G//A	nonsynonymous	1	0
ITPR1	chr3	4716792/A//G	nonsynonymous	1	0
SSUH2	chr3	8672503/C//G	synonymous	1	0
ATP2B2	chr3	10491043/G//T	nonsynonymous	1	0
SLC6A11	chr3	10857933/G//C	synonymous	1	0
SLC6A11	chr3	10861150/G//T	nonsynonymous	1	0
TAMM41	chr3	11885583/C//A	nonsynonymous	1	0
CAND2	chr3	12867122/G//T	nonsynonymous	1	0
FGD5	chr3	14860882/C//A	nonsynonymous	1	0
FGD5	chr3	14861255/C//G	nonsynonymous	1	0
METTL6	chr3	15466582/C//T	synonymous	1	0
COLQ	chr3	15520491/C//A	nonsynonymous	1	0
	chr3	15643492/G//T	synonymous	1	0

DAZL	chr3	16646786/C//A	synonymous	1	0
SUSD5	chr3	33195325/C//A	nonsynonymous	1	0
CLASP2	chr3	33592748/C//T	nonsynonymous	1	0
PDCD6IP	chr3	33883490/G//T	nonsynonymous	1	0
ACAA1	chr3	38168047/C//A	synonymous	1	0
SCN10A	chr3	38802813/G//T	synonymous	1	0
SCN11A	chr3	38926791/ATC//A	nonsynonymous	1	0
MYRIP	chr3	40085571/C//A	synonymous	1	0
ULK4	chr3	41795921/T//A	nonsynonymous	1	0
LRRC2	chr3	46571471/C//A	nonsynonymous	1	0
SMARCC1	chr3	47702844/T//A	nonsynonymous	1	0
CCDC51	chr3	48475289/G//A	synonymous	1	0
COL7A1	chr3	48615930/C//A	nonsynonymous	1	0
RNF123	chr3	49749985/C//A	nonsynonymous	1	0
RAD54L2	chr3	51624396/G//T	synonymous	1	0
GRM2	chr3	51749607/C//G	nonsynonymous	1	0
NEK4	chr3	52802349/A//T	synonymous	1	0
ITIH1	chr3	52824883/G//T	nonsynonymous	1	0
ITIH1	chr3	52825822/G//T	nonsynonymous	1	0
MUSTN1	chr3	52868957/T//A	nonsynonymous	1	0
CACNA2D3	chr3	54537635/C//A	nonsynonymous	1	0
LRTM1	chr3	54959232/G//A	synonymous	1	0
DNAH12	chr3	57487094/A//G	nonsynonymous	1	0
FLNB	chr3	58134498/C//T	nonsynonymous	1	0
DNASE1L3	chr3	58186833/A//G	nonsynonymous	1	0
CADPS	chr3	62860600/G//A	synonymous	1	0
SLC25A26	chr3	66271483/C//T	synonymous	1	0
TMF1	chr3	69084199/C//G	nonsynonymous	1	0
MITF	chr3	70014224/A//G	nonsynonymous	1	0
FRG2C	chr3	75714737/G//T	nonsynonymous	1	0
FRG2C	chr3	75714921/A//T	nonsynonymous	1	0
OR5H6	chr3	97983911/C//A	synonymous	1	0
NIT2	chr3	100057971/C//T	synonymous	1	0
IMPG2	chr3	100951835/C//A	nonsynonymous	1	0
ZPLD1	chr3	102175170/G//T	nonsynonymous	1	0
ALCAM	chr3	105243264/T//C	synonymous	1	0
IFT57	chr3	107910491/C//A	nonsynonymous	1	0
KIAA1524	chr3	108308120/C//A	nonsynonymous	1	0
TAGLN3	chr3	111732275/G//C	synonymous	1	0
GOLGB1	chr3	121414978/C//A	synonymous	1	0
PARP14	chr3	122420046/G//T	nonsynonymous	1	0
CCDC14	chr3	123680121/A//T	nonsynonymous	1	0
ROPN1B	chr3	125702118/C//A	synonymous	1	0
ZXDC	chr3	126193978/C//A	nonsynonymous	1	0
PLXNA1	chr3	126737242/C//T	synonymous	1	0
COL6A5	chr3	130110136/C//A	nonsynonymous	1	0
SLCO2A1	chr3	133672604/G//T	synonymous	1	0
IL20RB	chr3	136701157/G//A	nonsynonymous	1	0
DZIP1L	chr3	137822709/C//A	nonsynonymous	1	0
PIK3CB	chr3	138384018/T//A	synonymous	1	0
PIK3CB	chr3	138474626/C//T	nonsynonymous	1	0
PRR23B	chr3	138739506/C//T	synonymous	1	0
RBP1	chr3	139258431/C//A	nonsynonymous	1	0
CLSTN2	chr3	140178590/C//A	nonsynonymous	1	0
TRIM42	chr3	140401930/C//T	nonsynonymous	1	0
SPSB4	chr3	140866094/T//C	nonsynonymous	1	0
GRK7	chr3	141499551/C//A	nonsynonymous	1	0
XRN1	chr3	142074287/C//A	nonsynonymous	1	0
ZIC4	chr3	147106540/G//T	synonymous	1	0
P2RY12	chr3	151055703/T//A	nonsynonymous	1	0
RAP2B	chr3	152880959/G//T	nonsynonymous	1	0
GPR149	chr3	154145477/G//A	synonymous	1	0
PLCH1	chr3	155212309/G//A	synonymous	1	0
PLCH1	chr3	155232542/C//A	synonymous	1	0
IQCJ-SCHIP1	chr3	159606728/A//G	nonsynonymous	1	0
OTOL1	chr3	161214841/C//A	synonymous	1	0
SI	chr3	164714319/A//G	synonymous	1	0
SI	chr3	164716422/A//C	nonsynonymous	1	0
BCHE	chr3	165548222/C//T	synonymous	1	0
GOLIM4	chr3	167747760/T//C	nonsynonymous	1	0

MECOM	chr3	168807961/C//A	synonymous	1	0
SLC7A14	chr3	170218997/C//A	nonsynonymous	1	0
MCCC1	chr3	182804545/G//A	nonsynonymous	1	0
LAMP3	chr3	182872022/C//A	nonsynonymous	1	0
ABCC5	chr3	183660561/C//A	synonymous	1	0
DVL3	chr3	183883999/G//C	nonsynonymous	1	0
	chr3	183994326/G//C	synonymous	1	0
ST6GAL1	chr3	186790693/A//G	synonymous	1	0
MB21D2	chr3	192517385/A//C	nonsynonymous	1	0
ATP13A5	chr3	193029694/G//A	nonsynonymous	1	0
HES1	chr3	193854438/A//T	nonsynonymous	1	0
LRRC15	chr3	194080213/G//T	synonymous	1	0
SENP5	chr3	196612853/G//T	nonsynonymous	1	0
MELTF	chr3	196743106/G//T	synonymous	1	0
NAT8L	chr4	2065684/G//T	nonsynonymous	1	0
CFAP99	chr4	2453039/G//T	nonsynonymous	1	0
FAM193A	chr4	2691244/A//T	synonymous	1	0
NOP14	chr4	2964897/G//A	synonymous	1	0
NOP14	chr4	2965026/G//T	synonymous	1	0
NOP14	chr4	2965132/C//T	synonymous	1	0
HGFAC	chr4	3444590/C//T	synonymous	1	0
LRPAP1	chr4	3534108/C//A	nonsynonymous	1	0
MSX1	chr4	4861766/A//T	nonsynonymous	1	0
CRMP1	chr4	5862929/C//A	nonsynonymous	1	0
MAN2B2	chr4	6599017/G//T	nonsynonymous	1	0
BLOC1S4	chr4	6717914/G//T	synonymous	1	0
ACOX3	chr4	8394057/T//A	synonymous	1	0
SLC2A9	chr4	9889266/C//A	nonsynonymous	1	0
ZNF518B	chr4	10446725/C//G	nonsynonymous	1	0
CD38	chr4	15826640/G//A	nonsynonymous	1	0
PROM1	chr4	15982144/C//A	nonsynonymous	1	0
LDB2	chr4	16504200/G//T	synonymous	1	0
QDPR	chr4	17506086/C//A	nonsynonymous	1	0
FAM184B	chr4	17643820/G//T	nonsynonymous	1	0
ADGRA3	chr4	22390729/C//T	nonsynonymous	1	0
PPARGC1A	chr4	23831154/T//A	nonsynonymous	1	0
SEL1L3	chr4	25834710/A//G	synonymous	1	0
PCDH7	chr4	30724855/G//T	nonsynonymous	1	0
ARAP2	chr4	36150059/C//T	nonsynonymous	1	0
LIMCH1	chr4	41621280/G//T	nonsynonymous	1	0
YIPF7	chr4	44626637/C//A	nonsynonymous	1	0
GABRA4	chr4	46930534/G//T	nonsynonymous	1	0
GABRB1	chr4	47322206/G//T	nonsynonymous	1	0
KIT	chr4	55592184/A//C	nonsynonymous	1	0
SRD5A3	chr4	56230328/G//C	nonsynonymous	1	0
KIAA1211	chr4	57180640/G//C	nonsynonymous	1	0
KIAA1211	chr4	57181920/T//C	nonsynonymous	1	0
THEGL	chr4	57462760/G//T	nonsynonymous	1	0
NOA1	chr4	57843708/A//C	nonsynonymous	1	0
ADGRL3	chr4	62812631/G//T	nonsynonymous	1	0
ADGRL3	chr4	62936098/C//A	synonymous	1	0
ADGRL3	chr4	62936494/C//G	nonsynonymous	1	0
EPHA5	chr4	66217196/A//T	nonsynonymous	1	0
TMPRSS11B	chr4	69097015/A//G	nonsynonymous	1	0
UGT2A2	chr4	70465048/C//A	nonsynonymous	1	0
UGT2A2	chr4	70505209/T//A	nonsynonymous	1	0
CABS1	chr4	71201849/G//T	nonsynonymous	1	0
SMR3A	chr4	71232454/G//T	nonsynonymous	1	0
AMTN	chr4	71389580/T//G	synonymous	1	0
SLC4A4	chr4	72363337/C//A	synonymous	1	0
ANKRD17	chr4	74021406/G//C	synonymous	1	0
AFM	chr4	74363415/A//C	nonsynonymous	1	0
CXCL6	chr4	74703392/C//A	synonymous	1	0
FRAS1	chr4	79188412/G//T	synonymous	1	0
FRAS1	chr4	79188517/G//A	synonymous	1	0
PRKG2	chr4	82095997/C//G	nonsynonymous	1	0
HELQ	chr4	84358040/T//A	synonymous	1	0
HELQ	chr4	84362564/T//A	synonymous	1	0
GPAT3	chr4	84519220/G//C	nonsynonymous	1	0
WDFY3	chr4	85638147/T//A	nonsynonymous	1	0

IBSP	chr4	88732694/G//T	nonsynonymous	1	0
HERC3	chr4	89583578/C//A	synonymous	1	0
MMRN1	chr4	90816122/G//T	synonymous	1	0
SMARCAD1	chr4	95147344/A//G	nonsynonymous	1	0
BMPR1B	chr4	96046230/G//A	synonymous	1	0
PDHA2	chr4	96762261/T//C	synonymous	1	0
STPG2	chr4	98480246/C//A	nonsynonymous	1	0
NFKB1	chr4	103516131/G//A	nonsynonymous	1	0
CISD2	chr4	103806475/G//T	nonsynonymous	1	0
TET2	chr4	106157540/G//T	nonsynonymous	1	0
TBCK	chr4	107163632/T//G	synonymous	1	0
TBCK	chr4	107168385/G//A	nonsynonymous	1	0
ETNPPL	chr4	109681429/G//T	synonymous	1	0
COL25A1	chr4	109769914/C//A	nonsynonymous	1	0
COL25A1	chr4	110223055/G//A	synonymous	1	0
MCUB	chr4	110481584/C//G	nonsynonymous	1	0
ENPEP	chr4	111398017/G//T	nonsynonymous	1	0
ZGRF1	chr4	113540220/C//T	synonymous	1	0
ANK2	chr4	114170946/T//A	synonymous	1	0
NDST3	chr4	118975429/G//C	nonsynonymous	1	0
PDE5A	chr4	120527900/C//A	synonymous	1	0
TRPC3	chr4	122833121/A//T	nonsynonymous	1	0
KIAA1109	chr4	123141564/G//C	nonsynonymous	1	0
KIAA1109	chr4	123268856/G//T	nonsynonymous	1	0
KIAA1109	chr4	123274294/G//C	nonsynonymous	1	0
FAT4	chr4	126242219/G//T	nonsynonymous	1	0
MGARP	chr4	140188176/C//A	synonymous	1	0
MAML3	chr4	140811710/T//A	nonsynonymous	1	0
MAML3	chr4	140812033/G//C	nonsynonymous	1	0
TBC1D9	chr4	141543364/C//A	synonymous	1	0
OTUD4	chr4	146064557/G//C	nonsynonymous	1	0
PRMT9	chr4	148575024/A//G	nonsynonymous	1	0
DCLK2	chr4	151142877/A//G	nonsynonymous	1	0
SH3D19	chr4	152060987/C//A	nonsynonymous	1	0
FGA	chr4	155505917/C//G	nonsynonymous	1	0
NPY1R	chr4	164247228/G//A	nonsynonymous	1	0
NPY5R	chr4	164272571/G//A	nonsynonymous	1	0
TLL1	chr4	166946488/G//T	nonsynonymous	1	0
ANXA10	chr4	169060729/C//T	nonsynonymous	1	0
AADAT	chr4	170990363/G//A	nonsynonymous	1	0
GALNTL6	chr4	173734773/A//T	nonsynonymous	1	0
WDR17	chr4	177082105/C//T	nonsynonymous	1	0
AGA	chr4	178358661/CT//C	nonsynonymous	1	0
CDKN2AIP	chr4	184368109/C//G	nonsynonymous	1	0
FAM149A	chr4	187088167/G//A	nonsynonymous	1	0
CLPTM1L	chr5	1341794/C//G	nonsynonymous	1	0
IRX1	chr5	3600239/G//C	nonsynonymous	1	0
ADAMTS16	chr5	5235135/A//T	nonsynonymous	1	0
PAPD7	chr5	6743862/A//C	nonsynonymous	1	0
PAPD7	chr5	6751220/C//T	synonymous	1	0
SEMA5A	chr5	9066693/C//G	nonsynonymous	1	0
CTNND2	chr5	11117671/C//A	nonsynonymous	1	0
CTNND2	chr5	11159846/G//A	synonymous	1	0
DNAH5	chr5	13824368/T//A	synonymous	1	0
MARCH11	chr5	16067696/G//T	nonsynonymous	1	0
MARCH11	chr5	16091060/C//T	nonsynonymous	1	0
PRDM9	chr5	23509092/C//A	synonymous	1	0
PRDM9	chr5	23526554/G//T	nonsynonymous	1	0
PRDM9	chr5	23526896/C//A	nonsynonymous	1	0
CDH9	chr5	26915837/C//A	nonsynonymous	1	0
CDH6	chr5	31297434/G//T	nonsynonymous	1	0
DROSHA	chr5	31401560/C//G	nonsynonymous	1	0
RXFP3	chr5	33936787/G//T	synonymous	1	0
RAD1	chr5	34914869/G//C	nonsynonymous	1	0
DNAJC21	chr5	34945907/A//T	nonsynonymous	1	0
SPEF2	chr5	35709106/G//T	nonsynonymous	1	0
SLC1A3	chr5	36671224/G//C	nonsynonymous	1	0
NIPBL	chr5	37022379/A//T	nonsynonymous	1	0
C5orf42	chr5	37247790/C//A	nonsynonymous	1	0
NUP155	chr5	37304912/T//G	nonsynonymous	1	0

NUP155	chr5	37327790/T//C	synonymous	1	0
EGFLAM	chr5	38338839/G//C	nonsynonymous	1	0
EGFLAM	chr5	38448399/C//A	synonymous	1	0
OSMR	chr5	38904576/G//A	nonsynonymous	1	0
C9	chr5	39331843/C//T	nonsynonymous	1	0
CARD6	chr5	40853115/G//T	nonsynonymous	1	0
C7	chr5	40934560/G//T	nonsynonymous	1	0
C7	chr5	40964879/G//A	nonsynonymous	1	0
MROH2B	chr5	40998268/G//T	synonymous	1	0
C6	chr5	41149449/G//T	nonsynonymous	1	0
HMGCS1	chr5	43298858/C//A	nonsynonymous	1	0
NNT	chr5	43644761/C//G	nonsynonymous	1	0
GZMK	chr5	54329652/T//A	synonymous	1	0
ANKRD55	chr5	55528683/C//A	nonsynonymous	1	0
MAP3K1	chr5	56160642/C//T	nonsynonymous	1	0
GPBP1	chr5	56527134/G//C	nonsynonymous	1	0
PLK2	chr5	57755534/C//A	nonsynonymous	1	0
MCCC2	chr5	70900260/A//T	nonsynonymous	1	0
ARHGEF28	chr5	73069798/G//T	nonsynonymous	1	0
SV2C	chr5	75427838/T//A	nonsynonymous	1	0
IQGAP2	chr5	75950794/A//T	nonsynonymous	1	0
BHMT	chr5	78423613/C//T	nonsynonymous	1	0
CMYA5	chr5	79033166/G//T	nonsynonymous	1	0
VCAN	chr5	82833527/A//G	nonsynonymous	1	0
GPR150	chr5	94956899/G//A	nonsynonymous	1	0
PCSK1	chr5	95733161/A//C	nonsynonymous	1	0
MAN2A1	chr5	109110505/G//C	nonsynonymous	1	0
APC	chr5	112102927/C//G	nonsynonymous	1	0
ZNF474	chr5	121488730/C//G	nonsynonymous	1	0
SNCAIP	chr5	121787263/T//A	synonymous	1	0
SLC12A2	chr5	127420410/C//T	synonymous	1	0
FBN2	chr5	127700419/C//A	nonsynonymous	1	0
ADAMTS19	chr5	129039964/T//C	synonymous	1	0
CHSY3	chr5	129240891/G//A	synonymous	1	0
RAPGEF6	chr5	130764603/T//A	nonsynonymous	1	0
PDLIM4	chr5	131607985/C//T	synonymous	1	0
TXNDC15	chr5	134235171/TC//T	synonymous	1	0
BRD8	chr5	137476493/C//A	synonymous	1	0
KDM3B	chr5	137766067/C//T	nonsynonymous	1	0
NDUFA2	chr5	140027251/C//G	synonymous	1	0
DND1	chr5	140050979/T//A	nonsynonymous	1	0
PCDHA2	chr5	140176844/C//A	synonymous	1	0
PCDHA4	chr5	140187786/G//A	synonymous	1	0
PCDHA5	chr5	140201567/C//A	synonymous	1	0
PCDHA6	chr5	140208077/C//T	nonsynonymous	1	0
PCDHA7	chr5	140214372/C//T	nonsynonymous	1	0
PCDHA8	chr5	140222981/C//T	nonsynonymous	1	0
PCDHA13	chr5	140262032/T//G	nonsynonymous	1	0
PCDHAC1	chr5	140306672/C//A	nonsynonymous	1	0
PCDHB15	chr5	140626811/C//A	nonsynonymous	1	0
PCDHGA1	chr5	140710805/G//T	nonsynonymous	1	0
PCDHGA1	chr5	140710996/A//G	nonsynonymous	1	0
PCDHGB1	chr5	140731562/G//T	nonsynonymous	1	0
PCDHGA5	chr5	140744459/G//T	nonsynonymous	1	0
PCDHGA8	chr5	140773615/G//T	nonsynonymous	1	0
PCDHGB6	chr5	140789004/G//T	nonsynonymous	1	0
PCDHGB7	chr5	140797347/G//T	synonymous	1	0
POU4F3	chr5	145719603/C//G	nonsynonymous	1	0
SYNPO	chr5	150028545/C//T	synonymous	1	0
FAT2	chr5	150947928/T//C	nonsynonymous	1	0
HAND1	chr5	153855442/A//T	nonsynonymous	1	0
KIF4B	chr5	154396951/G//T	nonsynonymous	1	0
ITK	chr5	156608093/A//G	synonymous	1	0
C5orf52	chr5	157098770/G//T	nonsynonymous	1	0
EBF1	chr5	158526457/C//T	synonymous	1	0
ADRA1B	chr5	159399272/G//A	nonsynonymous	1	0
GABRG2	chr5	161569217/G//T	nonsynonymous	1	0
GABRG2	chr5	161576293/G//T	nonsynonymous	1	0
WWC1	chr5	167882503/C//G	nonsynonymous	1	0
SLIT3	chr5	168093559/C//A	nonsynonymous	1	0

SPDL1	chr5	169015475/G//C	nonsynonymous	1	0
FAM196B	chr5	169310379/G//T	nonsynonymous	1	0
DOCK2	chr5	169446112/G//T	nonsynonymous	1	0
TLX3	chr5	170736411/G//T	nonsynonymous	1	0
STK10	chr5	171520813/C//A	nonsynonymous	1	0
BOD1	chr5	173040178/C//A	nonsynonymous	1	0
HRH2	chr5	175110360/G//A	nonsynonymous	1	0
HRH2	chr5	175110583/G//T	nonsynonymous	1	0
UNC5A	chr5	176305314/G//A	nonsynonymous	1	0
HK3	chr5	176310839/T//C	nonsynonymous	1	0
TBC1D9B	chr5	179290699/C//T	nonsynonymous	1	0
RASGEF1C	chr5	179545653/C//A	nonsynonymous	1	0
CNOT6	chr5	179991699/G//T	nonsynonymous	1	0
BTNL3	chr5	180432593/C//T	synonymous	1	0
TRIM7	chr5	180622424/G//A	synonymous	1	0
NQO2	chr6	3017147/G//T	synonymous	1	0
SLC22A23	chr6	3273493/GGGCA//G	nonsynonymous	1	0
RREB1	chr6	7231982/G//T	nonsynonymous	1	0
DSP	chr6	7581097/G//T	synonymous	1	0
SNRNP48	chr6	7590627/G//A	nonsynonymous	1	0
ERVFRD-1	chr6	11104735/G//T	nonsynonymous	1	0
HIVEP1	chr6	12162090/G//T	nonsynonymous	1	0
RBM24	chr6	17291999/C//A	nonsynonymous	1	0
CAP2	chr6	17421836/G//A	nonsynonymous	1	0
NRSN1	chr6	24145944/G//T	nonsynonymous	1	0
HIST1H1C	chr6	26056277/T//A	nonsynonymous	1	0
HIST1H2AE	chr6	26217264/G//T	nonsynonymous	1	0
BTN3A1	chr6	26408051/G//A	nonsynonymous	1	0
BTN3A1	chr6	26408148/G//T	nonsynonymous	1	0
BTN1A1	chr6	26509263/G//C	nonsynonymous	1	0
BTN1A1	chr6	26509383/C//A	nonsynonymous	1	0
HIST1H2AH	chr6	27115117/G//T	synonymous	1	0
PRSS16	chr6	27215574/C//T	nonsynonymous	1	0
POM121L2	chr6	27279105/G//A	nonsynonymous	1	0
HIST1H1B	chr6	27834960/G//A	synonymous	1	0
ZSCAN9	chr6	28194918/G//T	nonsynonymous	1	0
ZSCAN9	chr6	28200828/C//G	nonsynonymous	1	0
	chr6	28226980/C//A	synonymous	1	0
ZSCAN26	chr6	28244062/T//G	nonsynonymous	1	0
ZBED9	chr6	28543639/T//A	synonymous	1	0
LOC100129636	chr6	29044139/C//T	synonymous	1	0
LOC100129636	chr6	29044433/G//T	synonymous	1	0
OR14J1	chr6	29275288/A//T	synonymous	1	0
OR12D2	chr6	29364752/C//A	nonsynonymous	1	0
OR10C1	chr6	29407933/G//T	synonymous	1	0
MAS1L	chr6	29455386/C//A	nonsynonymous	1	0
ZFP57	chr6	29640630/A//G	nonsynonymous	1	0
TRIM10	chr6	30122144/G//T	synonymous	1	0
TRIM15	chr6	30139725/G//T	nonsynonymous	1	0
TRIM39-RPP21	chr6	30314583/A//T	nonsynonymous	1	0
ABCF1	chr6	30550284/G//T	nonsynonymous	1	0
ABCF1	chr6	30553948/A//G	nonsynonymous	1	0
FLOT1	chr6	30707967/G//A	nonsynonymous	1	0
CDSN	chr6	31084463/C//T	nonsynonymous	1	0
VARS	chr6	31747511/G//A	synonymous	1	0
SLC44A4	chr6	31839177/G//A	synonymous	1	0
TNXB	chr6	32032716/C//A	synonymous	1	0
NOTCH4	chr6	32180374/A//G	nonsynonymous	1	0
COL11A2	chr6	33139278/C//A	nonsynonymous	1	0
ITPR3	chr6	33648145/G//T	nonsynonymous	1	0
LEMD2	chr6	33756773/C//T	nonsynonymous	1	0
PACSIN1	chr6	34497479/G//T	nonsynonymous	1	0
ANKS1A	chr6	35021901/G//T	nonsynonymous	1	0
CLPSL1	chr6	35755752/G//A	nonsynonymous	1	0
SLC26A8	chr6	35911776/G//T	synonymous	1	0
PNPLA1	chr6	36259132/G//C	nonsynonymous	1	0
ZFAND3	chr6	38120201/G//T	synonymous	1	0
DNAH8	chr6	38810206/G//A	nonsynonymous	1	0
MOCS1	chr6	39880004/T//A	synonymous	1	0
TTBK1	chr6	43220605/C//A	synonymous	1	0

TMEM151B	chr6	44240868/G//T	synonymous	1	0
AARS2	chr6	44272471/A//T	nonsynonymous	1	0
RUNX2	chr6	45399717/A//G	nonsynonymous	1	0
MEP1A	chr6	46800925/A//T	nonsynonymous	1	0
ADGRF1	chr6	46996788/C//A	nonsynonymous	1	0
PTCHD4	chr6	47846790/G//T	nonsynonymous	1	0
RHAG	chr6	49573464/G//A	synonymous	1	0
GSTA5	chr6	52696736/C//A	synonymous	1	0
ICK	chr6	52881027/G//A	nonsynonymous	1	0
MLIP	chr6	54095667/T//C	synonymous	1	0
HMGCLL1	chr6	55406886/C//T	nonsynonymous	1	0
DST	chr6	56504516/T//C	nonsynonymous	1	0
PHF3	chr6	64422853/G//T	nonsynonymous	1	0
EYS	chr6	66044999/C//A	nonsynonymous	1	0
ADGRB3	chr6	70049285/G//C	synonymous	1	0
COL9A1	chr6	70964213/C//A	nonsynonymous	1	0
MTO1	chr6	74171719/G//T	nonsynonymous	1	0
MTO1	chr6	74175987/G//C	nonsynonymous	1	0
EEF1A1	chr6	74227593/C//T	synonymous	1	0
TTK	chr6	80721642/A//T	nonsynonymous	1	0
PRSS35	chr6	84233758/C//G	nonsynonymous	1	0
SRSF12	chr6	89814919/G//C	nonsynonymous	1	0
MDN1	chr6	90459089/C//G	nonsynonymous	1	0
GJA10	chr6	90605139/G//A	nonsynonymous	1	0
EPHA7	chr6	93979309/G//T	nonsynonymous	1	0
FUT9	chr6	96651112/C//A	synonymous	1	0
KLHL32	chr6	97562198/G//T	nonsynonymous	1	0
FBXL4	chr6	99347359/T//A	nonsynonymous	1	0
SIM1	chr6	100841670/G//T	nonsynonymous	1	0
SIM1	chr6	100896056/G//T	nonsynonymous	1	0
SIM1	chr6	100911207/C//T	synonymous	1	0
PRDM1	chr6	106552902/C//A	synonymous	1	0
PRDM1	chr6	106553096/G//A	nonsynonymous	1	0
AIM1	chr6	106968452/G//A	nonsynonymous	1	0
AIM1	chr6	106991426/C//T	nonsynonymous	1	0
NR2E1	chr6	108497817/G//T	nonsynonymous	1	0
ZBTB24	chr6	109802806/T//A	nonsynonymous	1	0
CDC40	chr6	110547371/G//C	nonsynonymous	1	0
TUBE1	chr6	112408680/G//T	synonymous	1	0
RFPL4B	chr6	112671411/T//A	synonymous	1	0
FAM26F	chr6	116784652/G//T	synonymous	1	0
FAM26E	chr6	116836823/G//T	nonsynonymous	1	0
SLC35F1	chr6	118606414/G//T	nonsynonymous	1	0
SLC35F1	chr6	118635279/C//A	nonsynonymous	1	0
CEP85L	chr6	118786709/C//A	nonsynonymous	1	0
NKAIN2	chr6	124604242/T//C	nonsynonymous	1	0
THEMIS	chr6	128150620/C//A	nonsynonymous	1	0
L3MBTL3	chr6	130370512/C//T	nonsynonymous	1	0
ENPP1	chr6	132181576/C//A	nonsynonymous	1	0
STX7	chr6	132781897/T//G	nonsynonymous	1	0
TAAR9	chr6	132859515/A//T	synonymous	1	0
TAAR1	chr6	132966777/G//T	nonsynonymous	1	0
VNN1	chr6	133013437/C//T	synonymous	1	0
PDE7B	chr6	136512962/A//C	nonsynonymous	1	0
IL20RA	chr6	137329835/C//A	nonsynonymous	1	0
ARFGEF3	chr6	138599586/A//T	nonsynonymous	1	0
TXLNB	chr6	139609618/C//A	nonsynonymous	1	0
ADGRG6	chr6	142688780/G//T	nonsynonymous	1	0
GRM1	chr6	146351211/A//T	synonymous	1	0
ADGB	chr6	147109625/G//T	nonsynonymous	1	0
ZC3H12D	chr6	149771994/G//A	nonsynonymous	1	0
ULBP1	chr6	150289960/T//A	synonymous	1	0
ULBP3	chr6	150390199/C//G	nonsynonymous	1	0
AKAP12	chr6	151671402/C//A	nonsynonymous	1	0
RMND1	chr6	151766935/T//A	synonymous	1	0
CCDC170	chr6	151917613/C//A	nonsynonymous	1	0
SCAF8	chr6	155153496/C//T	nonsynonymous	1	0
SCAF8	chr6	155153581/G//C	synonymous	1	0
TIAM2	chr6	155450542/G//A	nonsynonymous	1	0
TMEM181	chr6	159005074/AAGGT//A	nonsynonymous	1	0

PNLDC1	chr6	160241533/G//T	nonsynonymous	1	0
LPA	chr6	160966532/G//T	nonsynonymous	1	0
PLG	chr6	161155115/A//C	nonsynonymous	1	0
PARK2	chr6	161970011/C//CA	nonsynonymous	1	0
C6orf118	chr6	165715186/C//T	nonsynonymous	1	0
T	chr6	166575993/C//A	synonymous	1	0
GPR31	chr6	167570464/T//A	nonsynonymous	1	0
GPR31	chr6	167570606/G//T	nonsynonymous	1	0
THBS2	chr6	169648544/C//A	nonsynonymous	1	0
WDR27	chr6	170072470/T//C	nonsynonymous	1	0
TCTE3	chr6	170144201/GC//G	nonsynonymous	1	0
MICALL2	chr7	1479575/C//A	nonsynonymous	1	0
INTS1	chr7	1510208/C//A	synonymous	1	0
SNX8	chr7	2296556/G//A	nonsynonymous	1	0
IQCE	chr7	2623287/A//G	nonsynonymous	1	0
FOXK1	chr7	4794096/C//T	synonymous	1	0
RADIL	chr7	4838973/G//T	synonymous	1	0
RADIL	chr7	4855873/T//A	nonsynonymous	1	0
PAPOLB	chr7	4899648/G//T	synonymous	1	0
RBAK	chr7	5105367/C//T	synonymous	1	0
TNRC18	chr7	5401331/T//C	nonsynonymous	1	0
ICA1	chr7	8268273/C//A	nonsynonymous	1	0
MEOX2	chr7	15725605/C//A	synonymous	1	0
ISPD	chr7	16348239/T//A	nonsynonymous	1	0
SOSTDC1	chr7	16502118/A//T	synonymous	1	0
PRPS1L1	chr7	18066687/C//A	nonsynonymous	1	0
HDAC9	chr7	18868781/C//A	synonymous	1	0
ITGB8	chr7	20418799/G//C	nonsynonymous	1	0
DNAH11	chr7	21640292/A//T	nonsynonymous	1	0
GPNMB	chr7	23309689/G//A	nonsynonymous	1	0
IGF2BP3	chr7	23383417/C//A	nonsynonymous	1	0
FAM221A	chr7	23731030/A//T	nonsynonymous	1	0
CYCS	chr7	25163650/C//A	nonsynonymous	1	0
C7orf31	chr7	25218904/G//C	synonymous	1	0
HOXA10	chr7	27211464/G//C	synonymous	1	0
NOD1	chr7	30492618/G//C	nonsynonymous	1	0
ADCYAP1R1	chr7	31117664/G//T	nonsynonymous	1	0
ANLN	chr7	36450717/A//T	nonsynonymous	1	0
GPR141	chr7	37780193/G//A	synonymous	1	0
NME8	chr7	37916535/T//A	nonsynonymous	1	0
NME8	chr7	37928024/C//A	nonsynonymous	1	0
SFRP4	chr7	37953976/G//T	synonymous	1	0
INHBA	chr7	41729343/G//A	synonymous	1	0
GLI3	chr7	42005514/C//A	nonsynonymous	1	0
GLI3	chr7	42012010/G//T	nonsynonymous	1	0
STK17A	chr7	43659224/C//G	nonsynonymous	1	0
TNS3	chr7	47384411/C//T	synonymous	1	0
C7orf57	chr7	48092436/C//G	nonsynonymous	1	0
ABCA13	chr7	48314375/A//T	nonsynonymous	1	0
DDC	chr7	50531049/C//A	nonsynonymous	1	0
VSTM2A	chr7	54612470/G//A	nonsynonymous	1	0
ZNF727	chr7	63538262/A//T	nonsynonymous	1	0
ZNF735	chr7	63667597/G//T	nonsynonymous	1	0
ZNF735	chr7	63667605/G//A	nonsynonymous	1	0
ZNF117	chr7	64439078/T//C	nonsynonymous	1	0
	chr7	71868383/G//C	synonymous	1	0
ABHD11	chr7	73151550/C//A	nonsynonymous	1	0
ELN	chr7	73477523/C//A	synonymous	1	0
HIP1	chr7	75172244/G//A	nonsynonymous	1	0
YWHAG	chr7	75958818/C//T	synonymous	1	0
MAGI2	chr7	77814994/G//T	synonymous	1	0
MAGI2	chr7	77885262/C//A	nonsynonymous	1	0
PCLO	chr7	82544135/C//A	nonsynonymous	1	0
PCLO	chr7	82581187/C//A	nonsynonymous	1	0
SEMA3A	chr7	83590806/G//T	nonsynonymous	1	0
ABCB4	chr7	87082305/C//A	nonsynonymous	1	0
ABCB1	chr7	87170710/G//T	nonsynonymous	1	0
C7orf62	chr7	88423478/C//A	synonymous	1	0
COL1A2	chr7	94042394/G//C	nonsynonymous	1	0
PEG10	chr7	94294151/G//T	nonsynonymous	1	0

DLX6	chr7	96639390/C//A	synonymous	1	0
TRRAP	chr7	98557020/G//T	synonymous	1	0
SMURF1	chr7	98639755/A//T	nonsynonymous	1	0
BUD31	chr7	99013890/A//T	synonymous	1	0
ATP5J2-PTCD1	chr7	99063824/C//G	synonymous	1	0
CYP3A5	chr7	99270212/T//G	synonymous	1	0
PPP1R35	chr7	100033001/C//T	synonymous	1	0
ACTL6B	chr7	100253510/G//T	synonymous	1	0
ZAN	chr7	100349588/C//T	synonymous	1	0
ZAN	chr7	100350737/C//A	synonymous	1	0
EPHB4	chr7	100417352/C//T	nonsynonymous	1	0
MUC12	chr7	100634129/C//A	synonymous	1	0
MUC12	chr7	100648071/A//C	nonsynonymous	1	0
MUC12	chr7	100660888/G//C	nonsynonymous	1	0
MUC17	chr7	100677405/G//A	nonsynonymous	1	0
COL26A1	chr7	101192503/A//T	nonsynonymous	1	0
CUX1	chr7	101840138/C//T	nonsynonymous	1	0
RELN	chr7	103252095/G//T	nonsynonymous	1	0
RELN	chr7	103368594/G//T	synonymous	1	0
RINT1	chr7	105190556/C//T	synonymous	1	0
PIK3CG	chr7	106508696/C//G	nonsynonymous	1	0
PRKAR2B	chr7	106685496/C//T	synonymous	1	0
COG5	chr7	106938787/C//A	synonymous	1	0
SLC26A3	chr7	107434261/G//T	nonsynonymous	1	0
LAMB1	chr7	107626690/G//T	nonsynonymous	1	0
TMEM168	chr7	112424925/C//G	synonymous	1	0
CFTR	chr7	117232334/A//G	nonsynonymous	1	0
KCND2	chr7	120381687/C//A	synonymous	1	0
WNT16	chr7	120969652/G//C	nonsynonymous	1	0
PTPRZ1	chr7	121652513/C//T	nonsynonymous	1	0
AASS	chr7	121733004/C//A	nonsynonymous	1	0
CADPS2	chr7	122131475/C//A	nonsynonymous	1	0
TMEM229A	chr7	123672057/C//G	nonsynonymous	1	0
GRM8	chr7	126086196/C//T	synonymous	1	0
GRM8	chr7	126410118/C//G	synonymous	1	0
FLNC	chr7	128492955/C//T	synonymous	1	0
TNPO3	chr7	128620088/G//A	synonymous	1	0
SMO	chr7	128851931/G//T	nonsynonymous	1	0
PLXNA4	chr7	131895739/C//A	nonsynonymous	1	0
PLXNA4	chr7	131912367/T//C	synonymous	1	0
EXOC4	chr7	132937834/CT//C	synonymous	1	0
STRA8	chr7	134931354/C//A	nonsynonymous	1	0
NUP205	chr7	135262745/A//T	nonsynonymous	1	0
DGKI	chr7	137150709/G//T	nonsynonymous	1	0
DENND2A	chr7	140218533/T//A	nonsynonymous	1	0
DENND2A	chr7	140255506/C//G	nonsynonymous	1	0
WEE2	chr7	141424969/C//G	synonymous	1	0
PRSS1	chr7	142459771/G//T	nonsynonymous	1	0
TRPV5	chr7	142612146/G//T	nonsynonymous	1	0
KEL	chr7	142641427/G//T	synonymous	1	0
OR9A2	chr7	142723760/A//G	nonsynonymous	1	0
CLCN1	chr7	143043323/C//A	nonsynonymous	1	0
CLCN1	chr7	143047702/C//T	synonymous	1	0
EPHA1	chr7	143096887/C//A	nonsynonymous	1	0
CNTNAP2	chr7	146805381/G//T	nonsynonymous	1	0
CNTNAP2	chr7	146818087/C//G	synonymous	1	0
CNTNAP2	chr7	147844655/C//A	nonsynonymous	1	0
CNTNAP2	chr7	147869415/G//C	nonsynonymous	1	0
EZH2	chr7	148525888/C//A	nonsynonymous	1	0
EZH2	chr7	148529770/C//A	nonsynonymous	1	0
ZNF398	chr7	148875791/C//T	nonsynonymous	1	0
SSPO	chr7	149482637/G//T	nonsynonymous	1	0
SSPO	chr7	149488947/C//G	nonsynonymous	1	0
SSPO	chr7	149517834/G//C	nonsynonymous	1	0
SSPO	chr7	149520942/G//T	synonymous	1	0
GIMAP1	chr7	150417558/G//A	nonsynonymous	1	0
AOC1	chr7	150553627/G//T	synonymous	1	0
ASB10	chr7	150878190/G//T	nonsynonymous	1	0
PRKAG2	chr7	151273506/G//A	synonymous	1	0
ACTR3B	chr7	152550643/G//C	nonsynonymous	1	0

ARHGEF10	chr8	1851439/G//T	synonymous	1	0
MCPH1	chr8	6302738/G//T	nonsynonymous	1	0
PINX1	chr8	10622970/C//A	nonsynonymous	1	0
DLC1	chr8	12957837/C//A	nonsynonymous	1	0
DLC1	chr8	13251154/G//T	nonsynonymous	1	0
MSR1	chr8	16026281/C//A	nonsynonymous	1	0
PIWIL2	chr8	22173801/G//A	nonsynonymous	1	0
PDLIM2	chr8	22447129/G//A	nonsynonymous	1	0
BIN3	chr8	22526602/A//G	synonymous	1	0
EBF2	chr8	25744343/C//G	nonsynonymous	1	0
SCARA3	chr8	27507276/G//T	nonsynonymous	1	0
INTS9	chr8	28635450/C//T	nonsynonymous	1	0
TEX15	chr8	30695008/G//A	nonsynonymous	1	0
TEX15	chr8	30700593/G//T	nonsynonymous	1	0
UNC5D	chr8	35093301/G//T	synonymous	1	0
KCNU1	chr8	36793271/A//T	nonsynonymous	1	0
ZNF703	chr8	37555184/G//T	synonymous	1	0
PROSC	chr8	37623143/G//A	nonsynonymous	1	0
ADRB3	chr8	37823289/G//T	synonymous	1	0
FGFR1	chr8	38314916/T//G	nonsynonymous	1	0
ANK1	chr8	41575669/C//A	nonsynonymous	1	0
ANK1	chr8	41580695/C//G	nonsynonymous	1	0
HGSNAT	chr8	43025814/C//T	synonymous	1	0
PRKDC	chr8	48689467/G//T	synonymous	1	0
SNTG1	chr8	51617178/C//A	synonymous	1	0
PXDNL	chr8	52320840/G//T	nonsynonymous	1	0
PXDNL	chr8	52320921/G//C	nonsynonymous	1	0
SOX17	chr8	55370639/G//A	synonymous	1	0
SOX17	chr8	55371890/C//G	nonsynonymous	1	0
TGS1	chr8	56686263/C//T	nonsynonymous	1	0
	chr8	62588621/C//G	synonymous	1	0
BHLHE22	chr8	65493589/G//C	nonsynonymous	1	0
TRIM55	chr8	67047252/G//T	nonsynonymous	1	0
CSPP1	chr8	68018198/G//T	nonsynonymous	1	0
SULF1	chr8	70515483/G//T	nonsynonymous	1	0
SULF1	chr8	70533472/A//G	nonsynonymous	1	0
EYA1	chr8	72233985/G//T	nonsynonymous	1	0
PI15	chr8	75757682/G//T	nonsynonymous	1	0
ZFHX4	chr8	77616356/G//T	nonsynonymous	1	0
ZFHX4	chr8	77616531/G//C	nonsynonymous	1	0
ZFHX4	chr8	77766766/T//A	nonsynonymous	1	0
RALYL	chr8	85762228/G//A	nonsynonymous	1	0
LRRCC1	chr8	86042248/C//G	nonsynonymous	1	0
CA2	chr8	86376223/G//A	synonymous	1	0
DCAF4L2	chr8	88885461/C//A	nonsynonymous	1	0
DCAF4L2	chr8	88885779/G//T	nonsynonymous	1	0
DCAF4L2	chr8	88885860/C//A	nonsynonymous	1	0
DCAF4L2	chr8	88886131/G//T	synonymous	1	0
MMP16	chr8	89068384/C//A	nonsynonymous	1	0
MMP16	chr8	89180039/A//T	nonsynonymous	1	0
SLC26A7	chr8	92352778/A//T	nonsynonymous	1	0
RUNX1T1	chr8	93004084/C//A	nonsynonymous	1	0
TMEM67	chr8	94821380/C//T	synonymous	1	0
CDH17	chr8	95186481/G//A	synonymous	1	0
GDF6	chr8	97156968/G//T	synonymous	1	0
GDF6	chr8	97157062/A//T	nonsynonymous	1	0
PTDSS1	chr8	97342446/C//T	synonymous	1	0
ODF1	chr8	103572827/C//G	nonsynonymous	1	0
RIMS2	chr8	104897962/G//T	nonsynonymous	1	0
EIF3E	chr8	109228731/T//A	synonymous	1	0
TRHR	chr8	110131396/C//G	synonymous	1	0
PKHD1L1	chr8	110408346/C//T	nonsynonymous	1	0
PKHD1L1	chr8	110467003/G//C	nonsynonymous	1	0
SYBU	chr8	110631068/G//A	synonymous	1	0
CSMD3	chr8	113253963/C//G	nonsynonymous	1	0
CSMD3	chr8	113259326/C//A	nonsynonymous	1	0
CSMD3	chr8	113364693/C//A	nonsynonymous	1	0
CSMD3	chr8	113529397/C//G	nonsynonymous	1	0
CSMD3	chr8	113650978/C//A	nonsynonymous	1	0
ATAD2	chr8	124346562/T//A	nonsynonymous	1	0

ATAD2	chr8	124371897/C//T	nonsynonymous	1	0
KLHL38	chr8	124665005/G//T	synonymous	1	0
FER1L6	chr8	125015549/C//T	synonymous	1	0
SQLE	chr8	126030440/C//G	nonsynonymous	1	0
ADCY8	chr8	131848578/C//T	nonsynonymous	1	0
TG	chr8	133899603/T//C	synonymous	1	0
NDRG1	chr8	134266834/G//T	nonsynonymous	1	0
ST3GAL1	chr8	134478165/C//A	nonsynonymous	1	0
COL22A1	chr8	139697504/G//A	nonsynonymous	1	0
MROH5	chr8	142483034/C//G	synonymous	1	0
ZFP41	chr8	144332101/G//T	nonsynonymous	1	0
GSDMD	chr8	144644683/C//T	nonsynonymous	1	0
PLEC	chr8	145001464/C//T	nonsynonymous	1	0
GPAA1	chr8	145138660/G//T	nonsynonymous	1	0
HGH1	chr8	145193656/C//T	nonsynonymous	1	0
FOXH1	chr8	145700005/G//A	synonymous	1	0
LRRC24	chr8	145750302/G//T	synonymous	1	0
DOCK8	chr9	312135/A//T	nonsynonymous	1	0
KANK1	chr9	713469/G//T	synonymous	1	0
INSL4	chr9	5233695/G//T	nonsynonymous	1	0
PTPRD	chr9	8341964/C//A	nonsynonymous	1	0
FREM1	chr9	14851545/C//G	nonsynonymous	1	0
CCDC171	chr9	15724769/G//T	synonymous	1	0
PLIN2	chr9	19121167/T//A	synonymous	1	0
IFNA7	chr9	21202158/G//T	synonymous	1	0
IFNA5	chr9	21305197/C//T	nonsynonymous	1	0
PLAA	chr9	26947038/C//A	synonymous	1	0
LINGO2	chr9	27949588/C//T	nonsynonymous	1	0
KIAA1161	chr9	34372433/C//A	nonsynonymous	1	0
CD72	chr9	35612979/G//T	nonsynonymous	1	0
GBA2	chr9	35738036/C//T	nonsynonymous	1	0
OR13J1	chr9	35869646/G//T	nonsynonymous	1	0
GLIPR2	chr9	36150906/G//C	nonsynonymous	1	0
POLR1E	chr9	37486092/G//C	synonymous	1	0
SHB	chr9	37919910/C//A	nonsynonymous	1	0
TJP2	chr9	71836170/G//T	nonsynonymous	1	0
CEP78	chr9	80868174/A//C	nonsynonymous	1	0
PSAT1	chr9	80919645/G//T	synonymous	1	0
SPATA31D1	chr9	84606340/A//T	nonsynonymous	1	0
SPATA31D1	chr9	84607183/A//G	nonsynonymous	1	0
RASEF	chr9	85637272/T//C	synonymous	1	0
SPATA31E1	chr9	90500395/T//A	synonymous	1	0
CCDC180	chr9	100128881/A//G	nonsynonymous	1	0
TDRD7	chr9	100201566/G//T	nonsynonymous	1	0
ALDOB	chr9	104184125/G//T	nonsynonymous	1	0
GRIN3A	chr9	104500066/T//A	nonsynonymous	1	0
CYLC2	chr9	105767423/G//T	nonsynonymous	1	0
PTPN3	chr9	112153744/T//C	synonymous	1	0
ASTN2	chr9	119382637/G//T	nonsynonymous	1	0
CNTRL	chr9	123906258/G//T	synonymous	1	0
LHX6	chr9	124966934/G//A	synonymous	1	0
	chr9	124990646/C//T	synonymous	1	0
OR1B1	chr9	125391069/G//T	nonsynonymous	1	0
RC3H2	chr9	125627694/T//C	nonsynonymous	1	0
GOLGA2	chr9	131025336/G//C	synonymous	1	0
ZER1	chr9	131515742/T//G	synonymous	1	0
NUP188	chr9	131749108/A//C	nonsynonymous	1	0
C9orf78	chr9	132591485/C//G	nonsynonymous	1	0
USP20	chr9	132637214/G//T	nonsynonymous	1	0
QRFP	chr9	133768899/G//A	synonymous	1	0
	chr9	134585068/C//A	synonymous	1	0
TSC1	chr9	135776204/G//A	synonymous	1	0
SARDH	chr9	136584084/C//A	nonsynonymous	1	0
PPP1R26	chr9	138377778/G//T	synonymous	1	0
NACC2	chr9	138903608/G//A	synonymous	1	0
NOTCH1	chr9	139397700/C//A	nonsynonymous	1	0
CACNA1B	chr9	140811886/G//A	synonymous	1	0
CACNA1B	chr9	141013140/G//T	nonsynonymous	1	0
DIP2C	chr10	454910/T//G	nonsynonymous	1	0
PFKP	chr10	3155576/G//T	nonsynonymous	1	0

FBXO18	chr10	5945022/G//T	nonsynonymous	1	0
SFMBT2	chr10	7409775/T//A	nonsynonymous	1	0
ITIH5	chr10	7627979/G//T	nonsynonymous	1	0
USP6NL	chr10	11505405/C//A	nonsynonymous	1	0
CUBN	chr10	17083205/C//A	nonsynonymous	1	0
CUBN	chr10	17151725/C//A	nonsynonymous	1	0
KIAA1217	chr10	24762772/G//T	nonsynonymous	1	0
MYO3A	chr10	26459403/A//T	synonymous	1	0
SVIL	chr10	29812603/G//T	nonsynonymous	1	0
RET	chr10	43604528/G//C	nonsynonymous	1	0
TMEM72	chr10	45430384/G//A	synonymous	1	0
ALOX5	chr10	45938962/G//T	nonsynonymous	1	0
GDF2	chr10	48413793/C//A	nonsynonymous	1	0
FRMPD2	chr10	49400755/C//G	nonsynonymous	1	0
WDFY4	chr10	49934009/G//A	synonymous	1	0
WDFY4	chr10	49994877/C//A	nonsynonymous	1	0
FAM170B	chr10	50339851/A//T	nonsynonymous	1	0
ASAH2	chr10	52005042/A//T	nonsynonymous	1	0
PRKG1	chr10	52912983/T//A	nonsynonymous	1	0
LRRTM3	chr10	68687097/C//T	synonymous	1	0
CTNNA3	chr10	69299402/C//T	synonymous	1	0
MYPN	chr10	69959315/T//C	nonsynonymous	1	0
TET1	chr10	70334014/G//A	synonymous	1	0
CCAR1	chr10	70549649/G//A	nonsynonymous	1	0
HKDC1	chr10	71007239/C//A	synonymous	1	0
ADAMTS14	chr10	72434476/C//T	nonsynonymous	1	0
ADAMTS14	chr10	72498706/G//T	nonsynonymous	1	0
CDH23	chr10	73199655/G//T	nonsynonymous	1	0
CDH23	chr10	73337717/G//T	nonsynonymous	1	0
CDH23	chr10	73501685/G//C	synonymous	1	0
DNAJB12	chr10	74100593/C//A	nonsynonymous	1	0
DLG5	chr10	79589245/T//G	nonsynonymous	1	0
ZMIZ1	chr10	81070858/G//T	nonsynonymous	1	0
SH2D4B	chr10	82348405/G//T	nonsynonymous	1	0
CDHR1	chr10	85971462/G//T	nonsynonymous	1	0
GRID1	chr10	87614323/G//A	nonsynonymous	1	0
GRID1	chr10	87615808/G//C	nonsynonymous	1	0
WAPL	chr10	88260218/A//G	nonsynonymous	1	0
LDB3	chr10	88476504/G//T	nonsynonymous	1	0
PPP1R3C	chr10	93390074/G//C	nonsynonymous	1	0
CYP26C1	chr10	94822523/C//T	nonsynonymous	1	0
MYOF	chr10	95126156/C//A	nonsynonymous	1	0
RBP4	chr10	95360210/C//A	nonsynonymous	1	0
PLCE1	chr10	95791186/C//G	nonsynonymous	1	0
PLCE1	chr10	96006251/C//A	nonsynonymous	1	0
NOC3L	chr10	96114825/C//A	nonsynonymous	1	0
CYP2C18	chr10	96493197/T//A	nonsynonymous	1	0
DNTT	chr10	98079040/A//T	nonsynonymous	1	0
PIK3AP1	chr10	98369619/C//A	nonsynonymous	1	0
LCOR	chr10	98715591/G//T	nonsynonymous	1	0
PGAM1	chr10	99192140/G//A	synonymous	1	0
PYROXD2	chr10	100148271/C//A	synonymous	1	0
ABCC2	chr10	101556931/C//T	nonsynonymous	1	0
PAX2	chr10	102541053/G//T	nonsynonymous	1	0
LBX1	chr10	102988405/C//A	synonymous	1	0
NEURL1	chr10	105254216/C//A	synonymous	1	0
SORCS3	chr10	106675604/G//T	nonsynonymous	1	0
SORCS1	chr10	108439399/C//A	nonsynonymous	1	0
SORCS1	chr10	108923885/C//T	nonsynonymous	1	0
SMC3	chr10	112327469/C//A	synonymous	1	0
CCDC186	chr10	115885698/G//A	nonsynonymous	1	0
VWA2	chr10	116037671/A//T	nonsynonymous	1	0
PNLIPRP2	chr10	118396397/G//A	synonymous	1	0
WDR11	chr10	122619647/G//C	nonsynonymous	1	0
FGFR2	chr10	123263358/C//A	nonsynonymous	1	0
DMBT1	chr10	124336167/G//T	nonsynonymous	1	0
C10orf120	chr10	124457799/G//T	nonsynonymous	1	0
	chr10	128810519/G//T	synonymous	1	0
MKI67	chr10	129905753/T//A	nonsynonymous	1	0
MKI67	chr10	129907047/T//C	synonymous	1	0

MKI67	chr10	129907064/G//C	nonsynonymous	1	0
KNDC1	chr10	135032421/G//T	synonymous	1	0
	chr10	135125185/G//T	synonymous	1	0
PHRF1	chr11	592681/G//T	synonymous	1	0
PHRF1	chr11	606454/T//C	synonymous	1	0
KCNQ1	chr11	2594172/C//T	nonsynonymous	1	0
PGAP2	chr11	3819154/G//T	synonymous	1	0
STIM1	chr11	4103454/C//G	nonsynonymous	1	0
OR52K2	chr11	4470742/A//G	nonsynonymous	1	0
OR51E1	chr11	4673811/G//T	nonsynonymous	1	0
OR51T1	chr11	4903894/G//T	synonymous	1	0
OR51G1	chr11	4944957/C//G	nonsynonymous	1	0
OR51A2	chr11	4976732/G//T	nonsynonymous	1	0
OR52J3	chr11	5068616/T//A	synonymous	1	0
OR52A1	chr11	5173599/T//C	nonsynonymous	1	0
HBG2	chr11	5274501/G//T	synonymous	1	0
OR51M1	chr11	5410623/C//T	synonymous	1	0
OR51M1	chr11	5411121/C//A	nonsynonymous	1	0
OR51Q1	chr11	5443974/C//A	nonsynonymous	1	0
TRIM5	chr11	5701037/C//A	nonsynonymous	1	0
OR52E6	chr11	5862797/C//G	nonsynonymous	1	0
OR56A5	chr11	5989180/C//T	nonsynonymous	1	0
OR52L1	chr11	6007406/C//T	nonsynonymous	1	0
OR56A1	chr11	6048404/C//A	synonymous	1	0
OR56A1	chr11	6048729/A//G	nonsynonymous	1	0
RRP8	chr11	6622155/C//A	nonsynonymous	1	0
TAF10	chr11	6632676/T//A	nonsynonymous	1	0
DCHS1	chr11	6643086/G//T	nonsynonymous	1	0
OR2AG2	chr11	6789356/T//A	nonsynonymous	1	0
OR2AG1	chr11	6806433/C//G	synonymous	1	0
ZNF214	chr11	7021511/T//A	nonsynonymous	1	0
NLRP14	chr11	7067915/A//T	nonsynonymous	1	0
OVCH2	chr11	7718312/C//A	synonymous	1	0
WEE1	chr11	9607037/G//T	nonsynonymous	1	0
CTR9	chr11	10800342/G//T	nonsynonymous	1	0
MICALCL	chr11	12315696/C//T	synonymous	1	0
ABCC8	chr11	17436057/AC//A	nonsynonymous	1	0
ABCC8	chr11	17449841/C//A	nonsynonymous	1	0
	chr11	17803235/C//A	synonymous	1	0
MRGPRX3	chr11	18159382/G//A	synonymous	1	0
MRGPRX4	chr11	18194973/G//T	nonsynonymous	1	0
MRGPRX4	chr11	18195199/C//A	nonsynonymous	1	0
GTF2H1	chr11	18373889/C//T	nonsynonymous	1	0
LDHA	chr11	18422424/G//A	synonymous	1	0
LDHC	chr11	18472566/G//T	synonymous	1	0
IGSF22	chr11	18741769/C//A	nonsynonymous	1	0
PRMT3	chr11	20419192/G//T	synonymous	1	0
LUZP2	chr11	24518785/G//T	synonymous	1	0
ANO3	chr11	26646015/G//A	nonsynonymous	1	0
KCNA4	chr11	30032503/C//A	nonsynonymous	1	0
KCNA4	chr11	30032534/G//T	nonsynonymous	1	0
KCNA4	chr11	30033370/C//A	nonsynonymous	1	0
MPPED2	chr11	30557666/C//A	nonsynonymous	1	0
DCDC5	chr11	30914526/G//T	nonsynonymous	1	0
DCDC1	chr11	31327897/C//G	nonsynonymous	1	0
WT1	chr11	32450152/G//A	synonymous	1	0
CCDC73	chr11	32637504/C//G	nonsynonymous	1	0
QSER1	chr11	32955806/C//G	nonsynonymous	1	0
QSER1	chr11	32997884/C//T	nonsynonymous	1	0
KIAA1549L	chr11	33566608/A//T	synonymous	1	0
CD59	chr11	33743949/C//A	nonsynonymous	1	0
LDLRAD3	chr11	36248924/G//C	nonsynonymous	1	0
COMMD9	chr11	36310986/G//C	synonymous	1	0
PHF21A	chr11	45959820/C//A	nonsynonymous	1	0
CKAP5	chr11	46772944/C//A	nonsynonymous	1	0
FNBP4	chr11	47789022/G//T	synonymous	1	0
NUP160	chr11	47801933/C//A	nonsynonymous	1	0
OR4X1	chr11	48285761/G//T	nonsynonymous	1	0
OR4C6	chr11	55433526/C//T	nonsynonymous	1	0
OR5D14	chr11	55563348/C//A	nonsynonymous	1	0

OR5L1	chr11	55579551/C//A	synonymous	1	0
OR5D18	chr11	55587556/G//T	nonsynonymous	1	0
OR5L2	chr11	55595193/C//T	nonsynonymous	1	0
TRIM51	chr11	55653123/G//T	nonsynonymous	1	0
OR5F1	chr11	55761363/C//G	nonsynonymous	1	0
OR8H2	chr11	55872778/T//A	nonsynonymous	1	0
OR5M10	chr11	56344622/G//A	synonymous	1	0
OR5M10	chr11	56344665/T//C	nonsynonymous	1	0
APLNR	chr11	57003468/G//T	nonsynonymous	1	0
TNKS1BP1	chr11	57080570/G//T	nonsynonymous	1	0
SLC43A1	chr11	57259326/G//A	nonsynonymous	1	0
OR1S2	chr11	57970952/G//C	nonsynonymous	1	0
OR10Q1	chr11	57995952/G//C	nonsynonymous	1	0
OR5B2	chr11	58190380/C//T	nonsynonymous	1	0
OR5B2	chr11	58190654/G//T	synonymous	1	0
OR5B12	chr11	58206869/T//C	synonymous	1	0
MPEG1	chr11	58979250/G//C	nonsynonymous	1	0
OSBP	chr11	59361614/C//T	nonsynonymous	1	0
MS4A10	chr11	60557844/T//A	nonsynonymous	1	0
TMEM109	chr11	60689365/G//T	nonsynonymous	1	0
VWCE	chr11	61048411/C//A	nonsynonymous	1	0
FADS1	chr11	61584129/C//A	synonymous	1	0
FADS3	chr11	61646847/G//A	synonymous	1	0
AHNAK	chr11	62298088/G//A	synonymous	1	0
TUT1	chr11	62359102/C//A	synonymous	1	0
EML3	chr11	62369941/C//G	synonymous	1	0
B3GAT3	chr11	62389402/C//G	nonsynonymous	1	0
GANAB	chr11	62393809/G//A	synonymous	1	0
SLC22A8	chr11	62763206/C//G	nonsynonymous	1	0
	chr11	62848389/C//G	synonymous	1	0
STIP1	chr11	63961802/G//C	nonsynonymous	1	0
FERMT3	chr11	63987025/G//A	synonymous	1	0
ESRRA	chr11	64082681/G//T	nonsynonymous	1	0
NRXN2	chr11	64375181/C//A	synonymous	1	0
ATG2A	chr11	64675324/G//T	nonsynonymous	1	0
AP5B1	chr11	65546222/C//G	nonsynonymous	1	0
SART1	chr11	65733406/A//T	nonsynonymous	1	0
KLC2	chr11	66033220/C//T	nonsynonymous	1	0
CARNS1	chr11	67188583/C//T	nonsynonymous	1	0
TBX10	chr11	67402462/C//T	synonymous	1	0
GAL	chr11	68458524/C//T	synonymous	1	0
CPT1A	chr11	68529087/C//A	nonsynonymous	1	0
PPFIA1	chr11	70218588/G//C	nonsynonymous	1	0
CTTN	chr11	70260715/G//T	nonsynonymous	1	0
NUMA1	chr11	71724325/C//A	nonsynonymous	1	0
PLEKHB1	chr11	73372575/C//A	synonymous	1	0
UCP3	chr11	73712560/G//C	nonsynonymous	1	0
RNF169	chr11	74460106/C//T	nonsynonymous	1	0
XRRA1	chr11	74562206/C//T	nonsynonymous	1	0
NEU3	chr11	74717068/G//T	nonsynonymous	1	0
WNT11	chr11	75907762/C//A	synonymous	1	0
MYO7A	chr11	76866947/C//A	synonymous	1	0
MYO7A	chr11	76883889/C//G	nonsynonymous	1	0
MYO7A	chr11	76885964/C//G	synonymous	1	0
INTS4	chr11	77702202/C//G	synonymous	1	0
INTS4	chr11	77705693/T//A	synonymous	1	0
TENM4	chr11	78565192/G//A	synonymous	1	0
TENM4	chr11	78780912/G//A	synonymous	1	0
FAM181B	chr11	82443842/C//A	synonymous	1	0
PCF11	chr11	82868648/C//G	nonsynonymous	1	0
CCDC89	chr11	85396457/C//A	nonsynonymous	1	0
TRIM49C	chr11	89774566/C//A	nonsynonymous	1	0
FAT3	chr11	92507345/A//T	nonsynonymous	1	0
FAT3	chr11	92600318/G//T	nonsynonymous	1	0
CEP295	chr11	93432482/T//C	synonymous	1	0
ENDOD1	chr11	94823064/C//A	synonymous	1	0
ENDOD1	chr11	94861718/C//T	nonsynonymous	1	0
SESN3	chr11	94923061/C//A	nonsynonymous	1	0
ARHGAP42	chr11	100784255/G//T	nonsynonymous	1	0
ARHGAP42	chr11	100846899/G//T	nonsynonymous	1	0

PGR	chr11	100998906/G//T	nonsynonymous	1	0
ANGPTL5	chr11	101762050/G//A	nonsynonymous	1	0
ANGPTL5	chr11	101762322/T//A	synonymous	1	0
C11orf70	chr11	101946741/G//C	synonymous	1	0
MMP12	chr11	102743681/G//A	synonymous	1	0
DYNC2H1	chr11	103006492/G//T	nonsynonymous	1	0
DYNC2H1	chr11	103062832/C//G	nonsynonymous	1	0
PDGFD	chr11	104034536/T//C	synonymous	1	0
CASP12	chr11	104762132/C//A	nonsynonymous	1	0
GRIA4	chr11	105732794/G//T	nonsynonymous	1	0
GRIA4	chr11	105789589/C//G	nonsynonymous	1	0
MSANTD4	chr11	105880398/T//G	nonsynonymous	1	0
ALKBH8	chr11	107393275/G//A	nonsynonymous	1	0
ATM	chr11	108224518/C//G	nonsynonymous	1	0
DIXDC1	chr11	111888609/G//T	nonsynonymous	1	0
HTR3A	chr11	113846017/C//A	synonymous	1	0
ZBTB16	chr11	114121323/G//C	synonymous	1	0
NXPE1	chr11	114393059/C//A	synonymous	1	0
PCSK7	chr11	117097897/C//A	nonsynonymous	1	0
CEP164	chr11	117233192/C//T	nonsynonymous	1	0
CEP164	chr11	117261811/G//T	nonsynonymous	1	0
DSCAML1	chr11	117302371/G//T	synonymous	1	0
TTC36	chr11	118398324/G//A	nonsynonymous	1	0
BCL9L	chr11	118772545/C//A	nonsynonymous	1	0
DPAGT1	chr11	118967976/T//C	nonsynonymous	1	0
HINFP	chr11	119005064/C//T	synonymous	1	0
CCDC153	chr11	119061132/C//A	synonymous	1	0
MFRP	chr11	119216997/G//T	nonsynonymous	1	0
NECTIN1	chr11	119549447/G//A	synonymous	1	0
TRIM29	chr11	120008359/C//A	synonymous	1	0
TECTA	chr11	120989065/G//T	nonsynonymous	1	0
SORL1	chr11	121485676/G//C	nonsynonymous	1	0
GRAMD1B	chr11	123448259/G//T	nonsynonymous	1	0
GRAMD1B	chr11	123466731/A//G	nonsynonymous	1	0
OR6M1	chr11	123676929/G//T	synonymous	1	0
OR10G4	chr11	123886301/T//A	nonsynonymous	1	0
OR10G9	chr11	123894411/A//G	nonsynonymous	1	0
OR8G1	chr11	124120552/G//T	nonsynonymous	1	0
OR8D1	chr11	124179974/G//T	nonsynonymous	1	0
OR8D1	chr11	124180066/A//T	synonymous	1	0
OR8B12	chr11	124413090/C//A	nonsynonymous	1	0
HEPACAM	chr11	124793778/G//T	nonsynonymous	1	0
CCDC15	chr11	124908487/A//G	nonsynonymous	1	0
PATE1	chr11	125617639/G//A	nonsynonymous	1	0
PATE2	chr11	125647880/A//T	nonsynonymous	1	0
	chr11	128807380/A//T	synonymous	1	0
ST14	chr11	130067783/G//T	nonsynonymous	1	0
ST14	chr11	130067792/T//A	nonsynonymous	1	0
ADAMTS8	chr11	130278773/C//T	nonsynonymous	1	0
NTM	chr11	132204979/G//T	nonsynonymous	1	0
GLB1L3	chr11	134151949/G//T	synonymous	1	0
GLB1L3	chr11	134182264/G//T	nonsynonymous	1	0
GLB1L2	chr11	134201992/A//T	nonsynonymous	1	0
LRTM2	chr12	1943596/A//T	synonymous	1	0
CCND2	chr12	4398124/G//T	nonsynonymous	1	0
ANO2	chr12	6031811/C//A	nonsynonymous	1	0
VWF	chr12	6105322/C//A	nonsynonymous	1	0
NOP2	chr12	6669641/T//G	nonsynonymous	1	0
LPAR5	chr12	6729130/TC//T	synonymous	1	0
P3H3	chr12	6940423/G//C	synonymous	1	0
P3H3	chr12	6948587/A//T	nonsynonymous	1	0
USP5	chr12	6974360/G//C	nonsynonymous	1	0
CD163L1	chr12	7556166/C//G	nonsynonymous	1	0
GDF3	chr12	7842966/G//A	synonymous	1	0
RIMKLB	chr12	8906546/G//T	nonsynonymous	1	0
RIMKLB	chr12	8926141/C//A	nonsynonymous	1	0
PHC1	chr12	9072434/A//G	nonsynonymous	1	0
A2M	chr12	9252033/C//T	nonsynonymous	1	0
A2M	chr12	9268386/G//T	synonymous	1	0
TAS2R19	chr12	11174782/A//G	nonsynonymous	1	0

TAS2R42	chr12	11338815/G//A	synonymous	1	0
CREBL2	chr12	12788817/G//T	nonsynonymous	1	0
CDKN1B	chr12	12871149/G//A	nonsynonymous	1	0
GUCY2C	chr12	14849249/G//C	nonsynonymous	1	0
PDE3A	chr12	20522704/G//T	synonymous	1	0
SLCO1B1	chr12	21350032/C//A	nonsynonymous	1	0
SLCO1B1	chr12	21355424/G//T	nonsynonymous	1	0
SLCO1B1	chr12	21370223/C//A	synonymous	1	0
CMAS	chr12	22199282/G//T	synonymous	1	0
ST8SIA1	chr12	22408213/C//G	nonsynonymous	1	0
MRPS35	chr12	27877032/G//T	nonsynonymous	1	0
KLHL42	chr12	27950851/G//A	nonsynonymous	1	0
	chr12	30829874/T//C	synonymous	1	0
DENND5B	chr12	31579256/C//G	nonsynonymous	1	0
H3F3C	chr12	31944940/T//C	nonsynonymous	1	0
PKP2	chr12	32949213/A//G	synonymous	1	0
SYT10	chr12	33535343/G//T	nonsynonymous	1	0
SYT10	chr12	33560162/G//T	synonymous	1	0
PDZRN4	chr12	41967242/G//T	nonsynonymous	1	0
ADAMTS20	chr12	43822166/C//A	nonsynonymous	1	0
PUS7L	chr12	44148690/T//C	nonsynonymous	1	0
ADCY6	chr12	49165633/A//G	nonsynonymous	1	0
KMT2D	chr12	49420273/C//A	nonsynonymous	1	0
KMT2D	chr12	49427265/TTGCTGCTGC//T	nonsynonymous	1	0
KMT2D	chr12	49443597/C//A	synonymous	1	0
LOC101927267	chr12	49690606/G//A	synonymous	1	0
AQP6	chr12	50369418/G//C	nonsynonymous	1	0
SLC4A8	chr12	51851227/C//A	nonsynonymous	1	0
SCN8A	chr12	52182495/G//T	nonsynonymous	1	0
ACVRL1	chr12	52306905/G//T	synonymous	1	0
	chr12	52376534/G//C	synonymous	1	0
EIF4B	chr12	53400213/A//G	synonymous	1	0
PDE1B	chr12	54971053/G//T	nonsynonymous	1	0
NEUROD4	chr12	55420479/A//T	nonsynonymous	1	0
OR6C70	chr12	55863137/C//G	synonymous	1	0
OR6C68	chr12	55886917/C//G	nonsynonymous	1	0
SMARCC2	chr12	56571855/C//T	nonsynonymous	1	0
ATP5B	chr12	57037360/C//A	nonsynonymous	1	0
LRP1	chr12	57606218/C//T	synonymous	1	0
R3HDM2	chr12	57682815/C//A	nonsynonymous	1	0
GLI1	chr12	57863246/C//T	synonymous	1	0
MBD6	chr12	57918764/T//C	nonsynonymous	1	0
OS9	chr12	58110298/G//A	synonymous	1	0
USP15	chr12	62778029/A//T	nonsynonymous	1	0
MON2	chr12	62949796/G//T	nonsynonymous	1	0
SRGAP1	chr12	64456709/G//T	nonsynonymous	1	0
LEMD3	chr12	65639941/G//T	nonsynonymous	1	0
MSRB3	chr12	65857048/C//A	synonymous	1	0
HMGA2	chr12	66221817/G//T	nonsynonymous	1	0
PTPRB	chr12	70980817/T//A	nonsynonymous	1	0
PTPRR	chr12	71078479/C//A	synonymous	1	0
TPH2	chr12	72335408/G//T	nonsynonymous	1	0
TPH2	chr12	72338125/A//T	nonsynonymous	1	0
TPH2	chr12	72425355/AC//A	nonsynonymous	1	0
TRHDE	chr12	72969035/A//T	nonsynonymous	1	0
KCNC2	chr12	75444843/G//A	synonymous	1	0
KCNC2	chr12	75601576/G//T	nonsynonymous	1	0
NAV3	chr12	78594354/G//A	nonsynonymous	1	0
NAV3	chr12	78598916/C//G	nonsynonymous	1	0
OTOGL	chr12	80672820/AG//A	nonsynonymous	1	0
PTPRQ	chr12	81072849/G//C	synonymous	1	0
MYF6	chr12	81102695/G//T	nonsynonymous	1	0
MYF6	chr12	81102696/A//T	nonsynonymous	1	0
PPFIA2	chr12	81688615/G//T	nonsynonymous	1	0
PPFIA2	chr12	81769636/A//G	nonsynonymous	1	0
METTL25	chr12	82780637/G//T	synonymous	1	0
MGAT4C	chr12	86373411/C//A	nonsynonymous	1	0
CCER1	chr12	91347974/G//T	synonymous	1	0
KERA	chr12	91449666/A//G	synonymous	1	0
EEA1	chr12	93221789/C//A	nonsynonymous	1	0

VEZT	chr12	95676371/C//T	nonsynonymous	1	0
APAF1	chr12	99102402/A//G	nonsynonymous	1	0
GAS2L3	chr12	101018272/A//T	synonymous	1	0
ANO4	chr12	101442156/T//C	nonsynonymous	1	0
ANO4	chr12	101491493/A//T	synonymous	1	0
UTP20	chr12	101731826/C//T	synonymous	1	0
SPIC	chr12	101876609/C//G	nonsynonymous	1	0
NUP37	chr12	102468233/C//G	nonsynonymous	1	0
ASCL1	chr12	103352554/G//T	nonsynonymous	1	0
C12orf42	chr12	103696026/G//C	nonsynonymous	1	0
STAB2	chr12	104140399/C//G	nonsynonymous	1	0
BTBD11	chr12	108011206/G//C	nonsynonymous	1	0
ASCL4	chr12	108169066/C//A	nonsynonymous	1	0
SART3	chr12	108931945/G//C	nonsynonymous	1	0
	chr12	109185927/G//C	synonymous	1	0
MYO1H	chr12	109849759/G//T	nonsynonymous	1	0
ATXN2	chr12	112037230/G//A	nonsynonymous	1	0
ACAD10	chr12	112150418/C//T	synonymous	1	0
RBM19	chr12	114385265/G//A	synonymous	1	0
TBX3	chr12	115110115/G//A	synonymous	1	0
TBX3	chr12	115117302/G//C	synonymous	1	0
NOS1	chr12	117657889/G//A	synonymous	1	0
NOS1	chr12	117698385/C//A	nonsynonymous	1	0
NOS1	chr12	117768189/G//T	nonsynonymous	1	0
NOS1	chr12	117768678/G//T	nonsynonymous	1	0
KSR2	chr12	117922343/G//A	synonymous	1	0
SRRM4	chr12	119552176/G//T	synonymous	1	0
CIT	chr12	120128029/C//A	nonsynonymous	1	0
PXN	chr12	120651717/G//A	synonymous	1	0
	chr12	121088351/G//A	synonymous	1	0
ACADS	chr12	121163547/C//T	synonymous	1	0
ACADS	chr12	121163551/C//T	synonymous	1	0
RNF34	chr12	121861441/G//T	synonymous	1	0
PSMD9	chr12	122326724/C//T	synonymous	1	0
MLXIP	chr12	122614647/C//T	nonsynonymous	1	0
CCDC62	chr12	123270291/C//G	nonsynonymous	1	0
PITPNM2	chr12	123494534/C//A	nonsynonymous	1	0
DDX55	chr12	124086680/G//T	synonymous	1	0
TMEM132C	chr12	128899735/C//A	synonymous	1	0
SLC15A4	chr12	129283887/C//A	nonsynonymous	1	0
TMEM132D	chr12	130184999/C//CT	nonsynonymous	1	0
ADGRD1	chr12	131490549/A//C	nonsynonymous	1	0
ADGRD1	chr12	131490566/G//A	synonymous	1	0
POLE	chr12	133249241/C//A	nonsynonymous	1	0
GOLGA3	chr12	133393193/C//A	nonsynonymous	1	0
ZNF268	chr12	133781105/G//T	nonsynonymous	1	0
LATS2	chr13	21549504/C//G	nonsynonymous	1	0
LNX2	chr13	28130387/C//A	nonsynonymous	1	0
STARD13	chr13	33685942/C//T	nonsynonymous	1	0
POSTN	chr13	38160353/G//T	nonsynonymous	1	0
FREM2	chr13	39264676/G//T	nonsynonymous	1	0
TNFSF11	chr13	43148532/C//G	nonsynonymous	1	0
SPERT	chr13	46276932/G//C	nonsynonymous	1	0
CAB39L	chr13	49918344/C//T	nonsynonymous	1	0
TRIM13	chr13	50586977/C//G	nonsynonymous	1	0
FAM124A	chr13	51855302/C//A	synonymous	1	0
ATP7B	chr13	52548881/T//A	nonsynonymous	1	0
PCDH8	chr13	53421216/C//T	synonymous	1	0
PCDH20	chr13	61985597/T//A	nonsynonymous	1	0
DIS3	chr13	73347866/T//G	nonsynonymous	1	0
SLITRK1	chr13	84453499/G//C	synonymous	1	0
SLITRK1	chr13	84454830/G//T	synonymous	1	0
UGGT2	chr13	96592252/C//A	nonsynonymous	1	0
HS6ST3	chr13	97485380/G//T	nonsynonymous	1	0
NALCN	chr13	101755645/C//G	nonsynonymous	1	0
TPP2	chr13	103288034/A//T	nonsynonymous	1	0
CCDC168	chr13	103394128/C//A	synonymous	1	0
BIVM-ERCC5	chr13	103510765/A//G	synonymous	1	0
PROZ	chr13	113814337/C//A	nonsynonymous	1	0
GRK1	chr13	114322206/C//A	nonsynonymous	1	0

OR4K5	chr14	20389206/C//A	synonymous	1	0
OR4K15	chr14	20444472/T//A	synonymous	1	0
OR11H4	chr14	20711804/C//A	nonsynonymous	1	0
TEP1	chr14	20849105/C//A	nonsynonymous	1	0
APEX1	chr14	20924985/C//T	synonymous	1	0
PNP	chr14	20943396/G//T	nonsynonymous	1	0
OR6S1	chr14	21108975/G//T	synonymous	1	0
SALL2	chr14	21993414/T//C	nonsynonymous	1	0
OR10G3	chr14	22038589/C//G	nonsynonymous	1	0
AJUBA	chr14	23442653/C//A	nonsynonymous	1	0
CDH24	chr14	23517630/C//A	synonymous	1	0
CDH24	chr14	23524925/C//T	synonymous	1	0
SLC22A17	chr14	23816933/C//A	nonsynonymous	1	0
PSME1	chr14	24607318/G//A	nonsynonymous	1	0
EMC9	chr14	24610492/C//T	nonsynonymous	1	0
REC8	chr14	24646668/C//A	synonymous	1	0
TGM1	chr14	24724011/T//A	synonymous	1	0
PRKD1	chr14	30046410/C//A	synonymous	1	0
HECTD1	chr14	31618140/T//A	nonsynonymous	1	0
AKAP6	chr14	33291860/G//A	nonsynonymous	1	0
NPAS3	chr14	34029334/A//T	nonsynonymous	1	0
MDGA2	chr14	47426572/C//T	synonymous	1	0
KLHDC1	chr14	50177065/C//T	nonsynonymous	1	0
ABHD12B	chr14	51345532/T//A	nonsynonymous	1	0
TRIM9	chr14	51444074/G//A	synonymous	1	0
PTGER2	chr14	52781547/C//A	nonsynonymous	1	0
CCDC175	chr14	59988395/C//A	nonsynonymous	1	0
RTN1	chr14	60193636/C//A	nonsynonymous	1	0
KCNH5	chr14	63269087/G//T	synonymous	1	0
SYNE2	chr14	64547323/G//A	synonymous	1	0
ESR2	chr14	64701719/G//T	nonsynonymous	1	0
ESR2	chr14	64727259/G//T	nonsynonymous	1	0
HSPA2	chr14	65008481/T//C	nonsynonymous	1	0
FUT8	chr14	66028211/A//G	synonymous	1	0
ZFYVE26	chr14	68222714/T//A	nonsynonymous	1	0
ZFYVE26	chr14	68268852/A//G	nonsynonymous	1	0
ERH	chr14	69861635/G//A	synonymous	1	0
SLC8A3	chr14	70634521/C//T	nonsynonymous	1	0
SIPA1L1	chr14	72205845/A//G	synonymous	1	0
PROX2	chr14	75329523/G//T	nonsynonymous	1	0
ACYP1	chr14	75530186/C//T	nonsynonymous	1	0
FOS	chr14	75746734/C//A	nonsynonymous	1	0
NRXN3	chr14	80164281/G//T	nonsynonymous	1	0
SEL1L	chr14	81993082/T//A	nonsynonymous	1	0
PTPN21	chr14	88945852/G//A	synonymous	1	0
UNC79	chr14	94173301/T//A	synonymous	1	0
SERPINA12	chr14	94955997/A//T	nonsynonymous	1	0
SERPINA5	chr14	95054061/C//A	nonsynonymous	1	0
ATG2B	chr14	96777955/T//C	nonsynonymous	1	0
AK7	chr14	96949469/G//T	synonymous	1	0
BCL11B	chr14	99641393/C//A	nonsynonymous	1	0
HHIPL1	chr14	100134606/C//A	nonsynonymous	1	0
ANKRD9	chr14	102973775/C//A	nonsynonymous	1	0
AHNAK2	chr14	105411664/T//A	nonsynonymous	1	0
AHNAK2	chr14	105415040/C//T	nonsynonymous	1	0
C14orf80	chr14	105964992/G//T	synonymous	1	0
NPAP1	chr15	24924492/C//A	synonymous	1	0
HERC2	chr15	28459400/C//A	nonsynonymous	1	0
CHRM5	chr15	34355377/G//T	nonsynonymous	1	0
KNL1	chr15	40914184/G//A	synonymous	1	0
MAP1A	chr15	43818747/A//C	nonsynonymous	1	0
EIF3J	chr15	44852463/G//C	nonsynonymous	1	0
DUOX2	chr15	45389858/C//T	nonsynonymous	1	0
SEMA6D	chr15	48054389/G//T	synonymous	1	0
SLC12A1	chr15	48499924/T//G	nonsynonymous	1	0
FBN1	chr15	48808560/C//G	nonsynonymous	1	0
MYO5A	chr15	52821026/C//A	synonymous	1	0
MYO1E	chr15	59480345/G//GA	nonsynonymous	1	0
HERC1	chr15	63958255/C//G	synonymous	1	0
HERC1	chr15	64017564/C//A	nonsynonymous	1	0

CSNK1G1	chr15	64506312/C//A	nonsynonymous	1	0
TRIP4	chr15	64701827/A//T	nonsynonymous	1	0
ANKDD1A	chr15	65209588/G//T	nonsynonymous	1	0
IGDCC3	chr15	65621762/G//C	nonsynonymous	1	0
CALML4	chr15	68489856/C//T	nonsynonymous	1	0
SPESP1	chr15	69238241/C//T	nonsynonymous	1	0
KIF23	chr15	69714356/G//T	synonymous	1	0
UACA	chr15	70976750/C//A	nonsynonymous	1	0
THSD4	chr15	72057496/C//G	synonymous	1	0
HEXA	chr15	72642949/C//T	nonsynonymous	1	0
MAN2C1	chr15	75658909/T//A	nonsynonymous	1	0
PEAK1	chr15	77473368/G//C	nonsynonymous	1	0
SH2D7	chr15	78390365/G//C	nonsynonymous	1	0
KIAA1024	chr15	79749899/C//A	nonsynonymous	1	0
CEMIP	chr15	81173261/C//A	nonsynonymous	1	0
EFL1	chr15	82530655/C//A	nonsynonymous	1	0
BTBD1	chr15	83735804/G//C	nonsynonymous	1	0
ADAMTSL3	chr15	84659837/G//A	nonsynonymous	1	0
ZNF592	chr15	85345616/C//G	nonsynonymous	1	0
AKAP13	chr15	86262351/A//C	nonsynonymous	1	0
NTRK3	chr15	88669501/C//A	nonsynonymous	1	0
NTRK3	chr15	88678614/C//A	nonsynonymous	1	0
DET1	chr15	89074850/T//A	synonymous	1	0
ACAN	chr15	89398247/C//A	nonsynonymous	1	0
HAPLN3	chr15	89430551/G//A	synonymous	1	0
RHCG	chr15	90020401/C//G	synonymous	1	0
BLM	chr15	91306241/G//T	nonsynonymous	1	0
	chr15	96869512/G//T	synonymous	1	0
SPATA8	chr15	97326950/C//G	nonsynonymous	1	0
CERS3	chr15	100942919/T//C	nonsynonymous	1	0
CHSY1	chr15	101775343/C//T	nonsynonymous	1	0
POLR3K	chr16	97503/C//A	nonsynonymous	1	0
AXIN1	chr16	359994/C//A	synonymous	1	0
RHOT2	chr16	720248/G//T	nonsynonymous	1	0
RHOT2	chr16	723098/C//G	nonsynonymous	1	0
RHOT2	chr16	723608/G//T	synonymous	1	0
JMJD8	chr16	734140/C//A	synonymous	1	0
FBXL16	chr16	746768/C//T	synonymous	1	0
UNKL	chr16	1421514/C//A	synonymous	1	0
UNKL	chr16	1453234/C//A	nonsynonymous	1	0
TBL3	chr16	2027641/G//A	synonymous	1	0
PKD1	chr16	2159393/C//A	synonymous	1	0
PKD1	chr16	2161498/C//A	nonsynonymous	1	0
ZNF213	chr16	3190814/C//T	synonymous	1	0
SLX4	chr16	3647660/G//A	nonsynonymous	1	0
ADCY9	chr16	4016883/G//C	synonymous	1	0
ADCY9	chr16	4033442/C//A	nonsynonymous	1	0
CORO7-PAM16	chr16	4405287/C//G	nonsynonymous	1	0
C16orf96	chr16	4638321/G//T	nonsynonymous	1	0
C16orf71	chr16	4793068/G//T	nonsynonymous	1	0
NAGPA-AS1	chr16	5083946/C//T	synonymous	1	0
RBFOX1	chr16	7629883/C//A	synonymous	1	0
PMM2	chr16	8941684/G//T	synonymous	1	0
GSPT1	chr16	11980389/G//C	nonsynonymous	1	0
SNX29	chr16	12450060/G//T	nonsynonymous	1	0
SNX29	chr16	12450130/G//T	synonymous	1	0
CPPED1	chr16	12897565/T//A	synonymous	1	0
SHISA9	chr16	12996146/G//A	synonymous	1	0
ERCC4	chr16	14041558/G//T	nonsynonymous	1	0
SMG1	chr16	18858914/C//A	nonsynonymous	1	0
GPR139	chr16	20043223/G//T	nonsynonymous	1	0
GP2	chr16	20334225/C//A	nonsynonymous	1	0
GP2	chr16	20335256/G//T	synonymous	1	0
UMOD	chr16	20352607/C//A	synonymous	1	0
ANKS4B	chr16	21261347/C//A	nonsynonymous	1	0
EEF2K	chr16	22277761/G//C	synonymous	1	0
COG7	chr16	23428424/C//G	nonsynonymous	1	0
HS3ST4	chr16	26147506/C//A	nonsynonymous	1	0
KDM8	chr16	27224957/G//A	nonsynonymous	1	0
NSMCE1	chr16	27268778/C//A	synonymous	1	0

ATP2A1	chr16	28909713/A//C	nonsynonymous	1	0
SPNS1	chr16	28992845/G//T	nonsynonymous	1	0
SEZ6L2	chr16	29900018/G//A	synonymous	1	0
ITGAL	chr16	30484220/G//A	nonsynonymous	1	0
ZNF688	chr16	30582359/C//A	nonsynonymous	1	0
SRCAP	chr16	30734517/A//C	nonsynonymous	1	0
SRCAP	chr16	30734519/G//T	nonsynonymous	1	0
SRCAP	chr16	30747669/A//T	nonsynonymous	1	0
SETD1A	chr16	30976455/C//T	synonymous	1	0
ITGAX	chr16	31366590/G//A	synonymous	1	0
ITGAD	chr16	31419164/G//A	synonymous	1	0
C16orf58	chr16	31508278/G//T	synonymous	1	0
VPS35	chr16	46705610/C//A	synonymous	1	0
VPS35	chr16	46708293/G//T	nonsynonymous	1	0
MYLK3	chr16	46766530/G//A	nonsynonymous	1	0
LONP2	chr16	48278443/G//T	nonsynonymous	1	0
LONP2	chr16	48296701/G//C	nonsynonymous	1	0
ZNF423	chr16	49671987/G//A	nonsynonymous	1	0
ZNF423	chr16	49672519/C//T	nonsynonymous	1	0
NOD2	chr16	50731185/G//T	nonsynonymous	1	0
NOD2	chr16	50745329/G//T	nonsynonymous	1	0
SALL1	chr16	51174769/GC//G	nonsynonymous	1	0
CHD9	chr16	53319515/GA//G	nonsynonymous	1	0
RPGRIP1L	chr16	53708937/G//A	nonsynonymous	1	0
NUP93	chr16	56865832/G//T	synonymous	1	0
NUP93	chr16	56867263/G//T	synonymous	1	0
CX3CL1	chr16	57416770/G//T	nonsynonymous	1	0
ADGRG5	chr16	57597045/C//T	synonymous	1	0
ADGRG1	chr16	57685160/G//T	nonsynonymous	1	0
DRC7	chr16	57741396/G//T	nonsynonymous	1	0
CNGB1	chr16	57917989/G//T	synonymous	1	0
CNGB1	chr16	57965695/G//C	nonsynonymous	1	0
NDRG4	chr16	58540381/T//A	synonymous	1	0
GOT2	chr16	58750025/T//C	synonymous	1	0
GOT2	chr16	58750581/T//C	nonsynonymous	1	0
CDH16	chr16	66950251/T//C	nonsynonymous	1	0
FBXL8	chr16	67197688/G//T	nonsynonymous	1	0
EXOC3L1	chr16	67218408/C//A	synonymous	1	0
PSMB10	chr16	67969577/G//A	nonsynonymous	1	0
NOB1	chr16	69782838/T//C	nonsynonymous	1	0
DDX19B	chr16	70359501/G//C	nonsynonymous	1	0
VAC14	chr16	70778345/G//A	synonymous	1	0
HYDIN	chr16	70893981/T//C	nonsynonymous	1	0
HYDIN	chr16	71054131/A//T	synonymous	1	0
CALB2	chr16	71411620/G//T	nonsynonymous	1	0
ZNF23	chr16	71482490/C//A	nonsynonymous	1	0
ZNF19	chr16	71509461/G//A	nonsynonymous	1	0
PHLPP2	chr16	71712864/G//A	synonymous	1	0
PKD1L3	chr16	72012187/C//A	nonsynonymous	1	0
ZFHX3	chr16	72993519/C//A	nonsynonymous	1	0
CFDP1	chr16	75467340/C//A	synonymous	1	0
	chr16	75678303/C//A	synonymous	1	0
CNTNAP4	chr16	76350418/A//T	nonsynonymous	1	0
CNTNAP4	chr16	76482815/G//A	synonymous	1	0
VAT1L	chr16	77822687/C//CA	nonsynonymous	1	0
C16orf46	chr16	81097443/G//A	nonsynonymous	1	0
CDH13	chr16	83520145/T//A	nonsynonymous	1	0
EMC8	chr16	85822616/C//A	nonsynonymous	1	0
PIEZO1	chr16	88808793/G//A	synonymous	1	0
DPEP1	chr16	89704240/A//T	synonymous	1	0
TCF25	chr16	89973708/T//G	nonsynonymous	1	0
RILP	chr17	1552950/G//T	nonsynonymous	1	0
OR1D2	chr17	2995605/G//T	nonsynonymous	1	0
OR3A3	chr17	3324517/C//A	nonsynonymous	1	0
TRPV3	chr17	3432251/C//G	synonymous	1	0
ITGAE	chr17	3680859/G//A	nonsynonymous	1	0
PELP1	chr17	4576658/C//A	nonsynonymous	1	0
USP6	chr17	5076110/C//A	nonsynonymous	1	0
ZNF594	chr17	5086176/G//A	nonsynonymous	1	0
LOC728392	chr17	5403911/G//C	synonymous	1	0

NLRP1	chr17	5462784/C//A	nonsynonymous	1	0
BCL6B	chr17	6930038/C//A	nonsynonymous	1	0
ACADVL	chr17	7125004/G//A	synonymous	1	0
TMEM256	chr17	7307433/G//A	synonymous	1	0
POLR2A	chr17	7417135/C//T	nonsynonymous	1	0
TP53	chr17	7577129/A//C	nonsynonymous	1	0
TP53	chr17	7578457/C//A	nonsynonymous	1	0
DNAH2	chr17	7643196/G//T	nonsynonymous	1	0
CHD3	chr17	7814274/C//T	nonsynonymous	1	0
ALOX15B	chr17	7942620/T//G	synonymous	1	0
ALOX15B	chr17	7943235/C//A	nonsynonymous	1	0
ALOX12B	chr17	7980329/G//T	synonymous	1	0
AURKB	chr17	8110631/C//A	nonsynonymous	1	0
PIK3R6	chr17	8730608/G//C	nonsynonymous	1	0
MYH13	chr17	10210265/G//T	synonymous	1	0
MYH13	chr17	10250042/C//T	synonymous	1	0
MYH8	chr17	10297646/C//A	nonsynonymous	1	0
MYH8	chr17	10323386/A//G	synonymous	1	0
MYH1	chr17	10401056/C//G	nonsynonymous	1	0
PIRT	chr17	10728537/C//A	synonymous	1	0
ARHGAP44	chr17	12893345/C//G	synonymous	1	0
TEKT3	chr17	15231396/C//A	synonymous	1	0
UBB	chr17	16285415/C//G	nonsynonymous	1	0
UBB	chr17	16285615/C//A	nonsynonymous	1	0
FAM83G	chr17	18881321/G//A	nonsynonymous	1	0
NOS2	chr17	26101295/G//C	nonsynonymous	1	0
NOS2	chr17	26107896/G//T	nonsynonymous	1	0
VTN	chr17	26696823/A//T	nonsynonymous	1	0
ALDOC	chr17	26901161/G//A	synonymous	1	0
TLCD1	chr17	27051713/G//C	nonsynonymous	1	0
SLC6A4	chr17	28542646/G//A	synonymous	1	0
NF1	chr17	29556863/C//A	nonsynonymous	1	0
NF1	chr17	29687566/C//T	nonsynonymous	1	0
C17orf75	chr17	30665233/A//T	nonsynonymous	1	0
SLFN11	chr17	33690306/T//C	nonsynonymous	1	0
CCL23	chr17	34340208/G//T	synonymous	1	0
ACACA	chr17	35562762/T//A	nonsynonymous	1	0
ARHGAP23	chr17	36623553/G//T	synonymous	1	0
PLXDC1	chr17	37235352/C//G	synonymous	1	0
KRT26	chr17	38927963/T//A	nonsynonymous	1	0
KRTAP16-1	chr17	39464493/C//A	nonsynonymous	1	0
KRT33A	chr17	39504790/G//T	nonsynonymous	1	0
KRT38	chr17	39596984/G//T	nonsynonymous	1	0
KRT36	chr17	39646037/C//A	nonsynonymous	1	0
KRT15	chr17	39672192/T//A	nonsynonymous	1	0
KRT15	chr17	39672368/C//A	nonsynonymous	1	0
KRT15	chr17	39673158/G//C	nonsynonymous	1	0
KRT16	chr17	39766247/C//A	nonsynonymous	1	0
GAST	chr17	39871744/C//A	nonsynonymous	1	0
CNP	chr17	40129366/G//C	synonymous	1	0
DHX58	chr17	40257080/C//A	nonsynonymous	1	0
KCNH4	chr17	40315650/G//A	synonymous	1	0
GHDC	chr17	40342735/C//T	nonsynonymous	1	0
PTRF	chr17	40556818/C//A	nonsynonymous	1	0
PTGES3L-AARSD1	chr17	41131270/G//A	nonsynonymous	1	0
MEOX1	chr17	41738499/C//A	nonsynonymous	1	0
NAGS	chr17	42083516/G//T	nonsynonymous	1	0
SLC4A1	chr17	42328934/G//A	synonymous	1	0
SLC4A1	chr17	42335414/C//A	nonsynonymous	1	0
ITGA2B	chr17	42457485/G//T	nonsynonymous	1	0
DBF4B	chr17	42828488/A//G	nonsynonymous	1	0
ADAM11	chr17	42847393/GA//G	nonsynonymous	1	0
KANSL1	chr17	44115913/G//A	synonymous	1	0
B4GALNT2	chr17	47237983/A//T	nonsynonymous	1	0
SLC35B1	chr17	47778773/G//C	synonymous	1	0
CACNA1G	chr17	48653158/C//T	synonymous	1	0
KIF2B	chr17	51901604/G//C	nonsynonymous	1	0
NOG	chr17	54671882/G//T	nonsynonymous	1	0
TRIM25	chr17	54969303/C//A	nonsynonymous	1	0
MPO	chr17	56353051/G//T	nonsynonymous	1	0

TRIM37	chr17	57078967/G//C	nonsynonymous	1	0
TRIM37	chr17	57139927/C//T	nonsynonymous	1	0
TBX2	chr17	59485543/C//A	synonymous	1	0
EFCAB3	chr17	60491093/G//T	nonsynonymous	1	0
TANC2	chr17	61432714/G//A	nonsynonymous	1	0
SCN4A	chr17	62018656/G//C	nonsynonymous	1	0
SCN4A	chr17	62020340/G//T	nonsynonymous	1	0
POLG2	chr17	62474014/T//C	nonsynonymous	1	0
ARSG	chr17	66339930/T//C	nonsynonymous	1	0
ABCA8	chr17	66920894/G//A	nonsynonymous	1	0
ABCA6	chr17	67080565/G//A	synonymous	1	0
KCNJ2	chr17	68172019/A//G	nonsynonymous	1	0
SDK2	chr17	71412066/T//C	nonsynonymous	1	0
	chr17	72245124/A//G	synonymous	1	0
KIF19	chr17	72345392/G//T	nonsynonymous	1	0
TMEM104	chr17	72832699/G//A	nonsynonymous	1	0
FADS6	chr17	72877274/G//A	nonsynonymous	1	0
OTOP3	chr17	72942980/G//T	nonsynonymous	1	0
HID1	chr17	72947628/G//A	synonymous	1	0
HID1	chr17	72960657/A//G	nonsynonymous	1	0
CDR2L	chr17	72998255/G//A	synonymous	1	0
NT5C	chr17	73127749/G//A	synonymous	1	0
ZACN	chr17	74075387/G//T	synonymous	1	0
JMJD6	chr17	74716473/G//C	nonsynonymous	1	0
TK1	chr17	76170840/T//G	nonsynonymous	1	0
PGS1	chr17	76399952/A//G	nonsynonymous	1	0
DNAH17	chr17	76522754/G//C	nonsynonymous	1	0
RPTOR	chr17	78882720/C//T	synonymous	1	0
CEP131	chr17	79180542/A//G	nonsynonymous	1	0
TEPSIN	chr17	79202762/G//A	nonsynonymous	1	0
BAHCC1	chr17	79425466/G//A	nonsynonymous	1	0
SLC25A10	chr17	79684060/A//T	synonymous	1	0
SLC25A10	chr17	79684441/G//T	nonsynonymous	1	0
P4HB	chr17	79801796/T//A	synonymous	1	0
	chr17	79857322/C//T	synonymous	1	0
FASN	chr17	80050393/C//G	nonsynonymous	1	0
CCDC57	chr17	80151999/T//A	nonsynonymous	1	0
NARF	chr17	80430441/G//T	synonymous	1	0
EPB41L3	chr18	5416317/T//C	nonsynonymous	1	0
MTCL1	chr18	8826213/G//A	nonsynonymous	1	0
RAB31	chr18	9859237/C//T	nonsynonymous	1	0
PIEZO2	chr18	10672698/C//A	synonymous	1	0
ANKRD30B	chr18	14752838/G//T	nonsynonymous	1	0
GAREM1	chr18	29848246/T//A	nonsynonymous	1	0
ASXL3	chr18	31326400/C//T	synonymous	1	0
NOL4	chr18	31709981/C//A	nonsynonymous	1	0
C18orf25	chr18	43820053/G//T	synonymous	1	0
TMX3	chr18	66350192/C//A	nonsynonymous	1	0
ZNF407	chr18	72632570/G//A	nonsynonymous	1	0
CTDP1	chr18	77477977/C//T	nonsynonymous	1	0
ATP8B3	chr19	1790823/C//T	nonsynonymous	1	0
ATP8B3	chr19	1805386/C//A	nonsynonymous	1	0
LMNB2	chr19	2434320/C//A	nonsynonymous	1	0
MATK	chr19	3778297/G//A	nonsynonymous	1	0
ANKRD24	chr19	4216281/G//T	nonsynonymous	1	0
SAFB2	chr19	5610094/C//A	nonsynonymous	1	0
DUS3L	chr19	5787321/G//A	nonsynonymous	1	0
ACER1	chr19	6312203/A//G	nonsynonymous	1	0
KHSRP	chr19	6415383/C//T	synonymous	1	0
VAV1	chr19	6854109/G//T	synonymous	1	0
ADGRE1	chr19	6902018/A//T	nonsynonymous	1	0
MCEMP1	chr19	7742534/C//A	nonsynonymous	1	0
PRAM1	chr19	8563430/C//A	nonsynonymous	1	0
MUC16	chr19	9014569/A//G	synonymous	1	0
MUC16	chr19	9046500/T//A	nonsynonymous	1	0
ZNF317	chr19	9268657/G//T	synonymous	1	0
ZNF426	chr19	9639182/G//A	synonymous	1	0
ZNF426	chr19	9641719/T//A	nonsynonymous	1	0
OLFM2	chr19	9968428/C//CGGA	nonsynonymous	1	0
CNN1	chr19	11660410/G//T	nonsynonymous	1	0

CC2D1A	chr19	14023138/C//A	nonsynonymous	1	0
UNC13A	chr19	17735760/G//T	nonsynonymous	1	0
FCHO1	chr19	17898550/G//T	nonsynonymous	1	0
KIAA1683	chr19	18368384/G//A	nonsynonymous	1	0
COMP	chr19	18898363/C//T	nonsynonymous	1	0
COMP	chr19	18899479/G//C	nonsynonymous	1	0
CERS1	chr19	18991133/C//A	synonymous	1	0
MAU2	chr19	19456183/G//T	synonymous	1	0
ZNF90	chr19	20229627/G//T	nonsynonymous	1	0
ZNF85	chr19	21132012/A//C	nonsynonymous	1	0
ZNF100	chr19	21909418/C//A	synonymous	1	0
ZNF100	chr19	21909492/T//A	nonsynonymous	1	0
ZNF729	chr19	22498184/T//C	synonymous	1	0
ZNF536	chr19	30934860/C//A	nonsynonymous	1	0
ZNF536	chr19	30935811/G//T	nonsynonymous	1	0
TSHZ3	chr19	31768674/C//A	synonymous	1	0
ANKRD27	chr19	33095286/C//A	synonymous	1	0
LRP3	chr19	33697950/C//T	synonymous	1	0
SLC7A10	chr19	33702142/G//T	nonsynonymous	1	0
KIAA0355	chr19	34791609/G//T	nonsynonymous	1	0
CD22	chr19	35827175/G//C	nonsynonymous	1	0
ATP4A	chr19	36049533/G//A	synonymous	1	0
PRODH2	chr19	36298003/G//C	synonymous	1	0
KIRREL2	chr19	36351545/C//G	nonsynonymous	1	0
ZNF382	chr19	37118408/G//A	nonsynonymous	1	0
ZNF568	chr19	37427656/G//T	synonymous	1	0
ZNF585A	chr19	37643669/C//A	nonsynonymous	1	0
ZNF571	chr19	38055398/C//G	synonymous	1	0
ZNF540	chr19	38091998/A//G	nonsynonymous	1	0
ZNF573	chr19	38260735/C//T	nonsynonymous	1	0
WDR87	chr19	38376695/C//A	nonsynonymous	1	0
WDR87	chr19	38377985/G//A	nonsynonymous	1	0
WDR87	chr19	38380317/G//T	nonsynonymous	1	0
SIPA1L3	chr19	38572331/G//A	synonymous	1	0
CATSPERG	chr19	38860880/G//T	synonymous	1	0
RYR1	chr19	38949779/G//A	synonymous	1	0
RYR1	chr19	39008114/G//T	synonymous	1	0
RYR1	chr19	39058427/A//T	nonsynonymous	1	0
ECH1	chr19	39322368/C//T	synonymous	1	0
SIRT2	chr19	39374274/C//T	nonsynonymous	1	0
FBXO27	chr19	39521668/C//A	nonsynonymous	1	0
PAK4	chr19	39660254/G//C	nonsynonymous	1	0
IFNL3	chr19	39734529/C//A	nonsynonymous	1	0
ZFP36	chr19	39899149/C//G	nonsynonymous	1	0
PLEKHG2	chr19	39914609/C//A	nonsynonymous	1	0
FCGBP	chr19	40364190/C//G	nonsynonymous	1	0
C19orf47	chr19	40842065/C//A	synonymous	1	0
HIPK4	chr19	40887028/C//T	synonymous	1	0
SPTBN4	chr19	41009752/G//C	nonsynonymous	1	0
SPTBN4	chr19	41019335/C//A	nonsynonymous	1	0
HNRNPUL1	chr19	41800316/C//A	nonsynonymous	1	0
ZNF574	chr19	42583753/G//T	nonsynonymous	1	0
CIC	chr19	42797947/G//T	synonymous	1	0
PSG1	chr19	43375931/G//T	nonsynonymous	1	0
PSG11	chr19	43529054/C//A	nonsynonymous	1	0
ZNF225	chr19	44619934/G//C	synonymous	1	0
ZNF226	chr19	44680409/G//A	nonsynonymous	1	0
ZNF233	chr19	44777937/G//A	nonsynonymous	1	0
ZNF235	chr19	44791615/T//A	nonsynonymous	1	0
ZNF229	chr19	44933116/C//G	nonsynonymous	1	0
ZNF229	chr19	44933153/C//A	synonymous	1	0
PVR	chr19	45150826/C//G	nonsynonymous	1	0
TOMM40	chr19	45396092/A//T	nonsynonymous	1	0
APOE	chr19	45412283/C//T	nonsynonymous	1	0
CLASRP	chr19	45567877/G//T	synonymous	1	0
PPM1N	chr19	46001921/G//A	nonsynonymous	1	0
PPM1N	chr19	46002668/A//G	nonsynonymous	1	0
SNRPD2	chr19	46195241/C//T	synonymous	1	0
FBXO46	chr19	46215940/G//A	nonsynonymous	1	0
DMWD	chr19	46289299/G//T	synonymous	1	0

HIF3A	chr19	46811544/G//T	nonsynonymous	1	0
PRKD2	chr19	47204141/C//A	nonsynonymous	1	0
AP2S1	chr19	47341683/C//A	synonymous	1	0
ARHGAP35	chr19	47440562/C//T	synonymous	1	0
C5AR2	chr19	47844084/T//A	nonsynonymous	1	0
LIG1	chr19	48631262/C//A	nonsynonymous	1	0
KDELR1	chr19	48886536/G//T	synonymous	1	0
SULT2B1	chr19	49090537/C//T	nonsynonymous	1	0
SULT2B1	chr19	49094890/C//A	synonymous	1	0
PPP1R15A	chr19	49377789/G//T	synonymous	1	0
TRPM4	chr19	49714531/G//T	synonymous	1	0
BCL2L12	chr19	50173593/G//A	nonsynonymous	1	0
TSKS	chr19	50243091/C//T	nonsynonymous	1	0
TSKS	chr19	50247621/C//T	nonsynonymous	1	0
MED25	chr19	50340195/C//T	synonymous	1	0
PTOV1	chr19	50363310/C//T	nonsynonymous	1	0
VRK3	chr19	50511078/C//A	nonsynonymous	1	0
ZNF473	chr19	50550187/C//G	synonymous	1	0
EMC10	chr19	50979826/A//T	nonsynonymous	1	0
SHANK1	chr19	51192118/C//A	nonsynonymous	1	0
CLEC11A	chr19	51228421/C//G	synonymous	1	0
KLK6	chr19	51465134/C//T	nonsynonymous	1	0
SIGLEC7	chr19	51645629/G//A	nonsynonymous	1	0
CD33	chr19	51729163/C//A	nonsynonymous	1	0
IGLON5	chr19	51827090/C//A	synonymous	1	0
ETFB	chr19	51848526/C//A	nonsynonymous	1	0
LIM2	chr19	51890643/G//T	nonsynonymous	1	0
CEACAM18	chr19	51983775/G//C	nonsynonymous	1	0
SIGLEC6	chr19	52034422/C//G	nonsynonymous	1	0
FPR3	chr19	52327613/C//G	synonymous	1	0
ZNF350	chr19	52469067/T//A	nonsynonymous	1	0
ZNF578	chr19	53008021/C//A	nonsynonymous	1	0
ZNF578	chr19	53014906/G//C	nonsynonymous	1	0
ZNF808	chr19	53058407/G//A	synonymous	1	0
ZNF808	chr19	53058636/G//T	nonsynonymous	1	0
ERVV-1	chr19	53518663/G//C	synonymous	1	0
ZNF160	chr19	53578372/G//A	nonsynonymous	1	0
ZNF665	chr19	53667950/TA//T	nonsynonymous	1	0
ZNF665	chr19	53668867/A//G	synonymous	1	0
VN1R2	chr19	53762662/G//T	nonsynonymous	1	0
BIRC8	chr19	53792999/C//A	nonsynonymous	1	0
ZNF845	chr19	53856308/A//G	nonsynonymous	1	0
LILRB3	chr19	54721227/C//G	nonsynonymous	1	0
LILRA2	chr19	55085566/C//T	nonsynonymous	1	0
LILRA2	chr19	55086222/C//G	nonsynonymous	1	0
LILRA2	chr19	55086840/A//T	nonsynonymous	1	0
LILRB1	chr19	55143550/C//A	nonsynonymous	1	0
LILRB1	chr19	55148182/G//T	nonsynonymous	1	0
KIR2DL3	chr19	55253486/C//G	nonsynonymous	1	0
KIR2DS4	chr19	55350998/A//T	synonymous	1	0
KIR3DL2	chr19	55378172/G//A	nonsynonymous	1	0
NCR1	chr19	55420655/C//T	nonsynonymous	1	0
GP6	chr19	55526059/C//T	synonymous	1	0
BRSK1	chr19	55814070/G//T	nonsynonymous	1	0
ISOC2	chr19	55964713/G//A	synonymous	1	0
SSC5D	chr19	56001678/T//A	synonymous	1	0
SSC5D	chr19	56005047/G//T	synonymous	1	0
SBK2	chr19	56041362/G//T	nonsynonymous	1	0
ZNF865	chr19	56127165/C//G	synonymous	1	0
EPN1	chr19	56203176/A//T	synonymous	1	0
NLRP9	chr19	56249456/C//G	synonymous	1	0
NLRP8	chr19	56485189/G//T	nonsynonymous	1	0
NLRP5	chr19	56538626/T//A	nonsynonymous	1	0
NLRP5	chr19	56538871/C//A	synonymous	1	0
NLRP5	chr19	56544913/G//C	nonsynonymous	1	0
NLRP5	chr19	56544917/T//A	nonsynonymous	1	0
ZNF470	chr19	57088611/G//T	nonsynonymous	1	0
ZNF71	chr19	57132653/G//T	synonymous	1	0
ZIM2	chr19	57286819/G//T	nonsynonymous	1	0
PEG3	chr19	57326681/G//T	nonsynonymous	1	0

DUXA	chr19	57670636/T//C	nonsynonymous	1	0
ZNF805	chr19	57752269/G//T	nonsynonymous	1	0
ZNF548	chr19	57908467/G//T	nonsynonymous	1	0
ZNF416	chr19	58084083/G//C	nonsynonymous	1	0
ZNF134	chr19	58130779/G//T	nonsynonymous	1	0
ZSCAN4	chr19	58187725/C//G	nonsynonymous	1	0
ZSCAN4	chr19	58189279/C//G	synonymous	1	0
ZNF776	chr19	58265601/G//T	nonsynonymous	1	0
ZNF606	chr19	58499575/C//A	nonsynonymous	1	0
	chr19	58574942/C//A	synonymous	1	0
ZSCAN18	chr19	58598731/C//A	nonsynonymous	1	0
ZNF584	chr19	58929136/G//T	nonsynonymous	1	0
MZF1	chr19	59074759/G//C	synonymous	1	0
ATRN	chr20	3526464/A//T	nonsynonymous	1	0
SPEF1	chr20	3758745/C//T	synonymous	1	0
CENPB	chr20	3766334/T//A	nonsynonymous	1	0
RASSF2	chr20	4781654/G//A	nonsynonymous	1	0
LRRN4	chr20	6033059/G//A	synonymous	1	0
HAO1	chr20	7894876/G//C	nonsynonymous	1	0
SNAP25	chr20	10273910/G//C	nonsynonymous	1	0
CFAP61	chr20	20055873/C//T	nonsynonymous	1	0
RALGAPA2	chr20	20582413/T//A	nonsynonymous	1	0
NKX2-4	chr20	21376969/C//A	synonymous	1	0
PAX1	chr20	21687565/C//A	nonsynonymous	1	0
DEFB121	chr20	29993950/C//T	synonymous	1	0
TTLL9	chr20	30527076/A//G	synonymous	1	0
HCK	chr20	30686924/G//C	nonsynonymous	1	0
KIF3B	chr20	30897727/G//T	synonymous	1	0
MAPRE1	chr20	31424498/A//T	nonsynonymous	1	0
PXMP4	chr20	32298566/A//G	synonymous	1	0
DYNLRB1	chr20	33104259/C//T	synonymous	1	0
NDRG3	chr20	35294750/G//A	nonsynonymous	1	0
DSN1	chr20	35395237/A//T	nonsynonymous	1	0
KIAA1755	chr20	36869658/C//T	nonsynonymous	1	0
SLC32A1	chr20	37356570/C//A	nonsynonymous	1	0
PPP1R16B	chr20	37464500/C//G	synonymous	1	0
ACOT8	chr20	44472211/G//A	nonsynonymous	1	0
ZNF335	chr20	44589137/T//C	nonsynonymous	1	0
SLC12A5	chr20	44664035/C//A	synonymous	1	0
SLC12A5	chr20	44664458/C//A	nonsynonymous	1	0
TP53RK	chr20	45315488/C//T	synonymous	1	0
ZMYND8	chr20	45875147/G//C	nonsynonymous	1	0
PREX1	chr20	47248858/C//T	nonsynonymous	1	0
PREX1	chr20	47351188/C//A	nonsynonymous	1	0
DDX27	chr20	47835894/T//A	nonsynonymous	1	0
KCNB1	chr20	48098594/C//A	nonsynonymous	1	0
	chr20	48732129/G//A	synonymous	1	0
NFATC2	chr20	50139984/C//G	nonsynonymous	1	0
PFDN4	chr20	52824668/G//T	synonymous	1	0
DOK5	chr20	53092396/G//T	synonymous	1	0
ZBP1	chr20	56179629/C//G	nonsynonymous	1	0
SYCP2	chr20	58496366/C//A	nonsynonymous	1	0
LAMA5	chr20	60886351/T//A	nonsynonymous	1	0
COL20A1	chr20	61940835/C//A	nonsynonymous	1	0
CHRNA4	chr20	61981435/T//G	nonsynonymous	1	0
KCNQ2	chr20	62038668/T//G	nonsynonymous	1	0
KCNQ2	chr20	62044826/G//A	synonymous	1	0
KCNQ2	chr20	62078168/G//T	nonsynonymous	1	0
POTED	chr21	14982850/T//A	nonsynonymous	1	0
SAMSN1	chr21	15872855/G//C	nonsynonymous	1	0
BTG3	chr21	18977207/G//A	synonymous	1	0
ATP5J	chr21	27096928/C//A	synonymous	1	0
KRTAP25-1	chr21	31661555/G//T	nonsynonymous	1	0
KRTAP27-1	chr21	31709654/A//G	synonymous	1	0
ITSN1	chr21	35147320/C//G	nonsynonymous	1	0
B3GALT5	chr21	41033164/G//T	synonymous	1	0
DSCAM	chr21	41684032/G//C	nonsynonymous	1	0
SPATC1L	chr21	47588375/T//A	nonsynonymous	1	0
DIP2A	chr21	47918488/A//G	synonymous	1	0
CCT8L2	chr22	17073012/G//C	synonymous	1	0

CLTCL1	chr22	19209109/C//A	nonsynonymous	1	0
CLTCL1	chr22	19217462/G//A	nonsynonymous	1	0
CLTCL1	chr22	19220999/C//A	synonymous	1	0
ARVCF	chr22	19968810/C//A	nonsynonymous	1	0
RSPH14	chr22	23482439/G//T	nonsynonymous	1	0
PIWIL3	chr22	25115777/A//T	nonsynonymous	1	0
MYO18B	chr22	26242210/T//A	nonsynonymous	1	0
SEZ6L	chr22	26707791/C//T	nonsynonymous	1	0
SEZ6L	chr22	26747144/T//C	nonsynonymous	1	0
MN1	chr22	28193128/A//T	nonsynonymous	1	0
MN1	chr22	28196499/G//C	synonymous	1	0
RHBDD3	chr22	29656024/G//A	synonymous	1	0
NEFH	chr22	29879383/G//T	synonymous	1	0
DEPDC5	chr22	32188751/C//T	nonsynonymous	1	0
C22orf42	chr22	32555006/A//G	nonsynonymous	1	0
TOM1	chr22	35713940/C//T	synonymous	1	0
MYH9	chr22	36678779/C//A	nonsynonymous	1	0
TMPRSS6	chr22	37465288/G//C	synonymous	1	0
MFNG	chr22	37870605/A//G	nonsynonymous	1	0
KCNJ4	chr22	38823589/C//A	nonsynonymous	1	0
APOBEC3D	chr22	39421288/G//A	nonsynonymous	1	0
SCUBE1	chr22	43716000/A//T	nonsynonymous	1	0
PHF21B	chr22	45279153/G//A	nonsynonymous	1	0
BRD1	chr22	50217497/C//A	nonsynonymous	1	0
GYG2	chrX	2779695/G//T	nonsynonymous	1	0
ARSH	chrX	2936637/C//A	nonsynonymous	1	0
SHROOM2	chrX	9864211/A//T	nonsynonymous	1	0
ADGRG2	chrX	19032183/C//G	nonsynonymous	1	0
MAP7D2	chrX	20030552/C//T	nonsynonymous	1	0
ZNF645	chrX	22291168/C//A	nonsynonymous	1	0
MAGEB3	chrX	30254540/G//C	nonsynonymous	1	0
CXorf21	chrX	30577936/T//A	nonsynonymous	1	0
DMD	chrX	32407663/C//A	nonsynonymous	1	0
DMD	chrX	32486681/G//T	synonymous	1	0
FAM47A	chrX	34150245/C//G	nonsynonymous	1	0
FAM47A	chrX	34150301/T//A	nonsynonymous	1	0
FAM47A	chrX	34150304/G//C	nonsynonymous	1	0
UXT	chrX	47517043/G//A	synonymous	1	0
SMC1A	chrX	53441703/C//T	synonymous	1	0
HUWE1	chrX	53610797/G//A	synonymous	1	0
FAM120C	chrX	54106712/C//G	nonsynonymous	1	0
ZC4H2	chrX	64196281/C//A	synonymous	1	0
NLGN3	chrX	70367627/C//T	synonymous	1	0
NLGN3	chrX	70367698/G//A	synonymous	1	0
CXCR3	chrX	70837068/A//T	nonsynonymous	1	0
UPRT	chrX	74513326/T//C	nonsynonymous	1	0
CYLC1	chrX	83128936/C//A	nonsynonymous	1	0
HDX	chrX	83723641/C//A	nonsynonymous	1	0
DACH2	chrX	85403684/G//GT	nonsynonymous	1	0
COL4A6	chrX	107402841/C//A	nonsynonymous	1	0
RGAG1	chrX	109695624/G//C	nonsynonymous	1	0
DCX	chrX	110574250/T//A	synonymous	1	0
DCX	chrX	110644517/C//G	nonsynonymous	1	0
KIAA1210	chrX	118222153/C//A	nonsynonymous	1	0
UPF3B	chrX	118977255/T//C	nonsynonymous	1	0
TEX13C	chrX	124456905/G//T	nonsynonymous	1	0
DCAF12L1	chrX	125685562/G//A	synonymous	1	0
SMARCA1	chrX	128605237/C//A	nonsynonymous	1	0
XPNPEP2	chrX	128880298/T//G	nonsynonymous	1	0
ENOX2	chrX	129759374/T//A	nonsynonymous	1	0
GPC4	chrX	132458216/T//C	nonsynonymous	1	0
ADGRG4	chrX	135429016/G//A	nonsynonymous	1	0
ADGRG4	chrX	135455135/G//A	nonsynonymous	1	0
ADGRG4	chrX	135485397/C//A	synonymous	1	0
CD40LG	chrX	135730523/C//A	nonsynonymous	1	0
	chrX	138727739/G//T	synonymous	1	0
MAGEC3	chrX	140969250/C//A	nonsynonymous	1	0
SLITRK2	chrX	144904417/C//A	nonsynonymous	1	0
PRRG3	chrX	150868534/A//T	nonsynonymous	1	0
PNMA5	chrX	152158994/C//A	nonsynonymous	1	0

ATP2B3	chrX	152807190/G//T	nonsynonymous	1	0
ATP2B3	chrX	152826171/G//T	synonymous	1	0
L1CAM	chrX	153132281/C//A	nonsynonymous	1	0
FLNA	chrX	153594450/G//A	synonymous	1	0
DVL1	chr1	1273393/A//C	nonsynonymous	1	0
MIIP	chr1	12091409/C//T	synonymous	1	0
FHAD1	chr1	15623224/G//T	nonsynonymous	1	0
CSMD2	chr1	34209214/G//A	synonymous	1	0
PRKAA2	chr1	57161792/C//A	nonsynonymous	1	0
C1orf168	chr1	57206384/C//G	synonymous	1	0
SLC44A5	chr1	75681456/T//C	nonsynonymous	1	0
LRRC8D	chr1	90399473/G//C	nonsynonymous	1	0
PTPN22	chr1	114391172/G//C	nonsynonymous	1	0
FCRL3	chr1	157665392/C//T	nonsynonymous	1	0
KIFAP3	chr1	169941688/C//T	nonsynonymous	1	0
KIAA1614	chr1	180897634/G//A	nonsynonymous	1	0
CACNA1E	chr1	181752817/G//T	nonsynonymous	1	0
KCNH1	chr1	211192226/C//T	nonsynonymous	1	0
ARID4B	chr1	235377296/T//C	synonymous	1	0
OR2L2	chr1	248202347/C//T	nonsynonymous	1	0
KCNF1	chr2	11052922/G//C	nonsynonymous	1	0
RHOB	chr2	20647357/CCGACATTGAGGTGG	nonsynonymous	1	0
SLC3A1	chr2	44502819/A//T	nonsynonymous	1	0
SOCS5	chr2	46987160/G//A	synonymous	1	0
ZNF2	chr2	95847499/C//G	nonsynonymous	1	0
IL1RL2	chr2	102835512/A//G	nonsynonymous	1	0
CCDC74A	chr2	132290992/G//T	synonymous	1	0
SCN9A	chr2	167085373/C//G	nonsynonymous	1	0
XIRP2	chr2	168105485/C//G	nonsynonymous	1	0
TTN	chr2	179480098/C//G	nonsynonymous	1	0
TTN	chr2	179569253/A//G	synonymous	1	0
TTN	chr2	179597376/C//G	nonsynonymous	1	0
TTN	chr2	179639041/C//T	nonsynonymous	1	0
ITGA4	chr2	182360510/G//T	nonsynonymous	1	0
TMEFF2	chr2	193056608/T//G	nonsynonymous	1	0
ZFAND2B	chr2	220074021/T//A	nonsynonymous	1	0
STK11IP	chr2	220479822/C//T	nonsynonymous	1	0
	chr2	230127406/G//C	synonymous	1	0
GRM7	chr3	7494476/C//T	nonsynonymous	1	0
TRANK1	chr3	36898248/G//C	nonsynonymous	1	0
KRBOX1	chr3	42984095/A//AC	nonsynonymous	1	0
PTH1R	chr3	46943307/C//T	nonsynonymous	1	0
FHIT	chr3	59737930/C//T	synonymous	1	0
TRMT10C	chr3	101284182/A//C	nonsynonymous	1	0
TRMT10C	chr3	101284645/A//G	synonymous	1	0
COL6A5	chr3	130107927/A//C	nonsynonymous	1	0
IL20RB	chr3	136708337/T//C	nonsynonymous	1	0
SOX14	chr3	137483863/T//G	synonymous	1	0
GPR87	chr3	151012787/T//C	nonsynonymous	1	0
EHHADH	chr3	184911074/T//A	nonsynonymous	1	0
NFXL1	chr4	47857086/C//G	nonsynonymous	1	0
UGT2B10	chr4	69885514/C//G	nonsynonymous	1	0
PPEF2	chr4	76805822/A//G	nonsynonymous	1	0
SEPT11	chr4	77932958/C//T	nonsynonymous	1	0
ADH4	chr4	100063874/C//T	nonsynonymous	1	0
ENPEP	chr4	111397959/G//A	nonsynonymous	1	0
MAB21L2	chr4	151505231/C//A	synonymous	1	0
FBXW7	chr4	153258983/G//A	nonsynonymous	1	0
FBXW7	chr4	153259090/T//C	nonsynonymous	1	0
GUCY1B3	chr4	156725843/C//G	nonsynonymous	1	0
IRX1	chr5	3599817/C//T	nonsynonymous	1	0
FGF10	chr5	44310629/A//G	nonsynonymous	1	0
N4BP3	chr5	177547629/G//A	nonsynonymous	1	0
RNF130	chr5	179393859/G//A	nonsynonymous	1	0
ELOVL2	chr6	11010983/C//T	synonymous	1	0
JARID2	chr6	15501478/C//T	synonymous	1	0
ATXN1	chr6	16326864/C//T	nonsynonymous	1	0
IP6K3	chr6	33695934/C//T	nonsynonymous	1	0
TTBK1	chr6	43225634/G//A	nonsynonymous	1	0
TDRD6	chr6	46656026/C//T	nonsynonymous	1	0

PDSS2	chr6	107475951/C//A	nonsynonymous	1	0
GPR6	chr6	110300986/G//A	nonsynonymous	1	0
GOPC	chr6	117888198/C//G	nonsynonymous	1	0
BCLAF1	chr6	136596705/A//G	nonsynonymous	1	0
NMBR	chr6	142409753/C//T	nonsynonymous	1	0
ZDHHC14	chr6	157963716/C//T	synonymous	1	0
DNAH11	chr7	21932181/G//C	nonsynonymous	1	0
HECW1	chr7	43477654/A//G	nonsynonymous	1	0
NPC1L1	chr7	44575567/G//A	nonsynonymous	1	0
ZNF107	chr7	64168217/C//T	nonsynonymous	1	0
TYW1	chr7	66703525/G//A	synonymous	1	0
PCLO	chr7	82584638/T//TA	nonsynonymous	1	0
TFPI2	chr7	93519522/G//A	synonymous	1	0
BUD31	chr7	99017026/G//A	nonsynonymous	1	0
LAMB4	chr7	107703363/G//A	synonymous	1	0
MET	chr7	116340231/T//C	nonsynonymous	1	0
PPP3CC	chr8	22398303/A//G	synonymous	1	0
RP1	chr8	55537356/A//C	nonsynonymous	1	0
SDCBP	chr8	59490749/C//G	nonsynonymous	1	0
CA8	chr8	61139429/T//C	synonymous	1	0
ADHFE1	chr8	67364310/C//T	nonsynonymous	1	0
DEPTOR	chr8	120977486/A//T	nonsynonymous	1	0
SCRIB	chr8	144886823/G//A	nonsynonymous	1	0
FGD3	chr9	95738925/C//T	synonymous	1	0
ANKS6	chr9	101540530/C//T	synonymous	1	0
RGS3	chr9	116258120/A//C	synonymous	1	0
OLFML2A	chr9	127572200/G//A	nonsynonymous	1	0
NOXA1	chr9	140328756/C//T	nonsynonymous	1	0
TUBB8	chr10	93817/G//A	nonsynonymous	1	0
PTCHD3	chr10	27702947/G//A	nonsynonymous	1	0
SLC18A3	chr10	50819673/G//T	nonsynonymous	1	0
	chr10	55570431/T//A	synonymous	1	0
ANK3	chr10	61815562/G//T	nonsynonymous	1	0
SLIT1	chr10	98806442/T//G	nonsynonymous	1	0
FOXI2	chr10	129535766/G//A	nonsynonymous	1	0
KNDC1	chr10	134981792/C//T	synonymous	1	0
TMEM80	chr11	703154/C//T	synonymous	1	0
CTSD	chr11	1774747/C//A	nonsynonymous	1	0
SLC22A18	chr11	2930890/C//T	synonymous	1	0
RBMXL2	chr11	7110884/G//A	nonsynonymous	1	0
OR4C15	chr11	55322186/T//C	nonsynonymous	1	0
ZDHHC5	chr11	57457857/T//TCACG	nonsynonymous	1	0
CHRDL2	chr11	74441961/C//T	synonymous	1	0
CREBZF	chr11	85375769/G//C	nonsynonymous	1	0
GUCY1A2	chr11	106558439/G//A	nonsynonymous	1	0
CWF19L2	chr11	107260824/C//T	synonymous	1	0
	chr11	118127937/T//G	synonymous	1	0
CCND2	chr12	4383276/G//T	nonsynonymous	1	0
CLEC6A	chr12	8618211/A//T	nonsynonymous	1	0
SOX5	chr12	24048943/T//A	synonymous	1	0
LETMD1	chr12	51450004/G//A	nonsynonymous	1	0
KRT84	chr12	52774993/C//T	synonymous	1	0
KRT79	chr12	53228026/G//A	nonsynonymous	1	0
HNRNPA1	chr12	54675574/C//G	synonymous	1	0
PAN2	chr12	56717656/G//A	nonsynonymous	1	0
LRRC10	chr12	70004402/G//T	nonsynonymous	1	0
NEDD1	chr12	97334208/C//G	nonsynonymous	1	0
ANKRD13A	chr12	110474093/G//C	nonsynonymous	1	0
NCOR2	chr12	124857079/C//A	nonsynonymous	1	0
KPNA3	chr13	50321089/C//T	nonsynonymous	1	0
LMO7	chr13	76395543/C//T	nonsynonymous	1	0
CCDC168	chr13	103394425/C//T	synonymous	1	0
SSTR1	chr14	38679494/C//T	synonymous	1	0
SIX6	chr14	60976301/T//G	nonsynonymous	1	0
SLC8A3	chr14	70512676/G//T	synonymous	1	0
EML5	chr14	89130911/A//C	nonsynonymous	1	0
UNC79	chr14	94173265/G//A	synonymous	1	0
SNAP23	chr15	42821882/T//C	synonymous	1	0
DYX1C1	chr15	55759243/T//A	nonsynonymous	1	0
HERC1	chr15	63904583/C//T	nonsynonymous	1	0

ABCA3	chr16	2329008/C//T	nonsynonymous	1	0
FBXL19	chr16	30941840/C//T	synonymous	1	0
LCAT	chr16	67976617/G//A	synonymous	1	0
MLKL	chr16	74708872/C//T	nonsynonymous	1	0
ADAMTS18	chr16	77398085/C//T	nonsynonymous	1	0
PKD1L2	chr16	81222545/G//A	nonsynonymous	1	0
NXN	chr17	726880/C//T	nonsynonymous	1	0
GSG2	chr17	3627597/CGCAGAAGT//C	nonsynonymous	1	0
C17orf107	chr17	4804527/C//T	synonymous	1	0
NSRP1	chr17	28505172/A//G	synonymous	1	0
WNK4	chr17	40948553/G//A	nonsynonymous	1	0
SDK2	chr17	71397881/G//A	synonymous	1	0
CD300LB	chr17	72527512/G//A	synonymous	1	0
SEH1L	chr18	12978753/A//C	nonsynonymous	1	0
SMAD2	chr18	45375020/A//C	nonsynonymous	1	0
DCC	chr18	50985827/A//G	synonymous	1	0
FZR1	chr19	3527007/C//G	synonymous	1	0
TBXA2R	chr19	3600361/G//A	nonsynonymous	1	0
SPC24	chr19	11257825/C//T	synonymous	1	0
ZNF700	chr19	12060374/T//A	nonsynonymous	1	0
NOTCH3	chr19	15285087/G//A	nonsynonymous	1	0
KLF2	chr19	16436633/G//A	nonsynonymous	1	0
CHERP	chr19	16639022/C//T	nonsynonymous	1	0
CRLF1	chr19	18707451/G//A	synonymous	1	0
CRTC1	chr19	18882313/C//T	nonsynonymous	1	0
ZNF708	chr19	21476980/C//T	nonsynonymous	1	0
TSHZ3	chr19	31768995/C//A	synonymous	1	0
PSG4	chr19	43698559/A//C	nonsynonymous	1	0
RSPH6A	chr19	46307573/G//A	synonymous	1	0
LILRA1	chr19	55106668/C//G	nonsynonymous	1	0
KIF16B	chr20	16410518/TATAAG//T	nonsynonymous	1	0
SYNDIG1	chr20	24523935/G//C	nonsynonymous	1	0
PPP1R16B	chr20	37524319/G//C	nonsynonymous	1	0
ZNF831	chr20	57829109/C//T	nonsynonymous	1	0
LTN1	chr21	30338728/A//T	nonsynonymous	1	0
HUNK	chr21	33371231/A//G	nonsynonymous	1	0
GART	chr21	34901156/C//G	nonsynonymous	1	0
DYRK1A	chr21	38884489/C//G	synonymous	1	0
DSCAM	chr21	41550901/C//A	nonsynonymous	1	0
MCM3AP	chr21	47660800/C//T	nonsynonymous	1	0
CABIN1	chr22	24445671/G//A	nonsynonymous	1	0
RHBDD3	chr22	29656780/G//A	synonymous	1	0
CELSR1	chr22	46787523/C//T	synonymous	1	0
PIM3	chr22	50356713/C//T	nonsynonymous	1	0
FAM47B	chrX	34962424/C//T	synonymous	1	0
USP11	chrX	47098475/T//C	synonymous	1	0
STARD8	chrX	67937376/G//A	nonsynonymous	1	0
MID2	chrX	107084562/C//T	nonsynonymous	1	0
ADGRF5	chr6	46874420/T//C	nonsynonymous	0	1
CALCOCO1	chr12	54110076/T//C	nonsynonymous	0	1
GOLGA3	chr12	133360757/T//C	nonsynonymous	0	1
ZNF26	chr12	133587370/T//C	nonsynonymous	0	1
PSEN1	chr14	73678592/A//G	synonymous	0	1
ZNF236	chr18	74590101/T//C	nonsynonymous	0	1
GAMT	chr19	1399793/T//C	nonsynonymous	0	1
LAMA5	chr20	60904921/A//G	nonsynonymous	0	1
MED12	chrX	70349922/A//G	nonsynonymous	0	1
BTK	chrX	100611125/T//C	nonsynonymous	0	1
ESPN	chr1	6511915/T//C	nonsynonymous	0	1
PRAMEF1	chr1	12854544/T//C	synonymous	0	1
HFM1	chr1	91779578/C//A	nonsynonymous	0	1
CD1B	chr1	158299176/G//T	nonsynonymous	0	1
CACNA1E	chr1	181754450/C//G	synonymous	0	1
PPP4R3B	chr2	55792037/C//G	nonsynonymous	0	1
ABCB11	chr2	169851904/C//A	nonsynonymous	0	1
ZGRF1	chr4	113538953/C//A	nonsynonymous	0	1
CDH9	chr5	26885886/G//A	synonymous	0	1
NUDT12	chr5	102894631/A//T	nonsynonymous	0	1
SLC12A2	chr5	127477569/G//A	nonsynonymous	0	1
LOC100505635	chr6	39856612/G//A	synonymous	0	1

PRIM2	chr6	57512614/A//T	nonsynonymous	0	1
NCOA2	chr8	71036916/C//T	synonymous	0	1
C9orf57	chr9	74674163/CGAATAAGATAACACC	nonsynonymous	0	1
ATRNL1	chr10	117607480/T//C	synonymous	0	1
LOC100128386	chr11	100558592/C//A	synonymous	0	1
TAS2R9	chr12	10962246/A//G	synonymous	0	1
BCAT1	chr12	24985748/G//A	nonsynonymous	0	1
RTN1	chr14	60212955/A//G	synonymous	0	1
ADAMTS18	chr16	77398216/G//A	nonsynonymous	0	1
TMEM132E	chr17	32959799/G//A	nonsynonymous	0	1
ERN1	chr17	62152614/A//C	synonymous	0	1
RAB8A	chr19	16240421/G//A	synonymous	0	1
POLD1	chr19	50916731/G//T	nonsynonymous	0	1
APOL5	chr22	36123119/A//G	nonsynonymous	0	1
CCDC120	chrX	48921951/C//T	synonymous	0	1
UPRT	chrX	74517333/G//T	nonsynonymous	0	1
TRMT2B	chrX	100276951/G//A	synonymous	0	1
PLXNB3	chrX	153037046/G//A	nonsynonymous	0	1
CDK11B	chr1	1573181/C//A	synonymous	0	1
SEMA4F	chr2	74902748/T//C	nonsynonymous	0	1
SLC34A2	chr4	25674815/G//GA	nonsynonymous	0	1
TSSK1B	chr5	112769824/G//A	nonsynonymous	0	1
HIST1H2AM	chr6	27860566/G//T	nonsynonymous	0	1
EYS	chr6	66006012/T//A	nonsynonymous	0	1
ZMIZ2	chr7	44802518/C//T	synonymous	0	1
GALNTL5	chr7	151699921/G//A	nonsynonymous	0	1
OR1K1	chr9	125562887/G//C	synonymous	0	1
CFAP46	chr10	134671213/C//T	nonsynonymous	0	1
KNDC1	chr10	134997427/G//A	nonsynonymous	0	1
PUS7L	chr12	44148918/T//C	nonsynonymous	0	1
ALPK3	chr15	85400817/C//T	nonsynonymous	0	1
XYLT1	chr16	17228330/C//T	nonsynonymous	0	1
LRRC3C	chr17	38100737/C//T	nonsynonymous	0	1
EPN3	chr17	48618218/G//T	synonymous	0	1
JPH2	chr20	42789014/C//T	nonsynonymous	0	1
NONO	chrX	70514339/C//G	nonsynonymous	0	1
GABRE	chrX	151123552/C//T	nonsynonymous	0	1
ITPR1	chr3	4856159/G//A	synonymous	0	1
BTN2A2	chr6	26390402/GGA//G	nonsynonymous	0	1
ATOH7	chr10	69991146/C//T	nonsynonymous	0	1
PEX16	chr11	45935930/T//C	nonsynonymous	0	1
ARHGAP23	chr17	36633932/C//T	nonsynonymous	0	1
ZNF253	chr19	20002701/T//C	synonymous	0	1
HTR3D	chr3	183756699/T//C	nonsynonymous	0	1
UGT2B28	chr4	70156392/A//G	synonymous	0	1
CCNK	chr14	99967172/T//C	nonsynonymous	0	1
HEXA	chr15	72641577/A//G	nonsynonymous	0	1
SS18	chr18	23637667/T//C	nonsynonymous	0	1
UBIAD1	chr1	11346108/G//A	nonsynonymous	0	1
NID1	chr1	236157057/G//A	synonymous	0	1
SNTG2	chr2	1079274/C//T	nonsynonymous	0	1
CNTNAP5	chr2	124999841/C//T	synonymous	0	1
ECE2	chr3	184008356/C//A	nonsynonymous	0	1
MUC4	chr3	195512990/C//G	nonsynonymous	0	1
SHROOM3	chr4	77631405/G//A	synonymous	0	1
PIK3R1	chr5	67589585/C//CATGAAT	nonsynonymous	0	1
APC	chr5	112175579/AC//A	nonsynonymous	0	1
TRPA1	chr8	72951161/G//T	nonsynonymous	0	1
ANKRD18A	chr9	38575653/C//T	synonymous	0	1
	chr9	73398840/T//C	synonymous	0	1
CARD9	chr9	139258965/C//T	nonsynonymous	0	1
PFKFB3	chr10	6262673/C//T	nonsynonymous	0	1
POLR2G	chr11	62529139/A//G	synonymous	0	1
UGGT2	chr13	96515941/C//A	nonsynonymous	0	1
ZNF423	chr16	49672282/C//A	nonsynonymous	0	1
DHRS7B	chr17	21094356/GCTGACTTA//G	nonsynonymous	0	1
COL1A1	chr17	48262937/C//G	nonsynonymous	0	1
APOH	chr17	64210685/T//C	nonsynonymous	0	1
TMC8	chr17	76128488/T//C	nonsynonymous	0	1
MISP	chr19	757199/G//A	nonsynonymous	0	1

INSR	chr19	7172330/A//G	synonymous	0	1
RFX1	chr19	14074381/TGGC//T	nonsynonymous	0	1
CPAMD8	chr19	17032727/G//A	nonsynonymous	0	1
ZNF831	chr20	57767250/G//A	synonymous	0	1
SON	chr21	34923991/C//T	synonymous	0	1
CSF2RA	chrX	1428601/G//A	synonymous	0	1
APEX2	chrX	55033203/G//A	nonsynonymous	0	1
TMSB15A	chrX	101769048/G//T	synonymous	0	1
KCNE5	chrX	108868103/G//A	synonymous	0	1
CNGA2	chrX	150908126/G//A	nonsynonymous	0	1
PTPRU	chr1	29641949/A//G	nonsynonymous	1	0
HRNR	chr1	152185792/G//A	synonymous	1	0
HRNR	chr1	152185796/A//G	nonsynonymous	1	0
IARS2	chr1	220300149/T//C	nonsynonymous	1	0
TP53BP2	chr1	223984275/T//C	nonsynonymous	1	0
GIGYF2	chr2	233712069/G//A	nonsynonymous	1	0
ZNF717	chr3	75786866/G//T	synonymous	1	0
ZNF717	chr3	75786892/A//C	nonsynonymous	1	0
ZNF717	chr3	75786899/T//C	synonymous	1	0
ZNF717	chr3	75786910/T//C	nonsynonymous	1	0
ZNF717	chr3	75786916/C//A	nonsynonymous	1	0
ZNF717	chr3	75786919/A//T	nonsynonymous	1	0
SYNE1	chr6	152527482/A//G	nonsynonymous	1	0
ABHD17B	chr9	74489533/G//A	nonsynonymous	1	0
NTRK2	chr9	87635188/C//T	nonsynonymous	1	0
PRRX2	chr9	132483040/T//C	nonsynonymous	1	0
KCNT1	chr9	138641988/G//A	nonsynonymous	1	0
ELMOD1	chr11	107488835/CACTT//C	synonymous	1	0
RALGAPA1	chr14	36217801/ATC//A	nonsynonymous	1	0
DYNC1H1	chr14	102431038/C//T	nonsynonymous	1	0
ATP2A1	chr16	28899001/T//C	nonsynonymous	1	0
STAT5B	chr17	40353745/A//G	synonymous	1	0
RBBP7	chrX	16876906/T//C	nonsynonymous	1	0
AP4B1	chr1	114438987/G//GTTT	nonsynonymous	0	1
PROS1	chr3	93617382/A//G	synonymous	0	1
ITGB8	chr7	20371466/T//C	nonsynonymous	0	1
TOP1MT	chr8	144403494/A//G	synonymous	0	1
MOB2	chr11	1491473/G//C	nonsynonymous	0	1
TGM7	chr15	43571911/G//A	synonymous	0	1
ABR	chr17	910560/G//A	synonymous	0	1
GPCPD1	chr20	5560674/T//A	nonsynonymous	0	1
LRRC39	chr1	100626082/T//C	synonymous	0	1
AKNAD1	chr1	109395309/C//T	synonymous	0	1
SLC8A1	chr2	40656878/G//T	synonymous	0	1
RGPD3	chr2	107073562/G//T	nonsynonymous	0	1
XIRP1	chr3	39230071/C//T	nonsynonymous	0	1
USF3	chr3	113378383/G//C	nonsynonymous	0	1
HIST1H3B	chr6	26031900/C//A	nonsynonymous	0	1
HMGN4	chr6	26545440/C//G	synonymous	0	1
HLA-G	chr6	29797674/C//T	nonsynonymous	0	1
RCAN2	chr6	46214568/G//C	nonsynonymous	0	1
HOXA9	chr7	27204728/C//T	nonsynonymous	0	1
KIAA1147	chr7	141362399/T//C	synonymous	0	1
TOX	chr8	59750758/G//A	nonsynonymous	0	1
ATRNL1	chr10	117185754/C//T	synonymous	0	1
NUTM1	chr15	34646640/C//A	synonymous	0	1
TP53	chr17	7579371/T//TG	nonsynonymous	0	1
KRTAP4-11	chr17	39274415/C//T	synonymous	0	1
PEG3	chr19	57326387/T//A	synonymous	0	1
MACROD2	chr20	15948246/A//G	nonsynonymous	0	1
NCOA3	chr20	46252789/A//T	nonsynonymous	0	1
ARSF	chrX	2994688/G//C	synonymous	0	1
DISP3	chr1	11595182/G//T	nonsynonymous	1	0
SPEN	chr1	16265283/C//A	nonsynonymous	1	0
COL11A1	chr1	103480122/C//A	nonsynonymous	1	0
LCE2B	chr1	152659572/C//A	synonymous	1	0
FCRL4	chr1	157557286/G//A	synonymous	1	0
MNDA	chr1	158812124/T//G	nonsynonymous	1	0
TNN	chr1	175092610/C//T	nonsynonymous	1	0
CACNA1S	chr1	201019515/A//T	nonsynonymous	1	0

USH2A	chr1	216062206/G//T	synonymous	1	0
NID1	chr1	236205258/G//C	nonsynonymous	1	0
RYR2	chr1	237814807/T//C	synonymous	1	0
OR2G2	chr1	247752006/C//T	synonymous	1	0
TRIM58	chr1	248020840/C//A	synonymous	1	0
KCNK12	chr2	47797150/G//T	synonymous	1	0
INHBB	chr2	121104205/C//T	synonymous	1	0
LRP1B	chr2	141607809/T//C	nonsynonymous	1	0
EPC2	chr2	149522657/A//G	nonsynonymous	1	0
FRZB	chr2	183707250/A//T	nonsynonymous	1	0
SF3B1	chr2	198267331/C//A	nonsynonymous	1	0
SATB2	chr2	200173490/G//T	nonsynonymous	1	0
CARF	chr2	203846859/A//C	nonsynonymous	1	0
NRP2	chr2	206628605/G//A	nonsynonymous	1	0
INPP5D	chr2	234078810/G//C	nonsynonymous	1	0
ABHD14B	chr3	52003974/G//C	nonsynonymous	1	0
CMSS1	chr3	99881241/G//A	nonsynonymous	1	0
	chr3	113022826/C//A	synonymous	1	0
UVSSA	chr4	1347033/G//T	nonsynonymous	1	0
CD38	chr4	15780146/G//T	nonsynonymous	1	0
LRRC66	chr4	52861180/G//C	nonsynonymous	1	0
TMPRSS11F	chr4	68934500/T//A	nonsynonymous	1	0
COQ2	chr4	84205984/C//G	synonymous	1	0
PDHA2	chr4	96761561/G//T	nonsynonymous	1	0
EGF	chr4	110883113/G//T	synonymous	1	0
PCDH10	chr4	134073775/C//T	nonsynonymous	1	0
TENM3	chr4	183601874/A//C	synonymous	1	0
MARCH11	chr5	16067552/G//A	synonymous	1	0
MAP1B	chr5	71495801/G//C	nonsynonymous	1	0
PCDHA1	chr5	140166455/CT//C	nonsynonymous	1	0
PCDHA5	chr5	140202975/G//T	nonsynonymous	1	0
PCDHB8	chr5	140559094/G//A	synonymous	1	0
PCDHGA9	chr5	140783302/G//C	synonymous	1	0
CLINT1	chr5	157214599/T//A	synonymous	1	0
SQSTM1	chr5	179264918/T//A	synonymous	1	0
OR12D3	chr6	29343022/G//T	nonsynonymous	1	0
NOTCH4	chr6	32172160/G//T	nonsynonymous	1	0
PRSS35	chr6	84233975/C//T	nonsynonymous	1	0
LAMA2	chr6	129612776/G//T	nonsynonymous	1	0
TCP10L2	chr6	167592535/G//A	nonsynonymous	1	0
THBS2	chr6	169625405/G//T	nonsynonymous	1	0
CCDC129	chr7	31617976/A//T	nonsynonymous	1	0
SUGCT	chr7	40535948/C//G	nonsynonymous	1	0
	chr7	44197726/G//A	synonymous	1	0
HUS1	chr7	48018368/C//T	nonsynonymous	1	0
PCLO	chr7	82581762/T//C	nonsynonymous	1	0
PRKAR2B	chr7	106797494/G//T	nonsynonymous	1	0
ZNF800	chr7	127013707/T//C	nonsynonymous	1	0
FLNC	chr7	128494041/G//T	nonsynonymous	1	0
MGAM	chr7	141752730/G//C	synonymous	1	0
CSMD1	chr8	3047589/C//A	nonsynonymous	1	0
	chr8	15998529/A//T	synonymous	1	0
LZTS1	chr8	20112393/C//A	synonymous	1	0
PTK2B	chr8	27277560/A//G	nonsynonymous	1	0
FGFR1	chr8	38271189/CGG//C	nonsynonymous	1	0
TACC1	chr8	38677955/G//A	nonsynonymous	1	0
PLAT	chr8	42040278/G//T	synonymous	1	0
UHRF2	chr9	6481682/A//G	synonymous	1	0
TMC1	chr9	75431091/C//T	synonymous	1	0
SPATA31C2	chr9	90746191/G//A	synonymous	1	0
DEC1	chr9	118164396/G//T	nonsynonymous	1	0
CRB2	chr9	126125336/G//C	nonsynonymous	1	0
NR5A1	chr9	127253424/C//A	synonymous	1	0
PLPP7	chr9	134165413/C//A	nonsynonymous	1	0
KCNT1	chr9	138649162/C//T	nonsynonymous	1	0
CCDC3	chr10	13040417/A//C	nonsynonymous	1	0
KIAA1462	chr10	30315216/T//A	nonsynonymous	1	0
ZNF33A	chr10	38343828/G//T	nonsynonymous	1	0
	chr10	73377267/C//A	synonymous	1	0
HPSE2	chr10	100992223/G//T	synonymous	1	0

SEC31B	chr10	102267260/T//C	nonsynonymous	1	0
CFAP46	chr10	134690380/A//T	nonsynonymous	1	0
RRP8	chr11	6622791/T//A	nonsynonymous	1	0
OLFML1	chr11	7509471/C//A	nonsynonymous	1	0
SLC1A2	chr11	35282400/C//A	synonymous	1	0
OR4S2	chr11	55418764/C//G	nonsynonymous	1	0
OR5F1	chr11	55761788/A//G	nonsynonymous	1	0
FAT3	chr11	92523337/G//T	nonsynonymous	1	0
MAML2	chr11	95826222/C//T	nonsynonymous	1	0
BIN2	chr12	51693409/A//G	synonymous	1	0
DPY19L2	chr12	63974442/C//T	nonsynonymous	1	0
GLIPR1	chr12	75874824/T//A	nonsynonymous	1	0
TCTN1	chr12	111066634/A//T	nonsynonymous	1	0
PIWIL1	chr12	130842082/C//A	nonsynonymous	1	0
RNF219	chr13	79191012/C//A	nonsynonymous	1	0
SLITRK1	chr13	84454508/C//A	nonsynonymous	1	0
CCDC168	chr13	103388737/C//G	nonsynonymous	1	0
ADSSL1	chr14	105212671/C//A	nonsynonymous	1	0
MYO9A	chr15	72144574/T//C	nonsynonymous	1	0
CPLX3	chr15	75119111/G//A	nonsynonymous	1	0
SLC28A1	chr15	85447387/A//G	nonsynonymous	1	0
ITGAM	chr16	31336614/G//A	synonymous	1	0
C16orf87	chr16	46843589/CTG//C	nonsynonymous	1	0
ZNF423	chr16	49670452/T//A	nonsynonymous	1	0
CSNK2A2	chr16	58200564/C//A	nonsynonymous	1	0
CDH1	chr16	68844241/C//T	nonsynonymous	1	0
DVL2	chr17	7131073/C//A	nonsynonymous	1	0
PER1	chr17	8045237/C//A	synonymous	1	0
C17orf51	chr17	21438800/C//G	nonsynonymous	1	0
RHBDL3	chr17	30625227/G//T	synonymous	1	0
	chr17	31618741/G//A	synonymous	1	0
KRT31	chr17	39553453/G//C	synonymous	1	0
TMC6	chr17	76118747/T//C	nonsynonymous	1	0
CD226	chr18	67614141/T//G	nonsynonymous	1	0
SALL3	chr18	76754265/C//G	nonsynonymous	1	0
STK11	chr19	1220703/G//C	nonsynonymous	1	0
APC2	chr19	1462120/C//T	synonymous	1	0
CELF5	chr19	3290264/C//A	nonsynonymous	1	0
MUC16	chr19	9059662/C//A	nonsynonymous	1	0
MUC16	chr19	9066300/G//T	nonsynonymous	1	0
CYP4F12	chr19	15784523/G//C	nonsynonymous	1	0
ZNF208	chr19	22157602/A//G	synonymous	1	0
PLEKHG2	chr19	39908648/G//T	nonsynonymous	1	0
ZNF155	chr19	44501157/G//T	nonsynonymous	1	0
PRR12	chr19	50097767/G//C	nonsynonymous	1	0
CPT1C	chr19	50210807/TC//T	nonsynonymous	1	0
	chr19	51857916/T//C	synonymous	1	0
LZTS3	chr20	3146206/C//A	nonsynonymous	1	0
KRTAP13-1	chr21	31768549/G//T	nonsynonymous	1	0
ITSN1	chr21	35147292/A//G	synonymous	1	0
SPATC1L	chr21	47581895/C//T	nonsynonymous	1	0
ASCC2	chr22	30185098/T//A	nonsynonymous	1	0
ZXDB	chrX	57619483/T//C	synonymous	1	0
HEPH	chrX	65428059/C//A	nonsynonymous	1	0
P2RY10	chrX	78216918/G//T	nonsynonymous	1	0
DCAF12L2	chrX	125298724/G//T	nonsynonymous	1	0
PER3	chr1	7861166/T//C	synonymous	1	0
DISP3	chr1	11562131/T//C	nonsynonymous	1	0
FHAD1	chr1	15578302/G//T	nonsynonymous	1	0
PLA2G2F	chr1	20465939/G//T	nonsynonymous	1	0
GRIK3	chr1	37267612/C//T	nonsynonymous	1	0
PLPPR4	chr1	99730211/G//C	nonsynonymous	1	0
PDE4DIP	chr1	144918984/C//T	nonsynonymous	1	0
FLG	chr1	152277216/A//T	synonymous	1	0
NUP210L	chr1	154112418/A//C	nonsynonymous	1	0
THBS3	chr1	155168003/C//T	nonsynonymous	1	0
SPTA1	chr1	158623112/C//T	nonsynonymous	1	0
GORAB	chr1	170508677/G//C	nonsynonymous	1	0
DHX9	chr1	182852339/C//T	nonsynonymous	1	0
F13B	chr1	197024881/C//T	nonsynonymous	1	0

KLHL29	chr2	23918513/C//T	synonymous	1	0
CAD	chr2	27440417/C//T	synonymous	1	0
XDH	chr2	31621545/G//A	synonymous	1	0
EML6	chr2	55176059/CAGA//C	nonsynonymous	1	0
SMYD1	chr2	88387520/G//A	nonsynonymous	1	0
ANKRD36	chr2	97790189/T//C	synonymous	1	0
ACTR3	chr2	114699805/A//C	nonsynonymous	1	0
CLASP1	chr2	122227422/C//T	nonsynonymous	1	0
THSD7B	chr2	138417321/G//C	synonymous	1	0
HOXD4	chr2	177017390/G//A	nonsynonymous	1	0
TTN	chr2	179571347/C//T	nonsynonymous	1	0
TTN	chr2	179583978/A//G	synonymous	1	0
ZNF804A	chr2	185731223/A//G	nonsynonymous	1	0
CCDC150	chr2	197593930/T//C	nonsynonymous	1	0
FN1	chr2	216249706/A//C	synonymous	1	0
NMUR1	chr2	232390072/G//A	synonymous	1	0
COL6A3	chr2	238287349/A//G	synonymous	1	0
SNED1	chr2	242007219/G//A	synonymous	1	0
ATP2B2	chr3	10392216/G//A	nonsynonymous	1	0
WNT7A	chr3	13860466/C//T	nonsynonymous	1	0
EPM2AIP1	chr3	37033458/G//A	nonsynonymous	1	0
DLEC1	chr3	38139012/G//A	nonsynonymous	1	0
CACNA2D3	chr3	55038847/C//T	synonymous	1	0
COL8A1	chr3	99509553/G//T	nonsynonymous	1	0
NFKBIZ	chr3	101576213/G//T	synonymous	1	0
B4GALT4	chr3	118931488/C//G	nonsynonymous	1	0
HEG1	chr3	124739861/G//A	synonymous	1	0
TOPBP1	chr3	133342258/T//C	nonsynonymous	1	0
PRR23B	chr3	138739379/G//A	nonsynonymous	1	0
TTC14	chr3	180328219/C//G	nonsynonymous	1	0
TTC14	chr3	180328251/A//G	nonsynonymous	1	0
AHSG	chr3	186338557/C//T	synonymous	1	0
RNF168	chr3	196198626/C//T	synonymous	1	0
BOD1L1	chr4	13605364/T//C	nonsynonymous	1	0
DCAF4L1	chr4	41984887/G//A	nonsynonymous	1	0
TMPRSS11A	chr4	68780439/TG//T	nonsynonymous	1	0
UGT2A2	chr4	70504662/G//T	nonsynonymous	1	0
THAP9	chr4	83838887/A//T	nonsynonymous	1	0
MAPK10	chr4	87023126/T//C	nonsynonymous	1	0
PPM1K	chr4	89189971/C//T	nonsynonymous	1	0
CASP6	chr4	110618804/G//A	synonymous	1	0
ALPK1	chr4	113303556/T//G	nonsynonymous	1	0
MAML3	chr4	140810650/T//C	nonsynonymous	1	0
MAML3	chr4	140812099/T//A	nonsynonymous	1	0
MARCH1	chr4	164775218/G//A	synonymous	1	0
TENM3	chr4	183664409/C//T	nonsynonymous	1	0
ERAP1	chr5	96127767/A//C	nonsynonymous	1	0
APC	chr5	112173602/G//T	nonsynonymous	1	0
PCDHB13	chr5	140595403/G//A	nonsynonymous	1	0
PCDHB13	chr5	140595792/G//A	synonymous	1	0
GRIA1	chr5	153144043/A//G	nonsynonymous	1	0
MED7	chr5	156565696/A//G	synonymous	1	0
RANBP17	chr5	170346473/A//G	nonsynonymous	1	0
OR2W1	chr6	29012329/G//C	synonymous	1	0
PKHD1	chr6	51887691/C//T	nonsynonymous	1	0
MCM3	chr6	52144358/A//G	nonsynonymous	1	0
HCRTR2	chr6	55147027/A//C	nonsynonymous	1	0
COL12A1	chr6	75899397/T//C	nonsynonymous	1	0
MDN1	chr6	90394651/C//G	nonsynonymous	1	0
EPHA7	chr6	93967222/T//C	synonymous	1	0
AGPAT4	chr6	161570289/T//C	nonsynonymous	1	0
DMTF1	chr7	86817422/C//G	nonsynonymous	1	0
COL1A2	chr7	94043217/T//C	synonymous	1	0
NPTX2	chr7	98257924/C//T	nonsynonymous	1	0
AASS	chr7	121733177/T//G	nonsynonymous	1	0
LMOD2	chr7	123296147/C//T	nonsynonymous	1	0
TBXAS1	chr7	139529161/G//C	synonymous	1	0
	chr7	143417995/C//T	synonymous	1	0
ABCB8	chr7	150737687/C//T	nonsynonymous	1	0
DLC1	chr8	13357107/T//A	nonsynonymous	1	0

PDLIM2	chr8	22442865/C//T	nonsynonymous	1	0
NRG1	chr8	32453445/G//A	nonsynonymous	1	0
ST18	chr8	53044521/C//T	nonsynonymous	1	0
NPBWR1	chr8	53852752/C//T	synonymous	1	0
RP1	chr8	55540963/A//C	synonymous	1	0
PREX2	chr8	68989671/G//A	nonsynonymous	1	0
	chr8	69017372/G//C	synonymous	1	0
RIMS2	chr8	104898339/C//T	synonymous	1	0
NOV	chr8	120430477/C//G	nonsynonymous	1	0
ATAD2	chr8	124373857/G//A	nonsynonymous	1	0
GSDMD	chr8	144645026/C//A	synonymous	1	0
SCRIB	chr8	144892707/CG//C	nonsynonymous	1	0
SPATA31C2	chr9	90744737/G//A	nonsynonymous	1	0
GABBR2	chr9	101216287/G//A	synonymous	1	0
ACTL7B	chr9	111617355/C//T	nonsynonymous	1	0
KIAA0368	chr9	114133004/G//T	nonsynonymous	1	0
ALAD	chr9	116152915/G//A	nonsynonymous	1	0
CRB2	chr9	126139326/GGA//G	nonsynonymous	1	0
C9orf116	chr9	138387410/C//T	nonsynonymous	1	0
CACNA1B	chr9	140777318/C//T	synonymous	1	0
EIF4G2	chr11	10821740/A//T	synonymous	1	0
KIAA1549L	chr11	33689765/C//T	synonymous	1	0
OR9Q1	chr11	57947504/A//T	nonsynonymous	1	0
PC	chr11	66618659/G//A	nonsynonymous	1	0
MMP3	chr11	102713419/T//C	nonsynonymous	1	0
DYNC2H1	chr11	103019219/C//G	nonsynonymous	1	0
AKAP3	chr12	4737680/G//A	nonsynonymous	1	0
PZP	chr12	9304216/A//T	nonsynonymous	1	0
KLRF2	chr12	10034123/T//C	synonymous	1	0
TMEM52B	chr12	10342711/C//T	nonsynonymous	1	0
ST8SIA1	chr12	22354566/G//A	nonsynonymous	1	0
PCBP2	chr12	53848633/C//T	nonsynonymous	1	0
NEUROD4	chr12	55420486/G//A	nonsynonymous	1	0
SLC35E3	chr12	69140486/C//G	nonsynonymous	1	0
NTS	chr12	86272114/AT//A	synonymous	1	0
SH2B3	chr12	111856332/G//A	nonsynonymous	1	0
SRRM4	chr12	119540063/G//A	nonsynonymous	1	0
TUBA3C	chr13	19748274/G//A	nonsynonymous	1	0
NBEA	chr13	35738655/T//C	synonymous	1	0
ITGBL1	chr13	102220122/C//A	nonsynonymous	1	0
RPGRIP1	chr14	21796637/A//G	nonsynonymous	1	0
MYH7	chr14	23884924/C//T	nonsynonymous	1	0
CLEC14A	chr14	38724681/G//T	nonsynonymous	1	0
FAM181A	chr14	94395253/G//A	nonsynonymous	1	0
INO80	chr15	41346203/G//T	nonsynonymous	1	0
EHD4	chr15	42192954/C//T	synonymous	1	0
CALML4	chr15	68486344/TCTC//T	synonymous	1	0
SEMA7A	chr15	74710642/G//A	synonymous	1	0
PKD1L3	chr16	72016031/G//C	nonsynonymous	1	0
ADAMTS18	chr16	77328857/G//T	nonsynonymous	1	0
PKD1L2	chr16	81183469/C//T	nonsynonymous	1	0
RFLNB	chr17	293115/C//T	nonsynonymous	1	0
TP53	chr17	7578265/A//T	nonsynonymous	1	0
SPECC1	chr17	20013829/C//T	synonymous	1	0
RAD51D	chr17	33434077/G//C	nonsynonymous	1	0
FDXR	chr17	72869017/C//T	nonsynonymous	1	0
NOTUM	chr17	79914595/G//A	nonsynonymous	1	0
VAPA	chr18	9937043/C//T	nonsynonymous	1	0
TUBB6	chr18	12325216/C//T	nonsynonymous	1	0
SALL3	chr18	76754490/G//A	synonymous	1	0
SPPL2B	chr19	2352960/C//T	nonsynonymous	1	0
ZNF77	chr19	2944926/C//T	synonymous	1	0
MUC16	chr19	9062874/A//T	nonsynonymous	1	0
ZNF559-ZNF177	chr19	9489725/G//T	nonsynonymous	1	0
SMARCA4	chr19	11152080/C//T	nonsynonymous	1	0
ZNF708	chr19	21476148/G//A	synonymous	1	0
ZNF99	chr19	22940767/G//T	synonymous	1	0
CCNE1	chr19	30314573/A//C	synonymous	1	0
ZNF568	chr19	37441173/G//A	nonsynonymous	1	0
PRR12	chr19	50098446/A//G	nonsynonymous	1	0

MED25	chr19	50338372/C//T	nonsynonymous	1	0
MYH14	chr19	50783514/G//T	synonymous	1	0
ZNF480	chr19	52819101/G//C	nonsynonymous	1	0
THBD	chr20	23028840/G//A	synonymous	1	0
ITCH	chr20	33000446/G//A	nonsynonymous	1	0
CBLN4	chr20	54575882/A//C	nonsynonymous	1	0
SPATC1L	chr21	47581472/A//G	nonsynonymous	1	0
CABIN1	chr22	24459603/G//A	synonymous	1	0
CABIN1	chr22	24483532/C//T	nonsynonymous	1	0
HMGXB4	chr22	35659824/CAAG//C	nonsynonymous	1	0
CSF2RA	chrX	1407701/G//A	synonymous	1	0
MAP7D2	chrX	20071097/T//C	nonsynonymous	1	0
IL1RAPL1	chrX	29301060/G//T	nonsynonymous	1	0
CACNA1F	chrX	49066800/A//T	nonsynonymous	1	0
WNK3	chrX	54319643/G//A	nonsynonymous	1	0
CYSLTR1	chrX	77528882/C//T	nonsynonymous	1	0
RHOXF1	chrX	119249555/C//T	nonsynonymous	1	0
INTS6L	chrX	134683628/T//A	nonsynonymous	1	0
SLC9A6	chrX	135104829/A//G	nonsynonymous	1	0
CDK11A	chr1	1636044/G//A	synonymous	0	1
UBR4	chr1	19524187/T//C	synonymous	0	1
HNRNPR	chr1	23637496/A//G	synonymous	0	1
EPHA10	chr1	38227322/A//T	nonsynonymous	0	1
INPP5B	chr1	38341385/T//C	nonsynonymous	0	1
SLC44A5	chr1	75693487/T//C	synonymous	0	1
	chr1	170993788/A//G	synonymous	0	1
PPP4R3B	chr2	55825749/A//C	nonsynonymous	0	1
DYSF	chr2	71886082/C//T	synonymous	0	1
FER1L5	chr2	97359277/C//T	synonymous	0	1
SLC9A2	chr2	103324704/A//G	nonsynonymous	0	1
SATB1	chr3	18419810/G//C	nonsynonymous	0	1
TRIM71	chr3	32932914/G//A	nonsynonymous	0	1
SLC22A14	chr3	38357834/G//A	nonsynonymous	0	1
DHX30	chr3	47888361/C//T	nonsynonymous	0	1
HYAL1	chr3	50339817/G//A	nonsynonymous	0	1
LRRIQ4	chr3	169540417/G//T	synonymous	0	1
DCLK2	chr4	151023666/G//A	nonsynonymous	0	1
TENM3	chr4	183696257/G//A	nonsynonymous	0	1
TENM3	chr4	183717885/G//A	nonsynonymous	0	1
APC	chr5	112174112/G//T	nonsynonymous	0	1
SEMA6A	chr5	115783402/C//A	nonsynonymous	0	1
PCDHA10	chr5	140236799/C//T	nonsynonymous	0	1
PCDHB6	chr5	140530024/C//T	synonymous	0	1
DOCK2	chr5	169267790/C//G	nonsynonymous	0	1
RIMS1	chr6	72984080/A//G	nonsynonymous	0	1
COL12A1	chr6	75861712/T//G	synonymous	0	1
CNR1	chr6	88854742/CTTG//C	nonsynonymous	0	1
DDO	chr6	110736701/C//G	nonsynonymous	0	1
ROS1	chr6	117678052/T//C	nonsynonymous	0	1
SYNE1	chr6	152638096/T//C	nonsynonymous	0	1
	chr7	20698739/G//A	synonymous	0	1
TMEM120A	chr7	75616861/CAAAGA//C	nonsynonymous	0	1
MOSPD3	chr7	100211195/T//TC	nonsynonymous	0	1
RELN	chr7	103290763/G//T	nonsynonymous	0	1
DGKI	chr7	137092652/G//A	synonymous	0	1
TRPV5	chr7	142627220/C//T	synonymous	0	1
PSD3	chr8	18432710/G//A	nonsynonymous	0	1
TRPA1	chr8	72963036/C//G	nonsynonymous	0	1
HHLA1	chr8	133111180/C//G	nonsynonymous	0	1
RECK	chr9	36087884/A//G	synonymous	0	1
NOL8	chr9	95063881/C//T	nonsynonymous	0	1
ST8SIA6	chr10	17363105/G//A	synonymous	0	1
MYO3A	chr10	26357710/A//G	nonsynonymous	0	1
UNC5B	chr10	73047427/G//A	nonsynonymous	0	1
CNGA4	chr11	6261702/G//A	synonymous	0	1
UBTFL1	chr11	89819402/C//T	synonymous	0	1
APLP2	chr11	129979322/A//G	nonsynonymous	0	1
GRIN2B	chr12	13715772/C//T	nonsynonymous	0	1
PIK3C2G	chr12	18573933/G//T	nonsynonymous	0	1
FAM186A	chr12	50745501/A//G	nonsynonymous	0	1

DIP2B	chr12	51126148/G//A	nonsynonymous	0	1
KRT78	chr12	53242613/G//A	synonymous	0	1
TAOK3	chr12	118619344/C//T	synonymous	0	1
EP400	chr12	132514584/C//T	nonsynonymous	0	1
ZMYM2	chr13	20567940/C//A	nonsynonymous	0	1
OR4K5	chr14	20389501/G//A	nonsynonymous	0	1
LRFN5	chr14	42356190/G//A	nonsynonymous	0	1
ATP10A	chr15	25924588/G//A	nonsynonymous	0	1
WFIKKN1	chr16	682751/G//A	nonsynonymous	0	1
GGA2	chr16	23507075/G//T	nonsynonymous	0	1
GGA2	chr16	23507084/G//C	nonsynonymous	0	1
SETD1A	chr16	30991093/G//T	nonsynonymous	0	1
ZCCHC14	chr16	87446278/T//TCTTGGTCCAGATC	nonsynonymous	0	1
TP53	chr17	7578406/C//T	nonsynonymous	0	1
	chr17	17494838/T//C	synonymous	0	1
RAB11FIP4	chr17	29758896/C//T	synonymous	0	1
ASIC2	chr17	31350871/A//G	synonymous	0	1
SLC35B1	chr17	47782525/C//A	synonymous	0	1
SGCA	chr17	48245851/G//A	nonsynonymous	0	1
ABCC3	chr17	48741063/C//A	synonymous	0	1
ACE	chr17	61570998/G//C	synonymous	0	1
ZNF560	chr19	9577745/C//A	nonsynonymous	0	1
MYO9B	chr19	17314069/T//C	nonsynonymous	0	1
MVB12A	chr19	17531383/G//T	nonsynonymous	0	1
SPTBN4	chr19	41074188/G//A	nonsynonymous	0	1
GNAS	chr20	57485013/C//T	nonsynonymous	0	1
TPTE	chr21	10944666/A//G	nonsynonymous	0	1
KRTAP10-4	chr21	45994562/C//A	synonymous	0	1
CHEK2	chr22	29099506/T//C	nonsynonymous	0	1
DMC1	chr22	38934572/T//C	nonsynonymous	0	1
DMBX1	chr1	46972694/C//T	synonymous	0	1
VANGL1	chr1	116226705/G//T	synonymous	0	1
NHLH1	chr1	160340559/C//T	nonsynonymous	0	1
USH2A	chr1	215812555/C//T	synonymous	0	1
RNASEH1	chr2	3599780/G//A	synonymous	0	1
HADHA	chr2	26426986/G//A	nonsynonymous	0	1
BIRC6	chr2	32743931/A//G	synonymous	0	1
LTBP1	chr2	33246007/G//A	synonymous	0	1
PCBP1	chr2	70315174/T//C	nonsynonymous	0	1
CNTNAP5	chr2	125261907/G//T	synonymous	0	1
SCN9A	chr2	167145057/C//A	nonsynonymous	0	1
HOXD3	chr2	177036312/C//T	synonymous	0	1
UNC80	chr2	210704065/C//T	nonsynonymous	0	1
WNT10A	chr2	219758132/C//T	synonymous	0	1
CFAP65	chr2	219886994/T//C	nonsynonymous	0	1
SEMA5B	chr3	122645390/G//A	nonsynonymous	0	1
PLXNA1	chr3	126748890/C//T	nonsynonymous	0	1
MUC4	chr3	195477812/G//A	synonymous	0	1
KIAA1109	chr4	123192328/A//G	nonsynonymous	0	1
PLEKHG4B	chr5	181731/G//A	nonsynonymous	0	1
EMB	chr5	49698158/G//T	synonymous	0	1
KIF4B	chr5	154394116/C//T	nonsynonymous	0	1
NIPAL4	chr5	156899709/G//T	nonsynonymous	0	1
GRK6	chr5	176863196/C//T	nonsynonymous	0	1
ANKS1A	chr6	35047713/G//A	synonymous	0	1
RSPO3	chr6	127517142/C//A	nonsynonymous	0	1
AGMO	chr7	15405785/A//G	synonymous	0	1
PIP	chr7	142832356/G//A	synonymous	0	1
SSPO	chr7	149474416/C//T	nonsynonymous	0	1
RHEB	chr7	151167740/T//C	nonsynonymous	0	1
FAM110B	chr8	59059801/G//A	nonsynonymous	0	1
COL22A1	chr8	139895349/C//T	nonsynonymous	0	1
PLEC	chr8	145011337/C//T	nonsynonymous	0	1
LCN9	chr9	138555254/C//T	synonymous	0	1
CACNA1B	chr9	140846741/G//A	nonsynonymous	0	1
KIF5B	chr10	32337435/A//G	synonymous	0	1
B3GAT3	chr11	62388113/C//T	nonsynonymous	0	1
LOC105369332	chr11	62601218/G//A	synonymous	0	1
KCNK4	chr11	64060485/C//A	synonymous	0	1
EMSY	chr11	76248847/A//C	nonsynonymous	0	1

	chr11	105836750/G//A	synonymous	0	1
LRRK2	chr12	40716198/G//T	nonsynonymous	0	1
NCKAP5L	chr12	50188879/C//T	nonsynonymous	0	1
LYZ	chr12	69746964/G//T	nonsynonymous	0	1
UBE3B	chr12	109967795/G//A	nonsynonymous	0	1
MAPKAPK5	chr12	112326720/G//A	nonsynonymous	0	1
GALNT9	chr12	132685702/C//A	nonsynonymous	0	1
MPHOSPH8	chr13	20224261/C//T	synonymous	0	1
MTUS2	chr13	29933548/C//A	nonsynonymous	0	1
	chr14	100341280/T//G	synonymous	0	1
ZNF609	chr15	64970645/C//T	nonsynonymous	0	1
NGRN	chr15	90809548/C//A	synonymous	0	1
PITPNM3	chr17	6377928/C//T	synonymous	0	1
MEIOC	chr17	42750761/C//T	nonsynonymous	0	1
RSAD1	chr17	48557446/G//A	nonsynonymous	0	1
TRIM65	chr17	73886916/A//G	nonsynonymous	0	1
DLGAP1	chr18	3879542/G//A	nonsynonymous	0	1
DSG1	chr18	28935130/C//A	nonsynonymous	0	1
LOXHD1	chr18	44140268/C//T	nonsynonymous	0	1
CNDP1	chr18	72250924/G//A	synonymous	0	1
FBXL12	chr19	9922085/G//T	synonymous	0	1
LDLR	chr19	11223997/G//A	synonymous	0	1
CYP4F3	chr19	15769553/G//A	nonsynonymous	0	1
ZNF14	chr19	19823119/G//A	nonsynonymous	0	1
FCGBP	chr19	40366143/G//A	synonymous	0	1
ATF5	chr19	50436236/C//T	nonsynonymous	0	1
ZNF415	chr19	53612491/C//A	nonsynonymous	0	1
KIR3DL2	chr19	55363712/C//A	nonsynonymous	0	1
KIR3DL2	chr19	55365342/C//T	nonsynonymous	0	1
NKX2-4	chr20	21377055/C//T	nonsynonymous	0	1
REM1	chr20	30071987/C//T	synonymous	0	1
KRTAP26-1	chr21	31692266/A//G	nonsynonymous	0	1
ELFN2	chr22	37770921/C//T	synonymous	0	1
AMER1	chrX	63411576/G//A	nonsynonymous	0	1
SAGE1	chrX	134978399/C//A	synonymous	0	1
GPR50	chrX	150348341/C//A	nonsynonymous	0	1
HSPG2	chr1	22199551/T//C	nonsynonymous	1	0
DHDDS	chr1	26786593/C//T	synonymous	1	0
THEMIS2	chr1	28212387/A//C	nonsynonymous	1	0
ZCCHC17	chr1	31821732/C//T	nonsynonymous	1	0
PHC2	chr1	33832876/G//A	nonsynonymous	1	0
LRRIQ3	chr1	74507272/A//C	nonsynonymous	1	0
BCL9	chr1	147091996/C//T	nonsynonymous	1	0
LRRC52	chr1	165532949/G//A	nonsynonymous	1	0
RC3H1	chr1	173931152/T//C	nonsynonymous	1	0
TOR3A	chr1	179054984/G//A	nonsynonymous	1	0
MARK1	chr1	220826478/C//T	nonsynonymous	1	0
PARP1	chr1	226576444/G//A	synonymous	1	0
TPO	chr2	1481150/C//T	nonsynonymous	1	0
RNASEH1	chr2	3605795/C//T	nonsynonymous	1	0
EHD3	chr2	31467209/G//A	synonymous	1	0
CTNNA2	chr2	80782972/T//A	synonymous	1	0
IL1RL1	chr2	102968037/C//T	nonsynonymous	1	0
POTEF	chr2	130877752/T//C	nonsynonymous	1	0
ACVR2A	chr2	148684725/TAGGTG//T	nonsynonymous	1	0
TTN	chr2	179478950/C//A	nonsynonymous	1	0
SPHKAP	chr2	228881614/C//T	nonsynonymous	1	0
PDE6D	chr2	232602764/C//T	nonsynonymous	1	0
IL5RA	chr3	3146655/G//A	nonsynonymous	1	0
VGLL4	chr3	11643490/G//A	nonsynonymous	1	0
TRANK1	chr3	36897248/G//A	nonsynonymous	1	0
ACKR2	chr3	42906001/G//A	nonsynonymous	1	0
DCAF1	chr3	51451440/C//T	nonsynonymous	1	0
EPHA6	chr3	97202787/C//A	nonsynonymous	1	0
TFDP2	chr3	141682808/G//C	synonymous	1	0
TMPRSS11A	chr4	68777170/G//T	nonsynonymous	1	0
GK2	chr4	80329159/C//T	nonsynonymous	1	0
MTTP	chr4	100529959/C//T	nonsynonymous	1	0
NFKB1	chr4	103517422/C//T	synonymous	1	0
PCDH10	chr4	134071730/C//T	synonymous	1	0

EDNRA	chr4	148441044/C//T	synonymous	1	0
TDO2	chr4	156831221/G//A	nonsynonymous	1	0
SORBS2	chr4	186545448/C//T	nonsynonymous	1	0
TPPP	chr5	665227/C//T	nonsynonymous	1	0
APC	chr5	112116511/A//T	nonsynonymous	1	0
APC	chr5	112175621/C//T	nonsynonymous	1	0
PCDHA6	chr5	140207946/C//T	synonymous	1	0
PCDHA10	chr5	140237804/C//T	nonsynonymous	1	0
PCDHA12	chr5	140256419/G//A	synonymous	1	0
FOXI1	chr5	169533108/C//T	synonymous	1	0
FOXI1	chr5	169535586/C//T	nonsynonymous	1	0
HMP19	chr5	173534413/G//A	nonsynonymous	1	0
GPLD1	chr6	24437453/G//A	synonymous	1	0
ADGRB3	chr6	69703861/G//T	synonymous	1	0
COL19A1	chr6	70866045/T//A	nonsynonymous	1	0
SIM1	chr6	100897265/C//T	nonsynonymous	1	0
GPR6	chr6	110300314/C//T	synonymous	1	0
ENPP1	chr6	132173319/G//C	nonsynonymous	1	0
SLC35D3	chr6	137245157/G//A	nonsynonymous	1	0
ADGB	chr6	147045335/C//A	nonsynonymous	1	0
CARD11	chr7	2983996/C//T	synonymous	1	0
ADCY1	chr7	45717490/C//T	nonsynonymous	1	0
SEMA3E	chr7	83016364/C//T	nonsynonymous	1	0
SEMA3A	chr7	83764138/T//A	nonsynonymous	1	0
	chr7	94913395/G//A	synonymous	1	0
TMEM130	chr7	98460911/G//A	synonymous	1	0
RELN	chr7	103180705/C//T	nonsynonymous	1	0
TRPV6	chr7	142573516/G//A	nonsynonymous	1	0
NOBOX	chr7	144097333/G//A	nonsynonymous	1	0
ZFHX4	chr8	77617406/C//T	synonymous	1	0
MMP16	chr8	89086977/A//C	synonymous	1	0
CYP11B1	chr8	143957721/G//A	nonsynonymous	1	0
PCSK5	chr9	78854084/A//G	synonymous	1	0
SPATA31C1	chr9	90535953/G//A	synonymous	1	0
CYLC2	chr9	105767662/A//T	nonsynonymous	1	0
SLC2A6	chr9	136341428/C//T	nonsynonymous	1	0
PROSER2	chr10	11912287/G//A	nonsynonymous	1	0
GJD4	chr10	35896751/G//A	nonsynonymous	1	0
	chr10	64239692/G//T	synonymous	1	0
PNLIPRP3	chr10	118220557/C//T	synonymous	1	0
TUBGCP2	chr10	135096577/C//T	synonymous	1	0
RAG1	chr11	36596118/G//A	nonsynonymous	1	0
CREB3L1	chr11	46342370/C//T	synonymous	1	0
TMEM151A	chr11	66062222/C//T	nonsynonymous	1	0
LRRC32	chr11	76371781/C//A	nonsynonymous	1	0
ANKK1	chr11	113270181/C//T	nonsynonymous	1	0
PRICKLE1	chr12	42853583/T//C	synonymous	1	0
GALNT4	chr12	89916549/T//C	synonymous	1	0
CAMKK2	chr12	121686496/C//T	synonymous	1	0
MMP17	chr12	132326315/G//A	nonsynonymous	1	0
SPERT	chr13	46287785/C//T	nonsynonymous	1	0
DLEU7	chr13	51417861/G//A	synonymous	1	0
PCDH17	chr13	58208595/G//A	nonsynonymous	1	0
SLC10A2	chr13	103718272/T//C	nonsynonymous	1	0
COL4A2	chr13	111164495/G//A	nonsynonymous	1	0
ATP11A	chr13	113487324/G//A	nonsynonymous	1	0
HECTD1	chr14	31576189/GT//G	nonsynonymous	1	0
TTLL5	chr14	76249837/A//T	nonsynonymous	1	0
AHNAK2	chr14	105414243/G//A	synonymous	1	0
ALPK3	chr15	85370777/C//T	nonsynonymous	1	0
RHCG	chr15	90022647/C//T	nonsynonymous	1	0
IGF1R	chr15	99465479/G//A	synonymous	1	0
METRN	chr16	767278/G//A	nonsynonymous	1	0
LCMT1	chr16	25123284/G//A	nonsynonymous	1	0
KIF22	chr16	29810609/C//G	nonsynonymous	1	0
PKD1L3	chr16	72001870/C//T	nonsynonymous	1	0
DHODH	chr16	72045954/G//C	synonymous	1	0
SLFN13	chr17	33768598/G//A	synonymous	1	0
KIF18B	chr17	43009581/G//A	synonymous	1	0
CRHR1	chr17	43861938/G//A	nonsynonymous	1	0

PDK2	chr17	48172713/G//A	synonymous	1	0
MGAT5B	chr17	74942515/G//A	nonsynonymous	1	0
PIGN	chr18	59805519/G//C	nonsynonymous	1	0
NETO1	chr18	70526183/C//T	nonsynonymous	1	0
DPP9-AS1	chr19	4683019/C//T	nonsynonymous	1	0
TUBB4A	chr19	6496178/T//C	nonsynonymous	1	0
MUC16	chr19	9007540/G//A	nonsynonymous	1	0
RGL3	chr19	11516009/C//G	nonsynonymous	1	0
DPF1	chr19	38702616/C//T	synonymous	1	0
RYR1	chr19	38960106/G//T	nonsynonymous	1	0
AXL	chr19	41748815/C//T	nonsynonymous	1	0
DMPK	chr19	46274894/C//T	nonsynonymous	1	0
EHD2	chr19	48220206/G//A	nonsynonymous	1	0
ZNF577	chr19	52376037/T//C	synonymous	1	0
ZNF432	chr19	52537864/T//C	synonymous	1	0
SEC23B	chr20	18523798/C//G	nonsynonymous	1	0
GNAS	chr20	57484421/G//A	nonsynonymous	1	0
ZNF831	chr20	57766932/G//A	synonymous	1	0
SMTN	chr22	31492844/G//A	nonsynonymous	1	0
TTLL1	chr22	43442433/G//A	synonymous	1	0
CFAP47	chrX	36091427/T//C	nonsynonymous	1	0
TAF9B	chrX	77387107/T//A	nonsynonymous	1	0
LPAR4	chrX	78010673/C//T	nonsynonymous	1	0
POU3F4	chrX	82764372/T//C	nonsynonymous	1	0
KLHL4	chrX	86872940/C//A	nonsynonymous	1	0
PRR32	chrX	125954664/C//T	nonsynonymous	1	0
NES	chr1	156639520/G//A	nonsynonymous	0	1
B4GALT3	chr1	161143523/G//A	synonymous	0	1
TGFB2	chr1	218609331/A//G	synonymous	0	1
LRP1B	chr2	140990784/C//A	nonsynonymous	0	1
SEMA3F	chr3	50220861/TC//T	nonsynonymous	0	1
UROC1	chr3	126229551/G//A	synonymous	0	1
KLKB1	chr4	187177207/T//C	synonymous	0	1
TMEM232	chr5	109954212/A//T	nonsynonymous	0	1
THSD7A	chr7	11521483/C//T	nonsynonymous	0	1
ETV1	chr7	13940432/C//T	synonymous	0	1
KLF10	chr8	103663846/C//A	synonymous	0	1
KRTAP5-3	chr11	1629052/G//A	synonymous	0	1
BTBD18	chr11	57512864/C//A	nonsynonymous	0	1
MT1F	chr16	56692659/G//A	synonymous	0	1
CHST4	chr16	71571421/C//T	nonsynonymous	0	1
EVI2A	chr17	29645961/A//G	nonsynonymous	0	1
RBM28	chr7	127983751/A//G	nonsynonymous	1	0
ZNF462	chr9	109689505/C//T	synonymous	1	0
ZNF385C	chr17	40179712/G//T	nonsynonymous	1	0
ZNF729	chr19	22499446/G//A	nonsynonymous	1	0
LRFN3	chr19	36431240/C//T	synonymous	1	0
ITPKC	chr19	41223707/T//C	nonsynonymous	1	0
OFD1	chrX	13753162/A//G	synonymous	1	0
PIK3CD	chr1	9780821/C//G	nonsynonymous	0	1
PUS10	chr2	61180171/C//T	synonymous	0	1
RBM45	chr2	178981080/A//G	nonsynonymous	0	1
ARHGEF3	chr3	56785365/AAT//A	nonsynonymous	0	1
SEC24D	chr4	119644815/G//C	synonymous	0	1
LRRN3	chr7	110764067/G//T	nonsynonymous	0	1
MKRN1	chr7	140171736/A//G	synonymous	0	1
SOX17	chr8	55371625/G//A	synonymous	0	1
THRSP	chr11	77775015/G//A	nonsynonymous	0	1
ALKBH8	chr11	107431552/ATC//A	nonsynonymous	0	1
FAM124A	chr13	51855360/G//A	nonsynonymous	0	1
DIAPH3	chr13	60385061/A//C	synonymous	0	1
ZNF729	chr19	22499505/G//A	nonsynonymous	0	1
FIZ1	chr19	56103904/C//T	nonsynonymous	0	1
C1D	chr2	68273161/G//A	nonsynonymous	0	1
NEK10	chr3	27337127/C//T	nonsynonymous	0	1
ZNF860	chr3	32031962/A//G	nonsynonymous	0	1
INTU	chr4	128637633/A//G	synonymous	0	1
NPC1L1	chr7	44578771/G//C	nonsynonymous	0	1
SEMA3E	chr7	83098591/T//A	nonsynonymous	0	1
MUC12	chr7	100646776/A//G	nonsynonymous	0	1

CSMD3	chr8	113246655/T//A	nonsynonymous	0	1
MSANTD3-TMEFF1	chr9	103204573/A//T	nonsynonymous	0	1
OR5D14	chr11	55563035/A//G	nonsynonymous	0	1
ATM	chr11	108196179/A//G	nonsynonymous	0	1
ACRBP	chr12	6756044/A//G	nonsynonymous	0	1
TFDP1	chr13	114285954/A//C	nonsynonymous	0	1
LTB4R2	chr14	24780718/T//C	nonsynonymous	0	1
GRN	chr17	42428098/C//T	nonsynonymous	0	1
FSD1	chr19	4323377/C//G	nonsynonymous	0	1
ZNF701	chr19	53086598/G//A	nonsynonymous	0	1
KIZ	chr20	21186184/G//A	synonymous	0	1
PISD	chr22	32017064/A//G	synonymous	0	1
MYH9	chr22	36691680/T//C	nonsynonymous	0	1
CDR1	chrX	139865913/T//C	nonsynonymous	0	1
MASP2	chr1	11097837/A//C	synonymous	1	0
ARHGEF2	chr1	155934781/T//G	nonsynonymous	1	0
ZNF512	chr2	27838099/A//G	nonsynonymous	1	0
SLC4A5	chr2	74491425/A//G	synonymous	1	0
SLC9A2	chr2	103299860/C//A	nonsynonymous	1	0
TTN	chr2	179585815/C//T	nonsynonymous	1	0
RNF168	chr3	196214338/T//C	nonsynonymous	1	0
PTCD2	chr5	71654050/A//C	nonsynonymous	1	0
OPN1SW	chr7	128413815/A//T	nonsynonymous	1	0
CSMD3	chr8	113988171/C//T	nonsynonymous	1	0
SCN3B	chr11	123508952/C//T	nonsynonymous	1	0
ABCC9	chr12	22001134/C//T	nonsynonymous	1	0
DDX23	chr12	49226219/T//G	synonymous	1	0
MYH7	chr14	23890230/G//A	synonymous	1	0
RHCG	chr15	90030220/C//T	synonymous	1	0
CACNA1H	chr16	1262079/C//T	nonsynonymous	1	0
INO80E	chr16	30016789/C//G	synonymous	1	0
ZNF267	chr16	31926731/A//G	synonymous	1	0
ZFP1	chr16	75203489/A//G	nonsynonymous	1	0
MYO5B	chr18	47421492/T//C	nonsynonymous	1	0
PDCD5	chr19	33076764/T//C	nonsynonymous	1	0
TNNI3	chr19	55666153/C//T	nonsynonymous	1	0
PTPRT	chr20	40735466/C//T	nonsynonymous	1	0
CSE1L	chr20	47688880/G//A	nonsynonymous	1	0
KRTAP19-1	chr21	31852534/G//A	nonsynonymous	1	0
OSM	chr22	30659978/C//T	nonsynonymous	1	0
ARHGEF16	chr1	3395124/C//T	nonsynonymous	0	1
IL22RA1	chr1	24449813/C//A	synonymous	0	1
PABPC4	chr1	40030855/T//A	nonsynonymous	0	1
NRAS	chr1	115256536/C//A	nonsynonymous	0	1
SNTG2	chr2	1093900/C//T	nonsynonymous	0	1
COL4A4	chr2	227966244/C//G	synonymous	0	1
ING5	chr2	242650831/C//T	nonsynonymous	0	1
CMTM6	chr3	32544121/G//A	synonymous	0	1
DNAH1	chr3	52430484/G//A	nonsynonymous	0	1
PRKCD	chr3	53223190/C//T	synonymous	0	1
LRRC15	chr3	194081584/C//T	synonymous	0	1
SMN1	chr5	70247825/A//T	synonymous	0	1
GRIA1	chr5	153078481/C//T	nonsynonymous	0	1
HCG4	chr6	29760024/G//A	synonymous	0	1
MAP3K4	chr6	161470762/G//A	synonymous	0	1
CSMD1	chr8	3565996/A//C	nonsynonymous	0	1
EBAG9	chr8	110576735/G//A	nonsynonymous	0	1
COL17A1	chr10	105801241/A//ACC	nonsynonymous	0	1
TMEM223	chr11	62558071/G//A	synonymous	0	1
SPDYC	chr11	64940375/G//A	nonsynonymous	0	1
OR10S1	chr11	123847434/C//T	nonsynonymous	0	1
SLC6A12	chr12	301696/A//G	nonsynonymous	0	1
TNS2	chr12	53457027/G//A	synonymous	0	1
USP44	chr12	95918558/C//T	nonsynonymous	0	1
SPIRE2	chr16	89916915/C//T	synonymous	0	1
LIG3	chr17	33313007/A//T	nonsynonymous	0	1
IL12RB1	chr19	18179253/C//T	nonsynonymous	0	1
ZNF229	chr19	44934441/T//A	nonsynonymous	0	1
ZNF541	chr19	48047998/C//T	synonymous	0	1
MACROD2	chr20	14665491/G//A	nonsynonymous	0	1

DNMT3B	chr20	31388105/G//A	nonsynonymous	0	1
BPI	chr20	36948654/A//C	nonsynonymous	0	1
NPEPL1	chr20	57268837/C//G	nonsynonymous	0	1
DSCAM	chr21	41550943/G//T	nonsynonymous	0	1
IL9R	chrX	155239561/C//T	synonymous	0	1
TXLNA	chr1	32646844/G//T	synonymous	0	1
MACF1	chr1	39854065/G//A	nonsynonymous	0	1
KANK4	chr1	62737149/C//A	nonsynonymous	0	1
PDE4B	chr1	66723370/T//C	synonymous	0	1
USP21	chr1	161132788/C//T	nonsynonymous	0	1
ADAMTS4	chr1	161166415/G//A	nonsynonymous	0	1
ASTN1	chr1	177030209/C//G	synonymous	0	1
HMCN1	chr1	185892536/G//A	nonsynonymous	0	1
CDC42BPA	chr1	227300109/T//G	synonymous	0	1
TBCE	chr1	235564884/C//G	nonsynonymous	0	1
VN1R5	chr1	247419516/T//G	nonsynonymous	0	1
EGR4	chr2	73519111/G//A	nonsynonymous	0	1
SULT1C3	chr2	108881786/A//C	synonymous	0	1
AMER3	chr2	131520837/G//T	nonsynonymous	0	1
KYNU	chr2	143718250/T//C	nonsynonymous	0	1
IFIH1	chr2	163163335/G//T	nonsynonymous	0	1
SCN3A	chr2	165947430/A//G	nonsynonymous	0	1
LRP2	chr2	170096286/C//G	nonsynonymous	0	1
DFNB59	chr2	179325821/C//A	synonymous	0	1
TTN	chr2	179401235/C//T	nonsynonymous	0	1
FSIP2	chr2	186669529/T//C	nonsynonymous	0	1
MAP2	chr2	210560347/G//C	nonsynonymous	0	1
CFAP65	chr2	219893099/C//A	nonsynonymous	0	1
PSMD1	chr2	231951864/G//A	nonsynonymous	0	1
SAG	chr2	234229442/G//T	synonymous	0	1
GRM7	chr3	7721735/G//C	nonsynonymous	0	1
RPUSD3	chr3	9881892/G//A	synonymous	0	1
FANCD2	chr3	10091136/G//C	nonsynonymous	0	1
CYP8B1	chr3	42916094/G//A	synonymous	0	1
CCRL2	chr3	46449963/G//A	synonymous	0	1
HYAL2	chr3	50355786/C//G	nonsynonymous	0	1
DCAF1	chr3	51456298/T//C	nonsynonymous	0	1
CADPS	chr3	62543153/C//T	synonymous	0	1
CNTN3	chr3	74347275/A//C	nonsynonymous	0	1
CLDND1	chr3	98241576/G//A	synonymous	0	1
GTPBP8	chr3	112714031/T//C	nonsynonymous	0	1
SPSB4	chr3	140785261/G//A	synonymous	0	1
ZIC1	chr3	147131143/C//T	synonymous	0	1
TTC14	chr3	180326584/A//T	nonsynonymous	0	1
GMNC	chr3	190573066/G//C	synonymous	0	1
	chr4	17847444/A//G	synonymous	0	1
PPARGC1A	chr4	23833296/C//T	nonsynonymous	0	1
DHX15	chr4	24550576/T//C	nonsynonymous	0	1
TECRL	chr4	65194270/T//A	synonymous	0	1
TMPRSS11D	chr4	68699001/G//A	nonsynonymous	0	1
NEUROG2	chr4	113435919/A//C	nonsynonymous	0	1
WDR17	chr4	177056272/C//T	nonsynonymous	0	1
TENM3	chr4	183594221/G//A	nonsynonymous	0	1
TENM3	chr4	183675793/A//C	nonsynonymous	0	1
ZBED3	chr5	76373616/C//A	nonsynonymous	0	1
AP3B1	chr5	77473201/T//C	nonsynonymous	0	1
ST8SIA4	chr5	100191911/T//A	nonsynonymous	0	1
FTMT	chr5	121188029/A//C	nonsynonymous	0	1
PCDHB2	chr5	140475758/G//A	nonsynonymous	0	1
PCDHB10	chr5	140573503/G//A	nonsynonymous	0	1
PCDHB11	chr5	140580943/C//T	synonymous	0	1
PCDHGB6	chr5	140789997/A//G	nonsynonymous	0	1
ABLIM3	chr5	148590370/CAG//C	nonsynonymous	0	1
GABRG2	chr5	161524717/T//C	nonsynonymous	0	1
CNOT6	chr5	179996248/A//T	nonsynonymous	0	1
PIM1	chr6	37138951/C//G	nonsynonymous	0	1
RPL7L1	chr6	42852469/G//A	nonsynonymous	0	1
REV3L	chr6	111696422/G//C	nonsynonymous	0	1
TRDN	chr6	123653045/G//A	nonsynonymous	0	1
T	chr6	166580201/G//A	nonsynonymous	0	1

INTS1	chr7	1529315/G//A	synonymous	0	1
CARD11	chr7	2946427/G//A	nonsynonymous	0	1
ABCA13	chr7	48335319/C//T	nonsynonymous	0	1
ABCA13	chr7	48467363/G//T	nonsynonymous	0	1
C7orf72	chr7	50198761/A//G	nonsynonymous	0	1
PEG10	chr7	94293280/G//C	nonsynonymous	0	1
ZNF394	chr7	99092032/T//C	nonsynonymous	0	1
LHFPL3	chr7	103969677/G//C	synonymous	0	1
TMEM168	chr7	112423979/A//T	nonsynonymous	0	1
DGKI	chr7	137304637/C//A	nonsynonymous	0	1
RAB19	chr7	140125855/C//A	nonsynonymous	0	1
TUSC3	chr8	15531294/C//T	synonymous	0	1
PENK	chr8	57354130/G//A	nonsynonymous	0	1
PREX2	chr8	69058465/CAGAAGG//C	nonsynonymous	0	1
OPLAH	chr8	145113215/G//A	synonymous	0	1
NDUFB6	chr9	32573058/T//A	synonymous	0	1
OSTF1	chr9	77761688/T//G	synonymous	0	1
SVEP1	chr9	113233669/G//C	synonymous	0	1
ZBTB34	chr9	129643020/A//T	nonsynonymous	0	1
CACNA1B	chr9	141016473/G//A	synonymous	0	1
DIP2C	chr10	403880/A//G	synonymous	0	1
KIAA1217	chr10	24832907/A//C	nonsynonymous	0	1
WDFY4	chr10	50011396/A//G	synonymous	0	1
USP54	chr10	75276561/A//T	nonsynonymous	0	1
GRID1	chr10	87487760/C//T	nonsynonymous	0	1
CFAP58	chr10	106152039/G//C	nonsynonymous	0	1
HPX	chr11	6461742/C//G	nonsynonymous	0	1
MRGPRX4	chr11	18194816/G//A	nonsynonymous	0	1
OR4X1	chr11	48285454/A//G	synonymous	0	1
OR5AK2	chr11	56757097/T//C	nonsynonymous	0	1
B3GAT3	chr11	62384575/G//C	nonsynonymous	0	1
PRR4	chr12	10999672/G//A	nonsynonymous	0	1
KRT83	chr12	52711549/G//A	synonymous	0	1
ZNF385A	chr12	54764212/C//A	nonsynonymous	0	1
INHBE	chr12	57850213/C//A	nonsynonymous	0	1
OTOGL	chr12	80750213/T//G	nonsynonymous	0	1
RPH3A	chr12	113304599/G//A	nonsynonymous	0	1
VSIG10	chr12	118517273/C//T	nonsynonymous	0	1
TMEM132B	chr12	126139163/C//T	synonymous	0	1
C1QTNF9B	chr13	24471039/T//G	synonymous	0	1
C1QTNF9B	chr13	24471048/C//T	synonymous	0	1
KL	chr13	33590997/G//A	nonsynonymous	0	1
ZFHX2	chr14	24001800/G//A	synonymous	0	1
C14orf105	chr14	57938004/A//T	synonymous	0	1
ARID4A	chr14	58817854/G//T	nonsynonymous	0	1
SYNE2	chr14	64600834/G//C	synonymous	0	1
GALNT16	chr14	69806254/C//T	nonsynonymous	0	1
LTBP2	chr14	75078222/G//A	synonymous	0	1
TTC7B	chr14	91252523/G//A	nonsynonymous	0	1
TDRD9	chr14	104497583/G//A	nonsynonymous	0	1
CHRNA7	chr15	32460346/C//T	nonsynonymous	0	1
AP3B2	chr15	83346442/C//A	nonsynonymous	0	1
SLC6A2	chr16	55703581/G//A	nonsynonymous	0	1
CDH16	chr16	66943266/C//T	nonsynonymous	0	1
ENKD1	chr16	67698913/T//C	nonsynonymous	0	1
PSKH1	chr16	67961297/C//T	nonsynonymous	0	1
COTL1	chr16	84600401/G//A	synonymous	0	1
ZC3H18	chr16	88643557/G//A	nonsynonymous	0	1
NF1	chr17	29657373/G//T	nonsynonymous	0	1
ASIC2	chr17	32483099/G//C	nonsynonymous	0	1
ERN1	chr17	62131627/C//T	nonsynonymous	0	1
SGSH	chr17	78194122/G//A	synonymous	0	1
MIR6786	chr17	79660885/C//G	synonymous	0	1
THOC1	chr18	267989/G//C	nonsynonymous	0	1
ELOA2	chr18	44561248/G//A	nonsynonymous	0	1
DOHH	chr19	3491666/C//T	nonsynonymous	0	1
FBN3	chr19	8153043/C//T	nonsynonymous	0	1
RAB11B	chr19	8467001/G//T	nonsynonymous	0	1
ADAMTS10	chr19	8650187/G//T	synonymous	0	1
OR2Z1	chr19	8842127/C//T	nonsynonymous	0	1

MAN2B1	chr19	12768335/G//A	synonymous	0	1
EPHX3	chr19	15342838/G//A	nonsynonymous	0	1
ABHD8	chr19	17412293/G//A	nonsynonymous	0	1
ZNF527	chr19	37870107/G//C	nonsynonymous	0	1
RYR1	chr19	39019018/G//A	nonsynonymous	0	1
SPTBN4	chr19	41040025/G//A	synonymous	0	1
PAFAH1B3	chr19	42806422/C//A	nonsynonymous	0	1
APOC1	chr19	45419444/C//G	synonymous	0	1
ACPT	chr19	51298030/CCT//C	synonymous	0	1
NLRP7	chr19	55451569/G//A	synonymous	0	1
CPXM1	chr20	2779403/C//T	synonymous	0	1
VSX1	chr20	25062706/G//A	synonymous	0	1
DNMT3B	chr20	31375235/A//T	nonsynonymous	0	1
ARHGAP40	chr20	37255767/G//A	nonsynonymous	0	1
ACTR5	chr20	37378710/C//T	nonsynonymous	0	1
CDH4	chr20	60511855/G//A	nonsynonymous	0	1
CHRNA4	chr20	61981703/G//A	nonsynonymous	0	1
TPTE	chr21	10907047/A//C	synonymous	0	1
KRTAP13-2	chr21	31743924/GT//G	synonymous	0	1
PAXBP1	chr21	34117939/C//A	nonsynonymous	0	1
RRP1B	chr21	45107674/A//G	synonymous	0	1
ITGB2	chr21	46310083/G//A	synonymous	0	1
CSF2RA	chrX	1413358/G//A	synonymous	0	1
AMER1	chrX	63411405/C//T	nonsynonymous	0	1
AMER1	chrX	63412323/C//A	nonsynonymous	0	1
ATRX	chrX	76907606/C//T	nonsynonymous	0	1
BRWD3	chrX	79964973/G//T	nonsynonymous	0	1
RGAG1	chrX	109694799/A//C	nonsynonymous	0	1
SAGE1	chrX	134992624/C//G	nonsynonymous	0	1
CRTC2	chr1	153924670/G//C	nonsynonymous	0	1
FMO1	chr1	171252304/C//A	nonsynonymous	0	1
ADSS	chr1	244615009/C//T	synonymous	0	1
DNAH6	chr2	84932760/G//C	nonsynonymous	0	1
EDAR	chr2	109545718/G//A	nonsynonymous	0	1
ARHGEF4	chr2	131688536/C//CT	nonsynonymous	0	1
AAMP	chr2	219134255/C//T	nonsynonymous	0	1
LRRC2	chr3	46586591/T//C	nonsynonymous	0	1
NUDT16	chr3	131100696/G//T	synonymous	0	1
UVSSA	chr4	1347046/C//T	nonsynonymous	0	1
ADRA2C	chr4	3769626/G//T	synonymous	0	1
BOD1L1	chr4	13600640/T//A	synonymous	0	1
SLIT2	chr4	20598253/G//T	nonsynonymous	0	1
TBC1D19	chr4	26675410/A//G	nonsynonymous	0	1
KIAA1211	chr4	57182581/C//T	synonymous	0	1
EPHA5	chr4	66217156/C//A	nonsynonymous	0	1
UGT2B11	chr4	70074069/C//T	synonymous	0	1
SLC4A4	chr4	72222724/G//C	nonsynonymous	0	1
UGT8	chr4	115589437/A//G	synonymous	0	1
FAT4	chr4	126372614/G//A	synonymous	0	1
GUCY1B3	chr4	156696211/A//G	nonsynonymous	0	1
ETFDH	chr4	159606275/C//G	synonymous	0	1
HAND2	chr4	174450394/T//C	nonsynonymous	0	1
WDR17	chr4	177094438/T//A	nonsynonymous	0	1
TRIML2	chr4	189022294/A//T	synonymous	0	1
TLX3	chr5	170737189/C//T	nonsynonymous	0	1
GJA10	chr6	90604298/T//A	synonymous	0	1
BACH2	chr6	90661498/G//T	synonymous	0	1
VGLL2	chr6	117593671/G//T	synonymous	0	1
ROS1	chr6	117662756/G//C	nonsynonymous	0	1
FAM184A	chr6	119345815/A//G	nonsynonymous	0	1
ACTB	chr7	5567757/C//T	nonsynonymous	0	1
C7orf49	chr7	134851484/A//G	nonsynonymous	0	1
PIWIL2	chr8	22146176/G//A	nonsynonymous	0	1
CCNE2	chr8	95893875/T//C	synonymous	0	1
FAM208B	chr10	5784296/C//G	nonsynonymous	0	1
ITGA8	chr10	15686213/G//T	synonymous	0	1
SORCS3	chr10	107015561/T//C	nonsynonymous	0	1
PPP1R14B	chr11	64012671/C//T	synonymous	0	1
HEPHL1	chr11	93808527/C//T	synonymous	0	1
PAN2	chr12	56720595/G//A	synonymous	0	1

LTA4H	chr12	96429220/G//C	nonsynonymous	0	1
OR4Q3	chr14	20216160/G//T	nonsynonymous	0	1
ZFHX2	chr14	23993108/T//C	nonsynonymous	0	1
HECTD1	chr14	31626181/G//A	synonymous	0	1
AKAP6	chr14	32902960/A//T	synonymous	0	1
AKAP6	chr14	33291740/G//T	nonsynonymous	0	1
TDP1	chr14	90499490/G//T	nonsynonymous	0	1
	chr14	94430817/G//T	synonymous	0	1
DICER1	chr14	95569869/C//A	nonsynonymous	0	1
BDKRB1	chr14	96730781/G//T	synonymous	0	1
YY1	chr14	100705985/T//A	nonsynonymous	0	1
KIF26A	chr14	104643225/G//T	nonsynonymous	0	1
LOC727924	chr15	22369055/G//T	synonymous	0	1
APBA2	chr15	29347016/G//T	nonsynonymous	0	1
FAN1	chr15	31197051/A//T	nonsynonymous	0	1
PLCB2	chr15	40584601/G//A	synonymous	0	1
FAM63B	chr15	59064243/C//T	synonymous	0	1
AGBL1	chr15	87572156/T//A	synonymous	0	1
BCAR1	chr16	75276497/G//A	synonymous	0	1
ATAD5	chr17	29182286/T//C	nonsynonymous	0	1
FFAR1	chr19	35842664/G//A	synonymous	0	1
SHKBP1	chr19	41096684/A//G	nonsynonymous	0	1
ZNF541	chr19	48049123/G//A	synonymous	0	1
KIR3DL1	chr19	55341643/G//A	synonymous	0	1
KRTAP10-10	chr21	46058107/C//A	synonymous	0	1
LZTR1	chr22	21348869/C//T	synonymous	0	1
LZTR1	chr22	21349173/C//T	synonymous	0	1
LZTR1	chr22	21350048/C//T	synonymous	0	1
ITIH6	chrX	54777533/T//C	synonymous	0	1
ERCC3	chr2	128044339/C//T	nonsynonymous	0	1
NEB	chr2	152397217/G//T	synonymous	0	1
ZNF732	chr4	265482/T//C	synonymous	0	1
ARL15	chr5	53467715/G//T	nonsynonymous	0	1
TNXB	chr6	32035620/C//T	nonsynonymous	0	1
MDN1	chr6	90383955/C//T	nonsynonymous	0	1
EHMT1	chr9	140706014/C//T	synonymous	0	1
MUC5B	chr11	1272548/T//C	nonsynonymous	0	1
KIF5A	chr12	57962815/C//A	nonsynonymous	0	1
MDGA2	chr14	47426801/T//G	nonsynonymous	0	1
RABEP1	chr17	5235383/A//G	synonymous	0	1
SOX9	chr17	70119928/C//CG	nonsynonymous	0	1
ZNF57	chr19	2901036/G//T	synonymous	0	1
TDRD12	chr19	33263733/A//C	nonsynonymous	0	1
PAK5	chr20	9561313/C//T	nonsynonymous	0	1
KCNS1	chr20	43726359/C//T	nonsynonymous	0	1
ZCCHC18	chrX	103358711/C//T	synonymous	0	1
DLGAP3	chr1	35332794/T//G	nonsynonymous	0	1
FPGT-TNNI3K	chr1	74957901/G//A	nonsynonymous	0	1
GFI1	chr1	92948553/A//G	nonsynonymous	0	1
OR10X1	chr1	158548766/C//A	nonsynonymous	0	1
SPTA1	chr1	158632690/T//A	nonsynonymous	0	1
OR10J1	chr1	159409945/C//T	nonsynonymous	0	1
HSPA6	chr1	161496264/C//T	nonsynonymous	0	1
ATP6V1G3	chr1	198492396/T//A	synonymous	0	1
CYB5R1	chr1	202932244/C//T	nonsynonymous	0	1
PTPN14	chr1	214557025/G//A	nonsynonymous	0	1
DISC1	chr1	231830153/C//G	nonsynonymous	0	1
CHRM3	chr1	240071698/C//A	nonsynonymous	0	1
PEX13	chr2	61275825/GAATCTGTTTTTGTTG/	nonsynonymous	0	1
SH3RF3	chr2	110015057/C//T	synonymous	0	1
RAB3GAP1	chr2	135872936/C//G	nonsynonymous	0	1
ABCA12	chr2	215797303/T//C	synonymous	0	1
TNS1	chr2	218683158/G//A	synonymous	0	1
OBSL1	chr2	220435552/G//T	synonymous	0	1
NGEF	chr2	233748731/G//C	nonsynonymous	0	1
EPHA3	chr3	89259150/G//T	nonsynonymous	0	1
FILIP1L	chr3	99569771/G//A	nonsynonymous	0	1
IMPG2	chr3	100994581/G//C	nonsynonymous	0	1
EREG	chr4	75231097/G//A	synonymous	0	1
NAAA	chr4	76836059/A//C	nonsynonymous	0	1

HERC5	chr4	89390310/T//A	nonsynonymous	0	1
TRAM1L1	chr4	118006515/G//T	nonsynonymous	0	1
USP53	chr4	120161062/A//C	synonymous	0	1
CDH9	chr5	26906093/G//A	synonymous	0	1
MRPS30	chr5	44815317/G//C	synonymous	0	1
APC	chr5	112173704/C//T	nonsynonymous	0	1
SLC12A2	chr5	127420206/CCGG//C	nonsynonymous	0	1
CSF2	chr5	131409503/A//C	synonymous	0	1
ANKHD1	chr5	139781756/G//A	synonymous	0	1
PCDHA7	chr5	140215714/G//A	synonymous	0	1
PCDHA11	chr5	140250356/C//T	synonymous	0	1
PCDHB2	chr5	140476595/G//A	nonsynonymous	0	1
DOCK2	chr5	169412924/C//G	nonsynonymous	0	1
DBN1	chr5	176894033/G//A	nonsynonymous	0	1
HIST1H2BG	chr6	26216831/C//T	nonsynonymous	0	1
DNAH8	chr6	38819406/C//T	nonsynonymous	0	1
CRIP3	chr6	43274012/G//T	nonsynonymous	0	1
COL21A1	chr6	56032966/G//T	nonsynonymous	0	1
RNGTT	chr6	89554232/G//A	synonymous	0	1
METTL24	chr6	110620290/A//G	synonymous	0	1
RAET1L	chr6	150342092/G//C	nonsynonymous	0	1
ERMARD	chr6	170175446/C//T	synonymous	0	1
FERD3L	chr7	19184566/G//A	synonymous	0	1
CCDC129	chr7	31614266/G//A	nonsynonymous	0	1
IKZF1	chr7	50358674/G//A	nonsynonymous	0	1
POM121L12	chr7	53103944/G//A	nonsynonymous	0	1
RUNDC3B	chr7	87370873/C//A	nonsynonymous	0	1
ZNF804B	chr7	88964521/T//A	nonsynonymous	0	1
KRBA1	chr7	149430772/G//A	nonsynonymous	0	1
CRYGN	chr7	151127133/C//A	synonymous	0	1
SNTG1	chr8	51621474/G//T	nonsynonymous	0	1
RIMS2	chr8	104898124/C//T	nonsynonymous	0	1
SAMD12	chr8	119391749/C//G	nonsynonymous	0	1
TRMT12	chr8	125463346/C//G	nonsynonymous	0	1
ELAVL2	chr9	23701434/T//C	nonsynonymous	0	1
CKS2	chr9	91930143/G//A	nonsynonymous	0	1
	chr9	96846925/G//A	synonymous	0	1
ANKS6	chr9	101552690/C//T	synonymous	0	1
KIAA0368	chr9	114180257/C//T	nonsynonymous	0	1
NOTCH1	chr9	139399152/C//G	nonsynonymous	0	1
ANKRD30A	chr10	37421172/G//T	nonsynonymous	0	1
TET1	chr10	70451227/G//A	nonsynonymous	0	1
KIF20B	chr10	91514322/A//G	synonymous	0	1
R3HCC1L	chr10	99995191/C//T	nonsynonymous	0	1
EMX2	chr10	119302967/C//G	synonymous	0	1
SLC5A12	chr11	26692651/A//G	nonsynonymous	0	1
BDNF	chr11	27679922/C//T	nonsynonymous	0	1
WT1	chr11	32456807/C//G	nonsynonymous	0	1
HIPK3	chr11	33373166/A//G	synonymous	0	1
LRRC4C	chr11	40136829/C//T	synonymous	0	1
ACCS	chr11	44089364/C//T	synonymous	0	1
CD6	chr11	60785783/C//A	nonsynonymous	0	1
SLC22A11	chr11	64331795/C//T	synonymous	0	1
CTTN	chr11	70265950/G//A	nonsynonymous	0	1
GRM5	chr11	88780843/A//G	synonymous	0	1
FAT3	chr11	92533820/C//G	nonsynonymous	0	1
DYNC2H1	chr11	103158334/G//T	nonsynonymous	0	1
	chr11	113101946/G//T	synonymous	0	1
C11orf63	chr11	122756647/G//C	nonsynonymous	0	1
NRGN	chr11	124615541/G//A	nonsynonymous	0	1
VSIG2	chr11	124621378/T//C	nonsynonymous	0	1
SRPRA	chr11	126138664/C//T	synonymous	0	1
SLC2A14	chr12	7981463/C//A	nonsynonymous	0	1
ADAMTS20	chr12	43826173/G//A	synonymous	0	1
KITLG	chr12	88926287/A//G	synonymous	0	1
POC1B	chr12	89885733/T//C	synonymous	0	1
NUAK1	chr12	106461121/G//T	nonsynonymous	0	1
LHX5	chr12	113901366/C//T	synonymous	0	1
DNAH10	chr12	124383261/C//T	synonymous	0	1
SFSWAP	chr12	132241165/G//A	nonsynonymous	0	1

MMP17	chr12	132328545/T//C	nonsynonymous	0	1
ULK1	chr12	132405699/C//T	nonsynonymous	0	1
PABPC3	chr13	25671361/C//T	nonsynonymous	0	1
FRY	chr13	32805413/T//C	nonsynonymous	0	1
SOHLH2	chr13	36744743/G//A	synonymous	0	1
POSTN	chr13	38138692/G//T	nonsynonymous	0	1
PCDH9	chr13	67477676/G//T	nonsynonymous	0	1
KCTD12	chr13	77460146/C//T	synonymous	0	1
DCT	chr13	95112407/G//T	nonsynonymous	0	1
ING1	chr13	111368158/C//G	nonsynonymous	0	1
AHNAK2	chr14	105410732/G//T	nonsynonymous	0	1
SNRPN	chr15	25213215/C//A	synonymous	0	1
REC114	chr15	73832904/A//G	nonsynonymous	0	1
CRAMP1	chr16	1664834/C//T	nonsynonymous	0	1
MEFV	chr16	3293258/G//A	synonymous	0	1
APOBR	chr16	28509421/T//C	nonsynonymous	0	1
CNOT1	chr16	58610468/G//A	nonsynonymous	0	1
ZC3H18	chr16	88694073/T//G	nonsynonymous	0	1
CBFA2T3	chr16	88947860/C//T	nonsynonymous	0	1
VPS53	chr17	422408/C//T	nonsynonymous	0	1
ASIC2	chr17	31355278/G//A	nonsynonymous	0	1
RUNDC3A	chr17	42392583/C//G	nonsynonymous	0	1
EFCAB3	chr17	60469270/G//C	nonsynonymous	0	1
ANKRD30B	chr18	14782537/G//C	nonsynonymous	0	1
PIGN	chr18	59828548/A//C	nonsynonymous	0	1
MBD3L2	chr19	7051420/G//A	synonymous	0	1
TIMM44	chr19	7999018/C//T	nonsynonymous	0	1
PRAM1	chr19	8563583/G//A	nonsynonymous	0	1
OR10H4	chr19	16060413/T//G	nonsynonymous	0	1
SIN3B	chr19	16976437/G//A	nonsynonymous	0	1
MYBPC2	chr19	50954553/C//T	synonymous	0	1
ZNF766	chr19	52794023/T//G	nonsynonymous	0	1
UBE2S	chr19	55915737/G//T	nonsynonymous	0	1
ZFP28	chr19	57050365/G//T	synonymous	0	1
HSPA12B	chr20	3731488/C//T	nonsynonymous	0	1
GINS1	chr20	25397760/G//A	nonsynonymous	0	1
SLC35C2	chr20	44983780/A//C	nonsynonymous	0	1
COL20A1	chr20	61940018/C//A	synonymous	0	1
ADAMTS1	chr21	28214737/C//A	nonsynonymous	0	1
PI4KA	chr22	21064236/C//A	nonsynonymous	0	1
PI4KA	chr22	21097060/A//T	synonymous	0	1
C22orf42	chr22	32548049/C//T	synonymous	0	1
SHANK3	chr22	51160349/G//A	nonsynonymous	0	1
RBMXL3	chrX	114427128/C//T	nonsynonymous	0	1
MYOM3	chr1	24419461/G//A	nonsynonymous	0	1
DBT	chr1	100701028/A//G	nonsynonymous	0	1
SUSD4	chr1	223402618/G//T	nonsynonymous	0	1
RYR2	chr1	237872811/G//A	nonsynonymous	0	1
IL1R2	chr2	102641110/C//T	synonymous	0	1
RGPD4	chr2	108443467/G//A	synonymous	0	1
RGPD4	chr2	108455303/G//T	synonymous	0	1
	chr3	18392838/C//T	synonymous	0	1
STAC	chr3	36534690/C//T	synonymous	0	1
UBA7	chr3	49849858/C//T	nonsynonymous	0	1
CHST2	chr3	142840967/C//T	nonsynonymous	0	1
PCDHB11	chr5	140580124/G//A	synonymous	0	1
FAT2	chr5	150932762/C//T	nonsynonymous	0	1
ATF6B	chr6	32084298/C//T	nonsynonymous	0	1
PLG	chr6	161174017/G//A	nonsynonymous	0	1
MUC17	chr7	100676930/G//A	nonsynonymous	0	1
C9orf85	chr9	74561949/G//A	nonsynonymous	0	1
WDFY4	chr10	49931515/C//A	nonsynonymous	0	1
TRIM22	chr11	5719757/G//A	synonymous	0	1
ATM	chr11	108115600/C//T	nonsynonymous	0	1
KIAA1551	chr12	32137850/A//G	nonsynonymous	0	1
NABP2	chr12	56619209/G//A	synonymous	0	1
TNFSF11	chr13	43155352/C//G	nonsynonymous	0	1
DNAJC3	chr13	96412373/G//A	nonsynonymous	0	1
NPAP1	chr15	24921354/G//A	nonsynonymous	0	1
IGDCC4	chr15	65681297/G//A	synonymous	0	1

ZNF814	chr19	58385416/C//T	nonsynonymous	0	1
ZNF814	chr19	58385457/T//C	nonsynonymous	0	1
ZNF814	chr19	58385460/T//G	nonsynonymous	0	1
WFDC3	chr20	44417634/C//G	nonsynonymous	0	1
HDAC1	chr1	32796474/A//G	nonsynonymous	0	1
MAST2	chr1	46476408/G//A	nonsynonymous	0	1
MRPS9	chr2	105713637/C//CA	nonsynonymous	0	1
RTP5	chr2	242814314/G//A	nonsynonymous	0	1
OR5H1	chr3	97852211/G//T	nonsynonymous	0	1
ST3GAL6	chr3	98512520/A//C	nonsynonymous	0	1
AADACL2	chr3	151475326/C//T	nonsynonymous	0	1
IBSP	chr4	88732780/C//A	synonymous	0	1
DCLK2	chr4	151114378/T//C	nonsynonymous	0	1
SLC25A4	chr4	186066084/A//G	nonsynonymous	0	1
PCDHB7	chr5	140553838/C//T	synonymous	0	1
CYP21A2	chr6	32008819/C//T	nonsynonymous	0	1
VPS52	chr6	33231635/G//A	nonsynonymous	0	1
PTCRA	chr6	42883834/C//T	synonymous	0	1
NXPH1	chr7	8790740/T//A	nonsynonymous	0	1
DOCK4	chr7	111644164/G//A	synonymous	0	1
FAM160B2	chr8	21955651/G//A	nonsynonymous	0	1
PPP3CC	chr8	22333119/A//G	synonymous	0	1
GATA3	chr10	8100283/G//A	nonsynonymous	0	1
ANO9	chr11	428571/G//A	synonymous	0	1
ARHGEF17	chr11	73074395/G//A	nonsynonymous	0	1
DNM1L	chr12	32866200/C//T	nonsynonymous	0	1
CCDC184	chr12	48578199/C//A	synonymous	0	1
TMEM119	chr12	108985378/T//A	nonsynonymous	0	1
NOS1	chr12	117685275/C//T	nonsynonymous	0	1
	chr13	24471274/G//T	synonymous	0	1
SLITRK5	chr13	88329327/G//A	nonsynonymous	0	1
LRP10	chr14	23346636/C//T	nonsynonymous	0	1
AP5M1	chr14	57753041/A//C	synonymous	0	1
SYNE2	chr14	64460673/T//G	nonsynonymous	0	1
SIPA1L1	chr14	72055119/A//G	nonsynonymous	0	1
PPIP5K1	chr15	43827084/T//G	nonsynonymous	0	1
CEMIP	chr15	81212481/C//T	nonsynonymous	0	1
CNOT1	chr16	58573716/G//A	nonsynonymous	0	1
UNC45B	chr17	33486449/C//A	nonsynonymous	0	1
KRTAP1-5	chr17	39183295/C//T	nonsynonymous	0	1
EFCAB13	chr17	45425299/A//T	nonsynonymous	0	1
CD300E	chr17	72613478/G//A	nonsynonymous	0	1
TMEM94	chr17	73494367/C//T	nonsynonymous	0	1
MAPK4	chr18	48190167/C//T	synonymous	0	1
PPP1R13L	chr19	45885980/G//A	synonymous	0	1
ZNF835	chr19	57175405/G//A	nonsynonymous	0	1
EEF1A2	chr20	62127303/A//T	nonsynonymous	0	1
TUBGCP6	chr22	50659539/T//C	synonymous	0	1
TUBGCP6	chr22	50659548/G//A	synonymous	0	1
NYX	chrX	41333011/C//T	nonsynonymous	0	1
NRK	chrX	105167136/A//C	nonsynonymous	0	1
USP9Y	chrY	14883020/C//T	nonsynonymous	0	1
SLC2A7	chr1	9063388/C//A	nonsynonymous	0	1
PIGK	chr1	77620288/T//A	nonsynonymous	0	1
AQP10	chr1	154296188/A//G	nonsynonymous	0	1
CD1E	chr1	158324351/C//A	synonymous	0	1
OR6K3	chr1	158686988/A//G	synonymous	0	1
KCNJ9	chr1	160053820/C//A	synonymous	0	1
NOS1AP	chr1	162325150/A//G	synonymous	0	1
MAEL	chr1	166990904/G//T	nonsynonymous	0	1
ANKRD45	chr1	173616131/G//T	nonsynonymous	0	1
PIK3C2B	chr1	204438710/C//A	nonsynonymous	0	1
CR1L	chr1	207890911/C//G	nonsynonymous	0	1
USH2A	chr1	215931944/T//A	synonymous	0	1
RPS7	chr2	3624113/A//G	nonsynonymous	0	1
PLB1	chr2	28763270/A//T	nonsynonymous	0	1
XDH	chr2	31572984/G//A	nonsynonymous	0	1
THUMPD2	chr2	39995575/G//T	nonsynonymous	0	1
SLC8A1	chr2	40656725/G//T	synonymous	0	1
SOWAHC	chr2	110372705/C//T	synonymous	0	1

PKP4	chr2	159481856/C//G	nonsynonymous	0	1
XIRP2	chr2	168107831/A//G	nonsynonymous	0	1
FSIP2	chr2	186665802/C//T	synonymous	0	1
SATB2	chr2	200245097/G//T	nonsynonymous	0	1
ERBB4	chr2	212530049/C//A	nonsynonymous	0	1
PER2	chr2	239161814/G//A	synonymous	0	1
CHL1	chr3	440757/G//T	nonsynonymous	0	1
ZCWPW2	chr3	28454850/T//C	synonymous	0	1
TOPAZ1	chr3	44285531/C//A	synonymous	0	1
PLXNB1	chr3	48461659/A//G	nonsynonymous	0	1
COL7A1	chr3	48626415/C//A	synonymous	0	1
CADM2	chr3	86114834/G//A	synonymous	0	1
CPOX	chr3	98311967/T//A	nonsynonymous	0	1
SLC9C1	chr3	112005686/A//G	synonymous	0	1
TF	chr3	133475157/T//A	nonsynonymous	0	1
PPP2R3A	chr3	135721613/A//T	nonsynonymous	0	1
GABRA2	chr4	46312240/A//T	nonsynonymous	0	1
PTPN13	chr4	87662855/A//G	synonymous	0	1
ALPK1	chr4	113362167/G//C	nonsynonymous	0	1
NIPBL	chr5	37017244/A//G	nonsynonymous	0	1
BDP1	chr5	70840887/C//A	nonsynonymous	0	1
VCAN	chr5	82789425/A//G	nonsynonymous	0	1
HAPLN1	chr5	82937440/C//A	nonsynonymous	0	1
PCDHGA12	chr5	140811150/C//T	nonsynonymous	0	1
GABRA6	chr5	161113004/G//T	synonymous	0	1
ADAMTS2	chr5	178699986/T//G	nonsynonymous	0	1
GFPT2	chr5	179765495/G//T	nonsynonymous	0	1
C4B	chr6	31996613/A//G	nonsynonymous	0	1
TNXB	chr6	32041679/G//A	nonsynonymous	0	1
HLA-DQB1-AS1	chr6	32628473/T//C	synonymous	0	1
ADGB	chr6	147061710/T//C	nonsynonymous	0	1
GPR31	chr6	167571229/C//T	nonsynonymous	0	1
DNAAF5	chr7	794237/G//C	nonsynonymous	0	1
EIF2AK1	chr7	6066486/G//A	nonsynonymous	0	1
ADCY1	chr7	45725769/C//A	nonsynonymous	0	1
HEPACAM2	chr7	92821669/CT//C	nonsynonymous	0	1
MUC17	chr7	100678426/C//G	synonymous	0	1
GPR22	chr7	107115527/C//G	nonsynonymous	0	1
MKRN1	chr7	140158824/T//C	nonsynonymous	0	1
BRAF	chr7	140481405/A//G	nonsynonymous	0	1
CSMD1	chr8	2807761/C//G	nonsynonymous	0	1
CSMD1	chr8	3216856/T//A	nonsynonymous	0	1
TNKS	chr8	9414078/A//G	nonsynonymous	0	1
INTS10	chr8	19700402/G//T	nonsynonymous	0	1
HR	chr8	21985178/C//T	synonymous	0	1
DPYSL2	chr8	26439579/T//C	synonymous	0	1
PXDNL	chr8	52361653/G//T	synonymous	0	1
OPRK1	chr8	54142031/A//G	synonymous	0	1
FBXO32	chr8	124553374/G//A	synonymous	0	1
ZNF517	chr8	146033387/G//T	nonsynonymous	0	1
ADAMTSL1	chr9	18636003/C//A	nonsynonymous	0	1
APBA1	chr9	72082830/T//C	nonsynonymous	0	1
FRMD3	chr9	86004519/C//T	synonymous	0	1
SPATA31C1	chr9	90537104/G//T	nonsynonymous	0	1
WNK2	chr9	95992125/C//T	synonymous	0	1
TGFBR1	chr9	101891260/A//T	nonsynonymous	0	1
RNF20	chr9	104324622/G//A	nonsynonymous	0	1
FAM107B	chr10	14816374/T//A	synonymous	0	1
FAM171A1	chr10	15256602/T//A	nonsynonymous	0	1
ANKRD26	chr10	27324503/G//C	nonsynonymous	0	1
RBP3	chr10	48381922/G//T	nonsynonymous	0	1
DLG5	chr10	79588697/C//A	synonymous	0	1
CRTAC1	chr10	99695973/G//C	nonsynonymous	0	1
PHRF1	chr11	607990/G//T	nonsynonymous	0	1
OR51G2	chr11	4936814/C//G	nonsynonymous	0	1
ARHGAP1	chr11	46702071/TACA//T	nonsynonymous	0	1
OR8H1	chr11	56058176/G//T	synonymous	0	1
LRRC55	chr11	56949613/C//A	synonymous	0	1
OR5B17	chr11	58126195/C//A	nonsynonymous	0	1
PTGDR2	chr11	60620580/G//T	nonsynonymous	0	1

FERMT3	chr11	63987048/C//T	nonsynonymous	0	1
BBS1	chr11	66294198/C//T	nonsynonymous	0	1
INPPL1	chr11	71943716/G//T	nonsynonymous	0	1
PHOX2A	chr11	71952294/T//C	nonsynonymous	0	1
CEP126	chr11	101834572/A//G	nonsynonymous	0	1
ARHGAP20	chr11	110454276/T//C	nonsynonymous	0	1
NXPE4	chr11	114453065/A//G	nonsynonymous	0	1
C11orf63	chr11	122817475/A//G	nonsynonymous	0	1
CACNA1C	chr12	2676860/G//T	nonsynonymous	0	1
A2ML1	chr12	9004443/G//C	nonsynonymous	0	1
	chr12	19500079/A//G	synonymous	0	1
HELB	chr12	66709068/A//G	synonymous	0	1
CPSF6	chr12	69645098/T//C	nonsynonymous	0	1
C12orf50	chr12	88383124/C//A	nonsynonymous	0	1
GAS2L3	chr12	101018412/G//C	nonsynonymous	0	1
RPH3A	chr12	113306312/G//T	nonsynonymous	0	1
LHX5	chr12	113901093/C//A	nonsynonymous	0	1
MPHOSPH9	chr12	123665710/C//A	nonsynonymous	0	1
ZNF268	chr12	133780052/G//T	nonsynonymous	0	1
IL17D	chr13	21296149/G//A	synonymous	0	1
ELF1	chr13	41508168/T//C	synonymous	0	1
RUBCNL	chr13	46924319/G//T	nonsynonymous	0	1
ZNF219	chr14	21558970/G//A	nonsynonymous	0	1
CHD8	chr14	21873881/T//C	nonsynonymous	0	1
FBXO33	chr14	39868589/T//C	synonymous	0	1
GOLGA5	chr14	93263997/A//G	nonsynonymous	0	1
SERPINA6	chr14	94780755/C//A	synonymous	0	1
OCA2	chr15	28171368/G//C	nonsynonymous	0	1
HERC2	chr15	28501322/G//A	nonsynonymous	0	1
CTDSPL2	chr15	44791968/C//T	nonsynonymous	0	1
STARD5	chr15	81616503/G//T	synonymous	0	1
ACAN	chr15	89400351/G//C	nonsynonymous	0	1
GRIN2A	chr16	10031932/GC//G	nonsynonymous	0	1
DNAH3	chr16	21065827/A//C	nonsynonymous	0	1
ERN2	chr16	23706116/G//T	nonsynonymous	0	1
VKORC1	chr16	31102617/G//C	synonymous	0	1
CES5A	chr16	55893494/C//G	nonsynonymous	0	1
ZFHX3	chr16	72830277/T//G	nonsynonymous	0	1
MAF	chr16	79632636/G//A	synonymous	0	1
CYB5D2	chr17	4053213/C//A	synonymous	0	1
TP53	chr17	7578556/T//C	nonsynonymous	0	1
NF1	chr17	29684286/G//C	nonsynonymous	0	1
HEATR9	chr17	34186082/G//T	synonymous	0	1
LRRC37A2	chr17	44625995/G//T	nonsynonymous	0	1
COPZ2	chr17	46114262/CT//C	nonsynonymous	0	1
LPO	chr17	56329718/G//A	nonsynonymous	0	1
	chr17	63822349/C//T	synonymous	0	1
ABCA8	chr17	66890327/C//A	nonsynonymous	0	1
MYOM1	chr18	3100355/A//G	synonymous	0	1
CIDEA	chr18	12262838/T//C	nonsynonymous	0	1
ANKRD30B	chr18	14763954/G//C	nonsynonymous	0	1
TMX3	chr18	66344103/T//C	synonymous	0	1
CNDP2	chr18	72173113/T//A	synonymous	0	1
ZNF221	chr19	44470737/A//G	nonsynonymous	0	1
EPS8L1	chr19	55591205/G//A	nonsynonymous	0	1
TGM3	chr20	2312943/G//T	synonymous	0	1
ADAM33	chr20	3650290/C//A	synonymous	0	1
BFSP1	chr20	17475180/A//G	nonsynonymous	0	1
DSTN	chr20	17581599/A//G	nonsynonymous	0	1
RRBP1	chr20	17610500/T//C	nonsynonymous	0	1
CTSA	chr20	44520316/C//T	nonsynonymous	0	1
FAM65C	chr20	49219088/C//A	nonsynonymous	0	1
KCNG1	chr20	49621035/C//G	synonymous	0	1
CTCFL	chr20	56099210/C//A	nonsynonymous	0	1
COL9A3	chr20	61460973/T//A	synonymous	0	1
FBXO7	chr22	32875097/G//A	synonymous	0	1
CACNA1I	chr22	40075205/G//T	nonsynonymous	0	1
MXRA5	chrX	3235264/G//T	nonsynonymous	0	1
ZNF645	chrX	22291576/T//C	synonymous	0	1
MAGEB18	chrX	26157185/C//T	nonsynonymous	0	1

GK	chrX	30709277/T//C	synonymous	0	1
KDM6A	chrX	44922694/C//T	nonsynonymous	0	1
ITIH6	chrX	54785415/T//A	synonymous	0	1
SLC16A2	chrX	73745723/T//C	synonymous	0	1
KLHL4	chrX	86887267/C//A	nonsynonymous	0	1
IRS4	chrX	107978566/T//C	nonsynonymous	0	1
TRPC5	chrX	111155562/T//C	nonsynonymous	0	1
ATP11C	chrX	138850522/C//A	nonsynonymous	0	1
PANK4	chr1	2458066/A//C	synonymous	0	1
INPP5B	chr1	38353986/C//G	synonymous	0	1
ERICH3	chr1	75097618/A//G	synonymous	0	1
CYB561D1	chr1	110038394/C//T	nonsynonymous	0	1
FCGR3B	chr1	161599823/C//G	nonsynonymous	0	1
RRP15	chr1	218475632/A//G	synonymous	0	1
ARID4B	chr1	235383732/T//G	nonsynonymous	0	1
LYST	chr1	235969303/T//C	nonsynonymous	0	1
C1orf101	chr1	244745115/G//C	nonsynonymous	0	1
OR14A16	chr1	247978783/A//T	synonymous	0	1
VAX2	chr2	71160273/C//T	nonsynonymous	0	1
NEB	chr2	152394698/G//C	nonsynonymous	0	1
SCN2A	chr2	166237194/T//A	synonymous	0	1
TLK1	chr2	171910364/C//G	nonsynonymous	0	1
THUMPD3	chr3	9413046/G//A	synonymous	0	1
WNT7A	chr3	13916473/C//T	nonsynonymous	0	1
TTC21A	chr3	39178525/T//C	synonymous	0	1
TTC21A	chr3	39178527/T//G	nonsynonymous	0	1
PTPN23	chr3	47447807/C//T	synonymous	0	1
TLR9	chr3	52256186/C//T	nonsynonymous	0	1
DHX36	chr3	154042189/T//A	nonsynonymous	0	1
NLGN1	chr3	173998959/A//G	nonsynonymous	0	1
ATP13A4	chr3	193232645/G//A	nonsynonymous	0	1
BEND4	chr4	42145714/A//T	nonsynonymous	0	1
TEC	chr4	48158723/T//A	nonsynonymous	0	1
WDFY3	chr4	85696073/T//C	nonsynonymous	0	1
FAT4	chr4	126367446/A//G	synonymous	0	1
MIER3	chr5	56231254/C//T	nonsynonymous	0	1
AGGF1	chr5	76326763/C//G	nonsynonymous	0	1
OTP	chr5	76933026/G//T	nonsynonymous	0	1
PCDHA8	chr5	140221210/T//G	nonsynonymous	0	1
PCDHA8	chr5	140222384/C//G	nonsynonymous	0	1
SOX4	chr6	21595443/C//T	synonymous	0	1
GPX5	chr6	28500126/C//G	nonsynonymous	0	1
PKHD1	chr6	51875159/G//T	nonsynonymous	0	1
RIMS1	chr6	72952062/C//A	nonsynonymous	0	1
GJA1	chr6	121768087/T//C	nonsynonymous	0	1
ETV1	chr7	13935712/C//G	nonsynonymous	0	1
FERD3L	chr7	19184586/C//T	nonsynonymous	0	1
NOD1	chr7	30491162/AGGT//A	nonsynonymous	0	1
NOD1	chr7	30491167/TGCCCACAGG//T	nonsynonymous	0	1
MUC17	chr7	100684021/A//G	synonymous	0	1
PTPRZ1	chr7	121652018/A//C	nonsynonymous	0	1
PAX4	chr7	127253804/C//T	synonymous	0	1
CHRM2	chr7	136699742/G//C	nonsynonymous	0	1
ASH2L	chr8	37990996/T//G	nonsynonymous	0	1
ANK1	chr8	41575628/G//C	nonsynonymous	0	1
PLAT	chr8	42037773/G//T	nonsynonymous	0	1
PDE7A	chr8	66639535/A//T	nonsynonymous	0	1
ZFHX4	chr8	77767286/C//A	nonsynonymous	0	1
CSMD3	chr8	113323376/T//C	synonymous	0	1
GSDMC	chr8	130760986/C//A	nonsynonymous	0	1
OC90	chr8	133036881/G//C	nonsynonymous	0	1
RANBP6	chr9	6013490/T//G	nonsynonymous	0	1
CCDC171	chr9	15729604/A//G	synonymous	0	1
	chr9	18684851/A//G	synonymous	0	1
IPPK	chr9	95432371/C//T	synonymous	0	1
NR4A3	chr9	102606946/C//T	nonsynonymous	0	1
MUSK	chr9	113457811/G//T	nonsynonymous	0	1
TTC16	chr9	130487133/G//A	nonsynonymous	0	1
EBLN1	chr10	22498085/T//G	synonymous	0	1
NRP1	chr10	33502469/T//C	nonsynonymous	0	1

SUPV3L1	chr10	70968608/C//G	synonymous	0	1
DLG5	chr10	79601840/C//T	synonymous	0	1
RBM20	chr10	112595661/G//A	synonymous	0	1
C10orf120	chr10	124457815/T//C	nonsynonymous	0	1
PHRF1	chr11	608928/C//T	nonsynonymous	0	1
CD44	chr11	35250781/G//T	synonymous	0	1
OR9G4	chr11	56510374/T//C	nonsynonymous	0	1
ZNHIT2	chr11	64885138/C//T	synonymous	0	1
NAALAD2	chr11	89883672/A//G	nonsynonymous	0	1
LETMD1	chr12	51449611/C//A	synonymous	0	1
SRGAP1	chr12	64521781/G//A	nonsynonymous	0	1
ATXN7L3B	chr12	74931916/C//T	synonymous	0	1
GLIPR1L2	chr12	75807414/G//A	nonsynonymous	0	1
HPD	chr12	122294261/A//T	nonsynonymous	0	1
AACS	chr12	125626685/C//T	synonymous	0	1
SMAD9	chr13	37446937/G//C	synonymous	0	1
UGGT2	chr13	96454072/A//C	synonymous	0	1
COL4A1	chr13	110855905/T//G	synonymous	0	1
ACIN1	chr14	23549382/G//T	nonsynonymous	0	1
IFT43	chr14	76549605/G//A	synonymous	0	1
LTK	chr15	41799849/T//C	nonsynonymous	0	1
GLDN	chr15	51692573/C//A	nonsynonymous	0	1
NLRC3	chr16	3614068/G//A	synonymous	0	1
SF3B3	chr16	70588956/C//G	synonymous	0	1
TUBB3	chr16	90001295/G//A	nonsynonymous	0	1
USP6	chr17	5045324/C//G	nonsynonymous	0	1
ALOXE3	chr17	8014817/A//T	nonsynonymous	0	1
GPR179	chr17	36486026/G//A	synonymous	0	1
ATP9B	chr18	77063705/T//G	nonsynonymous	0	1
ZNF382	chr19	37117178/C//T	nonsynonymous	0	1
ZNF585A	chr19	37644138/T//A	nonsynonymous	0	1
ZNF574	chr19	42583621/G//A	nonsynonymous	0	1
ZNF473	chr19	50548644/C//G	nonsynonymous	0	1
ZNF761	chr19	53958582/AGT//A	nonsynonymous	0	1
USP29	chr19	57641511/A//T	nonsynonymous	0	1
SIGLEC1	chr20	3677789/G//A	nonsynonymous	0	1
CHGB	chr20	5903660/C//T	synonymous	0	1
CHGB	chr20	5905728/C//A	synonymous	0	1
CFAP61	chr20	20071507/C//G	nonsynonymous	0	1
KRTAP19-6	chr21	31914019/C//T	nonsynonymous	0	1
KRTAP22-1	chr21	31973566/T//C	nonsynonymous	0	1
KRTAP10-2	chr21	45970820/G//T	synonymous	0	1
CECR2	chr22	18028831/A//T	nonsynonymous	0	1
KIAA1671	chr22	25434890/A//G	nonsynonymous	0	1
TTC28	chr22	28379401/G//C	nonsynonymous	0	1
SLC7A3	chrX	70149555/T//C	nonsynonymous	0	1
H6PD	chr1	9305286/A//G	nonsynonymous	0	1
ALDH4A1	chr1	19228965/GTCCAGGGGCGGGAC	nonsynonymous	0	1
NBPF3	chr1	21798079/T//C	nonsynonymous	0	1
CDCA8	chr1	38167486/A//G	nonsynonymous	0	1
UTP11	chr1	38489310/G//C	synonymous	0	1
MACF1	chr1	39950406/A//C	synonymous	0	1
TCHH	chr1	152080106/C//G	nonsynonymous	0	1
CRTC2	chr1	153931096/G//C	synonymous	0	1
ATP8B2	chr1	154315609/C//G	nonsynonymous	0	1
RUSC1	chr1	155296719/C//A	nonsynonymous	0	1
DDX59	chr1	200617700/C//G	synonymous	0	1
NAV1	chr1	201755706/G//C	nonsynonymous	0	1
NVL	chr1	224455731/C//T	synonymous	0	1
PARP1	chr1	226550865/T//C	synonymous	0	1
POMC	chr2	25384395/C//T	nonsynonymous	0	1
GALNT14	chr2	31360996/G//A	synonymous	0	1
USP34	chr2	61417725/A//G	synonymous	0	1
CHCHD5	chr2	113343579/T//C	nonsynonymous	0	1
LCT	chr2	136575345/T//C	nonsynonymous	0	1
SLC4A10	chr2	162804145/G//T	nonsynonymous	0	1
TTC21B	chr2	166781115/G//A	nonsynonymous	0	1
SACM1L	chr3	45761098/G//C	synonymous	0	1
QRICH1	chr3	49070214/A//G	synonymous	0	1
ABHD6	chr3	58256696/A//G	nonsynonymous	0	1

DZIP3	chr3	108391435/C//T	synonymous	0	1
ZNF80	chr3	113955443/A//G	nonsynonymous	0	1
PARP15	chr3	122354967/G//C	synonymous	0	1
PARP15	chr3	122354977/A//C	synonymous	0	1
SEMA5B	chr3	122667372/GTGCTTGCTAAGGG(	nonsynonymous	0	1
RBP2	chr3	139195319/C//A	synonymous	0	1
CPA3	chr3	148601493/G//C	nonsynonymous	0	1
TMEM44	chr3	194336434/G//T	synonymous	0	1
CPEB2	chr4	15009985/A//G	synonymous	0	1
C1QTNF7	chr4	15444081/G//A	synonymous	0	1
CCDC158	chr4	77317449/G//C	synonymous	0	1
THAP9	chr4	83840137/C//G	synonymous	0	1
NDNF	chr4	121957926/ATTCTGAGACAGAAG	nonsynonymous	0	1
BRD9	chr5	864662/A//G	nonsynonymous	0	1
RAD1	chr5	34908983/C//T	nonsynonymous	0	1
DNAJC21	chr5	34950370/G//A	synonymous	0	1
MARVELD2	chr5	68716011/A//G	nonsynonymous	0	1
NR2F1	chr5	92923981/G//T	synonymous	0	1
UBE2D2	chr5	138994367/A//T	synonymous	0	1
PXDC1	chr6	3727807/C//A	nonsynonymous	0	1
DPCR1	chr6	30917219/T//C	synonymous	0	1
PHIP	chr6	79656573/G//A	nonsynonymous	0	1
PDSS2	chr6	107780219/G//A	nonsynonymous	0	1
TMEM248	chr7	66418290/C//G	nonsynonymous	0	1
HIP1	chr7	75189110/C//T	nonsynonymous	0	1
CYP3A7-CYP3A51F	chr7	99319952/A//G	nonsynonymous	0	1
GALNT11	chr7	151805155/G//A	nonsynonymous	0	1
GALNT11	chr7	151805233/G//C	nonsynonymous	0	1
DNAJB6	chr7	157175039/C//G	nonsynonymous	0	1
CHMP7	chr8	23116316/A//T	synonymous	0	1
SLC39A4	chr8	145641404/C//T	synonymous	0	1
NFX1	chr9	33354118/G//A	nonsynonymous	0	1
KIAA1161	chr9	34372821/C//T	nonsynonymous	0	1
PAPPA	chr9	118989827/A//C	synonymous	0	1
KCNT1	chr9	138678253/G//A	nonsynonymous	0	1
FBXW5	chr9	139836132/G//C	nonsynonymous	0	1
AKR1C3	chr10	5147786/G//C	nonsynonymous	0	1
ST8SIA6	chr10	17369110/C//A	nonsynonymous	0	1
SLC18A3	chr10	50818816/C//A	synonymous	0	1
CSTF2T	chr10	53458329/TCGAGGAGGCAGACC	nonsynonymous	0	1
MAT1A	chr10	82045329/C//G	nonsynonymous	0	1
PTEN	chr10	89720680/A//T	synonymous	0	1
NRAP	chr10	115380395/G//A	nonsynonymous	0	1
CTBP2	chr10	126678262/G//A	nonsynonymous	0	1
OR52I2	chr11	4608800/A//G	nonsynonymous	0	1
PPFIBP2	chr11	7672991/G//C	nonsynonymous	0	1
TMEM86A	chr11	18720415/AGCC//A	synonymous	0	1
DCDC5	chr11	30942323/A//C	nonsynonymous	0	1
CRY2	chr11	45893783/G//A	synonymous	0	1
OR4C16	chr11	55340534/T//A	nonsynonymous	0	1
APLNR	chr11	57004359/G//C	synonymous	0	1
CD6	chr11	60775301/A//G	nonsynonymous	0	1
NDUFV1	chr11	67378617/C//G	nonsynonymous	0	1
AMOTL1	chr11	94602619/A//G	synonymous	0	1
CDON	chr11	125887003/G//C	nonsynonymous	0	1
ADAMTS15	chr11	130319382/T//G	nonsynonymous	0	1
OPCML	chr11	132812943/C//T	synonymous	0	1
B4GALNT3	chr12	653514/CGTGCCAACCT//C	nonsynonymous	0	1
TIGAR	chr12	4430410/G//A	synonymous	0	1
VWF	chr12	6166139/T//C	nonsynonymous	0	1
CLEC4D	chr12	8673933/T//C	synonymous	0	1
KRT84	chr12	52771743/C//G	synonymous	0	1
OSBPL8	chr12	76796469/CTTAAGAGATTCTTTT	nonsynonymous	0	1
APAF1	chr12	99043418/C//G	nonsynonymous	0	1
SLC41A2	chr12	105238246/T//C	synonymous	0	1
ANKRD13A	chr12	110450976/A//G	synonymous	0	1
ALDH2	chr12	112223142/A//G	synonymous	0	1
TBX3	chr12	115112499/G//A	nonsynonymous	0	1
SETD1B	chr12	122242664/CCA//C	nonsynonymous	0	1
SETD1B	chr12	122242666/A//C	nonsynonymous	0	1

BRCA2	chr13	32910685/A//G	synonymous	0	1
ELF1	chr13	41533147/A//T	synonymous	0	1
IRS2	chr13	110435229/C//T	nonsynonymous	0	1
ADPRHL1	chr13	114107738/C//G	nonsynonymous	0	1
PRKD1	chr14	30102069/T//C	synonymous	0	1
LRR1	chr14	50069124/G//A	nonsynonymous	0	1
UNC79	chr14	94053172/C//G	nonsynonymous	0	1
CA12	chr15	63634225/G//C	synonymous	0	1
HERC1	chr15	64067047/C//G	nonsynonymous	0	1
CPLX3	chr15	75122727/C//T	synonymous	0	1
TBC1D2B	chr15	78305552/T//C	nonsynonymous	0	1
ARL2BP	chr16	57279230/C//A	synonymous	0	1
CDH11	chr16	65032582/T//A	nonsynonymous	0	1
TXNL4B	chr16	72120574/T//G	nonsynonymous	0	1
RFWD3	chr16	74695257/C//G	nonsynonymous	0	1
CBFA2T3	chr16	88958307/C//G	nonsynonymous	0	1
RNF135	chr17	29298321/T//A	nonsynonymous	0	1
TMEM94	chr17	73492483/C//G	synonymous	0	1
UBE2O	chr17	74392547/A//G	nonsynonymous	0	1
BAHCC1	chr17	79409306/G//C	nonsynonymous	0	1
ANKRD12	chr18	9281004/G//A	nonsynonymous	0	1
TSPAN16	chr19	11407040/G//A	nonsynonymous	0	1
EPOR	chr19	11493822/C//A	nonsynonymous	0	1
ZNF254	chr19	24270043/C//G	synonymous	0	1
ZNF845	chr19	53855304/G//T	nonsynonymous	0	1
ZNF628	chr19	55994600/C//G	synonymous	0	1
CDC25B	chr20	3783027/C//T	synonymous	0	1
PFDN4	chr20	52824652/A//G	nonsynonymous	0	1
EDN3	chr20	57876689/C//A	synonymous	0	1
MYT1	chr20	62839046/C//G	nonsynonymous	0	1
SYNJ1	chr21	34059355/C//G	nonsynonymous	0	1
ATXN10	chr22	46125462/G//C	nonsynonymous	0	1
PUDP	chrX	7023768/G//A	nonsynonymous	0	1
MAGEB16	chrX	35820253/G//C	synonymous	0	1
ALAS2	chrX	55035643/C//T	nonsynonymous	0	1
DLG3	chrX	69665169/G//A	nonsynonymous	0	1
GLUD2	chrX	120182192/C//T	synonymous	0	1
VGLL1	chrX	135630883/C//T	nonsynonymous	0	1
MAGEA11	chrX	148794836/G//T	nonsynonymous	0	1
KCNT2	chr1	196577357/TG//T	nonsynonymous	0	1
IL1R1	chr2	102791961/G//A	nonsynonymous	0	1
THSD7B	chr2	138417308/T//C	nonsynonymous	0	1
CNTN4	chr3	3067878/C//T	synonymous	0	1
KLF15	chr3	126062742/C//A	synonymous	0	1
GRXCR1	chr4	42965137/G//A	nonsynonymous	0	1
GAR1	chr4	110743600/G//A	nonsynonymous	0	1
FREM3	chr4	144621111/C//T	nonsynonymous	0	1
SLC6A3	chr5	1422122/C//T	nonsynonymous	0	1
MYO10	chr5	16769229/G//A	synonymous	0	1
HSPA4	chr5	132406060/T//C	synonymous	0	1
PCDHGA3	chr5	140724411/G//A	nonsynonymous	0	1
KIF4B	chr5	154394377/C//A	nonsynonymous	0	1
DBN1	chr5	176885086/G//T	synonymous	0	1
HIST1H3I	chr6	27839744/C//T	nonsynonymous	0	1
ESR1	chr6	152129315/G//A	nonsynonymous	0	1
TRPM6	chr9	77377786/G//A	synonymous	0	1
BRD3	chr9	136916791/T//G	nonsynonymous	0	1
GLT6D1	chr9	138516030/G//A	synonymous	0	1
KIF5B	chr10	32310022/GTTTCTCTATGGCTCT	nonsynonymous	0	1
VSTM4	chr10	50227728/G//A	synonymous	0	1
LBX1	chr10	102987483/C//T	synonymous	0	1
VWA5A	chr11	123994075/A//T	nonsynonymous	0	1
	chr12	25055951/G//C	synonymous	0	1
GCN1	chr12	120591162/G//A	nonsynonymous	0	1
B3GLCT	chr13	31848665/A//G	nonsynonymous	0	1
RNF219	chr13	79233264/T//G	synonymous	0	1
RTL1	chr14	101347435/C//T	nonsynonymous	0	1
MYO5A	chr15	52615641/T//C	nonsynonymous	0	1
ST8SIA2	chr15	92987949/A//G	nonsynonymous	0	1
CDH5	chr16	66436878/G//A	nonsynonymous	0	1

ZNF469	chr16	88503808/C//T	synonymous	0	1
KANSL1	chr17	44109636/G//T	nonsynonymous	0	1
B3GNTL1	chr17	80923600/G//A	nonsynonymous	0	1
HDGFRP2	chr19	4499538/C//G	synonymous	0	1
ZNF441	chr19	11892490/C//T	synonymous	0	1
CACNA1A	chr19	13394205/C//A	nonsynonymous	0	1
ZNF583	chr19	56918467/C//A	synonymous	0	1
NFS1	chr20	34268698/G//T	nonsynonymous	0	1
TMPRSS15	chr21	19666606/C//T	nonsynonymous	0	1
CLDN17	chr21	31538697/G//A	nonsynonymous	0	1
PDPN	chr1	13910449/G//T	nonsynonymous	0	1
NPL	chr1	182783998/G//A	synonymous	0	1
DHX57	chr2	39095403/C//T	nonsynonymous	0	1
LRRTM4	chr2	77746472/T//C	nonsynonymous	0	1
BIN1	chr2	127811516/T//C	nonsynonymous	0	1
ATR	chr3	142274794/A//G	nonsynonymous	0	1
UNC5C	chr4	96127865/C//T	nonsynonymous	0	1
ASIC5	chr4	156775280/G//T	nonsynonymous	0	1
ZNF862	chr7	149545271/G//T	nonsynonymous	0	1
LETM2	chr8	38257906/G//GCT	nonsynonymous	0	1
RGS22	chr8	101118166/G//A	synonymous	0	1
OR13C9	chr9	107379770/G//A	nonsynonymous	0	1
MASTL	chr10	27454024/A//G	synonymous	0	1
EHBP1L1	chr11	65352963/C//T	synonymous	0	1
HECTD4	chr12	112631430/T//C	nonsynonymous	0	1
SNTB2	chr16	69221110/C//T	nonsynonymous	0	1
SEH1L	chr18	12984136/A//G	synonymous	0	1
CD22	chr19	35827128/G//A	nonsynonymous	0	1
ZNF814	chr19	58385037/C//A	nonsynonymous	0	1
DYNLRB1	chr20	33104272/C//T	synonymous	0	1
USP26	chrX	132161709/C//T	synonymous	0	1
TMEM240	chr1	1470870/G//A	synonymous	0	1
HIVEP3	chr1	42048811/G//T	nonsynonymous	0	1
AK5	chr1	77752748/C//G	nonsynonymous	0	1
FCER1A	chr1	159277538/C//T	nonsynonymous	0	1
EDAR	chr2	109526924/G//T	synonymous	0	1
COL3A1	chr2	189869012/G//A	synonymous	0	1
CADPS	chr3	62739267/C//T	nonsynonymous	0	1
FAT1	chr4	187509932/G//A	synonymous	0	1
GABRP	chr5	170239043/T//C	synonymous	0	1
HIST1H2BC	chr6	26124106/G//A	synonymous	0	1
TDRD6	chr6	46655880/C//T	synonymous	0	1
PEG10	chr7	94294093/C//T	nonsynonymous	0	1
CLCN1	chr7	143016929/G//A	nonsynonymous	0	1
CA2	chr8	86376278/G//T	synonymous	0	1
ADAMTS14	chr10	72511262/G//A	nonsynonymous	0	1
ATRNL1	chr10	117001471/C//T	synonymous	0	1
PYROXD1	chr12	21621501/G//C	nonsynonymous	0	1
	chr12	71029740/C//G	synonymous	0	1
PHLDA1	chr12	76424958/T//C	synonymous	0	1
TRPM1	chr15	31319165/C//A	nonsynonymous	0	1
RGMA	chr15	93588632/G//A	nonsynonymous	0	1
PPL	chr16	4943378/A//G	synonymous	0	1
KCTD19	chr16	67325614/A//ATGCTG	nonsynonymous	0	1
FOXC2	chr16	86600950/G//A	synonymous	0	1
ZFP3	chr17	4996100/C//T	nonsynonymous	0	1
	chr17	61957928/C//T	synonymous	0	1
CARD14	chr17	78163644/C//T	synonymous	0	1
DOT1L	chr19	2226917/G//T	nonsynonymous	0	1
LONP1	chr19	5707830/C//T	nonsynonymous	0	1
RAVER1	chr19	10439448/T//C	nonsynonymous	0	1
VN1R4	chr19	53770752/GCTAAGAAGT//G	nonsynonymous	0	1
HAO1	chr20	7894894/G//A	synonymous	0	1
SPON2	chr4	1161296/T//C	synonymous	0	1
SLC34A2	chr4	25675931/C//G	synonymous	0	1
PDZD2	chr5	32048710/C//T	nonsynonymous	0	1
GSTA3	chr6	52770596/G//A	nonsynonymous	0	1
SLC30A8	chr8	118147641/A//G	synonymous	0	1
FADS2	chr11	61615663/C//G	nonsynonymous	0	1
RASGRF1	chr15	79288118/A//G	synonymous	0	1

ACAN	chr15	89398293/C//A	nonsynonymous	0	1
NTHL1	chr16	2089886/G//T	synonymous	0	1
TNFRSF8	chr1	12123670/C//T	synonymous	0	1
ATP13A2	chr1	17316423/A//T	nonsynonymous	0	1
XKR8	chr1	28293119/C//T	nonsynonymous	0	1
B4GALT2	chr1	44447406/G//A	nonsynonymous	0	1
ZRANB2	chr1	71538161/A//G	synonymous	0	1
BARHL2	chr1	91182581/C//T	nonsynonymous	0	1
LSG1	chr3	194371867/G//A	nonsynonymous	0	1
RGS12	chr4	3318662/C//T	synonymous	0	1
ATP10D	chr4	47582456/T//C	synonymous	0	1
FGA	chr4	155508048/C//T	nonsynonymous	0	1
C5orf42	chr5	37165697/G//A	nonsynonymous	0	1
ADGRV1	chr5	89949052/C//A	nonsynonymous	0	1
TRPC7	chr5	135692974/G//A	synonymous	0	1
PCDHA1	chr5	140166685/C//T	synonymous	0	1
PCDHGA3	chr5	140725839/C//T	nonsynonymous	0	1
HIVEP2	chr6	143074917/C//T	nonsynonymous	0	1
COL1A2	chr7	94045753/G//A	nonsynonymous	0	1
ZAN	chr7	100373129/C//T	nonsynonymous	0	1
BRAF	chr7	140453155/C//T	nonsynonymous	0	1
DENND3	chr8	142146838/C//T	synonymous	0	1
CDKN2A	chr9	21974704/C//T	synonymous	0	1
DENND1A	chr9	126319867/G//A	synonymous	0	1
PLPP7	chr9	134183364/G//A	nonsynonymous	0	1
HMX2	chr10	124907975/C//T	synonymous	0	1
RBMXL2	chr11	7111385/G//A	nonsynonymous	0	1
PLEKHA7	chr11	16812744/T//A	nonsynonymous	0	1
FKBP2	chr11	64011440/G//GC	nonsynonymous	0	1
FOSL1	chr11	65660577/A//G	nonsynonymous	0	1
TPCN2	chr11	68822681/C//T	nonsynonymous	0	1
GRM5	chr11	88780735/C//T	synonymous	0	1
HEBP1	chr12	13128357/C//T	nonsynonymous	0	1
KIAA1551	chr12	32136552/ATACAT//A	nonsynonymous	0	1
OR6C6	chr12	55688832/C//T	nonsynonymous	0	1
UHRF1BP1L	chr12	100453754/T//C	synonymous	0	1
GPR180	chr13	95273389/G//A	nonsynonymous	0	1
CHD8	chr14	21871794/G//A	synonymous	0	1
MYH6	chr14	23869561/G//A	synonymous	0	1
SIX4	chr14	61180093/G//T	synonymous	0	1
TP53BP1	chr15	43783907/G//A	nonsynonymous	0	1
FBN1	chr15	48760714/C//A	nonsynonymous	0	1
DLG4	chr17	7100227/G//A	nonsynonymous	0	1
TBCD	chr17	80890597/C//T	synonymous	0	1
PSMA8	chr18	23713864/C//T	synonymous	0	1
CDH2	chr18	25589803/G//A	nonsynonymous	0	1
SMAD4	chr18	48575111/C//T	nonsynonymous	0	1
MAP2K2	chr19	4101129/G//A	nonsynonymous	0	1
MUC16	chr19	9089079/A//G	synonymous	0	1
C19orf73	chr19	49622133/G//A	synonymous	0	1
AKT1S1	chr19	50373052/G//C	synonymous	0	1
ZSCAN18	chr19	58596036/G//A	synonymous	0	1
TGM6	chr20	2375142/G//A	nonsynonymous	0	1
KIAA1755	chr20	36888951/G//A	synonymous	0	1
COL18A1	chr21	46925297/C//CCCCCCTGGG	nonsynonymous	0	1
SMG5	chr1	156237325/C//T	synonymous	0	1
LOC100506422	chr9	26114453/C//A	nonsynonymous	0	1
STX2	chr12	131283110/T//C	nonsynonymous	0	1
RBPMS2	chr15	65041626/T//C	synonymous	0	1
WDR93	chr15	90276385/C//T	synonymous	0	1
PARD6G	chr18	77960643/T//C	nonsynonymous	0	1
KIAA1755	chr20	36842060/C//A	nonsynonymous	0	1
MYBL2	chr20	42311444/A//G	nonsynonymous	0	1
MMP11	chr22	24124432/C//T	synonymous	0	1
CCDC134	chr22	42209333/G//A	nonsynonymous	0	1
ARHGEF16	chr1	3391282/T//C	nonsynonymous	0	1
PADI6	chr1	17698711/CTGAG//C	synonymous	0	1
C1QB	chr1	22985947/A//C	synonymous	0	1
MATN1	chr1	31194277/C//T	nonsynonymous	0	1
AGO3	chr1	36439119/G//A	synonymous	0	1

BEND5	chr1	49208391/C//T	synonymous	0	1
ZFYVE9	chr1	52704746/C//G	nonsynonymous	0	1
MOV10	chr1	113237176/G//A	nonsynonymous	0	1
PGLYRP3	chr1	153276376/T//G	nonsynonymous	0	1
DNAH14	chr1	225445715/A//C	nonsynonymous	0	1
WDR64	chr1	241901777/G//A	nonsynonymous	0	1
OR2G6	chr1	248685240/T//TG	nonsynonymous	0	1
EFR3B	chr2	25360300/G//T	nonsynonymous	0	1
EPAS1	chr2	46609689/G//A	nonsynonymous	0	1
MTIF2	chr2	55490777/T//C	nonsynonymous	0	1
RNF149	chr2	101911602/C//A	nonsynonymous	0	1
NEB	chr2	152500425/G//A	synonymous	0	1
SCN3A	chr2	165994536/T//G	nonsynonymous	0	1
TFPI	chr2	188332626/G//T	nonsynonymous	0	1
OR5H2	chr3	98002256/T//C	synonymous	0	1
DPPA2	chr3	109023487/A//C	nonsynonymous	0	1
IGSF11	chr3	118621651/A//C	nonsynonymous	0	1
STIM2	chr4	27024514/A//C	nonsynonymous	0	1
CHRNA9	chr4	40337843/G//A	nonsynonymous	0	1
TRPC3	chr4	122853894/C//T	synonymous	0	1
OTUD4	chr4	146071818/T//C	nonsynonymous	0	1
CDH10	chr5	24498507/C//T	synonymous	0	1
MCIDAS	chr5	54518198/C//G	nonsynonymous	0	1
DDX4	chr5	55083757/G//A	synonymous	0	1
MCC	chr5	112363068/C//A	nonsynonymous	0	1
GMNN	chr6	24781720/T//C	nonsynonymous	0	1
TRIM15	chr6	30140162/C//T	synonymous	0	1
DST	chr6	56399921/T//A	nonsynonymous	0	1
ETV1	chr7	13975345/G//T	nonsynonymous	0	1
CACNA2D1	chr7	81589082/C//A	synonymous	0	1
PCLO	chr7	82586203/T//C	nonsynonymous	0	1
ANKIB1	chr7	92015955/G//C	nonsynonymous	0	1
C7orf76	chr7	96114171/A//C	synonymous	0	1
TAS2R39	chr7	142881457/C//T	nonsynonymous	0	1
ASAH1	chr8	17933073/T//G	synonymous	0	1
EIF4EBP1	chr8	37888171/T//C	nonsynonymous	0	1
HTRA4	chr8	38832175/C//T	synonymous	0	1
TXN	chr9	113018803/C//T	synonymous	0	1
CDK5RAP2	chr9	123234136/T//G	nonsynonymous	0	1
COL5A1	chr9	137696825/A//C	nonsynonymous	0	1
COL5A1	chr9	137696830/G//T	nonsynonymous	0	1
DHTKD1	chr10	12143179/A//G	nonsynonymous	0	1
PSAP	chr10	73594216/C//T	synonymous	0	1
ZNF503	chr10	77159396/G//A	nonsynonymous	0	1
APBB1	chr11	6416829/G//A	nonsynonymous	0	1
ILK	chr11	6631688/C//T	synonymous	0	1
BAD	chr11	64039154/G//C	nonsynonymous	0	1
GRK2	chr11	67052440/G//A	nonsynonymous	0	1
GPR152	chr11	67219675/A//G	nonsynonymous	0	1
ARHGEF17	chr11	73067305/G//A	nonsynonymous	0	1
ARHGEF17	chr11	73074283/G//A	nonsynonymous	0	1
GXYLT1	chr12	42512884/T//G	nonsynonymous	0	1
SCAF11	chr12	46321385/G//T	nonsynonymous	0	1
OR6C65	chr12	55795059/T//C	synonymous	0	1
OTOGL	chr12	80729744/C//A	nonsynonymous	0	1
PPFIA2	chr12	81675103/T//A	nonsynonymous	0	1
NR1H4	chr12	100934553/G//A	synonymous	0	1
VWA8	chr13	42164866/T//G	nonsynonymous	0	1
FOXG1	chr14	29237700/C//T	synonymous	0	1
DNAAF2	chr14	50100943/G//A	nonsynonymous	0	1
TJP1	chr15	30053866/G//A	nonsynonymous	0	1
EMC7	chr15	34388111/G//A	nonsynonymous	0	1
FBN1	chr15	48780370/A//G	nonsynonymous	0	1
UNC13C	chr15	54838938/A//C	nonsynonymous	0	1
TCF25	chr16	89962463/C//T	nonsynonymous	0	1
AIPL1	chr17	6338325/T//C	synonymous	0	1
TBX21	chr17	45821620/C//T	synonymous	0	1
PDK2	chr17	48172699/G//A	synonymous	0	1
SDK2	chr17	71364645/A//C	nonsynonymous	0	1
NPLOC4	chr17	79534502/T//C	nonsynonymous	0	1

ARHGDIA	chr17	79827691/T//C	nonsynonymous	0	1
SIRT7	chr17	79870317/C//T	nonsynonymous	0	1
ZBTB7C	chr18	45567343/G//A	nonsynonymous	0	1
ACTL9	chr19	8808608/G//T	nonsynonymous	0	1
FCGBP	chr19	40424321/C//T	nonsynonymous	0	1
HAS1	chr19	52216790/C//T	nonsynonymous	0	1
MAPRE1	chr20	31413783/G//A	nonsynonymous	0	1
ZHX3	chr20	39831911/C//T	nonsynonymous	0	1
DDX17	chr22	38902154/T//C	nonsynonymous	0	1
HFM1	chr1	91859749/A//G	nonsynonymous	0	1
OR6K3	chr1	158687597/G//C	nonsynonymous	0	1
ABL2	chr1	179077710/G//A	nonsynonymous	0	1
CEP170	chr1	243349335/G//A	nonsynonymous	0	1
SIX3	chr2	45169955/G//T	nonsynonymous	0	1
PLEK	chr2	68622889/C//G	nonsynonymous	0	1
RAB11FIP5	chr2	73315611/C//G	nonsynonymous	0	1
LRP2	chr2	170030522/G//A	nonsynonymous	0	1
PDZRN3	chr3	73433966/G//A	nonsynonymous	0	1
CPB1	chr3	148575271/G//C	nonsynonymous	0	1
USP46	chr4	53468139/G//T	synonymous	0	1
TBCK	chr4	107163676/G//A	nonsynonymous	0	1
ANK2	chr4	114204002/G//T	nonsynonymous	0	1
WDR17	chr4	177083249/A//C	nonsynonymous	0	1
COL4A3BP	chr5	74681787/C//T	nonsynonymous	0	1
PCDHB3	chr5	140482102/A//G	synonymous	0	1
SLC22A3	chr6	160828102/C//T	nonsynonymous	0	1
RADIL	chr7	4839097/C//T	nonsynonymous	0	1
GLI3	chr7	42085029/G//C	synonymous	0	1
POM121L12	chr7	53104248/G//T	nonsynonymous	0	1
ZNF395	chr8	28217164/C//T	nonsynonymous	0	1
PRKDC	chr8	48686925/G//A	synonymous	0	1
ANGPT1	chr8	108276561/G//T	nonsynonymous	0	1
ITIH2	chr10	7774309/G//A	synonymous	0	1
SHOC2	chr10	112769139/T//C	nonsynonymous	0	1
MUC5B	chr11	1263343/C//T	synonymous	0	1
FGF4	chr11	69589552/C//T	nonsynonymous	0	1
MOGAT2	chr11	75440016/C//T	nonsynonymous	0	1
PRDM10	chr11	129805108/G//A	nonsynonymous	0	1
VWF	chr12	6182790/C//A	nonsynonymous	0	1
KIF21A	chr12	39713822/T//C	synonymous	0	1
ZFC3H1	chr12	72017325/C//T	nonsynonymous	0	1
PAN3	chr13	28750693/C//T	nonsynonymous	0	1
PAN3	chr13	28751978/AAACTTACGT//A	synonymous	0	1
GMPR2	chr14	24706336/C//T	synonymous	0	1
NID2	chr14	52481893/G//A	synonymous	0	1
MYO9A	chr15	72292250/A//G	nonsynonymous	0	1
C16orf62	chr16	19659098/G//C	synonymous	0	1
ABCA6	chr17	67083578/T//C	synonymous	0	1
OR7G3	chr19	9236774/G//T	nonsynonymous	0	1
ZNF93	chr19	20026183/T//TGTA	nonsynonymous	0	1
NPHS1	chr19	36342383/G//A	nonsynonymous	0	1
SERTAD1	chr19	40929344/G//A	nonsynonymous	0	1
ZNF222	chr19	44537136/C//T	nonsynonymous	0	1
SPATA25	chr20	44515476/G//A	nonsynonymous	0	1
PRPF6	chr20	62626355/C//G	synonymous	0	1
HCFC1	chrX	153219646/G//C	nonsynonymous	0	1
STIL	chr1	47775999/G//A	synonymous	0	1
DYNC1LI1	chr3	32570080/A//G	synonymous	0	1
SLC22A1	chr6	160579669/TGCA//T	synonymous	0	1
LMTK2	chr7	97820130/TAG//T	nonsynonymous	0	1
CTSD	chr11	1780768/G//A	synonymous	0	1
CTSF	chr11	66333616/G//T	nonsynonymous	0	1
DDN	chr12	49391534/T//C	synonymous	0	1
TOX4	chr14	21966576/A//G	synonymous	0	1
MOK	chr14	102698043/C//T	nonsynonymous	0	1
SERF2	chr15	44084481/A//G	synonymous	0	1
KCTD19	chr16	67329255/T//C	synonymous	0	1
AKAP8	chr19	15471675/T//C	nonsynonymous	0	1
ZNF628	chr19	55995074/G//A	synonymous	0	1
TNFRSF8	chr1	12198444/G//A	synonymous	0	1

TCTEX1D4	chr1	45271804/G//A	synonymous	0	1
PTCH2	chr1	45307587/A//T	nonsynonymous	0	1
RABGGTB	chr1	76257955/A//G	synonymous	0	1
BARHL2	chr1	91177957/C//T	nonsynonymous	0	1
TCHHL1	chr1	152058065/T//C	nonsynonymous	0	1
SPTA1	chr1	158606546/T//G	nonsynonymous	0	1
PDC	chr1	186418587/T//C	nonsynonymous	0	1
BRINP3	chr1	190234094/A//T	synonymous	0	1
TLR5	chr1	223286055/T//G	nonsynonymous	0	1
TRIM58	chr1	248020665/C//T	synonymous	0	1
NTSR2	chr2	11798827/G//C	nonsynonymous	0	1
KCNS3	chr2	18113187/C//A	synonymous	0	1
WDCP	chr2	24261940/A//G	nonsynonymous	0	1
THADA	chr2	43814066/C//A	nonsynonymous	0	1
SLC3A1	chr2	44508577/T//A	nonsynonymous	0	1
USP34	chr2	61546459/A//C	nonsynonymous	0	1
LRRTM4	chr2	77746912/G//A	nonsynonymous	0	1
SLC9A4	chr2	103148983/T//G	nonsynonymous	0	1
RANBP2	chr2	109383316/G//C	nonsynonymous	0	1
KIF5C	chr2	149633146/C//T	synonymous	0	1
NEB	chr2	152411464/A//T	nonsynonymous	0	1
TTN	chr2	179410185/G//A	synonymous	0	1
NCKAP1	chr2	183829518/A//T	nonsynonymous	0	1
ALS2CR11	chr2	202410299/G//A	nonsynonymous	0	1
UGT1A6	chr2	234601704/A//C	nonsynonymous	0	1
GRM7	chr3	7503394/C//T	synonymous	0	1
SLC6A1	chr3	11067472/C//T	nonsynonymous	0	1
ZNF620	chr3	40557651/A//G	nonsynonymous	0	1
ERC2	chr3	55733469/ATGG//A	nonsynonymous	0	1
ATG3	chr3	112277257/T//C	nonsynonymous	0	1
DNAJC13	chr3	132179188/T//G	nonsynonymous	0	1
GK5	chr3	141944337/G//C	synonymous	0	1
XRN1	chr3	142051245/G//A	synonymous	0	1
SKIL	chr3	170102401/C//G	nonsynonymous	0	1
CCDC39	chr3	180359814/A//C	nonsynonymous	0	1
KNG1	chr3	186445072/C//T	nonsynonymous	0	1
MXD4	chr4	2254237/G//A	synonymous	0	1
HS3ST1	chr4	11400799/G//A	synonymous	0	1
KIT	chr4	55604670/G//T	nonsynonymous	0	1
TENM3	chr4	183696167/T//G	nonsynonymous	0	1
TENM3	chr4	183714273/C//T	nonsynonymous	0	1
TLR3	chr4	186998105/T//A	nonsynonymous	0	1
ADAMTS16	chr5	5306848/G//A	synonymous	0	1
APC	chr5	112164616/C//T	nonsynonymous	0	1
FBN2	chr5	127700425/T//G	synonymous	0	1
PCDHB1	chr5	140432841/G//C	nonsynonymous	0	1
FAM114A2	chr5	153407729/G//C	nonsynonymous	0	1
DNAH8	chr6	38875805/T//C	nonsynonymous	0	1
CUL7	chr6	43011180/A//G	synonymous	0	1
B3GAT2	chr6	71666052/C//T	synonymous	0	1
MED23	chr6	131925373/G//T	nonsynonymous	0	1
RADIL	chr7	4854917/G//C	nonsynonymous	0	1
RPA3	chr7	7680060/C//A	synonymous	0	1
TYW1B	chr7	72297499/T//C	nonsynonymous	0	1
PPP1R9A	chr7	94881071/A//C	synonymous	0	1
CNOT4	chr7	135098235/A//G	nonsynonymous	0	1
	chr8	22452063/G//A	synonymous	0	1
C8orf86	chr8	38370144/C//A	nonsynonymous	0	1
PSMB7	chr9	127177648/G//A	synonymous	0	1
CFAP157	chr9	130472970/A//G	nonsynonymous	0	1
BRD3	chr9	136917510/A//C	nonsynonymous	0	1
CELF2	chr10	11330406/C//A	nonsynonymous	0	1
FAM171A1	chr10	15255460/G//A	synonymous	0	1
FAM35A	chr10	88911893/G//C	nonsynonymous	0	1
FBXL15	chr10	104180744/C//A	synonymous	0	1
LARGE2	chr11	45946136/C//G	nonsynonymous	0	1
PSMC3	chr11	47447811/A//G	nonsynonymous	0	1
OR5L2	chr11	55594859/C//A	synonymous	0	1
NARS2	chr11	78154724/T//A	nonsynonymous	0	1
SLC37A4	chr11	118895968/T//C	nonsynonymous	0	1

PDZD3	chr11	119057995/G//A	nonsynonymous	0	1
GLB1L3	chr11	134151979/C//A	nonsynonymous	0	1
KDM5A	chr12	427536/A//G	nonsynonymous	0	1
CPNE8	chr12	39079360/G//A	synonymous	0	1
ADAMTS20	chr12	43792989/G//T	nonsynonymous	0	1
ADAMTS20	chr12	43846451/C//T	nonsynonymous	0	1
DPY19L2	chr12	64062058/T//G	nonsynonymous	0	1
P2RX4	chr12	121648064/C//G	nonsynonymous	0	1
RNASEH2B	chr13	51528104/G//C	nonsynonymous	0	1
COL4A1	chr13	110959381/G//A	synonymous	0	1
TRPM7	chr15	50875363/G//C	synonymous	0	1
VPS13C	chr15	62312706/C//G	nonsynonymous	0	1
IGF1R	chr15	99467760/C//T	nonsynonymous	0	1
UBN1	chr16	4907995/A//G	nonsynonymous	0	1
RSL1D1	chr16	11945438/A//C	synonymous	0	1
CCP110	chr16	19547474/A//C	nonsynonymous	0	1
ERN2	chr16	23712363/A//T	nonsynonymous	0	1
CES5A	chr16	55907864/A//T	synonymous	0	1
PFN1	chr17	4849108/C//G	synonymous	0	1
CAMTA2	chr17	4885456/C//G	nonsynonymous	0	1
EPN2	chr17	19213264/T//C	nonsynonymous	0	1
TMUB2	chr17	42266646/G//A	nonsynonymous	0	1
ELOA2	chr18	44560055/C//T	synonymous	0	1
SH3GL1	chr19	4366968/C//A	synonymous	0	1
MUC16	chr19	9024136/G//C	nonsynonymous	0	1
CACNA1A	chr19	13356068/G//A	synonymous	0	1
	chr19	16186935/A//G	synonymous	0	1
NWD1	chr19	16918642/A//AT	nonsynonymous	0	1
COMP	chr19	18896844/TGTC//T	nonsynonymous	0	1
ZNF99	chr19	22941741/G//T	nonsynonymous	0	1
ZNF30	chr19	35434931/G//C	nonsynonymous	0	1
MAMSTR	chr19	49217620/C//T	synonymous	0	1
ZNF211	chr19	58153424/C//CG	nonsynonymous	0	1
CBLN4	chr20	54578942/C//A	nonsynonymous	0	1
LSM14B	chr20	60708464/G//C	nonsynonymous	0	1
DONSON	chr21	34953757/C//G	nonsynonymous	0	1
TCF20	chr22	42608342/C//A	synonymous	0	1
IL1RAPL2	chrX	104440305/C//G	nonsynonymous	0	1
COL4A5	chrX	107866060/G//T	synonymous	0	1
AKAP14	chrX	119037548/C//T	nonsynonymous	0	1
TMEM54	chr1	33360926/C//G	nonsynonymous	0	1
LHCGR	chr2	48958379/C//A	nonsynonymous	0	1
PTPN23	chr3	47448038/G//A	nonsynonymous	0	1
CELSR3	chr3	48692616/C//T	nonsynonymous	0	1
FOXL2	chr3	138665037/G//T	nonsynonymous	0	1
SLC10A4	chr4	48485815/C//T	synonymous	0	1
SV2C	chr5	75427755/G//A	synonymous	0	1
SLC22A5	chr5	131719864/G//A	nonsynonymous	0	1
KLC4	chr6	43039994/G//A	nonsynonymous	0	1
EYS	chr6	66115069/T//G	nonsynonymous	0	1
SYNE1	chr6	152728226/A//AGTT	nonsynonymous	0	1
DENND2A	chr7	140301437/G//A	nonsynonymous	0	1
TNFRSF10C	chr8	22972207/G//A	synonymous	0	1
NEFM	chr8	24773231/C//T	synonymous	0	1
ARFGEF1	chr8	68204316/G//A	nonsynonymous	0	1
TOPORS	chr9	32544029/G//A	nonsynonymous	0	1
TRIM14	chr9	100850295/G//A	synonymous	0	1
ABCA2	chr9	139912149/G//A	synonymous	0	1
C10orf71	chr10	50534757/C//T	synonymous	0	1
GBF1	chr10	104128002/G//A	synonymous	0	1
CPNE8	chr12	39047733/G//A	nonsynonymous	0	1
PLEKHH1	chr14	68042573/C//CG	nonsynonymous	0	1
RBBP6	chr16	24580635/T//A	nonsynonymous	0	1
CHST6	chr16	75513510/C//T	nonsynonymous	0	1
KCNAB3	chr17	7826455/C//T	nonsynonymous	0	1
ZNF521	chr18	22805922/T//C	nonsynonymous	0	1
AP3D1	chr19	2102191/GCCTTCATCTCTTCTAA	nonsynonymous	0	1
SCAF1	chr19	50154898/C//T	nonsynonymous	0	1
AURKC	chr19	57743436/G//A	nonsynonymous	0	1
DYRK1A	chr21	38858806/TTGCCATTAAAATAA//[illegible]	nonsynonymous	0	1

FTHL17	chrX	31089453/C//G	synonymous	0	1
EFHC2	chrX	44202815/G//A	nonsynonymous	0	1
AKAP4	chrX	49958993/C//A	nonsynonymous	0	1
PABPC5	chrX	90691465/A//G	nonsynonymous	0	1
PCDH19	chrX	99661761/C//T	nonsynonymous	0	1
ZMAT1	chrX	101138612/C//T	nonsynonymous	0	1
ARMCX5-GPRASP2	chrX	101971493/G//A	nonsynonymous	0	1
SLC10A3	chrX	153717021/C//T	nonsynonymous	0	1
SAMD11	chr1	877582/T//C	synonymous	1	0
TAS1R3	chr1	1267428/C//A	nonsynonymous	1	0
PEX10	chr1	2337144/A//C	synonymous	1	0
CHD5	chr1	6214827/A//G	nonsynonymous	1	0
MASP2	chr1	11087239/G//A	synonymous	1	0
MTHFR	chr1	11852427/CTAAG//C	nonsynonymous	1	0
TNFRSF8	chr1	12123678/T//G	nonsynonymous	1	0
PRAMEF12	chr1	12835929/C//T	synonymous	1	0
IFFO2	chr1	19235151/C//T	synonymous	1	0
ARID1A	chr1	27089634/G//T	nonsynonymous	1	0
NR0B2	chr1	27240121/G//T	nonsynonymous	1	0
SYTL1	chr1	27680354/CCT//C	synonymous	1	0
MAP3K6	chr1	27682935/G//T	nonsynonymous	1	0
MARCKSL1	chr1	32800513/TTTC//T	nonsynonymous	1	0
BSDC1	chr1	32852373/T//C	synonymous	1	0
RNF19B	chr1	33404103/T//C	nonsynonymous	1	0
SCMH1	chr1	41625463/CAGA//C	synonymous	1	0
PTCH2	chr1	45291982/G//A	synonymous	1	0
POMGNT1	chr1	46657828/T//C	nonsynonymous	1	0
ZYG11B	chr1	53262047/C//G	nonsynonymous	1	0
JAK1	chr1	65325832/C//T	synonymous	1	0
SYDE2	chr1	85648595/G//A	nonsynonymous	1	0
GBP4	chr1	89654412/G//A	synonymous	1	0
ZNF644	chr1	91403277/A//G	synonymous	1	0
HFM1	chr1	91859664/T//C	synonymous	1	0
FAM69A	chr1	93309641/C//T	nonsynonymous	1	0
GNAT2	chr1	110145946/A//G	synonymous	1	0
RBM15	chr1	110883879/C//T	nonsynonymous	1	0
CD101	chr1	117568206/G//T	nonsynonymous	1	0
ITGA10	chr1	145536090/C//T	nonsynonymous	1	0
RPRD2	chr1	150418882/G//A	synonymous	1	0
RPRD2	chr1	150445572/A//G	nonsynonymous	1	0
SETDB1	chr1	150921688/C//A	nonsynonymous	1	0
FLG	chr1	152277610/G//C	nonsynonymous	1	0
FLG	chr1	152278660/G//A	nonsynonymous	1	0
SPRR2F	chr1	153084926/G//A	synonymous	1	0
INTS3	chr1	153740222/C//A	synonymous	1	0
DCST1	chr1	155020368/G//A	nonsynonymous	1	0
GON4L	chr1	155735240/G//A	nonsynonymous	1	0
ARHGEF11	chr1	156928583/G//A	nonsynonymous	1	0
OR6K2	chr1	158669865/G//A	nonsynonymous	1	0
CFAP45	chr1	159846426/C//T	synonymous	1	0
NCSTN	chr1	160326056/C//T	nonsynonymous	1	0
CACNA1E	chr1	181453037/T//C	nonsynonymous	1	0
PLA2G4A	chr1	186823437/G//A	synonymous	1	0
KIF21B	chr1	200977992/C//T	nonsynonymous	1	0
LAD1	chr1	201355836/ATCTTCTCTGATAGA(	nonsynonymous	1	0
IPO9	chr1	201823730/C//A	nonsynonymous	1	0
PM20D1	chr1	205814517/G//C	nonsynonymous	1	0
ITPKB	chr1	226822530/T//C	nonsynonymous	1	0
URB2	chr1	229783467/G//A	nonsynonymous	1	0
KIF26B	chr1	245704144/G//A	synonymous	1	0
RSAD2	chr2	7018057/A//G	synonymous	1	0
APOB	chr2	21230763/C//T	nonsynonymous	1	0
MFSD2B	chr2	24246521/T//C	nonsynonymous	1	0
WDCP	chr2	24253859/C//A	nonsynonymous	1	0
DTNB	chr2	25655840/G//A	nonsynonymous	1	0
SELENOI	chr2	26569067/C//T	synonymous	1	0
ALK	chr2	29551312/A//C	nonsynonymous	1	0
HEATR5B	chr2	37297405/C//T	nonsynonymous	1	0
PREPL	chr2	44571084/T//A	nonsynonymous	1	0
SRBD1	chr2	45616595/T//C	nonsynonymous	1	0

EML6	chr2	55054778/C//A	nonsynonymous	1	0
PAPOLG	chr2	61021263/A//G	synonymous	1	0
DYSF	chr2	71816731/G//A	synonymous	1	0
SFXN5	chr2	73198726/C//A	synonymous	1	0
NAT8B	chr2	73928012/G//A	nonsynonymous	1	0
HK2	chr2	75100475/C//T	nonsynonymous	1	0
USP39	chr2	85843295/T//C	synonymous	1	0
POLR1A	chr2	86267636/G//A	nonsynonymous	1	0
ARID5A	chr2	97213172/C//T	nonsynonymous	1	0
SULT1C3	chr2	108869815/C//T	nonsynonymous	1	0
SH3RF3	chr2	110259146/C//T	synonymous	1	0
WDR33	chr2	128464133/CAA//C	synonymous	1	0
HS6ST1	chr2	129025784/G//A	synonymous	1	0
PTPN18	chr2	131129928/AGACGGG//A	nonsynonymous	1	0
KIF5C	chr2	149633153/C//A	synonymous	1	0
XIRP2	chr2	168101882/G//A	nonsynonymous	1	0
DLX2	chr2	172967139/C//T	nonsynonymous	1	0
RAPGEF4	chr2	173855540/C//T	synonymous	1	0
TTN	chr2	179413088/G//A	nonsynonymous	1	0
TTN	chr2	179594431/G//A	synonymous	1	0
INPP1	chr2	191236199/G//T	synonymous	1	0
IDH1	chr2	209104603/C//T	synonymous	1	0
ANKZF1	chr2	220095103/C//T	nonsynonymous	1	0
OBSL1	chr2	220419233/C//T	synonymous	1	0
DGKD	chr2	234375849/G//T	nonsynonymous	1	0
UGT1A10	chr2	234545231/C//T	synonymous	1	0
TRPM8	chr2	234891703/G//A	nonsynonymous	1	0
COL6A3	chr2	238285505/G//T	nonsynonymous	1	0
OTOS	chr2	241078556/C//T	synonymous	1	0
RNPEPL1	chr2	241516033/G//A	nonsynonymous	1	0
CAPN10	chr2	241531391/C//T	nonsynonymous	1	0
RTP5	chr2	242815165/C//T	synonymous	1	0
SLC6A11	chr3	10980080/C//T	nonsynonymous	1	0
TMEM40	chr3	12777099/C//T	nonsynonymous	1	0
MLH1	chr3	37083821/C//A	nonsynonymous	1	0
SCN5A	chr3	38671832/C//T	nonsynonymous	1	0
NKTR	chr3	42679324/C//T	nonsynonymous	1	0
KLHL40	chr3	42727373/A//G	nonsynonymous	1	0
TDGF1	chr3	46622674/A//G	synonymous	1	0
PLXNB1	chr3	48448281/AGTCT//A	nonsynonymous	1	0
BSN	chr3	49701060/A//G	synonymous	1	0
IP6K1	chr3	49764641/C//T	nonsynonymous	1	0
MANF	chr3	51426378/G//A	nonsynonymous	1	0
FLNB	chr3	58139197/G//A	nonsynonymous	1	0
MORC1	chr3	108833237/T//A	nonsynonymous	1	0
POLQ	chr3	121264723/A//G	nonsynonymous	1	0
SLC12A8	chr3	124826966/C//A	nonsynonymous	1	0
SLCO2A1	chr3	133667794/C//T	nonsynonymous	1	0
STAG1	chr3	136057079/T//C	synonymous	1	0
RASA2	chr3	141277771/T//C	nonsynonymous	1	0
CP	chr3	148895693/C//G	synonymous	1	0
MED12L	chr3	150876506/A//T	nonsynonymous	1	0
IGSF10	chr3	151154496/G//A	nonsynonymous	1	0
LRRC31	chr3	169579608/G//C	synonymous	1	0
PLD1	chr3	171417613/C//A	synonymous	1	0
HRG	chr3	186394973/C//T	synonymous	1	0
TMEM44	chr3	194331664/G//A	nonsynonymous	1	0
XXYLT1	chr3	194991774/C//T	nonsynonymous	1	0
TACC3	chr4	1741737/C//T	synonymous	1	0
WFS1	chr4	6302801/A//AT	nonsynonymous	1	0
MAN2B2	chr4	6590814/C//T	nonsynonymous	1	0
MAN2B2	chr4	6602378/G//A	synonymous	1	0
SH3TC1	chr4	8237257/G//A	nonsynonymous	1	0
BOD1L1	chr4	13571721/G//A	nonsynonymous	1	0
BOD1L1	chr4	13605849/A//G	nonsynonymous	1	0
ADGRA3	chr4	22444461/G//A	synonymous	1	0
PCDH7	chr4	30724309/G//A	nonsynonymous	1	0
SLAIN2	chr4	48424031/C//A	synonymous	1	0
USP46	chr4	53494214/C//T	synonymous	1	0
OPRPN	chr4	71275630/A//G	synonymous	1	0

ENAM	chr4	71508957/C//CA	nonsynonymous	1	0
USO1	chr4	76711856/C//T	synonymous	1	0
DNAJB14	chr4	100822278/G//A	synonymous	1	0
EGF	chr4	110909805/G//A	nonsynonymous	1	0
ANK2	chr4	114276056/A//C	nonsynonymous	1	0
MAD2L1	chr4	120981302/C//T	nonsynonymous	1	0
PCDH18	chr4	138452088/C//T	synonymous	1	0
RAPGEF2	chr4	160262846/C//T	nonsynonymous	1	0
WDR17	chr4	177071618/G//T	nonsynonymous	1	0
SEMA5A	chr5	9136609/C//T	synonymous	1	0
FBXL7	chr5	15936744/G//A	nonsynonymous	1	0
CDH10	chr5	24593511/G//T	nonsynonymous	1	0
C7	chr5	40962246/C//A	nonsynonymous	1	0
C6	chr5	41199899/T//C	nonsynonymous	1	0
TNPO1	chr5	72189299/A//G	synonymous	1	0
CMYA5	chr5	79029009/T//C	nonsynonymous	1	0
ZFYVE16	chr5	79732665/C//T	nonsynonymous	1	0
VCAN	chr5	82817967/C//T	nonsynonymous	1	0
CDKL3	chr5	133644333/C//T	nonsynonymous	1	0
KIF20A	chr5	137515356/C//T	synonymous	1	0
SIL1	chr5	138356887/G//A	nonsynonymous	1	0
ANKHD1	chr5	139906629/GAGA//G	nonsynonymous	1	0
PCDHA10	chr5	140236727/G//A	nonsynonymous	1	0
PCDHAC1	chr5	140306753/C//A	synonymous	1	0
PCDHGA2	chr5	140720014/G//A	synonymous	1	0
PCDHGA2	chr5	140720392/G//A	synonymous	1	0
PCDHGB1	chr5	140731657/C//T	synonymous	1	0
CPEB4	chr5	173317413/T//C	nonsynonymous	1	0
RACK1	chr5	180670753/G//A	synonymous	1	0
ELOVL2	chr6	10990020/C//T	synonymous	1	0
HIST1H2BH	chr6	26251926/G//C	nonsynonymous	1	0
PPP1R18	chr6	30647003/A//T	nonsynonymous	1	0
NRM	chr6	30658638/G//A	synonymous	1	0
BAG6	chr6	31617128/G//C	nonsynonymous	1	0
COL11A2	chr6	33136298/G//A	nonsynonymous	1	0
CPNE5	chr6	36724068/T//C	nonsynonymous	1	0
NCR2	chr6	41303603/G//A	synonymous	1	0
TRERF1	chr6	42236559/A//T	nonsynonymous	1	0
TJAP1	chr6	43473227/C//T	synonymous	1	0
ADGRF5	chr6	46826342/C//T	nonsynonymous	1	0
KIAA1586	chr6	56918789/G//A	nonsynonymous	1	0
EYS	chr6	65707581/G//A	nonsynonymous	1	0
COL12A1	chr6	75865450/G//A	nonsynonymous	1	0
COX7A2	chr6	75953513/G//A	nonsynonymous	1	0
MDN1	chr6	90365627/G//A	nonsynonymous	1	0
OSTM1	chr6	108395631/C//T	synonymous	1	0
TBC1D32	chr6	121481194/T//C	nonsynonymous	1	0
MED23	chr6	131929203/C//T	synonymous	1	0
TAAR2	chr6	132938636/T//C	nonsynonymous	1	0
KATNA1	chr6	149916344/C//A	nonsynonymous	1	0
LRP11	chr6	150157367/G//A	nonsynonymous	1	0
LPA	chr6	161027527/C//G	nonsynonymous	1	0
RPS6KA2	chr6	166826178/C//T	synonymous	1	0
FOXK1	chr7	4801880/C//T	nonsynonymous	1	0
HDAC9	chr7	18767228/G//A	nonsynonymous	1	0
DFNA5	chr7	24738636/C//T	synonymous	1	0
ELMO1	chr7	37262253/C//T	synonymous	1	0
ZMIZ2	chr7	44801090/G//A	nonsynonymous	1	0
MYO1G	chr7	45016247/C//A	nonsynonymous	1	0
SEMA3C	chr7	80432078/C//T	synonymous	1	0
PCLO	chr7	82582851/A//G	nonsynonymous	1	0
PCLO	chr7	82764328/A//G	synonymous	1	0
CROT	chr7	86978407/C//G	nonsynonymous	1	0
CDK14	chr7	90585032/G//A	nonsynonymous	1	0
RBM48	chr7	92166172/C//T	nonsynonymous	1	0
SAMD9	chr7	92732520/G//C	nonsynonymous	1	0
ARPC1B	chr7	98987568/G//A	nonsynonymous	1	0
MUC12	chr7	100638776/AAC//A	nonsynonymous	1	0
TRIM56	chr7	100731470/G//T	nonsynonymous	1	0
VGF	chr7	100806796/G//C	nonsynonymous	1	0

TES	chr7	115890549/A//G	nonsynonymous	1	0
CTTNBP2	chr7	117432272/C//T	nonsynonymous	1	0
LMOD2	chr7	123302169/A//G	nonsynonymous	1	0
OR9A4	chr7	141619394/C//T	nonsynonymous	1	0
OR2A14	chr7	143827014/A//G	nonsynonymous	1	0
ATP6V0E2	chr7	149576809/G//A	synonymous	1	0
ATG9B	chr7	150714980/A//G	nonsynonymous	1	0
CRYGN	chr7	151133308/A//G	nonsynonymous	1	0
KMT2C	chr7	152012318/GT//G	nonsynonymous	1	0
CSMD1	chr8	2820896/G//A	nonsynonymous	1	0
SH2D4A	chr8	19221585/C//T	nonsynonymous	1	0
BNIP3L	chr8	26268008/A//G	synonymous	1	0
CHRNA2	chr8	27327362/G//A	synonymous	1	0
KIF13B	chr8	28928143/C//T	nonsynonymous	1	0
PURG	chr8	30890262/G//C	nonsynonymous	1	0
BRF2	chr8	37707227/C//A	nonsynonymous	1	0
ADAM32	chr8	38994187/C//A	synonymous	1	0
ANK1	chr8	41552155/C//T	synonymous	1	0
OPRK1	chr8	54142387/C//T	nonsynonymous	1	0
NSMAF	chr8	59496766/GAA//G	synonymous	1	0
VCPIP1	chr8	67547585/A//G	synonymous	1	0
MAL2	chr8	120220834/G//A	nonsynonymous	1	0
FAM83A	chr8	124219528/C//T	nonsynonymous	1	0
SQLE	chr8	126017873/A//T	nonsynonymous	1	0
COL22A1	chr8	139895417/G//A	synonymous	1	0
CYP11B1	chr8	143958291/C//T	synonymous	1	0
EPPK1	chr8	144942834/C//G	nonsynonymous	1	0
TAF1L	chr9	32634890/G//A	nonsynonymous	1	0
ABHD17B	chr9	74481643/T//C	synonymous	1	0
RORB	chr9	77249561/G//C	nonsynonymous	1	0
SLC28A3	chr9	86905135/G//A	synonymous	1	0
CDK20	chr9	90589313/T//C	nonsynonymous	1	0
NCBP1	chr9	100420936/A//G	nonsynonymous	1	0
TGFBR1	chr9	101900330/G//A	nonsynonymous	1	0
FSD1L	chr9	108297340/T//G	synonymous	1	0
TNC	chr9	117844085/G//A	synonymous	1	0
DAB2IP	chr9	124535112/G//A	nonsynonymous	1	0
FBXW5	chr9	139837924/C//T	synonymous	1	0
FUT7	chr9	139926173/G//A	synonymous	1	0
NOXA1	chr9	140327534/C//T	synonymous	1	0
DIP2C	chr10	518437/G//A	synonymous	1	0
PITRM1	chr10	3215024/G//T	synonymous	1	0
	chr10	12211341/C//T	synonymous	1	0
CAMK1D	chr10	12803082/C//G	synonymous	1	0
BMS1	chr10	43293948/A//G	nonsynonymous	1	0
SLC18A3	chr10	50820314/G//A	nonsynonymous	1	0
PRKG1	chr10	54053595/A//C	nonsynonymous	1	0
SAR1A	chr10	71913675/A//G	synonymous	1	0
PALD1	chr10	72298858/G//C	nonsynonymous	1	0
VCL	chr10	75865016/G//A	nonsynonymous	1	0
KAT6B	chr10	76790263/C//T	nonsynonymous	1	0
ZNF503	chr10	77159617/C//A	synonymous	1	0
DLG5	chr10	79603415/G//A	nonsynonymous	1	0
FGFBP3	chr10	93668701/G//C	nonsynonymous	1	0
TWNK	chr10	102750242/C//T	nonsynonymous	1	0
SORCS3	chr10	106970916/T//C	synonymous	1	0
HABP2	chr10	115338440/G//A	nonsynonymous	1	0
DCLRE1A	chr10	115612525/C//T	nonsynonymous	1	0
HSPA12A	chr10	118434709/G//A	synonymous	1	0
SLC18A2	chr10	119003780/C//A	synonymous	1	0
TACC2	chr10	123848029/G//T	synonymous	1	0
GPR26	chr10	125447469/G//A	synonymous	1	0
CPXM2	chr10	125521473/G//A	synonymous	1	0
DHX32	chr10	127527595/GCTTT//G	nonsynonymous	1	0
ZNF511	chr10	135126453/G//T	synonymous	1	0
PGGHG	chr11	294170/G//T	synonymous	1	0
B4GALNT4	chr11	380434/G//A	nonsynonymous	1	0
MUC2	chr11	1093878/C//T	synonymous	1	0
BTBD10	chr11	13410543/CTT//C	nonsynonymous	1	0
OR4C6	chr11	55433079/C//T	nonsynonymous	1	0

TNKS1BP1	chr11	57076872/C//A	nonsynonymous	1	0
OR6Q1	chr11	57799100/G//A	nonsynonymous	1	0
PATL1	chr11	59423200/C//T	nonsynonymous	1	0
DAGLA	chr11	61488343/C//T	synonymous	1	0
MYRF	chr11	61548268/C//T	synonymous	1	0
AHNAK	chr11	62288599/T//C	synonymous	1	0
HNRNPUL2	chr11	62491159/T//C	nonsynonymous	1	0
SLC22A8	chr11	62763171/G//A	synonymous	1	0
SLC22A10	chr11	63059095/A//T	synonymous	1	0
CDC42BPG	chr11	64606636/C//A	nonsynonymous	1	0
ATG2A	chr11	64677380/G//A	nonsynonymous	1	0
IGHMBP2	chr11	68682330/C//A	nonsynonymous	1	0
CCND1	chr11	69462749/CCTT//C	synonymous	1	0
KRTAP5-10	chr11	71277137/C//T	synonymous	1	0
KRTAP5-10	chr11	71277185/G//T	nonsynonymous	1	0
P2RY6	chr11	73008384/C//G	nonsynonymous	1	0
DYNC2H1	chr11	103082677/A//G	synonymous	1	0
CASP12	chr11	104757456/G//C	synonymous	1	0
ZBTB16	chr11	113934582/T//C	nonsynonymous	1	0
CEP164	chr11	117265138/C//T	nonsynonymous	1	0
RNF26	chr11	119207148/G//A	synonymous	1	0
OR8G5	chr11	124135531/C//A	nonsynonymous	1	0
HYLS1	chr11	125769907/G//A	nonsynonymous	1	0
KIRREL3	chr11	126326297/G//A	synonymous	1	0
GLB1L3	chr11	134147246/C//T	nonsynonymous	1	0
KDM5A	chr12	420151/G//C	nonsynonymous	1	0
FOXM1	chr12	2981358/C//T	nonsynonymous	1	0
RHNO1	chr12	2997405/A//G	nonsynonymous	1	0
RAD51AP1	chr12	4653057/A//C	nonsynonymous	1	0
LPAR5	chr12	6729554/G//A	synonymous	1	0
COPS7A	chr12	6839962/G//A	nonsynonymous	1	0
USP5	chr12	6970623/C//T	synonymous	1	0
RASSF8	chr12	26221731/C//A	nonsynonymous	1	0
C12orf40	chr12	40114947/C//G	nonsynonymous	1	0
TWF1	chr12	44190865/T//C	nonsynonymous	1	0
SLC38A1	chr12	46591792/G//A	nonsynonymous	1	0
ASB8	chr12	48543447/C//T	nonsynonymous	1	0
CCNT1	chr12	49088064/A//C	synonymous	1	0
PRPF40B	chr12	50026833/CG//C	nonsynonymous	1	0
KRT6B	chr12	52842736/T//C	nonsynonymous	1	0
KRT6A	chr12	52884470/C//T	synonymous	1	0
KRT6A	chr12	52885442/TCA//T	nonsynonymous	1	0
KRT77	chr12	53088529/G//T	nonsynonymous	1	0
TNS2	chr12	53453966/G//C	nonsynonymous	1	0
NXPH4	chr12	57619520/A//G	nonsynonymous	1	0
MON2	chr12	62946730/G//A	nonsynonymous	1	0
DPY19L2	chr12	63974589/G//A	nonsynonymous	1	0
HELB	chr12	66725366/C//T	nonsynonymous	1	0
SART3	chr12	108929156/A//G	nonsynonymous	1	0
SVOP	chr12	109306170/C//G	synonymous	1	0
TMEM116	chr12	112381143/C//T	synonymous	1	0
TBX3	chr12	115118812/C//T	nonsynonymous	1	0
MLEC	chr12	121134453/G//C	synonymous	1	0
ANAPC5	chr12	121746346/CA//C	nonsynonymous	1	0
CLIP1	chr12	122839778/G//A	nonsynonymous	1	0
ABCB9	chr12	123428979/C//T	nonsynonymous	1	0
BRI3BP	chr12	125509769/C//T	synonymous	1	0
ULK1	chr12	132404021/G//A	nonsynonymous	1	0
EP400	chr12	132446105/C//T	nonsynonymous	1	0
EP400	chr12	132547138/A//G	synonymous	1	0
GOLGA3	chr12	133363320/C//T	synonymous	1	0
CHFR	chr12	133434124/C//T	synonymous	1	0
MTMR6	chr13	25839949/C//A	synonymous	1	0
	chr13	31287943/C//A	synonymous	1	0
SMAD9	chr13	37453599/G//A	synonymous	1	0
DGKH	chr13	42789726/A//G	nonsynonymous	1	0
NUFIP1	chr13	45554909/C//T	nonsynonymous	1	0
WDFY2	chr13	52277743/G//A	synonymous	1	0
THSD1	chr13	52952468/G//A	nonsynonymous	1	0
RNF113B	chr13	98829149/G//A	synonymous	1	0

ZIC5	chr13	100623858/C//G	synonymous	1	0
MCF2L	chr13	113656253/G//A	nonsynonymous	1	0
ZNF219	chr14	21558749/C//A	nonsynonymous	1	0
HNRNPC	chr14	21681236/TAGG//T	nonsynonymous	1	0
AP1G2	chr14	24029223/G//A	nonsynonymous	1	0
G2E3	chr14	31066642/C//T	nonsynonymous	1	0
HEATR5A	chr14	31771695/T//C	nonsynonymous	1	0
PLEKHH1	chr14	68041041/T//C	nonsynonymous	1	0
VRTN	chr14	74824137/G//A	synonymous	1	0
PROX2	chr14	75322010/A//G	synonymous	1	0
ZC2HC1C	chr14	75544237/T//C	synonymous	1	0
ZC3H14	chr14	89039358/A//T	synonymous	1	0
CATSPERB	chr14	92105595/C//A	nonsynonymous	1	0
PPP4R4	chr14	94732232/C//G	synonymous	1	0
SLC25A47	chr14	100793707/C//T	synonymous	1	0
ANKRD9	chr14	102973591/G//A	synonymous	1	0
TRMT61A	chr14	103999107/G//A	nonsynonymous	1	0
CEP170B	chr14	105344299/C//T	synonymous	1	0
AHNAK2	chr14	105404331/T//C	synonymous	1	0
TMEM121	chr14	105995640/C//A	nonsynonymous	1	0
GABRB3	chr15	26793246/C//A	synonymous	1	0
RYR3	chr15	34157391/C//T	synonymous	1	0
FAM98B	chr15	38765785/C//T	nonsynonymous	1	0
RASGRP1	chr15	38818504/T//C	nonsynonymous	1	0
EPB42	chr15	43502627/T//C	nonsynonymous	1	0
FBN1	chr15	48797235/G//A	synonymous	1	0
SHC4	chr15	49118149/T//C	synonymous	1	0
HDC	chr15	50534698/C//T	nonsynonymous	1	0
GLDN	chr15	51696834/C//T	synonymous	1	0
LDHAL6B	chr15	59499365/G//T	nonsynonymous	1	0
DIS3L	chr15	66618310/T//A	synonymous	1	0
PML	chr15	74335484/G//A	synonymous	1	0
CSPG4	chr15	76005058/GCC//G	nonsynonymous	1	0
LINGO1	chr15	77906503/C//G	nonsynonymous	1	0
TBC1D2B	chr15	78301339/CT//C	nonsynonymous	1	0
ALPK3	chr15	85402497/C//T	nonsynonymous	1	0
IFT140	chr16	1639613/ACTT//A	nonsynonymous	1	0
SPSB3	chr16	1827832/G//A	nonsynonymous	1	0
SRRM2	chr16	2812809/GTC//G	nonsynonymous	1	0
GRIN2A	chr16	9858553/G//C	nonsynonymous	1	0
CIITA	chr16	11017153/T//TGAA	nonsynonymous	1	0
PARN	chr16	14702131/T//C	synonymous	1	0
SMG1	chr16	18856743/T//C	synonymous	1	0
GPR139	chr16	20043388/C//T	nonsynonymous	1	0
ACSM5	chr16	20442558/G//A	nonsynonymous	1	0
DNAH3	chr16	21049299/G//A	synonymous	1	0
TNRC6A	chr16	24801413/C//T	nonsynonymous	1	0
XPO6	chr16	28113013/C//T	synonymous	1	0
CD19	chr16	28943721/A//C	nonsynonymous	1	0
PRSS53	chr16	31097474/C//T	nonsynonymous	1	0
ZNF720	chr16	31734623/A//G	nonsynonymous	1	0
GNAO1	chr16	56388837/C//T	nonsynonymous	1	0
CDH11	chr16	65016008/C//T	nonsynonymous	1	0
SLC9A5	chr16	67304745/G//A	nonsynonymous	1	0
CDH1	chr16	68863659/C//T	nonsynonymous	1	0
NFAT5	chr16	69726373/G//A	nonsynonymous	1	0
WWP2	chr16	69973852/G//A	synonymous	1	0
MARVELD3	chr16	71668324/C//A	nonsynonymous	1	0
CHST6	chr16	75512953/G//C	synonymous	1	0
KLHL36	chr16	84695504/C//T	nonsynonymous	1	0
KLHL36	chr16	84695655/C//T	synonymous	1	0
ZNF469	chr16	88503047/G//C	nonsynonymous	1	0
RILP	chr17	1553159/G//T	synonymous	1	0
PELP1	chr17	4575752/G//A	nonsynonymous	1	0
NLRP1	chr17	5424277/G//A	nonsynonymous	1	0
ZBTB4	chr17	7365648/C//A	nonsynonymous	1	0
TP53	chr17	7574034/C//T	nonsynonymous	1	0
TP53	chr17	7577518/T//A	nonsynonymous	1	0
PEMT	chr17	17415839/G//A	synonymous	1	0
MTRNR2L1	chr17	22023530/A//C	synonymous	1	0

SDF2	chr17	26976023/T//C	nonsynonymous	1	0
TAOK1	chr17	27857627/C//T	nonsynonymous	1	0
CPD	chr17	28754462/C//T	nonsynonymous	1	0
GAS2L2	chr17	34074134/G//A	nonsynonymous	1	0
PIP4K2B	chr17	36934060/G//A	synonymous	1	0
RPL19	chr17	37359334/C//T	nonsynonymous	1	0
ERBB2	chr17	37864609/T//A	synonymous	1	0
ERBB2	chr17	37881117/C//T	nonsynonymous	1	0
SEPT4	chr17	56604288/C//T	nonsynonymous	1	0
USP32	chr17	58257965/C//T	nonsynonymous	1	0
MRC2	chr17	60769762/GAGA//G	nonsynonymous	1	0
HELZ	chr17	65174911/A//G	nonsynonymous	1	0
USH1G	chr17	72916114/G//A	nonsynonymous	1	0
ZACN	chr17	74077751/G//A	synonymous	1	0
ARHGAP28	chr18	6912088/A//G	nonsynonymous	1	0
LAMA3	chr18	21492704/A//T	nonsynonymous	1	0
	chr18	47801738/A//G	synonymous	1	0
KIAA1468	chr18	59958838/A//T	nonsynonymous	1	0
TNFRSF11A	chr18	60017115/C//T	synonymous	1	0
RPS15	chr19	1440483/A//G	synonymous	1	0
ADAMTSL5	chr19	1510222/T//C	synonymous	1	0
SCAMP4	chr19	1918235/G//A	synonymous	1	0
CACTIN	chr19	3612040/C//T	nonsynonymous	1	0
MCOLN1	chr19	7589905/G//A	synonymous	1	0
CAMSAP3	chr19	7682305/G//A	synonymous	1	0
XAB2	chr19	7691028/G//A	synonymous	1	0
RDH8	chr19	10131976/G//A	synonymous	1	0
ICAM5	chr19	10404957/C//T	synonymous	1	0
DNM2	chr19	10897376/T//C	nonsynonymous	1	0
RGL3	chr19	11513326/T//C	synonymous	1	0
ECSIT	chr19	11623974/G//T	nonsynonymous	1	0
MED26	chr19	16687225/G//A	synonymous	1	0
MAST3	chr19	18246590/A//G	synonymous	1	0
KIAA1683	chr19	18377225/G//C	nonsynonymous	1	0
WDR62	chr19	36590368/G//A	nonsynonymous	1	0
SIPA1L3	chr19	38610519/G//A	synonymous	1	0
RYR1	chr19	38983263/G//T	nonsynonymous	1	0
ACTN4	chr19	39218599/C//T	nonsynonymous	1	0
MRPS12	chr19	39421403/C//T	synonymous	1	0
CIC	chr19	42797897/G//A	nonsynonymous	1	0
CD177	chr19	43866319/C//T	synonymous	1	0
PPP1R13L	chr19	45900161/C//T	synonymous	1	0
EML2	chr19	46112990/CAAG//C	nonsynonymous	1	0
LMTK3	chr19	49001687/G//T	nonsynonymous	1	0
SPHK2	chr19	49130982/G//A	nonsynonymous	1	0
IZUMO1	chr19	49249059/C//T	nonsynonymous	1	0
GFY	chr19	49930172/G//A	nonsynonymous	1	0
ZNF880	chr19	52888185/T//C	nonsynonymous	1	0
DEFB128	chr20	168578/A//G	synonymous	1	0
ANKEF1	chr20	10032460/C//T	nonsynonymous	1	0
ISM1	chr20	13279968/C//T	synonymous	1	0
XKR7	chr20	30584994/C//T	nonsynonymous	1	0
ZNF341	chr20	32354736/G//A	synonymous	1	0
AHCY	chr20	32873320/G//A	nonsynonymous	1	0
RBM12	chr20	34242709/G//A	nonsynonymous	1	0
RPRD1B	chr20	36687923/G//A	nonsynonymous	1	0
JPH2	chr20	42788528/C//T	nonsynonymous	1	0
PCIF1	chr20	44572014/G//A	nonsynonymous	1	0
SLC12A5	chr20	44675053/C//T	nonsynonymous	1	0
ELMO2	chr20	45000037/A//AT	nonsynonymous	1	0
CSTF1	chr20	54974209/G//A	nonsynonymous	1	0
VAPB	chr20	56993394/T//C	synonymous	1	0
EDN3	chr20	57876579/A//G	nonsynonymous	1	0
EDN3	chr20	57899449/G//A	nonsynonymous	1	0
RTEL1	chr20	62321766/T//C	synonymous	1	0
ZNF512B	chr20	62597541/C//T	synonymous	1	0
SCAF4	chr21	33065620/C//T	synonymous	1	0
GART	chr21	34889745/T//C	nonsynonymous	1	0
CRYZL1	chr21	34975783/C//T	nonsynonymous	1	0
TRPM2	chr21	45845653/C//G	synonymous	1	0

CCT8L2	chr22	17073274/C//T	nonsynonymous	1	0
	chr22	19754165/C//T	synonymous	1	0
ARVCF	chr22	19966562/C//T	nonsynonymous	1	0
SLC7A4	chr22	21384159/T//C	synonymous	1	0
CRYBB3	chr22	25598651/A//G	nonsynonymous	1	0
MYO18B	chr22	26423180/C//A	nonsynonymous	1	0
MYO18B	chr22	26423555/G//A	nonsynonymous	1	0
PES1	chr22	30984072/T//C	nonsynonymous	1	0
SELENOM	chr22	31500945/C//T	synonymous	1	0
ANKRD54	chr22	38240040/T//C	nonsynonymous	1	0
SUN2	chr22	39135889/G//A	synonymous	1	0
MCHR1	chr22	41075523/C//T	nonsynonymous	1	0
TSPO	chr22	43555376/G//A	synonymous	1	0
MAPK8IP2	chr22	51045120/G//A	nonsynonymous	1	0
OFD1	chrX	13775826/C//T	nonsynonymous	1	0
POLA1	chrX	24906168/C//T	nonsynonymous	1	0
NUDT10	chrX	51076184/G//A	nonsynonymous	1	0
MAGED1	chrX	51640086/G//A	synonymous	1	0
PFKFB1	chrX	54978512/G//A	synonymous	1	0
SATL1	chrX	84363646/G//A	nonsynonymous	1	0
UBE2A	chrX	118708946/T//C	nonsynonymous	1	0
PRR32	chrX	125953839/G//A	nonsynonymous	1	0
MAGEC1	chrX	140995221/G//T	nonsynonymous	1	0
FUNDC2	chrX	154255300/T//C	synonymous	1	0
VPS13D	chr1	12445319/A//G	nonsynonymous	1	0
SLC44A3	chr1	95360480/T//C	synonymous	1	0
FRRS1	chr1	100174463/T//A	synonymous	1	0
RPS27	chr1	153964631/G//C	synonymous	1	0
SLC30A1	chr1	211749375/T//C	synonymous	1	0
RYR2	chr1	237664160/G//A	nonsynonymous	1	0
IFT172	chr2	27686052/T//C	synonymous	1	0
HEATR5B	chr2	37234186/G//A	synonymous	1	0
STK39	chr2	169020398/G//C	synonymous	1	0
TTN	chr2	179569262/G//A	synonymous	1	0
C2orf80	chr2	209047702/C//T	nonsynonymous	1	0
PID1	chr2	229890713/A//G	nonsynonymous	1	0
PLCD1	chr3	38050093/C//T	nonsynonymous	1	0
KIF15	chr3	44841826/C//T	synonymous	1	0
RAD54L2	chr3	51663425/C//T	synonymous	1	0
PLCXD2	chr3	111564693/T//C	synonymous	1	0
ATR	chr3	142176574/T//A	nonsynonymous	1	0
ABCE1	chr4	146041246/T//G	nonsynonymous	1	0
COL4A3BP	chr5	74677794/C//T	nonsynonymous	1	0
KCNK5	chr6	39196818/C//G	nonsynonymous	1	0
TRDN	chr6	123637635/C//T	nonsynonymous	1	0
KMT2E	chr7	104753697/C//G	nonsynonymous	1	0
LAMB4	chr7	107696158/G//C	nonsynonymous	1	0
DMTN	chr8	21938306/G//T	synonymous	1	0
INTS8	chr8	95862307/T//A	nonsynonymous	1	0
RNF19A	chr8	101270869/T//C	nonsynonymous	1	0
TG	chr8	133899078/A//G	synonymous	1	0
PLEC	chr8	144997124/G//A	nonsynonymous	1	0
SHB	chr9	38068530/G//T	nonsynonymous	1	0
UPF2	chr10	12043762/C//T	nonsynonymous	1	0
SGPL1	chr10	72604402/A//G	synonymous	1	0
ATRNL1	chr10	117704243/G//T	nonsynonymous	1	0
TSG101	chr11	18502110/G//C	nonsynonymous	1	0
ATG2A	chr11	64684591/C//A	nonsynonymous	1	0
ATM	chr11	108205715/A//G	nonsynonymous	1	0
DIP2B	chr12	51089627/A//G	synonymous	1	0
MARS	chr12	57884360/A//G	nonsynonymous	1	0
TEP1	chr14	20852283/G//T	nonsynonymous	1	0
ZFHX2	chr14	24003001/C//G	nonsynonymous	1	0
SNX22	chr15	64448109/G//A	synonymous	1	0
CHRNB4	chr15	78927918/C//T	nonsynonymous	1	0
	chr16	1538427/G//A	synonymous	1	0
PRKCB	chr16	24166020/C//T	nonsynonymous	1	0
CNOT1	chr16	58559155/A//T	nonsynonymous	1	0
AP1G1	chr16	71783814/C//A	nonsynonymous	1	0
TP53	chr17	7578413/C//A	nonsynonymous	1	0

THRA	chr17	38233768/C//A	synonymous	1	0
LRRC37A3	chr17	62856675/C//G	nonsynonymous	1	0
GALNT1	chr18	33270984/G//C	nonsynonymous	1	0
ZNF407	chr18	72775831/G//A	nonsynonymous	1	0
POLRMT	chr19	622638/G//A	nonsynonymous	1	0
SIN3B	chr19	16952697/G//A	nonsynonymous	1	0
OSCAR	chr19	54600341/T//G	synonymous	1	0
ZNF132	chr19	58946103/G//A	synonymous	1	0
ITCH	chr20	33057906/A//G	nonsynonymous	1	0
CXADR	chr21	18931369/C//G	nonsynonymous	1	0
TTC3	chr21	38563669/A//G	nonsynonymous	1	0
TCN2	chr22	31022450/T//A	nonsynonymous	1	0
ZMAT1	chrX	101139265/A//G	synonymous	1	0
RAB9B	chrX	103080648/G//A	nonsynonymous	1	0
SAGE1	chrX	134988659/C//T	nonsynonymous	1	0
HNRNPCL2	chr1	13183853/T//G	nonsynonymous	1	0
PADI2	chr1	17420055/C//T	synonymous	1	0
GLIS1	chr1	53972275/C//T	synonymous	1	0
PRKAA2	chr1	57169717/A//G	nonsynonymous	1	0
PKN2	chr1	89298839/G//A	nonsynonymous	1	0
AXDND1	chr1	179502923/C//G	synonymous	1	0
ZBTB41	chr1	197144168/C//T	nonsynonymous	1	0
ZP4	chr1	238050843/C//T	nonsynonymous	1	0
TRIM58	chr1	248039452/G//A	synonymous	1	0
OR2T12	chr1	248458297/T//C	nonsynonymous	1	0
CLEC4F	chr2	71036884/C//T	nonsynonymous	1	0
CNNM4	chr2	97474314/C//G	nonsynonymous	1	0
PSD4	chr2	113956779/G//A	synonymous	1	0
	chr2	119988596/A//T	synonymous	1	0
ITGB6	chr2	160982983/C//T	nonsynonymous	1	0
STAB1	chr3	52536044/C//A	synonymous	1	0
GABRR3	chr3	97720604/G//T	nonsynonymous	1	0
PARP14	chr3	122447262/C//T	nonsynonymous	1	0
KIT	chr4	55564590/T//G	nonsynonymous	1	0
	chr4	79366897/C//T	synonymous	1	0
PRSS12	chr4	119253011/G//A	synonymous	1	0
CCDC110	chr4	186382206/A//G	synonymous	1	0
MARCH6	chr5	10387177/A//G	nonsynonymous	1	0
RUFY1	chr5	178996325/T//A	nonsynonymous	1	0
	chr6	56480266/C//T	synonymous	1	0
SLC13A1	chr7	122768934/G//A	synonymous	1	0
MGAM	chr7	141727444/C//T	nonsynonymous	1	0
OR9A2	chr7	142724086/A//G	nonsynonymous	1	0
RIMS2	chr8	105261014/C//T	nonsynonymous	1	0
WASHC5	chr8	126040860/A//G	nonsynonymous	1	0
NFX1	chr9	33301295/C//T	synonymous	1	0
CUTC	chr10	101502932/G//A	synonymous	1	0
SLC17A6	chr11	22398954/C//T	nonsynonymous	1	0
PPP1R32	chr11	61252831/G//A	nonsynonymous	1	0
TYR	chr11	88924593/T//G	synonymous	1	0
ROBO4	chr11	124754768/G//A	synonymous	1	0
RYR3	chr15	33939653/A//G	nonsynonymous	1	0
HS3ST4	chr16	25704462/G//T	nonsynonymous	1	0
HEATR3	chr16	50134219/G//A	nonsynonymous	1	0
CTNS	chr17	3563552/A//G	synonymous	1	0
TP53	chr17	7577570/C//T	nonsynonymous	1	0
MYH3	chr17	10539129/A//G	nonsynonymous	1	0
KRT27	chr17	38935798/T//C	nonsynonymous	1	0
ERN1	chr17	62131714/G//GC	nonsynonymous	1	0
CAMSAP3	chr19	7676432/C//T	nonsynonymous	1	0
PGLYRP2	chr19	15586561/C//T	nonsynonymous	1	0
HIF3A	chr19	46823722/G//A	nonsynonymous	1	0
ZNF615	chr19	52496302/C//T	nonsynonymous	1	0
CDH4	chr20	60448812/C//T	synonymous	1	0
ODF3B	chr22	50968939/C//T	synonymous	1	0
NPHP4	chr1	5965747/C//T	nonsynonymous	1	0
RNF207	chr1	6272454/G//T	nonsynonymous	1	0
PER3	chr1	7880583/A//G	nonsynonymous	1	0
KIF1B	chr1	10435439/G//A	synonymous	1	0
EPHA2	chr1	16475231/G//C	nonsynonymous	1	0

RLF	chr1	40703752/G//A	synonymous	1	0
SLC5A9	chr1	48695053/G//T	nonsynonymous	1	0
ZCCHC11	chr1	52991624/T//C	nonsynonymous	1	0
FPGT	chr1	74670869/G//A	nonsynonymous	1	0
RBMXL1	chr1	89448360/C//T	nonsynonymous	1	0
SLC35A3	chr1	100483364/C//T	nonsynonymous	1	0
SV2A	chr1	149878248/G//A	synonymous	1	0
FLG	chr1	152275272/C//T	synonymous	1	0
C1orf68	chr1	152692046/G//T	nonsynonymous	1	0
UBQLN4	chr1	156021535/G//A	synonymous	1	0
SPTA1	chr1	158592931/T//C	nonsynonymous	1	0
PAPPA2	chr1	176564215/C//T	nonsynonymous	1	0
KIF21B	chr1	200957951/G//A	nonsynonymous	1	0
MYBPH	chr1	203139438/G//A	synonymous	1	0
KCNH1	chr1	211192148/G//A	nonsynonymous	1	0
PROX1	chr1	214170759/G//T	nonsynonymous	1	0
DISP1	chr1	223177211/G//T	nonsynonymous	1	0
OBSCN	chr1	228412325/C//A	nonsynonymous	1	0
OBSCN	chr1	228467628/G//C	nonsynonymous	1	0
ACTN2	chr1	236902765/C//T	nonsynonymous	1	0
NLRP3	chr1	247588899/T//G	nonsynonymous	1	0
EFR3B	chr2	25360213/T//C	nonsynonymous	1	0
LRPPRC	chr2	44161330/T//A	nonsynonymous	1	0
DYSF	chr2	71891491/T//G	synonymous	1	0
LRRTM4	chr2	77746041/G//T	nonsynonymous	1	0
IMMT	chr2	86398377/A//T	synonymous	1	0
RFX8	chr2	102029410/C//T	nonsynonymous	1	0
RABL2A	chr2	114398961/T//G	nonsynonymous	1	0
MYO7B	chr2	128387277/AGGGGCTGCTGGCC	nonsynonymous	1	0
EPC2	chr2	149520259/G//A	nonsynonymous	1	0
RIF1	chr2	152321302/C//T	synonymous	1	0
KCNJ3	chr2	155555917/G//A	synonymous	1	0
LY75	chr2	160661546/T//C	synonymous	1	0
GCG	chr2	163002150/G//C	nonsynonymous	1	0
IFIH1	chr2	163124655/C//T	nonsynonymous	1	0
TLK1	chr2	171850380/G//A	synonymous	1	0
TTN	chr2	179483024/G//T	nonsynonymous	1	0
TTN	chr2	179489185/T//C	synonymous	1	0
TTN	chr2	179547952/G//C	nonsynonymous	1	0
ALS2	chr2	202574690/G//A	synonymous	1	0
PLEKHM3	chr2	208866116/GTTTC//G	nonsynonymous	1	0
FN1	chr2	216242892/A//T	synonymous	1	0
PLCD4	chr2	219496858/G//T	nonsynonymous	1	0
DOCK10	chr2	225658118/C//A	nonsynonymous	1	0
HDLBP	chr2	242169368/G//A	synonymous	1	0
CHL1	chr3	367721/TGAAGCTAAAGGAAATC(	nonsynonymous	1	0
VILL	chr3	38035917/G//A	nonsynonymous	1	0
GPR62	chr3	51989671/G//T	nonsynonymous	1	0
ROBO2	chr3	77638071/C//A	synonymous	1	0
ROBO1	chr3	78701027/C//A	nonsynonymous	1	0
C3orf38	chr3	88199071/G//T	synonymous	1	0
B4GALT4	chr3	118935087/C//T	nonsynonymous	1	0
SNX4	chr3	125216764/G//A	nonsynonymous	1	0
CLSTN2	chr3	140282841/G//A	nonsynonymous	1	0
CHST2	chr3	142840531/C//T	synonymous	1	0
SI	chr3	164712164/A//C	synonymous	1	0
SI	chr3	164750461/C//T	nonsynonymous	1	0
PHC3	chr3	169814896/A//G	synonymous	1	0
PIK3CA	chr3	178937391/C//G	nonsynonymous	1	0
ATP11B	chr3	182603777/C//T	synonymous	1	0
STIM2	chr4	27019406/G//A	synonymous	1	0
SLC4A4	chr4	72433713/A//T	synonymous	1	0
ANKRD17	chr4	74019594/T//C	synonymous	1	0
PPEF2	chr4	76794285/G//A	nonsynonymous	1	0
RASGEF1B	chr4	82366714/G//A	synonymous	1	0
CCSER1	chr4	92520156/ACCACAATTTACACAG	nonsynonymous	1	0
CCSER1	chr4	92520174/C//T	nonsynonymous	1	0
CCSER1	chr4	92520175/TGAGCTGCAAA//T	nonsynonymous	1	0
UBE2D3	chr4	103747635/G//A	synonymous	1	0
DKK2	chr4	107956805/G//T	synonymous	1	0

ETNPPL	chr4	109670581/T//A	nonsynonymous	1	0
ZGRF1	chr4	113469451/C//T	nonsynonymous	1	0
TDO2	chr4	156835591/G//A	synonymous	1	0
TKTL2	chr4	164393564/G//T	nonsynonymous	1	0
DROSHA	chr5	31466282/T//C	synonymous	1	0
ADAMTS12	chr5	33881391/C//T	nonsynonymous	1	0
MCCC2	chr5	70922531/ACATCATTGTACGCAA	nonsynonymous	1	0
MCCC2	chr5	70922553/T//A	synonymous	1	0
ANKRD34B	chr5	79855537/A//G	nonsynonymous	1	0
SLC27A6	chr5	128320968/C//A	nonsynonymous	1	0
NEUROG1	chr5	134871299/C//T	nonsynonymous	1	0
TRPC7	chr5	135587379/G//A	nonsynonymous	1	0
PCDHA1	chr5	140167538/G//A	nonsynonymous	1	0
PCDHA5	chr5	140201441/C//T	synonymous	1	0
PCDHA5	chr5	140202764/G//A	synonymous	1	0
PCDHB13	chr5	140594759/G//T	nonsynonymous	1	0
PCDHGA3	chr5	140725379/G//A	synonymous	1	0
PCDHGA6	chr5	140753744/C//T	nonsynonymous	1	0
PCDHGB7	chr5	140798986/C//T	synonymous	1	0
PCDHGC4	chr5	140866165/C//T	synonymous	1	0
CANX	chr5	179150724/A//AT	nonsynonymous	1	0
MAPK9	chr5	179669720/C//A	nonsynonymous	1	0
IRF4	chr6	395850/C//T	nonsynonymous	1	0
FARS2	chr6	5369019/C//T	synonymous	1	0
NHLRC1	chr6	18122670/G//A	synonymous	1	0
OR2W1	chr6	29012626/T//G	synonymous	1	0
ZBTB12	chr6	31867627/C//T	synonymous	1	0
TMEM217	chr6	37186731/C//T	nonsynonymous	1	0
ZFAND3	chr6	38050189/C//A	nonsynonymous	1	0
PGC	chr6	41710065/C//T	nonsynonymous	1	0
PHF3	chr6	64394926/A//G	nonsynonymous	1	0
MTO1	chr6	74183155/T//C	synonymous	1	0
ORC3	chr6	88375530/A//C	nonsynonymous	1	0
MDN1	chr6	90398499/G//T	synonymous	1	0
RTN4IP1	chr6	107067184/T//C	synonymous	1	0
ADGRG6	chr6	142688724/G//A	nonsynonymous	1	0
ADGB	chr6	147067127/C//T	nonsynonymous	1	0
AGMO	chr7	15405821/C//T	synonymous	1	0
ABCB5	chr7	20738104/G//T	synonymous	1	0
GPNMB	chr7	23293803/C//T	nonsynonymous	1	0
NME8	chr7	37907316/G//A	nonsynonymous	1	0
INHBA	chr7	41739831/G//A	nonsynonymous	1	0
ZMIZ2	chr7	44804069/A//T	nonsynonymous	1	0
PPIA	chr7	44836318/T//C	synonymous	1	0
ABHD11	chr7	73151688/T//C	nonsynonymous	1	0
PCLO	chr7	82579199/T//C	nonsynonymous	1	0
COL1A2	chr7	94055176/T//G	synonymous	1	0
MCM7	chr7	99694962/G//C	nonsynonymous	1	0
ACHE	chr7	100491410/C//A	nonsynonymous	1	0
BCAP29	chr7	107258995/G//C	synonymous	1	0
LAMB4	chr7	107689976/T//A	nonsynonymous	1	0
ANKRD7	chr7	117874934/T//A	synonymous	1	0
KCND2	chr7	119915254/G//T	nonsynonymous	1	0
KCNH2	chr7	150649608/G//A	nonsynonymous	1	0
LZTS1	chr8	20112394/G//A	nonsynonymous	1	0
NRG1	chr8	32621347/G//T	synonymous	1	0
ANK1	chr8	41575636/G//A	synonymous	1	0
RP1	chr8	55537987/A//G	synonymous	1	0
CSPP1	chr8	68105701/T//C	synonymous	1	0
PREX2	chr8	68965402/G//C	nonsynonymous	1	0
CALB1	chr8	91072901/C//G	nonsynonymous	1	0
VPS13B	chr8	100147903/G//A	nonsynonymous	1	0
KLHL38	chr8	124663910/C//G	synonymous	1	0
RNF139	chr8	125498278/C//G	nonsynonymous	1	0
TG	chr8	133980140/G//T	nonsynonymous	1	0
ADGRB1	chr8	143545648/C//T	nonsynonymous	1	0
GLI4	chr8	144351647/C//T	synonymous	1	0
TOP1MT	chr8	144399908/G//A	nonsynonymous	1	0
MROH6	chr8	144651916/C//T	nonsynonymous	1	0
ACER2	chr9	19423888/C//T	nonsynonymous	1	0

NUDT2	chr9	34343282/G//A	synonymous	1	0
TRPM3	chr9	73478010/C//G	synonymous	1	0
GDA	chr9	74856086/C//A	nonsynonymous	1	0
ABCA1	chr9	107549250/G//C	nonsynonymous	1	0
OLFM1	chr9	138011714/A//G	nonsynonymous	1	0
PNPLA7	chr9	140356418/C//T	nonsynonymous	1	0
CUBN	chr10	16979675/C//T	nonsynonymous	1	0
ARHGAP21	chr10	24874952/C//T	synonymous	1	0
GAD2	chr10	26506787/C//T	synonymous	1	0
TET1	chr10	70406266/G//A	synonymous	1	0
SGPL1	chr10	72633183/G//A	nonsynonymous	1	0
GLUD1	chr10	88822577/C//A	nonsynonymous	1	0
MGEA5	chr10	103553759/T//C	synonymous	1	0
SFXN4	chr10	120907319/C//A	synonymous	1	0
PTPRE	chr10	129864349/A//G	nonsynonymous	1	0
STK32C	chr10	134041533/G//A	nonsynonymous	1	0
OR52H1	chr11	5566493/AGGCACACCAGCTGT//	nonsynonymous	1	0
OR6A2	chr11	6816354/C//A	nonsynonymous	1	0
MRGPRX1	chr11	18955962/C//T	nonsynonymous	1	0
OR9G4	chr11	56511209/A//G	nonsynonymous	1	0
OR1S2	chr11	57971225/C//T	synonymous	1	0
NUMA1	chr11	71726928/G//A	nonsynonymous	1	0
NOX4	chr11	89073252/T//G	nonsynonymous	1	0
TRIM49C	chr11	89774527/A//T	nonsynonymous	1	0
DYNC2H1	chr11	103062891/C//T	nonsynonymous	1	0
GRIK4	chr11	120530973/C//T	synonymous	1	0
ARHGAP32	chr11	128932218/G//T	nonsynonymous	1	0
B3GAT1	chr11	134253812/G//A	nonsynonymous	1	0
TARBP2	chr12	53899960/C//A	synonymous	1	0
OR6C6	chr12	55689001/T//C	nonsynonymous	1	0
PPFIA2	chr12	81733050/G//T	nonsynonymous	1	0
CCER1	chr12	91348462/C//T	nonsynonymous	1	0
TMEM119	chr12	108985818/G//A	synonymous	1	0
TRAFD1	chr12	112585936/G//C	nonsynonymous	1	0
MED13L	chr12	116421060/C//A	nonsynonymous	1	0
DNAH10	chr12	124408866/G//A	nonsynonymous	1	0
POSTN	chr13	38158129/G//T	nonsynonymous	1	0
GPC5	chr13	93518681/G//A	nonsynonymous	1	0
TMTC4	chr13	101287132/A//T	nonsynonymous	1	0
ADCY4	chr14	24787685/G//T	synonymous	1	0
TRIM9	chr14	51467448/C//T	nonsynonymous	1	0
TRIM9	chr14	51467547/C//A	nonsynonymous	1	0
HIF1A	chr14	62207766/T//G	synonymous	1	0
SLC8A3	chr14	70515754/C//A	nonsynonymous	1	0
SERPINA10	chr14	94750292/G//A	synonymous	1	0
SETD3	chr14	99927594/C//T	nonsynonymous	1	0
AMN	chr14	103390301/C//T	synonymous	1	0
	chr14	104151344/C//T	synonymous	1	0
ATP10A	chr15	25966967/G//A	synonymous	1	0
MAPKBP1	chr15	42067505/G//A	nonsynonymous	1	0
SPTBN5	chr15	42169084/G//A	synonymous	1	0
LARP6	chr15	71124800/G//A	nonsynonymous	1	0
THSD4	chr15	72069673/G//A	nonsynonymous	1	0
MYO9A	chr15	72190523/T//C	nonsynonymous	1	0
ISLR2	chr15	74427076/G//C	nonsynonymous	1	0
HMG20A	chr15	77750782/G//A	synonymous	1	0
EFL1	chr15	82422801/G//A	synonymous	1	0
ADAMTSL3	chr15	84611680/C//T	nonsynonymous	1	0
ZNF710	chr15	90610813/C//T	synonymous	1	0
HBM	chr16	216040/A//G	nonsynonymous	1	0
CEMP1	chr16	2580972/G//T	nonsynonymous	1	0
SLX4	chr16	3658764/T//G	nonsynonymous	1	0
PPL	chr16	4943378/A//T	synonymous	1	0
EMP2	chr16	10631936/G//A	synonymous	1	0
EEF2K	chr16	22295375/A//C	synonymous	1	0
ERN2	chr16	23724559/A//T	synonymous	1	0
TBC1D10B	chr16	30371102/G//A	synonymous	1	0
SRCAP	chr16	30727660/A//T	nonsynonymous	1	0
SRCAP	chr16	30731580/G//A	nonsynonymous	1	0
ZNF668	chr16	31075268/G//A	synonymous	1	0

ZNF423	chr16	49670198/A//T	synonymous	1	0
CETP	chr16	57007295/C//T	nonsynonymous	1	0
PARD6A	chr16	67696275/G//C	nonsynonymous	1	0
VAC14	chr16	70820167/C//T	nonsynonymous	1	0
FOXL1	chr16	86612266/C//T	synonymous	1	0
CDH15	chr16	89256813/C//T	nonsynonymous	1	0
USP6	chr17	5072170/C//T	nonsynonymous	1	0
KDM6B	chr17	7750200/C//A	nonsynonymous	1	0
ALOX12B	chr17	7976477/G//A	nonsynonymous	1	0
MYH3	chr17	10538794/G//C	synonymous	1	0
NBR1	chr17	41338330/G//A	nonsynonymous	1	0
MAPT	chr17	44068843/G//A	synonymous	1	0
TBX21	chr17	45822603/C//T	synonymous	1	0
TEX14	chr17	56663371/C//A	nonsynonymous	1	0
INTS2	chr17	60002444/C//T	nonsynonymous	1	0
ARSG	chr17	66391299/G//A	nonsynonymous	1	0
	chr17	72667707/G//A	synonymous	1	0
NUP85	chr17	73214287/TCTC//T	nonsynonymous	1	0
CD7	chr17	80274657/C//G	nonsynonymous	1	0
LPIN2	chr18	2921573/C//T	synonymous	1	0
EPB41L3	chr18	5406959/T//A	nonsynonymous	1	0
LAMA1	chr18	7046328/C//T	nonsynonymous	1	0
GREB1L	chr18	19032092/A//G	nonsynonymous	1	0
MEP1B	chr18	29790683/C//A	synonymous	1	0
ZNF24	chr18	32920350/C//T	nonsynonymous	1	0
MISP	chr19	758033/G//A	nonsynonymous	1	0
EFNA2	chr19	1299902/C//T	synonymous	1	0
ATP8B3	chr19	1784913/T//C	nonsynonymous	1	0
PLIN4	chr19	4510585/T//C	synonymous	1	0
RGL3	chr19	11517233/G//A	synonymous	1	0
ZNF878	chr19	12155697/A//G	synonymous	1	0
CEBPA	chr19	33792754/GGGCGGCGGC//G	nonsynonymous	1	0
	chr19	37487699/G//T	synonymous	1	0
ARHGAP35	chr19	47440570/G//A	nonsynonymous	1	0
CARD8	chr19	48744220/G//A	nonsynonymous	1	0
PLEKHA4	chr19	49363634/C//T	nonsynonymous	1	0
VRK3	chr19	50498537/G//A	synonymous	1	0
MYH14	chr19	50766645/A//C	synonymous	1	0
ZNF845	chr19	53854443/C//T	nonsynonymous	1	0
A1BG	chr19	58861803/G//A	synonymous	1	0
MZF1	chr19	59073457/G//A	synonymous	1	0
SIGLEC1	chr20	3678673/C//T	nonsynonymous	1	0
NAA20	chr20	20013266/C//T	synonymous	1	0
FOXA2	chr20	22563310/C//T	synonymous	1	0
ZNF335	chr20	44580961/G//A	nonsynonymous	1	0
EEF1A2	chr20	62126340/G//A	nonsynonymous	1	0
UCKL1	chr20	62577847/T//C	nonsynonymous	1	0
TPTE	chr21	10907042/T//G	nonsynonymous	1	0
PCBP3	chr21	47361674/C//T	synonymous	1	0
CLTCL1	chr22	19188972/G//A	synonymous	1	0
MORC2	chr22	31330793/G//A	nonsynonymous	1	0
INPP5J	chr22	31522413/C//A	synonymous	1	0
BPIFC	chr22	32853349/G//A	nonsynonymous	1	0
MYH9	chr22	36681980/C//T	nonsynonymous	1	0
ENTHD1	chr22	40161360/C//A	nonsynonymous	1	0
FAM109B	chr22	42474090/T//TGAGTC	synonymous	1	0
FBLN1	chr22	45937188/C//T	synonymous	1	0
P2RY8	chrX	1584491/G//A	nonsynonymous	1	0
CLCN4	chrX	10176264/C//T	synonymous	1	0
BRWD3	chrX	79965017/A//C	nonsynonymous	1	0
PCDH19	chrX	99551400/G//A	nonsynonymous	1	0
GPRASP1	chrX	101912273/C//T	synonymous	1	0
SLITRK4	chrX	142718916/C//A	synonymous	1	0
ZSWIM5	chr1	45484674/C//A	nonsynonymous	1	0
DEPDC1	chr1	68948165/A//G	synonymous	1	0
MAN1A2	chr1	117957438/C//T	synonymous	1	0
FLG2	chr1	152328187/C//G	nonsynonymous	1	0
RAB1A	chr2	65357067/C//T	synonymous	1	0
NEB	chr2	152432785/G//A	synonymous	1	0
TTN	chr2	179659227/C//T	nonsynonymous	1	0

FBLN2	chr3	13671395/G//A	nonsynonymous	1	0
PTX3	chr3	157160492/G//A	synonymous	1	0
PIK3CA	chr3	178916936/G//A	nonsynonymous	1	0
CFAP97	chr4	186112021/A//T	synonymous	1	0
OSMR	chr5	38925333/T//C	nonsynonymous	1	0
APC	chr5	112175245/TC//T	nonsynonymous	1	0
PCDHA11	chr5	140248682/G//A	synonymous	1	0
ATP10B	chr5	160047493/G//C	nonsynonymous	1	0
EXOC2	chr6	598940/C//A	nonsynonymous	1	0
BEND3	chr6	107391775/G//A	nonsynonymous	1	0
MUC17	chr7	100681278/A//C	nonsynonymous	1	0
DOCK4	chr7	111484893/T//C	nonsynonymous	1	0
MGAM	chr7	141765153/G//A	synonymous	1	0
HOOK3	chr8	42823319/A//T	nonsynonymous	1	0
C8orf34	chr8	69445403/AATTTCT//A	synonymous	1	0
LOC286238	chr9	91262346/G//T	synonymous	1	0
ERCC6	chr10	50669503/G//A	nonsynonymous	1	0
ACTA2	chr10	90697848/C//T	synonymous	1	0
AP5B1	chr11	65545684/G//T	nonsynonymous	1	0
BCL9L	chr11	118779015/C//CT	nonsynonymous	1	0
PLCZ1	chr12	18858172/C//A	nonsynonymous	1	0
WSCD2	chr12	108620832/G//A	synonymous	1	0
THSD1	chr13	52952647/C//T	synonymous	1	0
RYR3	chr15	33955932/G//T	nonsynonymous	1	0
DUOX2	chr15	45403675/C//T	nonsynonymous	1	0
CACNA1G	chr17	48650041/C//T	synonymous	1	0
TEX14	chr17	56693619/C//T	synonymous	1	0
SLC25A41	chr19	6426476/G//A	nonsynonymous	1	0
ADGRE5	chr19	14492302/C//G	synonymous	1	0
CLPTM1	chr19	45489832/A//G	synonymous	1	0
DACT3	chr19	47152167/G//A	nonsynonymous	1	0
PCSK2	chr20	17462427/C//T	synonymous	1	0
ASPHD2	chr22	26830109/G//A	synonymous	1	0
ACO2	chr22	41895722/T//C	synonymous	1	0
MAPK8IP2	chr22	51042652/C//G	nonsynonymous	1	0
MAOA	chrX	43595532/G//A	synonymous	1	0
MAGEA6	chrX	151869877/G//T	synonymous	1	0
ARHGEF10L	chr1	18023593/G//A	synonymous	0	1
IPO13	chr1	44422877/T//G	nonsynonymous	0	1
SGIP1	chr1	67199451/C//G	nonsynonymous	0	1
PKP1	chr1	201282605/T//C	synonymous	0	1
SDCCAG8	chr1	243579026/C//G	nonsynonymous	0	1
BIRC6	chr2	32718730/G//A	nonsynonymous	0	1
EFEMP1	chr2	56097856/C//A	nonsynonymous	0	1
AFF3	chr2	100217921/C//T	synonymous	0	1
ZNF804A	chr2	185802473/A//C	nonsynonymous	0	1
PARD3B	chr2	206166358/A//C	nonsynonymous	0	1
DCBLD2	chr3	98519481/G//C	nonsynonymous	0	1
DCBLD2	chr3	98519491/T//C	nonsynonymous	0	1
PDZD2	chr5	32058185/G//A	nonsynonymous	0	1
NUP155	chr5	37305262/T//TC	nonsynonymous	0	1
	chr5	141237011/G//A	synonymous	0	1
MAGI2	chr7	77885726/A//C	synonymous	0	1
CCDC71L	chr7	106301212/C//A	nonsynonymous	0	1
SAPCD2	chr9	139959012/C//T	synonymous	0	1
CDC123	chr10	12280490/T//C	synonymous	0	1
LALBA	chr12	48962994/A//C	nonsynonymous	0	1
NUP37	chr12	102505932/C//A	nonsynonymous	0	1
COX6A1	chr12	120875976/C//T	nonsynonymous	0	1
HPD	chr12	122286974/C//T	nonsynonymous	0	1
GGACT	chr13	101184755/G//A	nonsynonymous	0	1
CLMN	chr14	95688083/C//T	nonsynonymous	0	1
ATP2C2	chr16	84456270/C//CG	nonsynonymous	0	1
FOXF1	chr16	86544988/G//T	synonymous	0	1
BRIP1	chr17	59885954/C//G	synonymous	0	1
ZNF714	chr19	21300194/C//T	nonsynonymous	0	1
ZNF765	chr19	53911861/A//T	synonymous	0	1
UCKL1	chr20	62571789/A//G	nonsynonymous	0	1
ZNF182	chrX	47836178/T//C	synonymous	0	1
ZMYM3	chrX	70462939/G//A	synonymous	0	1

CFAP74	chr1	1890563/C//A	nonsynonymous	0	1
GPSM2	chr1	109440141/G//A	synonymous	0	1
AHCYL1	chr1	110560623/G//A	nonsynonymous	0	1
MCM6	chr2	136626367/C//A	synonymous	0	1
EPHA3	chr3	89457240/A//G	nonsynonymous	0	1
FREM3	chr4	144620845/G//A	synonymous	0	1
TLL1	chr4	166963171/T//G	synonymous	0	1
C7orf43	chr7	99754476/C//T	nonsynonymous	0	1
MUC12	chr7	100635850/C//A	nonsynonymous	0	1
ASZ1	chr7	117067500/C//T	synonymous	0	1
JPH1	chr8	75227735/C//T	nonsynonymous	0	1
MYC	chr8	128750487/G//A	synonymous	0	1
RLN1	chr9	5339789/TCAGGACTGCGGCTGC	synonymous	0	1
NR4A3	chr9	102590882/G//A	synonymous	0	1
NFKB2	chr10	104156535/C//T	synonymous	0	1
CFAP46	chr10	134705879/C//T	nonsynonymous	0	1
OR4C16	chr11	55339962/G//A	nonsynonymous	0	1
LAYN	chr11	111420336/C//T	synonymous	0	1
C14orf28	chr14	45369645/A//T	nonsynonymous	0	1
IFT43	chr14	76488697/C//T	nonsynonymous	0	1
AHNAK2	chr14	105413407/C//T	nonsynonymous	0	1
PLCB2	chr15	40591424/G//A	synonymous	0	1
TMEM62	chr15	43427838/G//C	nonsynonymous	0	1
VPS53	chr17	531460/T//C	synonymous	0	1
ELOA2	chr18	44560391/G//A	synonymous	0	1
ZNF737	chr19	20728011/C//A	nonsynonymous	0	1
ZNF737	chr19	20728013/T//C	synonymous	0	1
ZNF208	chr19	22154511/C//T	nonsynonymous	0	1
CEACAM16	chr19	45206952/T//C	nonsynonymous	0	1
GMEB2	chr20	62236139/G//A	synonymous	0	1
ZFP92	chrX	152686999/G//T	synonymous	0	1
CPSF3L	chr1	1249684/A//G	nonsynonymous	0	1
SEMA4A	chr1	156127900/G//A	nonsynonymous	0	1
ZNF678	chr1	227843007/A//T	nonsynonymous	0	1
HADHB	chr2	26492866/G//A	nonsynonymous	0	1
PASK	chr2	242066002/G//A	synonymous	0	1
PDCD10	chr3	167402146/G//C	nonsynonymous	0	1
SMCO1	chr3	196234787/C//T	nonsynonymous	0	1
PCDHGA5	chr5	140745777/G//A	nonsynonymous	0	1
HIST1H2AG	chr6	27101057/C//G	nonsynonymous	0	1
ZBTB9	chr6	33423989/T//C	nonsynonymous	0	1
CRIP3	chr6	43273827/G//A	synonymous	0	1
PPP1R9A	chr7	94540745/G//A	synonymous	0	1
TECPR1	chr7	97860542/A//G	synonymous	0	1
MUC17	chr7	100676295/C//T	nonsynonymous	0	1
EPPK1	chr8	144940620/C//T	nonsynonymous	0	1
ADARB2	chr10	1405393/C//T	nonsynonymous	0	1
JMJD1C	chr10	64973827/C//T	synonymous	0	1
HELLS	chr10	96336485/C//T	nonsynonymous	0	1
CFAP58	chr10	106152121/A//T	nonsynonymous	0	1
FZD4	chr11	86662230/CCT//C	nonsynonymous	0	1
MIDN	chr19	1257449/C//T	synonymous	0	1
PPAN	chr19	10217177/T//C	nonsynonymous	0	1
RFX1	chr19	14080862/G//A	synonymous	0	1
CEP250	chr20	34087961/G//A	synonymous	0	1
KRTAP19-1	chr21	31852432/C//A	nonsynonymous	0	1
IFT27	chr22	37163414/C//T	synonymous	0	1
GBP1	chr1	89525071/G//A	synonymous	0	1
AGL	chr1	100346636/G//A	nonsynonymous	0	1
SLC16A4	chr1	110918178/A//G	synonymous	0	1
GON4L	chr1	155735502/T//C	synonymous	0	1
NFASC	chr1	204943900/G//A	nonsynonymous	0	1
TTN	chr2	179469438/A//G	synonymous	0	1
RAPH1	chr2	204305016/G//A	nonsynonymous	0	1
	chr3	138426143/A//G	synonymous	0	1
PRR23B	chr3	138739416/G//A	nonsynonymous	0	1
HPF1	chr4	170658831/C//T	nonsynonymous	0	1
PRR16	chr5	120021946/C//G	nonsynonymous	0	1
HK3	chr5	176308099/C//T	nonsynonymous	0	1
LRFN2	chr6	40399758/G//A	synonymous	0	1

PEX6	chr6	42942645/G//A	synonymous	0	1
NKX3-1	chr8	23538803/G//A	synonymous	0	1
ZMAT4	chr8	40532405/G//A	nonsynonymous	0	1
LYNX1	chr8	143845995/C//T	synonymous	0	1
SNAPC4	chr9	139282287/C//T	nonsynonymous	0	1
CACNA1B	chr9	141015185/G//A	nonsynonymous	0	1
PFKFB3	chr10	6265954/C//T	nonsynonymous	0	1
AGAP5	chr10	75457445/C//T	synonymous	0	1
SNCG	chr10	88718492/A//G	nonsynonymous	0	1
PNLIPRP1	chr10	118355777/G//A	nonsynonymous	0	1
TMEM258	chr11	61560074/C//T	synonymous	0	1
USP28	chr11	113683112/A//G	nonsynonymous	0	1
MCAM	chr11	119183010/C//T	synonymous	0	1
LRRK2	chr12	40715848/C//T	nonsynonymous	0	1
UHRF1BP1L	chr12	100441959/G//A	nonsynonymous	0	1
MYH7	chr14	23896796/G//A	nonsynonymous	0	1
PCK2	chr14	24566162/C//T	nonsynonymous	0	1
FLRT2	chr14	86088784/A//C	nonsynonymous	0	1
RYR3	chr15	33876607/C//T	nonsynonymous	0	1
JMJD7-PLA2G4B	chr15	42139597/C//T	synonymous	0	1
SRCAP	chr16	30751037/C//T	nonsynonymous	0	1
PKD1L2	chr16	81201640/G//A	nonsynonymous	0	1
ENOSF1	chr18	691257/G//A	nonsynonymous	0	1
POTEC	chr18	14542648/C//A	nonsynonymous	0	1
SETBP1	chr18	42532776/G//T	nonsynonymous	0	1
WDR7	chr18	54385199/G//A	nonsynonymous	0	1
ATCAY	chr19	3907818/G//A	nonsynonymous	0	1
EEF2	chr19	3983205/G//A	synonymous	0	1
DOCK6	chr19	11338085/G//A	synonymous	0	1
FAM90A27P	chr19	53787365/C//T	synonymous	0	1
DSCAM	chr21	41465775/G//A	synonymous	0	1
AJAP1	chr1	4832521/G//A	nonsynonymous	0	1
FMO2	chr1	171154949/A//G	nonsynonymous	0	1
ABL2	chr1	179078030/A//G	nonsynonymous	0	1
THSD7B	chr2	138375963/G//A	synonymous	0	1
SLC4A10	chr2	162661051/A//G	nonsynonymous	0	1
CCR1	chr3	46245808/G//A	synonymous	0	1
AMIGO3	chr3	49755551/C//T	nonsynonymous	0	1
ALDH1L1	chr3	125877280/G//A	synonymous	0	1
DNAJC13	chr3	132235626/C//T	nonsynonymous	0	1
	chr5	58571249/A//G	synonymous	0	1
SAP25	chr7	100170337/C//T	synonymous	0	1
GLIS3	chr9	4118370/T//C	nonsynonymous	0	1
FREM1	chr9	14784563/G//A	nonsynonymous	0	1
EFEMP2	chr11	65635424/C//T	nonsynonymous	0	1
MON2	chr12	62877952/T//C	synonymous	0	1
RAP1B	chr12	69050127/T//A	nonsynonymous	0	1
ADCK1	chr14	78285410/A//G	nonsynonymous	0	1
FBN1	chr15	48936846/G//A	nonsynonymous	0	1
CHRNB4	chr15	78917532/C//T	synonymous	0	1
ZNF710	chr15	90610910/G//A	nonsynonymous	0	1
GALNS	chr16	88907438/C//T	synonymous	0	1
MEX3C	chr18	48703397/C//T	nonsynonymous	0	1
MEGF8	chr19	42860040/C//T	synonymous	0	1
ERCC2	chr19	45867073/A//G	nonsynonymous	0	1
CHD6	chr20	40053922/GCA//G	nonsynonymous	0	1
NFATC2	chr20	50133447/C//T	nonsynonymous	0	1
IL17RA	chr22	17589328/G//A	nonsynonymous	0	1
SYNGR1	chr22	39770350/C//T	synonymous	0	1
CHI3L2	chr1	111783981/G//A	synonymous	0	1
SLC30A3	chr2	27480105/C//T	nonsynonymous	0	1
AGTR1	chr3	148459397/C//T	nonsynonymous	0	1
PCDHB5	chr5	140515309/C//T	nonsynonymous	0	1
PRRC2A	chr6	31595623/T//C	nonsynonymous	0	1
GLI3	chr7	42004666/C//T	synonymous	0	1
BAIAP2L1	chr7	97937129/C//G	nonsynonymous	0	1
DOCK5	chr8	25198452/T//C	nonsynonymous	0	1
SAPCD2	chr9	139959999/C//T	nonsynonymous	0	1
CUBN	chr10	16955909/G//A	synonymous	0	1
SFTPA1	chr10	81371729/C//G	nonsynonymous	0	1

TRHDE	chr12	72666890/C//T	nonsynonymous	0	1
TMEM255B	chr13	114507917/C//T	synonymous	0	1
SALL2	chr14	22004995/C//T	nonsynonymous	0	1
AHNAK2	chr14	105418119/G//C	nonsynonymous	0	1
AHNAK2	chr14	105418136/C//T	nonsynonymous	0	1
ITGAM	chr16	31340553/G//T	nonsynonymous	0	1
SMAD4	chr18	48593520/A//T	nonsynonymous	0	1
BIRC7	chr20	61870941/G//A	nonsynonymous	0	1
TRIOBP	chr22	38155256/G//A	synonymous	0	1
TTLL10	chr1	1115453/A//G	nonsynonymous	0	1
KDM3B	chr5	137767213/T//C	nonsynonymous	0	1
ZBED5	chr11	10874389/A//G	synonymous	0	1
ENPP7	chr17	77704901/C//T	synonymous	0	1
C1orf127	chr1	11007984/T//C	synonymous	0	1
LCE2A	chr1	152671571/G//T	nonsynonymous	0	1
BCL11A	chr2	60780416/G//T	synonymous	0	1
PEX13	chr2	61275603/T//C	synonymous	0	1
MAP4K4	chr2	102450883/T//C	nonsynonymous	0	1
LNP1	chr3	100170628/A//G	synonymous	0	1
LNP1	chr3	100170634/T//C	synonymous	0	1
PCDH18	chr4	138450913/G//A	nonsynonymous	0	1
FBXW7	chr4	153245429/C//T	nonsynonymous	0	1
ELOVL7	chr5	60050634/G//A	synonymous	0	1
PCDHB1	chr5	140432415/G//A	nonsynonymous	0	1
SPDL1	chr5	169028581/A//G	nonsynonymous	0	1
NFKBIE	chr6	44233209/C//T	nonsynonymous	0	1
COL21A1	chr6	56006630/G//A	nonsynonymous	0	1
ZDHHC14	chr6	157963680/C//T	synonymous	0	1
POU6F2	chr7	39472801/C//T	synonymous	0	1
ABCA13	chr7	48391989/C//T	synonymous	0	1
APBA1	chr9	72131426/C//T	nonsynonymous	0	1
PTEN	chr10	89717739/T//TA	nonsynonymous	0	1
PHC1	chr12	9086467/C//T	nonsynonymous	0	1
ITPR2	chr12	26733093/A//G	synonymous	0	1
RTN1	chr14	60063453/G//A	synonymous	0	1
CLMN	chr14	95660927/A//G	synonymous	0	1
DNAH2	chr17	7679343/C//T	nonsynonymous	0	1
PSG2	chr19	43579605/G//T	nonsynonymous	0	1
CPT1C	chr19	50208269/C//T	synonymous	0	1
ARSA	chr22	51064645/T//C	nonsynonymous	0	1
TSPYL2	chrX	53111715/C//T	nonsynonymous	0	1
ZBTB38	chr3	141162413/A//G	nonsynonymous	0	1
PCDHGB4	chr5	140769366/C//T	nonsynonymous	0	1
NOTCH4	chr6	32185874/G//T	nonsynonymous	0	1
TBX20	chr7	35242128/G//A	nonsynonymous	0	1
TBC1D31	chr8	124164138/G//T	nonsynonymous	0	1
PTK2	chr8	141696754/C//T	nonsynonymous	0	1
FUBP3	chr9	133511413/C//T	nonsynonymous	0	1
MRGPRF	chr11	68772827/G//A	synonymous	0	1
ARF3	chr12	49333497/T//C	nonsynonymous	0	1
ZNF26	chr12	133563452/C//T	nonsynonymous	0	1
	chr14	91360732/C//T	synonymous	0	1
ANPEP	chr15	90349291/T//C	nonsynonymous	0	1
KRT33A	chr17	39503345/G//A	nonsynonymous	0	1
ZNF208	chr19	22155346/T//C	synonymous	0	1
AP2A1	chr19	50305338/T//G	nonsynonymous	0	1
COL20A1	chr20	61952375/A//G	nonsynonymous	0	1
AGL	chr1	100379186/G//T	nonsynonymous	1	0
CEPT1	chr1	111703876/C//T	nonsynonymous	1	0
CELF3	chr1	151679703/G//T	nonsynonymous	1	0
KPRP	chr1	152733220/C//A	synonymous	1	0
ADAM15	chr1	155026879/A//G	nonsynonymous	1	0
LMX1A	chr1	165175245/C//A	nonsynonymous	1	0
CR1	chr1	207796399/A//G	synonymous	1	0
LYPLAL1	chr1	219384961/A//T	nonsynonymous	1	0
OR14I1	chr1	248845401/G//T	nonsynonymous	1	0
C2orf70	chr2	26798938/G//A	synonymous	1	0
EIF5B	chr2	99993088/C//T	nonsynonymous	1	0
CPS1	chr2	211476870/G//T	nonsynonymous	1	0
SLC23A3	chr2	220034589/AC//A	nonsynonymous	1	0

COL4A4	chr2	227942718/C//A	nonsynonymous	1	0
COL4A4	chr2	227942727/C//T	nonsynonymous	1	0
KLHL30	chr2	239049746/G//T	synonymous	1	0
GRM7	chr3	7620401/T//C	nonsynonymous	1	0
CACNA2D2	chr3	50414974/T//C	nonsynonymous	1	0
USF3	chr3	113389050/A//G	nonsynonymous	1	0
DTX3L	chr3	122288740/TC//T	nonsynonymous	1	0
SLC7A14	chr3	170198241/G//T	synonymous	1	0
SLC34A2	chr4	25674867/A//G	nonsynonymous	1	0
HELQ	chr4	84350691/C//A	nonsynonymous	1	0
CDKN2AIP	chr4	184367519/G//A	nonsynonymous	1	0
MYO10	chr5	16668494/T//A	nonsynonymous	1	0
CDH18	chr5	19473767/T//C	synonymous	1	0
MCTP1	chr5	94230365/A//G	synonymous	1	0
PJA2	chr5	108704383/C//A	nonsynonymous	1	0
PCDHGA1	chr5	140712160/G//C	nonsynonymous	1	0
PCDHGA2	chr5	140720256/C//G	nonsynonymous	1	0
PCDHGA3	chr5	140723858/C//T	synonymous	1	0
FAT2	chr5	150945851/A//G	nonsynonymous	1	0
GMDS	chr6	1961106/G//A	nonsynonymous	1	0
DPCR1	chr6	30919157/C//G	nonsynonymous	1	0
HLA-DPB1	chr6	33053609/G//A	nonsynonymous	1	0
FAM83B	chr6	54804557/G//C	nonsynonymous	1	0
GPR63	chr6	97246864/G//T	synonymous	1	0
ASCC3	chr6	101214507/T//A	synonymous	1	0
IL22RA2	chr6	137476112/C//T	nonsynonymous	1	0
FNDC1	chr6	159654470/G//T	nonsynonymous	1	0
GRM3	chr7	86493741/T//C	synonymous	1	0
SLC25A13	chr7	95761188/G//C	synonymous	1	0
	chr7	99672896/C//A	synonymous	1	0
DOCK4	chr7	111398834/T//C	nonsynonymous	1	0
TRPV5	chr7	142612711/T//C	nonsynonymous	1	0
DLGAP2	chr8	1497624/C//A	nonsynonymous	1	0
CSMD1	chr8	3076954/C//A	nonsynonymous	1	0
PRKDC	chr8	48846522/T//A	synonymous	1	0
CPSF1	chr8	145634480/G//C	nonsynonymous	1	0
LMX1B	chr9	129377736/A//T	nonsynonymous	1	0
NLRP6	chr11	284313/G//C	nonsynonymous	1	0
MYOD1	chr11	17741493/C//G	nonsynonymous	1	0
PDHX	chr11	34991723/G//A	nonsynonymous	1	0
OR5L2	chr11	55594856/G//A	synonymous	1	0
PATL1	chr11	59426726/T//C	nonsynonymous	1	0
PGA5	chr11	61017239/C//A	nonsynonymous	1	0
TUT1	chr11	62342891/C//T	nonsynonymous	1	0
KAT5	chr11	65482108/G//A	nonsynonymous	1	0
MUS81	chr11	65631139/T//A	nonsynonymous	1	0
SF3B2	chr11	65836262/G//C	synonymous	1	0
PC	chr11	66636358/G//A	synonymous	1	0
PPP1CA	chr11	67168357/CG//C	nonsynonymous	1	0
DDX6	chr11	118656787/TTGC//T	nonsynonymous	1	0
PKP2	chr12	33003830/A//G	synonymous	1	0
NAV3	chr12	78415564/G//T	nonsynonymous	1	0
C12orf50	chr12	88379816/T//C	nonsynonymous	1	0
GAS2L3	chr12	101017586/G//T	nonsynonymous	1	0
PAN3	chr13	28771473/A//T	nonsynonymous	1	0
COG3	chr13	46108878/G//T	synonymous	1	0
DNAJC3	chr13	96438192/G//T	nonsynonymous	1	0
NALCN	chr13	101997627/A//C	nonsynonymous	1	0
SLC12A1	chr15	48521473/C//T	nonsynonymous	1	0
UBE2Q2	chr15	76152321/C//A	nonsynonymous	1	0
TARSL2	chr15	102224408/C//A	nonsynonymous	1	0
PHKB	chr16	47495269/G//T	nonsynonymous	1	0
CLEC18B	chr16	74455173/C//G	synonymous	1	0
KIAA0513	chr16	85120727/A//G	nonsynonymous	1	0
ZNF469	chr16	88505640/C//G	nonsynonymous	1	0
ZMYND15	chr17	4644237/G//A	nonsynonymous	1	0
MYH8	chr17	10317509/A//T	nonsynonymous	1	0
MYH2	chr17	10427977/T//G	nonsynonymous	1	0
MYH3	chr17	10535011/G//C	nonsynonymous	1	0
CDRT15	chr17	14140080/C//G	nonsynonymous	1	0

SLC5A10	chr17	18923150/A//G	nonsynonymous	1	0
KIF18B	chr17	43010313/C//T	synonymous	1	0
LPO	chr17	56342245/C//T	nonsynonymous	1	0
UNC13D	chr17	73836181/G//A	nonsynonymous	1	0
ZACN	chr17	74075406/T//G	nonsynonymous	1	0
ZACN	chr17	74075424/T//G	nonsynonymous	1	0
SPHK1	chr17	74383640/G//A	synonymous	1	0
DNAH17	chr17	76571111/T//C	nonsynonymous	1	0
RPTOR	chr17	78867632/G//T	nonsynonymous	1	0
ANKRD27	chr19	33137525/G//A	synonymous	1	0
PLEKHG2	chr19	39914291/C//T	nonsynonymous	1	0
FBXO46	chr19	46215814/G//C	nonsynonymous	1	0
KLK2	chr19	51378077/C//A	nonsynonymous	1	0
KIR2DL1	chr19	55281308/G//T	synonymous	1	0
ACSS1	chr20	24994676/G//A	nonsynonymous	1	0
PHACTR3	chr20	58348392/C//A	synonymous	1	0
BIRC7	chr20	61867694/C//A	synonymous	1	0
ADAMTS5	chr21	28305319/C//T	synonymous	1	0
U2AF1L5	chr21	44524456/G//A	nonsynonymous	1	0
C22orf29	chr22	19839350/G//T	synonymous	1	0
SGSM1	chr22	25308632/C//T	synonymous	1	0
KIAA1671	chr22	25437223/C//T	nonsynonymous	1	0
SEC14L2	chr22	30803506/T//C	nonsynonymous	1	0
SHOX	chrX	595558/G//T	synonymous	1	0
RBM10	chrX	47044971/G//T	nonsynonymous	1	0
PHF8	chrX	53964525/G//A	synonymous	1	0
F9	chrX	138619263/G//A	synonymous	1	0
PRDM16	chr1	3328690/C//T	synonymous	0	1
UBE4B	chr1	10195238/G//GAA	nonsynonymous	0	1
ELOVL1	chr1	43830094/G//A	synonymous	0	1
KLF17	chr1	44595254/C//T	nonsynonymous	0	1
BARHL2	chr1	91182225/G//A	synonymous	0	1
TRIM33	chr1	114944006/C//G	synonymous	0	1
PLEKHA6	chr1	204226980/G//C	nonsynonymous	0	1
GKN1	chr2	69201706/T//A	synonymous	0	1
DNAH6	chr2	84774502/C//T	synonymous	0	1
ELMOD3	chr2	85617353/G//A	nonsynonymous	0	1
SNRNP200	chr2	96956422/C//T	synonymous	0	1
MYO7B	chr2	128384823/C//G	nonsynonymous	0	1
UGGT1	chr2	128938499/C//T	synonymous	0	1
SCN9A	chr2	167134663/C//G	nonsynonymous	0	1
ITGA4	chr2	182322985/C//T	nonsynonymous	0	1
ANO7	chr2	242135264/C//T	synonymous	0	1
ARPC4	chr3	9847973/C//T	synonymous	0	1
DYNC1LI1	chr3	32582581/G//A	nonsynonymous	0	1
PDE12	chr3	57542128/C//T	nonsynonymous	0	1
CBLB	chr3	105423021/G//T	synonymous	0	1
USP13	chr3	179458118/A//G	synonymous	0	1
JAKMIP1	chr4	6107415/G//A	nonsynonymous	0	1
SGCB	chr4	52894246/C//G	nonsynonymous	0	1
TMPRSS11D	chr4	68719895/G//T	nonsynonymous	0	1
STBD1	chr4	77231103/G//T	nonsynonymous	0	1
SLC9B2	chr4	103949940/A//G	synonymous	0	1
SEC24D	chr4	119665298/G//A	nonsynonymous	0	1
NPY1R	chr4	164247692/T//C	synonymous	0	1
NPY1R	chr4	164247697/T//G	nonsynonymous	0	1
TMEM192	chr4	166021955/G//C	nonsynonymous	0	1
CDK7	chr5	68555645/G//C	nonsynonymous	0	1
TAF9	chr5	68661256/A//C	synonymous	0	1
VCAN	chr5	82832940/C//T	nonsynonymous	0	1
PCDHB11	chr5	140581797/T//C	synonymous	0	1
DUSP1	chr5	172195881/C//T	nonsynonymous	0	1
KIAA1191	chr5	175777715/A//G	synonymous	0	1
IRF4	chr6	395840/C//T	synonymous	0	1
	chr6	5144453/C//T	synonymous	0	1
MED20	chr6	41877176/A//C	nonsynonymous	0	1
TTBK1	chr6	43230894/C//T	nonsynonymous	0	1
PKHD1	chr6	51920455/C//T	nonsynonymous	0	1
HCRTR2	chr6	55128625/A//C	synonymous	0	1
EYS	chr6	64431459/A//T	nonsynonymous	0	1

LAMA2	chr6	129775418/G//A	nonsynonymous	0	1
ADCY1	chr7	45717527/C//T	synonymous	0	1
MUC17	chr7	100684125/A//G	nonsynonymous	0	1
EPHB6	chr7	142568072/C//T	nonsynonymous	0	1
NEFM	chr8	24771311/G//C	nonsynonymous	0	1
PLAT	chr8	42033531/G//A	nonsynonymous	0	1
SOX17	chr8	55370939/G//T	nonsynonymous	0	1
VPS13B	chr8	100833581/A//T	synonymous	0	1
RIMS2	chr8	104897758/G//C	nonsynonymous	0	1
COL27A1	chr9	117044776/G//A	nonsynonymous	0	1
SHOC2	chr10	112767291/C//G	nonsynonymous	0	1
TCERG1L	chr10	133058540/G//A	nonsynonymous	0	1
KNDC1	chr10	135009284/C//T	nonsynonymous	0	1
OR52J3	chr11	5068646/C//A	synonymous	0	1
CSTF3	chr11	33106637/C//T	nonsynonymous	0	1
DDB1	chr11	61069791/C//G	synonymous	0	1
ESRRA	chr11	64074913/G//A	nonsynonymous	0	1
CD163L1	chr12	7519901/C//T	nonsynonymous	0	1
PRH1-TAS2R14	chr12	11091072/C//A	synonymous	0	1
HECTD4	chr12	112600304/G//A	nonsynonymous	0	1
ADGRD1	chr12	131490558/T//C	nonsynonymous	0	1
TEX26	chr13	31540449/A//G	nonsynonymous	0	1
PCDH9	chr13	66878829/T//C	synonymous	0	1
PCDH9	chr13	67801069/C//A	nonsynonymous	0	1
SLC10A2	chr13	103705040/A//T	nonsynonymous	0	1
BTBD7	chr14	93712396/A//C	nonsynonymous	0	1
OR4N4	chr15	22383353/T//A	nonsynonymous	0	1
TGM5	chr15	43552352/C//T	nonsynonymous	0	1
VWA9	chr15	65903475/T//G	nonsynonymous	0	1
UBE2Q2	chr15	76165911/T//G	nonsynonymous	0	1
KIAA0556	chr16	27732876/T//G	synonymous	0	1
TRPV1	chr17	3475439/C//A	nonsynonymous	0	1
TP53	chr17	7577556/C//T	nonsynonymous	0	1
RANGRF	chr17	8192147/T//C	nonsynonymous	0	1
MIEN1	chr17	37886737/C//T	synonymous	0	1
HEATR6	chr17	58121048/C//T	nonsynonymous	0	1
HELZ	chr17	65212007/A//C	synonymous	0	1
DNAH17	chr17	76450734/G//A	synonymous	0	1
PLPP2	chr19	281377/G//A	synonymous	0	1
RNF126	chr19	649678/C//G	nonsynonymous	0	1
HOOK2	chr19	12874192/C//G	nonsynonymous	0	1
ADGRL1	chr19	14263204/C//T	nonsynonymous	0	1
FAM129C	chr19	17650170/G//C	synonymous	0	1
CCNE1	chr19	30312932/T//A	synonymous	0	1
ZNF569	chr19	37904944/T//G	synonymous	0	1
CEACAM19	chr19	45175243/A//G	nonsynonymous	0	1
RSPH6A	chr19	46299073/C//G	synonymous	0	1
MYBPC2	chr19	50939377/C//T	nonsynonymous	0	1
RDH13	chr19	55558828/G//C	nonsynonymous	0	1
ZNF671	chr19	58231972/G//A	synonymous	0	1
FASTKD5	chr20	3127774/G//C	nonsynonymous	0	1
ADRA1D	chr20	4202749/C//T	synonymous	0	1
TRPC4AP	chr20	33592341/C//T	nonsynonymous	0	1
PTPRT	chr20	41306736/T//G	nonsynonymous	0	1
WFDC5	chr20	43738592/G//A	synonymous	0	1
SLC2A10	chr20	45354651/G//A	nonsynonymous	0	1
NFATC2	chr20	50140054/G//A	synonymous	0	1
TSHZ2	chr20	51870291/C//T	synonymous	0	1
LOC102724428	chr21	44837507/G//A	nonsynonymous	0	1
GTSE1	chr22	46709786/G//A	nonsynonymous	0	1
USP9X	chrX	41031097/T//C	nonsynonymous	0	1
MAGED2	chrX	54837359/C//T	nonsynonymous	0	1
ATP11C	chrX	138871574/T//TC	nonsynonymous	0	1
L1CAM	chrX	153129882/T//C	nonsynonymous	0	1
KLHDC7A	chr1	18809442/G//A	nonsynonymous	0	1
CSF1	chr1	110466468/C//T	nonsynonymous	0	1
SETDB1	chr1	150933325/G//A	synonymous	0	1
DCST2	chr1	154999106/C//T	synonymous	0	1
KLHL12	chr1	202880263/T//C	synonymous	0	1
FBXO28	chr1	224302099/G//A	nonsynonymous	0	1

ADI1	chr2	3502803/C//T	synonymous	0	1
THADA	chr2	43819540/G//T	synonymous	0	1
ETAA1	chr2	67624760/G//A	synonymous	0	1
	chr2	163279775/G//A	synonymous	0	1
FIGN	chr2	164467524/G//A	nonsynonymous	0	1
CCDC150	chr2	197559888/G//A	synonymous	0	1
GRIP2	chr3	14536452/C//T	nonsynonymous	0	1
CCR4	chr3	32995178/C//T	synonymous	0	1
SCN11A	chr3	38888976/C//T	nonsynonymous	0	1
NICN1	chr3	49463806/C//T	nonsynonymous	0	1
ZBTB11	chr3	101378824/T//G	nonsynonymous	0	1
ATR	chr3	142281185/G//T	nonsynonymous	0	1
AADACL2	chr3	151474834/C//T	nonsynonymous	0	1
KIT	chr4	55592200/T//C	synonymous	0	1
ADAMTS3	chr4	73175190/C//A	nonsynonymous	0	1
PPEF2	chr4	76794329/C//T	nonsynonymous	0	1
FBXL7	chr5	15937113/C//T	nonsynonymous	0	1
SPEF2	chr5	35704734/A//G	nonsynonymous	0	1
APC	chr5	112164586/C//T	nonsynonymous	0	1
PCDHA9	chr5	140229947/C//T	nonsynonymous	0	1
PCDHA13	chr5	140264052/G//A	synonymous	0	1
PCDHB8	chr5	140558899/G//T	synonymous	0	1
PCDHGA8	chr5	140774124/G//A	nonsynonymous	0	1
PCDHGB5	chr5	140779299/G//A	synonymous	0	1
GRM6	chr5	178410141/C//T	nonsynonymous	0	1
TBC1D9B	chr5	179318427/G//A	synonymous	0	1
WRNIP1	chr6	2783720/G//A	nonsynonymous	0	1
SIM1	chr6	100838544/C//T	nonsynonymous	0	1
HECA	chr6	139487600/G//A	nonsynonymous	0	1
UTRN	chr6	144852241/A//G	nonsynonymous	0	1
POM121	chr7	72416150/G//C	synonymous	0	1
BUD31	chr7	99008704/T//G	synonymous	0	1
SH2B2	chr7	101943925/G//A	nonsynonymous	0	1
SSPO	chr7	149521649/C//T	synonymous	0	1
RP1	chr8	55538827/C//A	synonymous	0	1
ADGRB1	chr8	143557999/C//T	nonsynonymous	0	1
RECQL4	chr8	145738405/C//T	synonymous	0	1
C9orf72	chr9	27567114/G//A	nonsynonymous	0	1
TMC1	chr9	75387463/C//T	synonymous	0	1
COL15A1	chr9	101797707/C//A	synonymous	0	1
NSMF	chr9	140348257/C//T	nonsynonymous	0	1
LARP4B	chr10	875655/T//C	nonsynonymous	0	1
ARHGAP21	chr10	24874763/C//T	synonymous	0	1
RAB6A	chr11	73471723/C//G	synonymous	0	1
TENM4	chr11	78614377/C//T	nonsynonymous	0	1
FAT3	chr11	92086867/C//T	nonsynonymous	0	1
CD163	chr12	7653830/C//T	nonsynonymous	0	1
TAS2R19	chr12	11174253/G//C	synonymous	0	1
TM7SF3	chr12	27149722/G//T	nonsynonymous	0	1
CEP290	chr12	88508240/C//A	nonsynonymous	0	1
NALCN	chr13	101742266/C//T	synonymous	0	1
NALCN	chr13	101763480/G//A	nonsynonymous	0	1
ADCY4	chr14	24795549/C//G	nonsynonymous	0	1
MDGA2	chr14	47770692/G//A	synonymous	0	1
HSPA2	chr14	65008263/C//T	synonymous	0	1
SPTB	chr14	65239526/C//T	synonymous	0	1
KCNK10	chr14	88707049/A//G	nonsynonymous	0	1
SERPINA4	chr14	95030071/G//A	synonymous	0	1
NPAP1	chr15	24921625/G//A	nonsynonymous	0	1
EMC4	chr15	34519894/C//T	nonsynonymous	0	1
DET1	chr15	89074729/G//A	nonsynonymous	0	1
CHSY1	chr15	101718073/T//C	synonymous	0	1
ST3GAL2	chr16	70422430/C//T	nonsynonymous	0	1
HYDIN	chr16	70952256/G//A	nonsynonymous	0	1
ZNF469	chr16	88501038/G//A	nonsynonymous	0	1
VTN	chr17	26694784/C//T	nonsynonymous	0	1
SCRN2	chr17	45915942/G//A	nonsynonymous	0	1
TTLL6	chr17	46882326/G//A	nonsynonymous	0	1
TMEM94	chr17	73495353/C//T	nonsynonymous	0	1
APBA3	chr19	3752566/G//A	synonymous	0	1

ARHGEF18	chr19	7523547/G//C	nonsynonymous	0	1
ZNF559	chr19	9449894/C//G	nonsynonymous	0	1
MAST3	chr19	18260384/C//T	nonsynonymous	0	1
ZNF430	chr19	21240355/C//T	nonsynonymous	0	1
ZNF729	chr19	22497472/C//A	nonsynonymous	0	1
ZNF536	chr19	30934537/G//C	nonsynonymous	0	1
	chr19	37487807/C//A	synonymous	0	1
ERICH4	chr19	41950106/C//T	nonsynonymous	0	1
SLC6A16	chr19	49812370/G//A	nonsynonymous	0	1
LRRC4B	chr19	51021572/G//A	synonymous	0	1
TNNI3	chr19	55667601/C//G	nonsynonymous	0	1
CHGB	chr20	5903187/C//T	nonsynonymous	0	1
CPNE1	chr20	34214650/C//T	nonsynonymous	0	1
LAMA5	chr20	60912702/G//A	nonsynonymous	0	1
SOX18	chr20	62679445/G//A	synonymous	0	1
PSMG1	chr21	40550578/CAT//C	synonymous	0	1
MX1	chr21	42818018/G//A	nonsynonymous	0	1
PCBP3	chr21	47361645/G//A	synonymous	0	1
TTC28	chr22	28702592/G//A	nonsynonymous	0	1
ASCC2	chr22	30184977/C//T	synonymous	0	1
LARGE1	chr22	33673142/C//T	synonymous	0	1
PNPLA5	chr22	44276620/G//T	synonymous	0	1
SPANXD	chrX	140785688/T//G	nonsynonymous	0	1
TARDBP	chr1	11082266/A//G	nonsynonymous	1	0
SNIP1	chr1	38019815/T//C	nonsynonymous	1	0
KIAA0754	chr1	39876785/G//A	nonsynonymous	1	0
CD53	chr1	111434097/A//T	synonymous	1	0
SPRR2E	chr1	153066078/G//A	synonymous	1	0
FCRL3	chr1	157668419/C//T	nonsynonymous	1	0
USH2A	chr1	216173745/T//A	nonsynonymous	1	0
PSEN2	chr1	227079037/C//A	synonymous	1	0
THADA	chr2	43798965/T//A	nonsynonymous	1	0
NAT8B	chr2	73928403/C//T	synonymous	1	0
LRP1B	chr2	141458092/C//A	nonsynonymous	1	0
LRP2	chr2	170068492/G//A	nonsynonymous	1	0
NFE2L2	chr2	178098969/G//T	nonsynonymous	1	0
TTN	chr2	179464408/T//G	nonsynonymous	1	0
TTN	chr2	179585928/T//A	nonsynonymous	1	0
	chr2	219188033/G//A	synonymous	1	0
TBC1D5	chr3	17333470/A//T	nonsynonymous	1	0
FAM208A	chr3	56657753/T//C	synonymous	1	0
COL6A5	chr3	130116760/G//T	nonsynonymous	1	0
MBNL1	chr3	152163154/T//C	synonymous	1	0
RAP2B	chr3	152881045/GCGCGCCGGCCGCG	synonymous	1	0
TP63	chr3	189582020/G//A	nonsynonymous	1	0
HTT	chr4	3158865/A//G	nonsynonymous	1	0
KDR	chr4	55964358/G//A	nonsynonymous	1	0
CTNND2	chr5	11411745/T//C	synonymous	1	0
LNPEP	chr5	96364105/G//A	nonsynonymous	1	0
UBTD2	chr5	171661207/C//A	nonsynonymous	1	0
DSP	chr6	7584497/A//G	synonymous	1	0
HIST1H3J	chr6	27858366/G//A	nonsynonymous	1	0
PRPH2	chr6	42666108/A//G	synonymous	1	0
CEP162	chr6	84913736/T//A	nonsynonymous	1	0
RSPO3	chr6	127476479/G//T	nonsynonymous	1	0
SLC2A12	chr6	134350441/C//T	nonsynonymous	1	0
UPK3B	chr7	76143298/G//T	nonsynonymous	1	0
NCAPG2	chr7	158486067/C//T	synonymous	1	0
CSMD1	chr8	3047488/G//C	nonsynonymous	1	0
KHDRBS3	chr8	136594216/G//C	nonsynonymous	1	0
ELAVL2	chr9	23761997/T//C	synonymous	1	0
DOLK	chr9	131709597/G//C	synonymous	1	0
SORCS1	chr10	108459131/G//A	synonymous	1	0
TNNI2	chr11	1862698/C//T	nonsynonymous	1	0
CHRNA10	chr11	3690513/C//A	nonsynonymous	1	0
OR52D1	chr11	5510070/A//G	nonsynonymous	1	0
NADSYN1	chr11	71189473/C//T	synonymous	1	0
KRT4	chr12	53207386/C//T	nonsynonymous	1	0
SLC16A7	chr12	60098658/G//C	nonsynonymous	1	0
BEST3	chr12	70049528/T//C	nonsynonymous	1	0

PPP1R12A	chr12	80199379/T//C	nonsynonymous	1	0
UTP20	chr12	101674936/A//C	nonsynonymous	1	0
ZNF84	chr12	133633903/G//C	nonsynonymous	1	0
UFM1	chr13	38924148/C//G	synonymous	1	0
OR4K17	chr14	20586282/G//A	synonymous	1	0
TRIP11	chr14	92470402/T//A	nonsynonymous	1	0
ATP6V0D1	chr16	67472364/GCA//G	synonymous	1	0
NFATC3	chr16	68225364/C//T	nonsynonymous	1	0
MYH4	chr17	10368822/G//A	nonsynonymous	1	0
MYO19	chr17	34867247/G//A	synonymous	1	0
GJD3	chr17	38519387/G//A	synonymous	1	0
KRTAP4-8	chr17	39254142/A//G	synonymous	1	0
MPP2	chr17	41981858/TCTCCTGGAG//T	nonsynonymous	1	0
MTCL1	chr18	8783701/C//T	synonymous	1	0
APBA3	chr19	3751324/A//G	nonsynonymous	1	0
VMAC	chr19	5909108/G//A	synonymous	1	0
MLLT1	chr19	6227019/G//A	nonsynonymous	1	0
FAM187B	chr19	35715900/T//A	nonsynonymous	1	0
HIPK4	chr19	40886982/G//A	nonsynonymous	1	0
C19orf54	chr19	41248607/A//G	nonsynonymous	1	0
FBXO46	chr19	46216077/C//T	nonsynonymous	1	0
ARHGAP35	chr19	47440632/A//T	nonsynonymous	1	0
LILRA5	chr19	54818795/C//T	nonsynonymous	1	0
ATRN	chr20	3557645/G//A	nonsynonymous	1	0
SNX21	chr20	44469994/C//T	synonymous	1	0
DIDO1	chr20	61538658/C//A	synonymous	1	0
RUNX1	chr21	36259134/C//T	synonymous	1	0
BRWD1	chr21	40559045/G//A	synonymous	1	0
THAP7	chr22	21356176/C//T	nonsynonymous	1	0
TLR8	chrX	12937291/A//T	synonymous	1	0
ACTRT2	chr1	2939042/C//T	synonymous	1	0
MAN1C1	chr1	26104704/G//A	nonsynonymous	1	0
PTPRU	chr1	29587363/C//T	synonymous	1	0
CSMD2	chr1	34052741/G//A	nonsynonymous	1	0
HPDL	chr1	45793826/T//TTC	nonsynonymous	1	0
MROH7	chr1	55144480/C//T	nonsynonymous	1	0
CD5L	chr1	157804407/G//A	nonsynonymous	1	0
SPTA1	chr1	158612287/G//A	nonsynonymous	1	0
OR2T4	chr1	248525275/G//T	nonsynonymous	1	0
GREB1	chr2	11774474/C//T	nonsynonymous	1	0
SPAST	chr2	32289188/G//A	synonymous	1	0
BIRC6	chr2	32626290/A//ATG	nonsynonymous	1	0
KCNK12	chr2	47748369/C//T	nonsynonymous	1	0
GLI2	chr2	121747048/C//CG	nonsynonymous	1	0
KYNU	chr2	143798027/C//T	nonsynonymous	1	0
KCNE4	chr2	223917884/G//A	synonymous	1	0
COLQ	chr3	15520497/C//T	nonsynonymous	1	0
PBRM1	chr3	52637555/G//A	nonsynonymous	1	0
ROBO2	chr3	77147279/C//T	nonsynonymous	1	0
OR5H6	chr3	97983658/C//A	nonsynonymous	1	0
COL8A1	chr3	99509727/C//A	synonymous	1	0
MED12L	chr3	151078286/C//T	synonymous	1	0
TMPRSS11A	chr4	68784770/A//T	synonymous	1	0
MAPK10	chr4	86938374/C//T	synonymous	1	0
NFKB1	chr4	103527722/G//A	nonsynonymous	1	0
FAM134B	chr5	16474917/T//C	nonsynonymous	1	0
ARSB	chr5	78181573/C//T	nonsynonymous	1	0
MEGF10	chr5	126732462/C//T	synonymous	1	0
	chr5	131534058/C//T	synonymous	1	0
PCDHGA6	chr5	140755848/C//T	nonsynonymous	1	0
GABRA1	chr5	161277795/T//C	synonymous	1	0
PLA2G7	chr6	46684223/G//A	nonsynonymous	1	0
TBX18	chr6	85446668/G//C	nonsynonymous	1	0
GRM1	chr6	146720509/C//T	synonymous	1	0
MIOS	chr7	7612552/G//T	nonsynonymous	1	0
FERD3L	chr7	19184766/C//T	nonsynonymous	1	0
GLI3	chr7	42004329/C//T	nonsynonymous	1	0
COBL	chr7	51111322/C//T	synonymous	1	0
	chr8	31498184/C//T	synonymous	1	0
RUNX1T1	chr8	93004052/G//C	nonsynonymous	1	0

COL27A1	chr9	116930013/G//A	nonsynonymous	1	0
POLR3A	chr10	79784800/C//T	nonsynonymous	1	0
GALNT18	chr11	11394142/G//A	nonsynonymous	1	0
KIAA1549L	chr11	33604953/C//T	nonsynonymous	1	0
MYRF	chr11	61541579/C//T	nonsynonymous	1	0
TRIM77	chr11	89443484/G//A	synonymous	1	0
TTC36	chr11	118398228/C//T	nonsynonymous	1	0
P3H3	chr12	6948564/G//A	nonsynonymous	1	0
GRIN2B	chr12	13906587/C//A	nonsynonymous	1	0
LDHB	chr12	21796917/T//A	nonsynonymous	1	0
PKP2	chr12	33021996/C//G	synonymous	1	0
HNRNPA1	chr12	54676359/C//A	synonymous	1	0
OR6C65	chr12	55795024/A//T	nonsynonymous	1	0
TBC1D15	chr12	72274354/G//C	nonsynonymous	1	0
C12orf42	chr12	103696309/G//T	synonymous	1	0
CKAP2	chr13	53036547/C//G	nonsynonymous	1	0
CCDC168	chr13	103388680/G//A	synonymous	1	0
FBN1	chr15	48937088/C//G	synonymous	1	0
GALK2	chr15	49509471/C//T	nonsynonymous	1	0
GRIN2A	chr16	9943750/G//A	synonymous	1	0
NETO2	chr16	47162361/T//A	nonsynonymous	1	0
LRRC36	chr16	67418886/C//A	nonsynonymous	1	0
HYDIN	chr16	71065612/G//A	nonsynonymous	1	0
JPH3	chr16	87677973/C//A	synonymous	1	0
ACACA	chr17	35557125/TC//T	nonsynonymous	1	0
BPTF	chr17	65907995/T//G	nonsynonymous	1	0
CANT1	chr17	76989693/C//T	nonsynonymous	1	0
CTDP1	chr18	77475022/C//T	nonsynonymous	1	0
RFX2	chr19	6002749/C//A	nonsynonymous	1	0
PNPLA6	chr19	7625640/G//A	nonsynonymous	1	0
HAPLN4	chr19	19369607/G//A	nonsynonymous	1	0
ATP4A	chr19	36054173/G//T	synonymous	1	0
FOSB	chr19	45974139/G//A	nonsynonymous	1	0
FBXO46	chr19	46216673/C//T	synonymous	1	0
ZSCAN1	chr19	58565250/G//A	nonsynonymous	1	0
APMAP	chr20	24944599/G//A	synonymous	1	0
	chr20	60074490/G//T	synonymous	1	0
CRYBA4	chr22	27019251/C//T	synonymous	1	0
ARSF	chrX	3030585/C//T	synonymous	1	0
CA5B	chrX	15792527/T//A	synonymous	1	0
NTNG1	chr1	107937956/A//C	synonymous	0	1
RGSL1	chr1	182491205/T//C	nonsynonymous	0	1
TMCC2	chr1	205241043/G//A	nonsynonymous	0	1
OBSCN	chr1	228494222/C//T	nonsynonymous	0	1
SCAP	chr3	47470070/A//G	nonsynonymous	0	1
KIAA1524	chr3	108304006/T//A	synonymous	0	1
LGI2	chr4	25019735/C//G	nonsynonymous	0	1
MAP1B	chr5	71492853/G//T	nonsynonymous	0	1
APC	chr5	112164578/T//A	nonsynonymous	0	1
LARS	chr5	145509709/T//C	nonsynonymous	0	1
MAPK14	chr6	36063811/G//A	nonsynonymous	0	1
IKZF1	chr7	50367289/G//A	synonymous	0	1
PCLO	chr7	82581167/C//G	synonymous	0	1
MUC12	chr7	100635178/A//T	nonsynonymous	0	1
PLXNA4	chr7	132192823/C//T	synonymous	0	1
CDH17	chr8	95174368/C//T	synonymous	0	1
C5	chr9	123776206/G//A	synonymous	0	1
COL5A1	chr9	137642661/C//T	nonsynonymous	0	1
ARMC3	chr10	23326330/C//T	synonymous	0	1
LCOR	chr10	98742016/C//T	nonsynonymous	0	1
CNNM1	chr10	101089444/C//T	synonymous	0	1
OR5M9	chr11	56230253/G//T	nonsynonymous	0	1
TMX2	chr11	57480265/T//C	nonsynonymous	0	1
ZFP91	chr11	58346873/C//T	nonsynonymous	0	1
TYR	chr11	88924452/C//T	nonsynonymous	0	1
EXPH5	chr11	108385606/A//G	synonymous	0	1
GNB3	chr12	6953097/C//T	synonymous	0	1
DBX2	chr12	45444578/G//A	nonsynonymous	0	1
FAM186B	chr12	49992607/C//T	synonymous	0	1
KRT6B	chr12	52845400/C//T	nonsynonymous	0	1

EIF4B	chr12	53421646/G//A	nonsynonymous	0	1
MAP3K12	chr12	53876679/G//T	synonymous	0	1
LRP1	chr12	57553669/C//T	synonymous	0	1
CNOT2	chr12	70732324/G//A	synonymous	0	1
DEPDC4	chr12	100656060/G//C	nonsynonymous	0	1
P2RX7	chr12	121613220/T//C	nonsynonymous	0	1
KDM2B	chr12	121987380/G//A	synonymous	0	1
WDR66	chr12	122413496/G//A	nonsynonymous	0	1
TMEM132B	chr12	126068449/C//A	nonsynonymous	0	1
WASF3	chr13	27255395/G//A	synonymous	0	1
UNC13C	chr15	54914521/T//A	synonymous	0	1
CD19	chr16	28943799/A//G	nonsynonymous	0	1
MYLK3	chr16	46781887/C//T	synonymous	0	1
PLEKHG4	chr16	67316439/C//G	nonsynonymous	0	1
CDK5R1	chr17	30814997/G//C	nonsynonymous	0	1
MYBPC2	chr19	50939082/T//G	synonymous	0	1
SIGLEC5	chr19	52130495/G//A	nonsynonymous	0	1
RBPJL	chr20	43945122/C//T	synonymous	0	1
LAMA5	chr20	60888834/G//A	synonymous	0	1
DDX17	chr22	38881951/AAGG//A	nonsynonymous	0	1
MXRA5	chrX	3241585/A//T	nonsynonymous	0	1
P2RY4	chrX	69479282/G//A	nonsynonymous	0	1
POU3F4	chrX	82763633/G//A	nonsynonymous	0	1
PUM1	chr1	31406102/C//G	nonsynonymous	0	1
FOXE3	chr1	47883043/A//G	synonymous	0	1
ANGPTL1	chr1	178820238/T//C	synonymous	0	1
VN1R5	chr1	247419716/G//T	nonsynonymous	0	1
OR2T1	chr1	248569354/T//G	nonsynonymous	0	1
HDLBP	chr2	242179108/G//C	nonsynonymous	0	1
PIK3CA	chr3	178938934/G//A	nonsynonymous	0	1
RNF168	chr3	196199258/T//C	nonsynonymous	0	1
MAPK10	chr4	86989041/C//A	nonsynonymous	0	1
PDLIM4	chr5	131607507/C//T	nonsynonymous	0	1
ADRB2	chr5	148207046/G//A	nonsynonymous	0	1
COL1A2	chr7	94024390/C//A	nonsynonymous	0	1
CNTNAP2	chr7	147926846/G//A	nonsynonymous	0	1
MRPL15	chr8	55047798/T//A	synonymous	0	1
SMARCA2	chr9	2191355/A//C	nonsynonymous	0	1
CER1	chr9	14722224/G//T	synonymous	0	1
HSD17B3	chr9	99064310/C//T	nonsynonymous	0	1
MYOF	chr10	95089444/G//A	synonymous	0	1
LGR5	chr12	71918204/T//C	synonymous	0	1
CEP83	chr12	94772672/C//T	synonymous	0	1
RPLP0	chr12	120638651/A//G	synonymous	0	1
OAZ2	chr15	64981025/G//A	nonsynonymous	0	1
MLYCD	chr16	83933211/G//A	synonymous	0	1
FASN	chr17	80045615/G//A	nonsynonymous	0	1
EPG5	chr18	43534537/T//C	synonymous	0	1
MUC16	chr19	9070918/A//G	nonsynonymous	0	1
DOCK6	chr19	11312753/G//T	nonsynonymous	0	1
CILP2	chr19	19651121/C//T	nonsynonymous	0	1
TMEM74B	chr20	1161796/G//A	nonsynonymous	0	1
DGCR14	chr22	19121981/G//A	nonsynonymous	0	1
MN1	chr22	28194681/G//A	synonymous	0	1
FAM46D	chrX	79698430/T//C	nonsynonymous	0	1
H6PD	chr1	9324740/A//G	nonsynonymous	0	1
EPHA8	chr1	22903038/G//A	nonsynonymous	0	1
SLC5A9	chr1	48713132/G//A	nonsynonymous	0	1
TSSC1	chr2	3358345/C//T	synonymous	0	1
SLC25A12	chr2	172650226/G//A	nonsynonymous	0	1
XIRP1	chr3	39228823/G//T	nonsynonymous	0	1
MED12L	chr3	151067877/C//T	nonsynonymous	0	1
SH3TC1	chr4	8238150/C//CT	nonsynonymous	0	1
ADAMTS16	chr5	5186274/C//T	synonymous	0	1
OR2J2	chr6	29141439/G//A	synonymous	0	1
MEP1A	chr6	46802394/C//T	synonymous	0	1
ADGRF2	chr6	47650350/C//A	synonymous	0	1
SFRP4	chr7	37947249/C//A	synonymous	0	1
ABCA13	chr7	48411803/C//A	nonsynonymous	0	1
RINT1	chr7	105187352/T//C	synonymous	0	1

CNGB3	chr8	87683247/G//A	nonsynonymous	0	1
CASP1	chr11	104905166/G//T	nonsynonymous	0	1
FLI1	chr11	128680482/C//T	nonsynonymous	0	1
B3GAT1	chr11	134253667/C//T	nonsynonymous	0	1
ERC1	chr12	1292584/A//G	synonymous	0	1
ACSS3	chr12	81568722/A//G	synonymous	0	1
TSC22D1	chr13	45148196/G//C	nonsynonymous	0	1
BEGAIN	chr14	101005343/C//T	nonsynonymous	0	1
CRAMP1	chr16	1676080/C//T	synonymous	0	1
C16orf45	chr16	15609187/G//A	synonymous	0	1
SLC39A6	chr18	33706857/C//T	synonymous	0	1
ZNF91	chr19	23544317/C//T	synonymous	0	1
GDF5	chr20	34022455/G//A	nonsynonymous	0	1
KCNQ2	chr20	62071011/G//A	synonymous	0	1
	chr21	15954671/C//A	synonymous	0	1
KRTAP10-6	chr21	46011496/C//A	synonymous	0	1
SSX5	chrX	48053616/C//T	nonsynonymous	0	1
SMC1A	chrX	53439105/G//A	nonsynonymous	0	1
CASP9	chr1	15850675/C//A	synonymous	0	1
PGM1	chr1	64095214/G//T	nonsynonymous	0	1
LCE1C	chr1	152777698/T//A	nonsynonymous	0	1
MYOG	chr1	203054632/C//T	nonsynonymous	0	1
RYR2	chr1	237796976/C//G	synonymous	0	1
OR2T10	chr1	248756761/G//C	nonsynonymous	0	1
NFE2L2	chr2	178095511/ATC//A	nonsynonymous	0	1
FBLN2	chr3	13668033/A//T	synonymous	0	1
XRN1	chr3	142030262/T//C	synonymous	0	1
SI	chr3	164727160/G//C	nonsynonymous	0	1
NAALADL2	chr3	174974208/C//A	synonymous	0	1
TBC1D14	chr4	6925307/T//C	nonsynonymous	0	1
SLC34A2	chr4	25667723/TTTTG//T	synonymous	0	1
SLC34A2	chr4	25667735/GTTTGT//G	synonymous	0	1
TRIM60	chr4	165961979/C//A	nonsynonymous	0	1
DST	chr6	56504969/TC//T	nonsynonymous	0	1
ABCB5	chr7	20782631/CG//C	nonsynonymous	0	1
HGF	chr7	81336653/C//A	nonsynonymous	0	1
PCLO	chr7	82390101/C//G	nonsynonymous	0	1
TRRAP	chr7	98506345/C//G	synonymous	0	1
EPPK1	chr8	144943280/C//T	nonsynonymous	0	1
ZNF34	chr8	145999825/G//C	nonsynonymous	0	1
WHRN	chr9	117266726/G//A	nonsynonymous	0	1
C10orf107	chr10	63519814/C//T	synonymous	0	1
FAM204A	chr10	120095716/C//T	nonsynonymous	0	1
GLRX3	chr10	131959192/T//A	nonsynonymous	0	1
IGF2	chr11	2154144/T//C	synonymous	0	1
OR52K2	chr11	4470702/T//C	nonsynonymous	0	1
TNKS1BP1	chr11	57080756/T//C	nonsynonymous	0	1
PATL1	chr11	59417078/G//C	nonsynonymous	0	1
FCHSD2	chr11	72553678/C//T	nonsynonymous	0	1
CHRDL2	chr11	74429831/G//A	synonymous	0	1
FAR2	chr12	29462148/TG//T	nonsynonymous	0	1
KCNH3	chr12	49943951/C//A	nonsynonymous	0	1
PPFIA2	chr12	81746949/G//T	nonsynonymous	0	1
NR1H4	chr12	100926290/G//C	nonsynonymous	0	1
UCHL3	chr13	76179930/T//G	nonsynonymous	0	1
LCTL	chr15	66853575/C//G	nonsynonymous	0	1
MYLPF	chr16	30387236/G//T	synonymous	0	1
HS3ST3A1	chr17	13503886/G//A	synonymous	0	1
EVI2B	chr17	29631196/C//G	synonymous	0	1
AFG3L2	chr18	12340252/G//C	nonsynonymous	0	1
FCGBP	chr19	40373955/G//A	synonymous	0	1
CRLS1	chr20	5986868/G//C	synonymous	0	1
TAF7L	chrX	100533046/C//A	nonsynonymous	0	1
TEX13C	chrX	124454433/C//G	synonymous	0	1
DCAF12L1	chrX	125686100/C//T	synonymous	0	1
UBE2J2	chr1	1192642/T//G	nonsynonymous	0	1
SGIP1	chr1	67147979/A//C	synonymous	0	1
OBSCN	chr1	228495136/C//T	nonsynonymous	0	1
GGCX	chr2	85781396/C//T	synonymous	0	1
TTC30A	chr2	178482604/C//T	nonsynonymous	0	1

GNAT1	chr3	50231182/G//A	synonymous	0	1
SEMA3G	chr3	52475330/C//T	nonsynonymous	0	1
EPHA3	chr3	89259166/C//T	nonsynonymous	0	1
ADAD1	chr4	123301400/C//T	synonymous	0	1
PCDHB2	chr5	140475170/G//A	nonsynonymous	0	1
IER3	chr6	30711911/G//A	synonymous	0	1
TMEM63B	chr6	44121819/C//T	nonsynonymous	0	1
FAM135A	chr6	71235897/G//A	nonsynonymous	0	1
MICAL1	chr6	109773533/C//T	nonsynonymous	0	1
HIP1	chr7	75221753/G//A	synonymous	0	1
NYAP1	chr7	100086957/C//T	nonsynonymous	0	1
EXTL3	chr8	28574802/C//T	nonsynonymous	0	1
SPATA31C2	chr9	90745357/C//T	synonymous	0	1
CNTRL	chr9	123880693/C//T	nonsynonymous	0	1
HRASLS5	chr11	63233779/G//A	nonsynonymous	0	1
FAT3	chr11	92600302/C//T	synonymous	0	1
MAN2C1	chr15	75654008/G//A	synonymous	0	1
PKD1	chr16	2142522/T//C	nonsynonymous	0	1
SMAD4	chr18	48591894/T//A	nonsynonymous	0	1
	chr19	15754855/G//A	synonymous	0	1
HAPLN4	chr19	19371745/C//T	nonsynonymous	0	1
ZNF98	chr19	22575496/A//T	nonsynonymous	0	1
FLRT3	chr20	14307034/T//A	synonymous	0	1
TRPM2	chr21	45789077/G//A	nonsynonymous	0	1
SULT4A1	chr22	44225047/C//A	nonsynonymous	0	1
FAM47B	chrX	34962445/G//C	nonsynonymous	0	1
CYLC1	chrX	83128550/G//A	synonymous	0	1
DCAF12L2	chrX	125298766/T//A	nonsynonymous	0	1
GPR50	chrX	150348846/C//T	nonsynonymous	0	1
TKTL1	chrX	153539368/G//A	nonsynonymous	0	1
DOCK7	chr1	63113932/AG//A	nonsynonymous	0	1
CCT3	chr1	156307997/G//A	synonymous	0	1
SEMA4F	chr2	74907086/G//A	nonsynonymous	0	1
ZMIZ1	chr10	81051993/CGCGGCAGCCGCTGC	nonsynonymous	0	1
CSPG4	chr15	75981709/A//G	nonsynonymous	0	1
ERBB2	chr17	37881345/T//C	nonsynonymous	0	1
ACP7	chr19	39589686/G//A	nonsynonymous	0	1
SBK2	chr19	56047541/C//T	nonsynonymous	0	1
TFAP2C	chr20	55206670/C//T	nonsynonymous	0	1
GTPBP1	chr22	39125525/A//G	nonsynonymous	0	1
GPR50	chrX	150345216/C//G	nonsynonymous	0	1
PCDHA3	chr5	140183048/C//A	nonsynonymous	0	1
TNFSF11	chr13	43148506/G//A	nonsynonymous	0	1
MSI2	chr17	55693409/A//G	nonsynonymous	0	1
SLC16A3	chr17	80195184/T//C	nonsynonymous	0	1
CHERP	chr19	16640563/AGCT//A	nonsynonymous	0	1
ZNF676	chr19	22363008/C//G	nonsynonymous	0	1
SAMM50	chr22	44368781/G//A	nonsynonymous	0	1
PIP4K2A	chr10	23003239/T//C	nonsynonymous	1	0
GCN1	chr12	120599831/A//G	nonsynonymous	1	0
SERINC4	chr15	44087251/T//C	nonsynonymous	1	0
INSL3	chr19	17932375/A//G	synonymous	1	0
ST6GAL2	chr2	107460201/T//C	nonsynonymous	1	0
TRAF2	chr9	139793362/G//C	nonsynonymous	1	0
ADIPOR2	chr12	1863603/A//G	nonsynonymous	1	0
ATP10A	chr15	25959150/C//T	nonsynonymous	1	0
NAE1	chr16	66852506/A//G	synonymous	1	0
ZNRF1	chr16	75033852/T//C	nonsynonymous	1	0
ABCA9	chr17	67022530/C//T	nonsynonymous	1	0
MMEL1	chr1	2542728/A//G	nonsynonymous	1	0
	chr1	247150542/T//A	synonymous	1	0
	chr1	247150551/G//A	synonymous	1	0
PXDN	chr2	1639248/C//G	nonsynonymous	1	0
C5orf42	chr5	37165766/T//C	nonsynonymous	1	0
KCNK5	chr6	39162513/T//C	nonsynonymous	1	0
SSC4D	chr7	76022934/T//C	nonsynonymous	1	0
PODXL	chr7	131241050/C//T	synonymous	1	0
MBOAT4	chr8	29990036/A//G	nonsynonymous	1	0
DMRT1	chr9	841741/T//C	synonymous	1	0
ECD	chr10	74914059/A//G	synonymous	1	0

LARP4	chr12	50867931/C//CG	nonsynonymous	1	0
C14orf2	chr14	104379017/G//A	nonsynonymous	1	0
IGDCC4	chr15	65702650/T//C	synonymous	1	0
EFNB3	chr17	7613025/T//C	synonymous	1	0
ZNF626	chr19	20807769/A//C	nonsynonymous	1	0
ZNF676	chr19	22363615/T//C	nonsynonymous	1	0
ZNF676	chr19	22363631/G//A	synonymous	1	0
ZNF676	chr19	22363634/C//A	nonsynonymous	1	0
MYH14	chr19	50770179/G//A	nonsynonymous	1	0
DTD1	chr20	18724797/A//G	synonymous	1	0
JADE3	chrX	46884272/A//G	nonsynonymous	1	0
ZCCHC12	chrX	117959635/A//G	nonsynonymous	1	0
AGRN	chr1	979794/C//T	synonymous	1	0
DPYD	chr1	97839203/T//A	synonymous	1	0
FLG	chr1	152280074/G//A	nonsynonymous	1	0
CHRNB2	chr1	154543930/C//T	nonsynonymous	1	0
KCNJ10	chr1	160012074/G//T	synonymous	1	0
OR2L3	chr1	248224727/T//G	synonymous	1	0
LRP2	chr2	170063416/G//A	nonsynonymous	1	0
IRS1	chr2	227662489/G//A	synonymous	1	0
TRAK1	chr3	42264846/G//A	nonsynonymous	1	0
UBA3	chr3	69104537/A//G	synonymous	1	0
HSD17B13	chr4	88226425/C//T	nonsynonymous	1	0
LNPEP	chr5	96358084/T//C	synonymous	1	0
	chr5	112043461/C//T	synonymous	1	0
PCDHA2	chr5	140176778/C//A	nonsynonymous	1	0
PCDHGB3	chr5	140751848/G//A	synonymous	1	0
PRSS35	chr6	84234147/C//T	synonymous	1	0
GOPC	chr6	117890852/C//T	synonymous	1	0
CPA1	chr7	130023270/C//T	synonymous	1	0
CUL1	chr7	148484180/G//A	nonsynonymous	1	0
GIMAP6	chr7	150324902/C//T	nonsynonymous	1	0
CSMD1	chr8	3047474/G//A	synonymous	1	0
SLC39A14	chr8	22269720/G//A	nonsynonymous	1	0
CYHR1	chr8	145690232/A//T	nonsynonymous	1	0
TAF1L	chr9	32632961/G//A	nonsynonymous	1	0
OR1L6	chr9	125512606/C//T	synonymous	1	0
KIF5B	chr10	32317393/G//T	nonsynonymous	1	0
SORCS1	chr10	108431069/C//T	synonymous	1	0
RNF169	chr11	74546991/C//A	nonsynonymous	1	0
FOXM1	chr12	2967884/T//G	nonsynonymous	1	0
PZP	chr12	9307325/C//T	nonsynonymous	1	0
PUS7L	chr12	44124241/CAA//C	nonsynonymous	1	0
POLR3B	chr12	106804719/G//A	nonsynonymous	1	0
TCTN2	chr12	124184288/G//A	nonsynonymous	1	0
TMEM132B	chr12	126138932/G//C	nonsynonymous	1	0
SUGT1	chr13	53241033/G//A	synonymous	1	0
SLITRK1	chr13	84454176/G//A	synonymous	1	0
SLITRK1	chr13	84454768/G//C	nonsynonymous	1	0
CCDC168	chr13	103401346/C//T	synonymous	1	0
LRP10	chr14	23346254/C//T	nonsynonymous	1	0
GZMB	chr14	25101548/G//A	synonymous	1	0
GZMB	chr14	25101589/G//C	nonsynonymous	1	0
APBA2	chr15	29409362/A//G	synonymous	1	0
TSC2	chr16	2108832/T//C	synonymous	1	0
TMC7	chr16	19049234/A//G	synonymous	1	0
GTF3C1	chr16	27514295/T//A	nonsynonymous	1	0
CTU2	chr16	88780897/CTG//C	synonymous	1	0
SMYD4	chr17	1690801/G//A	synonymous	1	0
CHD3	chr17	7798351/C//G	nonsynonymous	1	0
SLC35G3	chr17	33521220/G//A	nonsynonymous	1	0
SP6	chr17	45925699/G//A	nonsynonymous	1	0
CDR2L	chr17	72999831/C//T	nonsynonymous	1	0
LBP	chr20	36982731/C//A	nonsynonymous	1	0
PTPRT	chr20	41419897/C//T	nonsynonymous	1	0
JPH2	chr20	42788732/C//T	nonsynonymous	1	0
OPRL1	chr20	62729459/G//C	nonsynonymous	1	0
ADAMTS5	chr21	28338183/T//G	nonsynonymous	1	0
ZCCHC5	chrX	77912749/A//G	nonsynonymous	1	0
TAF7L	chrX	100547857/G//A	synonymous	1	0

ZIC3	chrX	136649333/G//T	synonymous	1	0
SPANXD	chrX	140785625/C//A	nonsynonymous	1	0
FMR1	chrX	147014079/G//A	nonsynonymous	1	0
ARHGAP4	chrX	153174570/C//T	nonsynonymous	1	0
HIVEP3	chr1	42045813/C//T	synonymous	0	1
CYP4B1	chr1	47264788/C//T	nonsynonymous	0	1
MYSM1	chr1	59165695/G//A	synonymous	0	1
PTGFR	chr1	79002253/C//A	nonsynonymous	0	1
MCOLN2	chr1	85405335/G//A	synonymous	0	1
GBP7	chr1	89599000/C//G	nonsynonymous	0	1
COL11A1	chr1	103540170/T//C	synonymous	0	1
HRNR	chr1	152191365/C//T	nonsynonymous	0	1
UBAP2L	chr1	154221739/G//C	nonsynonymous	0	1
KIAA0040	chr1	175130048/G//T	synonymous	0	1
CFHR3	chr1	196743997/T//G	synonymous	0	1
CRB1	chr1	197390836/T//C	synonymous	0	1
SRGAP2	chr1	206634422/G//A	nonsynonymous	0	1
TRIM11	chr1	228588676/C//G	nonsynonymous	0	1
MAP3K21	chr1	233507833/G//A	synonymous	0	1
TPO	chr2	1426881/C//A	synonymous	0	1
HECW2	chr2	197118754/C//A	nonsynonymous	0	1
SCG2	chr2	224462870/T//A	synonymous	0	1
ARL4C	chr2	235404992/T//A	nonsynonymous	0	1
AGAP1	chr2	236659131/C//T	synonymous	0	1
COL6A3	chr2	238243285/G//A	synonymous	0	1
SUSD5	chr3	33194840/G//A	synonymous	0	1
COL7A1	chr3	48601795/C//T	synonymous	0	1
MITF	chr3	70008562/C//T	synonymous	0	1
USF3	chr3	113376663/G//A	nonsynonymous	0	1
SH3TC1	chr4	8233747/G//A	nonsynonymous	0	1
KIT	chr4	55561681/C//G	nonsynonymous	0	1
KIAA1211	chr4	57182264/CAGA//C	nonsynonymous	0	1
GC	chr4	72611869/A//T	synonymous	0	1
	chr5	59284438/G//A	synonymous	0	1
APC	chr5	112128191/C//T	nonsynonymous	0	1
REEP5	chr5	112257816/G//C	synonymous	0	1
FBN2	chr5	127626533/C//G	nonsynonymous	0	1
PCDHB4	chr5	140502210/C//T	synonymous	0	1
PCDH1	chr5	141243762/C//T	nonsynonymous	0	1
STC2	chr5	172744904/G//A	synonymous	0	1
RMND5B	chr5	177570001/C//T	synonymous	0	1
NUP153	chr6	17625046/C//T	nonsynonymous	0	1
GPSM3	chr6	32159157/T//G	nonsynonymous	0	1
PTCRA	chr6	42893213/C//T	synonymous	0	1
PKHD1	chr6	51524320/T//C	nonsynonymous	0	1
DDO	chr6	110714549/C//T	synonymous	0	1
SLC16A10	chr6	111540010/G//A	synonymous	0	1
MYCT1	chr6	153043221/G//C	nonsynonymous	0	1
FERD3L	chr7	19184863/GT//G	nonsynonymous	0	1
IKZF1	chr7	50455035/T//G	synonymous	0	1
SEMA3C	chr7	80378263/T//C	nonsynonymous	0	1
STAG3	chr7	99796170/C//T	synonymous	0	1
OR9A4	chr7	141619376/G//A	nonsynonymous	0	1
PRKAG2	chr7	151372634/G//A	nonsynonymous	0	1
XKR5	chr8	6669809/T//C	nonsynonymous	0	1
INTS9	chr8	28627517/C//T	synonymous	0	1
GPIHBP1	chr8	144295103/G//A	synonymous	0	1
PRUNE2	chr9	79321992/T//G	nonsynonymous	0	1
GABBR2	chr9	101216403/C//T	nonsynonymous	0	1
OLFM1	chr9	138011488/G//A	nonsynonymous	0	1
MYO3A	chr10	26491973/C//G	nonsynonymous	0	1
SLIT1	chr10	98819920/G//A	synonymous	0	1
ANKRD2	chr10	99340708/C//T	nonsynonymous	0	1
NOLC1	chr10	103917211/C//T	synonymous	0	1
ACTR1A	chr10	104244007/G//A	synonymous	0	1
TCF7L2	chr10	114912200/G//A	nonsynonymous	0	1
VAX1	chr10	118896030/G//A	nonsynonymous	0	1
RAB11FIP2	chr10	119768573/G//A	nonsynonymous	0	1
DOCK1	chr10	128830019/T//C	nonsynonymous	0	1
MUC6	chr11	1016713/T//C	nonsynonymous	0	1

MUC5B	chr11	1272623/T//C	nonsynonymous	0	1
MS4A3	chr11	59834472/G//C	nonsynonymous	0	1
BBS1	chr11	66287225/C//T	synonymous	0	1
MRE11	chr11	94192684/T//C	nonsynonymous	0	1
PDGFD	chr11	103797727/C//T	synonymous	0	1
DSCAML1	chr11	117651528/G//A	synonymous	0	1
AAAS	chr12	53703472/G//A	synonymous	0	1
CCDC92	chr12	124428910/T//A	synonymous	0	1
SLC46A3	chr13	29284912/G//A	nonsynonymous	0	1
ELF1	chr13	41507644/C//T	nonsynonymous	0	1
SLITRK6	chr13	86369306/G//A	synonymous	0	1
C14orf178	chr14	78235922/G//A	synonymous	0	1
TMEM121	chr14	105995632/C//T	nonsynonymous	0	1
GABRB3	chr15	26793096/C//T	synonymous	0	1
SPATA5L1	chr15	45694622/T//C	synonymous	0	1
KIF7	chr15	90176203/G//A	synonymous	0	1
ABCA3	chr16	2369616/C//T	nonsynonymous	0	1
ABCA3	chr16	2374411/C//T	synonymous	0	1
PPL	chr16	4935100/T//G	nonsynonymous	0	1
FTO	chr16	54145851/G//A	synonymous	0	1
FUK	chr16	70499064/C//G	nonsynonymous	0	1
ZFHX3	chr16	72830219/A//G	nonsynonymous	0	1
SYCE1L	chr16	77233354/G//A	synonymous	0	1
TP53	chr17	7579311/C//A	nonsynonymous	0	1
KDM6B	chr17	7749185/T//TCC	nonsynonymous	0	1
MPRIP	chr17	17041372/C//T	synonymous	0	1
CORO6	chr17	27946176/C//T	nonsynonymous	0	1
IFI35	chr17	41165164/G//A	nonsynonymous	0	1
	chr17	42987481/C//T	synonymous	0	1
CDC27	chr17	45234685/G//A	nonsynonymous	0	1
KPNB1	chr17	45754410/G//T	nonsynonymous	0	1
RPS6KB1	chr17	57970599/C//A	nonsynonymous	0	1
SMAD4	chr18	48591921/T//G	nonsynonymous	0	1
C2CD4C	chr19	407709/G//C	nonsynonymous	0	1
ELANE	chr19	855598/A//T	nonsynonymous	0	1
ZNF98	chr19	22586201/G//T	nonsynonymous	0	1
LRP3	chr19	33696821/A//G	nonsynonymous	0	1
SPIB	chr19	50925794/G//A	nonsynonymous	0	1
ZNF814	chr19	58384769/C//G	synonymous	0	1
CHGB	chr20	5903078/C//T	synonymous	0	1
NAA20	chr20	19998003/G//T	synonymous	0	1
EIF6	chr20	33867388/G//A	nonsynonymous	0	1
MC3R	chr20	54824062/G//C	nonsynonymous	0	1
MRPL39	chr21	26976123/T//A	synonymous	0	1
SON	chr21	34922321/C//T	nonsynonymous	0	1
FTCD	chr21	47571516/G//C	nonsynonymous	0	1
PRODH	chr22	18912607/G//A	synonymous	0	1
GAS2L1	chr22	29707795/A//G	nonsynonymous	0	1
ASCC2	chr22	30184940/C//T	synonymous	0	1
CSDC2	chr22	41968063/A//G	nonsynonymous	0	1
KLHL4	chrX	86868879/G//T	nonsynonymous	0	1
DCAF12L1	chrX	125685849/C//T	nonsynonymous	0	1
HS6ST2	chrX	131762287/G//C	synonymous	0	1
DFFB	chr1	3773968/C//T	synonymous	0	1
AIM1L	chr1	26648652/T//C	synonymous	0	1
AIM1L	chr1	26672434/T//C	nonsynonymous	0	1
IPP	chr1	46206798/A//C	nonsynonymous	0	1
LEPR	chr1	66101983/G//A	nonsynonymous	0	1
OTUD7B	chr1	149943040/A//G	synonymous	0	1
RORC	chr1	151787583/G//C	nonsynonymous	0	1
MSTO1	chr1	155581968/T//C	synonymous	0	1
MSTO1	chr1	155582074/C//T	synonymous	0	1
OR6K3	chr1	158687886/G//C	nonsynonymous	0	1
SLAMF7	chr1	160722899/G//A	nonsynonymous	0	1
	chr1	168161569/G//A	synonymous	0	1
CACNA1E	chr1	181767545/A//G	nonsynonymous	0	1
FH	chr1	241682991/G//C	nonsynonymous	0	1
RAD51AP2	chr2	17699418/ACGAGTCTGTGGAGT	nonsynonymous	0	1
FNDC4	chr2	27717530/T//C	nonsynonymous	0	1
GLI2	chr2	121746043/C//T	synonymous	0	1

TOP2B	chr3	25648812/T//G	nonsynonymous	0	1
SNRK	chr3	43390077/C//T	synonymous	0	1
TGM4	chr3	44952766/C//A	nonsynonymous	0	1
SLC9C1	chr3	111962844/A//G	nonsynonymous	0	1
PLA1A	chr3	119348280/A//G	nonsynonymous	0	1
DGKG	chr3	186006613/G//C	nonsynonymous	0	1
DDX60L	chr4	169383142/G//C	nonsynonymous	0	1
DNAH5	chr5	13792118/G//T	synonymous	0	1
IPO11	chr5	61785974/C//T	nonsynonymous	0	1
ERBIN	chr5	65374334/C//A	synonymous	0	1
DMXL1	chr5	118485294/A//T	nonsynonymous	0	1
SNX2	chr5	122130970/T//G	nonsynonymous	0	1
ADAMTS19	chr5	129019821/G//A	synonymous	0	1
KDM3B	chr5	137753171/G//A	nonsynonymous	0	1
CSF1R	chr5	149459893/G//A	nonsynonymous	0	1
GEMIN5	chr5	154317583/G//A	synonymous	0	1
WWC1	chr5	167841359/C//A	synonymous	0	1
BTNL3	chr5	180424438/T//C	nonsynonymous	0	1
CNPY3-GNMT	chr6	42897355/C//G	nonsynonymous	0	1
ADGRF5	chr6	46821794/A//T	nonsynonymous	0	1
CD2AP	chr6	47471039/T//A	nonsynonymous	0	1
ADGRB3	chr6	70071139/A//G	nonsynonymous	0	1
CARD11	chr7	2953083/C//T	nonsynonymous	0	1
ZNF679	chr7	63727011/A//C	nonsynonymous	0	1
PTPRZ1	chr7	121650586/C//A	nonsynonymous	0	1
AKR1B10	chr7	134223693/G//T	nonsynonymous	0	1
PREX2	chr8	69058521/G//T	nonsynonymous	0	1
NCALD	chr8	102705045/A//C	nonsynonymous	0	1
JRK	chr8	143740372/T//G	synonymous	0	1
HACD4	chr9	21026716/A//G	nonsynonymous	0	1
CTNNA3	chr10	68940172/G//A	nonsynonymous	0	1
RRM1	chr11	4141165/T//C	synonymous	0	1
HSD17B12	chr11	43775675/A//C	synonymous	0	1
KCNK4	chr11	64065584/G//A	nonsynonymous	0	1
FAT3	chr11	92534407/T//C	nonsynonymous	0	1
DDX10	chr11	108547863/G//A	nonsynonymous	0	1
OR6X1	chr11	123624395/T//C	nonsynonymous	0	1
GPD1	chr12	50501525/C//T	nonsynonymous	0	1
TRHDE	chr12	72863615/C//G	nonsynonymous	0	1
TMEM132B	chr12	125811211/C//T	synonymous	0	1
SACS	chr13	23914023/T//A	nonsynonymous	0	1
MRPL52	chr14	23299422/G//A	nonsynonymous	0	1
MOK	chr14	102695928/G//C	nonsynonymous	0	1
KBTBD13	chr15	65369489/G//A	synonymous	0	1
ALPK3	chr15	85403072/A//G	nonsynonymous	0	1
TNRC6A	chr16	24802997/A//G	nonsynonymous	0	1
PRSS8	chr16	31143475/G//T	nonsynonymous	0	1
WDR59	chr16	74985432/G//A	nonsynonymous	0	1
TTLL6	chr17	46882309/G//A	nonsynonymous	0	1
ASXL3	chr18	31324205/A//G	nonsynonymous	0	1
MC4R	chr18	58038661/C//A	nonsynonymous	0	1
ATP5D	chr19	1244502/C//G	synonymous	0	1
CACTIN	chr19	3612034/T//C	nonsynonymous	0	1
ZNF266	chr19	9529218/A//G	synonymous	0	1
ZNF560	chr19	9578136/A//G	nonsynonymous	0	1
RYR1	chr19	38946168/C//T	nonsynonymous	0	1
HNRNPL	chr19	39334645/T//C	synonymous	0	1
CEACAM1	chr19	43023292/C//T	nonsynonymous	0	1
LILRB2	chr19	54783839/T//C	synonymous	0	1
ZNF543	chr19	57840365/G//A	nonsynonymous	0	1
ZNF335	chr20	44589259/T//C	nonsynonymous	0	1
BCAS1	chr20	52570096/C//T	nonsynonymous	0	1
APP	chr21	27372459/C//T	nonsynonymous	0	1
WRB-SH3BGR	chr21	40762768/A//G	nonsynonymous	0	1
DSCAM	chr21	41459142/T//C	nonsynonymous	0	1
PI4KA	chr22	21159452/G//C	nonsynonymous	0	1
TCF20	chr22	42610563/G//A	nonsynonymous	0	1
PRR5	chr22	45130952/G//A	nonsynonymous	0	1
PLXNB2	chr22	50727463/T//C	nonsynonymous	0	1
PLXNB2	chr22	50727469/T//C	nonsynonymous	0	1

GCNA	chrX	70832706/T//C	synonymous	0	1
CHD5	chr1	6203938/C//A	nonsynonymous	0	1
HIVEP3	chr1	41976299/C//T	synonymous	0	1
HIVEP3	chr1	42048697/G//A	nonsynonymous	0	1
DOCK7	chr1	62940924/C//T	nonsynonymous	0	1
GON4L	chr1	155823384/G//A	nonsynonymous	0	1
OR10Z1	chr1	158576251/C//T	nonsynonymous	0	1
FMO1	chr1	171251173/G//A	nonsynonymous	0	1
LBX2-AS1	chr2	74729977/T//C	synonymous	0	1
ACMSD	chr2	135621089/C//T	nonsynonymous	0	1
TTN	chr2	179455089/C//A	nonsynonymous	0	1
ECT2	chr3	172534570/T//C	synonymous	0	1
TBCK	chr4	107037433/A//G	nonsynonymous	0	1
TBCK	chr4	107157555/G//A	synonymous	0	1
DCHS2	chr4	155191083/T//C	synonymous	0	1
SDHA	chr5	236582/G//A	nonsynonymous	0	1
DMGDH	chr5	78328642/C//T	nonsynonymous	0	1
TRIM38	chr6	25983369/GTTTGT//G	synonymous	0	1
BACH2	chr6	90661231/G//A	synonymous	0	1
PCLO	chr7	82475921/T//C	nonsynonymous	0	1
OPN1SW	chr7	128414653/G//A	nonsynonymous	0	1
KIF27	chr9	86518279/C//T	nonsynonymous	0	1
FBXW5	chr9	139835562/G//A	synonymous	0	1
PLXDC2	chr10	20335806/A//T	synonymous	0	1
ZNF37A	chr10	38407653/G//T	nonsynonymous	0	1
PCDH15	chr10	55973710/C//A	nonsynonymous	0	1
HECTD2	chr10	93238009/C//T	nonsynonymous	0	1
MKI67	chr10	129906964/G//A	nonsynonymous	0	1
BIRC2	chr11	102248928/A//G	synonymous	0	1
CRTAM	chr11	122735343/G//A	nonsynonymous	0	1
OR6C4	chr12	55945557/G//A	nonsynonymous	0	1
CEMIP	chr15	81221441/G//T	synonymous	0	1
ITGAD	chr16	31434461/A//G	nonsynonymous	0	1
NOD2	chr16	50745219/C//T	nonsynonymous	0	1
PLLP	chr16	57295959/C//T	synonymous	0	1
ZZEF1	chr17	3919723/A//G	nonsynonymous	0	1
VTN	chr17	26694933/C//T	nonsynonymous	0	1
	chr17	30536468/A//C	synonymous	0	1
CSH1	chr17	61972886/C//T	nonsynonymous	0	1
CYGB	chr17	74524594/G//T	synonymous	0	1
DLGAP1	chr18	3499087/A//G	synonymous	0	1
PHLPP1	chr18	60630622/T//G	nonsynonymous	0	1
ZNF414	chr19	8576744/G//A	nonsynonymous	0	1
RGL3	chr19	11505038/G//A	synonymous	0	1
ZNF676	chr19	22364007/C//T	nonsynonymous	0	1
ZNF404	chr19	44377857/A//G	nonsynonymous	0	1
LILRB4	chr19	55174509/G//T	synonymous	0	1
PPP1R12C	chr19	55603035/G//A	synonymous	0	1
KDM6A	chrX	44942840/G//A	synonymous	0	1
ERAS	chrX	48688034/A//T	synonymous	0	1
TIMM17B	chrX	48751030/G//A	synonymous	0	1
CSMD2	chr1	34158614/A//G	nonsynonymous	0	1
FHL3	chr1	38463188/C//T	nonsynonymous	0	1
DNM3	chr1	172007462/C//G	nonsynonymous	0	1
MRPS22	chr3	139069035/C//T	synonymous	0	1
ADAMTS2	chr5	178562922/G//A	synonymous	0	1
POM121L2	chr6	27278501/C//T	synonymous	0	1
HLA-DQA1	chr6	32610495/A//G	nonsynonymous	0	1
CALCR	chr7	93116297/T//G	synonymous	0	1
CPED1	chr7	120655759/G//A	nonsynonymous	0	1
POT1	chr7	124464095/G//C	nonsynonymous	0	1
NPBWR1	chr8	53852834/A//G	nonsynonymous	0	1
OR5B2	chr11	58189875/A//G	nonsynonymous	0	1
STX5	chr11	62592546/G//T	nonsynonymous	0	1
PCNX3	chr11	65404148/C//T	synonymous	0	1
PPME1	chr11	73957239/T//C	nonsynonymous	0	1
SCAF11	chr12	46342214/A//G	synonymous	0	1
GGACT	chr13	101184483/C//T	synonymous	0	1
FBN1	chr15	48807622/T//C	nonsynonymous	0	1
UNKL	chr16	1451571/T//C	nonsynonymous	0	1

TMC7	chr16	19051704/T//C	nonsynonymous	0	1
POTEC	chr18	14537949/A//G	nonsynonymous	0	1
SRRM5	chr19	44118129/G//A	nonsynonymous	0	1
FTL	chr19	49470013/C//T	synonymous	0	1
ZNF135	chr19	58579349/C//T	synonymous	0	1
PADI2	chr1	17409099/C//T	nonsynonymous	0	1
PAX7	chr1	19027245/C//T	synonymous	0	1
FCRL4	chr1	157559050/C//T	nonsynonymous	0	1
F13B	chr1	197031010/C//T	nonsynonymous	0	1
TMEM183A	chr1	202984027/C//G	nonsynonymous	0	1
NFASC	chr1	204966527/C//T	synonymous	0	1
OR2L3	chr1	248224569/G//T	nonsynonymous	0	1
OR2L3	chr1	248224595/C//T	synonymous	0	1
ZNF513	chr2	27601830/A//T	synonymous	0	1
SEMA4C	chr2	97527309/A//G	synonymous	0	1
TTN	chr2	179606148/G//C	nonsynonymous	0	1
IL17RD	chr3	57199319/G//A	synonymous	0	1
PHLDB2	chr3	111659406/C//A	nonsynonymous	0	1
PIK3CB	chr3	138374293/C//T	nonsynonymous	0	1
IQGAP2	chr5	75757409/G//T	nonsynonymous	0	1
EFNA5	chr5	106763129/G//A	synonymous	0	1
HSD17B4	chr5	118813117/A//G	nonsynonymous	0	1
PEX6	chr6	42946321/T//C	nonsynonymous	0	1
GCLC	chr6	53409465/C//CG	synonymous	0	1
GCLC	chr6	53409465/C//CTCCTCCG	synonymous	0	1
HTR1B	chr6	78172545/G//A	synonymous	0	1
CSMD1	chr8	3057290/G//A	nonsynonymous	0	1
CIZ1	chr9	130942710/C//T	nonsynonymous	0	1
PELI3	chr11	66239923/C//T	synonymous	0	1
RAB38	chr11	87882860/A//G	nonsynonymous	0	1
MTUS2	chr13	29933457/G//A	synonymous	0	1
CMA1	chr14	24974805/G//A	nonsynonymous	0	1
THBS1	chr15	39876364/G//A	synonymous	0	1
SLX4	chr16	3639710/A//T	nonsynonymous	0	1
NRN1L	chr16	67919762/C//T	synonymous	0	1
ZNF469	chr16	88496341/C//T	synonymous	0	1
OR1A1	chr17	3119050/G//A	nonsynonymous	0	1
MC5R	chr18	13826508/C//T	synonymous	0	1
BCAM	chr19	45322885/C//T	synonymous	0	1
ZNF880	chr19	52887265/A//G	synonymous	0	1
NLRP8	chr19	56466873/A//G	synonymous	0	1
DDRGK1	chr20	3171273/CTG//C	synonymous	0	1
MFAP2	chr1	17303651/G//T	nonsynonymous	0	1
SYTL1	chr1	27676154/G//A	synonymous	0	1
PATJ	chr1	62299378/C//T	nonsynonymous	0	1
THSD7B	chr2	137814196/C//T	nonsynonymous	0	1
EVX2	chr2	176945373/G//A	nonsynonymous	0	1
TTN	chr2	179603090/G//T	synonymous	0	1
PAK2	chr3	196509683/A//G	nonsynonymous	0	1
COL10A1	chr6	116446581/G//A	synonymous	0	1
GLI3	chr7	42005498/C//T	nonsynonymous	0	1
SLC7A2	chr8	17422715/G//A	synonymous	0	1
MROH5	chr8	142480822/G//A	nonsynonymous	0	1
SCRIB	chr8	144885992/C//T	nonsynonymous	0	1
SLC39A4	chr8	145641986/C//T	nonsynonymous	0	1
MPDZ	chr9	13168554/A//G	nonsynonymous	0	1
DNAJA1	chr9	33026899/A//G	nonsynonymous	0	1
SLC25A51	chr9	37888320/T//G	synonymous	0	1
GSN	chr9	124091200/T//G	synonymous	0	1
CERS5	chr12	50537804/G//A	synonymous	0	1
LARP4	chr12	50834237/T//C	nonsynonymous	0	1
BAZ2A	chr12	56996496/C//T	nonsynonymous	0	1
C15orf32	chr15	93015692/C//A	nonsynonymous	0	1
ABAT	chr16	8875199/T//C	nonsynonymous	0	1
ITGAD	chr16	31426282/C//T	synonymous	0	1
PKD1L2	chr16	81198302/C//T	nonsynonymous	0	1
NFKBID	chr19	36379425/G//A	synonymous	0	1
RNF225	chr19	58908419/C//T	synonymous	0	1
GABRA3	chrX	151336737/C//T	nonsynonymous	0	1
AHDC1	chr1	27875122/C//A	nonsynonymous	0	1

LYST	chr1	235840823/T//C	nonsynonymous	0	1
TTN	chr2	179479268/C//T	nonsynonymous	0	1
ATG9A	chr2	220089279/C//T	nonsynonymous	0	1
ESPNL	chr2	239039325/G//C	nonsynonymous	0	1
GRIP2	chr3	14566940/T//C	synonymous	0	1
KALRN	chr3	124193514/T//A	nonsynonymous	0	1
AFF1	chr4	88052234/G//C	nonsynonymous	0	1
FAM134B	chr5	16475010/T//C	nonsynonymous	0	1
BASP1	chr5	17275900/C//T	nonsynonymous	0	1
LIFR	chr5	38530608/C//T	nonsynonymous	0	1
UBE3D	chr6	83763916/ATTCT//A	nonsynonymous	0	1
TMEM106B	chr7	12263950/G//C	nonsynonymous	0	1
ZNF679	chr7	63726759/A//G	nonsynonymous	0	1
PVRIG	chr7	99818921/G//A	synonymous	0	1
GRM8	chr7	126173489/G//A	synonymous	0	1
C7orf49	chr7	134851405/T//TTCC	nonsynonymous	0	1
ZNF746	chr7	149171807/C//T	nonsynonymous	0	1
DCAF4L2	chr8	88885717/C//T	synonymous	0	1
ZHX1	chr8	124265919/C//T	synonymous	0	1
SEC24C	chr10	75520133/C//G	nonsynonymous	0	1
TIAL1	chr10	121342039/C//T	nonsynonymous	0	1
ANKRD42	chr11	82947521/C//T	nonsynonymous	0	1
ALG10B	chr12	38714040/T//G	synonymous	0	1
MYO16	chr13	109535426/G//T	nonsynonymous	0	1
DHRS4	chr14	24438039/G//A	nonsynonymous	0	1
RPS29	chr14	50053002/C//T	nonsynonymous	0	1
NOXRED1	chr14	77860955/T//C	synonymous	0	1
HERC2	chr15	28459114/T//C	nonsynonymous	0	1
HERC2	chr15	28483904/C//CAA	nonsynonymous	0	1
MYH10	chr17	8413169/C//A	nonsynonymous	0	1
MYH13	chr17	10258282/A//C	nonsynonymous	0	1
ABCA9	chr17	66981227/G//A	synonymous	0	1
ALKBH7	chr19	6372996/G//T	nonsynonymous	0	1
JADE3	chrX	46887268/G//GATTGATTGTTTGT	nonsynonymous	0	1
DIAPH2	chrX	96369753/G//A	nonsynonymous	0	1
PLEKHG5	chr1	6533322/C//T	nonsynonymous	0	1
SPOCD1	chr1	32256897/G//A	synonymous	0	1
MSH4	chr1	76363656/T//C	nonsynonymous	0	1
ADGRL4	chr1	79402092/G//C	synonymous	0	1
DENND4B	chr1	153916529/C//T	synonymous	0	1
OR6K2	chr1	158670091/C//T	nonsynonymous	0	1
CD244	chr1	160832428/G//A	nonsynonymous	0	1
SLC19A2	chr1	169437963/T//C	nonsynonymous	0	1
NAV1	chr1	201752850/A//G	nonsynonymous	0	1
OR2C3	chr1	247694809/C//T	synonymous	0	1
USP34	chr2	61528083/C//A	synonymous	0	1
LRRTM4	chr2	77745564/C//A	nonsynonymous	0	1
WIPF1	chr2	175436483/C//T	synonymous	0	1
TTN	chr2	179486606/C//G	nonsynonymous	0	1
ADAM23	chr2	207395676/C//T	synonymous	0	1
ERBB4	chr2	212812147/T//A	synonymous	0	1
ABCA12	chr2	215823114/C//T	nonsynonymous	0	1
IRS1	chr2	227661675/G//A	nonsynonymous	0	1
CHL1	chr3	440023/G//A	nonsynonymous	0	1
TCAIM	chr3	44438259/G//A	nonsynonymous	0	1
TRIM42	chr3	140401368/G//T	nonsynonymous	0	1
ZIC1	chr3	147131293/C//T	synonymous	0	1
KIT	chr4	55593430/C//T	synonymous	0	1
TENM3	chr4	183710311/C//T	synonymous	0	1
SSBP2	chr5	80736382/G//A	nonsynonymous	0	1
SLC25A2	chr5	140683054/T//A	nonsynonymous	0	1
N4BP3	chr5	177547328/C//T	synonymous	0	1
IP6K3	chr6	33703227/G//A	synonymous	0	1
ZNF318	chr6	43322670/C//T	nonsynonymous	0	1
CYP39A1	chr6	46593234/C//T	nonsynonymous	0	1
DEFB112	chr6	50011452/G//A	nonsynonymous	0	1
SYNE1	chr6	152605182/C//T	synonymous	0	1
NOX3	chr6	155750136/G//A	nonsynonymous	0	1
ASB4	chr7	95157246/C//T	synonymous	0	1
NCAPG2	chr7	158439151/C//T	nonsynonymous	0	1

BLK	chr8	11400849/C//T	nonsynonymous	0	1
PREX2	chr8	69129868/C//T	nonsynonymous	0	1
TAF1L	chr9	32634324/G//A	synonymous	0	1
FAM13C	chr10	61022280/T//C	nonsynonymous	0	1
EGR2	chr10	64573638/C//T	nonsynonymous	0	1
MUC5B	chr11	1274132/G//A	nonsynonymous	0	1
OR4S2	chr11	55419115/G//A	nonsynonymous	0	1
TCIRG1	chr11	67812576/G//A	synonymous	0	1
ABCD2	chr12	39947887/G//A	nonsynonymous	0	1
NEMP1	chr12	57463069/G//T	nonsynonymous	0	1
TMEM132C	chr12	128899885/G//A	nonsynonymous	0	1
HERC1	chr15	63990951/C//T	nonsynonymous	0	1
SIAH1	chr16	48395513/T//C	nonsynonymous	0	1
CKLF-CMTM1	chr16	66612775/G//A	synonymous	0	1
SP2	chr17	45973596/G//T	synonymous	0	1
DNAH17	chr17	76472727/T//C	nonsynonymous	0	1
SYT4	chr18	40857219/C//T	nonsynonymous	0	1
PDE4A	chr19	10559813/G//A	nonsynonymous	0	1
ZSWIM4	chr19	13941909/G//A	synonymous	0	1
LILRA1	chr19	55106722/C//T	synonymous	0	1
NCAM2	chr21	22782747/A//T	nonsynonymous	0	1
TIAM1	chr21	32624335/C//T	synonymous	0	1
MID1	chrX	10535293/C//T	nonsynonymous	0	1
HS6ST2	chrX	132092561/G//A	nonsynonymous	0	1
MAGEC2	chrX	141290955/G//C	synonymous	0	1
AFF2	chrX	148039947/C//A	nonsynonymous	0	1
MAGEA8	chrX	149013066/G//T	nonsynonymous	0	1
CNGA2	chrX	150912504/A//G	nonsynonymous	0	1
ZNF185	chrX	152097142/G//A	nonsynonymous	0	1
INPP5B	chr1	38348496/G//T	nonsynonymous	0	1
LYSMD1	chr1	151134475/G//T	nonsynonymous	0	1
GLI2	chr2	121746594/T//C	nonsynonymous	0	1
TTN	chr2	179430120/C//A	nonsynonymous	0	1
ZNF717	chr3	75786042/G//A	nonsynonymous	0	1
ZNF717	chr3	75786059/C//T	synonymous	0	1
ZNF717	chr3	75786071/C//A	nonsynonymous	0	1
PI4K2B	chr4	25256710/C//A	synonymous	0	1
TBC1D19	chr4	26675480/T//C	synonymous	0	1
ANK2	chr4	114278787/G//A	nonsynonymous	0	1
PPP2CA	chr5	133536123/C//T	nonsynonymous	0	1
PCDHB13	chr5	140595360/C//T	synonymous	0	1
ITK	chr5	156679654/G//A	nonsynonymous	0	1
FAF2	chr5	175934053/ATAT//A	synonymous	0	1
COL11A2	chr6	33136485/G//T	nonsynonymous	0	1
TRMT11	chr6	126307689/C//T	synonymous	0	1
MAD1L1	chr7	2041758/T//C	nonsynonymous	0	1
ACTL6B	chr7	100253425/C//G	nonsynonymous	0	1
EPHB6	chr7	142561875/G//T	nonsynonymous	0	1
TRPV6	chr7	142569542/C//T	nonsynonymous	0	1
POTEA	chr8	43152158/C//A	synonymous	0	1
KLHL38	chr8	124664843/C//T	nonsynonymous	0	1
TAF5	chr10	105145255/C//T	synonymous	0	1
MYOD1	chr11	17742502/C//T	synonymous	0	1
TMEM151A	chr11	66062950/C//T	synonymous	0	1
BBS1	chr11	66283410/A//G	synonymous	0	1
TECTA	chr11	120989027/G//A	nonsynonymous	0	1
DPY19L2	chr12	63974603/A//G	nonsynonymous	0	1
PPP1CC	chr12	111157895/T//C	synonymous	0	1
PARP4	chr13	25033444/G//A	nonsynonymous	0	1
SAMD4A	chr14	55241822/CT//C	nonsynonymous	0	1
RGMA	chr15	93588783/T//C	synonymous	0	1
BCL7C	chr16	30903974/C//T	synonymous	0	1
CCDC40	chr17	78071113/C//A	nonsynonymous	0	1
MUC16	chr19	9033248/G//T	nonsynonymous	0	1
KLK7	chr19	51487126/A//G	synonymous	0	1
ZFP28	chr19	57061890/G//A	nonsynonymous	0	1
KIF16B	chr20	16492165/C//A	nonsynonymous	0	1
TPTE	chr21	10908896/C//A	nonsynonymous	0	1
RPS6KA6	chrX	83357077/G//A	nonsynonymous	0	1
ARMCX4	chrX	100743633/C//A	synonymous	0	1

CELA3B	chr1	22313034/G//C	nonsynonymous	0	1
KDM4A	chr1	44133562/G//A	synonymous	0	1
CC2D1B	chr1	52820607/A//G	synonymous	0	1
UCK2	chr1	165865424/C//T	synonymous	0	1
MAP4K4	chr2	102450875/A//G	nonsynonymous	0	1
RHBDD1	chr2	227729455/C//A	nonsynonymous	0	1
ZIC1	chr3	147128358/C//T	synonymous	0	1
PTPN13	chr4	87655539/A//G	nonsynonymous	0	1
MANBA	chr4	103585980/G//T	synonymous	0	1
RXFP1	chr4	159569665/G//A	nonsynonymous	0	1
PRDM9	chr5	23524440/C//A	synonymous	0	1
MUC22	chr6	30995078/G//A	nonsynonymous	0	1
MUC22	chr6	30995080/G//C	nonsynonymous	0	1
NOTCH4	chr6	32169865/G//A	nonsynonymous	0	1
EYS	chr6	65622601/C//A	nonsynonymous	0	1
WASF1	chr6	110434573/C//T	nonsynonymous	0	1
CCR6	chr6	167550167/C//T	nonsynonymous	0	1
BAZ1B	chr7	72891640/A//C	synonymous	0	1
CSMD3	chr8	113332217/A//C	synonymous	0	1
NDOR1	chr9	140109288/A//G	nonsynonymous	0	1
OR52L1	chr11	6007523/T//A	nonsynonymous	0	1
OR5AN1	chr11	59132672/A//G	synonymous	0	1
GDPD5	chr11	75146518/G//C	synonymous	0	1
IQSEC3	chr12	247927/C//T	synonymous	0	1
MBD6	chr12	57919636/G//A	nonsynonymous	0	1
NAA25	chr12	112513538/C//T	synonymous	0	1
SERTM1	chr13	37269146/C//A	synonymous	0	1
MYH6	chr14	23853924/G//A	synonymous	0	1
TGM7	chr15	43579514/C//T	nonsynonymous	0	1
IGFALS	chr16	1840942/G//T	nonsynonymous	0	1
ACSM1	chr16	20651826/C//T	nonsynonymous	0	1
EEF2K	chr16	22291519/G//C	nonsynonymous	0	1
FANCA	chr16	89883001/T//C	nonsynonymous	0	1
GRB7	chr17	37900456/C//G	nonsynonymous	0	1
DNAH17	chr17	76533466/A//G	nonsynonymous	0	1
RPTOR	chr17	78858924/C//T	synonymous	0	1
ELAC1	chr18	48513179/G//A	synonymous	0	1
ZNF358	chr19	7585709/T//C	synonymous	0	1
IL12RB1	chr19	18179307/C//G	nonsynonymous	0	1
SAMSN1	chr21	15858259/T//A	nonsynonymous	0	1
DEPDC5	chr22	32302495/A//G	synonymous	0	1
EFCAB6	chr22	43933328/C//T	nonsynonymous	0	1
FAM9B	chrX	9001022/C//T	synonymous	0	1
PABPC5	chrX	90690776/G//T	nonsynonymous	0	1
PRAMEF1	chr1	12854530/C//G	nonsynonymous	0	1
MTHFD2	chr2	74441262/A//G	nonsynonymous	0	1
POTEE	chr2	131976292/T//C	nonsynonymous	0	1
NMI	chr2	152132156/A//G	nonsynonymous	0	1
PMS1	chr2	190719819/A//G	synonymous	0	1
WDR17	chr4	177046439/C//A	synonymous	0	1
SEMA6A	chr5	115782419/G//A	synonymous	0	1
PCDHA10	chr5	140236219/C//T	nonsynonymous	0	1
SH3RF2	chr5	145439782/G//A	nonsynonymous	0	1
NFKBIE	chr6	44233189/G//A	synonymous	0	1
PAQR8	chr6	52268020/C//T	synonymous	0	1
FAM184A	chr6	119300649/G//T	nonsynonymous	0	1
PRKAR1B	chr7	720200/A//G	nonsynonymous	0	1
PTPRZ1	chr7	121679583/C//A	nonsynonymous	0	1
SLC25A37	chr8	23429097/C//A	nonsynonymous	0	1
SLC18A2	chr10	119013956/G//T	nonsynonymous	0	1
OR5P3	chr11	7846745/A//G	nonsynonymous	0	1
ADCY6	chr12	49164680/C//T	nonsynonymous	0	1
COQ5	chr12	120960166/A//G	nonsynonymous	0	1
BIVM-ERCC5	chr13	103527666/C//T	nonsynonymous	0	1
PPP2R3C	chr14	35585829/C//T	nonsynonymous	0	1
CORO6	chr17	27944639/T//C	nonsynonymous	0	1
ZNF135	chr19	58570679/G//A	nonsynonymous	0	1
BAGE3	chr21	11058248/G//A	synonymous	0	1
SEC14L2	chr22	30803125/T//C	synonymous	0	1
CCNB3	chrX	50094622/G//A	nonsynonymous	0	1

ALG13	chrX	110971470/C//T	nonsynonymous	0	1
ZNF185	chrX	152090804/A//G	synonymous	0	1
DPYD	chr1	97544644/C//T	nonsynonymous	0	1
RGS4	chr1	163039301/G//A	synonymous	0	1
MYOC	chr1	171621174/C//T	nonsynonymous	0	1
WNT10A	chr2	219747043/G//A	nonsynonymous	0	1
ILKAP	chr2	239093880/T//C	synonymous	0	1
OSBPL10	chr3	31921235/A//C	synonymous	0	1
ZNF197	chr3	44688028/G//A	synonymous	0	1
CC2D2A	chr4	15599051/C//T	nonsynonymous	0	1
ANK2	chr4	114279295/C//T	nonsynonymous	0	1
ANKRD31	chr5	74400466/C//T	nonsynonymous	0	1
APC	chr5	112176020/GA//G	nonsynonymous	0	1
PCDHA6	chr5	140207609/C//T	synonymous	0	1
PRRC2A	chr6	31601740/C//T	nonsynonymous	0	1
ADGRB3	chr6	70071251/C//G	nonsynonymous	0	1
GRIK2	chr6	102516279/C//T	nonsynonymous	0	1
LAMA2	chr6	129704345/G//T	nonsynonymous	0	1
SYNE1	chr6	152603121/G//C	synonymous	0	1
SYNE1	chr6	152861133/G//A	nonsynonymous	0	1
LAMB1	chr7	107600203/G//A	synonymous	0	1
SOX17	chr8	55370863/G//A	synonymous	0	1
MOB3B	chr9	27455594/C//T	synonymous	0	1
PCSK5	chr9	78547404/G//GA	synonymous	0	1
KCNT1	chr9	138660705/G//A	nonsynonymous	0	1
RBMXL2	chr11	7110849/G//A	synonymous	0	1
AGBL2	chr11	47698916/C//T	nonsynonymous	0	1
CWC15	chr11	94699549/C//T	nonsynonymous	0	1
OR10S1	chr11	123847954/C//T	nonsynonymous	0	1
ACTR10	chr14	58690341/G//A	synonymous	0	1
SIX6	chr14	60976625/C//T	nonsynonymous	0	1
FAM227B	chr15	49620866/C//T	nonsynonymous	0	1
CNTNAP4	chr16	76311635/G//A	synonymous	0	1
CSH1	chr17	61973942/T//A	synonymous	0	1
WDR7	chr18	54444034/C//T	nonsynonymous	0	1
ZNF20	chr19	12244616/C//T	nonsynonymous	0	1
CYP4F12	chr19	15807894/G//T	nonsynonymous	0	1
SYNJ1	chr21	34018017/C//T	synonymous	0	1
TSPEAR	chr21	45919566/G//T	synonymous	0	1
OTUD5	chrX	48814388/T//G	nonsynonymous	0	1
RPS6KA6	chrX	83403107/C//T	nonsynonymous	0	1
MAP7D3	chrX	135323365/T//C	synonymous	0	1
VPS13D	chr1	12353595/A//G	nonsynonymous	0	1
RBM44	chr2	238726845/C//T	nonsynonymous	0	1
DAG1	chr3	49569331/A//G	nonsynonymous	0	1
CC2D2A	chr4	15538699/A//G	synonymous	0	1
RSPH3	chr6	159403579/T//C	nonsynonymous	0	1
EPHB6	chr7	142568064/G//A	nonsynonymous	0	1
MBOAT4	chr8	29996089/C//T	synonymous	0	1
GHITM	chr10	85909880/A//G	nonsynonymous	0	1
TCIRG1	chr11	67817215/G//A	nonsynonymous	0	1
AVIL	chr12	58203626/C//T	nonsynonymous	0	1
MGA	chr15	42000392/C//T	nonsynonymous	0	1
ANKDD1A	chr15	65242165/C//T	synonymous	0	1
ITGAE	chr17	3620035/C//T	nonsynonymous	0	1
LRRC37A3	chr17	62893331/C//T	synonymous	0	1
ZNF91	chr19	23543263/C//T	nonsynonymous	0	1
ZNF528	chr19	52919325/G//A	nonsynonymous	0	1
TMEM255A	chrX	119419077/G//T	synonymous	0	1
LRRC38	chr1	13802561/T//C	nonsynonymous	0	1
MUTYH	chr1	45799093/T//C	nonsynonymous	0	1
MYSM1	chr1	59147985/T//G	nonsynonymous	0	1
GBP5	chr1	89729430/T//A	nonsynonymous	0	1
RNPC3	chr1	104094349/G//C	nonsynonymous	0	1
NBPF8	chr1	144621658/T//G	nonsynonymous	0	1
LCE2C	chr1	152648569/T//A	synonymous	0	1
SCAMP3	chr1	155228743/G//A	nonsynonymous	0	1
OR6N1	chr1	158736147/G//A	nonsynonymous	0	1
POGK	chr1	166819586/G//T	nonsynonymous	0	1
KCNT2	chr1	196250113/T//C	synonymous	0	1

C1orf116	chr1	207196244/G//A	nonsynonymous	0	1
TMEM131	chr2	98413855/C//T	nonsynonymous	0	1
IL1RL1	chr2	102968245/G//T	nonsynonymous	0	1
EVX2	chr2	176948176/G//T	nonsynonymous	0	1
CARF	chr2	203836232/T//C	nonsynonymous	0	1
MAP2	chr2	210561765/G//A	synonymous	0	1
SP140	chr2	231120238/C//T	nonsynonymous	0	1
EOMES	chr3	27763427/G//C	nonsynonymous	0	1
FAM19A1	chr3	68466564/G//A	nonsynonymous	0	1
HAUS3	chr4	2241787/C//G	nonsynonymous	0	1
FREM3	chr4	144617847/G//A	nonsynonymous	0	1
GLRA3	chr4	175565216/C//T	nonsynonymous	0	1
FAT1	chr4	187540068/TTTCTCGATCAAG//T	nonsynonymous	0	1
FRG2	chr4	190948352/T//A	nonsynonymous	0	1
RXFP3	chr5	33937865/G//T	synonymous	0	1
SCAMP1	chr5	77656534/T//C	synonymous	0	1
HSD17B4	chr5	118829509/C//G	synonymous	0	1
RAD50	chr5	131915051/T//G	nonsynonymous	0	1
PCDHGA6	chr5	140754276/G//A	nonsynonymous	0	1
MGAT1	chr5	180218699/G//A	nonsynonymous	0	1
SYCP2L	chr6	10959016/G//C	nonsynonymous	0	1
SCGN	chr6	25701495/G//A	nonsynonymous	0	1
ZKSCAN8	chr6	28121893/C//A	synonymous	0	1
CDC5L	chr6	44394293/C//G	nonsynonymous	0	1
ENPP5	chr6	46135255/A//G	synonymous	0	1
RSPH4A	chr6	116943973/GCGTC//G	nonsynonymous	0	1
CARD11	chr7	2962266/A//T	nonsynonymous	0	1
TYW1	chr7	66482898/G//A	nonsynonymous	0	1
AUTS2	chr7	69064955/G//A	synonymous	0	1
MUC17	chr7	100680299/G//C	nonsynonymous	0	1
MUC17	chr7	100680313/A//G	nonsynonymous	0	1
TRIM56	chr7	100732173/A//C	nonsynonymous	0	1
HBP1	chr7	106829753/G//C	nonsynonymous	0	1
KCNH2	chr7	150648736/C//T	nonsynonymous	0	1
HEY1	chr8	80677647/C//A	nonsynonymous	0	1
RALYL	chr8	85441637/T//C	synonymous	0	1
RGS22	chr8	100977666/T//G	synonymous	0	1
PKHD1L1	chr8	110456029/T//C	synonymous	0	1
FRMPD1	chr9	37746153/C//T	nonsynonymous	0	1
AKR1C4	chr10	5254670/C//A	nonsynonymous	0	1
MORN4	chr10	99379362/G//A	nonsynonymous	0	1
GPR26	chr10	125426542/G//A	nonsynonymous	0	1
PKP3	chr11	400618/G//A	synonymous	0	1
GALNT18	chr11	11400819/G//T	synonymous	0	1
LUZP2	chr11	24750780/G//A	nonsynonymous	0	1
FLRT1	chr11	63885482/C//T	synonymous	0	1
RAD9A	chr11	67164800/C//T	synonymous	0	1
DDI1	chr11	103907619/C//T	synonymous	0	1
FAR2	chr12	29469913/C//G	nonsynonymous	0	1
LRRK2	chr12	40702925/A//G	nonsynonymous	0	1
FAM186A	chr12	50749751/A//G	synonymous	0	1
OR9K2	chr12	55524558/T//C	nonsynonymous	0	1
TPH2	chr12	72425445/A//C	nonsynonymous	0	1
OTOGL	chr12	80615924/C//A	nonsynonymous	0	1
PLXNC1	chr12	94603431/G//C	nonsynonymous	0	1
SLC5A8	chr12	101598313/G//A	nonsynonymous	0	1
GOLGA3	chr12	133349592/A//T	synonymous	0	1
PCDH8	chr13	53421105/G//A	synonymous	0	1
PCDH17	chr13	58208780/C//G	nonsynonymous	0	1
ITGBL1	chr13	102227855/G//A	nonsynonymous	0	1
IRF2BPL	chr14	77492342/C//T	synonymous	0	1
WDR24	chr16	737323/G//A	synonymous	0	1
	chr16	28884515/C//T	synonymous	0	1
CDH11	chr16	65022055/C//A	synonymous	0	1
SLC22A31	chr16	89265448/G//T	synonymous	0	1
MYH13	chr17	10222374/G//T	synonymous	0	1
RHBDL3	chr17	30648020/G//A	synonymous	0	1
ITGA3	chr17	48154017/G//A	nonsynonymous	0	1
ABCA10	chr17	67189713/T//G	nonsynonymous	0	1
ALPK2	chr18	56204912/C//G	nonsynonymous	0	1

TPGS1	chr19	518944/C//T	nonsynonymous	0	1
ATP8B3	chr19	1808340/A//G	synonymous	0	1
ZNF726	chr19	24115679/A//G	nonsynonymous	0	1
NLRP2	chr19	55494947/A//C	nonsynonymous	0	1
ZNF337	chr20	25656991/C//G	nonsynonymous	0	1
BPIFA3	chr20	31805365/T//C	nonsynonymous	0	1
RALGAPB	chr20	37202853/A//G	synonymous	0	1
ADNP	chr20	49508232/T//C	nonsynonymous	0	1
NCAM2	chr21	22664433/C//T	nonsynonymous	0	1
MXRA5	chrX	3238549/G//T	nonsynonymous	0	1
CFAP47	chrX	36103526/A//G	nonsynonymous	0	1
NUP62CL	chrX	106397472/C//G	nonsynonymous	0	1
MAGEC2	chrX	141291657/G//C	synonymous	0	1
ZFY	chrY	2844843/T//G	nonsynonymous	0	1
CAMTA1	chr1	7724661/C//T	nonsynonymous	0	1
LRP8	chr1	53728212/C//T	synonymous	0	1
SDCCAG8	chr1	243468033/T//G	nonsynonymous	0	1
LRP2	chr2	170048434/G//A	synonymous	0	1
SPEG	chr2	220313045/C//T	nonsynonymous	0	1
MST1	chr3	49723782/T//C	nonsynonymous	0	1
GFM2	chr5	74026131/G//A	synonymous	0	1
PCDHA9	chr5	140229362/C//T	nonsynonymous	0	1
NSD1	chr5	176662828/G//A	nonsynonymous	0	1
TRIM38	chr6	25966908/G//A	nonsynonymous	0	1
PHF1	chr6	33383777/C//T	synonymous	0	1
AMZ1	chr7	2742379/A//C	nonsynonymous	0	1
FAM188B	chr7	30890150/G//A	nonsynonymous	0	1
EBF2	chr8	25899604/C//T	synonymous	0	1
ELP3	chr8	27964289/A//G	synonymous	0	1
HTRA4	chr8	38831710/A//G	synonymous	0	1
TSPYL5	chr8	98289905/G//A	synonymous	0	1
GPR107	chr9	132816332/G//T	nonsynonymous	0	1
AP3M1	chr10	75896414/G//A	nonsynonymous	0	1
ST5	chr11	8732781/G//T	synonymous	0	1
AKAP3	chr12	4736440/C//A	nonsynonymous	0	1
CNTN1	chr12	41408023/G//T	synonymous	0	1
UBC	chr12	125396902/T//C	synonymous	0	1
SOS2	chr14	50655424/A//G	synonymous	0	1
VPS39	chr15	42456660/T//C	nonsynonymous	0	1
ABCC12	chr16	48119514/C//T	nonsynonymous	0	1
KISS1R	chr19	917587/G//A	nonsynonymous	0	1
ZNF90	chr19	20216132/G//A	synonymous	0	1
ADAM33	chr20	3651662/G//A	nonsynonymous	0	1
MBTPS2	chrX	21887662/T//C	nonsynonymous	0	1
DCAF8L1	chrX	27997984/G//T	nonsynonymous	0	1
NYX	chrX	41333489/G//T	synonymous	0	1
VAMP7	chrX	155127842/G//A	nonsynonymous	0	1
ATP13A2	chr1	17316482/GCAGCCTGGTCAGGA	nonsynonymous	0	1
SLC9C2	chr1	173472456/T//C	nonsynonymous	0	1
ZNF648	chr1	182025734/G//A	nonsynonymous	0	1
PTPRC	chr1	198713209/T//A	synonymous	0	1
SIX3	chr2	45170034/CGGCGGCCAA//C	nonsynonymous	0	1
PPP4R3B	chr2	55826111/C//T	nonsynonymous	0	1
IGFBP5	chr2	217543705/G//T	synonymous	0	1
IGFBP5	chr2	217543709/A//C	nonsynonymous	0	1
IGFBP5	chr2	217543710/TGCGGGTGTGTTTGC	nonsynonymous	0	1
CUL3	chr2	225346704/G//T	nonsynonymous	0	1
SCAP	chr3	47463998/CATGATGG//C	nonsynonymous	0	1
NMNAT3	chr3	139279975/C//G	synonymous	0	1
CLSTN2	chr3	140167547/G//T	nonsynonymous	0	1
MED12L	chr3	151105838/C//G	nonsynonymous	0	1
NUP54	chr4	77039339/G//A	synonymous	0	1
TRIML1	chr4	189068012/A//G	nonsynonymous	0	1
PCDHGC5	chr5	140871078/C//T	synonymous	0	1
HIST1H4F	chr6	26240894/A//G	nonsynonymous	0	1
C6orf89	chr6	36887349/TC//T	synonymous	0	1
PKHD1	chr6	51524322/G//T	nonsynonymous	0	1
PKHD1	chr6	51735420/T//C	synonymous	0	1
AKAP9	chr7	91630223/T//C	nonsynonymous	0	1
SVOPL	chr7	138333845/A//G	nonsynonymous	0	1

RIPK2	chr8	90775204/A//G	synonymous	0	1
RNF19A	chr8	101270867/A//C	nonsynonymous	0	1
MCMBP	chr10	121598061/T//C	nonsynonymous	0	1
OR4C11	chr11	55371106/G//T	nonsynonymous	0	1
OSBP	chr11	59368019/T//C	nonsynonymous	0	1
KMT5B	chr11	67925927/G//C	nonsynonymous	0	1
ZFC3H1	chr12	72008437/TGTTTCTGGATCCA//T	nonsynonymous	0	1
RALGAPA1	chr14	36194328/T//C	nonsynonymous	0	1
ZBTB1	chr14	64989053/TTCTGCACA//T	nonsynonymous	0	1
NFAT5	chr16	69726598/T//A	nonsynonymous	0	1
USP32	chr17	58290970/G//T	synonymous	0	1
DCC	chr18	50741946/C//T	synonymous	0	1
PALM	chr19	734172/G//C	nonsynonymous	0	1
CPAMD8	chr19	17017834/C//T	nonsynonymous	0	1
WDR87	chr19	38375926/C//T	synonymous	0	1
ZNF845	chr19	53855029/A//C	nonsynonymous	0	1
LILRB2	chr19	54781761/C//T	nonsynonymous	0	1
GDAP1L1	chr20	42907841/G//A	synonymous	0	1
ADNP	chr20	49510859/G//A	nonsynonymous	0	1
PAGE1	chrX	49459312/T//A	nonsynonymous	0	1
GPR173	chrX	53106920/A//T	nonsynonymous	0	1
NEXN	chr1	78407879/G//A	nonsynonymous	0	1
COL11A1	chr1	103540216/G//C	nonsynonymous	0	1
SCN1A	chr2	166848842/C//T	nonsynonymous	0	1
PIKFYVE	chr2	209179106/G//A	synonymous	0	1
MRPL47	chr3	179306730/C//A	nonsynonymous	0	1
KDR	chr4	55973990/C//G	synonymous	0	1
SLC4A4	chr4	72432748/G//A	nonsynonymous	0	1
DNAH5	chr5	13776589/A//G	nonsynonymous	0	1
IQGAP2	chr5	75991435/A//G	nonsynonymous	0	1
CDHR2	chr5	176011529/G//A	synonymous	0	1
KRBA1	chr7	149427259/G//A	synonymous	0	1
SSPO	chr7	149519637/G//A	nonsynonymous	0	1
ADCY8	chr8	132002775/G//T	nonsynonymous	0	1
CNTNAP3	chr9	39103763/A//G	synonymous	0	1
CIZ1	chr9	130938724/G//C	nonsynonymous	0	1
ITIH5	chr10	7708826/C//T	synonymous	0	1
TET1	chr10	70333434/T//A	nonsynonymous	0	1
MMS19	chr10	99230532/C//T	synonymous	0	1
MYBPC3	chr11	47359100/CTCT//C	nonsynonymous	0	1
TRIM49	chr11	89531678/T//G	nonsynonymous	0	1
CD163L1	chr12	7528296/G//T	synonymous	0	1
CD163L1	chr12	7585175/A//G	synonymous	0	1
ATP8A2	chr13	26402282/T//C	synonymous	0	1
NEMF	chr14	50319513/C//G	synonymous	0	1
	chr15	75628513/G//A	synonymous	0	1
FSD2	chr15	83434735/C//A	synonymous	0	1
PRSS36	chr16	31157273/G//A	nonsynonymous	0	1
SMARCE1	chr17	38802079/C//T	synonymous	0	1
ANKRD29	chr18	21218907/C//T	nonsynonymous	0	1
STXBP2	chr19	7708104/G//A	synonymous	0	1
AXL	chr19	41749607/C//A	nonsynonymous	0	1
FCAR	chr19	55399556/C//T	nonsynonymous	0	1
FAM209A	chr20	55100941/T//G	nonsynonymous	0	1
ZNF831	chr20	57768939/C//A	synonymous	0	1
BRWD1	chr21	40570928/G//C	nonsynonymous	0	1
KRTAP10-7	chr21	46020644/C//T	synonymous	0	1
CSF2RA	chrX	1409241/A//G	nonsynonymous	0	1
POF1B	chrX	84534346/A//G	synonymous	0	1
HCFC1	chrX	153220119/C//T	nonsynonymous	0	1
PINK1	chr1	20975612/C//T	nonsynonymous	0	1
YTHDF2	chr1	29069595/T//C	synonymous	0	1
SIKE1	chr1	115321902/A//G	nonsynonymous	0	1
QSOX1	chr1	180135680/G//A	nonsynonymous	0	1
RFX8	chr2	102038191/G//A	nonsynonymous	0	1
CYP20A1	chr2	204161617/T//C	nonsynonymous	0	1
MAP4	chr3	47969797/T//C	synonymous	0	1
KLHDC8B	chr3	49213177/T//C	synonymous	0	1
MON1A	chr3	49946481/T//C	nonsynonymous	0	1
TECRL	chr4	65180469/C//T	nonsynonymous	0	1

LRIT3	chr4	110791180/T//C	synonymous	0	1
FAT4	chr4	126372061/G//A	nonsynonymous	0	1
MYO10	chr5	16689934/T//C	nonsynonymous	0	1
HIST1H1D	chr6	26235059/T//C	nonsynonymous	0	1
UBR2	chr6	42627423/C//T	nonsynonymous	0	1
AIM1	chr6	106978139/A//G	nonsynonymous	0	1
CYP2W1	chr7	1026860/G//A	nonsynonymous	0	1
CCDC90B	chr11	82972912/G//A	synonymous	0	1
KRT8	chr12	53295822/T//C	nonsynonymous	0	1
RPGRIP1	chr14	21762911/G//A	nonsynonymous	0	1
IPO4	chr14	24652224/G//A	synonymous	0	1
SUSD6	chr14	70175467/G//A	nonsynonymous	0	1
E4F1	chr16	2283567/T//C	synonymous	0	1
GALNS	chr16	88907406/C//T	nonsynonymous	0	1
SGSM3	chr22	40800403/T//C	nonsynonymous	0	1
ACOT9	chrX	23723644/G//C	nonsynonymous	0	1
ATP6AP2	chrX	40464884/A//G	synonymous	0	1
HDAC6	chrX	48675752/T//C	nonsynonymous	0	1
PRAMEF4	chr1	12941680/C//A	synonymous	0	1
KCNA3	chr1	111216901/G//A	synonymous	0	1
RFWD2	chr1	176176257/G//C	synonymous	0	1
TET3	chr2	74273509/G//A	synonymous	0	1
DNAH12	chr3	57456171/G//A	nonsynonymous	0	1
BMP2K	chr4	79747233/T//G	nonsynonymous	0	1
PCDHGC3	chr5	140856394/G//A	synonymous	0	1
SOX7	chr8	10584155/G//A	nonsynonymous	0	1
MMP26	chr11	5012654/T//A	nonsynonymous	0	1
FANCF	chr11	22646511/A//C	synonymous	0	1
ANO3	chr11	26484601/C//T	nonsynonymous	0	1
KIAA1549L	chr11	33667510/G//A	synonymous	0	1
TCN1	chr11	59631454/T//G	nonsynonymous	0	1
RIN1	chr11	66102322/G//A	synonymous	0	1
C12orf60	chr12	14976048/T//G	nonsynonymous	0	1
DENND5B	chr12	31555559/A//G	nonsynonymous	0	1
USP44	chr12	95927389/C//T	nonsynonymous	0	1
HECTD1	chr14	31642787/A//G	nonsynonymous	0	1
TPM1	chr15	63354429/G//A	nonsynonymous	0	1
MAP2K4	chr17	12028670/G//T	nonsynonymous	0	1
SPIRE1	chr18	12449800/A//G	nonsynonymous	0	1
PLIN5	chr19	4531683/G//A	nonsynonymous	0	1
TSHZ2	chr20	51871248/G//T	nonsynonymous	0	1
	chr1	6420694/T//C	synonymous	0	1
OXCT2	chr1	40235983/G//A	synonymous	0	1
BCL9	chr1	147091709/C//T	nonsynonymous	0	1
TNN	chr1	175087903/G//T	nonsynonymous	0	1
GREM2	chr1	240656333/T//G	nonsynonymous	0	1
HEATR5B	chr2	37217794/T//G	synonymous	0	1
ANKRD53	chr2	71209151/G//A	nonsynonymous	0	1
TTC21B	chr2	166737286/A//G	synonymous	0	1
SLC25A12	chr2	172644390/G//A	synonymous	0	1
FSIP2	chr2	186657168/C//G	nonsynonymous	0	1
HECW2	chr2	197189872/A//T	nonsynonymous	0	1
CCDC66	chr3	56649169/T//C	nonsynonymous	0	1
KALRN	chr3	123983480/C//T	synonymous	0	1
LIPH	chr3	185252683/A//G	nonsynonymous	0	1
NWD2	chr4	37447890/C//T	nonsynonymous	0	1
ATP8A1	chr4	42424855/G//C	nonsynonymous	0	1
ANK2	chr4	114275660/T//G	synonymous	0	1
CDH9	chr5	26881625/C//T	nonsynonymous	0	1
C5orf42	chr5	37221521/T//C	nonsynonymous	0	1
SEC24A	chr5	134039496/G//A	nonsynonymous	0	1
OR12D3	chr6	29342986/C//T	nonsynonymous	0	1
MICA	chr6	31380222/G//A	nonsynonymous	0	1
BAG6	chr6	31608723/A//G	synonymous	0	1
PEX6	chr6	42932552/G//A	nonsynonymous	0	1
MAP3K5	chr6	137113134/G//T	nonsynonymous	0	1
FNDC1	chr6	159618542/A//T	nonsynonymous	0	1
	chr7	87811379/C//T	synonymous	0	1
RAD54B	chr8	95404018/A//G	nonsynonymous	0	1
POLR3A	chr10	79784862/T//C	synonymous	0	1

KCNQ1	chr11	2608914/T//C	nonsynonymous	0	1
OR4C6	chr11	55432790/A//G	nonsynonymous	0	1
AHNAK	chr11	62301239/G//A	nonsynonymous	0	1
LRFN4	chr11	66627629/G//A	nonsynonymous	0	1
PLEKHB1	chr11	73372584/G//A	nonsynonymous	0	1
FAT3	chr11	92531514/A//C	nonsynonymous	0	1
KDELC1	chr13	103449241/A//G	nonsynonymous	0	1
BIVM	chr13	103468910/G//A	synonymous	0	1
GTF3C1	chr16	27483098/C//T	nonsynonymous	0	1
PLCG2	chr16	81969845/G//A	nonsynonymous	0	1
RALBP1	chr18	9530855/A//G	synonymous	0	1
SMAD2	chr18	45368290/T//TCCAGCAAGGAGTA	nonsynonymous	0	1
SALL3	chr18	76755414/C//T	synonymous	0	1
GPI	chr19	34890117/C//A	synonymous	0	1
GYS1	chr19	49472836/C//T	synonymous	0	1
PRR12	chr19	50101077/C//T	nonsynonymous	0	1
BMP7	chr20	55777673/C//T	synonymous	0	1
KLHDC7B	chr22	50986764/G//A	nonsynonymous	0	1
USP9X	chrX	41055518/T//C	nonsynonymous	0	1
TSPYL2	chrX	53115119/C//T	synonymous	0	1
NRK	chrX	105190363/A//G	synonymous	0	1
SLC45A1	chr1	8384561/C//G	nonsynonymous	0	1
SGIP1	chr1	67147733/A//C	nonsynonymous	0	1
GIPC2	chr1	78560669/A//T	nonsynonymous	0	1
RPAP2	chr1	92772775/T//C	nonsynonymous	0	1
TMED5	chr1	93620441/G//A	nonsynonymous	0	1
FMO3	chr1	171083429/C//A	synonymous	0	1
PRRC2C	chr1	171492400/G//C	nonsynonymous	0	1
EIF2D	chr1	206765094/A//G	synonymous	0	1
LAMB3	chr1	209797343/C//T	nonsynonymous	0	1
TRAF3IP3	chr1	209933527/C//A	nonsynonymous	0	1
NLRP3	chr1	247592921/C//T	nonsynonymous	0	1
ADD2	chr2	70931598/C//T	synonymous	0	1
DCTN1	chr2	74597826/C//A	nonsynonymous	0	1
LRP2	chr2	170113721/A//C	nonsynonymous	0	1
TTN	chr2	179504779/T//G	nonsynonymous	0	1
HDAC11	chr3	13538327/G//A	nonsynonymous	0	1
GLYCTK	chr3	52326467/C//A	nonsynonymous	0	1
SIDT1	chr3	113286500/C//T	nonsynonymous	0	1
KLHL24	chr3	183390154/T//C	nonsynonymous	0	1
TNK2	chr3	195611741/C//T	nonsynonymous	0	1
NUDT9	chr4	88362994/C//T	nonsynonymous	0	1
ANK2	chr4	114277457/A//C	nonsynonymous	0	1
	chr4	123533996/A//G	synonymous	0	1
FAT4	chr4	126411985/A//T	nonsynonymous	0	1
RBM46	chr4	155717975/C//T	nonsynonymous	0	1
KLKB1	chr4	187175881/G//A	nonsynonymous	0	1
ADCY2	chr5	7709500/G//A	synonymous	0	1
SEMA5A	chr5	9119162/G//A	nonsynonymous	0	1
ROPN1L	chr5	10465069/G//T	synonymous	0	1
FAM196B	chr5	169309995/G//T	nonsynonymous	0	1
PKHD1	chr6	51720847/A//C	synonymous	0	1
DAGLB	chr7	6485689/C//A	nonsynonymous	0	1
ZNF716	chr7	57529721/C//T	synonymous	0	1
GNAI1	chr7	79840327/G//C	nonsynonymous	0	1
SEMA3A	chr7	83636759/C//A	nonsynonymous	0	1
SRI	chr7	87838715/G//T	synonymous	0	1
CFAP69	chr7	89915712/A//G	nonsynonymous	0	1
TBXAS1	chr7	139655357/C//A	nonsynonymous	0	1
CSMD1	chr8	3165330/A//G	synonymous	0	1
TRIM35	chr8	27168600/C//T	synonymous	0	1
POU5F1B	chr8	128428581/G//C	nonsynonymous	0	1
FAM83H	chr8	144810435/G//A	nonsynonymous	0	1
SPATA31C2	chr9	90747134/A//T	nonsynonymous	0	1
ABCA1	chr9	107568585/G//A	synonymous	0	1
FAM166A	chr9	140140207/G//T	nonsynonymous	0	1
CDH23	chr10	73461953/G//A	nonsynonymous	0	1
PLAU	chr10	75672711/G//A	nonsynonymous	0	1
SORCS3	chr10	107012532/G//T	nonsynonymous	0	1
CFAP46	chr10	134686336/G//A	synonymous	0	1

DCHS1	chr11	6644235/C//A	nonsynonymous	0	1
CYB5R2	chr11	7686635/A//T	synonymous	0	1
TAF1D	chr11	93472420/C//T	synonymous	0	1
TRPC6	chr11	101347219/C//G	nonsynonymous	0	1
CADM1	chr11	115088647/C//T	synonymous	0	1
RB1	chr13	49037902/G//A	synonymous	0	1
C14orf93	chr14	23456494/T//C	nonsynonymous	0	1
JPH4	chr14	24040488/C//T	synonymous	0	1
AHNAK2	chr14	105444530/G//A	synonymous	0	1
UNC13C	chr15	54305975/G//A	nonsynonymous	0	1
RASGRF1	chr15	79310104/C//G	synonymous	0	1
SCNN1G	chr16	23223381/A//C	synonymous	0	1
SNTB2	chr16	69318042/A//G	nonsynonymous	0	1
LIG3	chr17	33326870/C//T	nonsynonymous	0	1
ABCA8	chr17	66933201/A//C	nonsynonymous	0	1
CCDC40	chr17	78055849/A//G	nonsynonymous	0	1
ENDOV	chr17	78410012/C//T	synonymous	0	1
MOB3A	chr19	2078284/C//T	synonymous	0	1
PIK3R2	chr19	18273784/G//A	nonsynonymous	0	1
ZNF676	chr19	22375810/T//C	synonymous	0	1
KIR3DL2	chr19	55365201/G//A	nonsynonymous	0	1
ZNF154	chr19	58213218/G//A	nonsynonymous	0	1
C20orf194	chr20	3233304/C//T	nonsynonymous	0	1
NCOA6	chr20	33337488/T//C	nonsynonymous	0	1
CD40	chr20	44747009/C//T	synonymous	0	1
PHACTR3	chr20	58420269/A//T	nonsynonymous	0	1
GRIK1	chr21	30961177/A//T	synonymous	0	1
BPIFC	chr22	32831766/G//A	synonymous	0	1
CDKL5	chrX	18622187/C//T	synonymous	0	1
MTRNR2L10	chrX	55208416/T//C	synonymous	0	1
FAM46D	chrX	79698851/T//C	synonymous	0	1
MAGEC1	chrX	140993372/A//T	nonsynonymous	0	1
SV2A	chr1	149881036/G//A	nonsynonymous	0	1
REG3A	chr2	79385557/C//A	synonymous	0	1
EIF5B	chr2	99985055/G//A	nonsynonymous	0	1
GLI2	chr2	121746637/C//T	synonymous	0	1
FRG2C	chr3	75715033/T//C	synonymous	0	1
APC	chr5	112175411/G//GA	nonsynonymous	0	1
KDM3B	chr5	137734080/C//T	synonymous	0	1
BCAP29	chr7	107259020/A//G	synonymous	0	1
CADPS2	chr7	122091459/AAG//A	nonsynonymous	0	1
SLC1A1	chr9	4573916/A//G	synonymous	0	1
PHF2	chr9	96411381/T//C	synonymous	0	1
DIP2C	chr10	465011/C//T	nonsynonymous	0	1
PHYH	chr10	13336420/T//C	synonymous	0	1
WDFY4	chr10	50165327/G//A	nonsynonymous	0	1
CACNA1C	chr12	2675648/T//C	synonymous	0	1
HELB	chr12	66718891/T//A	synonymous	0	1
SCFD1	chr14	31191086/A//G	synonymous	0	1
ARID4A	chr14	58832006/A//G	nonsynonymous	0	1
VAT1L	chr16	77822654/G//A	synonymous	0	1
MKNK2	chr19	2041042/C//T	synonymous	0	1
DSCAM	chr21	41427743/C//T	synonymous	0	1
SPSB1	chr1	9427658/C//T	synonymous	0	1
AIM1L	chr1	26670912/T//C	nonsynonymous	0	1
SPOCD1	chr1	32263851/G//C	nonsynonymous	0	1
KIAA1522	chr1	33236153/G//A	nonsynonymous	0	1
STIL	chr1	47755171/T//C	nonsynonymous	0	1
LRRC7	chr1	70486808/T//A	nonsynonymous	0	1
FCRL6	chr1	159778143/G//C	nonsynonymous	0	1
WNT9A	chr1	228109596/T//TG	nonsynonymous	0	1
SCTR	chr2	120236445/C//T	synonymous	0	1
LRP2	chr2	170083030/T//C	nonsynonymous	0	1
LRP2	chr2	170163901/C//T	nonsynonymous	0	1
	chr2	223065774/G//A	synonymous	0	1
SETD5	chr3	9475559/T//C	synonymous	0	1
FAM131A	chr3	184059910/C//G	nonsynonymous	0	1
LRBA	chr4	151186893/T//C	nonsynonymous	0	1
ZFR	chr5	32388697/C//G	nonsynonymous	0	1
CHD1	chr5	98217047/G//A	nonsynonymous	0	1

ABLIM3	chr5	148632379/T//C	synonymous	0	1
FABP6	chr5	159656566/T//C	nonsynonymous	0	1
N4BP3	chr5	177548154/A//G	nonsynonymous	0	1
GCNT2	chr6	10529827/G//C	nonsynonymous	0	1
VPS52	chr6	33235094/G//C	nonsynonymous	0	1
EPHA7	chr6	93967896/T//C	synonymous	0	1
HACE1	chr6	105243536/A//G	nonsynonymous	0	1
COL22A1	chr8	139845307/AT//A	nonsynonymous	0	1
TMEM26	chr10	63212742/T//C	nonsynonymous	0	1
ZNF215	chr11	6953397/G//C	synonymous	0	1
SCGB1D4	chr11	62063818/T//C	synonymous	0	1
CCDC88B	chr11	64111734/G//A	nonsynonymous	0	1
ANO4	chr12	101365177/C//T	nonsynonymous	0	1
ITPK1	chr14	93542947/A//G	nonsynonymous	0	1
NUDT14	chr14	105639561/A//G	nonsynonymous	0	1
GABRG3	chr15	27777755/C//G	nonsynonymous	0	1
ALDH1A2	chr15	58284974/C//G	nonsynonymous	0	1
PTPN9	chr15	75809668/C//CA	nonsynonymous	0	1
EFL1	chr15	82444165/G//C	nonsynonymous	0	1
ADAMTS17	chr15	100537659/C//T	synonymous	0	1
CCP110	chr16	19559307/A//G	synonymous	0	1
ASPHD1	chr16	29912996/C//A	nonsynonymous	0	1
OSGIN1	chr16	83994372/G//A	synonymous	0	1
SGSM2	chr17	2264987/C//T	nonsynonymous	0	1
PIRT	chr17	10729022/G//C	synonymous	0	1
GAA	chr17	78085814/A//T	nonsynonymous	0	1
YES1	chr18	745824/T//C	nonsynonymous	0	1
	chr18	3874693/G//A	synonymous	0	1
SETBP1	chr18	42618502/C//T	synonymous	0	1
PTPRS	chr19	5244233/C//T	nonsynonymous	0	1
C19orf44	chr19	16623834/T//C	nonsynonymous	0	1
ZNF536	chr19	31040162/A//G	synonymous	0	1
GSK3A	chr19	42738778/A//G	nonsynonymous	0	1
TRAPPC6A	chr19	45666421/C//T	synonymous	0	1
ZNF667	chr19	56953038/A//G	synonymous	0	1
TRIB3	chr20	368679/C//T	nonsynonymous	0	1
RALGAPA2	chr20	20596774/A//G	nonsynonymous	0	1
	chr21	44323657/A//G	synonymous	0	1
PATZ1	chr22	31740379/A//G	nonsynonymous	0	1
SLC5A1	chr22	32498215/G//A	synonymous	0	1
DMD	chrX	32613949/A//G	synonymous	0	1
CFAP47	chrX	36366284/G//C	nonsynonymous	0	1
L1TD1	chr1	62672694/A//T	nonsynonymous	0	1
MFSD14A	chr1	100533672/T//G	nonsynonymous	0	1
SPAG17	chr1	118596713/T//C	nonsynonymous	0	1
KPRP	chr1	152733512/C//T	nonsynonymous	0	1
SPTA1	chr1	158606546/T//C	nonsynonymous	0	1
FSIP2	chr2	186654153/G//C	nonsynonymous	0	1
MRAS	chr3	138121034/C//CAGA	nonsynonymous	0	1
ATR	chr3	142185265/C//T	nonsynonymous	0	1
RGS12	chr4	3318133/A//C	nonsynonymous	0	1
BEND4	chr4	42119508/G//T	synonymous	0	1
TMPRSS11E	chr4	69343269/G//T	nonsynonymous	0	1
WDFY3	chr4	85696294/A//G	synonymous	0	1
NR3C2	chr4	149356613/T//C	nonsynonymous	0	1
DNAH5	chr5	13792177/A//G	nonsynonymous	0	1
MSH3	chr5	80074660/G//T	synonymous	0	1
APC	chr5	112163618/A//G	synonymous	0	1
TRPC7	chr5	135567066/T//C	nonsynonymous	0	1
PCDHA12	chr5	140255189/C//T	synonymous	0	1
CD74	chr5	149786486/C//T	synonymous	0	1
	chr6	10557388/A//T	synonymous	0	1
PEX6	chr6	42946000/AT//A	synonymous	0	1
B3GAT2	chr6	71665652/T//G	nonsynonymous	0	1
IBTK	chr6	82925956/T//C	nonsynonymous	0	1
GJA10	chr6	90605007/T//C	nonsynonymous	0	1
MUC17	chr7	100676559/C//T	nonsynonymous	0	1
MUC17	chr7	100676569/C//T	synonymous	0	1
SHH	chr7	155596171/A//G	nonsynonymous	0	1
ANGPT2	chr8	6420375/G//A	synonymous	0	1

CSMD3	chr8	113303878/T//G	nonsynonymous	0	1
SMARCA2	chr9	2191332/G//T	nonsynonymous	0	1
PCSK5	chr9	78973573/C//A	nonsynonymous	0	1
VPS13A	chr9	79932549/C//T	nonsynonymous	0	1
JMJD1C	chr10	64977092/C//A	nonsynonymous	0	1
WNT8B	chr10	102240768/A//G	synonymous	0	1
VAX1	chr10	118896088/G//A	synonymous	0	1
DMBT1	chr10	124344787/A//T	nonsynonymous	0	1
MUC6	chr11	1029360/G//T	nonsynonymous	0	1
MUC15	chr11	26581396/C//T	synonymous	0	1
FBXO3	chr11	33777497/A//G	synonymous	0	1
OR5AS1	chr11	55798636/G//T	nonsynonymous	0	1
RIN1	chr11	66103805/C//T	synonymous	0	1
RSF1	chr11	77409696/G//C	nonsynonymous	0	1
IGSF9B	chr11	133790800/G//A	synonymous	0	1
PRMT8	chr12	3659109/T//C	nonsynonymous	0	1
BICD1	chr12	32480722/G//A	nonsynonymous	0	1
ADCY6	chr12	49176786/G//A	synonymous	0	1
FIGNL2	chr12	52216076/C//G	nonsynonymous	0	1
GLI1	chr12	57864645/G//C	nonsynonymous	0	1
PPM1H	chr12	63061003/G//A	nonsynonymous	0	1
CPM	chr12	69260751/G//A	nonsynonymous	0	1
ACSS3	chr12	81503383/G//A	nonsynonymous	0	1
CEP83	chr12	94706755/T//G	nonsynonymous	0	1
TRMT5	chr14	61445948/C//T	nonsynonymous	0	1
MLH3	chr14	75513492/T//C	nonsynonymous	0	1
ISM2	chr14	77948673/A//G	nonsynonymous	0	1
UNC45A	chr15	91489959/A//G	nonsynonymous	0	1
CHST5	chr16	75563186/C//T	nonsynonymous	0	1
KRT27	chr17	38936050/C//T	nonsynonymous	0	1
CDC27	chr17	45229240/T//C	synonymous	0	1
ZNF878	chr19	12155742/A//G	synonymous	0	1
ZNF98	chr19	22574480/C//T	synonymous	0	1
FAM83E	chr19	49113191/T//C	nonsynonymous	0	1
C20orf144	chr20	32250263/C//T	nonsynonymous	0	1
FTCD	chr21	47571833/G//A	synonymous	0	1
EIF2S3	chrX	24078283/A//T	synonymous	0	1
IGBP1	chrX	69385835/T//G	synonymous	0	1
MAGEC2	chrX	141291549/C//A	synonymous	0	1
CETN2	chrX	151996389/T//C	nonsynonymous	0	1
EIF4G3	chr1	21151697/A//G	synonymous	1	0
NCF2	chr1	183525366/C//T	nonsynonymous	1	0
OBSCN	chr1	228558877/C//T	nonsynonymous	1	0
TBCE	chr1	235590465/G//T	nonsynonymous	1	0
MSH6	chr2	48032088/G//A	nonsynonymous	1	0
ABCA12	chr2	215868978/C//T	nonsynonymous	1	0
PRR21	chr2	240981417/G//A	nonsynonymous	1	0
TBL1XR1	chr3	176744171/G//A	nonsynonymous	1	0
SLC26A1	chr4	985137/G//A	nonsynonymous	1	0
CMYA5	chr5	79026966/C//T	nonsynonymous	1	0
PCDHA4	chr5	140188217/C//T	nonsynonymous	1	0
PCDHA5	chr5	140203211/G//A	synonymous	1	0
PCDHGB4	chr5	140769248/C//T	synonymous	1	0
GLTSCR1L	chr6	42821414/G//A	nonsynonymous	1	0
CEP162	chr6	84865156/G//A	nonsynonymous	1	0
ARID1B	chr6	157511296/A//G	nonsynonymous	1	0
SYNJ2	chr6	158402913/G//A	synonymous	1	0
INHBA	chr7	41729399/C//T	nonsynonymous	1	0
NUPR2	chr7	56184055/G//A	synonymous	1	0
ADAM22	chr7	87762230/G//C	synonymous	1	0
SLC13A4	chr7	135412196/C//T	nonsynonymous	1	0
ZNF395	chr8	28210108/A//G	nonsynonymous	1	0
NBN	chr8	90967786/G//C	synonymous	1	0
RNF139	chr8	125498606/G//A	nonsynonymous	1	0
EPPK1	chr8	144946427/A//G	nonsynonymous	1	0
ERP44	chr9	102861120/C//CA	nonsynonymous	1	0
PTPN3	chr9	112145834/G//C	synonymous	1	0
ARL3	chr10	104436591/T//C	synonymous	1	0
NLRP10	chr11	7981533/C//T	synonymous	1	0
ZBTB44	chr11	130131055/T//G	synonymous	1	0

CD163	chr12	7637769/G//A	nonsynonymous	1	0
AQP6	chr12	50367139/C//T	synonymous	1	0
GRK1	chr13	114435967/G//A	synonymous	1	0
ESRRB	chr14	76964674/T//A	nonsynonymous	1	0
FES	chr15	91434222/C//T	nonsynonymous	1	0
IFT140	chr16	1652566/G//A	synonymous	1	0
USP7	chr16	8988907/A//C	nonsynonymous	1	0
TMC5	chr16	19483450/C//T	nonsynonymous	1	0
VAT1L	chr16	77850877/C//G	nonsynonymous	1	0
TP53	chr17	7577097/C//A	nonsynonymous	1	0
WIPF2	chr17	38416927/C//T	synonymous	1	0
BAHCC1	chr17	79425853/C//T	synonymous	1	0
PRTN3	chr19	846342/ACTTT//A	nonsynonymous	1	0
MUC16	chr19	9002493/T//C	synonymous	1	0
MUC16	chr19	9064376/C//T	synonymous	1	0
ZNF559-ZNF177	chr19	9490741/A//G	nonsynonymous	1	0
BEST2	chr19	12865463/C//T	synonymous	1	0
CD22	chr19	35823532/C//T	synonymous	1	0
RASGRP4	chr19	38905649/C//A	nonsynonymous	1	0
EML2-AS1	chr19	46145261/G//A	synonymous	1	0
SPHK2	chr19	49132587/G//C	nonsynonymous	1	0
PHACTR3	chr20	58330412/C//T	synonymous	1	0
CACNG2	chr22	36960675/C//T	nonsynonymous	1	0
FGD1	chrX	54495224/T//A	nonsynonymous	1	0
PGK1	chrX	77380388/T//C	synonymous	1	0
TENM1	chrX	123787547/G//A	nonsynonymous	1	0
MAGEC3	chrX	140983157/G//T	nonsynonymous	1	0
CNGA2	chrX	150911942/G//C	nonsynonymous	1	0
CLCN6	chr1	11894611/C//T	nonsynonymous	0	1
ST6GALNAC5	chr1	77510290/C//A	synonymous	0	1
MCOLN2	chr1	85405351/C//T	nonsynonymous	0	1
COL11A1	chr1	103483396/G//A	nonsynonymous	0	1
PROK1	chr1	110996598/G//A	nonsynonymous	0	1
CCDC185	chr1	223566940/C//T	synonymous	0	1
OBSCN	chr1	228465546/G//C	nonsynonymous	0	1
CNGA3	chr2	99013440/C//T	nonsynonymous	0	1
UBXN4	chr2	136540312/G//A	synonymous	0	1
INPP5D	chr2	233986934/G//A	nonsynonymous	0	1
HSPBAP1	chr3	122487662/A//G	synonymous	0	1
YEATS2	chr3	183436371/A//G	synonymous	0	1
IRX1	chr5	3601238/T//C	synonymous	0	1
DNAH5	chr5	13777433/G//T	nonsynonymous	0	1
CLPSL1	chr6	35754859/G//A	nonsynonymous	0	1
TDRD6	chr6	46656075/C//T	synonymous	0	1
BCKDHB	chr6	80881069/G//T	nonsynonymous	0	1
OSTM1	chr6	108370594/C//T	nonsynonymous	0	1
LAMA4	chr6	112476836/A//G	synonymous	0	1
LAMA2	chr6	129794482/C//T	nonsynonymous	0	1
MYCT1	chr6	153019200/A//C	nonsynonymous	0	1
GRB10	chr7	50660647/G//A	synonymous	0	1
TBL2	chr7	72992843/A//G	nonsynonymous	0	1
NUP205	chr7	135282890/C//T	nonsynonymous	0	1
ABCA2	chr9	139903855/C//T	nonsynonymous	0	1
MAP3K8	chr10	30747086/C//T	nonsynonymous	0	1
SLC35G1	chr10	95660897/G//A	nonsynonymous	0	1
MFRP	chr11	119215585/G//A	synonymous	0	1
CCDC184	chr12	48578223/C//T	synonymous	0	1
MBD6	chr12	57922052/C//T	synonymous	0	1
VPS29	chr12	110930973/C//T	nonsynonymous	0	1
FZD10	chr12	130648864/C//T	synonymous	0	1
SACS	chr13	23911813/T//A	nonsynonymous	0	1
PDS5B	chr13	33349272/A//G	synonymous	0	1
PCDH9	chr13	67802580/T//C	synonymous	0	1
NPAS3	chr14	34266717/G//A	synonymous	0	1
DDHD1	chr14	53619018/C//T	nonsynonymous	0	1
HIF1A	chr14	62212491/A//G	nonsynonymous	0	1
RYR3	chr15	34137131/G//A	synonymous	0	1
POLG	chr15	89862280/C//T	nonsynonymous	0	1
BLM	chr15	91326140/A//G	nonsynonymous	0	1
ZNF500	chr16	4815622/C//T	nonsynonymous	0	1

DNAJA2	chr16	47005385/C//T	nonsynonymous	0	1
PIEZO1	chr16	88793442/C//A	synonymous	0	1
KRT25	chr17	38906816/C//T	nonsynonymous	0	1
ARHGAP27	chr17	43473166/G//A	synonymous	0	1
GATA6	chr18	19752098/C//T	synonymous	0	1
PLIN5	chr19	4523889/C//T	nonsynonymous	0	1
ZNRF4	chr19	5455644/T//C	nonsynonymous	0	1
ZNF667	chr19	56952783/A//G	synonymous	0	1
ZNF835	chr19	57175897/A//T	nonsynonymous	0	1
AURKC	chr19	57746684/C//A	nonsynonymous	0	1
ZMYND8	chr20	45874842/C//T	nonsynonymous	0	1
TPTE	chr21	10906990/C//T	nonsynonymous	0	1
MPPED1	chr22	43831052/C//T	nonsynonymous	0	1
ADGRG2	chrX	19024222/A//G	synonymous	0	1
PRAMEF11	chr1	12885005/C//T	nonsynonymous	0	1
ADAR	chr1	154558767/A//C	nonsynonymous	0	1
YY1AP1	chr1	155657923/G//A	synonymous	0	1
LMNA	chr1	156084829/T//C	synonymous	0	1
DNM3	chr1	172062020/G//T	synonymous	0	1
ST6GAL2	chr2	107450547/T//G	nonsynonymous	0	1
CELSR3	chr3	48699529/T//C	nonsynonymous	0	1
VGLL3	chr3	86996104/A//C	synonymous	0	1
	chr3	184971669/A//G	synonymous	0	1
GRXCR1	chr4	42964937/G//A	nonsynonymous	0	1
BASP1	chr5	17275433/C//T	synonymous	0	1
PARP8	chr5	50055515/G//A	nonsynonymous	0	1
TMEM232	chr5	109961044/T//C	nonsynonymous	0	1
MUC22	chr6	30994181/T//A	nonsynonymous	0	1
TAP2	chr6	32802958/C//T	synonymous	0	1
ICK	chr6	52878460/C//T	synonymous	0	1
SEC63	chr6	108204310/T//G	nonsynonymous	0	1
HDAC9	chr7	18684313/G//A	nonsynonymous	0	1
HGF	chr7	81399273/T//C	synonymous	0	1
SLC24A2	chr9	19786519/C//A	nonsynonymous	0	1
AFAP1L2	chr10	116083010/C//T	nonsynonymous	0	1
DSCAML1	chr11	117303079/C//T	nonsynonymous	0	1
GRASP	chr12	52408482/G//T	synonymous	0	1
TBK1	chr12	64860826/G//A	synonymous	0	1
STYX	chr14	53226442/T//G	nonsynonymous	0	1
TLE6	chr19	2987752/G//A	nonsynonymous	0	1
ZNF98	chr19	22574648/T//C	synonymous	0	1
ZNF645	chrX	22292056/A//C	synonymous	0	1
CCDC120	chrX	48925238/G//A	nonsynonymous	0	1
CXorf57	chrX	105876162/G//T	nonsynonymous	0	1
H6PD	chr1	9323631/A//G	nonsynonymous	0	1
TAS1R2	chr1	19180787/A//G	nonsynonymous	0	1
KIF17	chr1	21014345/A//G	nonsynonymous	0	1
USP48	chr1	22074644/A//G	nonsynonymous	0	1
ATF6	chr1	161789538/T//C	nonsynonymous	0	1
ZEB2	chr2	145147559/G//A	nonsynonymous	0	1
GALNT13	chr2	155158098/C//CCCAGG	nonsynonymous	0	1
SCN1A	chr2	166929960/C//T	nonsynonymous	0	1
COPS8	chr2	238006426/A//G	synonymous	0	1
RAF1	chr3	12645688/G//A	nonsynonymous	0	1
GALNT15	chr3	16254202/G//C	nonsynonymous	0	1
CCDC66	chr3	56655658/C//G	synonymous	0	1
PLXNA1	chr3	126707515/G//A	nonsynonymous	0	1
MELTF	chr3	196744076/G//A	synonymous	0	1
KIAA1211	chr4	57180504/C//T	nonsynonymous	0	1
NKAPL	chr6	28227744/A//G	nonsynonymous	0	1
FAM26E	chr6	116833227/C//A	nonsynonymous	0	1
AGBL3	chr7	134719261/C//A	nonsynonymous	0	1
MGAM	chr7	141736007/T//C	synonymous	0	1
FAM167A	chr8	11302004/C//T	synonymous	0	1
ADAM2	chr8	39694694/C//A	nonsynonymous	0	1
RBM12B	chr8	94746810/T//C	nonsynonymous	0	1
GNE	chr9	36249369/T//C	synonymous	0	1
ZNF618	chr9	116769651/A//G	nonsynonymous	0	1
DHTKD1	chr10	12148351/T//C	nonsynonymous	0	1
EXT2	chr11	44255775/G//A	synonymous	0	1

SYVN1	chr11	64899815/C//T	nonsynonymous	0	1
PRDM10	chr11	129784891/C//T	nonsynonymous	0	1
NCAPD3	chr11	134023249/C//T	nonsynonymous	0	1
KLHL42	chr12	27951087/A//G	synonymous	0	1
NAV3	chr12	78511807/C//T	synonymous	0	1
MYF5	chr12	81110767/C//T	synonymous	0	1
MMP17	chr12	132334601/G//A	nonsynonymous	0	1
EP400	chr12	132514302/G//A	nonsynonymous	0	1
SLC10A2	chr13	103718363/C//T	nonsynonymous	0	1
SMOC1	chr14	70478273/G//A	nonsynonymous	0	1
DYNC1H1	chr14	102504831/T//C	nonsynonymous	0	1
ZNF423	chr16	49671973/C//T	nonsynonymous	0	1
SALL1	chr16	51174084/A//G	synonymous	0	1
ADAMTS18	chr16	77369685/G//C	synonymous	0	1
KIAA0513	chr16	85112552/A//G	nonsynonymous	0	1
CD300LG	chr17	41931398/C//A	synonymous	0	1
CDC27	chr17	45234709/A//G	synonymous	0	1
SERPINB2	chr18	61570438/C//A	nonsynonymous	0	1
CDH19	chr18	64178923/C//G	nonsynonymous	0	1
ZNF709	chr19	12575005/C//T	nonsynonymous	0	1
ZNF709	chr19	12575020/A//G	synonymous	0	1
LILRA1	chr19	55107378/C//T	synonymous	0	1
NECAB3	chr20	32260202/A//G	nonsynonymous	0	1
EYA2	chr20	45718026/C//A	synonymous	0	1
KRTAP10-11	chr21	46066659/G//A	nonsynonymous	0	1
TTLL12	chr22	43575656/C//T	nonsynonymous	0	1
LONRF3	chrX	118109075/T//C	nonsynonymous	0	1
SPANXN1	chrX	144329162/C//T	nonsynonymous	0	1
HOOK1	chr1	60338669/C//A	synonymous	0	1
ASB17	chr1	76384632/G//A	synonymous	0	1
RPAP2	chr1	92799008/G//A	nonsynonymous	0	1
IFI16	chr1	158988272/A//G	nonsynonymous	0	1
OBSCN	chr1	228444454/C//T	nonsynonymous	0	1
CCT7	chr2	73470142/G//A	nonsynonymous	0	1
SLC4A7	chr3	27439173/A//G	nonsynonymous	0	1
SLC4A7	chr3	27439198/G//A	nonsynonymous	0	1
MUC4	chr3	195512287/G//A	nonsynonymous	0	1
GAK	chr4	875835/C//T	synonymous	0	1
PRDM5	chr4	121828693/A//G	nonsynonymous	0	1
SDHA	chr5	226082/G//C	nonsynonymous	0	1
ICE1	chr5	5460898/C//G	nonsynonymous	0	1
WDR36	chr5	110446009/A//T	synonymous	0	1
PKD2L2	chr5	137228217/T//C	nonsynonymous	0	1
SLC22A7	chr6	43267462/A//G	nonsynonymous	0	1
SLC22A7	chr6	43271939/C//A	nonsynonymous	0	1
SAMD3	chr6	130535536/G//A	nonsynonymous	0	1
LPA	chr6	160961159/A//G	nonsynonymous	0	1
ZC3HAV1	chr7	138768750/C//T	nonsynonymous	0	1
LOXL2	chr8	23155621/C//T	nonsynonymous	0	1
WISP1	chr8	134239766/A//G	nonsynonymous	0	1
MBL2	chr10	54531407/T//G	synonymous	0	1
ADAMTS14	chr10	72492020/C//T	synonymous	0	1
HPS1	chr10	100185390/G//C	nonsynonymous	0	1
OAF	chr11	120099816/C//T	synonymous	0	1
ITPR2	chr12	26580958/A//G	nonsynonymous	0	1
ANO6	chr12	45742085/C//A	nonsynonymous	0	1
PRPH	chr12	49691261/C//T	nonsynonymous	0	1
CLIP1	chr12	122773095/A//C	synonymous	0	1
CENPJ	chr13	25487125/C//T	synonymous	0	1
KBTBD7	chr13	41766649/C//T	nonsynonymous	0	1
ADCY4	chr14	24798353/G//A	nonsynonymous	0	1
TTC7B	chr14	91044455/G//A	nonsynonymous	0	1
NHLRC4	chr16	618123/G//A	nonsynonymous	0	1
ADCY7	chr16	50338418/G//A	nonsynonymous	0	1
WRAP53	chr17	7604781/G//T	nonsynonymous	0	1
ARHGAP44	chr17	12799726/G//T	synonymous	0	1
MUC16	chr19	9002576/T//C	nonsynonymous	0	1
MUC16	chr19	9002587/C//T	nonsynonymous	0	1
MUC16	chr19	9002597/C//T	nonsynonymous	0	1
CLASRP	chr19	45570617/C//T	nonsynonymous	0	1

LILRA4	chr19	54849846/C//T	nonsynonymous	0	1
KIR3DL1	chr19	55330036/G//A	nonsynonymous	0	1
MCM8	chr20	5953761/C//T	synonymous	0	1
LRRC3	chr21	45877153/T//C	nonsynonymous	0	1
NCF4	chr22	37267747/C//A	nonsynonymous	0	1
CELSR1	chr22	46931025/A//G	synonymous	0	1
PJA1	chrX	68381401/C//T	nonsynonymous	0	1
DRP2	chrX	100496661/C//G	synonymous	0	1
LMNA	chr1	156107521/T//A	nonsynonymous	0	1
F5	chr1	169510376/G//C	nonsynonymous	0	1
F5	chr1	169510379/C//T	nonsynonymous	0	1
RC3H1	chr1	173931175/A//G	synonymous	0	1
GPR45	chr2	105858707/A//G	nonsynonymous	0	1
MYO7B	chr2	128393453/C//T	nonsynonymous	0	1
MARCH4	chr2	217234857/G//A	nonsynonymous	0	1
PRKCI	chr3	169977774/T//C	nonsynonymous	0	1
TPRG1	chr3	188956657/C//A	synonymous	0	1
LRRC14B	chr5	195416/C//G	nonsynonymous	0	1
WDR70	chr5	37438084/G//T	nonsynonymous	0	1
APC	chr5	112175709/AT//A	nonsynonymous	0	1
RAD50	chr5	131939715/A//G	nonsynonymous	0	1
KHDC1	chr6	73951235/G//A	synonymous	0	1
TAF6	chr7	99709804/A//G	nonsynonymous	0	1
KCNMA1	chr10	78669784/C//T	synonymous	0	1
PHLDB1	chr11	118498087/GT//G	nonsynonymous	0	1
BAZ1A	chr14	35264914/T//C	nonsynonymous	0	1
AVEN	chr15	34163232/CTA//C	nonsynonymous	0	1
ABCC6	chr16	16315537/AG//A	nonsynonymous	0	1
PSMD11	chr17	30771580/GTC//G	nonsynonymous	0	1
CCL5	chr17	34207330/T//C	synonymous	0	1
PDE4C	chr19	18331984/A//G	nonsynonymous	0	1
WDR87	chr19	38376445/T//C	synonymous	0	1
POTED	chr21	14982621/G//C	nonsynonymous	0	1
DYRK1A	chr21	38884639/C//T	synonymous	0	1
COMT	chr22	19956272/C//G	synonymous	0	1
STAG2	chrX	123179101/C//T	nonsynonymous	0	1
CHD5	chr1	6212507/C//T	nonsynonymous	1	0
H6PD	chr1	9324725/C//A	nonsynonymous	1	0
MFAP2	chr1	17301829/C//T	nonsynonymous	1	0
PTAFR	chr1	28476568/G//A	nonsynonymous	1	0
MANEAL	chr1	38265374/G//A	synonymous	1	0
SCP2	chr1	53504615/C//T	synonymous	1	0
LEPR	chr1	66036390/G//A	nonsynonymous	1	0
OTUD7B	chr1	149916153/C//T	nonsynonymous	1	0
FLG2	chr1	152327673/T//A	synonymous	1	0
SHE	chr1	154461721/C//T	nonsynonymous	1	0
RC3H1	chr1	173930299/C//T	nonsynonymous	1	0
AGBL5	chr2	27278796/C//T	synonymous	1	0
STRN	chr2	37111134/G//A	nonsynonymous	1	0
TTN	chr2	179476813/A//C	synonymous	1	0
COL5A2	chr2	189898826/G//A	synonymous	1	0
OBSL1	chr2	220423977/C//T	nonsynonymous	1	0
TMPPE	chr3	33134833/G//A	synonymous	1	0
CELSR3	chr3	48697673/G//A	nonsynonymous	1	0
STAB1	chr3	52557672/C//T	nonsynonymous	1	0
ERC2	chr3	55922488/G//A	synonymous	1	0
ATR	chr3	142177876/C//T	nonsynonymous	1	0
EVC2	chr4	5576419/C//G	nonsynonymous	1	0
TBC1D14	chr4	6925581/C//T	synonymous	1	0
C4orf22	chr4	81884889/G//T	synonymous	1	0
UNC5C	chr4	96199454/C//A	nonsynonymous	1	0
USP53	chr4	120189443/C//T	nonsynonymous	1	0
KLHL2	chr4	166129016/G//A	synonymous	1	0
DNAH5	chr5	13735348/G//A	nonsynonymous	1	0
TRIO	chr5	14369586/C//T	nonsynonymous	1	0
CD180	chr5	66481798/T//C	synonymous	1	0
RASA1	chr5	86685331/G//A	nonsynonymous	1	0
JADE2	chr5	133873748/G//A	nonsynonymous	1	0
PCDHGA9	chr5	140783737/G//A	synonymous	1	0
GABRA1	chr5	161302638/A//T	nonsynonymous	1	0

PSMB9	chr6	32823941/C//T	synonymous	1	0
GRIK2	chr6	101847196/C//T	nonsynonymous	1	0
ABCB5	chr7	20689758/G//A	synonymous	1	0
CCDC129	chr7	31594123/G//T	synonymous	1	0
GLI3	chr7	42005049/C//T	nonsynonymous	1	0
NACAD	chr7	45124497/C//T	nonsynonymous	1	0
BAZ1B	chr7	72858318/T//G	nonsynonymous	1	0
PRSS3P2	chr7	142479915/T//C	synonymous	1	0
ZNF862	chr7	149559068/G//A	nonsynonymous	1	0
ZNF705G	chr8	7215769/T//C	nonsynonymous	1	0
NCOA2	chr8	71025784/A//G	synonymous	1	0
KLHL38	chr8	124664458/C//T	nonsynonymous	1	0
ANKRD18A	chr9	38596183/G//C	nonsynonymous	1	0
C9orf135	chr9	72471585/G//A	nonsynonymous	1	0
GOLGA2	chr9	131021523/C//T	nonsynonymous	1	0
FUBP3	chr9	133499103/G//T	synonymous	1	0
PITRM1	chr10	3181098/T//G	nonsynonymous	1	0
WDFY4	chr10	49986708/C//A	nonsynonymous	1	0
C10orf53	chr10	50902599/T//C	nonsynonymous	1	0
SGPL1	chr10	72633346/A//C	nonsynonymous	1	0
COX15	chr10	101487284/A//G	synonymous	1	0
C10orf76	chr10	103607202/C//A	synonymous	1	0
AFAP1L2	chr10	116060437/C//T	nonsynonymous	1	0
FAM204A	chr10	120095930/T//C	synonymous	1	0
DOCK1	chr10	129249705/T//C	synonymous	1	0
CNGA4	chr11	6261451/C//T	nonsynonymous	1	0
C11orf49	chr11	47179457/A//G	synonymous	1	0
	chr11	85623734/T//C	synonymous	1	0
FAT3	chr11	92086555/C//T	nonsynonymous	1	0
FOXRED1	chr11	126143248/C//T	synonymous	1	0
B4GALNT3	chr12	645417/AG//A	nonsynonymous	1	0
KIF21A	chr12	39763833/G//GC	synonymous	1	0
METTL25	chr12	82793062/TAAAG//T	nonsynonymous	1	0
ACTR6	chr12	100613796/G//A	synonymous	1	0
FNDC3A	chr13	49741419/A//G	synonymous	1	0
PCDH8	chr13	53418998/G//A	synonymous	1	0
NALCN	chr13	101714451/C//T	nonsynonymous	1	0
CCDC168	chr13	103397221/A//G	synonymous	1	0
TNFSF13B	chr13	108959287/C//T	synonymous	1	0
FAM161B	chr14	74411118/T//G	nonsynonymous	1	0
CHGA	chr14	93397608/G//T	nonsynonymous	1	0
AQR	chr15	35202498/C//T	nonsynonymous	1	0
SPTBN5	chr15	42174905/C//T	nonsynonymous	1	0
ATP8B4	chr15	50264849/G//A	synonymous	1	0
DNAH3	chr16	20976493/G//A	nonsynonymous	1	0
SETD1A	chr16	30972657/C//T	nonsynonymous	1	0
CES1	chr16	55857480/C//T	nonsynonymous	1	0
CRISPLD2	chr16	84872243/C//T	nonsynonymous	1	0
NLGN2	chr17	7320721/G//A	nonsynonymous	1	0
DHRS13	chr17	27228235/A//T	nonsynonymous	1	0
NF1	chr17	29560133/C//A	synonymous	1	0
SLFN14	chr17	33884942/C//T	nonsynonymous	1	0
ACACA	chr17	35581967/G//A	synonymous	1	0
SRCIN1	chr17	36717944/G//A	synonymous	1	0
ERBB2	chr17	37883719/C//T	nonsynonymous	1	0
COG1	chr17	71189223/A//G	synonymous	1	0
CCDC102B	chr18	66678211/G//C	nonsynonymous	1	0
SBNO2	chr19	1113634/T//A	nonsynonymous	1	0
COL5A3	chr19	10090042/G//T	nonsynonymous	1	0
DNMT1	chr19	10277298/G//A	synonymous	1	0
ITPKC	chr19	41239150/C//T	nonsynonymous	1	0
ZNF283	chr19	44351423/C//T	nonsynonymous	1	0
MYH14	chr19	50810310/C//T	nonsynonymous	1	0
KIR3DL2	chr19	55363644/G//A	nonsynonymous	1	0
SBK2	chr19	56041354/G//A	nonsynonymous	1	0
NLRP4	chr19	56369450/G//A	nonsynonymous	1	0
ZNF329	chr19	58640467/A//G	nonsynonymous	1	0
ZNF274	chr19	58723898/C//T	nonsynonymous	1	0
HELZ2	chr20	62194317/G//A	nonsynonymous	1	0
GART	chr21	34878379/G//A	nonsynonymous	1	0

TMPRSS2	chr21	42852430/C//T	nonsynonymous	1	0
COL6A2	chr21	47532733/G//A	nonsynonymous	1	0
KDELR3	chr22	38864230/G//A	synonymous	1	0
RBMXL3	chrX	114424164/G//A	nonsynonymous	1	0
DKC1	chrX	153993212/C//T	nonsynonymous	1	0
GLMN	chr1	92713437/T//C	nonsynonymous	1	0
DPYD	chr1	98060621/T//C	nonsynonymous	1	0
KIT	chr4	55593610/T//A	nonsynonymous	1	0
ORAI2	chr7	102087372/T//C	nonsynonymous	1	0
DCAF4L2	chr8	88885315/C//T	synonymous	1	0
BARX1	chr9	96714528/A//C	nonsynonymous	1	0
BTAF1	chr10	93773457/T//C	nonsynonymous	1	0
JAKMIP3	chr10	133931086/G//T	synonymous	1	0
SMCO3	chr12	14959140/G//A	nonsynonymous	1	0
SLC8B1	chr12	113754385/A//G	synonymous	1	0
GCN1	chr12	120611959/T//G	nonsynonymous	1	0
COG6	chr13	40229871/A//G	nonsynonymous	1	0
HYDIN	chr16	70942291/A//G	synonymous	1	0
SNORA21	chr17	37009236/T//C	synonymous	1	0
ZNF236	chr18	74593374/C//G	synonymous	1	0
ZNF547	chr19	57888697/CAGCACATCTTCACCA	nonsynonymous	1	0
RIBC1	chrX	53456919/A//G	nonsynonymous	1	0
ZBTB17	chr1	16271244/T//C	nonsynonymous	0	1
C1orf112	chr1	169796282/T//TA	nonsynonymous	0	1
LAX1	chr1	203743233/T//C	synonymous	0	1
ZNF678	chr1	227842973/G//T	nonsynonymous	0	1
APOB	chr2	21263811/TG//T	nonsynonymous	0	1
ZAP70	chr2	98351805/A//T	nonsynonymous	0	1
EVX2	chr2	176948135/C//T	nonsynonymous	0	1
HDAC4	chr2	240033295/C//T	synonymous	0	1
NCAPG	chr4	17826628/T//C	nonsynonymous	0	1
AFF1	chr4	88036350/C//T	nonsynonymous	0	1
TENM3	chr4	183696228/C//T	synonymous	0	1
ADAMTS2	chr5	178634713/G//A	nonsynonymous	0	1
PUS7	chr7	105103066/A//G	nonsynonymous	0	1
LZTS1	chr8	20110449/C//T	synonymous	0	1
ZFHX4	chr8	77690462/G//T	nonsynonymous	0	1
DDX50	chr10	70706170/A//G	synonymous	0	1
MCU	chr10	74620223/T//C	nonsynonymous	0	1
BAG3	chr10	121429451/G//A	nonsynonymous	0	1
OSBPL5	chr11	3111908/G//A	nonsynonymous	0	1
MYOD1	chr11	17741923/C//T	synonymous	0	1
NBEA	chr13	35632917/C//T	nonsynonymous	0	1
FANCM	chr14	45667892/T//C	nonsynonymous	0	1
RBM25	chr14	73569928/A//G	nonsynonymous	0	1
KNL1	chr15	40907540/A//G	nonsynonymous	0	1
STARD9	chr15	42983333/G//A	nonsynonymous	0	1
DUOX1	chr15	45454025/A//G	nonsynonymous	0	1
VPS13C	chr15	62305266/G//A	nonsynonymous	0	1
ADAMTSL3	chr15	84581991/C//T	synonymous	0	1
PDE8A	chr15	85669608/A//G	synonymous	0	1
CACNG3	chr16	24372949/G//A	nonsynonymous	0	1
ZNF768	chr16	30536217/G//A	nonsynonymous	0	1
HSD3B7	chr16	30998269/G//A	nonsynonymous	0	1
ZNF532	chr18	56586480/A//G	nonsynonymous	0	1
UBXN6	chr19	4454018/T//C	synonymous	0	1
PRX	chr19	40900428/G//A	synonymous	0	1
ZNF880	chr19	52888071/G//T	nonsynonymous	0	1
PIM3	chr22	50356733/T//C	synonymous	0	1
ATRX	chrX	76938655/T//C	nonsynonymous	0	1
COL4A5	chrX	107936120/C//G	synonymous	0	1
ERICH6	chr3	150419932/T//C	nonsynonymous	0	1
UGT2B28	chr4	70146901/A//G	nonsynonymous	0	1
HHIP	chr4	145580881/G//A	nonsynonymous	0	1
AFF4	chr5	132270046/A//G	synonymous	0	1
CLVS2	chr6	123319002/T//C	nonsynonymous	0	1
FAM120B	chr6	170657386/G//A	nonsynonymous	0	1
OR4C16	chr11	55339875/C//T	nonsynonymous	0	1
ALKBH8	chr11	107424662/A//G	nonsynonymous	0	1
GALNS	chr16	88904164/A//G	synonymous	0	1

SALL3	chr18	76754108/C//T	nonsynonymous	0	1
ONECUT3	chr19	1775286/A//G	nonsynonymous	0	1
CD209	chr19	7808070/G//A	nonsynonymous	0	1
ST7L	chr1	113153521/T//C	synonymous	0	1
TCHH	chr1	152081651/T//C	nonsynonymous	0	1
HSPA6	chr1	161494828/G//A	nonsynonymous	0	1
CTSE	chr1	206325291/A//G	synonymous	0	1
WDR43	chr2	29136953/A//G	nonsynonymous	0	1
PLSCR2	chr3	146167020/G//A	synonymous	0	1
CXCL8	chr4	74607731/T//C	nonsynonymous	0	1
RADIL	chr7	4845300/T//C	synonymous	0	1
CCDC146	chr7	76908116/A//G	synonymous	0	1
MUC17	chr7	100681292/C//A	nonsynonymous	0	1
UBAP1	chr9	34241774/A//G	nonsynonymous	0	1
DDB2	chr11	47238437/T//C	nonsynonymous	0	1
MAP6	chr11	75379229/C//T	synonymous	0	1
MAP6	chr11	75379269/G//A	nonsynonymous	0	1
KDM5A	chr12	443534/C//CG	nonsynonymous	0	1
GJA3	chr13	20716597/C//T	synonymous	0	1
MDGA2	chr14	47351327/G//A	nonsynonymous	0	1
ADCY9	chr16	4016877/A//G	synonymous	0	1
CNP	chr17	40120522/T//C	nonsynonymous	0	1
TBX21	chr17	45822704/G//C	nonsynonymous	0	1
FADS6	chr17	72889527/A//G	nonsynonymous	0	1
CACNA1A	chr19	13397325/A//G	nonsynonymous	0	1
CBLC	chr19	45293281/A//G	nonsynonymous	0	1
EMID1	chr22	29622523/A//G	nonsynonymous	0	1
NPPA	chr1	11907422/C//T	synonymous	1	0
PLEKHM2	chr1	16058989/G//A	nonsynonymous	1	0
TMEM39B	chr1	32560430/C//G	nonsynonymous	1	0
MPL	chr1	43818424/G//A	nonsynonymous	1	0
GBP5	chr1	89730585/C//A	nonsynonymous	1	0
AMY2B	chr1	104117020/A//G	synonymous	1	0
DENND2C	chr1	115130018/C//T	synonymous	1	0
PTGFRN	chr1	117503952/C//T	nonsynonymous	1	0
SETDB1	chr1	150923423/C//T	synonymous	1	0
TDRKH	chr1	151747253/C//T	synonymous	1	0
HRNR	chr1	152193217/G//A	synonymous	1	0
CREB3L4	chr1	153945514/C//T	nonsynonymous	1	0
ASH1L	chr1	155449291/C//T	nonsynonymous	1	0
CD48	chr1	160654960/G//A	synonymous	1	0
ILDR2	chr1	166890167/C//T	nonsynonymous	1	0
QSOX1	chr1	180165695/C//T	synonymous	1	0
CFHR1	chr1	196794617/T//G	nonsynonymous	1	0
SYT2	chr1	202571661/C//T	nonsynonymous	1	0
SLC30A3	chr2	27479356/G//A	nonsynonymous	1	0
HEATR5B	chr2	37259911/G//A	synonymous	1	0
ABCG8	chr2	44100976/G//A	nonsynonymous	1	0
KCNK12	chr2	47748156/C//T	nonsynonymous	1	0
DYSF	chr2	71780148/T//A	nonsynonymous	1	0
FAM178B	chr2	97559765/C//T	synonymous	1	0
INPP4A	chr2	99163134/C//G	nonsynonymous	1	0
MYO7B	chr2	128381808/C//T	synonymous	1	0
EVX2	chr2	176948362/C//T	nonsynonymous	1	0
FSIP2	chr2	186660301/A//T	nonsynonymous	1	0
MFSD6	chr2	191301934/C//A	nonsynonymous	1	0
CDK15	chr2	202698592/C//T	nonsynonymous	1	0
PTPRN	chr2	220174060/C//T	synonymous	1	0
ITPR1	chr3	4699845/G//A	nonsynonymous	1	0
BAP1	chr3	52443744/A//G	nonsynonymous	1	0
ABI3BP	chr3	100527026/T//C	nonsynonymous	1	0
POLQ	chr3	121179010/G//A	nonsynonymous	1	0
MYLK	chr3	123366171/G//A	nonsynonymous	1	0
PLXNA1	chr3	126722211/C//T	synonymous	1	0
ARHGEF26	chr3	153847382/A//C	nonsynonymous	1	0
MCCC1	chr3	182740248/G//A	nonsynonymous	1	0
ECE2	chr3	184009919/C//T	nonsynonymous	1	0
POLN	chr4	2200402/G//A	nonsynonymous	1	0
KIAA0232	chr4	6865691/C//T	synonymous	1	0
CCDC149	chr4	24838995/C//A	nonsynonymous	1	0

PLEKHG4B	chr5	162961/T//C	nonsynonymous	1	0
IRX4	chr5	1878646/C//T	nonsynonymous	1	0
FBXL7	chr5	15937053/T//A	nonsynonymous	1	0
ERBIN	chr5	65288598/C//T	nonsynonymous	1	0
PCDHA13	chr5	140263557/C//T	synonymous	1	0
PCDHB16	chr5	140562537/G//A	nonsynonymous	1	0
PCDHGB4	chr5	140768431/C//T	nonsynonymous	1	0
PCDHGB7	chr5	140797794/T//C	nonsynonymous	1	0
RELL2	chr5	141019113/C//T	nonsynonymous	1	0
RBM27	chr5	145648798/G//A	nonsynonymous	1	0
HTR4	chr5	147889584/C//A	nonsynonymous	1	0
TNIP1	chr5	150411928/C//T	nonsynonymous	1	0
DOCK2	chr5	169506060/G//C	nonsynonymous	1	0
MBOAT1	chr6	20113247/G//T	synonymous	1	0
HDGFL1	chr6	22570420/G//A	nonsynonymous	1	0
PRRC2A	chr6	31599035/G//A	nonsynonymous	1	0
TNXB	chr6	32024600/T//C	nonsynonymous	1	0
BRPF3	chr6	36182160/A//T	nonsynonymous	1	0
BMP5	chr6	55620361/A//T	nonsynonymous	1	0
SESN1	chr6	109315684/ACTCT//A	nonsynonymous	1	0
COL10A1	chr6	116441549/T//A	nonsynonymous	1	0
TAGAP	chr6	159456894/C//A	nonsynonymous	1	0
TMEM184A	chr7	1588220/C//T	nonsynonymous	1	0
	chr7	34917702/G//A	synonymous	1	0
GLI3	chr7	42005604/G//A	nonsynonymous	1	0
STYXL1	chr7	75659845/G//C	synonymous	1	0
PEG10	chr7	94293470/C//T	nonsynonymous	1	0
TFR2	chr7	100218589/C//T	nonsynonymous	1	0
NOBOX	chr7	144098359/C//A	nonsynonymous	1	0
GIMAP1	chr7	150417247/G//A	nonsynonymous	1	0
SLC39A14	chr8	22273438/G//A	nonsynonymous	1	0
TNFRSF10A	chr8	23049319/A//C	nonsynonymous	1	0
SCARA3	chr8	27516790/A//T	nonsynonymous	1	0
DDHD2	chr8	38110500/G//A	nonsynonymous	1	0
ZFHX4	chr8	77617081/C//T	nonsynonymous	1	0
TSPYL5	chr8	98289299/G//C	nonsynonymous	1	0
DCSTAMP	chr8	105361214/T//G	nonsynonymous	1	0
ADGRB1	chr8	143602278/C//T	nonsynonymous	1	0
CPSF1	chr8	145625083/G//A	synonymous	1	0
KIFC2	chr8	145694882/G//A	nonsynonymous	1	0
IFNA5	chr9	21305261/A//C	synonymous	1	0
IDNK	chr9	86243103/G//A	nonsynonymous	1	0
SMC2	chr9	106889690/C//G	nonsynonymous	1	0
OR13C3	chr9	107298301/C//T	nonsynonymous	1	0
AIF1L	chr9	133995718/C//T	synonymous	1	0
INPP5E	chr9	139329284/C//T	nonsynonymous	1	0
SEC16A	chr9	139366460/C//T	nonsynonymous	1	0
NOTCH1	chr9	139401282/G//A	nonsynonymous	1	0
ABCA2	chr9	139915179/G//A	nonsynonymous	1	0
WDR37	chr10	1175265/C//T	nonsynonymous	1	0
ITIH5	chr10	7679200/G//A	nonsynonymous	1	0
CUBN	chr10	16882537/G//A	synonymous	1	0
WDFY4	chr10	49997923/A//T	nonsynonymous	1	0
ITPRIP	chr10	106074472/G//A	synonymous	1	0
	chr10	126715245/C//T	synonymous	1	0
OR52E2	chr11	5080489/G//T	nonsynonymous	1	0
LYVE1	chr11	10585749/C//A	nonsynonymous	1	0
OR4A47	chr11	48511134/C//G	nonsynonymous	1	0
OR8J3	chr11	55904785/G//A	nonsynonymous	1	0
OR8K1	chr11	56114169/A//G	nonsynonymous	1	0
OR1S2	chr11	57970709/T//C	synonymous	1	0
SLC22A12	chr11	64359388/G//A	nonsynonymous	1	0
CDC42BPG	chr11	64603955/C//A	nonsynonymous	1	0
TMEM151A	chr11	66062570/G//T	nonsynonymous	1	0
CTTN	chr11	70263206/C//T	nonsynonymous	1	0
ARHGEF17	chr11	73063876/G//A	nonsynonymous	1	0
MAP6	chr11	75298665/A//T	nonsynonymous	1	0
FOLH1B	chr11	89405072/G//T	nonsynonymous	1	0
NCAM1	chr11	113105857/C//T	nonsynonymous	1	0
BACE1	chr11	117163828/T//C	nonsynonymous	1	0

BACE1	chr11	117163830/ATAATACCAC//A	nonsynonymous	1	0
RPS25	chr11	118888166/G//A	synonymous	1	0
BLID	chr11	121986642/A//G	synonymous	1	0
OR8D4	chr11	123777849/G//A	synonymous	1	0
IGSF9B	chr11	133789874/C//T	nonsynonymous	1	0
IQSEC3	chr12	247859/G//A	nonsynonymous	1	0
CACNA2D4	chr12	1992094/C//T	nonsynonymous	1	0
A2M	chr12	9225467/C//T	nonsynonymous	1	0
EPS8	chr12	15793674/A//G	nonsynonymous	1	0
SLCO1B3	chr12	20968669/A//G	synonymous	1	0
OVCH1	chr12	29596303/T//A	nonsynonymous	1	0
LRRK2	chr12	40693045/G//A	nonsynonymous	1	0
SCAF11	chr12	46342225/A//G	synonymous	1	0
SLC4A8	chr12	51852393/A//C	nonsynonymous	1	0
ZNF385A	chr12	54765375/G//T	synonymous	1	0
GPR182	chr12	57389371/C//T	synonymous	1	0
TBK1	chr12	64882283/G//A	nonsynonymous	1	0
FZD10	chr12	130648358/G//A	nonsynonymous	1	0
PDS5B	chr13	33261315/T//A	nonsynonymous	1	0
FREM2	chr13	39261908/G//A	nonsynonymous	1	0
VWA8	chr13	42249395/GACTTCTATATA//G	nonsynonymous	1	0
RASA3	chr13	114751165/T//C	nonsynonymous	1	0
RNASE11	chr14	21058393/A//T	synonymous	1	0
PPP1R13B	chr14	104208420/G//C	nonsynonymous	1	0
GCHFR	chr15	41056294/C//T	synonymous	1	0
MYO9A	chr15	72192034/C//T	nonsynonymous	1	0
ODF3L1	chr15	76018502/CTCCAACCTGCGT//C	nonsynonymous	1	0
LRRK1	chr15	101601402/A//T	nonsynonymous	1	0
GRIN2A	chr16	10274008/G//A	synonymous	1	0
KCTD13	chr16	29918255/C//T	nonsynonymous	1	0
NOD2	chr16	50756560/G//A	synonymous	1	0
CMTM2	chr16	66622002/C//T	nonsynonymous	1	0
TP53	chr17	7577106/G//A	nonsynonymous	1	0
SPDYE4	chr17	8660666/G//A	nonsynonymous	1	0
NLK	chr17	26518108/G//A	nonsynonymous	1	0
KRT33B	chr17	39521752/G//A	synonymous	1	0
TANC2	chr17	61498656/G//A	synonymous	1	0
CDH20	chr18	59221875/C//T	nonsynonymous	1	0
NETO1	chr18	70526207/A//C	nonsynonymous	1	0
SMARCA4	chr19	11107217/C//T	synonymous	1	0
MAST1	chr19	12958617/AGCCTTCGGGCACAG	nonsynonymous	1	0
CEBPA	chr19	33792445/G//A	synonymous	1	0
FFAR2	chr19	35941124/G//A	nonsynonymous	1	0
PLEKHG2	chr19	39915013/G//T	nonsynonymous	1	0
CYP2B6	chr19	41512843/C//G	nonsynonymous	1	0
CYP2B6	chr19	41512851/G//A	nonsynonymous	1	0
ZNF574	chr19	42584308/A//G	nonsynonymous	1	0
BCAT2	chr19	49300484/T//G	nonsynonymous	1	0
GFY	chr19	49930708/A//G	nonsynonymous	1	0
NLRP12	chr19	54314260/G//A	nonsynonymous	1	0
SLC4A11	chr20	3211790/C//T	synonymous	1	0
SRC	chr20	36012673/C//T	synonymous	1	0
KIAA1755	chr20	36854140/T//A	nonsynonymous	1	0
SLC32A1	chr20	37356840/G//A	nonsynonymous	1	0
KCNG1	chr20	49620963/G//A	synonymous	1	0
	chr20	57415777/G//T	synonymous	1	0
TPTE	chr21	10942951/T//G	nonsynonymous	1	0
DSCAM	chr21	41710035/G//A	synonymous	1	0
COL18A1	chr21	46930131/G//A	nonsynonymous	1	0
COL6A1	chr21	47412686/C//T	nonsynonymous	1	0
MOSPD2	chrX	14934432/A//G	nonsynonymous	1	0
HDAC6	chrX	48682366/G//C	nonsynonymous	1	0
DGKK	chrX	50123071/G//A	nonsynonymous	1	0
TRO	chrX	54955077/C//T	synonymous	1	0
CPXCR1	chrX	88008985/C//T	synonymous	1	0
	chrX	119738707/A//T	synonymous	1	0
BGN	chrX	152772062/G//C	nonsynonymous	1	0
TMCO2	chr1	40713707/GTC//G	nonsynonymous	1	0
HECTD3	chr1	45476788/G//A	nonsynonymous	1	0
OTUD7B	chr1	149936239/G//A	nonsynonymous	1	0

HAPLN2	chr1	156593663/G//C	synonymous	1	0
ASTN1	chr1	176915236/A//T	nonsynonymous	1	0
SPATA17	chr1	217804736/C//T	nonsynonymous	1	0
CHML	chr1	241798781/A//C	nonsynonymous	1	0
LRP1B	chr2	140992451/G//A	synonymous	1	0
C3orf35	chr3	37458982/A//AT	nonsynonymous	1	0
KIAA1109	chr4	123140626/T//C	synonymous	1	0
WWC2	chr4	184186916/A//G	synonymous	1	0
SNCAIP	chr5	121787043/G//A	nonsynonymous	1	0
PCDHGA3	chr5	140725053/G//A	nonsynonymous	1	0
ZNF727	chr7	63529900/A//G	nonsynonymous	1	0
POMZP3	chr7	76241077/T//C	synonymous	1	0
MUC17	chr7	100676309/A//T	nonsynonymous	1	0
KMT2C	chr7	151855981/G//T	nonsynonymous	1	0
SMU1	chr9	33056166/G//C	nonsynonymous	1	0
CNTRL	chr9	123931981/T//G	nonsynonymous	1	0
STRBP	chr9	125920339/A//T	nonsynonymous	1	0
	chr9	138393568/C//T	synonymous	1	0
ACTR1A	chr10	104241820/A//G	nonsynonymous	1	0
TRIM64C	chr11	49080646/G//A	nonsynonymous	1	0
NELL2	chr12	44913920/GAC//G	nonsynonymous	1	0
HELB	chr12	66698565/C//G	nonsynonymous	1	0
MYCBP2	chr13	77755956/C//T	synonymous	1	0
HS3ST2	chr16	22826085/G//A	nonsynonymous	1	0
MEIOC	chr17	42745600/A//G	nonsynonymous	1	0
TMPRSS9	chr19	2396559/C//T	synonymous	1	0
FEM1A	chr19	4793002/A//G	nonsynonymous	1	0
ARFGEF2	chr20	47605115/A//G	nonsynonymous	1	0
XPNPEP3	chr22	41253221/C//T	synonymous	1	0
B3GALT6	chr1	1168669/C//T	synonymous	0	1
DISP3	chr1	11561071/T//A	nonsynonymous	0	1
ZRANB2	chr1	71546772/G//A	synonymous	0	1
COL24A1	chr1	86554881/G//C	synonymous	0	1
DNAH7	chr2	196774754/A//G	synonymous	0	1
SRGAP3	chr3	9068665/A//G	synonymous	0	1
POLQ	chr3	121263724/C//T	nonsynonymous	0	1
C3orf70	chr3	184800761/G//A	synonymous	0	1
PRDM9	chr5	23526431/T//C	nonsynonymous	0	1
CDH9	chr5	26885845/C//T	nonsynonymous	0	1
F2RL2	chr5	75913625/T//C	nonsynonymous	0	1
SLC35D3	chr6	137245656/A//G	nonsynonymous	0	1
ABHD11	chr7	73152048/A//G	synonymous	0	1
MUC17	chr7	100681244/A//G	nonsynonymous	0	1
CSMD1	chr8	3009026/C//T	nonsynonymous	0	1
DOCK5	chr8	25177166/C//T	nonsynonymous	0	1
ZBTB26	chr9	125682099/T//C	nonsynonymous	0	1
CUL2	chr10	35360234/T//C	synonymous	0	1
HK1	chr10	71142545/C//T	nonsynonymous	0	1
MARCH5	chr10	94070962/A//G	nonsynonymous	0	1
CDK2AP2	chr11	67275709/C//A	synonymous	0	1
PRH1	chr12	11034872/G//A	nonsynonymous	0	1
FAM186A	chr12	50744528/G//A	synonymous	0	1
ENOX1	chr13	43987121/G//T	synonymous	0	1
ZSCAN29	chr15	43661940/C//T	nonsynonymous	0	1
FMNL1	chr17	43315934/T//C	nonsynonymous	0	1
ZNF57	chr19	2917793/T//C	nonsynonymous	0	1
ZNF57	chr19	2917807/A//C	nonsynonymous	0	1
PLIN4	chr19	4511213/C//G	nonsynonymous	0	1
PPP6R1	chr19	55743204/C//T	nonsynonymous	0	1
SYNGR1	chr22	39772130/C//A	nonsynonymous	0	1
ZNF674	chrX	46359599/C//T	synonymous	0	1
PARS2	chr1	55223403/G//A	synonymous	1	0
SLC26A9	chr1	205890772/G//A	synonymous	1	0
GALNT14	chr2	31155034/C//T	nonsynonymous	1	0
USP34	chr2	61575022/ATGG//A	nonsynonymous	1	0
FEV	chr2	219846695/C//T	synonymous	1	0
PPP4R2	chr3	73046176/A//G	synonymous	1	0
CLOCK	chr4	56315622/T//C	nonsynonymous	1	0
POLR2B	chr4	57891137/A//T	nonsynonymous	1	0
RAPGEF6	chr5	130782227/C//T	nonsynonymous	1	0

TAAR9	chr6	132860062/G//A	nonsynonymous	1	0
TBX20	chr7	35288458/CAG//C	synonymous	1	0
DCLRE1C	chr10	14950898/T//C	nonsynonymous	1	0
PRKG1	chr10	54041962/CTT//C	nonsynonymous	1	0
OR4A5	chr11	51411527/T//C	nonsynonymous	1	0
GIF	chr11	59599212/C//T	synonymous	1	0
	chr11	65338051/A//G	synonymous	1	0
AMIGO2	chr12	47471582/T//A	nonsynonymous	1	0
NFE2	chr12	54686443/T//C	synonymous	1	0
TMEM132B	chr12	126135412/G//A	synonymous	1	0
NBEA	chr13	36180639/A//T	nonsynonymous	1	0
ACIN1	chr14	23548806/T//C	nonsynonymous	1	0
HECTD1	chr14	31576161/G//C	nonsynonymous	1	0
NRDE2	chr14	90754649/T//C	nonsynonymous	1	0
TRPM1	chr15	31334283/A//G	nonsynonymous	1	0
NEO1	chr15	73581512/T//C	synonymous	1	0
IREB2	chr15	78730661/GA//G	synonymous	1	0
SH3GL1	chr19	4363391/T//C	nonsynonymous	1	0
DOCK6	chr19	11311047/G//A	nonsynonymous	1	0
CPAMD8	chr19	17085971/C//T	nonsynonymous	1	0
FAM47A	chrX	34148721/C//T	nonsynonymous	1	0
RPGR	chrX	38186752/A//G	synonymous	1	0
SPANXN3	chrX	142596954/T//A	nonsynonymous	1	0
SEMA4F	chr2	74902121/C//T	nonsynonymous	0	1
ZNF804A	chr2	185802754/C//A	nonsynonymous	0	1
CLGN	chr4	141313486/G//A	nonsynonymous	0	1
DTNBP1	chr6	15533633/A//G	synonymous	0	1
WASF1	chr6	110423026/C//T	synonymous	0	1
PCLO	chr7	82764260/T//C	nonsynonymous	0	1
ZNF425	chr7	148802240/A//G	synonymous	0	1
TJP2	chr9	71851980/C//T	nonsynonymous	0	1
SPATA31E1	chr9	90499929/C//T	nonsynonymous	0	1
FAM186A	chr12	50754631/T//C	nonsynonymous	0	1
RPLP0	chr12	120634677/TGGCAGCAGCCACA(	nonsynonymous	0	1
SPNS2	chr17	4439624/G//A	nonsynonymous	0	1
GIPC3	chr19	3586966/G//A	nonsynonymous	0	1
PRODH2	chr19	36293934/C//T	nonsynonymous	0	1
	chrX	131803182/A//C	synonymous	0	1
GPR153	chr1	6310542/G//A	synonymous	0	1
CR1	chr1	207758050/T//C	nonsynonymous	0	1
TRMT10C	chr3	101284533/A//G	nonsynonymous	0	1
RAPGEF1	chr9	134459746/C//T	synonymous	0	1
OR5T1	chr11	56043679/G//A	nonsynonymous	0	1
HCRTR1	chr1	32092517/G//A	nonsynonymous	0	1
AKIRIN1	chr1	39469797/T//C	synonymous	0	1
FPGT	chr1	74670339/A//G	nonsynonymous	0	1
PPM1J	chr1	113255378/G//A	synonymous	0	1
RGS13	chr1	192617084/G//T	nonsynonymous	0	1
SRP9	chr1	225977131/G//A	synonymous	0	1
RYR2	chr1	237670088/A//T	nonsynonymous	0	1
RYR2	chr1	237947543/C//A	synonymous	0	1
ASAP2	chr2	9531238/A//T	nonsynonymous	0	1
TCF23	chr2	27372158/C//A	nonsynonymous	0	1
FAM179A	chr2	29222324/G//A	synonymous	0	1
BUB1	chr2	111415202/C//G	nonsynonymous	0	1
DDX18	chr2	118579558/G//T	nonsynonymous	0	1
CWC22	chr2	180818987/C//T	nonsynonymous	0	1
COL5A2	chr2	189917491/C//A	nonsynonymous	0	1
HDLBP	chr2	242203873/A//G	nonsynonymous	0	1
UBE2E1	chr3	23848790/C//T	synonymous	0	1
DCLK3	chr3	36778802/G//A	nonsynonymous	0	1
LRIG1	chr3	66448555/T//C	nonsynonymous	0	1
MORC1	chr3	108746613/T//C	synonymous	0	1
TMEM39A	chr3	119155808/G//A	synonymous	0	1
SLC41A3	chr3	125786785/C//T	synonymous	0	1
HPS3	chr3	148863228/A//C	nonsynonymous	0	1
LETM1	chr4	1824714/C//T	nonsynonymous	0	1
DRD5	chr4	9783698/C//T	synonymous	0	1
DRD5	chr4	9784550/G//A	synonymous	0	1
LIAS	chr4	39462474/A//G	nonsynonymous	0	1

TMPRSS11A	chr4	68788489/C//A	nonsynonymous	0	1
MMRN1	chr4	90816521/C//G	synonymous	0	1
CCDC110	chr4	186381255/G//A	synonymous	0	1
ZFP42	chr4	188923974/C//T	synonymous	0	1
ADCY2	chr5	7698487/A//C	nonsynonymous	0	1
DNAH5	chr5	13810221/G//A	nonsynonymous	0	1
FAM159B	chr5	63986531/C//T	synonymous	0	1
APC	chr5	112174682/C//T	nonsynonymous	0	1
PCDHA6	chr5	140209347/C//T	synonymous	0	1
PCDHA8	chr5	140221985/G//A	nonsynonymous	0	1
PCDHAC1	chr5	140307126/G//A	nonsynonymous	0	1
POU4F3	chr5	145719850/G//A	nonsynonymous	0	1
FAM135A	chr6	71212502/A//C	synonymous	0	1
POPDC3	chr6	105609486/C//T	nonsynonymous	0	1
LAMA2	chr6	129823898/A//G	nonsynonymous	0	1
VNN2	chr6	133065614/C//T	nonsynonymous	0	1
THSD7A	chr7	11468741/C//T	nonsynonymous	0	1
AMPH	chr7	38501011/T//C	nonsynonymous	0	1
HIP1	chr7	75168714/T//C	nonsynonymous	0	1
COPS6	chr7	99689400/GCT//G	nonsynonymous	0	1
NRG1	chr8	32453527/A//C	synonymous	0	1
ENPP2	chr8	120581552/A//G	nonsynonymous	0	1
MPDZ	chr9	13123176/G//T	synonymous	0	1
USP6NL	chr10	11551593/C//A	nonsynonymous	0	1
ITGA8	chr10	15714661/G//A	nonsynonymous	0	1
SPAG6	chr10	22676862/G//T	nonsynonymous	0	1
	chr10	34601309/G//C	synonymous	0	1
OR13A1	chr10	45799046/G//A	synonymous	0	1
C10orf71	chr10	50530702/C//T	nonsynonymous	0	1
TNKS2	chr10	93608295/C//A	synonymous	0	1
DNMBP	chr10	101716047/G//A	nonsynonymous	0	1
TACC2	chr10	123843946/C//T	nonsynonymous	0	1
DOCK1	chr10	128780201/A//G	nonsynonymous	0	1
CYP2E1	chr10	135352362/A//T	nonsynonymous	0	1
TRIM49B	chr11	49059206/C//A	nonsynonymous	0	1
OR5D13	chr11	55541284/G//A	nonsynonymous	0	1
TAF6L	chr11	62554636/G//C	nonsynonymous	0	1
CPT1A	chr11	68564405/G//A	synonymous	0	1
HIKESHI	chr11	86056645/C//T	nonsynonymous	0	1
ALKBH8	chr11	107396214/C//T	nonsynonymous	0	1
ARHGAP20	chr11	110501514/C//T	nonsynonymous	0	1
USP28	chr11	113705016/A//G	synonymous	0	1
OR6X1	chr11	123624624/GGTTGCTATGA//G	nonsynonymous	0	1
PLEKHG6	chr12	6436475/G//A	nonsynonymous	0	1
CD163	chr12	7639546/G//A	nonsynonymous	0	1
BCAT1	chr12	24989497/A//G	nonsynonymous	0	1
KMT2D	chr12	49424555/C//T	synonymous	0	1
	chr12	92818457/A//G	synonymous	0	1
GNPTAB	chr12	102159906/G//A	synonymous	0	1
OAS3	chr12	113386732/C//T	nonsynonymous	0	1
TMEM132C	chr12	129190089/C//T	nonsynonymous	0	1
XPO4	chr13	21357798/C//T	synonymous	0	1
SAP18	chr13	21721057/C//T	nonsynonymous	0	1
AMER2	chr13	25745730/C//T	nonsynonymous	0	1
FREM2	chr13	39432107/C//T	synonymous	0	1
	chr13	96086049/G//A	synonymous	0	1
NALCN	chr13	101720386/C//A	nonsynonymous	0	1
TOX4	chr14	21957375/A//G	nonsynonymous	0	1
ITPK1	chr14	93408244/C//T	nonsynonymous	0	1
ALDH1A2	chr15	58306379/T//C	nonsynonymous	0	1
CDH8	chr16	61935181/G//A	nonsynonymous	0	1
ALOX12B	chr17	7983246/C//T	synonymous	0	1
UBE2Z	chr17	46993462/C//G	nonsynonymous	0	1
TSPOAP1	chr17	56386507/T//C	nonsynonymous	0	1
TMEM94	chr17	73494579/G//A	synonymous	0	1
B4GALT6	chr18	29238056/G//A	synonymous	0	1
ATP8B3	chr19	1799980/G//A	synonymous	0	1
FEM1A	chr19	4791935/C//T	synonymous	0	1
	chr19	5687800/G//A	synonymous	0	1
DOCK6	chr19	11332565/A//G	nonsynonymous	0	1

WIZ	chr19	15538265/G//A	synonymous	0	1
LGI4	chr19	35617783/C//T	nonsynonymous	0	1
NANOS2	chr19	46417485/G//C	synonymous	0	1
RPL18	chr19	49118695/G//A	synonymous	0	1
PPFIA3	chr19	49637354/C//T	synonymous	0	1
NLRP12	chr19	54313563/C//T	synonymous	0	1
ZNF584	chr19	58928939/C//T	nonsynonymous	0	1
CDC25B	chr20	3784059/G//A	synonymous	0	1
COL18A1	chr21	46895448/C//T	nonsynonymous	0	1
SHOX	chrX	591574/C//T	synonymous	0	1
PDHA1	chrX	19377042/C//T	nonsynonymous	0	1
DMD	chrX	32841411/C//A	nonsynonymous	0	1
SHROOM4	chrX	50378129/G//A	nonsynonymous	0	1
IQSEC2	chrX	53271080/G//T	synonymous	0	1
DGAT2L6	chrX	69424201/A//T	nonsynonymous	0	1
BCORL1	chrX	129159242/G//A	synonymous	0	1
ATP11C	chrX	138882251/A//C	nonsynonymous	0	1
	chr1	157103884/C//G	synonymous	0	1
DPP4	chr2	162877202/G//C	synonymous	0	1
COL3A1	chr2	189854168/G//T	nonsynonymous	0	1
GDNF	chr5	37834848/C//T	synonymous	0	1
GIN1	chr5	102440306/A//AC	nonsynonymous	0	1
ZNF311	chr6	28963755/T//C	nonsynonymous	0	1
ECHDC1	chr6	127652004/C//T	nonsynonymous	0	1
PURB	chr7	44924879/G//A	synonymous	0	1
C8orf89	chr8	74171655/A//G	synonymous	0	1
ST6GALNAC6	chr9	130649011/T//C	nonsynonymous	0	1
CCDC42	chr17	8647039/G//A	nonsynonymous	0	1
CDC27	chr17	45199943/C//T	synonymous	0	1
ENOSF1	chr18	678713/T//C	nonsynonymous	0	1
HAUS1	chr18	43705706/A//T	synonymous	0	1
SREBF2	chr22	42229302/C//G	nonsynonymous	0	1
GCNA	chrX	70823702/A//G	nonsynonymous	0	1
TDRD5	chr1	179562603/G//C	nonsynonymous	0	1
HMCN1	chr1	186158894/C//T	nonsynonymous	0	1
CHRNG	chr2	233405129/A//G	nonsynonymous	0	1
RARB	chr3	25502811/C//T	synonymous	0	1
LAMB2	chr3	49161966/C//T	synonymous	0	1
PCOLCE2	chr3	142606512/G//T	nonsynonymous	0	1
EPHA5	chr4	66189863/A//T	nonsynonymous	0	1
KIAA0922	chr4	154541914/G//A	nonsynonymous	0	1
FBXL21	chr5	135272438/A//G	nonsynonymous	0	1
GRIA1	chr5	153190725/G//T	nonsynonymous	0	1
EBF1	chr5	158250213/G//A	nonsynonymous	0	1
MDFIC	chr7	114563005/G//A	synonymous	0	1
PLEC	chr8	144995433/G//A	synonymous	0	1
DNAJA1	chr9	33026819/G//T	nonsynonymous	0	1
CHD4	chr12	6711318/C//T	synonymous	0	1
GSX1	chr13	28367040/C//T	synonymous	0	1
CPB2	chr13	46679079/C//T	synonymous	0	1
OXA1L	chr14	23239471/T//C	nonsynonymous	0	1
PSMB11	chr14	23511665/T//C	synonymous	0	1
KRT17	chr17	39775799/C//T	synonymous	0	1
KIF19	chr17	72348091/A//G	nonsynonymous	0	1
SIGLEC12	chr19	52000187/C//T	nonsynonymous	0	1
	chrX	69672457/A//G	synonymous	0	1
NLGN4Y	chrY	16942322/C//T	synonymous	0	1
VWA5B1	chr1	20668960/C//A	synonymous	1	0
HTR1D	chr1	23519991/G//T	nonsynonymous	1	0
PUM1	chr1	31465372/C//A	nonsynonymous	1	0
SV2A	chr1	149881131/C//G	synonymous	1	0
OR10Z1	chr1	158577159/G//T	nonsynonymous	1	0
OR6K2	chr1	158669897/T//G	synonymous	1	0
CD247	chr1	167403267/A//T	nonsynonymous	1	0
CEP350	chr1	180063298/A//T	nonsynonymous	1	0
KCNT2	chr1	196397376/C//A	nonsynonymous	1	0
SNAP47	chr1	227923135/C//T	nonsynonymous	1	0
OR2T34	chr1	248737978/C//G	nonsynonymous	1	0
APOB	chr2	21235191/C//A	nonsynonymous	1	0
USP34	chr2	61605554/T//A	nonsynonymous	1	0

EPC2	chr2	149541191/C//G	nonsynonymous	1	0
INO80D	chr2	206921160/C//A	nonsynonymous	1	0
TADA2B	chr4	7056494/G//T	nonsynonymous	1	0
BMP3	chr4	81952578/A//T	nonsynonymous	1	0
ANK2	chr4	114238917/G//T	synonymous	1	0
NEIL3	chr4	178281779/G//T	nonsynonymous	1	0
MYO10	chr5	16758261/T//TAG	nonsynonymous	1	0
CDH12	chr5	22078575/C//A	nonsynonymous	1	0
CDH10	chr5	24593449/G//A	nonsynonymous	1	0
SPEF2	chr5	35646829/C//G	nonsynonymous	1	0
EGFLAM	chr5	38435326/C//T	synonymous	1	0
HCN1	chr5	45262432/G//A	nonsynonymous	1	0
RASGRF2	chr5	80256768/T//A	nonsynonymous	1	0
SEMA6A	chr5	115782441/G//C	synonymous	1	0
IL3	chr5	131396687/C//G	nonsynonymous	1	0
PCDHA4	chr5	140188982/C//A	nonsynonymous	1	0
PCDHB15	chr5	140625923/C//A	synonymous	1	0
TNXB	chr6	32063521/A//G	synonymous	1	0
CLPS	chr6	35762910/T//A	synonymous	1	0
DNAH8	chr6	38754707/A//G	synonymous	1	0
DOPEY1	chr6	83851689/A//G	nonsynonymous	1	0
LAMA2	chr6	129722460/C//T	nonsynonymous	1	0
ZNF92	chr7	64864461/A//G	synonymous	1	0
LRCH4	chr7	100175450/C//A	nonsynonymous	1	0
DLGAP2	chr8	1496886/C//T	synonymous	1	0
ADAM2	chr8	39624772/T//A	nonsynonymous	1	0
CHRNB3	chr8	42587179/C//A	synonymous	1	0
PXDNL	chr8	52321549/C//A	nonsynonymous	1	0
CA2	chr8	86388044/G//A	synonymous	1	0
ZFPM2	chr8	106813993/T//C	synonymous	1	0
ENPP2	chr8	120569930/C//A	nonsynonymous	1	0
DMRTA1	chr9	22447455/GAGCGCCAGCGCGTC	nonsynonymous	1	0
RASEF	chr9	85615135/G//C	nonsynonymous	1	0
CACNA1B	chr9	141016465/G//A	synonymous	1	0
CUBN	chr10	17107643/C//A	synonymous	1	0
OGDHL	chr10	50960684/C//A	nonsynonymous	1	0
CDH23	chr10	73567730/C//A	nonsynonymous	1	0
NOLC1	chr10	103922036/A//G	synonymous	1	0
PCGF6	chr10	105110675/T//C	nonsynonymous	1	0
CCDC186	chr10	115896990/T//A	synonymous	1	0
MUC5B	chr11	1274040/T//C	nonsynonymous	1	0
PIK3C2A	chr11	17190653/T//A	nonsynonymous	1	0
OR4C6	chr11	55433119/C//T	synonymous	1	0
OR5T1	chr11	56043911/C//A	nonsynonymous	1	0
MS4A4A	chr11	60059727/A//T	nonsynonymous	1	0
GRM5	chr11	88337961/G//C	nonsynonymous	1	0
CASP5	chr11	104868212/C//G	synonymous	1	0
KDM5A	chr12	427465/G//T	synonymous	1	0
PLEKHG6	chr12	6436568/C//A	nonsynonymous	1	0
NR4A1	chr12	52451312/C//A	nonsynonymous	1	0
CSAD	chr12	53553991/A//G	nonsynonymous	1	0
NAV3	chr12	78388609/C//A	nonsynonymous	1	0
PAH	chr12	103271235/C//A	synonymous	1	0
WSCD2	chr12	108620819/C//A	nonsynonymous	1	0
RPH3A	chr12	113285606/T//A	synonymous	1	0
ADGRD1	chr12	131589421/G//T	nonsynonymous	1	0
TMCO3	chr13	114150008/G//T	nonsynonymous	1	0
ADCY4	chr14	24792643/T//C	synonymous	1	0
PPP2R5E	chr14	63856322/C//T	synonymous	1	0
RGS6	chr14	73029475/G//T	synonymous	1	0
OR4N4	chr15	22382855/G//T	nonsynonymous	1	0
UACA	chr15	70960066/G//T	nonsynonymous	1	0
NTRK3	chr15	88476318/C//A	nonsynonymous	1	0
SSTR5	chr16	1129976/G//T	synonymous	1	0
CACNA1H	chr16	1252200/G//A	nonsynonymous	1	0
MEFV	chr16	3304364/G//T	nonsynonymous	1	0
HS3ST2	chr16	22926931/C//A	synonymous	1	0
LCMT1	chr16	25186278/T//A	nonsynonymous	1	0
	chr16	56374872/C//A	synonymous	1	0
AP1G1	chr16	71780585/T//C	nonsynonymous	1	0

TAX1BP3	chr17	3571889/TCGCTGCTTAACAAAG(	synonymous	1	0
FBXO39	chr17	6684205/C//A	nonsynonymous	1	0
ACAP1	chr17	7252458/C//A	nonsynonymous	1	0
ALOX15B	chr17	7950049/G//C	nonsynonymous	1	0
CCDC144NL	chr17	20799219/C//A	nonsynonymous	1	0
LRRC37B	chr17	30348571/C//G	nonsynonymous	1	0
ZPBP2	chr17	38027862/T//C	synonymous	1	0
TUBG2	chr17	40815492/A//C	nonsynonymous	1	0
NBR1	chr17	41338394/C//T	nonsynonymous	1	0
CDC42EP4	chr17	71282614/G//A	nonsynonymous	1	0
USP36	chr17	76799755/T//C	nonsynonymous	1	0
CARD14	chr17	78162192/A//C	nonsynonymous	1	0
STK11	chr19	1220382/C//T	nonsynonymous	1	0
THOP1	chr19	2808262/G//T	synonymous	1	0
MUC16	chr19	9066044/C//A	synonymous	1	0
FBXW9	chr19	12800847/T//A	nonsynonymous	1	0
MAST1	chr19	12976211/G//C	nonsynonymous	1	0
NCAN	chr19	19329754/G//A	nonsynonymous	1	0
ZNF208	chr19	22156995/C//A	nonsynonymous	1	0
CCNE1	chr19	30308172/C//T	synonymous	1	0
PPP2R1A	chr19	52714593/C//T	synonymous	1	0
NLRP2	chr19	55494243/C//A	nonsynonymous	1	0
SLC4A11	chr20	3210383/T//C	nonsynonymous	1	0
KIF16B	chr20	16407878/C//A	nonsynonymous	1	0
PHACTR3	chr20	58330413/G//A	nonsynonymous	1	0
C21orf33	chr21	45564833/G//T	synonymous	1	0
CCT8L2	chr22	17071686/C//T	synonymous	1	0
FRMPD4	chrX	12736441/C//G	nonsynonymous	1	0
CDK16	chrX	47088182/C//T	synonymous	1	0
HUWE1	chrX	53585952/C//A	nonsynonymous	1	0
TCEAL2	chrX	101381932/G//A	nonsynonymous	1	0
PCDH11Y	chrY	4968513/C//A	nonsynonymous	1	0
TDRD5	chr1	179620112/T//C	synonymous	0	1
RGL1	chr1	183854066/C//T	synonymous	0	1
GGPS1	chr1	235506134/T//C	synonymous	0	1
DUSP7	chr3	52090309/G//C	nonsynonymous	0	1
COL6A5	chr3	130110186/C//A	nonsynonymous	0	1
ACTL6B	chr7	100244598/A//G	nonsynonymous	0	1
SERPINE1	chr7	100775226/C//G	synonymous	0	1
MASTL	chr10	27454346/A//G	nonsynonymous	0	1
MS4A10	chr11	60563040/A//G	nonsynonymous	0	1
KMT2D	chr12	49425823/AGCT//A	nonsynonymous	0	1
NFIC	chr19	3425108/A//G	synonymous	0	1
C3	chr19	6720645/A//G	synonymous	0	1
SUGP1	chr19	19387334/A//G	synonymous	0	1
ZNF681	chr19	23926692/C//T	nonsynonymous	0	1
FBXO17	chr19	39440692/C//G	nonsynonymous	0	1
RBM10	chrX	47030582/G//T	nonsynonymous	0	1
SUV39H1	chrX	48558976/C//T	synonymous	0	1
C1orf64	chr1	16332708/C//T	nonsynonymous	0	1
TESK2	chr1	45923247/C//T	nonsynonymous	0	1
IVL	chr1	152882617/G//A	nonsynonymous	0	1
CREB3L4	chr1	153945249/TGAG//T	nonsynonymous	0	1
CRB1	chr1	197396959/T//A	nonsynonymous	0	1
PROX1	chr1	214170220/C//T	synonymous	0	1
TP53BP2	chr1	223988399/C//T	synonymous	0	1
BCL11A	chr2	60687882/G//A	nonsynonymous	0	1
USP34	chr2	61415157/AAAAC//A	synonymous	0	1
USP34	chr2	61441268/C//T	nonsynonymous	0	1
CPS1	chr2	211507306/G//C	nonsynonymous	0	1
CACNA1D	chr3	53757573/C//T	synonymous	0	1
MAGI1	chr3	65342524/G//A	synonymous	0	1
EPHA6	chr3	97466367/G//C	nonsynonymous	0	1
RUBCN	chr3	197409421/C//T	synonymous	0	1
TRIO	chr5	14477072/C//CGTAA	synonymous	0	1
TTC23L	chr5	34864627/T//A	nonsynonymous	0	1
PCDHB10	chr5	140574175/G//C	nonsynonymous	0	1
ARAP3	chr5	141052424/G//C	nonsynonymous	0	1
KIF4B	chr5	154397196/T//G	synonymous	0	1
EBF1	chr5	158526374/T//C	nonsynonymous	0	1

USP49	chr6	41773423/G//A	synonymous	0	1
PTCHD4	chr6	47847063/A//G	nonsynonymous	0	1
PKHD1	chr6	51612715/G//T	synonymous	0	1
SGK1	chr6	134491989/ATCT//A	nonsynonymous	0	1
DNAH11	chr7	21631098/G//C	nonsynonymous	0	1
SSPO	chr7	149488783/C//T	nonsynonymous	0	1
CTSB	chr8	11704681/G//C	synonymous	0	1
CARNMT1	chr9	77631350/GTT//G	synonymous	0	1
PRDX3	chr10	120934022/G//A	synonymous	0	1
PAOX	chr10	135195017/T//A	nonsynonymous	0	1
ABTB2	chr11	34192517/G//A	nonsynonymous	0	1
MED17	chr11	93540780/A//G	synonymous	0	1
DYNC2H1	chr11	103041005/T//C	nonsynonymous	0	1
VPS11	chr11	118947782/G//C	nonsynonymous	0	1
ARHGEF12	chr11	120351130/C//T	nonsynonymous	0	1
A2M	chr12	9258949/A//G	synonymous	0	1
KANSL2	chr12	49063018/G//C	synonymous	0	1
ITGA7	chr12	56086623/C//A	synonymous	0	1
LRP1	chr12	57598290/A//T	synonymous	0	1
POLR3B	chr12	106848487/G//A	nonsynonymous	0	1
AACS	chr12	125570949/A//T	synonymous	0	1
ZNF84	chr12	133634654/GAC//G	nonsynonymous	0	1
DIS3	chr13	73342966/T//G	nonsynonymous	0	1
SRP54	chr14	35492268/A//G	nonsynonymous	0	1
RTN1	chr14	60194198/C//A	nonsynonymous	0	1
GANC	chr15	42630650/TACTC//T	nonsynonymous	0	1
MYO5C	chr15	52571772/C//T	nonsynonymous	0	1
CORO2B	chr15	69004035/G//T	nonsynonymous	0	1
EFL1	chr15	82444297/G//A	nonsynonymous	0	1
XYLT1	chr16	17211800/G//A	nonsynonymous	0	1
CHD3	chr17	7810967/G//T	nonsynonymous	0	1
GGNBP2	chr17	34923614/A//G	nonsynonymous	0	1
PRKCA	chr17	64684448/C//T	nonsynonymous	0	1
C19orf45	chr19	7566044/C//A	nonsynonymous	0	1
	chr19	18375326/G//T	synonymous	0	1
ZNF486	chr19	20307806/C//A	nonsynonymous	0	1
ZNF527	chr19	37880479/G//A	nonsynonymous	0	1
DHX34	chr19	47856561/G//T	nonsynonymous	0	1
ZNF541	chr19	48052653/T//A	nonsynonymous	0	1
PRAME	chr22	22893313/G//A	nonsynonymous	0	1
ZXDA	chrX	57936978/C//G	synonymous	0	1
KLHL13	chrX	117043440/T//G	nonsynonymous	0	1
PRDM16	chr1	3301849/A//G	nonsynonymous	0	1
KLF17	chr1	44595163/T//C	nonsynonymous	0	1
ELAVL4	chr1	50666631/C//T	synonymous	0	1
NOS1AP	chr1	162124255/C//T	nonsynonymous	0	1
DPT	chr1	168670333/C//T	nonsynonymous	0	1
PRRC2C	chr1	171491368/G//A	nonsynonymous	0	1
DENND1B	chr1	197510108/T//C	nonsynonymous	0	1
PXDN	chr2	1668851/C//T	synonymous	0	1
GYPC	chr2	127447870/A//T	nonsynonymous	0	1
FMNL2	chr2	153483202/C//A	nonsynonymous	0	1
KIT	chr4	55603420/A//G	nonsynonymous	0	1
SLCO4C1	chr5	101606411/C//T	nonsynonymous	0	1
TBC1D9B	chr5	179306701/G//A	nonsynonymous	0	1
MUC21	chr6	30955382/C//T	nonsynonymous	0	1
EYS	chr6	64498113/G//A	synonymous	0	1
LAMA2	chr6	129573315/A//G	synonymous	0	1
BCLAF1	chr6	136599103/C//T	nonsynonymous	0	1
FERD3L	chr7	19184820/G//A	nonsynonymous	0	1
	chr7	94903240/G//T	synonymous	0	1
KDM7A	chr7	139829417/C//T	synonymous	0	1
MSR1	chr8	16001066/C//T	nonsynonymous	0	1
UBXN8	chr8	30614303/A//G	nonsynonymous	0	1
GRHPR	chr9	37436696/C//T	nonsynonymous	0	1
CNTNAP3	chr9	39132943/C//T	nonsynonymous	0	1
MSANTD3-TMEFF1	chr9	103310072/T//C	nonsynonymous	0	1
PPA1	chr10	71977608/G//A	nonsynonymous	0	1
DOCK1	chr10	129216764/G//A	nonsynonymous	0	1
DNHD1	chr11	6568201/G//A	nonsynonymous	0	1

SLC35C1	chr11	45832477/G//A	nonsynonymous	0	1
PPME1	chr11	73958143/A//G	synonymous	0	1
JAML	chr11	118081198/G//A	synonymous	0	1
KCNA5	chr12	5153956/G//A	nonsynonymous	0	1
C12orf57	chr12	7053665/T//C	nonsynonymous	0	1
AICDA	chr12	8757413/C//T	nonsynonymous	0	1
SLCO1B3	chr12	21054390/C//T	synonymous	0	1
KIF21A	chr12	39730984/T//C	nonsynonymous	0	1
SLC41A2	chr12	105255083/G//A	nonsynonymous	0	1
DNAH10	chr12	124403440/G//A	nonsynonymous	0	1
GLT1D1	chr12	129360490/G//A	nonsynonymous	0	1
RIMBP2	chr12	130935810/G//A	nonsynonymous	0	1
PCDH9	chr13	67801737/G//T	nonsynonymous	0	1
CRIP2	chr14	105944815/C//A	nonsynonymous	0	1
SRCAP	chr16	30750376/G//A	synonymous	0	1
ZNF319	chr16	58030510/C//T	nonsynonymous	0	1
ATP2A3	chr17	3831618/C//A	nonsynonymous	0	1
STAT5B	chr17	40362215/A//G	nonsynonymous	0	1
MEIOC	chr17	42734017/T//C	synonymous	0	1
ZNF136	chr19	12298752/A//G	nonsynonymous	0	1
ZNF563	chr19	12430623/A//G	synonymous	0	1
SPTBN4	chr19	41009902/C//T	nonsynonymous	0	1
CCDC114	chr19	48805993/G//A	nonsynonymous	0	1
TARM1	chr19	54578369/G//A	synonymous	0	1
MYLK2	chr20	30419606/G//A	nonsynonymous	0	1
FAAH	chr1	46871046/C//T	synonymous	0	1
CELSR2	chr1	109813852/T//C	nonsynonymous	0	1
MEX3A	chr1	156046686/AGAG//A	nonsynonymous	0	1
CACNA1E	chr1	181546947/G//C	synonymous	0	1
ZNF648	chr1	182026134/G//C	nonsynonymous	0	1
MAP3K21	chr1	233463917/A//G	nonsynonymous	0	1
MAP3K21	chr1	233490692/T//A	nonsynonymous	0	1
FMN2	chr1	240256023/TGCA//T	nonsynonymous	0	1
HNRNPU	chr1	245018760/C//G	nonsynonymous	0	1
SLC30A6	chr2	32396422/C//G	nonsynonymous	0	1
CTNNA2	chr2	80085164/C//T	synonymous	0	1
TTN	chr2	179407204/C//G	nonsynonymous	0	1
PLCL1	chr2	198950551/G//A	synonymous	0	1
NKTR	chr3	42679465/A//T	nonsynonymous	0	1
GYG1	chr3	148714146/C//T	synonymous	0	1
SLITRK3	chr3	164908099/T//C	nonsynonymous	0	1
GRID2	chr4	94006379/G//A	nonsynonymous	0	1
DDIT4L	chr4	101111166/A//C	synonymous	0	1
VCAN	chr5	82836034/A//T	nonsynonymous	0	1
APC	chr5	112175198/C//T	nonsynonymous	0	1
ADGRB3	chr6	69685195/G//C	nonsynonymous	0	1
PHIP	chr6	79664962/G//A	nonsynonymous	0	1
REV3L	chr6	111656707/T//TG	nonsynonymous	0	1
ABCA13	chr7	48319472/T//A	nonsynonymous	0	1
NOS3	chr7	150705989/TCCTCAGCCCTCACC	synonymous	0	1
CPSF1	chr8	145626084/G//A	nonsynonymous	0	1
FAM122A	chr9	71395653/C//G	nonsynonymous	0	1
ASB13	chr10	5693202/G//A	nonsynonymous	0	1
NCOA4	chr10	51580937/C//G	nonsynonymous	0	1
OR52K1	chr11	4510924/G//A	nonsynonymous	0	1
EXPH5	chr11	108385394/A//G	synonymous	0	1
TECTA	chr11	120976656/C//T	nonsynonymous	0	1
TAS2R46	chr12	11214879/C//A	synonymous	0	1
GPR19	chr12	12814146/T//C	nonsynonymous	0	1
LRRK2	chr12	40672050/C//T	nonsynonymous	0	1
DIP2B	chr12	51089093/T//C	nonsynonymous	0	1
ULK1	chr12	132393283/C//T	synonymous	0	1
POLE2	chr14	50133051/T//C	synonymous	0	1
FAH	chr15	80467429/G//C	synonymous	0	1
ACSM2B	chr16	20554464/A//G	nonsynonymous	0	1
ARHGAP27	chr17	43472704/C//T	synonymous	0	1
UTP18	chr17	49338127/T//C	nonsynonymous	0	1
SCN4A	chr17	62050003/T//C	nonsynonymous	0	1
NEDD4L	chr18	55996241/A//G	nonsynonymous	0	1
MADCAM1	chr19	498079/G//C	nonsynonymous	0	1

PTPRS	chr19	5222958/GGAGAAGGAC//G	nonsynonymous	0	1
ZNF331	chr19	54080960/G//A	synonymous	0	1
SNTA1	chr20	32005712/C//T	nonsynonymous	0	1
JPH2	chr20	42744819/G//A	nonsynonymous	0	1
CDH4	chr20	60511941/C//T	synonymous	0	1
PI4KA	chr22	21158696/C//G	synonymous	0	1
PRAME	chr22	22893212/G//T	synonymous	0	1
LARGE1	chr22	34046553/G//A	nonsynonymous	0	1
UXT	chrX	47518241/T//G	nonsynonymous	0	1
STARD8	chrX	67937719/TGAGGATGG//T	nonsynonymous	0	1
MED12	chrX	70354569/C//G	synonymous	0	1
ARMCX2	chrX	100911414/A//G	synonymous	0	1
COL4A5	chrX	107865997/A//G	synonymous	0	1
EFCAB7	chr1	64022832/G//C	nonsynonymous	1	0
CLCA1	chr1	86952406/C//T	synonymous	1	0
SPAG17	chr1	118550809/C//T	nonsynonymous	1	0
HRNR	chr1	152192648/C//T	nonsynonymous	1	0
GATAD2B	chr1	153784275/C//T	nonsynonymous	1	0
WNT9A	chr1	228109374/G//A	nonsynonymous	1	0
MAP3K21	chr1	233498021/C//T	nonsynonymous	1	0
RYR2	chr1	237890502/C//T	synonymous	1	0
EPAS1	chr2	46609650/G//A	nonsynonymous	1	0
EIF2AK3	chr2	88888278/C//T	nonsynonymous	1	0
ZAP70	chr2	98340650/G//A	nonsynonymous	1	0
EIF5B	chr2	100009444/G//A	synonymous	1	0
FAP	chr2	163074615/A//G	nonsynonymous	1	0
SNED1	chr2	242007271/G//A	nonsynonymous	1	0
TRANK1	chr3	36873092/C//T	nonsynonymous	1	0
CCNL1	chr3	156867901/T//C	nonsynonymous	1	0
KDR	chr4	55953916/C//G	nonsynonymous	1	0
TACR3	chr4	104511030/C//T	nonsynonymous	1	0
FAT1	chr4	187557801/G//A	nonsynonymous	1	0
DNAH5	chr5	13829794/C//T	nonsynonymous	1	0
ADGRV1	chr5	89981640/G//A	synonymous	1	0
APC	chr5	112175907/C//G	nonsynonymous	1	0
PCDHA1	chr5	140167762/C//T	synonymous	1	0
PCDHA12	chr5	140256686/G//A	synonymous	1	0
PCDHB15	chr5	140625359/C//T	synonymous	1	0
HIST1H3E	chr6	26225656/G//T	nonsynonymous	1	0
MDC1	chr6	30675208/G//T	nonsynonymous	1	0
EYS	chr6	65300806/A//G	nonsynonymous	1	0
LATS1	chr6	150004255/C//T	nonsynonymous	1	0
LOXL2	chr8	23190988/C//T	nonsynonymous	1	0
OPRK1	chr8	54142294/C//T	nonsynonymous	1	0
NBN	chr8	90958412/T//A	nonsynonymous	1	0
RIMS2	chr8	104934016/T//C	synonymous	1	0
FAM135B	chr8	139153536/C//T	nonsynonymous	1	0
PUF60	chr8	144898899/C//T	nonsynonymous	1	0
MPDZ	chr9	13121870/G//A	nonsynonymous	1	0
OR13J1	chr9	35870034/C//T	nonsynonymous	1	0
PRRX2	chr9	132484619/T//A	synonymous	1	0
DIP2C	chr10	436702/C//T	synonymous	1	0
MLLT10	chr10	21884287/A//G	nonsynonymous	1	0
CDH23	chr10	73571109/C//T	nonsynonymous	1	0
HBD	chr11	5255663/T//C	nonsynonymous	1	0
CABP2	chr11	67287283/G//A	synonymous	1	0
IGHMBP2	chr11	68673583/G//A	nonsynonymous	1	0
HEPHL1	chr11	93796831/C//T	synonymous	1	0
CEP57	chr11	95546688/T//C	synonymous	1	0
EPS8	chr12	15819385/G//A	synonymous	1	0
KRAS	chr12	25378562/C//G	nonsynonymous	1	0
TRHDE	chr12	73046109/C//A	nonsynonymous	1	0
C12orf74	chr12	93100450/G//C	nonsynonymous	1	0
POLR3B	chr12	106889916/G//T	nonsynonymous	1	0
MTUS2	chr13	29855976/C//T	nonsynonymous	1	0
NBEA	chr13	35745571/C//T	nonsynonymous	1	0
KLF5	chr13	73636639/C//G	nonsynonymous	1	0
TCL1A	chr14	96178712/G//A	nonsynonymous	1	0
PPP1R13B	chr14	104204182/G//A	synonymous	1	0
NSMCE3	chr15	29561026/G//A	nonsynonymous	1	0

TGM7	chr15	43571406/C//T	nonsynonymous	1	0
FBN1	chr15	48787439/C//A	nonsynonymous	1	0
KIF23	chr15	69708349/C//T	nonsynonymous	1	0
BNC1	chr15	83931848/G//A	nonsynonymous	1	0
TRAF7	chr16	2226323/G//A	nonsynonymous	1	0
PRSS8	chr16	31144207/C//T	nonsynonymous	1	0
HPR	chr16	72110819/G//A	nonsynonymous	1	0
GSE1	chr16	85696586/G//A	nonsynonymous	1	0
PABPN1L	chr16	88930646/G//A	nonsynonymous	1	0
MYH4	chr17	10358985/C//T	nonsynonymous	1	0
SHISA6	chr17	11461388/A//G	nonsynonymous	1	0
COX10	chr17	14110308/A//AGCCCCTGT	nonsynonymous	1	0
FBXW10	chr17	18647651/T//C	nonsynonymous	1	0
FAM83G	chr17	18874593/C//T	synonymous	1	0
EPOP	chr17	36829808/C//T	nonsynonymous	1	0
CACNA1G	chr17	48650035/C//T	synonymous	1	0
PRR11	chr17	57262793/A//T	synonymous	1	0
VMP1	chr17	57851137/A//G	nonsynonymous	1	0
CSH2	chr17	61949599/C//T	nonsynonymous	1	0
SCN4A	chr17	62024482/G//T	nonsynonymous	1	0
SLC39A11	chr17	70845904/G//A	nonsynonymous	1	0
SEC14L1	chr17	75189555/A//T	nonsynonymous	1	0
LOXHD1	chr18	44057524/C//T	nonsynonymous	1	0
DOT1L	chr19	2199932/A//G	nonsynonymous	1	0
THOP1	chr19	2810693/G//A	synonymous	1	0
HSD11B1L	chr19	5688297/C//T	synonymous	1	0
RFX2	chr19	6044281/G//A	nonsynonymous	1	0
PPAN-P2RY11	chr19	10225241/C//T	nonsynonymous	1	0
ZNF536	chr19	31039467/C//T	nonsynonymous	1	0
TSHZ3	chr19	31770391/G//A	nonsynonymous	1	0
ATP1A3	chr19	42482320/G//A	nonsynonymous	1	0
ZNF415	chr19	53611776/G//A	nonsynonymous	1	0
ZNF530	chr19	58117659/C//T	nonsynonymous	1	0
GNAS	chr20	57429236/G//A	nonsynonymous	1	0
GNAS	chr20	57484420/C//T	nonsynonymous	1	0
TPTE	chr21	10914363/C//T	synonymous	1	0
HSF2BP	chr21	45076539/C//T	nonsynonymous	1	0
NLGN4X	chrX	5821879/G//A	synonymous	1	0
TLR8	chrX	12940255/T//C	synonymous	1	0
POLA1	chrX	24735881/C//T	synonymous	1	0
GK	chrX	30737580/G//A	nonsynonymous	1	0
MAOB	chrX	43637944/G//A	synonymous	1	0
USP11	chrX	47104443/G//A	synonymous	1	0
SYN1	chrX	47434143/C//T	nonsynonymous	1	0
SATL1	chrX	84362915/G//A	nonsynonymous	1	0
RBMXL3	chrX	114426744/G//A	nonsynonymous	1	0
THOC2	chrX	122830662/G//A	nonsynonymous	1	0
MTM1	chrX	149818250/A//G	nonsynonymous	1	0
MTCP1	chrX	154294192/C//T	nonsynonymous	1	0
TARDBP	chr1	11078910/T//A	nonsynonymous	1	0
PRAMEF1	chr1	12855898/C//T	nonsynonymous	1	0
RNPEP	chr1	201951920/C//T	synonymous	1	0
PGBD5	chr1	230486805/C//T	nonsynonymous	1	0
MAP3K21	chr1	233497916/C//T	nonsynonymous	1	0
NCKAP5	chr2	133618083/A//T	synonymous	1	0
HOXD8	chr2	176995085/T//C	synonymous	1	0
ERBB4	chr2	212578372/A//G	synonymous	1	0
ERBB4	chr2	212615399/C//T	nonsynonymous	1	0
KIT	chr4	55593603/TGGAAGG//T	nonsynonymous	1	0
KIT	chr4	55594258/T//C	nonsynonymous	1	0
	chr5	126387609/G//A	synonymous	1	0
TENM2	chr5	167689675/G//A	nonsynonymous	1	0
PCLO	chr7	82580650/G//A	nonsynonymous	1	0
ATP6V0A4	chr7	138394426/G//A	nonsynonymous	1	0
HR	chr8	21980020/C//A	nonsynonymous	1	0
FANCC	chr9	97879628/A//G	synonymous	1	0
RGS3	chr9	116346364/G//A	nonsynonymous	1	0
MUC6	chr11	1016756/G//A	synonymous	1	0
OR8I2	chr11	55861245/A//G	synonymous	1	0
APOBEC1	chr12	7805221/G//C	synonymous	1	0

YBX3	chr12	10862670/C//T	nonsynonymous	1	0
RHEBL1	chr12	49460397/C//G	synonymous	1	0
FAIM2	chr12	50264381/G//A	nonsynonymous	1	0
CCDC92	chr12	124421990/C//T	nonsynonymous	1	0
DAAM1	chr14	59806785/T//C	synonymous	1	0
ZC2HC1C	chr14	75537903/G//T	nonsynonymous	1	0
TOX3	chr16	52473242/G//T	synonymous	1	0
SLFN11	chr17	33690685/G//A	nonsynonymous	1	0
PDK2	chr17	48186661/G//A	nonsynonymous	1	0
QRICH2	chr17	74276944/C//A	nonsynonymous	1	0
FSCN2	chr17	79495674/C//T	synonymous	1	0
FASN	chr17	80043515/A//G	nonsynonymous	1	0
SPTBN4	chr19	41066078/A//C	nonsynonymous	1	0
LENG8	chr19	54967868/G//C	nonsynonymous	1	0
URB1	chr21	33709798/C//T	synonymous	1	0
CECR2	chr22	18022054/C//T	nonsynonymous	1	0
NPTXR	chr22	39222613/G//A	synonymous	1	0
SNU13	chr22	42070861/A//G	synonymous	1	0
PAGE1	chrX	49455858/C//T	nonsynonymous	1	0
AMER1	chrX	63410313/G//T	nonsynonymous	1	0
MAP7D1	chr1	36642310/GAGA//G	nonsynonymous	0	1
STXBP3	chr1	109289364/C//T	nonsynonymous	0	1
DENND4B	chr1	153914805/T//C	nonsynonymous	0	1
KCNK2	chr1	215368364/G//T	nonsynonymous	0	1
PXDN	chr2	1652633/G//A	synonymous	0	1
ALMS1	chr2	73678590/G//T	nonsynonymous	0	1
VWA3B	chr2	98809455/A//T	nonsynonymous	0	1
MARCH7	chr2	160585594/AGTGC//A	nonsynonymous	0	1
KCNH7	chr2	163360995/C//T	synonymous	0	1
SCN3A	chr2	166011159/C//T	nonsynonymous	0	1
XIRP2	chr2	168115731/G//A	synonymous	0	1
TTN	chr2	179430818/G//A	nonsynonymous	0	1
EPHA3	chr3	89391072/GTCCGCT//G	nonsynonymous	0	1
SNX4	chr3	125208266/C//T	synonymous	0	1
PRR23A	chr3	138724770/G//A	nonsynonymous	0	1
DGKG	chr3	185986591/G//A	nonsynonymous	0	1
DKK2	chr4	107847019/G//A	nonsynonymous	0	1
ANK2	chr4	114279112/G//A	nonsynonymous	0	1
PCDH18	chr4	138452494/G//T	nonsynonymous	0	1
SLC6A3	chr5	1406366/G//A	synonymous	0	1
DNAH5	chr5	13810172/G//A	synonymous	0	1
CDH12	chr5	22078802/A//G	synonymous	0	1
CDH6	chr5	31267728/C//T	nonsynonymous	0	1
RASA1	chr5	86674288/C//T	nonsynonymous	0	1
SLC23A1	chr5	138714339/C//T	nonsynonymous	0	1
SPRY4	chr5	141694154/G//A	nonsynonymous	0	1
CSNK1A1	chr5	148929690/C//T	nonsynonymous	0	1
ARSI	chr5	149677099/C//T	nonsynonymous	0	1
SLC36A3	chr5	150666876/G//A	synonymous	0	1
HFE	chr6	26091613/T//C	nonsynonymous	0	1
MUC22	chr6	31002760/C//T	synonymous	0	1
TNXB	chr6	32021419/C//T	nonsynonymous	0	1
HDAC2	chr6	114277295/G//A	nonsynonymous	0	1
HEY2	chr6	126080810/C//T	synonymous	0	1
ESR1	chr6	152265629/C//T	nonsynonymous	0	1
SYNE1	chr6	152454621/T//C	synonymous	0	1
ARID1B	chr6	157528133/G//T	nonsynonymous	0	1
INTS1	chr7	1510498/G//A	synonymous	0	1
INTS1	chr7	1511235/C//T	nonsynonymous	0	1
NXPH1	chr7	8790753/G//A	nonsynonymous	0	1
C7orf71	chr7	26686141/C//T	synonymous	0	1
CAMK2B	chr7	44259764/C//T	nonsynonymous	0	1
IGFBP1	chr7	45928224/G//A	synonymous	0	1
ABCA13	chr7	48311785/G//A	nonsynonymous	0	1
DGKI	chr7	137080408/C//T	nonsynonymous	0	1
ATP6V0A4	chr7	138482810/A//G	synonymous	0	1
DLGAP2	chr8	1496964/C//T	synonymous	0	1
CSMD1	chr8	2813210/C//T	nonsynonymous	0	1
RIMS2	chr8	105261803/C//T	synonymous	0	1
LRP12	chr8	105509515/C//T	nonsynonymous	0	1

	chr8	144671575/T//C	synonymous	0	1
TYRP1	chr9	12694273/C//T	nonsynonymous	0	1
TAF1L	chr9	32630463/G//A	synonymous	0	1
APBA1	chr9	72131174/G//A	nonsynonymous	0	1
MSANTD3-TMEFF1	chr9	103323731/T//C	synonymous	0	1
SVEP1	chr9	113198774/C//T	synonymous	0	1
CUL2	chr10	35320420/T//A	nonsynonymous	0	1
NRAP	chr10	115410252/G//A	nonsynonymous	0	1
OR51A4	chr11	4967809/G//T	nonsynonymous	0	1
TUB	chr11	8122144/G//A	nonsynonymous	0	1
MYOD1	chr11	17741920/C//T	synonymous	0	1
	chr11	17801104/C//T	synonymous	0	1
EXT2	chr11	44219449/T//C	nonsynonymous	0	1
CREB3L1	chr11	46339016/C//T	synonymous	0	1
SLC39A13	chr11	47435028/C//T	synonymous	0	1
OR8H2	chr11	55872750/G//A	nonsynonymous	0	1
INCENP	chr11	61897635/G//A	synonymous	0	1
LRP5	chr11	68193589/A//G	nonsynonymous	0	1
NXPE2	chr11	114577600/C//T	nonsynonymous	0	1
USP5	chr12	6969584/C//T	nonsynonymous	0	1
DDIT3	chr12	57910578/A//G	synonymous	0	1
CAND1	chr12	67700353/C//T	nonsynonymous	0	1
BEST3	chr12	70048984/G//A	synonymous	0	1
MIPEP	chr13	24411824/A//T	synonymous	0	1
MIPEP	chr13	24411825/AG//A	nonsynonymous	0	1
COG6	chr13	40253690/C//A	nonsynonymous	0	1
CNMD	chr13	53313270/C//T	nonsynonymous	0	1
FAM155A	chr13	108518419/C//T	nonsynonymous	0	1
C14orf169	chr14	73959080/T//C	nonsynonymous	0	1
APBA2	chr15	29346112/G//A	nonsynonymous	0	1
RPS27L	chr15	63446193/A//G	synonymous	0	1
KBTBD13	chr15	65370123/G//A	nonsynonymous	0	1
PRSS41	chr16	2848596/G//A	synonymous	0	1
GRIN2A	chr16	9943712/G//A	nonsynonymous	0	1
MKL2	chr16	14342896/G//A	synonymous	0	1
PRKCB	chr16	24135187/C//T	nonsynonymous	0	1
ZNF688	chr16	30581294/G//T	nonsynonymous	0	1
RNF40	chr16	30773833/CAG//C	synonymous	0	1
ADGRG3	chr16	57713101/G//A	nonsynonymous	0	1
UTP4	chr16	69177217/G//A	nonsynonymous	0	1
ZNF778	chr16	89288555/A//G	synonymous	0	1
ZNF232	chr17	5009805/A//G	nonsynonymous	0	1
RNF222	chr17	8296744/A//G	synonymous	0	1
RAI1	chr17	17699461/C//T	nonsynonymous	0	1
TBX2	chr17	59485586/C//T	nonsynonymous	0	1
LRRC37A3	chr17	62893346/C//A	synonymous	0	1
NARF	chr17	80417947/A//G	nonsynonymous	0	1
FN3K	chr17	80699254/C//T	synonymous	0	1
MEP1B	chr18	29775442/A//AGCTTGGG	nonsynonymous	0	1
GTF2F1	chr19	6381587/C//T	synonymous	0	1
ZNF560	chr19	9577878/G//A	nonsynonymous	0	1
MAST1	chr19	12979553/G//A	nonsynonymous	0	1
LGI4	chr19	35622424/C//T	nonsynonymous	0	1
TRAPPC6A	chr19	45667170/TCACAGACGGGCAGG	nonsynonymous	0	1
HRC	chr19	49657913/C//T	synonymous	0	1
TARM1	chr19	54577356/G//A	synonymous	0	1
ZNF549	chr19	58049854/C//T	synonymous	0	1
ANKEF1	chr20	10025071/G//A	synonymous	0	1
DLGAP4	chr20	35060415/C//T	nonsynonymous	0	1
SEMG2	chr20	43851947/T//G	nonsynonymous	0	1
FAM65C	chr20	49226184/C//T	nonsynonymous	0	1
CTCFL	chr20	56099365/GAGTTGAAACA//G	synonymous	0	1
COL20A1	chr20	61943878/C//T	nonsynonymous	0	1
PRPF6	chr20	62664380/A//C	synonymous	0	1
DSCAM	chr21	41710046/C//T	nonsynonymous	0	1
PPIL2	chr22	22039136/A//G	synonymous	0	1
SEC14L6	chr22	30928795/G//A	nonsynonymous	0	1
RFPL3	chr22	32754103/A//C	nonsynonymous	0	1
EP300	chr22	41545858/C//T	nonsynonymous	0	1
	chr22	45958897/G//A	synonymous	0	1

NRK	chrX	105153818/C//T	nonsynonymous	0	1
OPN1LW	chrX	153421829/A//G	nonsynonymous	0	1
